# Health sector spending and spending on HIV/AIDS, tuberculosis, and malaria, and development assistance for health: progress towards Sustainable Development Goal 3

**DOI:** 10.1016/S0140-6736(20)30608-5

**Published:** 2020-09-05

**Authors:** Global Burden of Disease Health Financing Collaborator Network, Angela E Micah, Yanfang Su, Steven D Bachmeier, Abigail Chapin, Ian E Cogswell, Sawyer W Crosby, Brandon Cunningham, Anton C Harle, Emilie R Maddison, Modhurima Moitra, Maitreyi Sahu, Matthew T Schneider, Kyle E Simpson, Hayley N Stutzman, Golsum Tsakalos, Rahul R Zende, Bianca S Zlavog, Cristiana Abbafati, Zeleke Hailemariam Abebo, Hassan Abolhassani, Michael R M Abrigo, Muktar Beshir Ahmed, Rufus Olusola Akinyemi, Khurshid Alam, Saqib Ali, Cyrus Alinia, Vahid Alipour, Syed Mohamed Aljunid, Ali Almasi, Nelson Alvis-Guzman, Robert Ancuceanu, Tudorel Andrei, Catalina Liliana Andrei, Mina Anjomshoa, Carl Abelardo T Antonio, Jalal Arabloo, Morteza Arab-Zozani, Olatunde Aremu, Desta Debalkie Atnafu, Marcel Ausloos, Leticia Avila-Burgos, Martin Amogre Ayanore, Samad Azari, Tesleem Kayode Babalola, Mojtaba Bagherzadeh, Atif Amin Baig, Ahad Bakhtiari, Maciej Banach, Srikanta K Banerjee, Till Winfried Bärnighausen, Sanjay Basu, Bernhard T Baune, Mohsen Bayati, Adam E Berman, Reshmi Bhageerathy, Pankaj Bhardwaj, Mehdi Bohluli, Reinhard Busse, Lucero Cahuana-Hurtado, Luis LA Alberto Cámera, Carlos A Castañeda-Orjuela, Ferrán Catalá-López, Muge Cevik, Vijay Kumar Chattu, Lalit Dandona, Rakhi Dandona, Mostafa Dianatinasab, Hoa Thi Do, Leila Doshmangir, Maha El Tantawi, Sharareh Eskandarieh, Firooz Esmaeilzadeh, Anwar Faraj, Farshad Farzadfar, Florian Fischer, Nataliya A Foigt, Nancy Fullman, Mohamed M Gad, Mansour Ghafourifard, Ahmad Ghashghaee, Asadollah Gholamian, Salime Goharinezhad, Ayman Grada, Hassan Haghparast Bidgoli, Samer Hamidi, Hilda L Harb, Edris Hasanpoor, Simon I Hay, Delia Hendrie, Nathaniel J Henry, Claudiu Herteliu, Michael K Hole, Mehdi Hosseinzadeh, Sorin Hostiuc, Tanvir M Huda, Ayesha Humayun, Bing-Fang Hwang, Olayinka Stephen Ilesanmi, Usman Iqbal, Seyed Sina N Irvani, Sheikh Mohammed Shariful Islam, M Mofizul Islam, Mohammad Ali Jahani, Mihajlo Jakovljevic, Spencer L James, Zohre Javaheri, Jost B Jonas, Farahnaz Joukar, Jacek Jerzy Jozwiak, Mikk Jürisson, Rohollah Kalhor, Behzad Karami Matin, Salah Eddin Karimi, Gbenga A Kayode, Ali Kazemi Karyani, Yohannes Kinfu, Adnan Kisa, Stefan Kohler, Hamidreza Komaki, Soewarta Kosen, Anirudh Kotlo, Ai Koyanagi, G Anil Kumar, Dian Kusuma, Van C Lansingh, Anders O Larsson, Savita Lasrado, Shaun Wen Huey Lee, Lee-Ling Lim, Rafael Lozano, Hassan Magdy Abd El Razek, Mokhtar Mahdavi Mahdavi, Shokofeh Maleki, Reza Malekzadeh, Fariborz Mansour-Ghanaei, Mohammad Ali Mansournia, Lorenzo Giovanni Mantovani, Gabriel Martinez, Seyedeh Zahra Masoumi, Benjamin Ballard Massenburg, Ritesh G Menezes, Endalkachew Worku Mengesha, Tuomo J Meretoja, Atte Meretoja, Tomislav Mestrovic, Neda Milevska Kostova, Ted R Miller, Andreea Mirica, Erkin M Mirrakhimov, Masoud Moghadaszadeh, Bahram Mohajer, Efat Mohamadi, Aso Mohammad Darwesh, Abdollah Mohammadian-Hafshejani, Reza Mohammadpourhodki, Shafiu Mohammed, Farnam Mohebi, Ali H Mokdad, Shane Douglas Morrison, Jonathan F Mosser, Seyyed Meysam Mousavi, Moses K Muriithi, Saravanan Muthupandian, Chaw-Yin Myint, Mehdi Naderi, Ahamarshan Jayaraman Nagarajan, Cuong Tat Nguyen, Huong Lan Thi Nguyen, Justice Nonvignon, Jean Jacques Noubiap, In-Hwan Oh, Andrew T Olagunju, Jacob Olusegun Olusanya, Bolajoko Olubukunola Olusanya, Ahmed Omar Bali, Obinna E Onwujekwe, Stanislav S Otstavnov, Nikita Otstavnov, Mayowa Ojo Owolabi, Jagadish Rao Padubidri, Raffaele Palladino, Songhomitra Panda-Jonas, Anamika Pandey, Maarten J Postma, Sergio I Prada, Dimas Ria Angga Pribadi, Mohammad Rabiee, Navid Rabiee, Fakher Rahim, Chhabi Lal Ranabhat, Sowmya J Rao, Priya Rathi, Salman Rawaf, David Laith Rawaf, Lal Rawal, Reza Rawassizadeh, Aziz Rezapour, Siamak Sabour, Mohammad Ali Sahraian, Omar Mukhtar Salman, Joshua A Salomon, Abdallah M Samy, Juan Sanabria, João Vasco Santos, Milena M Santric Milicevic, Bruno Piassi Sao Jose, Miloje Savic, Falk Schwendicke, Subramanian Senthilkumaran, Sadaf G Sepanlou, Edson Serván-Mori, Hamidreza Setayesh, Masood Ali Shaikh, Aziz Sheikh, Kenji Shibuya, Mark G Shrime, Biagio Simonetti, Jasvinder A Singh, Pushpendra Singh, Valentin Yurievich Skryabin, Amin Soheili, Shahin Soltani, Simona Cătălina Ștefan, Rafael Tabarés-Seisdedos, Roman Topor-Madry, Marcos Roberto Tovani-Palone, Bach Xuan Tran, Ravensara Travillian, Eduardo A Undurraga, Pascual R Valdez, Job F M van Boven, Tommi Juhani Vasankari, Francesco S Violante, Vasily Vlassov, Theo Vos, Charles D A Wolfe, Junjie Wu, Sanni Yaya, Vahid Yazdi-Feyzabadi, Paul Yip, Naohiro Yonemoto, Mustafa Z Younis, Chuanhua Yu, Zoubida Zaidi, Sojib Bin Zaman, Mikhail Sergeevich Zastrozhin, Zhi-Jiang Zhang, Yingxi Zhao, Christopher J L Murray, Joseph L Dieleman

## Abstract

**Background:**

Sustainable Development Goal (SDG) 3 aims to “ensure healthy lives and promote well-being for all at all ages”. While a substantial effort has been made to quantify progress towards SDG3, less research has focused on tracking spending towards this goal. We used spending estimates to measure progress in financing the priority areas of SDG3, examine the association between outcomes and financing, and identify where resource gains are most needed to achieve the SDG3 indicators for which data are available.

**Methods:**

We estimated domestic health spending, disaggregated by source (government, out-of-pocket, and prepaid private) from 1995 to 2017 for 195 countries and territories. For disease-specific health spending, we estimated spending for HIV/AIDS and tuberculosis for 135 low-income and middle-income countries, and malaria in 106 malaria-endemic countries, from 2000 to 2017. We also estimated development assistance for health (DAH) from 1990 to 2019, by source, disbursing development agency, recipient, and health focus area, including DAH for pandemic preparedness. Finally, we estimated future health spending for 195 countries and territories from 2018 until 2030. We report all spending estimates in inflation-adjusted 2019 US$, unless otherwise stated.

**Findings:**

Since the development and implementation of the SDGs in 2015, global health spending has increased, reaching $7·9 trillion (95% uncertainty interval 7·8–8·0) in 2017 and is expected to increase to $11·0 trillion (10·7–11·2) by 2030. In 2017, in low-income and middle-income countries spending on HIV/AIDS was $20·2 billion (17·0–25·0) and on tuberculosis it was $10·9 billion (10·3–11·8), and in malaria-endemic countries spending on malaria was $5·1 billion (4·9–5·4). Development assistance for health was $40·6 billion in 2019 and HIV/AIDS has been the health focus area to receive the highest contribution since 2004. In 2019, $374 million of DAH was provided for pandemic preparedness, less than 1% of DAH. Although spending has increased across HIV/AIDS, tuberculosis, and malaria since 2015, spending has not increased in all countries, and outcomes in terms of prevalence, incidence, and per-capita spending have been mixed. The proportion of health spending from pooled sources is expected to increase from 81·6% (81·6–81·7) in 2015 to 83·1% (82·8–83·3) in 2030.

**Interpretation:**

Health spending on SDG3 priority areas has increased, but not in all countries, and progress towards meeting the SDG3 targets has been mixed and has varied by country and by target. The evidence on the scale-up of spending and improvements in health outcomes suggest a nuanced relationship, such that increases in spending do not always results in improvements in outcomes. Although countries will probably need more resources to achieve SDG3, other constraints in the broader health system such as inefficient allocation of resources across interventions and populations, weak governance systems, human resource shortages, and drug shortages, will also need to be addressed.

**Funding:**

The Bill & Melinda Gates Foundation.

## Introduction

In 2015, the 193 member states of the United Nations (UN) adopted the 2030 Agenda for Sustainable Development. The agenda identified 17 Sustainable Development Goals (SDGs) and 169 targets intended to catalyse “peace and prosperity for people and the planet”. Of the 17 goals, many address health indirectly (eg, zero hunger [SDG2], gender equality [SDG5], and clean water and sanitation [SDG6]), while SDG3 focuses directly on health, with the objective being to “ensure healthy lives and promote well-being for all at all ages.”

Substantial effort has been made to quantify the progress towards meeting the targets set in SDG3.^1^, ^2^ Examples include WHO's Thirteenth General Programme of Work, which provides a framework for tracking progress towards the health-related SDGs and research done by the Global Burden of Diseases, Injuries, and Risk Factors study (GBD) Collaborator Network, while less research has focused on tracking spending on SDG priority areas, especially how they relate to specific SDG3 indicators.^3^ Tracking financial resources for SDG3 priority areas is crucial for two distinct reasons. First, any scale-up of the interventions needed to achieve the ambitious health goals will probably require some additional resources. As such, tracking how many resources are spent on health, when and where those resources are spent, and who benefits from them is vital for transparency and assessment of progress towards the goals.^4^ Furthermore, the amount of financial investment in health and how it is spent might be used as a proxy for governments' commitment to achieving SDG3 and health services more broadly. Even in instances where more resources are not needed to achieve the goals (because gains can be made through improvements in efficiency of health systems), knowing precisely how much is being spent and for what purpose is essential for tracking effectiveness and ensuring an equitable distribution of resources. Second, SDG3 target 3.8 identifies financial risk protection and access to essential services as key targets.^5^ Financial risk protection is ensuring that no household endures financial hardship due to large spending on health. Achieving SDG3 target 3.8 not only requries enough resources are available to provide the services and interventions needed to prevent and treat ill health, but also that an awareness of the source of those funds is key. Ensuring that health spending does not lead to financial hardship and impoverishment, known as catastrophic health spending, requires that funds for health be prepaid and pooled across individuals via public or private insurance schemes.^6^ The alternative to prepaid and pooled resources for health is reliance on out-of-pocket spending, which forces households without sufficient resources to choose between receiving health care or medical impoverishment.

**Research in context**

**Evidence before this study**

The Sustainable Development Goals (SDGs) and their related indicators and targets mark a consensus among global leaders about the importance of improving and maintaining health worldwide. To monitor progress towards the health-related SDGs, the United Nation's Voluntary National Reviews Database, WHO, and the Global Burden of Diseases, Injuries, and Risk Factors study (GBD) Collaborator Network have measured health indicators to monitor achievement of SDG3, and the World Bank created the SDG atlas. A multitude of voices are championing progress towards achieving the SDGs, with some also proposing estimates of the financing needs to meet the related health goals. To track financing inputs for health, previous studies by the GBD Health Financing Collaborator Network have estimated past and projected future total health spending in 195 countries and territories from 1995 to 2050, and health investment from international donors to low-income and middle-income countries between 1990 and 2050. In the most recent study, in which spending was estimated in 2018 US$, global health spending was found to reach $8·0 trillion (95% uncertainty interval 7·8–8·1) comprising 8·6% (8·4–8·7) of the global economy in 2016 and was projected to increase to $15·0 trillion (14·0–16·0), that is 9·4% (7·6–11·3), of the global economy by 2050. Additionally, estimates have been published for HIV/AIDS spending in low-income and middle-income countries and malaria spending in 106 malaria-endemic countries (also from the GBD Collaborator Network). Similarly, UNAIDS and WHO have estimated for spending on HIV/AIDS, tuberculosis, and malaria in many low-income and middle-income countries. The studies from the GBD Health Financing Collaborator Network showed that in 2016, US$19·9 billion (15·8–26·3) was spent on HIV/AIDS and $4·3 billion (4·2–4·4) was spent on malaria. The World Malaria Report published by WHO in 2019 showed that US$2·7 billion was invested in malaria control and elimination activities by international partners and governments of malaria endemic countries. For HIV/AIDS, UNAIDS Global AIDS monitoring report showed that in 2018, $19 billion (in 2016 US$) from international and domestic sources was spent and the WHO's Global Report on tuberculosis reported that in 2019 $6·8 billion was spent on tuberculosis diagnosis, prevention, and treatment services. Additionally, the Sustainable Development Solutions Network, the International Monetary Fund, World Bank, and Organization for Economic Co-operation and Development have offered different methods, assumptions, and measures related to the financing needs for SDG3. The Working Group on SDG Costing and Financing has worked to mobilise costing practices and tools to achieve the SDGs. For SDG3 specifically, the Third Edition of the Disease Control Priorities in Developing Countries assessed financial needs for universal health coverage, while researchers at the Institute of Health Metrics and Evaluation have estimated funding gaps to achieve universal health coverage. The Department of Health Systems Governance and Financing at WHO has also projected resource needs to finance transformative health systems towards achievement of SDG3. Beyond estimated financing targets, needs, and gaps, only four of 27 SDG3 indicators have estimates of past or current total spending. These financial estimates are not directly comparable due to differences in study designs, scopes, and completeness.

**Added value of this study**

This study is the first to our knowledge, that assesses spending on and explores the association with health gains for key SDG3 targets related to HIV/AIDS (3.3.1), tuberculosis (3.3.2), malaria (3.3.3), universal health coverage (3.8.1), financial risk protection (3.8.2), and development assistance for health (DAH; 3.b.2). We focused on quantifying total health spending on HIV/AIDS, malaria, and tuberculosis and DAH contributions. Additionally, we provide updated estimates using consistent methods for retrospective and prospective total health sector spending. This work adds value to existing literature by using similar methods as previous studies to quantify progress in financing SDG3 priority by estimating domestic spending by source spending on four SDG3 indicators and DAH funding on eight SDG3 indicators.

**Implications of all available evidence**

Tracking progress towards the financing of health systems and specific targets associated with SDG3 draws attention to the need for sufficient resources to achieve health gains without placing financial hardship on households. Monitoring this progress requires comparable and consistent estimates in financing for health. By providing these estimates, we create a foundation for stakeholders to discuss, set, and reach achievable financial goals. In particular, for some low-income countries our results highlight that the available resources seem insufficient to achieve the SDG3 targets by 2030. This study also highlights the need to estimate the financing available for the other SDG3 priority areas. Furthermore, the nuanced evidence on the scale-up of spending and improvements in health outcomes suggest a complex association between spending and health outcomes. This complexity highlights that, although more resources are probably needed to achieve SDG3, other constraints such as inefficient resource allocation, weak governance systems, inadequate health workforce, and drug shortages will likely need to be addressed to achieve the SDG3 targets.

This study builds on past work and aims to make progress towards filling the current gap in knowledge on the financing of SDG3 priority areas.^7^, ^8^, ^9^ Little evidence exists on how much is being spent towards the SDG3 targets and how this spending relates to changes in health outcomes of interest. The objectives of this study are to measure spending on SDG3 priority areas where estimates are relatively complete and comparable, examine the association between outcomes and financings levels, and identify where resource shortages are most apparent for four SDG3 indicators. We quantified health spending for universal health coverage; domestic and DAH spending on HIV/AIDS, tuberculosis, malaria; and DAH spending for reproductive, maternal, newborn, and child health, tobacco control, non-communicable diseases, vaccines, and human resources. We also evaluated spending against key SDG3 indicators for HIV/AIDS, tuberculosis, malaria, universal health coverage, and pandemic preparedness. Additionally, this research estimates future spending on health up to 2030 and 2050 to highlight the expected resource availability and, in particular, provides information that can be used to identify where more prepaid and pooled resources are needed.

## Methods

### Overview

We measured health sector spending by source; domestic spending on HIV/AIDS, tuberculosis, and malaria; and development assistance for health (DAH; ie, from donors) for as many years as possible with the availability of input data. For total health sector spending and domestic health spending, we generated estimates for 1995–2017 for 195 countries and territories; for domestic spending on HIV, tuberculosis, and malaria, we generated estimates for 2000–17 for 135 low-income and middle-income countries (although for malaria, 28 low-income and middle-income countries without endemic malaria were excluded); and for DAH, we generated estimates for 1990–2019 and all low-income and middle-income countries. Using these health spending estimates, we projected health sector spending to 2030 and 2050. We define health spending similarly to the System of Health Accounts 2011 and the WHO Global Health Expenditure Database as spending on basic infrastructure, services, and supplies to deliver health care. This health spending is exclusive of informal care spending and major capital investments, such as building hospitals.

### Domestic health spending 1995–2017

We estimated three sources of domestic health spending: government, out-of-pocket, and prepaid private spending.^7^ The sum of spending from these three domestic sources, plus DAH, equate to total spending on health, meaning these four sources are mutually exclusive and collectively exhaustive. Government health spending is an aggregate of social health insurance and government public health programmes. Out-of-pocket health spending captures health-care spending by an individual patient or their household, excluding insurance premiums paid before needing care. Prepaid private-health spending includes non-governmental agency spending on health and private insurance. To estimate the three domestic health spending variables, we extracted data from the WHO Global Health Expenditure database for all available countries.^10^ We downloaded the data in current national currency units, adjusted for inflation, and then converted to 2019 $US, completed our analysis, and then also converted our estimates into 2019 purchasing-power parity-adjusted $. We used deflator series and exchange rate data based on data from the International Monetary Fund World Economic Outlook.^11^ For each extracted datapoint, we used the metadata provided by WHO to qualitatively assess the quality of data. We assigned a weight to each downloaded datapoint on the basis of documented source information, completeness of metadata, and documented methods of estimation (more details are in the appendix [pp 14–21]). We then used a spatiotemporal Gaussian process model to generate a complete time series of data from 1995 until 2017 for each country, and 95% uncertainty intervals (UIs).^12^

### Domestic spending on HIV/AIDS, tuberculosis, and malaria 2000–17

We generated estimates of domestic spending for three communicable diseases included in the SDG target 3.3: HIV/AIDS, tuberculosis, and malaria. To generate the three disease-specific spending estimates, we used a similar overarching strategy as for domestic health spending estimates. First, we did a comprehensive search and extracted all available and applicable data, which we put into a common currency for comparability (2019 US$). The input data for our disease-specific spending estimates came from multiple sources.

For HIV/AIDS, we extracted spending data for 135 low-income and middle-income countries from the National AIDS Spending Assessments,^13^ the Global Fund (including concept notes, proposals, and funding landscape documents), National Health Accounts and subaccounts, UNAIDS Global AIDS response progress reports, and three online public databases provided by UNAIDS: the AIDSinfo database, the HIV financing dashboard, and the Asia-Pacific region AIDS Data Hub. Additional details on the data sources we used are in the appendix (pp 113–16).

For tuberculosis, we extracted spending data for 135 low-income and middle-income countries from the WHO Global Tuberculosis database, Global Fund (proposals, concept notes, and funding landscaping documents), National Health Accounts and sub-accounts, WHO Global Health Expenditure database,^10^ National Tuberculosis Reports, Ministry of Health Reports, GBD data, and unit cost data from WHO-Choosing Interventions that are Cost Effective (CHOICE), and Moses et al.^14^ Additional details on the data sources we used are in the appendix (pp 13–21).

For malaria, we extracted spending data for 106 malaria-endemic low-income and middle-income countries from the World Malaria Report, the Global Fund (including concept notes, proposals, and funding landscape documents), National Health Accounts and sub-accounts, the Global Fund Price Quality Reporting, WHO Global Price Reporting Mechanism, Management Sciences for Health reference prices, Global Affordable Medicine Facility, Health Action International database, treatment data provided by the Malaria Atlas Project, Demographic and Health Surveys, malaria out-of-pocket cost literature, malaria inpatient and outpatient cost literature, and inpatient and outpatient unit costs from Moses et al.^14^ Further details on the data sources we used are in the appendix (pp 89–90).

Second, we used a spatiotemporal Gaussian process model to generate a complete time series of estimates by disease from 2000 to 2017 for each country included. For our HIV/AIDS spending estimates, tabulated data of annual spending of all components—government, out-of-pocket, and prepaid private spending—were available, so we used those to generate our estimates. For malaria and tuberculosis, little tabulated data and estimates on out-of-pocket spending were available, so we developed out-of-pocket spending estimates by taking the product of coverage (ie, volume) and unit costs for key services for which users pay out of pocket.

### Universal health coverage, 2000–17

We extracted the universal health coverage service index from the GBD 2017 SDG Collaborators.^1^ The index aggregates across a diverse set of intermediate coverage estimates, such as vaccine coverage, and measured of health system performance. We extracted data on 195 countries from 2000 to 2017 used these data in this analysis.

No commonly agreed on system exists to differentiate between which health spending is intended to help countries achieve universal health coverage. Because of this, we track pooled health spending as a proxy for tracking progress towards financing universal health service coverage. Pooled spending is health-care spending collected in advance and spread across a large set of individuals, and includes government and prepaid private spending and DAH.

### Estimating DAH, 1990–2019

We defined DAH as the financial and in-kind resources transferred through international development agencies to low-income and middle-income countries for the primary purpose of maintaining and improving health. We extracted project disbursement data from online databases, annual reports, and financial statements of the major international development agencies and philanthropic institutions including the World Bank, the Organisation for Economic Co-operation and Development's (OECD's) Creditor Reporting System, and the Bill & Melinda Gates Foundation; details on the agencies and institutions included are in the appendix (pp 28–33). The estimates of DAH include the expenses incurred to administer the grants and loans.

We classified estimates of how DAH funds were disbursed into ten mutually exclusive and collectively exhaustive health focus areas and 52 programme areas on the basis of project descriptions, project titles, including pandemic preparedness, and budget documents. Disbursement of DAH funds to single countries were identified as such, while global initiatives and administrative costs were classified separately. Administrative costs capture the operational cost of running projects—eg, staff salaries. The research and development funds that are included in our DAH estimates are those that are disbursed through international development agencies with the primary intent of the improvement and maintenance of health in low-income and middle-income countries. The DAH contributions towards human resources we captured here include indirect funding for human resources activities, such as per diems, and direct funding for human resources for health projects that invest in human resources activities, such as training, education, and policy development. The health focus areas included in this study are HIV/AIDS; tuberculosis; malaria; reproductive, maternal, newborn, and child health; other infectious disease; non-communicable diseases; sector-wide approaches; and health system strengthening. Detailed descriptions of the methods we used to isolate the disbursements of DAH funds for relevant health focus areas and preliminary estimates are in the appendix (pp 34–45) and elsewhere.^7^, ^15^

The estimates presented here of DAH incorporated improvements in our methods compared with previous years, such as using additional project-level descriptions from the Creditor Reporting System for the allocation of disbursements channelled through non-governmental organisations and refinement of our keyword search list (appendix 34–42).

The Millennium Development Goals (MDGs) were eight development goals adopted by the UN in 2000. The goals, to be achieved by 2015, included the eradication of extreme poverty and hunger; achievement of universal primary enrolment; promotion of gender equality and empowerment of women; reduction in child mortality, HIV/AIDS, malaria, and other diseases; and improvement in maternal health. Like the SDGs, the MDGs included health specific goals and goals focused on other sectors indirectly linked to health. In our analyses, we examine spending over the duration of the MDGs, starting in 2000 up to 2015.

DAH data for 2018 and 2019 are preliminary estimates based on budget data and estimation. Detailed information on the sources of the budget data and the estimation approaches we used are provided in the appendix (pp 29–33).

### Financial risk protection

We extracted incidence data on catastrophic health spending estimates from World Bank World Development Indicators database for all years and countries for which data were available. Reliance on out-of-pocket spending has been shown to be associated with catastrophic health spending (also known as medical impoverishment),^16^, ^17^ which defined by the World Bank World Development index as when a household spends more than 25% of annual household income on health.

### Health spending in the future: 2018 to 2030 and 2050

We estimated gross domestic product (GDP); general government spending (across all sectors); government, out-of-pocket, and prepaid private health spending; and total DAH provided and received from 2018 to 2030 and 2050. The methods used for these projections draw heavily from our previous research,^7^, ^18^, ^19^ with the key updates being the improvement of the retrospective estimates on which these projections are based.

We generated each projection using ensemble modelling techniques, such that the estimates are the mean of 1000 estimated time series from a broad set of models. We determined model selection on the basis of out-of-sample validation and selection was country and year specific. We completed projections sequentially, such that previously projected values could be used as covariates and for bounding other models. For example, government health spending was modelled as a fraction of general government spending, which was modelled as a fraction of GDP. On the basis of model performance, we modelled GDP as a proportion of the population who were of working age, which for this study was determined to be aged 20–65 years.

We modelled DAH as a fraction of the donor country's general government spending, or, for private donors, on the basis of autoregressive integrated moving average (ARIMA) modelling techniques.^20^ We aggregated total DAH across sources. We constructed a separate model that projected the fraction of total DAH that each recipient was expected to receive. As a country's own GDP per-capita increases, the fraction of total DAH received by the country is expected to go down. We also modelled when countries transitioned to being high-income and are no longer eligible to receive DAH.

All projections incorporated several types of uncertainty. We used ensemble modelling techniques to propagate model uncertainty.^21^ We took draws of the variance-covariance matrix of each estimate's model to propagate parameter uncertainty. We based our projection models on the draws of the retrospective estimates to propagate data uncertainty. Finally, we added a random walk residual to each country's and draw's projection to propagate fundamental uncertainty—ie, to mimic the inherent randomness of the observed data. We generated 95% uncertainty intervals (UIs) by taking the 2·5th and 97·5th percentile of the 1000 estimated random draws.

More details are in the appendix (pp 121–41).

### Statistical analysis

We report all spending estimates in inflation-adjusted 2019 US$, although some data are also presented in 2019 purchasing-power parity-adjusted $ and proportion of GDP. We report spending estimates for Venezuela in 2014 US$ because necessary exchange rates for more recent years were not reliable. We evaluated health spending against key indicators relative to SDG3. In particular, we extracted estimates of incidence of HIV/AIDS, tuberculosis, and malaria from GBD 2017,^22^ and the universal health coverage service coverage index.^1^, ^23^

We used different measures to report findings from our spending and outcomes analyses. For HIV/AIDS, we report spending per prevalent case, because a lot of HIV/AIDS spending is determined by the number of people undergoing treatment. For malaria, we report spending per capita, because as countries move towards elimination a lot of malaria spending is on surveillance. For tuberculosis, we report spending per incident case, because a lot of tuberculosis spending is determined by detection of incident cases. Population estimates, both retrospective and prospective were also extracted from the GBD 2017 study.^24^ We analysed the association between universal health coverage service index and pooled health spending by calculating the annualised rate of change in each metric from 2000 up to 2017. For our financial risk protection analysis, we used the estimates of catastrophic health spending and report catastrophic health spending estimates from the World Bank World Development Indicators database. We divided the extracted estimates by total domestic spending on health and then regressed on national income using loess regression methods. Annualised rate of change is only calculated for countries with more than 1 year of catastrophic health spending estimates and when catastrophic health spending was greater than zero.^25^, ^26^ We report estimates of DAH from 1990 up to 2019 for low-income and middle-income countries. The data for 2018 and 2019 are preliminary estimates based on budget data and estimation. We compared DAH contributions over two periods: 2000 up to 2015 and 2015 up to 2019. We also analysed DAH by health focus area specifically reporting contributions towards reproductive, maternal, newborn and child health, tobacco control, vaccines, non-communicable diseases, and human resources. Finally, we report global, income group, region, and country-specific estimates. Income groups are based on World Bank income group classification from 2019, while regions are GBD super-regions (central Europe, eastern Europe, and central Asia; high-income; Latin America and Caribbean; north Africa and the Middle East; south Asia; southeast Asia, east Asia, and Oceania; and sub-Saharan Africa). Argentina is the only country in the World Bank category of low-income and middle-income countries to fall in the GBD high-income super-region; hence, in the present study we do not include Argentina, and its GBD super-region, in figures that disaggregate by GBD super-region. We report aggregate rates that reflect the group of countries or region as a whole, rather than a mean across the countries in that group or region.

We did all analyses using R (version 3.6.0) and Stata (version 15). All spending estimates used in this analysis are publicly available on the Global Health Data Exchange website.

### Role of the funding source

The funder of this study had no role in study design, data collection, data analysis, data interpretation, or writing of the report. All authors had full access to all the data in the study, and AEM and JLD had final responsibility for the decision to submit for publication.

## Results

Table 1 lists the SDG3 targets and the associated indicators for monitoring these targets, and reports existing estimates of financing needed for attaining these targets and our spending estimates. The targets and indicators were determined and agreed on by the member states of the UN, while the financing targets are unofficial estimates of resources needed produced by other researchers. Our estimates of disease-specific spending focus on domestic and DAH spending among 135 low-income and middle-income countries while spending on universal health coverage is measured for 195 countries and territories including high-income countries.

**Table 1:** Health-related goals, health indicators, health targets, and related spending for SDG3 targets

	**SDG target**	**Spending estimate**	**Existing unofficial financing target**
**Target 3.1: by 2030, reduce the global maternal mortality ratio <70 per 100 000 livebirths**			
3.1.1: maternal mortality ratio	Reduce to <70 deaths per 100 000 livebirths by 2030	DAH funding on maternal health was $1·5 billion for 135 low-income and middle-income countries in 2019	$10·5 billion per year in 120 low-income and middle-income countries (UNFPA Nairobi Summit ICPD25,^27^ estimated $115·5 in 2020–30); $3·3 billion* (2014 US$) per year in 67 low-income and middle-income countries^28^
3.1.2: skilled birth attendance	Universal access (100%)	..	..
**Target 3.2: by 2030, end preventable deaths of newborn babies and children younger than 5 years, with all countries aiming to reduce neonatal mortality to at least as low as 12 per 1000 livebirths and under-5 mortality to at least as low as 25 per 1000 livebirths**			
3.2.1: under-5 mortality	Reduce to ≤25 deaths per 1000 livebirths by 2030	DAH on child health was $8·5 billion for 135 low-income and middle-income countries in 2019	..
3.2.2: neonatal mortality	Reduce to ≤12 deaths per 1000 livebirths by 2030	DAH on child health was $8·5 billion for 135 low-income and middle-income countries in 2019	..
**Target 3.3: by 2030, end the epidemics of AIDS, tuberculosis, malaria, and neglected tropical diseases and combat hepatitis, water-borne diseases, and other communicable diseases**			
3.3.1: HIV incidence	Eliminate by 2030	Domestic spending in 2017 was $10·6 billion and DAH was $9·5 billion in 2019 for 135 low-income and middle-income countries	$26·2 billion per year by 2020 and $22·3 billion per year by 2030 in 116 low-income and middle-income countries;^29^ $6·8 billion* per year in 67 low-income and middle-income countries^28^
3.3.2: tuberculosis incidence	Eliminate by 2030	Domestic spending was $9·2 billion in 2017 and DAH was $1·7 billion in 2019 for 135 low-income and middle-income countries	$13 billion by 2022 in 119 low-income and middle-income countries;^30^ $3·8 billion* per year in 67 low-income and middle-income countries^28^
3.3.3: malaria incidence	Eliminate by 2030	Domestic spending in 2017 was $2·6 billion and DAH was $1·1 billion in 2019 on malaria for 106 malaria-endemic countries	$6·6 billion per year by 2020^31^
3.3.4: hepatitis B incidence	Undefined	..	$6 billion* per year in 67 low-income and middle-income countries^28^, ^32^
3.3.5: neglected tropical diseases prevalence	Eliminate by 2030	..	$2·1 billion per year in low-income and middle-income countries^33^
**Target 3.4: by 2030, reduce premature mortality from non-communicable diseases by a third through prevention and treatment and promotion of mental health and wellbeing**			
3.4.2: non-communicable disease mortality	Reduce by a third by 2030	DAH on non-communicable disease was $0·7 billion for 135 low-income and middle-income countries in 2019	$28 billion* per year in 67 low-income and middle-income countries^28^
3.4.2: suicide mortality	Reduce by a third by 2030	..	..
**Target 3.5: strengthen the prevention and treatment of substance abuse, including narcotic drug abuse and harmful use of alcohol**			
3.5.1: substance abuse coverage	Undefined	..	$2 billion* per year in 67 low-income and middle-income countries^28^
3.5.2: alcohol use	Undefined	..	..
**Target 3.6: by 2020, halve the number of global deaths and injuries from road traffic accidents**			
3.6.1: road injury mortality	Reduce by half by 2020	..	..
**Target 3.7: by 2030, ensure universal access to sexual and reproductive health-care services, including for family planning, information and education, and the integration of reproductive health into national strategies and programmes**			
3.7.2: family planning need met, modern contraception methods	Universal access (100%)	DAH on family planning was $1·2 billion for 135 low-income and middle-income countries in 2019	$6·2 billion per year in 120 low-income and middle-income countries (UNFPA Nairobi Summit ICPD25, estimated $68·5 billion for 2020–30)^34^
3.7.2: adolescent birth rate	Undefined	..	..
**Target 3.8: achieve universal health coverage, including financial risk protection, access to quality essential health-care services, and access to safe, effective, quality, and affordable essential medicines and vaccines for all**			
3.8.1: universal health coverage service coverage index	Universal access (100%)	Domestic spending in 2017 and donor funding in 2019 on health was $7·9 trillion (95% UI 7·8–8·0) and $40·6 billion for 195 countries	$274–371 billion* per year in 67 low-income and middle-income countries;^28^ $575·57 billion† in 188 countries;^14^ $113–223 billion† in 83 low-income and lower-middle income countries;^14^, ^35^ $76 per captia per year in 34 low-income countries and $110 per capita per year in 49 lower-middle income countries;^36^ $110‡ per capita per year in 32 low-income developing countries; and $175‡ per captia in 27 other low-income developing countries (required budget outlays)^37^
3.8.2: financial risk protection	<10% or <25% of total expenditure or income	..	..
**Target 3.9: by 2030, substantially reduce the number of deaths and illnesses from hazardous chemicals and air, water, and soil pollution and contamination**			
3.9.1: air pollution mortality	Undefined	..	$8·1 billion* per year in 67 low-income and middle-income countries^28^
3.9.2: WaSH mortality,	Undefined	..	..
3.9.3: poisoning mortality	Undefined	..	..
**Target 3.a: strengthen the implementation of the WHO Framework Convention on Tobacco Control in all countries, as appropriate**			
3.a.1: smoking prevalence	Undefined	DAH on tobacco control was $0·1 billion for 135 low-income and middle-income countries in 2019	
**Target 3.b: support the research and development of vaccines and medicines for communicable and non-communicable diseases that primarily affect developing countries; provide access to affordable essential medicines and vaccines, in accordance with the Doha Declaration on the TRIPS Agreement and Public Health, which affirms the right of developing countries to use to the full the provisions in the Agreement on TRIPS regarding flexibilities to protect public health, and, in particular, provide access to medicines for all**			
3.b.1: vaccine coverage	Coverage of all target populations (100%)	DAH on immunisation was $3·1 billion for 135 low-income and middle-income countries in 2019	$1·4 billion* per year in 67 low-income and middle-income countries^28^
3.b.2: developmental assistance for research and health	Undefined	DAH on health was $40·6 billion for 135 low-income and middle-income countries in 2019	..
3.b.3: essential medicines	Coverage of all target populations (100%)	DAH on immunisation was $3·1 billion for low-income and middle-income countries in 2019	$1·4 billion* per year in 67 low-income and middle-income countries^28^
**Target 3.c: substantially increase health financing and the recruitment, development, training, and retention of the health workforce in developing countries, especially in the least developed countries and small island developing states**			
3.c.1: health worker density	Undefined	DAH on human resources was $4·0 billion for 135 low-income and middle-income countries in 2019	$8·1 billion* per year in 67 low-income and middle-income countries^28^
**Target 3.d: strengthen the capacity of all countries, particularly developing countries, for early warning, risk reduction, and management of national and global health risks**			
3.d.1: international health regulation capacity	Undefined	DAH on human resources was $4·0 billion for 135 low-income and middle-income countries in 2019	$8·1 billion* per year in 67 low-income and middle-income countries^28^

Spending data are reported in inflation adjusted 2019 US$, unless otherwise indicated. Data for HIV/AIDS and tuberculosis are reported for 135 low-income and middle-income countries, for malaria are for 106 malaria-endemic countries, for universal health coverage for 195 countries and territories, and for DAH for each SDG3 indicator for 135 low-income and middle-income countries except malaria. Existing unofficial financing targets have been extracted from literature review. Low-income and middle-income countries are grouped as defined by 2019 World Bank classification. SDG=Sustainable Development Goal. DAH=development assistance for health. UNFPA=United Nations Population Fund. ICPD25=25th International Conference on Development. WaSH=water, sanitation, and hygiene. TRIPS=Trade-Related Aspects of Intellectual Property Rights.

* 2014 US$.

† 2017 US$.

‡ 2018 US$.

Globally, total health spending has increased since the start of the SDGs in 2015, reaching $7·9 trillion (95% UI **7·8**–8·0) in 2017, and is expected to increase to $11·0 trillion (10·7–11·2) by 2030, and $16·7 trillion (16·0–17·4) in 2050, although with substantial disparity across countries. In 2017, in low-income and middle-income countries, $20·2 billion (17·0–25·0) was spent on HIV/AIDS, $10·9 billion (10·3–11·8) was spent on tuberculosis, and in 106 malaria-endemic countries, $5·1 billion (4·9–5·4) was spent on malaria. DAH was estimated to be $40·6 billion in 2019, the most recent year for which data are available. Estimates of DAH in 2019were also available for the following SDG3 health areas: maternal health ($1·5 billion), neonatal and child health ($8·5 billion), HIV/AIDS ($9·5 billion), tuberculosis ($1·7 billion), malaria ($2·3 billion), non-communicable diseases ($735·0 million), family planning ($1·2 billion), tobacco control ($66·2 million), vaccine ($3·1 billion), and human resources ($4·0 billion). Spending for several SDG3 indicators, including hepatitis B incidence (3.3.4), substance abuse (3.5.1–5.2), road injuries (3.6.1), adolescent birth rate (3.7.2), and chemical and environmental pollution (3.9.1–9.3) do not have a large, comparable set of spending estimates for either development assistance or domestic spending and so are not included in these analyses.

In 2019, DAH for pandemic preparedness was estimated to be $374 million (<1% of total DAH). $2·4 billion (6%) of all DAH was for infectious diseases (other than HIV/AIDS, tuberculosis, and malaria) in 2019, but these funds were generally spent on treatment or disease focused efforts rather than pandemic preparedness more broadly. Despite DAH for pandemic preparedness being such a small fraction of total DAH, DAH for pandemic preparedness has grown faster than total DAH over the past 10 years. Since 2010, DAH for pandemic preparedness has more than doubled (increasing 8·1% annually from $185·8 million in 2010), while total DAH has increased by only 1·9% annually. The development agency that provided the most DAH for pandemic preparedness in 2019 was the WHO.

In 2017, global health spending per capita was $1048 (95% UI 1034–1062). Of this amount, 81·3% (80·7–81·8) was financed by domestic government and prepaid private health spending (table 2). Most health spending was in high-income countries, where health spending was $5307 (5262–5351) per capita in 2017, of which 86·0% (85·7–86·2) was government and prepaid private health spending. In 2017, spending in upper-middle-income countries was $487 (457–520) per capita and in lower-middle-income countries was $84 (76–93) per capita. Of $37 (36–39) spent per capita in low-income countries in 2017, 30·9% (28·5–33·6) was government and prepaid health spending.

**Table 2:** Total health spending, by World bank income group, and GBD super-region, 2017 and 2030

		**Health spending per capita, 2019 US$**		**Health spending per capita, 2019 purchasing-power parity-adjusted $**		**Total health spending per GDP, %**		**Total government health spending and prepaid private spending per total health spending, %**	
		2017	2030	2017	2030	2017	2030	2017	2030
**Global**		**1048 (1034–1062)**	**1285 (1257–1316)**	**1418 (1393–1445)**	**1816 (1766–1871)**	**9·7% (9·6–9·8)**	**10·5% (10·1–10·9)**	**81·3% (80·7–81·8)**	**82·9% (82·1–83·6)**
**World Bank income groups**									
High-income		5307 (5262–5351)	6596 (6482–6708)	5825 (5777–5872)	7265 (7147–7385)	12·2% (12·1–12·3)	14·0% (13·5–14·4)	86·0% (85·7–86·2)	87·8% (87·5–88·1)
Upper-middle-income		487 (457–520)	808 (740–885)	1053 (995–1118)	1701 (1571–1852)	5·7% (5·3–6·1)	6·8% (6·0–7·6)	66·9% (64·2–69·6)	73·0% (69·6–76·1)
Lower-middle-income		84 (76–93)	127 (114–141)	289 (261–322)	439 (391–496)	3·9% (3·5–4·3)	4·1% (3·6–4·6)	41·6% (36·9–46·1)	45·7% (40·1–51·0)
Low-income		37 (36–39)	45 (42–48)	119 (113–126)	141 (132–152)	5·3% (5·0–5·7)	4·8% (4·4–5·3)	30·9% (28·5–33·6)	36·9% (33·6–40·4)
**Central Europe, eastern Europe, and central Asia**		**538 (518–560)**	**700 (672–730)**	**1332 (1276–1390)**	**1726 (1656–1806)**	**5·9% (5·7–6·2)**	**6·4% (6·0–6·8)**	**65·9% (64·1–67·7)**	**68·6% (66·8–70·5)**
Central Asia									
	Armenia	403 (364–447)	538 (483–597)	966 (872–1070)	1287 (1156–1428)	9·7% (8·5–10·9)	9·7% (8·3–11·2)	16·0% (12·8–19·6)	17·7% (13·9–21·8)
	Azerbaijan	304 (267–343)	368 (321–418)	1268 (1115–1433)	1535 (1339–1747)	6·6% (5·8–7·4)	6·7% (5·6–8·0)	17·4% (13·6–22·0)	18·3% (13·5–23·8)
	Georgia	307 (267–354)	521 (449–606)	870 (757–1003)	1477 (1274–1718)	8·0% (6·9–9·3)	10·3% (8·2–12·9)	41·4% (34·5–48·5)	55·8% (48·6–63·0)
	Kazakhstan	292 (249–340)	344 (286–411)	949 (811–1105)	1118 (930–1339)	3·4% (2·9–3·9)	3·1% (2·5–3·9)	67·0% (60·2–73·1)	63·8% (55·2–71·5)
	Kyrgyzstan	82 (68–99)	99 (80–121)	256 (210–309)	307 (250–376)	6·6% (5·4–7·9)	7·1% (5·6–9·0)	38·9% (30·8–48·1)	44·3% (34·0–55·1)
	Mongolia	162 (139–188)	226 (191–267)	563 (484–653)	786 (664–929)	4·2% (3·6–4·9)	4·4% (3·6–5·4)	57·1% (50·0–63·9)	61·1% (53·6–68·0)
	Tajikistan	59 (48–74)	68 (54–86)	247 (200–305)	284 (224–358)	7·3% (5·9–9·0)	7·1% (5·4–9·3)	28·5% (20·5–37·3)	29·0% (20·3–39·5)
	Turkmenistan	585 (523–656)	768 (683–859)	1417 (1265–1588)	1858 (1654–2079)	8·1% (7·3–9·1)	7·7% (6·6–9·1)	26·7% (21·8–32·3)	25·5% (19·8–32·0)
	Uzbekistan	88 (72–106)	124 (101–151)	479 (390–577)	676 (548–823)	5·8% (4·4–7·4)	6·3% (4·4–8·6)	46·0% (36·6–56·0)	50·4% (40·4–60·1)
Central Europe									
	Albania	364 (312–428)	607 (516–725)	933 (799–1096)	1554 (1321–1856)	7·3% (6·3–8·7)	9·7% (7·8–12·0)	67·4% (57·5–75·4)	74·5% (66·5–81·2)
	Bosnia and Herzegovina	531 (474–590)	838 (741–953)	1325 (1182–1471)	2090 (1848–2378)	9·7% (8·6–10·9)	11·9% (10·1–13·8)	71·2% (66·5–76·0)	76·9% (72·2–80·8)
	Bulgaria	713 (657–774)	1161 (1052–1280)	1853 (1707–2012)	3018 (2733–3327)	8·1% (7·5–8·8)	9·6% (8·1–11·1)	52·8% (48·8–57·2)	59·9% (55·5–64·2)
	Croatia	900 (824–980)	1165 (1011–1330)	1680 (1539–1831)	2175 (1889–2484)	6·5% (5·9–7·1)	7·1% (6·0–8·3)	89·1% (86·5–91·3)	89·9% (87·4–92·0)
	Czech Republic	1585 (1515–1665)	2308 (2120–2529)	2694 (2575–2829)	3922 (3602–4298)	7·2% (6·6–7·9)	8·4% (6·9–10·5)	85·2% (83·5–86·7)	87·1% (85·3–88·7)
	Hungary	1107 (1042–1180)	1445 (1343–1559)	2157 (2030–2299)	2816 (2617–3039)	7·0% (6·5–7·4)	7·2% (6·5–8·0)	73·0% (70·6–75·3)	74·1% (71·4–76·7)
	Montenegro	672 (518–870)	877 (674–1143)	1555 (1198–2012)	2029 (1559–2645)	8·4% (6·4–10·8)	8·7% (6·5–11·4)	72·0% (62·2–81·1)	73·6% (62·8–82·7)
	North Macedonia	433 (342–550)	600 (466–759)	1170 (925–1487)	1623 (1259–2051)	7·6% (6·0–9·7)	8·8% (6·7–11·1)	67·9% (57·7–77·1)	71·7% (62·0–80·3)
	Poland	882 (827–945)	1381 (1274–1498)	2003 (1879–2145)	3135 (2894–3402)	6·5% (6·1–6·9)	7·5% (6·6–8·6)	77·1% (74·4–79·6)	80·1% (77·5–82·5)
	Romania	585 (535–641)	955 (819–1104)	1320 (1207–1446)	2155 (1849–2492)	5·1% (4·7–5·6)	5·9% (4·8–7·1)	79·0% (74·9–82·4)	81·9% (77·6–85·6)
	Serbia	465 (423–512)	666 (596–743)	1163 (1059–1281)	1666 (1492–1858)	7·0% (6·3–8·0)	7·3% (6·1–8·7)	58·2% (53·8–62·9)	59·7% (54·6–64·4)
	Slovakia	1249 (1184–1315)	1640 (1489–1798)	2336 (2214–2459)	3067 (2785–3364)	6·8% (6·5–7·2)	7·1% (6·1–8·2)	81·7% (79·5–83·8)	82·6% (79·9–85·2)
	Slovenia	2014 (1913–2120)	2621 (2471–2777)	2974 (2825–3130)	3870 (3649–4101)	8·3% (7·8–8·7)	9·2% (8·5–10·2)	87·8% (86·5–89·0)	88·9% (87·6–90·0)
Eastern Europe									
	Belarus	373 (330–422)	470 (387–576)	1173 (1038–1327)	1478 (1215–1813)	5·9% (5·2–6·7)	6·3% (5·0–7·9)	70·3% (64·5–75·4)	72·1% (64·8–78·8)
	Estonia	1400 (1338–1462)	1812 (1662–1970)	2164 (2069–2261)	2802 (2569–3045)	6·4% (6·1–6·7)	7·0% (6·2–7·9)	76·7% (74·8–78·5)	78·5% (76·2–80·7)
	Latvia	1005 (953–1061)	1278 (1186–1377)	1741 (1651–1839)	2213 (2054–2386)	6·0% (5·7–6·4)	6·3% (5·6–7·1)	57·7% (54·9–60·3)	59·1% (55·5–62·4)
	Lithuania	1139 (1081–1201)	1595 (1477–1713)	2171 (2062–2289)	3041 (2816–3267)	6·5% (6·1–6·8)	7·4% (6·4–8·5)	67·8% (65·3–70·1)	71·5% (68·6–73·9)
	Moldova	215 (184–250)	288 (245–340)	500 (428–583)	671 (570–792)	7·9% (5·9–11·1)	8·9% (5·8–14·6)	51·8% (44·6–59·5)	56·7% (48·1–64·6)
	Russia	574 (526–630)	681 (612–756)	1537 (1409–1688)	1825 (1639–2025)	5·3% (4·8–5·8)	5·4% (4·7–6·3)	59·9% (55·3–64·3)	61·1% (56·4–66·3)
	Ukraine	219 (187–255)	248 (212–294)	618 (527–719)	701 (599–828)	6·8% (5·8–8·0)	7·0% (5·7–8·7)	46·1% (39·2–53·7)	48·1% (40·7–56·6)
**High-income**		**5760 (5707–5808)**	**7106 (6973–7229)**	**6175 (6121–6225)**	**7597 (7460–7725)**	**12·6% (12·5–12·8)**	**14·5% (14·0–15·0)**	**86·2% (86·0–86·5)**	**88·0% (87·7–88·3)**
Australasia									
	Australia	5195 (5108–5280)	6003 (5868–6154)	5181 (5095–5266)	5987 (5852–6137)	9·9% (9·3–10·8)	10·6% (9·5–12·0)	81·8% (81·1–82·5)	83·3% (82·5–84·1)
	New Zealand	4068 (3970–4174)	4755 (4562–4936)	4066 (3969–4172)	4754 (4560–4934)	9·9% (9·6–10·3)	11·1% (10·2–11·9)	86·4% (85·4–87·2)	87·7% (86·7–88·6)
High-income Asia Pacific									
	Brunei	690 (634–750)	766 (637–902)	1919 (1764–2085)	2130 (1773–2509)	2·4% (2·2–2·6)	2·6% (2·1–3·2)	94·7% (93·3–95·8)	95·2% (93·6–96·4)
	Japan	4290 (4148–4438)	5321 (5037–5597)	4784 (4626–4950)	5934 (5617–6242)	10·7% (10·4–11·1)	12·0% (11·0–12·9)	87·1% (86·3–87·9)	88·6% (87·6–89·5)
	Singapore	2739 (2624–2873)	3698 (3314–4168)	4393 (4208–4608)	5931 (5314–6685)	4·5% (4·2–4·8)	5·6% (4·8–6·5)	67·7% (65·9–69·5)	73·7% (70·4–77·0)
	South Korea	2118 (2041–2205)	3384 (3118–3613)	2993 (2885–3116)	4782 (4406–5107)	7·2% (6·8–7·6)	10·2% (8·8–11·7)	66·5% (65·0–68·0)	74·6% (72·1–76·8)
High-income North America									
	Canada	4919 (4840–5003)	5601 (5451–5762)	5410 (5323–5501)	6159 (5994–6337)	10·7% (10·6–10·9)	12·1% (11·4–12·8)	85·8% (85·1–86·4)	87·3% (86·6–87·9)
	Greenland	6559 (6196–6981)	8140 (7578–8745)	4880 (4610–5195)	6057 (5638–6507)	11·8% (10·7–13·2)	13·1% (10·7–16·1)	100·0% (100·0–100·0)	100·0% (100·0–100·0)
	USA	10 243 (10 087–10 390)	12 734 (12 337–13 115)	10 243 (10 087–10 390)	12 734 (12 337–13 115)	16·4% (16·2–16·6)	19·1% (17·8–20·4)	88·5% (88·1–88·9)	90·3% (89·8–90·8)
Southern Latin America									
	Argentina	907 (830–987)	844 (731–970)	2006 (1837–2184)	1866 (1617–2147)	8·5% (7·7–9·2)	8·7% (7·2–10·5)	85·0% (82·3–87·5)	85·4% (82·2–88·3)
	Chile	1379 (1311–1460)	1829 (1712–1955)	2365 (2248–2504)	3136 (2937–3353)	9·2% (8·7–9·7)	10·7% (9·4–12·2)	66·0% (63·7–68·1)	70·4% (68·1–72·6)
	Uruguay	1582 (1497–1670)	2048 (1846–2271)	2218 (2099–2341)	2871 (2588–3184)	9·3% (8·8–9·8)	10·3% (8·8–12·1)	82·6% (80·9–84·3)	84·5% (82·3–86·5)
Western Europe									
	Andorra	4491 (4310–4675)	5125 (4766–5519)	9712 (9320–10 109)	11 083 (10 308–11 936)	7·9% (7·2–8·6)	9·4% (7·6–11·9)	58·6% (56·7–60·6)	63·3% (60·0–66·4)
	Austria	5062 (4941–5183)	5602 (5335–5873)	5391 (5263–5521)	5966 (5683–6255)	10·4% (10·1–10·6)	10·9% (10·1–11·7)	80·8% (80·0–81·5)	81·8% (80·7–82·8)
	Belgium	4595 (4475–4727)	5387 (5107–5686)	4995 (4865–5139)	5857 (5552–6182)	10·4% (10·1–10·7)	11·7% (10·9–12·6)	82·3% (81·4–83·2)	84·4% (83·3–85·5)
	Cyprus	1184 (1111–1261)	1452 (1350–1566)	1780 (1671–1897)	2184 (2031–2355)	5·1% (4·0–6·7)	5·5% (3·9–8·1)	54·9% (52·0–58·2)	58·7% (55·3–62·0)
	Denmark	5933 (5782–6079)	6537 (6302–6768)	5364 (5227–5496)	5911 (5698–6119)	10·1% (9·9–10·4)	10·6% (9·9–11·3)	86·3% (85·7–86·9)	87·1% (86·4–87·8)
	Finland	4386 (4253–4523)	4894 (4595–5181)	4298 (4168–4432)	4796 (4503–5077)	9·3% (9·0–9·6)	9·5% (8·8–10·3)	79·6% (78·6–80·6)	80·6% (79·1–82·0)
	France	4530 (4455–4600)	5127 (5026–5235)	5100 (5015–5178)	5772 (5658–5893)	11·4% (11·0–11·8)	12·1% (11·2–12·9)	90·6% (89·9–91·3)	91·1% (90·4–91·8)
	Germany	5110 (4991–5225)	6162 (5794–6512)	5864 (5727–5995)	7070 (6648–7472)	11·1% (10·9–11·4)	12·5% (11·0–14·1)	87·3% (86·7–87·9)	88·7% (87·8–89·6)
	Greece	1571 (1477–1676)	1836 (1664–2020)	2368 (2227–2526)	2768 (2509–3046)	8·3% (7·8–9·0)	8·7% (7·5–10·0)	64·5% (61·5–67·5)	66·8% (63·3–70·2)
	Iceland	5538 (5290–5805)	5656 (5264–6079)	4680 (4470–4905)	4780 (4448–5137)	8·3% (7·9–8·7)	8·5% (7·6–9·5)	83·4% (82·4–84·5)	83·5% (82·0–84·9)
	Ireland	4979 (4718–5249)	6150 (5670–6662)	5433 (5148–5728)	6711 (6187–7270)	7·0% (6·6–7·4)	7·0% (6·2–8·1)	87·6% (86·5–88·6)	87·8% (86·5–89·1)
	Israel	2961 (2864–3068)	3711 (3474–3960)	2710 (2620–2807)	3396 (3178–3623)	7·0% (6·6–7·4)	7·6% (6·6–8·6)	77·4% (76·0–78·7)	79·1% (77·4–80·8)
	Italy	2879 (2784–2971)	3130 (2927–3355)	3535 (3419–3649)	3844 (3594–4121)	8·8% (8·4–9·1)	9·2% (8·3–10·4)	76·7% (75·3–77·9)	78·0% (76·2–79·8)
	Luxembourg	6066 (5714–6448)	6708 (6057–7439)	5928 (5584–6301)	6555 (5918–7268)	5·4% (5·1–5·8)	6·0% (5·3–6·9)	89·2% (87·7–90·6)	90·2% (88·5–91·7)
	Malta	2831 (2731–2939)	4020 (3768–4277)	4353 (4199–4519)	6182 (5794–6577)	9·8% (9·2–10·4)	11·2% (9·9–12·7)	65·0% (63·3–66·6)	69·2% (67·1–71·5)
	Netherlands	5143 (4950–5341)	6023 (5611–6462)	5753 (5537–5974)	6738 (6277–7228)	10·2% (9·8–10·5)	10·8% (9·9–11·9)	88·8% (87·9–89·7)	89·4% (88·3–90·5)
	Norway	8102 (7841–8368)	9313 (8824–9819)	7959 (7703–8220)	9148 (8668–9646)	10·6% (10·3–11·0)	11·8% (10·6–12·9)	85·8% (85·0–86·7)	87·4% (86·4–88·3)
	Portugal	1889 (1797–1988)	2127 (1918–2371)	2744 (2610–2888)	3089 (2785–3444)	8·8% (8·2–9·6)	9·0% (7·7–10·5)	72·5% (70·5–74·2)	72·6% (69·4–75·9)
	Spain	2554 (2461–2657)	3110 (2950–3287)	3526 (3398–3668)	4293 (4073–4538)	8·9% (8·6–9·3)	9·9% (8·9–11·0)	76·4% (75·1–77·8)	78·4% (76·8–80·0)
	Sweden	5561 (5344–5766)	7007 (6544–7470)	5917 (5685–6135)	7455 (6962–7948)	11·0% (10·6–11·4)	12·7% (11·6–13·8)	84·9% (84·0–85·8)	87·3% (86·2–88·3)
	Switzerland	9903 (9669–10151)	11 319 (10796–11888)	7898 (7711–8095)	9027 (8610–9481)	12·1% (11·8–12·5)	13·5% (12·5–14·6)	70·9% (70·0–71·8)	73·8% (72·3–75·2)
	UK	3883 (3766–4004)	4623 (4350–4916)	4430 (4297–4569)	5275 (4963–5609)	9·6% (9·3–9·9)	10·9% (9·8–12·1)	84·0% (82·8–85·2)	86·0% (84·7–87·3)
**Latin America and Caribbean**		**589 (570–611)**	**704 (682–729)**	**1189 (1150–1234)**	**1423 (1377–1476)**	**7·4% (7·1–7·7)**	**8·1% (7·7–8·6)**	**69·6% (67·8–71·4)**	**72·8% (71·1–74·6)**
Andean Latin America									
	Bolivia	217 (184–258)	288 (242–346)	443 (375–525)	587 (493–705)	6·2% (5·3–7·4)	6·8% (5·6–8·2)	70·4% (62·9–77·2)	73·3% (66·5–79·7)
	Ecuador	524 (464–591)	565 (496–646)	996 (881–1124)	1074 (943–1229)	8·2% (7·2–9·2)	8·6% (7·3–10·1)	59·2% (53·9–64·6)	61·6% (55·8–67·5)
	Peru	330 (283–384)	434 (369–514)	687 (589–799)	903 (768–1069)	4·9% (4·2–5·7)	5·4% (4·3–6·6)	70·6% (64·7–76·3)	73·5% (67·0–79·4)
Caribbean									
	Antigua and Barbuda	668 (588–750)	909 (774–1065)	1063 (935–1194)	1447 (1232–1694)	4·2% (3·6–4·9)	4·8% (3·8–5·9)	62·0% (56·7–66·8)	65·6% (59·4–71·4)
	The Bahamas	1990 (1863–2113)	2144 (1969–2335)	1967 (1841–2088)	2119 (1946–2308)	6·2% (5·7–6·8)	6·7% (5·6–7·9)	70·8% (68·4–73·1)	71·2% (68·3–74·1)
	Barbados	1180 (1119–1246)	1066 (989–1152)	1224 (1160–1291)	1106 (1025–1195)	6·5% (6·1–7·0)	5·9% (5·2–6·5)	53·1% (50·6–55·6)	46·4% (42·5–50·5)
	Belize	287 (247–337)	344 (297–401)	505 (435–593)	605 (522–706)	5·7% (5·0–6·7)	5·9% (4·8–7·3)	73·7% (67·8–78·9)	74·6% (68·9–80·4)
	Bermuda	7027 (5973–8208)	8358 (6986–9870)	4430 (3765–5174)	5269 (4404–6222)	6·4% (4·7–9·4)	25·3% (5·0–18·6)	90·1% (86·9–92·8)	91·8% (89·1–94·0)
	Cuba	1208 (1129–1304)	1724 (1566–1899)	3262 (3050–3522)	4659 (4231–5131)	11·3% (10·1–12·8)	14·5% (11·7–18·7)	89·8% (87·6–91·7)	91·7% (89·7–93·3)
	Dominica	493 (445–550)	644 (536–774)	699 (631–780)	915 (761–1099)	6·6% (5·9–7·4)	6·5% (5·2–8·0)	68·3% (63·3–72·7)	67·5% (60·5–73·6)
	Dominican Republic	436 (383–493)	714 (610–828)	1037 (911–1174)	1698 (1450–1969)	5·7% (5·0–6·5)	6·6% (5·3–8·1)	54·5% (48·3–60·6)	60·3% (53·7–66·6)
	Grenada	528 (474–593)	642 (553–748)	772 (692–867)	937 (808–1093)	5·0% (4·5–5·6)	4·9% (4·1–6·0)	46·1% (40·6–52·0)	46·9% (39·7–54·8)
	Guyana	258 (226–299)	621 (485–793)	456 (399–528)	1097 (855–1400)	5·3% (4·6–6·1)	6·4% (4·8–8·2)	62·6% (55·7–69·1)	70·1% (61·6–78·1)
	Haiti	48 (40–57)	50 (42–59)	117 (99–139)	122 (102–145)	6·0% (5·1–7·2)	6·0% (5·0–7·3)	19·3% (14·2–25·3)	17·3% (11·9–23·6)
	Jamaica	329 (280–389)	395 (322–482)	583 (497–690)	700 (571–855)	6·2% (5·3–7·3)	7·0% (5·6–8·8)	79·9% (74·5–84·5)	81·5% (76·0–86·2)
	Puerto Rico	1276 (1101–1487)	1742 (1499–2034)	1611 (1390–1878)	2199 (1892–2568)	4·1% (3·4–5·0)	5·4% (4·4–6·7)	77·4% (69·9–83·6)	81·9% (75·5–87·2)
	Saint Lucia	549 (494–609)	685 (595–781)	743 (668–824)	926 (805–1056)	5·0% (4·5–5·6)	5·6% (4·8–6·6)	51·0% (45·9–56·1)	56·6% (50·1–62·8)
	Saint Vincent and the Grenadines	335 (293–382)	439 (377–507)	532 (465–606)	696 (599–806)	4·4% (3·9–5·1)	5·0% (4·2–5·9)	65·5% (59·7–71·7)	67·8% (61·1–73·9)
	Suriname	414 (356–477)	526 (439–637)	1044 (899–1203)	1328 (1107–1606)	6·6% (5·6–7·6)	7·7% (6·1–9·5)	73·4% (67·8–78·7)	75·9% (69·9–81·2)
	Trinidad and Tobago	1117 (1042–1202)	1400 (1229–1577)	2247 (2096–2419)	2817 (2473–3174)	6·8% (6·3–7·3)	8·1% (6·9–9·5)	59·2% (56·0–62·3)	64·5% (59·6–69·1)
	Virgin Islands	1696 (1377–2117)	2011 (1556–2585)	1696 (1377–2117)	2011 (1556–2585)	4·2% (3·4–5·4)	4·8% (3·5–6·7)	75·6% (67·3–83·3)	78·0% (69·5–84·9)
Central Latin America									
	Colombia	481 (416–555)	709 (605–827)	1147 (992–1325)	1691 (1445–1973)	7·6% (6·6–8·8)	9·3% (7·8–11·2)	83·6% (79·0–87·1)	86·2% (82·3–89·3)
	Costa Rica	944 (869–1026)	1189 (1045–1352)	1408 (1296–1530)	1773 (1559–2017)	8·1% (7·4–8·8)	8·9% (7·6–10·3)	78·2% (75·0–81·4)	80·3% (76·5–83·5)
	El Salvador	315 (275–366)	411 (355–482)	650 (568–756)	850 (733–996)	8·0% (6·9–9·4)	9·0% (7·5–10·7)	69·8% (63·9–75·4)	73·5% (68·3–78·7)
	Guatemala	265 (227–311)	322 (270–382)	493 (424–580)	600 (504–712)	5·9% (5·0–6·9)	6·0% (5·0–7·3)	44·3% (36·3–52·8)	48·0% (39·9–57·1)
	Honduras	185 (155–218)	229 (189–275)	387 (324–457)	479 (396–576)	7·6% (6·3–9·0)	7·7% (6·3–9·3)	47·9% (39·1–57·0)	50·9% (41·9–59·6)
	Mexico	562 (502–629)	641 (569–721)	1158 (1035–1297)	1322 (1172–1486)	5·7% (5·0–6·4)	6·1% (5·2–7·2)	59·0% (54·0–63·8)	62·4% (57·0–67·5)
	Nicaragua	188 (161–222)	210 (180–247)	516 (441–608)	576 (492–677)	8·7% (7·4–10·3)	10·4% (8·5–12·6)	60·3% (52·5–67·7)	64·3% (56·8–70·9)
	Panama	1147 (1067–1235)	1588 (1429–1766)	1883 (1752–2028)	2608 (2346–2899)	7·4% (6·9–8·0)	7·7% (6·6–8·9)	68·6% (65·6–71·7)	69·2% (65·1–73·0)
	Venezuela	107 (89–127)	80 (64–101)	555 (466–663)	417 (334–526)	2·2% (1·8–2·8)	2·1% (1·6–2·8)	55·1% (47·0–63·1)	53·9% (43·2–64·3)
Tropical Latin America									
	Brazil	799 (766–834)	942 (909–978)	1505 (1443–1570)	1774 (1712–1841)	9·2% (8·8–9·6)	10·1% (9·2–11·2)	72·4% (69·3–75·3)	75·6% (72·8–78·2)
	Paraguay	389 (338–453)	528 (451–619)	937 (813–1091)	1271 (1087–1490)	7·6% (6·0–10·0)	8·8% (6·5–12·6)	55·8% (49·2–62·6)	61·1% (54·8–67·4)
**North Africa and Middle East**		**353 (339–367)**	**426 (404–451)**	**1055 (1012–1104)**	**1263 (1198–1337)**	**5·3% (5·1–5·5)**	**6·4% (6·0–6·9)**	**68·0% (66·2–69·8)**	**72·6% (70·8–74·4)**
Afghanistan		50 (37–65)	53 (40–68)	203 (154–265)	219 (165–281)	9·9% (7·5–13·0)	9·2% (6·6–12·5)	6·7% (4·5–9·1)	7·9% (5·5–11·1)
Algeria		265 (232–304)	297 (249–350)	988 (866–1133)	1106 (928–1304)	6·6% (5·8–7·6)	7·3% (6·1–8·8)	69·1% (62·3–75·0)	72·0% (65·6–78·1)
Bahrain		1230 (1169–1292)	1386 (1287–1483)	2422 (2300–2543)	2728 (2534–2920)	4·9% (4·7–5·2)	5·9% (5·0–6·9)	70·6% (68·2–73·1)	74·0% (71·0–76·8)
Egypt		147 (121–178)	189 (153–228)	675 (552–815)	863 (702–1044)	5·5% (4·3–6·7)	5·3% (4·0–6·7)	39·6% (30·3–49·7)	38·4% (28·7–49·1)
Iran		555 (495–618)	580 (516–648)	1763 (1574–1966)	1845 (1642–2061)	8·1% (7·2–9·3)	10·1% (8·2–12·3)	58·7% (53·4–64·4)	65·2% (59·8–70·4)
Iraq		195 (166–230)	258 (214–306)	609 (520–719)	808 (670–958)	3·4% (2·9–4·0)	4·0% (3·1–5·1)	32·4% (26·1–39·0)	41·3% (32·4–49·9)
Jordan		293 (251–344)	348 (291–427)	643 (551–754)	764 (639–938)	6·7% (5·8–7·9)	7·2% (5·8–8·8)	66·9% (60·1–73·3)	69·0% (62·1–75·6)
Kuwait		1556 (1400–1725)	1630 (1391–1890)	3640 (3277–4036)	3815 (3256–4422)	4·9% (4·2–5·5)	5·5% (4·3–7·0)	85·9% (83·6–87·8)	86·4% (83·5–88·9)
Lebanon		935 (847–1028)	1123 (962–1301)	1481 (1341–1628)	1777 (1523–2059)	10·2% (8·8–11·9)	10·6% (8·2–13·6)	66·5% (62·2–70·7)	68·6% (63·1–73·7)
Libya		466 (392–548)	436 (348–546)	821 (690–966)	768 (614–963)	9·5% (3·6–20·4)	11·9% (3·8–23·1)	73·2% (66·4–78·8)	78·0% (71·0–84·2)
Morocco		171 (145–205)	225 (187–270)	471 (398–564)	620 (516–742)	5·3% (4·4–6·3)	5·7% (4·6–7·0)	44·4% (36·3–53·6)	47·6% (38·9–57·0)
Oman		664 (624–711)	626 (549–731)	1702 (1600–1822)	1606 (1406–1875)	4·0% (3·5–4·4)	4·0% (3·1–5·0)	93·7% (92·0–95·0)	93·1% (91·1–94·7)
Palestine		365 (300–436)	492 (393–615)	139 (115–167)	188 (150–235)	10·9% (8·9–13·0)	12·1% (9·5–15·5)	57·0% (48·1–65·9)	63·3% (53·6–71·7)
Qatar		1958 (1780–2155)	3458 (2762–4411)	3750 (3410–4128)	6624 (5291–8451)	2·8% (2·6–3·1)	5·0% (3·9–6·4)	91·9% (90·3–93·3)	94·4% (92·6–95·9)
Saudi Arabia		1206 (1107–1310)	1786 (1577–2080)	3046 (2796–3307)	4511 (3983–5253)	5·2% (4·8–5·7)	8·0% (6·7–9·7)	83·8% (81·7–85·8)	88·8% (86·9–90·7)
Sudan		54 (43–68)	56 (45–70)	315 (250–395)	327 (261–407)	5·8% (4·2–8·1)	6·4% (4·3–9·4)	26·5% (18·9–35·7)	30·5% (21·5–40·7)
Syria		57 (46–70)	75 (59–91)	922 (744–1133)	1210 (958–1478)	5·7% (4·3–7·4)	5·9% (4·3–8·3)	45·9% (35·8–56·6)	48·0% (36·9–58·9)
Tunisia		238 (203–280)	326 (278–383)	916 (782–1078)	1253 (1070–1472)	7·4% (6·3–8·7)	9·1% (7·6–10·9)	60·6% (52·4–68·2)	66·5% (59·1–73·9)
Turkey		379 (333–429)	486 (403–587)	1228 (1079–1392)	1578 (1307–1904)	4·2% (3·7–4·8)	4·8% (3·9–5·8)	82·2% (77·5–86·3)	84·4% (79·5–88·2)
United Arab Emirates		1485 (1380–1600)	1925 (1633–2240)	2630 (2444–2834)	3408 (2892–3966)	3·7% (3·3–4·1)	5·2% (4·1–6·5)	81·1% (78·3–83·7)	84·9% (81·5–88·0)
Yemen		50 (42–62)	58 (48–70)	123 (101–150)	141 (116–171)	5·4% (3·8–7·5)	5·8% (3·9–8·6)	9·6% (7·1–12·4)	17·6% (13·1–23·5)
**South Asia**		**62 (51–77)**	**104 (85–130)**	**236 (192–291)**	**396 (321–493)**	**3·4% (2·8–4·2)**	**3·6% (2·8–4·5)**	**35·3% (26·2–45·4)**	**39·6% (29·4–49·4)**
Bangladesh		40 (31–52)	68 (52–87)	107 (83–136)	178 (138–231)	2·5% (1·9–3·2)	2·5% (1·8–3·4)	21·1% (14·9–27·9)	21·4% (15·3–28·3)
Bhutan		108 (90–129)	144 (109–185)	316 (262–378)	420 (320–542)	3·2% (2·6–3·9)	2·8% (2·0–3·7)	79·2% (72·9–84·4)	77·1% (68·4–83·9)
India		69 (54–87)	119 (94–152)	265 (209–336)	456 (360–583)	3·5% (2·8–4·5)	3·7% (2·8–4·8)	36·5% (25·6–48·0)	41·1% (29·3–52·4)
Nepal		50 (40–63)	79 (62–100)	162 (128–204)	255 (199–321)	5·5% (4·3–7·0)	5·6% (4·1–7·4)	31·6% (22·7–42·4)	35·7% (25·7–47·2)
Pakistan		37 (29–46)	49 (38–62)	159 (126–199)	211 (164–267)	2·8% (2·2–3·5)	2·9% (2·2–3·6)	33·0% (23·5–43·5)	36·1% (25·4–47·2)
**Southeast Asia, east Asia, and Oceania**		**365 (329–406)**	**730 (645–825)**	**757 (686–839)**	**1499 (1336–1683)**	**4·9% (4·4–5·5)**	**6·3% (5·3–7·4)**	**64·2% (59·7–68·8)**	**72·4% (67·6–76·7)**
East Asia									
	China	455 (400–517)	984 (850–1132)	875 (769–994)	1891 (1634–2176)	5·1% (4·4–5·7)	6·6% (5·4–8·0)	64·5% (59·2–69·8)	73·4% (67·8–78·2)
	North Korea	77 (60–96)	87 (68–110)	45 (35–56)	51 (39–64)	5·6% (4·3–7·0)	5·9% (4·4–7·8)	63·8% (52·5–73·6)	66·7% (55·5–76·0)
	Taiwan (province of China)	1477 (1312–1677)	1903 (1677–2172)	3270 (2905–3711)	4213 (3713–4808)	6·2% (5·5–7·1)	6·9% (5·7–8·4)	69·0% (64·9–73·7)	72·2% (67·9–76·5)
Oceania									
	American Samoa	694 (572–841)	711 (573–869)	694 (572–841)	711 (573–869)	5·5% (4·5–6·8)	6·0% (4·7–7·8)	78·4% (71·0–85·0)	79·7% (72·2–86·3)
	Federated States of Micronesia	141 (119–166)	224 (185–270)	136 (115–160)	215 (178–259)	4·1% (3·3–5·0)	5·9% (4·4–7·8)	84·3% (80·7–87·5)	88·9% (85·8–91·5)
	Fiji	198 (160–243)	255 (200–322)	356 (288–438)	459 (360–580)	3·5% (2·7–4·5)	3·8% (2·8–5·0)	78·6% (71·7–84·5)	79·5% (72·5–85·5)
	Guam	1468 (1143–1903)	1607 (1236–2088)	1468 (1143–1903)	1607 (1236–2088)	3·7% (2·9–4·9)	4·1% (3·1–5·8)	75·1% (65·2–83·2)	77·2% (68·4–84·6)
	Kiribati	214 (188–246)	234 (195–282)	290 (255–334)	317 (264–382)	14·1% (12·2–16·3)	15·0% (12·0–18·6)	59·3% (52·9–65·3)	61·2% (53·0–68·7)
	Marshall Islands	416 (370–472)	554 (468–648)	408 (362–462)	544 (459–636)	11·1% (9·6–12·9)	12·9% (10·4–15·8)	67·2% (62·3–71·9)	71·5% (65·9–76·7)
	Northern Mariana Islands	752 (585–992)	806 (614–1071)	752 (585–992)	806 (614–1071)	2·4% (1·9–3·2)	2·9% (2·1–4·1)	73·4% (63·2–81·8)	76·1% (66·8–84·1)
	Papua New Guinea	54 (44–64)	71 (57–87)	82 (67–98)	108 (87–133)	2·1% (1·6–2·5)	2·6% (1·9–3·3)	73·5% (67·6–78·7)	78·0% (72·1–83·0)
	Samoa	222 (194–254)	248 (209–293)	298 (259–339)	332 (280–393)	5·0% (4·3–5·7)	5·3% (4·2–6·4)	78·7% (74·2–82·8)	79·6% (74·3–84·2)
	Solomon Islands	119 (103–135)	127 (95–175)	119 (103–135)	128 (95–175)	5·6% (4·8–6·5)	5·4% (3·8–7·6)	60·9% (55·2–65·8)	60·6% (48·7–72·7)
	Tonga	218 (196–243)	277 (235–329)	291 (261–325)	369 (314–439)	5·1% (4·4–5·8)	5·0% (3·9–6·3)	65·4% (61·2–69·5)	69·3% (63·5–74·7)
	Vanuatu	98 (82–118)	117 (89–154)	89 (74–107)	107 (81–140)	3·0% (2·5–3·6)	3·1% (2·3–4·1)	67·7% (61·5–73·4)	70·5% (61·5–78·7)
Southeast Asia									
	Cambodia	83 (67–101)	129 (103–157)	236 (190–288)	368 (294–448)	5·7% (4·6–6·9)	5·8% (4·5–7·3)	25·7% (19·0–33·6)	28·4% (20·6–37·9)
	Indonesia	120 (94–152)	215 (166–277)	405 (316–511)	722 (558–931)	3·2% (2·5–4·0)	4·0% (3·0–5·2)	60·8% (49·1–72·0)	68·7% (58·5–77·6)
	Laos	58 (48–70)	83 (67–103)	173 (143–210)	247 (201–309)	2·4% (1·9–3·1)	2·3% (1·6–3·4)	36·9% (28·8–46·7)	36·5% (27·4–46·5)
	Malaysia	409 (353–475)	673 (575–790)	1190 (1029–1382)	1960 (1674–2299)	3·9% (3·4–4·6)	4·9% (4·1–5·9)	62·5% (56·6–68·7)	69·0% (63·3–75·1)
	Maldives	988 (905–1081)	1638 (1430–1884)	1479 (1355–1618)	2452 (2141–2821)	8·1% (6·4–10·1)	10·6% (7·4–14·8)	79·7% (76·5–82·7)	83·9% (80·4–86·9)
	Mauritius	606 (541–677)	969 (855–1106)	1309 (1170–1462)	2094 (1846–2389)	5·7% (5·1–6·4)	6·9% (5·9–8·2)	50·9% (45·7–56·3)	57·9% (52·2–63·5)
	Myanmar	52 (41–66)	87 (68–111)	279 (220–357)	470 (365–597)	4·4% (3·4–5·6)	4·4% (3·3–5·9)	16·3% (11·4–22·1)	18·6% (11·5–27·0)
	Philippines	133 (107–168)	204 (165–256)	374 (300–472)	573 (463–719)	4·4% (3·5–5·6)	4·8% (3·8–6·2)	44·5% (34·6–55·8)	50·0% (39·3–61·6)
	Sri Lanka	152 (124–182)	204 (164–247)	534 (437–641)	718 (579–871)	3·9% (3·2–4·8)	4·1% (3·2–5·1)	48·8% (39·4–58·4)	49·2% (38·9–59·2)
	Seychelles	754 (697–817)	992 (846–1161)	1394 (1288–1510)	1834 (1563–2146)	4·6% (4·3–5·0)	5·0% (4·2–6·0)	74·3% (70·8–77·8)	76·7% (72·2–81·3)
	Thailand	271 (229–326)	388 (326–465)	702 (593–843)	1005 (844–1205)	3·8% (3·2–4·5)	4·4% (3·6–5·4)	88·1% (84·1–91·4)	89·7% (86·1–92·7)
	Timor-Leste	86 (69–106)	114 (89–144)	197 (158–243)	261 (204–331)	3·6% (2·9–4·4)	4·0% (3·0–5·3)	72·5% (66·0–78·6)	77·7% (71·0–83·6)
	Vietnam	135 (111–164)	208 (167–257)	399 (327–484)	614 (492–758)	5·5% (4·5–6·7)	5·6% (4·3–7·3)	52·6% (43·5–62·7)	53·6% (43·7–64·5)
**Sub-Saharan Africa**		**81 (75–87)**	**92 (85–99)**	**204 (190–218)**	**232 (216–250)**	**5·2% (4·8–5·6)**	**5·1% (4·7–5·6)**	**54·0% (50·6–57·2)**	**55·8% (52·0–59·5)**
Central sub-Saharan Africa									
	Angola	90 (73–112)	82 (64–105)	205 (166–254)	186 (145–238)	2·9% (2·3–3·6)	2·7% (2·0–3·5)	62·6% (52·7–72·2)	58·6% (46·8–70·2)
	Central African Republic	21 (18–23)	26 (22–31)	36 (32–41)	45 (38–54)	4·7% (4·0–5·6)	4·7% (3·6–6·0)	14·6% (10·9–19·1)	21·0% (12·7–32·0)
	Congo (Brazzaville)	59 (48–71)	64 (49–84)	175 (143–210)	191 (147–248)	2·7% (2·0–3·5)	2·6% (1·7–3·7)	46·8% (36·3–56·2)	44·4% (32·7–56·9)
	Democratic Republic of the Congo	19 (16–22)	21 (17–26)	30 (26–36)	35 (28–42)	3·7% (3·0–4·5)	3·7% (2·6–4·9)	20·5% (13·6–29·5)	24·2% (16·1–34·9)
	Equatorial Guinea	299 (257–345)	352 (304–409)	711 (611–821)	837 (723–972)	2·8% (2·4–3·2)	3·1% (2·3–4·1)	20·9% (16·1–26·9)	29·2% (22·7–37·5)
	Gabon	289 (253–338)	386 (328–460)	702 (612–819)	936 (795–1115)	3·6% (3·2–4·2)	4·3% (3·5–5·3)	65·6% (59·6–71·4)	70·6% (64·3–76·3)
Eastern sub-Saharan Africa									
	Burundi	30 (26–36)	31 (26–39)	70 (60–84)	74 (60–93)	9·0% (7·5–10·9)	9·4% (7·4–12·0)	35·5% (26·3–46·7)	37·7% (26·4–50·7)
	Comoros	74 (60–91)	85 (70–104)	150 (122–185)	173 (142–211)	5·2% (4·0–6·5)	5·3% (4·0–7·0)	14·9% (10·4–20·4)	19·5% (13·3–26·8)
	Djibouti	57 (48–69)	63 (51–79)	104 (87–124)	113 (92–142)	2·5% (1·7–3·6)	2·4% (1·5–3·7)	56·8% (47·8–65·0)	55·6% (44·6–66·2)
	Eritrea	24 (20–30)	33 (26–40)	33 (27–41)	44 (35–54)	5·2% (2·7–10·2)	5·8% (2·3–17·2)	21·9% (16·0–28·8)	26·8% (17·9–37·1)
	Ethiopia	31 (25–38)	43 (34–57)	81 (67–101)	114 (89–149)	4·0% (3·1–5·1)	3·3% (2·3–4·7)	39·4% (28·8–51·5)	45·8% (33·3–58·9)
	Kenya	96 (78–120)	126 (100–161)	185 (150–230)	243 (192–310)	5·3% (4·3–6·7)	5·1% (3·9–6·7)	54·1% (43·3–63·5)	60·1% (49·6–69·8)
	Madagascar	22 (18–27)	28 (22–36)	81 (65–100)	102 (80–130)	4·8% (3·7–6·0)	5·3% (3·9–7·1)	58·1% (48·7–67·8)	64·7% (54·5–74·1)
	Malawi	45 (41–51)	46 (40–55)	151 (136–169)	154 (132–184)	11·9% (10·5–13·7)	9·7% (7·8–12·3)	29·1% (22·5–37·2)	34·3% (25·5–44·4)
	Mozambique	34 (32–36)	36 (32–42)	91 (85–98)	98 (85–114)	6·9% (6·4–7·4)	5·1% (4·2–6·2)	23·7% (19·5–28·9)	35·5% (27·4–44·4)
	Rwanda	45 (39–53)	60 (46–78)	133 (114–157)	175 (136–229)	6·2% (5·3–7·4)	5·3% (3·9–7·3)	46·2% (37·4–54·9)	63·8% (53·8–73·5)
	Somalia	6 (5–7)	6 (5–8)	14 (12–16)	14 (12–17)	4·7% (3·9–5·6)	4·7% (3·8–5·9)	19·3% (14·7–24·9)	19·3% (14·2–25·0)
	South Sudan	32 (29–35)	29 (25–33)	217 (201–237)	197 (173–229)	9·7% (6·1–13·2)	7·5% (3·3–11·9)	14·5% (10·9–19·0)	18·5% (15·2–22·1)
	Tanzania	43 (38–49)	50 (41–61)	129 (115–147)	151 (124–185)	4·2% (3·5–5·1)	3·8% (2·7–5·7)	35·7% (29·0–43·5)	44·7% (34·7–55·0)
	Uganda	44 (37–51)	54 (44–64)	149 (127–173)	184 (151–219)	6·5% (5·4–7·8)	5·9% (4·5–7·6)	19·5% (13·9–25·9)	30·1% (21·6–40·2)
	Zambia	66 (58–76)	67 (55–83)	210 (182–241)	212 (173–264)	5·0% (4·3–5·8)	4·7% (3·7–6·0)	40·8% (33·2–49·1)	44·0% (33·2–56·0)
Southern sub-Saharan Africa									
	Botswana	449 (393–516)	738 (633–859)	1017 (890–1170)	1671 (1433–1946)	5·9% (5·1–6·8)	7·5% (6·1–9·1)	89·6% (87·8–91·4)	93·0% (91·3–94·4)
	eSwatini	289 (249–340)	327 (270–399)	773 (666–909)	873 (722–1067)	7·1% (6·1–8·3)	6·9% (5·6–8·5)	59·2% (52·5–65·7)	58·9% (50·7–67·7)
	Lesotho	129 (113–147)	172 (148–199)	336 (296–383)	448 (387–519)	10·5% (9·0–12·3)	12·0% (9·6–15·0)	53·4% (47·2–59·6)	58·4% (51·1–65·0)
	Namibia	553 (488–629)	630 (546–729)	1100 (971–1252)	1253 (1085–1450)	9·4% (8·2–10·9)	10·1% (8·2–12·4)	83·8% (80·6–86·6)	84·7% (81·2–87·5)
	South Africa	533 (466–612)	673 (573–793)	1195 (1044–1372)	1509 (1285–1777)	8·6% (7·5–9·9)	10·5% (8·9–12·4)	89·9% (87·2–92·2)	91·4% (89·0–93·4)
	Zimbabwe	75 (62–92)	71 (56–91)	285 (235–349)	270 (213–347)	9·0% (6·5–12·8)	8·3% (5·5–12·8)	54·1% (44·9–63·2)	51·1% (39·1–62·8)
Western sub-Saharan Africa									
	Benin	35 (29–41)	41 (33–51)	97 (81–116)	114 (92–143)	3·7% (2·6–4·9)	3·2% (2·1–4·8)	27·8% (20·4–36·9)	33·0% (24·2–43·7)
	Burkina Faso	41 (33–49)	55 (43–68)	116 (95–141)	156 (122–194)	6·0% (5·0–7·3)	6·0% (4·7–7·6)	47·3% (36·7–57·3)	54·7% (43·9–65·4)
	Cameroon	60 (48–75)	75 (59–95)	158 (125–198)	196 (155–248)	4·1% (3·2–5·1)	3·9% (3·0–5·0)	15·9% (10·8–22·2)	17·9% (11·5–26·0)
	Cape Verde	162 (135–194)	237 (191–292)	349 (291–417)	509 (411–627)	4·8% (4·0–5·7)	5·3% (4·2–6·5)	67·1% (58·8–74·3)	70·8% (61·8–77·9)
	Chad	29 (23–37)	32 (25–41)	83 (66–106)	93 (72–118)	3·9% (2·8–5·3)	3·7% (2·5–5·1)	24·9% (17·5–33·3)	27·3% (18·1–38·7)
	Côte d'Ivoire	74 (58–92)	98 (76–128)	195 (153–241)	259 (200–336)	4·8% (3·8–5·9)	4·6% (3·4–6·0)	44·5% (33·1–57·1)	49·3% (36·5–61·5)
	The Gambia	43 (40–47)	42 (37–49)	162 (151–176)	158 (138–183)	7·1% (5·1–10·6)	5·3% (3·3–8·9)	20·3% (16·1–25·9)	20·5% (15·7–26·7)
	Ghana	66 (53–81)	111 (87–138)	208 (168–254)	349 (275–434)	3·7% (2·7–5·3)	4·4% (2·9–6·6)	46·5% (36·0–57·0)	58·8% (47·9–69·5)
	Guinea	45 (36–55)	60 (47–77)	110 (90–135)	149 (116–188)	4·4% (3·4–5·8)	4·2% (3·0–5·9)	23·7% (15·6–33·5)	29·7% (19·6–41·7)
	Guinea-Bissau	60 (49–73)	71 (56–89)	142 (116–172)	168 (132–211)	8·3% (6·7–10·3)	7·9% (6·1–10·2)	10·5% (6·9–15·0)	14·1% (8·7–20·6)
	Liberia	65 (56–75)	74 (62–88)	130 (112–151)	148 (124–177)	10·6% (7·6–15·3)	11·2% (7·1–18·7)	17·8% (12·2–25·2)	20·7% (13·1–30·6)
	Mali	32 (27–37)	38 (31–46)	85 (73–100)	102 (83–125)	3·4% (2·6–4·3)	3·2% (2·2–4·3)	31·1% (24·1–39·1)	40·2% (29·8–51·7)
	Mauritania	61 (50–74)	80 (65–99)	213 (175–257)	279 (226–345)	5·0% (4·0–6·2)	4·8% (3·6–6·2)	40·5% (31·1–50·2)	43·0% (33·1–54·0)
	Niger	26 (21–33)	33 (26–41)	69 (56–86)	87 (68–108)	6·8% (5·5–8·5)	7·0% (5·3–9·0)	30·6% (22·3–39·9)	40·5% (30·2–51·2)
	Nigeria	78 (62–96)	85 (68–107)	212 (170–262)	233 (185–291)	3·5% (2·8–4·3)	3·4% (2·6–4·4)	16·0% (11·2–21·6)	17·4% (11·2–25·5)
	São Tomé and PrÍncipe	113 (100–128)	136 (103–181)	184 (162–208)	221 (167–295)	5·8% (5·0–6·6)	5·6% (4·1–7·6)	46·3% (39·3–52·9)	50·5% (37·3–65·0)
	Senegal	65 (55–78)	76 (62–92)	170 (142–203)	197 (161–240)	4·8% (4·0–5·8)	4·4% (3·5–5·4)	29·2% (22·1–37·2)	30·8% (22·4–41·3)
	Sierra Leone	70 (58–84)	82 (67–101)	223 (185–268)	260 (213–322)	13·5% (11·2–16·3)	12·5% (9·8–16·0)	21·1% (14·1–29·9)	22·1% (14·2–31·9)
	Togo	41 (33–51)	54 (43–69)	111 (89–137)	147 (116–186)	6·4% (5·1–7·9)	6·6% (5·2–8·5)	26·3% (18·2–35·8)	34·8% (24·5–46·0)

Estimates in parentheses are 95% uncertainty intervals. Venezuela estimates are presented as 2014 US$. GBD=Global Burden of Diseases, Injuries, and Risk Factors study. GDP=Gross Domestic Product.

**Figure 1:**
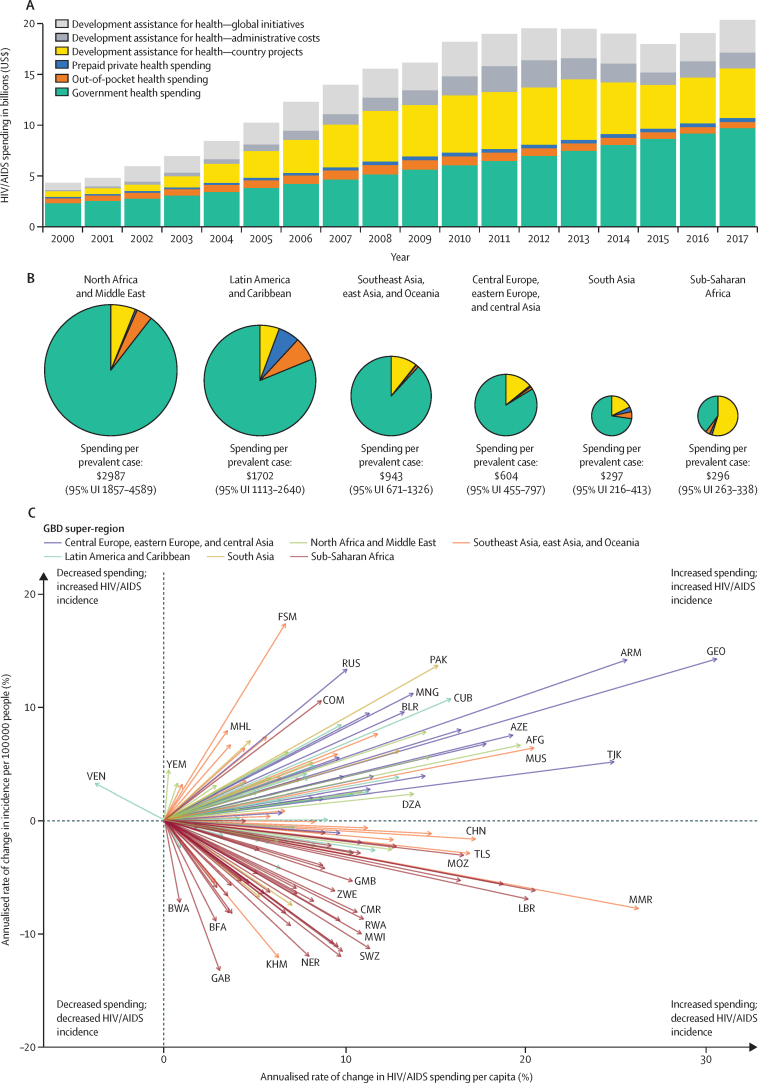
HIV/AIDS spending in low-income and middle-income countries (A) Total spending on HIV/AIDS by financing source, 2000 to 2017. (B) Breakdown of financing sources of HIV/AIDS spending and total HIV/AIDS spending per prevalent case, by GBD super-region, in 2017 with pie size proportional to spending per prevalent case of HIV/AIDS. (C) Annualised rates of change in HIV/AIDS prevalence and HIV/AIDS spending per capita, with each arrow showing one country moving from 2000 to 2017. Data are from all World Bank low-income and middle-income countries and spending estimates are presented in 2019 $US. Venezuela's spending is presented in 2014 $US. Administrative expenses that are only shown in panel A and reflect the operational expense of deploying the grant that is accrued in the donor country (eg, salaries of headquarters office staff). AFG=Afghanistan. ARM=Armenia. AZE=Azerbaijan. BFA=Burkina Faso. BLR=Belarus. BWA=Botswana. CHN=China. CMR=Cameroon. COM=Comoros. CUB=Cuba. DZA=Algeria. FSM=Federated States of Micronesia. GAB=Gabon. GBD=Global Burden of Diseases, Injuries, and Risk Factors study. GEO=Georgia. GMB=The Gambia. KHM=Cambodia. LBR=Liberia. MHL=Marshall Islands. MMR=Myanmar. MNG=Mongolia. MOZ=Mozambique. MUS=Mauritius MWI=Malawi. NER=Niger. PAK=Pakistan. RUS=Russia. RWA=Rwanda. SWZ=eSwatini. TJK=Tajikistan. TLS=Timor-Leste. VEN=Venezuela. YEM=Yemen. ZWE=Zimbabwe.

Total HIV/AIDS spending disaggregated by financing source in 135 low-income and middle-income countries for 2000–17 is shown in figure 1A. For these countries, which included 93·9% (95% UI 91·2–96·3) of the global HIV incidence and 98·3% (98·2–98·4) of global HIV/AIDS deaths in 2017, total spending on HIV/AIDS was $4·3 billion (3·2–5·9) in 2000 and increased to $20·2 billion (17·0–25·0) in 2017, increasing at an annualised rate of 9·62% (8·86–10·35) between 2000 and 2017.^23^, ^38^ Between 2000 and 2010, DAH for HIV/AIDS increased the fastest of all financing sources, growing at an annualised rate of 22·12%, although this annualised growth rate decreased to −1·64% between 2010 and 2017. In 2017, DAH for HIV/AIDS was $9·6 billion, with 49·4% being spent on grant administrations and global initiatives. In 2017, government spending on HIV/AIDS reached $9·7 billion (6·9–13·3), having increased at an annualised rate of 8·86% (8·40–9·34) since 2000. The amount sourced by out-of-pocket spending did not substantially increase, being $478·5 million (165·6–1069·9) in 2000 and $589·4 million (214·9–1347·9) in 2017. Total HIV/AIDS spending from prepaid private sources increased from $140·6 million (26·9–430·0) in 2000 to $395·8 million (93·2–1166·8) in 2017.

Since 2015, spending in low-income and middle-income countries to prevent and treat HIV/AIDS has increased from $10·6 billion (95% UI 8·3–13·9) to $12·0 billion (9·1–16·2) in 2017, primarily due to increases in government spending. Despite this growth, increases in spending levels have not been even across countries. 39 countries (including Zimbabwe and Kenya) spent less on HIV/AIDS in 2017 than in 2015, primarily because of reductions in DAH; a full list of country estimates is on the Global Health Data Exchange website.

The amount of HIV/AIDS spending per prevalent case for each region in 2017, and the fraction that is from each financing source is shown in figure 1B. Although more was spent in total in sub-Saharan Africa ($7660·6 million [95% UI 6736·2–8883·6]), HIV/AIDS spending per prevalent case was highest in the GBD super-region north Africa and the Middle East ($2987 [95% UI 1857–4589] per prevalent case), followed by Latin American and the Caribbean ($1702 [1113–2640] per prevalent case). Per prevalent case, spending was lowest in south Asia ($297 [216–413] per prevalent case) and sub-Saharan Africa ($296 [263–338] per prevalent case). Although domestic governments contributed the most to spending on HIV/AIDS in most regions, financing was dominated in sub-Saharan Africa by DAH (54·9% [48·0–61·5] of total HIV/AIDS spending; figure 1B).

SDG indicator 3.3.1 is to eliminate HIV/AIDS incidence. Change in HIV/AIDS incidence and HIV/AIDS spending per capita for each low-income and middle-income country for 2000–17 is shown in figure 1C. For all but one (Venezuela) of 135 countries, HIV/AIDS spending per capita has increased since 2000. 73 countries had reductions in HIV/AIDS incidence, while 62 had increases in incidence. While sub-Saharan Africa has had major decreases in HIV incidence and increases in spending per capita, other super-regions have had increases in HIV incidence since 2015.

**Figure 2:**
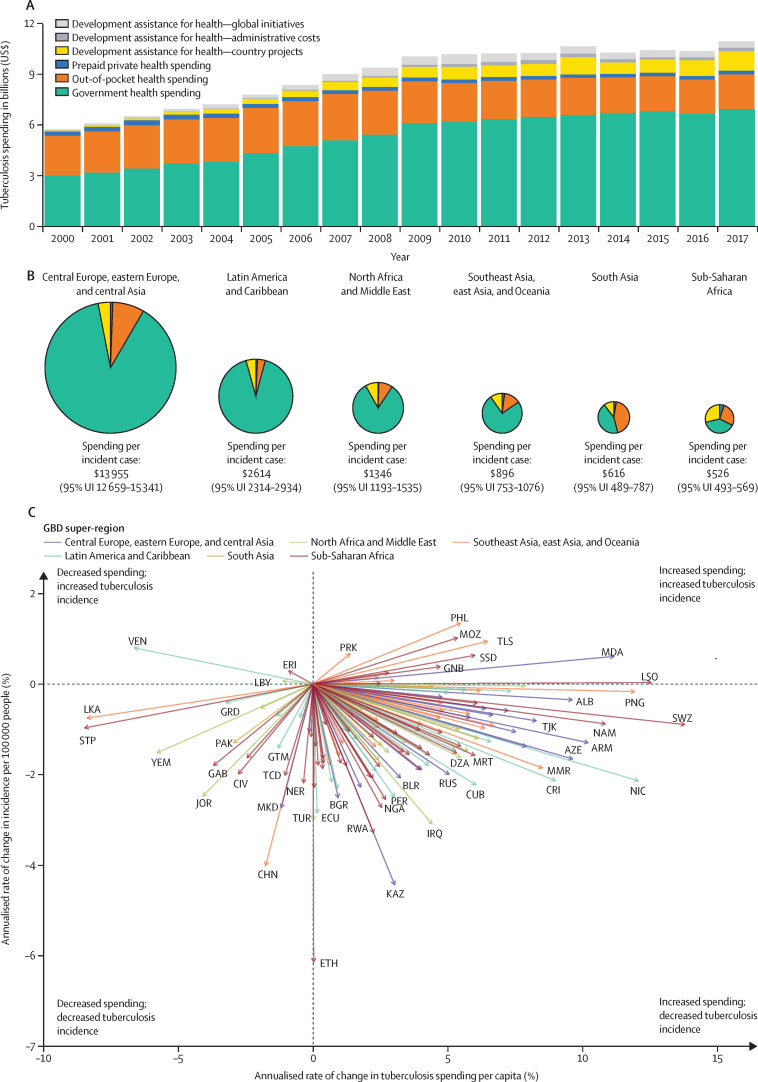
Tuberculosis spending in low-income and middle-income countries (A) Spending on tuberculosis by financing source, 2000 to 2017. (B) Breakdown of financing sources of tuberculosis spending and total tuberculosis spending for each incident case, by GBD super-region, in 2017, with pie size proportional to spending per prevalent case of tuberculosis. (C) Annualised rates of change in tuberculosis incidence and tuberculosis spending per capita, with each arrow showing one country moving from 2000 to 2017. Data are from all World Bank low-income and middle-income countries and spending estimates are presented in 2019 US$. Venezuela's spending is presented in 2014 US$. Administrative expenses that are only shown in panel A reflect the operational expense of deploying the grant that is accrued in the donor country (eg, salaries of headquarters office staff). ALB=Albania. ARM=Armenia. AZE=Azerbaijan. BGR=Bulgaria. BLR=Belarus. CHN=China. CIV=Côte d'Ivoire. CRI=Costa Rica. CUB=Cuba. DZA=Algeria. ECU=Ecuador. ERI=Eritrea. GAB=Gabon. GBD=Global Burden of Diseases, Injuries, and Risk Factors study. GNB=Guinea-Bissau. GRD=Grenada. GTM=Guatemala. IRQ=Iraq. JOR=Jordan. KAZ=Kazakhstan. LBY=Libya. LKA=Sri Lanka. LSO=Lesotho. MDA=Moldova. MKD=North Macedonia. MMR=Myanmar. MOZ=Mozambique. MRT=Mauritania. NAM=Namibia. NER=Niger. NGA=Nigeria. NIC=Nicaragua. PAK=Pakistan. PER=Peru. PHL=Philippines. PNG=Papua New Guinea. PRK=North Korea. RUS=Russia. RWA=Rwanda. SSD=South Sudan. STP=São Tomé and Príncipe. SWZ=eSwatini. TCD=Chad. TJK=Tajikistan. TLS=Timor-Leste. TUR=Turkey. VEN=Venezuela. YEM=Yemen.

Total tuberculosis spending disaggregated by source in 135 low-income and middle-income countries for 2000–17 is shown in figure 2A. These countries comprise 98·4% (95% UI 98·3–98·4) global tuberculosis incidence in 2017 and 98·7% (98·7–98·8) of tuberculosis deaths for the same year.^23^, ^38^ For these countries, spending on tuberculosis increased at an annualised rate of 3·87% (3·04–4·64), from $5·7 billion (5·2–6·5) in 2000 to $10·9 billion (10·3–11·8) in 2017. Government spending amounted to $6·9 billion (6·5–7·5) or 63·5% (59·2–66·8) of all tuberculosis spending in 2017 and increased annually by 5·06% (4·43 to 5·72) since 2000. Although DAH for tuberculosis increased at an even faster rate (15·91%), the amount in 2017 was $1·7 billion, of which 33·5% was spent on administrative costs and global initiatives. The amount of tuberculosis spending that was out-of-pocket has decreased steadily since 2000, as more tuberculosis cases have been treated in the public system. In 2000, an estimated $2·4 billion (1·9–3·1) was spent out-of-pocket on tuberculosis, while in 2017, $2·1 billion (1·6–2·7) was spent. Spending on tuberculosis from prepaid private sources was $246·9 million (171·9–368·7) in 2000 and $225·0 million (184·1–280·7) in 2017. Since the start of the SDGs in 2015, 87 of 135 low-income and middle-income countries we analysed increased their spending on tuberculosis (for more details see the WHO Global Health Data Exchange).

The amount of tuberculosis spending per incident case for each GBD super-region in 2017 (excluding the high-income region), and the fraction that is from each financing source are shown in figure 2B. Total spending was greatest in the central Europe, eastern Europe, and central Asia (appendix p 10), which also had the largest spending per incident case ($13 955 [95% UI 12 659–15 341]). The lowest spending per incident case was in sub-Saharan Africa ($526 [493–569]) and south Asia ($616 [489–787]). In most super-regions in 2017, domestic governments were the source of more than 75% of total tuberculosis funding. In south Asia, the dominant sources of spending were governments (44·1% [32·3–54·8]) and out-of-pocket spending (42·8% [30·0–56·8]), while in sub-Saharan Africa, governments (39·0% [35·4–42·7]) and DAH (28·8% [26·6–30·7]) are the main sources of funding for tuberculosis.

SDG indicator 3.3.2 is to eliminate tuberculosis incidence. Change in tuberculosis incidence and tuberculosis spending per capita for each low-income and middle-income country for 2000–17 is shown in figure 2C. 122 (90%) of 135 low-income and middle-income countries saw decreases in tuberculosis incidence between 2000 and 2017, with the few exceptions being primarily in sub-Saharan Africa and southeast Asia, east Asia, and Oceania. We saw substantial variation in spending patterns over time, with 113 (84%) of 135 countries increasing spending and 22 (16%) countries decreasing tuberculosis spending per capita. For tuberculosis, in eSwatini, Lesotho, and Nicaragua, we observed a more than 12% annualised rate of change in per capita spending with varying annualised rates of change in incidence (0·89% decrease for eSwatini, 0·04% increase for Lesotho, 2·14% decrease for Nicaragua; figure 2C).

**Figure 3:**
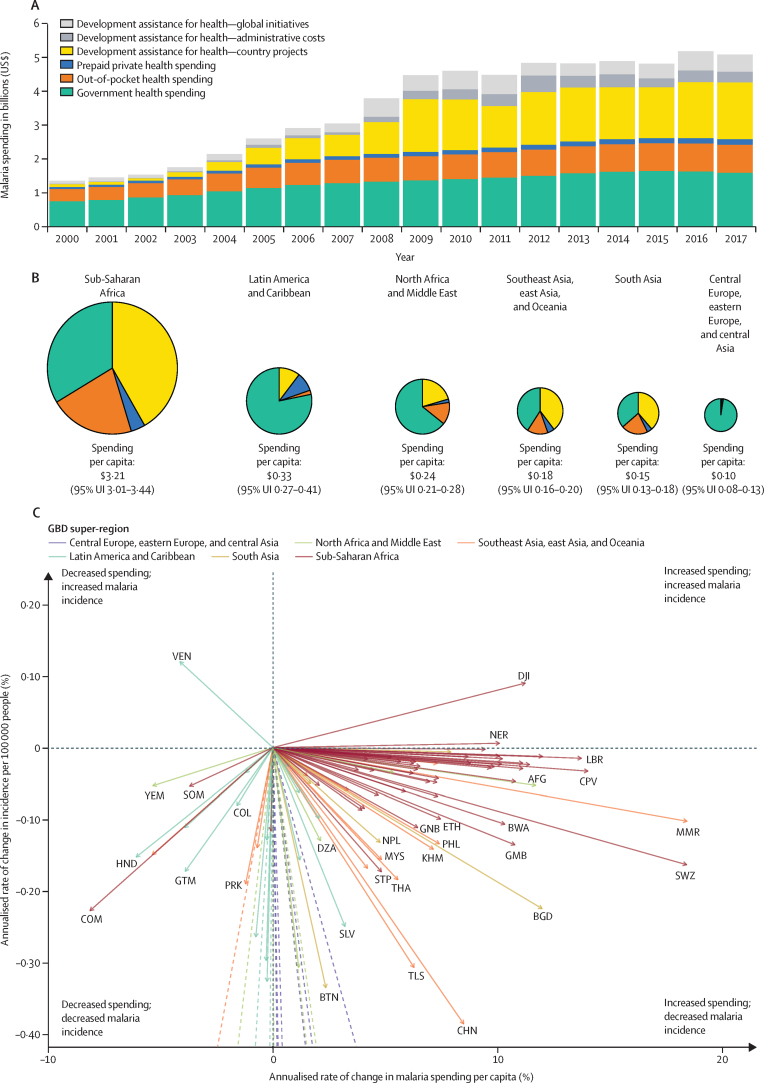
Malaria spending in 106 malaria endemic countries (A) Total spending on malaria by financing source, 2000 to 2017. (B) Breakdown of financing source of malaria spending and the total malaria spending for each incident case, by GBD super-region, in 2017, with pie size proportional to spending per prevalent case of malaria. (C) Annualised rates of change in malaria incidence and malaria spending per capita, with each arrow showing one country moving from 2000 to 2017. Data are from all malaria-endemic World Bank low-income and middle-income countries and spending estimates are presented in 2019 US$. Venezuela's spending is presented in 2014 US$. Administrative expenses are only shown in panel A reflect the operational expense of deploying the grant that is accrued in the donor country (eg, salaries of headquarters office staff). In panel C, dashed lines indicate countries that have eliminated malaria. World Bank low- and middle-income countries that have eliminated malaria since 2000 are Argentina, Armenia, Azerbaijan, Costa Rica, Georgia, Iraq, Kyrgyzstan, Morocco, Paraguay, Sri Lanka, Syria, Tajikistan, Turkey, and Uzbekistan. AFG=Afghanistan. BGD=Bangladesh. BTN=Bhutan. BWA=Botswana. CHN=China. COL=Colombia. COM=Comoros. CPV=Cape Verde. DJI=Djibouti. DZA=Algeria. ETH=Ethiopia. GBD=Global Burden of Diseases, Injuries, and Risk Factors study. GMB=The Gambia. GNB=Guinea-Bissau. GTM=Guatemala. HND=Honduras. KHM=Cambodia. LBR=Liberia. MMR=Myanmar. MYS=Malaysia. NER=Niger. NPL=Nepal. PHL=Philippines. PRK=North Korea. SLV=El Salvador. SOM=Somalia. STP=São Tomé and PrÍncipe. SWZ=eSwatini. THA=Thailand. TLS=Timor-Leste. VEN=Venezuela. YEM=Yemen.

Total malaria spending disaggregated by financing source in 106 countries with local malaria transmission since 2000 is shown in figure 3A. 102 (96%) of 106 countries are low-income or middle-income countries, and 99·98% (95% UI 99·97–99·98) of malaria deaths in 2017 were in these 106 countries.^38^ Spending on malaria increased annually by 7·96% (95% UI 8·20–7·74) from $1·4 billion (1·3–1·5) in 2000 to $5·1 billion (4·9–5·4) in 2017. Domestic governments have been a relatively stable source of funding for malaria, with spending changing from $0·8 billion (0·7–0·9) in 2000 to $1·6 billion (1·5–1·8) in 2017. Meanwhile, spending from DAH and out-of-pocket spending have substantially increased, comprising 48·7% (46·3–50·8) and 16·1% (13·4–19·8) of total spending in 2017. DAH contributed $2·5 billion in 2017, while $0·8 billion (0·7–1·0) was contributed by out-of-pocket spending.

Since 2015, spending on malaria increased from $4·8 billion (95% UI 4·6–5·1) to $5·1 billion (4·9–5·4) in 2017, and spending in 63 (59%) of 106 countries increased (figure 3). Increases in prepaid-private spending, out-of-pocket spending, and DAH all contributed to these increases.

The amount of malaria spending per capita for each region in 2017 and the fraction that is from each financing source are shown in figure 3B. The spending per capita in sub-Saharan Africa was $3·21 (95% UI 3·01–3·44), which was much larger than in the other malaria endemic GBD super-regions. The least spending per capita was in central Europe, eastern Europe, and central Asia ($0·10 [0·08–0·13]). Government spending constitutes the most spending on malaria in the super-regions of central Europe, eastern Europe, and central Asia; Latin America and Caribbean; north Africa and the Middle East; and southeast Asia, east Asia, and Oceania. DAH made up a larger share of the spending on malaria for sub-Saharan Africa than the other communicable diseases tracked in this study.

SDG indicator 3·3.3 is to eliminate malaria incidence. Change in malaria incidence and malaria spending per capita for each of 102 malaria endemic low-income and middle-income country for 2000–17 are shown in figure 3C. This figure highlights the 13 of 102 countries that have eliminated malaria since 2000 and relatively constant malaria spending per capita. Additionally, all but three of the remaining 93 remaining malaria endemic low-income and middle-income countries—Djibouti, Niger, and Venezuela—have seen reductions in malaria incidence. Meanwhile, malaria spending per capita has increased in 78 of 106 malaria endemic low-income and middle-income countries with the largest spending increases in sub-Saharan Africa. We observed that in Myanmar and eSwatini, per capita spending increased at an annualised rate of more than 15% from 2000 to 2017, and annualised incidence rate of the disease decreased by more than 10% (figure 3C).

**Figure 4:**
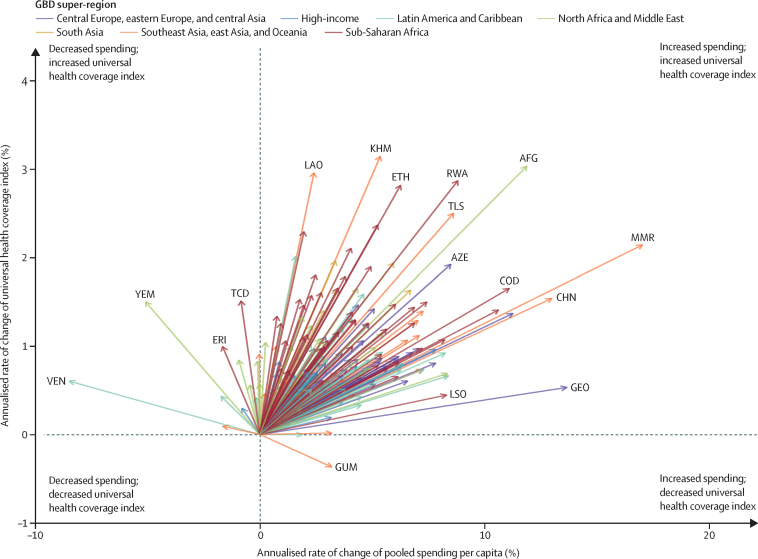
Annualised rate of change in universal health service coverage index and annualised rate of change in pooled health spending per capita, 2000 to 2017 Data are for 195 countries in territories, by GBD super-region. Spending estimates are presented in 2019 US$, and pooled health spending is the sum of government spending, prepaid private spending, and development assistance for health. Each arrow shows one country moving from 2000 to 2017. Spending estimates are presented in 2019 US$. AFG=Afghanistan. AZE=Azerbaijan. CHN=China. COD=Democratic Republic of the Congo. ERI=Eritrea. ETH=Ethiopia. GBD=Global Burden of Diseases, Injuries, and Risk Factors study. GEO=Georgia. GUM=Guam. KHM=Cambodia. LAO=Laos. LSO=Lesotho. MMR=Myanmar. RWA=Rwanda. TCD=Chad. TLS=Timor-Leste. VEN=Venezuela. YEM=Yemen.

Change in universal health coverage service coverage index and pooled health spending per capita for 2000–17 across all 195 countries and territories is shown in figure 4. We saw a strong association between increases in pooled health spending per capita and progress towards universal health coverage, with countries in the GBD super-regions of sub-Saharan Africa, south Asia, and southeast Asia, east Asia and Oceania making large gains in universal health coverage as pooled spending per capita increased. Since 2015, spending increased in 166 (85%) of 195 countries and universal health service coverage increased in 188 (96%) countries (data not shown).

**Figure 5:**
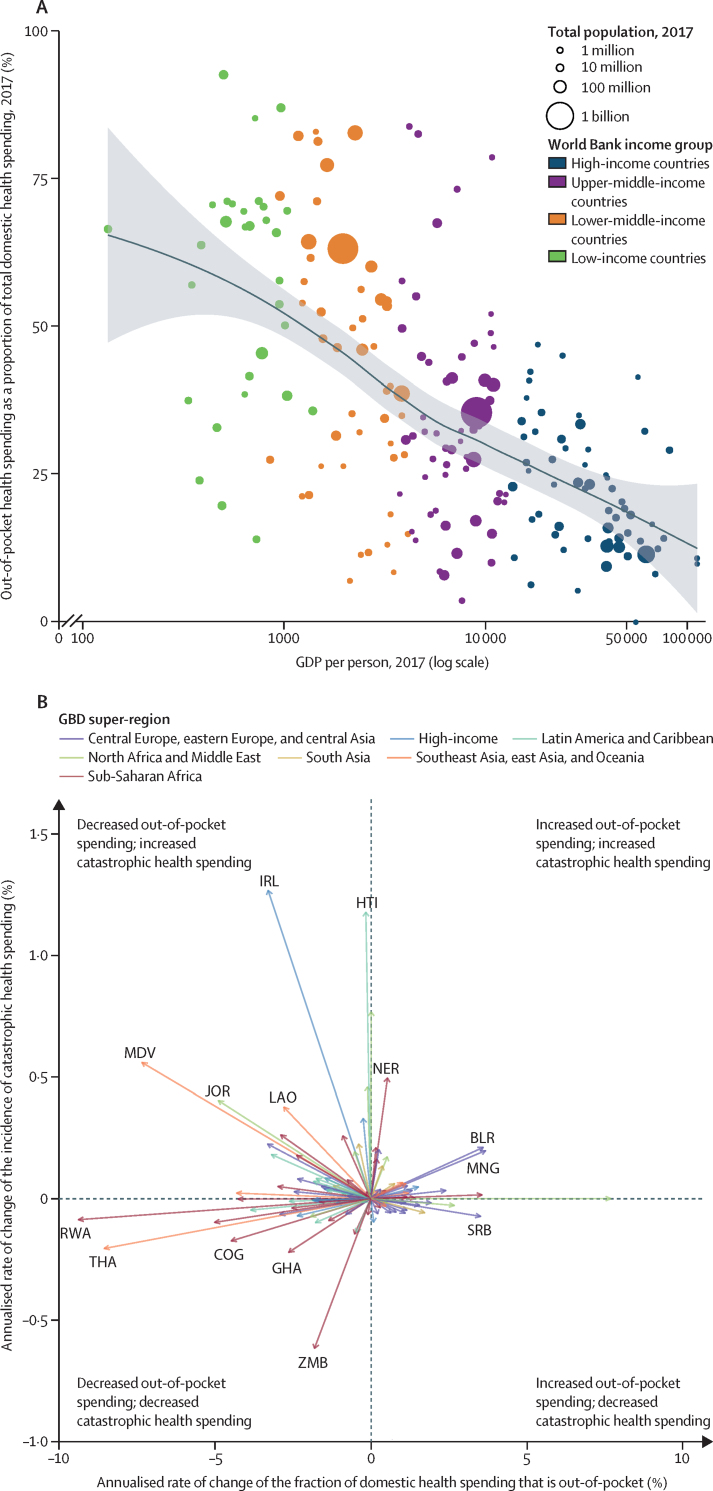
Out-of-pocket spending for health (A) Out-of-pocket spending as a share of total health spending, in 2017. (B) Change in proportion of households with catastrophic health spending versus change in proportion of domestic health spending that is out-of-pocket, 2000–17. Spending estimates are presented in 2019 US$. In panel A, estimates are plotted against GDP per capita with a loess regression line (span=0·95) and 95% uncertainty intervals shaded in grey. Timor-Leste is excluded from panel B because the World Bank estimates for 2000–17 showed that no households in the country had catastrophic health spending. BLR=Belarus. GBD=Global Burden of Diseases, Injuries, and Risk Factors study. GDP=Gross Domestic Product. HTI=Haiti. IRL=Ireland. JOR=Jordan. LAO=Laos. MDV=Maldives. MNG=Mongolia. NER=Niger. RWA=Rwanda. SRB=Serbia. THA=Thailand. ZMB=Zambia.

Although economic development is associated with reducing the domestic health financing burden that is funded by out-of-pocket spending, considerable variation exists in this association (figure 5A). For any one level of GDP per capita, a sizeable range of the fraction of domestic health spending is financed by out-of-pocket spending, suggesting that economic development does not solely determine the transition away from household financing. Additionally, large variation exists across countries in the association between rate of change in the fraction of domestic health spending that is out-of-pocket and the rate of change in the proportion of households with catastrophic health expenditure (figure 5B). A reliance on domestic government, prepaid, and pooled health financing is a means towards achieving universal health coverage and financial risk protection. Globally, this fraction contributing to universal health coverage ranges from 6·7% (95% UI 4·5–9·1) in Afghanistan to 100% (100–100) in Greenland (for more details see the WHO Global Health Data Exchange).

**Figure 6:**
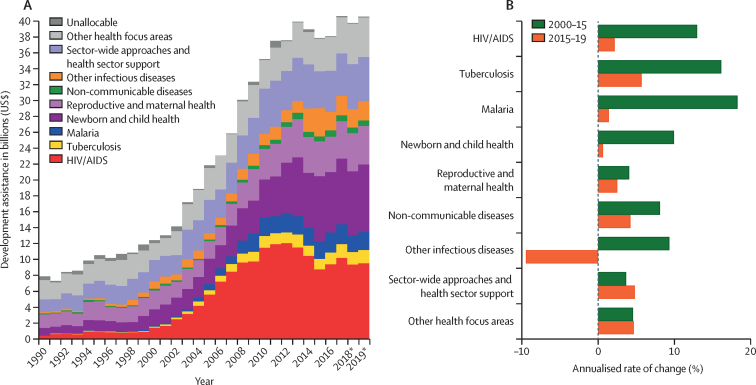
Development assistance for health (A) Changes in development assistance for health by health focus area, 1990–2019. (B) Annualised rate of change in development assistance for health by health focus area, 2000–15 and 2015–19. Estimates are presented in billions of 2019 US$. GBD=Global Burden of Diseases, Injuries, and Risk Factors study. *Data for 2018 and 2019 are preliminary estimates based on budget data and estimation.

In 2019, $40·6 billion of DAH was disbursed and increased at an annualised rate of 1·74% since 2015 (figure 6A). Over 1990 to 2019, reproductive and maternal health has consistently received substantial contributions, starting from $1·7 billion in 1990 to $4·8 billion in 2019. This change constitutes an annualised rate of change of 3·65%. However, since in 2004, DAH for HIV/AIDS has received the highest contributions of all health focus areas, peaking at $12·0 billion in 2012.

The annualised rate of change across the health focus areas for the time periods associated with the Millennium Development Goals (MDGs) and the SDGs are shown in figure 6B. Between 2000 and 2015, DAH increased by 7·75%, with disbursements for malaria increasing by 18·32%, for tuberculosis by 16·18%, and for HIV/AIDS by 13·02%. For the period 2015–19, the annualised growth rate for tuberculosis spending is 5·75%, for HIV/AIDS is 2·18%, and for malaria is 1·43%. Other key health focus areas that are funding progress on specific SDG indicators have had annual rates of change for 2015–19 of 4·25% for non-communicable diseases, 2·53% for reproductive and maternal health, and 0·66% for newborn and child health. The annualised growth rate of DAH for other infectious diseases has decreased for the period 2015–19, which is driven by the increased contributions that went towards the Ebola outbreak in 2014–15 and the subsequent re-alignment of resources after the Ebola crisis.

Based on past trends and associations, we estimate that an additional $238 (95% UI 209–267) per capita will be available for health globally in 2030 compared with 2017, with persistent disparities in spending between countries and across income groups (table 2). The proportion of health spending from pooled sources is expected to increase from 81·6% (81·6–81·7) in 2015 to 83·1% (82·8–83·3) in 2030. In high-income countries, health spending is expected to continue to increase, with expected annualised growth rates of 1·93% (1·77–2·10), reaching $6596 (6482–6708) per capita in 2030. For high-income countries, government and prepaid private spending as the financing sources of health is expected to increase to 87·8% (87·5–88·1) of total health spending. Health spending growth is expected to be fastest in lower-middle-income countries, where the annual growth rate is expected to be 4·38% (4·13–4·66) between 2018 and 2030, with per-capita spending reaching $127 (114–141). In these countries, government spending is also expected to be the financing source with the fastest growth (5·12% [4·73–5·54]), with government and prepaid private health spending making up 45·7% (40·1–51·0) of overall spending. For countries currently considered to be low income by The World Bank, resources for health are expected to grow annually by 4·13% (3·75–4·55) between 2018 and 2030, although in per capita terms, annualised health spending growth is expected to be only 1·52% (1·15–1·93), reaching $45 (42–48) per capita in 2030.

## Discussion

Disease-specific spending studies are valuable because they can provide policy makers and planners with insights into the financial burden created by specific diseases. This knowledge can subsequently be used in prioritisation, planning, budgeting, and evaluation of programmes; programme and policy interventions and development; and ultimately in better management of health systems. Our analysis quantified health sector spending and health spending on HIV/AIDS, tuberculosis, and malaria relative to outcomes, which are all priorities under SDG3. We also examined future availability of resources for health. Our results highlight that, globally, total health spending has increased since the state of the SDGs in 2015, reaching $7·9 trillion (7·8–8·0) in 2017, and is expected to increase to $11·0 trillion (10·7–11·2) by 2030, although with substantial disparity across countries. In 2017, in low-income and middle-income countries, an estimated $20·2 billion was spent on HIV/AIDS, $10·9 billion was spent on tuberculosis, and $5·1 billion was spent on malaria in endemic countries. Although both domestic government and DAH spending, has increased across these three diseases, the accompanied changes in outcomes have varied. We found that malaria had the most consistent decreases in outcomes across countries as spending has increased.

These health spending estimates enable further examination of the existing publicly available estimates of the financing required to achieve the SDG3 targets. Existing estimates suggest that the additional annual financing required to achieve SDG3 in 67 low-income and middle-income countries is $274 billion (progress scenario in which the attainment of goals is limited by countries' health systems existing absorptive capacity), and to reach health system targets for SDG3 including scale-up of health workforce and infrastructure is $371 billion.^28^ Stenberg and colleagues estimated that to achieve SDG3 in 67 low-income and middle-income countries, the corresponding per capita spending would need to increase to $249 per year (progress scenario) or $271 per year (ambitious scenario) by 2030.^28^ Another study by the Sustainable Development Solutions Network that included 59 low-income countries estimated that to achieve SDG3 would cost, approximately $225 billion between 2019 and 2030, with a per capita cost of $86 for low-income countries and $134 for lower-middle-income countries as the minimum needed to provide care consistent with basic human rights.^39^ A few other studies have also generated estimates of the resources needed using different methods.^36^, ^37^, ^40^ Our estimates suggest that 81 (60%) of 135 low-income and middle-income countries have not yet reached health spending of $249 per capita, and our projections suggest that 75 countries might still not reach these goals by 2030.

Although these financing goals can be benchmarks to encourage more spending and increased health system efficiency, they do not ensure that SDG3 will be achieved. Ultimately, costing estimates like these need to be continuously improved to make them locally relevant and price appropriate, with realistic assumptions about health system inefficiency and the distribution of spending in a country, and to incorporate any challenges associated with preventing and treating disease in difficult to reach contexts.

For all three diseases for which a complete and comparable series of spending estimates exist in low-income and middle-income countries—HIV/AIDS, tuberculosis, and malaria—comparing the relative contributions from the different financing sources highlights interesting patterns. Governments contribute substantially across all three diseases. This observation is important because domestic resource mobilisation has received renewed interest as a key strategy for generating resources to finance the SDGs.^4^ While DAH contributions to malaria and HIV/AIDS are substantial, contributions to tuberculosis are smaller. This pattern brings into light longstanding concerns and debate regarding the allocation of DAH especially across health focus areas.^41^, ^42^, ^43^ These concerns and debate include whether the current criteria that rely mainly on a country's level of development are the most appropriate to use for allocation, donor preference for implementing vertical programmes with short-term measurable effects, and prioritisation of such diseases to broader health system challenges. Also, the relative dependence on household out-of-pocket spending across the three diseases is notable, with the proportion of out-of-pocket spending for tuberculosis and malaria being much larger than the proportion for HIV/AIDS. Changes in policy, such as making HIV treatment available and free to all, has transformed management of care for HIV/AIDS and its by-source funding distribution. Previous studies have shown that high out-of-pocket spending promotes health impoverishment.^17^, ^44^, ^45^ Hence, targeted efforts, such as public education campaigns on enrolment in national health insurance or free provision of services where appropriate, aimed at increasing the share of national health spending that is financed through pooled resources might improve financial protection.

The distribution of health spending by source across different regions also highlights heterogeneous health financing patterns for the three diseases globally. For some geographical regions (eg, Central Europe, eastern Europe, and central Asia, and sub-Saharan Africa), governments carry the primary burden of providing resources for these diseases, while in other regions (eg, southeast Asia, east Asia, and Oceania, and south Asia) the pattern of financing changes with the type of disease. Similarly, health spending globally has distinct patterns. In the high-income GBD super-region, spending by governments dominates, while in other super-regions, such as south Asia and sub-Saharan Africa, DAH and out-of-pocket spending are prominent. Preferably, resources for financing health care should be pooled to restrict the risk of health impoverishment for the population and delays in accessing needed care. Resources for financing health care could be pooled through government facilitation of the development of viable prepaid mandatory insurance programmes.

The association between spending on health and health outcomes is of interest to many audiences, especially because of the increases in spending on health that have been observed in the past two decades with the adoption of the MDGs and now the SDGs. Our results highlight a nuanced and complex picture regarding the link between health spending and its associated effect on outcomes—here, disease-specific outcomes. Although substantial reductions in the incidence of some diseases were observed as spending increased in some countries, in others decreases in the incidence of other disease were minimal or even increases in incidence were seen. For example, for malaria, we observed that in Myanmar and eSwatini, per-capita spending increased at an annualised rate of more than 15% from 2000 to 2017, and the annualised incidence rate decreased by more than 10%, while for tuberculosis, in eSwatini, Lesotho, and Nicaragua, we observed a more than 12% annualised rate of growth in per-capita spending with varying annualised rates of change in incidence (0·89% decrease for eSwatini, 0·04% increase for Lesotho, 2·14% decrease for Nicaragua). Because these findings are not causal, interest in understanding this link between spending on health and health outcomes is strong and more efforts would be needed to understand the drivers of success.

Overall, these results highlight the continued importance of domestic resource mobilisation in securing the financing required for the SDGs and the health-related SDGs in particular. Although donor contributions will be necessary to meet spending targets in some low-income countries, governments were an important source of funding in the broader health system and among the three disease areas for which data were available. For most middle-income countries, the aspiration is that national economic growth will also bolster what resources are allocated to the health sector by the government. Furthermore, although DAH will continue to be needed in some low-income countries, a continued need for and value in DAH provision to cover so-called global public goods or common goods for health exists (such as pandemic preparedness or research and development for neglected tropical diseases).^35^, ^46^, ^47^, ^48^, ^49^, ^50^, ^51^, ^52^ This continued need is because the multisectoral nature of the SDGs and the increasingly interconnected world we live in present shared global challenges that need to be addressed beyond the individual country support framework that DAH has typically addressed.

In addition to the need to generate more resources to finance the health-related SDGs, the need to efficiently use existing resources should be highlighted. The comparison of the annualised rates of change of pooled health spending per capita and universal health coverage index highlighted some of the best performers at each level of development. For example, Myanmar and Georgia show annualised growth in per-capita-pooled spending of more than 13%, which was associated with 2·14% annualised growth in universal health coverage index in Myanmar and 0·05% in Georgia. Peer-to-peer country learning might facilitate the transfer of best practices in both the delivery and administration of the health sector in countries that are not yet performing optimally with their available resources. The second annual Universal Health Coverage Financing Forum organised by the World Bank highlighted strategies such as strategic purchasing, improvement in data management systems, and organisational management that can be adopted to promote better efficiency for health.^53^ However, while important health gains can probably be made by increasing efficiency and investing in allied sectors, our future health spending estimates indicate that spending is expected to remain low in many countries, which raises concerns about the viability of reaching crucial SDG3 goals in those countries. In such countries, additional efforts to mobilise revenue, such as tax reforms where appropriate, are needed to ensure that adequate resources are available to support the achievement of the SDG3 goals.

Furthermore, the nuanced evidence on the scale-up of spending and improvements in health outcomes suggest a complex association between spending and health outcomes. This complexity highlights that, although more resources are likely to be needed to achieve SDG3, other constraints such as inefficient resource allocation, weak governance systems, drug shortages, and inadequate health workforce and management systems for health information in the broader health system that constrain improvements in health outcomes will need to be addressed to achieve the SDG3 targets.

Finally, this study has also shown the gaps in current resource tracking efforts as they relate to the health-related SDGs. Most comparative data are available for the three diseases we studied but little comparable data on financing for most of the remaining indicators are available. This pattern might reflect funding priorities spurred by the MDGs. Given the broader orientation of the goals under the SDGs, a need exists for increased understanding on the financing for the other SDG3 targets.

Future research areas might include efforts targeted at financing health-related SDGs, such as hepatitis B, neglected tropical diseases, and non-communicable diseases, including substance abuse, alcohol use, road injury, adolescent birth, hazardous chemicals, and air, water, and soil pollution. Additionally, studies that aim to determine the types of spending that promote improvements in outcomes are needed to guide resource investments.

This study has several limitations. First, the data we used reported using different research units for each SDG3 indicator. HIV/AIDS, tuberculosis, and malaria spending estimates were available for a subset of total countries. As such, although the available data is meaningful and contributes to our knowledge of spending on the SDGs, a directly comparable analysis of global spending inclusive of all countries on these indicators is currently constrained by gaps in the available data. Also, multiple competing cost estimates exist, and so the existing financing targets that we found for comparison with our spending estimates often differed in geographical scope, methods, and currency. These comparisons require precise and context-specific costing estimates that incorporate realistic levels of efficacy. Second, some of the input data on global health spending used to generate total health spending estimates had questionable annual growth trends or did not provide direct information about the sources of the data. We used modelling methods to enable incorporation of these data but we acknowledge that challenges exist in terms of the quality of the available global health spending data. Additionally, we provided UIs for the estimates to provide information on the quality and precision of the estimates generated. Third, while the use of keywords to isolate relevant health focus and programme areas for our DAH analysis is the best existing strategy for a comprehensive effort, it relies heavily on the project description provided in the databases and in some instances might not accurately reflect what the funds actually contributed to. Fourth, presenting spending at disease level might not be the level of aggregation that is most relevant for ministries of health and disaggregating at facility level (primary, secondary, and tertiary) or at expenditure-item level (staff, commodities) might be more readily useful to them. We plan to provide estimates that include these levels of disaggregation in the near future. Notably, our analysis of spending and outcomes was not designed to detect causality, but was primarily a descriptive analysis of the associations between these two metrics. Therefore, our findings should not be interpreted as causal. Fifth, when forecasting health expenditures, we are unable to incorporate fundamentally new and different policies or innovations that are outside of the bounds of the observed data. Current estimates do not directly account for mass migration due to conflict and are only able to be incorporated on the basis of these events being reflected in the underlying covariates.

Furthermore, due to data availability, our analyses mainly covered four SDG priority areas. Spending on several SDG3 targets, including hepatitis B incidence, substance abuse, chemical and environmental pollution do not yet have a comparable set of spending estimates, which would prohibit analyses. Ideally, estimates on DAH and domestic spending on all the priority areas under SDG3 will provide a more comprehensive picture. Nonetheless, we believe that the data and estimates we have provided are an adequate first step in monitoring the spending for these key SDG3 areas.

Finally, while each set of spending estimates is consistent and comparable on its own, the input data vary enough between diseases that the spending estimates between diseases are not perfectly comparable. For example, the HIV/AIDS and malaria government spending estimates are modelled on the basis of tabulated data, generally reporting total spending or budgets, while government spending estimates for tuberculosis and for out-of-pocket malaria and tuberculosis spending were based on taking the product of unit cost and service coverage estimates. These distinct estimation strategies will drive some differences, with unit cost and service coverage estimates not comprehensively including inefficient spending that does not yield increases in service coverage.

As of publication of this Article, health systems throughout the world are stretched thin addressing the effects of coronavirus disease 2019 (COVID-19). Over the past 5 months it has become increasingly clear that, although not yet fully realised, both the health and economic losses caused by this novel coronavirus will be immense. Because these costs are not yet known in full and because the pandemic is ongoing, the effects of COVID-19 have not been considered in the financing projections reported in this Article. If these costs lead to reductions in health spending as nations focus inward on economic woes or if these costs can be a catalyst for investment in robust public health systems and in shared vision of global health security remains to be seen.

The link between spending and changes in outcomes remains complex, and realistic country-specific spending targets for most SDG3 indicators do not exist. Understanding how much is being spent and where crucial gaps exist are the first steps in providing evidence for a global dialogue about how much investment is needed for health, where it should come from, and where and on whom it should be spent. Using the resources available more efficiently and addressing broader health system constraints to service delivery, such as inadequate health information management systems, weak governance systems, shortages in health workforce and pharmaceuticals, are crucial if substantial progress is to be made towards achieving the health-related SDGs. A key tenet underlying the SDG era is that “no one is left behind”. To achieve this goal by 2030, current efforts must expand to include the tracking of spending in all of these areas, increasing resources, and spending those resources more efficiently.

## Data sharing

Data used for this study were extracted from publicly available sources that are listed in the appendix. Further details are available on the Global Health Data Exchange website.
