# Supplementary material for: Health sector spending and spending on HIV/AIDS, tuberculosis, and malaria, and development assistance for health: progress towards Sustainable Development Goal 3
Source: Lancet. 2020 Sep 5;396(10252):693–724. doi: 10.1016/S0140-6736(20)30608-5 (PMC7180045; doi:10.1016/S0140-6736(20)30608-5)
Supplement: Supplementary appendix [file mmc1.pdf]

THE LANCET

Supplementary appendix

This appendix formed part of the original submission and has been peer reviewed. We post it as supplied by the authors.

Supplement to: Global Burden of Disease Health Financing Collaborator Network. Health sector spending and spending on HIV/AIDS, tuberculosis, and malaria, and development assistance for health: progress towards Sustainable Development Goal 3. *Lancet* 2020; published online April 23. http://dx.doi.org/10.1016/S0140-6736(20)30608-5.

SUPPLEMENTARY METHODS ANNEX

Health spending for Sustainable Development Goal 3: total, HIV/AIDS, tuberculosis, malaria, universal health service coverage, financial risk protection, and donor spending, 2000 to 2030 and 2050

Version: March 26, 2020

Global Burden of Disease Health Financing Collaborator Network

Table of Contents

SECTION 1. METHODS OVERVIEW

S1.1 Overview

S1.2 Estimating domestic health spending: 1995-2016

S1.3 Estimating development assistance for health: 1990-2018

S1.4 Reporting and uncertainty analysis

SECTION 2. TRACKING TOTAL HEALTH SPENDING AND ITS COMPONENTS

S2.1 Overview

S2.2 Statistical model to fill missingness in health expenditure variables

S2.3 Currency exchange and deflation

SECTION 3. TRACKING DEVELOPMENT ASSISTANCE FOR HEALTH FROM TRADITIONAL DONORS

S3.1 Overview

S3.2 Tracking development assistance for health from bilateral aid agencies and the European Commission

S3.3 Tracking development assistance for health from the development banks

S3.4 Tracking contributions from GFATM and GAVI

S3.5 Tracking expenditure by United Nations Agencies active in the health domain

S3.6 Tracking development assistance for health from private foundations

S3.7 Tracking non-governmental organizations

S3.8 Tracking Development Assistance for health from China

S3.9 Calculating the technical assistance and program support component of development assistance for health from loan-and grant-making channels of assistance

S3.10 References

SECTION 4. TRACKING TOTAL SPENDING ON MALARIA BY SOURCE IN 106 COUNTRIES,

2000–2017

S4.1 Currency conversion

S4.2 Spatiotemporal Gaussian process regression (ST-GPR)

S4.3 Government health expenditure as a source on malaria

S4.3.1 Data

S4.3.2 Modeling government expenditure on malaria patient care

S4.3.3 Modeling total government expenditure on malaria

S4.4 Out-of-pocket expenditure on malaria

S4.4.1 Price-volume model

S4.4.2 Modeling total out-of-pocket expenditure on malaria

S4.4.3 Excluding data from an NHA from Nigeria

S4.5 Prepaid private expenditure on malaria

S4.6 Malaria elimination status of the 106 countries

S4.7 Data density

S4.8 References

SECTION 5. TRACKING TOTAL SPENDING ON HIV/AIDS BY SOURCE IN 135 LOW- AND MIDDLE-INCOME COUNTRIES, 2000 THROUGH 2017

S5.1 Acronyms and abbreviations

S5.2 Overview

S5.3 Removing non-HIV focused spending from GARPR/GAM datasets

S5.4 Currency conversion

S5.5 Modeling HIV/AIDS spending with ST-GPR

S5.6 Enforcing internal consistency

S5.7 Development assistance for health

S5.8 References

SECTION 6. FORECASTING HEALTH SPENDING FROM 2017 through 2050

S6.1 Data

S6.1.1 Summary of Data Sources

S6.1.2 World Health Organization's Global Health Expenditure Database

S6.1.3 WB WDI, IMF WEO, PWT, and Maddison

S6.1.4 Institute for Health Metrics and Evaluation's Development Assistance for Health Database

S6.1.5 Institute for Health Metrics and Evaluation's Global Burden of Disease Study 2017

S6.2 Covariates

S6.2.1 List of Forecasted Variables

S6.2.2 Covariates Used for Forecasting

S6.3 Ensemble Modeling

S6.3.1 Sub-model Setup

S6.3.2 Covariates

S6.3.3 Convergence to Global Growth Rates

S6.3.4 Specifications

S6.4 Inclusion and Exclusion Criteria

S6.5 Creating the Forecasts

S6.5.1 Ranking Sub-Models

S6.5.2 Uncertainty Estimation

S6.6 Ad-hoc Draws Correlation

S6.6.1 Motivation

S6.6.2 Bivariate Correlated Distributions

S6.7 Future Health Scenarios

S6.7.1 Long-term Growth Regressions

S6.7.2 Forecasting Better and Worse Scenarios

S6.7.3 Uncertainty Estimation

S6.8 Package and Architecture

S6.8.1 Architecture

S6.8.2 Template Model Builder

S6.9 Tables and Figures

S6.10 Additional Tables and Figures

Abbreviations

ADB	Asian Development Bank and
AfDB	African Development Bank
BMGF	Bill & Melinda Gates Foundation
CRS	Creditor Reporting System
DAC	Development Assistance Committee
DAH	Development assistance for health
EC	European Commission
GDP	Gross domestic product
GFATM	Global Fund for AIDS, Tuberculosis and Malaria
GGHE	General government health expenditure
GHED	Global Health Expenditure Database
GHES	Government health spending (Aggregate of "Social insurance contributions" and "Transfers from government domestic revenue (allocated to health purposes)", as defined in GHED)
HSS	Health System Strengthening
IBRD	International Bank for Reconstruction and Development
IDA	International Development Association
IDB	Inter-American Development Bank
LCU	Local currency units
NASA	National AIDS Spending Assessments
NGO	Non-governmental organizations
NHA	National Health Accounts
NPISH	Non-profit institutions serving households
ODA	Official development assistance
OECD	Organisation for Economic Co-operation and Development
OOP	Out-of-pocket health spending (defined in GHED: “Other revenues from households n.e.c")
PAHO	Pan American Regional Office for WHO
PI	Private insurance
PPP	Prepaid private health spending (Aggregate of "Voluntary prepayment", "Other revenues from corporations n.e.c.", "Other revenues from NPISH n.e.c.", and "Compulsory prepayment (Other, and unspecified, than FS.3)", as defined in GHED)

SHA 2011	System of Health Accounts 2011
SWAps	Sector Wide Approaches
TB	Tuberculosis
THE	Total health spending (defined in GHED: “Current health expenditure by revenues of health care financing schemes”)
UNICEF	United Nations Children’s Fund
UNAIDS	Joint United Nations Programme on HIV/AIDS
UNFPA	United Nations Population Fund
USD	US dollars
VolAg	Report of Voluntary Agencies
WHO	World Health Organization

SECTION 1. METHODS OVERVIEW

S1.1 Overview

The methods presented here summarize the various components of the estimation process; interested readers are encouraged to consult the appendix for additional details on data sources, methods, and for additional results presented in alternative units. "Health spending" is defined as money spent on services, supplies, and basic infrastructure to deliver health care, using the same definition employed by the System of Health Accounts 2011 and the WHO Global Health Expenditure Database (GHED).[1,2] Health "financing" and "funding" are used interchangeably to refer to the source, as opposed to the utilization, of financial resources. "Economic development" refers to GDP per capita.

We estimated health spending from four main sources – government, out-of-pocket, prepaid private, and DAH – for 195 countries and territories. For brevity, "countries and territories" are referred to only as "countries", all of which are categorized into four World Bank income groups (high-, upper-middle-, lower-middle-, and low-income) and seven Global Burden of Disease (GBD) super-regions (Central Europe, Eastern Europe, and Central Asia; GBD high income; Latin America and Caribbean; North Arica and Middle East; South Asia; Southeast Asia, East Asia, and Oceania; and sub-Saharan Africa). Data tracking government, out-of-pocket, and prepaid private health spending, which together comprise total domestic health spending, were available from 1995 through 2017. Government health spending includes social health insurance and mandated private health insurance, as well as government public health programmes. Out-of-pocket health spending includes health care spending paid by the patient or his or her household, excluding insurance premiums paid in advance of care. Prepaid private health spending includes voluntary private insurance and non-governmental agency spending on health.

Estimates of DAH, defined as the financial and in-kind contributions from major development agencies to low- and middle-income countries for the purpose of maintaining or improving population health, were generated from 1990 through 2019, although transfers to recipient countries can only be estimated until 2017. In other words, the total amount of DAH, by source, is estimated through 2019, but is not allocated by recipient country for 2018 and 2019. The sum of domestic health spending and DAH, net of the administrative costs needed to run the development agencies, form the envelope of total health spending for each country and year.

Domestic health spending from each of the three sources was projected for each country from 2018 to 2050, and DAH was projected from 2018 to 2050, by modelling rates of change across time. These models incorporate country-specific time trends that attenuate across time and converge to the global average, consider a broad set of covariates and time-series modelling techniques, and propagate four types of uncertainty - model, data, parameter, and fundamental uncertainty.

S1.2 Estimating domestic health spending: 1995–2017

We extracted data on gross domestic product (GDP) per capita from five leading sources of these estimates (World Bank, International Monetary Fund [IMF], United Nations, Penn World Tables, and Maddison) and built from methods described by James and colleagues to generate a single series of GDP per capita using Gaussian processes.[3-7] This method incorporated data from all five GDP series and also propagated uncertainty through the estimates.[8] The resulting series spans 195 countries from 1950 to 2019, and larger uncertainty intervals highlight countries where the input data from the five sources was discordant or estimates were missing.

We extracted data from the WHO's Global Health Expenditure Database (GHED) on government domestic revenue transfers allocated for health, compulsory prepayment, voluntary prepayment, social insurance contributions, and other domestic revenue from households, corporations, and non-profit institutions serving households.[9] Data from GHED excludes spending on major investments such as hospital construction, health worker education and training, and research and development. Countries report these data and the data source to WHO; when possible, these are based on National Health Accounts, but alternative sources are used in many cases. Health spending estimates were extracted in current national currency units, deflated 2019 national currency units, and exchanged to 2019 US dollars. Deflator series and exchanges rates used were based on those reported in the IMF World Economic Outlook.[5]

We estimated domestic government spending on health by aggregating transfers from government domestic revenue for health purposes, compulsory prepayment, and social insurance contributions. Then, to estimate domestic prepaid private health spending, we aggregated voluntary prepayment, other domestic revenues from non-profit institutions serving the household, and other domestic revenues from corporations. All payments by households other than social insurance contributions were designated as out-of-pocket spending. To generate domestic health spending estimates in purchasing-power parity-adjusted (PPP) dollars, we multiplied the health spending fractions by GDP per capita measured in 2019 PPP dollars.

The extracted data were evaluated for quality. Some of the extracted data points were not tied to an underlying data source and were estimated using a diverse set or poorly defined methods. Furthermore, for a given country, some data varied substantially across time. To overcome these concerns, we used a spatiotemporal Gaussian process regression model to estimate health spending across time, country, and spending category.[10] To prevent data with insufficiently described estimation methods or without data source identification from substantially influencing our model estimation, each data point was assessed and assigned a weight between one and five using the point-specific metadata provided in the GHED. We based weights on metadata completeness, documented source information, and documented methods for estimation. The distribution of data points assigned to each of the five data quality scores, by country income group, is presented in the appendix. Our guidelines for assessing the metadata, a detailed description of how the weights were created, and additional information about the spatiotemporal Gaussian process regression model can also be found in the appendix.

S1.3 Estimating development assistance for health: 1990–2019

To track DAH, we relied on revenue data in financial statements, annual reports, budget documents, and project disbursement records reported by international development agencies such as the Organisation for Economic Co-operation and Development (OECD) Creditor Reporting System, the World Bank, the Global Fund, and major philanthropic entities like the Bill & Melinda Gates Foundation, Susan Thompson Buffet Foundation, and Rotary International.[11-20] We used budget and commitment data to generate estimates for recent years for which disbursement data were not yet available; for most channels data extends to 2017 or 2018 so that at most two years of estimation were needed.

Our DAH estimates tracked disbursements from the originating donor (called the source); to the development agency responsible for disbursing the funds to the recipient country (called the channel); to the recipient country. We used revenue and disbursement data to count only once the transfers between development agencies to limit double counting. In addition to reporting the source, channel, and recipient of DAH, spending was disaggregated into nine major health focus areas (as well as more detailed programme areas) by searching project titles and descriptions for keywords, as well as using other indicators in the data. Health focus areas included HIV/AIDS; malaria; tuberculosis; reproductive and maternal health; newborn and child health; non-communicable diseases; other infectious diseases; sector-wide approaches

and health system strengthening; and other. The “Other” category captured projects such as general support for a conference on the Sustainable Development Goals that were not allocated to any of the eight specific health focus areas; remaining funds for which no project descriptions were available were classified as “unallocable.” The list of keywords used to isolate relevant health projects for each of the health focus areas are included in the supplemental appendix Table S3.4.

While the majority of the methods used for tracking DAH have been described previously, the current estimates of DAH incorporated improvements in methodology such as leveraging additional project level descriptions from the Creditor Reporting System for the allocation of disbursements channeled through NGOs and ongoing refinement of our keyword search list.[21-25] For all DAH tracking, we include on funds that were transferred through major development agencies included in our research, as well as private foundation and non-governmental agencies for whom we have data. This definition of DAH excludes spending on basic bench science which may have down-stream effects on the development of new drugs.

S1.4 Reporting and uncertainty analysis

All inflation-adjusted health spending estimates are reported using 2019 prices. Future values are not discounted. We report health spending per capita in US dollars and purchasing-power parity-adjusted dollars and as a fraction of GDP. When not otherwise indicated, estimates are reported in 2019 US dollars. We report country spending estimates using 2019 Global Burden of Disease super-regions and 2019 World Bank income groups, regardless of whether a country changed, or is projected to change, income groups during the study period.[28,29] GBD regions can be seen in Figure S1.4 below. Rates were calculated to reflect each group, rather than the average of countries within the group, such that spending per capita estimates for an income group or region more heavily reflect rates in more populous countries. The uncertainty interval around each estimate was computed using the 2.5th and 97.5th percentiles of the 1,000 draws. All analyses were done using R (version 3.6.0), Stata (version 15.1), and Python (version 3.6). Table S1.4 presents all packages used for the analyses in this paper.

Table S1.4 Programming packages used for analyses

Packages used within R (version 3.6)					Packages used within Python (version 3.7.3)		Packages used within Stata (version 15.1)	
AFModel	doParallel	influence.ME	pacman	tidyr	json	xarray	egenmore	findname
argparse	dplyr	iterators	parallel	tidyverse	csv	pandas	xfill	grc1leg
arm	feather	lme4	plotly	TMB				
boot	foreach	lmtest	RColorBrewer	zoo				
Cairo	forecast	MASS	readstata13					
car	frontier	Matrix	readxl					
cowplot	GauProMod	micEcon	RMySQL					
data.table	ggplot2	MuMIn	scales					
DBI	gridExtra	ncdf4	shiny					
devtools	haven	ncdf4.helpers	stringr					

Figure S1.4 Global Burden of Disease super-regions and regions (source www.healthdata.org)

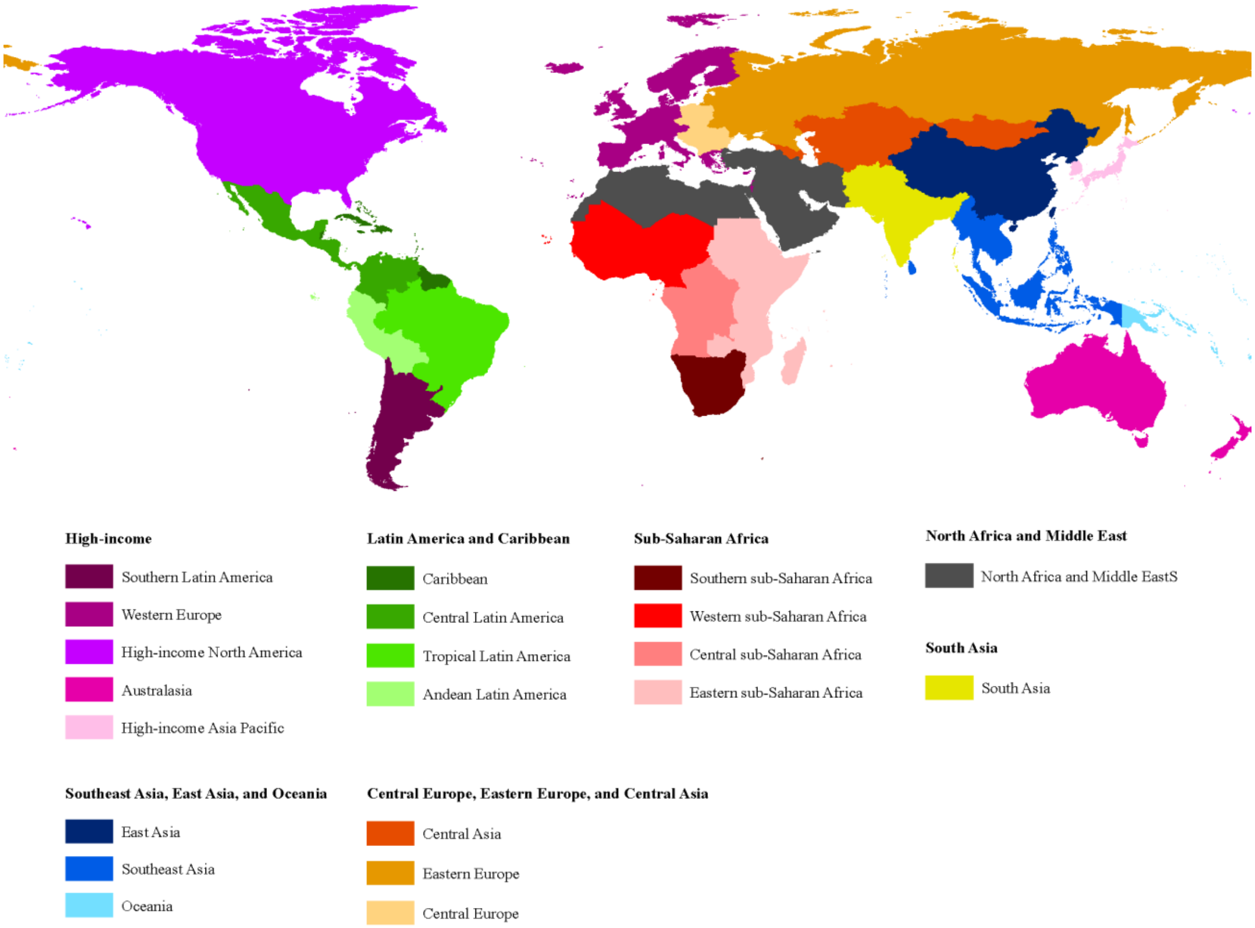

Table S1.5 Total Spending by disease

**Tuberculosis Total Spend**			
**GBD Super Region**	**Total Spending**	**Lower Bound**	**Upper Bound**
Southeast Asia, East Asia, and Oceania	1879.2	1580	2257.2
Central Europe, Eastern Europe, and Central Asia	3370.8	3057.8	3705.6
High-income	72.6	50.3	105.8
Latin America and Caribbean	518.3	458.9	581.8
North Africa and Middle East	326.7	289.5	372.6
South Asia	2166.7	1720.8	2769.2
Sub-Saharan Africa	2031.5	1902.4	2196.4
**Malaria Total Spend**			
**GBD Super Region**	**Total Spending**	**Lower Bound**	**Upper Bound**
Southeast Asia, East Asia, and Oceania	375.8	334.2	426.2
Central Europe, Eastern Europe, and Central Asia	8.9	7.2	11
High-income	0.9	0.5	1.5
Latin America and Caribbean	184.3	151.1	227.7
North Africa and Middle East	119.2	105.2	138.8
South Asia	267.3	223.1	324.7
Sub-Saharan Africa	3282.7	3082.9	3526
**HIV/AIDS Total Spend**			
**GBD Super Region**	**Total Spending**	**Lower Bound**	**Upper Bound**
Southeast Asia, East Asia, and Oceania	2231.2	1580.7	3159.6
Central Europe, Eastern Europe, and Central Asia	776.3	581.9	1037.3
High-income	446.9	325.9	616.5
Latin America and Caribbean	3288.4	2107.2	5313.3
North Africa and Middle East	490.1	301.3	764.9
South Asia	611.7	438.8	862.2
Sub-Saharan Africa	7660.6	6736.2	8883.6

References

1WHO | Global Health Expenditure Database. WHO. http://www.who.int/health-accounts/ghed/en/ (accessed Dec 23, 2017).

2OECD, Eurostat and World Health Organization. A System of Health Accounts 2011: Revised edition. Paris: OECD Publishing, 2017 https://read.oecd-ilibrary.org/social-issues-migration-health/a-system-of-health-accounts-2011_9789264270985-en (accessed March 7, 2019).

3The Database | Penn World Table version 9.0. Groningen Growth and Development Centre: Faculty of Economics and Business. https://www.rug.nl/ggdc/productivity/pwt/ (accessed Dec 21, 2018).

4DataBank | The World Bank. The World Bank. http://databank.worldbank.org/data/home.aspx (accessed Dec 21, 2018).

5International Monetary Fund. World Economic Outlook, October 2018: Challenges to Steady Growth. International Monetary Fund, 2018 https://www.imf.org/en/Publications/WEO/Issues/2018/09/24/world-economic-outlook-october-2018 (accessed Dec 21, 2018).

6Maddison Project Database 2018. Groningen Growth and Development Centre: Faculty of Economics and Business. https://www.rug.nl/ggdc/historicaldevelopment/maddison/releases/maddison-project-database-2018 (accessed Dec 21, 2018).

7National Accounts Main Aggregates Database. United Nations Statistics Division - National Health Accounts. https://unstats.un.org/unsd/snaama/Introduction.asp (accessed Dec 21, 2018).

8James SL, Gubbins P, Murray CJ, Gakidou E. Developing a comprehensive time series of GDP per capita for 210 countries from 1950 to 2015. *Population Health Metrics* 2012; **10**: 12.

9Global Health Expenditure Database. World Health Organization. http://apps.who.int/nha/database (accessed Dec 21, 2018).

10GBD 2015 Risk Factors Collaborators. Global, regional, and national comparative risk assessment of 79 behavioural, environmental and occupational, and metabolic risks or clusters of risks, 1990–2015: a systematic analysis for the Global Burden of Disease Study 2015. *The Lancet* 2016; **388**: 1659–724.

11Find a Grant. https%3a%2f%2fwww.theglobalfund.org%2fen%2fportfolio%2ffind%2f (accessed Dec 22, 2018).

12OECD Statistics. https://stats.oecd.org/ (accessed Dec 22, 2018).

13Malaria Operational Plans (MOPs) | PMI. https://www.pmi.gov/resource-library/mops/ (accessed Dec 22, 2018).

14Key figures: donor contributions & pledges. https://www.gavi.org/investing/funding/donor-contributions-pledges/ (accessed Dec 22, 2018).

15Trustees reports and financial statements - International Finance Facility for Immunisation. https://www.iffim.org/finance/trustees-reports-and-financial-statements/ (accessed Dec 22, 2018).

16Financial reports. https://www.gavi.org/investing/funding/financial-reports/ (accessed Dec 22, 2018).

17Disbursements and commitments. https://www.gavi.org/results/disbursements/ (accessed Dec 22, 2018).

18PCB Archive. http://www.unaids.org/en/aboutunaids/unaidsprogrammecoordinatingboard/pcbmeetingarchive (accessed Dec 22, 2018).

19Executive Board. /executive-board (accessed Dec 22, 2018).

20GB | Governing Body Documentation. http://apps.who.int/gb/ (accessed Dec 22, 2018).

21Ravishankar N, Gubbins P, Cooley RJ, *et al.* Financing of global health: tracking development assistance for health from 1990 to 2007. *The Lancet* 2009; **373**: 2113–24.

22Leach-Kemon K, Chou DP, Schneider MT, *et al.* The Global Financial Crisis Has Led To A Slowdown In Growth Of Funding To Improve Health In Many Developing Countries. *Health Affairs* 2012; **31**: 228–35.

23Dieleman JL, Graves C, Johnson E, *et al.* Sources and Focus of Health Development Assistance, 1990–2014. *JAMA* 2015; **313**: 2359–68.

24Dieleman JL, Graves CM, Templin T, *et al.* Global health development assistance remained steady in 2013 but did not align with recipients' disease burden. *Health Aff (Millwood)* 2014; **33**: 878–86.

25Dieleman JL, Templin T, Sadat N, *et al.* National spending on health by source for 184 countries between 2013 and 2040. *The Lancet* 2016; **387**: 2521–35.

26Micah AE, Zhao Y, Chen CS, *et al.* Tracking development assistance for health from China, 2000–2018. *BMJ Global Health* under review.

27Das Gupta P. Standardization and Decompostion of Rates: A User's Manual. U.S. Bureau of the Census, 1993 https://www.census.gov/content/dam/Census/library/publications/1993/demo/p23-186.pdf (accessed Dec 21, 2018).

28World Bank Country and Lending Groups – World Bank Data Help Desk. The World Bank. https://datahelpdesk.worldbank.org/knowledgebase/articles/906519-world-bank-country-and-lending-groups (accessed Dec 21, 2018).

29Abate KH, Abay SM, Abbafati C, *et al.* Global, regional, and national age-sex-specific mortality and life expectancy, 1950–2017: a systematic analysis for the Global Burden of Disease Study 2017. *The Lancet* 2018; **392**: 1684–735.

SECTION 2. TRACKING TOTAL HEALTH SPENDING AND ITS COMPONENTS

List of Tables and Figures

Table S2.1 Rules for assigning weight values to metadata

Table S2.2 Countries and income groups, number of assigned weights

Figure S2.1 Comparison of WHO GHED extracted and IHME ST-GPR THE in per capita space, 2000-2017

Figure S2.2 Comparison of WHO GHED extracted and IHME ST-GPR THE in per GDP space, 2000-2017

Figure S2.3 Currency conversion process diagram

S 2.1 Overview of GHED data cleaning process

We used Global Health Expenditure Database (GHED) data from the World Health Organization (WHO) to generate our estimates.[1] From the GHED, we extracted "Current health expenditure by revenues of health care financing schemes" for total health expenditure (THE), "Other revenues from households n.e.c" for out-of-pocket (OOP), "Gross Domestic Product" for GDP. We summed "Social insurance contributions" and "Transfers from government domestic revenue (allocated to health purposes)" for government health spending (GHE). We summed "Voluntary prepayment", "Other revenues from corporations n.e.c.", "Compulsory prepayment (Other, and unspecified, than FS.3)", and "Other revenues from NPISH n.e.c." for pre-paid private (PPP).

To ensure we used the best possible data from the GHED, we evaluated the metadata also provided by GHED to establish the reliability of the data. To do so, we downloaded the metadata from the GHED website for each data point for the five indicators. We used the metadata to decide how each given data point should be weighted, from 0 to 5, with 0 meaning drop, and 1 through 5 meaning keep and treated these weights as inverse variance weights.

To assign the weights, we established guidelines for the metadata that informed how the underlying data points should be weighted. We gave priority to factors such as complete, documented source information and penalized factors such as having been derived or estimated. Table S2.1 below describes the guidelines we created; any metadata that did not meet any of the disqualifying factors were given a value of 5 to reflect highest reliability.

We used the four primary metadata variables from the GHED database: data type, method of estimation, comments, and sources. We applied the guidelines to each unique set of metadata across these four variables. For a subset of data points, the metadata indicated that the data point was the sum of other data points. In these cases, if the indicator was a sub-indicator of GHES, PPP, or OOP, we assigned the data point a value of 2 to reflect that even though we could not determine if the sub-components existed, as they are not reported in GHED, we did not feel that being a sum warranted dropping the data. We assigned the summed GHES, PPP, and OOP indicators the lowest value of its sub-indicators. If the summed data point was THE, however, we assigned the data point the lowest value of its sub-components, the summed GHES, PPP, and OOP indicators.

After designating each of the 3,730 unique sets of metadata a value weight, we applied these weights to the underlying data points. In total, we had 30,590 data points, as multiple data points shared the same unique set of metadata. Once the weights were applied to the data, we reassigned all high-income country data points that were a 0 based on the metadata to 3. We made this change to reflect that high-income countries have higher quality data and thus should not be dropped, but should also not be given the

highest weight value. The high-income classification comes from the World Bank.[2] For all countries across all years of data their relative weighting scores can be seen in Table S2.2.

Each component of health expenditure was aggregated for all component-year-country combinations by taking component values (downloaded in current national unit); dividing by GHED reported GDP for the corresponding year-country (also in current national unit) to convert each component into its fraction of GDP; and finally multiplying those fractions by IHME GDP series measured in USD 2019. (Note: though we harvested and cleaned metadata for THE and GDP, we do not model THE (we model GHE, PPP, OOP and then aggregate) or use the WHO's GDP data since we have our own methods with multiple sources for determination of our value).

Each component of health expenditure was then converted into three spending metrics for the time period between 2000-2017, measured in purchasing power parity (PPP): health spending per capita ($), annualized rate of change in health spending per capita 2000-2017 (%), and annualized rate of change in health spending 2000-2017 (%). Then, each component of health expenditure was aggregated and converted into six spending metrics for the time period between 2018-2050, measured in PPP: health spending per capita ($), annualized rate of change in health spending per capita 2018-2050 (%), annualized rate of change in health spending 2018-2050 (%), Government health spending per capita ($), difference between GHEpc (government health expenditure per capita) reference scenario and better scenario 1, 2050 ($), and difference between GHEpc reference scenario and better scenario 2, 2050 ($).

Formula for calculating AROC (Annualized rate of change):

If you apply this growth rate n times (where n is the number of steps from starting to ending year) to the value in the starting year, you will get the value in the ending year. For example –

$$\underset{1990\rightarrow 2019}{\text{AROC}} = \left(\frac{value\ in\ 2019}{value\ in\ 1990}\right)^{\frac{1}{2019-1990}} - 1$$

Table S2.1 Rules for assigning weight values to metadata

Data type	Methods of estimation	Sources and/or Comments	Weight
**Blank**			0
**Estimated**			0
	Derived by applying the sum of the components		Lowest weight of the components
	Interpolated but with additional information		2
	Method description is unclear or provides very little information		0
	Time trend interpolation		1
	Uses data from other countries		0

Data type	Methods of estimation	Sources and/or Comments	Weight
		Abstract that's not from something documented	1 or method (whichever is bigger)
		Adjusted	0 or method (whichever is bigger)
		Adjusted using something	2 or method (whichever is bigger)
		Any suggestion that the WHO is unclear or unsure about some aspect of the data point's metadata	0 or method (whichever is bigger)
		Approximation	0 or method (whichever is bigger)
		Assumption	0 or method (whichever is bigger)
		Both blank	0 or method (whichever is bigger)
		Both with no intelligible information	0 or method (whichever is bigger)
		Budget address	1 or method (whichever is smaller)
		Calculation was used to generate the estimate	1 or method (whichever is bigger)
		Consultation/contact (without an additional documented source)	1 or method (whichever is smaller)
		Consultations with additional source, but no specifics and just consult is documented	1 or method (whichever is bigger)
		Currency conversion	1 or method (whichever is bigger)
		Data delivered/provided/reported by (a non-documented source)	1 or method (whichever is smaller)
		Data provided but not clear by whom, with an additional source if additional source is not documented	1 or method (whichever is smaller)
		Derived	0 or method (whichever is bigger)
		Estimated based on	1 or method (whichever is bigger)
		Estimation	0 or method (whichever is bigger)
		Excludes (if it excludes what we do want)	0 (supersedes method)
		Extrapolated	0 or method (whichever is bigger)
		Forecasted	0 or method (whichever is bigger)
		Government department, no explicit documented source	1 or method (whichever is bigger)
		Government ministry, but no explicit documented source	1 or method (whichever is bigger)
		Includes (if it includes what we don't want)	0 (supersedes method)
		Inferred	0 or method (whichever is bigger)
		Interpolation	0 or method (whichever is bigger)
		Missing (if missing something that should be included)	0 (supersedes method)
		Modified	0 or method (whichever is bigger)

Data type	Methods of estimation	Sources and/or Comments	Weight
		Modified from something/modified using something	2 or method (whichever is bigger)
		Needs assessment	1 or method (whichever is smaller)
		Needs discussion	1 or method (whichever is smaller)
		Needs validation	1 or method (whichever is smaller)
		Needs verification	1 or method (whichever is smaller)
		Only provides hint of a source	1 or method (whichever is smaller)
		Projected	0 or method (whichever is bigger)
		Provides only a vague term that does not provide adequate information to infer or determine what the source is	0 or method (whichever is bigger)
		Reply	1 or method (whichever is smaller)
		Response	1 or method (whichever is smaller)
		Speech	1 or method (whichever is smaller)
		Sum of	2 (except for THE, which is lowest weight of components)
		Total of	3 (except for THE, which is lowest weight of components)
		Underestimated	0 (supersedes method)
		Unpublished	1 or method (whichever is smaller)
		Validated figures, but without specifics	2 or method (whichever is bigger)
		Weights	0 or method (whichever is bigger)

Table S2.2 Countries and income groups, number of assigned weights

Country	0	1	2	3	4	5
**Afghanistan**	162	0	0	0	0	0
**Albania**	162	0	0	0	0	0
**Algeria**	162	0	0	0	0	0
**Andorra**	0	0	0	108	0	0
**Angola**	162	0	0	0	0	0
**Antigua and Barbuda**	63	0	0	98	0	0
**Argentina**	153	0	0	9	0	0
**Armenia**	162	0	0	0	0	0
**Australia**	0	0	0	162	0	0
**Austria**	0	0	0	162	0	0
**Azerbaijan**	126	0	0	0	0	0
**Bahamas**	0	0	0	162	0	0
**Bahrain**	9	0	0	153	0	0
**Bangladesh**	162	0	0	0	0	0

**Barbados**	36	0	0	126	0	0
**Belarus**	126	0	0	0	0	0
**Belgium**	0	0	0	126	0	0
**Belize**	162	0	0	0	0	0
**Benin**	162	0	0	0	0	0
**Bhutan**	162	0	0	0	0	0
**Bolivia (Plurinational State of)**	162	0	0	0	0	0
**Bosnia and Herzegovina**	144	0	0	0	0	0
**Botswana**	162	0	0	0	0	0
**Brazil**	162	0	0	0	0	0
**Brunei Darussalam**	0	0	0	162	0	0
**Bulgaria**	162	0	0	0	0	0
**Burkina Faso**	162	0	0	0	0	0
**Burundi**	162	0	0	0	0	0
**Cabo Verde**	162	0	0	0	0	0
**Cambodia**	162	0	0	0	0	0
**Cameroon**	162	0	0	0	0	0
**Canada**	0	0	0	162	0	0
**Central African Republic**	162	0	0	0	0	0
**Chad**	162	0	0	0	0	0
**Chile**	108	0	0	54	0	0
**China**	162	0	0	0	0	0
**Colombia**	162	0	0	0	0	0
**Comoros**	162	0	0	0	0	0
**Congo**	162	0	0	0	0	0
**Cook Islands**	162	0	0	0	0	0
**Costa Rica**	162	0	0	0	0	0
**Croatia**	90	0	0	72	0	0
**Cuba**	162	0	0	0	0	0
**Cyprus**	0	0	0	162	0	0
**Czechia**	54	0	0	108	0	0
**Côte d'Ivoire**	162	0	0	0	0	0
**Democratic Republic of the Congo**	162	0	0	0	0	0
**Denmark**	0	0	0	144	0	0
**Djibouti**	162	0	0	0	0	0
**Dominica**	162	0	0	0	0	0
**Dominican Republic**	162	0	0	0	0	0
**Ecuador**	162	0	0	0	0	0
**Egypt**	162	0	0	0	0	0
**El Salvador**	162	0	0	0	0	0
**Equatorial Guinea**	80	0	0	64	0	0
**Eritrea**	162	0	0	0	0	0
**Estonia**	54	0	0	108	0	0

**Eswatini**	162	0	0	0	0	0
**Ethiopia**	162	0	0	0	0	0
**Fiji**	162	0	0	0	0	0
**Finland**	0	0	0	162	0	0
**France**	0	0	0	162	0	0
**Gabon**	162	0	0	0	0	0
**Gambia**	162	0	0	0	0	0
**Georgia**	144	0	0	0	0	0
**Germany**	0	0	0	162	0	0
**Ghana**	162	0	0	0	0	0
**Greece**	0	0	0	162	0	0
**Grenada**	162	0	0	0	0	0
**Guatemala**	162	0	0	0	0	0
**Guinea**	162	0	0	0	0	0
**Guinea-Bissau**	162	0	0	0	0	0
**Guyana**	162	0	0	0	0	0
**Haiti**	162	0	0	0	0	0
**Honduras**	162	0	0	0	0	0
**Hungary**	81	0	0	81	0	0
**Iceland**	0	0	0	162	0	0
**India**	162	0	0	0	0	0
**Indonesia**	162	0	0	0	0	0
**Iran (Islamic Republic of)**	162	0	0	0	0	0
**Iraq**	162	0	0	0	0	0
**Ireland**	0	0	0	162	0	0
**Israel**	0	0	0	162	0	0
**Italy**	0	0	0	144	0	0
**Jamaica**	162	0	0	0	0	0
**Japan**	0	0	0	162	0	0
**Jordan**	162	0	0	0	0	0
**Kazakhstan**	144	0	0	0	0	0
**Kenya**	162	0	0	0	0	0
**Kiribati**	162	0	0	0	0	0
**Kuwait**	0	0	0	162	0	0
**Kyrgyzstan**	144	0	0	0	0	0
**Lao People's Democratic Republic**	162	0	0	0	0	0
**Latvia**	99	0	0	63	0	0
**Lebanon**	162	0	0	0	0	0
**Lesotho**	126	0	0	0	0	0
**Liberia**	162	0	0	0	0	0
**Libya**	162	0	0	0	0	0
**Lithuania**	108	0	0	54	0	0
**Luxembourg**	0	0	0	162	0	0

**Madagascar**	162	0	0	0	0	0
**Malawi**	162	0	0	0	0	0
**Malaysia**	162	0	0	0	0	0
**Maldives**	162	0	0	0	0	0
**Mali**	162	0	0	0	0	0
**Malta**	7	0	0	119	0	0
**Marshall Islands**	162	0	0	0	0	0
**Mauritania**	162	0	0	0	0	0
**Mauritius**	162	0	0	0	0	0
**Mexico**	162	0	0	0	0	0
**Micronesia (Federated States of)**	162	0	0	0	0	0
**Monaco**	0	0	0	108	0	0
**Mongolia**	162	0	0	0	0	0
**Morocco**	162	0	0	0	0	0
**Mozambique**	162	0	0	0	0	0
**Myanmar**	162	0	0	0	0	0
**Namibia**	162	0	0	0	0	0
**Nauru**	153	0	0	9	0	0
**Nepal**	162	0	0	0	0	0
**Netherlands**	0	0	0	162	0	0
**New Zealand**	0	0	0	162	0	0
**Nicaragua**	162	0	0	0	0	0
**Niger**	162	0	0	0	0	0
**Nigeria**	162	0	0	0	0	0
**Niue**	147	3	0	0	0	12
**Norway**	0	0	0	162	0	0
**Oman**	63	0	0	99	0	0
**Pakistan**	162	0	0	0	0	0
**Palau**	144	0	0	18	0	0
**Panama**	162	0	0	0	0	0
**Papua New Guinea**	162	0	0	0	0	0
**Paraguay**	162	0	0	0	0	0
**Peru**	162	0	0	0	0	0
**Philippines**	162	0	0	0	0	0
**Poland**	81	0	0	81	0	0
**Portugal**	0	0	0	162	0	0
**Qatar**	0	0	0	162	0	0
**Republic of Korea**	9	0	0	153	0	0
**Republic of Moldova**	162	0	0	0	0	0
**Romania**	162	0	0	0	0	0
**Russian Federation**	120	0	0	24	0	0
**Rwanda**	162	0	0	0	0	0
**Saint Kitts and Nevis**	99	0	0	63	0	0

**Saint Lucia**	162	0	0	0	0	0
**Saint Vincent and the Grenadines**	162	0	0	0	0	0
**Samoa**	162	0	0	0	0	0
**San Marino**	0	0	0	108	0	0
**Sao Tome and Principe**	162	0	0	0	0	0
**Saudi Arabia**	36	0	0	126	0	0
**Senegal**	162	0	0	0	0	0
**Serbia**	162	0	0	0	0	0
**Seychelles**	126	0	0	36	0	0
**Sierra Leone**	162	0	0	0	0	0
**Singapore**	0	0	0	162	0	0
**Slovakia**	63	0	0	99	0	0
**Slovenia**	0	0	0	144	0	0
**Solomon Islands**	162	0	0	0	0	0
**South Africa**	162	0	0	0	0	0
**South Sudan**	162	0	0	0	0	0
**Spain**	0	0	0	162	0	0
**Sri Lanka**	162	0	0	0	0	0
**Sudan**	162	0	0	0	0	0
**Suriname**	162	0	0	0	0	0
**Sweden**	0	0	0	162	0	0
**Switzerland**	0	0	0	162	0	0
**Syrian Arab Republic**	162	0	0	0	0	0
**Tajikistan**	162	0	0	0	0	0
**Thailand**	162	0	0	0	0	0
**Timor-Leste**	160	0	0	0	0	2
**Togo**	162	0	0	0	0	0
**Tonga**	162	0	0	0	0	0
**Trinidad and Tobago**	54	0	0	108	0	0
**Tunisia**	162	0	0	0	0	0
**Turkey**	162	0	0	0	0	0
**Turkmenistan**	162	0	0	0	0	0
**Tuvalu**	162	0	0	0	0	0
**Uganda**	162	0	0	0	0	0
**Ukraine**	162	0	0	0	0	0
**United Arab Emirates**	0	0	0	162	0	0
**United Kingdom**	0	0	0	162	0	0
**United Republic of Tanzania**	162	0	0	0	0	0
**United States Virgin Islands**	0	0	0	162	0	0
**United States of America**	0	0	0	162	0	0
**Uruguay**	108	0	0	54	0	0
**Uzbekistan**	162	0	0	0	0	0
**Vanuatu**	162	0	0	0	0	0

**Venezuela (Bolivarian Republic of)**	145	0	0	8	0	0
**Viet Nam**	162	0	0	0	0	0
**Yemen**	162	0	0	0	0	0
**Zambia**	162	0	0	0	0	0
**Zimbabwe**	162	0	0	0	0	0
**GBD High-income**	385	0	0	5015	0	0
**High income**	1654	0	0	7453	0	0
**Low income**	4698	0	0	0	0	0
**Lower-middle income**	7396	0	0	0	0	2
**Upper-middle income**	8787	0	0	114	0	0

S2.2 Statistical model to fill missingness in health expenditure variables

After the cleaning up of the data previously described above, we used Spatiotemporal Gaussian process regression (ST-GPR) in order to predict and fill out the missingness that existed in the resulting health expenditure dataset. ST-GPR is a stochastic modeling technique designed to detect signals amidst noisy data. It also serves as a powerful tool for interpolating non-linear trends. Unlike classical linear models that assume that the trend underlying data follows a definitive functional form, GPR assumes that the specific trend of interest follows a Gaussian process, where each point can be estimated with a mean and covariance function.

The first step to implementing ST-GPR is to identify relevant covariates that would be helpful in predicting each health expenditure variable of interest. Using the following set of covariates, we estimated the first stage of the process (space-time) in order to predict and fill up the dependent variables. The covariates used are:

a) All-sector government expenditure per capita, logged
b) Lag Distributed Income, logged
c) Healthcare Access and Quality Index, logged
d) Proportion of total population above the age of 65, logit transformed.

The dependent variables were logs of GHE per capita, PPP per capita and OOP per capita. We also run these models sequentially so that the following model can include the previous component as a covariate. GHE per capita uses only the above covariates, while PPP per capita additionally included GHE per capita as a covariate, and OOP per capita additionally uses GHE per capita and OOP per capita as covariates.

Given the weight of data, we were able to adjust the weight of each data point that contributed to the likelihood function of the Gaussian process, by inflating the pointwise variance for data points with lower weights. For missing data points, the resulting uncertainty was determined by region specific estimates. The final resulting dataset was a complete set of GHE, PPP and OOP per capita estimates for 195 countries from 1995 through 2017, where the uncertainty around each point was constructed by simulating from a normal distribution. Goodness-of-fit was determined using out-of-sample validation and choosing the models with the best performing set of hyperparameters. Overall, ST-GPR is a stochastic modeling technique that performs well at detecting signals among datasets that are noisy. It deviates from classic linear models that assume trend of its underlying data must follow a definitive functional form. Instead, GPR assumes the trend must follow a Gaussian Process form, having a covariance function and a pointwise mean. For the distribution of the Gaussian process, we used a Matérn covariance matrix. GPRs were run repeatedly to ensure that every country-year estimate per metric had 1,000 draws. This ST-GPR process follows the same form of that found in the Global Burden of Disease Study 2017 appendix.[3] This analysis was conducted in the following programs: Stata (version 13.1) and R (version 3.6.0).

Figure S2.1 below shows the comparison of WHO GHED extracted data and IHME ST-GPR THE in GDP per capita space from 2000-2017. Figure S2.2 shows the comparison of WHO GHED extracted data and IHME ST-GPR THE in per GDP space from 2000-2017.

Figure S2.1 Comparison of WHO GHED extracted and IHME ST-GPR THE *in per capita* space, 2000-2017

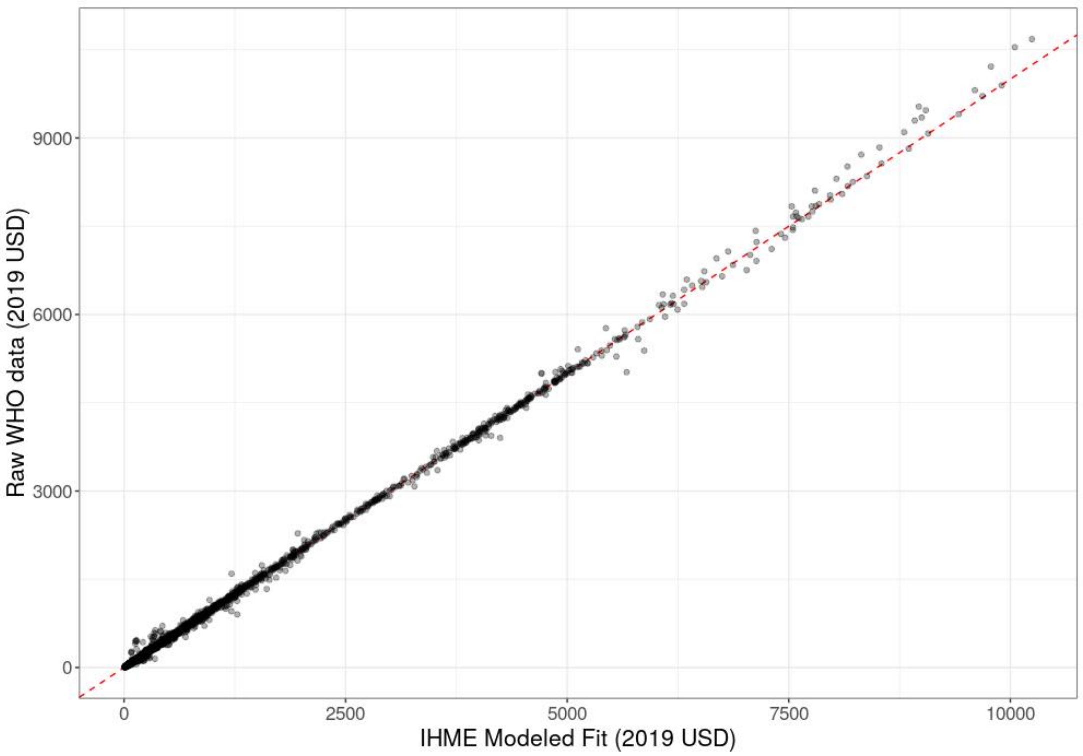

Figure S2.2 Comparison of WHO GHED extracted and IHME ST-GPR THE *in per GDP* space, 2000-2017

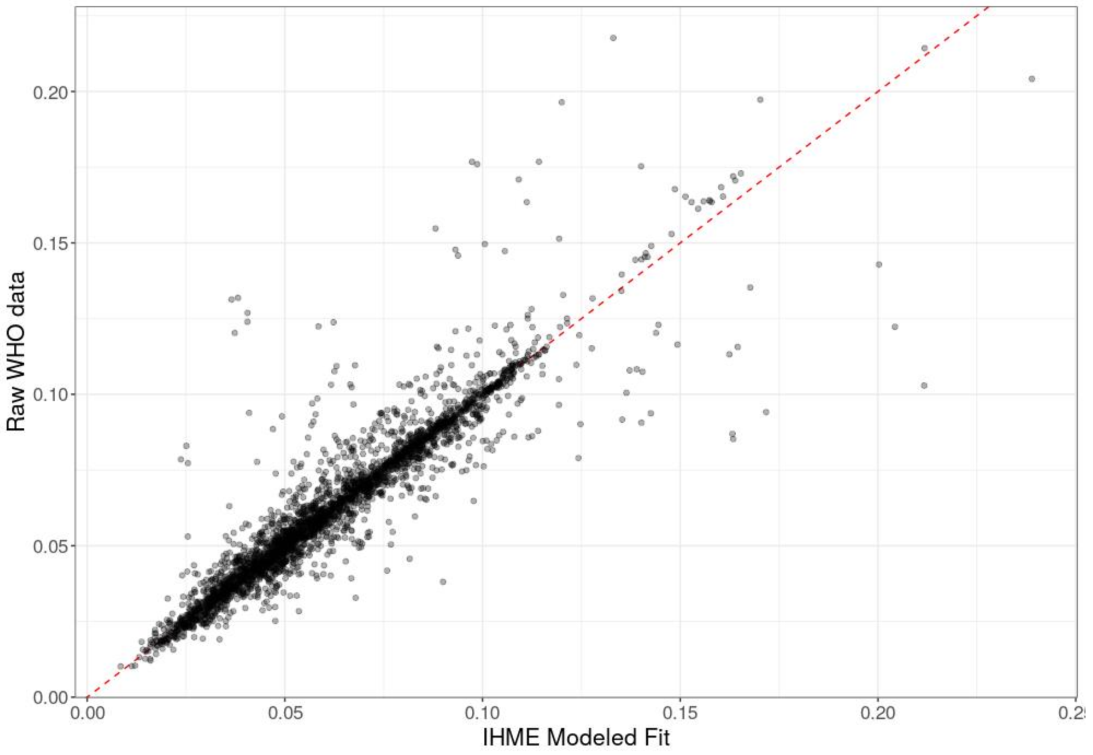

S2.3 Currency exchange and deflation

To convert a metric from real 2019 USD to real 2019 PPP, the following steps were taken. Real USD was exchanged to real LCU (local currency units) by multiplying with country-year specific USD to LCU exchange rates. Then, values were converted from real LCU to real PPP (purchasing-power parity-adjusted dollars) using the country-year specific LCU to PPP conversion series. Any currency deflation from nominal to real currency units or between real currency units in different base years were performed after converting either USD or PPP to LCU, and used country-specific deflator series. All deflators and exchange rates were extracted from the World Bank[4], International Monetary Fund[5], Penn World Tables[6], the United Nations National Accounts[7], and the World Health Organization[8], and were imputed to provide a complete series for each of the variables between 1950 and 2019. We then used several models including ordinary least-squares regression and mixed effects models, to complete each source series from 1950 to 2019.

Figure S2.3 shows the currency conversion process used in this work.

Figure S2.3 Currency conversion process diagram

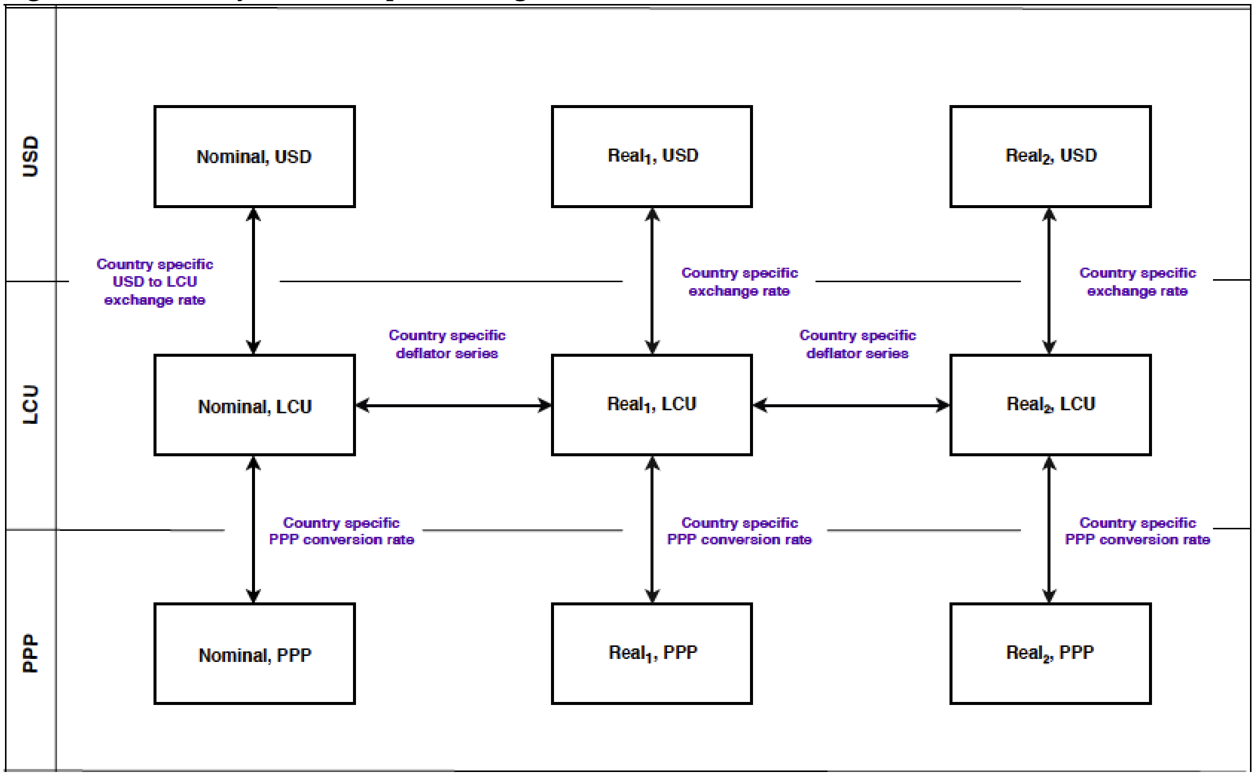

References

1 Global Health Expenditure Database. World Health Organization. http://apps.who.int/nha/database (accessed Jan 7, 2020).

2 World Bank Country and Lending Groups – World Bank Data Help Desk. World Bank. https://datahelpdesk.worldbank.org/knowledgebase/articles/906519-world-bank-country-and-lending-groups (accessed Oct 31, 2019).

3 Stanaway JD, Afshin A, Gakidou E, *et al.* Global, regional, and national comparative risk assessment of 84 behavioural, environmental and occupational, and metabolic risks or clusters of risks for 195 countries and territories, 1990–2017: a systematic analysis for the Global Burden of Disease Study 2017. *The Lancet* 2018; **392**: 1923–94.

4 World Development Indicators (WDI) | Data Catalog. https://datacatalog.worldbank.org/dataset/world-development-indicators (accessed Oct 31, 2019).

5 World Economic Outlook Database October 2018.https://www.imf.org/external/pubs/ft/weo/2018/02/weodata/index.aspx (accessed Oct 31, 2019).

6 Robert C. Feenstra RI. Penn World Table 9.0. 2016. DOI:10.15141/S5J01T.

7 National Accounts - Analysis of Main Aggregates (AMA). https://unstats.un.org/unsd/snaama/Index (accessed Oct 31, 2019).

8 WHO | Global Health Observatory (GHO) data. WHO. http://www.who.int/gho/en/ (accessed Oct 31, 2019).

SECTION 3. TRACKING DEVELOPMENT ASSISTANCE FOR HEALTH FROM TRADITIONAL DONORS

List of Figures

Figure S3.1 Comparing CRS commitments, CRS disbursements, and DAC commitments

Figure S3.2 CRS disbursement to commitment ratio and cutoff points by donor agency

Figure S3.3 One- to six-year disbursement schedules for bilateral channels

Figure S3.4 DAH as a percentage of corresponding budget data by bilateral agency

Figure S3.5 Malaria DAH to program areas as assigned by keyword search and PMI reports

Figure S3.6 Comparison of world Bank IDA Replenishment and DAC

Figure S3.7 Disbursements by the African Development Bank

Figure S3.8 Disbursements by Asian Development Bank

Figure S3.9 Disbursements by Inter-American Development Bank

Figure S3.10 Contributions received by the Global Fund to Fight AIDS, Tuberculosis and Malaria

Figure S3.11 The Global Fund to Fight AIDS, Tuberculosis and Malaria's commitments and disbursements

Figure S3.12 Gavi's income and disbursements

Figure S3.13 Total revenue received by non-governmental organizations

Figure S3.14 Expenditure by non-governmental organizations

Figure S3.15 In-kind contributions by loan- and grant-making DAH channels of assistance

List of Tables

Table S3.1. Summary of primary data sources and databases

Table S3.2. Data sources received via personal correspondence

Table S3.3. Additional data sources, databases and model choices used for preliminary estimates of DAH

Table S3.4. Terms for keyword searches

Table S3.5. Additional health focus area categorizations

Table S3.6. World Bank's health sector and theme codes

Table S3.7 Summary of data sources for the regional development banks

Table S3.8 Summary of US non-governmental organizations in the study

Table S3.9 Summary of data sources for calculating in-kind contributions

Box 3.1. EXAMPLE - Australia's primary and additional data sources

Box 3.2. EXAMPLE. Post-keyword search weighting

Box 3.3. EXAMPLE. Australia's CRS disbursement to commitment ratio and cutoff year

Box 3.4. EXAMPLE. Australia's one- to six-year disbursement schedules

Box 3.5. EXAMPLE. Australia's DAH as a percentage of corresponding budget data

S3.1 Overview

We summarize the original methodology as well as updates for this year's estimates below. A more detailed description of the original methodology used to obtain the estimates can be found in Dieleman et al.[1] All known, systematically reported, available data on health-related disbursements and expenditures were extracted, as well as income and revenue from existing project databases, annual reports, and audited financial statements. The channels included and the corresponding data sources are summarized in Table S3.1. Data sources obtained via personal correspondence are summarized in Table S3.2.

DAH for bilateral agencies included all health-related disbursements from bilateral donor agencies, excluding funds that they transferred to any of the other channels we tracked in order to avoid double-counting. This information was extracted from the Creditor Reporting System (CRS) and Development Assistance Committee (DAC) databases of the Development Assistance Committee of the Organisation for Economic Co-operation and Development (OECD-DAC). In some cases, donor agencies did not report disbursement data to the CRS. A method for predicting disbursements from commitment data was implemented to address this challenge. For detailed description of this method see Tracking Development Assistance for Health from Bilateral Aid Agencies and the European Commission section below as well as in Dieleman et al. [1]

For other grant- and loan-making institutions, annual disbursements on health grants and loans were similarly included, excluding transfers to any other channels and ignoring any repayments on outstanding debts. For a more detailed description of this process see Dieleman et al.[1] The annual disbursements for grant- and loan-making institutions only reflect the financial transfers made by these agencies. Therefore, in-kind transfers from these institutions in the form of staff time for providing technical assistance and the costs of managing programs were estimated separately.[1]

Estimates of DAH for the United Nations (UN) agencies included annual expenditures on health both from their core budgets and from voluntary contributions. Calculating DAH for the United Nations Children's Fund (UNICEF) involved estimating the fraction of its total expenditure spent on health prior to 2001.[1]

Non-governmental organizations' (NGOs) DAH estimates utilized data from US government sources, GuideStar Research Fundamental Plus dataset and a survey of health expenditure for a sample of NGOs to estimate DAH from US-based and internationally based NGOs receiving support from the US government. We were unable to include other NGOs due to the lack of audited and comparable data.

The database also included an analysis of the composition of health funding by recipient country, health focus area and program area. In this round of updates to the database we incorporated improvements in methodology such as leveraging additional project level descriptions from the Creditor Reporting System for the allocation of disbursements channeled through NGOs and ongoing refinement of our keyword search list. All methodological updates made are detailed in their relevant channel sub-section below. The improvements to our health focus area keyword search terms are detailed in Table S3.4.

For many channels, reporting-time lags prevent primary disbursement data for the most recent year(s). For those years, the values of DAH were predicted, using channel-specific time trends. The methods employed to obtain these predictions are summarized in Table S3.3. In general, these methods depend on data availability. The estimates are based on channel-specific budget, commitment, and appropriations data, and in many cases assume the most recent disbursement patterns persist. Due to the lack of more detailed disaggregated data, estimates for the most recent year are not provided for recipient countries.

We predicted ebola funding in 2018-2019 for bilateral sources and the European Commission by assuming that 2018 and 2019 ebola funding was equal to 2017 ebola funding.

In addition, for SWAp/HSS funding, we allocated SWAP/HSS projects with multiple health focus areas identified by a proportional allocation based on the relative proportions of the project going to the various health focus areas.

For countries that only began existing in certain years, we backcasted DAH in years before their existence as follows. For countries that split off from parent countries, we calculated a three-year average ratio of child country DAH received to parent country DAH received. In years before the child country split off, DAH received by the parent country would have included DAH received in the region that would split off to become the child country. Therefore, we reallocated funding from the parent country, in all years before the child country split off, adding this proportion of the parent country's DAH to the child country's DAH and subtracting out this value from the parent country's DAH. By this method, total annual DAH between the parent and child country do not change, but the allocation of funding between the parent country and child country change. For any country that ceased to exist (such as former Yugoslavia and former USSR) and that had observed DAH received in certain years, we split the funding equally among its new constituent countries.

S3.1.1 Currency exchange and deflation

All results are presented in real 2019 US dollars. All disbursement sequences were converted into real 2019 US dollars by taking disbursements in nominal US dollars in the year of disbursement and adjusting these sequences into real 2019 US dollars using US gross domestic product (GDP) deflators[99]. Analyses were conducted in Stata (versions 13.1 or 15.1).

Table S3.1. Summary of primary data sources and databases

Channel	Source
**Bilateral agencies**	OECD-DAC and CRS databases[2]
**European Commission**	OECD-DAC and CRS databases[3]
**Joint United Nations Programme on HIV/AIDS (UNAIDS)**	Financial reports and audited financial statements[4]
**United Nations Children's Fund (UNICEF)**	Financial reports and audited financial statements[5–7]
**United Nations Population Fund (UNFPA)**	Financial reports and audited financial statements[8]
**UNITAID**	Project level data from IATI database shared through correspondence, financial reports and audited financial statements[9]
**Pan American Health Organization (PAHO)**	Financial reports and audited financial statements[10]
**World Health Organization (WHO)**	Financial reports and audited financial statements[11]
**World Bank**	Online project database and correspondence[12,13]
**African Development Bank (AfDB)**	Correspondence and compendium of statistics[14,15]
**Asian Development Bank (ADB)**	Online project database and correspondence[81,98]
**Inter-American Development Bank (IDB)**	Online project database and correspondence[16,17]
**Gavi, the Vaccine Alliance**	Online project database, cash received database, International Finance Facility for Immunisation (IFFIm) annual reports, Advance Market Commitment for Pneumococcal Vaccines (AMC) annual reports, and annual reports[18–21]
**The Global Fund to Fight AIDS, Tuberculosis and Malaria (GFATM)**	Online grant database, contributions report and annual reports[22–24]
**Coalition for Epidemic Preparedness and Innovation (CEPI)**	Project level data through correspondence

**NGOs registered in the US**	United States Agency for International Development (USAID) Report of Voluntary Agencies (VolAg), tax filings, annual reports, financial statements, RED BOOK Expanded Database, and WHO's Model List of Essential Medicines, Guidestar Research Fundamentals Plus dataset[25–29]
**Bill & Melinda Gates Foundation (BMGF)**	Online grant database, IRS 990 tax forms, and correspondence[30,31]
**Other private US foundations**	Foundation Center's grants database[32]
**Wellcome Trust**	Project level data through correspondence
**European Economic Area**	Online project database[33] and correspondence[34]
**China**	Financial reports and yearbooks

Table S3.2. Data sources received via personal correspondence

Channel	Data received
**World Bank**	Health project-level disbursement data, 1990 – August 2018[13]
**BMGF**	Health disbursement data, 2018[31]
**CEPI**	Health disbursement data, 2019
**IDB**	Health project-level loan disbursement data, 2019[17]
**AfDB**	Health project-level disbursement data, 2002 – August 2019[35]
**ADB**	Health project-level disbursement data, 1990 – 6 November 2019[98]
**UNITAID**	Health project-level disbursement data, 2007 – 2019[36]
**UAE bilateral**	UAE Foreign Assistance in Health 1990 - 2008[37]
**European Economic Area**	Health project-level disbursement data, 2007-2019

Table S3.3. Additional data sources, databases and model choices used for preliminary estimates of DAH

Channel	Data source	Variables used	Years of budget data used for modeling*	Years underlying DAH data not available; thus modeled*	Model used
**National agencies**					
**Australia**	Australia's International Development Assistance (2008-2019); Australia's Overseas Aid Program (1998-2008)[38,39]	Health official development assistance (ODA): International development assistance budget	1998-2019	2018-2019	Weighted average of actual DAH/budgeted DAH
**Austria**	Austria Federal Ministry of Finance budget[40]	General ODA: Federal ODA budget	2007-2019	2018-2019	Weighted average of

					DAH/budgeted ODA
**Belgium**	Project Budget General – general expenses[41]	General ODA: Foreign affairs, foreign trade development and cooperation	2000-2019	2018-2019	Weighted average of DAH/budgeted ODA
**Canada**	Canadian International Development Agency – Report on Plans and Priorities[42]	General ODA: Financial summary – planned spending	1996-2019	2018-2019	Weighted average of DAH/budgeted ODA
**Denmark**	Danish Ministry of Foreign Affairs Budget[43]	General ODA: Budgeted expenditures on overseas development assistance	2000-2019	2018-2019	Weighted average of DAH/budgeted ODA
**European Commission**	General budget[44]	Data not used as they were inconsistent with disbursements	–	2018-2019	Based on weighted average of trends in member countries
**Finland**	Document Assembly in budget years 1998-2019[45]	General ODA: Ministry of Foreign Affairs' administrative appropriations, international development	2002-2019	2018-2019	Weighted average of DAH/budgeted ODA
**France**	Budget and Financial documents[46,47]	General ODA: aggregated project data; Total ODA	2009-2019	2018-2019	Weighted average of DAH/budgeted ODA
**Germany**	Plan of the Federal Budget[48]	General ODA: Development expenditure	2001-2019	2018-2019	Weighted average of DAH/budgeted ODA
**Greece**	Ministry of Finance Budget (2013-2018); OECD Data (1996-2019)[2,49,50]	General ODA; ODA commitments	1996-2019	2018-2019	Weighted average of DAH/budgeted ODA
**Ireland**	Department of Finance – budget 2000-2004; Estimates for Public Services and Summary Public Capital Programme, 2005-2019 [51]	General ODA: Summary of adjustments to gross current estimates – international co-operation	2002-2019	2018-2019	Weighted average of DAH/budgeted ODA

**Italy**	The Italian Agency for Development Cooperation[52]	General ODA: Net development corporation	2007-2019	2018-2019	Weighted average of DAH/budgeted ODA
**Japan**	Highlights of the Budget for FY1999-2019[53]	General ODA: Major budget expenditures	2003-2019	2018-2019	Weighted average of DAH/budgeted ODA
**Korea, South**	ODA Korea comprehensive implementation plan[54]	General ODA: Plan for international development cooperation	2008-2019	2018-2019	Weighted average of DAH/budgeted ODA
**Luxembourg**	State Budget[55]	General ODA: Ministry of Foreign Affairs – budgeted international development cooperation and humanitarian aid	2001-2019	2018-2019	Weighted average of DAH/budgeted ODA
**Netherlands**	Netherlands International Cooperation Budget (2001-2019)	General ODA: Total annual official development assistance expenditure	2001-2019	2018-2019	Weighted average of DAH/budgeted ODA
**New Zealand**	Vote Foreign Affairs and Trade (1998-2001); VOTE Official Development Assistance (2002-2019)[56]	General ODA: Total annual official development assistance expenditure	1998-2019	2018-2019	Weighted average of DAH/budgeted ODA
**Norway**	Norwegian Ministry of Finance National Budget (2014-2019); Correspondence (2000-2013)[57,58]	General ODA: ODA budget	2000-2019	2018-2019	Weighted average of DAH/budgeted ODA
**Portugal**	Ministry of Finance and Public Administration State Budget 2003-2019[59]	General ODA: Integrated service expenditure – external cooperation budget	2003-2019	2018-2019	Weighted average of DAH/budgeted ODA
**Spain**	Annual Plans of Spanish International Cooperation[60]	General ODA: Spanish total development cooperation	2003-2019	2018-2019	Weighted average of DAH/budgeted ODA

**Sweden**	Correspondence (2000-2010); Ministry of Foreign Affairs Budget (2010-2019)[61]	General ODA: Ministry for Foreign Affairs budgets for expenditure – international development cooperation	2000-2019	2018-2019	Weighted average of DAH/budgeted ODA
**Switzerland**	Foreign Affairs (2000-2006); Budget – Further Explanations and Statistics (2007-2019)	General ODA: Direction of development and cooperation (2000-2006); foreign affairs – international cooperation, development aid (in the South and East) (2007-2016)	2000-2019	2018-2019	Weighted average of DAH/budgeted ODA
**United Kingdom**	IATA (Department for International Development (DFID)) [62,63]	General ODA: assistance for international development; Sum (revised) - aggregated project data	1998-2019	2018-2019	Weighted average of DAH/budgeted ODA
**United States**	Foreign Assistance Dashboard (2006-2019); Budget of the US Government (2005-2019)[64,65]	Global health ODA: Planned foreign assistance for health; Department of Health and Human Services global health budget	2005-2019	2018-2019	Weighted average of actual DAH/budgeted DAH
**UN agencies**					
**WHO**	Programme budget[66]	DAH budget: Programme budget	2002-2018	2019	Weighted average of DAH/budget
**UNAIDS**	Unified Budget and Workplan, bienniums 2002-2017 and 2018-2019[67,68]	DAH budget: Unified Budget and Workplan	2002-2018	2019	Weighted average of DAH/Core Budget
**UNICEF**	Financial report and audited financial statements[7,69,70]	Total expenditure;	1990-2018	2019	Weighted average of DAH/budget

		Total health expenditure			
**UNFPA**	Audited Financial report, contributions report, annual report, and Structured Funding dialogue [71,72]	Total health expenditure	2002-2018	2019	2018 DAH/budget
**PAHO**	Proposed program budget[10]	Total regular budget, estimated voluntary contributions	2000-2018	2019	Weighted average of DAH/budget
**NGOs**	VolAg (1990-2014), GuideStar (2015-2016), sample of top NGOs [25,26]	Revenue breakdowns for: US public, non-US public, private, in-kind, BMGF; total overseas expenditures	1990-2016	2017-2019	Regression on DAH, US GDP, and USAID and private voluntary organization (PVO) revenue

* Years of budget data used for modeling versus years underlying DAH data unavailable thus modeled: The data used to estimate DAH by channel vary across channels. Table S3.1 reports our primary data used for each channel. Due to reporting lags there are some years we need to estimate disbursement using additional data sources. These additional data sources, the years in which the primary data is modeled, the years the additional data is available, and the methods for this estimating these modeled years are reported in Table S3.3. Years of budget data used for modeling are the years of additional data available to us. We rely on historic trends to inform our estimates so we rely on many years of additional data despite only modeling a few years of primary data. Years underlying DAH data unavailable thus modeled are the years the primary data is incomplete and thus estimated using additional data. See example below for more details for Australia.

**Box 3.1. EXAMPLE - Australia's primary and additional data sources**
**Project-level data for health-related projects funded by Australia's bilateral aid agencies are available from the OECD's CRS database through 2017. This is the primary data source used to estimate DAH channeled by Australian aid agencies, as described in Table S3.1. 2018 and 2019 are incomplete because of lags in reporting. To estimate DAH disbursed for 2018-2019, additional data are available from Australia's International Development Assistance budget (2008-2019) and Australia's Overseas Aid Program budget (1998-2008), as described in Table S3.3. These sources provide health-specific official development assistance (ODA) budgeted by Australia, 1998-2019. We convert countries' budgeted ODA, as given in nominal local currency units, to nominal US dollars using the OECD's currency exchange rate series based on USD monthly averages. To estimate DAH disbursed in 2018-2019, we calculated the ratio of disbursed DAH (from the CRS database) relative to budgeted DAH (from the International Development Assistance and Overseas Aid Program budgets) for 1998-2017. We combine the most recent three ratios into a single estimate by taking a weighted average, weighting substantially higher the most recent year. We multiply this ratio – the estimated disbursed DAH to budgeted DAH – by the 2018 or 2019 budgeted DAH to estimate disbursed DAH in those years. These methods are described more fully in Dieleman et al.[1]**

S3.1.2 DISAGGREGATING BY HEALTH FOCUS AREA

Similar to our previous work, the analysis of health focus areas included assessments of development assistance for HIV/AIDS, tuberculosis (TB), malaria, reproductive and maternal health, newborn and child health, other infectious diseases, non-communicable diseases, and SWAps and health system strengthening by using keyword searches within descriptive fields. These were chosen as the areas of focus because of their relevance to current policy debates about global health financing and data availability.

In effect, DAH was disaggregated into nine health focus areas: HIV/AIDS, tuberculosis, malaria, reproductive and maternal health, newborn and child health, non-communicable diseases, SWAps/health sector support, other infectious diseases, and other. For most data sources, project-level data were available only through 2018. Methods to estimate health focus area allocations for 2019 are described in more detail below. Keyword searches were performed for a subset of global health channels that provide project-level data with project titles or descriptions. These sources include the bilateral development assistance agencies from 24 DAC member countries, one DAC participant country, GFATM, the World Bank, Asian Development Bank, African Development Bank, Inter-American Development Bank, Bill & Melinda Gates Foundations, Non-government Organizations, US foundations, Wellcome Trust and the European Economic Area. The keywords used are outlined in Table S3.4 below. Descriptive fields were adjusted so that they were all in capitalized letters, special characters were modified, and search terms with multiple words were put between quotation marks. All keywords were translated into nine major languages (English, Spanish, French, Portuguese, Italian, Dutch, German, Norwegian, and Swedish) used in the OECD CRS, checked for double meanings across all languages, and adjusted accordingly.

Total DAH was split across the health focus areas using weighted averages based on the number of keywords present in each project's descriptive variables. If, for example, three keywords suggested the project focused on HIV/AIDS and two keywords related to tuberculosis were also tagged, three-fifths of the project's total DAH was allocated to HIV/AIDS and two-fifths was allocated to tuberculosis. To account for the sensitivity of this method, several checks were implemented after the keyword searches to ensure the project was accurately categorized. First, projects that were tagged as child and newborn vaccines and other infectious diseases were categorized as child and newborn vaccines only. Second, projects that were tagged as one of the three major infectious diseases (HIV/AIDS, tuberculosis, or malaria) and other infectious diseases were categorized under only HIV/AIDS, tuberculosis, or malaria.

Box 3.2. EXAMPLE. Post-keyword search weighting
**A project in the CRS database had a value of $1,000 of DAH. A keyword search conducted on this project's title and description tagged five keywords: 3 keywords related to HIV/AIDs and 2 keywords related to tuberculosis. Therefore, $600, or 3/5 of total DAH, was allocated to HIV/AIDS, while $400, or 2/5 of total DAH, was allocated to tuberculosis.**

In addition to keyword searches, funds were allocated to health focus areas based on characteristics of the channel or additional channel variables. For the bilateral agencies and the EC, purpose codes from the CRS were used to supplement keyword searches. For the World Bank-IDA and -IBRD, health focus areas were also determined by the project sector codes and theme codes, which included percentages of health funds that targeted each theme. All funds from Gavi were allocated to child and newborn vaccines, health system strengthening and non-communicable diseases and all funds from UNICEF to HIV/AIDS prevention, newborn and child health, ebola, and reproductive and maternal health – other (plus a very small amount being unallocable). Funds from GFATM were distributed to malaria, HIV/AIDS, TB, and health sector support based on disease components. Within each disease component, keyword searches on programmatic budget data and project descriptions were conducted to distribute among program areas. Funds from UNAIDS were allocated to HIV/AIDS and TB with specific program areas determined by budget information. UNITAID funds were allocated based on assignments made by correspondence. UNFPA funds were allocated to specific health focus areas based on project expenditure data from its financial review report. PAHO and WHO funds were allocated to specific health focus areas based on project expenditure data from

correspondence budget data and their proposed programme budgets. For all channels, projects listed as HIV/TB were distributed evenly among the two health focus categories. See Table S3.5 below for more details on these categorizations.

Table S3.4. Terms for keyword searches

**Health focus area level**	**Program area**	**Keywords**
**HIV/AIDS**	HIV envelope/other	" HUMANIMMUNODEFVIRUS " " SIDA " " OVC " " H I V " " HIV " " AIDS " " HUMAN IMMUNODEFICIENCY " " REVERSE TRANSCRIPTASE INHIBITOR " " ACQUIRED IMMUNE DEFICIENCY SYNDROME " " ACQUIRED IMMUNNODEFICIENCY " "RETROVIRAL " " VCT " " MALE CIRCUMCISION" " ART " " ARV " " CD4 COUNT " " HAART " " PMTCT " " MOTHER TO CHILD TRANSMISSION" " MOTHER TO CHILD AIDS TRANSMISSION" " PARENT TO CHILD TRANSMISSION" " PRESIDENT S EMERGENCY PLAN FOR AIDS RELIEF " " PEPFAR " " THREE DISEASES FUND " " 3 DISEASES FUND " " EMTCT " " 90 90 90 " " MOTHERTOCHILD"
	Care and Support	" CAREANDSUPPORT " " CARE ACTIVIT" " PAIN RELIEF " " SYMPTOM RELIEF " " PSYCHO SOCIAL SUPPORT " " CHRONICALLY ILL " " CLINICAL MONITORING " " CARE AND SUPPORT " " PSYCHOLOGICAL SERVICE" " PSYCHOLOGICAL SUPPORT " " PSYCHOSOCIAL SUPPORT " " PSYCHOSOCIAL SERVICE" " MATERIAL SUPPORT " " HOME BASED CARE " " PALLIATIVE CARE "
	Counseling and Testing	" COUNSELING " " TESTING " " VCT " " COUNSELLING " " COUNSELINGANDTESTING " " DIAGNOS"
	Orphans and Vulnerable Children	" VULNERABLECHILD" " OVC " " ORPHAN" " VULNERABLE CHILD" " INFECTED CHILD"
	Prevention of mother-to-child transmission (PMTCT)	" MOTHERTOCHILD" " MOTHER TO CHILD" " PARENT TO CHILD" " PMTCT " " EMTCT " " OPTION B "
	Prevention	" CONDOM" " PREVENT" " HIV EDUCATION " " AIDS EDUCATION " " REDUCING THE TRANSMISSION OF HIV " " REDUCE THE TRANSMISSION OF HIV " " MALE CIRCUMCISION" " SAFE BLOOD SUPPL" " SAFE INJECTION" " ABSTINENCE " " BLOOD SAFETY " " MICROBICIDE" " HARM REDUCTION " " PREP " " PEP " " PROPHYLAXIS " " ABCD " " BE FAITHFUL " " EARLY DETECTION "
	Treatment	"RETROVIRAL " " TREAT" " ART " " ARV " " CD4 COUNT " " HAART " " VIRAL LOAD " " VIRAL BURDEN " " VIRAL TITER " " DRUG REGIMEN" " FIRST LINE " " REVERSE TRANSCRIPTASE INHIBITOR "
	Antimicrobial resistance	" ANTIMICROBIAL RESISTAN" " ANTI MICROBIAL RESISTAN" " ANTIBIOTIC RESISTAN" " AMR " "DRUG RESISTAN" " MDR " " XDR " " RESISTANCE TESTING " " DRUG SUSCEPTIBILITY TESTING " " DST " " SECOND LINE "
**Tuberculosis**	Tuberculosis envelope/other	" TUBERCULOSIS " " TB " " TBC " " TUBERCULAR" " DOTS " " DIRECTLY OBSERVED TREATMENT " " RIFAMPICIN " " ISONIAZID " " THREE DISEASES FUND " " 3 DISEASES FUND " " RIFAMPIN "
	Treatment	" TREAT" " DOTS " " FIRST LINE " " DRUGS " " RIFAMPICIN " " RIFAMPIN " " ISONIAZID " " INH " " PYRAZINAMIDE " " PZA "

Health focus area level	Program area	Keywords
		" ETHAMBUTOL " " EMB " " STREPTOMYCIN " " SM " " STM " " PATIENT KIT " " INJECTABLE AGENT" " FLUOROQUINOLONES " " REGIMEN" " CASE MANAGEMENT " " ANTIMICROBIAL THERAPY " " DRUG SUSCEPTIBLE " " DRUG SENSITIVE "
	Diagnosis	" MICROSCOPY " " SPUTUM " " SMEAR " " CULTURE " " BACTERIOLOGICALLY CONFIRMED " " LABORATORY CONFIRMED " " GENEXPERT " " XPERT MTB RIF " " CHEST X RAY" " DIAGNOS" " LINE PROBE ASSAY "
	Antimicrobial resistance	" ANTIMICROBIAL RESISTAN" " ANTI MICROBIAL RESISTAN" " ANTIBIOTIC RESISTAN" " AMR " "DRUG RESISTAN" " MDR " " XDR " " RESISTANCE TESTING " " DRUG SUSCEPTIBILITY TESTING " " DST " " SECOND LINE "
**Malaria**	Malaria envelope/other	" MALARIA " " FALCIPARUM " " ANOPHELES " " ARTEMISININ " " PRIMAQUINE " " INDOOR RESIDUAL SPRAY" " INDOORRESIDUALSPRAY" " IRS " "VIVAX " " BEDNET" " BED NET" " SMITN " " ITN " " LLIN " " INSECTICIDAL NET" " INSECTICIDE TREATED NET" " THREE DISEASES FUND " " 3 DISEASES FUND " " CHLOROQUINE " " ANTI MALARIAL " " ANTIMALARIAL "
	Diagnosis	" DIAGNOS" " CASE DETECTION " " MICROSCOPY " " BLOOD SURVEY" " BIOLOGICAL TESTING " " EDT " " LAMP " " RDT "
	Community outreach	" COMMUNITYOUTREACH " " OUTREACH " " COMMUNITY MOBILIZATION" " AWARE" " COMMUNICATION STRATEGY " " SOCIAL COMMUNICATION " " PARTNERSHIP" " ACTIVITIES NEAR COMMUNITIES " " BCC " " BEHAVIORAL CHANGE COMMUNICATION " " BEHAVIOURAL CHANGE COMMUNICATION " " BEHAVIOR CHANGE COMMUNICATION " " BEHAVIOUR CHANGE COMMUNICATION " " SOCIAL MOBILIZATION "
	Vector control: bednets	" BEDNET" " BED NET" " SMITN " " ITN " " LLIN " " INSECTICIDAL NET" " INSECTICIDE TREAT"
	Vector control: irs	" INDOORRESIDUALSPRAY" " IRS " " REDUCE THE PARASITE RESERVOIR " " FOGGING " " COILS " " LARVICID" " LARVACID" " VECTOR CONTROL" "RESIDUAL SPRAY" " RESIDUALSSPRAY " "INDOOR SPRAY" " INDOORSPRAY "
	Vector control: other than bednets and irs	" PREVENT" " IPT " " SMC " " SEASONAL MALARIA CHEMOPREVENTION "
	Treatment	" ARTEMISININ " " PRIMAQUINE " " ACT " " DRUG" " TREAT" " CASE MANAGEMENT " " COMBINATION THERAPY " " ANTI MALARIAL " " ANTIMALARIAL " " CHLOROQUINE "
	Antimicrobial resistance	" ANTIMICROBIAL RESISTAN" " ANTI MICROBIAL RESISTAN" " ANTIBIOTIC RESISTAN" " AMR " "DRUG RESISTAN" " MDR " " XDR " " RESISTANCE TESTING " " DRUG SUSCEPTIBILITY TESTING " " DST " " SECOND LINE "
**Reproductive and maternal health**	envelope/other	" FERTILITY " " FAMILY PLANNING " " FP " " BIRTH" " WOMEN HEALTH " " WOMEN S HEALTH " " WOMENS HEALTH " " CONTRACEP" " IPPF " " PLANNED PARENTHOOD " " ABORTION" " UNFPA " " POSTPARTUM " " POST PARTUM " " MATERNAL " " MATERNITY " " MOTHER" " SBA " " ANTENATAL " " PRENATAL " " PERINATAL " " POSTNATAL " " FETUS" " FETAL" " IPTP " " REPRODUCTIVE HEALTH " " OBSTETRIC" " PREGNANC" " RH " " REPROD " " RHCS " "

Health focus area level	Program area	Keywords
		SEXUAL HEALTH " " SYPHILIS " " FISTULA " " ANEMI" " ANAEMI" " FOETUS" " FOETAL " " FGM " " FEMALE GENITAL MUTILATION " " FEMALE GENITAL CUTTING " " FEMALE CIRCUMCISION " " SBAS " " OBSTRUCTED LABOR " " OBSTRUCTED LABOUR " " MNCH" " RNCH " " RCH " " RNH " " MNH " " MCH " " EMAS " " MCNH " " PMNCH " "ECLAMPSIA " " PRETERM " " ANC " " WCAH " " TBA " " TBAS " " BIRTH ATTENDANT" " CESAREAN" " CAESAREAN" " C SECTION" " STI " " STD " " SEXUALLY TRANSMITTED "
	Family planning	" FERTILITY " " FAMILY PLANNING " " FP " " BIRTH SPACING " " CONTRACEPT" " FAMILY SIZE" " IPPF " " PLANNED PARENTHOOD " " ABORTION" " BIRTH CONTROL " " CONDOM" " IUD " " VASECTOMY " " TUBULAR LIGATION"
	Maternal health	" POSTPARTUM " " POST PARTUM " " MATERNAL HEALTH " " MATERNAL MORTALITY " " MATERNAL DEATH" " SAFE MOTHERHOOD " " BIRTH ATTENDANT" " SBA " " ANTENATAL " " PRENATAL " " PERINATAL " " POSTNATAL " " FETUS" " FETAL" " IPTP " " MATERNITY " " OBSTETRIC" " PREGNANC" " FISTULA " " SEPSIS " " SEPTICEMIA " " ANEMI" " ANAEMI" " FOETUS" " FOETAL " " SBAS " " OBSTRUCTED LABOR " " OBSTRUCTURED LABOUR " " DELIVERY ROOM" " CHILD DELIVERY " " MIDWIV" " MIDWIFE" "ECLAMPSIA " " PRETERM " " ANC " " TBA " " TBAS " " CESAREAN" " CAESAREAN" " C SECTION" " MNH " " MCH " " MNCH " " MCNH "
**Newborn and child health**	envelope/other	" NEONATAL " " PERINATAL " " POSTNATAL " " MALNUTRITION " " VITAMIN A " " BREAST FE" " BREASTFE" " MICRONUTRIENT" " FORTIFICATION " " STUNT" " WASTING " " BABY FRIENDLY HOSPITAL INITIATIVE " " BREASTMILK " " BREAST MILK " " IODINE " " IODIZED " " IODIZATION " " VAD " " LACTAT" " FOLIC ACID " " FOLAT" " VACCIN" " IMMUNIZ" " POLIO " " DIPHTHERIA " " TETANUS " " PERTUSSIS " " DTP " " HIB " " ROTAVIRUS " " MEASLES " " IMMUNIS" " HEPB " " INJECTION SAFETY " " RUBELLA " " MENINGITIS " " PENTA " " PENTAVALENT " " PNEUMONIA " " PNEUMOCOCC" " HAEMOPHILUS INFLUENZAE " " TETRA " " GAVI " " CHILDHEALTH " " CHILD HEALTH " " CHILDREN " " INFANT" " NEWBORN" " CHILD MORTALITY " " UNDER FIVE MORTALITY " " CHILD SURVIVAL " " CHILDHOOD ILLNESS" " LRI " " RESPIRATORY INFECTION" " DIARRHEA" " DIARRHOEA" " ORAL REHYDRATION " " ORT " " ORS " " UNICEF " " MNCH" " RNCH " " RCH " " RNH " " MNH " " MCH " " EMAS " " MCNH " " POLIOVIRUS "
	Child/newborn nutrition	" NUTRITION" " MALNUTRITION " " BIRTH WEIGHT " " BIRTHWEIGHT " " VITAMIN A " " BREAST FE" " BREASTFE" " FEEDING " " MICRONUTRIENT" " ZINC " " FORTIFICATION " " STUNT" " WASTING " " UNDERWEIGHT " " BABY FRIENDLY HOSPITAL INITIATIVE " " BREASTMILK " " BREAST MILK " " IODINE " " IODIZED " " IODIZATION " " VAD " " LACTAT" " FOLIC ACID " " FOLAT" " IRON " " DEWORMING "
	Child/newborn vaccines	" POLIO " " VACCIN" " IMMUNIZ" " DIPHTHERIA " " TETANUS " " PERTUSSIS " " DTP " " HIB " " ROTAVIRUS " " MEASLES " " IMMUNIS" " HEPB " " INJECTION SAFETY " " RUBELLA " " MENINGITIS " " PENTA " " PENTAVALENT " "

Health focus area level	Program area	Keywords
		PNEUMONIA " " PNEUMOCOCC" " HAEMOPHILUS INFLUENZAE " " TETRA " " GAVI " " POLIOVIRUS " " DPT " " PCV "
**Non-communicable diseases**	envelope/other	" TOBACCO" " SMOK" "CIGAR" " FCTC " " TFI " " SCHIZOPHRENIA " " MENTAL HEALTH " " NEUROTIC " " NEUROSIS " " NEUROSES " " NEUROLOGICAL" " PSYCHOLOG" " PSYCHIATR" " EMOTIONAL DISORDER" " OBSESSIVE COMPULSIVE " " OCD " " PTSD " " POST TRAUMATIC " " POSTTRAUMATIC " " ALCOHOL DEPENDEN" " ALCOHOL ABUSE " " ADDICTION " " BEHAVIORAL DISORDER" " DRUG ABUSE " " SUBSTANCE ABUSE " " OPIOID " " COCAINE " " AMPHETAMIN" " DEPRESSIVE DISORDER" " DEPRESSION " " DYSTHYMIA " " BIPOLAR " " ANXIETY " " EATING DISORDER " " PHOBIA " " DRUG DEPENDEN" " ATTENTION DEFICIT HYPERACTIVITY DISORDER " " ADHD " " PANIC DISORDER" " SELF HARM " " STRESS DISORDER" " SUBSTANCE USE DISORDER" " DRUG USE DISORDER" " MENTAL ILLNESS" " MENTAL DISORDER" " PSYCHOSOCIAL " " PSYCHO SOCIAL " " HEROIN " " OXYCODONE " " ATTENTION DEFICIT DISORDER " " SUICIDE PREVENTION" " HEADACHE " " ANOREXIA " " BULIMIA " " HYPERKINETIC DISORDER" " PERSONALITY DISORDER" " FETAL ALCOHOL SYNDROME" " PSYCHOSIS " " DOWN SYNDROME " " DOWN S SYNDROME " " DOWNS SYNDROME " " AUTISM " " ASPERGER " " DEVELOPMENTAL DISORDER" " CONDUCT DISORDER" " INTELLECTUAL DISABILIT" " MENTAL DISAB" " MENTAL RETARDATION " " ALZHEIMER" " DEMENTIA" " EPILEPSY " " MIGRAINE " " PARKINSON" " MENTALLY DISAB" " NERVOUS SYSTEM" " SYNAPSE" " NON COMMUNICABLE " " NONCOMMUNICABLE " " CANCER" " CHEMOTHERAPY " " RADIATION " " NEOPLAS" " TUMOR " " LEUKEMIA " " LYMPHOMA " " MYELOMA " " HPV " " HUMAN PAPILLOMA VIRUS " " HEP C " " HEPATITIS C " " DIABET" " INSULIN " " ENDOCRINE " " RHEUMAT" " ISCHAEMIC " " ISCHEMIC " " CIRCULATORY " " CIRRHOSIS " " DIGESTIVE DISEASE" " OTHER DIGESTIVE " " PEPTIC " " APPENDICITIS " " GASTRITIS " " GENITOURINARY " " UROGENITAL " " MUSCULOSKELETAL " " GOUT " " BACK PAIN " " MACULAR " " HEARING " " AUDIOLOG" " PERIODONTAL " " CARIES " " CONGENITAL " " OBESITY " " OVERWEIGHT " " GLAUCOMA " " HYPERTENSI" " HERNIA " "ARTHRITIS " " CLEFT LIP" " CLEFT PALATE" " PHENYLKETONURIA " " SICKLE CELL" " DREPANOCYTOSIS " " HEMOPHILIA " " HAEMOPHILIA " " THALASSEMIA " " GENETIC DISORDER" " HEART DISEASE" " CHRONIC RESPIRATORY " " COPD " " STROKE " " CATARACT" " CHRONIC OBSTRUCTIVE PULMONARY DISEASE" " ASTHMA " " SKIN DISEASE" " DERMATITIS " " PSORIASIS " " SCABIES " " PHYSICAL DISAB" " DENTAL " " ORAL HEALTH " " CVD " " IHD " " CKD " " KIDNEY DISEASE" " MSK " " EYE " " CEREBROVASCULAR " " VASCULAR " " BLOOD PRESSURE " " ACUTE GLOMERULONEPHRITIS " " ALOPECIA AREATA " " ANEURYSM " " ANGINA " " ARTERY "

Health focus area level	Program area	Keywords
		" ATHEROSCLEROSIS " " ATRIAL FIBRILLATION" " ATRIAL FLUTTER " " BENIGN PROSTATIC HYPERPLASIA " "BLASTOMA" " BLIND " " PREVENTABLE BLINDNESS " " AVOIDABLE BLINDNESS " " BLOOD DISORDER" " BRONCHITI" " CARCINOMA " " CARDIAC " " CARDIO" " CELLULITIS " " CEREBRAL " " CORONARY " " DEAF" " DECUBITUS ULCER " "DIALYSIS" " DUODENITIS " " ECZEMA " " EKZEMA " " EDENTULISM " " ENDOCARDITIS " " FIBROSIS " " G6PD DEFICIENCY " " GALL BLADDER " " BILE DUCT " " GLYCEMI" " GLYCAEMI" " HEMOGLOBINOPATH" " HEMOLYTIC ANEMIA " " HODGKIN" " INSOMNIA " " INTERSTITIAL LUNG DISEASE" " INTESTINAL OBSTRUCTION" " LEUKAEMIA " " MELANOMA" " MULTIPLE SCLEROSIS " " MYOCARD" " NCD " " NECK PAIN " " NEPHRITIS " " NEPHROSIS " " NEURAL TUBE DEFECT" " NEURODEGENERATIVE " " INFLAMMATORY BOWEL" " ONCOLOG" " OPTICAL " " OSTEOMYELITIS " " OTITIS MEDIA " " PANCREATITIS " " PARALYTIC ILEUS " " PERITONEAL " " PNEUMOCONIOSIS " " PROSTATE " " PRURITUS " " SARCOIDOSIS " " PYELONEPHRITIS " " REFRACTIVE ERROR" " RENAL " " RETINA " " SARCOMA " " SUBCUTANEOUS DISEASE" " URINARY DISEASE" " URINARY TRACT INFECTION" " UROLITHIASIS " " URTICARIA " " VENTRICULAR " " VISION LOSS " " ACCOMODATION DISORDER" " SENSE ORGAN " " GUILLAIN BARRE SYNDROME" " IMPETIGO " " LOSE WEIGHT " " BIRTH DEFECT" "PAPILLOMAVIRUS" " GENE DEFECT" " PHYSICALLY DISAB" " TUMOUR" " BRAIN INJUR" " MAMMOGRA" " ANTITUMOR " " ANTITUMOUR " " BARIATRIC" " FATTY LIVER" " IMMUNOTHERAPY " " CHROMOSOMAL ABERRATION" " PERIODONTITIS " " OSTEOPOROSIS " "MALIGNANC" " NEURON"
	Mental health	" SCHIZOPHRENIA " " MENTAL HEALTH " " NEUROTIC " " NEUROSIS " " NEUROSES " " NEUROLOGICAL" " PSYCHOLOG" " PSYCHIATR" " EMOTIONAL DISORDER" " OBSESSIVE COMPULSIVE " " OCD " " PTSD " " POST TRAUMATIC " " POSTTRAUMATIC " " ALCOHOL DEPENDEN" " ALCOHOL ABUSE " " ADDICTION " " BEHAVIORAL DISORDER" " DRUG ABUSE " " SUBSTANCE ABUSE " " OPIOID " " COCAINE " " AMPHETAMIN" " DEPRESSIVE DISORDER" " DEPRESSION " " DYSTHYMIA " " BIPOLAR " " ANXIETY " " EATING DISORDER " " PHOBIA " " DRUG DEPENDEN" " ATTENTION DEFICIT HYPERACTIVITY DISORDER " " ADHD " " PANIC DISORDER" " SELF HARM " " STRESS DISORDER" " SUBSTANCE USE DISORDER" " DRUG USE DISORDER" " MENTAL ILLNESS" " MENTAL DISORDER" " PSYCHOSOCIAL " " PSYCHO SOCIAL " " HEROIN " " OXYCODONE " " ATTENTION DEFICIT DISORDER " " SUICIDE PREVENTION" " HEADACHE " " ANOREXIA " " BULIMIA " " HYPERKINETIC DISORDER" " PERSONALITY DISORDER" " FETAL ALCOHOL SYNDROME" " PSYCHOSIS " " DOWN SYNDROME " " DOWN S SYNDROME " " DOWNS SYNDROME " " AUTISM " " ASPERGER " " DEVELOPMENTAL DISORDER" " CONDUCT DISORDER" " INTELLECTUAL

Health focus area level	Program area	Keywords
		DISABILIT" " MENTAL DISAB" " MENTAL RETARDATION " " ALZHEIMER" " DEMENTIA" " EPILEPSY " " MIGRAINE " " PARKINSON" " MENTALLY DISAB" " NERVOUS SYSTEM" " SYNAPSE"
	Tobacco	" TOBACCO" " SMOK" "CIGAR" " FCTC " " TFI "
**SWAps/ Health sector support**	envelope/other	" SWAP" " TRAINING " " CAPACIT" " DATA SYSTEM" " SECTOR WIDE APPROACH" " HEALTH SYSTEM" " SECTOR PROGRAM" " BUDGET SUPPORT" " SECTOR SUPPORT " " HSS " " TRACKING PROGRESS " " SKILLED WORKER" " HEALTH WORKER" " SKILLED STAFF " " HEALTH PROFESSIONAL" " FACILITIES " " ESSENTIAL MEDICINES " " POLICY DEVELOPMENT" " MEDICAL EQUIPMENT" " SURGICAL EQUIPMENT" " HOSPITAL EQUIPMENT" " HOSPITAL EQMT " " INSTITUTIONAL STRENGTHENING " " HSPSP " " M&E " " M & E " " MONITORING " " SURVEILLANCE " " GOVERNANCE " " HUMAN RESOURCE" " HUMAN CAPITAL " " SCALING UP " " REALLOCATE RESOURCES " " STRATEGIES AND PROGRAM" " HIV STRATEG" " PROGRAM IN COUNTRY ACTIVITIES " " STRATEGIC INFORMATION " " PROCUREMENT " " EVIDENCE BASED " " CASE REPORTING " " MEDICAL WORKER" " HEALTH CARE PERSONNEL " " OPERATIONAL RESEARCH " " SUPPORTIVE ENVIRONMENT " " INFORMATION SYSTEM" " WORKFORCE " " INFRASTRUCTUR" " MEDICAL EDUCATION " " CASE NOTIFICATION " " CASE FINDING " " LABORATORY STRENGTHENING " " LABORATORY QUALITY " " LABORATORY NETWORK" " CONTROL SERVICES " " INFECTION CONTROL " " CONTROL PROGRAM" " SCALE UP" " STOP TB STRATEGY " " HEALTH EDUCATION " " CONTINUING EDUCATION " " SUPPLY " " HEALTH MANAGEMENT" " HEALTH POLICY " " MANAGEMENT AND COORDINATION " " ADMINISTRATIVE MANAGEMENT " " MANAGEMENT AND ADMINISTRATION " " COLD CHAIN" " HEALTH PROMOTION " " TECHNICAL ASSISTANCE " " DSS " " DISTRIBUTION SYSTEMS " " SERVICE DELIVERY " " BUILDINGS " " HEALTH FACILIT" " CONSTRUCT" " MEDICAL SCHOOL" "CENTERS OF EXCELLENCE" "NURSE" "DOCTOR" "PHYSICIAN" "MEDICAL LABORATORY SCIENTIST" "SURGEON" "SPECIALIST" "PHARMACIST" "HEALTH LABOR" "LABOR MARKET" "PERSONNEL" "MEDICAL PRACTIONER" "DENTAL PRACTIONER" "TASK SHIFTING"

Health focus area level	Program area	Keywords
	Human resources	" INFRASTRUCTUR" " MEDICAL EQUIPMENT" " SURGICAL EQUIPMENT" " HOSPITAL EQUIPMENT" " HOSPITAL EQMT " " BUILDINGS " " HEALTH FACILIT" " CONSTRUCT" " MEDICAL SCHOOL" "CENTERS OF EXCELLENCE" " TRAINING " " CAPACIT" " SKILLED WORKER" " HEALTH WORKER" " SKILLED STAFF " " HEALTH PROFESSIONAL" " HUMAN RESOURCE" " HUMAN CAPITAL " " MEDICAL WORKER" " WORKFORCE " " MEDICAL EDUCATION " " HEALTH EDUCATION " " CONTINUING EDUCATION " " HEALTH MANAGEMENT" " MANAGEMENT AND COORDINATION " " ADMINISTRATIVE MANAGEMENT " " MANAGEMENT AND ADMINISTRATION " "NURSE" "DOCTOR" "PHYSICIAN" "MIDWIFE" "MIDWIVES" "MEDICAL LABORATORY SCIENTIST" "SURGEON" "SPECIALIST" "PHARMACIST" "HEALTH LABOR" "LABOR MARKET" "PERSONNEL" "MEDICAL PRACTIONER" "DENTAL PRACTIONER" "TASK SHIFTING" " TECHNICAL ASSISTANCE "
	Pandemic preparedness	" PANDEMIC PREPAREDNESS " " PANDEMIC RESPONSE" " PANDEMIC ALERT" " EPIDEMIC ALERT" " EPIDEMIC RESPONSE" " EPIDEMIC PREPAREDNESS " " OUTBREAK RESPONSE" " OUTBREAK ALERT" " OUTBREAK PREPAREDNESS " " PANDEMIC INFLUENZA " " EPIDEMIOLOGICAL INVESTIGATION" " CONTACT MANAGEMENT " " PREPAREDNESS AND RESPONSE PLAN" " PREPAREDNESS & RESPONSE PLAN" " BIOSAFETY MEASURE" "EARLY WARNING " " HEALTH SECURITY PREPAREDNESS " " HEALTH SECURITY RISK ASSESSMENT " " RAPID RESPONSE STRATEG"
**Other infectious diseases**	envelope/other	" INFECTIOUS " " COMMUNICABLE " " TRICHURIASIS " " YELLOW FEVER " " WHIPWORM " " TRACHOMA " " SCHISTOSOMIASIS " " BILHARZIA " " SNAIL FEVER " " KAYAYAMA FEVER " " RABIES " " ONCHOCERCIASIS " " RIVER BLINDNESS " " ROBLES DISEASE" " LYMPHATIC FILARIASIS " " ELEPHANTIASIS " " LEISHMANIASIS " " LEISHMANIOSIS " " HOOKWORM " " FOOD BORNE " " FOODBORNE " " ECHINOCOCCOSIS " " HYDATID DISEASE" " HYDATIDOSIS " " DENGUE " " CYSTICERCOSIS " " CHAGAS " " TRYPANOSOMIASIS " " SLEEPING SICKNESS " " ASCARIASIS " " TROPICAL DISEASE" " CHOLERA " " DYSENTERY " " PARASITE DISEASE" " FAO " " NEGLECTED TROPICAL DISEASE" "TYPHOID" " LEPROSY " " BURULI ULCER " " EBOLA" " EBOV " " EVD " " ZIKA " " ZIKV " " GUINEA WORM " " DRACUNCULIASIS " " FILARIASIS " " HEPATITIS E" " ENCEPHALITIS " " VARICELLA" " INFLUENZA" " FLU " " NTD " " HEPATITIS A " " HEPATITIS D " " HEP A " " HEP D " " ROUNDWORM" " RINGWORM" " TAPEWORM" " FLATWORM" " CHIKUNGUNYA " " LASSA " " MERS " " NIPAH " " MARBURG " " SARS "

Health focus area level	Program area	Keywords
	Antimicrobial resistance	" ANTIMICROBIAL RESISTAN" " ANTI MICROBIAL RESISTAN" " ANTIBIOTIC RESISTAN" " AMR " "DRUG RESISTAN" " MDR " " XDR " " RESISTANCE TESTING " " DRUG SUSCEPTIBILITY TESTING " " DST " " SECOND LINE "

Table S3.5. Additional health focus area categorizations

Channel	Allocation criteria	Health focus area
**Bilaterals and the EC**	CRS purpose code 13030, family planning	Family planning
	CRS purpose code 13020, reproductive health care	Maternal health, non-family planning
	CRS purpose code 12240, basic nutrition	Child and newborn nutrition
	CRS purpose code 12250, infectious disease control and the keywords “child” or “vaccine” present in descriptive variables	Child and newborn vaccines
	CRS purpose code 13040, STD control including HIV/AIDS	HIV/AIDS
	CRS purpose code 12262, malaria control	Malaria, unspecified
	CRS purpose code 12250, infectious disease control and no other keywords present in the descriptive variables	Other infectious diseases
	CRS purpose code 12263, tuberculosis control	Tuberculosis
	CRS purpose code 12230, basic health infrastructure	SWAPs/health system strengthening - other
	CRS purpose code 12281, health personnel development	SWAPs/health system strengthening - Human resources
	CRS purpose code 12281, medical education/training	SWAPs/health system strengthening - Human resources
**World Bank IDA and IBRD**	Theme code population and reproductive health	Reproductive and maternal health, other
	Theme code tuberculosis	Tuberculosis, other
	Theme code child health	Newborn and child health, other
	Theme code HIV/AIDS	HIV/AIDS, other
	Theme code malaria	Malaria, other
	Theme code injuries and non-communicable diseases	Non-communicable diseases, other
	Theme code nutrition and food security	Newborn and child health, nutrition
	Theme code other communicable diseases	Other infectious diseases, other
	Theme code health system performance	SWAPs/health systems strengthening, other
	Theme code social analysis and monitoring	SWAPs/health systems strengthening, other
**UNFPA**	Family planning, population and development strategies, population and development, population dynamics	Family planning
	Reproductive health, maternal and newborn health, young people’s SRH and sexuality education, HIV and STI prevention services,	Maternal health

Channel	Allocation criteria	Health focus area
	sexual and reproductive health, sexuality education	
	Gender equality and women's empowerment, gender equality and reproductive rights, program coordination and assistance, adolescents and youth, civil society and rights for all, ending harmful practices, marginalized girls, protection rights, other	Family planning and Maternal health, unspecified, according to proportions between the two.
	HIV and STI prevention services, HIV and AIDS	HIV prevention
	Sexual and reproductive health policies, adolescents and youth skills and capabilities, adolescents and youth policies, youth leadership and participation, and elimination of harmful practices	Reproductive and maternal health - other
	Integrated sexual and reproductive health services	Split equally between maternal health, family planning, other reproductive and maternal health, and other HIV/AIDS
	Health workforce capacity	Reproductive and maternal health HSS - human resources
	Delivery of sexual and reproductive health commodities, accountability for sexual and reproductive health, gender equality laws and policies, gender and sociocultural norms, prevention and addressing of gender-based violence, population data systems, demographic intelligence, improved programming for results, optimized management of resources, United Nationals coordination and coherence, and communication and resources mobilization and partnerships	Reproductive and maternal health HSS - other
**UNICEF**	HIV/AIDS results	HIV/AIDS – prevention, treatment, care, PMTCT, OVC, and other
	Nutrition results	Newborn and child health – nutrition
	Health results	Reproductive and maternal health – other<br>Newborn and child health – HSS, vaccines, other
	Ebola (starting in 2014)	Other infectious diseases - Ebola
	The keyword search was run on budget information for years 2008-2018<br>Program components in budget documents from 1998 to 2007	All program areas under HIV/AIDS and TB
**UNAIDS**	Assigned in database received through correspondence	HIV/AIDS (treatment, counseling and testing, prevention) , TB (treatment and diagnosis), Malaria (diagnosis and treatment), HSS
**UNITAID**	Vaccine DAH for HPV vaccine<br>All other vaccine DAH<br>HSS DAH	NCD other<br>Child and newborn vaccines<br>Maternal and child health HSS
**GAVI**	Disease components for Malaria, HIV/AIDS, TB, TB/HIV, and Other (health systems strengthening)	All program areas under Malaria, TB, HIV and Swap/HSS

Channel	Allocation criteria	Health focus area
	Keyword search on program service delivery areas	
**GLOBAL FUND**	Reproductive, maternal, newborn, child, and adolescent health (divided by 2); Research in human reproduction	Maternal health, unspecified
**WHO**	Nutrition	Child and newborn nutrition
	Vaccine-preventable diseases	Child and newborn vaccines
	Reproductive, maternal, newborn, child and adolescent health (divided by 2)	Child and newborn health, unspecified
	Aging and health;	Maternal, newborn, and child health, unspecified
	HIV/AIDS	HIV/AIDS
	Malaria	Malaria
	Tuberculosis	Tuberculosis
	Mental health and substance abuse	Non-communicable diseases, mental health
	Disabilities and rehabilitation; Non-communicable diseases; Violence and injuries	Non-communicable diseases, unspecified
	Neglected tropical diseases; Tropical disease research; Outbreak and crisis response (50%); Alert and response capacities (50%); Polio eradication	Other infectious diseases
	Health system information and evidence; Integrated people-centered health services; National health policies, strategies and plans; Access to medicines and health technologies and strengthening regulatory capacity; health emergency information and risk assessment (50%)	SWAps/health system strengthening
	Country health emergency preparedness and the International Health Regulations; health emergency information and risk assessment(50%); Emergency operations; Emergency core services; Outbreak and crisis response(50%); Epidemic- and pandemic-prone diseases; Alert and response capacities(50%); Infectious hazard management;	SWAps/health system strengthening, pandemic preparedness
	Social determinants for health; Health and the environment; Food safety; Antimicrobial resistance; gender, equity and human rights mainstreaming	Other
	HIV/AIDS and STIs; HIV/AIDS, TB and malaria (33%)	HIV/AIDS, unspecified
**PAHO**	Tuberculosis; HIV/AIDS, TB and malaria (33%)	Tuberculosis, unspecified
	HIV/AIDS, TB and malaria (33%); Malaria and other Vector-Borne Diseases (50%);	Malaria, unspecified
	Communciable diseases; Malaria and other Vector-Borne Diseases (50%); Neglected Tropical and zoonotic diseases	Other infectious diseases
	Nutrition; Nutrition food safety and food security	Child and newborn nutrition
	Vaccíne-Preventable Diseases	Child and newborn vaccines

Channel	Allocation criteria	Health focus area
	Women, maternal, newborn, child, and adolescent and adult health	Maternal and child health, unspecified
	Mental Health and psychoactive substance use disorders	Non-communicable diseases, mental health
	Non Communicable Diseases and Risk Factors; Chronic noncommunicable diseases	Non-communicable diseases, unspecified
	Health systems leadership and governance; Human resources for health; Social protection and financing; Health systems information and evidence; Health services; People-centered integrated health services; Access to medical products and strengthening regulatory capacity; Health governance and financing, national health policies, strategies and plans	Swap/health system strengthening
	Violence and Injuries; Disabilities and Rehabilitation; Antimicrobial resistance; Aging and health; Gender, equity, human rights and ethnicity; Social determinants of health; Health and the environment; Strategic communications; Management and administration; Flexible and learning organization	Other

S3.1.3 Disaggregating preliminary estimates by health focus area

Estimates by health focus area for years in which descriptive data were not available (usually 2019) were obtained by modeling channel-specific DAH per health focus area as a function of time. Out-of-sample validation was used to test the predictive accuracy of a large suite of models, estimating the models using 1990-2010 data and predicting 2011 and 2012. The potential models included fractional multinomial logit regression, OLS regression, autoregressive integrated moving average (ARIMA) models, Epanechnikov kernel-weighted local polynomial smoothing, and multivariable fractional polynomial models. For each model, time was modeled linearly, with splines, and by including lag-dependent variables. Other methodologies considered included modeling health-focus-area-specific DAH as a dollar amount and as a fraction of the channel-specific total DAH. Lastly, models that involved transforming the dependent variable in natural log and logit transformed space were considered. In order to accommodate zero values in the logit transformation, the transformation described in Smithson and Verkuilen were applied.[73] Over 40 models and specifications were evaluated in total.

Each of the potential model and specification described above were estimated using data from 1990 through 2010, and then the estimated model was used to predict DAH by health focus area for 2011 and 2012. Since we have DAH estimates for 2011 and 2012, we compared the modeled estimates and the observed estimates and calculated average percent deviation and average total absolute deviation for each model and specification across all the channels and health focus areas. A variant of the Epanechnikov kernel-weighted local polynomial smoothing had the smallest average percent deviations and average total absolute error. In this model and specification, health focus area-specific DAH fractions were independently estimated at the channel level after they were logit transformed. Time was the only independent variable included in the model. The health focus area-specific DAH estimates were adjusted so the sum of the channel's health focus area disbursements totaled channel-specific DAH envelope. Our preferred model, the Epanechnikov kernel-weighted local polynomial smoothing, minimized both the average percent deviation and the total absolute error out of sample, predicting two years ahead. See Dieleman et al. for a table that demonstrates the performance of four models, each with their optimal specification (as determined by the out-of-sample average percent deviation and total absolute error).[1]

S3.2 Tracking development assistance for health from bilateral aid agencies and the European Commission

OECD-DAC maintains two databases on aid flows: 1) the DAC annual aggregates database, which provides summaries of the total volume of flows from different donor countries and institutions, and 2) the CRS, which contains project- or activity-level data.[3] This year, we used the DAC databases to track health ODA from 24 OECD-DAC members (Austria, Australia, Belgium, Canada, Denmark, Finland, France, Germany, Greece, Ireland, Italy, Japan, Luxembourg, the Netherlands, New Zealand, Norway, Portugal, South Korea, Spain, Sweden, Switzerland, the United Kingdom, the United States, and the EC), and one DAC Participant country, United Arab Emirates, for the years 1990 to 2019. Observed data for the DAC members was available from 1990 to 2017, and observed data for the United Arab Emirates was available from 2009 to 2017. United Arab Emirates bilateral health ODA from 1990 to 2008 was obtained through personal correspondence.

These two DAC databases track the following types of resource flows:

Official development assistance (ODA), defined as "flows of official financing administered with the promotion of the economic development and welfare of developing countries as the main objective"[2] is tracked from its 30 members (Austria, Australia, Belgium, Canada, Czech Republic, Denmark, Finland, France, Germany, Greece, Hungary, Iceland, Ireland, Italy, Japan, Luxembourg, the Netherlands, New Zealand, Norway, Poland, Portugal, Slovakia, Slovenia, South Korea, Spain, Sweden, Switzerland, the United Kingdom, the United States, and the EC). The CRS also now includes some private ODA, such as that funded by BMGF and the Global Fund to Fight AIDS, Tuberculosis and Malaria (GFATM), as well as assistance from a number of non-DAC countries such as the United Arab Emirates and Kuwait.

ODA includes:

• Bilateral ODA, which is given directly by DAC members as aid to recipient governments, core contributions to NGOs and public-private partnerships, and earmarked funding to international organizations.

• Multilateral ODA, which includes core contributions to multilateral agencies such as WHO, UNFPA, GFATM, Gavi, UNAIDS, UNICEF, PAHO, the World Bank, and other regional development banks. Only regular budgetary contributions to these institutions can be reported to the OECD-DAC; hence, extrabudgetary funds, including earmarked contributions that donors can report as bilateral ODA, are not included as multilateral ODA. Only 70% of core contributions to WHO can be counted as multilateral ODA.

a. Official development finance (ODF), which includes grants and loans made by multilateral agencies.

b. Other official flows (OOF), which refers to transactions that "do not meet the conditions for eligibility as Official Development Assistance or Official Aid, either because they are not primarily aimed at development, or because they have a Grant Element of less than 25 percent."

The DAC aggregate tables include all multilateral development banks, GFATM, operational activities of UN agencies and funds, and a few other multilateral agencies. The project-level data in the CRS cover a smaller subset of multilateral institutions, including UNAIDS, UNFPA, UNICEF, public-private partnerships including Gavi and GFATM, some development banks, and BMGF, but do not reflect the core-funded operational activities of WHO prior to 2009, disbursements by Gavi prior to 2007 and BMGF prior to 2009, or all loans from the World Bank.

This research utilized the CRS as the principal source for tracking bilateral DAH. This is because the DAC aggregate tables do not report detailed project-level information about the recipient country and health focus area. The OECD sector codes for general health (121), basic health (122), and population programs (130) were used to identify health flows in the CRS. Only ODA related flows are used in our analysis, including OECD flow codes corresponding to ODA grants (11), ODA grant-like (12), ODA loans (13), and equity investment (19). In addition, we included select projects with OECD sector code for emergency aid (720) in order to capture additional funding for ebola and zika outbreaks. Projects with purpose code emergency food aid (72040) were excluded, and the

remaining project descriptive variables were parsed for the keywords “EBOLA” and “ZIKA” to identify emergency aid projects targeting these diseases. Based on our definition of DAH, we also excluded any remaining projects containing any of the following keywords: " WATER " " SANITATION " " FOOD " " EARTHQUAKE " " HURRICANE " " TERREMOTO " " HURACAN " " FAMILY CARE STRUCTURES ".

To avoid double-counting, all identifiable earmarked commitments and disbursements between one disbursing agency to another were tagged and removed before calculating final DAH estimates. Income statements, expenditure statements, and keyword searches in the descriptive project fields were used to identify potential sources of double-counting.

In multilateral agencies, public-private partnerships, development banks, and foundations, when expenditure by recipient agency information was available, double counting was tagged and dropped in the disbursing agency from whom the resources was transferred. In the absence of detailed expenditure data in the disbursing agency that transferred resources, the income statement of the receiving disbursing agency that reflected the disbursement was used to flag the disbursement in the initial disbursing agency. Preliminary estimates were generated based on the annual disbursed envelope exclusive of resources flagged as transfers to other agencies.

Channel codes in the CRS data were used to track DAH to international and donor-country-based non-government organizations. The names of NGOs that were captured in IHME’s NGO data (as detailed in section 3.7 titled “Tracking non-governmental organizations”) were searched for in the CRS descriptive variables and tagged as double-counting. Research funds for HIV/AIDS channeled by the US government through the National Institutes for Health (NIH) were also removed from the total since they do not meet the definition of DAH as contributions from institutions whose primary purpose is development assistance. Official development finance (ODF) from the CRS was not counted because these expenditures were included elsewhere, either in the analysis of multilateral institutions relevant to the study or in the assessment of health spending by BMGF, the data for which were obtained via correspondence and from their annual reports, audited financial statements, and project databases. To avoid double-counting, only health assistance flows from multilateral institutions to low- and middle-income countries were counted, and not transfers to multilateral institutions. Also, for regional projects the disbursements are split amongst all countries in the specified OECD region. For example, a project allocated to recipient “North of Sahara, regional” would have its disbursements split equally between all the countries in the corresponding OECD region: Algeria, Egypt, Libya, Morocco, and Tunisia.

Allocation of funding to health focus areas was assigned as described in the section “DISAGGREGATING BY HEALTH FOCUS AREA”, based on a keyword search of five descriptive variables in the CRS: project title, short description, long description, channel name, and channel reported name. Additional adjustments were made based on CRS purpose codes, as detailed in Table S3.5, in order to ensure that the specified purpose corresponded to the highest-weighted **bilateral** health focus area.

S3.2.1 Estimating disbursements for the 24 channels and the EC

Both the DAC tables and the CRS rely on information reported by DAC members and other institutions to the OECD-DAC. Hence, the quality of the data varies considerably over time and across donors. Three variables were used to estimate yearly donor disbursements: CRS commitments, CRS disbursements, and DAC commitments. There were two main challenges in using the data from the CRS for this research:

1. underreporting of aid activity to the CRS compared to what is reported to the DAC, and

2. underreporting of disbursement data to the CRS compared to commitment data reported to the CRS.

These issues are highlighted in Figure S3.1. Methods developed to account for both these challenges are discussed below. Details on how we estimated the cost of providing technical assistance and program support for these institutions are highlighted below in the section titled calculating the ‘Technical assistance and program support component of development assistance for health from loan-and grant-making channels of assistance’.

To address these two challenges, we determined a cutoff point for each channel. We defined this channel-specific cutoff year as when the ratio of total CRS disbursements to commitments was greater than 50% and did not drop subsequently below 30%. Figure S3.2 below shows each donor's CRS disbursement to commitment ratio in green, and the estimated cutoff year is marked with a vertical red line. For years after the cutoff year, DAH is measured using the unadjusted disbursement data. For the time prior to the cutoff year, it was determined that the disbursement data are not of high enough quality, and adjusted commitments were used instead.

Two adjustments were made to commitments to estimate disbursements before each donor-specific cutoff point:

I. The first adjustment addressed underreporting of aid activity to the CRS (relative to the DAC). To address this challenge, all CRS commitments for the health sector were adjusted upward using the DAC commitment to CRS commitment coverage ratio. The coverage ratio of the CRS was well below 10% before 1996 but has improved steadily over time.

II. The second adjustment addressed underreporting of disbursements data to the CRS (relative to commitments reported to the CRS). To address this challenge, we pooled completed projects in the CRS that have disbursement data for each channel and computed yearly project disbursement rates (the fraction of total commitments disbursed for each year of a project) and overall project disbursement rates (the fraction of total commitments disbursed over the life of each project) by project length. Yearly disbursement schedules were calculated for projects with lengths of one, two, three, four, five, and six years. When an observed project length was more than six years, all expenditure after the sixth year was aggregated and assumed to be expended in the sixth year. This does not happen often. Yearly disbursement rates were the median of these shares, averaged across projects for every donor in each project year. The sum of these averages equals one, so that all the disbursements were expended over the lifetime of a project. We also adjusted donor disbursement schedules by the ratio of disbursements to commitments in the CRS in order to reflect the fact that not all commitments are typically disbursed. The product of these donor-specific yearly disbursement rates, the donor-specific overall disbursement rates, and the donor-specific disbursement to commitment ratios produced the donor-specific disbursement schedules. The donor-specific disbursement schedules were applied to project-level DAC-adjusted commitments reported in the CRS. Figure S3.3 shows the yearly disbursement rates and overall disbursement rates for projects with one- to six-year lifespans for each of the 24 member countries and the EC.

Lastly, to address the challenge of underreporting of aid activity to the CRS compared to the DAC for all years, the difference between each donor's aggregate DAC health commitments and CRS health disbursements was added to each donor's yearly DAH. Since only aggregate commitments are reported to the DAC, several adjustments were made, based on more detailed CRS data:

I. First, each donor's yearly median project length was calculated by applying the donor-specific disbursement schedules described above to CRS projects that had disbursement in order to get adjusted DAC commitments.

II. Commitments for projects that have not opened yet were then subtracted, based on the open date reporting in the CRS. This ensured that future disbursements were not captured.

III. The donor-specific ratio of disbursements to commitments was applied to adjusted DAC commitments in order to account for the fact that not all commitments are usually disbursed.

IV. Lastly, these DAC-adjusted commitments were compared to CRS disbursements, inclusive of transfers that were later dropped as double-counting.

In addition to tracking disbursements from the EC, gross disbursements from the DAC were used to compile data on the sources of funding for the EC.

Figure S3.1 Comparing CRS commitments, CRS disbursements, and DAC commitments

This figure compares commitments and disbursements from the Creditor Reporting System (CRS) and Development Assistance Committee (DAC) databases of the Development Assistance Committee of the Organisation for Economic Co-operation and Development (OECD-DAC) from 1990 to 2017. CRS disbursements are usually underreported when compared to both CRS and DAC commitments data, especially in earlier years. Because of this gap between CRS and DAC, CRS disbursements data were adjusted to fit DAC commitments data.

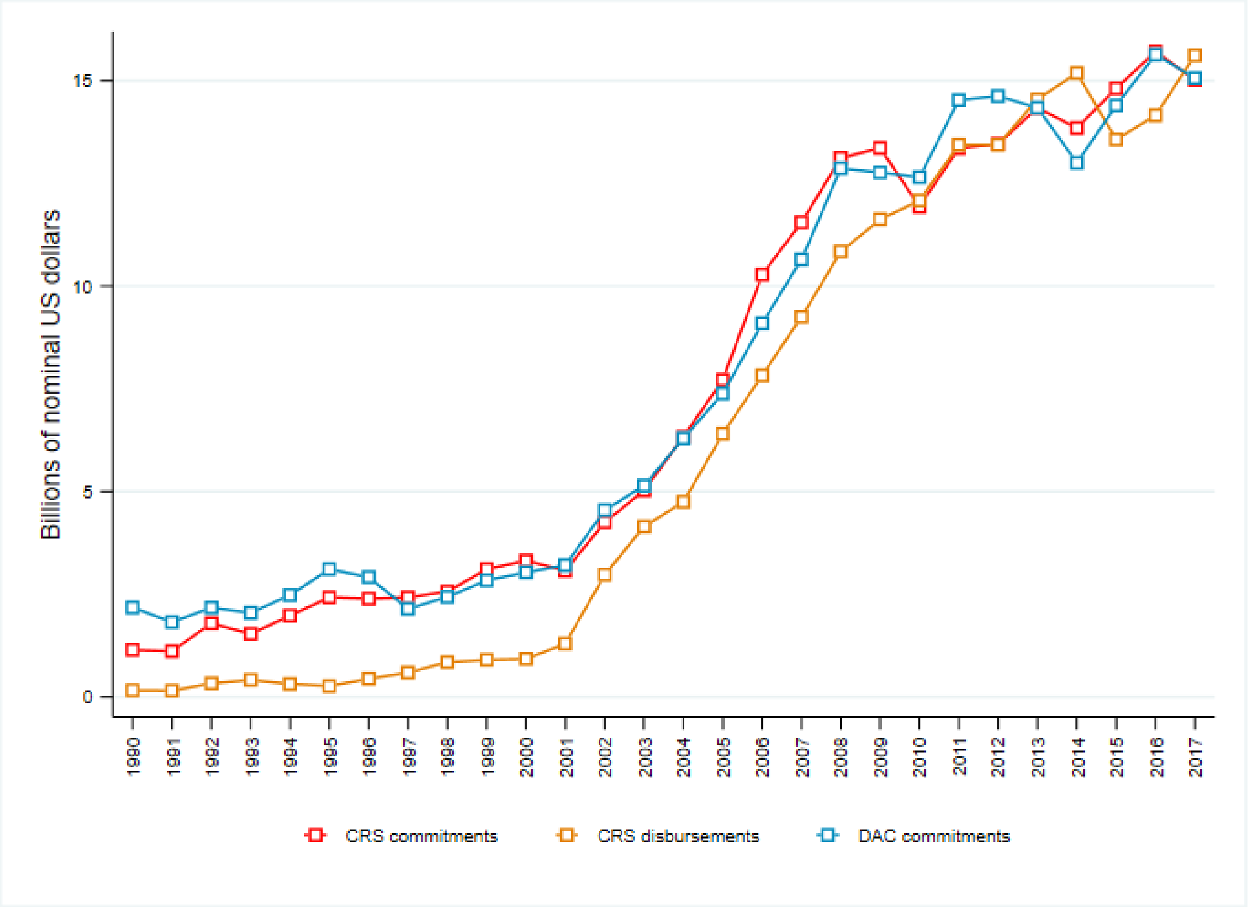

Source: OECD-DAC and OECD Creditor Reporting System

Figure S3.2 CRS disbursement to commitment ratio and cutoff points by donor agency

This figure shows the channel-specific cutoff year. Before this year, we adjust CRS commitments using disbursement schedules. After this cutoff we rely on CRS-reported disbursements. The total CRS disbursements to commitments ratio is in green, and the cutoff year is marked with a vertical red line. The cutoff year is determined to be when the ratio goes above 50% and does not fall back below 30%. The vertical axis represents the CRS disbursement to commitment ratio as a percentage. ARE = United Arab Emirates, AUS = Australia, AUT = Austria, BEL = Belgium, CAN = Canada, CHE = Switzerland, DEU = Germany, DNK = Denmark, EC = European Commission, ESP = Spain, FIN = Finland, FRA = France, GBR = Great Britain, GRC = Greece, IRL = Ireland, ITA = Italy, JPN = Japan, KOR = South Korea, LUX = Luxembourg, NLD = the Netherlands, NOR = Norway, NZL = New Zealand, PRT = Portugal, SWE = Sweden, USA = United States of America.

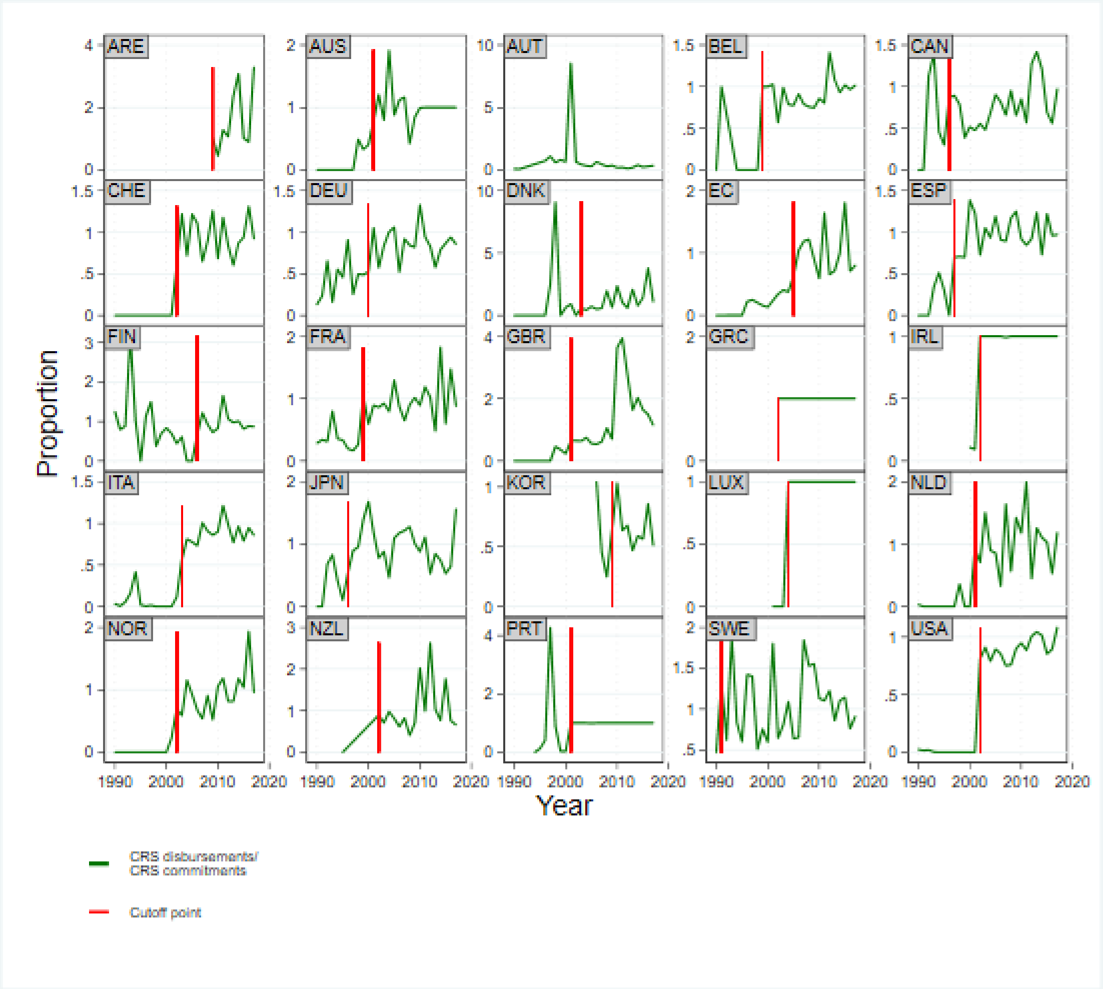

Source: OECD Creditor Reporting System

**Box 3.3. EXAMPLE. Australia's CRS disbursement to commitment ratio and cutoff year**
**The green line shows the ratio of Australia's disbursements to commitments, as reported in the CRS. Prior to 2001, the ratio was always below 50%. In 2001, the ratio rose above 50%; it did not fall below 30% in subsequent years, thereby defining 2001 as the cutoff year. Thus, for Australia, before 2001 DAH is based on adjusted CRS commitment data. These data are adjusted using disbursements schedules (Figure S3.3) and data from the DAC. After 2001, Australia's DAH is based on the disbursements reported in the CRS.**

Figure S3.3 One- to six-year disbursement schedules for bilateral channels

This figure shows the estimated disbursement schedules for bilateral channels. Before the channel-specific cutoff year, we rely on commitment data to inform our estimates of DAH. Commitment data are adjusted to reflect disbursements over time using schedules estimated from projects in the CRS that have both commitment and disbursement data. The vertical axis represents the percentage of the commitment disbursed. ARE = United Arab Emirates, AUS = Australia, AUT = Austria, BEL = Belgium, CAN = Canada, CHE = Switzerland, DEU = Germany, DNK = Denmark, EC = European Commission, ESP = Spain, FIN = Finland, FRA = France, GBR = Great Britain, GRC = Greece, IRL = Ireland, ITA = Italy, JPN = Japan, KOR = South Korea, LUX = Luxembourg, NLD = the Netherlands, NOR = Norway, NZL = New Zealand, PRT = Portugal, SWE = Sweden, USA = United States of America

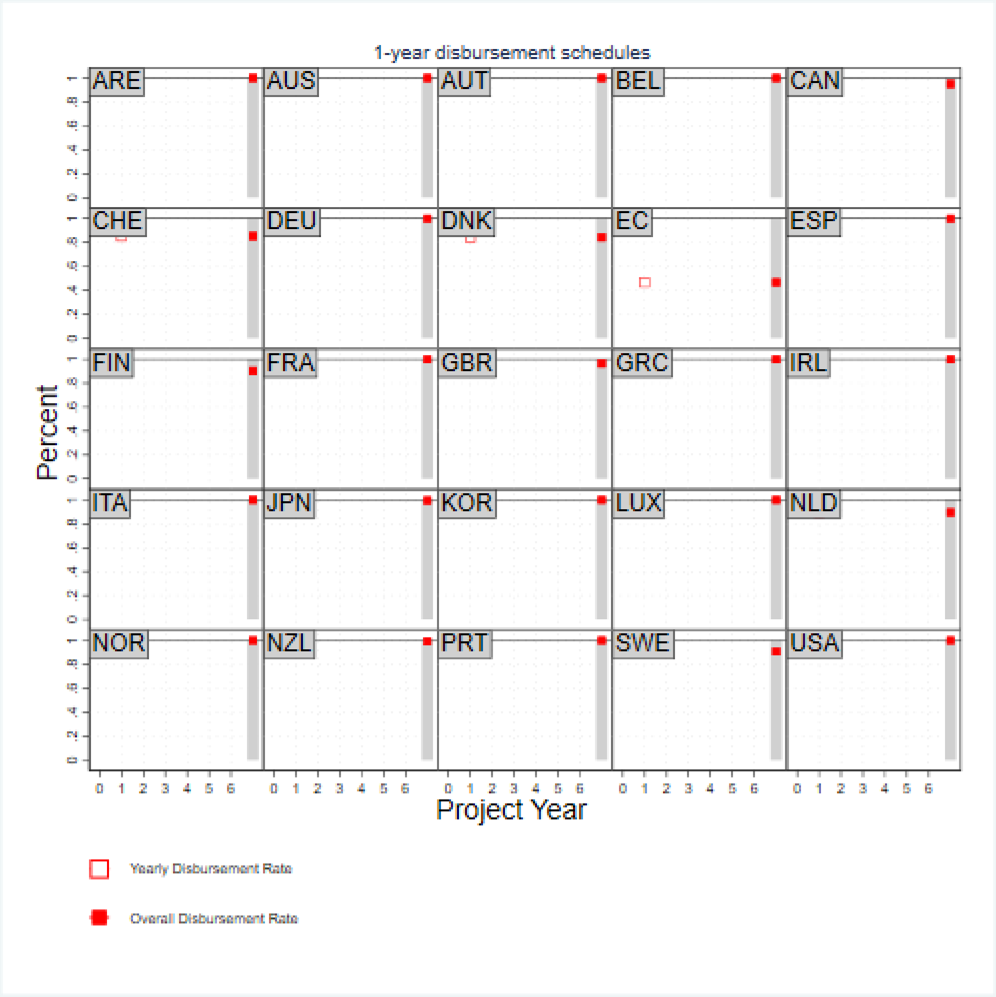
1-year disbursement schedules
ARE
AUS
AUT
BEL
CAN
CHE
DEU
DNK
EC
ESP
FIN
FRA
GBR
GRC
IRL
ITA
JPN
KOR
LUX
NLD
NOR
NZL
PRT
SWE
USA
Percent
Project Year
Yearly Disbursement Rate
Overall Disbursement Rate

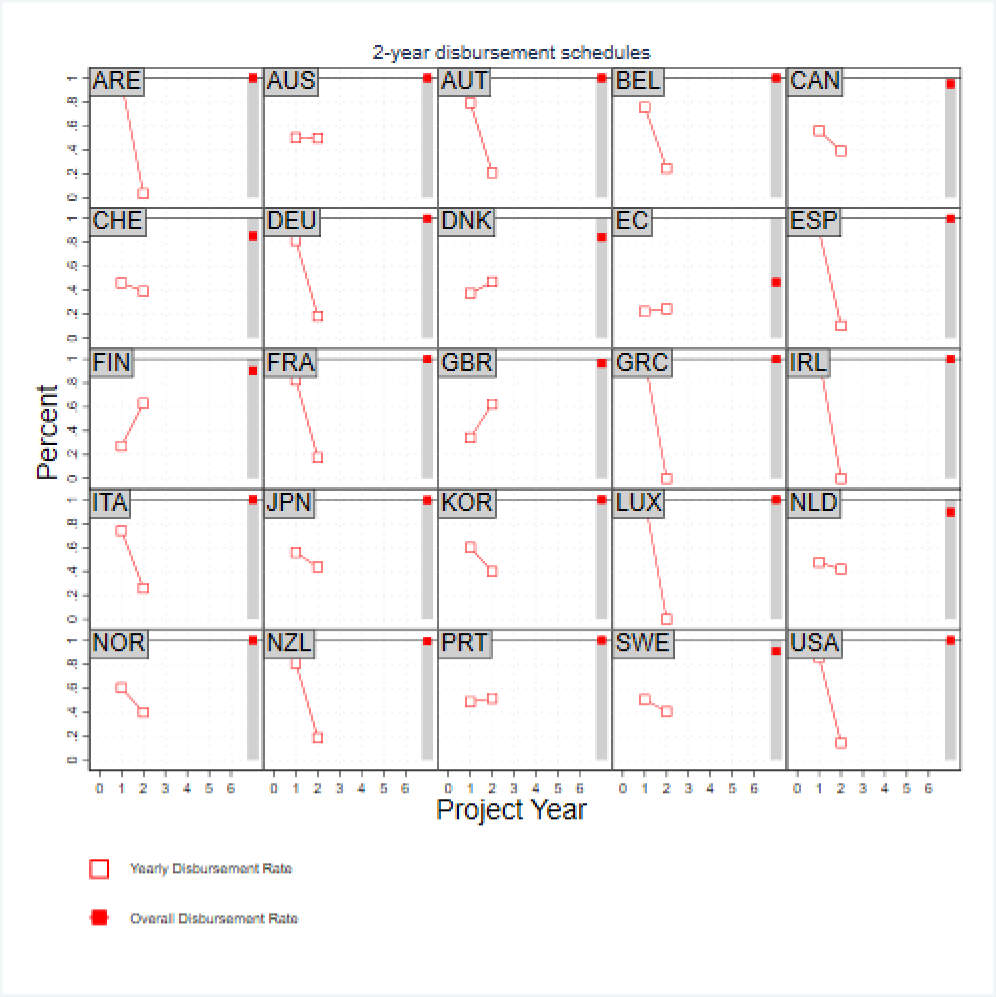

2-year disbursement schedules
ARE
AUS
AUT
BEL
CAN
CHE
DEU
DNK
EC
ESP
FIN
FRA
GBR
GRC
IRL
ITA
JPN
KOR
LUX
NLD
NOR
NZL
PRT
SWE
USA
Percent
Project Year
Yearly Disbursement Rate
Overall Disbursement Rate

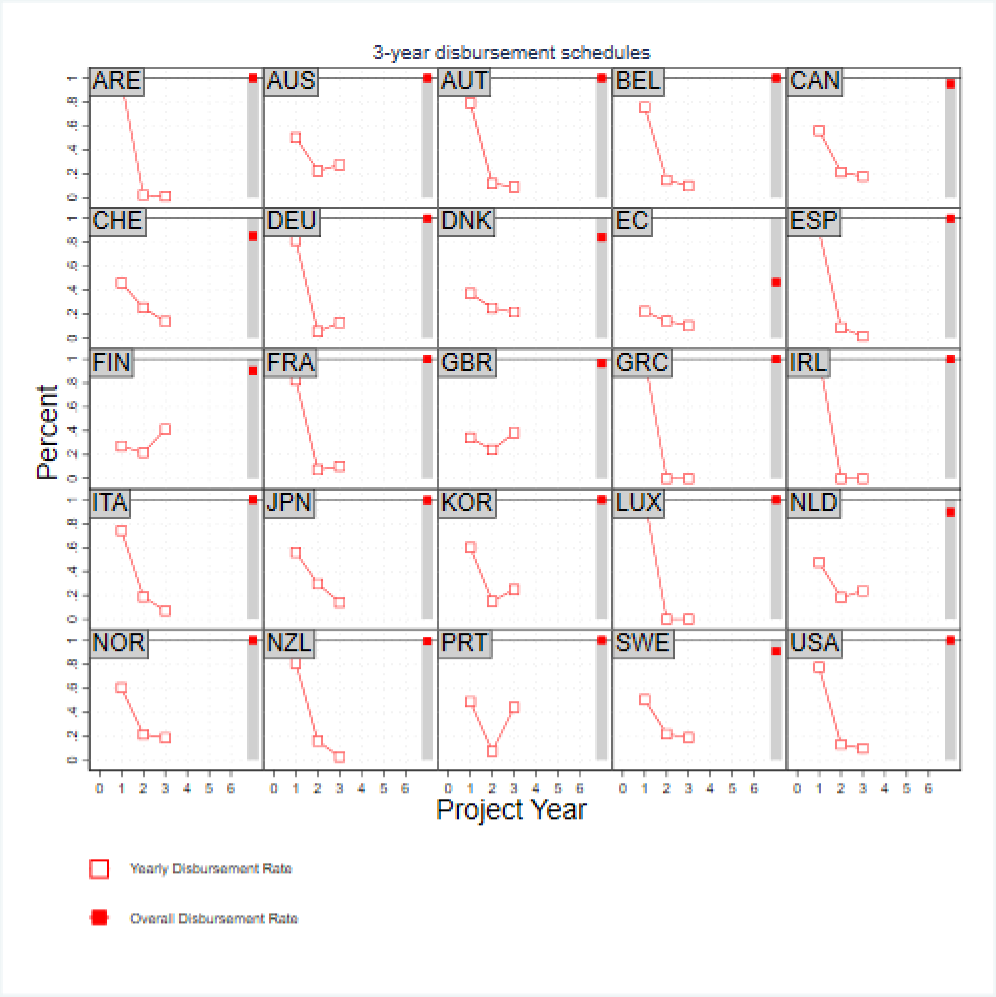

3-year disbursement schedules
ARE
AUS
AUT
BEL
CAN
CHE
DEU
DNK
EC
ESP
FIN
FRA
GBR
GRC
IRL
ITA
JPN
KOR
LUX
NLD
NOR
NZL
PRT
SWE
USA
Percent
Project Year
Yearly Disbursement Rate
Overall Disbursement Rate

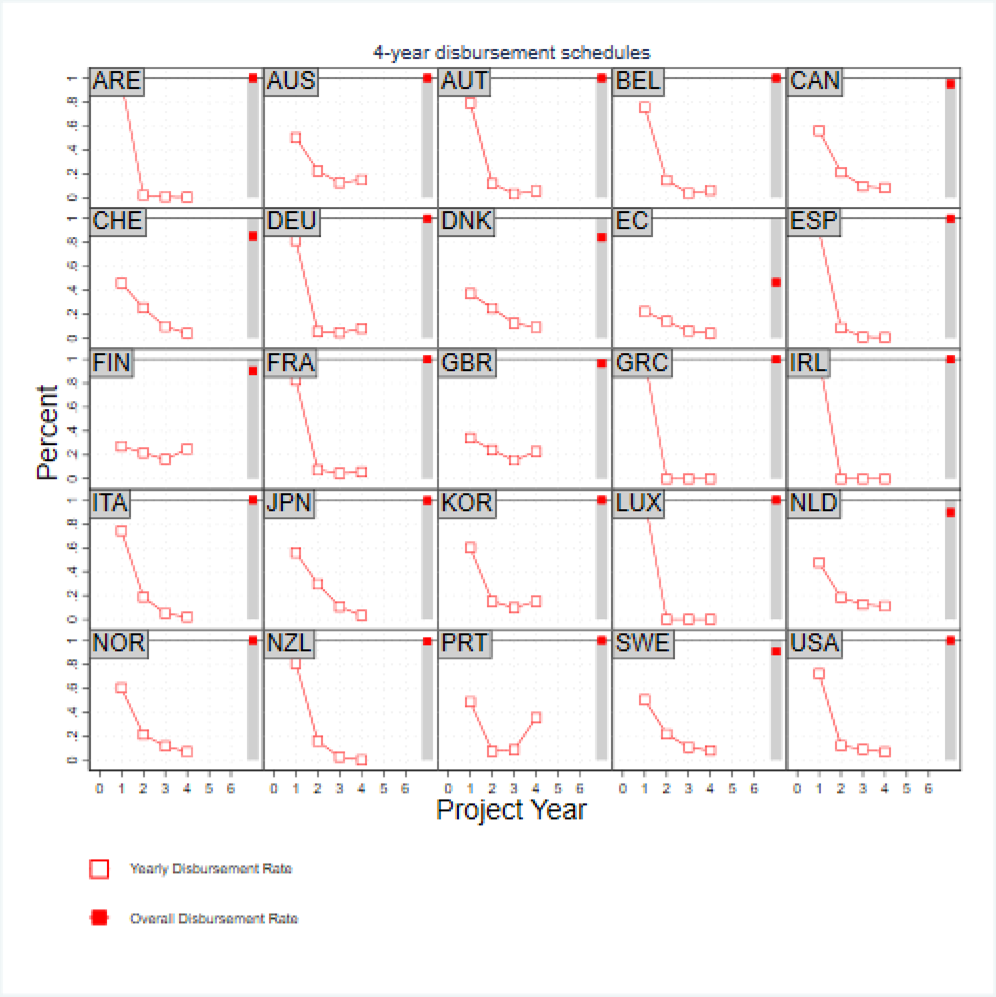

4-year disbursement schedules
ARE
AUS
AUT
BEL
CAN
CHE
DEU
DNK
EC
ESP
FIN
FRA
GBR
GRC
IRL
ITA
JPN
KOR
LUX
NLD
NOR
NZL
PRT
SWE
USA
Percent
Project Year
Yearly Disbursement Rate
Overall Disbursement Rate

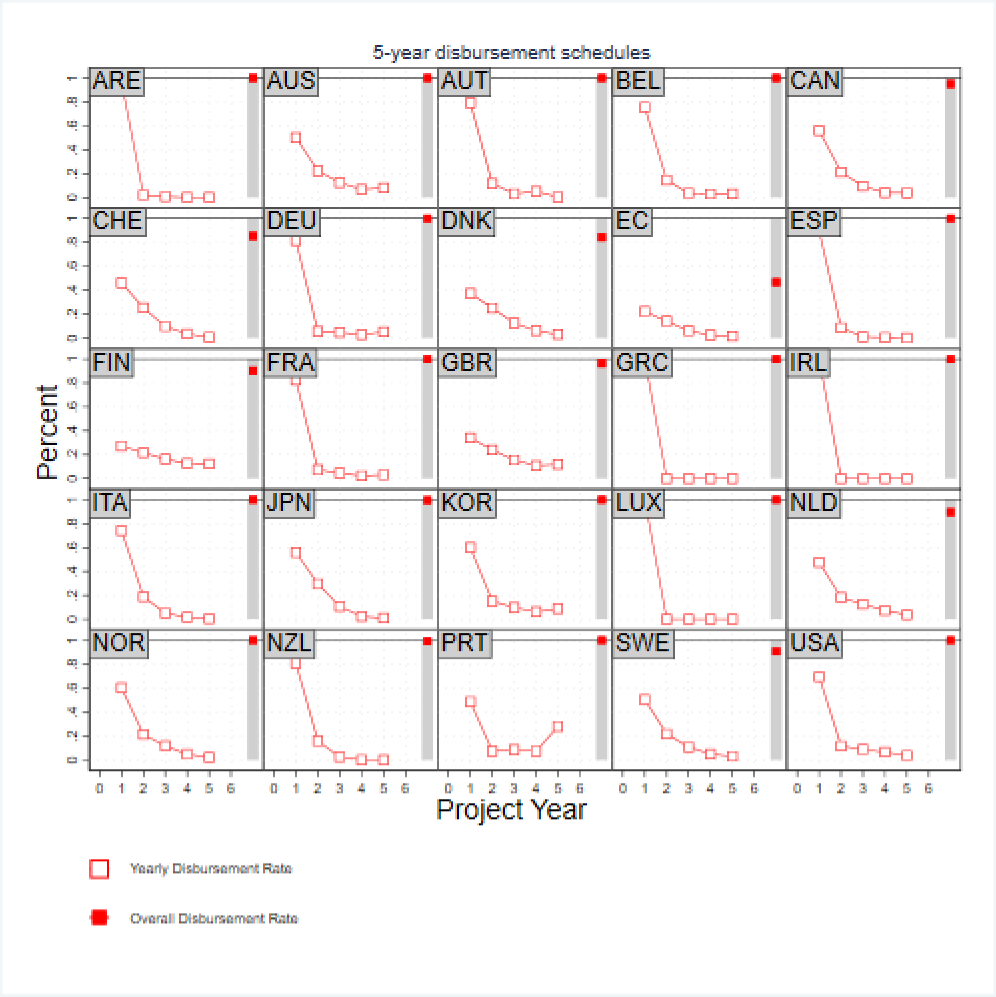
5-year disbursement schedules
ARE
AUS
AUT
BEL
CAN
CHE
DEU
DNK
EC
ESP
FIN
FRA
GBR
GRC
IRL
ITA
JPN
KOR
LUX
NLD
NOR
NZL
PRT
SWE
USA
Percent
Project Year
Yearly Disbursement Rate
Overall Disbursement Rate

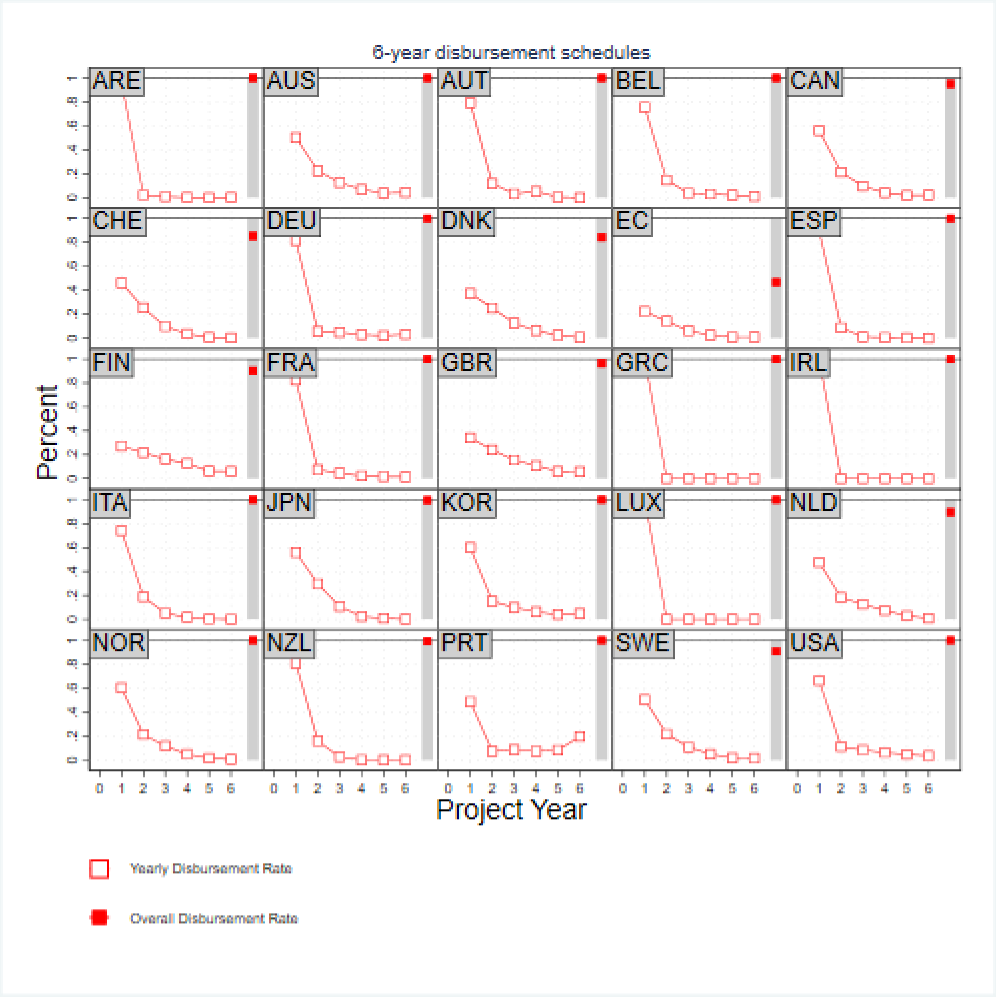
6-year disbursement schedules
ARE
AUS
AUT
BEL
CAN
CHE
DEU
DNK
EC
ESP
FIN
FRA
GBR
GRC
IRL
ITA
JPN
KOR
LUX
NLD
NOR
NZL
PRT
SWE
USA
Percent
Project Year
Yearly Disbursement Rate
Overall Disbursement Rate

**Box 3.4. EXAMPLE. Australia's one- to six-year disbursement schedules**
**To estimate disbursements using commitment data, we rely on disbursement schedules derived from CRS data that include both commitments and disbursements. Disbursement schedules are specific for each channel and the length of a project. These schedules also take into consideration the average amount of commitments for each channel that lead to disbursements. Across all Australian projects in the CRS with complete disbursements data, Australia disbursed 100% of the funds that it committed, as shown by the solid red dot on the right-hand side of the Australia panel (upper left corner of the first panel of Figure S3.3). In projects with a length of one year, Australia disbursed 100% of the funds that it committed in that year. For two-year projects, Australia disbursed 50% of total disbursements in year one and 50% of total disbursements in year two. In projects with lengths of three years, Australia disbursed about 50% of total disbursements in year one and 23% and 27% of total disbursements in years two and three, respectively. This is estimated for projects ranging from one to six years. The disbursement schedules were applied to commitment data from the CRS to estimate disbursements for years prior to the cutoff year, which is 2001 for Australia.**

To predict DAH for the recent years not reported in the CRS, budget data were extracted from a variety of sources. These data are listed in Table S3.3. Global health budgetary data were utilized whenever possible, but these detailed data were available as a complete time series only for Australia and the United States. For all other bilateral channels, general ODA budgets were used. In order to predict DAH for 2018-2019 for 24 bilateral agencies, the budget ratio for each donor was calculated by dividing DAH estimates by the corresponding budget data (ODA or global health). Budget ratios were projected using a weighted average of the previous three years (placing one-half weight on the one-year lagged ratio, one-third weight on the two-year lagged ratio, and one-sixth weight on the three-year lagged ratio), and this ratio was multiplied by the observed budgeted DAH for those same years. Figure S3.4 plots the budget ratio for each bilateral channel. Budget data for the EC were inconsistent and did not match the disbursement series. Instead, DAH for 2018-2019 was estimated based on trends in DAH for EC member countries. A weighted average was applied to the percent change in DAH from 2016-2019 for all EC member countries. The weighting was based on each country's total national contributions to the EC. These data were collected from the EC's financial statements.[74] The weighted average was then applied to the EC's 2017 DAH to forecast 2018-2019.

Figure S3.4 DAH as a percentage of corresponding budget data by bilateral agency

This figure shows the trend of the ratio of DAH measured as a share of budget data. Green dots indicate that a donor provided global-health-specific budget data, so in these cases the denominator is all global-health-specific budgeted data. The numerator is estimated DAH. Red dots indicate that a donor did not have global-health-specific budget data, so overall ODA budget data were used in calculating the DAH to budget ratios. The vertical axis represents estimated DAH as a fraction of corresponding budget data. ARE = United Arab Emirates, AUS = Australia, AUT = Austria, BEL = Belgium, CAN = Canada, CHE = Switzerland, DEU = Germany, DNK = Denmark, ESP = Spain, FIN = Finland, FRA = France, GBR = Great Britain, GRC = Greece, IRL = Ireland, ITA = Italy, JPN = Japan, KOR = South Korea, LUX = Luxembourg, NLD = the Netherlands, NOR = Norway, NZL = New Zealand, PRT = Portugal, SWE = Sweden, USA = United States of America.

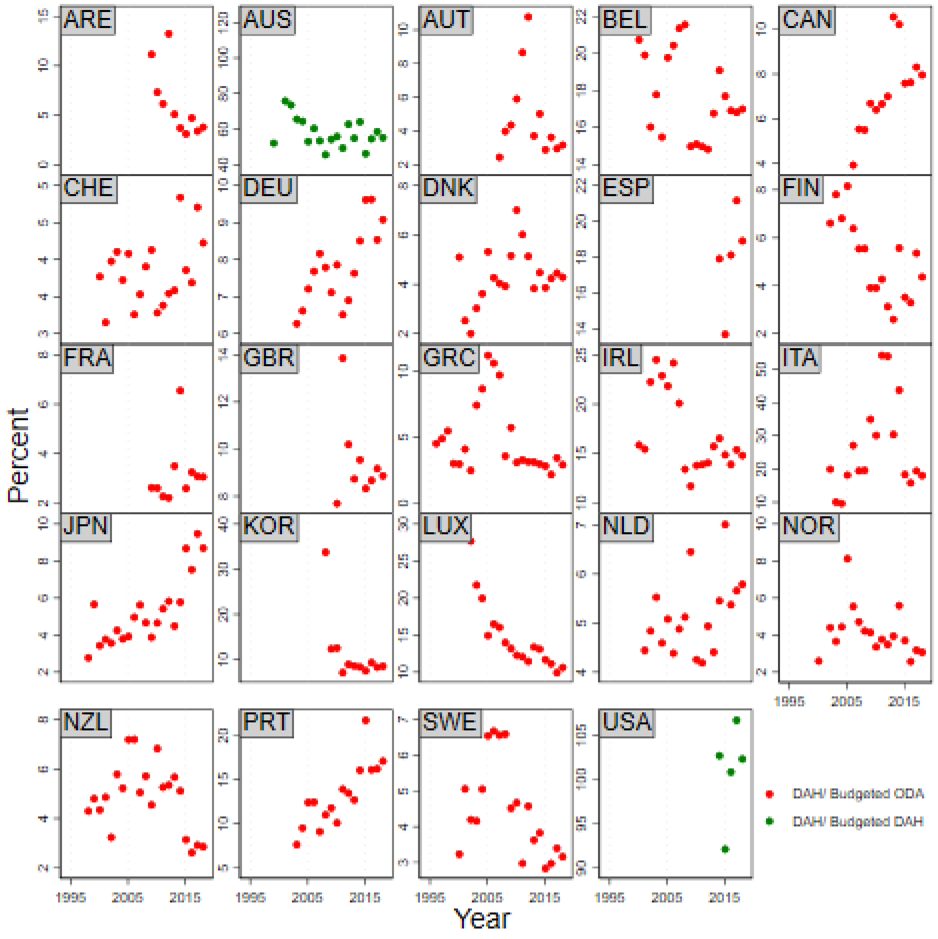

Source: IHME DAH Database (2019) and corresponding bilateral ODA/DAH budget documents outlined in Table S3.1 and 3.3.

**Box 3.5. EXAMPLE. Australia's DAH as a percentage of corresponding budget data**
Australia provided global-health-specific budget data for 1998-2019 through its International Development Assistance and Overseas Aid Program budgets. For 1998-2017, health ODA and observed DAH were used to create DAH to budget ratios. These budget ratios were then applied to 2018-2019 health ODA budget data to project DAH in 2018-2019, using a weighted average: $$(Total\ DAH_t) = \left(\frac{1}{2}\right)(Budget\ ratio_{t-1})\ (Budgeted\ GHE_t)\ + \left(\frac{1}{3}\right)(Budget\ ratio_{t-2})(Budgeted\ GHE_t) + \left(\frac{1}{6}\right)(Budget\ ratio_{t-3})(Budgeted\ GHE_t)$$ where t = year to be modeled.

To supplement our estimates of development assistance for health to HIV/AIDS and malaria program areas for the United States, we used additional available data from the President's Emergency Plan for AIDS Relief (PEPFAR) and the President's Malaria Initiative (PMI). We downloaded data on all planned funding by PEPFAR by recipient country, year, and program area from 2004 to 2019.[75] All PEPFAR projects were assigned to our eight HIV/AIDS program areas using PEPFAR budget codes, splitting out overhead costs equally to all other program areas. We then created country-year specific HIV/AIDS program area fractions out of total annual HIV/AIDS DAH, which we applied to all United States HIV/AIDS projects in the CRS from 2004 to 2017 by country-year. To inform malaria

funding by program areas, we downloaded the most recently available malaria funding tables from malaria operational plans for all countries and years.[76] We used a keyword search to assign each line item in these tables to our eight malaria program areas, and then created fractions for the malaria program areas out of the total annual malaria DAH specific to each country-year. These fractions were applied to all United States malaria projects in the CRS from 2006 to 2017 by country-year.

Figure S3.5 Malaria DAH to program areas as assigned by keyword search and PMI reports

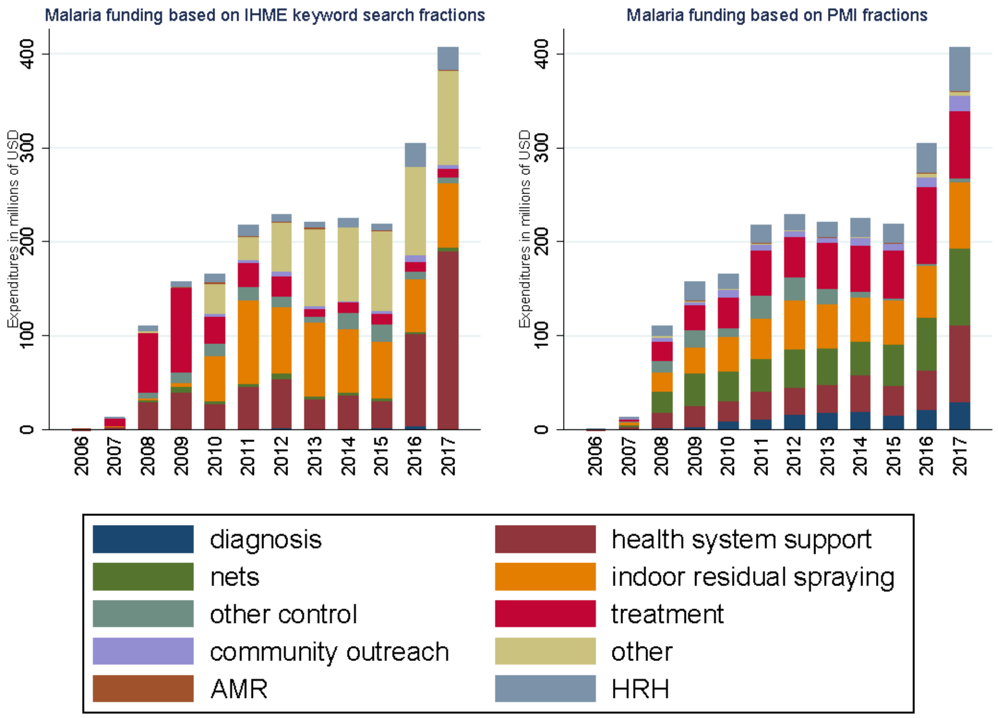

Source: IHME DAH Database (2019) and PMI malaria operational plans

This figure outlines the assignment of funding to malaria program areas for United States projects from the OECD CRS from 2006 onwards. The figure on the left shows how malaria funding is broken out based on keyword search. The figure on the right shows the breakdown of funding to malaria program areas based on PMI malaria funding tables. Using the data from PMI reduces the amount of funding allocated to the "other" program area. As such, in as often as more disaggregated information on project allocation is available, IHME uses such project information available in project budget documents or other project documents to disaggregate into program areas.

S3.3 Tracking development assistance for health from the development banks

The World Bank project-level health disbursement data for 1990 through September 2019 were obtained through correspondence with Kathleen E. Krackenberger and Miyuki T. Parris , Operations Analysts.[77] The World Bank recently underwent a recoding process for their disbursements. This recoding affected health disbursements, however the recoding was not completed for projects with disbursements prior to 2001. To create a comparable dataset adjustments had to be made. Regression analysis to predict health disbursements were explored, however, in the end the average percent change between project-level health disbursements before and after recoding was used

to adjust health disbursements prior to 2001. It was observed that on average, between 2001 and 2005 (inclusive) the recoding process decreased health disbursements by 0.22%. This number was used to adjust all project-level health disbursements prior to 2001.[78] Health disbursements included all health projects as well as other sector projects with a health sector code. In addition, data were collected from the World Bank online loans database in order to fill in descriptive information for loans from the two arms of the World Bank: the International Development Association (IDA) and the International Bank for Reconstruction and Development (IBRD).[79] Along with keyword searches, health theme codes were used to allocate disbursements by health focus area. The online database contains up to five sector codes and five theme codes that can be assigned to each project. Sector codes represent economic, political, and social subdivisions, while theme codes represent the goals or objectives of World Bank activities. The codes are summarized in Table S3.6. Emergency recovery loans were excluded since they do not fit the definition of DAH.

Table S3.6 World Bank's health sector and theme codes

**Health sector codes**<br>**Sector codes represent economic, political, or social subdivisions within society. World Bank projects are classified by up to five sectors.**	**Health theme codes**<br>**Theme codes represent the goals or objectives of World Bank activities.**
**Historic (prior to 2001):**<br>(1)**Basic health**<br>(2)**Other population health and nutrition**<br>(3)**Targeted health**<br>(4)**Primary health, including reproductive health, child health, and health promotion**<br><br>**Current (as of 2001):**<br>**(1) Health**<br>**(2) Compulsory health finance**<br>**(3) Public administration – health**<br>**(4) Noncompulsory health finance**	Current:<br>(1) HIV/AIDS<br>(2) Malaria<br>(3) Tuberculosis<br>(4) Other communicable diseases<br>(5) Population and reproductive health<br>(6) Child health<br>(7) Nutrition and food security<br>(8) Injuries and non-communicable diseases<br>(9) Health system performance<br>(10) Social analysis and monitoring

Data on yearly government contributions were obtained from the DAC statistics in order to disaggregate IDA flows by source. Because China does not report to DAC, we generated contribution estimates using replenishment data. We split the 3-year replenishment amount over the three years of the 16th-19th replenishment, to obtain China's contributions to the World Bank from 2008-2017. We validated our use of replenishment data by extracting China's and other countries' contributions to the World Bank International Development Association (IDA) 16th, 17th and 18th replenishment and comparing the number with the contribution we extracted from the OECD Creditor's Reporting System (CRS)[80]. The trend of contribution from major donors was similar, we therefore used China's replenishment contribution as a proxy of the contribution to World Bank IDA. We further disaggregated the DAH contribution to World Bank IDA from China by multiplying China's contributing proportion over World Bank IDA's total health envelope.

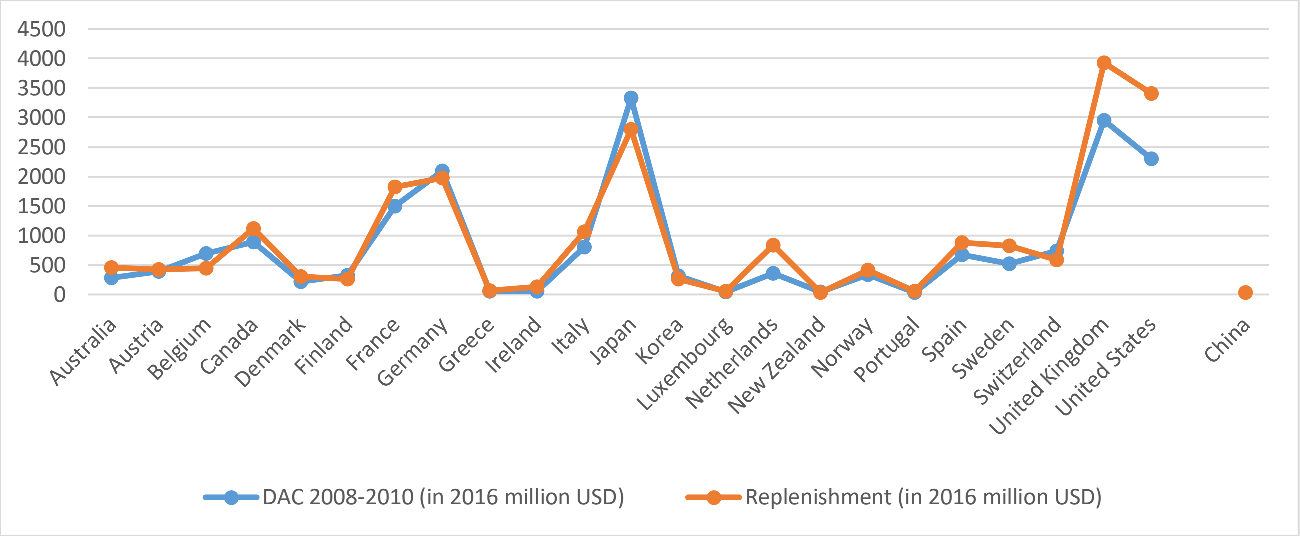

Panel a. Comparison of World Bank International Development Association 16th Replenishment

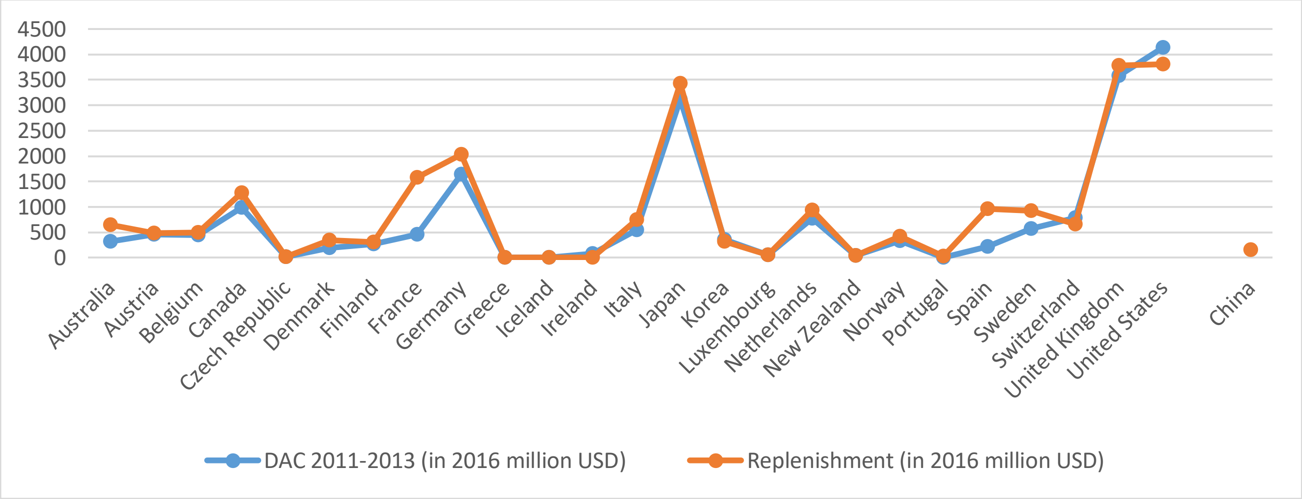

Panel b. Comparison of World Bank International Development Association 17th Replenishment

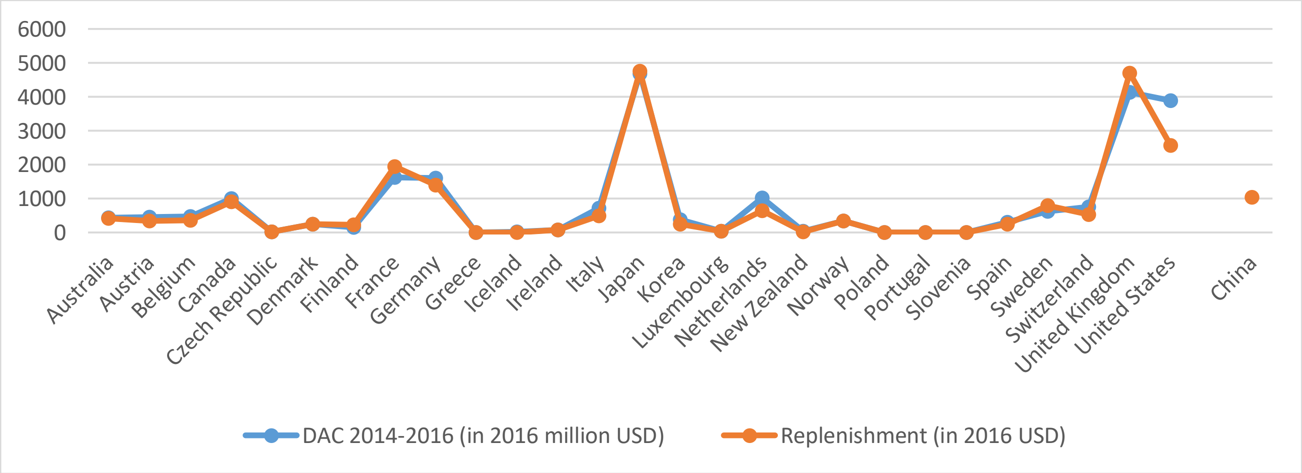

Panel c. Comparison of World Bank International Development Association 18th Replenishment

Figure S3.6 Comparison of World Bank IDA Replenishment and DAC

The three figures show the comparison of country commitment to World Bank IDA replenishment rounds, and yearly government contributions reported to DAC statistics. The orange line shows the country commitment to each IDA replenishment round. The blue line shows the sum of yearly reported contribution during the three-year replenishment round. Panel a is contributions and commitments from replenishment round 16; panel b is contributions and commitments from replenishment round 17; panel c is contributions and commitments from replenishment round 18.

Details on how we estimated the cost of providing technical assistance and program support for these institutions are highlighted below in the section titled "Calculating the technical assistance and program support component of development assistance for health from loan-and grant-making channels of assistance." The data received from the World Bank captured disbursements for Jan 1st to Sep 15th of 2019, so the 2019 total estimate for IDA and IBRD were rescaled to 12 months.

S3.3.1 Regional development banks

The Asian Development Bank (ADB), and Inter-American Development Bank (IDB) all maintain their own loan databases, which were used to estimate disbursements.[15,16,81] To estimate health disbursements from the African Development Bank (AfDB), data was received via correspondence with Mr. Yomi Ayodabo (Division Manager, Loan accounting) .[35] To estimate health disbursement from the Asian Development Bank (ADB), data were received via correspondence with Dr. Rouselle F. Lavado. Table S3.7 provides a summary of the data sources used across the regional banks. Furthermore, Figure S3.6 displays the disbursements for AfDB from 1990 to 2019 and eFigures 3.7, and 3.8 display commitments and disbursements from 1990 to 2019 for ADB and IDB.

For AfDB, we received project level disbursement data from 2001 through 2019. For pre-2001 estimates, data from the Compendium of Statistics were used. Donor information for projects funded by the African Development Fund was based on subscription information from the 9th to 14th replenishment reports[82]. The sole donor to projects implemented by the Nigeria Trust Fund was Nigeria, and all income for African Development Bank projects was attributable to other sources.

For ADB, we received project-level disbursement data from 1990 through 2019, including recipient, project title and description and annual disbursement. A keyword search was performed to identify health focus areas of the projects. For income source of ADB, data for country donations were extracted from the replenishment reports of Asian Development Fund and we manually divide the three or four years' replenishment into annual contributions[83]. We extracted the total revenue of Ordinary Capital Resources and Asian Development Fund from the Financial reports of ADB to generate the total envelope of income sources and subtracted the country contribution to provide the estimate for resources coming from the bank itself. Using the fraction for the Bank's own resources and country contribution, we provided the estimate of income source for each project.

The IDB's project database also provided commitments and disbursements for all projects. The same methods were used for estimating annual disbursements from the IDB as were used for the ADB. Through correspondence, 2019 health loan disbursements were obtained. These numbers were used in the 2019 estimates. The dataset used to estimate disbursements for IDB was updated in November 2019. However, since these channels have so few new projects each year, it was assumed that smoothing disbursements over time for reported projects captured the majority of total disbursements for 2019. The contribution quotas by donor for the Fund for Special Operations from 1990-2016 were extracted from Annual Financial reports for the IDB. The ratio of each donor's contribution to total contribution was applied to total disbursements to assign income sources. In 2017, the Fund for Special Operations was transferred to the Ordinary Capital. Donor assignments in 2017 onwards are unallocable.

Table S3.7 Summary of data sources for the regional development banks

This figure indicates the data available and used to estimate DAH. (X) indicates that project-level data are present in the dataset. (-) indicates that project-level data are not present in the dataset.

Institution	Data source	Commitments	Cumulative disbursements	Yearly disbursement	Notes
**African Development Bank (AfDB)**	Compendium of Statistics	X	-	(Aggregate - not at the project level)	The Compendium of Statistics was not available for 1990-1993, 1995, and 1998-1999; we estimated yearly disbursements using the average of neighboring disbursements
	Correspondence	-	-	X	Annual loan disbursements from 2001 through August 2019 were provided.
**Asian Development Bank (ADB)**	Correspondence	X	X	X	Annual grant loan disbursements from 1990 through 6 November 2019 were provided.
**InterAmerican Development Bank (IDB)**	Online projects database	-	X	-	As yearly disbursement amounts are not provided in the online database, we estimated yearly disbursements by allocating cumulative disbursements over each year of the project.
	Correspondence	-	-	X	Loan disbursements from January through 4 September 2019 were provided.

Figure S3.7 Disbursements by the African Development Bank

The orange line with squares shows estimated disbursements based on the Compendium of Statistics from 1990 through 2001 and actual disbursements received from 2001 onwards.

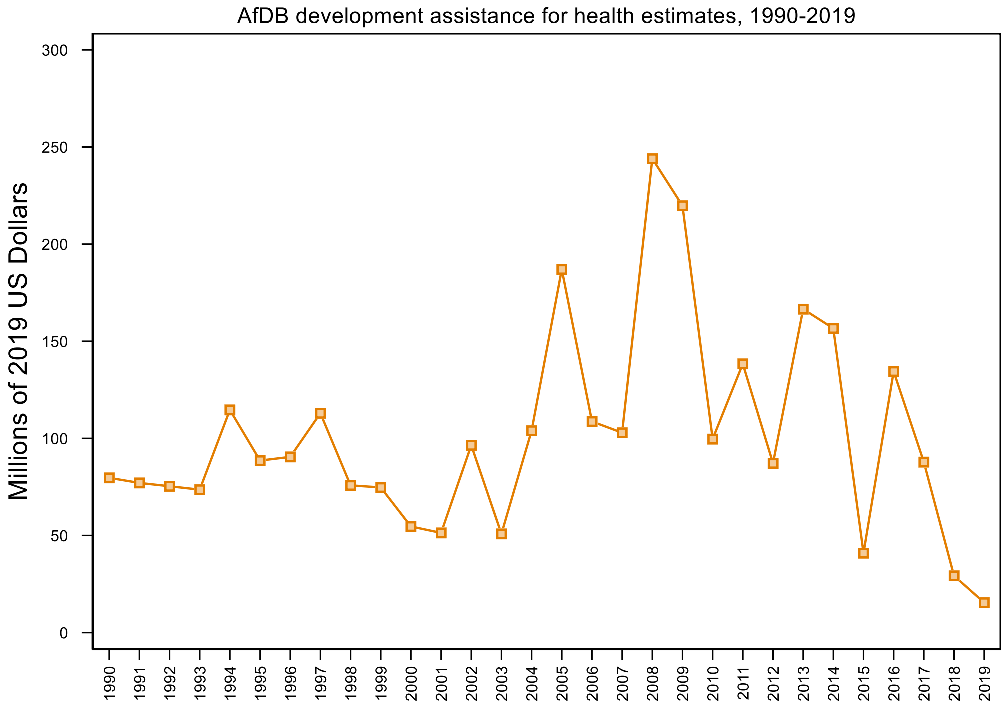

Source: IHME DAH Database (2019) and African Development Bank Compendium of Statistics.

Figure S3.8 Disbursements by Asian Development Bank

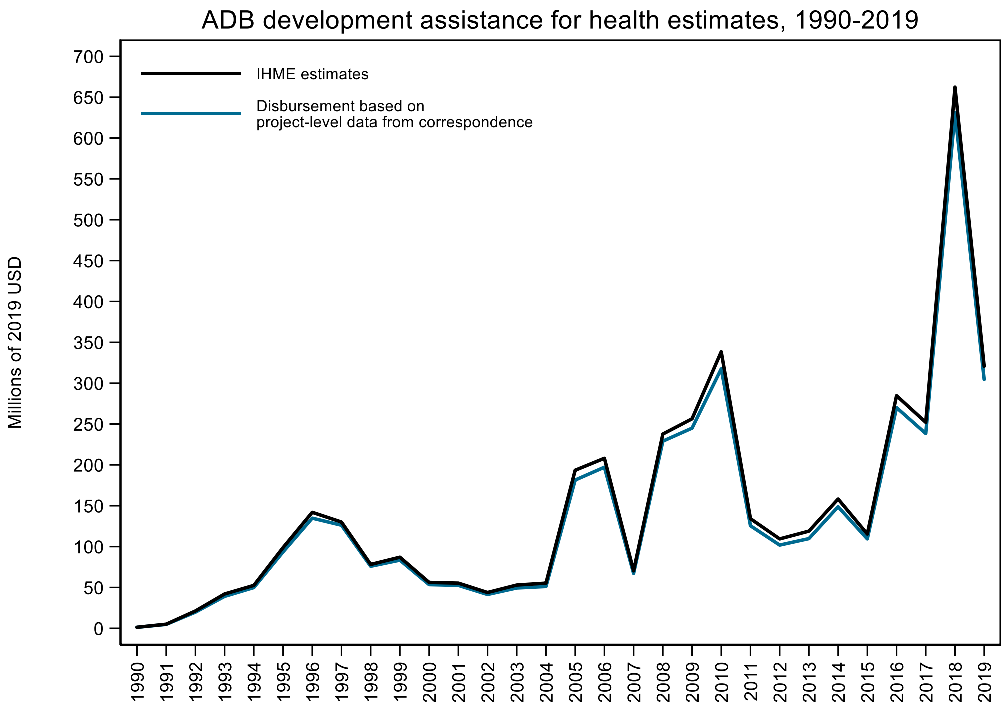

Source: IHME DAH Database (2019)

Figure S3.9 Commitments and disbursements by Inter-American Development Bank

The dashed green line shows commitments from the Inter-American Development Bank's (IDB) online projects database. The orange line shows smoothed disbursements from the online projects database and from correspondence for 2019.

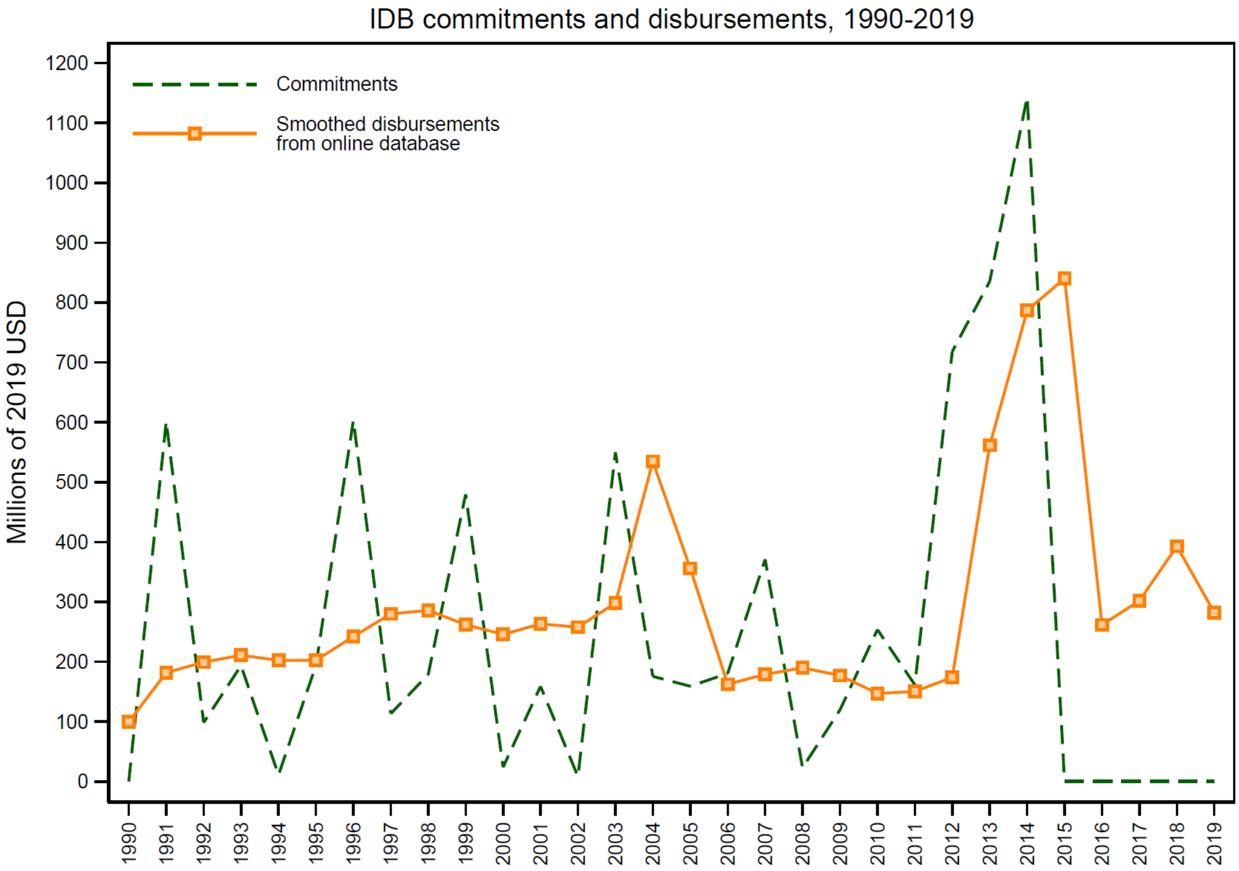

Source: IHME DAH Database (2019) and correspondence

S3.4 Tracking contributions from GFATM and GAVI

The Global Fund to Fight AIDS, Tuberculosis and Malaria

The grants database made available online by the Global Fund to Fight AIDS, Tuberculosis and Malaria (GFATM) provides grant-level commitments and annual disbursements from its inception in 2002 to the present year.[22] In addition, sources of funding were compiled from the GFATM contributions dataset and annual reports, all downloaded from the GFATM website.[23,24] Regional grants were split evenly between all countries identified in the regional grant documents found on the GFATM website. Figure S3.10 shows GFATM's annual contributions received from public and private sources. Figure S3.10 shows GFATM's annual commitments and disbursements from its project database from 2002 through 2019.

Figure S3.10 Contributions received by the Global Fund to Fight AIDS, Tuberculosis and Malaria

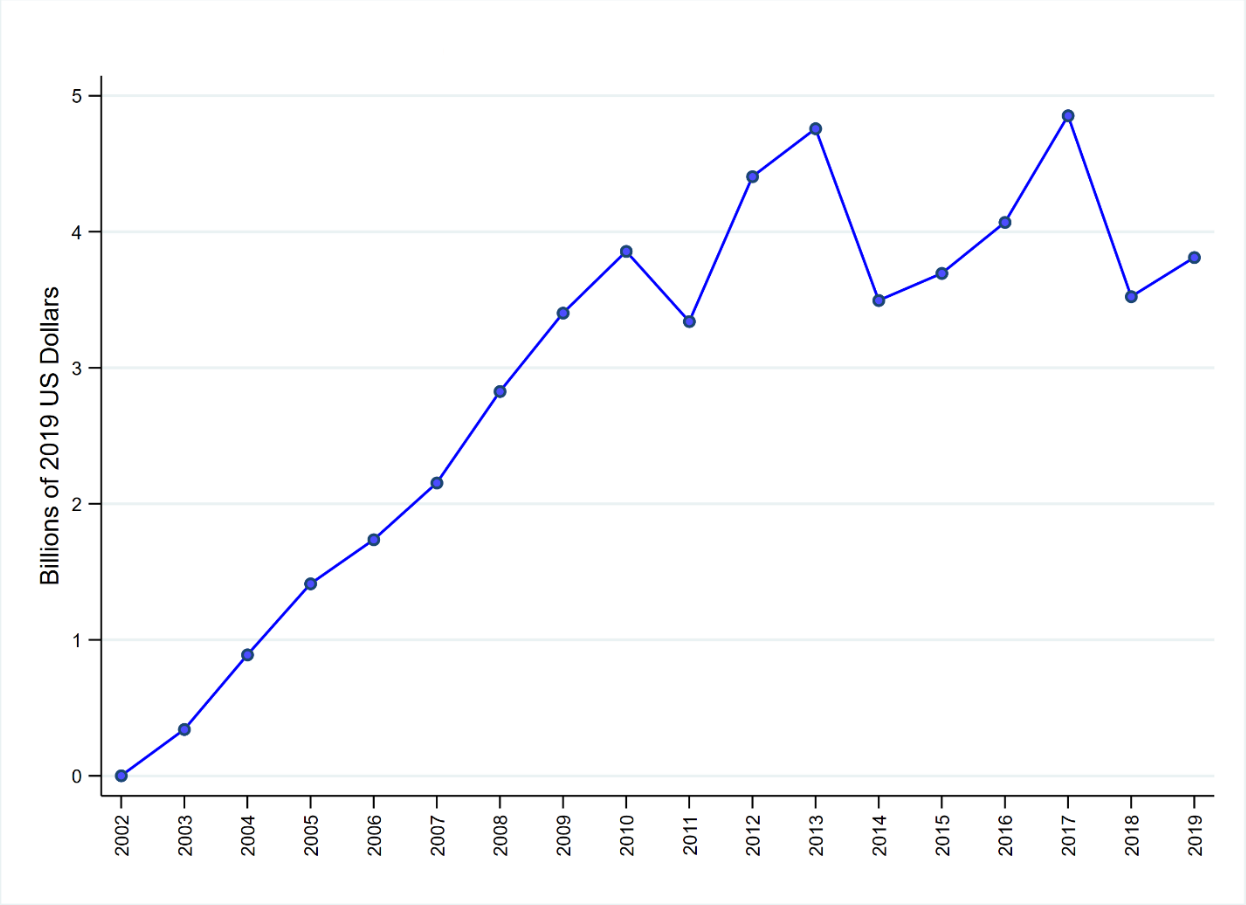

Source: GFATM pledges and contributions 2019

Figure S3.11 The Global Fund to Fight AIDS, Tuberculosis and Malaria's commitments and disbursements

The dashed green line shows commitments from the Global Fund to Fight AIDS, Tuberculosis and Malaria's (GFATM) online grants database. The orange line shows disbursements from the online grants database.

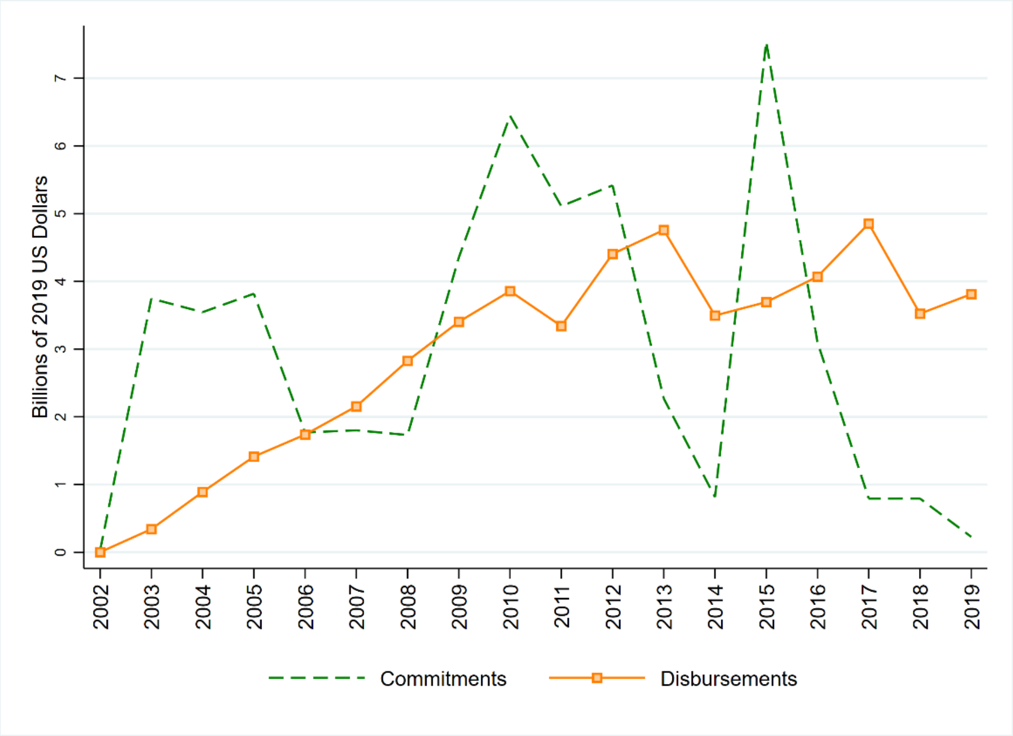

Source: IHME DAH Database (2019)

S3.4.1 Gavi, the Vaccine Alliance

Gavi provided publicly available project-level data on commitments, disbursements, and investment cases from 2000 through the present.[18,21] Gavi's annual DAH was defined as the sum of (1) project-level disbursements by year paid; (2) investment cases (one-time investments in disease prevention and control); and (3) administrative and work plan costs. Data from Gavi's online databases include expenditure for (1) and (2), but not (3). However, project level data from the CRS for 2007-2012 did include administrative and work plan costs, so disbursements data from the online database were adjusted to match the CRS in those years. The average fraction of administrative and work plan costs was added to total disbursements in 2000-2006 and 2013-2015, the years in which the CRS did not include these data. Contributions data from Gavi's website as well as annual reports from the International Finance Facility for Immunisation (IFFIm) and Advance Market Commitment for Pneumococcal Vaccines were used to determine Gavi's annual income.[19,20,84]

All of the data sources used for Gavi estimates were complete through 2018. Donor contributions received and outstanding pledges data were available on Gavi's website. The unadjusted total pledges were used as total disbursements for 2019. Gavi disbursements were assigned to health focus areas including child and newborn vaccines, HSS, and non-communicable disease as documented in Table S3.7 above. Of note, we reclassified all GAVI health system strengthening projects as maternal, newborn, child health specific health system strengthening disbursements

Figure S3.12 Gavi's income and disbursements

The dashed green line shows commitments from Gavi's online database. The dashed orange line shows the disbursements from Gavi's online database, which are the sum of project-level disbursements and investment cases. These data are adjusted using Gavi expenditure data reported to the Creditor Reporting System (CRS) to add administrative and work plan costs to the total. Adjusted disbursements are shown by the solid orange line.

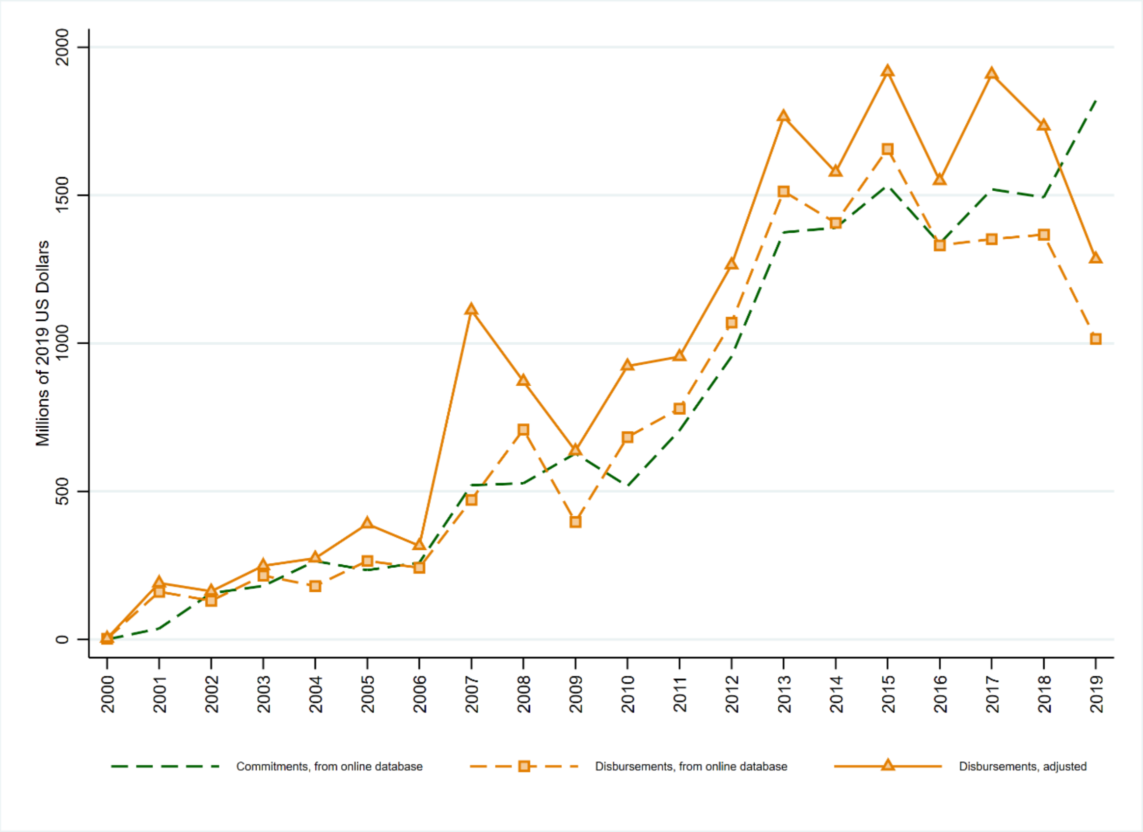

Source: IHME DAH Database (2019)

S3.4.2 Coalition for Epidemic Preparedness and Innovation

CEPI provided a complete dataset of all disbursed funding by disease from 2017-2019 and all income received from donors from 2018-2019. Funding data was provided per target vaccine and were classified between the other infectious diseases health focus area and the health systems strengthening other infectious diseases health focus area. Since CEPI donor data spans from 2017-2019, the proportions by donor for 2017-2018 was applied to total disbursements in 2018.

S3.5 Tracking expenditure by United Nations Agencies active in the health domain

Data on income and expenditures were collected for six UN agencies: WHO, UNICEF, UNFPA, UNAIDS, UNITAID and PAHO. The data sources and calculations for each are described in detail below. Similar to the bilateral channels, we extracted budget data for the UN agencies to predict DAH for years for which we did not have health expenditure data. Model choices and budget measures for UN agencies are presented in Table S3.3.

S3.5.1 World Health Organization

Data on WHO's budgetary and extrabudgetary income and expenditure were compiled from annual reports and audited financial statements released by WHO.[85] Income data were extracted from WHO's assessed and voluntary contributions, while expenditure data were extracted from both budgetary and extrabudgetary spending reports. For financial statements representing activities over a two-year period, both income and expenditure data were divided by two, in order to approximate yearly amounts, and dollars were deflated using the US GDP deflator specific to the reporting year. Expenditures from trust funds, regional offices tracked separately, and associated entities not part of WHO's program of activities, such as UNAIDS and GFATM trust funds were excluded. Expenditures from supply services funds were also excluded, as these expenditures pertain to services provided by WHO but paid for by recipient countries. Additionally, WHO projects tracked as paid Ebola expenditure were extracted from the UNOCHA Financial Tracking System database and included as WHO health expenditure.

Disbursement data were not available for WHO in 2019. Much like the bilateral agencies, the ratio of DAH to the total program budget was estimated for 1990-2018 and then predicted for 2019 using the single-year average of previous year. The predicted ratio was then multiplied by the observed program budget for 2019 to get the estimates of DAH.

S3.5.2 United Nations Population Fund

Data on income and expenditure were extracted for UNFPA from its audited financial statements.[72] As the 1990-2005 statements represent activities over a two-year period, income and expenditure data were divided by two in order to approximate yearly amounts.

Income and expenditures associated with procurement and cost-sharing activities were excluded from estimates of health assistance because UNFPA uses cost-sharing accounts when a donor contributes to UNFPA for a project to be conducted in the donor's own country. Since this money can be considered domestic spending that goes through UNFPA before being returned to the country in the form of a UNFPA program, it is not included in calculations of total DAH. UNFPA's additional expenditures for these projects come from trust funds or regular resources and are therefore, captured in our estimates. To estimate disbursements by health focus areas, UNFPA's total health expenditure was multiplied by the proportion of funding reported for each program area from annual reports from 1997 through 2013, from the UNFPA transparency portal for 2014 through 2018, and from the 2018 Statistical and Financial Review for 2019. Maternal and child health spending classified as "other" was split equally between the maternal and child health program areas. Health spending in 2018 that was classified as towards "Integrated sexual and reproductive health services" was split equally between maternal health, family planning, other reproductive health and other HIV/AIDS program areas. Additionally, UNFPA projects tracked as paid Ebola expenditure were extracted from the UNOCHA Financial Tracking System database and added to UNFPA health expenditure.

The disbursement data for UNFPA were available through 2018. For year 2019, the ratio of DAH and income was estimated to be the same as that for 2018. This approach was used rather than implementing a three-year weighted average of ratios because the change in funding structure was assumed to persist for the near future. The predicted ratio was then multiplied by observed income to estimate DAH for 2019.

S3.5.3 United Nations Children's Fund

Data on income and expenditure for UNICEF were extracted from its audited financial statements.[69] As these statements represent activities over a two-year period from 1990-2011, income and expenditure data were divided by two in order to approximate yearly amounts. The audited financial statements from 2012 onwards are produced on an annual basis.

Since UNICEF's activities are not limited to the health sector, the fraction of UNICEF's expenditure that was for health was estimated using either financial data from correspondence (2001-2013 observed data was used to estimate 1990 through 2000 expenditure) or a combination of annual reports and annual results reports from 2014

through 2018. The annual results reports provide the proportion of funding for each program area and the average of 2014 through 2016 proportions was used to estimate the spending proportion for the years 1990 through 2013. In the annual results report, HIV/AIDS funding and Nutrition funding (as of 2014) was reported separately from health funding so the percentages spent on each health program were proportioned based on total spending for Health. Furthermore, UNICEF projects tracked as paid Ebola expenditure were extracted from the UNOCHA Financial Tracking System and added to estimates for UNICEF's health expenditure.

The product of observed program budget and the three-year weighted average of the DAH to budget was used to predict DAH in 2019.

S3.5.4 Joint United Nations Programme on HIV/AIDS

UNAIDS income and expenditure data for both its core and noncore budgets were extracted from its audited financial statements.[67] As financial data are provided on a biennial basis in all years prior to 2012, those values were divided by two to obtain yearly amounts. Dollars were deflated using the US GDP deflator specific to the reporting year.

For UNAIDS, budget measures were available only for a subset of reported total disbursements. UNAIDS reported total expenditure, which combined Unified Budget and Workplan (UBW) and non-UWB components, but only UBW budget data were available.[68] To predict DAH for UNAIDS in 2019, disbursements for that year were estimated by multiplying the observed UBW budget by the three-year weighted average of the ratio of DAH to the UWB budget. UNAIDS disbursements were assigned to HIV/AIDS and TB program areas as documented in Table S3.7 above.

S3.5.5 UNITAID

Data on project level disbursement was obtained through correspondence with UNITAID. Income data was extracted from the annual financial statements downloaded from UNITAID's website. The project level data provided covered project disbursements from 2007 through 2018 and project budget commitments was provided for 2019.

S3.5.6 Pan American Health Organization

The Pan American Regional Office for WHO, or PAHO, reports its income and expenditure in its biennial financial report.[10,86] The funds transferred through the "Rotating Fund" were excluded because developing countries fund this procurement of health commodities which are then used within that funding country , and it therefore does not fit the definition of DAH.

As the financial data are provided on a biennial basis (with the exception of 2010 through 2016, where single-year financial reports were available), the quantities were divided by two to obtain yearly amounts. Dollars were deflated using the US GDP deflator specific to the reporting year.

Correspondence with PAHO revealed that data from the financial statements include both Program and nonProgram funds. The latter include funds that countries provide PAHO, so that PAHO can reinvest these funds into the countries' national health systems. These funds should not be included as development assistance for health, and PAHO provided corrected disbursement numbers for 2008 to 2013. The corresponding disbursement numbers for 2014 and 2015 were identified in the PAHO End-of-Biennium Assessment 2014-2015. These funds were provided as biennial disbursements, so they were divided by two to obtain yearly disbursements. The ratio of Program disbursements numbers provided by PAHO and the sum of Program and non-Program funds collected from financial statements was taken for the years 2008 to 2015. The average ratio was calculated, and this ratio was multiplied

through disbursement numbers collected from financial statements from earlier years. In this way, Program and non-Program funds collected from audited statements from earlier years were adjusted to estimate DAH.

For PAHO, disbursement data were not available for 2019. PAHO provided budget information along with disbursements for 2008 to 2018. The average ratio between spending and budget was calculated over the years 2008 to 2018, and this ratio was used to estimate 2019 disbursements.

S3.6 Tracking development assistance for health from private foundations

Previous studies on foundations outside the US have documented the severe paucity of reliable time series data and lack of comparability across countries.[87] Hence, this research focused efforts on tracking only US foundations.

S3.6.1 US Foundations

The Foundation Center maintains a database of all grants of $10,000 or more awarded by over 1,000 US foundations. The Foundation Center has coded each grant by sector and international focus and therefore is able to identify global health grants. IHME purchased a customized dataset with cross-border health grants and health grants to US-based international programs from 1992 to 2017 from the Foundation Center.[32] Grants from BMGF, which were tracked separately, were excluded. Additionally, grants to channels that this research already tracks were excluded.

The Foundation Center adopted a new classification methodology as of FGH 2016. The Foundation Center was able to provide historic data based on the new classification system from 2002 to 2012. In order to obtain the series from 1992 to 2001, we multiplied a weighted fraction calculated based on both old and new classification data values from 2002 through 2004 by the old data series (1992 to 2001) we had previously obtained.

$$
\begin{aligned}
(Weighted\ fraction) &= \left(\frac{1}{2}\right)\left(DAH_{new\ classification}\right)/\left(DAH_{old\ classification}\right)2002 \\
&+ \left(\frac{1}{3}\right)\left(DAH_{new\ classification}\right)/\left(DAH_{old\ classification}\right)2003 \\
&+ \left(\frac{1}{6}\right)\left(DAH_{new\ classification}\right)/\left(DAH_{old\ classification}\right)2004
\end{aligned}
$$

$$(DAH\ Estimate_t) = (Weighted\ fraction)(DAH\ Observed_t)$$

where DAH Observed is the old data values for the series 1992 through 2001

To estimate total health grants in 1990-1991 and 2017-2019, natural log of US foundation DAH was regressed on the lagged natural log of US GDP per capita and year using ordinary least squares estimation. The missing years of data were predicted based on estimated regression coefficients from the equation. Exponents of the predicted values were used as final estimates

$$(ln\ Foundation_t) = \alpha + 1.\beta_1(\ln US\ GDP\ per\ capita_t) + \beta_2(year_t) + \varepsilon$$

Details on how we estimated the cost of providing technical assistance and program support for these US foundations are highlighted below in the section titled calculating the technical assistance and program support component of development assistance for health from loan-and grant-making channels of assistance.

S3.6.2 Bill & Melinda Gates Foundation

BMGF has been the single largest grant-making institution in the health domain since 2000; hence, additional research was undertaken to accurately capture its annual disbursements. BMGF's IRS 990PF filings for years 1999-2008, which report all global health grants disbursed per year, were downloaded from the BMGF website. Additionally, disbursement data for years 2009-2018 were collected from the BMGF online grants database, the OECD CRS and personal correspondence. The OECD CRS data was used to identify NGOs that are double-counted from other data sources.

An ordinary least squares linear regression model was used to predict the disbursement for BMGF for 2019. Since there is a strong correlation between market trends and BMGF annual disbursements, market data including lagged US GDP, lagged yearly average of Berkshire stock returns, lagged yearly average of the Russell Index, and lagged total assets of the BMGF Trust were utilized to predict the total disbursement for year 2019.

$$
\begin{aligned}
(BMGF\ total\ disbursement_t) \\
&= \alpha + \beta_1\left(US\ GDP\ per\ capita_{t-2}\right) + \beta_2\left(Berkshire\ stock\ returns_{t-2}\right) \\
&+ \beta_3\left(Russell\ Index_{t-2}\right) + \beta_4\left(BMGF\ total\ asset_{t-2}\right) + \varepsilon
\end{aligned}
$$

BMGF's predicted DAH was adjusted to account for in-kind DAH and double-counting. The difference between

BMGF's final DAH and DAH without in-kind added and double-counting removed from 2003-2018 was regressed using ordinary least squares on DAH without in-kind added and double-counting removed and year. The predicted difference was then subtracted from the predicted DAH from the previous regression for 2019.

S3.6.3 European Economic Area

DAH estimates for the European Economic Area were based on two downloadable project datasets available online. IHME DAH data for 2014 to 2018 was obtained from projects in the 2009-2014 grant period dataset with programme area "public health initiatives", and 2007-2012 data was obtained from projects in the 2004-2009 grant period dataset with sector "health and childcare". Childcare projects not related to health were identified and excluded based on manual screening and keyword search for the following terms: "NURSERY" "ORPHANAGE" "RECREATION" "FOSTER" " CARE HOME " " CHILDREN S HOME " " CHILD DEVELOPMENT CENTRE " " YOUTH CENTER" " YOUTH CAMPS " " RESIDENTIAL CENTRE" " RESIDENTIAL CARE FACILITIES " "CHILDCARE" " INFORMAL EDUCATION FOR CHILDREN " " PREVENT INSTITUTIONALISATION " " SPORTS FIELD" " SPORT FIELD" " SPORTS FACILIT" " SPORTS ACTIVIT" " SPORTS INFRASTRUCTURE" " REINTEGRATION OF JUVENILE OFFENDERS "

We determined that no health projects to low- and middle-income countries were implemented by the EEA prior to the 2004-2009 grant period, based on the report on the Financial Instrument 1999-2003 and the Financial Mechanism 1994-1998.[88,89] Project summary variables were obtained through correspondence, and were used together with project titles to assign DAH to health focus areas using a keyword search. To assign annual funding for projects that were implemented across multiple years, project grants were split based on the average daily spending across the duration of the project and the proportion of the year that the project was in implementation. The relative contributions of donors to the EEA grants (Norway, Iceland, and Liechtenstein) were obtained as constant ratios based on the 2016-2017 Annual Report.[90]

An open call for projects in the EEA's third grant period occurred in 2014, the first projects of which began disbursing in 2019 and are set to conclude in 2021. Data for the 2019 grants came from personal correspondence.

S3.7 Tracking non-governmental organisations

Currently, there are no centralized, easily accessible databases for tracking program expenses of the thousands of NGOs based in high-income countries that are active in providing development assistance and humanitarian relief worldwide. This study relied on CRS data and the only comprehensive data source identified for a large subset of these NGOs, namely the United States Agency for International Development's Report of Voluntary Agencies (USAID's VolAg report).[27] The report, which includes both US-based and international NGOs that received funding from the US government, provides data from 1990 to 2014 on domestic and overseas expenditures for these NGOs as well as their revenue from US and other public sources, private contributions, and in-kind. Total revenue and expenditure data obtained from the NGOs' IRS tax forms, accessed through the GuideStar online database, were also used in tracking NGOs incorporated in the US.[26] The GuideStar Research Fundamentals Plus dataset was used to obtain information for US-based NGOs in fiscal years 2015 and 2016, including domestic, overseas, and total expenses, and revenues from all public and private sources.

First, in order to track disbursements from OECD donor countries to NGOs, we utilized channel codes present in the CRS database. The code 21000 identified international NGOs and the code 22000 identified donor-country-based NGOs. In order to remove double-counting, we conducted a keyword search on channels where the donor country was the United States to exclude NGOs present in the USAID VolAg report and the GuideStar dataset. Allocation of funding to health focus areas for NGOs tracked through the CRS was assigned as described in the section "DISAGGREGATING BY HEALTH FOCUS AREA", based on a keyword search of five descriptive variables in the CRS: project title, short description, long description, channel name, and channel reported name. For NGOs tracked in the USAID VolAg report and the GuideStar dataset, allocation of funding to health focus areas was assigned as described in the section "DISAGGREGATING BY HEALTH FOCUS AREA", based on a keyword search of the NGO's description given in the VolAg report. Due to CRS projects containing more complete descriptions than the VolAg report descriptions, the health focus area allocations from CRS were prioritized over those from the VolAg report for US NGO agencies that appeared in both sources.

In order to use the USAID VolAg data and the GuideStar dataset, several challenges were overcome. We outline these challenges here and discuss below the methods employed to estimate a consistent series of DAH channeled through NGOs despite these challenges. First, with the exception of BMGF, it was impossible to track the amount of funding from US foundations routed through US NGOs, which may have led to double-counting in estimates of total health assistance. The second challenge relates to the incompleteness of the universe of NGOs captured through the VolAg report and the GuideStar Research Fundamentals dataset. The VolAg report provides data on NGOs that received funding from the US government. While this covers many of the largest NGOs, it is not a comprehensive list. A related problem is that the VolAg report and GuideStar dataset only include NGOs that received funds in a given year. While many of the largest NGOs are consistently funded by the US government and are therefore in the dataset every year, not all NGOs are reported across all years. Third, health sector-specific expenditure is not reported in the VolAg or systematically reported in IRS tax forms. The VolAg does report overseas expenditure but does not disaggregate this expenditure by sector. Fourth, complete data are lacking in several time periods. The 2016 VolAg provided data through 2014. For NGOs incorporated in the US, IRS tax forms were obtained. Furthermore, prior to 1998 the VolAg report did not include international NGOs. Attempts were made to compile other data on the health expenditures of the top international NGOs, in terms of overseas expenditure, by searching other websites for financial documents and contacting these organizations directly. Getting reliable time series data before 2000 proved to be extremely difficult for even this small sample of international NGOs.

Estimates of the share of overseas expenditure spent on health-related projects drew upon a sample of NGOs for which such data were available. Collecting financial data on health expenditures for each NGO would have been prohibitively time-consuming. Therefore, a sample of NGOs was drawn from the list for each year; the sample included the top 30 NGOs in terms of overseas expenditure and 20 randomly selected US-based NGOs from the remaining pool, with the probability of being selected set proportional to overseas expenditure. Next, health expenditure data were collected for each NGO in this sample by seeking out annual reports, audited financial statements, 990 tax forms, and data from NGO websites. Health expenditure was carefully reviewed to ensure that

expenditures on food aid, food security, disaster relief, and water and sanitation projects were not included. Table S3.8 summarizes the number of NGOs included each year in the USAID report, the number of NGOs in the sample by year, and the number of NGOs for which health expenditure data were successfully compiled.

Table S3.8 Summary of US non-governmental organizations in the study

Year	Number of US NGOs in VolAG report	Number of international NGOs in VolAG report	Number of US NGOs in IHME sample	Number of US NGOs from sample for which data on health expenditure were found
**1990**	267	-	16	9
**1991**	334	-	19	14
**1992**	385	-	18	15
**1993**	411	-	17	12
**1994**	424	-	17	10
**1995**	416	-	16	12
**1996**	423	-	21	14
**1997**	425	-	23	18
**1998**	435	42	24	22
**1999**	438	-	33	28
**2000**	433	50	34	28
**2001**	442	51	33	26
**2002**	486	58	33	27
**2003**	507	54	42	32
**2004**	508	55	47	33
**2005**	494	59	45	36
**2006**	536	67	50	38
**2007**	556	68	50	40
**2008**	565	78	58	48
**2009**	580	90	57	45
**2010**	579	94	69	57
**2011**	595	112	73	63
**2012**	579	94	69	60
**2013**	519	113	69	52
**2014**	485	106	73	54
**2015**	-	-	69	53
**2016**	-	-	136	111

A random effects regression model was fit to predict health expenditure as a fraction of total expenditure using the data for the sampled NGOs. A random effects model was chosen because the sample included observations for several NGOs for multiple years. A random effects model allows for the effect of each type of NGO to be captured distinctly. This model was used to predict the fraction of expenditure spent on health for the remaining NGOs. To ensure that the predicted health fractions were bounded between zero and one, the regression utilized the logit-transformed health fraction as the dependent variable. Since several NGOs in the sample were observed for multiple years, the regression included a random effect that varied by NGO. Five of the nine variables used to predict the

health fraction were drawn from the VolAg reports and the GuideStar dataset. They were (1) fraction of revenue from in-kind donations, (2) fraction of revenue from the US government, (3) fraction of revenue from private financial contributions, (4) overseas expenditure as a fraction of total expenditure, and (5) calendar year. The remaining four variables used to predict the health fraction were binary indicators that were constructed based on keyword searches on the NGO name and NGO description found in the VolAg. For both the NGO name and description, a keyword search was conducted to indicate whether the name or description was sufficiently health-related. Another keyword search was conducted independently on the NGO names and descriptions for keywords that indicated if the NGOs might focus on something other than health. These four indicators proved excellent predictors of health fractions.

$$\begin{aligned} logit(\,NGO - specific\ DAH_{it}) &= \alpha + \ \beta_1(Inkind\ contributions\ fraction_{it}) \\ &+ \ \beta_2(US\ government\ contributions\ fraction\ _{it}) \\ &+ \beta_3(Private\ financial\ contributions\ fractions_{it}) \\ &+ \beta_4(Overseas\ expenditure\ as\ a\ fraction\ of\ total\ expenditure_{it}) \\ &+ \beta_5(Health - related\ name_{it}) + \beta_6(Non - health - related\ name_{it}) \\ &+ \beta_7(Health - related\ description_{it}) + \beta_8(Non - health - related\ desciption_{it}) + U_i + \ \varepsilon \end{aligned}$$

Overseas health expenditure was calculated for individual NGOs in each year by multiplying the estimated health fraction and total overseas expenditure. For the NGOs that were sampled, the observed health fraction acquired through data collection was used. For the unsampled NGOs, the fitted fraction from the previously described random effects regression was used. Total overseas expenditure, reported in the VolAg, was not available for 2015-2019. For US-based NGOs, the 2016 NGO overseas fraction was calculated by regressing the logit transformed observed overseas fraction on a linear time trend using ordinary least squares, for each NGO independently. For these cases, the overseas health fraction was calculated as the product of estimated overseas fraction, estimated health fraction, and total expenditure found in the IRS 990 forms.

$$logit(Observed\ overseas\ health\ expenditure_i) = \alpha + \ \beta_i(year_t) + U_i + \ \varepsilon$$

At this point three reasons remained why the overseas health expenditure for some NGOs remained unknown. First, if an observation was non-US-based, then IRS tax forms were not available and total overseas expenditure could not be calculated. Second, for 2017 to 2019, no data were available. Finally, if an NGO was reported in the VolAg in multiple years but not for an intermittent year, no NGO-specific data were available for the gap year. This would be the case if an NGO received support from the US government one year and then again in a nonconsecutive year. For all three of these scenarios, a panel-based hierarchical linear regression model was used to fill in the overseas health expenditure gaps. Total overseas health expenditure (measured at the NGO-year level) was regressed on US GDP per capita and US bilateral DAH disbursed. Because the US government funds many of these NGOs, US bilateral DAH was an excellent predictor of NGO DAH. A flexible model was employed to allow both the GDP and US government DAH coefficients to vary randomly across NGOs, such that each NGO employed a unique (but not independent) relationship between overseas health expenditure, GDP, and US government DAH. A random intercept was also included to capture the significant unobserved heterogeneity present in our set of NGOs. Once fit, this model was used to predict overseas health expenditure for all remaining gaps.

$$(\,NGO\ DAH_{it}) = \alpha + \ \beta_{1i}(US\ GDP\ per\ capita_t) + \ \beta_{2i}(US\ bilateral\ DAH\ per\ capita_t) + U_i + \ \varepsilon$$

Expenditures financed from each revenue source were then calculated by multiplying overseas health expenditure by NGO-specific revenue fractions. Expenditures from in-kind sources were deflated by a constant fraction. This was determined by comparing the federal upper limit and average wholesale price valuations of drugs on the WHO's Model List of Essential Medicines from the RED BOOK Expanded Database.[28,29] Figure S3.13 and Figure S3.14 show the income and estimated overseas health expenditure, respectively, of the NGOs in the universe of US- and non-US-based NGOs that were tracked in this study from 1990 to 2016 in constant 2019 US dollars.

Figure S3.13 Total revenue received by non-governmental organizations

The orange line shows total revenue for all sources, both public and private, received by NGOs. The green line shows estimates of private financial contributions to NGOs, while the blue line shows private in-kind donations to NGOs.

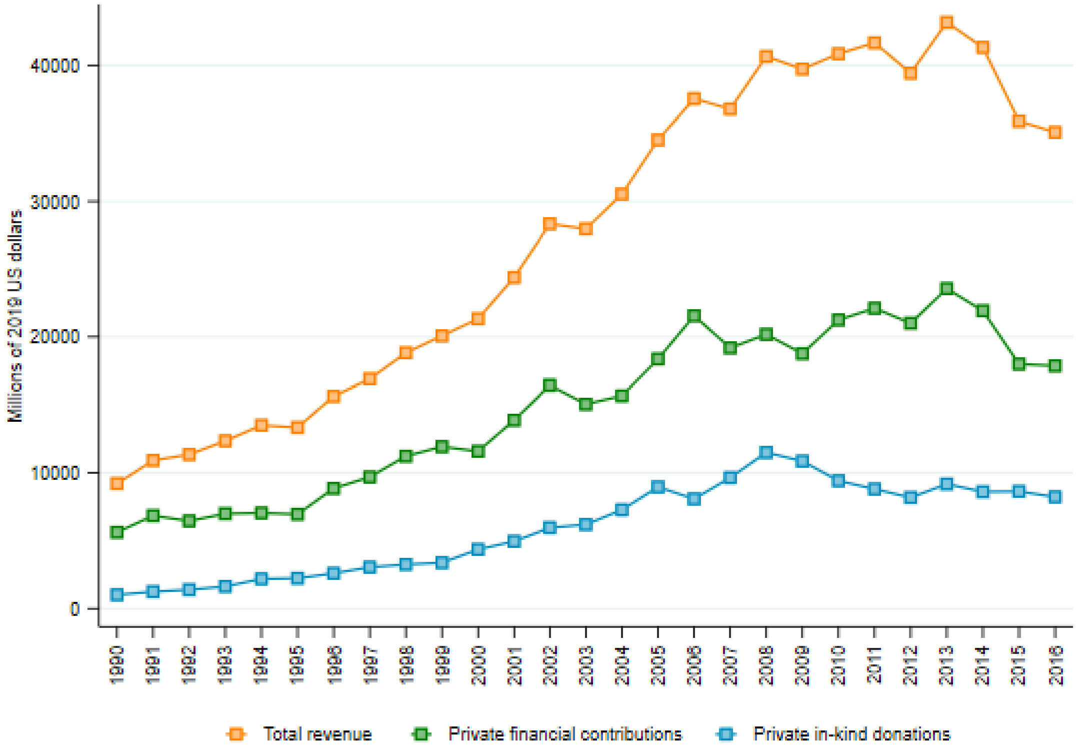

Source: IHME DAH Database (2019)

Figure S3.14 Expenditure by non-governmental organizations

The orange line illustrates total overseas expenditure by NGOs, regardless of sector. The green line shows overseas expenditure by NGOs to health-specific recipients, or DAH.

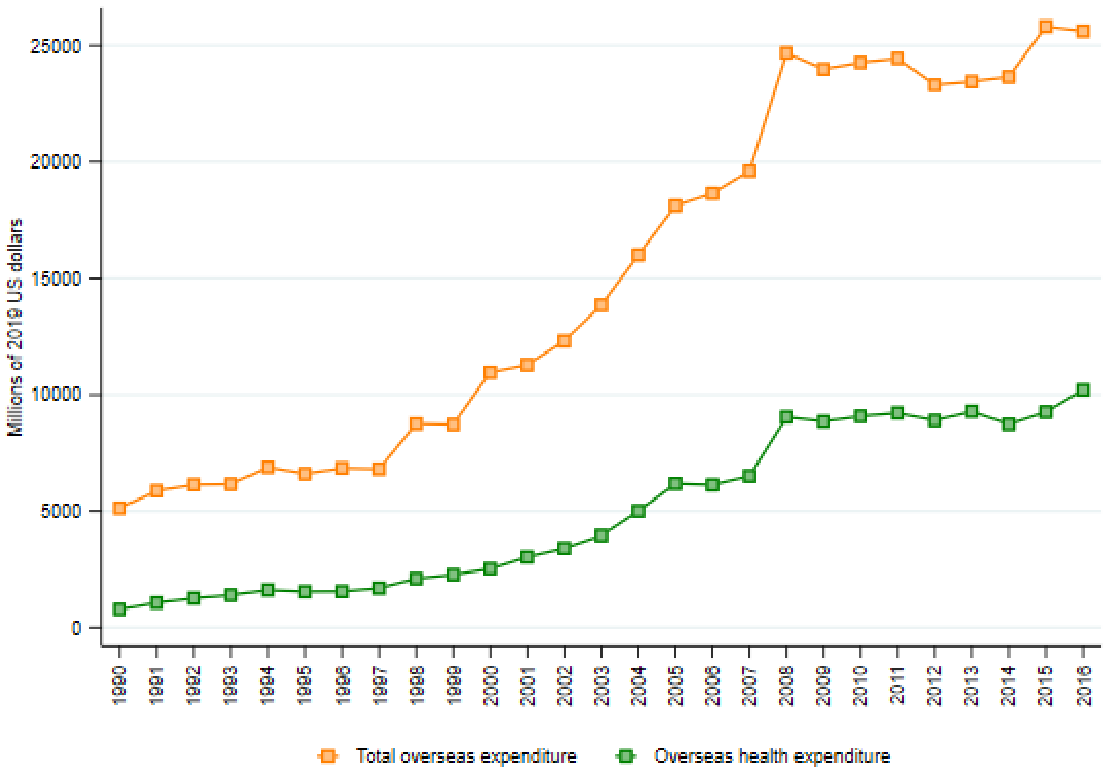

Source: IHME DAH Database (2019)

S3.8 Tracking development assistance for health from China

The estimates of DAH contributions from China is based on data from diverse sources including government reports and international development agencies databases. We generate estimates for bilateral and multilateral contributions from China separately and aggregate to obtain the total DAH contributions from China. We use project descriptions and reports where available to determine health focus areas of the contributions. For years with limited data, we use various methods inclusive of interpolation and modeling to generate estimates.

This year we updated our estimates of DAH contributions from China by adding the China International Development Cooperation Agency (CIDCA), a new ministry of the Chinese government founded in 2018. This ministry has been tasked with taking over the international aid duties of the Chinese Ministry of Commerce (MOFCOM). To estimate contributions from this agency, we collected data on pledges and contributions from their annual budget and department final account, and conducted a literature review of their press releases to calculate a scaling proportion for the health related projects disbursed. Based on the literature review, we identified three relevant health focus areas (other health systems strengthening/sector wide approaches, other infectious diseases – Ebola, and malaria treatment) disbursed to over the two years.

Detailed description of the methods for generating the estimates are reported in Micah AE, Zhao Y, Chen CS, et al[97].

S3.9 Calculating the technical assistance and program support component of development assistance for health from loan-and grant-making channels of assistance

The following methods were used to estimate the costs incurred by loan- and grant-making institutions for administering and supporting health sector loans and grants, which includes costs related to staffing and program management.

Data on the total administrative costs were compiled for institutions in our universe for which these data were readily available: IDA, IBRD, ADB, AfDB, IDB, BMGF, the Wellcome Trust, US Foundations, CEPI, GFATM, Gavi, UNICEF, UNFPA, WHO, UNITAID, PAHO, UNAIDS, USAID, and the UK Department for International Development (DFID), Japan International Cooperation Agency (JICA), Norwegian Aid Agency (NORAD) and the Swedish International Development Agency (SIDA). The sources of data for the institutions in this sample are summarized in Table S3.9. The ratio of total administrative costs to total grants and loans was calculated for each source by year. It was assumed that the percentage of operating and administrative costs devoted to health would be equal to the percentage of grants and loans that were for health. In other words, if 20% of a foundation's grants were for health, the model assumed that 20% of administrative costs of the foundation were spent on facilitating these health grants. Given this assumption, the ratios of the observed administrative costs to grants/loans were used to estimate the in-kind contribution made by each of these organizations toward maintaining their health grants and loans. For the institutions not in this sample, the ratio from the institution most similar to it was used to arrive at an estimate of in-kind contributions. For example, for US foundations we selected the top 10 and bottom 10 US foundations based on total disbursements across the entire time period. For each foundation, we calculated the ratio of the "cash basis" column of total operating and administrative expenses to grants paid. We then used the average of these top 10 and bottom 10 foundations ratio as the inkind ratio across time. Total in-kind contributions from all grant- and loan-making global health institutions are shown in Figure S3.14.

Table S3.9 Summary of data sources for calculating in-kind contributions

Organization	Source	Notes
**BMGF**	990 tax returns (1999-2006)<br>BMGF Trust financial statements (2007-2018)	Used "cash basis" column to calculate ratio of total operating and administrative expenses to grants paid.<br>Used "grants expenditure" statement to calculate ratio of administrative expenditure to grants/program expenditure.
**US Foundations**	990 tax returns	Calculated the average across time of the ratio of the "cash basis" column of total operating and administrative expenses to grants paid.
**Wellcome Trust**	Annual report	Calculated ratio of support costs to grants awarded by the trust
**CEPI**	Board of directors report, 2016-2018	Calculated ratio of operating expenses to total expenditure.
**GFATM**	Annual report financial statements	Calculated ratio of operating expenses to grants disbursed.

**Gavi**	Annual report financial statements	Calculated ratio of management, general, and fundraising expenses to program expenses.
**UNICEF**	Annual report financial statements	Calculated ratio of programme support to total expenditure (1990-2011)<br>Calculated ratio of institutional budget to total programme and emergency budgets (2012-2018)
**UNFPA**	Annual financial review	Calculated ratio of institutional budget to total expenses
**WHO**	Financial report and annual financial statements	Calculated ratio of general operating expenses to total expenses
**UNITAID**	Financial statement	Calculated ratio of operating expenses to program expenses
**USAID**	US government budget database	Used outlays spreadsheet to calculate ratio of total outlays for USAID operating account to sum of outlays for bilateral accounts.
**DFID**	Annual report expense summary	Calculated ratio of DFID’s administration expenses to DFID’s bilateral program expenses from 2002 onward.
**JICA**	Statement of income in annual report	Calculated ratio of general administrative expenses to operating expenses less depreciation
**SIDA and other Swedish agencies disbursing aid**	Swedish OpenAid website[91]	Calculated ratio of administrative costs to the sum of all program expenses
**NORAD and other Norwegian agencies disbursing aid**	Norwegian Aid statistics website[92]	Calculated ratio of general administrative expenses to operating expenses less depreciation
**IDA**	World Bank audited financial statements	Calculated ratio of management fee charged by IBRD to development credit disbursements.
**IBRD**	World Bank audited financial statements	Calculated ratio of administrative expenses to loan disbursements.
**AfDB**	Income statements in annual reports	Calculated ratio of administrative expenses to the sum of loan and grant disbursements
**ADB**	Annual report	Calculated ratio of administrative expenses to the sum of loan and grant disbursements
**IDB**	Annual report and financial statement	Calculated the ratio of administrative expenses to total loan disbursements
**EEA**	Administrative budget data[93]	Calculated ratio of donor states' management budget and donor programme partners' participation to Net allocation to beneficiary countries

**PAHO**	Audited financial statements	Calculated ratio of general operating expenses and contractual services to total expenses.
**UNAIDS**	Audited financial statements	Calculated ratio of general operating expenses to total expenses.

Figure S3.15 In-kind contributions by loan- and grant-making DAH channels of assistance

This figure illustrates the proportions of financial and in-kind DAH disbursed by loan- and grant-making institutions. The proportion of in-kind DAH varies, based on the channel. The overall proportion of in-kind DAH received across all channels has grown over time.

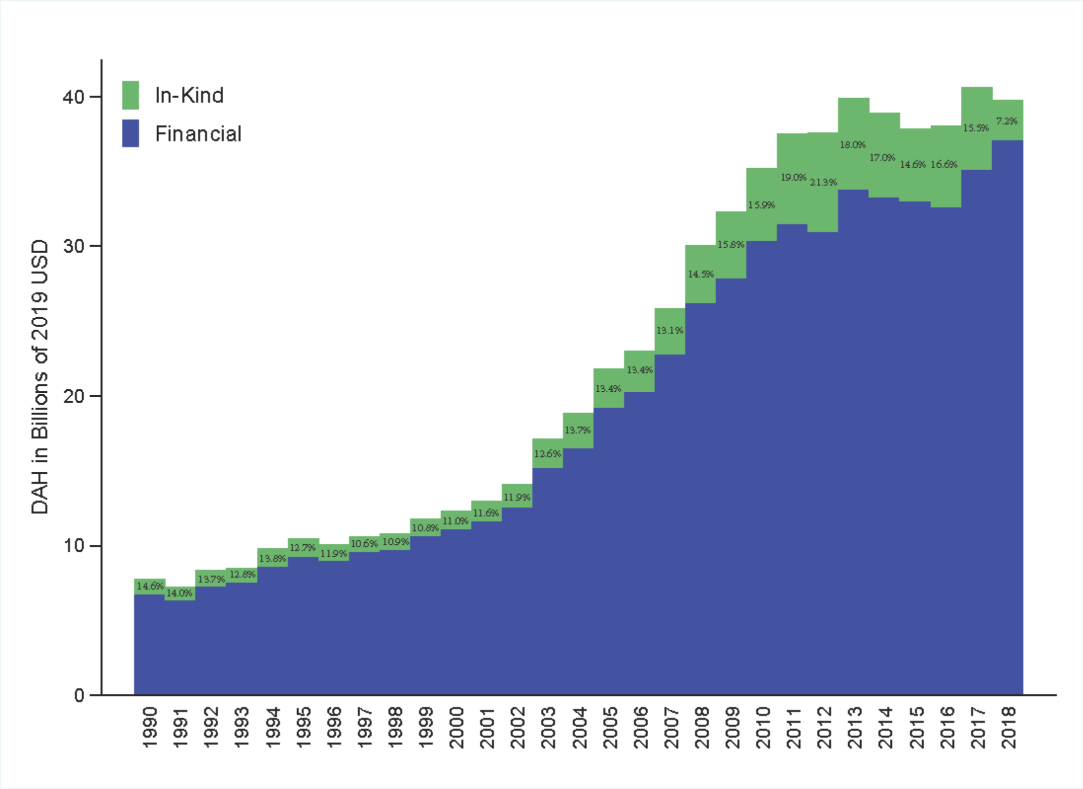

Source: IHME DAH Database (2019)

S3.10 REFERENCES

1 Dieleman JL, Schneider MT, Haakenstad A, *et al*. Development assistance for health: past trends, associations, and the future of international financial flows for health. *The Lancet* 2016; **387**: 2536–44.

2 OECD. International Development Statistics (IDS) online databases. https://www.oecd.org/development/stats/idsonline.htm (accessed Jan 7, 2017).

3 European Commission. Annual reports - international cooperation and development. Int. Coop. Dev. /europeaid/annual-reports_en (accessed Jan 26, 2017).

4 UNAIDS. PCB Archive. http://www.unaids.org/en/aboutunaids/unaidsprogrammecoordinatingboard/pcbmeetingarchive (accessed Aug 14, 2019).

5 UNICEF. UNICEF Strategic Plan: updated financial estimates, 2019-2022. 2019. https://www.unicef.org/about/execboard/files/2019-ABL5-SP_financial_estimates_2019-2022-EN-ODS.pdf

6 UNICEF. United Nations Children's Fund Financial report and audited financial statements for the year ended 31 December 2017 and Report of the Board of Auditors. United Nations: New York, 2018. https://www.unicef.org/about/execboard/files/A-73-5-Add3-BOA_report-ODS-EN.pdf

7 UNICEF. Annual Report 2018. UNICEF. https://www.unicef.org/reports/annual-report-2018 (accessed Sep 18, 2019).

8 United Nations Population Fund. Annual Report 2018. http://www.unfpa.org/annual-report (accessed Oct 28, 2019).

9 UNITAID. Audited Financial Statements (2007-2016). Unitaid. https://unitaid.eu/publications/ (accessed Dec 22, 2017).

10 Pan American Health Organization, World Health Organization. Executive Committee Session. 2017; published online Dec 20. http://iris.paho.org/xmlui/handle/123456789/34210 (accessed August 29, 2017).

11 World Health Organization. Financial report and audited financial statement, 2014. World Health Organization, 2015 http://apps.who.int/gb/ebwha/pdf_files/WHA68/A68_38-en.pdf.

12 The World Bank. Projects & Operations. http://projects.worldbank.org/ (accessed Jan 30, 2017).

13 Paris M. Project database 1990-2016 obtained through personal correspondence. 2016; published online Sept 29.

14 African Development Bank. Compendium of statistics on bank group operations. Tunis, Tunisia: Statistics Department, African Development Bank.

15 African Development Bank. Online project database. https://www.afdb.org/en/projects-and-operations/project-portfolio/ (accessed Jan 30, 2017).

16 Inter-American Development Bank. Projects database. https://www.iadb.org/en/projects-search?query%5Bcountry%5D=&query%5Bsector%5D=SA&query%5Bstatus%5D=&query%5Bquery%5D= (accessed Oct 14, 2019).

17 Neret M. health disbursement data. 2019; accessed Dec 19.

18 Gavi, the Vaccine Alliance. Disbursements and commitments. http://www.gavi.org/results/disbursements/ (accessed Sept 5, 2019).

19 Gavi, the Vaccine Alliance. Pneumococcal AMC. http://www.gavi.org/funding/pneumococcal-amc/ (accessed Sept 5, 2019).

20 Gavi, the Vaccine Alliance. Financial reports. http://www.gavi.org/funding/financial-reports/ (accessed Sept 5, 2019).

21 Gavi, the Vaccine Alliance. Cash Received database. http://www.gavi.org/funding/donor-contributions-pledges/cash-receipts/ (accessed Sept 5, 2019).

22 The Global Fund to fight AIDS, Tuberculosis and Malaria. Grants in detail and Disbursements. https://data-service.theglobalfund.org/downloads (accessed Sept 7, 2019).

23 The Global Fund to fight AIDS, Tuberculosis and Malaria. GFATM annual reports. http://www.theglobalfund.org/en/archive/annualreports/ (accessed Sept 7, 2019).

24 The Global Fund to fight AIDS, Tuberculosis and Malaria. GFATM pledges & contributions report. https://data-service.theglobalfund.org/downloads (accessed Sept 7, 2019).

25 GuideStar USA. Income tax filings. http://www2.guidestar.org/Home.aspx (accessed Oct 25, 2019).

26 United States Agency for International Development. VolAg report: report of voluntary agencies engaged in overseas relief and development. https://www.usaid.gov/pvo/volag-report (accessed Jan 30, 2017).

27 Thomson Reuters. Red book expanded database. New York: Thomson Reuters, 2009.

28 World Health Organization. WHO | Essential medicines. WHO. http://www.who.int/topics/essential_medicines/en/ (accessed Feb 15, 2017).

29 GuideStar. Data Sets for Research. https://learn.guidestar.org/products/data-sets-for-research (accessed Oct 25, 2019).

30 Bill & Melinda Gates Foundation. Audited Financial statements. Seattle, WA: Bill & Melinda Gates Foundation http://www.gatesfoundation.org/Who-We-Are/General-Information/Financials (accessed Oct 12, 2019).

31 Chan S. Foundation Awards and Payments. 2016; published online Aug 16.

32 Foundation Center. Grants database. Candid. https://candid.org/ (accessed Dec 19).

33 EEA Grants-Norway Grants, Financial Mechanism Office. EEA Grants project portal. https://eeagrants.org/resources?title=&field_resource_type_target_id=109 (accessed Sept 17, 2019).

34 Popic A. EEA project data obtained through personal correspondence. Brussels, Belgium. EEA and Norway Grants. 2019.

35 Fofana A. AfDB development support for health projects enquiry. 2019; accessed Dec 19.

36 Rouzinova R. Incorporating UNITAID into Financing Global Health Landscape. 2017; published online Aug 21.

37 Al Anood Al Abdool., United Arab Emirates Ministry of Foreign Affairs and International Cooperation. UAE Foreign Assistance in Health 1990-2008 through personal correspondence. 2018; published online Jan 24.

38 Australian Government. Department of Foreign Affairs and Trade. Aid budget and statistical information. Dep. Foreign Aff. Trade. http://dfat.gov.au/aid/aid-budgets-statistics/Pages/default.aspx (accessed Aug 5, 2019).

39 Department of Foreign Affairs and Trade. Annual reports. Dep. Foreign Aff. Trade. http://dfat.gov.au/about-us/publications/corporate/annual-reports/pages/annual-reports.aspx (accessed Aug 5, 2019).

40 Austria Federal Ministry of Finance. Federal budget. https://www.bmf.gv.at/budget/das-budget/das-budget.html (accessed Jul 26, 2019).

41 Belgium House of Representatives. Project budgets. https://www.lachambre.be/kvvcr/showpage.cfm?section=/pri/budget&language=fr&rightmenu=right_pri&story=2019-budget.xml (accessed Aug 5, 2019).

42 Government of Canada. Planning and performance. GAC. http://international.gc.ca/gac-amc/publications/plans/index.aspx?lang=eng#rpp (accessed Aug 14, 2019).

43 Danish Ministry of Foreign Affairs. Foreign affairs budget. http://www.oes-cs.dk/bevillingslove/ (accessed Aug 5, 2019).

44 European Commission. General Budget - budget on-line. https://ec.europa.eu/budget/graphs/revenue_expediture.html (accessed Aug 23, 2019).

45 Ministry of Finance Finland. State budget bills [in Finnish]. http://budjetti.vm.fi/indox/index.jsp (accessed Aug 6, 2019).

46 Ministry of Foreign Affairs and International Development. Politique francaise en faveur du developpement. Ministry of Foreign Affairs and International Development, 2019 https://www.performance-publique.budget.gouv.fr/sites/performance_publique/files/files/documents/dpt-2019/DPT2019_politique_developpement-W.pdf(accessed Aug 6, 2019).

47 Legifrance. Republique Francaise. Budget and financial documents. https://www.legifrance.gouv.fr/initRechTexte.do (accessed Aug 6, 2019).

48 German Federal Ministry of Economic Cooperation and Development. Plan of the federal budget. Fed. Minist. Econ. Coop. Dev. http://www.bmz.de/en/ministry/InDetail/budget/index.html (accessed Aug 5, 2019).

49 Greece Standing Committee on Economic Affairs. The state budget and budgets for certain special funds and services, 2013 and 2014 [in Greek]. 2014. http://www.hellenicparliament.gr/UserFiles/7b24652e-78eb-4807-9d68-e9a5d4576eff/Proyp2014-prak.pdf.

50 Greece Standing Committee on Economic Affairs. Ratification of the State Budget for the financial year 2017. http://www.hellenicparliament.gr/Nomothetiko-Ergo/Anazitisi-Nomothetikou-Ergou?law_id=76e87dd3-8cae-433f-963f-a6c50141de97 (accessed Feb 13, 2018).

51 Department of Finance, Government of Ireland. The Budget. http://www.budget.gov.ie/Budgets/2019/2019.aspx (accessed Aug 6, 2019).

52 Ministry of Foreign Affairs and International Cooperation. Stato di previsione del ministero degli affari esteri e della cooperazione internazionale. 2019. https://www.esteri.it/mae/resource/doc/2019/01/tabella_6_del_2019.pdf (accessed Aug 7, 2019).

53 Ministry of Finance Japan. Budget. http://www.mof.go.jp/english/budget/budget/index.html (accessed Aug 7, 2019).

54 Korea Official Development Assistance. Comprehensive Implementation plan for international development cooperation by year. http://www.odakorea.go.kr/hz.blltn2.YearPlanSlPL.do?brd_seq=3&blltn_div=oda (accessed Aug 14, 2019).

55 Ministry of Finance Luxembourg. State Budget[in French]. http://www.igf.etat.lu/ (accessed Aug 7, 2019).

56 New Zealand Treasury. Vote budget data - budgets of the New Zealand Government. 2019; published online May 30. http://www.treasury.govt.nz/budget (accessed Aug 8, 2019).

57 Ministry of Foreign Affairs Norway. Email correspondences. April 18, 2011, February 13, 2012, and August 14, 2013.

58 Norwegian Ministry of Finance. The National Budget 2019: a summary. https://www.statsbudsjettet.no/english/ (accessed Nov 20, 2019).

59 Ministry of Finance Portugal. State budget report. http://www.dgo.pt/Paginas/default.aspx (accessed Aug 8, 2019).

60 Ministry of Finance and Public Function Spain. State General Budget [in Spanish] http://www.congreso.es/docu/pge2019/pge_2019-tomos/PGE-ROM/doc/L_19_A_A4.PDF (accessed Aug 12, 2019).

61 Ministry of Foreign Affairs Sweden. International aid budget. http://www.regeringen.se/ (accessed Aug 8, 2019).

62 UK - Department for International Development. IATI Dashboard. http://dashboard.iatistandard.org/publisher/dfid.html (accessed Aug 12, 2019).

63 Her Majesty's Treasury United Kingdom. Budget. https://www.gov.uk/government/publications/spring-budget-2018-documents (accessed Feb 15, 2018).

64 Executive Office of the President of the United States. Budget of the United States Government. https://www.gpo.gov/fdsys/browse/collection.action?collectionCode=BUDGET&browsePath=Fiscal+Year+2014&isCollapsed=true&leafLevelBrowse=false&isDocumentResults=true&ycord=0 (accessed Jan 31, 2017).

65 US Foreign Assistance Dashboard. Foreign assistance by category, health-planned stage. https://www.foreignassistance.gov/explore (accessed Aug 14, 2019).

66 World Health Organization. Proposed programme budget 2018-2019. https://apps.who.int/iris/handle/10665/274693 (accessed Jul 13, 2017).

67 Joint United Nations Programme on HIV/AIDS. Financial report and audited financial statement. https://www.unaids.org/en/aboutunaids/unaidsprogrammecoordinatingboard/pcbmeetingarchive (accessed Aug 14, 2019).

68 Joint United Nations Programme on HIV/AIDS. Unified budget and workplan. https://www.unaids.org/en/aboutunaids/unaidsprogrammecoordinatingboard/pcbmeetingarchive/ (accessed Aug 14, 2019).

69 UNICEF. United Nations Children's Fund Financial report and audited financial statements for the year ended 31 December 2017 and Report of the Board of Auditors. United Nations: New York, 2018. https://www.unicef.org/about/execboard/files/A-73-5-Add3-BOA_report-ODS-EN.pdf

70 Sabbah L. Health expenditure data - UNICEF. 2015; published online Sept 29.

71 UNFPA. Report on contributions by member states and others to UNFPA and revenue projections. https://executiveboard.unfpa.org/ (accessed Aug 23, 2018).

72 UNFPA. Statistical and financial review. https://executiveboard.unfpa.org/ (accessed Aug 30, 2019).

73 Smithson M, Verkuilen J. A better lemon squeezer? Maximum-likelihood regression with beta-distributed dependent variables. *Psychol Methods* 2006; **11**: 54–71.

74 Office of the European Union. EU budget 2018 Financial report. Luxembourg: Office of the European Union, 2015 https://ec.europa.eu/info/publications/eu-budget-2018-financial-report_en (accessed Aug 23, 2019).

75 President's Emergency Plan for AIDS Relief/ US Government. PEPFAR Dashboards- PEPFAR OU Budgets by Budget code FY04-FY19. 2019. https://data.pepfar.gov/dashboards (accessed Jul 29, 2019).

76 President's Malaria Initiative/US Government. Malaria Operational Plans (MOPs). 2019. https://www.pmi.gov/resource-library/mops (accessed Jul 24, 2019).

77 Krackenberger K. IHME-WB FGH 2018 data request. 2018; published online Sept 10.

78 Projects & Operations - Sectors. http://projects.worldbank.org/sector (accessed Jan 26, 2018).

79 Miyuki Parris. Project database 1990-2016 obtained through personal correspondence. Washington D.C.: The World Bank, 2016.

80 World Bank Group - International Development Association. IDA18 Replenishment. Int. Dev. Assoc. - World Bank. 2016; published online Feb 16. http://ida.worldbank.org/financing/replenishments/ida18-replenishment (accessed Dec 21, 2018).

81 Asian Development Bank. Online project database. https://www.adb.org/projects (accessed Jan 30, 2017).

82 African Development Bank Group. Resource Mobilization and Partnerships. https://frmb.afdb.org/?page=adf&subpage=adf-ar (accessed Dec 21, 2018).

83 Asian Development Bank. Asian Development Fund Replenishments. Asian Dev. Bank. 2014; published online Aug 5. https://www.adb.org/site/adf/replenishments (accessed Dec 21, 2018).

84 Gavi, the Vaccine Alliance. Annual financial reports. https://www.gavi.org/search?s=financial&f%5B0%5D=type%3Adocument&page=0 (accessed Sept 5, 2019).

85 World Health Organization. WHO Audited Financial Statements for the year ended 31 December 2018. Geneva, Switzerland: World Health Organization, 2018 http://apps.who.int/gb/ebwha/pdf_files/WHA72/A72_36-en.pdf (accessed Aug 26, 2019).

86 Pan American Health Organization. Financial Report of the Director and Report of the External Auditor for 2018. 1 January 2018 - 31 December 2018 https://www.paho.org/hq/index.php?option=com_content&view=article&id=15194:official-document-357&Itemid=40453&lang=en er 2018. Washington D.C.: Pan American Health Organization, 2019. (accessed Aug 8, 2019)

87 Schlutter A, Volker T, Walkenhorst P. Foundations in Europe: International Reference Book on Society, Management and Law. Gutersloh, Germany; Washington, DC: Bertelsmann Stiftung; Brookings Institution Press, 2001.

88 European Economic Area. EEA grants report. Financial Instrument 1999-2003. Final report. http://eeagrants.org/content/download/10037/139187/version/3/file/141219+FI99-03+final+report.pdf.

89 European Economic Area. EEA report - Financial Mechanism 1994-1998. Final report. http://eeagrants.org/content/download/5915/65422/version/1/file/Financial+Mechanism+1994-1998+Final+Report.pdf.

90 European Economic Area. EEA Annual Report - 2016-2017. https://eeagrants.org/content/download/13045/175890/version/1/file/Annual+Report+2016-2017.pdf.

91 Swedish International Development Agency. Sweden’s aid to the world for all sectors in. Openaid.se. https://openaid.se/aid/ (accessed Dec 21, 2018).

92 NORAD. Norwegian Aid Statistics. NoradDev. https://norad.no/en/front/toolspublications/norwegian-aid-statistics/ (accessed Dec 21, 2018).

93 European Economic Area. Administrative budget - EEA Grants. https://eeagrants.org/Who-we-are/How-we-work/Administrative-budget (accessed Aug 27, 2019).

94 UNFPA. Report on the UNFPA Structured Funding Dialogue 2018-2019. https://www.unfpa.org/report-unfpa-structured-funding-dialogue-2018-2019-dpfpa20198 (accessed Oct 28, 2019).

95 World Health Organization. Status of collection of assessed contributions, including Member States in arrears in the payment of their contributions to an extent that would justify invoking Article 7 of the Constitution. Geneva, Switzerland: World Health Organization, 2019 http://apps.who.int/gb/ebwha/pdf_files/WHA72/A72_37-en.pdf (accessed Aug 26, 2019).

96 World Health Organization. Voluntary contributions by fund and by contributor, 2018. Geneva, Switzerland: World Health Organization, 2019. http://apps.who.int/gb/ebwha/pdf_files/WHA72/A72_INF5-en.pdf (accessed Aug 26, 2019).

97 Micah AE, Zhao Y, Chen CS, et al. Tracking development assistance for health from China, 2007–2017BMJ Global Health 2019;4:e001513.

98 Lavado R.F. ADB development support for health projects enquiry. 2019; accessed Dec 19.

99 International Monetary Fund. World Economic Outlook Database: October 2019. https://www.imf.org/external/pubs/ft/weo/2019/02/weodata/index.aspx (accessed Oct 20, 2019).

SECTION 4: TRACKING TOTAL SPENDING ON MALARIA BY SOURCE IN 106 COUNTRIES, 2000-2017

S4.1 Currency Conversion

All malaria expenditure estimates were made and reported in 2019 United States dollars (USD), with the exception of Venezuela, whose expenditure was reported in 2014 USD. Data sources reported expenditure in either nominal local currency units (LCUs) or nominal USD. To convert nominal LCUs to USD, we applied deflators to nominal LCUs to inflate to 2019 LCUs. We then applied exchange rates to produce 2019 USD. When LCUs were not reported, we extracted reported expenditure in nominal USD, applied corresponding nominal exchange rates to produce nominal LCUs, inflated nominal LCUs to 2019 LCUs with deflators, and finally exchanged 2019 LCUs to 2019 USD. More information can be found in Section 2 of this appendix.

S4.2 Spaciotemporal Gaussian process regression (ST-GPR)

We used spatiotemporal Gaussian process regression (ST-GPR) several times throughout the process of estimating malaria spending. ST-GPR generates a complete time-series of data for a set of locations, follows the data where it is available and borrows strength across time, geographic regions, and covariates' predictive power when data is not available. The interested reader may view a complete description of ST-GPR here[1]. Briefly, ST-GPR has three primary steps. First, a linear mixed effects model is run with a given set of predictors. Predictions from the first step provide the general trend within the data. In the second step, spatiotemporal patterns are estimated by applying a series of spatiotemporal weights to average the residuals of the first step linear model. These spatiotemporal patterns are then added to the linear predictions to generate spatiotemporally smoothed predictions. Finally, the smoothed predictions are passed as the mean function to a Gaussian process regression along with the data to produce final ST-GPR predictions. For every country-year, 1,000 draws were generated from the Gaussian process regression model and used in subsequent calculations.

S4.3 Government health expenditure as a source on malaria

S4.3.1 Data

Government spending on malaria was drawn from three main sources. First, we extracted data on government spending reported by National Malaria Control Programmes (NMCPs) to the World Health Organization (WHO) and published in the WHO's annual World Malaria Reports (WMRs).[2] We conducted a web search of all existing National Health Accounts, including those hosted by the WHO on the Global Health Expenditure Database, and found 61 reports that estimated government expenditure as a source dedicated to malaria prevention, control, and treatment.[3] Finally, we extracted the government spending reported by governments submitting proposals, concept notes, and funding requests to the Global Fund. We excluded any projections for spending reported in these documents submitted to the Global Fund. Table S4.1 shows the number of countries-years available for each data source.

Table S4.1: Main data sources for government health spending as a source for malaria

Data source	Country-years of data
NMCP reports in the WMRs	1010
NHAs	97
Global Fund proposals, concept notes, and funding requests	466

The spending reported by NMCPs to the WMRs typically capture expenditure on prevention activities, such as insecticide-treated nets, indoor residual spraying, and chemoprevention, as well as the purchase of antimalarial drugs and diagnostics. However, they do not include spending on patient care – e.g. spending on the labor, facilities, and others costs involved in providing care to malaria patients in government health facilities outside of drugs and diagnostics. Because the government spending numbers reported in the Global Fund proposals, concept notes, and funding requests are typically submitted by the same source – NMCPs – and aligned well with the reported numbers in the WMR, we assumed that this spending also did not include patient care.

S4.3.2 Modeling government expenditure on malaria patient care

To estimate government spending on patient care, we built a price-volume model focused on spending on inpatient and outpatient care, respectively, as shown in (1).

$$\text{Gov}_{\text{mal patient care}} = (\text{Gov cost per admission}_{\text{mal}} * \text{Public admissions}_{\text{mal}}) + (\text{Gov cost per outpatient visit}_{\text{mal}} * \text{Public outpatient visits}_{\text{mal}}) \qquad (1)$$

In (2) - (5) below, we show the equations used to estimate each element of $\text{Gov}_{\text{mal patient care}}$.

Estimates of $\text{Gov cost per admission}_{\text{mal}}$, shown in (2), were based on country-specific average inpatient unit cost of all health conditions from Moses et al. (2018).[4] We extracted estimates of malaria inpatient unit costs for 17 country-years from 13 peer-reviewed articles, as shown in Table S4.2.[5-17] Only 8 country-years from these studies reported inpatient unit costs without spending on drugs and diagnostics. Therefore, we took the median share of non-drug, non-diagnostic spending and applied it to the remaining 11 country-years to generate $\text{mal inpatient unit cost}_{\text{non drug \& non diagnostic}}$. We converted these values to 2019 US dollars and took the ratio of these values to the inpatient unit costs from Moses et al. (2018) in the country and year in which the study took place. The median of this ratio (0.33) was applied to all inpatient unit costs to calculate $\text{Gov cost per admission}_{\text{mal}}$.

$$\text{Gov cost per admission}_{\text{mal}} = \text{inpatient unit cost} * \text{med}\left(\frac{\text{mal inpatient unit cost}_{\text{non drug \& non diagnostic}}}{\text{inpatient unit cost}}\right) \qquad (2)$$

Table S4.2: Malaria inpatient unit costs extracted from peer-reviewed literature

Country	Year	Inpatient unit costs (2019 USD)	Non-drug, non-diagnostic inpatient unit costs (2019 USD)	Study
Bangladesh	2003	63.26		Lubell et al. 2009
Cameroon	2013	67.61	38.24	Maka et al. 2016
China	2014	905.84	171.27	Tang et al. 2017
Democratic Republic of the Congo	2005	37.12		Tsakala et al. 2005
Ghana	2009	26.89		Sicuri et al. 2013
India	1996	28.21	18.84	Gogtay et al. 2003
India	2003	71.85		Lubell et al. 2009
Indonesia	2003	73.25		Lubell et al. 2009
Kenya	2004	163.6		Ayieko et al. 2009
Kenya	2009	31.42		Sicuri et al. 2013
Kenya	2015	92.52		Rakuomi et al. 2017
Nigeria	2009	101.09	91.23	Lubell et al. 2011
South Africa	2004	419.14		Muheki et al. 2004
Tanzania	2009	69.24	46.16	Lubell et al. 2011
Tanzania	2009	21.26		Sicuri et al. 2013
Thailand	2001	233.95	122.22	Kyaw et al. 2014
Uganda	2009	59.25	49.12	Lubell et al. 2011
Zimbabwe	2000	1881.41	1755.82	Hongoro and McPake 2003

$\text{Public admissions}_{\text{mal}}$, shown in (3), represents the number of inpatient admissions for malaria in government-run health facilities. NMCP programs reported the total number of $\text{inpatient cases}_{\text{mal}}$ to the WHO (not disaggregated by the public and private sector but capturing admissions in both sectors) and they are published in WMRs annually. Some missingness characterizes these reported values – malaria inpatient cases were available for 29% of country-years in our sample of 106 countries. For this reason, we modeled logit-transformed malaria admissions as a share of malaria incident cases from the Global Burden of Disease (GBD) study 2017 with spatiotemporal Gaussian process regression (ST-GPR) and the following covariates: malaria Lysenko 1 from the Global Burden of Disease (GBD) study 2017,[18] natural log transformed ten-year lag-distributed income per capita (LDI per capita), and random effects on country, region, and super-region. We back-transformed the dependent variable and multiplied the ratio with the number of malaria incident cases to produce total malaria inpatient admissions for each of the 106 countries in our sample over 2000-2017.

$$\text{Public admissions}_{\text{mal}} \quad = \qquad (3)$$

$$\text{inpatient cases}_{\text{mal}} * \left(\frac{\text{public treated cases}_{\text{mal}}}{\text{treated cases}_{\text{mal}}}\right)$$

$\text{public treated cases}_{\text{mal}}$ were based on estimates of treatment-seeking in the public sector among children under five with a fever in the last two weeks, as estimated by Battle et al. (2016).[19] $\text{treated cases}_{\text{mal}}$ were from the same source but focused on all treatment-seeking among children under five with a fever in the last two weeks. These are the only comprehensive set of estimates of malaria treatment-seeking available to-date. Furthermore, treatment-seeking rates for malaria among children have been shown to be similar to treatment-seeking among adults.[20,21]

To impute data for countries not included in the original study, we used a similar approach to Battle et al. (2016) – we modeled logit-transformed public treatment-seeking as a share of children under five with a fever in the last two weeks with ST-GPR and the following covariates: logit-transformed, out of-pocket (OOP) expenditure as a share of loess-smoothed total OOP health expenditure from Global Burden of Disease Health Financing Collaborator Network (2019),[22] log LDI per capita, coverage of the diphtheria-tetanus-pertussis vaccine, four antenatal care visits, skilled birth attendance, and random effects on country, region, and super-region. $\text{treated cases}_{\text{mal}}$ were imputed with a linear mixed model with fixed effects on year and year squared and random effects on region and super-region. Since both $\text{public treated cases}_{\text{mal}}$ and $\text{treated cases}_{\text{mal}}$ were modeled as a share of fever cases, we multiplied these ratios by malaria incident cases from GBD 2017 to calculate counts of each measure. We assumed that the share of total admissions in the public sector is similar to the share of all treatment seeking that occurred in the public sector and thus apply the ratio $\frac{\text{public treated cases}_{\text{mal}}}{\text{treated cases}_{\text{mal}}}$ to $\text{inpatient cases}_{\text{mal}}$ to estimate Public $\text{admissions}_{\text{mal}}$.

Estimating $\text{Gov cost per outpatient visit}_{\text{mal}}$, shown in (4), employs a similar approach to $\text{Gov cost per admission}_{\text{mal}}$ in (2). Estimates of $\text{outpatient unit cost}$ for all health conditions were sourced from Moses et al. (2018).[23] Malaria inpatient unit costs were extracted from 10 peer-reviewed articles.[24-32] Only 4 country-years additionally reported outpatient unit costs without spending on drugs and diagnostics. The median share of non-drug, non-diagnostic spending was thus applied to the remaining 6 country-years studies to generate $\text{mal outpatient unit cost}_{\text{non drug \& non diagnostic}}$. We converted these values to 2019 US dollars and took the ratio of these values to the outpatient unit costs from Moses et al. (2018) in the country and year in which the study took place. The median of this ratio (1.04) was applied to all outpatient unit costs to calculate $\text{Gov cost per outpatient visit}_{\text{mal}}$. Table S4.3 shows each of the point estimates extracted from peer reviewed literature.

$$\text{Gov cost per outpatient visit}_{\text{mal}} = \qquad (4)$$

$$\text{outpatient unit cost} * \text{med}\left(\frac{\text{mal outpatient unit cost}_{\text{non drug \& non diagnostic}}}{\text{outpatient unit cost}}\right)$$

Table S4.3: Malaria outpatient unit costs extracted from peer-reviewed literature

Country	Year	Outpatient unit costs (2019 USD)	Non-drug, non-diagnostic outpatient unit costs (2019 USD)	Study
China	2013	1209.06		Liu et al. 2016
Ghana	2009	2.83		Sicuri et al. 2013
Kenya	2009	4.17		Sicuri et al. 2013
Nigeria	2013	25.56	21.09	Ezenduka et al. 2017
Nigeria	2016	16.32	1.68	Salwu et al. 2016
South Africa	2004	27.7		Muheki et al. 2004
Tanzania	2003	2.7		Njau et al. 2008
Tanzania	2009	2		Sicuri et al. 2013
Tanzania	2005	7.02	5.83	Wiseman et a. 2006
Zambia	2005	6.56	1.71	Chanda et al. 2007

Estimates of $\text{Public outpatient visits}_{\text{mal}}$, shown in (5), was based on $\text{Public admissions}_{\text{mal}}$, estimated as in (3), and the $\text{public treated cases}_{\text{mal}}$ which is an input to those estimates. $\text{Public admissions}_{\text{mal}}$ were subtracted from $\text{public treated cases}_{\text{mal}}$ to calculate all outpatient visits for malaria in each country over 2000-2017.

$$\text{Public outpatient visits}_{\text{mal}} = \text{public treated cases}_{\text{mal}} - \text{Public admissions}_{\text{mal}} \quad (5)$$

S4.3.3 Modeling total government expenditure on malaria

Total government spending is the sum of $\text{Gov}_{\text{mal patient care}}$ as described above and the government spending from the NMCP programs, as reported in WMRs and Global Fund proposals, concept notes, and funding requests ($\text{NMCP Gov}_{\text{mal}}$), appended by the government spending on malaria estimated in NHAs ($\text{NHA Gov}_{\text{mal}}$), as shown in (6).

$$\text{Gov total}_{\text{mal}} = \text{NMCP Gov}_{\text{mal}} + \text{Gov}_{\text{mal patient care}} = \text{NHA Gov}_{\text{mal}} \quad (6)$$

To estimate a full time series for all years and countries in our study, we modelled $\text{Gov total}_{\text{mal}}$ as a logit transformed fraction of loess smoothed, total government health spending using ST-GPR. We first considered the following covariates: malaria incidence, malaria prevalence, LDI per capita, coverage of indoor residual spraying (IRS), insecticide-treated nets (ITNs), malaria Lysenko 1, one- and four-visit antenatal care coverage, ACT coverage, the healthcare access and quality index (HAQI), the proportion of the population living in an urban area, coverage of skilled birth attendance (SBA), the sociodemographic index (SDI) and the universal health coverage (UHC) index. All the covariates measured as

a proportion were logit-transformed. We sourced all covariate estimates from the GBD Study 2017 and the Malaria Atlas Project.

Because the availability of covariate data is higher for sub-Saharan Africa and patterns of incidence and intervention strategies differ from other regions, we split the government malaria spending data into two groups – sub-Saharan African and non-sub-Saharan African countries – and modeled each group separately. We performed covariate selection for each dataset, starting with the set of 14 potential covariates.

We first conducted a lasso regression to determine which covariates were least correlated, conditional on other covariates, with the fraction of government spending on malaria as the dependent variable. Covariates with an estimated coefficient of zero were removed from the set of possible covariates. We then used linear mixed effects regression to estimate all models including all possible combinations of the remaining covariates. We then selected the intersection of 1000 best models with the lowest Akaike information criterion (AIC) and Bayesian information criterion (BIC) values. Finally, we completed a 10-fold cross-validation with out-of-sample predictions on these selected 1000 models. We selected the best model based on out-of-sample root mean squared error. The top-five models are shown in Table S4.4. Our final model is model 1 in both SSA and non-SSA sections.

Table S4.4: Top government spending models based on out-of-sample root-mean square error

SSA	Covariates	OOS RMSE
1	Insecticide-treated nets (ITNs), Coverage of indoor residual spraying (IRS), One visit antenatal care coverage, Coverage of skilled birth attendance (SBA), Malaria prevalence	1.539028
2	Insecticide-treated nets (ITNs), Coverage of indoor residual spraying (IRS), One visit antenatal care coverage, Coverage of skilled birth attendance (SBA), Malaria incidence	1.539187
3	Insecticide-treated nets (ITNs), Coverage of indoor residual spraying (IRS), One visit antenatal care coverage, Healthcare access and quality index (HAQI), Malaria prevalence	1.539274
4	Insecticide-treated nets (ITNs), One visit antenatal care coverage, Coverage of skilled birth attendance (SBA), Malaria prevalence	1.539577
5	Insecticide-treated nets (ITNs), Coverage of indoor residual spraying (IRS), One visit antenatal care coverage, Proportion of the population living in an urban area, Malaria incidence	1.539592
**Non - SSA**	**Covariates**	**OOS RMSE**
1	Coverage of indoor residual spraying (IRS), Malaria Lysenko 1, Proportion of the population living in an urban area, Coverage of skilled birth attendance (SBA), Malaria incidence, Malaria prevalence, Lag distributed income (LDI) per capita	0.800753

2	Coverage of indoor residual spraying (IRS), Proportion of the population living in an urban area, Coverage of skilled birth attendance (SBA), Malaria incidence, Malaria prevalence, Lag distributed income (LDI) per capita	0.800794
3	Malaria Lysenko 1, Proportion of the population living in an urban area, Coverage of skilled birth attendance (SBA), Malaria incidence, Malaria prevalence, Lag distributed income (LDI) per capita	0.801354
4	Proportion of the population living in an urban area, Coverage of skilled birth attendance (SBA), Malaria incidence, Malaria prevalence, Lag distributed income (LDI) per capita	0.801478
5	Coverage of indoor residual spraying (IRS), Malaria Lysenko 1, Coverage of skilled birth attendance (SBA), Malaria incidence, Malaria prevalence, Lag distributed income (LDI) per capita	0.801484

Finally, we used a spatiotemporal Gaussian process regression (ST-GPR) to model government spending on malaria, independently modeling sub-Saharan African countries and non-sub-Saharan African countries. The first stage of ST-GPR was a mixed-effect model with random effects on Global Burden of Disease region and country, as well as the covariates selected using the method described above. To detect and reduce the influence of outlier data points, we used the selected model to measure Cooke's distance for each data point. We excluded each data point if Cooke's distance was greater than 4/n where n is the total number of $\text{Gov total}_{\text{mal}}$ data points. Additionally, we removed several data points by visual inspection; these are listed in table S4.5 below.

Table S4.5: Malaria GHE data points removed by visual inspection

Country	Year	Source Type	Value (2019 USD)
Papua New Guinea	2012	Global Fund Concept Note	6,541,201
Papua New Guinea	2013	Global Fund Concept Note	6,823,760
Venezuela	2004	World Malaria Report	62,168,499
South Africa	2007	World Malaria Report	156,735,278
Zimbabwe	2006	Global Fund Proposal	16,939,048
Zimbabwe	2007	Global Fund Proposal	15,966,816

S4.4 Out-of-pocket spending on malaria

S4.4.1 Price-volume model

We found only 83 points of malaria OOP expenditure, all sourced from NHAs. To augment this sparse dataset, we developed a price-volume model of malaria OOP, focused on the OOP costs of malaria treatment. Because of the substantial investment of governments and donors into malaria prevention and

control, including financing of insecticide-treated nets, indoor residual spraying, community outreach and chemoprevention,[33] we assumed that the OOP spending on prevention is minimal, and focused on estimating the OOP spending on malaria treatment. Our price-volume model is shown in (7). The estimation strategy for each component of (7) is described in (8)-(12).

$$\text{OOP}_{\text{mal}} = (\text{OOP cost per admission}_{\text{mal}} * \text{Admissions}_{\text{mal}}) + (\text{OOP cost per outpatient visit}_{\text{mal}} * \text{Outpatient visits}_{\text{mal}}) + \text{OOP drug expenditure}_{\text{mal}} \quad (7)$$

The approach to estimating volume of malaria treatment-seeking, both $\text{Admissions}_{\text{mal}}$ and $\text{Outpatient visits}_{\text{mal}}$, are described above. Inpatient cases were sourced from the WHO's WMRs and were modeled as share of all inpatient stays with ST-GPR. $\text{Outpatient visits}_{\text{mal}}$ are $\text{treated cases}_{\text{mal}}$, or treatment-seeking among children under five with a fever in the last two weeks based on Battle et al. (2016), minus $\text{Admissions}_{\text{mal}}$.

$\text{OOP cost per admission}_{\text{mal}}$, as shown in (8), was estimated similarly to $\text{Gov cost per admission}_{\text{mal}}$, with some modifications. Moses et al. (2018) was the source of inpatient unit cost. We adjusted these country-specific values to reflect the share of unit costs sourced OOP using the fraction $\frac{\text{OOP inpatient spend}}{\text{Total inpatient spend}}$, calculated using 625 country-years of extracted NHA data. $\frac{\text{OOP inpatient spend}}{\text{Total inpatient spend}}$ was logit-transformed and modeled with ST-GPR with the following covariates: logit-transformed OOP/THE from the Global Burden of Disease Financing Global Health Collaborator Network (2019), natural log-transformed LDI per capita and country, region, and super-region effects. Estimates of OOP spending on malaria admissions (mal OOP inpatient unit cost) were extracted from nine peer-reviewed articles,[34-42] shown in Table S4.6, and converted to 2019 US dollars. To adjust average OOP inpatient unit costs to represent OOP costs per inpatient stay, we computed the median of the ratio $\frac{\text{mal OOP inpatient unit cost}}{\left(\text{inpatient unit cost} * \frac{\text{OOP inpatient spend}}{\text{Total inpatient spend}}\right)}$, which amounted to 0.32, and applied it to all other estimates of $\text{inpatient unit cost} * \frac{\text{OOP inpatient spend}}{\text{Total inpatient spend}}$ to generate $\text{OOP cost per admission}_{\text{mal}}$.

$$\text{OOP cost per admission}_{\text{mal}} = \text{inpatient unit cost} * \frac{\text{OOP inpatient spend}}{\text{Total inpatient spend}} * \text{med}\left(\frac{\text{mal OOP inpatient unit cost}}{\left(\text{inpatient unit cost} * \frac{\text{OOP inpatient spend}}{\text{Total inpatient spend}}\right)}\right) \quad (8)$$

Table S4.6: Malaria OOP inpatient unit costs extracted from peer-reviewed literature

Country	Year	OOP malaria inpatient unit costs (2019 USD)	Study
China	2012	424.29	Xia et al. 2016
Democratic Republic of the Congo	2011	235.37	Ilunga-Ilunga et al. 2015
Ethiopia	2003	115.57	Deressa et al. 2007
Ghana	2009	23.97	Sicuri et al. 2013
Kenya	2009	16.39	Sicuri et al. 2013
Malawi	2012	7.27	Hennessee et al. 2017
Mozambique	2001-2002	5.23	Castillo-Riquelme et al. 2008
Nigeria	2009	7.23	Onwujekwe O et al. 2013
South Africa	2001-2002	1.3	Castillo-Riquelme et al. 2008
Tanzania	2009	6.25	Sicuri et al. 2013
Uganda	2009	7.62	Nabyonga Orem et al. 2013
Zimbabwe	2014-2015	36.04	Gunda et al. 2017

Estimating $\text{OOP cost per outpatient visit}_{\text{mal}}$ employed a similar approach to strategy applied in (8). We used outpatient unit cost from Moses et al. (2018) and adjusted those country-specific estimates to reflect the OOP unit costs of outpatient spending by applying the fraction of outpatient spending sourced OOP: $(\frac{\text{OOP outpatient spend}}{\text{Total outpatient spend}})$. This fraction was calculated with 625 country-years of extracted NHA and modeled with ST-GPR . As covariates, we used logit-transformed OOP/THE, log LDI per capita and random effects on country, region and super region. As shown in Table S4.7, we extracted 13 country-years of data from 10 peer-reviewed articles to create $\text{mal OOP outpatient unit cost}_{\text{non drug}}$.[43-54] To adjust average OOP outpatient unit costs to represent malaria OOP non-drug unit costs, we computed the median of the ratio $\frac{\text{mal OOP outpatient unit cost}_{\text{non drug}}}{\left(\text{outpatient unit cost} * \frac{\text{OOP outpatient spend}}{\text{Total outpatient spend}}\right)}$, which amounted to 0.37, and applied it to all other estimates of $\text{outpatient unit cost} * \frac{\text{OOP outpatient spend}}{\text{Total outpatient spend}}$ to generate $\text{OOP cost per outpatient visit}_{\text{mal}}$.

$$\text{OOP cost per outpatient visit}_{\text{mal}} = \text{outpatient unit cost} * \frac{\text{OOP outpatient spend}}{\text{Total outpatient spend}} * \text{med}\left(\frac{\text{mal OOP outpatient unit cost}_{\text{non drug}}}{\left(\text{outpatient unit cost} * \frac{\text{OOP outpatient spend}}{\text{Total outpatient spend}}\right)}\right) \quad (9)$$

Table S4.7: Malaria OOP non-drug outpatient unit costs extracted from peer-reviewed literature

Country	Year	OOP non-drug outpatient unit cost (2019 USD)	Study
Burkina Faso	2000	22.35	Mugisha et al. 2002
China	2012	22.15	Xia et al. 2016
Kenya	2010	1.14	Chuma et al. 2010.
Malawi	2004	0.02	Mota et al. 2009.
Malawi	2009	0.02	Ewing et al. 2011
Mozambique	2002	0.24	Castillo-Riquelme M et al. 2008.
Nigeria	2013	0.37	Onwujekwe O et al. 2013.
South Africa	2002	0.27	Castillo-Riquelme M et al. 2008.
Sri Lanka	2004	5.97	Mustafa & Babiker 2007
Uganda	2009	3.29	Nabyonga Orem et al. 2013
Uganda	2012	0.04	Matovu et al. 2014
Vietnam	2004	1.14	Morel et al. 2008

We modeled drug expenditure on malaria broken down by spending on ACTs, which tend to be higher and have a different trend over 2000-2017, separately from other antimalarials, which included monotherapies, chloroquine, and other antimalarials, as shown in (10).

$$\text{OOP drug expenditure}_{\text{mal}} = \text{ACT price} * \text{ACT covered cases} + \text{Other antimarial price} * \text{Other antimalarial covered cases} \tag{10}$$

Data on OOP spending on ACTs and other antimalarials were sourced from reports on the outlet surveys conducted by ACTwatch (169),[55] Health Action International (738),[56] and as reported in a report by the Affordable Medicines Facility – malaria (AMFm).[57] The outlet surveys captured the price charged to patients for an array of malaria drugs as well as their availability in private and public drug outlets, including government-run health facilities. For each outlet survey country-year, we took the average across the different ACTs and other antimalarials, respectively, weighted by their availability in different outlet types to approximate access to public and private OOP prices of drugs. All OOP drug prices were converted to 2019 USD. We modeled ACT and other antimalarial OOP prices separately.
Since 2000, the average cost of an antimalarial treatment course has decreased substantially,[58] influenced by negotiations to lower prices by AMF-m and the Global Fund. Furthermore, since 2000, many countries

have instituted policies to provide ACTs free-of-charge in the public sector. Therefore, we modeled a country-specific trend for ACTs, incorporating changes to ACT-free policies.

Our main covariate for modeling OOP drug prices was the public procurement price of ACTs, sourced from the WHO's Global Price Reporting Mechanism (GPRM),[59] and the Global Fund's Price and Quality Reporting database,[60] which capture ACTs procured by NMCPs, non-governmental organizations and other institutions that received grants from the Global Fund or participate in the GPRM. For each country-year, we took the average procurement price paid for ACTs, weighted by the volume of drugs purchased, and converted all prices to 2019 US dollars. A full time series of ACT procurement prices was modeled with ST-GPR with log-transformed average global price of an ACT treatment course from Management Sciences for Health,[61] and random effects on country, region and super region. We log-transformed OOP ACT price, and applied a robust regression to log-transformed ACT procurement price and an indicator for whether not a ACTs were provided free in the public sector in that country-year as reported in the WHO's WMRs.

Average prices for other antimalarials have not changes substantially in real terms since 2000.[62] However, we wanted to capture regional and country variation in antimalarial OOP drug prices. We therefore modeled log-transformed other antimalarial OOP price with random effects on country, region and super-region with a linear mixed effects model.

The equations used for ACT covered cases and other antimalarial covered cases are shown in (11) and (12). We assumed that all treated cases for malaria received some sort of antimalarial drug. We relied on estimates of ACT coverage among children under five with a fever in the last two weeks estimated by the Bennett et al. (2017).[63] These data did not cover countries outside of sub-Saharan Africa, however, and so we imputed ACT coverage for these countries by replicating the same approach employed by Bennet et al. (2017). We extracted the ACTs delivered for each country, as reported in the WHO's WMR. To fill in missing values in this dataset, we modeled log ACTs delivered per incident case in ST-GPR, with log LDI per capita as the sole covariate and random effects on country, region and super-region. Finally, logit-transformed ACT coverage was modeled with ST-GPR with log ACTs per capita and log LDI per capita and random effects on country, region, and super region. We multiplied ACT coverage and incidence cases to calculate ACT treated cases. We assume that all treated cases that did not get an ACT received another antimalarial. As described in the government spending section, $\text{treated cases}_{\text{mal}}$ were based on Battle et al. (2016) and $\text{inpatient cases}_{\text{mal}}$ were modeled based on the inpatient cases reported in the WHO's WMRs. We subtracted $\text{inpatient cases}_{\text{mal}}$ from $\text{treated cases}_{\text{mal}}$ to focus on non-inpatient treatment seeking.

$$\text{ACT covered cases} = \qquad (11)$$

$$(\text{treated cases}_{\text{mal}} - \text{inpatient cases}_{\text{mal}}) * \frac{\text{ACT treated cases}}{\text{treated cases}_{\text{mal}}}$$

$$\text{Other antimalarial covered cases} = \qquad (12)$$

$$(\text{treated cases}_{\text{mal}} - \text{inpatient cases}_{\text{mal}}) * \left(1 - \frac{\text{ACT treated cases}}{\text{treated cases}_{\text{mal}}}\right)$$

S4.4.2 Modeling total out-of-pocket expenditure on malaria

We appended $\mathrm{OOP}_{\mathrm{mal}}$ from the price-volume model to the 81 NHA points to create the full malaria OOP dataset. Using these data, we used ST-GPR to model logit-transformed OOP spending on malaria as a share of total loess-smoothed OOP spending excluding OOP spending on HIV. Because the availability of covariate data is higher for sub-Saharan Africa and patterns of incidence and intervention strategies differ from other regions, we split the OOP malaria spending data into two groups – sub-Saharan African and non-sub-Saharan African countries – and modeled each group separately. Both models included the following covariates: LDI per capita and malaria incidence per capita, with random effects for country, region, and super-region. The sub-Saharan Africa model additionally included coverage of indoor residual spraying (IRS) and insecticide-treated nets (ITNs). To detect and reduce the influence of outlier data points, we used the selected model to measure Cooke's distance for each data point; outliers were detected at the country level, with an individual model run for each location, without the geographic random effects. We excluded data points whose Cooke's distance was greater than 4/n where n is the total number of OOP data points for each country.

S4.4.3 Excluding data from an NHA from Nigeria

As described above, after generating cost-volume estimates of OOP spending on malaria, we appended these estimates to any data on malaria OOP spending extracted from NHAs and ran ST-GPR on the combined dataset to generate a complete series. In the past, the NHA and cost-volume data have not diverged to the extent that they caused concern. However, this year, new data from a Nigerian NHA was so different from our cost-volume estimates and so much substantially larger than most other estimates of malaria OOP spending that it prompted additional investigations, which led us to exclude this NHA's data from our OOP model.

In order to determine whether to include data on malaria OOP spending from this NHA, we compared the NHA's data and our cost*volume estimates to our estimates of malaria OOP spending for 16 similar countries. In order to determine a list of similar countries, we used k-means clustering to group all 106 malaria endemic countries in terms of malaria incidence per capita, lag-distributed income per capita, and the fraction of total health sector spending that is OOP. The number of clusters (7) was determined based on the elbow of a scree-plot. The countries in Nigeria's cluster are shown below in Table S4.8. Once this group of 16 comparison countries was determined, we compared the cost-volume and NHA data on Nigeria's OOP malaria spending to the minimum, maximum, and mean values of OOP spending across the 16 other countries in Nigeria's cluster. Results are displayed in Table S4.9.

Table S4.8: List of countries in Nigeria's cluster

Countries in Nigeria's Cluster
Benin
Burkina Faso
Cameroon
Central African Republic
Chad
Congo
Cote d'Ivoire

Democratic Republic of the Congo
Ghana
Guinea
Liberia
Mali
Niger
Sierra Leone
Togo
Uganda

As shown below in Table S4.9, the NHA's reported spending per capita, per prevalent case, and per incident case are each about 10 times the maximum value of the cluster, while the cost-volume estimates are close to the mean values from the cluster. In comparison, although this is not shown below, Nigeria's government spending on malaria per capita, per incident case, and per prevalent case are very near the mean of this cluster. We believe this is strong evidence that the OOP malaria spending data from the recent Nigerian NHA is too high, and that the cost-volume estimates are well within reason.

Table S4.9: Results from clustering analysis

	Nigeria cost volume	Nigeria NHA	Comparator country min	Comparator country mean	Comparator country max
**Malaria OOP per capita**	0.97	25.64	0.27	1.21	2.82
**Malaria OOP per health sector OOP**	0.02	0.44	0.02	0.06	0.14
**Malaria OOP per prevalent case**	6.96	184.51	1.18	6.66	17.81
**Malaria OOP per incident case**	4.41	116.77	1.05	4.61	12.12

In addition to comparing the NHA and cost*volume estimates for Nigeria against malaria OOP estimates from comparator countries, we also identified seven articles on household spending on malaria in Nigeria and compared the estimates presented in these studies to data from the NHA and our cost-volume estimates. This comparison provided further evidence that the Nigerian NHA's data on OOP malaria spending was implausibly high. The studies we reviewed can be found in Table S4.10, below.

Table S4.10: Reviewed literature on Malaria OOP spending

Onwujekwe, O., Uguru, N., Etiaba, E., Chikezie, I., Uzochukwu, B., & Adjagba, A. (2013). The economic burden of malaria on households and the health system in Enugu State Southeast Nigeria. *PloS one*, *8*(11), e78362.
Onwujekwe, O., Chima, R., & Okonkwo, P. (2000). Economic burden of malaria illness on households versus that of all other illness episodes: a study in five malaria holo-endemic Nigerian communities. *Health policy*, *54*(2), 143-159.

Jimoh, A., Sofola, O., Petu, A., & Okorosobo, T. (2007). Quantifying the economic burden of malaria in Nigeria using the willingness to pay approach. *Cost effectiveness and resource allocation*, 5(1), 6.
Onwujekwe, O., Dike, N., Uzochukwu, B., & Ezeoke, O. (2010). Informal payments for healthcare: differences in expenditures from consumers and providers perspectives for treatment of malaria in Nigeria. *Health policy*, 96(1), 72-79.
Teklehaimanot, A., & Mejia, P. (2008). Malaria and poverty. *Annals of the New York Academy of Sciences*, 1136(1), 32-37.
Onwujekwe, O., Hanson, K., Uzochukwu, B., Ichoku, H., Ike, E., & Onwughalu, B. (2010). Are malaria treatment expenditures catastrophic to different socio-economic and geographic groups and how do they cope with payment? A study in southeast Nigeria. *Tropical medicine & international health*, 15(1), 18-25.
Okorosobo, T., Okorosobo, F., Mwabu, G., Orem, J. N., & Kirigia, J. M. (2011). Economic burden of malaria in six countries of Africa. *Eur J Bus Manag*, 3(6), 42-63.

S4.5 Prepaid private spending on malaria

Just 50 country-years of NHA data included prepaid private (PPP) expenditure on malaria. We combined these data with the estimates outlined above for OOP and government spending on malaria and malaria-focused development assistance for health (DAH) data from the Institute for Health Metrics and Evaluation to calculate total spending on malaria.[64] As a share of total spending on malaria, median PPP from the 50 NHA country-years was 0.8%. We computed the median ratio of malaria PPP share of non-PPP malaria spending to total loess-smoothed PPP over loess-smoothed non-PPP all health spending, as shown in (13), to calculate ρ (0.47).

$$\rho = \text{med}\left[\frac{\left(\frac{PPP_{mal}}{DAH_{mal}+GHES_{mal}+OOP_{mal}}\right)}{\left(\frac{PPP}{DAH+GHES+OOP}\right)}\right] \qquad (13)$$

In order to estimate PPP spending on malaria, we re-arranged terms and applied ρ to the estimated $DAH_{mal} + GHES_{mal} + OOP_{mal}$ and total health terms, as shown in (14).

$$PPP_{mal} = \rho * \left(\frac{PPP}{DAH+GHES+OOP}\right) * (DAH_{mal} + GHES_{mal} + OOP_{mal}) \qquad (14)$$

S.4.6 Malaria elimination status of the 106 countries

Table S4.11: Malaria elimination status by country

Elimination status in 2016	Countries
Control	Afghanistan<br>Angola<br>Benin<br>Bolivia<br>Brazil<br>Burkina Faso<br>Burundi

	Cameroon<br>Central African Republic<br>Chad<br>Colombia<br>Congo<br>Cote d'Ivoire<br>Democratic Republic of the Congo<br>Djibouti<br>Equatorial Guinea<br>Eritrea<br>Ethiopia<br>Gabon<br>Ghana<br>Guinea<br>Guinea-Bissau<br>Guyana<br>Kenya<br>Liberia<br>Madagascar<br>Malawi<br>Mali<br>Mauritania<br>Niger<br>Nigeria<br>Pakistan<br>Papua New Guinea<br>Peru<br>Rwanda<br>Senegal<br>Sierra Leone<br>Somalia<br>South Sudan<br>Sudan<br>Suriname<br>Tanzania<br>The Gambia<br>Togo<br>Uganda<br>Venezuela<br>Yemen
Eliminating	Bangladesh<br>Belize<br>Bhutan<br>Botswana<br>Cambodia<br>Cape Verde<br>China

	Comoros<br>Costa Rica<br>Dominican Republic<br>Ecuador<br>El Salvador<br>Guatemala<br>Haiti<br>Honduras<br>India<br>Indonesia<br>Iran<br>Laos<br>Malaysia<br>Mexico<br>Mozambique<br>Myanmar<br>Namibia<br>Nepal<br>Nicaragua<br>North Korea<br>Panama<br>Philippines<br>Sao Tome and Principe<br>Saudi Arabia<br>Solomon Islands<br>South Africa<br>South Korea<br>Swaziland<br>Thailand<br>Timor-Leste<br>Vanuatu<br>Vietnam<br>Zambia<br>Zimbabwe
Malaria-free	Algeria<br>Argentina<br>Armenia<br>Azerbaijan<br>Egypt<br>Georgia<br>Iraq<br>Kazakhstan<br>Kyrgyzstan<br>Morocco<br>Oman<br>Paraguay<br>Sri Lanka

	Syria<br>Tajikistan<br>Turkey<br>Turkmenistan<br>Uzbekistan

S4.7 Data density

Table S4.12: Availability of data by source and country elimination status

Source	Control	Eliminating	Malaria-Free	Total
**ACTwatch**	124	21		145
**Affordable Medicine Facility - Malaria**	127			127
**Battle, et al. (2016)**	1598	1394	544	3536
**Global Fund Concept Notes**	147	112	12	271
**Global Fund Funding Requests**	75	45	3	123
**Global Fund Price & Quality Reporting**	3135	973	47	4155
**Global Fund Proposals**	264	167	24	455
**Health Action International**	207	21	1	229
**Moses, et al. (2018)**	1692	1476	648	3816
**NHA**	193	32	1	226
**WHO Global Price Reporting Mechanism**	19165	3156	157	22478
**WMR**	2384	2493	683	5560
**Total**	29111	9890	2120	41121

Table S4.13: Availability of data by source and GBD super-region

Source	Central Europe, Eastern Europe, and Central Asia	High-income	Latin America and Caribbean	North Africa and Middle East	South Asia	Southeast Asia, East Asia, and Oceania	Sub-Saharan Africa	Total
ACTwatch						11	134	145
Affordable Medicine Facility - Malaria							127	127
Battle, et al. (2016)	238	68	646	374	170	510	1530	3536
Global Fund Concept Notes	4		28	10	18	56	155	271
Global Fund Funding Requests	3		3	3	15	15	84	123
Global Fund Price & Quality Reporting	37		66	166	240	517	3129	4155
Global Fund Proposals	24		51	30	32	76	242	455
Health Action International			3	22	4	15	185	229
Moses, et al. (2018)	288	72	684	432	180	540	1620	3816
NHA	1		3	2		9	211	226
WHO Global Price Reporting Mechanism	84		611	1337	365	2390	17691	22478
WMR	311	96	1178	472	311	914	2278	5560
Total	990	236	3273	2848	1335	5053	27386	41121

S4.7 References

1 Gakidou E, Afshin A, Abajobir AA, *et al.* Global, regional, and national comparative risk assessment of 84 behavioural, environmental and occupational, and metabolic risks or clusters of risks, 1990–2016: a systematic analysis for the Global Burden of Disease Study 2016. *The Lancet* 2017; 390: 1345–422.

2. World Health Organization (WHO). World Malaria Report. WHO; Geneva, Switzerland. Available at: https://www.who.int/malaria/publications/world_malaria_report/en/ (Accessed January 15, 2019).

3. WHO. Global Health Expenditure Database: Documentation Centre: National Reports. WHO; Geneva, Switzerland. Available at: http://apps.who.int/nha/database/DocumentationCentre/Index/en (Accessed January 15, 2019.

4. Moses MW, Pedroza P, Baral R, Bloom S, Brown J, Chapin A, Compton K, Eldrenkamp E, Fullman N, Mumford JE, Nandakumar V, Rosettie K, Sadat N, Shonka T, Flaxman A, Vos T, Murray CJL, Weaver MR. 2018. Funding and services needed to achieve universal health coverage: applications of global, regional, and national estimates of utilisation of outpatient visits and inpatient admissions from 1990 to 2016, and unit costs from 1995 to 2016. *The Lancet Public Health*.

5. Ayieko P, Akumu AO, Griffiths UK, English M. 2009. The economic burden of inpatient paediatric care in Kenya: household and provider costs for treatment of pneumonia, malaria and meningitis. *Cost-Effectiveness and Resoure Allocation.* 7:3.

6. Davis WA, Clarke PM, Siba PM, Karunajeewa HA, Davy C, Mueller I, Davis TME. 2011. Cost-effectiveness of artemisinin combination therapy for uncomplicated malaria in children: data from Papua New Guinea. *Bulletin of the World Health Organization*. 89(3):211-220.

7. Gogtay NJ, Kadam VS, Desai S, Kamtekar KD, Dalvi SS, Kshirsagar NA. 2003. A cost-effectiveness analysis of three antimalarial treatments for acute, uncomplicated Plasmodium falciparum malaria in Mumbai, India. *Journal of Association of Physicians of India.* 51:877-879.

8. Hongoro C, McPake B. 2003. Hospital costs of high-burden diseases: malaria and pulmonary tuberculosis in a high HIV prevalence context in Zimbabwe. *Tropical Medicine and International Health.* 8(3):242-50.

9. Kyaw SS, Drake T, Ruangveerayuth R, Chierakul W, White NJ, Newton PN, Lubell Y. 2014. Cost of treating inpatient falciparum malaria on the Thai-Myanmar border. *Malaria Journal.* 13:416.

10. Lubell Y, Riewpaiboon A, Dondorp AM, von Seidlein L, Mokuolu OA, Nansumba M, Gesase S, Kent A, Mtove G, Olaosebikan R *et al*. 2011. Cost-effectiveness of parenteral artesunate for treating children with severe malaria in sub-Saharan Africa. *Bulletin of the World Health Organization*. 89(7):504-512.

11. Maka DE, Chiabi A, Obadeyi B, Mah E, Nguefack S, Nana P, Mbacham W, Mbonda E. 2016. Economic evaluation of artesunate and three quinine regimens in the treatment of

severe malariain children at the Ebolowa Regional Hospital-Cameroon: a cost analysis. *Malaria Journal.* 15(1):587.

12. Muheki C, McIntyre D, Barnes KI. 2004. Artemisinin-based combination therapy reduces expenditure on malaria treatment in KwaZulu Natal, South Africa. *Tropical Medicine & International Health.* 9(9):959-966.

13. Rakuomi V, Okalebo F, Ndwigah S, Mbugua L. 2017. Cost effectiveness of pre-referral antimalarial treatment in severe malaria among children in sub-Saharan Africa. *Cost-effectiveness and Resource Allocation.* 15: 14.

14. Sicuri E, Vieta A, Lindner L, Constenla D, Sauboin C. 2013. The economic costs of malaria in children in three sub-Saharan countries: Ghana, Tanzania and Kenya. *Malaria Journal.* 12:307.

15. Svihrova V, Szilagyiova M, Krkoska D, Simekova K, Hudeckova H, Avdicova M. 2009. Analysis of the direct and indirect costs of treatment of imported malaria in the Slovak Republic. *Revista de Sociedade Brasiliera de Medicina Tropical.* 42(4):377-80.

16. Tang S, Feng D, Wang R, Ghose B, Hu T, Ji L, Wu T, Fu H, Huang Y, Feng Z. 2017. Economic burden of malaria inpatients during National Malaria Elimination Programme: estimation of hospitalization cost and its inter-province variation. *Malaria Journal.* 16(1):291. d

17. Tsakala TM, Tona GL, Mesia K, Mboma JC, Vangu JM, Voso SM, Kanja GL, Kodondi KK, Mabela M, Walo R. 2005. [Evaluation of prescriptions for inpatient treatment of malaria and gastroenteritis: Bondeko and St Joseph hospitals in Kinshasa]. Sante. 15(2):119-24.

18. James SL, Abate D, Abate KH, *et al.* Global, regional, and national incidence, prevalence, and years lived with disability for 354 diseases and injuries for 195 countries and territories, 1990–2017: a systematic analysis for the Global Burden of Disease Study 2017. *The Lancet* 2018; 392: 1789–858.

19. Battle KE, Bisanzio D, Gibson HS, Bhatt S, Cameron E, Weiss DJ, Mappin B, Dalrymple U, Howes RE, Hay SI, Gething PW. 2016. Treatment-seeking rates in malaria endemic countries. *Malaria Journal.* 15(20).

20. WHO. 2008. World malaria report 2008. WHO; Geneva, Switzerland. p. 215.

21. Deressa W. 2007. Treatment-seeking behaviour for febrile illness in an area of seasonal malaria transmission in rural Ethiopia. *Malaria Journal.* 6:49.

22. The Global Burden of Disease Financing Global Health Collaborator Network. 2019. Past, present, and future of global health financing: a review of development assistance, government, out-of-pocket, and other private spending on health for 195 countries, 1995-2050. *The Lancet.* Submitted Manuscript.

23. Moses MW, Pedroza P, Baral R, Bloom S, Brown J, Chapin A, Compton K, Eldrenkamp E, Fullman N, Mumford JE, Nandakumar V, Rosettie K, Sadat N, Shonka T, Flaxman A, Vos T, Murray CJL, Weaver MR. 2018. Funding and services needed to achieve universal health coverage: applications of global, regional, and national estimates of utilisation of outpatient visits

and inpatient admissions from 1990 to 2016, and unit costs from 1995 to 2016**.** *The Lancet Public Health*.

[24]. Chanda P, Masiye F, Chitah BM, Sipilanyambe N, Hawela M, Banda P, Okorosobo T. 2007. A cost-effectiveness analysis of artemether lumefantrine for treatment of uncomplicated malaria in Zambia. *Malaria Journal.* 6.

[25]. Davis WA, Clarke PM, Siba PM, Karunajeewa HA, Davy C, Mueller I, Davis TME. 2011. Cost-effectiveness of artemisinin combination therapy for uncomplicated malaria in children: data from Papua New Guinea. *Bulletin of the World Health Organization*. 89(3):211-220.

[26]. Ezenduka CC, Falleiros DR, Godman BB. 2017. Evaluating the Treatment Costs for Uncomplicated Malaria at a Public Healthcare Facility in Nigeria and the Implications. *Pharmacoeconomics.* 1(3):185-194.

[27]. Liu Y, Zhang YL, Gao LJ, Qian D, Yang CY, Zhou RM, Xu BL, Zhang HW. 2016. Medical Costs on Malaria in Henan Province and Analysis of the Influential Factors. *Zhongguo Ji Sheng Chong Xue Yu Ji Sheng Chong Bing Za Zhi.* (1):11-7.

[28]. Muheki C, McIntyre D, Barnes KI. 2004. Artemisinin-based combination therapy reduces expenditure on malaria treatment in KwaZulu Natal, South Africa. *Tropical Medicine & International Health.* 9(9):959-966.

[29]. Njau JD, Goodman CA, Kachur SP, Mulligan J, Munkondya JS, McHomvu N, Abdulla S, Bloland P, Mills A. 2008. The costs of introducing artemisinin-based combination therapy: evidence from district-wide implementation in rural Tanzania. *Malaria Journal.* 7.

[30]. Salawu AT, Fawole OI, Dairo MD. 2016. Patronage and Cost of Malaria Treatment in Private Hospitals in Ibadan North L.G.A. South Western Nigeria. *Analysis of Ibadan Post-graduate Medicine.* 14(2):81-84.

[31]. Sicuri E, Vieta A, Lindner L, Constenla D, Sauboin C. 2013. The economic costs of malaria in children in three sub-Saharan countries: Ghana, Tanzania and Kenya. *Malaria Journal.* 12:307.

[32]. Wiseman V, Kim M, Mutabingwa TK, Whitty CJM. 2006. Cost-effectiveness study of three antimalarial drug combinations in Tanzania. *Plos Medicine.* 3:1844-1850.

[33]. Institute for Health Metrics and Evaluation (IHME). 2018. Financing Global Health 2017:Funding Universal Health Coverage and the Unfinished HIV/AIDS Agenda. IHME; Seattle, WA. Available at: http://www.healthdata.org/policy-report/financing-global-health-2017 (Accessed January 16, 2019).

[34]. Castillo-Riquelme M, McIntyre D, Barnes K. 2008. Household burden of malaria in South Africa and Mozambique: is there a catastrophic impact? *Tropical Medicine and International Health.* 13:108–122.

[35]. Deressa W, Hailemariam D, Ali A. 2007. Economic costs of epidemic malaria to households in rural Ethiopia. *Tropical Medicine and International Health* 12(10):1148-56.

[36]. Gunda R, Shamu S, Chimbari MJ, Mukaratirwa S. 2017.Economic burden of malaria on rural households in Gwanda district, Zimbabwe. *African Journal of Primary Health Care and Family Medicine.* 9(1):e1-e6.

[37]. Hennessee I, Chinkhumba J, Briggs-Hagen M, Bauleni A, Shah MP, Chalira A, Moyo D, Dodoli W, Luhanga M, Sande J, Ali D, Gutman J, Lindblade KA, Njau J, Mathanga DP. 2017. Household costs among patients hospitalized with malaria: evidence from a national survey in Malawi, 2012. *Malaria Journal.* 16(1):395. doi: 10.1186/s12936-017-2038-y.

[38]. Ilunga-Ilunga F, Levêque A, Laokri S, Dramaix M. 2015. Incidence of catastrophic health expenditures for households: an example of medical attention for the treatment of severe childhood malaria in Kinshasa reference hospitals, Democratic Republic of Congo. *Journal of Infection and Public Health.* 8:136–144.

[39]. Nabyonga Orem J, Mugisha F, Okui AP, Musango L, Kirigia JM. 2013. Health care seeking patterns and determinants of out-of-pocket expenditure for malaria for the children under-five in Uganda. *Malaria Journal.* 12:175.

[40]. Onwujekwe O, Uguru N, Etiaba E, Chikezie I, Uzochukwu B, Adjagba A. 2013. The economic burden of malaria on households and the health system in Enugu State southeast Nigeria. *PLoS One.* 8(11):e78362.

[41]. Sicuri E, Vieta A, Lindner L, Constenla D, Sauboin C. 2013.The economic costs of malaria in children in three sub-Saharan countries: Ghana, Tanzania and Kenya. *Malaria Journal.* 12:307.

[42]. Xia S, Ma JX, Wang DQ, Li SZ, Rollinson D, Zhou SS, Zhou XN. 2016. Economic cost analysis of malaria case management at the household level during the malaria elimination phase in The People's Republic of China. *Infectious Diseases of Poverty.* 5(1):50.

[43]. Castellani J, Mihaylova B, Siribié M, Gansane Z, Ouerdraogo AZ, Fouque F, Sirima SB, Evers SMAA, Paulus ATG, Gomes M. 2018. Household costs and time to treatment for children with severe febrile illness in rural Burkina Faso: the role of rectal artesunate. *Malaria Journal.* 17: 380.

[44]. Castillo-Riquelme M, McIntyre D, Barnes K. 2008. Household burden of malaria in South Africa and Mozambique: is there a catastrophic impact? *Tropical Medicine and International Health.* 1:108-22.

[45]. Chuma J, Okungu V, Molyneux C. 2010. The economic costs of malaria in four Kenyan districts: do household costs differ by disease endemicity? *Malaria Journal.* 2 (9): 149.

[46]. Ewing VL, Lalloo DG, Phiri KS, Roca-Feltrer A, Mangham LJ, SanJoaquin MA. 2011. Seasonal and geographic differences in treatment-seeking and household cost of febrile illness among children in Malawi. *Malaria Journal.* 10:32.

[47]. Matovu F, Nanyiti A, Rutebemberwa E. 2014. Household health care-seeking costs: experiences from a randomized, controlled trial of community-based malaria and pneumonia treatment among under-fives in eastern Uganda. *Malaria Journal.* 13:222.

[48]. Morel CM, Thang ND, Xa NX, Hung le X, Thuan le K, Van Ky P, Erhart A, Mills AJ, D'Alessandro U. 2008. The economic burden of malaria on the household in south-central Vietnam. *Malaria Journal.* 7:166.

[49]. Mota RE, Lara AM, Kunkwenzu ED, Lalloo DG. 2009. Health seeking behavior after fever onset in a malaria-endemic area of Malawi. *American Journal of Tropical Medicine and Hygiene.* 81(6):935-43.

[50]. Mugisha F, Kouyate B, Gbangou A, Sauerborn R. 2002. Examining out-of-pocket expenditure on health care in Nouna, Burkina Faso: implications for health policy. *Tropical Medicine and International Health.*7(2):187-96.

[51]. Mustafa MH, Babiker MA. 2007. Economic cost of malaria on households during a transmission season in Khartoum State, Sudan. *Eastern Mediterranean Health Journal.* 13(6):1298-307.

[52]. Nabyonga Orem J, Mugisha F, Okui AP, Musango L, Kirigia JM. 2013. Health care seeking patterns and determinants of out-of-pocket expenditure for malaria for the children under-five in Uganda. *Malaria Journal.* 12:175.

[53]. Onwujekwe O, Uguru N, Etiaba E, Chikezie I, Uzochukwu B, Adjagba A. 2013.The economic burden of malaria on households and the health system in Enugu State southeast Nigeria. *PLoS One.* 8(11):e78362.

[54]. Xia S, Ma JX, Wang DQ, Li SZ, Rollinson D, Zhou SS, Zhou XN. 2016. Economic cost analysis of malaria case management at the household level during the malaria elimination phase in The People's Republic of China. *Infectious Disease of Poverty.* 5(1):50.

[55]. ACTwatch. News & Publications. Available at: http://www.actwatch.info/publications (Accessed January 16, 2019).

[56]. Health Action International (HAI). Price & Availability Data: Database of medicine prices, availability, affordability and price components. HAI. Available at: http://haiweb.org/what-we-do/price-availability-affordability/price-availability-data/ (Accessed January 16, 2019).

[57]. Arnold F, Ye Y, Ren R, Yoder S, Hanson K, Goodman C, Tougher S, Mann A, Willey B. 2012. Independent Evaluation of Phase 1 of the Affordable Medicines Facility - malaria (AMFm): multi-country independent evaluation report: final report. *The Global Fund to Fight AIDS, Tuberculosis and Malaria.* Available at: https://unitaid.org/assets/Mid-term-evaluation-Affordable-medicines-for-malaria-facility-AMFm-Phase-1.pdf. (Accessed January 18, 2019).

[58]. Management Sciences for Health (MSH). 2018. International Medical Products Price Guide. Available at: http://mshpriceguide.org/en/home/ (Accessed January 16, 2019).

[59]. WHO. Global Price Reporting Mechanism for HIV, tuberculosis and malaria. WHO; Geneva, Switzerland. Available at: https://www.who.int/hiv/amds/gprm/en/ (Accessed January 16, 2019).

[60]. The Global Fund to Fight AIDS, Tuberculosis and Malaria. Price & Quality Reporting. The Global Fund; Geneva, Switzerland. Available at: https://www.theglobalfund.org/en/sourcing-management/price-quality-reporting/ (Accessed January 16, 2019).

[61]. Management Sciences for Health (MSH). 2018. International Medical Products Price Guide.

Available at: http://mshpriceguide.org/en/home/ (Accessed January 16, 2019).

[62]. Based on an analysis of: Management Sciences for Health (MSH). 2018. International Medical Products Price Guide. Available at: http://mshpriceguide.org/en/home/ (Accessed January 16, 2019).

[63]. Bennett A, Bisanzio D, Yukich JO, Mappin B, Fergus CA, Lynch M, Cibulskis RE, Bhatt S, Weiss DJ, Cameron E, Gething PW, Eisele TP. 2017. Population coverage of artemisinin-based combination treatment in children younger than 5 years with fever and Plasmodium falciparum infection in Africa, 2003–2015: a modelling study using data from national surveys. *The Lancet Global Health.* 5:e418-27.

[64]. Global Burden of Disease Health Financing Collaborator Network. Past, present, and future of global health financing: a review of development assistance, government, out-of-pocket, and other private spending on health for 195 countries, 1995-2050. *The Lancet*; Submitted Manuscript.

SECTION 5. TRACKING POTENTIAL FOR ADDITIONAL GOVERNMENT SPENDING ON HIV/AIDS IN 135 LOW- AND MIDDLE-INCOME COUNTRIES, 2000 THROUGH 2017

S5.1 Acronyms and abbreviations

BIC, Bayesian information criteria
DAH-HIV/AIDS, Development assistance for health for HIV/AIDS
GAM, Global AIDS Monitoring
GARPR, Global Aids Response Progress Reports
HAQI, Healthcare access and quality index
HIV/AIDS, Human immunodeficiency virus, acquired immunodeficiency syndrome
LCU, Local currency units
LDI, 10-year lag distributed GDP per capita
NASAs, National AIDS Spending Assessments
NHA, National Health Accounts
PPP, Purchasing price parity
ST-GPR, Spatiotemporal Gaussian Process Regression
THE, Total health expenditure
UNAIDS, the Joint United Nations Programme on HIV/AIDS
USD, United states dollar

S5.2 Overview

In this study, we were interested in extracting data providing information on HIV/AIDS spending at the country-level by domestic financing source (domestic, government, private, prepaid private and out-of-pocket (OOP)) and domestic spending by spending category (care and treatment, prevention, and other). We extracted publicly available data from websites of international institutions and government data aggregators. HIV/AIDS spending data were extracted from seven sources:

- AIDSinfo database published by the Joint United Nations Programme on HIV/AIDS (UNAIDS)
- UNAIDS Global Aids Response Progress Reports (GARPR)
- UNAIDS health financing dashboard database (includes data from GARPR and Global Aids Monitoring (GAM))
- Government and private spending data reported by countries in proposals, concept notes, and funding requests submitted to the Global Fund to Fight AIDS, Tuberculosis and Malaria (the Global Fund)
- National Health Accounts that capture HIV/AIDS spending, including sub-accounts and accounts that adhere to the System of Health Accounts 2011 (SHA 2011) methodology
- National AIDS Spending Assessments (NASAs)
- Asia Pacific region data downloaded from the AIDS data hub

We leveraged the unique strengths across the different datasets, with the understanding that they were all generated to serve different purposes. The financing data collated by UNAIDS is sourced from annual reporting by countries to UNAIDS, in line with the 2000 Declaration on Commitment to HIV/AIDS.

Similarly, countries report domestic spending in proposals, concept notes, and funding requests submitted to the Global Fund to secure funding. The Global Fund requires countries submit these estimates as part of a requirement that they contribute funds to the disease area of focus, in addition to Global Fund contributions. Staff at both the Global Fund and UNAIDS verify the data submitted to them, but in general do not publish data that has been altered from what countries themselves report.

Not all extracted data sources used the same definition of health expenditures. For example, National AIDS Spending Assessment's (NASAs) definition of health expenditure on HIV/AIDS followed a broader definition than the definition of health expenditure provided by National Health Accounts (NHAs). Specifically, NASAs included expenditure on non-health spending categories such as orphans and vulnerable children, creation of an enabling environment, and other social protection services.

To harmonize the definition of HIV/AIDS related health expenditure amongst data sources, when provided, we subtracted expenditure related to orphans and vulnerable children, creation of an enabling environment, and social protection services, from the respective sources and functions of health expenditure reported in the NASAs. The three spending categories of orphans and vulnerable children, creation of an enabling environment, and social protection do not represent an exhaustive list of the deviations between NASAs and NHAs' HIV/AIDS spending definition, but do represent the vast majority of this deviation. Other spending categories that were included in NASAs but not included in NHAs were more granular and frequently not reported. Finally, in inspecting some of our data, we noticed the presence of duplicates. This was because many of our data sources drew from the same underlying data (AIDSinfo, UNAIDs' health financing dashboard, NASAs, GARPR). If multiple sources reported the same spending value for the same country, year, and financing source or health function, we removed all but one of these data points.

We extracted a total of 8,065 unique data points from 2000 to 2017. Data for government health spending on HIV/AIDS was most substantial, with more than 2,900 data points. Data was most sparse for out-of-pocket, prepaid private, and other domestic health function spending, for which there were 239, 280, and 329 data points, respectively. Table S5.1 provides breakdowns of the number of data points by year and quantity of interest.

Figure S5.1 captures the availability of HIV/AIDS spending data by country**.** Data density was highest in low- and middle-income countries with a large HIV/AIDS burden, notably Southern and Eastern sub-Saharan Africa; high-income countries had the fewest data points.

Table S5.1: Number of data points by year and prediction unit

Year	Domestic spending	Private spending	Out-of-pocket spending	Prepaid private spending	Government spending	Domestic other spending	Domestic care and treatment spending	Domestic prevention spending
2000	7	6	6	4	13	3	10	10
2001	24	7	2	2	14	2	9	9
2002	25	11	8	7	18	6	15	15
2003	26	10	3	3	19	5	9	11
2004	45	13	5	5	50	5	13	13
2005	57	34	10	13	101	12	30	32
2006	63	55	15	17	182	17	44	47
2007	57	68	16	22	233	16	59	59

2008	68	117	22	28	295	22	105	102
2009	68	124	21	25	289	27	115	117
2010	73	122	22	25	258	28	96	100
2011	60	105	17	22	211	22	77	75
2012	105	169	28	32	291	25	65	66
2013	115	133	20	21	259	20	62	61
2014	118	115	15	23	223	24	51	56
2015	101	101	13	14	226	35	50	49
2016	45	63	8	9	160	29	61	52
2017	20	56	8	8	132	31	55	57

Figure S5.1: Map of HIV/AIDS data availability

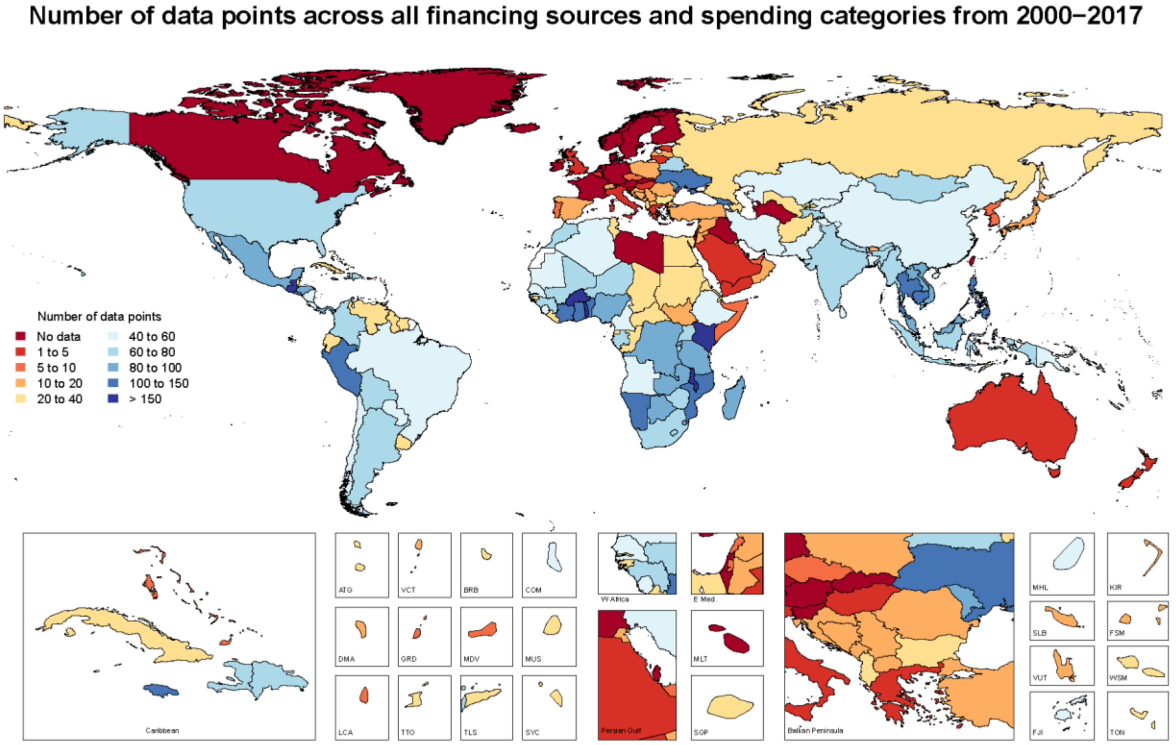

While the analysis presents on only domestic and government spending on HIV/AIDS in 135 low- and middle-income countries, we used data from high-income countries to inform and improve the overall fit of the models.

S5.3 Removing non-HIV focused spending from GARPR/GAM datasets

The most recent UNAIDS health financing dashboard dataset, which includes data from 2011-2018 from a number of GARPR and GAM reports, provided a detailed disaggregation of HIV and AIDS spending by health function (spending programme) and financing source. This allowed us to remove non-health spending from our estimates of total health spending on HIV and refine the definition of government spending on HIV. The spending we removed is on allied sectors, but does not meet the definition of

health spending defined by the System of Health Accounts. We removed spending on gender programmes, programmes for children and adolescents, social protection, synergies with development sectors, and tuberculosis from our estimates of total HIV spending. Furthermore, we removed spending sourced from reimbursable development bank loans from our estimates of total government spending on HIV. The specific mapping of GARPR/GAM HIV and AIDS programmes to our health function categories is presented below in Table S5.2.

We also used this dataset, which includes the disaggregation to these granular health programs, to adjust previously published GARPR/GAM datasets which did not include this level of detail. This process allowed us to remove what we expect to be the proportion of non-health spending and development bank loans from older, more aggregated GARPR/GAM datasets. We first calculated the fraction of health spending relative to total spending captured in the GARPR/GAM database using the granular data from the newest dataset. We calculated this ratio for each health function and financing source category, and then applied this ratio to older GARPR/GAM data which did not include this level of detail, using the country-level ratio when possible. When a country-level ratio was not available, we used the average ratio by health function and financing source. On average, this resulted in a 5% reduction of the previous GARPR/GAM database estimates. The main limitation of this approach is that we assume that the cross-year average proportion of non-health spending for each country and spending component is consistent between the new and old GARPR/GAM databases.

Table S5.2: Mapping GARPR and GAM spending programmes to health function categories

HIV and AIDS Programme	Health Function
1 Treatment, care and support	Curative Care
2 Prevention of vertical transmission of HIV (sub total)	Prevention
3 Prevention	Prevention
4 Gender programmes	Not included
5 Programmes for children and adolescents	Not included
6 Social protection	Not included
7 Community mobilization	Other health function
8 Governance and sustainability	Other health function
9 Critical enablers	Other health function
10 TB / HIV co infection, diagnosis and treatment (sub total)	Not included
1. Prevention of sexual transmission of HIV	Prevention
2. HIV prevention for people who inject drugs	Prevention
3. Prevention of mother to child transmission	Prevention
4. Universal access to treatment	Curative Care
5. TB	Not included
6. Governance and sustainability	Other health function
7. Critical enablers	Other health function
8. Synergies with development sectors	Not included

S5.4 Currency conversion

All HIV/AIDS expenditure estimates are presented in 2019 United States dollars), with the exception of Venezuela, whose expenditure was reported in 2014 USD. Data sources reported spending in either nominal local currency units (LCUs) or nominal United States Dollars (USD). To convert nominal LCUs to United States dollars, we applied deflators to nominal LCU to inflate to 2019 LCUs. We then applied exchange rates to produce 2019 United States dollars. When LCUs were not reported, we extracted reported expenditure in nominal USD, applied corresponding nominal exchange rates to produce nominal LCUs, inflated nominal LCUs to 2019 LCUs with deflators, and finally exchanged 2019 LCUs to 2019 United States dollars. More information can be found in Section 2 of this appendix.

S5.5 Modeling HIV/AIDS Spending with ST-GPR

As previously mentioned in the manuscript, we used spatiotemporal Gaussian process regression (ST-GPR) to model HIV/AIDS spending for five financing source models (domestic, private, government, prepaid private, and OOP) and three domestic spending category models (prevention, care and treatment, and other). ST-GPR generates a complete time-series of data for a set of locations, follows the data where it is available and borrows strength across time, geographic regions, and covariates' predictive power when data is not available. The interested reader may view a complete description of ST-GPR here[7]. ST-GPR has three primary steps. First, a linear mixed effects model is run with a given set of predictors. Predictions from the first step provide the general trend within the data. In the second step, spatiotemporal patterns are estimated by applying a series of spatiotemporal weights to average the residuals of the first step linear model. These spatiotemporal patterns are then added to the linear predictions to generate spatiotemporally smoothed predictions. Finally, the smoothed predictions are passed as the mean function to a Gaussian process regression along with the data to produce final ST-GPR predictions. For every country-year, 1,000 draws were generated from the Gaussian process regression model and used in subsequent calculations.

For the first step of ST-GPR—the linear model—we used a linear mixed effects regression with random effects on Global burden of disease (GBD) super region and GBD region. The exact set of fixed-effect covariates for each model is presented in Table S5.3. All covariates were sourced from GBD 2017[8,9]. Within the linear model step, we measured each data point's cook's distance, and after checking to ensure the data was properly extracted, we excluded every data point with a cook's distance exceeding 4/n, where n is the total number of data points within the model. Additionally, we removed several data points by visual inspection; these are listed in Table S5.4 below.

Table S5.3: Covariates used in ST-GPR

ST-GPR model	Covariates
Domestic	Lag distributed income per capita, ART price, HIV/AIDS prevalence
Government	Lag distributed income per capita, ART price, HIV prevalence
Private	Lag distributed income per capita, HIV/AIDS prevalence
OOP	Lag distributed income per capita, HIV/AIDS prevalence

Prepaid private	Lag distributed income per capita, ART price, HIV prevalence
Domestic HIV/AIDS spending on care and treatment	Lag distributed income per capita, ART price, HIV/AIDS prevalence
Domestic HIV/AIDS spending on prevention	Lag distributed income per capita, ART price, HIV/AIDS prevalence
Domestic HIV/AIDS spending on all other functions	Lag distributed income per capita, ART price, HIV/AIDS prevalence

Table S5.4: HIV data points removed by visual inspection

Country	Year	Component	Source Type	Value (2019 USD)
Comoros	2011	Government Spending	UNAIDS health financing dashboard database	821,723.40
The Gambia	2014	Government Spending	UNAIDS health financing dashboard database	4,107,382.70
The Gambia	2014	Government Spending	AIDSinfo database	4,107,382.30
Cote d'Ivoire	2009	Private Spending	National Health Account	18,864,402.10
Cote d'Ivoire	2010	Private Spending	National Health Account	20,271,147.20
Guinea	2013	Private Spending	National Health Account	13,430,795.40

S5.6 Enforcing internal consistency

To ensure internal consistency between the HIV/AIDS spending estimates and the all health spending estimates[6], HIV/AIDS spending by source was modeled as the logit transformed fraction of the respective, loess smoothed, all health spending by financing source estimate (e.g. domestic HIV/AIDS spending divided by all domestic health spending). As a consistency check, extracted data points were outliered if the fraction of HIV/AIDS spending by source and all health spending by source exceeded one.

While the above transformation helped ensure internal consistency between HIV/AIDS spending and all health spending, we were still required to ensure internal consistency within our estimates such that government HIV/AIDS spending did not exceed domestic HIV/AIDS spending, and further we wished to take advantage of all the extracted data within the implemented models. These objectives were accomplished by both aggregating and raking. Aggregating is the process of summing mutually exclusive and collectively exhaustive estimates of sub-components of health expenditure (e.g. OOP, prepaid private, government spending, development assistance) and using the sum as the estimate of total health expenditure. Raking is the exact opposite of aggregating. In raking, we used estimates of total health expenditure to evenly scale the estimated sub-components to ensure the sub-components sum to the estimated total health expenditure. Raking and aggregating are equally valid and widely used in health financing and in the Global Burden of Disease[8,9].

In our extracted dataset, few data sources (NHAs and NASAs) reported OOP, prepaid private, or total HIV/AIDS spending (sum of OOP, prepaid private, government, DAH-HIV/AIDS), while nearly all data sources reported expenditure of either government, private (sum of OOP and prepaid private, but not disaggregated), and total domestic (sum of government and private, but not disaggregated) HIV/AIDS spending. Given this inconsistency, we modeled the five financing source spending variables and raked and aggregated estimates to draw strength across areas with the highest data density. This process was implemented by averaging the domestic HIV/AIDS spending estimates with the aggregated domestic HIV/AIDS spending estimate formed by summing estimates of government and private HIV/AIDS spending. This averaged result represented our final estimate of domestic HIV/AIDS spending. We then raked estimates of government and private HIV/AIDS spending to the final domestic HIV/AIDS spending envelope to produced final private and government HIV/AIDS spending estimates. The final private HIV/AIDS spending estimates were then used as an envelope to rake OOP and prepaid private HIV/AIDS spending estimates. To propagate uncertainty, we conducted both aggregating and raking on the draw level. For estimates of total HIV/AIDS spending, we deterministically added development assistance, source from published literature[6], to total domestic HIV/AIDS spending

To generate estimate of domestic HIV/AIDS spending by spending category (prevention, care and treatment, and other), we estimated domestic spending by spending category as the logit transformed fraction of total domestic spending. Not only did these models draw on reported domestic spending by spending category, but they drew on data reporting total spending by spending category. We equated these latter data to domestic spending by spending category by subtracting estimates of development assistance from analogous spending category. This process required us to map development assistance by HIV/AIDS health focus area to our spending categories. This mapping is presented in Table S5.5. If this process of equating data resulted a negative value, after checking the data point of incorrect extraction, we dropped the data point.

Estimates of domestic spending by spend category were raked to the total domestic spending envelope. Final estimates of HIV/AIDS total spending by spending category (i.e. total HIV/AIDS spending and total HIV/AIDS spending for each health function) were created by deterministically adding domestic spending to development assistance by spending category.

Table S5.5: Mapping DAH focus areas to HIV/AIDS spending functions

HIV/AIDS spending functions	Development assistance for HIV/AIDS by health focus area.
Prevention	Prevention, PMTCT
Care and treatment	Treatment, Care, Counseling and Testing
Other	Health system strengthening
	Unidentified

S5.7 Development assistance for health

Development assistance for health (DAH) estimates were obtained from the Institute for Health Metrics and Evaluation's development assistance for health database. A more detailed description of the methodology used to obtain the estimates in the database can be found in Dieleman et al.[6] All known, systematically reported, available data on health-related disbursements and expenditures were extracted, as well as income and revenue from existing project databases, annual reports, and audited financial statements. DAH for bilateral agencies included all health-related disbursements from bilateral donor

agencies, excluding funds that they transferred to any of the other channels we tracked in order to avoid double-counting. This information was extracted from the Creditor Reporting System (CRS) and Development Assistance Committee (DAC) databases of the Development Assistance Committee of the Organisation for Economic Co-operation and Development (OECDDAC).

In some cases, donor agencies did not report disbursement data to the CRS. A method for predicting disbursements from commitment data was implemented to address this challenge. For other grant- and loan-making institutions, annual disbursements on health grants and loans were similarly included, excluding transfers to any other channels and ignoring any repayments on outstanding debts. The annual disbursements for grant- and loan-making institutions only reflect the financial transfers made by these agencies. Therefore, in-kind transfers from these institutions in the form of staff time for providing technical assistance and the costs of managing programs were estimated separately. Estimates of DAH for the United Nations (UN) agencies included annual expenditures on health both from their core budgets and from voluntary contributions. Non-governmental organizations' (NGOs) DAH estimates utilized data from US government sources, Guidestar Research Fundamental Plus dataset and a survey of health expenditure for a sample of NGOs to estimate DAH from US-based and internationally based NGOs receiving support from the US government. To allocate DAH to HIV/AIDS and the program areas within development assistance for HIV/AIDS, we used a keyword search and tagged spending based on a weighted-average of keywords. Extensive details about all the steps involved in estimating DAH are in Dieleman et al.

S5.8 References

1 World Development Indicators (WDI) | Data Catalog. https://datacatalog.worldbank.org/dataset/world-development-indicators (accessed Jan 31, 2019).

2 World Economic Outlook Database October 2018. https://www.imf.org/external/pubs/ft/weo/2018/02/weodata/index.aspx (accessed Jan 31, 2019).

3 Robert C. Feenstra RI. Penn World Table 9.0. 2016. DOI:10.15141/S5J01T.

4 National Accounts - Analysis of Main Aggregates (AMA). https://unstats.un.org/unsd/snaama/Index (accessed Jan 31, 2019).

5 WHO | Global Health Observatory (GHO) data. WHO. http://www.who.int/gho/en/ (accessed Jan 31, 2019).

6 Global Burden of Disease Health Financing Collaborator Network. Past, present, and future of global health financing: a review of development assistance, government, out-of-pocket, and other private spending on health for 195 countries, 1995-2050. *The Lancet*; **Submitted Manuscript**.

7 Gakidou E, Afshin A, Abajobir AA, *et al.* Global, regional, and national comparative risk assessment of 84 behavioural, environmental and occupational, and metabolic risks or clusters of risks, 1990–2016: a systematic analysis for the Global Burden of Disease Study 2016. *The Lancet* 2017; **390**: 1345–422.

8 Dicker D, Nguyen G, Abate D, *et al.* Global, regional, and national age-sex-specific mortality and life expectancy, 1950–2017: a systematic analysis for the Global Burden of Disease Study 2017. *The Lancet* 2018; **392**: 1684–735.

9 James SL, Abate D, Abate KH, *et al.* Global, regional, and national incidence, prevalence, and years lived with disability for 354 diseases and injuries for 195 countries and territories, 1990–2017: a systematic analysis for the Global Burden of Disease Study 2017. *The Lancet* 2018; **392**: 1789–858.

10 Hollingsworth B, Peacock S. Efficiency Measurement in Health and Health Care. Oxon: Routledge.

11 Remme M, Siapka M, Sterck O, Ncube M, Watts C, Vassall A. Financing the HIV response in sub-Saharan Africa from domestic sources: Moving beyond a normative approach. *Soc Sci Med 1982* 2016; **169**: 66–76.

Forecasting Health Spending from 2018 through 2050

Supplementary Appendix

Global Burden of Disease Financing Global Health Collaborator Network

Updated: Friday 21st February, 2020

Contents

S6.1 Data 2
S6.1.1 Summary of Data Sources 2
S6.1.2 World Health Organization's Global Health Expenditure Database 2
S6.1.3 WB WDI, IMF WEO, PWT, and Maddison 3
S6.1.4 Institute for Health Metrics and Evaluation's Development Assistance for Health Database 4
S6.1.5 Institute for Health Metrics and Evaluation's Global Burden of Disease Study 2017 4

S6.2 Covariates 6
S6.2.1 List of Forecasted Variables 6
S6.2.2 Covariates Used for Forecasting 6

S6.3 Ensemble Modeling 8
S6.3.1 Sub-model Setup 8
S6.3.2 Covariates 8
S6.3.3 Convergence to Global Growth Rates 8
S6.3.4 Specifications 9

S6.4 Inclusion and Exclusion Criteria 11

S6.5 Creating the Forecasts 12
S6.5.1 Ranking Sub-Models 12
S6.5.2 Uncertainty Estimation 12

S6.6 Ad-hoc Draws Correlation 14
S6.6.1 Motivation 14
S6.6.2 Bivariate Correlated Distributions 14

S6.7 Future Health Scenarios 14
S6.7.1 Long-term Growth Regressions 15
S6.7.2 Forecasting Better and Worse Scenarios 15
S6.7.3 Uncertainty Estimation 16

S6.8 Package and Architecture 17
S6.8.1 Architecture 17
S6.8.2 Template Model Builder 17

References **18**

S6.9 Tables and Figures **20**

Introduction

The objective of this study is to provide data on future health spending patterns that can guide decision-makers. These analyses produced comprehensive and comparable set of gross domestic product and all-sector government spending estimates, followed by all the components of a country's total health expenditure. Additionally, using observed past trends, we predicted the possible trajectories of the covariates of interest conditional on following an optimistic and pessimistic pattern based on global rates of change.

The purpose of this appendix is to describe in detail the methodology used in our analyses. Subsequent sections contain information on all data sources, ensemble forecasting strategies, inclusion criteria, and uncertainty estimation used to generate our estimates, as well as our guidelines on how our future health scenarios analyses were conducted.

SECTION 6. FORECASTING HEALTH SPENDING FROM 2018 through 2050

S6.1 Data

S6.1.1 Summary of Data Sources

We used data from seven sources for the analyses:

- World Health Organization's (WHO) Global Health Expenditure Database
- World Bank (WB) World Development Indicators database (WDI)
- International Monetary Fund's (IMF) World Economic Outlook (WEO) October 2019 database
- Penn World Tables 9.1 (PWT)
- Angus Maddison Project Database 2018
- Institute for Health Metrics and Evaluation's Development Assistance for Health Database 2019 (IHME)
- Institute for Health Metrics and Evaluation's Global Burden of Disease Study 2017 (IHME GBD 2017)

Specifically, we collected health expenditure information on all available sources that is comparable across countries and complete for most countries from WHO and IHME, and demographic data from the WPP, while the underlying data for producing gross domestic product (GDP) and general government expenditure (GGE) were extracted from the IMF, WB, and PWT. Table S6.1 presents the definitions for the various health expenditure sources.

S6.1.2 World Health Organization's Global Health Expenditure Database

We used Global Health Expenditure Database (GHED) data from the WHO to generate our estimates, which spans 191 countries between 2000 and 2017 (in the January 7th, 2020 version). From the GHED, we pulled the following variables:

(i) Compulsory prepayment (Other, and unspecified, than FS.3)

(ii) Other revenues from NPISH n.e.c.

(iii) Other revenues from corporations n.e.c.

(iv) Other revenues from households n.e.c.

(v) Social insurance contributions

(vi) Transfers from government domestic revenue (allocated to health purposes)

(vii) Gross Domestic Product

(viii) Voluntary prepayment

To ensure we were using the best possible data, we downloaded the metadata for each data point for all of the indicators from the GHED website. We used the metadata to decide how each given data point should be weighted, from 1 to 5, being applied as inverse variance weights. We established guidelines for the metadata that informed how the underlying data points should be weighted, giving priority to factors such as complete, documented source information and penalizing factors such as having been derived or estimated. We adjusted these data by converting them from current local currency to 2019 US dollars. Details of the weighting guidelines and classification are explained in the supplementary appendix our retrospective analysis paper.

Once we have an incomplete set of data points for the health expenditure variables we are interested in forecasting, we used Spatiotemporal Gaussian process regression (ST-GPR) to model the full time-series for each variable across a total of 195 countries. ST-GPR is a stochastic modeling technique that is designed to detect signals amidst noisy data. Unlike classical linear models that assume that the trend underlying data follows a definitive functional form, GPR assumes that the specific trend of interest follows a Gaussian Process, existing with some pointwise mean and covariance function [1]. The covariates that were used in order to determine the initial fit of our health expenditure variables are: lag-distributed income, all-sector government expenditure per capita, and proportion of total population over the age of 64; using a Matérn covariance function for the distribution of the Gaussian process. Once we determined an initial prediction of our dependent variables, using the variability of data across regions, Gaussian process regressions (GPR) were run in order to estimate 1,000 draws of each country-year estimate per metric.

S6.1.3 WB WDI, IMF WEO, PWT, and Maddison

The WDI Database provides data on a wide range of development related variables, including data on GDP and GDP per capita. Data series in this database begin in 1960 and span

through the present. The IMF's WEO Database provides data on various macroeconomic indicators. Macroeconomic series data are available from 1980 to present. The PWT is a database that provides real national accounts data for 182 countries and territories from 1950 to 2017. The Maddison Project database provides historical GDP, GDP per capita, and population data dating as far back as Roman times. We utilized GDP per capita as a primary covariate to produce forecasts. GDP per capita from 1950 through 2020 was constructed using the method described in *James et al* [2]. The method utilized extracted data from a number of sources (IMF, WB, PWT and Maddison), and used multiple random effects models to estimate a mean GDP per capita series to be used in our analysis. Similarly, we used the same methodology to produce a mean general government expenditure (GGE) per GDP series, from 1980 through 2019.

S6.1.4 Institute for Health Metrics and Evaluation's Development Assistance for Health Database

Development assistance for health estimates were obtained from the Institute for Health Metrics and Evaluation's Development Assistance for Health Database. To generate these estimates, IHME collected audited budgets, annual reports, and project records from the primary development agencies providing assistance for the health sector. These records are augmented by information acquired via correspondence, and are standardized and compiled to provide a comprehensive perspective on international financial flows for health. These estimates are tracked backward to the source of the funds and forward to the country recipient, and are available from 1990 through 2019 and 2017, respectively.

S6.1.5 Institute for Health Metrics and Evaluation's Global Burden of Disease Study 2017

Covariates used in forecasting health spending, such as populations and educational attainment per capita [3, 4], were obtained from the Institute for Health Metrics and Evaluation's Global Burden of Disease Study 2017. Population forecasts used were from IHME's Future Health Scenarios work, which uses a cohort component method of projection (CCMP [5]), and takes forecasts of age-specific fertility rates, mortality, migration, and the sex ratio at birth as inputs [6]. Age-specific fertility forecasts were projected by modeling completed cohort fertility as a function of met need for contraception and maternal education. Completed cohort fertility forecasts were then converted back to age-specific fertility rates. Net migration rates were forecasted as a function of the sociodemographic index, the crude popu-

lation growth rate, and mortality due to conflict, war and natural disasters. Total (all-cause) mortality was derived from forecasting cause-specific mortality from GBD 2017, using the three-component model described in Foreman et al [4]. Cause-specific mortality was then aggregated up the GBD 2017 cause hierarchy to generate total mortality forecasts, which were utilized to compute future life tables. Using past population from GBD 2017, the CCMP model advanced populations forward and produced country-age-sex specific estimates for single future calendar years.

S6.2 Covariates

S6.2.1 List of Forecasted Variables

The following are the list of variables which are forecasted in the manuscript:

- GDP: Gross Domestic Product (national income of a country)
- GGE: General Government Expenditure (all sector government expenditure in a country)
- DAH_d: Development Assistance for Health donated
- DAH_r: Development Assistance for Health received
- GHE: Government Health Expenditure
- OOP: Out-of-pocket Private Expenditure
- PPP: Prepaid Private Expenditure
- THE: Total Health Expenditure

S6.2.2 Covariates Used for Forecasting

The following covariates are used as predictors, or independent variables, in our models:

- Total population of a country (1950 - 2050)
- Proportion of total population below the age of 15 (1950 - 2050)
- Proportion of total population above the age of 64 (1950 - 2050)
- Educational attainment per capita (1980 - 2050)
- An indicator variable used to denote the anomalous disbursement of DAH in our DAH_d forecasts (1 for years in 2000 - 2010, 0 otherwise)
- An indicator variable used to denote the Ebola crisis in Guinea, Sierra Leone and Liberia in our DAH_r forecasts (1 for years in 2014 and 2015 for the three aforementioned countries, 0 otherwise).

Additionally, we also use the forecasted GDP and GGE per capita as covariates to predict the health expenditure variables. Figure S6.1 in the Tables and Figures section shows our full pathway for forecasting all our endogenous variables and how each of those variable fed into a succeeding model.

In order to forecast GDP per capita, we first convert GDP per capita to GDP per working population in the following manner:

$$\text{GDP}_{\text{working population}} = \text{GDP}_{\text{total population}} \times \frac{\text{Working population (20-64 years old)}}{\text{Total Population}}$$

We forecast GDP per working population from 2021-2050 and convert back to GDP per capita for further analysis. The out-of-sample root-mean-squared-error (RMSE) based on using GDP per working population was smaller than forecasting with GDP per capita.

S6.3 Ensemble Modeling

The purpose of ensemble modeling is to make sure that we capture the most out of what we have in our arsenal in terms of covariates and model specifications. We are agnostic about one model being the sole predictor of the future, and allow an ensemble of beliefs about predicting off of the past trends. 'Ensembling', in simple terms, is a way of pooling a number of sub-models, where the space of sub-models span different inclusions and combinations of predictors, and/or different econometric specifications.

S6.3.1 Sub-model Setup

Our basic sub-model is a linear mixed effect model of the following form, for country i and time t:

$$\Delta_t Y_{i,t} = (\alpha + \alpha_i) + \left(\sum_{p=1}^{3} \rho_p \Delta_t Y_{i,t-p} \right) + \Delta_t X' \beta + \varepsilon_{i,t} \tag{1}$$

where

$$\left(\alpha, \vec{\beta}\right) \qquad \text{(Fixed effects)} \tag{2}$$

$$\alpha_i \sim N(0, \sigma_a^2) \qquad \text{(Country specific random intercept)} \tag{3}$$

$$\varepsilon_{i,t} \sim N\left(\sum_{m=1}^{3} \phi_m \varepsilon_{i,t-m}, \sigma_i^2 \right) \qquad \text{(Autocorrelated residuals as time random effects)} \tag{4}$$

'Fixed effect' is the equivalent of a non-random coefficient in a linear regression setting, and is estimated globally across all countries and time periods. The likelihood function for the data were all set to be Gaussian distributions for all of our metrics and models.

S6.3.2 Covariates

Using our linear baseline model as defined in section S6.3.1, we created our set of ensemble sub-models by using all combinations of each of the covariates in Table S6.2. For example, if we were predicting GGE per GDP with Educational attainment and Population as the predictors, then we would get a possible combination of four specifications to use (including one with no fixed effects). All covariates were included as fixed effects, and all specifications had the random effect on country intercepts. The proportion of submodels used that contain particular submodel specifications are described in Table S6.3.

S6.3.3 Convergence to Global Growth Rates

We believe that the very-long-term trajectory of a variable forecasted for each country will gradually taper to the global growth rates. The way that we implemented this growth rate convergence is in the following manner:

1) Suppose that we are forecasting $Y_{i,t}$, which includes an intercept at the global level and the country level. Let us also assume that there were $\widetilde{T}$ time periods of Y observed in the past for each country i.

2) We start the tapering of the growth rate to start from 10 years before $\widetilde{T}$ into the future starting from the present, converging to the global value 10 years after $\widetilde{T}$. Therefore, if the present time period is H, then our decyaing scheme will start off from the year $h + \widetilde{T} - 10$ and end at $h + \widetilde{T} + 10$.

3) Our main objective is to slowly taper all the countries' random intercepts to decay to zero, so that they are all following a global intercept. In a first-difference model, since the intercepts represent the linear time trend, this scheme will slowly converge the linear time trends of all countries to decay to the global time trend.

4) Starting from the first year of decay, we will transform the country specific intercepts as such:
$$\hat{\alpha}_i = (\hat{\alpha}_i) \times \frac{20 - t}{20}$$
where t are all the time periods between $h + \widetilde{T} - 10$ and $h + \widetilde{T} + 10$.

5) By the time t reaches $h + \widetilde{T} + 10$, the value of $\hat{\alpha}_i$ will have decayed to zero, and therefore, all the countries will have reached a global linear time trend estimate.

S6.3.4 Specifications

1) **ARIMA (Autoregressive Integrated Moving Average) terms**: We allowed up to three degrees of lags in the model (traditional auto-regressive terms), where each degree of AR term will include itself and all other lower degrees of lags. For example, a GDP per capita model with AR(3) specification (predicting log of GDP per capita) will include once, twice and thrice lagged log GDP per capita term. These were included as fixed effects.

   Additionally, in order to predict the best set of fixed effect coefficients, we test and include auto-correlated residuals in our models (traditional moving-average terms in an ARIMA

setup). This basically means that we allow our models to estimate the residuals with an autoregressive process of their own. These were included as random effects, and we allowed this to exist at the country-year level.

All of the variables we forecasted used the first-differences transformation (across time) as the dependent variable, except DAH received which was forecasted in non-differenced space, because of the presence of very high noise, thereby making the differenced series very unstable to forecast.

2) **Recency weights**: One of the other specifications we included in our sub-models was the option of weighting the recent years higher. This is particularly helpful for countries like Ethiopia and Nigeria, where they had rapid economic growth in recent years, and we believe that is a better predictor of the GDP forecasts than the further past. The weighting function was defined as such:

$$Weight = f(\tau, t) = (T - t + 1)^{\tau} \tag{5}$$

where T is the final year of in-sample data we have, and t is the year at that data point. This is an exponential decaying weighting function, where the degree of decaying is determined by τ, and we test and include a set of values of τ ranging between 0 and 0.5, where $\tau = 0$ refers to equal weights (all time periods are weighed equally at 100). Given that $\left.\frac{df(\tau, t)}{dt}\right|_{\tau > 0} < 0$, this allows to weight our data with higher contributions to the likelihood from the recent past, and slowly decaying as we move further into the past towards the first observed time period. These recency weights were multiplied with the pointwise data variance parameter in order to weight the data points.

3) **Convergence Term**: We also allowed for the inclusion of a 'convergence term' in the list of sub-models. A convergence term is the one-year lag of the non-differenced dependent variable, and gets updated as each year is forecasted in the future. If a convergence term was considered in a sub-model, then we only included that sub-model if the coefficient on the convergence term was estimated to be negative (and statistically significant at 10% level).

S6.4 Inclusion and Exclusion Criteria

After we ran all possible combination of our sub-models and created a mean set of forecasts, we only want to keep the best possible set of sub-models. Hence, we implied the following set of inclusion and exclusion criteria in order to filter out the 'unrealistic' sub-models:

(1) All of the estimated coefficients must be **statistically significant at 10% level of significance**. For the fixed effects, we took the mean and the standard deviation of the posterior estimates, and filtered out the sub-model if the absolute z-score is below 1.645 (the absolute value of the one-sided 95th quantile of a standard normal distribution).

For the random effects, we look at the whether the measure of variance is statistically significant or not. The model outputs the mean and standard deviation of the *precision of the random effects*, and therefore allowed us to exclude the specification if the precisions are not statistically significant at 10% level.

(2) If there were any estimated coefficient that **defied a prior belief we have on the direction of the value**, then we dropped that sub-model from consideration. For example, we strongly believe that as a donor country's (high income countries) income (GDP per capita) grows, they will be able to donate more DAH to lower income countries, and so, if we ran a sub-model predicting DAH_d and get a negative coefficient on GDP per capita, we dropped that sub-model from consideration.

Our prior beliefs on the covariates for each dependent variable are listed in Table S6.2.

(3) The forecasted trajectory growth **must not exceed observed growth rates**. We believe that a country will not grow faster than how much it has grown in the past trend. In order to come up with the bounds, we run a stochastic frontier analysis (SFA) of the change in the predicted variable against the level value of the predicted variable. SFA is just like an ordinary least squares specification, except with the addition of an additional 'inefficiency' term with a half-normal distribution. This allows us to estimate (for example, for GDP per capita): conditional of a country's income, how much growth rate did we see in the country's GDP. We ran this analysis across all of the observed data points, and derived a relationship binding the growth rates of GDP against the absolute values of GDP.

S6.5 Creating the Forecasts

S6.5.1 Ranking Sub-Models

In order to find out with sub-models would be able to predict a country's future the best, we ran out-of-sample predictive validity (OOS-PV) tests [7]. Simply put: we took each sub-model that passed the criteria in section S6.4, and instead of running it on all of the past data, we left out some number of recent most years. Following that, we ran the sub-model on the truncated past, and forecasted those years left out. For example, our GDP data extends from 1970 through 2020; we left out 15 years of data, and reran a sub-model from 1970 - 2005, and use the results of that sub-model to forecast GDP for the out-of-sample years (2006 - 2020).

This gave us essentially two trajectories between 2006 through 2020: the truth and the out-of-sample predictions. For each year, we computed the squared error (the difference) between these two lines, and averaged these errors for each of the neighboring years. So for example, we had squared errors for 15 data points between 2006 and 2020, and so the first mean squared error sum was just the squared error at 2006 and 2007, the second was the mean of the sum of squared errors for 2006, 2007 and 2008, and so on. We then took the square root of this new series to get the running root mean squared errors (RMSE) for a given country.

We looked at a country's 2006 RMSE values for each sub-models ran, and listed out the best 10% of the sub-models (that is, the lowest 10% RMSE values), and we did so for every year out-of-sample. For a single country, we may potentially have completely different set of sub-models for each of the OOS years. Then, for the 10% of the sub-models selected in the *first* year OOS, we only used those models to predict the *first* year of forecast for each country; the set of 10% of the sub-models selected in the *second* year OOS were used to predict the *second* year of forecast for each country, and so on, until the last year OOS model selections are used to compute the forecast the remaining years. This allowed us to narrow down every country's trajectory with the best performing OOS-PV sub-models for each year.

S6.5.2 Uncertainty Estimation

To estimate the uncertainty intervals (UI), we reran the selected, ranked sub-models from section S6.5.1 and simulated draws instead of just getting a mean estimate of the future. There are four types of uncertainty we implemented in forecasting:

(i) **Model Uncertainty** : This type of uncertainty comes from having more than one

type of specification to create forecasts, and therefore we included a set of sub-models in the ensemble to incorporate for this uncertainty (which are ranked within each country-year).

(ii) **Data Uncertainty** : If our covariates themselves had draws of the future data (for example, when we forecasted GDP as a covariate to forecast PPP), then we picked randomly from the draws of the independent variable when predicting a sub-model's trajectory, if that covariate was included.

(iii) **Parameter Uncertainty** : This type of uncertainty is due to the variance for the posterior distributions. Once we have run a sub-model, we simulated from each of the estimated posterior distribution to create a set of simulated coefficients. This is done by simulating from the joint precision matrix of all the parameters estimated, and therefore produces a correlated set of coefficients.

(iv) **Fundamental Uncertainty** : The in-sample data and the fitted line in the past will never line up perfectly: there will always be errors from the model fit. We needed to reflect this level of uncertainty in our forecasts as well. By extracting these empirical residuals produced by a sub-model, we forecasted future country-specific residuals by using a random walk process, where the variance of the process is the variance of the residuals from the model fit σ_{ε}^2:

$$\hat{\varepsilon}_{i,t} \sim N(\varepsilon_{i,\hat{t}-1}, \sigma_{\varepsilon,t}^2) \tag{6}$$

A random walk is an AR(1) process with the coefficient equal to 1: in other words, the current value is independent of last year's value (except for the starting position), and will propagate forward with a random Gaussian noise of $\sigma_{\varepsilon,t}^2$ variance.

All of the above were used to simulate 1,000 forecasts (or draws), and so in order to construct our UIs, we took the mean and the values of the 2.5th and 97.5th quantiles to estimate 95% UIs.

Table S6.4 shows all of the retrospective and prospective estimates of our health spending outcomes in 2019 US dollars and 2019 purchasing-power parity-adjusted dollars in per capita space.

S6.6 Ad-hoc Draws Correlation

S6.6.1 Motivation

Given our current setup of compiling draws for each single year, there is no way of enforcing a temporal correlation across the draws right from the ensemble architecture. For example, the sub-models used in the first 15 years of GDP per capita forecasts were independently constructed and only depended on OOS-PV fits, while the 10th year (2036) onwards all draw from the same set of sub-models. This section details on the method used to generate the same correlation in the first 10 years of that example, drawing from the existing correlation from the 10th year onwards.

S6.6.2 Bivariate Correlated Distributions

Using the GDP per capita example: following from uncertainty estimation, once we generated approximately 1,000 forecast draws for a country and year for any of the covariates, we used the following strategy to achieve consistent temporal correlation across all time periods in the future:

(a) For each country, we recorded country-specific Spearman's correlation coefficient across all draws between 2036 and 2050, which gave us a country-specific correlation vector.

(b) For each value of correlation in step (a), we simulated a bivariate uniform distribution for each country and year (2018 through 2036). This simulated distribution was ranked in such a way that the marginal distributions in the joint distribution were correlated with that value of correlation coefficient we supplied (this joint distribution is known as a *copula*).

(c) We recorded the ranks of the copula, and sorted our draws (within each country) using those temporal ranks, and therefore we ended up with a complete time-series data for all draws, such that each country and year will follow the same rank correlation structure that exists between 2036 and 2050.

(d) Finally, we calculated our final set of uncertainty intervals by taking the 2.5th and 97.5th percentiles of these correlated draws.

We used this method at the end of forecasting every metric, since one metric fed into the other sequentially.

S6.7 Future Health Scenarios

We established the trajectories that our health expenditures are expected to take in the next 33 years using our ensemble models (from hereby referred to as the 'reference' case). The reference forecasts were built upon the basis of each country's past trends and expected future trends from covariates. We additionally also predicted what the possible trajectories for each country would look like if they were to follow the possible optimistic and pessimistic growth rates observed globally (referred to as 'better' and 'worse' cases, respectively).

S6.7.1 Long-term Growth Regressions

In order to determine what the possible better and worse growth rates for each country would be, we ran long-term growth regressions with the following specification:

$$Y_{i,T} - Y_{i,t} = \alpha + \beta Y_{i.t} + \epsilon_{i,t} \tag{7}$$

where the dependent variable represented the long term growth rate of Y for country i, which was computed either as logarithmic or logistic growth rates (for fractions).

The only independent variable we used ($Y_{i,t}$) was the value of Y_i at time t, and it served as a convergence term in this regression. This allowed us to predict the long-term growth rates of Y, conditional on a country's level of Y at time t.

S6.7.2 Forecasting Better and Worse Scenarios

In order to estimate what the future better and worse trajectories would be for each country, we followed these steps (assuming that we are forecasting from 2018 through 2050, with observed data between 1995 and 2017):

(i) We computed the 85th and 15th percentiles of the empirical residuals ϵ, as $Q_{0.85}(\hat{\epsilon_{i,t}})$ and $Q_{0.15}(\hat{\epsilon_{i,t}})$ respectively, where $Q_p(\cdot)$ is a quantile function for a percentile p.

(ii) We computed the starting annualized growth rate from the fitted scenario regression, such that, for country i:

$$\text{Better growth rate} = \exp(\hat{\alpha}) \times \exp(Q_{0.85}(\hat{\epsilon_{i,t}})) \times (Y_{i,2017}^{\hat{\beta}})^{(1/(2017-1995))} \tag{8}$$

$$\text{Worse growth rate} = \exp(\hat{\alpha}) \times \exp(Q_{0.15}(\hat{\epsilon_{i,t}})) \times (Y_{i,2017}^{\hat{\beta}})^{(1/(2017-1995))} \tag{9}$$

(iii) Finally, once we have established the growth rates as a function of the convergence term, we recursively created better and worse trajectories, conditional on the updated

growth rates every year, such that:

$$Y_{i,t+1} = Y_{i,t} \times \exp(\hat{\alpha}) \times \exp(Q_{0.85}(\hat{\epsilon_{i,t}})) \times (Y_{i,t}^{\hat{\beta}}) \tag{10}$$

where $\exp(\hat{\alpha}) \times \exp(Q_{0.85}(\hat{\epsilon_{i,t}})) \times (Y_{i,t}^{\hat{\beta}})$ was the conditional growth rate for a single year.

One condition that we imposed for the computed scenarios is: the better projection cannot be lower than the reference projection, and the worse projection cannot be higher than the reference projection. For countries with wide forecasts where this case did happen, we moved the better and worse forecasts down and up to overlap on top of the reference line respectively.

S6.7.3 Uncertainty Estimation

The uncertainty intervals around a scenario were expected to take the same shape as the uncertainty around our reference forecasts. Therefore, once we have propagated a mean set of better and worse forecasts in section S6.7.2, we created the draws around our scenarios in the following way:

(i) We took our reference forecast's mean line and the 1,000 draws around that line.

(ii) We computed the deviation of the mean from each of the draws (in logarithmic or logistic transformation, depending on the space of the covariate).

(iii) We took each of the scenario mean lines and added the deviations from the previous step to the mean lines, giving us 1,000 draws of the scenario projections.

Figure set S6.3 visualizes the future health scenarios of our health outcomes in per capita space (in 2019 US dollars).

S6.8 Package and Architecture

S6.8.1 Architecture

All analysis and forecasting were done on a parallel computing cluster with 26,000 cores with a CentOS interface. We compiled R [8] version 3.6.0 from source code on a Docker based on Debian OS, which was deployed as a Singularity container with all the necessary compilers and binaries (GCC, G++ and Fortran 8.2.0).

S6.8.2 Template Model Builder

We used the R library **TMB** [9] to run our baseline mixed effects models. TMB stands for Template Model Builder, and it's a powerful method of approximating the integral of the Gaussian probability distribution function (which doesn't exist in closed form) by using a Laplace Approximation, which gives very precise results, and is relatively faster than other approximation packages which exist like Stan or Bugs. TMB uses automatic differentiation on the negative log-likelihood function in order to optimize the model parameters, and also returns a sparse joint precision matrix (inverse of the variance-covariance matrix), from which we can simulate posterior parameter draws.

References

[1] GBD 2017 Risk Factor Collaborators. Global, regional, and national comparative risk assessment of 84 behavioural, environmental and occupational, and metabolic risks or clusters of risks for 195 countries and territories, 1990–2017: a systematic analysis for the Global Burden of Disease Study 2017. *The Lancet*, 392(10159):1923–1994, nov 2018. doi: 10.1016/S0140-6736(18)32225-6. URL `https://doi.org/10.1016/S0140-6736(18)32225-6`.

[2] Spencer L James, Paul Gubbins, Christopher JL Murray, and Emmanuela Gakidou. Developing a comprehensive time series of gdp per capita for 210 countries from 1950 to 2015. *Population health metrics*, 10(1):12, 2012. URL `https://pophealthmetrics.biomedcentral.com/articles/10.1186/1478-7954-10-12`.

[3] Emmanuela Gakidou, Krycia Cowling, Rafael Lozano, and Christopher JL Murray. Increased educational attainment and its effect on child mortality in 175 countries between 1970 and 2009: a systematic analysis. *The Lancet*, 376(9745):959–974, 2010. URL `https://doi.org/10.1016/S0140-6736(10)61257-3`.

[4] Kyle J Foreman, Neal Marquez, Andrew Dolgert, Kai Fukutaki, Nancy Fullman, Madeline McGaughey, Martin A Pletcher, Amanda E Smith, Kendrick Tang, Chun-Wei Yuan, et al. Forecasting life expectancy, years of life lost, and all-cause and cause-specific mortality for 250 causes of death: reference and alternative scenarios for 2016–40 for 195 countries and territories. *The Lancet*, 392(10159):2052–2090, 2018.

[5] Spencer Preston, Patrick Heuveline, and Michel Guillot. *Demography: measuring and modeling population processes*. Wiley-Blackwell, 1 edition. Malden, MA, USA, 2000.

[6] Stein Emil Vollset et al. Widespread population decline: robust fertility, mortality, migration, and population scenarios for 195 countries and territories from 2017 to 2100. *The Lancet*, 2019.

[7] Kyle J Foreman, Rafael Lozano, Alan D Lopez, and Christopher JL Murray. Modeling causes of death: an integrated approach using codem. *Population health metrics*, 10(1): 1, 2012.

[8] R Core Team. *R: A Language and Environment for Statistical Computing*. R Foundation for Statistical Computing, Vienna, Austria, 2017. URL `https://www.R-project.org/`. Accessed: 2017-12-20.

[9] Kasper Kristensen, Anders Nielsen, Casper W Berg, Hans Skaug, and Brad Bell. Tmb: automatic differentiation and laplace approximation. *arXiv preprint arXiv:1509.00660*, 2015.

S6.9 Tables and Figures

Table S6.1: Definitions of health expenditure sources

Health Expenditure Type	Definition
Development assistance for health	Financial and in-kind contributions from global health channels that aim to improve or maintain health in low- or middle-income countries.
Government health expenditure as source	Government health expenditure as source only includes domestically financed government expenditure on health, including public health spending.
Out-of-pocket expenditure	Paid by individuals for health services; considered catastrophic if exceeding 40% of a household's annual income.
Prepaid private health expenditure	Private risk pooling against catastrophic health expenditure; includes private insurance and non-governmental organizations.

Figure S6.1: State Space Diagram of Forecasting Components

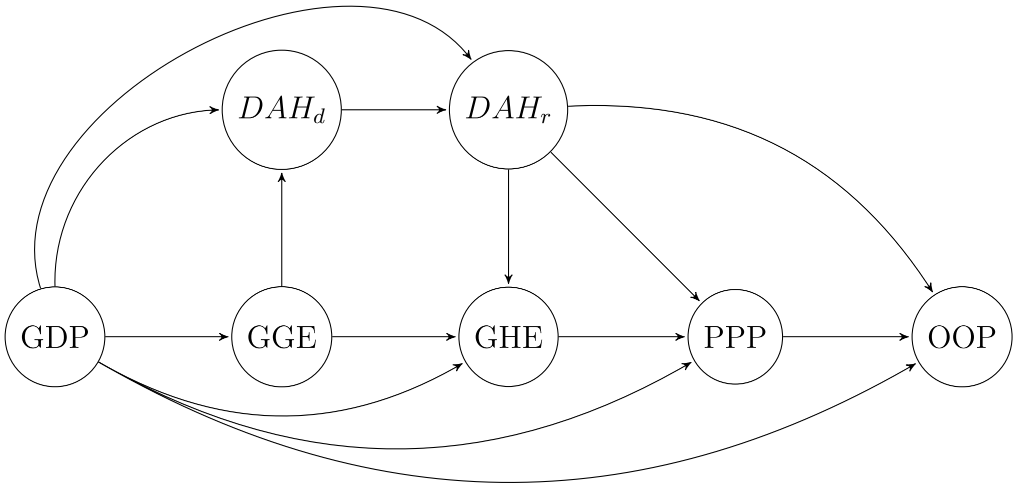

Table S6.2: Prediction and Covariates Map with Transformations

	**Predicted Variables**	**Covariates**	**Extra Specifications**
1	GDP per labour (log)		ARIMA(2-3, 1, 0)
2	GGE per GDP (logit)	GDPpc$^+$, Pop >64$^+$, Education$^+$	ARIMA(0-3, 1, 0-3)
3	DAH donated per GGE (logit)	GDPpc$^+$, GGE/GDP$^+$, Bush Era Dummy$^+$	ARIMA(0-3, 1, 0-3)
4	DAH received per total DAH donated (logit)	GDPpc$^-$, Pop <15$^+$, Ebola dummy$^+$, total DAH envelope	ARIMA(0-3, 0, 0-3)
5	GHE per GGE (logit)	GDPpc$^+$, Pop >64$^+$, GGE/GDP$^+$, DAH/GDP$^-$	ARIMA(0-3, 1, 0-3)
6	PPP per GDP (logit)	GDPpc$^+$, Pop >64$^+$, GGE/GDP. DAH/GDP, GHE/GDP,	ARIMA(0-3, 1, 0-3)
7	OOP per GDP (logit)	GDPpc$^-$, Pop >64$^+$, GGE/GDP, DAH/GDP, GHE/GDP, PPP/GDP	ARIMA(0-3, 1, 0-3)

Figure S6.2: Process diagram for estimating future GDP, all-sector government spending, and health spending by source

This diagram details the inputs and outputs of the ensemble modeling process.

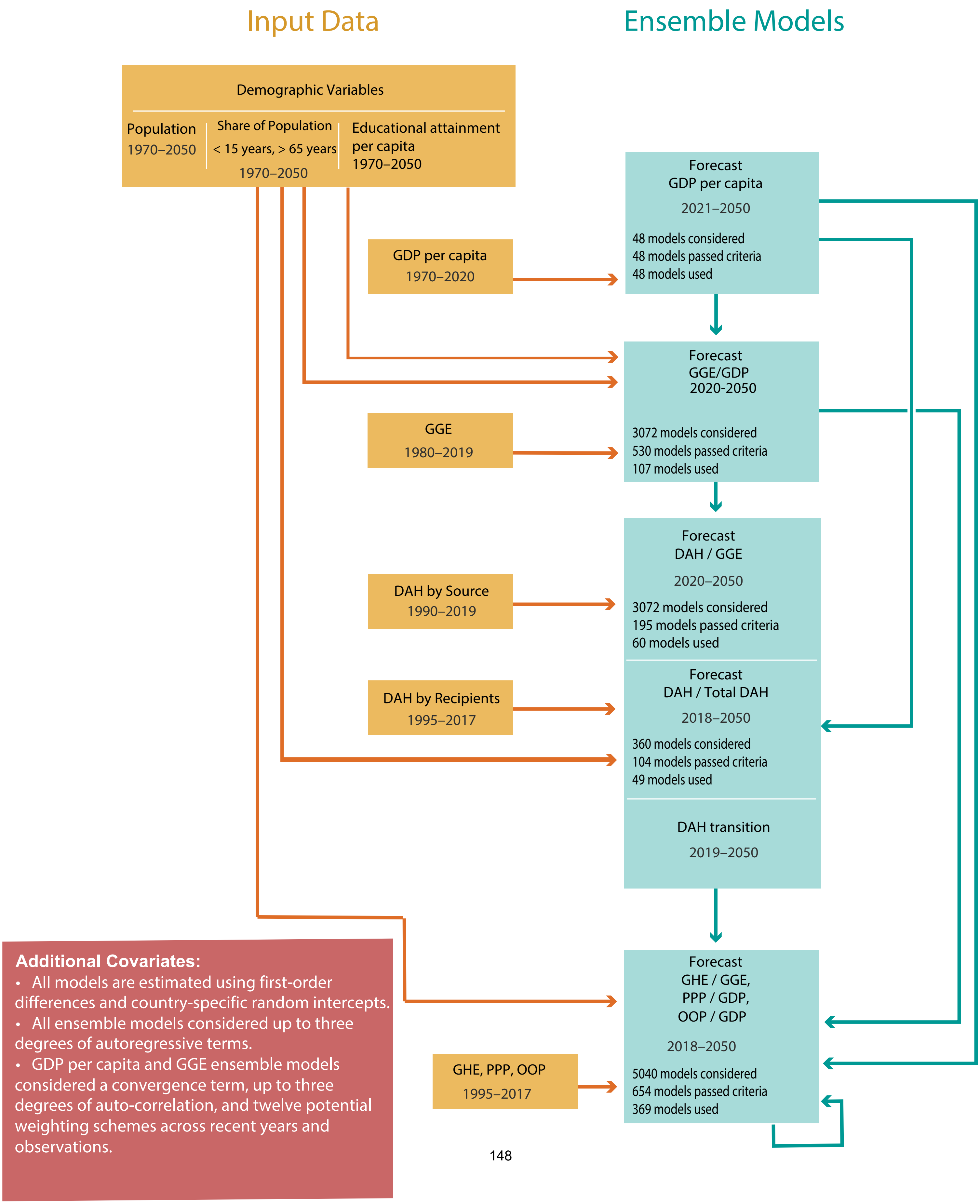
Input Data
Ensemble Models
Demographic Variables
Population
1970–2050
Share of Population
< 15 years, > 65 years
1970–2050
Educational attainment
per capita
1970–2050
GDP per capita
1970–2020
GGE
1980–2019
DAH by Source
1990–2019
DAH by Recipients
1995–2017
GHE, PPP, OOP
1995–2017
Forecast
GDP per capita
2021–2050
48 models considered
48 models passed criteria
48 models used
Forecast
GGE/GDP
2020-2050
3072 models considered
530 models passed criteria
107 models used
Forecast
DAH / GGE
2020–2050
3072 models considered
195 models passed criteria
60 models used
Forecast
DAH / Total DAH
2018–2050
360 models considered
104 models passed criteria
49 models used
DAH transition
2019–2050
Forecast
GHE / GGE,
PPP / GDP,
OOP / GDP
2018–2050
5040 models considered
654 models passed criteria
369 models used
Additional Covariates:
• All models are estimated using first-order differences and country-specific random intercepts.
• All ensemble models considered up to three degrees of autoregressive terms.
• GDP per capita and GGE ensemble models considered a convergence term, up to three degrees of auto-correlation, and twelve potential weighting schemes across recent years and observations.

Table S6.3: Ensemble submodel specifications used in forecasts

This table shows all submodel specifications considered in the ensemble, and the percentage of models with given specifications used in the forecasts.

	GDP		GGE		DAH Provided		DAH Received		GHE		PPP		OOP	
Number of out-of-sample years included	15		15		15		10		15		15		15	
Number of countries included	195		195		24		139		195		195		195	
Number of top models used per country and year	15		17		15		15		15		15		34	
Total number of models used across all countries and years	43875		49725		5400		20850		29250		29250		66300	
Covariate(s)	Value	Percentage of models	Value	Percentage of models	Value	Percentage of models	Value	Percentage of models	Value	Percentage of models	Value	Percentage of models	Value	Percentage of models
Autoregressive terms	3	50.1%	3	16.4%	3	0.0%	0	100.0%	2	25.6%	2	100.0%	3	0.0%
	2	49.9%	2	14.7%	2	0.0%			1	41.3%			2	100.0%
			1	9.6%	1	41.9%			0	33.1%				
			0	59.3%	0	58.1%								
Moving average terms	0	100.0%	3	14.8%	3	24.1%	0	100.0%	0	100.0%	0	100.0%	0	100.0%
			2	18.7%	2	15.0%								
			1	36.2%	1	60.9%								
			0	30.3%	0	0.0%								
Convergence term	Included	53.2%	Included	19.4%	Included	100.0%	Not included	100.0%	Not included	100.0%	Not included	100.0%	Not included	100.0%
	Not included	46.8%	Not included	80.6%	Not included	0.0%								
Recency weights (Values represent the tau value used in our recency weighting function described on page 10 of this appendix. Higher values indicate a stronger weight placed on recent years' data.)	0.70000	15.3%	0.70000	10.5%	0.70000	20.4%	0.80000	4.0%	0.20000	6.6%	0.20000	7.7%	0.20000	5.3%
	0.63636	15.5%	0.63636	10.9%	0.63636	17.1%	0.77241	4.1%	0.18571	6.6%	0.18571	7.7%	0.18571	5.2%
	0.57273	15.2%	0.57273	10.9%	0.57273	15.3%	0.74483	7.0%	0.17143	6.6%	0.17143	7.7%	0.17143	5.2%
	0.50909	11.8%	0.50909	11.7%	0.50909	14.1%	0.71724	5.8%	0.15714	6.6%	0.15714	7.7%	0.15714	5.2%
	0.44545	4.8%	0.44545	9.7%	0.44545	11.4%	0.68966	5.8%	0.14286	6.6%	0.14286	7.7%	0.14286	5.3%
	0.38182	4.1%	0.38182	6.9%	0.38182	2.5%	0.66207	5.8%	0.12857	6.7%	0.12857	7.8%	0.12857	5.5%
	0.31818	4.3%	0.31818	8.0%	0.31818	2.7%	0.63448	5.8%	0.11429	6.7%	0.11429	10.0%	0.11429	5.7%
	0.25455	4.8%	0.25455	5.0%	0.25455	3.1%	0.60690	5.8%	0.10000	6.7%	0.10000	7.9%	0.10000	5.8%
	0.19091	5.5%	0.19091	5.8%	0.19091	3.4%	0.57931	5.8%	0.08571	6.7%	0.08571	5.7%	0.08571	6.7%
	0.12727	6.0%	0.12727	6.5%	0.12727	3.4%	0.55172	5.8%	0.07143	6.7%	0.07143	5.7%	0.07143	6.9%
	0.06364	6.3%	0.06364	6.9%	0.06364	3.2%	0.52414	5.8%	0.05714	6.7%	0.05714	5.7%	0.05714	7.8%
	0.00000	6.5%	0.00000	7.2%	0.00000	3.3%	0.49655	5.8%	0.04286	6.7%	0.04286	5.8%	0.04286	8.2%
							0.46897	5.1%	0.028571429	6.7%	0.028571429	4.4%	0.028571429	8.6%
							0.44138	5.0%	0.014285714	6.7%	0.014285714	4.4%	0.014285714	9.1%
							0.41379	0.0%	0	6.7%	0	4.4%	0	9.5%
							0.38621	5.5%						
							0.35862	4.0%						
							0.33103	4.0%						
							0.30345	0.9%						
							0.27586	0.9%						
							0.24828	0.9%						
							0.22069	0.8%						
							0.1931	0.8%						
							0.16552	0.8%						
							0.13793	0.7%						
							0.11034	0.7%						
							0.08276	0.7%						
							0.05517	0.7%						
							0.02759	0.6%						
							0	0.6%						
Proportion of population under age 15							Included	3.6%						
							Not included	96.4%						
Proportion of population over age 65			Included	0.0%					Included	51.4%	Included	0.0%	Included	55.3%
			Not included	100.0%					Not included	48.6%	Not included	100.0%	Not included	44.7%
GDP per capita			Included	0.0%	Included	32.6%	Included	100.0%	Included	0.0%	Included	0.0%	Included	0.0%
			Not included	100.0%	Not included	67.4%	Not included	0.0%	Not included	100.0%	Not included	100.0%	Not included	100.0%
GGE per GDP					Included	0.0%			Included	0.0%	Included	0.0%	Included	0.0%
					Not included	100.0%			Not included	100.0%	Not included	100.0%	Not included	100.0%
DAH Received per GDP									Included	0.0%	Included	47.3%	Included	50.2%
									Not included	100.0%	Not included	52.7%	Not included	49.8%
GHE per GDP											Included	61.1%	Included	0.0%
											Not included	38.9%	Not included	100.0%
Educational attainment per capita			Included	6.3%										
			Not included	93.7%										
Total DAH Received envelope							Included	29.8%						
							Not included	70.2%						

Table S6.4: Health Spending from 1995-2050

This table shows all of our health spending outcomes for every country and year from 1995 through 2050. The variables are in per capita measures, and for currencies 2019 US dollars and 2019 purchasing-power parity-adjusted dollars.

Country	Year	2019 US dollars per capita: Total health spending	Government health spending	Out-of-pocket health spending	Prepaid private health spending	Development assistance for health	2019 US purchasing-power parity-adjusted dollars per capita: Total health spending	Government health spending	Out-of-pocket health spending	Prepaid private health spending	Development assistance for health
Afghanistan	1995	30 (21 to 42)	1 (1 to 1)	29 (20 to 41)	0 (0 to 1)	0 (0 to 0)	124 (87 to 172)	4 (3 to 5)	118 (81 to 166)	1 (1 to 2)	1 (1 to 1)
Afghanistan	1996	28 (20 to 39)	1 (1 to 1)	27 (18 to 37)	0 (0 to 0)	0 (0 to 0)	115 (82 to 159)	3 (3 to 4)	110 (76 to 153)	1 (0 to 2)	1 (1 to 1)
Afghanistan	1997	27 (19 to 37)	1 (1 to 1)	25 (17 to 35)	0 (0 to 0)	1 (1 to 1)	109 (78 to 150)	3 (2 to 4)	103 (71 to 143)	1 (0 to 2)	2 (2 to 2)
Afghanistan	1998	25 (18 to 34)	1 (0 to 1)	24 (16 to 33)	0 (0 to 0)	0 (0 to 0)	101 (72 to 138)	3 (2 to 4)	97 (68 to 133)	1 (0 to 2)	1 (1 to 1)
Afghanistan	1999	23 (17 to 32)	1 (0 to 1)	22 (15 to 30)	0 (0 to 0)	0 (0 to 0)	96 (68 to 129)	2 (2 to 3)	90 (63 to 123)	1 (0 to 1)	2 (2 to 2)
Afghanistan	2000	22 (16 to 29)	1 (0 to 1)	21 (15 to 28)	0 (0 to 0)	0 (0 to 0)	90 (65 to 120)	2 (2 to 3)	85 (60 to 116)	1 (0 to 1)	2 (2 to 2)
Afghanistan	2001	21 (15 to 28)	1 (0 to 1)	19 (14 to 27)	0 (0 to 0)	1 (1 to 1)	86 (62 to 116)	2 (2 to 3)	80 (57 to 110)	1 (0 to 1)	3 (3 to 3)
Afghanistan	2002	23 (17 to 31)	1 (0 to 1)	21 (15 to 29)	0 (0 to 0)	1 (1 to 1)	95 (71 to 126)	2 (2 to 3)	86 (61 to 117)	1 (0 to 1)	6 (6 to 6)
Afghanistan	2003	29 (21 to 37)	1 (1 to 2)	25 (18 to 34)	0 (0 to 1)	2 (2 to 2)	117 (88 to 153)	5 (4 to 7)	103 (73 to 138)	1 (1 to 2)	8 (8 to 8)
Afghanistan	2004	33 (25 to 42)	1 (1 to 2)	26 (18 to 35)	0 (0 to 1)	5 (5 to 5)	134 (103 to 171)	6 (5 to 8)	105 (75 to 142)	1 (1 to 3)	21 (21 to 21)
Afghanistan	2005	34 (27 to 43)	2 (1 to 2)	26 (19 to 35)	0 (0 to 1)	6 (6 to 6)	139 (109 to 175)	6 (5 to 8)	106 (76 to 142)	1 (1 to 3)	25 (25 to 25)
Afghanistan	2006	35 (28 to 44)	2 (1 to 2)	27 (19 to 36)	0 (0 to 1)	6 (6 to 6)	145 (114 to 182)	7 (5 to 9)	111 (80 to 147)	2 (1 to 3)	25 (25 to 25)

Afghanistan	2007	39 (32 to 49)	2 (2 to 3)	29 (21 to 38)	0 (0 to 1)	8 (8 to 8)	161 (129 to 200)	9 (6 to 11)	119 (87 to 158)	2 (1 to 4)	32 (32 to 32)
Afghanistan	2008	39 (31 to 48)	2 (2 to 3)	29 (21 to 38)	0 (0 to 1)	7 (7 to 7)	159 (127 to 197)	8 (6 to 11)	118 (86 to 156)	2 (1 to 3)	31 (31 to 31)
Afghanistan	2009	44 (35 to 53)	2 (2 to 3)	30 (22 to 40)	0 (0 to 1)	11 (11 to 11)	179 (146 to 219)	9 (7 to 12)	124 (91 to 163)	2 (1 to 4)	44 (44 to 44)
Afghanistan	2010	43 (35 to 53)	2 (2 to 3)	30 (22 to 40)	0 (0 to 1)	10 (10 to 10)	176 (143 to 216)	9 (7 to 11)	123 (90 to 163)	2 (1 to 4)	41 (41 to 41)
Afghanistan	2011	45 (37 to 55)	2 (2 to 3)	30 (22 to 40)	0 (0 to 1)	12 (12 to 12)	185 (153 to 226)	9 (7 to 11)	124 (91 to 164)	2 (1 to 4)	51 (51 to 51)
Afghanistan	2012	45 (37 to 56)	2 (2 to 3)	32 (24 to 43)	1 (0 to 1)	10 (10 to 10)	185 (151 to 229)	9 (7 to 12)	132 (97 to 176)	2 (1 to 4)	41 (41 to 41)
Afghanistan	2013	47 (38 to 58)	2 (2 to 3)	34 (25 to 45)	1 (0 to 1)	10 (10 to 10)	194 (158 to 238)	10 (7 to 13)	139 (104 to 184)	2 (1 to 4)	43 (43 to 43)
Afghanistan	2014	49 (40 to 60)	3 (2 to 3)	36 (27 to 48)	1 (0 to 1)	9 (9 to 9)	201 (163 to 247)	10 (8 to 13)	150 (112 to 197)	2 (1 to 4)	39 (39 to 39)
Afghanistan	2015	51 (40 to 63)	3 (2 to 3)	39 (28 to 51)	1 (0 to 1)	9 (9 to 9)	207 (165 to 258)	11 (8 to 14)	158 (116 to 209)	2 (1 to 4)	37 (37 to 37)
Afghanistan	2016	49 (37 to 62)	3 (2 to 4)	40 (29 to 54)	1 (0 to 1)	5 (5 to 5)	199 (154 to 256)	11 (8 to 15)	166 (120 to 223)	2 (1 to 4)	20 (20 to 20)
Afghanistan	2017	50 (37 to 65)	3 (2 to 4)	42 (30 to 56)	1 (0 to 1)	5 (5 to 5)	203 (154 to 265)	11 (8 to 15)	171 (121 to 231)	2 (1 to 4)	19 (19 to 19)
Afghanistan	2018	49 (37 to 64)	3 (2 to 4)	42 (29 to 56)	1 (0 to 1)	4 (4 to 4)	202 (153 to 263)	12 (9 to 16)	170 (121 to 230)	2 (1 to 4)	18 (18 to 18)
Afghanistan	2019	50 (38 to 64)	3 (2 to 4)	42 (30 to 56)	1 (0 to 1)	4 (4 to 4)	204 (154 to 264)	12 (9 to 16)	171 (122 to 231)	2 (1 to 4)	18 (18 to 18)
Afghanistan	2020	50 (38 to 65)	3 (2 to 4)	42 (30 to 57)	1 (0 to 1)	4 (4 to 5)	205 (156 to 265)	12 (9 to 16)	173 (123 to 232)	2 (1 to 4)	18 (17 to 19)
Afghanistan	2021	50 (38 to 65)	3 (2 to 4)	42 (30 to 57)	1 (0 to 1)	4 (4 to 5)	206 (156 to 266)	12 (9 to 17)	173 (124 to 233)	2 (1 to 4)	18 (17 to 19)

Afghanistan	2022	50 (38 to 65)	3 (2 to 4)	42 (30 to 57)	1 (0 to 1)	4 (4 to 5)	206 (157 to 266)	13 (9 to 17)	174 (124 to 234)	2 (1 to 4)	18 (16 to 20)
Afghanistan	2023	50 (38 to 65)	3 (2 to 4)	42 (30 to 57)	1 (0 to 1)	4 (4 to 5)	206 (155 to 268)	13 (9 to 17)	174 (124 to 234)	2 (1 to 4)	18 (16 to 20)
Afghanistan	2024	50 (38 to 65)	3 (2 to 4)	43 (30 to 57)	1 (0 to 1)	4 (4 to 5)	207 (156 to 267)	13 (9 to 17)	175 (124 to 235)	2 (1 to 4)	18 (16 to 20)
Afghanistan	2025	51 (38 to 66)	3 (2 to 4)	43 (31 to 58)	1 (0 to 1)	4 (4 to 5)	209 (157 to 270)	13 (9 to 17)	177 (126 to 238)	2 (1 to 4)	17 (15 to 20)
Afghanistan	2026	51 (39 to 66)	3 (2 to 4)	44 (31 to 59)	1 (0 to 1)	4 (4 to 5)	211 (159 to 272)	13 (9 to 17)	179 (127 to 240)	2 (1 to 4)	17 (15 to 20)
Afghanistan	2027	52 (39 to 68)	3 (2 to 4)	44 (31 to 59)	1 (0 to 1)	4 (4 to 5)	213 (161 to 278)	13 (9 to 18)	181 (128 to 243)	2 (1 to 4)	17 (15 to 20)
Afghanistan	2028	52 (40 to 68)	3 (2 to 4)	44 (32 to 60)	1 (0 to 1)	4 (4 to 5)	215 (162 to 278)	13 (9 to 18)	182 (130 to 245)	2 (1 to 4)	17 (15 to 20)
Afghanistan	2029	53 (40 to 68)	3 (2 to 5)	45 (32 to 60)	1 (0 to 1)	4 (4 to 5)	217 (163 to 280)	14 (10 to 19)	184 (131 to 247)	3 (1 to 5)	17 (15 to 20)
Afghanistan	2030	53 (40 to 68)	3 (2 to 5)	45 (32 to 61)	1 (0 to 1)	4 (3 to 5)	219 (165 to 281)	14 (10 to 20)	185 (132 to 249)	3 (1 to 5)	17 (14 to 20)
Afghanistan	2031	54 (40 to 69)	4 (3 to 5)	46 (32 to 61)	1 (0 to 1)	4 (3 to 5)	221 (165 to 285)	15 (10 to 21)	187 (133 to 251)	3 (1 to 5)	17 (14 to 20)
Afghanistan	2032	54 (41 to 70)	4 (3 to 5)	46 (33 to 62)	1 (0 to 1)	4 (3 to 5)	223 (167 to 288)	15 (11 to 21)	188 (134 to 253)	3 (1 to 5)	17 (14 to 20)
Afghanistan	2033	55 (41 to 71)	4 (3 to 5)	46 (33 to 62)	1 (0 to 1)	4 (3 to 5)	225 (170 to 290)	16 (11 to 22)	190 (135 to 256)	3 (1 to 5)	16 (14 to 20)
Afghanistan	2034	56 (42 to 72)	4 (3 to 6)	47 (33 to 63)	1 (0 to 1)	4 (3 to 5)	228 (171 to 294)	17 (12 to 23)	191 (136 to 258)	3 (1 to 5)	16 (14 to 20)
Afghanistan	2035	56 (42 to 72)	4 (3 to 6)	47 (33 to 63)	1 (0 to 1)	4 (3 to 5)	230 (174 to 297)	18 (12 to 25)	193 (137 to 260)	3 (2 to 5)	16 (14 to 20)
Afghanistan	2036	57 (43 to 73)	5 (3 to 6)	47 (34 to 64)	1 (0 to 1)	4 (3 to 5)	232 (176 to 300)	18 (13 to 26)	194 (138 to 262)	3 (2 to 6)	16 (13 to 20)

Afghanistan	2037	57 (43 to 74)	5 (3 to 7)	48 (34 to 65)	1 (0 to 1)	4 (3 to 5)	235 (178 to 305)	19 (13 to 27)	197 (139 to 265)	3 (2 to 6)	16 (13 to 20)
Afghanistan	2038	58 (44 to 75)	5 (3 to 7)	48 (34 to 65)	1 (0 to 1)	4 (3 to 5)	238 (179 to 308)	20 (14 to 28)	199 (141 to 268)	3 (2 to 6)	16 (13 to 20)
Afghanistan	2039	59 (44 to 76)	5 (3 to 7)	49 (35 to 66)	1 (0 to 1)	4 (3 to 5)	241 (182 to 312)	21 (14 to 29)	201 (143 to 271)	3 (2 to 6)	16 (13 to 20)
Afghanistan	2040	60 (45 to 77)	5 (4 to 7)	50 (35 to 67)	1 (0 to 2)	4 (3 to 5)	245 (184 to 315)	21 (15 to 30)	204 (145 to 274)	3 (2 to 6)	16 (13 to 20)
Afghanistan	2041	60 (46 to 78)	5 (4 to 8)	50 (36 to 68)	1 (0 to 2)	4 (3 to 5)	248 (188 to 320)	22 (15 to 31)	207 (147 to 278)	4 (2 to 7)	16 (13 to 20)
Afghanistan	2042	62 (47 to 79)	6 (4 to 8)	51 (36 to 69)	1 (0 to 2)	4 (3 to 5)	252 (191 to 324)	23 (15 to 32)	210 (149 to 283)	4 (2 to 7)	16 (13 to 20)
Afghanistan	2043	63 (48 to 81)	6 (4 to 8)	52 (37 to 70)	1 (0 to 2)	4 (3 to 5)	256 (196 to 330)	23 (16 to 33)	214 (151 to 288)	4 (2 to 7)	16 (13 to 20)
Afghanistan	2044	64 (48 to 82)	6 (4 to 8)	53 (37 to 72)	1 (0 to 2)	4 (3 to 5)	261 (196 to 336)	24 (16 to 34)	217 (154 to 294)	4 (2 to 7)	16 (13 to 21)
Afghanistan	2045	65 (49 to 83)	6 (4 to 9)	54 (38 to 73)	1 (0 to 2)	4 (3 to 5)	265 (203 to 342)	25 (17 to 35)	221 (156 to 300)	4 (2 to 7)	16 (12 to 20)
Afghanistan	2046	66 (50 to 86)	6 (4 to 9)	55 (39 to 75)	1 (0 to 2)	4 (3 to 5)	271 (205 to 351)	25 (17 to 37)	226 (159 to 306)	4 (2 to 7)	16 (12 to 20)
Afghanistan	2047	67 (51 to 87)	6 (4 to 9)	56 (40 to 76)	1 (1 to 2)	4 (3 to 5)	276 (210 to 357)	26 (17 to 38)	230 (162 to 312)	4 (2 to 8)	16 (12 to 20)
Afghanistan	2048	68 (51 to 89)	7 (4 to 10)	57 (40 to 77)	1 (1 to 2)	4 (3 to 5)	281 (211 to 365)	27 (18 to 39)	234 (165 to 317)	4 (2 to 8)	15 (12 to 21)
Afghanistan	2049	70 (53 to 90)	7 (4 to 10)	58 (41 to 79)	1 (1 to 2)	4 (3 to 5)	286 (215 to 369)	28 (18 to 40)	238 (169 to 323)	4 (2 to 8)	15 (12 to 20)
Afghanistan	2050	71 (54 to 92)	7 (5 to 10)	59 (42 to 80)	1 (1 to 2)	4 (3 to 5)	291 (221 to 379)	28 (19 to 42)	243 (172 to 329)	5 (2 to 8)	15 (12 to 21)
Albania	1995	62 (52 to 75)	29 (23 to 35)	26 (18 to 37)	2 (1 to 4)	5 (5 to 5)	159 (133 to 193)	74 (60 to 90)	68 (46 to 95)	5 (2 to 10)	12 (12 to 12)

Albania	1996	67 (56 to 81)	32 (26 to 39)	28 (19 to 40)	2 (1 to 4)	4 (4 to 4)	170 (142 to 207)	82 (67 to 99)	72 (48 to 103)	6 (3 to 10)	10 (10 to 10)
Albania	1997	66 (54 to 80)	34 (28 to 40)	29 (19 to 41)	2 (1 to 4)	1 (1 to 1)	168 (139 to 205)	86 (71 to 103)	74 (50 to 105)	6 (3 to 10)	3 (3 to 3)
Albania	1998	80 (67 to 96)	42 (35 to 49)	32 (22 to 46)	3 (1 to 5)	3 (3 to 3)	204 (171 to 246)	107 (89 to 127)	83 (55 to 118)	7 (3 to 13)	8 (8 to 8)
Albania	1999	99 (84 to 117)	52 (44 to 61)	37 (25 to 53)	3 (1 to 6)	7 (7 to 7)	253 (216 to 300)	134 (113 to 157)	94 (64 to 135)	8 (4 to 15)	18 (18 to 18)
Albania	2000	108 (92 to 129)	58 (49 to 68)	40 (27 to 58)	3 (2 to 6)	7 (7 to 7)	278 (237 to 330)	148 (126 to 174)	102 (69 to 147)	9 (4 to 16)	19 (19 to 19)
Albania	2001	119 (102 to 141)	64 (55 to 75)	43 (29 to 62)	4 (2 to 7)	8 (8 to 8)	304 (261 to 360)	164 (140 to 192)	111 (75 to 158)	10 (5 to 18)	19 (19 to 19)
Albania	2002	125 (108 to 149)	69 (59 to 80)	46 (32 to 66)	4 (2 to 8)	6 (6 to 6)	321 (276 to 381)	176 (151 to 204)	118 (81 to 169)	11 (5 to 20)	16 (16 to 16)
Albania	2003	136 (117 to 160)	74 (63 to 86)	50 (35 to 70)	5 (2 to 9)	8 (8 to 8)	349 (300 to 410)	189 (162 to 219)	127 (89 to 180)	12 (6 to 22)	21 (21 to 21)
Albania	2004	151 (130 to 177)	83 (72 to 96)	55 (38 to 77)	5 (3 to 10)	8 (8 to 8)	387 (333 to 453)	213 (184 to 246)	140 (96 to 196)	14 (6 to 25)	21 (21 to 21)
Albania	2005	162 (139 to 189)	88 (76 to 100)	58 (40 to 83)	6 (3 to 11)	10 (10 to 10)	415 (356 to 484)	224 (194 to 257)	149 (102 to 212)	15 (7 to 27)	26 (26 to 26)
Albania	2006	179 (154 to 209)	97 (85 to 111)	63 (43 to 90)	7 (3 to 12)	11 (11 to 11)	458 (393 to 534)	250 (217 to 285)	162 (111 to 229)	17 (8 to 32)	28 (28 to 28)
Albania	2007	195 (168 to 228)	112 (99 to 128)	70 (47 to 99)	8 (3 to 14)	6 (6 to 6)	500 (429 to 584)	287 (254 to 328)	178 (121 to 253)	20 (9 to 37)	15 (15 to 15)
Albania	2008	243 (211 to 282)	145 (129 to 164)	80 (54 to 114)	9 (5 to 17)	9 (9 to 9)	621 (541 to 723)	370 (330 to 420)	204 (139 to 292)	24 (12 to 44)	23 (23 to 23)
Albania	2009	273 (238 to 316)	168 (151 to 189)	87 (59 to 128)	11 (5 to 19)	7 (7 to 7)	698 (610 to 810)	430 (386 to 483)	223 (152 to 327)	27 (13 to 49)	18 (18 to 18)
Albania	2010	283 (247 to 327)	175 (156 to 195)	90 (61 to 132)	11 (5 to 19)	6 (6 to 6)	724 (632 to 838)	449 (400 to 499)	232 (157 to 339)	27 (13 to 48)	15 (15 to 15)

Albania	2011	301 (264 to 346)	184 (163 to 204)	95 (63 to 135)	11 (5 to 20)	12 (12 to 12)	771 (677 to 887)	470 (418 to 522)	242 (162 to 346)	29 (14 to 50)	30 (30 to 30)
Albania	2012	304 (266 to 351)	189 (168 to 209)	98 (66 to 141)	12 (6 to 21)	4 (4 to 4)	777 (682 to 898)	484 (431 to 536)	252 (168 to 361)	31 (14 to 55)	11 (11 to 11)
Albania	2013	312 (273 to 360)	197 (176 to 218)	102 (68 to 144)	13 (6 to 23)	1 (1 to 1)	800 (698 to 921)	503 (450 to 559)	261 (174 to 369)	33 (15 to 60)	3 (3 to 3)
Albania	2014	343 (300 to 393)	215 (194 to 238)	109 (73 to 156)	14 (7 to 27)	5 (5 to 5)	878 (769 to 1006)	549 (496 to 610)	278 (187 to 399)	37 (17 to 68)	13 (13 to 13)
Albania	2015	344 (299 to 397)	216 (194 to 240)	110 (74 to 158)	15 (7 to 28)	3 (3 to 3)	880 (766 to 1017)	553 (496 to 616)	283 (190 to 405)	38 (18 to 71)	6 (6 to 6)
Albania	2016	348 (301 to 404)	218 (194 to 245)	113 (76 to 162)	15 (7 to 30)	2 (2 to 2)	891 (772 to 1034)	559 (498 to 629)	289 (194 to 416)	39 (18 to 76)	4 (4 to 4)
Albania	2017	364 (312 to 428)	229 (200 to 262)	117 (80 to 169)	16 (8 to 32)	2 (2 to 2)	933 (799 to 1096)	586 (513 to 670)	301 (205 to 432)	42 (20 to 81)	5 (5 to 5)
Albania	2018	388 (326 to 457)	246 (215 to 281)	123 (84 to 176)	18 (8 to 34)	2 (2 to 2)	993 (834 to 1171)	629 (552 to 720)	314 (214 to 451)	45 (21 to 86)	4 (4 to 4)
Albania	2019	411 (352 to 481)	264 (231 to 302)	127 (87 to 182)	19 (9 to 36)	2 (2 to 2)	1053 (901 to 1233)	676 (591 to 773)	325 (222 to 465)	48 (23 to 91)	4 (4 to 4)
Albania	2020	436 (369 to 514)	283 (247 to 324)	132 (90 to 189)	20 (9 to 38)	2 (2 to 2)	1118 (944 to 1316)	725 (632 to 830)	338 (232 to 484)	50 (24 to 97)	5 (4 to 5)
Albania	2021	454 (388 to 532)	297 (258 to 339)	135 (93 to 193)	20 (10 to 39)	2 (2 to 2)	1162 (993 to 1362)	760 (662 to 869)	345 (237 to 493)	52 (25 to 101)	5 (4 to 5)
Albania	2022	470 (399 to 557)	310 (270 to 355)	137 (94 to 196)	21 (10 to 41)	2 (2 to 2)	1205 (1021 to 1428)	795 (691 to 909)	351 (240 to 502)	54 (26 to 104)	5 (4 to 5)
Albania	2023	487 (418 to 572)	324 (281 to 370)	139 (95 to 199)	22 (10 to 42)	2 (2 to 2)	1247 (1072 to 1465)	830 (720 to 948)	357 (244 to 509)	56 (26 to 108)	5 (4 to 5)

Albania	2024	504 (426 to 594)	338 (294 to 388)	141 (97 to 202)	23 (11 to 43)	2 (2 to 2)	1291 (1092 to 1523)	866 (753 to 993)	362 (248 to 519)	58 (27 to 111)	5 (4 to 6)
Albania	2025	520 (449 to 610)	352 (306 to 405)	143 (98 to 205)	23 (11 to 45)	2 (2 to 2)	1333 (1151 to 1563)	902 (784 to 1037)	366 (251 to 525)	59 (28 to 114)	5 (4 to 6)
Albania	2026	537 (458 to 637)	366 (318 to 421)	145 (99 to 208)	24 (11 to 46)	2 (2 to 2)	1376 (1173 to 1633)	938 (814 to 1079)	371 (255 to 534)	61 (29 to 117)	5 (5 to 6)
Albania	2027	554 (474 to 651)	381 (330 to 439)	147 (101 to 212)	25 (12 to 47)	2 (2 to 2)	1419 (1215 to 1667)	975 (844 to 1124)	376 (258 to 542)	63 (30 to 121)	5 (5 to 6)
Albania	2028	572 (487 to 675)	395 (342 to 458)	149 (102 to 215)	25 (12 to 48)	2 (2 to 2)	1464 (1248 to 1728)	1013 (877 to 1172)	381 (262 to 550)	65 (31 to 124)	5 (5 to 6)
Albania	2029	589 (500 to 695)	410 (355 to 476)	151 (103 to 217)	26 (12 to 50)	2 (2 to 2)	1509 (1281 to 1780)	1051 (908 to 1218)	386 (265 to 557)	67 (32 to 127)	5 (5 to 6)
Albania	2030	607 (516 to 725)	425 (366 to 495)	153 (105 to 220)	27 (13 to 51)	2 (2 to 3)	1554 (1321 to 1856)	1089 (937 to 1267)	391 (269 to 563)	69 (32 to 131)	6 (5 to 7)
Albania	2031	625 (530 to 733)	440 (379 to 514)	155 (106 to 223)	28 (13 to 53)	2 (2 to 3)	1601 (1358 to 1878)	1128 (970 to 1318)	396 (272 to 571)	71 (33 to 135)	6 (5 to 7)
Albania	2032	644 (549 to 769)	456 (391 to 533)	157 (108 to 226)	28 (13 to 54)	2 (2 to 3)	1649 (1406 to 1969)	1168 (1001 to 1366)	402 (276 to 579)	73 (34 to 139)	6 (5 to 7)
Albania	2033	664 (565 to 775)	473 (403 to 553)	160 (109 to 230)	29 (14 to 56)	2 (2 to 3)	1700 (1448 to 1986)	1210 (1033 to 1416)	409 (280 to 589)	75 (35 to 143)	6 (5 to 7)

Albania	2034	684 (582 to 808)	489 (417 to 574)	162 (111 to 234)	30 (14 to 58)	2 (2 to 3)	1751 (1492 to 2070)	1253 (1069 to 1469)	416 (284 to 599)	77 (36 to 148)	6 (5 to 7)
Albania	2035	705 (600 to 827)	506 (431 to 595)	165 (113 to 238)	31 (15 to 59)	2 (2 to 3)	1806 (1537 to 2118)	1297 (1104 to 1524)	423 (289 to 610)	79 (37 to 152)	6 (5 to 8)
Albania	2036	727 (622 to 867)	524 (447 to 616)	168 (115 to 243)	32 (15 to 61)	2 (2 to 3)	1861 (1592 to 2221)	1342 (1145 to 1577)	431 (294 to 623)	82 (38 to 157)	6 (5 to 8)
Albania	2037	749 (636 to 893)	542 (461 to 640)	172 (117 to 248)	33 (15 to 63)	3 (2 to 3)	1919 (1629 to 2288)	1389 (1181 to 1639)	440 (301 to 636)	84 (40 to 162)	6 (5 to 8)
Albania	2038	772 (650 to 921)	561 (476 to 663)	175 (120 to 253)	34 (16 to 65)	3 (2 to 3)	1978 (1665 to 2359)	1436 (1218 to 1699)	449 (306 to 649)	87 (41 to 167)	7 (5 to 8)
Albania	2039	796 (673 to 935)	580 (491 to 686)	179 (122 to 258)	35 (16 to 67)	3 (2 to 3)	2039 (1723 to 2394)	1485 (1257 to 1758)	458 (312 to 662)	89 (42 to 172)	7 (6 to 9)
Albania	2040	820 (687 to 980)	599 (506 to 710)	183 (125 to 264)	36 (17 to 69)	3 (2 to 3)	2102 (1761 to 2509)	1534 (1297 to 1819)	468 (319 to 676)	92 (43 to 178)	7 (6 to 9)
Albania	2041	845 (711 to 1000)	618 (523 to 735)	187 (127 to 270)	37 (17 to 72)	3 (2 to 3)	2165 (1820 to 2561)	1584 (1340 to 1882)	479 (326 to 691)	95 (44 to 184)	7 (6 to 9)
Albania	2042	870 (728 to 1051)	638 (540 to 756)	191 (130 to 276)	38 (18 to 74)	3 (2 to 4)	2229 (1865 to 2693)	1634 (1382 to 1935)	489 (333 to 708)	98 (46 to 189)	7 (6 to 9)
Albania	2043	895 (758 to 1055)	657 (556 to 779)	195 (133 to 283)	39 (18 to 76)	3 (2 to 4)	2292 (1940 to 2702)	1684 (1423 to 1996)	500 (341 to 725)	101 (47 to 194)	7 (6 to 10)

Albania	2044	920 (768 to 1103)	677 (570 to 802)	200 (136 to 290)	41 (19 to 78)	3 (2 to 4)	2357 (1967 to 2826)	1734 (1461 to 2055)	512 (349 to 742)	104 (48 to 199)	7 (6 to 10)
Albania	2045	945 (794 to 1118)	696 (587 to 827)	204 (139 to 297)	42 (19 to 80)	3 (2 to 4)	2421 (2035 to 2864)	1784 (1503 to 2118)	523 (357 to 760)	107 (50 to 205)	8 (6 to 10)
Albania	2046	970 (815 to 1161)	715 (601 to 850)	208 (142 to 302)	43 (20 to 82)	3 (2 to 4)	2484 (2088 to 2974)	1833 (1539 to 2177)	534 (365 to 773)	110 (51 to 211)	8 (6 to 10)
Albania	2047	994 (831 to 1190)	734 (617 to 871)	213 (145 to 307)	44 (21 to 85)	3 (2 to 4)	2546 (2127 to 3047)	1881 (1579 to 2230)	545 (372 to 786)	113 (53 to 217)	8 (6 to 10)
Albania	2048	1018 (851 to 1212)	753 (631 to 893)	217 (148 to 312)	45 (21 to 87)	3 (2 to 4)	2608 (2179 to 3105)	1929 (1617 to 2287)	555 (380 to 799)	116 (54 to 224)	8 (6 to 11)
Albania	2049	1041 (870 to 1249)	771 (646 to 916)	221 (151 to 317)	46 (22 to 90)	3 (2 to 4)	2668 (2229 to 3200)	1975 (1654 to 2347)	566 (387 to 811)	119 (56 to 230)	8 (6 to 11)
Albania	2050	1065 (901 to 1264)	789 (662 to 937)	225 (154 to 321)	47 (22 to 92)	3 (3 to 4)	2727 (2307 to 3239)	2022 (1695 to 2401)	576 (394 to 823)	121 (57 to 236)	8 (6 to 11)
Algeria	1995	101 (85 to 118)	72 (58 to 86)	27 (20 to 36)	2 (1 to 4)	0 (0 to 0)	376 (318 to 439)	267 (215 to 320)	101 (75 to 136)	8 (4 to 16)	0 (0 to 0)
Algeria	1996	99 (84 to 115)	70 (58 to 84)	26 (20 to 35)	2 (1 to 4)	0 (0 to 0)	369 (314 to 427)	263 (216 to 313)	98 (73 to 131)	8 (4 to 16)	0 (0 to 0)
Algeria	1997	101 (87 to 117)	71 (59 to 84)	27 (21 to 36)	2 (1 to 4)	0 (0 to 0)	377 (325 to 436)	266 (221 to 315)	102 (77 to 135)	9 (4 to 16)	0 (0 to 0)
Algeria	1998	103 (89 to 119)	73 (61 to 86)	28 (21 to 37)	2 (1 to 4)	0 (0 to 0)	384 (333 to 442)	271 (228 to 319)	105 (79 to 138)	9 (4 to 16)	0 (0 to 0)
Algeria	1999	105 (92 to 120)	74 (63 to 87)	28 (21 to 37)	2 (1 to 4)	0 (0 to 0)	391 (341 to 445)	277 (237 to 325)	105 (79 to 138)	9 (4 to 16)	0 (0 to 0)

Algeria	2000	106 (93 to 120)	76 (65 to 89)	27 (21 to 35)	2 (1 to 4)	0 (0 to 0)	394 (345 to 447)	283 (243 to 330)	102 (78 to 131)	9 (4 to 16)	0 (0 to 0)
Algeria	2001	112 (99 to 126)	82 (71 to 95)	27 (21 to 35)	3 (1 to 5)	0 (0 to 0)	417 (368 to 471)	305 (263 to 354)	102 (78 to 132)	9 (5 to 17)	0 (0 to 0)
Algeria	2002	119 (105 to 135)	88 (76 to 101)	28 (22 to 37)	3 (1 to 5)	0 (0 to 0)	445 (393 to 502)	329 (283 to 378)	106 (81 to 136)	10 (5 to 18)	0 (0 to 0)
Algeria	2003	120 (106 to 135)	88 (76 to 102)	29 (23 to 37)	3 (1 to 5)	0 (0 to 0)	447 (395 to 504)	328 (284 to 379)	108 (84 to 136)	10 (5 to 19)	0 (0 to 0)
Algeria	2004	120 (106 to 135)	86 (75 to 99)	31 (24 to 39)	3 (1 to 5)	0 (0 to 0)	447 (395 to 504)	321 (278 to 370)	115 (90 to 145)	11 (5 to 19)	0 (0 to 0)
Algeria	2005	118 (105 to 133)	83 (72 to 95)	32 (25 to 41)	3 (1 to 5)	0 (0 to 0)	440 (391 to 495)	309 (269 to 355)	121 (94 to 151)	10 (5 to 19)	0 (0 to 0)
Algeria	2006	127 (113 to 142)	89 (77 to 102)	35 (28 to 44)	3 (1 to 5)	0 (0 to 0)	473 (420 to 531)	330 (286 to 378)	132 (104 to 166)	11 (5 to 19)	0 (0 to 0)
Algeria	2007	144 (128 to 162)	101 (88 to 116)	40 (31 to 49)	3 (1 to 6)	0 (0 to 0)	536 (476 to 603)	376 (328 to 430)	148 (117 to 183)	11 (6 to 21)	0 (0 to 0)
Algeria	2008	166 (148 to 186)	118 (102 to 134)	45 (35 to 56)	3 (2 to 6)	0 (0 to 0)	620 (553 to 692)	440 (382 to 501)	167 (132 to 208)	12 (6 to 22)	1 (1 to 1)
Algeria	2009	185 (166 to 206)	131 (115 to 150)	50 (40 to 63)	3 (2 to 6)	0 (0 to 0)	690 (620 to 768)	490 (428 to 557)	188 (150 to 234)	13 (6 to 23)	0 (0 to 0)
Algeria	2010	189 (170 to 209)	132 (117 to 150)	53 (42 to 65)	3 (2 to 6)	0 (0 to 0)	703 (634 to 781)	494 (435 to 560)	197 (157 to 244)	12 (6 to 22)	0 (0 to 0)
Algeria	2011	210 (189 to 234)	149 (132 to 167)	57 (46 to 71)	3 (2 to 6)	0 (0 to 0)	782 (705 to 874)	555 (491 to 623)	214 (172 to 265)	13 (6 to 24)	1 (1 to 1)
Algeria	2012	235 (212 to 260)	169 (149 to 189)	63 (51 to 77)	4 (2 to 7)	0 (0 to 0)	876 (790 to 969)	628 (557 to 704)	234 (189 to 287)	13 (7 to 26)	0 (0 to 0)
Algeria	2013	226 (204 to 250)	159 (141 to 179)	64 (52 to 79)	3 (2 to 6)	0 (0 to 0)	843 (760 to 931)	593 (524 to 666)	237 (192 to 294)	12 (6 to 23)	1 (1 to 1)
Algeria	2014	253 (227 to 279)	178 (157 to 201)	71 (57 to 88)	4 (2 to 7)	0 (0 to 0)	942 (848 to 1040)	664 (586 to 748)	263 (213 to 328)	14 (7 to 25)	1 (1 to 1)

Algeria	2015	277 (248 to 308)	195 (172 to 220)	78 (63 to 99)	4 (2 to 7)	0 (0 to 0)	1033 (924 to 1146)	727 (639 to 821)	291 (235 to 368)	15 (7 to 28)	0 (0 to 0)
Algeria	2016	270 (239 to 303)	186 (161 to 211)	80 (64 to 103)	4 (2 to 7)	0 (0 to 0)	1007 (890 to 1128)	693 (602 to 787)	300 (239 to 385)	15 (7 to 28)	0 (0 to 0)
Algeria	2017	265 (232 to 304)	179 (153 to 208)	82 (63 to 106)	4 (2 to 7)	0 (0 to 0)	988 (866 to 1133)	668 (570 to 775)	305 (236 to 393)	15 (7 to 27)	0 (0 to 0)
Algeria	2018	262 (228 to 302)	176 (151 to 204)	82 (63 to 106)	4 (2 to 7)	0 (0 to 0)	975 (850 to 1127)	655 (564 to 761)	305 (235 to 393)	15 (7 to 27)	0 (0 to 0)
Algeria	2019	268 (234 to 308)	181 (154 to 213)	82 (64 to 106)	4 (2 to 7)	0 (0 to 0)	997 (871 to 1149)	675 (575 to 794)	307 (237 to 397)	15 (7 to 27)	0 (0 to 0)
Algeria	2020	270 (234 to 310)	183 (155 to 215)	83 (64 to 107)	4 (2 to 7)	0 (0 to 0)	1006 (872 to 1154)	683 (579 to 802)	308 (238 to 398)	15 (7 to 28)	0 (0 to 0)
Algeria	2021	272 (235 to 315)	185 (156 to 218)	83 (64 to 107)	4 (2 to 7)	0 (0 to 0)	1014 (876 to 1174)	691 (582 to 813)	308 (238 to 399)	15 (7 to 28)	0 (0 to 0)
Algeria	2022	274 (235 to 317)	188 (158 to 222)	82 (64 to 107)	4 (2 to 8)	0 (0 to 0)	1021 (877 to 1181)	699 (590 to 827)	307 (238 to 398)	15 (7 to 28)	0 (0 to 0)
Algeria	2023	276 (240 to 319)	190 (159 to 223)	82 (64 to 107)	4 (2 to 8)	0 (0 to 0)	1029 (895 to 1187)	707 (593 to 832)	307 (237 to 399)	15 (7 to 28)	0 (0 to 0)
Algeria	2024	278 (239 to 321)	192 (160 to 226)	82 (64 to 107)	4 (2 to 8)	0 (0 to 0)	1036 (891 to 1195)	715 (598 to 844)	306 (237 to 398)	15 (7 to 28)	0 (0 to 0)
Algeria	2025	280 (240 to 325)	194 (161 to 231)	82 (64 to 106)	4 (2 to 8)	0 (0 to 0)	1045 (894 to 1211)	724 (600 to 862)	306 (237 to 397)	15 (7 to 29)	0 (0 to 0)

Algeria	2026	283 (241 to 329)	197 (162 to 235)	82 (64 to 106)	4 (2 to 8)	0 (0 to 0)	1054 (898 to 1225)	733 (604 to 876)	306 (237 to 396)	15 (7 to 29)	0 (0 to 0)
Algeria	2027	286 (244 to 335)	199 (165 to 236)	82 (63 to 106)	4 (2 to 8)	0 (0 to 0)	1065 (907 to 1247)	743 (614 to 880)	306 (237 to 396)	16 (8 to 29)	0 (0 to 0)
Algeria	2028	289 (245 to 342)	203 (167 to 243)	82 (64 to 106)	4 (2 to 8)	0 (0 to 0)	1078 (912 to 1274)	756 (623 to 904)	307 (237 to 395)	16 (8 to 29)	0 (0 to 0)
Algeria	2029	293 (249 to 343)	206 (169 to 249)	83 (64 to 106)	4 (2 to 8)	0 (0 to 0)	1093 (928 to 1280)	769 (631 to 927)	308 (237 to 396)	16 (8 to 30)	0 (0 to 0)
Algeria	2030	297 (249 to 350)	210 (171 to 253)	83 (64 to 106)	4 (2 to 8)	0 (0 to 0)	1106 (928 to 1304)	781 (637 to 944)	309 (237 to 396)	16 (8 to 30)	0 (0 to 0)
Algeria	2031	301 (255 to 350)	213 (172 to 259)	83 (64 to 107)	4 (2 to 8)	0 (0 to 0)	1122 (950 to 1303)	796 (641 to 964)	310 (238 to 397)	16 (8 to 30)	0 (0 to 0)
Algeria	2032	306 (257 to 359)	218 (175 to 265)	84 (64 to 107)	4 (2 to 8)	0 (0 to 0)	1139 (959 to 1340)	811 (650 to 988)	312 (239 to 399)	16 (8 to 31)	0 (0 to 0)
Algeria	2033	311 (260 to 368)	222 (179 to 272)	84 (64 to 108)	4 (2 to 8)	0 (0 to 0)	1159 (970 to 1370)	828 (666 to 1014)	314 (240 to 403)	17 (8 to 31)	0 (0 to 0)
Algeria	2034	317 (268 to 376)	227 (183 to 279)	85 (65 to 109)	5 (2 to 9)	0 (0 to 0)	1181 (997 to 1400)	847 (681 to 1039)	317 (242 to 407)	17 (8 to 32)	0 (0 to 0)
Algeria	2035	323 (271 to 381)	232 (186 to 286)	86 (66 to 110)	5 (2 to 9)	0 (0 to 0)	1203 (1009 to 1421)	866 (692 to 1066)	320 (244 to 411)	17 (8 to 32)	0 (0 to 0)

Algeria	2036	329 (275 to 392)	238 (191 to 293)	87 (66 to 112)	5 (2 to 9)	0 (0 to 0)	1227 (1025 to 1460)	887 (711 to 1092)	323 (246 to 416)	17 (8 to 33)	0 (0 to 0)
Algeria	2037	336 (280 to 401)	244 (194 to 303)	87 (67 to 113)	5 (2 to 9)	0 (0 to 0)	1251 (1045 to 1495)	908 (725 to 1130)	326 (249 to 421)	18 (8 to 34)	0 (0 to 0)
Algeria	2038	342 (285 to 407)	249 (198 to 309)	88 (67 to 114)	5 (2 to 9)	0 (0 to 0)	1275 (1062 to 1515)	928 (736 to 1152)	329 (251 to 426)	18 (9 to 34)	0 (0 to 0)
Algeria	2039	349 (289 to 416)	255 (202 to 316)	89 (68 to 115)	5 (2 to 9)	0 (0 to 0)	1300 (1076 to 1552)	950 (752 to 1177)	332 (254 to 430)	18 (9 to 35)	0 (0 to 0)
Algeria	2040	355 (296 to 430)	260 (207 to 325)	90 (69 to 117)	5 (2 to 9)	0 (0 to 0)	1324 (1105 to 1604)	970 (770 to 1210)	335 (256 to 436)	19 (9 to 35)	0 (0 to 0)
Algeria	2041	362 (300 to 434)	266 (211 to 331)	91 (69 to 118)	5 (2 to 10)	0 (0 to 0)	1347 (1119 to 1616)	990 (785 to 1235)	338 (258 to 440)	19 (9 to 35)	0 (0 to 0)
Algeria	2042	368 (303 to 442)	271 (213 to 341)	91 (70 to 119)	5 (2 to 10)	0 (0 to 0)	1370 (1129 to 1648)	1011 (795 to 1269)	340 (259 to 444)	19 (9 to 36)	0 (0 to 0)
Algeria	2043	374 (306 to 455)	277 (216 to 350)	92 (70 to 120)	5 (2 to 10)	0 (0 to 0)	1393 (1141 to 1695)	1030 (805 to 1305)	343 (261 to 446)	19 (9 to 36)	0 (0 to 0)
Algeria	2044	379 (309 to 461)	281 (218 to 358)	92 (70 to 120)	5 (3 to 10)	0 (0 to 0)	1413 (1151 to 1718)	1049 (814 to 1333)	344 (262 to 448)	20 (9 to 37)	0 (0 to 0)
Algeria	2045	384 (310 to 471)	286 (223 to 364)	93 (71 to 121)	5 (3 to 10)	0 (0 to 0)	1432 (1156 to 1755)	1066 (830 to 1358)	346 (263 to 450)	20 (9 to 37)	0 (0 to 0)

Algeria	2046	389 (318 to 476)	291 (226 to 371)	93 (71 to 121)	5 (3 to 10)	0 (0 to 0)	1451 (1185 to 1774)	1084 (842 to 1382)	347 (263 to 452)	20 (10 to 37)	0 (0 to 0)
Algeria	2047	394 (321 to 481)	295 (229 to 374)	93 (71 to 122)	5 (3 to 10)	0 (0 to 0)	1467 (1197 to 1792)	1099 (853 to 1395)	348 (263 to 454)	20 (10 to 37)	0 (0 to 0)
Algeria	2048	398 (322 to 490)	299 (232 to 380)	93 (71 to 122)	5 (3 to 10)	0 (0 to 0)	1483 (1201 to 1824)	1114 (864 to 1417)	348 (263 to 454)	20 (10 to 38)	0 (0 to 0)
Algeria	2049	402 (325 to 492)	303 (235 to 384)	93 (71 to 122)	5 (3 to 10)	0 (0 to 0)	1497 (1213 to 1834)	1128 (876 to 1432)	348 (263 to 453)	20 (10 to 38)	0 (0 to 0)
Algeria	2050	405 (325 to 495)	306 (236 to 389)	93 (71 to 122)	6 (3 to 10)	0 (0 to 0)	1510 (1213 to 1844)	1142 (879 to 1450)	348 (263 to 454)	21 (10 to 38)	0 (0 to 0)
American Samoa	1995	806 (646 to 1009)	652 (505 to 840)	141 (95 to 201)	12 (6 to 22)	0 (0 to 0)	806 (646 to 1009)	652 (505 to 840)	141 (95 to 201)	12 (6 to 22)	0 (0 to 0)
American Samoa	1996	812 (666 to 997)	660 (518 to 827)	140 (96 to 202)	12 (6 to 21)	0 (0 to 0)	812 (666 to 997)	660 (518 to 827)	140 (96 to 202)	12 (6 to 21)	0 (0 to 0)
American Samoa	1997	825 (679 to 996)	673 (541 to 829)	140 (96 to 201)	12 (5 to 21)	0 (0 to 0)	825 (679 to 996)	673 (541 to 829)	140 (96 to 201)	12 (5 to 21)	0 (0 to 0)
American Samoa	1998	833 (696 to 990)	682 (559 to 831)	140 (97 to 200)	11 (5 to 21)	0 (0 to 0)	833 (696 to 990)	682 (559 to 831)	140 (97 to 200)	11 (5 to 21)	0 (0 to 0)
American Samoa	1999	844 (716 to 985)	693 (581 to 828)	141 (99 to 203)	10 (5 to 20)	0 (0 to 0)	844 (716 to 985)	693 (581 to 828)	141 (99 to 203)	10 (5 to 20)	0 (0 to 0)
American Samoa	2000	862 (748 to 983)	710 (613 to 825)	143 (100 to 202)	9 (4 to 18)	0 (0 to 0)	862 (748 to 983)	710 (613 to 825)	143 (100 to 202)	9 (4 to 18)	0 (0 to 0)
American Samoa	2001	874 (779 to 974)	721 (642 to 815)	145 (100 to 205)	8 (4 to 15)	0 (0 to 0)	874 (779 to 974)	721 (642 to 815)	145 (100 to 205)	8 (4 to 15)	0 (0 to 0)
American Samoa	2002	888 (806 to 970)	731 (676 to 794)	148 (103 to 210)	9 (4 to 16)	0 (0 to 0)	888 (806 to 970)	731 (676 to 794)	148 (103 to 210)	9 (4 to 16)	0 (0 to 0)

American Samoa	2003	902 (837 to 980)	741 (695 to 790)	152 (106 to 217)	9 (4 to 17)	0 (0 to 0)	902 (837 to 980)	741 (695 to 790)	152 (106 to 217)	9 (4 to 17)	0 (0 to 0)
American Samoa	2004	898 (825 to 983)	733 (675 to 790)	155 (108 to 224)	10 (5 to 19)	0 (0 to 0)	898 (825 to 983)	733 (675 to 790)	155 (108 to 224)	10 (5 to 19)	0 (0 to 0)
American Samoa	2005	879 (789 to 981)	712 (633 to 790)	156 (108 to 224)	11 (5 to 21)	0 (0 to 0)	879 (789 to 981)	712 (633 to 790)	156 (108 to 224)	11 (5 to 21)	0 (0 to 0)
American Samoa	2006	839 (740 to 952)	672 (583 to 765)	154 (107 to 219)	13 (6 to 23)	0 (0 to 0)	839 (740 to 952)	672 (583 to 765)	154 (107 to 219)	13 (6 to 23)	0 (0 to 0)
American Samoa	2007	834 (727 to 955)	663 (567 to 769)	156 (108 to 220)	15 (7 to 28)	0 (0 to 0)	834 (727 to 955)	663 (567 to 769)	156 (108 to 220)	15 (7 to 28)	0 (0 to 0)
American Samoa	2008	805 (701 to 926)	631 (538 to 735)	156 (109 to 218)	19 (9 to 34)	0 (0 to 0)	805 (701 to 926)	631 (538 to 735)	156 (109 to 218)	19 (9 to 34)	0 (0 to 0)
American Samoa	2009	766 (665 to 885)	591 (502 to 685)	154 (107 to 214)	22 (10 to 39)	0 (0 to 0)	766 (665 to 885)	591 (502 to 685)	154 (107 to 214)	22 (10 to 39)	0 (0 to 0)
American Samoa	2010	752 (656 to 857)	573 (498 to 651)	154 (108 to 212)	25 (12 to 46)	0 (0 to 0)	752 (656 to 857)	573 (498 to 651)	154 (108 to 212)	25 (12 to 46)	0 (0 to 0)
American Samoa	2011	739 (653 to 832)	556 (489 to 626)	154 (108 to 212)	28 (13 to 51)	0 (0 to 0)	739 (653 to 832)	556 (489 to 626)	154 (108 to 212)	28 (13 to 51)	0 (0 to 0)
American Samoa	2012	702 (627 to 785)	522 (470 to 577)	150 (104 to 207)	30 (13 to 53)	0 (0 to 0)	702 (627 to 785)	522 (470 to 577)	150 (104 to 207)	30 (13 to 53)	0 (0 to 0)
American Samoa	2013	681 (612 to 757)	502 (458 to 551)	148 (102 to 203)	31 (14 to 55)	0 (0 to 0)	681 (612 to 757)	502 (458 to 551)	148 (102 to 203)	31 (14 to 55)	0 (0 to 0)
American Samoa	2014	688 (611 to 769)	504 (456 to 559)	150 (104 to 207)	34 (16 to 59)	0 (0 to 0)	688 (611 to 769)	504 (456 to 559)	150 (104 to 207)	34 (16 to 59)	0 (0 to 0)
American Samoa	2015	699 (612 to 797)	512 (449 to 587)	151 (105 to 209)	35 (17 to 62)	0 (0 to 0)	699 (612 to 797)	512 (449 to 587)	151 (105 to 209)	35 (17 to 62)	0 (0 to 0)
American Samoa	2016	702 (606 to 823)	515 (437 to 605)	151 (103 to 209)	36 (17 to 65)	0 (0 to 0)	702 (606 to 823)	515 (437 to 605)	151 (103 to 209)	36 (17 to 65)	0 (0 to 0)
American Samoa	2017	694 (572 to 841)	508 (418 to 617)	150 (102 to 209)	36 (17 to 66)	0 (0 to 0)	694 (572 to 841)	508 (418 to 617)	150 (102 to 209)	36 (17 to 66)	0 (0 to 0)

American Samoa	2018	699 (575 to 841)	514 (420 to 626)	149 (101 to 208)	36 (17 to 66)	0 (0 to 0)	699 (575 to 841)	514 (420 to 626)	149 (101 to 208)	36 (17 to 66)	0 (0 to 0)
American Samoa	2019	697 (568 to 857)	514 (418 to 626)	147 (100 to 206)	36 (17 to 66)	0 (0 to 0)	697 (568 to 857)	514 (418 to 626)	147 (100 to 206)	36 (17 to 66)	0 (0 to 0)
American Samoa	2020	698 (576 to 837)	515 (422 to 627)	147 (100 to 205)	36 (17 to 66)	0 (0 to 0)	698 (576 to 837)	515 (422 to 627)	147 (100 to 205)	36 (17 to 66)	0 (0 to 0)
American Samoa	2021	704 (572 to 864)	520 (424 to 635)	148 (100 to 206)	36 (17 to 67)	0 (0 to 0)	704 (572 to 864)	520 (424 to 635)	148 (100 to 206)	36 (17 to 67)	0 (0 to 0)
American Samoa	2022	709 (580 to 854)	524 (428 to 645)	149 (101 to 207)	36 (17 to 67)	0 (0 to 0)	709 (580 to 854)	524 (428 to 645)	149 (101 to 207)	36 (17 to 67)	0 (0 to 0)
American Samoa	2023	713 (576 to 874)	527 (428 to 655)	149 (101 to 207)	37 (17 to 67)	0 (0 to 0)	713 (576 to 874)	527 (428 to 655)	149 (101 to 207)	37 (17 to 67)	0 (0 to 0)
American Samoa	2024	715 (583 to 866)	530 (429 to 650)	148 (100 to 206)	37 (17 to 68)	0 (0 to 0)	715 (583 to 866)	530 (429 to 650)	148 (100 to 206)	37 (17 to 68)	0 (0 to 0)
American Samoa	2025	717 (578 to 878)	532 (431 to 657)	148 (100 to 206)	37 (18 to 68)	0 (0 to 0)	717 (578 to 878)	532 (431 to 657)	148 (100 to 206)	37 (18 to 68)	0 (0 to 0)
American Samoa	2026	718 (583 to 874)	533 (429 to 656)	147 (100 to 205)	37 (18 to 69)	0 (0 to 0)	718 (583 to 874)	533 (429 to 656)	147 (100 to 205)	37 (18 to 69)	0 (0 to 0)
American Samoa	2027	717 (579 to 878)	534 (431 to 662)	147 (99 to 204)	37 (18 to 69)	0 (0 to 0)	717 (579 to 878)	534 (431 to 662)	147 (99 to 204)	37 (18 to 69)	0 (0 to 0)
American Samoa	2028	716 (579 to 875)	533 (429 to 657)	146 (99 to 203)	37 (18 to 69)	0 (0 to 0)	716 (579 to 875)	533 (429 to 657)	146 (99 to 203)	37 (18 to 69)	0 (0 to 0)
American Samoa	2029	714 (578 to 879)	532 (425 to 656)	145 (98 to 202)	37 (18 to 69)	0 (0 to 0)	714 (578 to 879)	532 (425 to 656)	145 (98 to 202)	37 (18 to 69)	0 (0 to 0)
American Samoa	2030	711 (573 to 869)	531 (422 to 657)	143 (97 to 200)	37 (18 to 69)	0 (0 to 0)	711 (573 to 869)	531 (422 to 657)	143 (97 to 200)	37 (18 to 69)	0 (0 to 0)
American Samoa	2031	708 (569 to 878)	529 (417 to 654)	142 (96 to 198)	37 (18 to 69)	0 (0 to 0)	708 (569 to 878)	529 (417 to 654)	142 (96 to 198)	37 (18 to 69)	0 (0 to 0)
American Samoa	2032	705 (561 to 867)	528 (413 to 655)	141 (95 to 196)	37 (17 to 69)	0 (0 to 0)	705 (561 to 867)	528 (413 to 655)	141 (95 to 196)	37 (17 to 69)	0 (0 to 0)

American Samoa	2033	702 (564 to 870)	526 (412 to 652)	139 (94 to 194)	37 (17 to 69)	0 (0 to 0)	702 (564 to 870)	526 (412 to 652)	139 (94 to 194)	37 (17 to 69)	0 (0 to 0)
American Samoa	2034	699 (557 to 866)	524 (406 to 649)	138 (93 to 192)	37 (17 to 69)	0 (0 to 0)	699 (557 to 866)	524 (406 to 649)	138 (93 to 192)	37 (17 to 69)	0 (0 to 0)
American Samoa	2035	696 (555 to 862)	523 (408 to 648)	137 (92 to 190)	37 (17 to 69)	0 (0 to 0)	696 (555 to 862)	523 (408 to 648)	137 (92 to 190)	37 (17 to 69)	0 (0 to 0)
American Samoa	2036	694 (549 to 858)	522 (403 to 651)	136 (91 to 189)	37 (17 to 69)	0 (0 to 0)	694 (549 to 858)	522 (403 to 651)	136 (91 to 189)	37 (17 to 69)	0 (0 to 0)
American Samoa	2037	692 (549 to 863)	521 (402 to 652)	135 (91 to 187)	37 (17 to 69)	0 (0 to 0)	692 (549 to 863)	521 (402 to 652)	135 (91 to 187)	37 (17 to 69)	0 (0 to 0)
American Samoa	2038	691 (546 to 858)	521 (401 to 655)	134 (90 to 186)	37 (17 to 69)	0 (0 to 0)	691 (546 to 858)	521 (401 to 655)	134 (90 to 186)	37 (17 to 69)	0 (0 to 0)
American Samoa	2039	690 (541 to 865)	521 (403 to 656)	133 (89 to 184)	37 (17 to 69)	0 (0 to 0)	690 (541 to 865)	521 (403 to 656)	133 (89 to 184)	37 (17 to 69)	0 (0 to 0)
American Samoa	2040	690 (541 to 866)	522 (401 to 658)	132 (89 to 183)	37 (17 to 69)	0 (0 to 0)	690 (541 to 866)	522 (401 to 658)	132 (89 to 183)	37 (17 to 69)	0 (0 to 0)
American Samoa	2041	691 (542 to 863)	523 (403 to 661)	131 (88 to 182)	37 (17 to 70)	0 (0 to 0)	691 (542 to 863)	523 (403 to 661)	131 (88 to 182)	37 (17 to 70)	0 (0 to 0)
American Samoa	2042	692 (542 to 870)	525 (407 to 669)	131 (88 to 182)	37 (17 to 70)	0 (0 to 0)	692 (542 to 870)	525 (407 to 669)	131 (88 to 182)	37 (17 to 70)	0 (0 to 0)
American Samoa	2043	694 (546 to 876)	527 (407 to 669)	130 (88 to 181)	37 (17 to 70)	0 (0 to 0)	694 (546 to 876)	527 (407 to 669)	130 (88 to 181)	37 (17 to 70)	0 (0 to 0)
American Samoa	2044	697 (540 to 876)	529 (407 to 678)	130 (88 to 181)	37 (17 to 71)	0 (0 to 0)	697 (540 to 876)	529 (407 to 678)	130 (88 to 181)	37 (17 to 71)	0 (0 to 0)
American Samoa	2045	699 (543 to 887)	532 (406 to 677)	130 (87 to 181)	38 (17 to 72)	0 (0 to 0)	699 (543 to 887)	532 (406 to 677)	130 (87 to 181)	38 (17 to 72)	0 (0 to 0)
American Samoa	2046	702 (542 to 884)	535 (407 to 686)	129 (87 to 181)	38 (18 to 72)	0 (0 to 0)	702 (542 to 884)	535 (407 to 686)	129 (87 to 181)	38 (18 to 72)	0 (0 to 0)
American Samoa	2047	706 (546 to 894)	539 (407 to 690)	129 (87 to 181)	38 (18 to 73)	0 (0 to 0)	706 (546 to 894)	539 (407 to 690)	129 (87 to 181)	38 (18 to 73)	0 (0 to 0)

American Samoa	2048	710 (550 to 897)	542 (408 to 699)	129 (87 to 181)	38 (18 to 74)	0 (0 to 0)	710 (550 to 897)	542 (408 to 699)	129 (87 to 181)	38 (18 to 74)	0 (0 to 0)
American Samoa	2049	714 (552 to 908)	547 (413 to 705)	129 (87 to 181)	39 (18 to 75)	0 (0 to 0)	714 (552 to 908)	547 (413 to 705)	129 (87 to 181)	39 (18 to 75)	0 (0 to 0)
American Samoa	2050	719 (551 to 912)	551 (414 to 712)	129 (87 to 180)	39 (18 to 75)	0 (0 to 0)	719 (551 to 912)	551 (414 to 712)	129 (87 to 180)	39 (18 to 75)	0 (0 to 0)
Andorra	1995	3516 (3370 to 3668)	1505 (1394 to 1619)	1628 (1536 to 1719)	382 (333 to 434)	0 (0 to 0)	7603 (7287 to 7933)	3256 (3015 to 3500)	3520 (3322 to 3716)	827 (719 to 939)	0 (0 to 0)
Andorra	1996	3601 (3487 to 3711)	1531 (1447 to 1617)	1665 (1602 to 1732)	406 (371 to 445)	0 (0 to 0)	7788 (7540 to 8026)	3310 (3129 to 3496)	3601 (3464 to 3746)	877 (803 to 962)	0 (0 to 0)
Andorra	1997	3829 (3718 to 3939)	1627 (1550 to 1705)	1754 (1688 to 1821)	448 (410 to 485)	0 (0 to 0)	8280 (8041 to 8519)	3518 (3352 to 3686)	3794 (3651 to 3937)	968 (888 to 1048)	0 (0 to 0)
Andorra	1998	3952 (3846 to 4058)	1675 (1597 to 1751)	1808 (1744 to 1872)	469 (432 to 508)	0 (0 to 0)	8547 (8317 to 8775)	3621 (3453 to 3788)	3910 (3772 to 4047)	1015 (935 to 1099)	0 (0 to 0)
Andorra	1999	3951 (3846 to 4055)	1662 (1590 to 1739)	1845 (1776 to 1910)	444 (409 to 482)	0 (0 to 0)	8545 (8318 to 8770)	3594 (3438 to 3761)	3990 (3840 to 4130)	961 (884 to 1042)	0 (0 to 0)
Andorra	2000	3934 (3826 to 4042)	1643 (1571 to 1724)	1872 (1802 to 1943)	419 (389 to 454)	0 (0 to 0)	8507 (8274 to 8740)	3553 (3396 to 3728)	4049 (3897 to 4202)	906 (840 to 982)	0 (0 to 0)
Andorra	2001	4001 (3891 to 4107)	1652 (1575 to 1727)	1929 (1854 to 1998)	420 (391 to 451)	0 (0 to 0)	8652 (8414 to 8882)	3573 (3407 to 3735)	4172 (4009 to 4321)	907 (845 to 975)	0 (0 to 0)
Andorra	2002	4060 (3955 to 4163)	1628 (1553 to 1703)	1997 (1930 to 2063)	434 (405 to 464)	0 (0 to 0)	8780 (8552 to 9004)	3521 (3359 to 3683)	4319 (4174 to 4462)	940 (875 to 1004)	0 (0 to 0)

Andorra	2003	4083 (3983 to 4189)	1577 (1502 to 1650)	2054 (1982 to 2122)	451 (420 to 482)	0 (0 to 0)	8829 (8612 to 9058)	3411 (3249 to 3568)	4442 (4287 to 4588)	976 (908 to 1043)	0 (0 to 0)
Andorra	2004	4080 (3975 to 4182)	1525 (1459 to 1600)	2093 (2026 to 2158)	462 (429 to 492)	0 (0 to 0)	8823 (8597 to 9043)	3299 (3155 to 3460)	4526 (4382 to 4668)	999 (928 to 1065)	0 (0 to 0)
Andorra	2005	4255 (4145 to 4361)	1591 (1521 to 1664)	2177 (2106 to 2245)	487 (454 to 518)	0 (0 to 0)	9202 (8964 to 9431)	3440 (3290 to 3598)	4709 (4555 to 4854)	1053 (981 to 1121)	0 (0 to 0)
Andorra	2006	4237 (4131 to 4339)	1593 (1522 to 1665)	2162 (2090 to 2230)	482 (451 to 513)	0 (0 to 0)	9164 (8933 to 9384)	3445 (3291 to 3600)	4676 (4521 to 4822)	1042 (975 to 1109)	0 (0 to 0)
Andorra	2007	4130 (4024 to 4229)	1597 (1526 to 1672)	2073 (2007 to 2146)	460 (428 to 493)	0 (0 to 0)	8931 (8701 to 9146)	3453 (3300 to 3615)	4483 (4340 to 4642)	995 (926 to 1067)	0 (0 to 0)
Andorra	2008	3977 (3876 to 4078)	1621 (1554 to 1697)	1929 (1864 to 1996)	426 (398 to 457)	0 (0 to 0)	8600 (8382 to 8818)	3506 (3360 to 3669)	4172 (4032 to 4316)	922 (862 to 989)	0 (0 to 0)
Andorra	2009	3963 (3855 to 4067)	1709 (1635 to 1785)	1845 (1779 to 1909)	408 (380 to 438)	0 (0 to 0)	8569 (8337 to 8794)	3696 (3535 to 3861)	3990 (3846 to 4129)	883 (821 to 946)	0 (0 to 0)
Andorra	2010	4003 (3896 to 4110)	1800 (1725 to 1880)	1801 (1738 to 1866)	402 (372 to 432)	0 (0 to 0)	8656 (8425 to 8887)	3893 (3730 to 4065)	3896 (3758 to 4034)	868 (804 to 933)	0 (0 to 0)
Andorra	2011	4064 (3958 to 4171)	1866 (1795 to 1941)	1799 (1733 to 1862)	399 (370 to 431)	0 (0 to 0)	8788 (8560 to 9020)	4035 (3883 to 4197)	3889 (3748 to 4026)	864 (801 to 932)	0 (0 to 0)
Andorra	2012	4177 (4074 to 4277)	1944 (1870 to 2022)	1829 (1765 to 1897)	404 (376 to 434)	0 (0 to 0)	9032 (8810 to 9248)	4204 (4044 to 4372)	3955 (3817 to 4102)	873 (814 to 940)	0 (0 to 0)

Andorra	2013	4297 (4195 to 4402)	2026 (1951 to 2108)	1861 (1800 to 1927)	410 (382 to 441)	0 (0 to 0)	9292 (9072 to 9519)	4381 (4219 to 4559)	4024 (3893 to 4167)	887 (825 to 954)	0 (0 to 0)
Andorra	2014	4322 (4219 to 4432)	2055 (1983 to 2132)	1863 (1802 to 1927)	404 (376 to 435)	0 (0 to 0)	9347 (9123 to 9583)	4444 (4288 to 4610)	4028 (3897 to 4168)	875 (813 to 940)	0 (0 to 0)
Andorra	2015	4336 (4227 to 4446)	2086 (2008 to 2166)	1850 (1787 to 1912)	400 (372 to 430)	0 (0 to 0)	9377 (9141 to 9615)	4512 (4342 to 4685)	4000 (3864 to 4135)	865 (805 to 930)	0 (0 to 0)
Andorra	2016	4531 (4416 to 4649)	2230 (2146 to 2319)	1882 (1818 to 1951)	418 (388 to 451)	0 (0 to 0)	9798 (9550 to 10054)	4823 (4640 to 5015)	4071 (3931 to 4219)	905 (839 to 974)	0 (0 to 0)
Andorra	2017	4491 (4310 to 4675)	2222 (2108 to 2336)	1860 (1766 to 1958)	409 (368 to 451)	0 (0 to 0)	9712 (9320 to 10109)	4806 (4559 to 5052)	4023 (3820 to 4234)	883 (796 to 976)	0 (0 to 0)
Andorra	2018	4556 (4349 to 4760)	2257 (2114 to 2399)	1880 (1785 to 1979)	419 (377 to 462)	0 (0 to 0)	9853 (9404 to 10294)	4880 (4572 to 5188)	4067 (3859 to 4279)	906 (816 to 1000)	0 (0 to 0)
Andorra	2019	4597 (4379 to 4817)	2293 (2147 to 2453)	1881 (1785 to 1981)	422 (380 to 466)	0 (0 to 0)	9940 (9469 to 10418)	4959 (4644 to 5304)	4069 (3861 to 4284)	913 (822 to 1008)	0 (0 to 0)
Andorra	2020	4674 (4440 to 4909)	2345 (2178 to 2517)	1901 (1806 to 2001)	428 (386 to 474)	0 (0 to 0)	10107 (9602 to 10615)	5070 (4711 to 5443)	4110 (3905 to 4328)	926 (835 to 1024)	0 (0 to 0)
Andorra	2021	4719 (4469 to 4972)	2386 (2205 to 2572)	1903 (1805 to 2001)	431 (387 to 477)	0 (0 to 0)	10206 (9664 to 10753)	5160 (4768 to 5563)	4115 (3903 to 4327)	931 (838 to 1031)	0 (0 to 0)
Andorra	2022	4762 (4497 to 5028)	2426 (2224 to 2627)	1903 (1805 to 2004)	432 (389 to 479)	0 (0 to 0)	10298 (9725 to 10873)	5247 (4810 to 5681)	4116 (3904 to 4334)	935 (841 to 1036)	0 (0 to 0)

Andorra	2023	4806 (4534 to 5091)	2470 (2267 to 2688)	1903 (1805 to 2005)	433 (390 to 481)	0 (0 to 0)	10393 (9804 to 11009)	5342 (4903 to 5813)	4115 (3904 to 4336)	937 (844 to 1041)	0 (0 to 0)
Andorra	2024	4848 (4562 to 5150)	2515 (2291 to 2740)	1900 (1803 to 2004)	434 (390 to 483)	0 (0 to 0)	10485 (9864 to 11138)	5438 (4954 to 5925)	4109 (3900 to 4334)	938 (844 to 1045)	0 (0 to 0)
Andorra	2025	4893 (4592 to 5208)	2562 (2325 to 2811)	1897 (1797 to 2006)	434 (390 to 483)	0 (0 to 0)	10582 (9930 to 11263)	5540 (5028 to 6078)	4103 (3886 to 4339)	939 (844 to 1044)	0 (0 to 0)
Andorra	2026	4937 (4631 to 5263)	2609 (2365 to 2872)	1894 (1792 to 2009)	434 (391 to 483)	0 (0 to 0)	10677 (10014 to 11381)	5642 (5115 to 6212)	4096 (3876 to 4344)	939 (845 to 1045)	0 (0 to 0)
Andorra	2027	4984 (4666 to 5331)	2659 (2405 to 2953)	1890 (1786 to 2007)	434 (390 to 484)	0 (0 to 0)	10778 (10090 to 11529)	5751 (5201 to 6387)	4088 (3863 to 4340)	939 (844 to 1047)	0 (0 to 0)
Andorra	2028	5031 (4690 to 5409)	2709 (2424 to 3024)	1887 (1778 to 2003)	435 (390 to 484)	0 (0 to 0)	10880 (10141 to 11698)	5859 (5241 to 6539)	4081 (3846 to 4332)	940 (843 to 1046)	0 (0 to 0)
Andorra	2029	5078 (4727 to 5471)	2760 (2459 to 3084)	1883 (1771 to 2005)	435 (390 to 484)	0 (0 to 0)	10981 (10223 to 11831)	5969 (5317 to 6669)	4072 (3830 to 4335)	940 (843 to 1047)	0 (0 to 0)
Andorra	2030	5125 (4766 to 5519)	2813 (2504 to 3139)	1878 (1763 to 2007)	434 (389 to 484)	0 (0 to 0)	11083 (10308 to 11936)	6083 (5416 to 6787)	4062 (3812 to 4341)	939 (841 to 1047)	0 (0 to 0)
Andorra	2031	5169 (4781 to 5587)	2864 (2535 to 3212)	1871 (1752 to 2006)	433 (388 to 485)	0 (0 to 0)	11178 (10339 to 12083)	6194 (5482 to 6945)	4047 (3789 to 4339)	937 (839 to 1049)	0 (0 to 0)

Andorra	2032	5198 (4799 to 5630)	2906 (2573 to 3270)	1860 (1739 to 1998)	432 (386 to 484)	0 (0 to 0)	11241 (10378 to 12176)	6284 (5564 to 7072)	4023 (3760 to 4322)	934 (835 to 1047)	0 (0 to 0)
Andorra	2033	5222 (4821 to 5679)	2944 (2600 to 3351)	1848 (1724 to 1991)	430 (384 to 482)	0 (0 to 0)	11294 (10426 to 12282)	6367 (5623 to 7246)	3997 (3727 to 4305)	930 (830 to 1042)	0 (0 to 0)
Andorra	2034	5238 (4835 to 5712)	2978 (2619 to 3383)	1833 (1706 to 1981)	428 (381 to 479)	0 (0 to 0)	11328 (10455 to 12353)	6439 (5664 to 7315)	3965 (3688 to 4285)	925 (825 to 1037)	0 (0 to 0)
Andorra	2035	5247 (4812 to 5733)	3006 (2627 to 3399)	1816 (1684 to 1968)	425 (378 to 476)	0 (0 to 0)	11347 (10407 to 12398)	6501 (5680 to 7351)	3927 (3641 to 4257)	918 (818 to 1030)	0 (0 to 0)
Andorra	2036	5248 (4805 to 5751)	3031 (2638 to 3437)	1796 (1658 to 1955)	421 (375 to 473)	0 (0 to 0)	11349 (10390 to 12436)	6554 (5704 to 7433)	3884 (3585 to 4228)	911 (810 to 1024)	0 (0 to 0)
Andorra	2037	5231 (4778 to 5742)	3043 (2638 to 3470)	1772 (1630 to 1937)	417 (370 to 470)	0 (0 to 0)	11312 (10333 to 12416)	6580 (5705 to 7503)	3831 (3525 to 4189)	901 (801 to 1016)	0 (0 to 0)
Andorra	2038	5238 (4786 to 5747)	3069 (2669 to 3493)	1755 (1608 to 1925)	414 (367 to 468)	0 (0 to 0)	11327 (10351 to 12428)	6638 (5771 to 7553)	3795 (3477 to 4164)	895 (794 to 1013)	0 (0 to 0)
Andorra	2039	5243 (4782 to 5775)	3093 (2664 to 3547)	1738 (1586 to 1916)	411 (364 to 466)	0 (0 to 0)	11337 (10340 to 12488)	6690 (5760 to 7671)	3758 (3429 to 4143)	889 (787 to 1008)	0 (0 to 0)

Andorra	2040	5239 (4743 to 5774)	3112 (2682 to 3568)	1719 (1562 to 1901)	408 (361 to 464)	0 (0 to 0)	11330 (10257 to 12486)	6731 (5800 to 7716)	3717 (3378 to 4111)	883 (780 to 1004)	0 (0 to 0)
Andorra	2041	5235 (4731 to 5774)	3129 (2688 to 3606)	1701 (1540 to 1886)	405 (357 to 462)	0 (0 to 0)	11321 (10230 to 12485)	6767 (5812 to 7799)	3677 (3329 to 4079)	876 (773 to 998)	0 (0 to 0)
Andorra	2042	5226 (4711 to 5788)	3142 (2685 to 3628)	1682 (1517 to 1870)	402 (354 to 459)	0 (0 to 0)	11302 (10188 to 12517)	6795 (5806 to 7846)	3637 (3281 to 4043)	870 (765 to 994)	0 (0 to 0)
Andorra	2043	5221 (4711 to 5791)	3156 (2696 to 3661)	1664 (1497 to 1856)	400 (350 to 457)	0 (0 to 0)	11290 (10188 to 12522)	6825 (5830 to 7918)	3600 (3237 to 4014)	865 (758 to 989)	0 (0 to 0)
Andorra	2044	5210 (4689 to 5802)	3166 (2708 to 3690)	1647 (1476 to 1842)	398 (347 to 455)	0 (0 to 0)	11268 (10140 to 12547)	6847 (5857 to 7980)	3561 (3191 to 3983)	860 (751 to 984)	0 (0 to 0)
Andorra	2045	5203 (4675 to 5816)	3177 (2710 to 3705)	1631 (1457 to 1829)	396 (345 to 454)	0 (0 to 0)	11251 (10109 to 12576)	6870 (5861 to 8013)	3526 (3150 to 3955)	856 (745 to 983)	0 (0 to 0)
Andorra	2046	5194 (4652 to 5817)	3185 (2718 to 3721)	1615 (1439 to 1816)	394 (342 to 454)	0 (0 to 0)	11233 (10061 to 12579)	6888 (5877 to 8046)	3493 (3112 to 3927)	852 (740 to 983)	0 (0 to 0)
Andorra	2047	5189 (4637 to 5832)	3193 (2711 to 3743)	1602 (1425 to 1806)	393 (341 to 454)	0 (0 to 0)	11221 (10029 to 12612)	6905 (5862 to 8094)	3465 (3082 to 3905)	850 (737 to 982)	0 (0 to 0)

Andorra	2048	5187 (4620 to 5828)	3202 (2723 to 3779)	1592 (1415 to 1796)	393 (340 to 454)	0 (0 to 0)	11218 (9991 to 12603)	6925 (5889 to 8172)	3442 (3060 to 3884)	850 (735 to 983)	0 (0 to 0)
Andorra	2049	5193 (4636 to 5846)	3215 (2728 to 3777)	1584 (1407 to 1789)	394 (340 to 457)	0 (0 to 0)	11229 (10027 to 12642)	6952 (5899 to 8167)	3425 (3042 to 3869)	852 (736 to 988)	0 (0 to 0)
Andorra	2050	5198 (4635 to 5865)	3225 (2720 to 3808)	1577 (1399 to 1784)	395 (341 to 459)	0 (0 to 0)	11241 (10023 to 12684)	6975 (5882 to 8235)	3411 (3025 to 3857)	855 (737 to 992)	0 (0 to 0)
Angola	1995	95 (79 to 115)	41 (32 to 51)	31 (23 to 42)	20 (10 to 34)	4 (4 to 4)	216 (179 to 260)	92 (72 to 116)	70 (51 to 96)	44 (23 to 76)	9 (9 to 9)
Angola	1996	74 (62 to 88)	32 (25 to 40)	20 (15 to 28)	14 (7 to 24)	7 (7 to 7)	168 (141 to 198)	73 (57 to 91)	46 (33 to 63)	32 (17 to 55)	17 (17 to 17)
Angola	1997	68 (57 to 81)	32 (25 to 40)	20 (15 to 28)	12 (6 to 21)	3 (3 to 3)	154 (129 to 183)	73 (58 to 91)	46 (33 to 62)	27 (14 to 47)	8 (8 to 8)
Angola	1998	63 (53 to 75)	30 (24 to 37)	21 (15 to 28)	10 (6 to 17)	2 (2 to 2)	142 (120 to 169)	68 (55 to 84)	47 (34 to 63)	23 (13 to 39)	4 (4 to 4)
Angola	1999	72 (61 to 85)	37 (30 to 46)	22 (16 to 29)	11 (6 to 19)	2 (2 to 2)	163 (137 to 192)	84 (67 to 103)	50 (37 to 66)	25 (14 to 42)	5 (5 to 5)
Angola	2000	66 (56 to 77)	33 (26 to 40)	23 (17 to 30)	8 (5 to 15)	2 (2 to 2)	150 (126 to 175)	74 (59 to 91)	51 (38 to 68)	19 (10 to 33)	5 (5 to 5)
Angola	2001	75 (64 to 89)	34 (27 to 42)	31 (23 to 41)	8 (4 to 14)	3 (3 to 3)	171 (144 to 201)	77 (62 to 94)	70 (52 to 93)	18 (10 to 31)	6 (6 to 6)
Angola	2002	80 (68 to 95)	34 (28 to 42)	35 (26 to 47)	8 (4 to 14)	2 (2 to 2)	181 (154 to 215)	78 (62 to 95)	80 (59 to 107)	18 (9 to 31)	6 (6 to 6)
Angola	2003	86 (73 to 102)	37 (30 to 45)	38 (28 to 51)	8 (4 to 14)	3 (3 to 3)	195 (166 to 231)	84 (68 to 103)	86 (65 to 115)	19 (10 to 33)	6 (6 to 6)
Angola	2004	85 (72 to 100)	38 (30 to 46)	37 (27 to 49)	8 (4 to 14)	3 (3 to 3)	193 (164 to 227)	85 (68 to 104)	83 (62 to 112)	18 (10 to 31)	7 (7 to 7)

Angola	2005	86 (73 to 100)	38 (31 to 46)	32 (24 to 43)	8 (4 to 13)	7 (7 to 7)	194 (165 to 226)	87 (70 to 105)	73 (55 to 97)	18 (10 to 30)	16 (16 to 16)
Angola	2006	89 (76 to 103)	47 (38 to 57)	30 (22 to 40)	9 (5 to 16)	3 (3 to 3)	202 (171 to 234)	106 (86 to 129)	68 (51 to 90)	21 (11 to 36)	7 (7 to 7)
Angola	2007	105 (90 to 121)	59 (49 to 72)	29 (22 to 39)	12 (6 to 20)	4 (4 to 4)	238 (204 to 275)	135 (110 to 164)	67 (49 to 89)	27 (14 to 44)	9 (9 to 9)
Angola	2008	132 (114 to 153)	81 (67 to 100)	30 (22 to 39)	16 (9 to 28)	5 (5 to 5)	300 (259 to 347)	183 (151 to 225)	67 (50 to 89)	37 (20 to 63)	12 (12 to 12)
Angola	2009	113 (96 to 131)	70 (57 to 86)	24 (17 to 31)	15 (8 to 25)	4 (4 to 4)	256 (219 to 297)	158 (129 to 194)	54 (39 to 71)	34 (18 to 56)	10 (10 to 10)
Angola	2010	103 (88 to 121)	64 (52 to 77)	21 (15 to 28)	14 (8 to 24)	4 (4 to 4)	234 (199 to 273)	144 (118 to 175)	48 (35 to 63)	33 (18 to 55)	9 (9 to 9)
Angola	2011	101 (86 to 118)	63 (51 to 77)	20 (15 to 27)	15 (8 to 24)	3 (3 to 3)	229 (196 to 267)	143 (116 to 175)	46 (34 to 61)	33 (19 to 55)	7 (7 to 7)
Angola	2012	101 (87 to 118)	61 (50 to 75)	21 (15 to 27)	15 (8 to 24)	4 (4 to 4)	229 (197 to 267)	139 (113 to 169)	47 (34 to 61)	34 (19 to 55)	10 (10 to 10)
Angola	2013	104 (89 to 121)	61 (49 to 74)	23 (17 to 31)	16 (9 to 26)	5 (5 to 5)	236 (203 to 275)	137 (111 to 168)	53 (38 to 69)	36 (20 to 58)	11 (11 to 11)
Angola	2014	103 (88 to 121)	57 (46 to 70)	26 (19 to 34)	17 (9 to 27)	3 (3 to 3)	232 (199 to 273)	129 (104 to 157)	59 (44 to 78)	38 (21 to 61)	6 (6 to 6)
Angola	2015	92 (79 to 110)	46 (37 to 56)	28 (20 to 36)	15 (8 to 25)	3 (3 to 3)	209 (178 to 248)	105 (85 to 128)	62 (46 to 82)	34 (18 to 56)	8 (8 to 8)
Angola	2016	87 (74 to 103)	40 (32 to 50)	28 (20 to 38)	14 (8 to 24)	4 (4 to 4)	196 (167 to 233)	91 (73 to 113)	64 (46 to 85)	32 (17 to 54)	9 (9 to 9)
Angola	2017	90 (73 to 112)	42 (33 to 53)	30 (22 to 41)	15 (8 to 26)	4 (4 to 4)	205 (166 to 254)	95 (76 to 119)	67 (49 to 92)	34 (18 to 58)	9 (9 to 9)
Angola	2018	79 (64 to 97)	34 (26 to 42)	29 (21 to 40)	13 (7 to 22)	4 (4 to 4)	179 (144 to 221)	76 (59 to 96)	65 (47 to 90)	29 (16 to 50)	9 (8 to 9)
Angola	2019	76 (61 to 95)	32 (25 to 41)	28 (20 to 39)	12 (7 to 21)	4 (4 to 4)	173 (139 to 215)	72 (57 to 93)	64 (46 to 87)	28 (15 to 48)	9 (9 to 9)

Angola	2020	75 (60 to 94)	31 (24 to 40)	28 (20 to 38)	12 (7 to 21)	4 (4 to 4)	170 (136 to 212)	71 (55 to 92)	63 (46 to 86)	27 (15 to 47)	9 (8 to 9)
Angola	2021	75 (61 to 93)	31 (24 to 41)	28 (20 to 38)	12 (7 to 21)	4 (4 to 4)	170 (137 to 211)	71 (55 to 92)	63 (46 to 86)	28 (15 to 48)	9 (8 to 9)
Angola	2022	76 (60 to 94)	32 (24 to 41)	28 (20 to 38)	12 (7 to 21)	4 (4 to 4)	171 (137 to 214)	72 (55 to 93)	63 (46 to 86)	28 (15 to 48)	9 (8 to 10)
Angola	2023	76 (61 to 96)	32 (25 to 41)	28 (20 to 38)	12 (7 to 21)	4 (3 to 4)	172 (138 to 216)	72 (56 to 94)	63 (46 to 87)	28 (15 to 49)	9 (8 to 10)
Angola	2024	77 (61 to 96)	32 (25 to 42)	28 (21 to 38)	13 (7 to 22)	4 (3 to 4)	174 (139 to 218)	73 (57 to 95)	64 (46 to 87)	28 (15 to 49)	9 (8 to 10)
Angola	2025	77 (61 to 97)	33 (25 to 42)	28 (21 to 39)	13 (7 to 22)	4 (3 to 4)	175 (139 to 220)	74 (56 to 96)	64 (47 to 88)	29 (16 to 50)	9 (8 to 10)
Angola	2026	78 (61 to 98)	33 (25 to 43)	29 (21 to 39)	13 (7 to 22)	4 (3 to 4)	177 (139 to 223)	75 (56 to 98)	65 (47 to 88)	29 (16 to 50)	9 (8 to 10)
Angola	2027	79 (62 to 100)	33 (25 to 44)	29 (21 to 39)	13 (7 to 22)	4 (3 to 4)	179 (140 to 226)	75 (56 to 101)	65 (47 to 89)	30 (16 to 51)	8 (7 to 10)
Angola	2028	80 (63 to 101)	34 (25 to 46)	29 (21 to 40)	13 (7 to 23)	4 (3 to 4)	181 (142 to 229)	76 (56 to 103)	66 (48 to 90)	30 (16 to 51)	8 (7 to 10)
Angola	2029	81 (63 to 103)	34 (25 to 46)	30 (21 to 40)	14 (7 to 23)	4 (3 to 4)	183 (143 to 233)	77 (56 to 104)	67 (49 to 91)	31 (17 to 52)	8 (7 to 10)
Angola	2030	82 (64 to 105)	35 (25 to 47)	30 (22 to 41)	14 (7 to 24)	4 (3 to 4)	186 (145 to 238)	78 (56 to 107)	68 (49 to 93)	31 (17 to 53)	8 (7 to 10)
Angola	2031	83 (65 to 108)	35 (25 to 49)	30 (22 to 42)	14 (8 to 24)	4 (3 to 4)	189 (147 to 244)	80 (57 to 111)	69 (50 to 94)	32 (17 to 54)	8 (7 to 10)
Angola	2032	85 (66 to 110)	36 (25 to 50)	31 (22 to 42)	14 (8 to 25)	4 (3 to 4)	193 (150 to 249)	81 (57 to 114)	70 (51 to 96)	33 (18 to 56)	8 (7 to 10)
Angola	2033	87 (67 to 113)	37 (26 to 51)	32 (23 to 43)	15 (8 to 25)	4 (3 to 4)	196 (153 to 255)	83 (58 to 117)	71 (52 to 98)	33 (18 to 57)	8 (7 to 10)
Angola	2034	88 (68 to 115)	37 (26 to 53)	32 (23 to 44)	15 (8 to 26)	4 (3 to 4)	200 (154 to 260)	85 (58 to 119)	73 (53 to 99)	34 (19 to 58)	8 (7 to 10)

Angola	2035	90 (70 to 118)	38 (26 to 54)	33 (24 to 45)	15 (8 to 26)	4 (3 to 4)	204 (157 to 266)	86 (59 to 122)	74 (53 to 101)	35 (19 to 60)	8 (7 to 10)
Angola	2036	92 (71 to 121)	39 (27 to 56)	33 (24 to 45)	16 (9 to 27)	4 (3 to 5)	207 (160 to 273)	88 (61 to 127)	75 (54 to 103)	36 (19 to 62)	8 (7 to 10)
Angola	2037	93 (72 to 123)	40 (27 to 58)	34 (24 to 46)	16 (9 to 28)	4 (3 to 4)	211 (162 to 278)	90 (61 to 130)	76 (55 to 104)	37 (20 to 63)	8 (7 to 10)
Angola	2038	95 (73 to 125)	40 (27 to 59)	34 (25 to 47)	17 (9 to 29)	4 (3 to 5)	215 (164 to 284)	91 (61 to 133)	78 (56 to 106)	37 (20 to 65)	8 (7 to 10)
Angola	2039	96 (73 to 127)	41 (27 to 60)	35 (25 to 48)	17 (9 to 29)	4 (3 to 5)	218 (166 to 289)	93 (62 to 135)	79 (57 to 108)	38 (21 to 67)	8 (7 to 10)
Angola	2040	98 (74 to 130)	42 (27 to 61)	36 (26 to 48)	17 (9 to 30)	4 (3 to 5)	222 (169 to 295)	94 (62 to 138)	80 (58 to 110)	39 (21 to 68)	8 (7 to 10)
Angola	2041	100 (75 to 133)	43 (28 to 63)	36 (26 to 49)	18 (10 to 31)	4 (3 to 5)	227 (170 to 301)	96 (63 to 142)	82 (59 to 112)	40 (22 to 70)	8 (7 to 10)
Angola	2042	102 (76 to 136)	43 (28 to 64)	37 (26 to 50)	18 (10 to 32)	4 (3 to 5)	231 (173 to 307)	98 (64 to 145)	83 (60 to 114)	41 (22 to 71)	8 (7 to 10)
Angola	2043	104 (78 to 139)	44 (28 to 65)	37 (27 to 51)	19 (10 to 32)	4 (3 to 5)	235 (176 to 314)	100 (64 to 148)	85 (61 to 116)	42 (23 to 73)	8 (7 to 11)
Angola	2044	106 (79 to 141)	45 (29 to 67)	38 (27 to 52)	19 (10 to 33)	4 (3 to 5)	240 (178 to 320)	102 (66 to 151)	86 (62 to 117)	43 (23 to 75)	8 (7 to 11)
Angola	2045	108 (79 to 143)	46 (29 to 68)	39 (28 to 53)	19 (10 to 34)	4 (3 to 5)	244 (179 to 324)	104 (66 to 154)	88 (63 to 120)	44 (24 to 77)	8 (7 to 11)
Angola	2046	110 (80 to 146)	47 (30 to 69)	39 (28 to 54)	20 (11 to 35)	4 (3 to 5)	249 (182 to 331)	106 (67 to 156)	89 (64 to 121)	45 (24 to 78)	8 (7 to 11)
Angola	2047	112 (81 to 150)	48 (30 to 70)	40 (29 to 55)	20 (11 to 35)	4 (3 to 5)	253 (184 to 340)	108 (68 to 160)	91 (65 to 124)	46 (24 to 80)	8 (7 to 11)
Angola	2048	114 (83 to 154)	48 (30 to 73)	41 (29 to 56)	21 (11 to 36)	4 (3 to 5)	257 (187 to 348)	109 (68 to 166)	93 (67 to 126)	47 (25 to 82)	8 (7 to 11)
Angola	2049	116 (84 to 156)	49 (30 to 74)	42 (30 to 57)	21 (11 to 37)	4 (3 to 5)	262 (190 to 354)	111 (68 to 167)	94 (68 to 128)	48 (25 to 83)	8 (7 to 11)

Angola	2050	118 (85 to 158)	50 (31 to 78)	42 (30 to 58)	22 (11 to 37)	4 (3 to 5)	267 (193 to 359)	113 (70 to 176)	96 (69 to 130)	49 (26 to 85)	8 (7 to 11)
Antigua and Barbuda	1995	710 (639 to 787)	430 (371 to 497)	148 (123 to 178)	133 (101 to 170)	0 (0 to 0)	1130 (1017 to 1252)	684 (591 to 791)	235 (196 to 283)	211 (161 to 271)	0 (0 to 0)
Antigua and Barbuda	1996	702 (641 to 769)	422 (370 to 478)	148 (125 to 174)	131 (104 to 160)	0 (0 to 0)	1116 (1021 to 1223)	672 (590 to 761)	236 (199 to 277)	209 (165 to 255)	0 (0 to 0)
Antigua and Barbuda	1997	662 (610 to 720)	396 (351 to 444)	142 (122 to 165)	123 (100 to 150)	0 (0 to 0)	1054 (970 to 1145)	631 (559 to 706)	227 (194 to 262)	196 (159 to 239)	0 (0 to 0)
Antigua and Barbuda	1998	730 (675 to 789)	431 (385 to 481)	147 (126 to 169)	135 (111 to 162)	18 (18 to 18)	1162 (1075 to 1256)	686 (613 to 765)	233 (201 to 269)	214 (176 to 258)	28 (28 to 28)
Antigua and Barbuda	1999	713 (660 to 769)	412 (370 to 461)	145 (125 to 165)	131 (110 to 156)	26 (26 to 26)	1135 (1051 to 1224)	656 (588 to 733)	230 (199 to 262)	208 (175 to 247)	41 (41 to 41)
Antigua and Barbuda	2000	707 (655 to 762)	408 (365 to 453)	151 (132 to 171)	132 (111 to 157)	17 (17 to 17)	1125 (1042 to 1213)	649 (580 to 722)	240 (210 to 273)	210 (177 to 250)	26 (26 to 26)
Antigua and Barbuda	2001	725 (673 to 781)	427 (385 to 473)	162 (142 to 182)	134 (113 to 158)	2 (2 to 2)	1154 (1071 to 1243)	680 (613 to 753)	257 (225 to 290)	214 (179 to 251)	2 (2 to 2)
Antigua and Barbuda	2002	764 (710 to 823)	454 (410 to 501)	172 (152 to 193)	138 (116 to 161)	0 (0 to 0)	1216 (1129 to 1309)	722 (653 to 797)	274 (242 to 307)	220 (185 to 257)	0 (0 to 0)
Antigua and Barbuda	2003	769 (714 to 825)	450 (406 to 497)	183 (162 to 203)	133 (112 to 154)	4 (4 to 4)	1223 (1136 to 1312)	716 (647 to 791)	291 (257 to 323)	211 (178 to 244)	6 (6 to 6)

Antigua and Barbuda	2004	747 (696 to 798)	425 (383 to 470)	191 (171 to 212)	125 (107 to 147)	6 (6 to 6)	1189 (1108 to 1270)	676 (610 to 748)	304 (272 to 338)	200 (171 to 234)	9 (9 to 9)
Antigua and Barbuda	2005	738 (690 to 792)	410 (370 to 454)	206 (185 to 227)	122 (106 to 142)	0 (0 to 0)	1175 (1098 to 1260)	652 (588 to 722)	327 (294 to 361)	195 (168 to 226)	0 (0 to 0)
Antigua and Barbuda	2006	846 (789 to 904)	469 (424 to 517)	238 (216 to 262)	139 (120 to 161)	0 (0 to 0)	1346 (1255 to 1438)	746 (675 to 823)	379 (343 to 417)	221 (191 to 256)	0 (0 to 0)
Antigua and Barbuda	2007	845 (790 to 905)	463 (420 to 511)	246 (223 to 271)	136 (117 to 157)	0 (0 to 0)	1345 (1257 to 1440)	736 (668 to 813)	391 (355 to 431)	217 (187 to 250)	0 (0 to 0)
Antigua and Barbuda	2008	821 (768 to 878)	449 (408 to 494)	236 (214 to 261)	135 (116 to 158)	0 (0 to 0)	1307 (1221 to 1397)	715 (649 to 785)	376 (340 to 415)	215 (185 to 251)	0 (0 to 0)
Antigua and Barbuda	2009	906 (844 to 971)	509 (461 to 563)	232 (208 to 259)	157 (132 to 185)	8 (8 to 8)	1442 (1343 to 1546)	810 (734 to 896)	370 (331 to 412)	249 (210 to 294)	13 (13 to 13)
Antigua and Barbuda	2010	721 (674 to 771)	391 (354 to 431)	201 (179 to 223)	122 (104 to 146)	6 (6 to 6)	1148 (1072 to 1227)	623 (563 to 686)	320 (286 to 356)	195 (166 to 232)	10 (10 to 10)
Antigua and Barbuda	2011	771 (723 to 821)	418 (378 to 462)	210 (188 to 233)	134 (114 to 158)	10 (10 to 10)	1228 (1150 to 1307)	665 (601 to 735)	334 (299 to 371)	214 (181 to 252)	15 (15 to 15)
Antigua and Barbuda	2012	754 (702 to 805)	410 (370 to 451)	212 (190 to 235)	132 (113 to 153)	0 (0 to 0)	1200 (1117 to 1281)	652 (589 to 718)	337 (302 to 374)	211 (181 to 244)	1 (1 to 1)
Antigua and Barbuda	2013	800 (743 to 854)	437 (394 to 485)	221 (198 to 245)	141 (121 to 163)	0 (0 to 0)	1273 (1183 to 1359)	696 (627 to 771)	352 (315 to 390)	225 (192 to 259)	0 (0 to 0)

Antigua and Barbuda	2014	775 (718 to 830)	415 (371 to 462)	222 (199 to 245)	138 (118 to 158)	0 (0 to 0)	1233 (1143 to 1321)	660 (591 to 735)	353 (317 to 389)	219 (188 to 251)	0 (0 to 0)
Antigua and Barbuda	2015	803 (743 to 865)	420 (373 to 476)	233 (209 to 256)	150 (128 to 172)	0 (0 to 0)	1278 (1182 to 1376)	669 (594 to 757)	371 (333 to 408)	238 (204 to 274)	0 (0 to 0)
Antigua and Barbuda	2016	749 (688 to 813)	359 (311 to 412)	244 (218 to 271)	146 (122 to 170)	0 (0 to 0)	1192 (1095 to 1294)	571 (496 to 656)	389 (346 to 431)	232 (195 to 271)	0 (0 to 0)
Antigua and Barbuda	2017	668 (588 to 750)	275 (233 to 319)	253 (220 to 286)	140 (114 to 171)	0 (0 to 0)	1063 (935 to 1194)	437 (371 to 508)	403 (351 to 455)	223 (181 to 272)	0 (0 to 0)
Antigua and Barbuda	2018	725 (638 to 816)	306 (258 to 357)	266 (232 to 300)	153 (125 to 186)	0 (0 to 0)	1154 (1015 to 1299)	487 (411 to 568)	423 (368 to 477)	243 (198 to 297)	0 (0 to 0)
Antigua and Barbuda	2019	742 (651 to 837)	313 (260 to 368)	273 (238 to 308)	157 (127 to 190)	0 (0 to 0)	1181 (1035 to 1332)	498 (414 to 585)	435 (379 to 490)	249 (202 to 303)	0 (0 to 0)
Antigua and Barbuda	2020	766 (668 to 863)	325 (267 to 388)	279 (244 to 315)	162 (132 to 197)	0 (0 to 0)	1219 (1063 to 1374)	517 (424 to 617)	444 (388 to 501)	258 (210 to 313)	0 (0 to 0)
Antigua and Barbuda	2021	784 (681 to 895)	335 (274 to 406)	283 (248 to 319)	166 (135 to 201)	0 (0 to 0)	1247 (1083 to 1425)	532 (436 to 645)	451 (394 to 508)	264 (214 to 320)	0 (0 to 0)
Antigua and Barbuda	2022	802 (694 to 920)	344 (280 to 422)	288 (251 to 324)	170 (138 to 206)	0 (0 to 0)	1276 (1105 to 1465)	548 (445 to 671)	458 (399 to 516)	270 (220 to 328)	0 (0 to 0)
Antigua and Barbuda	2023	822 (711 to 947)	356 (288 to 440)	292 (255 to 329)	174 (142 to 212)	0 (0 to 0)	1308 (1131 to 1507)	566 (458 to 701)	465 (405 to 524)	277 (225 to 337)	0 (0 to 0)

Antigua and Barbuda	2024	840 (725 to 972)	367 (297 to 452)	295 (257 to 333)	178 (145 to 217)	0 (0 to 0)	1337 (1154 to 1546)	584 (472 to 719)	470 (409 to 530)	284 (231 to 345)	0 (0 to 0)
Antigua and Barbuda	2025	860 (738 to 994)	379 (306 to 470)	298 (260 to 337)	183 (148 to 222)	0 (0 to 0)	1368 (1175 to 1581)	603 (488 to 749)	475 (414 to 536)	290 (236 to 354)	0 (0 to 0)
Antigua and Barbuda	2026	871 (750 to 1018)	384 (308 to 485)	301 (263 to 340)	185 (150 to 225)	0 (0 to 0)	1386 (1193 to 1619)	612 (490 to 771)	480 (418 to 541)	295 (239 to 359)	0 (0 to 0)
Antigua and Barbuda	2027	882 (760 to 1036)	390 (310 to 492)	304 (265 to 344)	188 (153 to 228)	0 (0 to 0)	1403 (1209 to 1648)	620 (493 to 782)	484 (422 to 547)	299 (243 to 363)	0 (0 to 0)
Antigua and Barbuda	2028	892 (764 to 1048)	395 (310 to 503)	307 (268 to 346)	190 (155 to 231)	0 (0 to 0)	1419 (1216 to 1667)	628 (494 to 801)	488 (426 to 551)	302 (246 to 368)	0 (0 to 0)
Antigua and Barbuda	2029	900 (768 to 1058)	399 (312 to 511)	309 (269 to 349)	192 (156 to 234)	0 (0 to 0)	1433 (1221 to 1683)	635 (497 to 812)	492 (428 to 556)	306 (248 to 372)	0 (0 to 0)
Antigua and Barbuda	2030	909 (774 to 1065)	403 (314 to 518)	311 (271 to 351)	194 (157 to 236)	0 (0 to 0)	1447 (1232 to 1694)	642 (500 to 825)	495 (431 to 559)	309 (251 to 376)	0 (0 to 0)
Antigua and Barbuda	2031	918 (777 to 1082)	408 (315 to 522)	313 (272 to 354)	197 (159 to 239)	0 (0 to 0)	1460 (1236 to 1722)	649 (501 to 831)	499 (433 to 563)	313 (253 to 381)	0 (0 to 0)
Antigua and Barbuda	2032	926 (783 to 1098)	412 (318 to 538)	316 (274 to 357)	199 (161 to 241)	0 (0 to 0)	1474 (1247 to 1747)	656 (506 to 856)	502 (435 to 568)	316 (257 to 384)	0 (0 to 0)
Antigua and Barbuda	2033	934 (786 to 1116)	416 (316 to 546)	318 (275 to 360)	201 (163 to 244)	0 (0 to 0)	1487 (1251 to 1776)	662 (502 to 869)	505 (437 to 573)	320 (259 to 388)	0 (0 to 0)

Antigua and Barbuda	2034	942 (793 to 1126)	420 (322 to 549)	319 (276 to 362)	203 (164 to 247)	0 (0 to 0)	1499 (1261 to 1791)	668 (513 to 873)	508 (439 to 576)	323 (262 to 393)	0 (0 to 0)
Antigua and Barbuda	2035	950 (795 to 1134)	423 (321 to 557)	321 (277 to 365)	205 (166 to 249)	0 (0 to 0)	1511 (1265 to 1804)	673 (511 to 886)	511 (440 to 581)	327 (265 to 396)	0 (0 to 0)
Antigua and Barbuda	2036	958 (803 to 1147)	427 (319 to 563)	323 (278 to 367)	207 (168 to 252)	0 (0 to 0)	1524 (1278 to 1826)	679 (507 to 896)	514 (443 to 583)	330 (267 to 400)	0 (0 to 0)
Antigua and Barbuda	2037	966 (813 to 1154)	431 (326 to 567)	325 (279 to 370)	210 (170 to 254)	0 (0 to 0)	1538 (1294 to 1836)	687 (519 to 902)	517 (444 to 588)	334 (271 to 404)	0 (0 to 0)
Antigua and Barbuda	2038	975 (812 to 1166)	436 (329 to 575)	327 (280 to 373)	212 (172 to 256)	0 (0 to 0)	1552 (1291 to 1855)	694 (524 to 914)	520 (445 to 593)	338 (273 to 408)	0 (0 to 0)
Antigua and Barbuda	2039	984 (817 to 1194)	441 (327 to 593)	329 (282 to 376)	215 (173 to 260)	0 (0 to 0)	1566 (1300 to 1900)	702 (520 to 944)	523 (448 to 598)	342 (276 to 413)	0 (0 to 0)
Antigua and Barbuda	2040	994 (824 to 1205)	446 (329 to 598)	331 (283 to 379)	217 (175 to 263)	0 (0 to 0)	1582 (1311 to 1917)	710 (524 to 951)	527 (451 to 604)	346 (279 to 419)	0 (0 to 0)
Antigua and Barbuda	2041	1004 (831 to 1208)	451 (328 to 598)	333 (285 to 383)	220 (177 to 267)	0 (0 to 0)	1597 (1323 to 1922)	718 (522 to 952)	530 (453 to 609)	350 (282 to 425)	0 (0 to 0)
Antigua and Barbuda	2042	1014 (835 to 1225)	457 (333 to 610)	335 (286 to 385)	223 (180 to 271)	0 (0 to 0)	1613 (1329 to 1949)	726 (529 to 970)	533 (456 to 613)	354 (286 to 431)	0 (0 to 0)
Antigua and Barbuda	2043	1023 (845 to 1237)	461 (333 to 621)	337 (288 to 389)	225 (181 to 274)	0 (0 to 0)	1628 (1345 to 1969)	733 (530 to 988)	536 (458 to 620)	359 (288 to 436)	0 (0 to 0)

Antigua and Barbuda	2044	1034 (857 to 1245)	467 (336 to 629)	339 (290 to 393)	228 (183 to 278)	0 (0 to 0)	1645 (1363 to 1981)	742 (534 to 1001)	539 (461 to 625)	363 (291 to 442)	0 (0 to 0)
Antigua and Barbuda	2045	1044 (861 to 1259)	472 (339 to 639)	341 (291 to 396)	231 (185 to 282)	0 (0 to 0)	1662 (1370 to 2004)	752 (539 to 1016)	543 (463 to 630)	368 (295 to 448)	0 (0 to 0)
Antigua and Barbuda	2046	1055 (866 to 1272)	478 (340 to 645)	343 (293 to 398)	234 (187 to 285)	0 (0 to 0)	1679 (1377 to 2024)	761 (541 to 1026)	546 (466 to 634)	373 (297 to 453)	0 (0 to 0)
Antigua and Barbuda	2047	1067 (874 to 1289)	484 (342 to 656)	345 (294 to 401)	237 (189 to 289)	0 (0 to 0)	1698 (1391 to 2051)	771 (545 to 1044)	549 (467 to 638)	377 (301 to 460)	0 (0 to 0)
Antigua and Barbuda	2048	1077 (882 to 1310)	490 (344 to 668)	347 (295 to 403)	240 (191 to 293)	0 (0 to 0)	1714 (1403 to 2084)	779 (547 to 1063)	552 (469 to 641)	382 (304 to 467)	0 (0 to 0)
Antigua and Barbuda	2049	1087 (879 to 1327)	495 (346 to 678)	349 (297 to 407)	243 (193 to 298)	0 (0 to 0)	1730 (1398 to 2112)	788 (551 to 1079)	556 (473 to 647)	387 (307 to 473)	0 (0 to 0)
Antigua and Barbuda	2050	1099 (889 to 1348)	501 (353 to 689)	351 (299 to 410)	246 (195 to 301)	0 (0 to 0)	1748 (1414 to 2145)	797 (561 to 1097)	559 (475 to 652)	392 (311 to 479)	0 (0 to 0)
Argentina	1995	739 (685 to 796)	452 (416 to 492)	186 (157 to 218)	95 (71 to 128)	5 (5 to 5)	1634 (1514 to 1760)	1000 (919 to 1089)	411 (347 to 482)	211 (156 to 283)	12 (12 to 12)
Argentina	1996	778 (730 to 828)	472 (440 to 505)	195 (169 to 224)	103 (81 to 130)	8 (8 to 8)	1721 (1614 to 1831)	1043 (973 to 1116)	432 (375 to 495)	228 (179 to 287)	18 (18 to 18)
Argentina	1997	796 (752 to 842)	475 (445 to 506)	202 (178 to 228)	110 (89 to 135)	8 (8 to 8)	1761 (1662 to 1862)	1052 (984 to 1119)	447 (393 to 505)	244 (196 to 299)	19 (19 to 19)

Argentina	1998	803 (757 to 845)	472 (443 to 501)	207 (183 to 232)	115 (93 to 139)	9 (9 to 9)	1776 (1675 to 1869)	1045 (979 to 1109)	459 (405 to 514)	253 (206 to 307)	19 (19 to 19)
Argentina	1999	784 (740 to 828)	456 (427 to 484)	209 (186 to 235)	116 (94 to 140)	4 (4 to 4)	1735 (1637 to 1831)	1008 (945 to 1071)	463 (412 to 520)	256 (207 to 311)	9 (9 to 9)
Argentina	2000	734 (690 to 776)	415 (388 to 443)	205 (184 to 229)	112 (91 to 137)	2 (2 to 2)	1623 (1527 to 1717)	918 (859 to 980)	453 (407 to 507)	247 (200 to 302)	5 (5 to 5)
Argentina	2001	696 (654 to 740)	385 (359 to 411)	199 (176 to 224)	106 (85 to 132)	6 (6 to 6)	1539 (1446 to 1636)	851 (793 to 910)	439 (389 to 495)	235 (188 to 292)	14 (14 to 14)
Argentina	2002	555 (522 to 590)	298 (278 to 320)	172 (151 to 194)	82 (66 to 102)	2 (2 to 2)	1228 (1154 to 1306)	660 (616 to 708)	380 (334 to 428)	182 (145 to 225)	5 (5 to 5)
Argentina	2003	579 (543 to 614)	300 (278 to 321)	172 (151 to 193)	86 (68 to 107)	21 (21 to 21)	1280 (1200 to 1358)	663 (615 to 711)	381 (333 to 426)	190 (150 to 236)	47 (47 to 47)
Argentina	2004	586 (550 to 622)	310 (289 to 333)	181 (160 to 203)	89 (70 to 108)	6 (6 to 6)	1297 (1216 to 1376)	686 (640 to 736)	401 (353 to 449)	196 (154 to 239)	13 (13 to 13)
Argentina	2005	637 (598 to 675)	338 (315 to 362)	200 (178 to 224)	96 (77 to 117)	2 (2 to 2)	1408 (1322 to 1492)	748 (696 to 800)	443 (393 to 494)	212 (171 to 260)	5 (5 to 5)
Argentina	2006	698 (655 to 739)	379 (353 to 404)	212 (188 to 237)	105 (85 to 128)	2 (2 to 2)	1544 (1449 to 1634)	839 (781 to 894)	468 (415 to 525)	232 (189 to 284)	4 (4 to 4)
Argentina	2007	784 (738 to 829)	441 (413 to 467)	218 (194 to 243)	117 (96 to 143)	8 (8 to 8)	1735 (1633 to 1834)	976 (915 to 1033)	481 (429 to 538)	259 (212 to 316)	18 (18 to 18)

Argentina	2008	826 (779 to 872)	489 (459 to 519)	209 (187 to 234)	123 (101 to 149)	4 (4 to 4)	1827 (1722 to 1930)	1083 (1016 to 1147)	462 (413 to 517)	272 (224 to 330)	10 (10 to 10)
Argentina	2009	857 (813 to 902)	533 (504 to 564)	194 (173 to 219)	125 (102 to 150)	4 (4 to 4)	1895 (1799 to 1995)	1180 (1115 to 1247)	430 (384 to 485)	277 (226 to 332)	9 (9 to 9)
Argentina	2010	888 (845 to 935)	579 (549 to 610)	178 (158 to 200)	126 (104 to 151)	4 (4 to 4)	1964 (1868 to 2067)	1280 (1213 to 1350)	394 (350 to 443)	279 (231 to 334)	10 (10 to 10)
Argentina	2011	933 (886 to 977)	637 (606 to 671)	164 (145 to 185)	124 (103 to 148)	8 (8 to 8)	2064 (1959 to 2161)	1409 (1341 to 1485)	363 (321 to 410)	274 (228 to 327)	17 (17 to 17)
Argentina	2012	932 (886 to 975)	666 (633 to 700)	147 (130 to 166)	113 (93 to 137)	6 (6 to 6)	2061 (1960 to 2157)	1472 (1401 to 1549)	325 (287 to 367)	249 (207 to 302)	14 (14 to 14)
Argentina	2013	930 (889 to 970)	684 (652 to 719)	139 (122 to 157)	100 (82 to 122)	7 (7 to 7)	2056 (1967 to 2147)	1513 (1443 to 1590)	307 (269 to 346)	221 (182 to 269)	15 (15 to 15)
Argentina	2014	895 (855 to 937)	676 (647 to 708)	132 (115 to 151)	87 (70 to 106)	0 (0 to 0)	1980 (1892 to 2072)	1495 (1432 to 1567)	292 (254 to 333)	192 (155 to 235)	0 (0 to 0)
Argentina	2015	924 (880 to 969)	700 (668 to 733)	133 (114 to 151)	87 (69 to 107)	5 (5 to 5)	2044 (1947 to 2144)	1549 (1478 to 1621)	293 (252 to 335)	192 (153 to 236)	10 (10 to 10)
Argentina	2016	881 (838 to 931)	658 (623 to 692)	130 (110 to 151)	86 (67 to 109)	7 (7 to 7)	1949 (1854 to 2059)	1456 (1379 to 1531)	288 (244 to 335)	190 (148 to 241)	16 (16 to 16)
Argentina	2017	907 (830 to 987)	674 (627 to 725)	136 (112 to 162)	97 (73 to 127)	0 (0 to 0)	2006 (1837 to 2184)	1491 (1386 to 1604)	300 (248 to 357)	215 (161 to 281)	0 (0 to 0)

Argentina	2018	845 (772 to 929)	623 (575 to 673)	132 (109 to 157)	91 (68 to 119)	0 (0 to 0)	1870 (1709 to 2054)	1378 (1273 to 1489)	291 (241 to 347)	201 (150 to 262)	0 (0 to 0)
Argentina	2019	798 (724 to 875)	582 (534 to 631)	127 (105 to 151)	88 (66 to 115)	0 (0 to 0)	1764 (1603 to 1936)	1288 (1181 to 1395)	281 (233 to 335)	195 (146 to 255)	0 (0 to 0)
Argentina	2020	789 (714 to 871)	577 (525 to 628)	125 (103 to 148)	87 (65 to 114)	0 (0 to 0)	1744 (1579 to 1928)	1276 (1162 to 1390)	276 (228 to 328)	193 (143 to 251)	0 (0 to 0)
Argentina	2021	793 (711 to 876)	581 (525 to 636)	124 (103 to 148)	87 (65 to 114)	0 (0 to 0)	1753 (1573 to 1937)	1285 (1161 to 1408)	275 (227 to 327)	194 (144 to 253)	0 (0 to 0)
Argentina	2022	797 (712 to 890)	585 (524 to 647)	124 (102 to 148)	88 (66 to 115)	0 (0 to 0)	1763 (1575 to 1968)	1294 (1160 to 1431)	274 (227 to 326)	195 (145 to 254)	0 (0 to 0)
Argentina	2023	803 (713 to 894)	590 (527 to 653)	124 (102 to 147)	88 (66 to 116)	0 (0 to 0)	1775 (1577 to 1977)	1306 (1165 to 1444)	273 (226 to 325)	196 (146 to 256)	0 (0 to 0)
Argentina	2024	807 (716 to 905)	595 (529 to 663)	123 (102 to 147)	89 (66 to 116)	0 (0 to 0)	1786 (1584 to 2003)	1316 (1171 to 1467)	273 (225 to 325)	197 (147 to 258)	0 (0 to 0)
Argentina	2025	813 (719 to 915)	600 (532 to 673)	123 (102 to 146)	89 (67 to 117)	0 (0 to 0)	1798 (1590 to 2024)	1328 (1177 to 1489)	272 (225 to 324)	198 (148 to 259)	0 (0 to 0)
Argentina	2026	818 (718 to 926)	605 (533 to 685)	123 (101 to 146)	90 (67 to 118)	0 (0 to 0)	1808 (1589 to 2047)	1338 (1179 to 1514)	271 (224 to 322)	199 (149 to 261)	0 (0 to 0)
Argentina	2027	823 (722 to 935)	610 (532 to 693)	122 (101 to 145)	90 (68 to 118)	0 (0 to 0)	1820 (1597 to 2068)	1349 (1177 to 1533)	271 (223 to 322)	200 (150 to 262)	0 (0 to 0)

Argentina	2028	828 (723 to 948)	615 (537 to 703)	122 (101 to 145)	91 (68 to 119)	0 (0 to 0)	1832 (1599 to 2097)	1360 (1187 to 1555)	270 (223 to 321)	201 (150 to 263)	0 (0 to 0)
Argentina	2029	834 (724 to 954)	620 (537 to 716)	122 (100 to 145)	91 (68 to 120)	0 (0 to 0)	1845 (1602 to 2110)	1372 (1189 to 1584)	270 (222 to 321)	202 (151 to 265)	0 (0 to 0)
Argentina	2030	844 (731 to 970)	629 (543 to 722)	122 (101 to 145)	92 (69 to 121)	0 (0 to 0)	1866 (1617 to 2147)	1391 (1201 to 1597)	270 (223 to 322)	204 (152 to 268)	0 (0 to 0)
Argentina	2031	854 (735 to 982)	638 (545 to 734)	123 (101 to 146)	93 (70 to 122)	0 (0 to 0)	1889 (1627 to 2173)	1410 (1206 to 1624)	271 (223 to 323)	207 (154 to 271)	0 (0 to 0)
Argentina	2032	866 (742 to 999)	648 (551 to 748)	123 (101 to 147)	95 (70 to 124)	0 (0 to 0)	1916 (1642 to 2210)	1434 (1218 to 1655)	272 (224 to 325)	209 (156 to 274)	0 (0 to 1)
Argentina	2033	880 (750 to 1018)	660 (553 to 766)	124 (102 to 148)	96 (71 to 126)	0 (0 to 0)	1947 (1659 to 2251)	1460 (1224 to 1695)	274 (226 to 327)	212 (158 to 278)	0 (0 to 1)
Argentina	2034	895 (761 to 1035)	672 (566 to 783)	125 (103 to 149)	97 (72 to 128)	0 (0 to 0)	1979 (1683 to 2290)	1487 (1252 to 1731)	276 (227 to 329)	215 (160 to 282)	0 (0 to 1)
Argentina	2035	909 (771 to 1058)	685 (570 to 798)	126 (103 to 150)	99 (73 to 129)	0 (0 to 0)	2011 (1705 to 2340)	1515 (1262 to 1765)	278 (228 to 331)	218 (162 to 286)	0 (0 to 1)
Argentina	2036	924 (787 to 1077)	698 (585 to 817)	126 (104 to 151)	100 (74 to 131)	0 (0 to 0)	2044 (1741 to 2381)	1543 (1294 to 1807)	279 (230 to 334)	221 (164 to 290)	0 (0 to 1)
Argentina	2037	940 (792 to 1100)	711 (592 to 838)	127 (104 to 152)	101 (75 to 133)	0 (0 to 0)	2078 (1752 to 2432)	1572 (1310 to 1854)	281 (231 to 336)	224 (166 to 293)	0 (0 to 1)

Argentina	2038	955 (810 to 1125)	724 (607 to 857)	128 (105 to 153)	103 (76 to 135)	0 (0 to 0)	2113 (1791 to 2488)	1602 (1343 to 1897)	283 (232 to 338)	228 (168 to 298)	0 (0 to 1)
Argentina	2039	970 (817 to 1141)	737 (613 to 873)	129 (105 to 154)	104 (77 to 137)	0 (0 to 0)	2145 (1807 to 2525)	1629 (1356 to 1931)	284 (233 to 340)	231 (170 to 302)	0 (0 to 1)
Argentina	2040	985 (824 to 1164)	750 (617 to 897)	129 (106 to 155)	106 (78 to 138)	0 (0 to 0)	2179 (1824 to 2575)	1659 (1365 to 1984)	286 (234 to 342)	234 (172 to 306)	0 (0 to 1)
Argentina	2041	999 (833 to 1184)	762 (623 to 905)	130 (106 to 155)	107 (79 to 140)	0 (0 to 0)	2210 (1842 to 2620)	1685 (1377 to 2002)	287 (235 to 344)	237 (174 to 310)	0 (0 to 1)
Argentina	2042	1013 (844 to 1198)	774 (635 to 926)	130 (107 to 156)	108 (80 to 142)	0 (0 to 0)	2241 (1868 to 2651)	1712 (1404 to 2049)	289 (236 to 346)	239 (176 to 313)	1 (0 to 1)
Argentina	2043	1029 (845 to 1222)	788 (641 to 942)	131 (107 to 157)	110 (81 to 143)	0 (0 to 0)	2276 (1868 to 2702)	1743 (1418 to 2083)	290 (237 to 348)	242 (178 to 317)	1 (0 to 1)
Argentina	2044	1044 (863 to 1237)	801 (650 to 960)	132 (108 to 158)	111 (81 to 145)	0 (0 to 0)	2309 (1910 to 2737)	1772 (1438 to 2124)	291 (238 to 350)	245 (180 to 321)	1 (0 to 1)
Argentina	2045	1059 (871 to 1264)	814 (657 to 982)	132 (108 to 159)	112 (82 to 147)	0 (0 to 0)	2342 (1926 to 2795)	1801 (1454 to 2173)	292 (240 to 352)	248 (182 to 325)	1 (0 to 1)
Argentina	2046	1073 (879 to 1281)	827 (664 to 1003)	133 (108 to 160)	114 (83 to 149)	0 (0 to 0)	2374 (1944 to 2833)	1829 (1469 to 2219)	293 (240 to 354)	251 (184 to 329)	1 (0 to 1)
Argentina	2047	1089 (896 to 1305)	841 (673 to 1022)	133 (109 to 161)	115 (84 to 150)	0 (0 to 0)	2408 (1981 to 2886)	1859 (1489 to 2260)	295 (240 to 356)	254 (185 to 332)	1 (0 to 1)

Argentina	2048	1104 (900 to 1332)	854 (682 to 1048)	134 (109 to 161)	116 (85 to 152)	0 (0 to 0)	2441 (1991 to 2947)	1888 (1509 to 2318)	296 (241 to 357)	257 (187 to 337)	1 (0 to 1)
Argentina	2049	1117 (913 to 1350)	865 (691 to 1059)	134 (109 to 162)	117 (85 to 154)	0 (0 to 0)	2471 (2019 to 2986)	1914 (1529 to 2343)	297 (242 to 358)	260 (188 to 341)	1 (0 to 1)
Argentina	2050	1131 (917 to 1364)	878 (698 to 1072)	135 (110 to 163)	119 (86 to 156)	0 (0 to 0)	2503 (2028 to 3017)	1942 (1545 to 2372)	298 (243 to 360)	263 (190 to 346)	1 (0 to 1)
Armenia	1995	54 (44 to 66)	16 (12 to 20)	37 (28 to 48)	1 (0 to 2)	0 (0 to 0)	130 (104 to 158)	37 (29 to 48)	90 (68 to 115)	2 (1 to 4)	1 (1 to 1)
Armenia	1996	57 (47 to 68)	16 (12 to 20)	39 (30 to 50)	1 (1 to 2)	1 (1 to 1)	136 (112 to 164)	37 (29 to 47)	93 (72 to 119)	2 (1 to 4)	3 (3 to 3)
Armenia	1997	58 (49 to 70)	15 (12 to 19)	41 (33 to 51)	1 (1 to 2)	1 (1 to 1)	139 (116 to 167)	37 (28 to 46)	99 (78 to 123)	3 (1 to 5)	1 (1 to 1)
Armenia	1998	61 (52 to 72)	16 (12 to 20)	43 (34 to 53)	1 (1 to 2)	2 (2 to 2)	147 (124 to 173)	37 (29 to 47)	103 (82 to 127)	3 (1 to 5)	4 (4 to 4)
Armenia	1999	63 (54 to 74)	16 (12 to 20)	44 (36 to 54)	1 (1 to 2)	3 (3 to 3)	152 (130 to 178)	38 (29 to 48)	105 (85 to 129)	3 (1 to 5)	6 (6 to 6)
Armenia	2000	66 (57 to 76)	16 (12 to 20)	44 (37 to 53)	1 (1 to 2)	5 (5 to 5)	157 (136 to 183)	37 (29 to 48)	106 (88 to 128)	3 (1 to 5)	11 (11 to 11)
Armenia	2001	73 (64 to 84)	18 (14 to 22)	51 (43 to 61)	1 (1 to 2)	3 (3 to 3)	175 (152 to 201)	42 (33 to 53)	123 (103 to 147)	3 (2 to 6)	7 (7 to 7)
Armenia	2002	94 (83 to 108)	20 (15 to 25)	69 (58 to 81)	1 (1 to 2)	5 (5 to 5)	226 (199 to 258)	47 (37 to 59)	165 (138 to 194)	3 (2 to 6)	11 (11 to 11)
Armenia	2003	110 (97 to 126)	24 (19 to 29)	83 (71 to 98)	1 (1 to 2)	2 (2 to 2)	263 (231 to 303)	57 (44 to 70)	199 (169 to 234)	3 (2 to 5)	5 (5 to 5)
Armenia	2004	119 (106 to 136)	27 (21 to 33)	88 (75 to 104)	1 (0 to 2)	3 (3 to 3)	286 (253 to 326)	65 (51 to 80)	212 (180 to 248)	2 (1 to 4)	7 (7 to 7)
Armenia	2005	134 (119 to 151)	32 (25 to 40)	95 (82 to 110)	1 (0 to 1)	6 (6 to 6)	320 (286 to 360)	77 (61 to 97)	227 (196 to 264)	1 (1 to 2)	14 (14 to 14)

Armenia	2006	153 (137 to 172)	38 (30 to 48)	105 (92 to 121)	0 (0 to 1)	9 (9 to 9)	366 (328 to 411)	92 (73 to 114)	252 (221 to 290)	1 (1 to 2)	21 (21 to 21)
Armenia	2007	182 (165 to 202)	47 (37 to 58)	125 (111 to 142)	1 (0 to 1)	10 (10 to 10)	436 (394 to 485)	112 (88 to 139)	299 (265 to 340)	1 (1 to 2)	24 (24 to 24)
Armenia	2008	208 (189 to 230)	49 (39 to 61)	151 (134 to 170)	1 (0 to 1)	8 (8 to 8)	498 (452 to 552)	117 (93 to 145)	361 (321 to 408)	2 (1 to 3)	19 (19 to 19)
Armenia	2009	246 (223 to 268)	52 (42 to 64)	178 (160 to 199)	1 (0 to 2)	14 (14 to 14)	588 (533 to 642)	124 (100 to 154)	427 (383 to 477)	2 (1 to 4)	34 (34 to 34)
Armenia	2010	262 (238 to 285)	50 (40 to 62)	200 (181 to 223)	1 (1 to 2)	10 (10 to 10)	626 (570 to 682)	120 (95 to 149)	479 (433 to 533)	3 (1 to 6)	23 (23 to 23)
Armenia	2011	289 (265 to 313)	51 (41 to 64)	226 (204 to 248)	2 (1 to 4)	10 (10 to 10)	692 (633 to 750)	123 (98 to 153)	541 (489 to 594)	5 (2 to 9)	24 (24 to 24)
Armenia	2012	309 (284 to 335)	50 (40 to 62)	245 (222 to 269)	3 (1 to 5)	11 (11 to 11)	739 (680 to 802)	119 (95 to 149)	587 (532 to 644)	7 (3 to 12)	26 (26 to 26)
Armenia	2013	338 (311 to 367)	51 (41 to 64)	274 (249 to 300)	4 (2 to 7)	9 (9 to 9)	810 (745 to 878)	123 (97 to 153)	657 (597 to 719)	9 (5 to 17)	21 (21 to 21)
Armenia	2014	354 (327 to 385)	53 (43 to 66)	292 (267 to 318)	4 (2 to 8)	5 (5 to 5)	848 (782 to 922)	127 (102 to 158)	698 (638 to 762)	10 (5 to 18)	13 (13 to 13)
Armenia	2015	376 (346 to 408)	58 (46 to 72)	310 (282 to 339)	5 (2 to 8)	4 (4 to 4)	901 (829 to 977)	139 (111 to 173)	742 (675 to 812)	11 (5 to 20)	9 (9 to 9)
Armenia	2016	389 (356 to 423)	59 (47 to 74)	318 (287 to 349)	5 (2 to 8)	7 (7 to 7)	930 (853 to 1013)	142 (113 to 177)	761 (687 to 837)	11 (5 to 20)	17 (17 to 17)
Armenia	2017	403 (364 to 447)	59 (47 to 75)	334 (298 to 376)	5 (2 to 9)	5 (5 to 5)	966 (872 to 1070)	142 (112 to 179)	800 (713 to 901)	12 (6 to 21)	12 (12 to 12)
Armenia	2018	413 (372 to 458)	58 (45 to 73)	345 (308 to 389)	5 (2 to 9)	5 (5 to 5)	988 (890 to 1097)	139 (109 to 175)	827 (737 to 930)	12 (6 to 21)	11 (11 to 11)
Armenia	2019	431 (390 to 478)	64 (50 to 81)	357 (318 to 403)	5 (3 to 10)	5 (5 to 5)	1032 (933 to 1145)	153 (119 to 194)	855 (761 to 964)	13 (6 to 23)	11 (11 to 11)

Armenia	2020	446 (402 to 493)	66 (51 to 84)	369 (329 to 416)	6 (3 to 10)	5 (5 to 5)	1067 (962 to 1179)	159 (122 to 201)	884 (788 to 996)	13 (7 to 24)	12 (11 to 12)
Armenia	2021	454 (408 to 505)	68 (52 to 86)	375 (335 to 423)	6 (3 to 10)	5 (5 to 5)	1086 (977 to 1209)	162 (126 to 206)	898 (801 to 1012)	13 (7 to 24)	12 (11 to 13)
Armenia	2022	462 (417 to 509)	69 (53 to 88)	381 (340 to 429)	6 (3 to 10)	5 (5 to 6)	1105 (998 to 1219)	166 (128 to 210)	913 (814 to 1028)	14 (7 to 25)	12 (11 to 13)
Armenia	2023	470 (422 to 521)	71 (55 to 89)	388 (346 to 437)	6 (3 to 11)	5 (5 to 6)	1124 (1010 to 1248)	170 (131 to 214)	928 (828 to 1045)	14 (7 to 25)	12 (11 to 14)
Armenia	2024	479 (432 to 532)	73 (56 to 93)	394 (351 to 444)	6 (3 to 11)	5 (5 to 6)	1146 (1035 to 1273)	176 (135 to 223)	943 (840 to 1062)	15 (7 to 26)	13 (11 to 14)
Armenia	2025	486 (437 to 541)	76 (58 to 96)	399 (356 to 449)	6 (3 to 11)	5 (5 to 6)	1164 (1045 to 1296)	181 (138 to 230)	955 (851 to 1074)	15 (7 to 27)	13 (12 to 15)
Armenia	2026	494 (446 to 547)	78 (59 to 100)	404 (360 to 455)	6 (3 to 11)	6 (5 to 6)	1183 (1068 to 1308)	187 (142 to 239)	968 (863 to 1089)	15 (8 to 27)	13 (12 to 15)
Armenia	2027	504 (452 to 555)	80 (61 to 103)	411 (366 to 462)	7 (3 to 12)	6 (5 to 7)	1205 (1083 to 1329)	192 (146 to 246)	984 (877 to 1106)	16 (8 to 28)	13 (12 to 16)
Armenia	2028	514 (459 to 572)	83 (62 to 106)	419 (373 to 471)	7 (3 to 12)	6 (5 to 7)	1231 (1099 to 1368)	198 (150 to 254)	1003 (893 to 1128)	16 (8 to 29)	14 (12 to 16)
Armenia	2029	526 (473 to 582)	85 (64 to 110)	428 (381 to 481)	7 (3 to 12)	6 (5 to 7)	1258 (1133 to 1394)	204 (154 to 262)	1023 (911 to 1152)	17 (8 to 30)	14 (12 to 16)

Armenia	2030	538 (483 to 597)	88 (66 to 114)	437 (389 to 492)	7 (3 to 13)	6 (5 to 7)	1287 (1156 to 1428)	210 (158 to 273)	1045 (930 to 1177)	17 (8 to 30)	14 (12 to 17)
Armenia	2031	550 (494 to 612)	90 (68 to 117)	446 (397 to 503)	7 (4 to 13)	6 (5 to 7)	1316 (1182 to 1466)	216 (162 to 280)	1068 (950 to 1204)	17 (9 to 31)	15 (12 to 17)
Armenia	2032	563 (504 to 632)	93 (70 to 121)	457 (406 to 515)	8 (4 to 13)	6 (5 to 8)	1348 (1205 to 1513)	222 (167 to 289)	1093 (972 to 1233)	18 (9 to 32)	15 (13 to 18)
Armenia	2033	577 (517 to 638)	95 (72 to 124)	468 (415 to 528)	8 (4 to 14)	6 (5 to 8)	1381 (1237 to 1528)	228 (171 to 297)	1119 (995 to 1263)	18 (9 to 33)	15 (13 to 18)
Armenia	2034	592 (527 to 659)	98 (74 to 129)	480 (425 to 541)	8 (4 to 14)	6 (5 to 8)	1418 (1262 to 1578)	235 (176 to 309)	1148 (1018 to 1296)	19 (9 to 34)	15 (13 to 19)
Armenia	2035	608 (543 to 676)	101 (75 to 132)	492 (435 to 555)	8 (4 to 15)	7 (5 to 8)	1455 (1299 to 1618)	242 (180 to 316)	1178 (1042 to 1329)	20 (9 to 35)	16 (13 to 19)
Armenia	2036	623 (557 to 695)	104 (77 to 137)	505 (445 to 569)	8 (4 to 15)	7 (6 to 8)	1492 (1333 to 1665)	248 (185 to 327)	1208 (1066 to 1362)	20 (10 to 36)	16 (13 to 20)
Armenia	2037	638 (569 to 713)	106 (79 to 141)	517 (455 to 583)	9 (4 to 15)	7 (6 to 8)	1528 (1362 to 1706)	255 (188 to 337)	1236 (1090 to 1394)	21 (10 to 37)	16 (13 to 20)
Armenia	2038	653 (585 to 727)	109 (80 to 145)	528 (465 to 595)	9 (4 to 16)	7 (6 to 9)	1563 (1399 to 1739)	261 (192 to 347)	1264 (1113 to 1425)	21 (10 to 38)	17 (14 to 21)
Armenia	2039	668 (595 to 746)	112 (83 to 149)	540 (475 to 609)	9 (4 to 16)	7 (6 to 9)	1599 (1423 to 1785)	268 (198 to 357)	1292 (1137 to 1457)	22 (10 to 39)	17 (14 to 22)

Armenia	2040	683 (610 to 761)	115 (85 to 153)	551 (485 to 622)	9 (4 to 17)	7 (6 to 9)	1634 (1459 to 1821)	275 (202 to 365)	1319 (1160 to 1489)	22 (11 to 40)	17 (14 to 22)
Armenia	2041	698 (622 to 777)	118 (87 to 157)	563 (494 to 636)	10 (5 to 17)	7 (6 to 9)	1670 (1488 to 1860)	282 (207 to 376)	1347 (1183 to 1522)	23 (11 to 41)	18 (14 to 23)
Armenia	2042	712 (634 to 794)	121 (89 to 162)	574 (503 to 649)	10 (5 to 17)	8 (6 to 10)	1704 (1518 to 1900)	289 (213 to 388)	1373 (1203 to 1553)	24 (11 to 42)	18 (15 to 24)
Armenia	2043	726 (647 to 814)	124 (91 to 166)	584 (512 to 660)	10 (5 to 18)	8 (6 to 10)	1737 (1548 to 1949)	297 (218 to 398)	1397 (1226 to 1581)	24 (12 to 43)	19 (15 to 24)
Armenia	2044	739 (655 to 822)	127 (93 to 171)	594 (521 to 672)	10 (5 to 18)	8 (6 to 11)	1770 (1567 to 1968)	305 (223 to 409)	1421 (1248 to 1609)	25 (12 to 44)	19 (15 to 25)
Armenia	2045	752 (666 to 844)	131 (95 to 177)	603 (529 to 683)	11 (5 to 19)	8 (6 to 10)	1800 (1593 to 2020)	312 (227 to 423)	1443 (1265 to 1634)	25 (12 to 45)	19 (15 to 25)
Armenia	2046	765 (681 to 858)	134 (98 to 181)	612 (536 to 694)	11 (5 to 19)	8 (7 to 11)	1832 (1629 to 2054)	321 (235 to 433)	1465 (1282 to 1660)	26 (12 to 46)	20 (16 to 26)
Armenia	2047	778 (692 to 881)	138 (100 to 187)	621 (542 to 704)	11 (5 to 20)	9 (7 to 11)	1863 (1657 to 2108)	330 (240 to 448)	1486 (1297 to 1685)	27 (13 to 47)	20 (16 to 27)
Armenia	2048	791 (701 to 891)	142 (103 to 192)	629 (548 to 713)	11 (5 to 20)	9 (7 to 12)	1893 (1678 to 2132)	339 (247 to 460)	1506 (1313 to 1707)	27 (13 to 48)	21 (16 to 28)
Armenia	2049	804 (712 to 907)	146 (105 to 200)	638 (555 to 722)	12 (6 to 21)	9 (7 to 12)	1925 (1705 to 2172)	349 (251 to 478)	1526 (1329 to 1728)	28 (13 to 49)	21 (17 to 29)

Armenia	2050	817 (724 to 924)	150 (109 to 204)	646 (561 to 731)	12 (6 to 21)	9 (7 to 12)	1956 (1732 to 2212)	360 (262 to 488)	1545 (1343 to 1751)	29 (14 to 50)	22 (17 to 30)
Australia	1995	2731 (2643 to 2819)	1811 (1748 to 1877)	465 (429 to 503)	455 (410 to 503)	0 (0 to 0)	2724 (2636 to 2811)	1806 (1744 to 1872)	463 (427 to 502)	454 (408 to 502)	0 (0 to 0)
Australia	1996	2844 (2774 to 2908)	1897 (1851 to 1942)	503 (473 to 533)	444 (409 to 479)	0 (0 to 0)	2836 (2766 to 2900)	1892 (1846 to 1937)	501 (471 to 531)	442 (408 to 477)	0 (0 to 0)
Australia	1997	2933 (2869 to 2996)	1978 (1932 to 2022)	546 (518 to 573)	409 (379 to 440)	0 (0 to 0)	2925 (2861 to 2988)	1973 (1927 to 2017)	544 (517 to 572)	408 (378 to 438)	0 (0 to 0)
Australia	1998	3100 (3039 to 3162)	2107 (2061 to 2153)	610 (581 to 638)	383 (354 to 412)	0 (0 to 0)	3092 (3031 to 3154)	2101 (2055 to 2147)	609 (580 to 637)	382 (353 to 411)	0 (0 to 0)
Australia	1999	3269 (3213 to 3333)	2242 (2199 to 2289)	657 (628 to 688)	370 (342 to 398)	0 (0 to 0)	3260 (3204 to 3324)	2236 (2193 to 2283)	655 (627 to 686)	369 (341 to 397)	0 (0 to 0)
Australia	2000	3340 (3279 to 3396)	2283 (2237 to 2327)	688 (659 to 716)	369 (344 to 397)	0 (0 to 0)	3331 (3270 to 3387)	2277 (2231 to 2320)	687 (657 to 714)	368 (343 to 396)	0 (0 to 0)
Australia	2001	3468 (3407 to 3530)	2365 (2319 to 2411)	707 (678 to 735)	396 (371 to 425)	0 (0 to 0)	3458 (3397 to 3520)	2359 (2313 to 2405)	705 (677 to 733)	394 (370 to 423)	0 (0 to 0)
Australia	2002	3601 (3538 to 3662)	2457 (2412 to 2505)	718 (691 to 746)	426 (399 to 455)	0 (0 to 0)	3591 (3528 to 3652)	2451 (2405 to 2498)	716 (689 to 744)	425 (398 to 453)	0 (0 to 0)
Australia	2003	3727 (3666 to 3791)	2542 (2497 to 2591)	726 (701 to 755)	460 (431 to 489)	0 (0 to 0)	3717 (3656 to 3781)	2535 (2490 to 2584)	724 (699 to 753)	459 (430 to 488)	0 (0 to 0)

Australia	2004	3872 (3813 to 3934)	2644 (2602 to 2692)	748 (721 to 776)	480 (449 to 511)	0 (0 to 0)	3861 (3802 to 3923)	2637 (2595 to 2684)	746 (719 to 774)	479 (448 to 510)	0 (0 to 0)
Australia	2005	3952 (3888 to 4017)	2703 (2657 to 2750)	767 (740 to 796)	481 (452 to 512)	0 (0 to 0)	3941 (3878 to 4006)	2696 (2650 to 2742)	765 (738 to 794)	480 (451 to 511)	0 (0 to 0)
Australia	2006	4017 (3951 to 4076)	2755 (2708 to 2799)	782 (754 to 812)	481 (452 to 514)	0 (0 to 0)	4006 (3940 to 4065)	2747 (2701 to 2792)	779 (752 to 810)	480 (450 to 513)	0 (0 to 0)
Australia	2007	4114 (4050 to 4175)	2837 (2789 to 2884)	792 (766 to 820)	486 (456 to 516)	0 (0 to 0)	4103 (4039 to 4164)	2829 (2781 to 2876)	790 (764 to 818)	484 (455 to 514)	0 (0 to 0)
Australia	2008	4234 (4174 to 4297)	2927 (2879 to 2973)	810 (784 to 839)	497 (468 to 526)	0 (0 to 0)	4223 (4163 to 4285)	2919 (2871 to 2965)	808 (782 to 837)	496 (467 to 525)	0 (0 to 0)
Australia	2009	4407 (4341 to 4467)	3051 (3004 to 3101)	842 (813 to 871)	514 (486 to 546)	0 (0 to 0)	4395 (4330 to 4455)	3043 (2996 to 3092)	839 (811 to 868)	513 (484 to 545)	0 (0 to 0)
Australia	2010	4433 (4365 to 4496)	3056 (3007 to 3107)	861 (833 to 892)	517 (488 to 548)	0 (0 to 0)	4421 (4353 to 4484)	3048 (2999 to 3099)	858 (830 to 889)	515 (486 to 547)	0 (0 to 0)
Australia	2011	4478 (4418 to 4544)	3070 (3020 to 3121)	877 (847 to 908)	531 (504 to 560)	0 (0 to 0)	4466 (4406 to 4532)	3062 (3012 to 3112)	875 (845 to 906)	529 (502 to 559)	0 (0 to 0)
Australia	2012	4597 (4529 to 4663)	3121 (3070 to 3167)	909 (879 to 941)	568 (539 to 598)	0 (0 to 0)	4585 (4517 to 4651)	3112 (3062 to 3159)	906 (877 to 938)	566 (537 to 597)	0 (0 to 0)
Australia	2013	4690 (4626 to 4758)	3165 (3117 to 3212)	928 (898 to 956)	597 (566 to 629)	0 (0 to 0)	4677 (4613 to 4745)	3156 (3109 to 3203)	925 (896 to 954)	595 (564 to 628)	0 (0 to 0)

Australia	2014	4864 (4795 to 4929)	3287 (3237 to 3333)	946 (917 to 978)	631 (598 to 664)	0 (0 to 0)	4851 (4782 to 4916)	3278 (3229 to 3324)	944 (914 to 976)	629 (596 to 662)	0 (0 to 0)
Australia	2015	5051 (4986 to 5118)	3439 (3395 to 3495)	956 (925 to 988)	656 (623 to 692)	0 (0 to 0)	5038 (4973 to 5105)	3430 (3386 to 3485)	953 (923 to 985)	655 (622 to 690)	0 (0 to 0)
Australia	2016	5168 (5104 to 5235)	3544 (3496 to 3595)	952 (924 to 984)	672 (637 to 707)	0 (0 to 0)	5154 (5090 to 5221)	3534 (3487 to 3585)	950 (921 to 982)	670 (635 to 705)	0 (0 to 0)
Australia	2017	5195 (5108 to 5280)	3577 (3511 to 3643)	943 (904 to 987)	674 (626 to 722)	0 (0 to 0)	5181 (5095 to 5266)	3568 (3501 to 3633)	941 (901 to 985)	672 (624 to 720)	0 (0 to 0)
Australia	2018	5274 (5179 to 5367)	3638 (3553 to 3718)	950 (911 to 995)	686 (637 to 734)	0 (0 to 0)	5260 (5165 to 5352)	3628 (3543 to 3708)	948 (909 to 992)	684 (635 to 732)	0 (0 to 0)
Australia	2019	5306 (5206 to 5403)	3663 (3571 to 3753)	952 (912 to 997)	691 (641 to 741)	0 (0 to 0)	5291 (5192 to 5389)	3653 (3561 to 3743)	949 (910 to 994)	689 (640 to 739)	0 (0 to 0)
Australia	2020	5368 (5267 to 5473)	3711 (3611 to 3810)	959 (920 to 1004)	699 (649 to 749)	0 (0 to 0)	5354 (5253 to 5458)	3701 (3601 to 3800)	956 (917 to 1001)	697 (647 to 747)	0 (0 to 0)
Australia	2021	5415 (5310 to 5522)	3747 (3644 to 3852)	963 (922 to 1009)	706 (655 to 757)	0 (0 to 0)	5400 (5295 to 5507)	3737 (3634 to 3841)	960 (920 to 1006)	704 (653 to 755)	0 (0 to 0)
Australia	2022	5464 (5354 to 5575)	3785 (3675 to 3905)	967 (926 to 1014)	712 (661 to 765)	0 (0 to 0)	5450 (5340 to 5560)	3775 (3665 to 3895)	964 (924 to 1011)	711 (659 to 763)	0 (0 to 0)
Australia	2023	5516 (5407 to 5632)	3826 (3712 to 3953)	970 (930 to 1017)	719 (668 to 774)	0 (0 to 0)	5501 (5392 to 5617)	3816 (3702 to 3942)	968 (927 to 1014)	718 (666 to 772)	0 (0 to 0)

Australia	2024	5568 (5448 to 5690)	3868 (3746 to 4001)	974 (932 to 1020)	726 (674 to 781)	0 (0 to 0)	5553 (5433 to 5675)	3857 (3736 to 3990)	971 (930 to 1017)	725 (672 to 779)	0 (0 to 0)
Australia	2025	5625 (5507 to 5752)	3914 (3785 to 4052)	977 (935 to 1023)	734 (680 to 790)	0 (0 to 0)	5610 (5492 to 5737)	3904 (3775 to 4041)	974 (933 to 1021)	732 (678 to 788)	0 (0 to 0)
Australia	2026	5695 (5578 to 5826)	3971 (3841 to 4118)	981 (939 to 1029)	743 (687 to 800)	0 (0 to 0)	5680 (5562 to 5810)	3960 (3830 to 4107)	978 (937 to 1026)	741 (686 to 798)	0 (0 to 0)
Australia	2027	5769 (5658 to 5902)	4031 (3894 to 4184)	986 (944 to 1034)	753 (694 to 810)	0 (0 to 0)	5754 (5643 to 5886)	4020 (3883 to 4173)	983 (941 to 1031)	751 (692 to 808)	0 (0 to 0)
Australia	2028	5845 (5717 to 5979)	4091 (3954 to 4252)	992 (949 to 1040)	762 (703 to 822)	0 (0 to 0)	5829 (5702 to 5963)	4079 (3943 to 4241)	989 (946 to 1038)	760 (701 to 820)	0 (0 to 0)
Australia	2029	5924 (5795 to 6057)	4153 (4013 to 4319)	998 (954 to 1048)	773 (710 to 835)	0 (0 to 0)	5908 (5779 to 6040)	4142 (4002 to 4307)	995 (951 to 1046)	771 (708 to 833)	0 (0 to 0)
Australia	2030	6003 (5868 to 6154)	4216 (4067 to 4388)	1004 (959 to 1057)	783 (719 to 848)	0 (0 to 0)	5987 (5852 to 6137)	4204 (4056 to 4376)	1002 (956 to 1054)	781 (717 to 846)	0 (0 to 0)
Australia	2031	6084 (5939 to 6241)	4279 (4128 to 4451)	1011 (965 to 1065)	794 (727 to 862)	0 (0 to 0)	6068 (5923 to 6224)	4267 (4117 to 4439)	1008 (963 to 1062)	792 (725 to 860)	0 (0 to 0)
Australia	2032	6164 (6018 to 6319)	4342 (4179 to 4521)	1017 (970 to 1072)	805 (735 to 875)	0 (0 to 0)	6147 (6001 to 6302)	4330 (4168 to 4509)	1014 (968 to 1070)	803 (733 to 873)	0 (0 to 0)
Australia	2033	6238 (6085 to 6402)	4400 (4234 to 4589)	1023 (975 to 1079)	815 (744 to 887)	0 (0 to 0)	6221 (6068 to 6385)	4388 (4223 to 4577)	1020 (972 to 1076)	813 (742 to 885)	0 (0 to 0)

Australia	2034	6311 (6159 to 6477)	4458 (4287 to 4648)	1028 (979 to 1086)	825 (753 to 901)	0 (0 to 0)	6294 (6142 to 6460)	4446 (4276 to 4635)	1025 (976 to 1083)	822 (751 to 898)	0 (0 to 0)
Australia	2035	6383 (6225 to 6546)	4515 (4336 to 4709)	1033 (982 to 1091)	834 (761 to 914)	0 (0 to 0)	6366 (6209 to 6528)	4503 (4324 to 4696)	1030 (980 to 1088)	832 (759 to 912)	0 (0 to 0)
Australia	2036	6459 (6295 to 6626)	4575 (4392 to 4775)	1039 (987 to 1099)	845 (769 to 928)	0 (0 to 0)	6442 (6278 to 6608)	4563 (4380 to 4762)	1036 (984 to 1096)	842 (767 to 926)	0 (0 to 0)
Australia	2037	6536 (6366 to 6715)	4635 (4442 to 4844)	1045 (991 to 1107)	855 (777 to 942)	0 (0 to 0)	6518 (6348 to 6697)	4623 (4430 to 4831)	1042 (988 to 1104)	853 (775 to 940)	0 (0 to 0)
Australia	2038	6618 (6437 to 6812)	4699 (4497 to 4909)	1052 (996 to 1118)	867 (786 to 957)	0 (0 to 0)	6600 (6419 to 6794)	4686 (4484 to 4896)	1049 (994 to 1115)	864 (784 to 954)	0 (0 to 0)
Australia	2039	6701 (6512 to 6898)	4763 (4554 to 4988)	1060 (1002 to 1128)	878 (794 to 974)	0 (0 to 0)	6683 (6494 to 6879)	4750 (4541 to 4975)	1057 (999 to 1125)	876 (792 to 971)	0 (0 to 0)
Australia	2040	6783 (6588 to 6984)	4826 (4613 to 5051)	1068 (1007 to 1139)	890 (803 to 989)	0 (0 to 0)	6765 (6570 to 6965)	4813 (4600 to 5038)	1065 (1005 to 1136)	888 (801 to 986)	0 (0 to 0)
Australia	2041	6867 (6684 to 7077)	4889 (4668 to 5128)	1076 (1013 to 1150)	902 (811 to 1004)	0 (0 to 0)	6849 (6665 to 7058)	4876 (4655 to 5114)	1073 (1010 to 1147)	900 (809 to 1002)	0 (0 to 0)
Australia	2042	6950 (6750 to 7173)	4952 (4725 to 5193)	1084 (1019 to 1160)	914 (820 to 1021)	0 (0 to 0)	6931 (6732 to 7154)	4938 (4712 to 5179)	1081 (1016 to 1157)	912 (818 to 1018)	0 (0 to 0)
Australia	2043	7033 (6821 to 7248)	5015 (4777 to 5271)	1092 (1025 to 1170)	926 (830 to 1037)	0 (0 to 0)	7014 (6803 to 7229)	5002 (4765 to 5257)	1089 (1022 to 1167)	924 (827 to 1035)	0 (0 to 0)

Australia	2044	7117 (6899 to 7343)	5079 (4826 to 5342)	1100 (1031 to 1180)	938 (838 to 1054)	0 (0 to 0)	7098 (6880 to 7323)	5065 (4813 to 5327)	1097 (1028 to 1177)	936 (836 to 1051)	0 (0 to 0)
Australia	2045	7200 (6966 to 7422)	5142 (4883 to 5410)	1108 (1036 to 1190)	950 (847 to 1069)	0 (0 to 0)	7180 (6947 to 7402)	5128 (4870 to 5395)	1105 (1033 to 1187)	948 (845 to 1066)	0 (0 to 0)
Australia	2046	7284 (7047 to 7542)	5206 (4942 to 5481)	1116 (1041 to 1200)	963 (856 to 1083)	0 (0 to 0)	7265 (7028 to 7522)	5192 (4928 to 5466)	1113 (1039 to 1197)	960 (853 to 1080)	0 (0 to 0)
Australia	2047	7368 (7115 to 7637)	5270 (4999 to 5552)	1123 (1046 to 1211)	975 (865 to 1098)	0 (0 to 0)	7348 (7095 to 7617)	5256 (4986 to 5537)	1120 (1043 to 1208)	972 (862 to 1095)	0 (0 to 0)
Australia	2048	7453 (7172 to 7744)	5335 (5053 to 5625)	1131 (1050 to 1221)	987 (873 to 1114)	0 (0 to 0)	7433 (7152 to 7723)	5321 (5040 to 5609)	1127 (1047 to 1218)	984 (870 to 1111)	0 (0 to 0)
Australia	2049	7539 (7233 to 7863)	5402 (5104 to 5713)	1138 (1054 to 1234)	999 (881 to 1134)	0 (0 to 0)	7519 (7214 to 7842)	5387 (5090 to 5697)	1135 (1051 to 1230)	997 (879 to 1131)	0 (0 to 0)
Australia	2050	7625 (7303 to 7972)	5469 (5165 to 5793)	1145 (1058 to 1243)	1012 (890 to 1153)	0 (0 to 0)	7605 (7283 to 7950)	5454 (5151 to 5777)	1142 (1055 to 1240)	1009 (887 to 1150)	0 (0 to 0)
Austria	1995	3454 (3362 to 3553)	2395 (2325 to 2471)	580 (544 to 621)	479 (428 to 537)	0 (0 to 0)	3679 (3581 to 3784)	2550 (2477 to 2632)	618 (580 to 661)	510 (456 to 572)	0 (0 to 0)
Austria	1996	3508 (3438 to 3576)	2468 (2413 to 2522)	599 (570 to 627)	441 (405 to 482)	0 (0 to 0)	3736 (3661 to 3808)	2628 (2571 to 2686)	638 (608 to 668)	470 (431 to 513)	0 (0 to 0)
Austria	1997	3522 (3450 to 3585)	2535 (2482 to 2587)	616 (590 to 642)	372 (338 to 405)	0 (0 to 0)	3751 (3675 to 3818)	2699 (2643 to 2756)	656 (628 to 684)	396 (360 to 431)	0 (0 to 0)

Austria	1998	3700 (3631 to 3768)	2711 (2660 to 2766)	646 (620 to 674)	343 (312 to 376)	0 (0 to 0)	3941 (3868 to 4013)	2888 (2833 to 2946)	688 (660 to 718)	366 (332 to 400)	0 (0 to 0)
Austria	1999	3735 (3674 to 3804)	2759 (2707 to 2814)	661 (635 to 688)	316 (288 to 345)	0 (0 to 0)	3978 (3913 to 4051)	2938 (2884 to 2997)	704 (676 to 733)	337 (307 to 368)	0 (0 to 0)
Austria	2000	3852 (3791 to 3916)	2854 (2800 to 2908)	689 (665 to 715)	310 (285 to 336)	0 (0 to 0)	4103 (4038 to 4171)	3039 (2982 to 3097)	734 (708 to 762)	330 (304 to 358)	0 (0 to 0)
Austria	2001	3897 (3834 to 3960)	2875 (2821 to 2925)	715 (692 to 738)	308 (282 to 335)	0 (0 to 0)	4151 (4084 to 4218)	3062 (3004 to 3115)	761 (737 to 786)	328 (300 to 357)	0 (0 to 0)
Austria	2002	4002 (3938 to 4061)	2938 (2890 to 2993)	746 (723 to 769)	318 (291 to 345)	0 (0 to 0)	4263 (4194 to 4325)	3129 (3078 to 3188)	795 (770 to 819)	339 (310 to 368)	0 (0 to 0)
Austria	2003	4010 (3947 to 4070)	2920 (2870 to 2973)	766 (742 to 792)	324 (298 to 351)	0 (0 to 0)	4271 (4204 to 4335)	3110 (3057 to 3166)	816 (791 to 843)	345 (318 to 374)	0 (0 to 0)
Austria	2004	4243 (4178 to 4304)	3081 (3030 to 3133)	808 (783 to 834)	353 (324 to 381)	0 (0 to 0)	4519 (4450 to 4584)	3282 (3227 to 3337)	861 (834 to 888)	376 (346 to 406)	0 (0 to 0)
Austria	2005	4250 (4181 to 4311)	3081 (3029 to 3132)	815 (791 to 841)	354 (328 to 382)	0 (0 to 0)	4527 (4453 to 4592)	3282 (3226 to 3336)	868 (842 to 896)	377 (349 to 407)	0 (0 to 0)
Austria	2006	4363 (4297 to 4435)	3175 (3121 to 3234)	825 (800 to 849)	363 (337 to 390)	0 (0 to 0)	4647 (4577 to 4724)	3381 (3324 to 3444)	878 (852 to 904)	387 (359 to 416)	0 (0 to 0)
Austria	2007	4475 (4410 to 4545)	3268 (3215 to 3323)	834 (811 to 860)	373 (344 to 402)	0 (0 to 0)	4767 (4697 to 4840)	3480 (3425 to 3539)	889 (863 to 916)	397 (367 to 428)	0 (0 to 0)

Austria	2008	4590 (4522 to 4657)	3360 (3306 to 3413)	843 (819 to 869)	387 (359 to 416)	0 (0 to 0)	4888 (4816 to 4960)	3579 (3521 to 3635)	898 (872 to 926)	412 (383 to 443)	0 (0 to 0)
Austria	2009	4723 (4656 to 4787)	3450 (3396 to 3503)	859 (834 to 884)	414 (385 to 443)	0 (0 to 0)	5030 (4959 to 5098)	3675 (3617 to 3731)	915 (889 to 942)	441 (410 to 472)	0 (0 to 0)
Austria	2010	4733 (4665 to 4800)	3441 (3389 to 3493)	874 (848 to 899)	419 (390 to 448)	0 (0 to 0)	5042 (4969 to 5112)	3665 (3610 to 3721)	931 (904 to 958)	446 (415 to 477)	0 (0 to 0)
Austria	2011	4747 (4676 to 4817)	3446 (3394 to 3498)	888 (863 to 914)	413 (383 to 443)	0 (0 to 0)	5056 (4980 to 5131)	3670 (3615 to 3726)	946 (920 to 973)	440 (408 to 472)	0 (0 to 0)
Austria	2012	4859 (4793 to 4925)	3529 (3472 to 3583)	913 (888 to 938)	417 (389 to 448)	0 (0 to 0)	5175 (5105 to 5245)	3758 (3698 to 3817)	973 (946 to 999)	445 (414 to 478)	0 (0 to 0)
Austria	2013	4866 (4797 to 4932)	3523 (3464 to 3581)	926 (899 to 951)	416 (388 to 447)	0 (0 to 0)	5182 (5109 to 5253)	3753 (3690 to 3814)	986 (958 to 1013)	443 (414 to 476)	0 (0 to 0)
Austria	2014	4919 (4849 to 4991)	3562 (3506 to 3624)	938 (912 to 966)	418 (390 to 449)	0 (0 to 0)	5239 (5165 to 5315)	3794 (3734 to 3860)	1000 (971 to 1029)	445 (416 to 478)	0 (0 to 0)
Austria	2015	4914 (4841 to 4982)	3554 (3499 to 3615)	943 (917 to 970)	416 (386 to 448)	0 (0 to 0)	5233 (5156 to 5307)	3785 (3727 to 3850)	1005 (976 to 1033)	443 (411 to 478)	0 (0 to 0)
Austria	2016	4976 (4906 to 5046)	3598 (3542 to 3656)	958 (930 to 986)	421 (391 to 452)	0 (0 to 0)	5300 (5225 to 5375)	3832 (3772 to 3894)	1020 (990 to 1051)	448 (416 to 481)	0 (0 to 0)
Austria	2017	5062 (4941 to 5183)	3661 (3589 to 3730)	973 (935 to 1011)	427 (386 to 471)	0 (0 to 0)	5391 (5263 to 5521)	3899 (3823 to 3973)	1037 (996 to 1077)	455 (411 to 501)	0 (0 to 0)

Austria	2018	5150 (5013 to 5284)	3724 (3635 to 3807)	990 (951 to 1029)	436 (393 to 479)	0 (0 to 0)	5485 (5339 to 5628)	3966 (3872 to 4055)	1055 (1013 to 1096)	464 (419 to 510)	0 (0 to 0)
Austria	2019	5160 (5015 to 5298)	3726 (3631 to 3817)	996 (956 to 1036)	438 (395 to 482)	0 (0 to 0)	5496 (5341 to 5643)	3969 (3867 to 4066)	1061 (1018 to 1103)	467 (421 to 514)	0 (0 to 0)
Austria	2020	5214 (5068 to 5371)	3766 (3659 to 3867)	1005 (964 to 1045)	443 (400 to 488)	0 (0 to 0)	5554 (5397 to 5720)	4011 (3897 to 4118)	1071 (1027 to 1113)	472 (426 to 520)	0 (0 to 0)
Austria	2021	5259 (5095 to 5417)	3802 (3693 to 3906)	1009 (968 to 1049)	448 (404 to 493)	0 (0 to 0)	5601 (5427 to 5770)	4050 (3933 to 4160)	1075 (1031 to 1117)	477 (431 to 525)	0 (0 to 0)
Austria	2022	5302 (5140 to 5470)	3837 (3717 to 3954)	1013 (972 to 1053)	452 (407 to 498)	0 (0 to 0)	5647 (5475 to 5826)	4087 (3959 to 4211)	1079 (1035 to 1122)	481 (434 to 530)	0 (0 to 0)
Austria	2023	5357 (5170 to 5535)	3884 (3754 to 4017)	1016 (975 to 1058)	456 (411 to 504)	0 (0 to 0)	5705 (5507 to 5895)	4137 (3998 to 4278)	1083 (1039 to 1126)	486 (438 to 537)	0 (0 to 0)
Austria	2024	5408 (5207 to 5624)	3927 (3779 to 4076)	1020 (978 to 1061)	461 (415 to 510)	0 (0 to 0)	5760 (5546 to 5990)	4183 (4025 to 4342)	1086 (1041 to 1130)	491 (442 to 543)	0 (0 to 0)
Austria	2025	5450 (5242 to 5663)	3965 (3800 to 4129)	1021 (979 to 1064)	464 (417 to 513)	0 (0 to 0)	5805 (5583 to 6032)	4223 (4047 to 4398)	1088 (1043 to 1133)	494 (444 to 547)	0 (0 to 0)
Austria	2026	5491 (5273 to 5720)	4001 (3832 to 4174)	1023 (980 to 1065)	468 (420 to 519)	0 (0 to 0)	5849 (5616 to 6092)	4261 (4081 to 4445)	1089 (1044 to 1135)	498 (447 to 553)	0 (0 to 0)
Austria	2027	5529 (5293 to 5761)	4035 (3859 to 4216)	1023 (981 to 1066)	471 (422 to 523)	0 (0 to 0)	5889 (5637 to 6136)	4298 (4111 to 4490)	1090 (1045 to 1136)	501 (449 to 558)	0 (0 to 0)

Austria	2028	5561 (5322 to 5812)	4064 (3876 to 4254)	1023 (980 to 1068)	473 (424 to 526)	0 (0 to 0)	5923 (5668 to 6191)	4329 (4128 to 4531)	1090 (1044 to 1138)	504 (451 to 561)	0 (0 to 0)
Austria	2029	5583 (5323 to 5841)	4087 (3889 to 4286)	1021 (977 to 1067)	475 (425 to 529)	0 (0 to 0)	5947 (5670 to 6221)	4353 (4142 to 4565)	1088 (1040 to 1136)	506 (453 to 563)	0 (0 to 0)
Austria	2030	5602 (5335 to 5873)	4106 (3894 to 4319)	1019 (973 to 1066)	477 (426 to 531)	0 (0 to 0)	5966 (5683 to 6255)	4374 (4148 to 4600)	1085 (1036 to 1136)	508 (453 to 565)	0 (0 to 0)
Austria	2031	5624 (5344 to 5910)	4128 (3909 to 4353)	1017 (970 to 1068)	478 (427 to 535)	0 (0 to 0)	5990 (5692 to 6294)	4397 (4164 to 4636)	1083 (1033 to 1137)	509 (454 to 570)	0 (0 to 0)
Austria	2032	5653 (5362 to 5961)	4156 (3924 to 4401)	1016 (967 to 1069)	481 (428 to 538)	0 (0 to 0)	6021 (5711 to 6349)	4427 (4179 to 4688)	1083 (1030 to 1139)	512 (456 to 573)	0 (0 to 0)
Austria	2033	5691 (5379 to 6006)	4189 (3938 to 4446)	1017 (965 to 1072)	484 (430 to 543)	0 (0 to 0)	6061 (5729 to 6397)	4462 (4194 to 4735)	1084 (1028 to 1141)	515 (458 to 578)	0 (0 to 0)
Austria	2034	5729 (5398 to 6076)	4223 (3955 to 4496)	1018 (964 to 1075)	487 (432 to 548)	0 (0 to 0)	6101 (5749 to 6471)	4498 (4212 to 4788)	1085 (1026 to 1145)	519 (460 to 583)	0 (0 to 0)
Austria	2035	5772 (5426 to 6129)	4261 (3978 to 4548)	1020 (963 to 1080)	491 (435 to 553)	0 (0 to 0)	6147 (5779 to 6528)	4538 (4237 to 4844)	1087 (1025 to 1151)	523 (463 to 589)	0 (0 to 0)
Austria	2036	5819 (5452 to 6184)	4302 (4002 to 4608)	1023 (963 to 1086)	495 (438 to 559)	0 (0 to 0)	6198 (5807 to 6587)	4582 (4262 to 4908)	1089 (1025 to 1156)	527 (466 to 596)	0 (0 to 0)
Austria	2037	5877 (5500 to 6274)	4350 (4035 to 4670)	1027 (965 to 1092)	500 (441 to 566)	0 (0 to 0)	6259 (5858 to 6682)	4633 (4298 to 4973)	1094 (1028 to 1163)	532 (470 to 603)	0 (0 to 0)

Austria	2038	5940 (5555 to 6356)	4402 (4072 to 4735)	1032 (968 to 1100)	505 (445 to 573)	0 (0 to 0)	6326 (5917 to 6770)	4689 (4337 to 5043)	1099 (1031 to 1171)	538 (474 to 610)	0 (0 to 0)
Austria	2039	6010 (5597 to 6446)	4460 (4115 to 4805)	1038 (972 to 1108)	512 (449 to 581)	0 (0 to 0)	6401 (5961 to 6866)	4751 (4382 to 5118)	1105 (1035 to 1180)	545 (478 to 619)	0 (0 to 0)
Austria	2040	6081 (5658 to 6535)	4520 (4157 to 4882)	1044 (976 to 1117)	518 (453 to 591)	0 (0 to 0)	6477 (6026 to 6960)	4814 (4427 to 5199)	1111 (1040 to 1190)	552 (482 to 629)	0 (0 to 0)
Austria	2041	6155 (5708 to 6640)	4580 (4205 to 4963)	1050 (980 to 1127)	525 (458 to 600)	0 (0 to 0)	6555 (6079 to 7072)	4879 (4478 to 5286)	1118 (1043 to 1200)	559 (488 to 639)	0 (0 to 0)
Austria	2042	6229 (5783 to 6707)	4641 (4253 to 5044)	1056 (983 to 1136)	531 (463 to 609)	0 (0 to 0)	6634 (6160 to 7144)	4943 (4530 to 5373)	1125 (1047 to 1210)	566 (493 to 648)	0 (0 to 0)
Austria	2043	6301 (5814 to 6825)	4701 (4299 to 5123)	1062 (987 to 1144)	538 (468 to 618)	0 (0 to 0)	6711 (6192 to 7270)	5007 (4579 to 5457)	1132 (1051 to 1219)	573 (499 to 658)	0 (0 to 0)
Austria	2044	6376 (5884 to 6908)	4763 (4350 to 5199)	1069 (992 to 1153)	544 (473 to 626)	0 (0 to 0)	6791 (6267 to 7358)	5073 (4633 to 5538)	1138 (1057 to 1228)	580 (504 to 667)	0 (0 to 0)
Austria	2045	6449 (5963 to 6991)	4823 (4402 to 5271)	1075 (996 to 1161)	551 (477 to 635)	0 (0 to 0)	6869 (6351 to 7446)	5137 (4688 to 5614)	1145 (1061 to 1237)	587 (508 to 677)	0 (0 to 0)
Austria	2046	6522 (6000 to 7055)	4883 (4453 to 5340)	1081 (999 to 1169)	558 (482 to 644)	0 (0 to 0)	6946 (6391 to 7514)	5201 (4742 to 5688)	1152 (1064 to 1246)	594 (514 to 686)	0 (0 to 0)
Austria	2047	6591 (6053 to 7170)	4940 (4500 to 5405)	1087 (1001 to 1179)	564 (487 to 653)	0 (0 to 0)	7020 (6447 to 7637)	5261 (4793 to 5757)	1158 (1067 to 1256)	601 (519 to 695)	0 (0 to 0)

Austria	2048	6654 (6114 to 7241)	4993 (4545 to 5465)	1092 (1003 to 1186)	570 (491 to 660)	0 (0 to 0)	7088 (6512 to 7712)	5318 (4841 to 5821)	1163 (1069 to 1263)	607 (523 to 703)	0 (0 to 0)
Austria	2049	6718 (6144 to 7316)	5045 (4589 to 5526)	1097 (1007 to 1193)	576 (494 to 668)	0 (0 to 0)	7155 (6544 to 7792)	5373 (4887 to 5886)	1168 (1072 to 1270)	613 (526 to 712)	0 (0 to 0)
Austria	2050	6778 (6215 to 7379)	5095 (4630 to 5587)	1102 (1009 to 1200)	581 (497 to 678)	0 (0 to 0)	7219 (6620 to 7859)	5427 (4931 to 5951)	1173 (1075 to 1278)	619 (530 to 722)	0 (0 to 0)
Azerbaijan	1995	54 (43 to 67)	13 (10 to 17)	41 (31 to 53)	0 (0 to 0)	0 (0 to 0)	226 (181 to 280)	56 (42 to 71)	171 (129 to 221)	0 (0 to 0)	0 (0 to 0)
Azerbaijan	1996	54 (43 to 66)	13 (10 to 17)	41 (31 to 52)	0 (0 to 0)	0 (0 to 0)	224 (181 to 274)	54 (41 to 70)	170 (129 to 217)	0 (0 to 0)	0 (0 to 0)
Azerbaijan	1997	52 (43 to 63)	12 (9 to 15)	40 (31 to 50)	0 (0 to 0)	0 (0 to 0)	217 (178 to 265)	51 (39 to 64)	166 (129 to 209)	0 (0 to 1)	0 (0 to 0)
Azerbaijan	1998	51 (43 to 62)	11 (8 to 14)	40 (32 to 49)	0 (0 to 0)	0 (0 to 0)	215 (178 to 259)	46 (35 to 59)	168 (133 to 207)	1 (0 to 2)	0 (0 to 0)
Azerbaijan	1999	55 (47 to 65)	11 (9 to 14)	42 (34 to 52)	0 (0 to 1)	2 (2 to 2)	231 (195 to 274)	47 (36 to 60)	176 (142 to 217)	1 (1 to 2)	7 (7 to 7)
Azerbaijan	2000	57 (49 to 68)	11 (9 to 14)	44 (36 to 54)	0 (0 to 1)	2 (2 to 2)	240 (205 to 284)	46 (36 to 59)	184 (151 to 224)	1 (1 to 2)	9 (9 to 9)
Azerbaijan	2001	59 (50 to 69)	11 (8 to 14)	47 (39 to 57)	0 (0 to 0)	0 (0 to 0)	245 (209 to 288)	44 (34 to 56)	198 (163 to 238)	1 (0 to 2)	2 (2 to 2)
Azerbaijan	2002	75 (64 to 87)	13 (10 to 16)	61 (51 to 73)	0 (0 to 1)	1 (1 to 1)	314 (269 to 364)	54 (42 to 69)	255 (213 to 304)	1 (1 to 2)	3 (3 to 3)
Azerbaijan	2003	93 (80 to 106)	13 (10 to 17)	78 (67 to 91)	0 (0 to 1)	0 (0 to 0)	387 (334 to 445)	56 (44 to 71)	327 (279 to 381)	1 (1 to 2)	2 (2 to 2)
Azerbaijan	2004	117 (102 to 133)	16 (12 to 20)	100 (86 to 116)	0 (0 to 1)	0 (0 to 0)	489 (426 to 556)	66 (51 to 83)	419 (358 to 483)	2 (1 to 3)	2 (2 to 2)
Azerbaijan	2005	141 (124 to 160)	19 (15 to 24)	120 (105 to 138)	1 (0 to 1)	1 (1 to 1)	590 (517 to 669)	80 (62 to 100)	503 (437 to 574)	2 (1 to 4)	5 (5 to 5)

Azerbaijan	2006	168 (151 to 189)	26 (20 to 33)	140 (122 to 160)	1 (0 to 1)	2 (2 to 2)	703 (631 to 790)	109 (85 to 137)	583 (511 to 667)	3 (1 to 5)	8 (8 to 8)
Azerbaijan	2007	183 (164 to 206)	33 (25 to 41)	148 (131 to 169)	1 (0 to 2)	1 (1 to 1)	765 (684 to 859)	136 (106 to 170)	618 (546 to 707)	4 (2 to 7)	6 (6 to 6)
Azerbaijan	2008	200 (180 to 223)	40 (31 to 50)	157 (140 to 178)	1 (1 to 2)	2 (2 to 2)	835 (753 to 932)	167 (132 to 210)	655 (584 to 744)	5 (2 to 9)	8 (8 to 8)
Azerbaijan	2009	215 (194 to 239)	45 (35 to 56)	166 (147 to 187)	1 (1 to 2)	3 (3 to 3)	899 (810 to 999)	188 (148 to 234)	694 (616 to 782)	6 (3 to 10)	11 (11 to 11)
Azerbaijan	2010	214 (193 to 237)	45 (36 to 55)	164 (145 to 185)	1 (1 to 2)	3 (3 to 3)	892 (805 to 989)	188 (150 to 231)	686 (606 to 774)	6 (3 to 10)	12 (12 to 12)
Azerbaijan	2011	222 (201 to 246)	48 (38 to 59)	168 (149 to 190)	1 (1 to 3)	5 (5 to 5)	929 (838 to 1027)	200 (160 to 244)	704 (623 to 793)	6 (3 to 11)	19 (19 to 19)
Azerbaijan	2012	233 (211 to 258)	51 (41 to 62)	178 (158 to 198)	2 (1 to 3)	2 (2 to 2)	973 (879 to 1078)	213 (169 to 261)	743 (659 to 828)	7 (3 to 12)	10 (10 to 10)
Azerbaijan	2013	250 (227 to 275)	53 (43 to 65)	192 (172 to 214)	2 (1 to 3)	3 (3 to 3)	1045 (950 to 1150)	222 (178 to 270)	804 (718 to 894)	7 (3 to 13)	13 (13 to 13)
Azerbaijan	2014	267 (242 to 294)	54 (43 to 67)	210 (187 to 233)	2 (1 to 3)	2 (2 to 2)	1116 (1012 to 1229)	225 (180 to 278)	875 (780 to 973)	7 (4 to 14)	8 (8 to 8)
Azerbaijan	2015	295 (267 to 327)	57 (45 to 70)	236 (210 to 263)	2 (1 to 4)	1 (1 to 1)	1233 (1117 to 1365)	237 (188 to 293)	984 (876 to 1099)	8 (4 to 15)	4 (4 to 4)
Azerbaijan	2016	295 (262 to 328)	52 (41 to 64)	241 (211 to 272)	2 (1 to 4)	1 (1 to 1)	1233 (1095 to 1369)	217 (170 to 269)	1005 (882 to 1135)	8 (4 to 15)	4 (4 to 4)
Azerbaijan	2017	304 (267 to 343)	51 (40 to 64)	250 (215 to 288)	2 (1 to 4)	1 (1 to 1)	1268 (1115 to 1433)	211 (167 to 266)	1043 (897 to 1203)	8 (4 to 16)	5 (5 to 5)

Azerbaijan	2018	306 (268 to 348)	48 (37 to 61)	255 (219 to 294)	2 (1 to 4)	1 (1 to 1)	1277 (1120 to 1456)	200 (156 to 255)	1064 (914 to 1229)	8 (4 to 15)	5 (5 to 5)
Azerbaijan	2019	315 (276 to 355)	50 (39 to 64)	261 (225 to 301)	2 (1 to 4)	1 (1 to 1)	1314 (1153 to 1484)	211 (164 to 268)	1090 (939 to 1256)	9 (4 to 16)	5 (5 to 5)
Azerbaijan	2020	326 (285 to 370)	53 (41 to 68)	269 (231 to 311)	2 (1 to 4)	1 (1 to 1)	1360 (1191 to 1544)	221 (170 to 283)	1125 (966 to 1297)	9 (4 to 17)	5 (5 to 5)
Azerbaijan	2021	330 (290 to 374)	54 (42 to 69)	273 (234 to 314)	2 (1 to 4)	1 (1 to 1)	1380 (1210 to 1561)	226 (174 to 290)	1140 (979 to 1313)	9 (4 to 17)	5 (5 to 6)
Azerbaijan	2022	334 (292 to 380)	55 (42 to 71)	275 (236 to 318)	2 (1 to 4)	1 (1 to 1)	1395 (1221 to 1588)	230 (176 to 297)	1150 (985 to 1327)	9 (4 to 17)	5 (5 to 6)
Azerbaijan	2023	337 (296 to 382)	56 (43 to 73)	278 (237 to 321)	2 (1 to 4)	1 (1 to 1)	1409 (1234 to 1597)	235 (179 to 305)	1159 (990 to 1339)	9 (5 to 17)	6 (5 to 6)
Azerbaijan	2024	340 (297 to 388)	57 (43 to 74)	279 (239 to 323)	2 (1 to 4)	1 (1 to 2)	1421 (1240 to 1619)	239 (181 to 308)	1167 (998 to 1348)	10 (5 to 18)	6 (5 to 6)
Azerbaijan	2025	343 (300 to 390)	58 (44 to 75)	281 (242 to 325)	2 (1 to 4)	1 (1 to 2)	1433 (1255 to 1629)	243 (183 to 315)	1175 (1010 to 1357)	10 (5 to 18)	6 (5 to 7)
Azerbaijan	2026	347 (302 to 395)	59 (44 to 77)	284 (244 to 328)	2 (1 to 4)	1 (1 to 2)	1448 (1263 to 1650)	247 (183 to 321)	1186 (1018 to 1368)	10 (5 to 18)	6 (5 to 7)
Azerbaijan	2027	351 (307 to 399)	60 (45 to 79)	287 (246 to 331)	2 (1 to 4)	1 (1 to 2)	1467 (1284 to 1666)	252 (186 to 331)	1198 (1028 to 1382)	10 (5 to 19)	6 (5 to 7)

Azerbaijan	2028	356 (310 to 406)	62 (45 to 82)	290 (249 to 334)	2 (1 to 5)	1 (1 to 2)	1487 (1296 to 1696)	258 (190 to 341)	1213 (1040 to 1397)	10 (5 to 19)	6 (5 to 7)
Azerbaijan	2029	362 (316 to 410)	63 (46 to 84)	294 (252 to 339)	3 (1 to 5)	1 (1 to 2)	1510 (1321 to 1711)	264 (193 to 349)	1229 (1055 to 1415)	11 (5 to 19)	6 (5 to 7)
Azerbaijan	2030	368 (321 to 418)	65 (47 to 86)	299 (256 to 343)	3 (1 to 5)	1 (1 to 2)	1535 (1339 to 1747)	271 (196 to 359)	1247 (1068 to 1434)	11 (5 to 20)	6 (5 to 7)
Azerbaijan	2031	373 (326 to 423)	66 (48 to 88)	303 (259 to 349)	3 (1 to 5)	2 (1 to 2)	1560 (1363 to 1765)	277 (199 to 369)	1265 (1081 to 1456)	11 (5 to 20)	6 (5 to 8)
Azerbaijan	2032	379 (330 to 431)	68 (49 to 90)	307 (263 to 353)	3 (1 to 5)	2 (1 to 2)	1585 (1379 to 1799)	284 (206 to 377)	1283 (1097 to 1475)	11 (5 to 21)	6 (6 to 8)
Azerbaijan	2033	386 (337 to 438)	70 (50 to 94)	312 (266 to 358)	3 (1 to 5)	2 (1 to 2)	1611 (1406 to 1828)	291 (210 to 392)	1302 (1111 to 1495)	11 (6 to 21)	7 (6 to 8)
Azerbaijan	2034	392 (341 to 446)	71 (52 to 96)	316 (270 to 364)	3 (1 to 5)	2 (1 to 2)	1638 (1426 to 1863)	298 (215 to 403)	1321 (1126 to 1520)	12 (6 to 22)	7 (6 to 8)
Azerbaijan	2035	399 (348 to 452)	73 (52 to 100)	321 (273 to 370)	3 (1 to 5)	2 (1 to 2)	1667 (1453 to 1890)	306 (217 to 419)	1342 (1142 to 1544)	12 (6 to 22)	7 (6 to 8)
Azerbaijan	2036	406 (354 to 462)	75 (52 to 102)	326 (278 to 375)	3 (1 to 5)	2 (1 to 2)	1694 (1479 to 1930)	313 (218 to 428)	1362 (1159 to 1565)	12 (6 to 23)	7 (6 to 9)
Azerbaijan	2037	412 (359 to 468)	77 (53 to 105)	331 (281 to 380)	3 (1 to 6)	2 (1 to 2)	1722 (1498 to 1954)	321 (221 to 438)	1381 (1175 to 1588)	13 (6 to 23)	7 (6 to 9)

Azerbaijan	2038	420 (365 to 478)	79 (55 to 108)	336 (286 to 387)	3 (1 to 6)	2 (1 to 2)	1753 (1526 to 1998)	330 (229 to 449)	1403 (1194 to 1616)	13 (6 to 24)	7 (6 to 9)
Azerbaijan	2039	427 (370 to 485)	81 (56 to 111)	341 (290 to 393)	3 (2 to 6)	2 (1 to 2)	1782 (1544 to 2024)	338 (234 to 465)	1424 (1212 to 1643)	13 (6 to 24)	7 (6 to 9)
Azerbaijan	2040	434 (377 to 496)	83 (57 to 114)	346 (294 to 400)	3 (2 to 6)	2 (1 to 2)	1813 (1574 to 2071)	346 (238 to 478)	1446 (1228 to 1671)	13 (7 to 25)	7 (6 to 9)
Azerbaijan	2041	441 (382 to 503)	85 (58 to 116)	351 (298 to 406)	3 (2 to 6)	2 (1 to 2)	1843 (1596 to 2099)	355 (242 to 486)	1466 (1244 to 1694)	14 (7 to 25)	8 (6 to 10)
Azerbaijan	2042	448 (389 to 513)	87 (59 to 120)	356 (301 to 411)	3 (2 to 6)	2 (2 to 2)	1872 (1625 to 2142)	363 (246 to 500)	1487 (1259 to 1719)	14 (7 to 26)	8 (6 to 10)
Azerbaijan	2043	455 (393 to 519)	89 (61 to 125)	360 (305 to 416)	3 (2 to 6)	2 (2 to 2)	1899 (1640 to 2170)	371 (253 to 520)	1505 (1272 to 1737)	14 (7 to 26)	8 (6 to 10)
Azerbaijan	2044	461 (400 to 528)	91 (61 to 126)	364 (307 to 421)	4 (2 to 6)	2 (2 to 3)	1924 (1672 to 2204)	379 (256 to 527)	1522 (1284 to 1757)	15 (7 to 27)	8 (6 to 11)
Azerbaijan	2045	467 (401 to 534)	93 (64 to 128)	369 (310 to 425)	4 (2 to 6)	2 (2 to 3)	1949 (1676 to 2231)	387 (266 to 536)	1539 (1296 to 1776)	15 (7 to 27)	8 (7 to 11)
Azerbaijan	2046	471 (409 to 542)	94 (65 to 132)	372 (313 to 429)	4 (2 to 7)	2 (2 to 3)	1969 (1708 to 2263)	394 (272 to 552)	1552 (1306 to 1794)	15 (7 to 28)	8 (7 to 11)
Azerbaijan	2047	477 (409 to 546)	96 (64 to 136)	375 (315 to 434)	4 (2 to 7)	2 (2 to 3)	1992 (1709 to 2281)	401 (269 to 570)	1566 (1315 to 1812)	15 (7 to 28)	9 (7 to 11)

Azerbaijan	2048	482 (418 to 554)	98 (67 to 140)	378 (317 to 438)	4 (2 to 7)	2 (2 to 3)	2012 (1745 to 2313)	409 (278 to 583)	1579 (1323 to 1829)	16 (8 to 28)	9 (7 to 12)
Azerbaijan	2049	487 (418 to 558)	100 (67 to 141)	381 (319 to 441)	4 (2 to 7)	2 (2 to 3)	2033 (1744 to 2332)	416 (278 to 591)	1592 (1331 to 1843)	16 (8 to 29)	9 (7 to 12)
Azerbaijan	2050	491 (423 to 566)	101 (68 to 145)	383 (320 to 445)	4 (2 to 7)	2 (2 to 3)	2050 (1768 to 2366)	423 (283 to 607)	1602 (1339 to 1857)	16 (8 to 29)	9 (7 to 12)
Bahrain	1995	973 (908 to 1036)	642 (596 to 692)	260 (227 to 296)	70 (51 to 95)	0 (0 to 0)	1915 (1787 to 2039)	1264 (1174 to 1362)	513 (446 to 583)	138 (101 to 187)	0 (0 to 0)
Bahrain	1996	943 (892 to 996)	620 (582 to 656)	255 (227 to 286)	68 (51 to 89)	0 (0 to 0)	1857 (1756 to 1961)	1220 (1146 to 1292)	502 (446 to 562)	135 (100 to 175)	0 (0 to 0)
Bahrain	1997	1055 (1005 to 1106)	707 (669 to 745)	270 (244 to 299)	78 (61 to 100)	0 (0 to 0)	2077 (1979 to 2178)	1391 (1317 to 1467)	532 (480 to 588)	154 (119 to 198)	0 (0 to 0)
Bahrain	1998	972 (928 to 1018)	642 (609 to 676)	257 (233 to 284)	73 (58 to 91)	0 (0 to 0)	1914 (1827 to 2005)	1264 (1199 to 1331)	506 (460 to 559)	143 (113 to 180)	0 (0 to 0)
Bahrain	1999	989 (944 to 1034)	656 (620 to 690)	258 (235 to 285)	76 (60 to 95)	0 (0 to 0)	1946 (1857 to 2035)	1290 (1221 to 1358)	507 (462 to 561)	149 (118 to 186)	0 (0 to 0)
Bahrain	2000	933 (893 to 974)	613 (581 to 646)	248 (227 to 273)	72 (57 to 89)	0 (0 to 0)	1837 (1758 to 1917)	1208 (1143 to 1272)	488 (447 to 537)	141 (113 to 174)	0 (0 to 0)
Bahrain	2001	977 (937 to 1020)	647 (616 to 680)	258 (236 to 283)	73 (58 to 90)	0 (0 to 0)	1924 (1844 to 2008)	1274 (1212 to 1339)	507 (465 to 556)	143 (115 to 177)	0 (0 to 0)

Bahrain	2002	996 (955 to 1038)	658 (628 to 690)	269 (247 to 294)	68 (55 to 84)	0 (0 to 0)	1960 (1881 to 2044)	1296 (1235 to 1358)	530 (486 to 579)	134 (108 to 165)	0 (0 to 0)
Bahrain	2003	947 (906 to 987)	617 (589 to 645)	273 (250 to 298)	57 (44 to 70)	0 (0 to 0)	1863 (1784 to 1942)	1214 (1159 to 1270)	538 (492 to 587)	111 (86 to 138)	0 (0 to 0)
Bahrain	2004	852 (815 to 893)	554 (527 to 585)	254 (232 to 276)	43 (34 to 54)	0 (0 to 0)	1677 (1605 to 1757)	1091 (1037 to 1152)	500 (457 to 543)	86 (67 to 107)	0 (0 to 0)
Bahrain	2005	827 (792 to 864)	547 (520 to 575)	241 (221 to 262)	40 (31 to 51)	0 (0 to 0)	1629 (1559 to 1701)	1076 (1023 to 1132)	474 (434 to 515)	78 (61 to 99)	0 (0 to 0)
Bahrain	2006	798 (762 to 832)	529 (502 to 557)	230 (210 to 249)	38 (30 to 49)	0 (0 to 0)	1570 (1500 to 1637)	1042 (987 to 1096)	452 (414 to 491)	75 (58 to 96)	0 (0 to 0)
Bahrain	2007	801 (766 to 838)	526 (497 to 554)	232 (213 to 252)	42 (34 to 53)	0 (0 to 0)	1577 (1509 to 1650)	1036 (979 to 1091)	457 (419 to 497)	84 (66 to 104)	0 (0 to 0)
Bahrain	2008	842 (804 to 878)	551 (522 to 582)	237 (217 to 258)	53 (43 to 65)	0 (0 to 0)	1657 (1582 to 1729)	1085 (1029 to 1145)	467 (427 to 508)	104 (84 to 128)	0 (0 to 0)
Bahrain	2009	837 (801 to 875)	538 (510 to 566)	240 (220 to 261)	60 (49 to 73)	0 (0 to 0)	1649 (1577 to 1722)	1058 (1003 to 1115)	472 (432 to 514)	119 (97 to 143)	0 (0 to 0)
Bahrain	2010	877 (839 to 918)	563 (534 to 592)	245 (224 to 267)	70 (57 to 84)	0 (0 to 0)	1727 (1651 to 1808)	1107 (1050 to 1165)	482 (441 to 526)	138 (113 to 166)	0 (0 to 0)
Bahrain	2011	860 (822 to 897)	548 (518 to 576)	240 (220 to 261)	72 (59 to 85)	0 (0 to 0)	1693 (1619 to 1767)	1080 (1019 to 1134)	472 (434 to 513)	141 (117 to 168)	0 (0 to 0)

Bahrain	2012	961 (919 to 1000)	622 (589 to 653)	258 (236 to 278)	81 (67 to 96)	0 (0 to 0)	1891 (1810 to 1968)	1225 (1159 to 1285)	507 (465 to 548)	159 (132 to 189)	0 (0 to 0)
Bahrain	2013	1060 (1016 to 1102)	690 (656 to 724)	278 (256 to 300)	92 (77 to 108)	0 (0 to 0)	2087 (2000 to 2170)	1359 (1292 to 1425)	547 (503 to 591)	181 (151 to 213)	0 (0 to 0)
Bahrain	2014	1019 (979 to 1061)	649 (621 to 681)	279 (258 to 301)	91 (77 to 107)	0 (0 to 0)	2005 (1927 to 2089)	1277 (1222 to 1340)	549 (509 to 592)	179 (151 to 211)	0 (0 to 0)
Bahrain	2015	1215 (1168 to 1266)	775 (739 to 812)	323 (299 to 347)	118 (100 to 137)	0 (0 to 0)	2392 (2300 to 2492)	1525 (1455 to 1599)	635 (589 to 684)	232 (196 to 269)	0 (0 to 0)
Bahrain	2016	1229 (1177 to 1279)	757 (720 to 795)	343 (316 to 369)	128 (108 to 152)	0 (0 to 0)	2419 (2317 to 2517)	1491 (1417 to 1565)	675 (623 to 727)	253 (213 to 299)	0 (0 to 0)
Bahrain	2017	1230 (1169 to 1292)	731 (687 to 777)	362 (326 to 400)	137 (112 to 167)	0 (0 to 0)	2422 (2300 to 2543)	1439 (1352 to 1529)	713 (641 to 787)	269 (221 to 328)	0 (0 to 0)
Bahrain	2018	1261 (1195 to 1327)	761 (704 to 815)	360 (324 to 398)	139 (114 to 170)	0 (0 to 0)	2481 (2353 to 2612)	1498 (1386 to 1604)	709 (638 to 783)	274 (225 to 334)	0 (0 to 0)
Bahrain	2019	1223 (1157 to 1285)	727 (667 to 785)	360 (323 to 397)	135 (111 to 165)	0 (0 to 0)	2407 (2277 to 2530)	1432 (1314 to 1546)	708 (636 to 782)	267 (218 to 325)	0 (0 to 0)
Bahrain	2020	1272 (1199 to 1340)	770 (703 to 836)	361 (325 to 399)	141 (115 to 172)	0 (0 to 0)	2503 (2360 to 2637)	1515 (1384 to 1647)	711 (640 to 785)	277 (227 to 338)	0 (0 to 0)
Bahrain	2021	1315 (1240 to 1391)	810 (733 to 885)	361 (325 to 399)	145 (119 to 176)	0 (0 to 0)	2590 (2441 to 2739)	1594 (1444 to 1742)	710 (640 to 785)	286 (233 to 347)	0 (0 to 0)

Bahrain	2022	1360 (1277 to 1446)	850 (767 to 938)	361 (326 to 400)	149 (122 to 181)	0 (0 to 0)	2678 (2514 to 2847)	1673 (1510 to 1847)	711 (641 to 787)	294 (240 to 357)	0 (0 to 0)
Bahrain	2023	1406 (1313 to 1496)	891 (797 to 983)	361 (326 to 399)	154 (125 to 186)	0 (0 to 0)	2767 (2585 to 2946)	1753 (1569 to 1935)	711 (641 to 785)	302 (247 to 367)	0 (0 to 0)
Bahrain	2024	1402 (1307 to 1493)	889 (795 to 984)	361 (325 to 398)	153 (125 to 186)	0 (0 to 0)	2760 (2573 to 2938)	1749 (1566 to 1937)	710 (640 to 783)	301 (246 to 366)	0 (0 to 0)
Bahrain	2025	1399 (1308 to 1493)	886 (791 to 982)	360 (324 to 399)	153 (124 to 186)	0 (0 to 0)	2754 (2576 to 2939)	1744 (1558 to 1934)	709 (638 to 785)	301 (245 to 366)	0 (0 to 0)
Bahrain	2026	1396 (1307 to 1491)	883 (786 to 981)	361 (325 to 399)	152 (124 to 185)	0 (0 to 0)	2748 (2572 to 2935)	1738 (1547 to 1930)	710 (639 to 785)	300 (244 to 364)	0 (0 to 0)
Bahrain	2027	1393 (1299 to 1492)	880 (783 to 981)	361 (323 to 400)	152 (123 to 184)	0 (0 to 0)	2742 (2557 to 2936)	1733 (1542 to 1932)	710 (637 to 788)	299 (243 to 363)	0 (0 to 0)
Bahrain	2028	1390 (1290 to 1487)	878 (779 to 979)	360 (322 to 401)	152 (123 to 185)	0 (0 to 0)	2737 (2539 to 2927)	1729 (1533 to 1926)	709 (634 to 790)	299 (242 to 363)	0 (0 to 0)
Bahrain	2029	1388 (1292 to 1486)	876 (774 to 979)	360 (321 to 402)	151 (123 to 184)	0 (0 to 0)	2732 (2542 to 2925)	1725 (1524 to 1926)	709 (632 to 791)	298 (241 to 362)	0 (0 to 0)
Bahrain	2030	1386 (1287 to 1483)	875 (770 to 977)	360 (320 to 403)	151 (122 to 183)	0 (0 to 0)	2728 (2534 to 2920)	1722 (1516 to 1924)	708 (629 to 794)	298 (241 to 361)	0 (0 to 0)
Bahrain	2031	1383 (1286 to 1483)	873 (765 to 979)	359 (318 to 404)	151 (122 to 183)	0 (0 to 0)	2723 (2532 to 2920)	1718 (1506 to 1928)	707 (627 to 796)	297 (240 to 361)	0 (0 to 0)

Bahrain	2032	1381 (1281 to 1482)	871 (762 to 981)	359 (317 to 405)	151 (122 to 183)	0 (0 to 0)	2718 (2521 to 2918)	1715 (1501 to 1932)	706 (623 to 797)	297 (239 to 361)	0 (0 to 0)
Bahrain	2033	1378 (1276 to 1489)	870 (759 to 984)	358 (315 to 406)	150 (121 to 183)	0 (0 to 0)	2713 (2512 to 2932)	1712 (1495 to 1937)	705 (620 to 800)	296 (239 to 361)	0 (0 to 0)
Bahrain	2034	1374 (1275 to 1485)	867 (756 to 985)	357 (312 to 407)	150 (121 to 183)	0 (0 to 0)	2706 (2510 to 2923)	1707 (1488 to 1938)	703 (615 to 801)	295 (238 to 360)	0 (0 to 0)
Bahrain	2035	1370 (1266 to 1478)	864 (751 to 985)	356 (310 to 406)	150 (120 to 183)	0 (0 to 0)	2697 (2491 to 2909)	1701 (1479 to 1939)	701 (610 to 800)	295 (237 to 360)	0 (0 to 0)
Bahrain	2036	1364 (1255 to 1477)	860 (745 to 983)	355 (307 to 406)	149 (119 to 182)	0 (0 to 0)	2685 (2471 to 2907)	1694 (1467 to 1935)	698 (604 to 800)	293 (235 to 359)	0 (0 to 0)
Bahrain	2037	1357 (1238 to 1473)	856 (740 to 981)	353 (304 to 405)	148 (119 to 182)	0 (0 to 0)	2670 (2436 to 2899)	1685 (1457 to 1930)	694 (598 to 798)	292 (233 to 358)	0 (0 to 0)
Bahrain	2038	1347 (1230 to 1467)	850 (733 to 976)	350 (300 to 405)	147 (118 to 180)	0 (0 to 0)	2651 (2421 to 2888)	1673 (1443 to 1922)	689 (591 to 797)	290 (231 to 355)	0 (0 to 0)
Bahrain	2039	1335 (1207 to 1463)	842 (725 to 970)	347 (296 to 404)	146 (116 to 179)	0 (0 to 0)	2628 (2376 to 2880)	1658 (1428 to 1910)	683 (583 to 796)	287 (229 to 352)	0 (0 to 0)
Bahrain	2040	1321 (1205 to 1445)	834 (717 to 963)	343 (292 to 403)	144 (115 to 177)	0 (0 to 0)	2601 (2372 to 2844)	1642 (1412 to 1895)	676 (574 to 792)	284 (226 to 349)	0 (0 to 0)
Bahrain	2041	1309 (1190 to 1435)	826 (709 to 955)	340 (287 to 400)	143 (114 to 176)	0 (0 to 0)	2576 (2343 to 2824)	1626 (1396 to 1881)	669 (565 to 787)	281 (223 to 346)	0 (0 to 0)

Bahrain	2042	1295 (1175 to 1419)	818 (701 to 948)	336 (282 to 396)	141 (112 to 174)	0 (0 to 0)	2550 (2313 to 2793)	1610 (1379 to 1866)	661 (556 to 780)	278 (221 to 343)	0 (0 to 0)
Bahrain	2043	1281 (1156 to 1405)	809 (692 to 939)	332 (278 to 394)	140 (111 to 172)	0 (0 to 0)	2521 (2276 to 2766)	1593 (1362 to 1848)	653 (547 to 775)	275 (218 to 339)	0 (0 to 0)
Bahrain	2044	1264 (1143 to 1396)	799 (682 to 928)	327 (272 to 390)	138 (109 to 170)	0 (0 to 0)	2488 (2250 to 2748)	1573 (1342 to 1826)	644 (536 to 768)	272 (214 to 335)	0 (0 to 0)
Bahrain	2045	1246 (1121 to 1378)	788 (672 to 916)	322 (267 to 387)	136 (107 to 168)	0 (0 to 0)	2454 (2206 to 2712)	1552 (1324 to 1804)	634 (526 to 762)	268 (211 to 331)	0 (0 to 0)
Bahrain	2046	1231 (1109 to 1363)	779 (665 to 906)	318 (262 to 383)	134 (106 to 166)	0 (0 to 0)	2424 (2184 to 2683)	1534 (1308 to 1784)	625 (516 to 755)	265 (208 to 328)	0 (0 to 0)
Bahrain	2047	1221 (1092 to 1353)	773 (660 to 900)	314 (258 to 380)	133 (105 to 166)	0 (0 to 0)	2403 (2149 to 2663)	1523 (1298 to 1771)	618 (509 to 749)	262 (206 to 326)	0 (0 to 0)
Bahrain	2048	1214 (1086 to 1353)	770 (657 to 896)	311 (255 to 378)	133 (104 to 165)	0 (0 to 0)	2389 (2137 to 2664)	1516 (1293 to 1764)	612 (503 to 744)	261 (204 to 325)	0 (0 to 0)
Bahrain	2049	1209 (1081 to 1345)	768 (656 to 894)	308 (253 to 376)	132 (103 to 165)	0 (0 to 0)	2380 (2128 to 2648)	1512 (1291 to 1759)	607 (497 to 740)	260 (203 to 325)	0 (0 to 0)
Bahrain	2050	1206 (1077 to 1349)	768 (655 to 894)	306 (250 to 374)	132 (103 to 165)	0 (0 to 0)	2375 (2121 to 2657)	1512 (1288 to 1759)	603 (493 to 737)	260 (202 to 325)	0 (0 to 0)
Bangladesh	1995	17 (13 to 22)	4 (3 to 6)	11 (8 to 16)	1 (0 to 1)	1 (1 to 1)	45 (36 to 57)	12 (9 to 15)	30 (21 to 41)	2 (1 to 3)	2 (2 to 2)
Bangladesh	1996	16 (13 to 21)	4 (3 to 5)	11 (7 to 15)	1 (0 to 1)	1 (1 to 1)	43 (34 to 54)	11 (8 to 14)	28 (19 to 39)	2 (1 to 3)	2 (2 to 2)

Bangladesh	1997	16 (12 to 20)	4 (3 to 5)	10 (7 to 14)	1 (0 to 1)	1 (1 to 1)	41 (33 to 52)	11 (8 to 14)	26 (18 to 36)	2 (1 to 3)	3 (3 to 3)
Bangladesh	1998	16 (13 to 20)	4 (3 to 5)	10 (7 to 13)	1 (0 to 1)	2 (2 to 2)	43 (35 to 54)	10 (8 to 14)	25 (18 to 35)	2 (1 to 3)	6 (6 to 6)
Bangladesh	1999	16 (13 to 20)	4 (3 to 5)	9 (7 to 13)	1 (0 to 1)	2 (2 to 2)	42 (34 to 53)	10 (7 to 13)	25 (17 to 35)	1 (1 to 3)	6 (6 to 6)
Bangladesh	2000	16 (13 to 21)	4 (3 to 5)	10 (7 to 14)	1 (0 to 1)	2 (2 to 2)	43 (35 to 54)	11 (8 to 14)	26 (19 to 37)	1 (1 to 3)	5 (5 to 5)
Bangladesh	2001	17 (14 to 22)	4 (3 to 6)	11 (8 to 15)	1 (0 to 1)	2 (2 to 2)	45 (36 to 58)	11 (8 to 15)	29 (20 to 40)	1 (1 to 3)	4 (4 to 4)
Bangladesh	2002	18 (14 to 23)	4 (3 to 6)	11 (8 to 16)	1 (0 to 1)	1 (1 to 1)	46 (37 to 59)	12 (9 to 15)	30 (21 to 42)	1 (1 to 3)	3 (3 to 3)
Bangladesh	2003	19 (15 to 24)	5 (3 to 6)	12 (9 to 17)	1 (0 to 1)	1 (1 to 1)	49 (39 to 63)	12 (9 to 16)	32 (23 to 46)	2 (1 to 3)	3 (3 to 3)
Bangladesh	2004	19 (15 to 25)	5 (4 to 6)	13 (9 to 18)	1 (0 to 1)	1 (1 to 1)	51 (40 to 66)	12 (9 to 16)	34 (24 to 48)	2 (1 to 3)	3 (3 to 3)
Bangladesh	2005	21 (17 to 27)	5 (4 to 6)	14 (10 to 20)	1 (0 to 1)	2 (2 to 2)	56 (44 to 72)	13 (10 to 17)	37 (26 to 52)	2 (1 to 3)	4 (4 to 4)
Bangladesh	2006	23 (18 to 28)	5 (4 to 7)	15 (11 to 21)	1 (0 to 1)	2 (2 to 2)	60 (47 to 75)	14 (10 to 18)	40 (28 to 56)	2 (1 to 4)	4 (4 to 4)
Bangladesh	2007	23 (18 to 30)	5 (4 to 7)	16 (11 to 22)	1 (0 to 1)	1 (1 to 1)	61 (47 to 78)	14 (10 to 18)	42 (30 to 58)	2 (1 to 4)	3 (3 to 3)
Bangladesh	2008	26 (20 to 34)	6 (4 to 8)	18 (13 to 24)	1 (0 to 2)	2 (2 to 2)	69 (54 to 89)	16 (11 to 21)	47 (33 to 64)	2 (1 to 4)	4 (4 to 4)
Bangladesh	2009	27 (21 to 34)	6 (4 to 8)	18 (13 to 25)	1 (0 to 2)	2 (2 to 2)	71 (56 to 90)	15 (11 to 20)	49 (35 to 66)	2 (1 to 4)	5 (5 to 5)
Bangladesh	2010	29 (23 to 36)	6 (5 to 8)	20 (14 to 27)	1 (0 to 2)	2 (2 to 2)	76 (59 to 95)	16 (12 to 21)	52 (37 to 71)	2 (1 to 4)	5 (5 to 5)
Bangladesh	2011	31 (24 to 39)	7 (5 to 9)	22 (16 to 30)	1 (0 to 2)	2 (2 to 2)	82 (65 to 104)	18 (13 to 24)	58 (42 to 79)	3 (1 to 5)	4 (4 to 4)

Bangladesh	2012	33 (26 to 42)	7 (5 to 9)	23 (17 to 32)	1 (0 to 2)	2 (2 to 2)	88 (70 to 110)	18 (14 to 24)	61 (44 to 83)	3 (1 to 5)	6 (6 to 6)
Bangladesh	2013	36 (28 to 45)	7 (5 to 9)	25 (18 to 34)	1 (1 to 2)	3 (3 to 3)	94 (75 to 119)	19 (14 to 25)	65 (47 to 89)	3 (1 to 5)	8 (8 to 8)
Bangladesh	2014	36 (28 to 46)	7 (5 to 9)	26 (19 to 36)	1 (1 to 2)	2 (2 to 2)	95 (75 to 122)	18 (14 to 24)	68 (49 to 94)	3 (1 to 5)	6 (6 to 6)
Bangladesh	2015	37 (29 to 48)	7 (5 to 9)	27 (19 to 37)	1 (1 to 2)	2 (2 to 2)	98 (77 to 127)	18 (14 to 24)	71 (51 to 98)	3 (1 to 5)	6 (6 to 6)
Bangladesh	2016	38 (30 to 49)	7 (5 to 9)	28 (20 to 38)	1 (1 to 2)	2 (2 to 2)	101 (78 to 129)	18 (14 to 24)	73 (53 to 101)	3 (1 to 6)	6 (6 to 6)
Bangladesh	2017	40 (31 to 52)	7 (6 to 9)	29 (21 to 40)	1 (1 to 2)	3 (3 to 3)	107 (83 to 136)	19 (15 to 25)	77 (55 to 107)	3 (1 to 6)	7 (7 to 7)
Bangladesh	2018	43 (34 to 55)	8 (6 to 10)	31 (22 to 43)	1 (1 to 2)	2 (2 to 2)	113 (89 to 145)	21 (16 to 27)	82 (59 to 114)	3 (2 to 7)	6 (6 to 6)
Bangladesh	2019	46 (36 to 59)	9 (7 to 11)	33 (24 to 46)	1 (1 to 3)	2 (2 to 2)	120 (94 to 155)	23 (17 to 29)	88 (63 to 121)	4 (2 to 7)	6 (6 to 6)
Bangladesh	2020	48 (38 to 62)	9 (7 to 12)	35 (25 to 49)	1 (1 to 3)	2 (2 to 3)	127 (100 to 163)	24 (18 to 31)	93 (67 to 129)	4 (2 to 7)	6 (6 to 7)
Bangladesh	2021	50 (39 to 64)	9 (7 to 12)	37 (26 to 51)	2 (1 to 3)	2 (2 to 3)	132 (104 to 170)	25 (19 to 32)	97 (69 to 134)	4 (2 to 8)	6 (6 to 7)
Bangladesh	2022	52 (40 to 66)	10 (7 to 13)	38 (27 to 52)	2 (1 to 3)	2 (2 to 3)	136 (107 to 176)	25 (19 to 33)	100 (72 to 138)	4 (2 to 8)	6 (6 to 7)
Bangladesh	2023	53 (42 to 69)	10 (7 to 13)	39 (28 to 54)	2 (1 to 3)	2 (2 to 3)	141 (110 to 181)	26 (20 to 34)	104 (75 to 144)	4 (2 to 8)	6 (6 to 7)
Bangladesh	2024	55 (43 to 71)	10 (8 to 13)	41 (29 to 56)	2 (1 to 3)	2 (2 to 3)	146 (114 to 188)	27 (20 to 35)	108 (77 to 149)	5 (2 to 9)	6 (6 to 7)
Bangladesh	2025	57 (44 to 73)	11 (8 to 14)	42 (30 to 59)	2 (1 to 3)	2 (2 to 3)	151 (117 to 194)	28 (21 to 36)	112 (80 to 155)	5 (2 to 9)	6 (6 to 7)
Bangladesh	2026	59 (46 to 76)	11 (8 to 14)	44 (31 to 61)	2 (1 to 4)	2 (2 to 3)	156 (122 to 200)	29 (21 to 37)	116 (83 to 160)	5 (2 to 9)	6 (6 to 7)

Bangladesh	2027	61 (47 to 78)	11 (8 to 15)	46 (33 to 63)	2 (1 to 4)	2 (2 to 3)	161 (125 to 207)	30 (22 to 39)	120 (86 to 166)	5 (2 to 10)	6 (6 to 7)
Bangladesh	2028	63 (49 to 82)	11 (9 to 15)	47 (34 to 65)	2 (1 to 4)	2 (2 to 3)	167 (129 to 216)	30 (22 to 40)	125 (89 to 172)	5 (2 to 10)	6 (6 to 8)
Bangladesh	2029	65 (51 to 84)	12 (9 to 16)	49 (35 to 68)	2 (1 to 4)	2 (2 to 3)	173 (134 to 223)	31 (23 to 41)	129 (93 to 178)	5 (3 to 10)	7 (6 to 8)
Bangladesh	2030	68 (52 to 87)	12 (9 to 16)	51 (36 to 70)	2 (1 to 4)	2 (2 to 3)	178 (138 to 231)	32 (24 to 42)	134 (96 to 185)	6 (3 to 11)	7 (6 to 8)
Bangladesh	2031	70 (53 to 91)	13 (9 to 17)	53 (38 to 72)	2 (1 to 4)	3 (2 to 3)	184 (141 to 239)	33 (24 to 44)	139 (100 to 191)	6 (3 to 11)	7 (6 to 8)
Bangladesh	2032	72 (57 to 93)	13 (9 to 17)	55 (39 to 75)	2 (1 to 4)	3 (2 to 3)	191 (149 to 246)	34 (25 to 45)	144 (103 to 198)	6 (3 to 11)	7 (6 to 8)
Bangladesh	2033	75 (58 to 97)	13 (10 to 18)	56 (40 to 78)	2 (1 to 4)	3 (2 to 3)	197 (153 to 256)	35 (26 to 47)	149 (106 to 205)	6 (3 to 12)	7 (6 to 8)
Bangladesh	2034	77 (60 to 99)	14 (10 to 18)	58 (42 to 80)	2 (1 to 5)	3 (2 to 3)	203 (159 to 262)	36 (26 to 48)	154 (110 to 212)	6 (3 to 12)	7 (6 to 8)
Bangladesh	2035	80 (61 to 104)	14 (10 to 19)	60 (43 to 83)	2 (1 to 5)	3 (2 to 3)	210 (162 to 275)	37 (27 to 50)	160 (114 to 220)	6 (3 to 12)	7 (6 to 8)
Bangladesh	2036	82 (64 to 106)	14 (11 to 19)	62 (44 to 86)	3 (1 to 5)	3 (2 to 3)	217 (168 to 280)	38 (28 to 51)	165 (117 to 228)	7 (3 to 13)	7 (6 to 9)
Bangladesh	2037	85 (65 to 110)	15 (11 to 20)	65 (46 to 89)	3 (1 to 5)	3 (2 to 3)	224 (172 to 290)	39 (29 to 53)	170 (121 to 236)	7 (3 to 13)	7 (6 to 9)
Bangladesh	2038	87 (67 to 113)	15 (11 to 21)	67 (47 to 92)	3 (1 to 5)	3 (2 to 3)	231 (177 to 299)	40 (29 to 55)	176 (125 to 244)	7 (3 to 14)	7 (6 to 9)
Bangladesh	2039	90 (69 to 117)	16 (11 to 21)	69 (49 to 95)	3 (1 to 5)	3 (2 to 3)	238 (183 to 308)	42 (30 to 56)	182 (128 to 252)	7 (3 to 14)	7 (6 to 9)
Bangladesh	2040	93 (71 to 122)	16 (12 to 22)	71 (50 to 98)	3 (1 to 5)	3 (2 to 4)	245 (188 to 321)	43 (31 to 58)	187 (132 to 260)	8 (4 to 14)	7 (6 to 9)
Bangladesh	2041	96 (74 to 124)	17 (12 to 23)	73 (51 to 101)	3 (1 to 6)	3 (2 to 4)	252 (195 to 327)	44 (32 to 60)	193 (136 to 268)	8 (4 to 15)	8 (6 to 10)

Bangladesh	2042	98 (75 to 129)	17 (12 to 23)	75 (53 to 104)	3 (1 to 6)	3 (2 to 4)	260 (198 to 342)	45 (33 to 61)	199 (139 to 275)	8 (4 to 15)	8 (6 to 10)
Bangladesh	2043	101 (77 to 132)	18 (13 to 24)	78 (54 to 107)	3 (1 to 6)	3 (2 to 4)	267 (202 to 349)	47 (34 to 63)	205 (143 to 284)	8 (4 to 16)	8 (6 to 10)
Bangladesh	2044	104 (80 to 137)	18 (13 to 25)	80 (56 to 111)	3 (1 to 6)	3 (2 to 4)	275 (211 to 363)	48 (35 to 65)	211 (147 to 293)	8 (4 to 16)	8 (6 to 11)
Bangladesh	2045	107 (82 to 141)	19 (14 to 25)	82 (57 to 115)	3 (2 to 6)	3 (2 to 4)	283 (216 to 373)	49 (36 to 67)	217 (151 to 303)	9 (4 to 17)	8 (6 to 10)
Bangladesh	2046	110 (84 to 145)	19 (14 to 26)	85 (59 to 118)	3 (2 to 6)	3 (2 to 4)	291 (223 to 382)	51 (37 to 69)	223 (156 to 313)	9 (4 to 17)	8 (7 to 11)
Bangladesh	2047	114 (87 to 149)	20 (14 to 27)	87 (61 to 122)	4 (2 to 7)	3 (3 to 4)	300 (229 to 393)	53 (38 to 71)	230 (160 to 322)	9 (4 to 18)	8 (7 to 11)
Bangladesh	2048	117 (88 to 153)	21 (15 to 28)	89 (62 to 125)	4 (2 to 7)	3 (3 to 4)	308 (233 to 404)	54 (39 to 74)	236 (164 to 330)	10 (4 to 18)	9 (7 to 11)
Bangladesh	2049	120 (92 to 157)	21 (15 to 29)	92 (64 to 128)	4 (2 to 7)	3 (3 to 4)	317 (243 to 414)	56 (40 to 76)	243 (169 to 338)	10 (5 to 19)	9 (7 to 12)
Bangladesh	2050	123 (93 to 164)	22 (16 to 30)	94 (66 to 131)	4 (2 to 7)	3 (3 to 5)	326 (246 to 432)	58 (42 to 79)	249 (173 to 347)	10 (5 to 19)	9 (7 to 12)
Barbados	1995	1075 (1005 to 1149)	596 (541 to 657)	344 (306 to 380)	79 (56 to 110)	56 (56 to 56)	1114 (1042 to 1192)	618 (561 to 681)	356 (318 to 394)	82 (58 to 114)	58 (58 to 58)
Barbados	1996	1139 (1074 to 1203)	637 (587 to 689)	362 (331 to 394)	85 (64 to 112)	55 (55 to 55)	1181 (1114 to 1247)	661 (609 to 715)	375 (343 to 409)	88 (66 to 116)	57 (57 to 57)
Barbados	1997	1158 (1099 to 1219)	642 (594 to 691)	376 (348 to 405)	86 (66 to 112)	54 (54 to 54)	1201 (1139 to 1264)	666 (616 to 717)	390 (361 to 420)	89 (68 to 116)	56 (56 to 56)
Barbados	1998	1104 (1049 to 1159)	597 (553 to 642)	373 (347 to 398)	81 (63 to 103)	53 (53 to 53)	1145 (1087 to 1202)	619 (573 to 666)	386 (360 to 413)	84 (66 to 107)	55 (55 to 55)

Barbados	1999	1090 (1038 to 1142)	579 (538 to 623)	377 (352 to 404)	81 (64 to 101)	52 (52 to 52)	1130 (1077 to 1184)	600 (558 to 646)	391 (365 to 419)	84 (66 to 105)	54 (54 to 54)
Barbados	2000	1023 (974 to 1075)	561 (519 to 606)	380 (356 to 406)	82 (65 to 100)	0 (0 to 0)	1061 (1010 to 1114)	581 (538 to 628)	395 (369 to 421)	85 (67 to 104)	0 (0 to 0)
Barbados	2001	1038 (989 to 1089)	538 (496 to 580)	373 (350 to 397)	77 (62 to 95)	50 (50 to 50)	1076 (1025 to 1129)	558 (514 to 601)	387 (363 to 412)	80 (64 to 99)	52 (52 to 52)
Barbados	2002	1159 (1099 to 1217)	659 (611 to 706)	412 (385 to 438)	88 (70 to 109)	0 (0 to 0)	1202 (1140 to 1262)	684 (633 to 732)	427 (400 to 454)	91 (73 to 113)	0 (0 to 0)
Barbados	2003	1253 (1194 to 1311)	681 (635 to 728)	424 (397 to 452)	82 (65 to 102)	65 (65 to 65)	1299 (1238 to 1359)	707 (658 to 755)	440 (412 to 469)	85 (68 to 105)	67 (67 to 67)
Barbados	2004	1295 (1236 to 1355)	708 (663 to 757)	436 (407 to 465)	81 (65 to 99)	69 (69 to 69)	1342 (1281 to 1405)	734 (687 to 785)	452 (422 to 482)	84 (67 to 103)	72 (72 to 72)
Barbados	2005	1308 (1249 to 1369)	726 (679 to 776)	447 (417 to 475)	83 (66 to 101)	52 (52 to 52)	1356 (1295 to 1420)	753 (704 to 805)	463 (433 to 493)	86 (69 to 105)	54 (54 to 54)
Barbados	2006	1223 (1167 to 1279)	699 (653 to 746)	443 (417 to 468)	81 (65 to 98)	0 (0 to 0)	1268 (1210 to 1326)	724 (677 to 774)	460 (433 to 486)	84 (68 to 101)	0 (0 to 0)
Barbados	2007	1260 (1205 to 1319)	721 (675 to 770)	456 (431 to 480)	83 (68 to 101)	0 (0 to 0)	1307 (1250 to 1368)	748 (700 to 798)	472 (447 to 497)	86 (70 to 105)	0 (0 to 0)
Barbados	2008	1265 (1210 to 1321)	720 (674 to 765)	464 (440 to 486)	81 (67 to 100)	0 (0 to 0)	1311 (1254 to 1370)	746 (699 to 793)	481 (456 to 504)	84 (69 to 104)	0 (0 to 0)

Barbados	2009	1266 (1213 to 1320)	711 (670 to 758)	476 (452 to 500)	79 (65 to 97)	0 (0 to 0)	1313 (1257 to 1369)	737 (694 to 785)	494 (469 to 519)	82 (67 to 101)	0 (0 to 0)
Barbados	2010	1256 (1203 to 1311)	689 (649 to 736)	488 (463 to 513)	78 (64 to 96)	0 (0 to 0)	1302 (1247 to 1359)	715 (673 to 763)	506 (480 to 532)	81 (67 to 99)	0 (0 to 0)
Barbados	2011	1221 (1170 to 1271)	645 (605 to 691)	500 (475 to 524)	76 (62 to 93)	0 (0 to 0)	1266 (1213 to 1318)	669 (627 to 716)	518 (492 to 543)	78 (65 to 96)	0 (0 to 0)
Barbados	2012	1281 (1228 to 1336)	671 (626 to 720)	530 (505 to 556)	80 (65 to 97)	0 (0 to 0)	1328 (1273 to 1385)	696 (649 to 746)	549 (524 to 576)	83 (68 to 101)	0 (0 to 0)
Barbados	2013	1266 (1214 to 1321)	642 (600 to 688)	541 (518 to 568)	83 (68 to 101)	0 (0 to 0)	1313 (1259 to 1370)	666 (622 to 713)	561 (537 to 589)	86 (70 to 105)	0 (0 to 0)
Barbados	2014	1203 (1152 to 1257)	584 (545 to 625)	537 (512 to 564)	83 (67 to 100)	0 (0 to 0)	1248 (1195 to 1303)	605 (565 to 648)	557 (531 to 584)	86 (69 to 104)	0 (0 to 0)
Barbados	2015	1223 (1168 to 1276)	582 (540 to 626)	553 (527 to 581)	88 (71 to 106)	0 (0 to 0)	1268 (1211 to 1323)	604 (560 to 649)	573 (546 to 602)	91 (74 to 110)	0 (0 to 0)
Barbados	2016	1200 (1140 to 1262)	559 (513 to 609)	554 (527 to 582)	88 (71 to 110)	0 (0 to 0)	1245 (1181 to 1308)	579 (531 to 632)	574 (546 to 603)	91 (73 to 114)	0 (0 to 0)
Barbados	2017	1180 (1119 to 1246)	538 (484 to 597)	554 (518 to 594)	88 (67 to 114)	0 (0 to 0)	1224 (1160 to 1291)	558 (502 to 619)	574 (537 to 616)	92 (69 to 118)	0 (0 to 0)
Barbados	2018	1118 (1046 to 1195)	479 (432 to 533)	551 (515 to 590)	89 (67 to 114)	0 (0 to 0)	1159 (1085 to 1239)	496 (448 to 553)	571 (534 to 612)	92 (70 to 118)	0 (0 to 0)

Barbados	2019	1088 (1033 to 1140)	448 (404 to 498)	551 (514 to 590)	89 (68 to 115)	0 (0 to 0)	1128 (1071 to 1182)	465 (419 to 516)	571 (533 to 612)	92 (70 to 119)	0 (0 to 0)
Barbados	2020	1094 (1023 to 1177)	450 (406 to 500)	554 (518 to 596)	90 (68 to 116)	0 (0 to 0)	1134 (1060 to 1221)	467 (421 to 519)	575 (538 to 618)	93 (71 to 120)	0 (0 to 0)
Barbados	2021	1097 (1047 to 1155)	450 (405 to 502)	557 (521 to 600)	90 (68 to 116)	0 (0 to 0)	1137 (1086 to 1198)	467 (419 to 521)	578 (540 to 622)	93 (71 to 120)	0 (0 to 0)
Barbados	2022	1099 (1026 to 1177)	449 (403 to 501)	559 (522 to 602)	91 (69 to 117)	0 (0 to 0)	1139 (1064 to 1221)	466 (417 to 520)	580 (541 to 624)	94 (72 to 122)	0 (0 to 0)
Barbados	2023	1098 (1047 to 1153)	445 (398 to 496)	561 (524 to 604)	91 (69 to 118)	0 (0 to 0)	1138 (1086 to 1195)	461 (412 to 515)	582 (544 to 626)	95 (72 to 123)	0 (0 to 0)
Barbados	2024	1094 (1018 to 1174)	438 (391 to 490)	563 (526 to 606)	92 (70 to 119)	0 (0 to 0)	1134 (1055 to 1217)	454 (406 to 508)	584 (546 to 628)	96 (72 to 124)	0 (0 to 0)
Barbados	2025	1089 (1042 to 1144)	431 (386 to 483)	565 (527 to 608)	93 (70 to 120)	0 (0 to 0)	1129 (1081 to 1186)	447 (400 to 501)	586 (546 to 630)	96 (73 to 125)	0 (0 to 0)
Barbados	2026	1084 (1013 to 1168)	424 (380 to 476)	567 (528 to 611)	94 (71 to 121)	0 (0 to 0)	1124 (1050 to 1211)	440 (394 to 494)	587 (547 to 633)	97 (73 to 126)	0 (0 to 0)
Barbados	2027	1080 (1030 to 1132)	418 (374 to 470)	568 (530 to 612)	94 (71 to 123)	0 (0 to 0)	1120 (1068 to 1173)	433 (387 to 487)	589 (549 to 635)	98 (74 to 127)	0 (0 to 0)
Barbados	2028	1076 (995 to 1160)	411 (366 to 464)	569 (532 to 614)	95 (72 to 124)	0 (0 to 0)	1115 (1032 to 1203)	427 (380 to 481)	590 (551 to 637)	98 (74 to 128)	0 (0 to 0)

Barbados	2029	1071 (1020 to 1125)	405 (360 to 457)	571 (533 to 615)	96 (72 to 125)	0 (0 to 0)	1110 (1057 to 1166)	420 (373 to 474)	592 (552 to 638)	99 (75 to 129)	0 (0 to 0)
Barbados	2030	1066 (989 to 1152)	399 (353 to 451)	571 (533 to 616)	96 (72 to 126)	0 (0 to 0)	1106 (1025 to 1195)	414 (366 to 468)	592 (553 to 639)	100 (75 to 130)	0 (0 to 0)
Barbados	2031	1062 (1013 to 1118)	393 (348 to 446)	572 (534 to 617)	97 (73 to 127)	0 (0 to 0)	1101 (1050 to 1159)	408 (361 to 462)	593 (553 to 640)	100 (76 to 131)	0 (0 to 0)
Barbados	2032	1058 (979 to 1146)	388 (342 to 441)	573 (534 to 618)	98 (73 to 128)	0 (0 to 0)	1097 (1015 to 1189)	402 (355 to 457)	594 (554 to 641)	101 (76 to 132)	0 (0 to 0)
Barbados	2033	1053 (1002 to 1108)	382 (336 to 435)	573 (533 to 619)	98 (74 to 129)	0 (0 to 0)	1092 (1039 to 1149)	396 (349 to 451)	594 (553 to 642)	102 (77 to 133)	0 (0 to 0)
Barbados	2034	1049 (971 to 1133)	376 (331 to 430)	574 (533 to 620)	99 (75 to 130)	0 (0 to 0)	1088 (1007 to 1175)	390 (343 to 446)	595 (552 to 643)	102 (77 to 135)	0 (0 to 0)
Barbados	2035	1045 (991 to 1104)	371 (325 to 425)	574 (532 to 620)	100 (75 to 131)	0 (0 to 0)	1083 (1028 to 1145)	385 (337 to 441)	595 (552 to 643)	103 (78 to 136)	0 (0 to 0)
Barbados	2036	1042 (958 to 1132)	367 (321 to 420)	574 (532 to 622)	100 (76 to 132)	0 (0 to 0)	1080 (993 to 1174)	381 (332 to 435)	595 (552 to 644)	104 (79 to 137)	0 (0 to 0)
Barbados	2037	1039 (984 to 1101)	364 (317 to 417)	575 (532 to 623)	101 (76 to 133)	0 (0 to 0)	1078 (1020 to 1141)	377 (328 to 432)	596 (551 to 646)	105 (79 to 138)	0 (0 to 0)
Barbados	2038	1038 (951 to 1132)	361 (313 to 415)	575 (531 to 625)	102 (77 to 134)	0 (0 to 0)	1076 (986 to 1174)	374 (324 to 430)	596 (551 to 648)	106 (80 to 139)	0 (0 to 0)

Barbados	2039	1038 (978 to 1099)	359 (310 to 412)	576 (532 to 627)	103 (78 to 136)	0 (0 to 0)	1076 (1014 to 1140)	372 (322 to 428)	597 (552 to 650)	107 (81 to 141)	0 (0 to 0)
Barbados	2040	1039 (957 to 1124)	358 (308 to 411)	577 (533 to 630)	104 (78 to 137)	0 (0 to 0)	1077 (993 to 1166)	371 (320 to 427)	599 (552 to 653)	108 (81 to 142)	0 (0 to 0)
Barbados	2041	1040 (976 to 1108)	356 (306 to 411)	578 (533 to 632)	105 (79 to 139)	0 (0 to 0)	1078 (1012 to 1149)	370 (318 to 426)	600 (553 to 655)	109 (82 to 144)	0 (0 to 0)
Barbados	2042	1042 (955 to 1130)	356 (305 to 411)	580 (534 to 635)	106 (80 to 140)	0 (0 to 0)	1080 (990 to 1172)	369 (316 to 426)	601 (553 to 658)	110 (83 to 145)	0 (0 to 0)
Barbados	2043	1045 (980 to 1112)	356 (304 to 412)	582 (534 to 638)	107 (81 to 142)	0 (0 to 0)	1083 (1016 to 1153)	369 (315 to 428)	603 (554 to 661)	111 (84 to 147)	0 (0 to 0)
Barbados	2044	1049 (962 to 1144)	357 (304 to 414)	584 (535 to 641)	108 (82 to 144)	0 (0 to 0)	1087 (997 to 1187)	370 (315 to 429)	605 (555 to 665)	112 (85 to 149)	0 (0 to 0)
Barbados	2045	1053 (989 to 1122)	358 (305 to 416)	586 (536 to 644)	109 (83 to 145)	0 (0 to 0)	1092 (1026 to 1164)	371 (316 to 431)	607 (556 to 668)	113 (86 to 151)	0 (0 to 0)
Barbados	2046	1058 (968 to 1155)	360 (306 to 419)	588 (537 to 647)	111 (84 to 147)	0 (0 to 0)	1097 (1003 to 1198)	373 (317 to 434)	609 (557 to 671)	115 (87 to 152)	0 (0 to 0)
Barbados	2047	1063 (996 to 1146)	362 (307 to 421)	590 (537 to 650)	112 (84 to 149)	0 (0 to 0)	1102 (1033 to 1188)	375 (319 to 437)	611 (557 to 674)	116 (87 to 154)	0 (0 to 0)
Barbados	2048	1069 (980 to 1173)	364 (309 to 425)	592 (538 to 653)	113 (85 to 151)	0 (0 to 0)	1109 (1016 to 1216)	378 (321 to 441)	614 (558 to 677)	117 (89 to 157)	0 (0 to 0)

Barbados	2049	1076 (1004 to 1159)	368 (312 to 429)	594 (539 to 657)	114 (86 to 153)	0 (0 to 0)	1116 (1041 to 1201)	381 (324 to 445)	616 (559 to 682)	119 (89 to 159)	0 (0 to 0)
Barbados	2050	1083 (988 to 1188)	371 (315 to 433)	596 (540 to 660)	116 (87 to 155)	0 (0 to 0)	1123 (1024 to 1231)	385 (326 to 449)	618 (560 to 685)	120 (91 to 161)	0 (0 to 0)
Belarus	1995	124 (108 to 143)	94 (79 to 111)	21 (16 to 28)	9 (4 to 15)	0 (0 to 0)	390 (339 to 451)	296 (249 to 350)	67 (50 to 89)	27 (13 to 47)	0 (0 to 0)
Belarus	1996	126 (110 to 144)	97 (83 to 114)	20 (15 to 26)	9 (4 to 15)	0 (0 to 0)	395 (347 to 452)	306 (262 to 358)	62 (47 to 82)	27 (13 to 47)	0 (0 to 0)
Belarus	1997	133 (117 to 152)	105 (91 to 121)	19 (14 to 25)	9 (4 to 16)	0 (0 to 0)	419 (369 to 477)	331 (287 to 381)	59 (45 to 78)	28 (14 to 49)	0 (0 to 0)
Belarus	1998	138 (123 to 156)	108 (94 to 123)	21 (16 to 27)	9 (4 to 16)	0 (0 to 0)	435 (385 to 490)	341 (297 to 388)	66 (50 to 85)	29 (14 to 49)	0 (0 to 0)
Belarus	1999	142 (126 to 159)	110 (96 to 124)	23 (18 to 29)	9 (4 to 15)	0 (0 to 0)	446 (396 to 500)	346 (302 to 391)	72 (56 to 92)	28 (14 to 47)	0 (0 to 0)
Belarus	2000	149 (133 to 166)	114 (100 to 128)	26 (20 to 33)	8 (4 to 15)	0 (0 to 0)	468 (417 to 521)	359 (315 to 403)	82 (64 to 103)	27 (13 to 47)	0 (0 to 0)
Belarus	2001	168 (150 to 187)	128 (113 to 144)	32 (25 to 40)	9 (4 to 16)	0 (0 to 0)	529 (470 to 587)	402 (355 to 451)	100 (79 to 125)	27 (14 to 49)	0 (0 to 0)
Belarus	2002	180 (161 to 200)	135 (120 to 152)	37 (29 to 45)	8 (4 to 15)	0 (0 to 0)	567 (506 to 629)	426 (376 to 478)	115 (92 to 143)	26 (13 to 47)	0 (0 to 0)
Belarus	2003	194 (174 to 215)	145 (129 to 162)	41 (32 to 49)	8 (4 to 15)	0 (0 to 0)	610 (546 to 676)	457 (405 to 509)	127 (102 to 155)	26 (13 to 48)	0 (0 to 0)
Belarus	2004	215 (194 to 238)	160 (142 to 178)	46 (37 to 56)	9 (5 to 17)	0 (0 to 0)	676 (609 to 747)	502 (448 to 559)	145 (116 to 175)	28 (14 to 53)	1 (1 to 1)
Belarus	2005	237 (215 to 261)	172 (154 to 192)	54 (44 to 65)	10 (5 to 18)	1 (1 to 1)	744 (678 to 820)	542 (485 to 604)	170 (139 to 204)	30 (15 to 57)	3 (3 to 3)
Belarus	2006	258 (236 to 283)	184 (167 to 204)	63 (52 to 75)	10 (5 to 20)	1 (1 to 1)	812 (741 to 889)	579 (524 to 642)	198 (164 to 236)	33 (16 to 62)	2 (2 to 2)

Belarus	2007	278 (254 to 304)	194 (174 to 215)	73 (61 to 87)	11 (5 to 20)	1 (1 to 1)	876 (799 to 956)	609 (548 to 676)	229 (192 to 274)	35 (17 to 64)	3 (3 to 3)
Belarus	2008	328 (298 to 360)	226 (203 to 251)	88 (74 to 104)	13 (6 to 24)	2 (2 to 2)	1033 (938 to 1132)	711 (638 to 790)	276 (232 to 328)	41 (20 to 76)	5 (5 to 5)
Belarus	2009	305 (277 to 330)	202 (182 to 224)	89 (75 to 105)	11 (5 to 20)	2 (2 to 2)	958 (871 to 1039)	636 (571 to 703)	281 (236 to 331)	35 (17 to 64)	5 (5 to 5)
Belarus	2010	309 (283 to 336)	202 (182 to 224)	95 (80 to 112)	10 (5 to 18)	2 (2 to 2)	972 (891 to 1057)	636 (574 to 704)	299 (251 to 353)	31 (15 to 56)	6 (6 to 6)
Belarus	2011	317 (292 to 347)	205 (184 to 227)	101 (86 to 119)	10 (5 to 18)	2 (2 to 2)	998 (918 to 1090)	644 (580 to 714)	319 (269 to 374)	31 (15 to 56)	5 (5 to 5)
Belarus	2012	330 (303 to 360)	212 (191 to 234)	107 (91 to 127)	10 (5 to 18)	2 (2 to 2)	1039 (953 to 1133)	665 (600 to 735)	338 (287 to 400)	31 (15 to 57)	5 (5 to 5)
Belarus	2013	354 (325 to 388)	226 (205 to 250)	117 (99 to 137)	10 (5 to 19)	2 (2 to 2)	1115 (1022 to 1220)	711 (644 to 787)	367 (312 to 431)	32 (15 to 60)	5 (5 to 5)
Belarus	2014	357 (328 to 390)	227 (206 to 252)	119 (100 to 140)	9 (4 to 17)	1 (1 to 1)	1122 (1030 to 1227)	714 (648 to 791)	374 (316 to 441)	30 (14 to 54)	4 (4 to 4)
Belarus	2015	373 (341 to 410)	242 (218 to 267)	121 (102 to 144)	10 (5 to 17)	1 (1 to 1)	1174 (1073 to 1290)	761 (686 to 840)	381 (319 to 454)	30 (14 to 54)	2 (2 to 2)
Belarus	2016	368 (334 to 406)	246 (220 to 274)	112 (93 to 136)	9 (4 to 16)	1 (1 to 1)	1156 (1051 to 1278)	774 (690 to 862)	352 (291 to 429)	27 (13 to 49)	3 (3 to 3)
Belarus	2017	373 (330 to 422)	254 (222 to 287)	110 (89 to 137)	9 (4 to 16)	1 (1 to 1)	1173 (1038 to 1327)	797 (699 to 902)	344 (278 to 432)	27 (13 to 50)	4 (4 to 4)

Belarus	2018	381 (337 to 431)	259 (225 to 295)	112 (90 to 140)	9 (4 to 16)	1 (1 to 1)	1198 (1061 to 1355)	815 (707 to 927)	351 (284 to 440)	28 (13 to 51)	4 (4 to 4)
Belarus	2019	402 (351 to 459)	278 (238 to 320)	113 (92 to 142)	9 (4 to 17)	1 (1 to 1)	1265 (1105 to 1444)	875 (747 to 1005)	357 (289 to 448)	30 (14 to 54)	4 (4 to 4)
Belarus	2020	410 (356 to 473)	284 (241 to 331)	116 (94 to 145)	10 (5 to 17)	1 (1 to 1)	1290 (1118 to 1487)	893 (759 to 1040)	364 (295 to 457)	30 (14 to 55)	4 (4 to 4)
Belarus	2021	416 (357 to 480)	288 (241 to 338)	117 (94 to 147)	10 (5 to 18)	1 (1 to 1)	1307 (1123 to 1510)	905 (758 to 1062)	367 (297 to 461)	31 (14 to 56)	4 (4 to 4)
Belarus	2022	421 (362 to 494)	292 (243 to 349)	118 (95 to 148)	10 (5 to 18)	1 (1 to 1)	1323 (1137 to 1553)	917 (765 to 1096)	370 (299 to 465)	31 (15 to 56)	4 (4 to 5)
Belarus	2023	425 (361 to 499)	295 (244 to 356)	119 (96 to 149)	10 (5 to 18)	1 (1 to 2)	1338 (1134 to 1569)	929 (766 to 1120)	373 (302 to 469)	31 (15 to 57)	4 (4 to 5)
Belarus	2024	430 (363 to 510)	299 (241 to 367)	120 (97 to 150)	10 (5 to 19)	1 (1 to 2)	1352 (1143 to 1602)	940 (757 to 1155)	376 (304 to 472)	32 (15 to 58)	4 (4 to 5)
Belarus	2025	435 (364 to 521)	303 (241 to 373)	120 (97 to 151)	10 (5 to 19)	1 (1 to 2)	1368 (1143 to 1638)	953 (758 to 1174)	379 (306 to 476)	32 (15 to 59)	4 (4 to 5)
Belarus	2026	440 (369 to 529)	307 (245 to 381)	122 (98 to 152)	10 (5 to 19)	1 (1 to 2)	1384 (1162 to 1663)	965 (769 to 1197)	382 (309 to 479)	33 (15 to 60)	5 (4 to 5)
Belarus	2027	447 (371 to 540)	312 (248 to 386)	123 (99 to 154)	11 (5 to 19)	1 (1 to 2)	1404 (1168 to 1698)	980 (779 to 1213)	386 (312 to 483)	33 (16 to 61)	5 (4 to 5)

Belarus	2028	453 (377 to 550)	316 (249 to 399)	124 (101 to 156)	11 (5 to 20)	2 (1 to 2)	1424 (1187 to 1728)	994 (782 to 1255)	391 (317 to 489)	34 (16 to 62)	5 (4 to 6)
Belarus	2029	461 (379 to 556)	322 (252 to 412)	126 (102 to 158)	11 (5 to 20)	2 (1 to 2)	1450 (1191 to 1749)	1013 (791 to 1295)	397 (321 to 497)	35 (17 to 63)	5 (4 to 6)
Belarus	2030	470 (387 to 576)	329 (255 to 424)	128 (104 to 161)	11 (5 to 21)	2 (1 to 2)	1478 (1215 to 1813)	1034 (802 to 1334)	404 (326 to 505)	36 (17 to 65)	5 (4 to 6)
Belarus	2031	479 (390 to 587)	335 (255 to 433)	131 (105 to 163)	12 (5 to 21)	2 (1 to 2)	1507 (1226 to 1847)	1055 (801 to 1362)	410 (332 to 514)	36 (17 to 66)	5 (4 to 6)
Belarus	2032	488 (396 to 599)	342 (258 to 439)	133 (107 to 166)	12 (6 to 22)	2 (1 to 2)	1535 (1246 to 1884)	1076 (812 to 1381)	417 (337 to 522)	37 (18 to 68)	5 (5 to 6)
Belarus	2033	499 (397 to 615)	350 (262 to 453)	135 (109 to 169)	12 (6 to 22)	2 (1 to 2)	1568 (1249 to 1935)	1099 (825 to 1424)	425 (343 to 532)	38 (18 to 69)	5 (5 to 7)
Belarus	2034	509 (406 to 633)	357 (264 to 466)	138 (111 to 172)	12 (6 to 23)	2 (1 to 2)	1600 (1277 to 1989)	1123 (831 to 1465)	433 (349 to 542)	39 (18 to 71)	6 (5 to 7)
Belarus	2035	520 (412 to 654)	365 (267 to 481)	140 (113 to 176)	13 (6 to 23)	2 (2 to 2)	1636 (1297 to 2055)	1148 (841 to 1513)	442 (356 to 554)	40 (19 to 73)	6 (5 to 7)
Belarus	2036	532 (422 to 673)	374 (274 to 500)	143 (115 to 179)	13 (6 to 24)	2 (2 to 2)	1672 (1329 to 2117)	1174 (861 to 1573)	450 (362 to 564)	41 (19 to 75)	6 (5 to 7)
Belarus	2037	542 (426 to 688)	381 (277 to 508)	146 (117 to 183)	13 (6 to 24)	2 (2 to 2)	1704 (1341 to 2165)	1197 (872 to 1598)	458 (368 to 574)	42 (20 to 76)	6 (5 to 7)

Belarus	2038	553 (438 to 698)	389 (283 to 525)	148 (119 to 186)	14 (6 to 25)	2 (2 to 2)	1738 (1377 to 2195)	1223 (888 to 1651)	466 (374 to 584)	43 (20 to 78)	6 (5 to 8)
Belarus	2039	563 (439 to 724)	397 (284 to 540)	150 (121 to 189)	14 (7 to 25)	2 (2 to 3)	1772 (1380 to 2278)	1248 (893 to 1698)	473 (380 to 594)	44 (21 to 80)	6 (5 to 8)
Belarus	2040	574 (450 to 741)	404 (288 to 551)	153 (123 to 192)	14 (7 to 26)	2 (2 to 3)	1804 (1417 to 2329)	1272 (905 to 1733)	481 (385 to 604)	45 (21 to 82)	7 (5 to 8)
Belarus	2041	584 (450 to 761)	412 (292 to 564)	155 (124 to 195)	15 (7 to 27)	2 (2 to 3)	1836 (1415 to 2393)	1295 (919 to 1773)	488 (391 to 613)	46 (21 to 84)	7 (5 to 8)
Belarus	2042	594 (463 to 771)	420 (299 to 583)	157 (126 to 198)	15 (7 to 27)	2 (2 to 3)	1868 (1455 to 2425)	1320 (940 to 1833)	495 (396 to 622)	47 (22 to 86)	7 (6 to 9)
Belarus	2043	604 (464 to 796)	427 (302 to 592)	159 (128 to 201)	15 (7 to 28)	2 (2 to 3)	1901 (1459 to 2502)	1344 (951 to 1862)	501 (401 to 631)	48 (22 to 87)	7 (6 to 9)
Belarus	2044	614 (468 to 819)	434 (302 to 615)	162 (129 to 203)	16 (7 to 28)	2 (2 to 3)	1930 (1471 to 2575)	1365 (950 to 1934)	508 (406 to 639)	49 (23 to 89)	7 (6 to 10)
Belarus	2045	623 (474 to 832)	441 (305 to 626)	164 (131 to 206)	16 (7 to 29)	2 (2 to 3)	1959 (1491 to 2618)	1388 (959 to 1968)	514 (411 to 648)	50 (23 to 91)	7 (6 to 10)
Belarus	2046	632 (481 to 835)	448 (312 to 638)	165 (132 to 209)	16 (8 to 29)	2 (2 to 3)	1987 (1512 to 2627)	1408 (980 to 2006)	520 (414 to 656)	51 (24 to 93)	8 (6 to 10)
Belarus	2047	640 (487 to 852)	454 (314 to 652)	167 (133 to 211)	16 (8 to 30)	2 (2 to 3)	2012 (1530 to 2679)	1427 (986 to 2050)	526 (418 to 663)	52 (24 to 94)	8 (6 to 10)

Belarus	2048	648 (490 to 878)	460 (316 to 665)	169 (134 to 213)	17 (8 to 30)	3 (2 to 3)	2036 (1541 to 2761)	1445 (994 to 2090)	531 (420 to 670)	53 (24 to 96)	8 (6 to 11)
Belarus	2049	654 (493 to 880)	464 (313 to 681)	170 (134 to 215)	17 (8 to 31)	3 (2 to 3)	2057 (1550 to 2766)	1460 (983 to 2141)	535 (423 to 675)	53 (25 to 97)	8 (7 to 11)
Belarus	2050	660 (490 to 897)	469 (311 to 685)	171 (135 to 216)	17 (8 to 31)	3 (2 to 4)	2077 (1542 to 2822)	1475 (976 to 2152)	539 (425 to 680)	54 (25 to 98)	8 (7 to 11)
Belgium	1995	2512 (2421 to 2610)	1936 (1853 to 2023)	497 (457 to 538)	79 (59 to 103)	0 (0 to 0)	2731 (2632 to 2838)	2105 (2015 to 2199)	541 (497 to 584)	86 (64 to 112)	0 (0 to 0)
Belgium	1996	2596 (2526 to 2670)	1991 (1930 to 2051)	520 (490 to 551)	85 (67 to 105)	0 (0 to 0)	2822 (2746 to 2903)	2164 (2098 to 2230)	565 (532 to 599)	93 (73 to 115)	0 (0 to 0)
Belgium	1997	2686 (2614 to 2756)	2034 (1974 to 2094)	555 (525 to 585)	97 (80 to 117)	0 (0 to 0)	2920 (2842 to 2996)	2211 (2146 to 2276)	603 (571 to 636)	105 (87 to 127)	0 (0 to 0)
Belgium	1998	2771 (2700 to 2842)	2079 (2020 to 2143)	581 (551 to 612)	111 (93 to 132)	0 (0 to 0)	3012 (2936 to 3089)	2261 (2196 to 2330)	631 (599 to 665)	120 (101 to 143)	0 (0 to 0)
Belgium	1999	2896 (2830 to 2966)	2165 (2107 to 2223)	604 (576 to 635)	127 (109 to 148)	0 (0 to 0)	3149 (3077 to 3225)	2354 (2290 to 2417)	657 (627 to 690)	138 (118 to 161)	0 (0 to 0)
Belgium	2000	3037 (2970 to 3105)	2271 (2215 to 2330)	624 (596 to 651)	142 (124 to 163)	0 (0 to 0)	3302 (3228 to 3376)	2469 (2408 to 2534)	679 (648 to 708)	154 (135 to 177)	0 (0 to 0)
Belgium	2001	3134 (3067 to 3202)	2342 (2284 to 2403)	641 (614 to 667)	151 (133 to 170)	0 (0 to 0)	3407 (3334 to 3481)	2546 (2483 to 2612)	696 (667 to 725)	165 (145 to 185)	0 (0 to 0)

Belgium	2002	3302 (3236 to 3366)	2465 (2407 to 2522)	673 (648 to 699)	163 (145 to 182)	0 (0 to 0)	3590 (3518 to 3660)	2680 (2616 to 2742)	732 (704 to 760)	177 (158 to 198)	0 (0 to 0)
Belgium	2003	3517 (3451 to 3585)	2639 (2583 to 2700)	700 (673 to 727)	178 (160 to 198)	0 (0 to 0)	3824 (3752 to 3897)	2869 (2808 to 2935)	761 (731 to 790)	194 (174 to 215)	0 (0 to 0)
Belgium	2004	3612 (3546 to 3682)	2733 (2673 to 2795)	693 (665 to 720)	186 (167 to 206)	0 (0 to 0)	3927 (3855 to 4003)	2972 (2906 to 3038)	753 (723 to 783)	203 (182 to 224)	0 (0 to 0)
Belgium	2005	3793 (3722 to 3868)	2896 (2835 to 2958)	699 (672 to 728)	199 (179 to 219)	0 (0 to 0)	4124 (4046 to 4205)	3148 (3082 to 3215)	760 (730 to 791)	216 (194 to 238)	0 (0 to 0)
Belgium	2006	3734 (3660 to 3803)	2842 (2784 to 2901)	699 (671 to 727)	193 (175 to 213)	0 (0 to 0)	4059 (3979 to 4135)	3090 (3027 to 3154)	760 (730 to 790)	210 (191 to 232)	0 (0 to 0)
Belgium	2007	3842 (3769 to 3910)	2929 (2865 to 2989)	722 (695 to 751)	191 (172 to 210)	0 (0 to 0)	4177 (4097 to 4251)	3185 (3115 to 3250)	785 (755 to 816)	207 (187 to 229)	0 (0 to 0)
Belgium	2008	4023 (3952 to 4096)	3094 (3034 to 3162)	746 (719 to 774)	183 (165 to 203)	0 (0 to 0)	4374 (4297 to 4453)	3364 (3298 to 3438)	811 (782 to 842)	199 (179 to 220)	0 (0 to 0)
Belgium	2009	4217 (4146 to 4285)	3269 (3207 to 3331)	769 (744 to 797)	179 (161 to 199)	0 (0 to 0)	4584 (4508 to 4659)	3554 (3487 to 3622)	836 (809 to 867)	194 (175 to 216)	0 (0 to 0)
Belgium	2010	4276 (4200 to 4351)	3322 (3257 to 3381)	778 (750 to 807)	176 (158 to 195)	0 (0 to 0)	4648 (4566 to 4730)	3612 (3541 to 3676)	845 (815 to 877)	191 (172 to 212)	0 (0 to 0)
Belgium	2011	4341 (4270 to 4413)	3376 (3314 to 3438)	786 (757 to 816)	179 (160 to 198)	0 (0 to 0)	4719 (4642 to 4798)	3671 (3602 to 3738)	855 (823 to 887)	194 (174 to 215)	0 (0 to 0)

Belgium	2012	4421 (4349 to 4498)	3445 (3382 to 3509)	791 (761 to 821)	185 (166 to 205)	0 (0 to 0)	4807 (4728 to 4890)	3746 (3677 to 3815)	860 (828 to 893)	201 (180 to 223)	0 (0 to 0)
Belgium	2013	4435 (4361 to 4508)	3453 (3387 to 3521)	791 (762 to 820)	191 (172 to 210)	0 (0 to 0)	4822 (4741 to 4901)	3754 (3683 to 3828)	860 (828 to 892)	208 (187 to 228)	0 (0 to 0)
Belgium	2014	4460 (4389 to 4531)	3471 (3409 to 3535)	788 (760 to 819)	201 (182 to 220)	0 (0 to 0)	4849 (4772 to 4926)	3773 (3706 to 3844)	857 (826 to 890)	218 (198 to 239)	0 (0 to 0)
Belgium	2015	4477 (4404 to 4551)	3473 (3408 to 3535)	791 (758 to 822)	213 (194 to 235)	0 (0 to 0)	4867 (4788 to 4947)	3775 (3705 to 3843)	860 (825 to 893)	232 (211 to 255)	0 (0 to 0)
Belgium	2016	4531 (4456 to 4609)	3503 (3437 to 3565)	803 (773 to 832)	225 (203 to 248)	0 (0 to 0)	4926 (4845 to 5011)	3808 (3737 to 3876)	873 (840 to 905)	245 (221 to 270)	0 (0 to 0)
Belgium	2017	4595 (4475 to 4727)	3547 (3454 to 3639)	813 (772 to 858)	235 (206 to 266)	0 (0 to 0)	4995 (4865 to 5139)	3856 (3755 to 3957)	884 (840 to 932)	256 (224 to 289)	0 (0 to 0)
Belgium	2018	4695 (4564 to 4827)	3635 (3539 to 3735)	821 (780 to 867)	239 (210 to 270)	0 (0 to 0)	5104 (4962 to 5248)	3951 (3847 to 4061)	892 (848 to 942)	260 (228 to 293)	0 (0 to 0)
Belgium	2019	4747 (4611 to 4893)	3677 (3570 to 3788)	827 (785 to 873)	243 (213 to 274)	0 (0 to 0)	5161 (5013 to 5319)	3998 (3882 to 4118)	899 (854 to 949)	264 (231 to 298)	0 (0 to 0)
Belgium	2020	4828 (4688 to 4978)	3747 (3638 to 3864)	834 (792 to 880)	247 (216 to 279)	0 (0 to 0)	5249 (5097 to 5412)	4074 (3955 to 4200)	907 (861 to 957)	268 (235 to 304)	0 (0 to 0)
Belgium	2021	4896 (4747 to 5051)	3808 (3694 to 3935)	838 (795 to 884)	250 (218 to 283)	0 (0 to 0)	5323 (5161 to 5492)	4140 (4016 to 4279)	911 (865 to 962)	271 (237 to 307)	0 (0 to 0)

Belgium	2022	4968 (4816 to 5130)	3874 (3750 to 4002)	842 (798 to 888)	253 (221 to 286)	0 (0 to 0)	5401 (5236 to 5578)	4212 (4077 to 4351)	915 (868 to 966)	275 (241 to 311)	0 (0 to 0)
Belgium	2023	5031 (4860 to 5203)	3933 (3802 to 4071)	843 (800 to 891)	255 (223 to 289)	0 (0 to 0)	5469 (5284 to 5657)	4275 (4133 to 4426)	917 (870 to 969)	277 (243 to 314)	0 (0 to 0)
Belgium	2024	5084 (4898 to 5273)	3985 (3837 to 4133)	843 (799 to 891)	257 (224 to 291)	0 (0 to 0)	5527 (5325 to 5733)	4332 (4171 to 4494)	916 (869 to 969)	279 (244 to 316)	0 (0 to 0)
Belgium	2025	5132 (4943 to 5338)	4031 (3876 to 4198)	842 (797 to 889)	259 (226 to 293)	0 (0 to 0)	5579 (5374 to 5803)	4383 (4214 to 4564)	915 (867 to 966)	281 (246 to 318)	0 (0 to 0)
Belgium	2026	5179 (4973 to 5399)	4078 (3909 to 4256)	841 (796 to 887)	261 (228 to 295)	0 (0 to 0)	5631 (5406 to 5870)	4433 (4250 to 4627)	914 (866 to 965)	284 (248 to 321)	0 (0 to 0)
Belgium	2027	5227 (5011 to 5454)	4124 (3942 to 4319)	840 (795 to 886)	263 (231 to 298)	0 (0 to 0)	5683 (5448 to 5929)	4484 (4285 to 4696)	913 (865 to 963)	286 (251 to 324)	0 (0 to 0)
Belgium	2028	5277 (5034 to 5531)	4173 (3980 to 4385)	839 (795 to 885)	266 (232 to 302)	0 (0 to 0)	5737 (5473 to 6014)	4537 (4327 to 4767)	912 (864 to 962)	289 (253 to 328)	0 (0 to 0)
Belgium	2029	5331 (5084 to 5603)	4224 (4008 to 4454)	838 (794 to 885)	268 (234 to 304)	0 (0 to 0)	5796 (5527 to 6091)	4593 (4357 to 4842)	912 (863 to 962)	291 (254 to 331)	0 (0 to 0)
Belgium	2030	5387 (5107 to 5686)	4278 (4047 to 4541)	839 (794 to 885)	271 (236 to 307)	0 (0 to 0)	5857 (5552 to 6182)	4651 (4400 to 4937)	912 (863 to 962)	294 (257 to 334)	0 (0 to 0)
Belgium	2031	5445 (5156 to 5768)	4332 (4074 to 4620)	839 (793 to 886)	273 (238 to 310)	0 (0 to 0)	5920 (5605 to 6271)	4710 (4429 to 5023)	913 (863 to 964)	297 (258 to 337)	0 (0 to 0)

Belgium	2032	5505 (5176 to 5856)	4388 (4103 to 4693)	841 (794 to 889)	276 (239 to 314)	0 (0 to 0)	5985 (5627 to 6366)	4770 (4461 to 5102)	914 (863 to 967)	300 (260 to 342)	0 (0 to 0)
Belgium	2033	5563 (5220 to 5926)	4442 (4132 to 4767)	842 (794 to 891)	279 (242 to 319)	0 (0 to 0)	6048 (5676 to 6443)	4829 (4492 to 5183)	916 (863 to 969)	303 (263 to 347)	0 (0 to 0)
Belgium	2034	5623 (5253 to 6013)	4496 (4172 to 4846)	844 (795 to 894)	282 (244 to 323)	0 (0 to 0)	6113 (5711 to 6538)	4888 (4536 to 5268)	918 (864 to 972)	307 (265 to 351)	0 (0 to 0)
Belgium	2035	5682 (5296 to 6089)	4551 (4205 to 4911)	846 (796 to 898)	285 (246 to 327)	0 (0 to 0)	6178 (5758 to 6620)	4948 (4571 to 5339)	920 (865 to 976)	310 (268 to 355)	0 (0 to 0)
Belgium	2036	5740 (5337 to 6180)	4603 (4240 to 4994)	849 (797 to 903)	288 (249 to 331)	0 (0 to 0)	6240 (5803 to 6718)	5005 (4610 to 5429)	923 (867 to 981)	313 (270 to 360)	0 (0 to 0)
Belgium	2037	5795 (5374 to 6236)	4653 (4272 to 5063)	851 (798 to 906)	291 (251 to 335)	0 (0 to 0)	6300 (5842 to 6780)	5059 (4645 to 5504)	925 (868 to 985)	316 (273 to 365)	0 (0 to 0)
Belgium	2038	5851 (5406 to 6324)	4704 (4307 to 5128)	853 (800 to 909)	294 (253 to 339)	0 (0 to 0)	6361 (5877 to 6875)	5114 (4682 to 5575)	928 (870 to 988)	319 (275 to 368)	0 (0 to 0)
Belgium	2039	5911 (5463 to 6370)	4757 (4347 to 5186)	857 (802 to 912)	297 (255 to 342)	0 (0 to 0)	6426 (5940 to 6925)	5172 (4726 to 5638)	932 (872 to 992)	323 (278 to 372)	0 (0 to 0)
Belgium	2040	5972 (5501 to 6465)	4811 (4380 to 5258)	861 (805 to 917)	300 (258 to 346)	0 (0 to 0)	6493 (5980 to 7029)	5230 (4762 to 5717)	936 (876 to 997)	327 (280 to 376)	0 (0 to 0)
Belgium	2041	6033 (5538 to 6543)	4864 (4420 to 5327)	865 (808 to 923)	304 (260 to 351)	0 (0 to 0)	6559 (6021 to 7114)	5288 (4805 to 5791)	941 (879 to 1003)	330 (283 to 381)	0 (0 to 0)

Belgium	2042	6093 (5613 to 6597)	4917 (4465 to 5386)	869 (811 to 929)	307 (263 to 355)	0 (0 to 0)	6624 (6102 to 7172)	5345 (4854 to 5856)	945 (882 to 1010)	334 (286 to 386)	0 (0 to 0)
Belgium	2043	6151 (5640 to 6680)	4967 (4506 to 5433)	873 (813 to 933)	310 (265 to 360)	0 (0 to 0)	6687 (6131 to 7263)	5400 (4899 to 5907)	950 (884 to 1015)	337 (288 to 391)	0 (0 to 0)
Belgium	2044	6209 (5681 to 6727)	5018 (4547 to 5488)	878 (816 to 941)	314 (267 to 364)	0 (0 to 0)	6751 (6176 to 7313)	5456 (4943 to 5967)	954 (887 to 1023)	341 (291 to 396)	0 (0 to 0)
Belgium	2045	6266 (5751 to 6786)	5067 (4581 to 5545)	882 (818 to 947)	317 (270 to 368)	0 (0 to 0)	6812 (6253 to 7378)	5509 (4980 to 6029)	959 (889 to 1029)	344 (293 to 401)	0 (0 to 0)
Belgium	2046	6323 (5764 to 6878)	5116 (4608 to 5612)	887 (822 to 953)	320 (272 to 372)	0 (0 to 0)	6874 (6267 to 7478)	5562 (5010 to 6102)	964 (894 to 1036)	348 (295 to 405)	0 (0 to 0)
Belgium	2047	6378 (5844 to 6933)	5164 (4657 to 5673)	891 (825 to 960)	323 (274 to 377)	0 (0 to 0)	6934 (6354 to 7537)	5614 (5063 to 6168)	969 (897 to 1044)	352 (298 to 410)	0 (0 to 0)
Belgium	2048	6432 (5867 to 7001)	5210 (4692 to 5718)	896 (828 to 967)	327 (276 to 382)	0 (0 to 0)	6993 (6379 to 7611)	5664 (5102 to 6217)	974 (900 to 1052)	355 (300 to 415)	0 (0 to 0)
Belgium	2049	6485 (5913 to 7059)	5254 (4730 to 5778)	900 (830 to 974)	330 (279 to 387)	0 (0 to 0)	7050 (6428 to 7674)	5712 (5142 to 6282)	979 (902 to 1059)	359 (303 to 421)	0 (0 to 0)
Belgium	2050	6537 (5974 to 7116)	5299 (4761 to 5843)	905 (836 to 981)	333 (281 to 391)	0 (0 to 0)	7107 (6494 to 7737)	5761 (5176 to 6353)	984 (909 to 1067)	362 (305 to 425)	0 (0 to 0)
Belize	1995	180 (154 to 207)	105 (88 to 124)	53 (39 to 70)	20 (11 to 35)	2 (2 to 2)	316 (271 to 364)	185 (155 to 219)	93 (69 to 123)	35 (19 to 61)	4 (4 to 4)
Belize	1996	171 (148 to 194)	97 (83 to 114)	52 (39 to 68)	19 (10 to 33)	2 (2 to 2)	300 (260 to 341)	171 (145 to 200)	91 (69 to 120)	34 (18 to 59)	4 (4 to 4)

Belize	1997	170 (148 to 193)	95 (81 to 111)	52 (41 to 68)	19 (10 to 32)	3 (3 to 3)	299 (261 to 339)	168 (143 to 196)	92 (72 to 120)	33 (18 to 56)	6 (6 to 6)
Belize	1998	171 (149 to 193)	95 (82 to 110)	54 (42 to 69)	19 (10 to 31)	2 (2 to 2)	300 (262 to 339)	168 (145 to 193)	95 (74 to 122)	33 (18 to 55)	4 (4 to 4)
Belize	1999	182 (160 to 207)	100 (87 to 115)	59 (46 to 74)	19 (10 to 31)	5 (5 to 5)	321 (282 to 364)	176 (153 to 202)	103 (81 to 130)	33 (18 to 55)	8 (8 to 8)
Belize	2000	200 (177 to 223)	105 (91 to 120)	64 (51 to 81)	19 (10 to 31)	11 (11 to 11)	351 (311 to 393)	185 (160 to 211)	113 (90 to 142)	33 (18 to 55)	20 (20 to 20)
Belize	2001	219 (195 to 245)	117 (102 to 132)	71 (56 to 87)	20 (11 to 33)	11 (11 to 11)	385 (344 to 430)	206 (180 to 233)	125 (99 to 153)	35 (19 to 58)	20 (20 to 20)
Belize	2002	219 (196 to 243)	118 (104 to 134)	73 (58 to 88)	19 (10 to 31)	9 (9 to 9)	385 (345 to 428)	208 (184 to 236)	128 (101 to 156)	33 (18 to 55)	15 (15 to 15)
Belize	2003	236 (212 to 262)	132 (117 to 149)	76 (61 to 92)	20 (11 to 32)	8 (8 to 8)	415 (372 to 461)	232 (205 to 262)	134 (108 to 162)	35 (19 to 56)	15 (15 to 15)
Belize	2004	237 (213 to 262)	134 (118 to 151)	75 (59 to 91)	19 (10 to 31)	8 (8 to 8)	417 (375 to 461)	237 (208 to 266)	131 (104 to 160)	34 (18 to 55)	15 (15 to 15)
Belize	2005	236 (212 to 258)	138 (122 to 154)	72 (57 to 88)	18 (10 to 30)	8 (8 to 8)	414 (373 to 455)	242 (214 to 271)	126 (100 to 154)	32 (17 to 53)	14 (14 to 14)
Belize	2006	240 (216 to 263)	144 (127 to 161)	69 (55 to 85)	17 (9 to 28)	10 (10 to 10)	422 (381 to 462)	253 (224 to 283)	122 (96 to 150)	31 (16 to 50)	17 (17 to 17)
Belize	2007	267 (239 to 294)	169 (149 to 189)	71 (57 to 88)	19 (10 to 30)	8 (8 to 8)	470 (421 to 517)	298 (263 to 333)	125 (100 to 155)	33 (18 to 53)	13 (13 to 13)
Belize	2008	260 (233 to 285)	168 (148 to 187)	68 (55 to 85)	17 (10 to 29)	7 (7 to 7)	458 (410 to 502)	295 (261 to 329)	120 (96 to 149)	31 (17 to 50)	12 (12 to 12)
Belize	2009	274 (247 to 303)	180 (160 to 202)	69 (56 to 86)	18 (10 to 29)	7 (7 to 7)	482 (434 to 533)	317 (281 to 355)	122 (99 to 152)	31 (18 to 51)	12 (12 to 12)
Belize	2010	282 (255 to 313)	185 (165 to 206)	69 (56 to 86)	17 (10 to 30)	11 (11 to 11)	497 (449 to 550)	325 (290 to 363)	122 (99 to 152)	31 (17 to 53)	19 (19 to 19)
Belize	2011	274 (247 to 302)	182 (162 to 203)	69 (56 to 85)	17 (10 to 29)	6 (6 to 6)	481 (435 to 532)	320 (285 to 358)	121 (98 to 150)	30 (17 to 52)	11 (11 to 11)

Belize	2012	270 (244 to 298)	177 (158 to 198)	68 (55 to 84)	17 (9 to 28)	9 (9 to 9)	475 (430 to 525)	311 (277 to 349)	119 (97 to 148)	29 (16 to 50)	15 (15 to 15)
Belize	2013	297 (272 to 324)	178 (159 to 199)	68 (54 to 85)	17 (9 to 28)	35 (35 to 35)	523 (478 to 571)	313 (279 to 350)	119 (95 to 149)	29 (17 to 50)	61 (61 to 61)
Belize	2014	274 (248 to 303)	181 (161 to 202)	68 (54 to 86)	17 (9 to 30)	7 (7 to 7)	482 (437 to 533)	319 (284 to 356)	120 (95 to 151)	30 (16 to 53)	13 (13 to 13)
Belize	2015	300 (273 to 332)	197 (176 to 221)	70 (55 to 89)	18 (10 to 32)	14 (14 to 14)	528 (480 to 585)	347 (309 to 390)	124 (98 to 157)	32 (17 to 56)	25 (25 to 25)
Belize	2016	292 (263 to 325)	195 (172 to 222)	70 (54 to 87)	18 (10 to 31)	10 (10 to 10)	513 (462 to 572)	343 (303 to 391)	122 (95 to 153)	31 (17 to 55)	17 (17 to 17)
Belize	2017	287 (247 to 337)	194 (168 to 225)	69 (52 to 88)	18 (10 to 30)	7 (7 to 7)	505 (435 to 593)	341 (295 to 397)	121 (92 to 155)	31 (17 to 53)	12 (12 to 12)
Belize	2018	271 (234 to 314)	178 (154 to 206)	69 (53 to 89)	18 (10 to 31)	6 (6 to 6)	477 (412 to 552)	312 (270 to 363)	122 (93 to 156)	32 (17 to 54)	11 (11 to 11)
Belize	2019	274 (236 to 322)	180 (157 to 209)	69 (53 to 89)	18 (10 to 31)	6 (6 to 6)	482 (416 to 566)	316 (275 to 368)	122 (93 to 156)	32 (17 to 55)	11 (11 to 11)
Belize	2020	275 (237 to 320)	181 (158 to 211)	69 (53 to 88)	18 (10 to 31)	7 (6 to 7)	484 (417 to 562)	319 (277 to 371)	122 (93 to 156)	32 (17 to 55)	12 (11 to 12)
Belize	2021	282 (243 to 331)	187 (162 to 217)	70 (54 to 90)	19 (10 to 32)	7 (6 to 7)	497 (428 to 582)	328 (285 to 381)	124 (94 to 158)	33 (18 to 56)	12 (11 to 13)
Belize	2022	290 (250 to 336)	193 (167 to 224)	72 (55 to 92)	19 (10 to 33)	7 (6 to 7)	510 (439 to 590)	339 (295 to 393)	126 (96 to 161)	34 (18 to 58)	12 (11 to 13)
Belize	2023	298 (257 to 349)	199 (173 to 231)	73 (56 to 93)	20 (11 to 34)	7 (6 to 8)	525 (452 to 614)	350 (304 to 407)	128 (98 to 164)	35 (19 to 59)	12 (11 to 13)
Belize	2024	306 (264 to 354)	205 (178 to 238)	74 (57 to 95)	20 (11 to 35)	7 (6 to 8)	538 (464 to 622)	361 (314 to 419)	130 (100 to 167)	36 (19 to 61)	12 (11 to 13)
Belize	2025	313 (269 to 366)	210 (183 to 244)	75 (58 to 96)	21 (11 to 35)	7 (6 to 8)	550 (473 to 644)	369 (321 to 429)	132 (101 to 169)	36 (19 to 62)	12 (10 to 13)
Belize	2026	319 (276 to 371)	215 (186 to 249)	76 (58 to 98)	21 (11 to 36)	7 (6 to 8)	562 (486 to 653)	378 (328 to 438)	134 (103 to 172)	37 (20 to 64)	12 (10 to 14)

Belize	2027	326 (280 to 381)	220 (191 to 255)	77 (59 to 99)	22 (11 to 37)	7 (6 to 8)	573 (492 to 671)	387 (335 to 449)	136 (104 to 174)	38 (20 to 65)	12 (10 to 14)
Belize	2028	332 (287 to 387)	225 (194 to 263)	78 (60 to 100)	22 (12 to 38)	7 (6 to 8)	584 (505 to 682)	395 (341 to 462)	138 (105 to 176)	39 (20 to 66)	12 (10 to 14)
Belize	2029	338 (290 to 398)	229 (198 to 268)	79 (60 to 101)	23 (12 to 38)	7 (6 to 8)	595 (510 to 700)	404 (348 to 472)	140 (106 to 178)	40 (21 to 67)	12 (10 to 14)
Belize	2030	344 (297 to 401)	234 (201 to 274)	80 (61 to 102)	23 (12 to 39)	7 (6 to 8)	605 (522 to 706)	412 (354 to 482)	141 (107 to 180)	40 (21 to 68)	12 (10 to 15)
Belize	2031	350 (299 to 415)	239 (205 to 280)	81 (61 to 103)	23 (12 to 39)	7 (6 to 8)	616 (526 to 731)	420 (360 to 493)	142 (108 to 182)	41 (22 to 69)	12 (11 to 15)
Belize	2032	356 (307 to 416)	243 (208 to 289)	82 (62 to 104)	24 (13 to 40)	7 (6 to 8)	626 (541 to 732)	428 (367 to 508)	144 (109 to 183)	42 (22 to 71)	12 (11 to 15)
Belize	2033	362 (306 to 430)	248 (211 to 295)	82 (62 to 106)	24 (13 to 41)	7 (6 to 9)	636 (539 to 756)	436 (372 to 518)	145 (110 to 186)	42 (22 to 72)	13 (11 to 15)
Belize	2034	367 (315 to 432)	252 (215 to 298)	83 (63 to 106)	25 (13 to 42)	7 (6 to 9)	646 (555 to 760)	444 (378 to 525)	146 (111 to 187)	43 (23 to 73)	13 (11 to 15)
Belize	2035	373 (315 to 442)	257 (218 to 305)	84 (63 to 107)	25 (13 to 42)	7 (6 to 9)	655 (554 to 778)	451 (383 to 536)	147 (111 to 188)	44 (23 to 74)	13 (11 to 16)
Belize	2036	378 (324 to 446)	261 (221 to 309)	84 (64 to 108)	25 (13 to 43)	7 (6 to 9)	665 (571 to 784)	459 (388 to 544)	149 (112 to 190)	45 (23 to 75)	13 (11 to 16)
Belize	2037	384 (322 to 459)	266 (224 to 315)	85 (64 to 109)	26 (14 to 44)	7 (6 to 9)	675 (567 to 807)	467 (394 to 555)	150 (113 to 192)	45 (24 to 77)	13 (11 to 16)
Belize	2038	390 (333 to 464)	270 (227 to 329)	86 (65 to 110)	26 (14 to 44)	8 (6 to 9)	686 (585 to 816)	475 (399 to 579)	151 (114 to 194)	46 (24 to 78)	13 (11 to 17)
Belize	2039	396 (331 to 478)	275 (231 to 334)	87 (65 to 111)	27 (14 to 45)	8 (6 to 10)	696 (581 to 841)	484 (406 to 588)	152 (115 to 195)	47 (24 to 79)	13 (11 to 17)
Belize	2040	402 (341 to 481)	280 (234 to 342)	87 (66 to 112)	27 (14 to 46)	8 (6 to 10)	706 (600 to 847)	492 (411 to 601)	154 (116 to 197)	48 (25 to 80)	14 (11 to 17)
Belize	2041	408 (340 to 499)	284 (237 to 351)	88 (66 to 113)	27 (14 to 46)	8 (6 to 10)	717 (599 to 878)	500 (417 to 617)	155 (116 to 199)	48 (25 to 82)	14 (11 to 18)

Belize	2042	414 (351 to 499)	289 (241 to 358)	89 (67 to 114)	28 (14 to 47)	8 (6 to 10)	728 (618 to 877)	508 (423 to 629)	156 (117 to 201)	49 (25 to 83)	14 (11 to 18)
Belize	2043	420 (350 to 518)	294 (244 to 368)	90 (67 to 115)	28 (15 to 48)	8 (6 to 11)	739 (616 to 911)	517 (430 to 648)	158 (118 to 203)	50 (26 to 85)	14 (11 to 18)
Belize	2044	427 (360 to 520)	299 (248 to 376)	90 (67 to 116)	29 (15 to 49)	8 (7 to 11)	751 (633 to 915)	526 (436 to 662)	159 (119 to 205)	51 (26 to 86)	14 (12 to 19)
Belize	2045	433 (360 to 538)	304 (251 to 383)	91 (68 to 117)	29 (15 to 50)	8 (7 to 11)	761 (633 to 946)	535 (442 to 674)	160 (119 to 206)	52 (27 to 88)	15 (12 to 19)
Belize	2046	439 (371 to 535)	309 (256 to 389)	92 (68 to 118)	30 (15 to 51)	8 (7 to 11)	772 (653 to 941)	543 (450 to 685)	162 (120 to 208)	53 (27 to 89)	15 (12 to 19)
Belize	2047	445 (370 to 553)	314 (260 to 394)	93 (69 to 119)	30 (16 to 52)	9 (7 to 11)	783 (651 to 972)	552 (457 to 693)	163 (121 to 210)	53 (28 to 91)	15 (12 to 20)
Belize	2048	452 (380 to 558)	319 (263 to 405)	93 (69 to 120)	31 (16 to 52)	9 (7 to 12)	795 (668 to 981)	561 (462 to 713)	164 (122 to 212)	54 (28 to 92)	15 (12 to 20)
Belize	2049	458 (379 to 575)	324 (267 to 412)	94 (70 to 121)	31 (16 to 53)	9 (7 to 12)	806 (667 to 1011)	570 (469 to 724)	166 (123 to 213)	55 (28 to 94)	16 (12 to 21)
Belize	2050	464 (390 to 575)	329 (271 to 420)	95 (70 to 122)	32 (16 to 54)	9 (7 to 12)	817 (686 to 1011)	578 (477 to 738)	167 (124 to 215)	56 (29 to 96)	16 (13 to 21)
Benin	1995	27 (22 to 33)	7 (5 to 9)	15 (11 to 22)	2 (1 to 3)	3 (3 to 3)	74 (61 to 93)	20 (15 to 26)	43 (30 to 61)	5 (2 to 8)	7 (7 to 7)
Benin	1996	27 (22 to 33)	7 (5 to 9)	15 (11 to 21)	2 (1 to 3)	4 (4 to 4)	76 (62 to 93)	20 (15 to 25)	42 (30 to 59)	5 (2 to 8)	10 (10 to 10)
Benin	1997	26 (21 to 32)	7 (5 to 9)	15 (11 to 21)	2 (1 to 3)	2 (2 to 2)	74 (59 to 91)	20 (15 to 26)	42 (30 to 59)	5 (2 to 8)	7 (7 to 7)
Benin	1998	29 (24 to 35)	7 (5 to 9)	15 (11 to 21)	2 (1 to 3)	6 (6 to 6)	82 (67 to 98)	19 (15 to 25)	42 (30 to 57)	4 (2 to 8)	16 (16 to 16)
Benin	1999	26 (21 to 32)	7 (5 to 9)	15 (10 to 20)	1 (1 to 3)	3 (3 to 3)	72 (59 to 89)	18 (14 to 24)	41 (29 to 56)	4 (2 to 7)	9 (9 to 9)
Benin	2000	29 (24 to 36)	8 (6 to 10)	16 (11 to 22)	2 (1 to 3)	4 (4 to 4)	82 (67 to 100)	22 (16 to 28)	44 (32 to 61)	5 (2 to 8)	12 (12 to 12)

Benin	2001	29 (24 to 36)	8 (6 to 10)	16 (11 to 22)	2 (1 to 3)	4 (4 to 4)	81 (67 to 99)	22 (16 to 28)	44 (32 to 61)	4 (2 to 8)	11 (11 to 11)
Benin	2002	29 (24 to 35)	8 (6 to 10)	16 (11 to 22)	2 (1 to 3)	4 (4 to 4)	80 (66 to 99)	21 (16 to 28)	44 (31 to 62)	5 (2 to 8)	11 (11 to 11)
Benin	2003	29 (25 to 36)	7 (5 to 9)	15 (11 to 21)	2 (1 to 3)	6 (6 to 6)	82 (69 to 100)	19 (15 to 25)	43 (30 to 60)	4 (2 to 8)	16 (16 to 16)
Benin	2004	31 (26 to 37)	7 (5 to 9)	15 (11 to 21)	2 (1 to 3)	8 (8 to 8)	87 (74 to 104)	19 (14 to 25)	42 (30 to 59)	4 (2 to 8)	22 (22 to 22)
Benin	2005	31 (27 to 37)	7 (5 to 9)	15 (10 to 20)	2 (1 to 3)	8 (8 to 8)	88 (75 to 105)	19 (15 to 25)	41 (29 to 57)	4 (2 to 8)	23 (23 to 23)
Benin	2006	29 (25 to 35)	6 (5 to 8)	14 (10 to 19)	2 (1 to 3)	7 (7 to 7)	82 (70 to 98)	18 (14 to 23)	39 (28 to 55)	4 (2 to 8)	21 (21 to 21)
Benin	2007	30 (25 to 36)	7 (5 to 9)	14 (10 to 20)	2 (1 to 3)	7 (7 to 7)	84 (71 to 100)	19 (15 to 25)	40 (29 to 55)	5 (2 to 9)	20 (20 to 20)
Benin	2008	30 (26 to 36)	7 (5 to 9)	13 (10 to 19)	2 (1 to 3)	9 (9 to 9)	85 (72 to 100)	18 (14 to 24)	38 (27 to 52)	5 (2 to 8)	24 (24 to 24)
Benin	2009	35 (30 to 41)	8 (6 to 10)	14 (10 to 19)	2 (1 to 3)	11 (11 to 11)	97 (84 to 114)	21 (16 to 28)	39 (28 to 54)	5 (2 to 9)	32 (32 to 32)
Benin	2010	31 (27 to 37)	7 (5 to 9)	13 (9 to 18)	2 (1 to 3)	10 (10 to 10)	87 (75 to 104)	19 (14 to 25)	37 (26 to 51)	4 (2 to 8)	27 (27 to 27)
Benin	2011	33 (29 to 39)	7 (5 to 9)	13 (9 to 18)	2 (1 to 3)	11 (11 to 11)	93 (80 to 109)	20 (15 to 26)	37 (26 to 50)	4 (2 to 8)	32 (32 to 32)
Benin	2012	29 (25 to 35)	7 (5 to 9)	13 (9 to 17)	2 (1 to 3)	8 (8 to 8)	81 (69 to 98)	20 (15 to 26)	36 (25 to 49)	4 (2 to 8)	22 (22 to 22)
Benin	2013	28 (24 to 35)	7 (5 to 9)	13 (9 to 18)	2 (1 to 3)	7 (7 to 7)	80 (67 to 97)	20 (15 to 26)	36 (25 to 49)	5 (2 to 9)	19 (19 to 19)
Benin	2014	31 (26 to 36)	7 (5 to 9)	12 (9 to 18)	2 (1 to 3)	10 (10 to 10)	86 (73 to 102)	19 (14 to 25)	35 (25 to 49)	5 (2 to 9)	28 (28 to 28)
Benin	2015	30 (25 to 36)	7 (5 to 10)	13 (9 to 18)	2 (1 to 3)	8 (8 to 8)	83 (70 to 99)	20 (15 to 27)	36 (26 to 51)	5 (3 to 10)	21 (21 to 21)

Benin	2016	29 (25 to 35)	7 (5 to 9)	12 (9 to 18)	2 (1 to 3)	8 (8 to 8)	82 (69 to 98)	18 (14 to 25)	35 (24 to 49)	5 (2 to 9)	24 (24 to 24)
Benin	2017	35 (29 to 41)	8 (6 to 10)	13 (9 to 19)	2 (1 to 3)	12 (12 to 12)	97 (81 to 116)	22 (16 to 29)	37 (26 to 53)	5 (2 to 10)	33 (33 to 33)
Benin	2018	34 (28 to 41)	8 (6 to 10)	14 (10 to 20)	2 (1 to 3)	11 (11 to 11)	95 (80 to 114)	21 (16 to 29)	39 (27 to 55)	5 (2 to 10)	29 (29 to 30)
Benin	2019	34 (29 to 42)	8 (6 to 11)	14 (10 to 20)	2 (1 to 4)	10 (10 to 10)	96 (81 to 116)	22 (17 to 30)	40 (28 to 56)	5 (3 to 10)	29 (29 to 29)
Benin	2020	35 (29 to 43)	8 (6 to 11)	15 (10 to 21)	2 (1 to 4)	10 (10 to 11)	99 (82 to 119)	23 (17 to 31)	41 (28 to 58)	6 (3 to 11)	29 (27 to 30)
Benin	2021	36 (30 to 43)	8 (6 to 11)	15 (10 to 21)	2 (1 to 4)	10 (10 to 11)	100 (83 to 121)	24 (18 to 32)	42 (29 to 59)	6 (3 to 11)	29 (27 to 31)
Benin	2022	36 (30 to 44)	9 (7 to 12)	15 (10 to 22)	2 (1 to 4)	10 (9 to 11)	101 (84 to 124)	24 (18 to 33)	42 (29 to 60)	6 (3 to 11)	29 (26 to 32)
Benin	2023	37 (30 to 45)	9 (7 to 12)	15 (11 to 22)	2 (1 to 4)	10 (9 to 11)	102 (84 to 125)	25 (19 to 33)	43 (30 to 61)	6 (3 to 11)	28 (26 to 32)
Benin	2024	37 (30 to 45)	9 (7 to 12)	16 (11 to 22)	2 (1 to 4)	10 (9 to 11)	104 (85 to 126)	25 (19 to 34)	44 (30 to 62)	6 (3 to 12)	28 (25 to 32)
Benin	2025	38 (31 to 46)	9 (7 to 13)	16 (11 to 23)	2 (1 to 4)	10 (9 to 11)	105 (87 to 128)	26 (19 to 35)	45 (31 to 63)	6 (3 to 12)	28 (25 to 32)
Benin	2026	38 (32 to 46)	10 (7 to 13)	16 (11 to 23)	2 (1 to 4)	10 (9 to 11)	106 (88 to 129)	27 (20 to 36)	46 (32 to 65)	7 (3 to 12)	28 (24 to 31)
Benin	2027	39 (32 to 47)	10 (7 to 13)	17 (11 to 24)	2 (1 to 5)	10 (9 to 11)	108 (90 to 131)	28 (21 to 37)	46 (32 to 66)	7 (3 to 13)	27 (24 to 32)
Benin	2028	39 (32 to 48)	10 (7 to 14)	17 (12 to 24)	2 (1 to 5)	10 (8 to 11)	110 (90 to 135)	28 (21 to 38)	47 (33 to 67)	7 (3 to 13)	27 (24 to 32)
Benin	2029	40 (33 to 49)	10 (8 to 14)	17 (12 to 25)	3 (1 to 5)	10 (8 to 11)	112 (91 to 137)	29 (21 to 40)	48 (33 to 69)	7 (3 to 13)	27 (23 to 32)
Benin	2030	41 (33 to 51)	11 (8 to 15)	18 (12 to 25)	3 (1 to 5)	10 (8 to 11)	114 (92 to 143)	30 (22 to 42)	49 (34 to 70)	7 (3 to 14)	27 (23 to 32)

Benin	2031	41 (34 to 52)	11 (8 to 15)	18 (12 to 26)	3 (1 to 5)	10 (8 to 11)	116 (94 to 144)	31 (23 to 43)	50 (35 to 71)	8 (4 to 14)	27 (23 to 32)
Benin	2032	42 (34 to 52)	11 (8 to 16)	18 (13 to 26)	3 (1 to 5)	9 (8 to 11)	118 (96 to 145)	32 (23 to 45)	51 (35 to 73)	8 (4 to 15)	26 (22 to 32)
Benin	2033	43 (35 to 52)	12 (8 to 16)	19 (13 to 27)	3 (1 to 5)	9 (8 to 11)	120 (97 to 146)	33 (24 to 46)	52 (36 to 75)	8 (4 to 15)	26 (22 to 32)
Benin	2034	43 (36 to 53)	12 (9 to 17)	19 (13 to 27)	3 (1 to 6)	9 (8 to 12)	122 (99 to 150)	34 (24 to 47)	53 (37 to 76)	8 (4 to 16)	26 (22 to 32)
Benin	2035	44 (36 to 55)	12 (9 to 17)	19 (13 to 28)	3 (1 to 6)	9 (8 to 11)	124 (99 to 154)	35 (25 to 48)	55 (37 to 78)	8 (4 to 16)	26 (22 to 32)
Benin	2036	45 (36 to 57)	13 (9 to 18)	20 (14 to 28)	3 (1 to 6)	9 (8 to 12)	126 (101 to 159)	36 (25 to 50)	56 (38 to 79)	9 (4 to 16)	26 (21 to 33)
Benin	2037	46 (37 to 57)	13 (9 to 18)	20 (14 to 29)	3 (1 to 6)	9 (8 to 11)	128 (102 to 161)	37 (26 to 51)	57 (39 to 81)	9 (4 to 17)	26 (21 to 32)
Benin	2038	47 (37 to 59)	13 (9 to 19)	21 (14 to 30)	3 (2 to 6)	9 (8 to 12)	131 (105 to 165)	38 (26 to 54)	58 (40 to 83)	9 (4 to 17)	26 (21 to 33)
Benin	2039	48 (39 to 59)	14 (10 to 20)	21 (14 to 30)	3 (2 to 6)	9 (7 to 12)	133 (108 to 166)	39 (27 to 55)	59 (40 to 84)	9 (4 to 18)	26 (21 to 33)
Benin	2040	48 (39 to 60)	14 (10 to 20)	22 (15 to 31)	3 (2 to 7)	9 (7 to 12)	135 (110 to 169)	40 (28 to 56)	60 (41 to 86)	9 (4 to 18)	26 (21 to 33)
Benin	2041	49 (39 to 62)	15 (10 to 21)	22 (15 to 31)	3 (2 to 7)	9 (7 to 12)	138 (109 to 173)	41 (28 to 58)	62 (42 to 88)	10 (5 to 19)	26 (21 to 33)
Benin	2042	50 (40 to 64)	15 (10 to 21)	22 (15 to 32)	4 (2 to 7)	9 (7 to 12)	141 (112 to 178)	42 (29 to 60)	63 (43 to 90)	10 (5 to 19)	26 (21 to 33)
Benin	2043	51 (41 to 65)	15 (11 to 22)	23 (16 to 33)	4 (2 to 7)	9 (7 to 12)	144 (115 to 183)	43 (30 to 61)	64 (44 to 92)	10 (5 to 20)	26 (21 to 33)
Benin	2044	52 (41 to 66)	16 (11 to 23)	23 (16 to 34)	4 (2 to 7)	9 (7 to 12)	146 (116 to 184)	44 (31 to 64)	66 (45 to 94)	11 (5 to 20)	26 (20 to 34)
Benin	2045	53 (42 to 68)	16 (11 to 23)	24 (16 to 34)	4 (2 to 7)	9 (7 to 12)	149 (119 to 189)	46 (32 to 65)	67 (46 to 96)	11 (5 to 21)	26 (20 to 34)

Benin	2046	54 (42 to 69)	17 (12 to 24)	24 (17 to 35)	4 (2 to 8)	9 (7 to 12)	152 (119 to 192)	47 (33 to 68)	69 (47 to 99)	11 (5 to 21)	26 (20 to 34)
Benin	2047	56 (43 to 71)	17 (12 to 25)	25 (17 to 36)	4 (2 to 8)	9 (7 to 13)	156 (121 to 198)	48 (34 to 70)	70 (48 to 101)	11 (5 to 22)	26 (20 to 35)
Benin	2048	57 (45 to 72)	18 (12 to 26)	26 (17 to 37)	4 (2 to 8)	9 (7 to 12)	159 (125 to 203)	50 (35 to 72)	71 (49 to 103)	12 (5 to 22)	26 (20 to 35)
Benin	2049	58 (46 to 73)	18 (13 to 26)	26 (18 to 38)	4 (2 to 8)	9 (7 to 13)	162 (128 to 204)	51 (36 to 73)	73 (50 to 106)	12 (6 to 23)	26 (21 to 35)
Benin	2050	59 (46 to 75)	19 (13 to 27)	27 (18 to 38)	4 (2 to 9)	9 (7 to 13)	165 (129 to 210)	52 (37 to 76)	75 (51 to 108)	12 (6 to 24)	26 (21 to 36)
Bermuda	1995	3477 (2621 to 4769)	1099 (842 to 1410)	983 (678 to 1342)	1395 (720 to 2573)	0 (0 to 0)	2192 (1653 to 3007)	693 (531 to 889)	620 (428 to 846)	880 (454 to 1622)	0 (0 to 0)
Bermuda	1996	3519 (2677 to 4732)	1128 (869 to 1435)	1089 (767 to 1489)	1302 (678 to 2395)	0 (0 to 0)	2218 (1687 to 2983)	711 (548 to 905)	686 (484 to 939)	821 (427 to 1510)	0 (0 to 0)
Bermuda	1997	3355 (2606 to 4319)	1076 (833 to 1357)	1206 (862 to 1629)	1073 (562 to 1956)	0 (0 to 0)	2115 (1643 to 2723)	678 (525 to 855)	760 (543 to 1027)	676 (354 to 1233)	0 (0 to 0)
Bermuda	1998	3395 (2696 to 4233)	1070 (833 to 1321)	1399 (1011 to 1868)	926 (490 to 1681)	0 (0 to 0)	2140 (1700 to 2669)	674 (525 to 833)	882 (637 to 1177)	584 (309 to 1060)	0 (0 to 0)
Bermuda	1999	3561 (2893 to 4378)	1075 (851 to 1318)	1644 (1220 to 2149)	842 (455 to 1494)	0 (0 to 0)	2245 (1824 to 2760)	678 (537 to 831)	1036 (769 to 1355)	531 (287 to 942)	0 (0 to 0)
Bermuda	2000	3857 (3206 to 4641)	1109 (899 to 1339)	1931 (1471 to 2471)	818 (460 to 1418)	0 (0 to 0)	2432 (2021 to 2926)	699 (567 to 844)	1217 (927 to 1558)	515 (290 to 894)	0 (0 to 0)
Bermuda	2001	4303 (3626 to 5063)	1192 (997 to 1417)	2263 (1787 to 2816)	848 (496 to 1388)	0 (0 to 0)	2713 (2286 to 3192)	752 (628 to 893)	1427 (1126 to 1775)	534 (313 to 875)	0 (0 to 0)

Bermuda	2002	4479 (3887 to 5186)	1181 (1018 to 1367)	2462 (2015 to 3004)	836 (515 to 1324)	0 (0 to 0)	2824 (2451 to 3269)	745 (642 to 862)	1552 (1270 to 1894)	527 (325 to 835)	0 (0 to 0)
Bermuda	2003	4820 (4273 to 5471)	1238 (1095 to 1399)	2694 (2276 to 3204)	887 (585 to 1304)	0 (0 to 0)	3038 (2693 to 3449)	781 (690 to 882)	1699 (1435 to 2020)	559 (369 to 822)	0 (0 to 0)
Bermuda	2004	5074 (4600 to 5636)	1272 (1151 to 1406)	2866 (2487 to 3326)	935 (657 to 1295)	0 (0 to 0)	3198 (2900 to 3553)	802 (726 to 887)	1807 (1568 to 2097)	590 (414 to 816)	0 (0 to 0)
Bermuda	2005	5065 (4651 to 5553)	1308 (1204 to 1421)	2668 (2368 to 3027)	1089 (817 to 1429)	0 (0 to 0)	3193 (2932 to 3500)	825 (759 to 896)	1682 (1493 to 1908)	686 (515 to 901)	0 (0 to 0)
Bermuda	2006	4990 (4598 to 5418)	1359 (1264 to 1454)	2201 (1953 to 2493)	1429 (1145 to 1767)	0 (0 to 0)	3146 (2899 to 3416)	857 (797 to 917)	1388 (1231 to 1572)	901 (722 to 1114)	0 (0 to 0)
Bermuda	2007	5223 (4809 to 5687)	1455 (1361 to 1561)	1491 (1298 to 1717)	2277 (1932 to 2664)	0 (0 to 0)	3292 (3031 to 3585)	917 (858 to 984)	940 (818 to 1083)	1435 (1218 to 1679)	0 (0 to 0)
Bermuda	2008	5644 (5236 to 6099)	1546 (1448 to 1652)	1202 (1015 to 1400)	2896 (2557 to 3278)	0 (0 to 0)	3558 (3301 to 3845)	975 (913 to 1042)	758 (640 to 883)	1826 (1612 to 2066)	0 (0 to 0)
Bermuda	2009	6074 (5670 to 6504)	1666 (1564 to 1772)	1059 (869 to 1252)	3350 (3018 to 3704)	0 (0 to 0)	3829 (3575 to 4100)	1050 (986 to 1117)	667 (548 to 789)	2112 (1903 to 2335)	0 (0 to 0)
Bermuda	2010	6619 (6208 to 7057)	1892 (1786 to 2003)	1008 (814 to 1210)	3720 (3362 to 4101)	0 (0 to 0)	4173 (3914 to 4449)	1193 (1126 to 1263)	635 (513 to 763)	2345 (2120 to 2585)	0 (0 to 0)
Bermuda	2011	6912 (6493 to 7356)	2043 (1937 to 2157)	964 (776 to 1164)	3905 (3557 to 4292)	0 (0 to 0)	4357 (4093 to 4637)	1288 (1221 to 1360)	607 (489 to 734)	2462 (2242 to 2705)	0 (0 to 0)

Bermuda	2012	6942 (6539 to 7379)	2068 (1962 to 2172)	909 (729 to 1104)	3965 (3600 to 4359)	0 (0 to 0)	4376 (4122 to 4652)	1304 (1237 to 1369)	573 (459 to 696)	2500 (2270 to 2748)	0 (0 to 0)
Bermuda	2013	6885 (6464 to 7362)	2041 (1934 to 2152)	825 (655 to 1010)	4019 (3645 to 4430)	0 (0 to 0)	4340 (4075 to 4641)	1286 (1219 to 1357)	520 (413 to 636)	2534 (2298 to 2793)	0 (0 to 0)
Bermuda	2014	6736 (6293 to 7205)	1969 (1858 to 2086)	734 (575 to 911)	4033 (3661 to 4423)	0 (0 to 0)	4246 (3967 to 4542)	1241 (1171 to 1315)	462 (362 to 574)	2543 (2308 to 2788)	0 (0 to 0)
Bermuda	2015	6720 (6252 to 7182)	1916 (1790 to 2042)	680 (524 to 859)	4124 (3749 to 4527)	0 (0 to 0)	4237 (3941 to 4528)	1208 (1129 to 1288)	429 (330 to 541)	2600 (2363 to 2854)	0 (0 to 0)
Bermuda	2016	6791 (6203 to 7388)	1892 (1740 to 2046)	674 (511 to 862)	4225 (3695 to 4799)	0 (0 to 0)	4281 (3911 to 4657)	1193 (1097 to 1290)	425 (322 to 543)	2664 (2329 to 3025)	0 (0 to 0)
Bermuda	2017	7027 (5973 to 8208)	1942 (1740 to 2159)	690 (506 to 905)	4395 (3589 to 5335)	0 (0 to 0)	4430 (3765 to 5174)	1224 (1097 to 1361)	435 (319 to 571)	2771 (2263 to 3363)	0 (0 to 0)
Bermuda	2018	7196 (6116 to 8388)	1992 (1776 to 2227)	703 (515 to 922)	4502 (3673 to 5454)	0 (0 to 0)	4537 (3855 to 5288)	1256 (1120 to 1404)	443 (325 to 581)	2838 (2315 to 3438)	0 (0 to 0)
Bermuda	2019	7454 (6321 to 8668)	2082 (1844 to 2331)	714 (524 to 937)	4658 (3799 to 5639)	0 (0 to 0)	4699 (3984 to 5464)	1312 (1163 to 1470)	450 (330 to 591)	2936 (2395 to 3555)	0 (0 to 0)
Bermuda	2020	7738 (6566 to 9039)	2176 (1932 to 2452)	731 (537 to 959)	4831 (3933 to 5846)	0 (0 to 0)	4878 (4139 to 5698)	1372 (1218 to 1546)	461 (338 to 605)	3046 (2480 to 3685)	0 (0 to 0)
Bermuda	2021	7877 (6664 to 9225)	2231 (1957 to 2528)	733 (538 to 962)	4913 (3993 to 5948)	0 (0 to 0)	4966 (4201 to 5815)	1406 (1234 to 1594)	462 (339 to 607)	3097 (2517 to 3750)	0 (0 to 0)

Bermuda	2022	8001 (6772 to 9333)	2282 (2004 to 2590)	734 (538 to 963)	4986 (4042 to 6042)	0 (0 to 0)	5044 (4269 to 5883)	1438 (1263 to 1633)	463 (339 to 607)	3143 (2548 to 3809)	0 (0 to 0)
Bermuda	2023	8133 (6862 to 9534)	2336 (2041 to 2656)	735 (539 to 963)	5063 (4099 to 6126)	0 (0 to 0)	5127 (4326 to 6010)	1472 (1287 to 1675)	463 (340 to 607)	3192 (2584 to 3862)	0 (0 to 0)
Bermuda	2024	8247 (6942 to 9657)	2386 (2078 to 2727)	734 (538 to 961)	5128 (4158 to 6215)	0 (0 to 0)	5199 (4376 to 6088)	1504 (1310 to 1719)	462 (339 to 606)	3232 (2621 to 3918)	0 (0 to 0)
Bermuda	2025	8311 (7037 to 9805)	2422 (2099 to 2803)	728 (534 to 953)	5161 (4183 to 6258)	0 (0 to 0)	5239 (4436 to 6181)	1527 (1323 to 1767)	459 (337 to 601)	3253 (2637 to 3945)	0 (0 to 0)
Bermuda	2026	8322 (6964 to 9775)	2443 (2119 to 2821)	718 (527 to 939)	5161 (4192 to 6248)	0 (0 to 0)	5246 (4390 to 6162)	1540 (1336 to 1778)	453 (332 to 592)	3254 (2642 to 3939)	0 (0 to 0)
Bermuda	2027	8331 (7019 to 9817)	2463 (2118 to 2853)	708 (520 to 926)	5160 (4198 to 6254)	0 (0 to 0)	5252 (4425 to 6188)	1553 (1335 to 1799)	446 (328 to 584)	3253 (2647 to 3942)	0 (0 to 0)
Bermuda	2028	8343 (6963 to 9812)	2483 (2134 to 2861)	699 (514 to 914)	5162 (4203 to 6248)	0 (0 to 0)	5260 (4390 to 6186)	1565 (1346 to 1804)	441 (324 to 576)	3254 (2650 to 3939)	0 (0 to 0)
Bermuda	2029	8354 (7028 to 9836)	2501 (2142 to 2892)	691 (507 to 901)	5162 (4198 to 6270)	0 (0 to 0)	5266 (4431 to 6200)	1577 (1350 to 1823)	435 (320 to 568)	3254 (2647 to 3953)	0 (0 to 0)
Bermuda	2030	8358 (6986 to 9870)	2517 (2148 to 2927)	682 (501 to 887)	5159 (4191 to 6268)	0 (0 to 0)	5269 (4404 to 6222)	1587 (1354 to 1845)	430 (316 to 559)	3252 (2642 to 3952)	0 (0 to 0)
Bermuda	2031	8356 (7014 to 9881)	2529 (2149 to 2964)	674 (494 to 878)	5152 (4181 to 6263)	0 (0 to 0)	5267 (4422 to 6229)	1594 (1355 to 1868)	425 (312 to 554)	3248 (2636 to 3948)	0 (0 to 0)

Bermuda	2032	8384 (6946 to 9940)	2551 (2145 to 3021)	668 (490 to 871)	5165 (4188 to 6278)	0 (0 to 0)	5285 (4379 to 6266)	1608 (1352 to 1904)	421 (309 to 549)	3256 (2640 to 3957)	0 (0 to 0)
Bermuda	2033	8417 (7012 to 9996)	2573 (2155 to 3046)	664 (486 to 864)	5180 (4193 to 6295)	0 (0 to 0)	5306 (4420 to 6301)	1622 (1359 to 1920)	418 (306 to 545)	3266 (2643 to 3968)	0 (0 to 0)
Bermuda	2034	8452 (7022 to 10090)	2595 (2170 to 3093)	660 (484 to 858)	5198 (4209 to 6316)	0 (0 to 0)	5328 (4426 to 6361)	1636 (1368 to 1950)	416 (305 to 541)	3277 (2653 to 3982)	0 (0 to 0)
Bermuda	2035	8486 (7079 to 10107)	2616 (2189 to 3130)	656 (480 to 853)	5215 (4217 to 6346)	0 (0 to 0)	5350 (4463 to 6372)	1649 (1380 to 1973)	413 (303 to 537)	3288 (2658 to 4001)	0 (0 to 0)
Bermuda	2036	8514 (7057 to 10240)	2633 (2196 to 3169)	652 (476 to 847)	5229 (4215 to 6356)	0 (0 to 0)	5367 (4449 to 6455)	1660 (1385 to 1998)	411 (300 to 534)	3296 (2657 to 4007)	0 (0 to 0)
Bermuda	2037	8540 (7091 to 10189)	2650 (2211 to 3198)	649 (474 to 842)	5242 (4216 to 6372)	0 (0 to 0)	5384 (4470 to 6423)	1670 (1394 to 2016)	409 (299 to 531)	3304 (2658 to 4017)	0 (0 to 0)
Bermuda	2038	8566 (7083 to 10295)	2665 (2214 to 3227)	646 (472 to 839)	5255 (4220 to 6401)	0 (0 to 0)	5400 (4465 to 6490)	1680 (1396 to 2034)	407 (298 to 529)	3313 (2660 to 4035)	0 (0 to 0)
Bermuda	2039	8595 (7064 to 10309)	2681 (2214 to 3229)	643 (471 to 836)	5271 (4222 to 6431)	0 (0 to 0)	5419 (4453 to 6499)	1690 (1396 to 2035)	406 (297 to 527)	3323 (2661 to 4054)	0 (0 to 0)
Bermuda	2040	8635 (7072 to 10399)	2700 (2216 to 3285)	641 (469 to 835)	5293 (4235 to 6473)	0 (0 to 0)	5443 (4458 to 6555)	1702 (1397 to 2071)	404 (296 to 526)	3337 (2669 to 4080)	0 (0 to 0)
Bermuda	2041	8662 (7111 to 10411)	2715 (2235 to 3301)	639 (467 to 833)	5308 (4242 to 6491)	0 (0 to 0)	5461 (4483 to 6563)	1711 (1409 to 2081)	403 (294 to 525)	3346 (2674 to 4092)	0 (0 to 0)

Bermuda	2042	8691 (7150 to 10531)	2728 (2239 to 3318)	638 (466 to 832)	5325 (4249 to 6518)	0 (0 to 0)	5479 (4507 to 6638)	1720 (1412 to 2092)	402 (293 to 524)	3357 (2678 to 4109)	0 (0 to 0)
Bermuda	2043	8716 (7143 to 10506)	2740 (2223 to 3324)	636 (464 to 830)	5339 (4261 to 6546)	0 (0 to 0)	5494 (4503 to 6623)	1727 (1401 to 2096)	401 (292 to 523)	3366 (2686 to 4126)	0 (0 to 0)
Bermuda	2044	8750 (7137 to 10556)	2756 (2240 to 3340)	635 (463 to 830)	5359 (4274 to 6579)	0 (0 to 0)	5516 (4499 to 6655)	1737 (1412 to 2106)	400 (292 to 523)	3379 (2694 to 4147)	0 (0 to 0)
Bermuda	2045	8779 (7200 to 10609)	2769 (2231 to 3363)	634 (461 to 830)	5377 (4284 to 6610)	0 (0 to 0)	5535 (4539 to 6688)	1745 (1406 to 2120)	400 (291 to 523)	3389 (2701 to 4167)	0 (0 to 0)
Bermuda	2046	8807 (7169 to 10707)	2781 (2250 to 3427)	633 (460 to 830)	5393 (4297 to 6639)	0 (0 to 0)	5552 (4519 to 6750)	1753 (1418 to 2160)	399 (290 to 523)	3400 (2709 to 4185)	0 (0 to 0)
Bermuda	2047	8845 (7215 to 10779)	2797 (2255 to 3444)	633 (460 to 831)	5416 (4321 to 6677)	0 (0 to 0)	5576 (4548 to 6795)	1763 (1422 to 2171)	399 (290 to 524)	3414 (2724 to 4209)	0 (0 to 0)
Bermuda	2048	8887 (7268 to 10768)	2814 (2276 to 3472)	632 (460 to 832)	5441 (4347 to 6715)	0 (0 to 0)	5602 (4582 to 6788)	1774 (1435 to 2189)	399 (290 to 524)	3430 (2740 to 4233)	0 (0 to 0)
Bermuda	2049	8921 (7249 to 10893)	2827 (2264 to 3516)	632 (459 to 833)	5462 (4351 to 6745)	0 (0 to 0)	5624 (4570 to 6867)	1782 (1427 to 2217)	398 (289 to 525)	3443 (2743 to 4252)	0 (0 to 0)
Bermuda	2050	8954 (7285 to 10984)	2840 (2270 to 3528)	632 (459 to 834)	5482 (4350 to 6778)	0 (0 to 0)	5644 (4592 to 6925)	1790 (1431 to 2224)	398 (289 to 526)	3456 (2742 to 4273)	0 (0 to 0)
Bhutan	1995	52 (43 to 63)	41 (32 to 51)	9 (7 to 13)	0 (0 to 1)	1 (1 to 1)	153 (125 to 184)	120 (94 to 149)	27 (19 to 38)	1 (1 to 3)	4 (4 to 4)
Bhutan	1996	55 (45 to 66)	44 (35 to 54)	10 (7 to 14)	0 (0 to 1)	0 (0 to 0)	160 (131 to 193)	128 (102 to 159)	29 (21 to 41)	1 (1 to 3)	1 (1 to 1)

Bhutan	1997	74 (64 to 85)	45 (35 to 55)	11 (8 to 15)	1 (0 to 1)	18 (18 to 18)	217 (189 to 249)	130 (103 to 161)	32 (22 to 44)	1 (1 to 3)	54 (54 to 54)
Bhutan	1998	64 (55 to 73)	41 (33 to 50)	10 (7 to 14)	0 (0 to 1)	12 (12 to 12)	186 (160 to 215)	120 (96 to 147)	30 (21 to 42)	1 (1 to 2)	34 (34 to 34)
Bhutan	1999	56 (46 to 66)	44 (36 to 54)	10 (7 to 14)	0 (0 to 1)	1 (1 to 1)	163 (135 to 195)	129 (104 to 159)	29 (20 to 40)	1 (1 to 3)	4 (4 to 4)
Bhutan	2000	58 (49 to 69)	44 (36 to 54)	8 (6 to 11)	0 (0 to 1)	6 (6 to 6)	170 (143 to 201)	129 (104 to 159)	23 (16 to 33)	1 (1 to 2)	16 (16 to 16)
Bhutan	2001	60 (50 to 71)	48 (39 to 59)	8 (5 to 11)	1 (0 to 1)	4 (4 to 4)	177 (148 to 209)	141 (113 to 172)	22 (15 to 31)	2 (1 to 3)	12 (12 to 12)
Bhutan	2002	56 (48 to 66)	44 (35 to 53)	8 (5 to 11)	0 (0 to 1)	4 (4 to 4)	165 (139 to 193)	128 (104 to 156)	23 (16 to 32)	1 (1 to 2)	13 (13 to 13)
Bhutan	2003	60 (51 to 69)	42 (33 to 50)	9 (6 to 13)	0 (0 to 1)	9 (9 to 9)	175 (150 to 202)	122 (98 to 147)	27 (19 to 37)	1 (0 to 2)	25 (25 to 25)
Bhutan	2004	58 (49 to 67)	41 (33 to 48)	10 (7 to 14)	0 (0 to 0)	7 (7 to 7)	168 (143 to 195)	119 (96 to 142)	29 (20 to 40)	1 (0 to 1)	20 (20 to 20)
Bhutan	2005	69 (59 to 79)	45 (36 to 54)	11 (8 to 15)	0 (0 to 0)	13 (13 to 13)	202 (174 to 232)	132 (106 to 159)	32 (22 to 44)	1 (0 to 1)	38 (38 to 38)
Bhutan	2006	68 (58 to 79)	45 (36 to 55)	11 (8 to 15)	0 (0 to 0)	12 (12 to 12)	199 (171 to 231)	130 (105 to 160)	32 (23 to 44)	1 (0 to 1)	36 (36 to 36)
Bhutan	2007	70 (59 to 82)	48 (39 to 59)	12 (8 to 16)	0 (0 to 0)	10 (10 to 10)	204 (173 to 240)	142 (114 to 174)	34 (24 to 47)	1 (0 to 1)	28 (28 to 28)
Bhutan	2008	67 (57 to 79)	49 (39 to 60)	12 (8 to 16)	0 (0 to 1)	7 (7 to 7)	197 (166 to 230)	143 (114 to 176)	34 (24 to 47)	1 (0 to 2)	20 (20 to 20)
Bhutan	2009	73 (61 to 86)	55 (44 to 67)	13 (9 to 18)	0 (0 to 1)	5 (5 to 5)	213 (178 to 251)	160 (128 to 196)	38 (27 to 52)	1 (0 to 2)	14 (14 to 14)
Bhutan	2010	82 (68 to 97)	63 (51 to 77)	14 (10 to 20)	0 (0 to 1)	4 (4 to 4)	240 (200 to 285)	185 (149 to 225)	42 (30 to 58)	1 (1 to 3)	11 (11 to 11)
Bhutan	2011	81 (68 to 96)	62 (49 to 76)	15 (11 to 21)	1 (0 to 1)	4 (4 to 4)	238 (198 to 282)	181 (144 to 221)	45 (32 to 60)	2 (1 to 3)	11 (11 to 11)

Bhutan	2012	89 (74 to 105)	65 (52 to 79)	17 (12 to 23)	1 (0 to 1)	5 (5 to 5)	259 (217 to 306)	191 (152 to 232)	51 (36 to 68)	2 (1 to 4)	15 (15 to 15)
Bhutan	2013	92 (76 to 108)	69 (55 to 83)	19 (13 to 25)	1 (0 to 2)	3 (3 to 3)	269 (223 to 318)	201 (162 to 244)	55 (39 to 75)	3 (1 to 5)	10 (10 to 10)
Bhutan	2014	92 (77 to 107)	68 (54 to 82)	19 (13 to 26)	1 (1 to 2)	4 (4 to 4)	270 (225 to 315)	198 (159 to 240)	57 (39 to 77)	3 (1 to 5)	12 (12 to 12)
Bhutan	2015	98 (82 to 115)	71 (57 to 87)	20 (14 to 27)	1 (1 to 2)	7 (7 to 7)	288 (241 to 337)	208 (168 to 254)	58 (41 to 79)	3 (2 to 5)	19 (19 to 19)
Bhutan	2016	109 (92 to 129)	82 (66 to 100)	21 (14 to 29)	1 (1 to 2)	5 (5 to 5)	320 (269 to 377)	240 (194 to 294)	61 (42 to 84)	4 (2 to 7)	16 (16 to 16)
Bhutan	2017	108 (90 to 129)	84 (68 to 104)	19 (14 to 26)	1 (1 to 2)	3 (3 to 3)	316 (262 to 378)	247 (198 to 305)	56 (40 to 77)	4 (2 to 7)	10 (10 to 10)
Bhutan	2018	114 (94 to 136)	90 (73 to 111)	19 (13 to 26)	1 (1 to 3)	3 (3 to 3)	334 (276 to 399)	265 (213 to 326)	56 (39 to 77)	4 (2 to 7)	10 (10 to 10)
Bhutan	2019	94 (78 to 112)	70 (56 to 87)	19 (14 to 27)	1 (1 to 2)	3 (3 to 3)	275 (228 to 329)	205 (165 to 253)	57 (40 to 78)	3 (2 to 6)	10 (10 to 10)
Bhutan	2020	99 (82 to 119)	74 (59 to 91)	20 (14 to 28)	1 (1 to 2)	3 (3 to 4)	289 (239 to 347)	216 (173 to 267)	59 (42 to 82)	4 (2 to 7)	10 (9 to 10)
Bhutan	2021	102 (84 to 122)	77 (61 to 95)	21 (15 to 29)	1 (1 to 2)	3 (3 to 4)	299 (247 to 357)	224 (179 to 277)	62 (44 to 85)	4 (2 to 7)	10 (9 to 11)
Bhutan	2022	106 (87 to 128)	80 (63 to 99)	22 (15 to 30)	1 (1 to 2)	3 (3 to 4)	311 (254 to 375)	233 (185 to 291)	64 (45 to 88)	4 (2 to 7)	10 (9 to 11)
Bhutan	2023	110 (89 to 134)	83 (65 to 105)	23 (16 to 31)	1 (1 to 3)	3 (3 to 4)	322 (261 to 394)	242 (189 to 308)	66 (47 to 91)	4 (2 to 8)	10 (9 to 11)
Bhutan	2024	114 (92 to 140)	86 (66 to 111)	23 (17 to 32)	1 (1 to 3)	3 (3 to 4)	335 (268 to 411)	252 (192 to 325)	69 (48 to 95)	4 (2 to 8)	10 (9 to 11)
Bhutan	2025	119 (95 to 146)	89 (68 to 116)	24 (17 to 34)	1 (1 to 3)	3 (3 to 4)	347 (278 to 428)	262 (198 to 339)	71 (50 to 98)	4 (2 to 8)	10 (9 to 11)
Bhutan	2026	123 (98 to 154)	93 (70 to 122)	25 (18 to 35)	2 (1 to 3)	3 (3 to 4)	360 (286 to 452)	272 (204 to 358)	74 (52 to 102)	4 (2 to 8)	10 (9 to 12)

Bhutan	2027	128 (100 to 161)	97 (72 to 128)	26 (18 to 36)	2 (1 to 3)	3 (3 to 4)	374 (294 to 472)	283 (210 to 376)	76 (54 to 105)	5 (2 to 9)	10 (9 to 12)
Bhutan	2028	133 (103 to 170)	101 (74 to 135)	27 (19 to 37)	2 (1 to 3)	3 (3 to 4)	388 (302 to 497)	294 (217 to 395)	79 (56 to 109)	5 (2 to 9)	10 (9 to 12)
Bhutan	2029	138 (107 to 178)	105 (76 to 142)	28 (20 to 39)	2 (1 to 3)	3 (3 to 4)	405 (312 to 520)	307 (221 to 416)	82 (58 to 113)	5 (2 to 9)	10 (9 to 12)
Bhutan	2030	144 (109 to 185)	109 (77 to 148)	29 (21 to 40)	2 (1 to 3)	3 (3 to 4)	420 (320 to 542)	320 (225 to 434)	85 (60 to 117)	5 (3 to 10)	10 (9 to 12)
Bhutan	2031	149 (113 to 195)	114 (80 to 159)	30 (21 to 41)	2 (1 to 3)	3 (3 to 4)	437 (331 to 569)	333 (235 to 464)	88 (63 to 121)	5 (3 to 10)	10 (9 to 12)
Bhutan	2032	155 (117 to 206)	118 (83 to 166)	31 (22 to 43)	2 (1 to 4)	4 (3 to 4)	453 (344 to 604)	346 (242 to 486)	91 (65 to 126)	6 (3 to 11)	10 (9 to 13)
Bhutan	2033	160 (120 to 212)	122 (85 to 173)	32 (23 to 44)	2 (1 to 4)	4 (3 to 4)	469 (351 to 619)	358 (250 to 508)	94 (67 to 130)	6 (3 to 11)	10 (9 to 13)
Bhutan	2034	166 (124 to 221)	127 (87 to 182)	33 (24 to 46)	2 (1 to 4)	4 (3 to 4)	487 (363 to 648)	373 (256 to 533)	98 (70 to 135)	6 (3 to 11)	10 (9 to 13)
Bhutan	2035	172 (127 to 230)	132 (89 to 189)	34 (25 to 48)	2 (1 to 4)	4 (3 to 4)	504 (373 to 674)	387 (261 to 552)	101 (72 to 139)	6 (3 to 12)	11 (9 to 13)
Bhutan	2036	179 (130 to 243)	137 (91 to 198)	36 (25 to 49)	2 (1 to 4)	4 (3 to 5)	523 (380 to 710)	402 (267 to 579)	104 (74 to 144)	6 (3 to 12)	11 (9 to 13)
Bhutan	2037	185 (133 to 253)	142 (94 to 204)	37 (26 to 51)	2 (1 to 4)	4 (3 to 5)	542 (388 to 740)	417 (276 to 598)	108 (77 to 149)	7 (3 to 13)	11 (9 to 13)
Bhutan	2038	191 (137 to 260)	148 (96 to 212)	38 (27 to 52)	2 (1 to 4)	4 (3 to 5)	560 (400 to 762)	432 (282 to 621)	111 (79 to 153)	7 (3 to 13)	11 (9 to 14)
Bhutan	2039	198 (141 to 271)	153 (101 to 221)	39 (28 to 54)	2 (1 to 5)	4 (3 to 5)	579 (413 to 793)	447 (296 to 646)	114 (81 to 157)	7 (3 to 14)	11 (9 to 14)
Bhutan	2040	205 (145 to 284)	158 (103 to 234)	40 (28 to 55)	3 (1 to 5)	4 (3 to 5)	599 (424 to 832)	464 (302 to 684)	117 (83 to 162)	7 (4 to 14)	11 (9 to 14)
Bhutan	2041	212 (149 to 290)	164 (107 to 240)	41 (29 to 57)	3 (1 to 5)	4 (3 to 5)	620 (437 to 849)	481 (312 to 704)	120 (86 to 166)	8 (4 to 14)	11 (9 to 14)

Bhutan	2042	218 (153 to 307)	170 (108 to 254)	42 (30 to 58)	3 (1 to 5)	4 (3 to 5)	639 (447 to 898)	497 (317 to 744)	123 (88 to 170)	8 (4 to 15)	11 (9 to 15)
Bhutan	2043	226 (156 to 315)	176 (112 to 265)	43 (31 to 60)	3 (1 to 5)	4 (3 to 5)	661 (458 to 922)	515 (327 to 776)	126 (90 to 174)	8 (4 to 15)	12 (9 to 15)
Bhutan	2044	233 (160 to 328)	182 (115 to 275)	44 (31 to 61)	3 (1 to 5)	4 (3 to 5)	682 (470 to 959)	533 (338 to 806)	129 (92 to 178)	8 (4 to 16)	12 (9 to 16)
Bhutan	2045	241 (166 to 337)	189 (120 to 285)	45 (32 to 62)	3 (1 to 6)	4 (3 to 5)	705 (486 to 986)	553 (350 to 834)	132 (94 to 182)	9 (4 to 16)	12 (9 to 15)
Bhutan	2046	249 (172 to 351)	195 (122 to 296)	46 (33 to 64)	3 (1 to 6)	4 (3 to 5)	728 (503 to 1027)	572 (358 to 866)	135 (96 to 186)	9 (4 to 17)	12 (9 to 16)
Bhutan	2047	256 (175 to 362)	202 (124 to 309)	47 (33 to 65)	3 (2 to 6)	4 (3 to 6)	750 (511 to 1061)	591 (364 to 904)	137 (98 to 191)	9 (4 to 17)	12 (9 to 17)
Bhutan	2048	264 (178 to 376)	209 (128 to 321)	48 (34 to 67)	3 (2 to 6)	4 (3 to 6)	772 (521 to 1100)	610 (376 to 940)	140 (99 to 195)	9 (5 to 18)	12 (10 to 17)
Bhutan	2049	271 (187 to 395)	215 (133 to 337)	49 (35 to 68)	3 (2 to 6)	4 (3 to 6)	794 (546 to 1157)	630 (389 to 986)	142 (101 to 198)	10 (5 to 18)	13 (10 to 17)
Bhutan	2050	279 (189 to 404)	222 (137 to 345)	49 (35 to 68)	3 (2 to 6)	4 (3 to 6)	816 (554 to 1183)	649 (401 to 1011)	144 (102 to 200)	10 (5 to 19)	13 (10 to 18)
Bolivia	1995	84 (71 to 100)	39 (31 to 48)	32 (24 to 44)	7 (3 to 12)	6 (6 to 6)	171 (144 to 203)	79 (63 to 98)	65 (48 to 89)	13 (6 to 25)	13 (13 to 13)
Bolivia	1996	93 (79 to 108)	44 (36 to 55)	34 (25 to 46)	7 (3 to 13)	8 (8 to 8)	189 (160 to 221)	90 (73 to 111)	69 (51 to 93)	14 (7 to 26)	16 (16 to 16)
Bolivia	1997	97 (83 to 113)	48 (39 to 58)	35 (26 to 47)	7 (3 to 13)	7 (7 to 7)	198 (170 to 230)	98 (80 to 118)	72 (53 to 96)	14 (7 to 27)	14 (14 to 14)
Bolivia	1998	108 (92 to 124)	53 (44 to 64)	38 (29 to 51)	7 (3 to 13)	9 (9 to 9)	220 (188 to 253)	108 (89 to 130)	78 (58 to 103)	14 (7 to 27)	19 (19 to 19)
Bolivia	1999	110 (94 to 126)	56 (46 to 66)	40 (30 to 53)	7 (3 to 13)	8 (8 to 8)	224 (192 to 256)	113 (93 to 135)	81 (61 to 107)	14 (6 to 26)	16 (16 to 16)
Bolivia	2000	111 (97 to 126)	56 (47 to 66)	38 (28 to 49)	6 (3 to 12)	11 (11 to 11)	226 (197 to 257)	113 (95 to 135)	77 (57 to 100)	13 (6 to 24)	23 (23 to 23)

Bolivia	2001	113 (99 to 130)	59 (49 to 70)	38 (28 to 49)	7 (3 to 13)	9 (9 to 9)	231 (202 to 264)	120 (101 to 143)	77 (58 to 101)	14 (7 to 26)	19 (19 to 19)
Bolivia	2002	113 (99 to 130)	61 (51 to 72)	38 (29 to 50)	7 (3 to 13)	7 (7 to 7)	231 (202 to 265)	124 (104 to 147)	78 (59 to 101)	15 (7 to 26)	15 (15 to 15)
Bolivia	2003	116 (102 to 131)	60 (50 to 72)	38 (29 to 49)	7 (3 to 13)	11 (11 to 11)	237 (208 to 268)	123 (102 to 146)	77 (59 to 99)	14 (7 to 26)	23 (23 to 23)
Bolivia	2004	117 (103 to 133)	62 (52 to 73)	38 (29 to 48)	7 (3 to 13)	10 (10 to 10)	238 (209 to 271)	125 (106 to 148)	77 (59 to 99)	15 (7 to 26)	21 (21 to 21)
Bolivia	2005	118 (104 to 134)	65 (54 to 77)	38 (29 to 50)	7 (4 to 13)	8 (8 to 8)	241 (212 to 274)	132 (111 to 156)	78 (60 to 101)	15 (7 to 27)	15 (15 to 15)
Bolivia	2006	117 (104 to 133)	64 (54 to 75)	37 (29 to 48)	7 (3 to 12)	9 (9 to 9)	239 (212 to 271)	130 (110 to 154)	76 (58 to 97)	14 (7 to 25)	19 (19 to 19)
Bolivia	2007	123 (108 to 138)	69 (59 to 82)	38 (29 to 49)	7 (3 to 12)	8 (8 to 8)	250 (221 to 282)	141 (119 to 166)	78 (60 to 100)	14 (7 to 25)	17 (17 to 17)
Bolivia	2008	132 (116 to 149)	76 (65 to 89)	40 (31 to 51)	7 (3 to 12)	8 (8 to 8)	269 (237 to 303)	155 (132 to 182)	82 (64 to 105)	14 (7 to 25)	17 (17 to 17)
Bolivia	2009	137 (121 to 155)	81 (68 to 95)	43 (33 to 55)	7 (3 to 12)	7 (7 to 7)	280 (247 to 316)	164 (139 to 193)	88 (68 to 112)	14 (7 to 25)	13 (13 to 13)
Bolivia	2010	137 (121 to 155)	79 (67 to 92)	44 (34 to 57)	6 (3 to 11)	7 (7 to 7)	278 (247 to 316)	161 (137 to 186)	90 (70 to 115)	13 (6 to 23)	14 (14 to 14)
Bolivia	2011	151 (134 to 171)	89 (76 to 103)	48 (37 to 62)	7 (3 to 12)	7 (7 to 7)	308 (272 to 349)	181 (155 to 210)	99 (75 to 127)	14 (7 to 25)	15 (15 to 15)
Bolivia	2012	157 (138 to 178)	93 (80 to 108)	51 (39 to 65)	7 (3 to 12)	6 (6 to 6)	320 (282 to 363)	189 (163 to 220)	104 (80 to 133)	14 (7 to 25)	12 (12 to 12)
Bolivia	2013	173 (153 to 197)	105 (90 to 121)	55 (42 to 70)	7 (4 to 14)	7 (7 to 7)	353 (311 to 401)	213 (183 to 246)	112 (85 to 143)	15 (7 to 28)	14 (14 to 14)
Bolivia	2014	196 (172 to 221)	123 (105 to 142)	59 (45 to 76)	8 (4 to 16)	5 (5 to 5)	399 (350 to 451)	251 (214 to 290)	121 (92 to 155)	17 (8 to 32)	11 (11 to 11)
Bolivia	2015	211 (184 to 239)	136 (116 to 159)	61 (46 to 78)	9 (4 to 17)	5 (5 to 5)	429 (375 to 486)	278 (237 to 325)	123 (94 to 159)	18 (9 to 34)	10 (10 to 10)

Bolivia	2016	209 (181 to 238)	137 (115 to 162)	59 (45 to 77)	9 (4 to 16)	4 (4 to 4)	426 (368 to 486)	279 (235 to 330)	121 (91 to 157)	18 (9 to 33)	8 (8 to 8)
Bolivia	2017	217 (184 to 258)	144 (119 to 174)	59 (44 to 78)	9 (5 to 17)	5 (5 to 5)	443 (375 to 525)	293 (243 to 354)	120 (89 to 159)	19 (9 to 34)	11 (11 to 11)
Bolivia	2018	223 (189 to 266)	148 (123 to 179)	60 (45 to 80)	9 (5 to 17)	5 (5 to 5)	454 (385 to 542)	302 (252 to 365)	123 (91 to 163)	19 (10 to 35)	10 (10 to 10)
Bolivia	2019	228 (194 to 270)	151 (126 to 183)	62 (46 to 82)	10 (5 to 18)	5 (5 to 5)	464 (396 to 550)	308 (257 to 372)	125 (93 to 166)	20 (10 to 36)	10 (10 to 10)
Bolivia	2020	233 (199 to 278)	155 (129 to 188)	63 (47 to 83)	10 (5 to 18)	5 (5 to 5)	475 (405 to 566)	316 (264 to 382)	128 (95 to 170)	20 (10 to 38)	10 (10 to 11)
Bolivia	2021	239 (203 to 283)	160 (133 to 193)	64 (48 to 85)	10 (5 to 19)	5 (5 to 6)	486 (414 to 578)	325 (271 to 392)	130 (97 to 172)	21 (11 to 38)	10 (10 to 11)
Bolivia	2022	244 (207 to 289)	163 (136 to 197)	65 (48 to 86)	10 (5 to 19)	5 (5 to 6)	496 (422 to 590)	333 (277 to 402)	132 (98 to 174)	21 (11 to 39)	11 (10 to 12)
Bolivia	2023	248 (211 to 295)	167 (139 to 202)	65 (49 to 87)	11 (5 to 20)	5 (5 to 6)	506 (430 to 601)	341 (283 to 411)	133 (99 to 176)	22 (11 to 40)	11 (10 to 12)
Bolivia	2024	253 (215 to 302)	171 (143 to 206)	66 (49 to 87)	11 (6 to 20)	5 (5 to 6)	516 (438 to 616)	349 (291 to 420)	134 (100 to 178)	22 (11 to 41)	11 (10 to 12)
Bolivia	2025	258 (218 to 308)	175 (146 to 211)	67 (50 to 88)	11 (6 to 20)	5 (5 to 6)	526 (444 to 627)	357 (298 to 429)	136 (101 to 180)	22 (11 to 42)	11 (10 to 12)
Bolivia	2026	263 (222 to 313)	179 (150 to 216)	67 (50 to 90)	11 (6 to 21)	5 (5 to 6)	537 (452 to 639)	365 (305 to 441)	138 (103 to 182)	23 (12 to 43)	11 (9 to 12)
Bolivia	2027	269 (225 to 320)	184 (153 to 222)	68 (51 to 91)	11 (6 to 21)	5 (5 to 6)	548 (459 to 652)	375 (312 to 452)	139 (104 to 185)	23 (12 to 43)	11 (9 to 13)
Bolivia	2028	275 (231 to 329)	189 (156 to 228)	69 (52 to 92)	12 (6 to 22)	5 (5 to 6)	560 (470 to 670)	384 (319 to 464)	141 (105 to 188)	24 (12 to 44)	11 (9 to 13)
Bolivia	2029	281 (236 to 337)	194 (160 to 234)	70 (52 to 93)	12 (6 to 22)	5 (5 to 6)	573 (481 to 688)	395 (327 to 477)	143 (107 to 190)	24 (13 to 45)	11 (9 to 13)
Bolivia	2030	288 (242 to 346)	199 (165 to 242)	71 (53 to 95)	12 (6 to 23)	5 (5 to 6)	587 (493 to 705)	406 (336 to 492)	145 (108 to 193)	25 (13 to 46)	11 (9 to 13)

Bolivia	2031	295 (247 to 356)	205 (169 to 248)	73 (54 to 96)	12 (6 to 23)	5 (5 to 6)	602 (503 to 725)	417 (344 to 506)	148 (110 to 196)	25 (13 to 47)	11 (9 to 13)
Bolivia	2032	303 (252 to 364)	211 (173 to 256)	74 (55 to 98)	13 (7 to 24)	5 (5 to 7)	617 (514 to 741)	429 (353 to 521)	150 (112 to 199)	26 (13 to 48)	11 (9 to 13)
Bolivia	2033	311 (261 to 373)	217 (178 to 263)	75 (56 to 100)	13 (7 to 24)	5 (5 to 7)	633 (532 to 760)	442 (362 to 536)	153 (114 to 203)	27 (14 to 49)	11 (9 to 13)
Bolivia	2034	319 (267 to 385)	224 (183 to 272)	77 (57 to 101)	13 (7 to 25)	5 (5 to 7)	650 (544 to 785)	455 (372 to 554)	156 (116 to 207)	27 (14 to 51)	11 (9 to 14)
Bolivia	2035	328 (274 to 396)	230 (188 to 280)	78 (58 to 103)	14 (7 to 26)	6 (5 to 7)	668 (559 to 807)	469 (382 to 571)	159 (119 to 211)	28 (15 to 52)	11 (9 to 14)
Bolivia	2036	337 (282 to 407)	237 (193 to 289)	80 (60 to 106)	14 (7 to 26)	6 (5 to 7)	686 (575 to 830)	483 (394 to 589)	162 (121 to 215)	29 (15 to 54)	11 (9 to 14)
Bolivia	2037	345 (290 to 415)	244 (199 to 298)	81 (61 to 108)	15 (8 to 27)	6 (5 to 7)	704 (591 to 846)	497 (405 to 607)	165 (124 to 219)	30 (15 to 55)	11 (9 to 14)
Bolivia	2038	354 (295 to 428)	251 (204 to 307)	83 (62 to 110)	15 (8 to 28)	6 (5 to 7)	722 (601 to 872)	511 (415 to 625)	169 (126 to 223)	30 (16 to 57)	11 (9 to 14)
Bolivia	2039	363 (303 to 439)	258 (209 to 316)	84 (63 to 112)	15 (8 to 28)	6 (5 to 7)	740 (616 to 895)	526 (427 to 644)	172 (128 to 228)	31 (16 to 58)	12 (9 to 15)
Bolivia	2040	373 (310 to 453)	266 (215 to 325)	86 (64 to 114)	16 (8 to 29)	6 (5 to 7)	759 (632 to 922)	541 (438 to 662)	175 (131 to 232)	32 (16 to 60)	12 (9 to 15)
Bolivia	2041	382 (318 to 466)	273 (221 to 335)	87 (65 to 116)	16 (8 to 30)	6 (5 to 7)	779 (649 to 950)	556 (449 to 682)	178 (133 to 236)	33 (17 to 61)	12 (10 to 15)
Bolivia	2042	392 (326 to 478)	280 (226 to 344)	89 (66 to 118)	16 (8 to 31)	6 (5 to 7)	798 (665 to 973)	571 (461 to 700)	181 (135 to 240)	34 (17 to 62)	12 (9 to 15)
Bolivia	2043	401 (334 to 488)	288 (232 to 353)	91 (68 to 120)	17 (9 to 31)	6 (5 to 8)	817 (679 to 994)	586 (473 to 719)	185 (138 to 244)	34 (18 to 64)	12 (10 to 16)
Bolivia	2044	410 (338 to 496)	295 (238 to 363)	92 (69 to 122)	17 (9 to 32)	6 (5 to 8)	836 (688 to 1011)	601 (484 to 739)	188 (140 to 249)	35 (18 to 65)	12 (10 to 16)
Bolivia	2045	420 (345 to 510)	302 (243 to 372)	94 (70 to 125)	18 (9 to 33)	6 (5 to 8)	855 (702 to 1039)	616 (496 to 759)	191 (142 to 254)	36 (18 to 67)	12 (10 to 16)

Bolivia	2046	429 (354 to 519)	310 (249 to 382)	95 (71 to 127)	18 (9 to 34)	6 (5 to 8)	874 (720 to 1057)	631 (508 to 778)	194 (144 to 258)	37 (19 to 69)	12 (10 to 16)
Bolivia	2047	438 (359 to 532)	317 (254 to 390)	97 (72 to 129)	18 (9 to 34)	6 (5 to 8)	893 (732 to 1084)	645 (518 to 796)	198 (147 to 263)	38 (19 to 70)	12 (10 to 16)
Bolivia	2048	447 (366 to 542)	324 (260 to 400)	99 (73 to 131)	19 (10 to 35)	6 (5 to 8)	911 (747 to 1104)	660 (530 to 815)	201 (149 to 268)	38 (20 to 72)	12 (10 to 16)
Bolivia	2049	456 (374 to 553)	331 (266 to 408)	100 (74 to 134)	19 (10 to 36)	6 (5 to 8)	929 (762 to 1127)	674 (541 to 832)	204 (151 to 273)	39 (20 to 73)	12 (10 to 17)
Bolivia	2050	465 (378 to 560)	338 (271 to 416)	102 (75 to 136)	20 (10 to 37)	6 (5 to 8)	948 (770 to 1141)	688 (552 to 847)	207 (153 to 277)	40 (20 to 75)	13 (10 to 17)
Bosnia and Herzegovina	1995	97 (83 to 113)	37 (30 to 44)	58 (46 to 72)	2 (1 to 4)	0 (0 to 0)	242 (207 to 282)	91 (76 to 109)	144 (114 to 179)	5 (3 to 10)	1 (1 to 1)
Bosnia and Herzegovina	1996	155 (136 to 177)	62 (52 to 72)	85 (69 to 103)	3 (2 to 7)	5 (5 to 5)	387 (340 to 442)	154 (130 to 181)	211 (171 to 257)	9 (4 to 16)	13 (13 to 13)
Bosnia and Herzegovina	1997	182 (161 to 206)	76 (65 to 88)	101 (83 to 121)	4 (2 to 8)	1 (1 to 1)	455 (402 to 514)	190 (163 to 220)	251 (206 to 302)	11 (5 to 21)	3 (3 to 3)
Bosnia and Herzegovina	1998	209 (187 to 232)	87 (76 to 100)	110 (92 to 131)	5 (2 to 10)	7 (7 to 7)	522 (467 to 579)	218 (190 to 249)	273 (228 to 326)	13 (6 to 25)	18 (18 to 18)
Bosnia and Herzegovina	1999	247 (225 to 271)	109 (96 to 125)	112 (95 to 132)	6 (3 to 12)	19 (19 to 19)	616 (561 to 677)	273 (241 to 311)	280 (236 to 330)	16 (8 to 30)	48 (48 to 48)
Bosnia and Herzegovina	2000	233 (212 to 257)	120 (106 to 135)	102 (86 to 119)	7 (3 to 13)	5 (5 to 5)	581 (529 to 641)	299 (264 to 336)	253 (216 to 298)	17 (8 to 33)	11 (11 to 11)
Bosnia and Herzegovina	2001	232 (212 to 255)	126 (113 to 140)	95 (80 to 111)	7 (4 to 14)	4 (4 to 4)	578 (530 to 636)	314 (281 to 348)	236 (198 to 278)	18 (9 to 34)	9 (9 to 9)

Bosnia and Herzegovina	2002	245 (224 to 268)	138 (124 to 153)	94 (80 to 111)	8 (4 to 15)	4 (4 to 4)	610 (560 to 669)	343 (310 to 382)	236 (199 to 278)	20 (10 to 36)	11 (11 to 11)
Bosnia and Herzegovina	2003	268 (246 to 295)	153 (139 to 170)	101 (86 to 119)	9 (4 to 16)	5 (5 to 5)	669 (614 to 735)	382 (346 to 423)	253 (214 to 298)	22 (10 to 41)	12 (12 to 12)
Bosnia and Herzegovina	2004	293 (268 to 320)	162 (146 to 179)	118 (100 to 139)	10 (5 to 18)	3 (3 to 3)	730 (670 to 799)	404 (364 to 446)	294 (249 to 346)	24 (11 to 44)	7 (7 to 7)
Bosnia and Herzegovina	2005	326 (299 to 356)	176 (159 to 194)	135 (115 to 158)	11 (5 to 20)	4 (4 to 4)	812 (746 to 888)	438 (396 to 483)	336 (286 to 394)	28 (13 to 50)	10 (10 to 10)
Bosnia and Herzegovina	2006	354 (326 to 386)	191 (172 to 209)	147 (126 to 171)	12 (6 to 23)	4 (4 to 4)	883 (813 to 964)	476 (430 to 522)	366 (315 to 426)	31 (15 to 58)	10 (10 to 10)
Bosnia and Herzegovina	2007	384 (355 to 417)	211 (191 to 231)	155 (134 to 178)	14 (7 to 27)	4 (4 to 4)	958 (885 to 1039)	526 (477 to 575)	386 (334 to 444)	35 (17 to 67)	11 (11 to 11)
Bosnia and Herzegovina	2008	429 (399 to 464)	239 (220 to 261)	156 (135 to 181)	16 (8 to 30)	17 (17 to 17)	1070 (995 to 1158)	597 (548 to 650)	389 (338 to 450)	40 (19 to 76)	43 (43 to 43)
Bosnia and Herzegovina	2009	420 (391 to 455)	255 (234 to 278)	140 (122 to 161)	17 (8 to 32)	8 (8 to 8)	1048 (976 to 1135)	635 (583 to 692)	350 (304 to 402)	42 (20 to 81)	21 (21 to 21)
Bosnia and Herzegovina	2010	438 (407 to 470)	272 (251 to 294)	133 (115 to 154)	18 (8 to 35)	15 (15 to 15)	1092 (1015 to 1173)	677 (625 to 732)	332 (286 to 383)	45 (21 to 88)	37 (37 to 37)
Bosnia and Herzegovina	2011	434 (404 to 467)	278 (257 to 301)	128 (110 to 149)	18 (8 to 35)	10 (10 to 10)	1083 (1007 to 1166)	694 (641 to 752)	320 (274 to 371)	45 (21 to 87)	24 (24 to 24)

Bosnia and Herzegovina	2012	453 (421 to 488)	296 (274 to 320)	129 (110 to 149)	19 (9 to 38)	9 (9 to 9)	1131 (1050 to 1218)	739 (684 to 798)	322 (274 to 372)	48 (22 to 94)	21 (21 to 21)
Bosnia and Herzegovina	2013	463 (432 to 498)	307 (285 to 331)	130 (111 to 150)	20 (9 to 39)	7 (7 to 7)	1156 (1079 to 1242)	765 (711 to 826)	324 (276 to 375)	50 (23 to 98)	17 (17 to 17)
Bosnia and Herzegovina	2014	490 (456 to 527)	326 (303 to 352)	134 (114 to 155)	21 (10 to 41)	9 (9 to 9)	1223 (1138 to 1314)	814 (756 to 879)	334 (284 to 387)	53 (25 to 103)	22 (22 to 22)
Bosnia and Herzegovina	2015	494 (458 to 535)	334 (308 to 362)	136 (115 to 160)	22 (10 to 42)	2 (2 to 2)	1232 (1141 to 1333)	832 (769 to 902)	340 (288 to 398)	54 (26 to 105)	6 (6 to 6)
Bosnia and Herzegovina	2016	520 (478 to 568)	346 (317 to 376)	140 (116 to 166)	23 (11 to 44)	12 (12 to 12)	1297 (1192 to 1418)	862 (791 to 938)	350 (289 to 415)	57 (27 to 110)	29 (29 to 29)
Bosnia and Herzegovina	2017	531 (474 to 590)	355 (319 to 391)	144 (117 to 174)	24 (11 to 46)	9 (9 to 9)	1325 (1182 to 1471)	885 (795 to 974)	360 (292 to 434)	59 (28 to 114)	22 (22 to 22)
Bosnia and Herzegovina	2018	562 (504 to 631)	379 (341 to 416)	150 (122 to 181)	25 (12 to 48)	8 (8 to 8)	1401 (1257 to 1575)	945 (850 to 1038)	373 (303 to 451)	63 (30 to 121)	20 (20 to 20)
Bosnia and Herzegovina	2019	582 (520 to 648)	394 (354 to 434)	153 (125 to 186)	26 (13 to 50)	8 (8 to 8)	1452 (1298 to 1615)	983 (883 to 1082)	383 (312 to 464)	65 (31 to 125)	21 (21 to 21)
Bosnia and Herzegovina	2020	607 (544 to 679)	413 (371 to 455)	157 (128 to 191)	27 (13 to 52)	9 (8 to 9)	1513 (1357 to 1692)	1031 (925 to 1135)	392 (320 to 476)	68 (33 to 131)	22 (21 to 23)
Bosnia and Herzegovina	2021	628 (558 to 703)	431 (386 to 475)	160 (130 to 194)	28 (13 to 54)	9 (8 to 10)	1567 (1392 to 1752)	1075 (962 to 1184)	400 (325 to 485)	70 (34 to 135)	22 (21 to 24)

Bosnia and Herzegovina	2022	650 (582 to 729)	449 (401 to 495)	163 (132 to 197)	29 (14 to 56)	9 (8 to 10)	1622 (1451 to 1819)	1120 (1001 to 1236)	406 (330 to 492)	72 (35 to 140)	23 (21 to 25)
Bosnia and Herzegovina	2023	673 (597 to 755)	468 (417 to 516)	166 (135 to 201)	30 (14 to 58)	9 (9 to 11)	1678 (1490 to 1882)	1166 (1040 to 1287)	414 (336 to 501)	75 (36 to 145)	24 (21 to 26)
Bosnia and Herzegovina	2024	695 (618 to 782)	486 (433 to 538)	168 (137 to 204)	31 (15 to 60)	10 (9 to 11)	1733 (1542 to 1950)	1212 (1080 to 1341)	420 (341 to 508)	77 (37 to 150)	24 (22 to 27)
Bosnia and Herzegovina	2025	716 (635 to 805)	504 (449 to 559)	170 (138 to 206)	32 (15 to 62)	10 (9 to 11)	1786 (1583 to 2009)	1257 (1119 to 1393)	425 (345 to 515)	80 (38 to 154)	25 (22 to 28)
Bosnia and Herzegovina	2026	738 (657 to 832)	523 (465 to 580)	172 (140 to 209)	33 (16 to 63)	10 (9 to 12)	1841 (1639 to 2076)	1304 (1161 to 1446)	430 (349 to 521)	82 (40 to 158)	25 (22 to 29)
Bosnia and Herzegovina	2027	761 (675 to 858)	542 (482 to 603)	175 (142 to 212)	34 (16 to 65)	10 (9 to 12)	1898 (1683 to 2139)	1353 (1203 to 1503)	435 (353 to 528)	85 (41 to 162)	26 (23 to 30)
Bosnia and Herzegovina	2028	786 (698 to 886)	563 (501 to 628)	177 (143 to 215)	35 (17 to 67)	11 (9 to 12)	1960 (1741 to 2210)	1404 (1248 to 1565)	441 (358 to 536)	87 (42 to 167)	27 (23 to 31)
Bosnia and Herzegovina	2029	812 (722 to 913)	585 (519 to 651)	180 (145 to 218)	36 (17 to 69)	11 (10 to 13)	2024 (1802 to 2276)	1459 (1295 to 1625)	448 (362 to 544)	90 (44 to 171)	27 (24 to 32)
Bosnia and Herzegovina	2030	838 (741 to 953)	607 (539 to 678)	182 (147 to 221)	37 (18 to 71)	11 (10 to 13)	2090 (1848 to 2378)	1515 (1346 to 1690)	455 (368 to 552)	93 (45 to 176)	28 (24 to 33)
Bosnia and Herzegovina	2031	866 (769 to 980)	631 (561 to 702)	185 (150 to 226)	38 (19 to 73)	11 (10 to 14)	2159 (1918 to 2443)	1573 (1400 to 1752)	462 (374 to 562)	96 (47 to 181)	29 (25 to 34)

Bosnia and Herzegovina	2032	894 (789 to 1020)	654 (580 to 729)	188 (153 to 229)	40 (19 to 75)	12 (10 to 14)	2229 (1968 to 2544)	1631 (1446 to 1817)	470 (380 to 572)	99 (48 to 187)	29 (25 to 36)
Bosnia and Herzegovina	2033	923 (817 to 1042)	678 (601 to 755)	192 (155 to 233)	41 (20 to 78)	12 (10 to 15)	2301 (2037 to 2600)	1691 (1499 to 1884)	478 (386 to 581)	102 (50 to 194)	30 (26 to 37)
Bosnia and Herzegovina	2034	952 (842 to 1085)	702 (622 to 785)	195 (158 to 237)	42 (21 to 80)	12 (10 to 15)	2375 (2100 to 2706)	1752 (1551 to 1957)	487 (393 to 592)	105 (51 to 200)	31 (26 to 38)
Bosnia and Herzegovina	2035	982 (869 to 1115)	727 (644 to 816)	199 (160 to 242)	44 (21 to 82)	13 (11 to 16)	2450 (2168 to 2780)	1814 (1605 to 2034)	496 (400 to 604)	109 (53 to 205)	32 (26 to 39)
Bosnia and Herzegovina	2036	1011 (894 to 1154)	751 (665 to 843)	202 (163 to 246)	45 (22 to 84)	13 (11 to 16)	2521 (2229 to 2879)	1873 (1658 to 2104)	504 (407 to 614)	112 (54 to 210)	33 (27 to 41)
Bosnia and Herzegovina	2037	1039 (916 to 1182)	774 (685 to 870)	205 (166 to 251)	46 (22 to 86)	13 (11 to 16)	2591 (2285 to 2948)	1931 (1708 to 2170)	512 (413 to 625)	115 (56 to 216)	33 (28 to 41)
Bosnia and Herzegovina	2038	1067 (937 to 1217)	797 (705 to 899)	208 (168 to 255)	47 (23 to 89)	14 (11 to 17)	2661 (2337 to 3034)	1989 (1758 to 2242)	520 (420 to 635)	118 (57 to 221)	34 (28 to 43)
Bosnia and Herzegovina	2039	1095 (962 to 1247)	820 (724 to 926)	212 (171 to 258)	49 (24 to 91)	14 (12 to 18)	2730 (2400 to 3111)	2046 (1805 to 2310)	528 (426 to 644)	121 (59 to 227)	35 (29 to 45)
Bosnia and Herzegovina	2040	1123 (986 to 1284)	843 (743 to 951)	215 (173 to 262)	50 (24 to 93)	15 (12 to 19)	2800 (2459 to 3202)	2103 (1854 to 2373)	536 (433 to 654)	124 (60 to 233)	36 (30 to 46)
Bosnia and Herzegovina	2041	1151 (1011 to 1314)	866 (762 to 976)	218 (176 to 266)	51 (25 to 96)	15 (12 to 19)	2870 (2521 to 3277)	2161 (1902 to 2435)	544 (439 to 664)	127 (62 to 239)	37 (30 to 47)

Bosnia and Herzegovina	2042	1180 (1029 to 1345)	890 (782 to 1005)	222 (179 to 271)	52 (25 to 99)	15 (13 to 20)	2942 (2565 to 3354)	2220 (1951 to 2507)	553 (446 to 677)	131 (63 to 246)	38 (31 to 49)
Bosnia and Herzegovina	2043	1210 (1060 to 1382)	914 (803 to 1034)	226 (182 to 276)	54 (26 to 101)	16 (13 to 21)	3017 (2645 to 3446)	2280 (2004 to 2579)	563 (454 to 689)	134 (65 to 252)	39 (32 to 52)
Bosnia and Herzegovina	2044	1240 (1086 to 1425)	938 (824 to 1062)	230 (185 to 281)	55 (27 to 104)	16 (13 to 22)	3092 (2708 to 3554)	2341 (2055 to 2650)	573 (462 to 701)	137 (66 to 259)	41 (33 to 54)
Bosnia and Herzegovina	2045	1269 (1111 to 1449)	962 (844 to 1090)	234 (189 to 286)	56 (27 to 107)	17 (13 to 21)	3166 (2772 to 3615)	2400 (2106 to 2719)	583 (471 to 714)	141 (68 to 266)	42 (33 to 54)
Bosnia and Herzegovina	2046	1298 (1132 to 1491)	985 (865 to 1117)	238 (192 to 291)	58 (28 to 110)	17 (14 to 22)	3239 (2824 to 3719)	2458 (2157 to 2786)	594 (478 to 727)	144 (70 to 273)	43 (34 to 55)
Bosnia and Herzegovina	2047	1328 (1162 to 1516)	1009 (883 to 1146)	242 (195 to 297)	59 (29 to 112)	18 (14 to 23)	3312 (2897 to 3782)	2516 (2202 to 2858)	604 (486 to 741)	148 (71 to 280)	44 (35 to 58)
Bosnia and Herzegovina	2048	1357 (1186 to 1556)	1032 (902 to 1172)	247 (199 to 303)	61 (29 to 115)	18 (14 to 24)	3385 (2959 to 3881)	2574 (2251 to 2924)	615 (495 to 755)	151 (73 to 287)	45 (36 to 60)
Bosnia and Herzegovina	2049	1387 (1205 to 1593)	1055 (923 to 1194)	251 (202 to 308)	62 (30 to 118)	19 (15 to 25)	3459 (3005 to 3974)	2632 (2302 to 2978)	626 (503 to 769)	155 (74 to 294)	47 (37 to 62)
Bosnia and Herzegovina	2050	1417 (1239 to 1630)	1079 (944 to 1221)	255 (205 to 314)	63 (30 to 120)	19 (15 to 26)	3534 (3089 to 4067)	2691 (2354 to 3044)	637 (511 to 783)	158 (76 to 300)	48 (37 to 65)
Botswana	1995	218 (191 to 249)	112 (96 to 131)	28 (20 to 39)	64 (46 to 87)	14 (14 to 14)	494 (433 to 565)	254 (216 to 296)	63 (44 to 87)	145 (103 to 197)	33 (33 to 33)
Botswana	1996	212 (186 to 241)	119 (103 to 137)	28 (20 to 38)	65 (48 to 85)	1 (1 to 1)	481 (420 to 545)	269 (233 to 311)	63 (45 to 87)	146 (108 to 193)	2 (2 to 2)

Botswana	1997	226 (201 to 256)	130 (113 to 148)	29 (20 to 39)	66 (50 to 86)	1 (1 to 1)	512 (454 to 579)	295 (257 to 334)	65 (46 to 88)	150 (112 to 194)	3 (3 to 3)
Botswana	1998	227 (202 to 256)	134 (118 to 151)	28 (20 to 38)	63 (48 to 81)	1 (1 to 1)	513 (457 to 579)	304 (267 to 342)	64 (46 to 86)	143 (109 to 183)	2 (2 to 2)
Botswana	1999	235 (211 to 262)	144 (128 to 161)	29 (21 to 39)	61 (47 to 79)	1 (1 to 1)	532 (478 to 593)	326 (289 to 365)	66 (48 to 89)	139 (107 to 179)	1 (1 to 1)
Botswana	2000	244 (220 to 270)	155 (139 to 173)	30 (21 to 40)	58 (45 to 75)	1 (1 to 1)	553 (498 to 612)	351 (315 to 392)	68 (49 to 91)	132 (101 to 170)	3 (3 to 3)
Botswana	2001	261 (235 to 288)	169 (152 to 188)	30 (22 to 41)	60 (46 to 76)	1 (1 to 1)	590 (532 to 651)	383 (345 to 426)	68 (49 to 92)	136 (104 to 172)	3 (3 to 3)
Botswana	2002	294 (267 to 323)	190 (171 to 211)	31 (22 to 42)	66 (51 to 83)	8 (8 to 8)	667 (606 to 731)	430 (388 to 477)	70 (50 to 95)	149 (115 to 188)	19 (19 to 19)
Botswana	2003	301 (274 to 328)	188 (169 to 209)	30 (22 to 40)	66 (51 to 83)	17 (17 to 17)	681 (620 to 743)	425 (383 to 473)	67 (49 to 91)	149 (116 to 188)	39 (39 to 39)
Botswana	2004	299 (273 to 327)	182 (163 to 202)	28 (20 to 38)	66 (51 to 83)	24 (24 to 24)	678 (618 to 740)	411 (370 to 457)	63 (46 to 86)	150 (116 to 189)	53 (53 to 53)
Botswana	2005	273 (249 to 298)	168 (151 to 185)	25 (18 to 35)	65 (51 to 82)	15 (15 to 15)	618 (564 to 675)	380 (341 to 420)	57 (42 to 79)	146 (115 to 185)	34 (34 to 34)
Botswana	2006	289 (264 to 314)	169 (151 to 186)	24 (17 to 33)	68 (53 to 86)	28 (28 to 28)	655 (599 to 711)	383 (341 to 421)	54 (39 to 75)	154 (121 to 194)	64 (64 to 64)
Botswana	2007	321 (295 to 350)	189 (169 to 207)	23 (17 to 32)	79 (62 to 98)	31 (31 to 31)	728 (667 to 792)	427 (383 to 469)	52 (38 to 72)	178 (140 to 222)	70 (70 to 70)
Botswana	2008	500 (469 to 534)	228 (207 to 250)	23 (17 to 32)	100 (80 to 124)	148 (148 to 148)	1132 (1062 to 1210)	516 (469 to 567)	53 (38 to 73)	227 (182 to 281)	336 (336 to 336)
Botswana	2009	510 (479 to 545)	232 (211 to 254)	22 (15 to 30)	119 (97 to 145)	138 (138 to 138)	1156 (1085 to 1234)	526 (478 to 576)	49 (35 to 68)	268 (219 to 328)	312 (312 to 312)
Botswana	2010	423 (390 to 457)	232 (211 to 254)	20 (14 to 28)	119 (97 to 145)	52 (52 to 52)	957 (884 to 1034)	525 (478 to 575)	45 (32 to 62)	270 (219 to 329)	117 (117 to 117)

Botswana	2011	424 (392 to 458)	237 (216 to 259)	18 (13 to 26)	113 (93 to 137)	54 (54 to 54)	959 (888 to 1038)	537 (489 to 587)	42 (30 to 58)	257 (211 to 311)	123 (123 to 123)
Botswana	2012	433 (401 to 467)	259 (237 to 283)	18 (13 to 25)	111 (91 to 134)	45 (45 to 45)	981 (909 to 1057)	586 (537 to 642)	41 (29 to 56)	252 (206 to 304)	102 (102 to 102)
Botswana	2013	436 (404 to 471)	270 (247 to 294)	17 (12 to 23)	103 (83 to 126)	47 (47 to 47)	988 (915 to 1066)	611 (560 to 666)	39 (28 to 52)	233 (189 to 285)	105 (105 to 105)
Botswana	2014	448 (413 to 485)	296 (271 to 323)	17 (12 to 23)	101 (81 to 124)	35 (35 to 35)	1015 (936 to 1098)	671 (614 to 731)	38 (27 to 51)	228 (184 to 280)	78 (78 to 78)
Botswana	2015	443 (406 to 480)	304 (278 to 332)	16 (11 to 22)	96 (75 to 121)	27 (27 to 27)	1002 (920 to 1086)	688 (629 to 752)	36 (26 to 49)	216 (170 to 273)	61 (61 to 61)
Botswana	2016	443 (405 to 484)	305 (277 to 337)	15 (11 to 21)	89 (67 to 117)	34 (34 to 34)	1003 (918 to 1097)	691 (628 to 764)	34 (25 to 47)	201 (152 to 264)	78 (78 to 78)
Botswana	2017	449 (393 to 516)	326 (292 to 364)	15 (11 to 21)	77 (55 to 105)	31 (31 to 31)	1017 (890 to 1170)	739 (661 to 824)	34 (24 to 47)	174 (125 to 238)	71 (71 to 71)
Botswana	2018	466 (406 to 541)	342 (304 to 385)	15 (11 to 21)	80 (58 to 110)	29 (29 to 29)	1056 (919 to 1224)	774 (687 to 872)	35 (25 to 48)	182 (131 to 249)	65 (65 to 65)
Botswana	2019	486 (420 to 565)	357 (314 to 404)	16 (11 to 22)	83 (60 to 114)	29 (29 to 29)	1100 (950 to 1279)	809 (711 to 915)	36 (25 to 49)	189 (135 to 258)	67 (66 to 67)
Botswana	2020	504 (434 to 587)	372 (327 to 422)	16 (11 to 22)	86 (62 to 118)	30 (28 to 31)	1142 (984 to 1329)	843 (739 to 955)	37 (26 to 51)	195 (140 to 268)	67 (64 to 71)
Botswana	2021	522 (449 to 605)	386 (339 to 436)	17 (12 to 23)	89 (64 to 122)	30 (28 to 33)	1182 (1017 to 1370)	875 (767 to 988)	37 (26 to 52)	201 (144 to 276)	68 (64 to 74)

Botswana	2022	541 (465 to 628)	402 (352 to 454)	17 (12 to 23)	92 (66 to 126)	30 (28 to 33)	1225 (1054 to 1422)	910 (797 to 1028)	38 (27 to 53)	208 (149 to 285)	69 (63 to 76)
Botswana	2023	562 (483 to 652)	419 (367 to 474)	17 (12 to 24)	95 (68 to 130)	30 (28 to 34)	1272 (1094 to 1476)	949 (832 to 1074)	39 (27 to 54)	215 (155 to 295)	69 (62 to 77)
Botswana	2024	583 (502 to 677)	437 (383 to 494)	18 (12 to 24)	98 (70 to 134)	31 (27 to 34)	1320 (1137 to 1534)	990 (867 to 1118)	40 (28 to 55)	221 (159 to 303)	69 (62 to 78)
Botswana	2025	606 (522 to 701)	457 (401 to 516)	18 (13 to 25)	100 (72 to 137)	31 (27 to 35)	1373 (1182 to 1586)	1036 (907 to 1170)	41 (28 to 56)	227 (163 to 311)	69 (62 to 79)
Botswana	2026	629 (541 to 730)	477 (418 to 540)	18 (13 to 25)	103 (74 to 141)	31 (27 to 35)	1425 (1226 to 1654)	1081 (947 to 1222)	41 (29 to 57)	233 (168 to 319)	70 (62 to 79)
Botswana	2027	656 (562 to 759)	500 (437 to 566)	19 (13 to 26)	106 (76 to 144)	31 (27 to 36)	1485 (1273 to 1719)	1133 (990 to 1282)	42 (30 to 58)	239 (172 to 327)	70 (62 to 81)
Botswana	2028	683 (587 to 792)	524 (458 to 594)	19 (13 to 26)	109 (78 to 148)	31 (27 to 36)	1546 (1329 to 1793)	1187 (1037 to 1345)	43 (30 to 59)	246 (177 to 336)	71 (62 to 82)
Botswana	2029	710 (610 to 826)	548 (478 to 622)	19 (14 to 27)	111 (80 to 152)	31 (27 to 37)	1609 (1380 to 1871)	1242 (1083 to 1409)	44 (31 to 60)	252 (182 to 344)	71 (62 to 83)
Botswana	2030	738 (633 to 859)	572 (501 to 651)	20 (14 to 27)	114 (83 to 156)	32 (27 to 38)	1671 (1433 to 1946)	1296 (1134 to 1474)	45 (31 to 61)	259 (187 to 353)	71 (61 to 86)
Botswana	2031	766 (656 to 890)	597 (522 to 678)	20 (14 to 28)	117 (85 to 160)	32 (27 to 38)	1735 (1485 to 2015)	1352 (1182 to 1536)	45 (32 to 63)	266 (192 to 362)	72 (62 to 86)

Botswana	2032	796 (686 to 927)	623 (545 to 713)	20 (14 to 28)	121 (87 to 164)	32 (27 to 39)	1804 (1554 to 2099)	1412 (1233 to 1614)	46 (32 to 64)	273 (197 to 372)	73 (62 to 88)
Botswana	2033	827 (712 to 968)	650 (568 to 744)	21 (15 to 29)	124 (89 to 169)	32 (28 to 39)	1873 (1612 to 2193)	1472 (1287 to 1685)	47 (33 to 65)	280 (202 to 382)	73 (62 to 89)
Botswana	2034	858 (738 to 1001)	677 (593 to 777)	21 (15 to 29)	127 (92 to 173)	33 (27 to 40)	1943 (1670 to 2267)	1534 (1344 to 1759)	48 (34 to 66)	287 (208 to 392)	74 (62 to 90)
Botswana	2035	890 (764 to 1041)	706 (616 to 809)	22 (15 to 30)	130 (94 to 178)	33 (28 to 40)	2016 (1730 to 2358)	1598 (1395 to 1833)	49 (34 to 67)	295 (213 to 403)	75 (62 to 91)
Botswana	2036	922 (791 to 1082)	734 (639 to 844)	22 (15 to 30)	134 (97 to 182)	33 (28 to 42)	2089 (1791 to 2450)	1661 (1448 to 1912)	50 (35 to 69)	302 (219 to 412)	75 (62 to 94)
Botswana	2037	956 (819 to 1122)	763 (664 to 880)	22 (16 to 31)	137 (99 to 186)	34 (28 to 41)	2166 (1856 to 2540)	1729 (1504 to 1993)	51 (35 to 70)	310 (224 to 422)	76 (63 to 93)
Botswana	2038	991 (845 to 1163)	794 (689 to 917)	23 (16 to 32)	140 (102 to 191)	34 (28 to 43)	2244 (1914 to 2634)	1798 (1559 to 2078)	52 (36 to 71)	318 (230 to 432)	77 (63 to 97)
Botswana	2039	1026 (876 to 1205)	825 (716 to 954)	23 (16 to 32)	144 (104 to 195)	34 (28 to 43)	2323 (1983 to 2728)	1868 (1621 to 2161)	52 (36 to 73)	325 (235 to 441)	77 (63 to 98)
Botswana	2040	1061 (908 to 1246)	857 (743 to 991)	24 (16 to 33)	147 (106 to 200)	34 (28 to 44)	2403 (2055 to 2821)	1940 (1683 to 2244)	53 (37 to 74)	332 (240 to 453)	78 (63 to 99)
Botswana	2041	1097 (935 to 1294)	889 (770 to 1027)	24 (16 to 33)	150 (109 to 205)	35 (28 to 44)	2485 (2118 to 2930)	2013 (1745 to 2326)	54 (37 to 75)	340 (246 to 464)	78 (63 to 100)

Botswana	2042	1133 (961 to 1335)	921 (798 to 1064)	24 (17 to 34)	153 (111 to 209)	35 (27 to 45)	2566 (2177 to 3022)	2085 (1808 to 2409)	55 (38 to 76)	347 (251 to 473)	78 (61 to 102)
Botswana	2043	1169 (998 to 1376)	953 (827 to 1103)	25 (17 to 34)	157 (113 to 213)	34 (0 to 47)	2647 (2259 to 3117)	2158 (1873 to 2498)	56 (38 to 78)	355 (255 to 483)	78 (0 to 106)
Botswana	2044	1205 (1024 to 1415)	986 (856 to 1138)	25 (17 to 35)	160 (115 to 217)	34 (0 to 48)	2728 (2319 to 3203)	2233 (1939 to 2576)	56 (39 to 79)	362 (261 to 492)	77 (0 to 108)
Botswana	2045	1240 (1058 to 1465)	1018 (884 to 1174)	25 (17 to 35)	163 (117 to 221)	34 (0 to 47)	2809 (2396 to 3318)	2305 (2003 to 2658)	57 (39 to 80)	369 (266 to 501)	78 (0 to 107)
Botswana	2046	1275 (1084 to 1501)	1050 (911 to 1209)	26 (18 to 36)	166 (120 to 226)	34 (0 to 48)	2888 (2454 to 3400)	2377 (2064 to 2738)	58 (40 to 81)	376 (271 to 512)	77 (0 to 108)
Botswana	2047	1310 (1108 to 1541)	1081 (941 to 1245)	26 (18 to 36)	169 (122 to 231)	34 (0 to 49)	2967 (2509 to 3489)	2449 (2130 to 2819)	59 (40 to 82)	383 (277 to 523)	76 (0 to 111)
Botswana	2048	1344 (1142 to 1581)	1112 (967 to 1279)	26 (18 to 37)	172 (124 to 235)	34 (0 to 51)	3043 (2586 to 3581)	2517 (2191 to 2897)	59 (41 to 84)	390 (282 to 532)	76 (0 to 115)
Botswana	2049	1376 (1170 to 1618)	1141 (992 to 1311)	26 (18 to 37)	175 (126 to 238)	33 (0 to 51)	3115 (2650 to 3663)	2583 (2247 to 2970)	60 (41 to 85)	396 (286 to 540)	76 (0 to 116)
Botswana	2050	1407 (1201 to 1650)	1169 (1017 to 1344)	27 (18 to 38)	178 (128 to 242)	33 (0 to 51)	3186 (2719 to 3737)	2648 (2303 to 3043)	60 (41 to 86)	403 (291 to 549)	75 (0 to 117)
Brazil	1995	538 (492 to 587)	222 (198 to 249)	210 (181 to 244)	105 (82 to 134)	1 (1 to 1)	1012 (927 to 1105)	418 (373 to 469)	396 (340 to 460)	197 (154 to 252)	2 (2 to 2)

Brazil	1996	564 (524 to 605)	235 (213 to 258)	216 (189 to 243)	112 (93 to 136)	1 (1 to 1)	1062 (988 to 1139)	442 (401 to 486)	407 (357 to 458)	210 (175 to 255)	2 (2 to 2)
Brazil	1997	568 (531 to 604)	237 (216 to 258)	216 (193 to 241)	113 (95 to 134)	1 (1 to 1)	1069 (1001 to 1137)	446 (407 to 485)	407 (363 to 454)	213 (180 to 252)	3 (3 to 3)
Brazil	1998	576 (540 to 612)	241 (220 to 262)	218 (195 to 241)	116 (99 to 138)	1 (1 to 1)	1084 (1017 to 1153)	453 (415 to 493)	410 (368 to 454)	219 (186 to 259)	2 (2 to 2)
Brazil	1999	574 (540 to 611)	239 (219 to 260)	217 (194 to 240)	117 (99 to 138)	1 (1 to 1)	1081 (1017 to 1151)	450 (413 to 489)	408 (366 to 453)	220 (187 to 260)	3 (3 to 3)
Brazil	2000	566 (533 to 602)	234 (215 to 255)	214 (191 to 237)	117 (100 to 136)	1 (1 to 1)	1067 (1004 to 1133)	441 (405 to 480)	404 (360 to 447)	220 (188 to 257)	2 (2 to 2)
Brazil	2001	585 (550 to 620)	243 (223 to 264)	218 (196 to 242)	122 (104 to 143)	2 (2 to 2)	1101 (1036 to 1167)	457 (420 to 497)	411 (368 to 455)	230 (196 to 269)	3 (3 to 3)
Brazil	2002	606 (570 to 643)	256 (234 to 278)	221 (198 to 245)	128 (109 to 149)	1 (1 to 1)	1142 (1074 to 1211)	482 (440 to 524)	416 (372 to 462)	242 (206 to 280)	2 (2 to 2)
Brazil	2003	610 (573 to 646)	259 (237 to 282)	218 (195 to 242)	131 (111 to 151)	3 (3 to 3)	1149 (1079 to 1217)	488 (447 to 532)	410 (368 to 456)	246 (210 to 284)	5 (5 to 5)
Brazil	2004	600 (565 to 636)	254 (233 to 277)	215 (192 to 237)	131 (112 to 151)	1 (1 to 1)	1130 (1064 to 1197)	479 (438 to 522)	404 (362 to 446)	246 (211 to 285)	1 (1 to 1)
Brazil	2005	620 (583 to 658)	263 (242 to 286)	217 (195 to 240)	139 (120 to 162)	1 (1 to 1)	1167 (1098 to 1239)	495 (455 to 538)	408 (367 to 453)	261 (226 to 304)	2 (2 to 2)

Brazil	2006	637 (600 to 676)	272 (251 to 295)	217 (195 to 241)	147 (128 to 170)	1 (1 to 1)	1201 (1131 to 1273)	513 (473 to 555)	409 (367 to 454)	277 (241 to 321)	1 (1 to 1)
Brazil	2007	654 (617 to 693)	280 (258 to 303)	216 (194 to 239)	157 (137 to 179)	1 (1 to 1)	1231 (1163 to 1305)	528 (485 to 571)	407 (365 to 450)	295 (257 to 337)	1 (1 to 1)
Brazil	2008	676 (638 to 717)	294 (269 to 317)	214 (193 to 237)	167 (145 to 193)	1 (1 to 1)	1273 (1201 to 1351)	553 (507 to 596)	404 (364 to 447)	315 (274 to 363)	1 (1 to 1)
Brazil	2009	673 (634 to 712)	294 (271 to 318)	210 (189 to 235)	168 (146 to 192)	1 (1 to 1)	1267 (1194 to 1341)	554 (510 to 598)	395 (355 to 442)	316 (274 to 362)	2 (2 to 2)
Brazil	2010	718 (673 to 759)	319 (294 to 345)	213 (191 to 238)	184 (159 to 209)	2 (2 to 2)	1352 (1268 to 1430)	602 (553 to 649)	402 (359 to 448)	346 (300 to 393)	3 (3 to 3)
Brazil	2011	718 (673 to 760)	319 (295 to 346)	211 (189 to 236)	187 (163 to 213)	1 (1 to 1)	1353 (1267 to 1430)	602 (556 to 651)	397 (355 to 444)	352 (307 to 402)	2 (2 to 2)
Brazil	2012	720 (677 to 760)	318 (294 to 345)	210 (188 to 234)	191 (168 to 218)	1 (1 to 1)	1355 (1275 to 1431)	599 (554 to 650)	395 (354 to 441)	360 (317 to 411)	2 (2 to 2)
Brazil	2013	747 (703 to 789)	329 (305 to 356)	213 (191 to 238)	203 (179 to 231)	1 (1 to 1)	1406 (1325 to 1485)	620 (573 to 670)	401 (359 to 448)	382 (337 to 436)	3 (3 to 3)
Brazil	2014	774 (732 to 819)	339 (315 to 366)	218 (195 to 246)	216 (190 to 246)	1 (1 to 1)	1458 (1379 to 1543)	638 (592 to 690)	411 (367 to 462)	407 (358 to 462)	2 (2 to 2)
Brazil	2015	785 (740 to 832)	339 (315 to 364)	220 (196 to 249)	225 (198 to 253)	0 (0 to 0)	1478 (1394 to 1567)	639 (594 to 685)	415 (368 to 470)	424 (374 to 477)	1 (1 to 1)

Brazil	2016	789 (739 to 837)	337 (310 to 365)	219 (194 to 250)	233 (204 to 265)	1 (1 to 1)	1486 (1392 to 1576)	635 (584 to 687)	412 (365 to 470)	438 (384 to 499)	1 (1 to 1)
Brazil	2017	799 (766 to 834)	337 (304 to 372)	220 (189 to 255)	241 (204 to 284)	1 (1 to 1)	1505 (1443 to 1570)	634 (573 to 701)	414 (356 to 479)	455 (384 to 535)	1 (1 to 1)
Brazil	2018	811 (778 to 846)	345 (311 to 381)	221 (189 to 255)	245 (207 to 289)	1 (1 to 1)	1528 (1465 to 1594)	650 (585 to 718)	415 (357 to 480)	462 (389 to 543)	1 (1 to 1)
Brazil	2019	815 (783 to 850)	347 (311 to 384)	221 (190 to 255)	247 (208 to 290)	1 (1 to 1)	1535 (1474 to 1601)	654 (586 to 723)	416 (357 to 481)	464 (391 to 547)	1 (1 to 1)
Brazil	2020	831 (797 to 866)	355 (319 to 392)	223 (192 to 258)	252 (212 to 296)	1 (1 to 1)	1565 (1501 to 1631)	669 (600 to 738)	421 (361 to 486)	474 (399 to 558)	1 (1 to 1)
Brazil	2021	840 (808 to 876)	360 (323 to 399)	224 (192 to 259)	255 (215 to 300)	1 (1 to 1)	1583 (1521 to 1650)	679 (609 to 751)	422 (362 to 488)	480 (404 to 565)	1 (1 to 2)
Brazil	2022	850 (815 to 886)	365 (328 to 405)	225 (193 to 260)	258 (217 to 304)	1 (1 to 1)	1600 (1535 to 1668)	688 (618 to 762)	424 (364 to 490)	486 (409 to 573)	1 (1 to 2)
Brazil	2023	859 (826 to 895)	371 (332 to 412)	226 (194 to 261)	261 (219 to 308)	1 (1 to 1)	1617 (1555 to 1685)	698 (626 to 776)	426 (365 to 492)	492 (413 to 580)	2 (1 to 2)
Brazil	2024	870 (836 to 905)	377 (339 to 419)	227 (194 to 261)	265 (222 to 312)	1 (1 to 1)	1638 (1575 to 1705)	710 (638 to 789)	427 (366 to 492)	499 (419 to 588)	2 (1 to 2)
Brazil	2025	880 (847 to 917)	384 (343 to 425)	227 (195 to 262)	269 (225 to 316)	1 (1 to 1)	1658 (1595 to 1728)	723 (646 to 800)	428 (367 to 493)	506 (424 to 596)	2 (1 to 2)

Brazil	2026	893 (860 to 929)	392 (351 to 434)	228 (195 to 262)	273 (229 to 322)	1 (1 to 1)	1681 (1619 to 1750)	738 (660 to 817)	429 (368 to 494)	513 (431 to 606)	2 (1 to 2)
Brazil	2027	905 (871 to 941)	400 (357 to 443)	228 (196 to 263)	277 (232 to 327)	1 (1 to 1)	1705 (1641 to 1773)	753 (673 to 835)	430 (369 to 495)	521 (437 to 616)	2 (1 to 2)
Brazil	2028	918 (884 to 952)	407 (365 to 453)	229 (196 to 264)	281 (235 to 333)	1 (1 to 1)	1728 (1665 to 1793)	767 (686 to 853)	430 (370 to 497)	529 (443 to 626)	2 (1 to 2)
Brazil	2029	930 (896 to 966)	415 (372 to 463)	229 (197 to 264)	285 (238 to 337)	1 (1 to 1)	1751 (1688 to 1819)	782 (700 to 872)	431 (370 to 498)	536 (449 to 635)	2 (1 to 2)
Brazil	2030	942 (909 to 978)	423 (378 to 473)	229 (197 to 264)	289 (241 to 342)	1 (1 to 1)	1774 (1712 to 1841)	797 (711 to 890)	432 (371 to 498)	544 (454 to 644)	2 (1 to 2)
Brazil	2031	955 (921 to 991)	431 (385 to 483)	230 (197 to 264)	293 (244 to 346)	1 (1 to 1)	1798 (1735 to 1866)	812 (725 to 909)	432 (372 to 498)	551 (459 to 652)	2 (2 to 2)
Brazil	2032	969 (935 to 1004)	440 (391 to 493)	230 (198 to 266)	297 (247 to 352)	1 (1 to 1)	1824 (1761 to 1892)	829 (737 to 929)	434 (373 to 501)	560 (465 to 663)	2 (2 to 2)
Brazil	2033	983 (949 to 1021)	449 (399 to 502)	231 (199 to 267)	302 (251 to 358)	1 (1 to 1)	1850 (1788 to 1923)	845 (751 to 946)	435 (374 to 503)	568 (473 to 673)	2 (2 to 2)
Brazil	2034	997 (963 to 1032)	457 (406 to 514)	232 (199 to 268)	306 (255 to 363)	1 (1 to 1)	1877 (1814 to 1944)	861 (765 to 968)	437 (375 to 504)	577 (481 to 684)	2 (2 to 2)
Brazil	2035	1011 (976 to 1049)	466 (413 to 524)	233 (200 to 269)	311 (259 to 369)	1 (1 to 1)	1904 (1839 to 1975)	878 (778 to 986)	439 (377 to 507)	586 (487 to 694)	2 (2 to 2)

Brazil	2036	1025 (991 to 1062)	475 (420 to 534)	234 (200 to 270)	316 (262 to 374)	1 (1 to 1)	1931 (1867 to 1999)	894 (792 to 1006)	440 (377 to 509)	594 (493 to 705)	2 (2 to 2)
Brazil	2037	1039 (1005 to 1077)	484 (428 to 545)	234 (201 to 271)	320 (265 to 380)	1 (1 to 1)	1957 (1893 to 2029)	911 (806 to 1027)	442 (379 to 510)	603 (499 to 716)	2 (2 to 2)
Brazil	2038	1054 (1020 to 1093)	493 (436 to 556)	235 (202 to 272)	325 (269 to 387)	1 (1 to 1)	1985 (1921 to 2058)	928 (821 to 1048)	443 (380 to 512)	612 (507 to 728)	2 (2 to 3)
Brazil	2039	1068 (1033 to 1108)	502 (444 to 568)	236 (203 to 273)	330 (273 to 393)	1 (1 to 1)	2012 (1946 to 2087)	945 (835 to 1069)	444 (382 to 514)	621 (515 to 740)	2 (2 to 3)
Brazil	2040	1083 (1050 to 1124)	511 (451 to 579)	237 (203 to 274)	334 (278 to 398)	1 (1 to 1)	2040 (1977 to 2116)	962 (849 to 1091)	446 (383 to 516)	630 (524 to 750)	2 (2 to 3)
Brazil	2041	1098 (1062 to 1139)	520 (459 to 591)	237 (204 to 275)	339 (282 to 404)	1 (1 to 1)	2068 (1999 to 2146)	980 (864 to 1112)	447 (384 to 518)	639 (532 to 762)	2 (2 to 3)
Brazil	2042	1113 (1079 to 1154)	530 (466 to 601)	238 (204 to 276)	344 (286 to 410)	1 (1 to 2)	2095 (2032 to 2173)	998 (879 to 1133)	448 (384 to 519)	647 (539 to 773)	2 (2 to 3)
Brazil	2043	1127 (1091 to 1168)	539 (475 to 612)	238 (204 to 277)	348 (290 to 417)	1 (1 to 2)	2123 (2055 to 2200)	1016 (894 to 1153)	449 (385 to 521)	656 (546 to 786)	2 (2 to 3)
Brazil	2044	1141 (1106 to 1185)	549 (483 to 623)	238 (205 to 277)	353 (293 to 423)	1 (1 to 2)	2150 (2083 to 2231)	1033 (909 to 1174)	449 (385 to 522)	665 (551 to 797)	2 (2 to 3)
Brazil	2045	1155 (1117 to 1197)	558 (491 to 635)	238 (205 to 277)	357 (296 to 429)	1 (1 to 2)	2176 (2104 to 2254)	1051 (924 to 1196)	449 (386 to 522)	673 (557 to 808)	2 (2 to 3)

Brazil	2046	1169 (1132 to 1213)	567 (500 to 646)	239 (205 to 278)	362 (299 to 436)	1 (1 to 2)	2202 (2132 to 2284)	1069 (941 to 1217)	449 (385 to 523)	681 (562 to 821)	2 (2 to 3)
Brazil	2047	1182 (1145 to 1225)	576 (507 to 656)	238 (204 to 278)	366 (301 to 441)	1 (1 to 2)	2226 (2157 to 2306)	1085 (955 to 1236)	449 (385 to 523)	689 (566 to 831)	3 (2 to 3)
Brazil	2048	1195 (1157 to 1242)	585 (514 to 667)	239 (204 to 278)	370 (303 to 447)	1 (1 to 2)	2251 (2180 to 2338)	1103 (969 to 1256)	449 (385 to 524)	697 (571 to 842)	3 (2 to 3)
Brazil	2049	1208 (1169 to 1252)	594 (522 to 677)	238 (204 to 278)	374 (306 to 452)	1 (1 to 2)	2275 (2201 to 2359)	1119 (983 to 1275)	449 (385 to 524)	705 (575 to 852)	3 (2 to 4)
Brazil	2050	1221 (1181 to 1266)	603 (530 to 688)	238 (204 to 278)	378 (308 to 457)	1 (1 to 2)	2299 (2225 to 2384)	1135 (999 to 1295)	449 (385 to 524)	712 (580 to 861)	3 (2 to 4)
Brunei	1995	982 (907 to 1059)	766 (700 to 835)	216 (183 to 253)	0 (0 to 0)	0 (0 to 0)	2731 (2524 to 2946)	2130 (1946 to 2323)	601 (510 to 703)	0 (0 to 1)	0 (0 to 0)
Brunei	1996	1014 (952 to 1076)	801 (743 to 854)	214 (187 to 243)	0 (0 to 0)	0 (0 to 0)	2822 (2647 to 2993)	2227 (2068 to 2375)	594 (519 to 676)	0 (0 to 1)	0 (0 to 0)
Brunei	1997	1034 (980 to 1090)	828 (780 to 876)	207 (182 to 231)	0 (0 to 0)	0 (0 to 0)	2877 (2727 to 3033)	2302 (2171 to 2437)	575 (507 to 642)	0 (0 to 1)	0 (0 to 0)
Brunei	1998	1088 (1034 to 1144)	888 (840 to 940)	199 (178 to 222)	0 (0 to 0)	0 (0 to 0)	3025 (2876 to 3183)	2471 (2336 to 2616)	554 (496 to 618)	0 (0 to 1)	0 (0 to 0)
Brunei	1999	1033 (979 to 1089)	854 (804 to 905)	179 (160 to 200)	0 (0 to 0)	0 (0 to 0)	2874 (2723 to 3029)	2375 (2237 to 2519)	499 (445 to 557)	0 (0 to 1)	0 (0 to 0)

Brunei	2000	901 (854 to 948)	747 (703 to 792)	153 (138 to 171)	0 (0 to 0)	0 (0 to 0)	2505 (2375 to 2637)	2078 (1957 to 2202)	427 (383 to 476)	1 (0 to 1)	0 (0 to 0)
Brunei	2001	852 (807 to 894)	707 (664 to 748)	145 (130 to 160)	0 (0 to 0)	0 (0 to 0)	2369 (2245 to 2488)	1965 (1848 to 2080)	403 (361 to 445)	1 (0 to 1)	0 (0 to 0)
Brunei	2002	922 (876 to 967)	773 (728 to 817)	149 (134 to 166)	0 (0 to 0)	0 (0 to 0)	2565 (2437 to 2691)	2150 (2026 to 2273)	415 (373 to 462)	0 (0 to 1)	0 (0 to 0)
Brunei	2003	802 (760 to 842)	665 (625 to 703)	137 (124 to 152)	0 (0 to 0)	0 (0 to 0)	2232 (2114 to 2343)	1850 (1738 to 1957)	382 (344 to 423)	1 (0 to 1)	0 (0 to 0)
Brunei	2004	817 (769 to 862)	684 (639 to 727)	133 (119 to 148)	0 (0 to 0)	0 (0 to 0)	2272 (2138 to 2398)	1902 (1777 to 2022)	370 (331 to 412)	0 (0 to 1)	0 (0 to 0)
Brunei	2005	732 (688 to 776)	617 (577 to 660)	115 (102 to 129)	0 (0 to 0)	0 (0 to 0)	2035 (1914 to 2160)	1715 (1604 to 1835)	319 (284 to 359)	1 (0 to 1)	0 (0 to 0)
Brunei	2006	691 (648 to 734)	592 (552 to 635)	99 (87 to 111)	0 (0 to 0)	0 (0 to 0)	1921 (1803 to 2043)	1646 (1536 to 1766)	274 (242 to 310)	1 (0 to 1)	0 (0 to 0)
Brunei	2007	674 (634 to 718)	589 (551 to 631)	85 (75 to 97)	0 (0 to 0)	0 (0 to 0)	1874 (1763 to 1997)	1637 (1532 to 1756)	236 (208 to 269)	0 (0 to 1)	0 (0 to 0)
Brunei	2008	641 (604 to 682)	569 (533 to 608)	72 (63 to 82)	0 (0 to 0)	0 (0 to 0)	1782 (1681 to 1896)	1582 (1483 to 1690)	200 (174 to 229)	1 (0 to 1)	0 (0 to 0)
Brunei	2009	696 (654 to 740)	627 (585 to 670)	68 (59 to 79)	0 (0 to 0)	0 (0 to 0)	1935 (1819 to 2058)	1745 (1627 to 1863)	190 (164 to 219)	0 (0 to 1)	0 (0 to 0)

Brunei	2010	697 (654 to 738)	635 (592 to 675)	62 (53 to 72)	0 (0 to 0)	0 (0 to 0)	1938 (1820 to 2054)	1766 (1647 to 1878)	172 (148 to 200)	0 (0 to 1)	0 (0 to 0)
Brunei	2011	616 (580 to 657)	562 (526 to 601)	54 (46 to 63)	0 (0 to 0)	0 (0 to 0)	1715 (1612 to 1828)	1564 (1463 to 1672)	150 (128 to 176)	1 (0 to 1)	0 (0 to 0)
Brunei	2012	614 (575 to 658)	563 (526 to 605)	51 (44 to 61)	0 (0 to 0)	0 (0 to 0)	1709 (1601 to 1830)	1566 (1462 to 1682)	142 (121 to 169)	0 (0 to 1)	0 (0 to 0)
Brunei	2013	619 (580 to 662)	571 (533 to 612)	48 (41 to 57)	0 (0 to 0)	0 (0 to 0)	1722 (1614 to 1841)	1588 (1482 to 1701)	134 (114 to 159)	0 (0 to 1)	0 (0 to 0)
Brunei	2014	624 (584 to 667)	581 (542 to 622)	43 (36 to 51)	0 (0 to 0)	0 (0 to 0)	1735 (1626 to 1855)	1615 (1507 to 1731)	119 (101 to 143)	0 (0 to 1)	0 (0 to 0)
Brunei	2015	682 (641 to 727)	642 (599 to 686)	40 (33 to 49)	0 (0 to 0)	0 (0 to 0)	1898 (1783 to 2022)	1786 (1667 to 1908)	111 (92 to 135)	0 (0 to 1)	0 (0 to 0)
Brunei	2016	697 (651 to 746)	659 (614 to 705)	38 (31 to 47)	0 (0 to 0)	0 (0 to 0)	1940 (1811 to 2076)	1834 (1709 to 1962)	106 (85 to 130)	0 (0 to 1)	0 (0 to 0)
Brunei	2017	690 (634 to 750)	653 (599 to 711)	37 (29 to 45)	0 (0 to 0)	0 (0 to 0)	1919 (1764 to 2085)	1816 (1665 to 1978)	102 (81 to 126)	0 (0 to 1)	0 (0 to 0)
Brunei	2018	620 (565 to 678)	584 (530 to 642)	36 (28 to 44)	0 (0 to 0)	0 (0 to 0)	1723 (1572 to 1885)	1623 (1475 to 1785)	100 (79 to 124)	0 (0 to 1)	0 (0 to 0)
Brunei	2019	691 (622 to 761)	655 (588 to 723)	36 (29 to 45)	0 (0 to 0)	0 (0 to 0)	1922 (1731 to 2116)	1822 (1636 to 2010)	100 (80 to 124)	0 (0 to 1)	0 (0 to 0)

Brunei	2020	721 (645 to 802)	683 (608 to 763)	37 (30 to 46)	0 (0 to 0)	0 (0 to 0)	2004 (1794 to 2231)	1900 (1691 to 2123)	104 (82 to 128)	0 (0 to 1)	0 (0 to 0)
Brunei	2021	731 (645 to 822)	693 (606 to 781)	37 (30 to 46)	0 (0 to 0)	0 (0 to 0)	2033 (1793 to 2285)	1928 (1685 to 2172)	104 (82 to 129)	0 (0 to 1)	0 (0 to 0)
Brunei	2022	738 (647 to 832)	701 (610 to 794)	37 (30 to 46)	0 (0 to 0)	0 (0 to 0)	2054 (1800 to 2315)	1949 (1697 to 2208)	104 (82 to 129)	0 (0 to 1)	0 (0 to 0)
Brunei	2023	743 (640 to 845)	706 (602 to 807)	37 (29 to 46)	0 (0 to 0)	0 (0 to 0)	2067 (1781 to 2352)	1963 (1675 to 2246)	104 (82 to 129)	0 (0 to 1)	0 (0 to 0)
Brunei	2024	748 (643 to 857)	710 (606 to 819)	37 (29 to 46)	0 (0 to 0)	0 (0 to 0)	2080 (1789 to 2383)	1976 (1686 to 2279)	104 (81 to 128)	0 (0 to 1)	0 (0 to 0)
Brunei	2025	752 (637 to 868)	714 (598 to 830)	37 (29 to 46)	0 (0 to 0)	0 (0 to 0)	2091 (1772 to 2414)	1987 (1664 to 2308)	104 (81 to 128)	0 (0 to 1)	0 (0 to 0)
Brunei	2026	756 (639 to 873)	718 (601 to 836)	37 (29 to 46)	0 (0 to 0)	0 (0 to 0)	2102 (1777 to 2430)	1998 (1672 to 2324)	103 (81 to 128)	0 (0 to 1)	0 (0 to 0)
Brunei	2027	759 (638 to 881)	722 (603 to 844)	37 (29 to 46)	0 (0 to 0)	0 (0 to 0)	2112 (1776 to 2450)	2009 (1677 to 2347)	103 (81 to 128)	1 (0 to 1)	0 (0 to 0)
Brunei	2028	761 (644 to 890)	724 (607 to 854)	37 (29 to 46)	0 (0 to 0)	0 (0 to 0)	2117 (1793 to 2476)	2014 (1688 to 2375)	103 (80 to 127)	1 (0 to 1)	0 (0 to 0)
Brunei	2029	764 (641 to 893)	727 (606 to 854)	37 (29 to 45)	0 (0 to 0)	0 (0 to 0)	2125 (1783 to 2483)	2022 (1687 to 2376)	102 (80 to 126)	1 (0 to 1)	0 (0 to 0)

Brunei	2030	766 (637 to 902)	729 (601 to 867)	37 (29 to 45)	0 (0 to 0)	0 (0 to 0)	2130 (1773 to 2509)	2027 (1671 to 2411)	102 (79 to 126)	1 (0 to 1)	0 (0 to 0)
Brunei	2031	768 (635 to 916)	731 (601 to 877)	36 (28 to 45)	0 (0 to 0)	0 (0 to 0)	2136 (1767 to 2548)	2034 (1671 to 2440)	101 (79 to 125)	1 (0 to 1)	0 (0 to 0)
Brunei	2032	769 (635 to 915)	733 (599 to 879)	36 (28 to 45)	0 (0 to 0)	0 (0 to 0)	2140 (1767 to 2545)	2038 (1667 to 2446)	101 (78 to 125)	1 (0 to 1)	0 (0 to 0)
Brunei	2033	772 (637 to 922)	736 (599 to 886)	36 (28 to 45)	0 (0 to 0)	0 (0 to 0)	2147 (1773 to 2565)	2046 (1667 to 2465)	100 (78 to 124)	1 (0 to 1)	0 (0 to 0)
Brunei	2034	773 (639 to 927)	737 (602 to 892)	36 (28 to 45)	0 (0 to 0)	0 (0 to 0)	2151 (1776 to 2578)	2050 (1674 to 2482)	100 (78 to 124)	1 (0 to 1)	0 (0 to 0)
Brunei	2035	775 (633 to 932)	739 (599 to 895)	36 (28 to 44)	0 (0 to 0)	0 (0 to 0)	2155 (1761 to 2591)	2055 (1665 to 2490)	100 (77 to 123)	1 (0 to 1)	0 (0 to 0)
Brunei	2036	776 (633 to 936)	741 (598 to 901)	36 (27 to 44)	0 (0 to 0)	0 (0 to 0)	2160 (1760 to 2603)	2060 (1663 to 2506)	99 (76 to 123)	1 (0 to 1)	0 (0 to 0)
Brunei	2037	779 (628 to 948)	743 (596 to 915)	35 (27 to 44)	0 (0 to 0)	0 (0 to 0)	2166 (1747 to 2637)	2067 (1658 to 2544)	99 (76 to 123)	1 (0 to 1)	0 (0 to 0)
Brunei	2038	783 (624 to 953)	747 (589 to 918)	35 (27 to 44)	0 (0 to 0)	0 (0 to 0)	2178 (1735 to 2650)	2079 (1640 to 2552)	98 (75 to 123)	1 (0 to 1)	0 (0 to 0)
Brunei	2039	785 (623 to 956)	750 (590 to 919)	35 (27 to 44)	0 (0 to 0)	0 (0 to 0)	2185 (1734 to 2658)	2086 (1641 to 2556)	98 (75 to 122)	1 (0 to 1)	0 (0 to 0)

Brunei	2040	788 (626 to 963)	753 (590 to 928)	35 (27 to 44)	0 (0 to 0)	0 (0 to 0)	2192 (1742 to 2680)	2094 (1642 to 2580)	98 (75 to 122)	1 (0 to 1)	0 (0 to 0)
Brunei	2041	792 (622 to 977)	756 (590 to 941)	35 (27 to 44)	0 (0 to 0)	0 (0 to 0)	2202 (1730 to 2717)	2104 (1641 to 2617)	97 (74 to 122)	1 (0 to 1)	0 (0 to 0)
Brunei	2042	795 (627 to 994)	760 (591 to 962)	35 (27 to 44)	0 (0 to 0)	0 (0 to 0)	2211 (1743 to 2764)	2113 (1643 to 2675)	97 (74 to 121)	1 (0 to 1)	0 (0 to 0)
Brunei	2043	796 (627 to 991)	761 (593 to 958)	35 (27 to 44)	0 (0 to 0)	0 (0 to 0)	2215 (1745 to 2757)	2118 (1651 to 2666)	97 (74 to 121)	1 (0 to 1)	0 (0 to 0)
Brunei	2044	798 (624 to 997)	763 (587 to 964)	35 (26 to 43)	0 (0 to 0)	0 (0 to 0)	2220 (1735 to 2773)	2123 (1633 to 2681)	96 (73 to 121)	1 (0 to 1)	0 (0 to 0)
Brunei	2045	799 (620 to 999)	765 (587 to 965)	34 (26 to 43)	0 (0 to 0)	0 (0 to 0)	2224 (1725 to 2780)	2127 (1633 to 2683)	96 (73 to 120)	1 (0 to 1)	0 (0 to 0)
Brunei	2046	801 (626 to 1012)	766 (593 to 978)	34 (26 to 43)	0 (0 to 0)	0 (0 to 0)	2228 (1742 to 2814)	2132 (1648 to 2721)	95 (72 to 120)	1 (0 to 1)	0 (0 to 0)
Brunei	2047	802 (627 to 1010)	768 (595 to 978)	34 (26 to 43)	0 (0 to 0)	0 (0 to 0)	2231 (1745 to 2810)	2135 (1654 to 2721)	95 (72 to 119)	1 (0 to 1)	0 (0 to 0)
Brunei	2048	802 (631 to 1022)	768 (598 to 989)	34 (26 to 43)	0 (0 to 0)	0 (0 to 0)	2231 (1755 to 2844)	2136 (1663 to 2751)	94 (72 to 118)	1 (0 to 1)	0 (0 to 0)
Brunei	2049	802 (623 to 1022)	768 (588 to 989)	34 (26 to 42)	0 (0 to 0)	0 (0 to 0)	2231 (1732 to 2843)	2137 (1637 to 2751)	94 (71 to 118)	1 (0 to 1)	0 (0 to 0)

Brunei	2050	802 (621 to 1025)	768 (586 to 991)	33 (25 to 42)	0 (0 to 1)	0 (0 to 0)	2231 (1728 to 2852)	2137 (1630 to 2757)	93 (71 to 117)	1 (0 to 1)	0 (0 to 0)
Bulgaria	1995	191 (171 to 214)	136 (118 to 158)	55 (43 to 68)	1 (0 to 1)	0 (0 to 0)	498 (446 to 557)	354 (306 to 411)	142 (113 to 176)	2 (1 to 3)	0 (0 to 0)
Bulgaria	1996	180 (163 to 199)	125 (111 to 141)	55 (45 to 66)	1 (0 to 1)	0 (0 to 0)	468 (424 to 516)	324 (287 to 367)	142 (116 to 172)	1 (1 to 3)	0 (0 to 0)
Bulgaria	1997	179 (163 to 195)	121 (107 to 135)	57 (47 to 67)	1 (0 to 1)	0 (0 to 0)	464 (422 to 508)	315 (279 to 351)	147 (122 to 174)	1 (1 to 2)	1 (1 to 1)
Bulgaria	1998	205 (188 to 223)	135 (121 to 150)	66 (56 to 77)	1 (0 to 1)	3 (3 to 3)	532 (488 to 579)	351 (314 to 390)	172 (146 to 201)	1 (1 to 3)	7 (7 to 7)
Bulgaria	1999	228 (209 to 248)	146 (131 to 162)	81 (69 to 93)	1 (0 to 1)	0 (0 to 0)	592 (543 to 644)	380 (342 to 422)	210 (181 to 241)	1 (1 to 3)	1 (1 to 1)
Bulgaria	2000	262 (242 to 283)	160 (145 to 177)	99 (86 to 112)	1 (0 to 1)	2 (2 to 2)	680 (628 to 736)	417 (377 to 461)	257 (224 to 292)	2 (1 to 3)	4 (4 to 4)
Bulgaria	2001	297 (275 to 321)	177 (161 to 194)	118 (104 to 133)	1 (1 to 2)	1 (1 to 1)	772 (715 to 835)	459 (418 to 505)	306 (270 to 346)	3 (1 to 6)	4 (4 to 4)
Bulgaria	2002	329 (306 to 355)	194 (178 to 213)	131 (116 to 147)	2 (1 to 4)	2 (2 to 2)	856 (795 to 924)	505 (462 to 554)	341 (302 to 383)	6 (3 to 11)	5 (5 to 5)
Bulgaria	2003	358 (333 to 384)	211 (193 to 231)	142 (125 to 159)	3 (2 to 6)	1 (1 to 1)	929 (865 to 997)	549 (503 to 600)	368 (326 to 412)	9 (5 to 17)	3 (3 to 3)
Bulgaria	2004	380 (354 to 407)	221 (203 to 241)	150 (133 to 167)	5 (2 to 8)	4 (4 to 4)	987 (921 to 1058)	574 (527 to 627)	391 (347 to 435)	12 (6 to 22)	10 (10 to 10)
Bulgaria	2005	399 (374 to 426)	229 (211 to 250)	160 (143 to 177)	6 (3 to 10)	5 (5 to 5)	1038 (973 to 1107)	595 (548 to 650)	415 (372 to 460)	15 (7 to 26)	13 (13 to 13)
Bulgaria	2006	402 (377 to 427)	226 (208 to 247)	167 (150 to 185)	6 (3 to 12)	2 (2 to 2)	1044 (979 to 1111)	589 (540 to 641)	435 (389 to 480)	16 (8 to 30)	4 (4 to 4)

Bulgaria	2007	444 (417 to 472)	245 (225 to 267)	181 (164 to 199)	7 (4 to 13)	10 (10 to 10)	1154 (1085 to 1227)	636 (584 to 694)	472 (425 to 517)	19 (10 to 35)	27 (27 to 27)
Bulgaria	2008	464 (436 to 493)	257 (235 to 278)	195 (177 to 214)	8 (4 to 15)	4 (4 to 4)	1205 (1134 to 1281)	667 (610 to 724)	507 (460 to 557)	22 (11 to 39)	9 (9 to 9)
Bulgaria	2009	499 (470 to 530)	264 (242 to 287)	208 (189 to 229)	9 (4 to 16)	19 (19 to 19)	1298 (1223 to 1378)	686 (628 to 746)	541 (491 to 594)	23 (11 to 41)	49 (49 to 49)
Bulgaria	2010	507 (476 to 540)	273 (252 to 298)	222 (202 to 244)	9 (4 to 16)	2 (2 to 2)	1317 (1237 to 1404)	710 (655 to 775)	578 (526 to 635)	23 (11 to 41)	6 (6 to 6)
Bulgaria	2011	523 (493 to 557)	276 (253 to 299)	237 (217 to 260)	8 (4 to 15)	2 (2 to 2)	1359 (1282 to 1447)	716 (659 to 778)	616 (564 to 677)	22 (11 to 40)	5 (5 to 5)
Bulgaria	2012	555 (524 to 591)	287 (266 to 311)	258 (237 to 284)	9 (4 to 16)	1 (1 to 1)	1443 (1361 to 1535)	746 (691 to 807)	671 (617 to 737)	23 (11 to 42)	3 (3 to 3)
Bulgaria	2013	599 (566 to 636)	310 (287 to 336)	280 (257 to 306)	9 (5 to 17)	1 (1 to 1)	1558 (1471 to 1652)	805 (746 to 873)	727 (668 to 794)	24 (12 to 45)	2 (2 to 2)
Bulgaria	2014	657 (620 to 696)	342 (318 to 367)	304 (279 to 331)	10 (5 to 19)	1 (1 to 1)	1707 (1612 to 1810)	889 (828 to 954)	789 (724 to 859)	27 (14 to 49)	3 (3 to 3)
Bulgaria	2015	682 (643 to 723)	352 (326 to 378)	318 (291 to 348)	11 (5 to 20)	1 (1 to 1)	1772 (1670 to 1879)	915 (846 to 984)	827 (757 to 905)	28 (14 to 51)	3 (3 to 3)
Bulgaria	2016	681 (639 to 725)	346 (317 to 375)	324 (295 to 355)	11 (5 to 20)	1 (1 to 1)	1770 (1660 to 1884)	898 (824 to 975)	841 (767 to 923)	28 (14 to 51)	3 (3 to 3)

Bulgaria	2017	713 (657 to 774)	365 (329 to 403)	336 (299 to 375)	12 (6 to 21)	1 (1 to 1)	1853 (1707 to 2012)	949 (855 to 1047)	873 (777 to 976)	30 (15 to 55)	2 (2 to 2)
Bulgaria	2018	765 (704 to 832)	404 (361 to 447)	349 (310 to 389)	12 (6 to 23)	1 (1 to 1)	1990 (1829 to 2163)	1049 (938 to 1161)	906 (806 to 1012)	32 (16 to 60)	2 (2 to 2)
Bulgaria	2019	842 (774 to 910)	463 (413 to 511)	364 (324 to 407)	14 (7 to 25)	1 (1 to 1)	2187 (2010 to 2364)	1203 (1072 to 1329)	947 (842 to 1058)	36 (18 to 66)	2 (2 to 2)
Bulgaria	2020	876 (804 to 952)	484 (432 to 535)	377 (336 to 421)	14 (7 to 26)	1 (1 to 1)	2277 (2090 to 2476)	1257 (1124 to 1392)	981 (873 to 1094)	37 (18 to 69)	2 (2 to 2)
Bulgaria	2021	896 (822 to 976)	496 (443 to 551)	384 (342 to 429)	15 (7 to 27)	1 (1 to 1)	2329 (2136 to 2536)	1289 (1152 to 1432)	999 (889 to 1114)	38 (19 to 70)	2 (2 to 2)
Bulgaria	2022	920 (845 to 1001)	513 (458 to 570)	391 (349 to 437)	15 (7 to 28)	1 (1 to 1)	2392 (2195 to 2603)	1333 (1191 to 1482)	1018 (906 to 1136)	39 (19 to 72)	2 (2 to 2)
Bulgaria	2023	946 (865 to 1033)	531 (474 to 591)	399 (355 to 445)	16 (8 to 29)	1 (1 to 1)	2460 (2247 to 2684)	1380 (1231 to 1536)	1037 (922 to 1157)	40 (20 to 75)	2 (2 to 2)
Bulgaria	2024	974 (891 to 1067)	550 (489 to 615)	407 (362 to 454)	16 (8 to 30)	1 (1 to 1)	2531 (2315 to 2773)	1430 (1271 to 1599)	1057 (941 to 1180)	42 (21 to 77)	2 (2 to 3)
Bulgaria	2025	1002 (915 to 1091)	570 (507 to 638)	415 (368 to 463)	17 (8 to 30)	1 (1 to 1)	2605 (2380 to 2835)	1482 (1317 to 1659)	1078 (957 to 1203)	43 (21 to 79)	2 (2 to 3)
Bulgaria	2026	1033 (943 to 1128)	591 (524 to 666)	424 (376 to 473)	17 (9 to 31)	1 (1 to 1)	2685 (2452 to 2933)	1537 (1361 to 1730)	1101 (978 to 1228)	44 (22 to 82)	2 (2 to 3)

Bulgaria	2027	1065 (965 to 1168)	613 (541 to 692)	433 (384 to 483)	18 (9 to 33)	1 (1 to 1)	2767 (2509 to 3037)	1594 (1406 to 1798)	1125 (999 to 1256)	46 (23 to 85)	2 (2 to 3)
Bulgaria	2028	1095 (999 to 1209)	634 (561 to 714)	442 (393 to 495)	18 (9 to 34)	1 (1 to 1)	2847 (2597 to 3143)	1647 (1457 to 1856)	1150 (1021 to 1286)	47 (24 to 87)	3 (2 to 3)
Bulgaria	2029	1128 (1020 to 1241)	655 (575 to 740)	453 (402 to 507)	19 (9 to 35)	1 (1 to 1)	2931 (2652 to 3227)	1703 (1496 to 1924)	1177 (1046 to 1318)	49 (24 to 90)	3 (2 to 3)
Bulgaria	2030	1161 (1052 to 1280)	677 (592 to 768)	464 (412 to 520)	19 (10 to 36)	1 (1 to 1)	3018 (2733 to 3327)	1759 (1539 to 1996)	1205 (1072 to 1352)	50 (25 to 93)	3 (2 to 3)
Bulgaria	2031	1197 (1085 to 1321)	702 (612 to 797)	474 (422 to 533)	20 (10 to 37)	1 (1 to 1)	3112 (2820 to 3433)	1824 (1589 to 2072)	1233 (1097 to 1384)	52 (26 to 96)	3 (2 to 3)
Bulgaria	2032	1235 (1114 to 1365)	728 (634 to 833)	485 (432 to 545)	21 (10 to 38)	1 (1 to 1)	3210 (2895 to 3549)	1892 (1647 to 2166)	1261 (1122 to 1416)	54 (27 to 100)	3 (2 to 3)
Bulgaria	2033	1272 (1151 to 1415)	754 (655 to 865)	496 (441 to 558)	21 (11 to 40)	1 (1 to 1)	3307 (2991 to 3678)	1959 (1703 to 2247)	1289 (1146 to 1451)	56 (28 to 103)	3 (2 to 4)
Bulgaria	2034	1309 (1176 to 1461)	780 (670 to 899)	506 (450 to 571)	22 (11 to 41)	1 (0 to 1)	3402 (3057 to 3797)	2026 (1742 to 2337)	1316 (1169 to 1483)	57 (29 to 106)	3 (0 to 4)
Bulgaria	2035	1347 (1209 to 1505)	806 (696 to 935)	517 (459 to 583)	23 (11 to 42)	1 (0 to 1)	3501 (3142 to 3910)	2096 (1809 to 2430)	1343 (1192 to 1515)	59 (30 to 110)	3 (0 to 4)
Bulgaria	2036	1385 (1241 to 1536)	834 (717 to 968)	527 (467 to 595)	23 (12 to 44)	1 (0 to 2)	3600 (3225 to 3991)	2167 (1864 to 2516)	1370 (1215 to 1546)	61 (30 to 113)	3 (0 to 4)

Bulgaria	2037	1423 (1269 to 1591)	861 (741 to 1002)	537 (476 to 607)	24 (12 to 45)	1 (0 to 2)	3699 (3298 to 4137)	2238 (1927 to 2605)	1397 (1237 to 1577)	62 (31 to 116)	2 (0 to 4)
Bulgaria	2038	1462 (1308 to 1632)	889 (765 to 1039)	547 (484 to 619)	25 (12 to 46)	1 (0 to 2)	3799 (3400 to 4242)	2310 (1989 to 2699)	1422 (1258 to 1608)	64 (32 to 120)	2 (0 to 4)
Bulgaria	2039	1501 (1334 to 1693)	918 (789 to 1076)	557 (492 to 631)	25 (13 to 47)	1 (0 to 2)	3902 (3467 to 4399)	2386 (2050 to 2798)	1448 (1279 to 1639)	66 (33 to 123)	2 (0 to 4)
Bulgaria	2040	1541 (1367 to 1738)	948 (807 to 1114)	567 (500 to 642)	26 (13 to 49)	1 (0 to 2)	4006 (3554 to 4518)	2463 (2097 to 2895)	1473 (1300 to 1669)	68 (34 to 126)	2 (0 to 4)
Bulgaria	2041	1581 (1411 to 1780)	977 (834 to 1153)	576 (508 to 653)	27 (13 to 50)	1 (0 to 2)	4108 (3668 to 4627)	2539 (2168 to 2997)	1498 (1319 to 1697)	70 (35 to 130)	2 (0 to 4)
Bulgaria	2042	1621 (1432 to 1827)	1006 (855 to 1187)	586 (517 to 664)	28 (14 to 51)	1 (0 to 2)	4212 (3723 to 4748)	2616 (2223 to 3086)	1523 (1343 to 1725)	72 (36 to 133)	2 (0 to 5)
Bulgaria	2043	1661 (1472 to 1884)	1036 (875 to 1222)	596 (525 to 675)	28 (14 to 53)	1 (0 to 2)	4318 (3827 to 4897)	2693 (2275 to 3177)	1549 (1364 to 1754)	73 (37 to 137)	1 (0 to 5)
Bulgaria	2044	1702 (1503 to 1938)	1066 (899 to 1263)	606 (533 to 687)	29 (15 to 54)	1 (0 to 2)	4423 (3907 to 5037)	2771 (2336 to 3282)	1576 (1386 to 1785)	75 (38 to 140)	1 (0 to 5)
Bulgaria	2045	1743 (1548 to 1961)	1096 (923 to 1301)	617 (541 to 698)	30 (15 to 55)	0 (0 to 2)	4530 (4023 to 5097)	2849 (2399 to 3383)	1602 (1406 to 1814)	77 (39 to 144)	1 (0 to 5)
Bulgaria	2046	1784 (1563 to 2037)	1126 (950 to 1335)	627 (549 to 710)	30 (15 to 57)	0 (0 to 2)	4637 (4061 to 5293)	2927 (2469 to 3470)	1629 (1427 to 1845)	79 (40 to 148)	1 (0 to 5)

Bulgaria	2047	1826 (1614 to 2087)	1157 (973 to 1372)	637 (557 to 724)	31 (16 to 58)	0 (0 to 2)	4746 (4195 to 5424)	3007 (2529 to 3565)	1656 (1447 to 1881)	81 (41 to 152)	1 (0 to 5)
Bulgaria	2048	1867 (1632 to 2119)	1188 (995 to 1406)	647 (565 to 737)	32 (16 to 60)	0 (0 to 2)	4854 (4242 to 5509)	3087 (2587 to 3656)	1683 (1468 to 1917)	83 (42 to 155)	1 (0 to 5)
Bulgaria	2049	1911 (1686 to 2178)	1220 (1019 to 1444)	658 (574 to 749)	33 (16 to 61)	0 (0 to 2)	4967 (4383 to 5660)	3170 (2648 to 3752)	1710 (1491 to 1947)	85 (43 to 159)	1 (0 to 5)
Bulgaria	2050	1955 (1709 to 2220)	1252 (1044 to 1481)	669 (582 to 761)	33 (17 to 63)	0 (0 to 2)	5081 (4441 to 5770)	3255 (2713 to 3851)	1738 (1512 to 1978)	87 (44 to 163)	1 (0 to 5)
Burkina Faso	1995	16 (14 to 19)	4 (3 to 6)	8 (5 to 11)	1 (0 to 2)	3 (3 to 3)	46 (39 to 56)	12 (9 to 16)	22 (15 to 30)	3 (1 to 5)	9 (9 to 9)
Burkina Faso	1996	14 (12 to 18)	5 (4 to 6)	8 (5 to 11)	1 (0 to 2)	1 (1 to 1)	41 (34 to 50)	13 (10 to 17)	22 (15 to 30)	3 (1 to 5)	3 (3 to 3)
Burkina Faso	1997	16 (14 to 20)	5 (4 to 6)	8 (5 to 11)	1 (0 to 2)	3 (3 to 3)	47 (39 to 57)	14 (10 to 18)	22 (15 to 30)	3 (1 to 5)	9 (9 to 9)
Burkina Faso	1998	16 (14 to 20)	5 (4 to 7)	7 (5 to 10)	1 (0 to 2)	3 (3 to 3)	47 (40 to 56)	14 (11 to 19)	21 (15 to 30)	3 (1 to 5)	9 (9 to 9)
Burkina Faso	1999	15 (13 to 18)	5 (4 to 7)	7 (5 to 10)	1 (0 to 2)	2 (2 to 2)	44 (36 to 53)	15 (11 to 20)	20 (14 to 29)	2 (1 to 5)	6 (6 to 6)
Burkina Faso	2000	14 (12 to 17)	5 (4 to 7)	6 (4 to 9)	1 (0 to 1)	2 (2 to 2)	41 (34 to 49)	14 (11 to 19)	18 (13 to 25)	2 (1 to 4)	7 (7 to 7)
Burkina Faso	2001	15 (13 to 18)	5 (4 to 7)	6 (4 to 9)	1 (0 to 1)	4 (4 to 4)	44 (37 to 51)	14 (11 to 19)	17 (12 to 24)	2 (1 to 3)	10 (10 to 10)
Burkina Faso	2002	15 (12 to 18)	5 (4 to 7)	6 (4 to 8)	1 (0 to 1)	3 (3 to 3)	43 (36 to 50)	15 (11 to 20)	17 (12 to 24)	2 (1 to 3)	9 (9 to 9)
Burkina Faso	2003	17 (14 to 20)	5 (4 to 7)	6 (4 to 8)	1 (0 to 1)	5 (5 to 5)	48 (41 to 56)	15 (11 to 21)	17 (12 to 24)	2 (1 to 3)	15 (15 to 15)

Burkina Faso	2004	20 (17 to 23)	7 (5 to 9)	6 (5 to 9)	1 (0 to 1)	6 (6 to 6)	56 (48 to 66)	19 (14 to 25)	18 (13 to 26)	2 (1 to 3)	17 (17 to 17)
Burkina Faso	2005	21 (18 to 25)	7 (5 to 9)	7 (5 to 10)	1 (0 to 1)	7 (7 to 7)	61 (52 to 71)	20 (15 to 25)	19 (14 to 28)	2 (1 to 3)	20 (20 to 20)
Burkina Faso	2006	23 (19 to 26)	8 (6 to 10)	7 (5 to 11)	1 (0 to 1)	7 (7 to 7)	65 (55 to 75)	22 (16 to 28)	21 (15 to 30)	2 (1 to 4)	20 (20 to 20)
Burkina Faso	2007	23 (20 to 27)	8 (6 to 10)	8 (5 to 11)	1 (0 to 2)	6 (6 to 6)	66 (57 to 77)	23 (17 to 29)	23 (16 to 32)	2 (1 to 4)	18 (18 to 18)
Burkina Faso	2008	24 (21 to 28)	7 (6 to 10)	8 (5 to 11)	1 (0 to 2)	8 (8 to 8)	70 (61 to 81)	21 (16 to 27)	23 (16 to 32)	2 (1 to 5)	24 (24 to 24)
Burkina Faso	2009	26 (23 to 30)	8 (6 to 11)	9 (6 to 12)	1 (1 to 2)	8 (8 to 8)	75 (65 to 87)	23 (17 to 30)	25 (17 to 35)	3 (2 to 6)	23 (23 to 23)
Burkina Faso	2010	28 (25 to 33)	8 (6 to 11)	9 (6 to 13)	1 (1 to 2)	10 (10 to 10)	81 (71 to 94)	23 (17 to 31)	26 (18 to 37)	4 (2 to 7)	28 (28 to 28)
Burkina Faso	2011	24 (21 to 29)	8 (6 to 10)	9 (6 to 13)	2 (1 to 3)	6 (6 to 6)	70 (59 to 82)	23 (17 to 30)	26 (18 to 37)	4 (2 to 8)	16 (16 to 16)
Burkina Faso	2012	29 (25 to 34)	9 (6 to 11)	10 (7 to 14)	2 (1 to 4)	8 (8 to 8)	83 (71 to 96)	25 (18 to 32)	28 (20 to 40)	6 (3 to 10)	24 (24 to 24)
Burkina Faso	2013	31 (26 to 37)	10 (7 to 13)	11 (8 to 15)	3 (1 to 5)	7 (7 to 7)	90 (76 to 105)	29 (21 to 38)	32 (22 to 44)	7 (4 to 13)	21 (21 to 21)
Burkina Faso	2014	31 (26 to 37)	10 (7 to 13)	11 (8 to 15)	3 (1 to 5)	7 (7 to 7)	89 (75 to 105)	29 (21 to 37)	32 (22 to 44)	8 (4 to 14)	21 (21 to 21)
Burkina Faso	2015	33 (28 to 39)	11 (8 to 14)	12 (8 to 16)	3 (1 to 5)	8 (8 to 8)	95 (81 to 112)	31 (23 to 40)	33 (23 to 47)	8 (4 to 15)	23 (23 to 23)
Burkina Faso	2016	36 (31 to 43)	13 (10 to 18)	13 (9 to 18)	3 (1 to 5)	8 (8 to 8)	104 (88 to 123)	38 (28 to 50)	36 (25 to 50)	8 (4 to 15)	22 (22 to 22)
Burkina Faso	2017	41 (33 to 49)	16 (12 to 21)	14 (9 to 19)	3 (1 to 6)	8 (8 to 8)	116 (95 to 141)	46 (34 to 60)	39 (27 to 54)	9 (4 to 17)	22 (22 to 22)
Burkina Faso	2018	40 (32 to 50)	16 (12 to 21)	14 (10 to 20)	3 (2 to 6)	7 (7 to 7)	115 (93 to 142)	45 (33 to 59)	41 (28 to 56)	10 (4 to 18)	20 (20 to 21)

Burkina Faso	2019	42 (34 to 51)	16 (12 to 22)	15 (10 to 20)	3 (2 to 6)	7 (7 to 7)	119 (97 to 146)	47 (35 to 62)	42 (29 to 58)	10 (5 to 18)	20 (20 to 20)
Burkina Faso	2020	43 (34 to 52)	17 (13 to 23)	15 (10 to 21)	4 (2 to 7)	7 (7 to 7)	123 (98 to 150)	49 (37 to 65)	43 (30 to 59)	10 (5 to 19)	20 (19 to 21)
Burkina Faso	2021	44 (35 to 54)	18 (13 to 23)	15 (11 to 21)	4 (2 to 7)	7 (7 to 8)	125 (102 to 155)	51 (38 to 67)	44 (30 to 60)	10 (5 to 20)	20 (19 to 22)
Burkina Faso	2022	45 (36 to 55)	18 (14 to 24)	16 (11 to 21)	4 (2 to 7)	7 (6 to 8)	128 (102 to 157)	52 (39 to 69)	44 (31 to 61)	11 (5 to 20)	20 (18 to 22)
Burkina Faso	2023	46 (37 to 56)	19 (14 to 25)	16 (11 to 22)	4 (2 to 7)	7 (6 to 8)	130 (105 to 162)	54 (40 to 71)	45 (31 to 62)	11 (5 to 21)	20 (18 to 22)
Burkina Faso	2024	47 (37 to 58)	20 (15 to 26)	16 (11 to 22)	4 (2 to 7)	7 (6 to 8)	133 (106 to 165)	56 (42 to 74)	46 (32 to 64)	11 (5 to 21)	19 (17 to 22)
Burkina Faso	2025	48 (38 to 60)	21 (15 to 27)	16 (11 to 23)	4 (2 to 8)	7 (6 to 8)	137 (110 to 170)	59 (44 to 77)	47 (33 to 65)	12 (6 to 22)	19 (17 to 22)
Burkina Faso	2026	49 (39 to 61)	21 (16 to 28)	17 (12 to 23)	4 (2 to 8)	7 (6 to 8)	140 (112 to 173)	61 (45 to 80)	48 (33 to 66)	12 (6 to 23)	19 (17 to 22)
Burkina Faso	2027	50 (40 to 63)	22 (16 to 29)	17 (12 to 24)	4 (2 to 8)	7 (6 to 8)	144 (114 to 180)	64 (47 to 84)	49 (34 to 68)	13 (6 to 24)	19 (17 to 22)
Burkina Faso	2028	52 (41 to 64)	23 (17 to 30)	17 (12 to 24)	5 (2 to 9)	6 (6 to 8)	148 (117 to 183)	66 (49 to 87)	50 (35 to 69)	13 (6 to 24)	19 (16 to 22)
Burkina Faso	2029	53 (42 to 66)	24 (18 to 32)	18 (12 to 25)	5 (2 to 9)	6 (6 to 8)	152 (122 to 189)	69 (51 to 91)	51 (35 to 71)	14 (6 to 25)	18 (16 to 22)
Burkina Faso	2030	55 (43 to 68)	25 (18 to 33)	18 (13 to 25)	5 (2 to 9)	6 (5 to 8)	156 (122 to 194)	72 (53 to 95)	52 (36 to 73)	14 (7 to 26)	18 (16 to 22)
Burkina Faso	2031	56 (44 to 70)	26 (19 to 34)	19 (13 to 26)	5 (2 to 9)	6 (5 to 7)	160 (127 to 200)	75 (55 to 99)	53 (37 to 74)	15 (7 to 27)	18 (15 to 21)
Burkina Faso	2032	58 (45 to 72)	27 (20 to 36)	19 (13 to 27)	5 (2 to 10)	6 (5 to 7)	165 (128 to 206)	78 (57 to 103)	55 (38 to 76)	15 (7 to 28)	18 (15 to 21)
Burkina Faso	2033	59 (47 to 74)	28 (21 to 37)	19 (13 to 27)	5 (3 to 10)	6 (5 to 7)	170 (134 to 213)	81 (60 to 107)	56 (39 to 78)	16 (7 to 29)	18 (15 to 21)

Burkina Faso	2034	61 (48 to 77)	30 (22 to 39)	20 (14 to 28)	6 (3 to 11)	6 (5 to 7)	175 (137 to 220)	85 (62 to 112)	57 (39 to 79)	16 (8 to 30)	17 (14 to 21)
Burkina Faso	2035	63 (50 to 79)	31 (23 to 41)	20 (14 to 28)	6 (3 to 11)	6 (5 to 7)	180 (142 to 227)	88 (65 to 116)	58 (40 to 81)	17 (8 to 31)	17 (14 to 21)
Burkina Faso	2036	65 (50 to 81)	32 (23 to 42)	21 (14 to 29)	6 (3 to 11)	6 (5 to 7)	186 (143 to 232)	92 (67 to 121)	59 (41 to 82)	17 (8 to 32)	17 (14 to 21)
Burkina Faso	2037	67 (52 to 85)	33 (24 to 44)	21 (15 to 29)	6 (3 to 12)	6 (5 to 7)	191 (150 to 245)	95 (70 to 126)	61 (42 to 84)	18 (8 to 34)	17 (14 to 21)
Burkina Faso	2038	69 (53 to 86)	35 (25 to 46)	22 (15 to 30)	6 (3 to 12)	6 (5 to 7)	197 (151 to 248)	99 (72 to 131)	62 (43 to 86)	19 (9 to 35)	17 (14 to 21)
Burkina Faso	2039	71 (55 to 91)	36 (26 to 48)	22 (15 to 31)	7 (3 to 13)	6 (5 to 7)	203 (158 to 261)	103 (75 to 136)	64 (44 to 88)	19 (9 to 36)	17 (13 to 21)
Burkina Faso	2040	73 (56 to 92)	37 (27 to 49)	23 (16 to 31)	7 (3 to 13)	6 (5 to 7)	209 (161 to 264)	107 (78 to 142)	65 (45 to 90)	20 (9 to 37)	17 (13 to 21)
Burkina Faso	2041	75 (58 to 96)	39 (28 to 51)	23 (16 to 32)	7 (3 to 13)	6 (5 to 7)	215 (167 to 275)	111 (82 to 147)	66 (46 to 92)	21 (9 to 39)	16 (13 to 21)
Burkina Faso	2042	77 (59 to 98)	40 (30 to 53)	24 (16 to 33)	7 (3 to 14)	6 (5 to 7)	221 (170 to 281)	116 (85 to 153)	68 (47 to 94)	21 (10 to 40)	16 (13 to 21)
Burkina Faso	2043	80 (62 to 103)	42 (31 to 56)	24 (17 to 34)	8 (4 to 14)	6 (4 to 7)	228 (178 to 295)	120 (88 to 159)	70 (48 to 97)	22 (10 to 41)	16 (13 to 21)
Burkina Faso	2044	82 (63 to 105)	44 (32 to 58)	25 (17 to 35)	8 (4 to 15)	6 (4 to 7)	235 (180 to 300)	125 (91 to 166)	71 (49 to 99)	23 (10 to 43)	16 (13 to 21)
Burkina Faso	2045	85 (66 to 109)	45 (33 to 60)	26 (18 to 35)	8 (4 to 15)	6 (4 to 7)	243 (188 to 312)	130 (94 to 172)	73 (51 to 102)	24 (11 to 44)	16 (13 to 21)
Burkina Faso	2046	88 (66 to 111)	47 (34 to 63)	26 (18 to 36)	9 (4 to 16)	6 (4 to 7)	250 (190 to 318)	135 (98 to 179)	75 (52 to 104)	24 (11 to 46)	16 (12 to 21)
Burkina Faso	2047	90 (70 to 117)	49 (36 to 65)	27 (18 to 37)	9 (4 to 17)	6 (4 to 7)	258 (199 to 334)	140 (102 to 186)	77 (53 to 106)	25 (11 to 47)	16 (12 to 21)
Burkina Faso	2048	93 (71 to 119)	51 (37 to 67)	27 (19 to 38)	9 (4 to 17)	5 (4 to 7)	266 (203 to 340)	146 (105 to 193)	79 (54 to 109)	26 (12 to 49)	16 (12 to 21)

Burkina Faso	2049	96 (74 to 125)	53 (38 to 70)	28 (19 to 39)	9 (4 to 18)	5 (4 to 7)	275 (211 to 357)	151 (109 to 201)	81 (56 to 112)	27 (12 to 51)	16 (12 to 21)
Burkina Faso	2050	99 (76 to 126)	55 (40 to 73)	29 (20 to 40)	10 (4 to 18)	5 (4 to 7)	284 (216 to 362)	157 (113 to 208)	83 (57 to 115)	28 (13 to 53)	16 (12 to 21)
Burundi	1995	24 (19 to 29)	7 (5 to 9)	9 (7 to 13)	5 (2 to 9)	2 (2 to 2)	56 (45 to 69)	17 (13 to 21)	22 (15 to 31)	12 (6 to 21)	5 (5 to 5)
Burundi	1996	22 (18 to 28)	7 (5 to 9)	9 (7 to 13)	5 (2 to 9)	1 (1 to 1)	52 (42 to 65)	16 (13 to 22)	22 (15 to 30)	12 (6 to 21)	3 (3 to 3)
Burundi	1997	20 (16 to 25)	6 (5 to 8)	9 (6 to 12)	4 (2 to 8)	1 (1 to 1)	48 (38 to 59)	14 (11 to 19)	20 (14 to 28)	10 (5 to 19)	3 (3 to 3)
Burundi	1998	21 (17 to 26)	6 (5 to 8)	9 (6 to 12)	5 (2 to 8)	1 (1 to 1)	49 (39 to 61)	15 (11 to 19)	21 (14 to 29)	11 (5 to 20)	3 (3 to 3)
Burundi	1999	21 (17 to 26)	6 (4 to 8)	9 (6 to 12)	5 (2 to 9)	1 (1 to 1)	49 (39 to 60)	14 (10 to 18)	21 (14 to 29)	11 (5 to 20)	3 (3 to 3)
Burundi	2000	21 (17 to 26)	6 (4 to 7)	9 (6 to 12)	5 (2 to 8)	2 (2 to 2)	49 (39 to 61)	13 (10 to 17)	21 (14 to 29)	11 (5 to 20)	4 (4 to 4)
Burundi	2001	20 (16 to 25)	6 (4 to 7)	9 (6 to 13)	4 (2 to 7)	2 (2 to 2)	47 (38 to 59)	13 (10 to 17)	21 (14 to 30)	10 (5 to 17)	4 (4 to 4)
Burundi	2002	20 (16 to 24)	5 (4 to 7)	9 (6 to 12)	4 (2 to 7)	2 (2 to 2)	46 (38 to 57)	12 (9 to 16)	20 (14 to 29)	9 (4 to 16)	5 (5 to 5)
Burundi	2003	23 (19 to 28)	6 (5 to 8)	10 (7 to 13)	4 (2 to 7)	3 (3 to 3)	54 (44 to 66)	15 (11 to 19)	22 (16 to 31)	10 (5 to 17)	7 (7 to 7)
Burundi	2004	26 (22 to 31)	7 (5 to 10)	10 (7 to 14)	4 (2 to 7)	4 (4 to 4)	61 (51 to 73)	17 (13 to 22)	24 (16 to 33)	10 (5 to 17)	10 (10 to 10)
Burundi	2005	26 (22 to 31)	7 (5 to 9)	10 (7 to 14)	4 (2 to 7)	6 (6 to 6)	62 (52 to 74)	16 (12 to 20)	23 (16 to 33)	9 (5 to 16)	14 (14 to 14)
Burundi	2006	29 (24 to 35)	7 (5 to 9)	11 (8 to 15)	4 (2 to 8)	7 (7 to 7)	68 (57 to 82)	17 (13 to 22)	26 (18 to 36)	11 (5 to 18)	15 (15 to 15)
Burundi	2007	29 (25 to 36)	7 (5 to 10)	11 (8 to 16)	5 (2 to 9)	6 (6 to 6)	69 (58 to 83)	17 (13 to 22)	26 (18 to 37)	11 (6 to 20)	14 (14 to 14)

Burundi	2008	31 (26 to 37)	7 (5 to 9)	11 (7 to 15)	5 (3 to 9)	8 (8 to 8)	73 (62 to 88)	17 (12 to 22)	26 (18 to 36)	12 (6 to 21)	19 (19 to 19)
Burundi	2009	31 (27 to 37)	7 (5 to 9)	10 (7 to 14)	5 (2 to 8)	9 (9 to 9)	72 (62 to 86)	16 (12 to 22)	24 (17 to 33)	11 (6 to 19)	21 (21 to 21)
Burundi	2010	34 (30 to 40)	8 (6 to 10)	10 (7 to 14)	5 (2 to 9)	11 (11 to 11)	80 (70 to 94)	18 (13 to 24)	23 (16 to 32)	12 (6 to 20)	27 (27 to 27)
Burundi	2011	35 (31 to 41)	9 (7 to 12)	9 (6 to 13)	5 (3 to 9)	12 (12 to 12)	83 (72 to 97)	21 (15 to 28)	21 (15 to 30)	12 (6 to 22)	28 (28 to 28)
Burundi	2012	32 (27 to 37)	8 (6 to 11)	8 (5 to 11)	5 (2 to 10)	10 (10 to 10)	74 (64 to 88)	20 (15 to 26)	19 (13 to 26)	12 (6 to 22)	24 (24 to 24)
Burundi	2013	31 (27 to 37)	8 (6 to 10)	7 (5 to 10)	5 (2 to 10)	11 (11 to 11)	73 (63 to 86)	18 (13 to 23)	17 (12 to 24)	12 (6 to 22)	26 (26 to 26)
Burundi	2014	32 (28 to 38)	8 (6 to 11)	7 (5 to 10)	5 (3 to 10)	11 (11 to 11)	76 (65 to 89)	19 (15 to 25)	18 (12 to 24)	13 (6 to 23)	26 (26 to 26)
Burundi	2015	30 (26 to 35)	8 (6 to 10)	7 (5 to 10)	5 (3 to 10)	10 (10 to 10)	70 (60 to 83)	18 (14 to 23)	17 (12 to 23)	12 (6 to 23)	23 (23 to 23)
Burundi	2016	30 (27 to 35)	6 (5 to 8)	6 (4 to 9)	5 (2 to 8)	13 (13 to 13)	71 (63 to 82)	15 (11 to 19)	15 (10 to 21)	11 (5 to 20)	31 (31 to 31)
Burundi	2017	30 (26 to 36)	6 (5 to 8)	6 (4 to 9)	5 (2 to 9)	13 (13 to 13)	70 (60 to 84)	14 (11 to 19)	15 (10 to 21)	11 (5 to 20)	30 (30 to 30)
Burundi	2018	29 (25 to 36)	6 (5 to 8)	6 (4 to 9)	5 (2 to 8)	12 (12 to 12)	68 (58 to 83)	14 (11 to 19)	15 (10 to 21)	11 (5 to 20)	28 (28 to 29)
Burundi	2019	29 (25 to 36)	6 (5 to 8)	6 (4 to 9)	4 (2 to 8)	13 (12 to 13)	69 (58 to 83)	14 (11 to 19)	14 (10 to 20)	11 (5 to 20)	29 (29 to 29)
Burundi	2020	29 (25 to 36)	6 (4 to 8)	6 (4 to 8)	4 (2 to 8)	13 (12 to 14)	69 (59 to 84)	14 (10 to 19)	14 (10 to 20)	10 (5 to 20)	31 (29 to 32)
Burundi	2021	30 (25 to 36)	6 (5 to 8)	6 (4 to 8)	4 (2 to 8)	13 (12 to 14)	70 (59 to 85)	15 (11 to 19)	14 (10 to 20)	11 (5 to 20)	31 (29 to 33)
Burundi	2022	30 (25 to 37)	6 (5 to 9)	6 (4 to 9)	5 (2 to 9)	13 (12 to 15)	71 (59 to 87)	15 (11 to 20)	14 (10 to 20)	11 (5 to 20)	31 (28 to 34)

Burundi	2023	30 (25 to 37)	6 (5 to 9)	6 (4 to 9)	5 (2 to 9)	13 (12 to 15)	71 (60 to 88)	15 (11 to 20)	14 (10 to 20)	11 (5 to 20)	31 (28 to 35)
Burundi	2024	30 (25 to 37)	6 (5 to 9)	6 (4 to 9)	5 (2 to 9)	13 (12 to 15)	71 (59 to 88)	15 (11 to 20)	14 (10 to 20)	11 (5 to 20)	31 (28 to 35)
Burundi	2025	31 (26 to 38)	7 (5 to 9)	6 (4 to 9)	5 (2 to 9)	13 (12 to 15)	72 (60 to 88)	15 (11 to 21)	14 (10 to 20)	11 (5 to 21)	31 (28 to 35)
Burundi	2026	31 (25 to 38)	7 (5 to 9)	6 (4 to 9)	5 (2 to 9)	13 (12 to 15)	72 (59 to 89)	15 (11 to 21)	14 (10 to 20)	11 (5 to 21)	31 (27 to 35)
Burundi	2027	31 (26 to 38)	7 (5 to 9)	6 (4 to 9)	5 (2 to 9)	13 (12 to 15)	72 (60 to 90)	16 (11 to 21)	14 (10 to 20)	11 (6 to 21)	31 (27 to 36)
Burundi	2028	31 (26 to 39)	7 (5 to 9)	6 (4 to 9)	5 (2 to 9)	13 (11 to 15)	73 (60 to 91)	16 (11 to 22)	15 (10 to 21)	12 (6 to 21)	31 (27 to 36)
Burundi	2029	31 (26 to 39)	7 (5 to 10)	6 (4 to 9)	5 (2 to 9)	13 (11 to 15)	73 (61 to 92)	16 (11 to 23)	15 (10 to 21)	12 (6 to 22)	31 (27 to 36)
Burundi	2030	31 (26 to 39)	7 (5 to 10)	6 (4 to 9)	5 (2 to 9)	13 (11 to 16)	74 (60 to 93)	16 (11 to 23)	15 (10 to 21)	12 (6 to 22)	31 (27 to 37)
Burundi	2031	32 (26 to 39)	7 (5 to 10)	6 (4 to 9)	5 (2 to 9)	13 (11 to 16)	74 (61 to 92)	16 (11 to 23)	15 (10 to 21)	12 (6 to 22)	31 (27 to 37)
Burundi	2032	32 (26 to 39)	7 (5 to 10)	6 (4 to 9)	5 (2 to 9)	13 (11 to 16)	74 (60 to 93)	17 (11 to 24)	15 (10 to 21)	12 (6 to 22)	32 (27 to 38)
Burundi	2033	32 (26 to 40)	7 (5 to 10)	6 (4 to 9)	5 (2 to 9)	14 (12 to 17)	75 (61 to 93)	17 (11 to 24)	14 (10 to 20)	12 (6 to 22)	32 (27 to 39)
Burundi	2034	32 (26 to 40)	7 (5 to 10)	6 (4 to 9)	5 (2 to 10)	14 (11 to 17)	75 (60 to 94)	17 (11 to 24)	14 (10 to 20)	12 (6 to 22)	32 (27 to 40)
Burundi	2035	32 (26 to 40)	7 (5 to 10)	6 (4 to 9)	5 (3 to 10)	14 (12 to 17)	76 (62 to 94)	17 (11 to 24)	14 (10 to 20)	12 (6 to 22)	33 (27 to 40)
Burundi	2036	32 (26 to 41)	7 (5 to 10)	6 (4 to 9)	5 (3 to 10)	14 (12 to 18)	76 (61 to 95)	17 (11 to 24)	14 (10 to 20)	12 (6 to 22)	33 (27 to 41)
Burundi	2037	33 (27 to 41)	7 (5 to 10)	6 (4 to 9)	5 (3 to 10)	14 (12 to 18)	77 (62 to 97)	17 (11 to 24)	14 (10 to 20)	12 (6 to 22)	34 (27 to 41)

Burundi	2038	33 (26 to 42)	7 (5 to 10)	6 (4 to 9)	5 (3 to 10)	15 (12 to 18)	77 (62 to 98)	17 (11 to 24)	14 (10 to 20)	12 (6 to 23)	34 (28 to 43)
Burundi	2039	33 (27 to 41)	7 (5 to 11)	6 (4 to 8)	5 (3 to 10)	15 (12 to 18)	77 (62 to 97)	17 (11 to 25)	14 (10 to 20)	12 (6 to 23)	34 (28 to 43)
Burundi	2040	33 (27 to 42)	7 (5 to 11)	6 (4 to 8)	5 (3 to 10)	15 (12 to 19)	78 (62 to 98)	17 (11 to 25)	14 (10 to 20)	12 (6 to 23)	35 (28 to 44)
Burundi	2041	33 (27 to 42)	7 (5 to 11)	6 (4 to 8)	5 (3 to 10)	15 (12 to 19)	78 (63 to 100)	17 (11 to 25)	14 (10 to 20)	12 (6 to 23)	35 (28 to 45)
Burundi	2042	34 (27 to 43)	7 (5 to 11)	6 (4 to 8)	5 (3 to 10)	15 (12 to 19)	79 (63 to 100)	17 (11 to 25)	14 (10 to 20)	12 (6 to 23)	36 (28 to 45)
Burundi	2043	34 (27 to 43)	7 (5 to 11)	6 (4 to 8)	5 (3 to 10)	15 (12 to 20)	79 (63 to 102)	17 (11 to 26)	14 (10 to 20)	12 (6 to 23)	36 (29 to 47)
Burundi	2044	34 (27 to 44)	7 (5 to 11)	6 (4 to 8)	5 (3 to 10)	15 (12 to 20)	80 (63 to 103)	17 (11 to 26)	14 (10 to 20)	12 (6 to 23)	36 (29 to 48)
Burundi	2045	34 (27 to 44)	7 (5 to 11)	6 (4 to 8)	5 (3 to 10)	16 (12 to 20)	80 (63 to 103)	17 (11 to 25)	14 (10 to 20)	12 (6 to 23)	37 (29 to 48)
Burundi	2046	35 (27 to 44)	7 (5 to 11)	6 (4 to 9)	5 (3 to 10)	16 (12 to 20)	81 (64 to 103)	17 (11 to 25)	14 (10 to 20)	12 (6 to 23)	37 (29 to 48)
Burundi	2047	35 (28 to 44)	7 (5 to 11)	6 (4 to 9)	5 (3 to 10)	16 (13 to 21)	82 (65 to 104)	17 (11 to 26)	14 (10 to 20)	13 (6 to 24)	37 (29 to 50)
Burundi	2048	35 (28 to 45)	8 (5 to 11)	6 (4 to 9)	5 (3 to 10)	16 (13 to 21)	82 (65 to 106)	18 (11 to 26)	14 (10 to 20)	13 (6 to 24)	38 (29 to 50)
Burundi	2049	35 (28 to 45)	8 (5 to 11)	6 (4 to 9)	5 (3 to 10)	16 (13 to 22)	83 (65 to 107)	18 (11 to 27)	14 (10 to 20)	13 (6 to 24)	38 (29 to 52)
Burundi	2050	36 (28 to 46)	8 (5 to 11)	6 (4 to 9)	5 (3 to 10)	17 (13 to 22)	84 (66 to 109)	18 (11 to 27)	14 (10 to 20)	13 (6 to 24)	39 (30 to 52)
Cambodia	1995	42 (35 to 49)	9 (7 to 12)	20 (14 to 27)	0 (0 to 1)	13 (13 to 13)	119 (100 to 140)	26 (19 to 34)	57 (41 to 76)	1 (1 to 2)	36 (36 to 36)
Cambodia	1996	38 (31 to 45)	10 (7 to 13)	21 (15 to 28)	0 (0 to 1)	7 (7 to 7)	108 (89 to 129)	28 (21 to 37)	60 (43 to 80)	1 (1 to 2)	19 (19 to 19)

Cambodia	1997	36 (29 to 43)	9 (7 to 12)	21 (15 to 28)	0 (0 to 1)	6 (6 to 6)	101 (83 to 122)	26 (19 to 34)	59 (43 to 79)	1 (0 to 2)	16 (16 to 16)
Cambodia	1998	34 (28 to 42)	9 (7 to 12)	21 (16 to 29)	0 (0 to 1)	4 (4 to 4)	97 (80 to 119)	25 (19 to 33)	61 (45 to 83)	1 (0 to 2)	10 (10 to 10)
Cambodia	1999	35 (28 to 43)	9 (7 to 11)	22 (17 to 30)	0 (0 to 1)	3 (3 to 3)	99 (81 to 122)	25 (19 to 32)	64 (47 to 87)	1 (0 to 2)	9 (9 to 9)
Cambodia	2000	36 (29 to 45)	8 (6 to 11)	23 (17 to 32)	0 (0 to 1)	4 (4 to 4)	102 (84 to 127)	23 (18 to 31)	67 (49 to 91)	1 (1 to 2)	11 (11 to 11)
Cambodia	2001	38 (31 to 47)	8 (6 to 10)	24 (18 to 33)	0 (0 to 1)	5 (5 to 5)	107 (88 to 133)	23 (17 to 29)	69 (51 to 95)	1 (1 to 2)	14 (14 to 14)
Cambodia	2002	39 (31 to 48)	9 (7 to 11)	26 (19 to 35)	0 (0 to 1)	3 (3 to 3)	111 (90 to 137)	25 (19 to 32)	75 (55 to 101)	1 (1 to 2)	10 (10 to 10)
Cambodia	2003	42 (35 to 52)	9 (7 to 12)	27 (20 to 36)	0 (0 to 1)	6 (6 to 6)	120 (100 to 147)	25 (19 to 33)	77 (56 to 103)	1 (1 to 2)	17 (17 to 17)
Cambodia	2004	43 (35 to 52)	9 (7 to 11)	27 (20 to 36)	0 (0 to 1)	7 (7 to 7)	122 (101 to 148)	25 (19 to 32)	77 (57 to 101)	1 (1 to 2)	19 (19 to 19)
Cambodia	2005	46 (38 to 54)	9 (6 to 11)	28 (20 to 36)	0 (0 to 1)	9 (9 to 9)	130 (108 to 155)	24 (18 to 31)	78 (58 to 103)	1 (1 to 2)	26 (26 to 26)
Cambodia	2006	46 (38 to 54)	9 (7 to 11)	27 (20 to 36)	1 (0 to 1)	9 (9 to 9)	130 (109 to 155)	25 (19 to 31)	78 (57 to 103)	1 (1 to 3)	26 (26 to 26)
Cambodia	2007	48 (40 to 58)	10 (7 to 12)	28 (21 to 37)	1 (0 to 1)	10 (10 to 10)	137 (115 to 164)	27 (21 to 35)	81 (60 to 106)	2 (1 to 3)	28 (28 to 28)
Cambodia	2008	53 (45 to 64)	10 (8 to 13)	32 (24 to 41)	1 (0 to 1)	11 (11 to 11)	152 (128 to 181)	30 (22 to 38)	90 (67 to 118)	2 (1 to 3)	30 (30 to 30)
Cambodia	2009	61 (51 to 73)	12 (9 to 16)	36 (27 to 48)	1 (0 to 1)	11 (11 to 11)	173 (145 to 207)	35 (27 to 45)	104 (77 to 136)	2 (1 to 3)	32 (32 to 32)
Cambodia	2010	65 (55 to 78)	13 (10 to 17)	39 (29 to 51)	1 (0 to 1)	12 (12 to 12)	186 (157 to 222)	38 (28 to 49)	111 (83 to 145)	2 (1 to 3)	35 (35 to 35)
Cambodia	2011	70 (59 to 83)	14 (10 to 18)	43 (32 to 56)	0 (0 to 1)	13 (13 to 13)	200 (168 to 238)	40 (30 to 51)	122 (90 to 159)	1 (1 to 3)	36 (36 to 36)

Cambodia	2012	71 (58 to 85)	15 (11 to 20)	46 (34 to 60)	0 (0 to 1)	9 (9 to 9)	202 (167 to 242)	44 (33 to 56)	132 (97 to 170)	1 (1 to 2)	25 (25 to 25)
Cambodia	2013	75 (61 to 89)	16 (12 to 21)	48 (35 to 62)	0 (0 to 0)	10 (10 to 10)	213 (174 to 253)	46 (35 to 60)	138 (100 to 177)	1 (0 to 1)	28 (28 to 28)
Cambodia	2014	76 (62 to 91)	17 (13 to 22)	49 (36 to 63)	0 (0 to 0)	10 (10 to 10)	217 (178 to 258)	48 (36 to 61)	139 (102 to 180)	1 (0 to 1)	30 (30 to 30)
Cambodia	2015	76 (63 to 91)	17 (13 to 22)	48 (36 to 63)	0 (0 to 1)	10 (10 to 10)	217 (178 to 260)	49 (37 to 64)	138 (102 to 179)	1 (0 to 2)	29 (29 to 29)
Cambodia	2016	79 (65 to 95)	19 (14 to 25)	50 (37 to 66)	0 (0 to 1)	10 (10 to 10)	226 (184 to 271)	54 (41 to 70)	144 (106 to 188)	1 (1 to 2)	27 (27 to 27)
Cambodia	2017	83 (67 to 101)	21 (15 to 27)	53 (39 to 70)	1 (0 to 1)	9 (9 to 9)	236 (190 to 288)	59 (44 to 76)	151 (111 to 198)	2 (1 to 3)	25 (25 to 25)
Cambodia	2018	88 (71 to 106)	23 (17 to 30)	56 (41 to 73)	1 (0 to 1)	8 (8 to 8)	250 (202 to 303)	66 (49 to 85)	159 (117 to 209)	2 (1 to 3)	23 (23 to 24)
Cambodia	2019	92 (74 to 111)	24 (18 to 31)	59 (43 to 77)	1 (0 to 1)	8 (8 to 8)	261 (212 to 317)	69 (51 to 90)	168 (123 to 220)	2 (1 to 3)	23 (23 to 24)
Cambodia	2020	96 (77 to 117)	25 (19 to 33)	62 (45 to 81)	1 (0 to 1)	8 (8 to 9)	273 (220 to 334)	72 (54 to 94)	176 (129 to 231)	2 (1 to 4)	24 (22 to 25)
Cambodia	2021	98 (79 to 120)	26 (19 to 34)	64 (47 to 84)	1 (0 to 1)	8 (8 to 9)	280 (226 to 343)	73 (55 to 96)	182 (133 to 239)	2 (1 to 4)	24 (22 to 26)
Cambodia	2022	101 (82 to 124)	26 (20 to 34)	66 (48 to 86)	1 (0 to 1)	8 (8 to 9)	288 (234 to 354)	75 (56 to 98)	187 (137 to 246)	2 (1 to 4)	24 (22 to 27)
Cambodia	2023	104 (84 to 127)	27 (20 to 36)	68 (50 to 89)	1 (0 to 1)	8 (8 to 10)	296 (240 to 363)	77 (58 to 101)	193 (142 to 253)	2 (1 to 4)	24 (22 to 27)
Cambodia	2024	107 (87 to 131)	28 (21 to 37)	70 (51 to 92)	1 (0 to 1)	8 (8 to 10)	305 (248 to 373)	80 (60 to 106)	199 (146 to 261)	2 (1 to 4)	24 (21 to 27)
Cambodia	2025	110 (89 to 134)	29 (22 to 39)	72 (53 to 94)	1 (0 to 1)	9 (8 to 10)	315 (255 to 381)	84 (62 to 111)	205 (150 to 269)	2 (1 to 4)	24 (22 to 28)
Cambodia	2026	114 (92 to 139)	30 (22 to 41)	74 (54 to 97)	1 (0 to 2)	9 (7 to 10)	324 (261 to 397)	87 (64 to 116)	211 (154 to 277)	2 (1 to 4)	24 (21 to 28)

Cambodia	2027	117 (94 to 143)	32 (23 to 42)	76 (56 to 100)	1 (0 to 2)	9 (7 to 10)	335 (268 to 408)	90 (66 to 121)	217 (159 to 286)	2 (1 to 5)	25 (21 to 29)
Cambodia	2028	121 (97 to 148)	33 (24 to 44)	79 (57 to 104)	1 (0 to 2)	9 (7 to 10)	345 (276 to 422)	94 (69 to 127)	224 (163 to 295)	2 (1 to 5)	25 (21 to 29)
Cambodia	2029	125 (101 to 153)	34 (25 to 46)	81 (59 to 107)	1 (0 to 2)	9 (8 to 10)	357 (287 to 435)	98 (72 to 132)	231 (168 to 305)	2 (1 to 5)	25 (21 to 29)
Cambodia	2030	129 (103 to 157)	36 (26 to 49)	84 (61 to 111)	1 (0 to 2)	9 (7 to 11)	368 (294 to 448)	102 (73 to 139)	239 (173 to 315)	3 (1 to 5)	25 (21 to 30)
Cambodia	2031	133 (106 to 163)	37 (27 to 50)	87 (63 to 114)	1 (0 to 2)	9 (7 to 11)	380 (302 to 465)	106 (77 to 144)	247 (179 to 326)	3 (1 to 5)	25 (21 to 31)
Cambodia	2032	138 (109 to 169)	39 (27 to 53)	89 (65 to 118)	1 (0 to 2)	9 (8 to 11)	393 (311 to 481)	110 (78 to 150)	255 (185 to 337)	3 (1 to 5)	26 (21 to 31)
Cambodia	2033	143 (114 to 175)	40 (29 to 55)	92 (67 to 122)	1 (0 to 2)	9 (8 to 11)	407 (324 to 499)	115 (82 to 158)	264 (191 to 349)	3 (1 to 6)	26 (21 to 31)
Cambodia	2034	148 (118 to 181)	42 (30 to 58)	96 (69 to 127)	1 (0 to 2)	9 (8 to 11)	421 (336 to 517)	119 (85 to 166)	272 (197 to 361)	3 (1 to 6)	26 (21 to 32)
Cambodia	2035	153 (123 to 187)	44 (31 to 61)	99 (72 to 131)	1 (1 to 2)	9 (7 to 11)	436 (349 to 532)	124 (88 to 174)	282 (204 to 373)	3 (1 to 6)	26 (21 to 32)
Cambodia	2036	158 (125 to 195)	46 (32 to 64)	102 (74 to 135)	1 (1 to 2)	9 (8 to 12)	451 (356 to 556)	130 (91 to 183)	292 (211 to 386)	3 (2 to 6)	26 (22 to 33)
Cambodia	2037	164 (131 to 203)	48 (33 to 66)	106 (76 to 140)	1 (1 to 2)	9 (8 to 12)	467 (373 to 579)	136 (95 to 189)	302 (218 to 399)	3 (2 to 7)	27 (21 to 33)
Cambodia	2038	170 (134 to 211)	50 (34 to 70)	110 (79 to 145)	1 (1 to 2)	9 (8 to 12)	484 (382 to 601)	142 (98 to 199)	312 (225 to 412)	4 (2 to 7)	27 (22 to 34)
Cambodia	2039	176 (138 to 218)	52 (36 to 74)	113 (82 to 149)	1 (1 to 2)	9 (8 to 12)	502 (394 to 621)	148 (101 to 211)	323 (233 to 426)	4 (2 to 7)	27 (22 to 35)
Cambodia	2040	182 (143 to 226)	54 (37 to 78)	117 (84 to 155)	1 (1 to 3)	10 (8 to 12)	519 (409 to 645)	155 (107 to 221)	334 (241 to 441)	4 (2 to 7)	27 (22 to 35)
Cambodia	2041	189 (147 to 233)	57 (39 to 81)	121 (87 to 160)	1 (1 to 3)	10 (8 to 13)	537 (419 to 665)	161 (111 to 231)	344 (248 to 456)	4 (2 to 8)	28 (22 to 36)

Cambodia	2042	195 (154 to 242)	59 (41 to 85)	125 (90 to 165)	1 (1 to 3)	10 (8 to 13)	556 (439 to 690)	168 (117 to 241)	356 (256 to 471)	4 (2 to 8)	28 (22 to 36)
Cambodia	2043	201 (159 to 249)	61 (43 to 89)	129 (92 to 170)	1 (1 to 3)	10 (8 to 13)	574 (454 to 709)	175 (121 to 253)	367 (264 to 486)	4 (2 to 8)	28 (22 to 37)
Cambodia	2044	208 (166 to 260)	64 (44 to 92)	133 (95 to 176)	2 (1 to 3)	10 (8 to 13)	593 (472 to 740)	182 (125 to 262)	378 (271 to 500)	4 (2 to 9)	28 (22 to 38)
Cambodia	2045	215 (170 to 266)	66 (45 to 95)	137 (98 to 181)	2 (1 to 3)	10 (8 to 13)	612 (485 to 759)	189 (129 to 272)	390 (278 to 516)	5 (2 to 9)	29 (22 to 38)
Cambodia	2046	222 (175 to 276)	69 (47 to 100)	141 (100 to 187)	2 (1 to 3)	10 (8 to 14)	632 (499 to 787)	197 (133 to 285)	402 (286 to 532)	5 (2 to 9)	29 (22 to 39)
Cambodia	2047	229 (180 to 284)	72 (49 to 104)	145 (103 to 192)	2 (1 to 3)	10 (8 to 14)	652 (513 to 809)	204 (139 to 296)	414 (294 to 548)	5 (2 to 9)	29 (22 to 40)
Cambodia	2048	236 (185 to 292)	74 (50 to 107)	149 (106 to 198)	2 (1 to 3)	10 (8 to 14)	672 (528 to 833)	212 (141 to 305)	426 (303 to 564)	5 (2 to 10)	30 (23 to 41)
Cambodia	2049	243 (191 to 303)	77 (52 to 110)	154 (109 to 204)	2 (1 to 4)	11 (8 to 15)	693 (544 to 864)	219 (148 to 314)	438 (311 to 581)	5 (2 to 10)	30 (23 to 41)
Cambodia	2050	250 (197 to 312)	80 (54 to 116)	158 (112 to 210)	2 (1 to 4)	11 (8 to 15)	714 (561 to 889)	227 (155 to 330)	450 (320 to 597)	5 (2 to 10)	31 (23 to 42)
Cameroon	1995	41 (32 to 54)	7 (5 to 9)	33 (24 to 45)	1 (0 to 2)	0 (0 to 0)	108 (84 to 141)	19 (14 to 25)	87 (63 to 118)	2 (1 to 4)	1 (1 to 1)
Cameroon	1996	48 (38 to 60)	7 (5 to 9)	35 (26 to 47)	1 (0 to 2)	5 (5 to 5)	125 (100 to 157)	19 (14 to 24)	92 (68 to 125)	2 (1 to 4)	12 (12 to 12)
Cameroon	1997	45 (36 to 57)	7 (6 to 10)	35 (26 to 47)	1 (0 to 1)	2 (2 to 2)	118 (94 to 150)	19 (15 to 25)	92 (69 to 124)	2 (1 to 4)	4 (4 to 4)
Cameroon	1998	45 (36 to 57)	8 (6 to 10)	35 (26 to 46)	1 (0 to 1)	2 (2 to 2)	119 (94 to 149)	20 (15 to 26)	92 (68 to 122)	2 (1 to 4)	5 (5 to 5)
Cameroon	1999	46 (37 to 58)	8 (6 to 11)	36 (27 to 47)	1 (0 to 2)	1 (1 to 1)	120 (97 to 152)	21 (16 to 28)	94 (70 to 123)	2 (1 to 4)	3 (3 to 3)
Cameroon	2000	47 (38 to 59)	9 (7 to 11)	37 (28 to 48)	1 (0 to 2)	1 (1 to 1)	123 (99 to 155)	22 (17 to 30)	96 (72 to 127)	2 (1 to 4)	2 (2 to 2)

Cameroon	2001	49 (40 to 62)	9 (7 to 12)	38 (28 to 50)	1 (0 to 1)	1 (1 to 1)	129 (105 to 163)	24 (19 to 32)	99 (75 to 131)	2 (1 to 4)	4 (4 to 4)
Cameroon	2002	49 (40 to 61)	10 (7 to 13)	38 (29 to 50)	1 (0 to 1)	1 (1 to 1)	129 (105 to 160)	25 (19 to 33)	99 (76 to 131)	2 (1 to 4)	2 (2 to 2)
Cameroon	2003	50 (41 to 62)	10 (7 to 13)	38 (29 to 50)	1 (0 to 1)	2 (2 to 2)	132 (108 to 163)	25 (19 to 33)	99 (75 to 131)	2 (1 to 4)	5 (5 to 5)
Cameroon	2004	51 (42 to 63)	9 (7 to 12)	38 (29 to 50)	1 (0 to 1)	3 (3 to 3)	133 (110 to 166)	24 (18 to 31)	99 (76 to 131)	2 (1 to 4)	8 (8 to 8)
Cameroon	2005	49 (40 to 61)	8 (6 to 11)	37 (28 to 48)	1 (0 to 1)	3 (3 to 3)	129 (105 to 160)	22 (17 to 29)	97 (74 to 127)	2 (1 to 3)	8 (8 to 8)
Cameroon	2006	49 (40 to 61)	8 (6 to 10)	37 (28 to 48)	1 (0 to 1)	4 (4 to 4)	130 (106 to 160)	21 (16 to 27)	97 (74 to 126)	2 (1 to 3)	10 (10 to 10)
Cameroon	2007	50 (40 to 62)	8 (6 to 10)	37 (28 to 49)	1 (0 to 1)	4 (4 to 4)	131 (105 to 163)	21 (16 to 27)	98 (73 to 128)	2 (1 to 4)	10 (10 to 10)
Cameroon	2008	52 (42 to 65)	8 (6 to 11)	39 (29 to 51)	1 (0 to 2)	4 (4 to 4)	137 (110 to 170)	22 (17 to 29)	102 (76 to 134)	2 (1 to 4)	10 (10 to 10)
Cameroon	2009	50 (40 to 62)	8 (6 to 11)	37 (27 to 49)	1 (0 to 2)	4 (4 to 4)	132 (105 to 163)	22 (17 to 29)	97 (72 to 128)	2 (1 to 4)	10 (10 to 10)
Cameroon	2010	48 (39 to 59)	9 (7 to 12)	35 (26 to 47)	1 (0 to 2)	3 (3 to 3)	126 (101 to 155)	23 (17 to 30)	93 (69 to 122)	2 (1 to 4)	8 (8 to 8)
Cameroon	2011	51 (42 to 62)	10 (7 to 13)	34 (25 to 45)	1 (1 to 2)	6 (6 to 6)	135 (110 to 162)	26 (19 to 33)	89 (67 to 117)	3 (1 to 5)	17 (17 to 17)
Cameroon	2012	50 (40 to 60)	8 (6 to 11)	36 (26 to 47)	1 (0 to 2)	5 (5 to 5)	130 (105 to 159)	22 (17 to 29)	93 (69 to 122)	3 (1 to 5)	12 (12 to 12)
Cameroon	2013	53 (43 to 65)	8 (6 to 11)	38 (28 to 51)	1 (1 to 2)	6 (6 to 6)	140 (113 to 172)	22 (17 to 29)	100 (75 to 133)	3 (1 to 6)	15 (15 to 15)
Cameroon	2014	54 (42 to 66)	8 (6 to 11)	40 (29 to 52)	1 (1 to 2)	4 (4 to 4)	140 (111 to 173)	21 (16 to 28)	104 (76 to 136)	3 (2 to 6)	12 (12 to 12)
Cameroon	2015	57 (45 to 71)	8 (6 to 11)	41 (30 to 55)	1 (1 to 2)	6 (6 to 6)	149 (119 to 187)	22 (16 to 29)	108 (78 to 144)	3 (2 to 6)	16 (16 to 16)

Cameroon	2016	56 (45 to 71)	8 (6 to 11)	42 (30 to 57)	1 (1 to 2)	5 (5 to 5)	148 (117 to 187)	22 (16 to 28)	110 (79 to 149)	4 (2 to 6)	12 (12 to 12)
Cameroon	2017	60 (48 to 75)	8 (6 to 11)	42 (30 to 57)	1 (1 to 2)	9 (9 to 9)	158 (125 to 198)	21 (16 to 28)	110 (80 to 150)	3 (2 to 6)	23 (23 to 23)
Cameroon	2018	60 (47 to 75)	8 (6 to 10)	42 (31 to 57)	1 (1 to 2)	8 (8 to 8)	157 (124 to 197)	21 (15 to 28)	111 (81 to 151)	3 (2 to 6)	22 (22 to 22)
Cameroon	2019	60 (48 to 76)	8 (6 to 11)	43 (31 to 58)	1 (1 to 2)	8 (8 to 8)	159 (126 to 199)	21 (16 to 28)	112 (81 to 153)	3 (2 to 6)	22 (22 to 22)
Cameroon	2020	61 (49 to 77)	8 (6 to 11)	43 (31 to 59)	1 (1 to 2)	8 (8 to 9)	161 (129 to 201)	21 (16 to 29)	114 (82 to 155)	4 (2 to 6)	22 (21 to 23)
Cameroon	2021	62 (49 to 78)	8 (6 to 12)	44 (32 to 60)	1 (1 to 3)	8 (8 to 9)	163 (129 to 205)	22 (16 to 30)	116 (84 to 157)	4 (2 to 7)	22 (21 to 24)
Cameroon	2022	63 (50 to 80)	9 (6 to 12)	45 (32 to 61)	1 (1 to 3)	8 (8 to 9)	166 (131 to 209)	23 (16 to 31)	118 (85 to 160)	4 (2 to 7)	22 (20 to 24)
Cameroon	2023	64 (51 to 82)	9 (6 to 12)	46 (33 to 62)	1 (1 to 3)	8 (8 to 9)	169 (133 to 215)	24 (17 to 32)	120 (87 to 163)	4 (2 to 7)	22 (20 to 25)
Cameroon	2024	66 (52 to 83)	9 (7 to 13)	47 (34 to 63)	2 (1 to 3)	8 (7 to 9)	173 (136 to 217)	24 (17 to 33)	122 (89 to 166)	4 (2 to 7)	22 (20 to 25)
Cameroon	2025	67 (53 to 84)	10 (7 to 13)	48 (35 to 65)	2 (1 to 3)	8 (7 to 9)	176 (138 to 221)	25 (18 to 35)	125 (91 to 170)	4 (2 to 7)	22 (19 to 25)
Cameroon	2026	69 (54 to 86)	10 (7 to 14)	49 (35 to 66)	2 (1 to 3)	8 (7 to 9)	180 (141 to 227)	26 (18 to 36)	128 (93 to 174)	4 (2 to 8)	22 (19 to 25)
Cameroon	2027	70 (55 to 88)	10 (7 to 14)	50 (36 to 68)	2 (1 to 3)	8 (7 to 10)	184 (144 to 231)	27 (18 to 38)	131 (95 to 178)	4 (2 to 8)	21 (19 to 25)
Cameroon	2028	72 (56 to 90)	11 (7 to 15)	51 (37 to 70)	2 (1 to 3)	8 (7 to 10)	188 (148 to 236)	28 (19 to 40)	134 (97 to 183)	4 (2 to 8)	21 (18 to 25)
Cameroon	2029	73 (57 to 92)	11 (7 to 16)	52 (38 to 71)	2 (1 to 3)	8 (7 to 10)	192 (148 to 241)	29 (20 to 41)	137 (100 to 187)	5 (2 to 8)	21 (18 to 25)
Cameroon	2030	75 (59 to 95)	12 (8 to 17)	53 (39 to 73)	2 (1 to 3)	8 (7 to 10)	196 (155 to 248)	30 (20 to 44)	140 (102 to 191)	5 (2 to 9)	21 (18 to 26)

Cameroon	2031	76 (59 to 97)	12 (8 to 18)	55 (40 to 74)	2 (1 to 3)	8 (7 to 10)	201 (156 to 255)	31 (21 to 46)	143 (104 to 195)	5 (2 to 9)	21 (17 to 25)
Cameroon	2032	78 (61 to 98)	12 (8 to 18)	56 (41 to 76)	2 (1 to 4)	8 (7 to 10)	205 (160 to 258)	32 (22 to 48)	146 (107 to 199)	5 (2 to 9)	21 (17 to 26)
Cameroon	2033	80 (62 to 100)	13 (9 to 19)	57 (41 to 78)	2 (1 to 4)	8 (7 to 10)	210 (162 to 263)	34 (23 to 50)	150 (109 to 204)	5 (2 to 9)	21 (17 to 26)
Cameroon	2034	82 (64 to 103)	13 (9 to 20)	58 (42 to 79)	2 (1 to 4)	8 (6 to 10)	214 (168 to 270)	35 (23 to 53)	153 (111 to 208)	5 (3 to 10)	21 (17 to 26)
Cameroon	2035	83 (65 to 105)	14 (9 to 21)	59 (43 to 81)	2 (1 to 4)	8 (6 to 10)	219 (171 to 276)	36 (24 to 54)	156 (113 to 212)	5 (3 to 10)	21 (16 to 26)
Cameroon	2036	85 (67 to 107)	14 (9 to 22)	60 (44 to 82)	2 (1 to 4)	8 (6 to 10)	223 (175 to 281)	38 (25 to 57)	159 (115 to 216)	6 (3 to 10)	21 (16 to 27)
Cameroon	2037	87 (68 to 109)	15 (10 to 22)	62 (45 to 84)	2 (1 to 4)	8 (6 to 10)	228 (178 to 286)	39 (25 to 59)	162 (117 to 220)	6 (3 to 11)	21 (16 to 27)
Cameroon	2038	89 (68 to 113)	15 (10 to 23)	63 (45 to 85)	2 (1 to 4)	8 (6 to 11)	233 (180 to 296)	40 (25 to 61)	165 (119 to 224)	6 (3 to 11)	21 (16 to 28)
Cameroon	2039	91 (70 to 114)	16 (10 to 24)	64 (46 to 87)	2 (1 to 4)	8 (6 to 11)	238 (184 to 300)	42 (26 to 64)	168 (122 to 228)	6 (3 to 11)	21 (15 to 28)
Cameroon	2040	93 (72 to 117)	17 (10 to 25)	65 (47 to 88)	2 (1 to 4)	8 (6 to 11)	243 (189 to 308)	44 (27 to 65)	171 (124 to 232)	6 (3 to 11)	22 (16 to 28)
Cameroon	2041	94 (74 to 120)	17 (11 to 26)	66 (48 to 90)	2 (1 to 4)	8 (6 to 11)	248 (194 to 314)	45 (28 to 68)	174 (126 to 237)	6 (3 to 12)	22 (16 to 28)
Cameroon	2042	96 (75 to 123)	18 (11 to 27)	68 (49 to 92)	2 (1 to 5)	8 (6 to 11)	253 (197 to 322)	47 (29 to 71)	178 (127 to 240)	6 (3 to 12)	22 (15 to 29)
Cameroon	2043	99 (76 to 126)	19 (12 to 28)	69 (49 to 94)	3 (1 to 5)	8 (6 to 11)	259 (201 to 330)	49 (30 to 74)	181 (130 to 246)	7 (3 to 12)	22 (15 to 29)
Cameroon	2044	101 (78 to 129)	19 (12 to 29)	70 (50 to 95)	3 (1 to 5)	8 (6 to 11)	264 (206 to 339)	51 (32 to 77)	184 (132 to 249)	7 (3 to 12)	22 (15 to 30)
Cameroon	2045	103 (79 to 132)	20 (12 to 30)	71 (51 to 96)	3 (1 to 5)	9 (6 to 12)	270 (208 to 345)	53 (33 to 80)	187 (134 to 253)	7 (3 to 13)	22 (15 to 30)

Cameroon	2046	105 (81 to 135)	21 (13 to 32)	73 (52 to 99)	3 (1 to 5)	9 (6 to 12)	275 (213 to 355)	55 (33 to 84)	191 (136 to 259)	7 (3 to 13)	23 (15 to 30)
Cameroon	2047	107 (82 to 139)	22 (13 to 33)	74 (53 to 101)	3 (1 to 5)	9 (6 to 12)	281 (216 to 366)	57 (34 to 88)	194 (138 to 265)	7 (3 to 13)	23 (15 to 32)
Cameroon	2048	109 (82 to 140)	23 (13 to 35)	75 (54 to 103)	3 (1 to 5)	9 (6 to 12)	287 (216 to 368)	59 (35 to 91)	197 (141 to 270)	7 (4 to 14)	23 (15 to 32)
Cameroon	2049	112 (85 to 143)	23 (14 to 37)	76 (54 to 105)	3 (1 to 5)	9 (6 to 12)	293 (223 to 374)	62 (38 to 96)	200 (143 to 275)	8 (4 to 14)	23 (15 to 33)
Cameroon	2050	114 (86 to 146)	24 (15 to 38)	77 (55 to 106)	3 (1 to 5)	9 (6 to 13)	299 (226 to 382)	64 (39 to 99)	203 (145 to 280)	8 (4 to 14)	24 (15 to 33)
Canada	1995	2876 (2802 to 2958)	2087 (2026 to 2151)	498 (458 to 539)	290 (259 to 327)	0 (0 to 0)	3162 (3081 to 3253)	2295 (2228 to 2365)	548 (504 to 593)	319 (285 to 360)	0 (0 to 0)
Canada	1996	2860 (2799 to 2918)	2064 (2018 to 2111)	502 (471 to 533)	294 (268 to 320)	0 (0 to 0)	3145 (3078 to 3209)	2270 (2220 to 2321)	552 (518 to 586)	323 (295 to 352)	0 (0 to 0)
Canada	1997	2918 (2860 to 2974)	2099 (2054 to 2144)	514 (486 to 542)	304 (281 to 331)	0 (0 to 0)	3208 (3145 to 3271)	2309 (2259 to 2358)	566 (535 to 596)	334 (309 to 364)	0 (0 to 0)
Canada	1998	3040 (2980 to 3098)	2193 (2147 to 2238)	527 (501 to 553)	319 (295 to 347)	0 (0 to 0)	3343 (3277 to 3406)	2412 (2361 to 2461)	580 (550 to 608)	351 (325 to 381)	0 (0 to 0)
Canada	1999	3152 (3098 to 3206)	2282 (2236 to 2324)	537 (512 to 562)	334 (312 to 358)	0 (0 to 0)	3467 (3407 to 3526)	2509 (2459 to 2556)	591 (563 to 618)	367 (343 to 394)	0 (0 to 0)
Canada	2000	3300 (3252 to 3355)	2398 (2355 to 2442)	546 (524 to 570)	356 (335 to 379)	0 (0 to 0)	3629 (3576 to 3689)	2637 (2590 to 2685)	601 (576 to 627)	391 (368 to 416)	0 (0 to 0)
Canada	2001	3457 (3402 to 3512)	2514 (2472 to 2558)	556 (533 to 580)	387 (367 to 409)	0 (0 to 0)	3802 (3741 to 3862)	2765 (2718 to 2813)	611 (586 to 638)	426 (404 to 450)	0 (0 to 0)

Canada	2002	3586 (3533 to 3639)	2608 (2567 to 2653)	565 (543 to 588)	413 (393 to 434)	0 (0 to 0)	3944 (3885 to 4002)	2868 (2822 to 2918)	621 (597 to 646)	454 (432 to 477)	0 (0 to 0)
Canada	2003	3722 (3668 to 3777)	2716 (2674 to 2760)	573 (552 to 596)	434 (410 to 456)	0 (0 to 0)	4093 (4034 to 4154)	2986 (2941 to 3036)	630 (607 to 655)	477 (451 to 502)	0 (0 to 0)
Canada	2004	3804 (3750 to 3855)	2778 (2735 to 2823)	582 (560 to 605)	443 (420 to 467)	0 (0 to 0)	4183 (4124 to 4240)	3055 (3008 to 3104)	640 (616 to 665)	488 (462 to 514)	0 (0 to 0)
Canada	2005	3912 (3858 to 3964)	2860 (2814 to 2906)	596 (575 to 619)	456 (434 to 480)	0 (0 to 0)	4302 (4243 to 4360)	3146 (3094 to 3195)	655 (632 to 681)	501 (477 to 528)	0 (0 to 0)
Canada	2006	4044 (3988 to 4099)	2962 (2916 to 3011)	610 (587 to 633)	472 (449 to 496)	0 (0 to 0)	4447 (4385 to 4508)	3258 (3207 to 3312)	670 (646 to 696)	519 (494 to 545)	0 (0 to 0)
Canada	2007	4143 (4088 to 4199)	3041 (2999 to 3088)	617 (595 to 641)	485 (461 to 509)	0 (0 to 0)	4556 (4496 to 4618)	3344 (3298 to 3396)	679 (654 to 704)	533 (507 to 560)	0 (0 to 0)
Canada	2008	4263 (4210 to 4317)	3137 (3093 to 3180)	628 (604 to 651)	498 (474 to 521)	0 (0 to 0)	4688 (4630 to 4747)	3450 (3402 to 3497)	690 (664 to 716)	548 (521 to 573)	0 (0 to 0)
Canada	2009	4474 (4411 to 4531)	3307 (3262 to 3356)	646 (622 to 671)	521 (498 to 546)	0 (0 to 0)	4920 (4851 to 4983)	3637 (3587 to 3691)	710 (684 to 738)	573 (547 to 601)	0 (0 to 0)
Canada	2010	4567 (4506 to 4624)	3384 (3337 to 3431)	653 (628 to 678)	531 (506 to 556)	0 (0 to 0)	5022 (4955 to 5085)	3721 (3669 to 3773)	718 (691 to 745)	583 (557 to 611)	0 (0 to 0)
Canada	2011	4554 (4492 to 4612)	3383 (3334 to 3428)	640 (615 to 664)	530 (505 to 555)	0 (0 to 0)	5008 (4940 to 5072)	3721 (3667 to 3770)	704 (677 to 730)	583 (555 to 610)	0 (0 to 0)

Canada	2012	4554 (4494 to 4616)	3390 (3342 to 3442)	630 (606 to 654)	534 (508 to 560)	0 (0 to 0)	5008 (4942 to 5076)	3728 (3675 to 3785)	692 (666 to 719)	587 (559 to 616)	0 (0 to 0)
Canada	2013	4569 (4508 to 4628)	3405 (3357 to 3456)	624 (602 to 647)	540 (517 to 566)	0 (0 to 0)	5025 (4958 to 5090)	3745 (3691 to 3801)	687 (662 to 712)	594 (568 to 622)	0 (0 to 0)
Canada	2014	4586 (4526 to 4648)	3415 (3368 to 3462)	628 (604 to 651)	544 (520 to 571)	0 (0 to 0)	5043 (4977 to 5112)	3755 (3704 to 3807)	690 (665 to 716)	598 (572 to 628)	0 (0 to 0)
Canada	2015	4764 (4700 to 4828)	3545 (3497 to 3593)	652 (628 to 675)	566 (540 to 592)	0 (0 to 0)	5239 (5169 to 5309)	3899 (3845 to 3951)	717 (691 to 742)	623 (594 to 652)	0 (0 to 0)
Canada	2016	4857 (4795 to 4920)	3597 (3547 to 3646)	679 (655 to 707)	581 (555 to 609)	0 (0 to 0)	5341 (5273 to 5410)	3956 (3901 to 4010)	747 (721 to 777)	639 (610 to 670)	0 (0 to 0)
Canada	2017	4919 (4840 to 5003)	3626 (3565 to 3690)	700 (668 to 736)	593 (559 to 632)	0 (0 to 0)	5410 (5323 to 5501)	3987 (3920 to 4058)	770 (735 to 810)	652 (614 to 695)	0 (0 to 0)
Canada	2018	5007 (4924 to 5092)	3698 (3631 to 3768)	706 (673 to 743)	603 (567 to 644)	0 (0 to 0)	5506 (5415 to 5600)	4067 (3993 to 4144)	776 (740 to 817)	663 (624 to 708)	0 (0 to 0)
Canada	2019	5071 (4988 to 5161)	3751 (3682 to 3823)	710 (677 to 747)	610 (574 to 651)	0 (0 to 0)	5576 (5486 to 5675)	4125 (4049 to 4204)	780 (744 to 822)	671 (631 to 716)	0 (0 to 0)
Canada	2020	5167 (5074 to 5266)	3830 (3755 to 3906)	717 (683 to 754)	621 (584 to 663)	0 (0 to 0)	5683 (5580 to 5791)	4212 (4129 to 4296)	788 (751 to 829)	683 (642 to 729)	0 (0 to 0)
Canada	2021	5233 (5143 to 5323)	3886 (3804 to 3971)	719 (685 to 758)	628 (590 to 671)	0 (0 to 0)	5755 (5656 to 5853)	4274 (4183 to 4367)	791 (753 to 833)	691 (649 to 738)	0 (0 to 0)

Canada	2022	5291 (5186 to 5399)	3937 (3848 to 4029)	720 (685 to 759)	634 (595 to 677)	0 (0 to 0)	5819 (5703 to 5938)	4330 (4231 to 4431)	792 (754 to 835)	697 (655 to 745)	0 (0 to 0)
Canada	2023	5321 (5222 to 5426)	3968 (3874 to 4059)	716 (682 to 756)	637 (598 to 681)	0 (0 to 0)	5852 (5742 to 5967)	4363 (4260 to 4464)	788 (750 to 831)	701 (657 to 749)	0 (0 to 0)
Canada	2024	5349 (5225 to 5475)	3996 (3895 to 4096)	713 (679 to 752)	640 (599 to 685)	0 (0 to 0)	5883 (5747 to 6021)	4395 (4283 to 4505)	785 (747 to 827)	704 (659 to 753)	0 (0 to 0)
Canada	2025	5380 (5249 to 5510)	4025 (3906 to 4141)	712 (677 to 751)	643 (601 to 688)	0 (0 to 0)	5916 (5773 to 6060)	4427 (4295 to 4554)	783 (745 to 826)	707 (661 to 757)	0 (0 to 0)
Canada	2026	5413 (5279 to 5547)	4057 (3935 to 4183)	710 (675 to 749)	646 (602 to 693)	0 (0 to 0)	5953 (5806 to 6100)	4462 (4327 to 4600)	781 (743 to 824)	710 (663 to 763)	0 (0 to 0)
Canada	2027	5450 (5306 to 5596)	4092 (3965 to 4221)	709 (675 to 748)	649 (605 to 699)	0 (0 to 0)	5994 (5836 to 6154)	4500 (4361 to 4642)	780 (742 to 823)	714 (665 to 769)	0 (0 to 0)
Canada	2028	5498 (5354 to 5639)	4134 (4001 to 4264)	710 (675 to 749)	655 (609 to 705)	0 (0 to 0)	6046 (5888 to 6202)	4546 (4400 to 4689)	781 (742 to 824)	720 (670 to 776)	0 (0 to 0)
Canada	2029	5549 (5381 to 5712)	4177 (4040 to 4320)	711 (676 to 751)	660 (614 to 713)	0 (0 to 0)	6102 (5918 to 6282)	4594 (4443 to 4750)	782 (743 to 826)	726 (675 to 784)	0 (0 to 0)
Canada	2030	5601 (5451 to 5762)	4221 (4078 to 4368)	713 (677 to 753)	666 (618 to 721)	0 (0 to 0)	6159 (5994 to 6337)	4642 (4485 to 4804)	784 (745 to 828)	732 (680 to 793)	0 (0 to 0)
Canada	2031	5654 (5482 to 5827)	4266 (4120 to 4422)	716 (679 to 755)	672 (622 to 729)	0 (0 to 0)	6218 (6029 to 6408)	4692 (4531 to 4863)	787 (747 to 831)	739 (685 to 801)	0 (0 to 0)

Canada	2032	5707 (5548 to 5874)	4310 (4157 to 4470)	718 (681 to 759)	678 (627 to 737)	0 (0 to 0)	6276 (6101 to 6460)	4740 (4571 to 4916)	790 (749 to 834)	746 (689 to 811)	0 (0 to 0)
Canada	2033	5760 (5582 to 5944)	4353 (4193 to 4518)	721 (683 to 762)	685 (631 to 744)	0 (0 to 0)	6334 (6139 to 6537)	4788 (4611 to 4969)	793 (752 to 838)	753 (694 to 819)	0 (0 to 0)
Canada	2034	5812 (5631 to 5999)	4395 (4224 to 4567)	725 (685 to 767)	691 (635 to 754)	0 (0 to 0)	6391 (6193 to 6597)	4834 (4646 to 5023)	797 (754 to 843)	760 (699 to 829)	0 (0 to 0)
Canada	2035	5862 (5665 to 6065)	4436 (4258 to 4613)	728 (688 to 771)	698 (640 to 763)	0 (0 to 0)	6447 (6230 to 6670)	4878 (4682 to 5073)	801 (756 to 848)	768 (704 to 839)	0 (0 to 0)
Canada	2036	5912 (5712 to 6103)	4475 (4295 to 4664)	732 (690 to 776)	705 (645 to 771)	0 (0 to 0)	6502 (6281 to 6712)	4922 (4723 to 5129)	805 (759 to 853)	775 (709 to 848)	0 (0 to 0)
Canada	2037	5961 (5752 to 6183)	4514 (4321 to 4705)	736 (693 to 781)	711 (649 to 780)	0 (0 to 0)	6556 (6326 to 6800)	4964 (4752 to 5174)	809 (762 to 859)	782 (714 to 858)	0 (0 to 0)
Canada	2038	6012 (5804 to 6217)	4554 (4363 to 4742)	740 (695 to 786)	718 (654 to 788)	0 (0 to 0)	6612 (6383 to 6837)	5008 (4798 to 5215)	814 (765 to 865)	790 (719 to 867)	0 (0 to 0)
Canada	2039	6064 (5840 to 6292)	4594 (4395 to 4790)	745 (698 to 792)	725 (658 to 799)	0 (0 to 0)	6668 (6423 to 6920)	5052 (4833 to 5268)	819 (768 to 871)	798 (723 to 878)	0 (0 to 0)
Canada	2040	6114 (5896 to 6330)	4632 (4427 to 4834)	749 (701 to 797)	733 (663 to 809)	0 (0 to 0)	6724 (6484 to 6961)	5094 (4868 to 5316)	824 (771 to 877)	806 (729 to 890)	0 (0 to 0)
Canada	2041	6166 (5920 to 6401)	4671 (4453 to 4882)	754 (704 to 804)	740 (667 to 820)	0 (0 to 0)	6780 (6511 to 7040)	5137 (4897 to 5369)	829 (775 to 884)	814 (734 to 902)	0 (0 to 0)

Canada	2042	6216 (5974 to 6455)	4710 (4480 to 4937)	759 (707 to 810)	747 (671 to 831)	0 (0 to 0)	6835 (6569 to 7099)	5179 (4927 to 5429)	835 (778 to 891)	822 (738 to 913)	0 (0 to 0)
Canada	2043	6267 (6027 to 6499)	4749 (4510 to 4982)	764 (711 to 816)	755 (675 to 842)	0 (0 to 0)	6892 (6628 to 7147)	5223 (4960 to 5479)	840 (782 to 898)	830 (742 to 926)	0 (0 to 0)
Canada	2044	6320 (6053 to 6594)	4789 (4545 to 5028)	769 (715 to 823)	762 (680 to 853)	0 (0 to 0)	6950 (6657 to 7251)	5266 (4998 to 5530)	846 (786 to 905)	838 (748 to 939)	0 (0 to 0)
Canada	2045	6372 (6126 to 6632)	4829 (4579 to 5077)	774 (718 to 829)	770 (684 to 864)	0 (0 to 0)	7008 (6736 to 7294)	5311 (5036 to 5583)	851 (790 to 911)	846 (752 to 950)	0 (0 to 0)
Canada	2046	6425 (6139 to 6716)	4869 (4609 to 5118)	779 (721 to 835)	777 (688 to 874)	0 (0 to 0)	7066 (6751 to 7385)	5354 (5068 to 5629)	856 (793 to 919)	855 (756 to 961)	0 (0 to 0)
Canada	2047	6478 (6197 to 6762)	4909 (4649 to 5171)	784 (725 to 843)	785 (691 to 884)	0 (0 to 0)	7124 (6815 to 7437)	5399 (5113 to 5686)	862 (797 to 927)	863 (760 to 972)	0 (0 to 0)
Canada	2048	6532 (6244 to 6838)	4950 (4680 to 5215)	789 (727 to 850)	793 (695 to 898)	0 (0 to 0)	7183 (6867 to 7520)	5444 (5147 to 5735)	867 (800 to 935)	872 (765 to 987)	0 (0 to 0)
Canada	2049	6585 (6289 to 6887)	4991 (4717 to 5260)	794 (730 to 857)	800 (699 to 910)	0 (0 to 0)	7242 (6916 to 7574)	5488 (5187 to 5784)	873 (803 to 943)	880 (769 to 1001)	0 (0 to 0)
Canada	2050	6639 (6319 to 6954)	5032 (4748 to 5315)	799 (733 to 864)	808 (704 to 922)	0 (0 to 0)	7301 (6949 to 7647)	5534 (5222 to 5845)	878 (806 to 951)	889 (774 to 1014)	0 (0 to 0)
Cape Verde	1995	83 (69 to 99)	69 (56 to 86)	11 (8 to 16)	1 (1 to 2)	1 (1 to 1)	178 (148 to 214)	149 (121 to 185)	24 (17 to 34)	2 (1 to 5)	2 (2 to 2)
Cape Verde	1996	83 (69 to 100)	70 (57 to 86)	12 (8 to 16)	1 (1 to 2)	0 (0 to 0)	179 (149 to 216)	152 (123 to 186)	25 (18 to 35)	2 (1 to 4)	0 (0 to 0)

Cape Verde	1997	93 (79 to 108)	69 (57 to 84)	13 (9 to 18)	1 (1 to 2)	9 (9 to 9)	200 (170 to 233)	149 (122 to 181)	28 (20 to 38)	2 (1 to 4)	20 (20 to 20)
Cape Verde	1998	89 (76 to 105)	68 (57 to 82)	16 (12 to 22)	1 (0 to 2)	4 (4 to 4)	192 (164 to 226)	146 (122 to 177)	35 (25 to 47)	2 (1 to 4)	9 (9 to 9)
Cape Verde	1999	100 (87 to 118)	74 (61 to 89)	19 (14 to 26)	1 (1 to 2)	7 (7 to 7)	216 (188 to 253)	158 (132 to 192)	41 (30 to 56)	2 (1 to 4)	14 (14 to 14)
Cape Verde	2000	104 (90 to 123)	79 (65 to 94)	22 (16 to 30)	1 (1 to 2)	3 (3 to 3)	225 (194 to 264)	169 (141 to 202)	48 (34 to 64)	2 (1 to 4)	6 (6 to 6)
Cape Verde	2001	110 (96 to 126)	69 (57 to 82)	22 (16 to 30)	1 (0 to 2)	18 (18 to 18)	237 (207 to 271)	148 (123 to 176)	47 (34 to 64)	2 (1 to 4)	40 (40 to 40)
Cape Verde	2002	110 (95 to 127)	78 (65 to 93)	25 (18 to 33)	1 (0 to 2)	6 (6 to 6)	236 (204 to 273)	168 (140 to 199)	53 (38 to 72)	2 (1 to 4)	13 (13 to 13)
Cape Verde	2003	116 (102 to 131)	71 (59 to 84)	24 (18 to 33)	1 (0 to 2)	19 (19 to 19)	249 (219 to 281)	153 (127 to 181)	53 (38 to 71)	2 (1 to 4)	41 (41 to 41)
Cape Verde	2004	127 (111 to 143)	78 (64 to 92)	26 (19 to 35)	1 (0 to 2)	22 (22 to 22)	273 (239 to 308)	167 (139 to 198)	56 (41 to 75)	2 (1 to 4)	47 (47 to 47)
Cape Verde	2005	136 (121 to 153)	79 (65 to 93)	27 (20 to 36)	1 (0 to 2)	29 (29 to 29)	292 (259 to 329)	169 (141 to 201)	58 (42 to 78)	2 (1 to 4)	63 (63 to 63)
Cape Verde	2006	138 (122 to 156)	81 (68 to 96)	28 (21 to 37)	1 (1 to 2)	28 (28 to 28)	297 (263 to 335)	174 (146 to 206)	61 (45 to 80)	2 (1 to 5)	60 (60 to 60)
Cape Verde	2007	135 (119 to 152)	80 (67 to 95)	29 (22 to 37)	1 (1 to 2)	25 (25 to 25)	290 (255 to 326)	173 (145 to 204)	61 (47 to 80)	3 (1 to 5)	53 (53 to 53)
Cape Verde	2008	139 (123 to 157)	82 (69 to 97)	29 (22 to 38)	1 (1 to 3)	26 (26 to 26)	298 (266 to 337)	176 (149 to 208)	63 (48 to 83)	3 (1 to 6)	56 (56 to 56)
Cape Verde	2009	130 (114 to 150)	86 (72 to 101)	31 (24 to 41)	2 (1 to 3)	11 (11 to 11)	280 (246 to 324)	185 (156 to 217)	68 (52 to 88)	4 (2 to 7)	24 (24 to 24)
Cape Verde	2010	157 (139 to 179)	95 (79 to 112)	36 (27 to 46)	3 (1 to 5)	24 (24 to 24)	339 (299 to 384)	205 (170 to 242)	77 (58 to 100)	6 (3 to 11)	51 (51 to 51)
Cape Verde	2011	172 (154 to 193)	91 (76 to 107)	36 (27 to 47)	3 (2 to 6)	42 (42 to 42)	371 (332 to 415)	196 (163 to 231)	78 (58 to 102)	7 (3 to 12)	90 (90 to 90)

Cape Verde	2012	166 (148 to 188)	97 (80 to 113)	39 (29 to 51)	4 (2 to 7)	26 (26 to 26)	357 (318 to 405)	208 (173 to 244)	84 (63 to 110)	8 (4 to 15)	57 (57 to 57)
Cape Verde	2013	180 (162 to 203)	96 (80 to 114)	40 (30 to 53)	4 (2 to 7)	40 (40 to 40)	387 (350 to 437)	206 (173 to 246)	87 (65 to 114)	9 (4 to 16)	86 (86 to 86)
Cape Verde	2014	168 (151 to 191)	93 (79 to 111)	41 (31 to 55)	4 (2 to 7)	30 (30 to 30)	362 (325 to 411)	201 (170 to 239)	89 (66 to 118)	8 (4 to 15)	64 (64 to 64)
Cape Verde	2015	165 (145 to 188)	97 (81 to 115)	43 (32 to 57)	4 (2 to 7)	22 (22 to 22)	355 (313 to 404)	208 (175 to 246)	92 (68 to 123)	8 (4 to 15)	47 (47 to 47)
Cape Verde	2016	154 (133 to 177)	97 (80 to 117)	44 (33 to 60)	4 (2 to 8)	9 (9 to 9)	331 (286 to 382)	209 (173 to 251)	95 (70 to 129)	9 (4 to 16)	18 (18 to 18)
Cape Verde	2017	162 (135 to 194)	104 (85 to 126)	47 (34 to 65)	4 (2 to 8)	6 (6 to 6)	349 (291 to 417)	225 (183 to 272)	101 (74 to 139)	10 (5 to 18)	13 (13 to 13)
Cape Verde	2018	167 (140 to 200)	108 (88 to 131)	49 (36 to 67)	5 (2 to 8)	6 (6 to 6)	360 (302 to 431)	231 (188 to 281)	106 (78 to 145)	10 (5 to 18)	13 (13 to 13)
Cape Verde	2019	184 (153 to 220)	122 (99 to 148)	51 (37 to 70)	5 (2 to 9)	6 (6 to 6)	396 (330 to 474)	263 (213 to 319)	110 (81 to 151)	11 (5 to 20)	13 (13 to 13)
Cape Verde	2020	192 (161 to 231)	128 (104 to 156)	53 (39 to 73)	5 (3 to 10)	6 (6 to 6)	414 (347 to 497)	276 (224 to 335)	114 (83 to 156)	11 (5 to 21)	13 (12 to 14)
Cape Verde	2021	199 (165 to 236)	133 (108 to 161)	54 (39 to 74)	5 (3 to 10)	6 (6 to 7)	428 (355 to 509)	286 (232 to 347)	117 (85 to 159)	12 (6 to 22)	13 (12 to 14)
Cape Verde	2022	203 (170 to 244)	136 (110 to 165)	55 (40 to 76)	6 (3 to 10)	6 (6 to 7)	437 (366 to 525)	293 (238 to 356)	119 (86 to 163)	12 (6 to 22)	13 (12 to 15)
Cape Verde	2023	208 (173 to 248)	140 (112 to 171)	56 (41 to 77)	6 (3 to 11)	6 (6 to 7)	447 (372 to 533)	300 (242 to 368)	121 (88 to 166)	12 (6 to 23)	13 (12 to 15)
Cape Verde	2024	212 (177 to 256)	143 (116 to 176)	57 (42 to 78)	6 (3 to 11)	6 (6 to 7)	456 (382 to 552)	307 (249 to 379)	123 (89 to 168)	13 (6 to 23)	13 (12 to 15)
Cape Verde	2025	216 (179 to 259)	146 (117 to 179)	58 (42 to 79)	6 (3 to 11)	6 (6 to 7)	465 (384 to 557)	314 (251 to 386)	125 (91 to 171)	13 (6 to 24)	14 (12 to 15)
Cape Verde	2026	220 (182 to 267)	149 (119 to 183)	59 (43 to 81)	6 (3 to 11)	6 (6 to 7)	474 (391 to 575)	320 (255 to 394)	127 (92 to 174)	13 (6 to 24)	14 (12 to 16)

Cape Verde	2027	224 (184 to 270)	152 (119 to 188)	60 (43 to 82)	6 (3 to 11)	6 (6 to 7)	482 (395 to 582)	326 (256 to 406)	128 (93 to 176)	13 (6 to 25)	14 (12 to 16)
Cape Verde	2028	228 (186 to 278)	155 (120 to 194)	60 (44 to 83)	6 (3 to 12)	7 (6 to 8)	491 (401 to 599)	333 (257 to 418)	130 (94 to 179)	14 (7 to 25)	14 (12 to 16)
Cape Verde	2029	232 (188 to 283)	158 (122 to 201)	61 (44 to 84)	6 (3 to 12)	7 (6 to 8)	500 (404 to 608)	340 (262 to 432)	132 (96 to 181)	14 (7 to 26)	14 (12 to 17)
Cape Verde	2030	237 (191 to 292)	161 (122 to 205)	62 (45 to 85)	7 (3 to 12)	7 (6 to 8)	509 (411 to 627)	347 (263 to 441)	134 (97 to 184)	14 (7 to 26)	14 (12 to 17)
Cape Verde	2031	241 (194 to 294)	165 (124 to 211)	63 (46 to 87)	7 (3 to 12)	7 (6 to 8)	519 (418 to 633)	354 (267 to 454)	136 (98 to 186)	14 (7 to 27)	15 (12 to 17)
Cape Verde	2032	245 (197 to 301)	167 (126 to 214)	64 (46 to 88)	7 (3 to 13)	7 (6 to 8)	527 (425 to 649)	360 (272 to 460)	137 (100 to 189)	15 (7 to 27)	15 (13 to 18)
Cape Verde	2033	249 (201 to 304)	170 (130 to 217)	65 (47 to 89)	7 (3 to 13)	7 (6 to 8)	536 (432 to 654)	367 (279 to 466)	139 (101 to 191)	15 (7 to 28)	15 (13 to 18)
Cape Verde	2034	253 (203 to 313)	174 (129 to 223)	65 (47 to 90)	7 (3 to 13)	7 (6 to 9)	545 (436 to 673)	373 (278 to 479)	141 (102 to 193)	15 (7 to 28)	15 (13 to 19)
Cape Verde	2035	258 (205 to 317)	177 (131 to 227)	66 (48 to 91)	7 (4 to 13)	7 (6 to 9)	554 (441 to 682)	381 (283 to 488)	143 (103 to 196)	15 (8 to 29)	15 (13 to 19)
Cape Verde	2036	262 (207 to 325)	180 (132 to 232)	67 (49 to 92)	7 (4 to 14)	7 (6 to 9)	565 (445 to 700)	388 (284 to 500)	145 (105 to 198)	16 (8 to 29)	16 (13 to 20)
Cape Verde	2037	267 (211 to 330)	184 (133 to 238)	68 (49 to 93)	7 (4 to 14)	7 (6 to 9)	575 (453 to 710)	396 (286 to 511)	147 (106 to 200)	16 (8 to 30)	16 (13 to 20)
Cape Verde	2038	272 (214 to 336)	188 (135 to 243)	69 (50 to 95)	8 (4 to 14)	8 (6 to 9)	585 (461 to 724)	404 (291 to 524)	149 (107 to 203)	16 (8 to 30)	16 (13 to 20)
Cape Verde	2039	277 (216 to 344)	191 (138 to 249)	70 (50 to 96)	8 (4 to 14)	8 (6 to 10)	595 (465 to 740)	412 (297 to 535)	150 (108 to 206)	17 (8 to 31)	16 (13 to 21)
Cape Verde	2040	281 (219 to 352)	195 (141 to 256)	71 (51 to 97)	8 (4 to 15)	8 (6 to 10)	606 (471 to 758)	420 (303 to 552)	152 (110 to 209)	17 (8 to 32)	17 (14 to 21)
Cape Verde	2041	287 (224 to 359)	199 (144 to 262)	72 (52 to 98)	8 (4 to 15)	8 (6 to 10)	617 (481 to 773)	428 (309 to 563)	154 (111 to 211)	17 (8 to 32)	17 (14 to 21)

Cape Verde	2042	292 (225 to 367)	203 (144 to 266)	73 (52 to 99)	8 (4 to 15)	8 (6 to 10)	627 (484 to 789)	436 (311 to 573)	156 (112 to 213)	18 (9 to 33)	17 (14 to 22)
Cape Verde	2043	296 (229 to 372)	206 (147 to 272)	74 (53 to 100)	8 (4 to 16)	8 (7 to 11)	638 (493 to 801)	444 (317 to 586)	158 (113 to 216)	18 (9 to 34)	17 (14 to 23)
Cape Verde	2044	301 (234 to 379)	210 (151 to 278)	74 (53 to 102)	8 (4 to 16)	8 (7 to 11)	648 (503 to 816)	452 (325 to 599)	160 (115 to 219)	18 (9 to 34)	18 (14 to 23)
Cape Verde	2045	306 (235 to 384)	214 (151 to 282)	75 (54 to 103)	9 (4 to 16)	8 (7 to 11)	658 (505 to 827)	460 (326 to 608)	162 (116 to 221)	19 (9 to 35)	18 (14 to 23)
Cape Verde	2046	310 (240 to 393)	217 (155 to 289)	76 (55 to 104)	9 (4 to 17)	9 (7 to 11)	668 (516 to 846)	467 (334 to 622)	164 (117 to 223)	19 (9 to 36)	18 (14 to 24)
Cape Verde	2047	315 (241 to 400)	220 (157 to 297)	77 (55 to 105)	9 (4 to 17)	9 (7 to 11)	677 (518 to 860)	474 (338 to 640)	165 (119 to 225)	19 (9 to 36)	19 (15 to 25)
Cape Verde	2048	319 (244 to 403)	223 (156 to 299)	77 (55 to 106)	9 (4 to 17)	9 (7 to 12)	686 (524 to 868)	481 (335 to 644)	167 (119 to 228)	19 (10 to 37)	19 (15 to 26)
Cape Verde	2049	322 (244 to 413)	226 (157 to 306)	78 (56 to 107)	9 (4 to 17)	9 (7 to 12)	694 (525 to 888)	487 (337 to 658)	168 (120 to 230)	20 (10 to 37)	19 (15 to 26)
Cape Verde	2050	326 (248 to 416)	229 (156 to 309)	78 (56 to 108)	9 (5 to 18)	9 (7 to 12)	702 (534 to 895)	493 (337 to 665)	169 (121 to 232)	20 (10 to 38)	20 (15 to 27)
Central African Republic	1995	26 (21 to 32)	9 (6 to 11)	13 (9 to 18)	0 (0 to 1)	4 (4 to 4)	45 (37 to 55)	15 (11 to 19)	23 (16 to 32)	1 (0 to 1)	7 (7 to 7)
Central African Republic	1996	19 (15 to 24)	7 (5 to 8)	12 (8 to 16)	0 (0 to 0)	0 (0 to 0)	33 (26 to 41)	11 (8 to 15)	20 (14 to 28)	0 (0 to 1)	1 (1 to 1)
Central African Republic	1997	20 (16 to 25)	7 (5 to 8)	12 (8 to 16)	0 (0 to 0)	2 (2 to 2)	35 (28 to 44)	11 (8 to 14)	21 (14 to 28)	0 (0 to 1)	3 (3 to 3)
Central African Republic	1998	26 (22 to 31)	7 (5 to 9)	12 (9 to 17)	0 (0 to 1)	7 (7 to 7)	45 (38 to 54)	12 (9 to 15)	21 (15 to 29)	0 (0 to 1)	12 (12 to 12)

Central African Republic	1999	25 (20 to 30)	8 (6 to 11)	12 (9 to 17)	0 (0 to 1)	4 (4 to 4)	43 (35 to 53)	14 (10 to 18)	22 (15 to 30)	0 (0 to 1)	7 (7 to 7)
Central African Republic	2000	23 (19 to 29)	9 (7 to 12)	13 (9 to 18)	0 (0 to 1)	2 (2 to 2)	40 (32 to 51)	15 (12 to 20)	22 (15 to 30)	0 (0 to 1)	3 (3 to 3)
Central African Republic	2001	23 (18 to 28)	8 (6 to 11)	12 (8 to 17)	0 (0 to 0)	2 (2 to 2)	39 (32 to 49)	15 (11 to 19)	20 (14 to 29)	0 (0 to 1)	4 (4 to 4)
Central African Republic	2002	25 (20 to 31)	10 (7 to 13)	12 (9 to 17)	0 (0 to 1)	3 (3 to 3)	44 (35 to 54)	17 (12 to 22)	21 (15 to 30)	0 (0 to 1)	5 (5 to 5)
Central African Republic	2003	21 (17 to 27)	8 (6 to 11)	11 (8 to 15)	0 (0 to 0)	2 (2 to 2)	37 (30 to 46)	14 (10 to 18)	19 (13 to 27)	0 (0 to 1)	4 (4 to 4)
Central African Republic	2004	22 (19 to 28)	8 (6 to 11)	11 (7 to 15)	0 (0 to 0)	3 (3 to 3)	39 (32 to 48)	14 (11 to 19)	19 (13 to 27)	0 (0 to 1)	6 (6 to 6)
Central African Republic	2005	25 (20 to 30)	9 (7 to 12)	11 (8 to 16)	0 (0 to 0)	4 (4 to 4)	43 (35 to 52)	16 (12 to 20)	19 (13 to 27)	0 (0 to 1)	7 (7 to 7)
Central African Republic	2006	24 (20 to 28)	8 (6 to 10)	10 (7 to 15)	0 (0 to 0)	5 (5 to 5)	41 (34 to 49)	14 (10 to 18)	18 (12 to 26)	0 (0 to 1)	9 (9 to 9)
Central African Republic	2007	20 (17 to 25)	7 (6 to 10)	10 (7 to 14)	0 (0 to 0)	3 (3 to 3)	35 (29 to 43)	13 (10 to 17)	17 (12 to 25)	0 (0 to 1)	5 (5 to 5)
Central African Republic	2008	26 (22 to 30)	7 (5 to 9)	10 (7 to 14)	0 (0 to 1)	8 (8 to 8)	44 (38 to 52)	12 (9 to 16)	17 (12 to 25)	0 (0 to 1)	14 (14 to 14)

Central African Republic	2009	19 (16 to 23)	6 (4 to 8)	10 (7 to 13)	0 (0 to 1)	3 (3 to 3)	33 (27 to 40)	10 (7 to 13)	17 (11 to 23)	0 (0 to 1)	5 (5 to 5)
Central African Republic	2010	21 (17 to 25)	6 (4 to 8)	10 (7 to 13)	0 (0 to 1)	5 (5 to 5)	36 (30 to 43)	10 (7 to 13)	17 (12 to 23)	1 (0 to 1)	8 (8 to 8)
Central African Republic	2011	19 (16 to 23)	5 (4 to 6)	9 (6 to 13)	0 (0 to 1)	5 (5 to 5)	33 (28 to 40)	8 (6 to 11)	16 (11 to 22)	0 (0 to 1)	9 (9 to 9)
Central African Republic	2012	19 (16 to 23)	5 (3 to 6)	9 (6 to 13)	0 (0 to 1)	5 (5 to 5)	33 (28 to 40)	8 (6 to 10)	16 (11 to 22)	1 (0 to 1)	9 (9 to 9)
Central African Republic	2013	17 (14 to 20)	3 (2 to 4)	7 (5 to 10)	0 (0 to 0)	6 (6 to 6)	29 (25 to 34)	5 (4 to 6)	13 (9 to 18)	0 (0 to 1)	11 (11 to 11)
Central African Republic	2014	21 (18 to 24)	3 (2 to 4)	8 (5 to 11)	0 (0 to 0)	9 (9 to 9)	35 (31 to 41)	6 (4 to 7)	14 (9 to 19)	0 (0 to 1)	16 (16 to 16)
Central African Republic	2015	15 (13 to 18)	3 (2 to 3)	7 (5 to 10)	0 (0 to 0)	5 (5 to 5)	26 (22 to 31)	4 (3 to 6)	12 (9 to 17)	0 (0 to 1)	8 (8 to 8)
Central African Republic	2016	20 (18 to 23)	2 (2 to 3)	7 (5 to 10)	0 (0 to 0)	11 (11 to 11)	35 (31 to 40)	4 (3 to 6)	12 (9 to 17)	0 (0 to 1)	18 (18 to 18)
Central African Republic	2017	21 (18 to 23)	3 (2 to 4)	7 (5 to 10)	0 (0 to 0)	10 (10 to 10)	36 (32 to 41)	5 (4 to 6)	13 (9 to 17)	0 (0 to 1)	18 (18 to 18)
Central African Republic	2018	20 (18 to 23)	3 (2 to 4)	8 (5 to 10)	0 (0 to 0)	9 (9 to 9)	35 (31 to 41)	6 (4 to 8)	13 (9 to 18)	0 (0 to 1)	16 (16 to 16)

Central African Republic	2019	21 (18 to 24)	4 (3 to 5)	8 (5 to 10)	0 (0 to 0)	9 (9 to 10)	36 (32 to 42)	6 (4 to 8)	13 (9 to 18)	0 (0 to 1)	16 (16 to 16)
Central African Republic	2020	21 (19 to 25)	4 (3 to 5)	8 (6 to 11)	0 (0 to 0)	10 (9 to 10)	37 (32 to 43)	6 (4 to 9)	14 (10 to 19)	0 (0 to 1)	17 (16 to 18)
Central African Republic	2021	22 (19 to 25)	4 (3 to 5)	8 (6 to 11)	0 (0 to 0)	10 (9 to 11)	38 (33 to 44)	7 (5 to 9)	14 (10 to 19)	0 (0 to 1)	17 (16 to 18)
Central African Republic	2022	22 (19 to 26)	4 (3 to 6)	8 (6 to 11)	0 (0 to 0)	10 (9 to 11)	39 (33 to 45)	7 (5 to 10)	14 (10 to 19)	0 (0 to 1)	17 (16 to 19)
Central African Republic	2023	23 (19 to 27)	4 (3 to 6)	8 (6 to 11)	0 (0 to 0)	10 (9 to 11)	39 (34 to 46)	7 (5 to 10)	14 (10 to 20)	0 (0 to 1)	18 (16 to 20)
Central African Republic	2024	23 (20 to 27)	4 (3 to 6)	8 (6 to 11)	0 (0 to 0)	10 (9 to 12)	40 (34 to 47)	7 (5 to 11)	15 (10 to 20)	0 (0 to 1)	18 (16 to 20)
Central African Republic	2025	24 (20 to 28)	4 (3 to 7)	9 (6 to 12)	0 (0 to 0)	10 (9 to 12)	41 (35 to 48)	8 (5 to 11)	15 (10 to 20)	0 (0 to 1)	18 (16 to 21)
Central African Republic	2026	24 (20 to 29)	5 (3 to 7)	9 (6 to 12)	0 (0 to 0)	10 (9 to 12)	41 (35 to 50)	8 (5 to 12)	15 (11 to 21)	0 (0 to 1)	18 (16 to 21)
Central African Republic	2027	24 (21 to 29)	5 (3 to 7)	9 (6 to 12)	0 (0 to 0)	11 (9 to 12)	42 (36 to 50)	8 (5 to 13)	15 (11 to 21)	0 (0 to 1)	18 (16 to 21)
Central African Republic	2028	25 (21 to 30)	5 (3 to 8)	9 (6 to 12)	0 (0 to 0)	11 (9 to 13)	43 (36 to 52)	8 (5 to 13)	16 (11 to 21)	0 (0 to 1)	19 (16 to 22)

Central African Republic	2029	25 (21 to 30)	5 (3 to 8)	9 (6 to 13)	0 (0 to 0)	11 (9 to 13)	44 (37 to 52)	9 (5 to 14)	16 (11 to 22)	0 (0 to 1)	19 (16 to 22)
Central African Republic	2030	26 (22 to 31)	5 (3 to 8)	9 (7 to 13)	0 (0 to 0)	11 (9 to 13)	45 (38 to 54)	9 (5 to 15)	16 (11 to 22)	0 (0 to 1)	19 (16 to 23)
Central African Republic	2031	26 (22 to 32)	5 (3 to 9)	10 (7 to 13)	0 (0 to 1)	11 (10 to 14)	46 (38 to 55)	9 (5 to 15)	16 (12 to 23)	1 (0 to 1)	19 (16 to 23)
Central African Republic	2032	27 (22 to 33)	6 (3 to 9)	10 (7 to 13)	0 (0 to 1)	11 (10 to 14)	47 (39 to 57)	10 (5 to 16)	17 (12 to 23)	1 (0 to 1)	20 (17 to 24)
Central African Republic	2033	28 (23 to 33)	6 (3 to 10)	10 (7 to 14)	0 (0 to 1)	12 (10 to 14)	48 (40 to 58)	10 (5 to 17)	17 (12 to 23)	1 (0 to 1)	20 (17 to 25)
Central African Republic	2034	28 (23 to 34)	6 (3 to 10)	10 (7 to 14)	0 (0 to 1)	12 (10 to 15)	49 (40 to 59)	10 (5 to 17)	17 (12 to 24)	1 (0 to 1)	21 (17 to 25)
Central African Republic	2035	29 (24 to 35)	6 (3 to 11)	10 (7 to 14)	0 (0 to 1)	12 (10 to 15)	50 (41 to 61)	11 (6 to 18)	17 (12 to 24)	1 (0 to 1)	21 (17 to 26)
Central African Republic	2036	29 (24 to 36)	6 (3 to 11)	10 (7 to 14)	0 (0 to 1)	13 (10 to 16)	51 (42 to 62)	11 (6 to 19)	18 (12 to 24)	1 (0 to 1)	22 (17 to 27)
Central African Republic	2037	30 (24 to 37)	7 (3 to 11)	10 (7 to 14)	0 (0 to 1)	13 (10 to 16)	52 (42 to 64)	11 (6 to 20)	18 (12 to 25)	1 (0 to 1)	22 (18 to 28)
Central African Republic	2038	31 (25 to 38)	7 (3 to 12)	10 (7 to 14)	0 (0 to 1)	13 (11 to 17)	53 (43 to 65)	12 (6 to 20)	18 (13 to 25)	1 (0 to 1)	23 (18 to 29)

Central African Republic	2039	31 (25 to 39)	7 (3 to 12)	11 (7 to 14)	0 (0 to 1)	13 (11 to 17)	54 (44 to 67)	12 (6 to 22)	18 (13 to 25)	1 (0 to 1)	23 (18 to 30)
Central African Republic	2040	32 (26 to 39)	7 (3 to 13)	11 (7 to 15)	0 (0 to 1)	14 (11 to 18)	55 (45 to 68)	12 (6 to 22)	18 (13 to 25)	1 (0 to 1)	24 (19 to 31)
Central African Republic	2041	33 (26 to 41)	7 (3 to 13)	11 (8 to 15)	0 (0 to 1)	14 (11 to 18)	56 (45 to 70)	13 (6 to 23)	19 (13 to 26)	1 (0 to 1)	25 (19 to 32)
Central African Republic	2042	33 (27 to 42)	7 (4 to 13)	11 (8 to 15)	0 (0 to 1)	15 (11 to 19)	58 (46 to 73)	13 (6 to 23)	19 (13 to 26)	1 (0 to 1)	25 (19 to 33)
Central African Republic	2043	34 (27 to 42)	8 (4 to 14)	11 (8 to 15)	0 (0 to 1)	15 (11 to 20)	59 (47 to 73)	13 (6 to 23)	19 (13 to 26)	1 (0 to 1)	26 (19 to 35)
Central African Republic	2044	35 (28 to 44)	8 (4 to 14)	11 (8 to 15)	0 (0 to 1)	15 (11 to 21)	60 (48 to 76)	14 (6 to 24)	19 (13 to 26)	1 (0 to 1)	27 (19 to 36)
Central African Republic	2045	35 (28 to 45)	8 (4 to 15)	11 (8 to 15)	0 (0 to 1)	16 (11 to 21)	61 (48 to 78)	14 (7 to 25)	19 (13 to 27)	1 (0 to 1)	27 (20 to 36)
Central African Republic	2046	36 (28 to 46)	8 (4 to 15)	11 (8 to 16)	0 (0 to 1)	16 (12 to 22)	63 (49 to 80)	14 (7 to 27)	20 (13 to 27)	1 (0 to 1)	28 (20 to 37)
Central African Republic	2047	37 (29 to 47)	9 (4 to 16)	11 (8 to 16)	0 (0 to 1)	17 (12 to 23)	64 (50 to 82)	15 (7 to 27)	20 (14 to 27)	1 (0 to 1)	29 (20 to 40)
Central African Republic	2048	38 (30 to 49)	9 (4 to 16)	11 (8 to 16)	0 (0 to 1)	17 (12 to 24)	65 (51 to 84)	15 (7 to 28)	20 (14 to 27)	1 (0 to 1)	30 (21 to 41)

Central African Republic	2049	39 (30 to 50)	9 (4 to 17)	12 (8 to 16)	0 (0 to 1)	18 (12 to 24)	67 (52 to 87)	16 (7 to 29)	20 (14 to 28)	1 (0 to 1)	31 (21 to 42)
Central African Republic	2050	40 (30 to 51)	9 (4 to 17)	12 (8 to 16)	0 (0 to 1)	18 (13 to 25)	69 (53 to 88)	16 (7 to 30)	20 (14 to 28)	1 (0 to 1)	32 (22 to 44)
Chad	1995	32 (26 to 40)	7 (5 to 10)	19 (13 to 26)	1 (1 to 3)	5 (5 to 5)	93 (76 to 116)	21 (16 to 27)	54 (38 to 76)	4 (2 to 8)	14 (14 to 14)
Chad	1996	29 (23 to 36)	7 (5 to 10)	18 (13 to 26)	1 (1 to 3)	2 (2 to 2)	83 (65 to 105)	21 (16 to 28)	53 (37 to 73)	4 (2 to 7)	5 (5 to 5)
Chad	1997	30 (24 to 38)	7 (6 to 10)	18 (13 to 26)	1 (1 to 3)	3 (3 to 3)	88 (70 to 110)	22 (16 to 29)	53 (37 to 74)	4 (2 to 8)	9 (9 to 9)
Chad	1998	29 (23 to 36)	7 (5 to 9)	18 (12 to 25)	1 (1 to 2)	3 (3 to 3)	83 (66 to 105)	21 (16 to 27)	51 (35 to 71)	4 (2 to 7)	8 (8 to 8)
Chad	1999	31 (25 to 39)	8 (6 to 11)	18 (12 to 25)	1 (1 to 2)	4 (4 to 4)	90 (73 to 112)	24 (18 to 31)	52 (36 to 72)	4 (2 to 7)	11 (11 to 11)
Chad	2000	30 (24 to 37)	9 (7 to 11)	17 (12 to 24)	1 (0 to 2)	3 (3 to 3)	85 (68 to 105)	25 (19 to 33)	49 (34 to 68)	3 (1 to 6)	8 (8 to 8)
Chad	2001	28 (22 to 35)	8 (6 to 11)	17 (11 to 23)	1 (0 to 2)	2 (2 to 2)	81 (65 to 101)	24 (18 to 32)	48 (33 to 65)	3 (1 to 5)	7 (7 to 7)
Chad	2002	31 (25 to 39)	9 (7 to 12)	18 (13 to 25)	1 (1 to 2)	3 (3 to 3)	91 (73 to 112)	26 (20 to 35)	52 (36 to 73)	3 (2 to 6)	9 (9 to 9)
Chad	2003	34 (27 to 41)	10 (8 to 13)	18 (13 to 25)	1 (1 to 2)	4 (4 to 4)	97 (79 to 119)	29 (22 to 38)	53 (37 to 73)	4 (2 to 7)	12 (12 to 12)
Chad	2004	34 (27 to 41)	9 (7 to 12)	19 (13 to 26)	1 (1 to 2)	4 (4 to 4)	97 (78 to 119)	27 (21 to 36)	54 (38 to 75)	4 (2 to 7)	12 (12 to 12)
Chad	2005	34 (28 to 42)	8 (6 to 11)	19 (14 to 27)	1 (1 to 3)	5 (5 to 5)	98 (79 to 121)	24 (18 to 32)	56 (40 to 77)	4 (2 to 8)	14 (14 to 14)
Chad	2006	33 (26 to 41)	8 (6 to 10)	20 (14 to 28)	2 (1 to 3)	3 (3 to 3)	94 (75 to 117)	22 (17 to 29)	58 (41 to 79)	5 (2 to 8)	10 (10 to 10)

Chad	2007	32 (25 to 40)	8 (6 to 10)	20 (14 to 28)	2 (1 to 3)	2 (2 to 2)	92 (73 to 115)	22 (17 to 29)	59 (41 to 80)	5 (2 to 9)	7 (7 to 7)
Chad	2008	32 (26 to 39)	7 (5 to 9)	20 (14 to 27)	2 (1 to 3)	3 (3 to 3)	91 (74 to 113)	20 (15 to 26)	57 (40 to 79)	5 (2 to 9)	9 (9 to 9)
Chad	2009	32 (26 to 40)	7 (5 to 10)	20 (14 to 28)	2 (1 to 4)	3 (3 to 3)	93 (75 to 116)	21 (16 to 27)	58 (41 to 81)	5 (3 to 10)	8 (8 to 8)
Chad	2010	35 (29 to 43)	8 (6 to 10)	21 (15 to 28)	2 (1 to 4)	5 (5 to 5)	101 (83 to 125)	22 (17 to 29)	59 (43 to 82)	6 (3 to 11)	14 (14 to 14)
Chad	2011	32 (26 to 40)	7 (5 to 9)	19 (14 to 26)	2 (1 to 3)	4 (4 to 4)	93 (76 to 114)	21 (16 to 27)	55 (40 to 76)	5 (2 to 10)	12 (12 to 12)
Chad	2012	33 (27 to 40)	8 (6 to 10)	20 (14 to 27)	2 (1 to 4)	3 (3 to 3)	94 (76 to 116)	23 (18 to 30)	57 (41 to 79)	5 (2 to 10)	9 (9 to 9)
Chad	2013	37 (31 to 45)	9 (6 to 11)	20 (14 to 28)	2 (1 to 4)	7 (7 to 7)	108 (89 to 130)	25 (19 to 31)	58 (41 to 80)	6 (2 to 10)	20 (20 to 20)
Chad	2014	36 (30 to 44)	9 (7 to 11)	21 (15 to 28)	2 (1 to 4)	5 (5 to 5)	104 (86 to 127)	25 (19 to 32)	59 (43 to 81)	6 (3 to 11)	14 (14 to 14)
Chad	2015	33 (26 to 40)	8 (6 to 10)	20 (15 to 28)	2 (1 to 4)	3 (3 to 3)	94 (75 to 116)	22 (17 to 28)	58 (42 to 81)	5 (3 to 10)	8 (8 to 8)
Chad	2016	32 (26 to 39)	6 (5 to 8)	19 (13 to 26)	2 (1 to 3)	5 (5 to 5)	91 (74 to 113)	17 (13 to 22)	54 (38 to 75)	5 (2 to 9)	15 (15 to 15)
Chad	2017	29 (23 to 37)	6 (4 to 7)	18 (13 to 25)	2 (1 to 3)	4 (4 to 4)	83 (66 to 106)	16 (12 to 21)	52 (36 to 73)	5 (2 to 9)	11 (11 to 11)
Chad	2018	28 (22 to 35)	5 (4 to 6)	18 (13 to 25)	2 (1 to 3)	4 (4 to 4)	80 (64 to 101)	14 (10 to 19)	52 (36 to 73)	5 (2 to 9)	10 (10 to 10)
Chad	2019	29 (23 to 36)	5 (4 to 7)	18 (13 to 25)	2 (1 to 3)	4 (4 to 4)	82 (66 to 105)	16 (12 to 21)	51 (36 to 72)	5 (2 to 9)	10 (10 to 10)
Chad	2020	29 (23 to 37)	6 (4 to 8)	18 (13 to 26)	2 (1 to 3)	4 (3 to 4)	84 (67 to 106)	16 (12 to 22)	53 (37 to 74)	5 (2 to 9)	10 (10 to 11)
Chad	2021	29 (23 to 37)	6 (4 to 8)	18 (13 to 26)	2 (1 to 3)	4 (3 to 4)	84 (67 to 106)	16 (12 to 22)	53 (37 to 74)	5 (2 to 9)	10 (10 to 11)

Chad	2022	29 (23 to 37)	6 (4 to 8)	19 (13 to 26)	2 (1 to 3)	4 (3 to 4)	85 (67 to 107)	16 (12 to 23)	53 (38 to 75)	5 (2 to 9)	10 (9 to 11)
Chad	2023	30 (23 to 38)	6 (4 to 8)	19 (13 to 26)	2 (1 to 3)	3 (3 to 4)	86 (67 to 110)	17 (12 to 23)	54 (38 to 76)	5 (2 to 9)	10 (9 to 11)
Chad	2024	30 (24 to 38)	6 (4 to 8)	19 (13 to 27)	2 (1 to 3)	3 (3 to 4)	86 (68 to 110)	17 (12 to 23)	55 (38 to 77)	5 (2 to 10)	10 (9 to 11)
Chad	2025	30 (24 to 39)	6 (4 to 9)	19 (13 to 27)	2 (1 to 3)	3 (3 to 4)	87 (69 to 112)	17 (12 to 25)	55 (39 to 78)	5 (3 to 10)	10 (9 to 11)
Chad	2026	31 (24 to 39)	6 (4 to 9)	19 (14 to 27)	2 (1 to 3)	3 (3 to 4)	88 (69 to 112)	18 (12 to 25)	56 (39 to 79)	5 (3 to 10)	9 (8 to 11)
Chad	2027	31 (24 to 40)	6 (4 to 9)	20 (14 to 28)	2 (1 to 4)	3 (3 to 4)	89 (70 to 115)	18 (12 to 26)	57 (40 to 79)	5 (3 to 10)	9 (8 to 11)
Chad	2028	31 (25 to 40)	6 (4 to 9)	20 (14 to 28)	2 (1 to 4)	3 (3 to 4)	91 (71 to 115)	19 (13 to 27)	57 (40 to 80)	6 (3 to 10)	9 (8 to 11)
Chad	2029	32 (25 to 41)	7 (4 to 10)	20 (14 to 28)	2 (1 to 4)	3 (3 to 4)	92 (71 to 118)	19 (13 to 27)	58 (41 to 82)	6 (3 to 11)	9 (8 to 11)
Chad	2030	32 (25 to 41)	7 (5 to 10)	20 (14 to 29)	2 (1 to 4)	3 (3 to 4)	93 (72 to 118)	20 (13 to 28)	59 (41 to 83)	6 (3 to 11)	9 (8 to 11)
Chad	2031	33 (25 to 42)	7 (5 to 10)	21 (14 to 29)	2 (1 to 4)	3 (3 to 4)	94 (73 to 122)	20 (13 to 29)	59 (42 to 84)	6 (3 to 11)	9 (7 to 10)
Chad	2032	33 (26 to 42)	7 (5 to 10)	21 (15 to 29)	2 (1 to 4)	3 (3 to 4)	95 (74 to 121)	20 (13 to 30)	60 (42 to 85)	6 (3 to 12)	9 (7 to 10)
Chad	2033	33 (26 to 44)	7 (5 to 11)	21 (15 to 30)	2 (1 to 4)	3 (2 to 4)	96 (74 to 127)	21 (13 to 31)	61 (42 to 86)	6 (3 to 12)	8 (7 to 10)
Chad	2034	34 (26 to 44)	7 (5 to 11)	21 (15 to 30)	2 (1 to 4)	3 (2 to 4)	97 (75 to 125)	21 (13 to 32)	62 (43 to 87)	6 (3 to 12)	8 (7 to 10)
Chad	2035	34 (27 to 45)	8 (5 to 11)	22 (15 to 30)	2 (1 to 4)	3 (2 to 3)	98 (76 to 128)	22 (14 to 33)	62 (43 to 88)	6 (3 to 12)	8 (7 to 10)
Chad	2036	35 (26 to 44)	8 (5 to 12)	22 (15 to 31)	2 (1 to 4)	3 (2 to 4)	100 (76 to 128)	22 (14 to 35)	63 (44 to 89)	6 (3 to 13)	8 (7 to 10)

Chad	2037	35 (27 to 46)	8 (5 to 12)	22 (15 to 31)	2 (1 to 4)	3 (2 to 3)	101 (77 to 132)	23 (14 to 36)	63 (44 to 90)	7 (3 to 13)	8 (7 to 10)
Chad	2038	35 (27 to 45)	8 (5 to 13)	22 (15 to 32)	2 (1 to 5)	3 (2 to 3)	102 (77 to 130)	23 (14 to 36)	64 (44 to 91)	7 (3 to 13)	8 (6 to 10)
Chad	2039	36 (27 to 47)	8 (5 to 13)	23 (16 to 32)	2 (1 to 5)	3 (2 to 3)	103 (77 to 135)	23 (14 to 37)	65 (45 to 92)	7 (3 to 13)	8 (6 to 10)
Chad	2040	36 (28 to 47)	8 (5 to 13)	23 (16 to 32)	2 (1 to 5)	3 (2 to 3)	104 (79 to 135)	24 (15 to 38)	66 (45 to 93)	7 (3 to 14)	8 (6 to 10)
Chad	2041	37 (27 to 48)	8 (5 to 13)	23 (16 to 33)	2 (1 to 5)	3 (2 to 3)	106 (79 to 138)	24 (15 to 39)	67 (46 to 94)	7 (3 to 14)	8 (6 to 10)
Chad	2042	37 (29 to 48)	9 (5 to 14)	23 (16 to 33)	3 (1 to 5)	3 (2 to 3)	107 (82 to 139)	25 (15 to 40)	67 (47 to 95)	7 (4 to 14)	8 (6 to 10)
Chad	2043	38 (28 to 49)	9 (5 to 14)	24 (16 to 34)	3 (1 to 5)	3 (2 to 3)	109 (81 to 142)	26 (15 to 41)	68 (47 to 97)	7 (4 to 14)	7 (6 to 10)
Chad	2044	38 (29 to 50)	9 (5 to 15)	24 (17 to 34)	3 (1 to 5)	3 (2 to 3)	110 (83 to 143)	26 (15 to 43)	69 (48 to 98)	8 (4 to 15)	7 (6 to 10)
Chad	2045	39 (30 to 52)	9 (5 to 15)	24 (17 to 35)	3 (1 to 5)	3 (2 to 3)	112 (85 to 149)	27 (16 to 43)	70 (49 to 99)	8 (4 to 15)	7 (6 to 9)
Chad	2046	39 (30 to 51)	10 (6 to 15)	25 (17 to 35)	3 (1 to 5)	2 (2 to 3)	114 (86 to 146)	27 (16 to 44)	71 (49 to 101)	8 (4 to 15)	7 (6 to 9)
Chad	2047	40 (30 to 53)	10 (6 to 16)	25 (17 to 36)	3 (1 to 5)	2 (2 to 3)	115 (87 to 151)	28 (16 to 45)	72 (50 to 102)	8 (4 to 16)	7 (6 to 9)
Chad	2048	41 (31 to 54)	10 (6 to 16)	25 (18 to 36)	3 (1 to 6)	2 (2 to 3)	117 (90 to 155)	29 (17 to 46)	73 (51 to 104)	8 (4 to 16)	7 (5 to 9)
Chad	2049	41 (31 to 54)	10 (6 to 17)	26 (18 to 37)	3 (1 to 6)	2 (2 to 3)	119 (89 to 156)	30 (17 to 49)	75 (52 to 106)	9 (4 to 16)	7 (5 to 9)
Chad	2050	42 (32 to 55)	11 (6 to 17)	26 (18 to 37)	3 (1 to 6)	2 (2 to 3)	122 (93 to 159)	30 (18 to 50)	76 (52 to 108)	9 (4 to 17)	7 (5 to 9)
Chile	1995	521 (481 to 566)	196 (177 to 218)	207 (182 to 234)	116 (92 to 147)	2 (2 to 2)	894 (825 to 971)	336 (304 to 375)	355 (312 to 401)	200 (158 to 252)	3 (3 to 3)

Chile	1996	587 (552 to 628)	219 (201 to 238)	238 (215 to 264)	129 (108 to 154)	2 (2 to 2)	1007 (946 to 1077)	375 (344 to 408)	409 (370 to 452)	221 (184 to 264)	3 (3 to 3)
Chile	1997	646 (609 to 684)	236 (218 to 253)	270 (248 to 293)	136 (115 to 161)	4 (4 to 4)	1107 (1045 to 1173)	404 (375 to 433)	463 (425 to 503)	234 (198 to 277)	6 (6 to 6)
Chile	1998	694 (657 to 732)	254 (237 to 272)	295 (271 to 319)	144 (123 to 168)	0 (0 to 0)	1190 (1127 to 1255)	436 (407 to 467)	505 (466 to 548)	248 (211 to 289)	1 (1 to 1)
Chile	1999	713 (676 to 753)	263 (246 to 281)	303 (279 to 329)	147 (125 to 170)	0 (0 to 0)	1223 (1160 to 1291)	451 (421 to 483)	520 (479 to 565)	252 (214 to 292)	0 (0 to 0)
Chile	2000	712 (673 to 747)	261 (243 to 279)	304 (280 to 328)	147 (124 to 170)	0 (0 to 0)	1221 (1154 to 1282)	447 (417 to 479)	521 (479 to 563)	251 (213 to 292)	0 (0 to 0)
Chile	2001	726 (687 to 762)	268 (250 to 286)	310 (284 to 333)	148 (126 to 172)	0 (0 to 0)	1245 (1178 to 1307)	459 (429 to 491)	531 (488 to 572)	253 (216 to 295)	1 (1 to 1)
Chile	2002	741 (704 to 781)	278 (260 to 296)	316 (292 to 341)	147 (126 to 171)	0 (0 to 0)	1271 (1207 to 1340)	476 (446 to 508)	543 (501 to 585)	252 (216 to 293)	0 (0 to 0)
Chile	2003	749 (711 to 787)	285 (266 to 303)	319 (294 to 345)	144 (124 to 169)	1 (1 to 1)	1284 (1220 to 1350)	489 (457 to 520)	548 (504 to 592)	247 (213 to 289)	1 (1 to 1)
Chile	2004	751 (716 to 790)	291 (273 to 310)	318 (294 to 343)	140 (121 to 162)	2 (2 to 2)	1289 (1228 to 1355)	500 (468 to 531)	545 (504 to 589)	241 (207 to 278)	3 (3 to 3)
Chile	2005	757 (721 to 793)	301 (283 to 321)	316 (291 to 341)	139 (120 to 162)	1 (1 to 1)	1299 (1236 to 1360)	517 (486 to 550)	541 (499 to 584)	238 (205 to 277)	2 (2 to 2)

Chile	2006	758 (722 to 796)	311 (293 to 331)	309 (284 to 334)	138 (118 to 160)	0 (0 to 0)	1300 (1238 to 1364)	534 (502 to 567)	529 (488 to 573)	236 (203 to 274)	1 (1 to 1)
Chile	2007	794 (756 to 833)	337 (317 to 357)	311 (287 to 335)	146 (125 to 168)	1 (1 to 1)	1362 (1297 to 1428)	578 (544 to 613)	533 (492 to 575)	250 (215 to 288)	1 (1 to 1)
Chile	2008	853 (815 to 897)	376 (356 to 398)	318 (294 to 342)	159 (138 to 184)	0 (0 to 0)	1464 (1397 to 1538)	646 (610 to 683)	545 (504 to 586)	273 (237 to 316)	1 (1 to 1)
Chile	2009	907 (865 to 949)	416 (395 to 440)	320 (298 to 346)	170 (148 to 195)	0 (0 to 0)	1555 (1484 to 1628)	714 (677 to 755)	549 (511 to 593)	292 (254 to 335)	0 (0 to 0)
Chile	2010	921 (881 to 964)	431 (407 to 455)	318 (296 to 343)	172 (151 to 196)	0 (0 to 0)	1579 (1512 to 1654)	738 (699 to 780)	546 (507 to 588)	295 (259 to 336)	0 (0 to 0)
Chile	2011	964 (927 to 1007)	456 (434 to 478)	328 (307 to 353)	180 (159 to 203)	0 (0 to 0)	1654 (1591 to 1727)	782 (744 to 820)	563 (526 to 605)	308 (272 to 348)	0 (0 to 0)
Chile	2012	1036 (999 to 1076)	494 (473 to 516)	349 (328 to 373)	193 (173 to 215)	0 (0 to 0)	1777 (1714 to 1846)	847 (811 to 885)	598 (562 to 640)	331 (296 to 369)	0 (0 to 0)
Chile	2013	1105 (1068 to 1144)	527 (508 to 548)	374 (352 to 398)	203 (182 to 223)	0 (0 to 0)	1894 (1832 to 1962)	904 (871 to 940)	642 (605 to 682)	348 (312 to 382)	0 (0 to 0)
Chile	2014	1184 (1149 to 1220)	568 (546 to 590)	404 (383 to 428)	211 (190 to 233)	0 (0 to 0)	2030 (1970 to 2092)	974 (937 to 1012)	694 (656 to 734)	363 (326 to 400)	0 (0 to 0)
Chile	2015	1269 (1230 to 1309)	615 (592 to 637)	435 (412 to 459)	218 (199 to 241)	0 (0 to 0)	2176 (2109 to 2245)	1056 (1015 to 1093)	746 (707 to 787)	374 (341 to 413)	0 (0 to 0)

Chile	2016	1328 (1287 to 1372)	654 (631 to 678)	456 (431 to 483)	218 (196 to 241)	0 (0 to 0)	2277 (2207 to 2354)	1122 (1082 to 1162)	782 (739 to 829)	373 (337 to 414)	0 (0 to 0)
Chile	2017	1379 (1311 to 1460)	687 (654 to 720)	469 (435 to 505)	223 (195 to 256)	0 (0 to 0)	2365 (2248 to 2504)	1178 (1122 to 1235)	805 (747 to 866)	383 (335 to 439)	0 (0 to 0)
Chile	2018	1421 (1347 to 1500)	709 (672 to 746)	482 (448 to 519)	230 (201 to 263)	0 (0 to 0)	2436 (2310 to 2572)	1215 (1152 to 1279)	827 (768 to 891)	394 (344 to 451)	0 (0 to 0)
Chile	2019	1433 (1359 to 1519)	716 (677 to 756)	485 (450 to 522)	232 (203 to 266)	0 (0 to 0)	2458 (2332 to 2606)	1228 (1161 to 1297)	832 (773 to 895)	398 (348 to 456)	0 (0 to 0)
Chile	2020	1460 (1383 to 1542)	734 (694 to 776)	489 (455 to 526)	237 (207 to 272)	0 (0 to 0)	2505 (2372 to 2644)	1259 (1190 to 1330)	839 (780 to 902)	407 (355 to 466)	0 (0 to 0)
Chile	2021	1489 (1410 to 1572)	754 (713 to 797)	493 (458 to 530)	242 (212 to 278)	0 (0 to 0)	2555 (2418 to 2696)	1294 (1223 to 1368)	845 (786 to 910)	416 (364 to 476)	0 (0 to 0)
Chile	2022	1523 (1443 to 1613)	777 (733 to 822)	498 (462 to 536)	248 (217 to 285)	0 (0 to 0)	2612 (2475 to 2767)	1333 (1258 to 1410)	854 (793 to 919)	426 (372 to 488)	0 (0 to 0)
Chile	2023	1558 (1474 to 1655)	801 (755 to 848)	503 (467 to 542)	254 (222 to 291)	0 (0 to 0)	2672 (2528 to 2838)	1374 (1295 to 1454)	863 (801 to 929)	436 (380 to 499)	0 (0 to 0)
Chile	2024	1594 (1504 to 1691)	826 (778 to 873)	508 (473 to 548)	260 (227 to 298)	0 (0 to 0)	2734 (2579 to 2901)	1417 (1334 to 1498)	871 (811 to 939)	446 (389 to 511)	0 (0 to 0)
Chile	2025	1630 (1539 to 1732)	851 (802 to 901)	513 (477 to 553)	266 (231 to 305)	0 (0 to 0)	2795 (2640 to 2971)	1459 (1375 to 1545)	880 (818 to 949)	456 (397 to 522)	0 (0 to 0)

Chile	2026	1667 (1572 to 1766)	877 (825 to 932)	518 (482 to 559)	272 (237 to 312)	0 (0 to 0)	2859 (2697 to 3030)	1504 (1415 to 1598)	889 (826 to 958)	466 (406 to 535)	0 (0 to 0)
Chile	2027	1704 (1607 to 1812)	903 (848 to 962)	523 (487 to 564)	278 (242 to 319)	0 (0 to 0)	2923 (2756 to 3108)	1549 (1454 to 1650)	898 (834 to 968)	477 (414 to 548)	0 (0 to 0)
Chile	2028	1744 (1641 to 1857)	931 (871 to 992)	529 (491 to 571)	284 (247 to 327)	0 (0 to 0)	2992 (2815 to 3184)	1597 (1494 to 1702)	907 (843 to 979)	488 (424 to 562)	0 (0 to 0)
Chile	2029	1786 (1678 to 1906)	960 (896 to 1026)	535 (496 to 579)	291 (253 to 335)	0 (0 to 0)	3064 (2878 to 3269)	1647 (1536 to 1760)	918 (851 to 993)	499 (433 to 575)	0 (0 to 0)
Chile	2030	1829 (1712 to 1955)	990 (921 to 1061)	541 (501 to 587)	298 (258 to 344)	0 (0 to 0)	3136 (2937 to 3353)	1697 (1580 to 1819)	928 (859 to 1006)	511 (443 to 589)	0 (0 to 0)
Chile	2031	1871 (1749 to 2004)	1019 (945 to 1096)	547 (507 to 594)	305 (264 to 352)	0 (0 to 0)	3209 (2999 to 3438)	1748 (1620 to 1880)	938 (869 to 1019)	523 (452 to 603)	0 (0 to 0)
Chile	2032	1913 (1785 to 2059)	1048 (969 to 1130)	553 (512 to 602)	312 (269 to 360)	0 (0 to 0)	3281 (3061 to 3531)	1797 (1662 to 1937)	949 (878 to 1032)	535 (462 to 618)	0 (0 to 0)
Chile	2033	1953 (1815 to 2098)	1076 (991 to 1163)	559 (516 to 608)	318 (274 to 369)	0 (0 to 0)	3349 (3113 to 3598)	1845 (1700 to 1996)	958 (885 to 1043)	546 (470 to 632)	0 (0 to 0)
Chile	2034	1993 (1854 to 2135)	1103 (1016 to 1198)	565 (521 to 615)	325 (279 to 376)	0 (0 to 0)	3419 (3180 to 3661)	1893 (1742 to 2054)	969 (894 to 1055)	557 (479 to 645)	0 (0 to 0)
Chile	2035	2034 (1888 to 2183)	1131 (1038 to 1232)	571 (525 to 621)	332 (285 to 385)	0 (0 to 0)	3488 (3239 to 3744)	1940 (1781 to 2113)	979 (901 to 1066)	569 (489 to 661)	0 (0 to 0)

Chile	2036	2075 (1925 to 2239)	1160 (1061 to 1265)	577 (530 to 628)	339 (291 to 394)	0 (0 to 0)	3560 (3302 to 3840)	1989 (1821 to 2170)	990 (910 to 1077)	581 (499 to 676)	0 (0 to 0)
Chile	2037	2118 (1958 to 2288)	1188 (1083 to 1298)	584 (535 to 636)	346 (296 to 403)	0 (0 to 0)	3633 (3358 to 3925)	2038 (1858 to 2227)	1001 (918 to 1091)	593 (508 to 692)	0 (0 to 0)
Chile	2038	2162 (2000 to 2339)	1218 (1109 to 1333)	591 (541 to 644)	353 (302 to 412)	0 (0 to 0)	3707 (3430 to 4012)	2089 (1903 to 2287)	1013 (929 to 1105)	606 (518 to 707)	0 (0 to 0)
Chile	2039	2205 (2024 to 2399)	1247 (1132 to 1368)	597 (546 to 652)	360 (308 to 422)	0 (0 to 0)	3781 (3472 to 4114)	2139 (1942 to 2346)	1024 (936 to 1119)	618 (528 to 724)	0 (0 to 0)
Chile	2040	2248 (2061 to 2453)	1276 (1156 to 1403)	604 (551 to 661)	368 (314 to 431)	0 (0 to 0)	3855 (3536 to 4207)	2188 (1983 to 2406)	1036 (945 to 1133)	631 (538 to 740)	0 (0 to 0)
Chile	2041	2290 (2099 to 2494)	1304 (1178 to 1437)	611 (556 to 668)	375 (320 to 440)	0 (0 to 0)	3928 (3600 to 4277)	2237 (2021 to 2465)	1047 (954 to 1146)	643 (548 to 755)	0 (0 to 0)
Chile	2042	2332 (2140 to 2541)	1333 (1201 to 1469)	617 (561 to 676)	382 (325 to 449)	0 (0 to 0)	4000 (3670 to 4359)	2286 (2059 to 2519)	1059 (963 to 1159)	655 (557 to 770)	0 (0 to 0)
Chile	2043	2375 (2171 to 2595)	1361 (1225 to 1505)	624 (567 to 684)	389 (330 to 458)	0 (0 to 0)	4073 (3724 to 4451)	2335 (2100 to 2582)	1070 (973 to 1173)	668 (566 to 785)	0 (0 to 0)
Chile	2044	2418 (2211 to 2641)	1390 (1249 to 1540)	631 (573 to 692)	397 (336 to 467)	0 (0 to 0)	4147 (3793 to 4530)	2384 (2143 to 2641)	1083 (983 to 1187)	681 (576 to 801)	0 (0 to 0)
Chile	2045	2461 (2242 to 2694)	1418 (1274 to 1571)	638 (579 to 701)	404 (341 to 476)	0 (0 to 0)	4220 (3845 to 4621)	2432 (2185 to 2695)	1095 (992 to 1202)	694 (585 to 816)	0 (0 to 0)

Chile	2046	2503 (2270 to 2740)	1446 (1298 to 1605)	645 (584 to 710)	412 (347 to 485)	0 (0 to 0)	4293 (3893 to 4700)	2480 (2226 to 2752)	1107 (1001 to 1217)	706 (595 to 832)	0 (0 to 0)
Chile	2047	2545 (2310 to 2783)	1474 (1322 to 1637)	652 (588 to 718)	419 (352 to 494)	0 (0 to 0)	4366 (3963 to 4773)	2528 (2268 to 2808)	1119 (1009 to 1231)	719 (604 to 847)	0 (0 to 0)
Chile	2048	2587 (2353 to 2832)	1502 (1345 to 1668)	659 (593 to 726)	427 (357 to 503)	0 (0 to 0)	4437 (4035 to 4857)	2575 (2307 to 2861)	1130 (1017 to 1246)	732 (613 to 863)	0 (0 to 0)
Chile	2049	2627 (2381 to 2876)	1528 (1366 to 1700)	665 (597 to 734)	434 (362 to 512)	0 (0 to 0)	4506 (4084 to 4932)	2621 (2344 to 2915)	1141 (1025 to 1260)	744 (621 to 878)	0 (0 to 0)
Chile	2050	2669 (2427 to 2922)	1556 (1391 to 1728)	672 (602 to 743)	441 (367 to 522)	0 (0 to 0)	4577 (4162 to 5013)	2668 (2386 to 2964)	1153 (1033 to 1274)	756 (630 to 895)	0 (0 to 0)
China	1995	55 (46 to 67)	15 (12 to 19)	28 (22 to 36)	12 (6 to 20)	0 (0 to 0)	106 (88 to 130)	30 (24 to 37)	54 (42 to 69)	23 (12 to 39)	0 (0 to 0)
China	1996	61 (51 to 73)	17 (13 to 20)	32 (25 to 40)	12 (7 to 21)	0 (0 to 0)	117 (98 to 140)	32 (26 to 39)	61 (48 to 78)	24 (13 to 39)	0 (0 to 0)
China	1997	67 (57 to 80)	18 (14 to 22)	37 (29 to 46)	13 (7 to 21)	0 (0 to 0)	130 (110 to 155)	34 (28 to 43)	71 (57 to 89)	24 (13 to 41)	0 (0 to 0)
China	1998	75 (64 to 88)	19 (16 to 24)	42 (34 to 52)	13 (7 to 22)	0 (0 to 0)	144 (123 to 169)	37 (30 to 45)	81 (66 to 100)	25 (14 to 42)	0 (0 to 0)
China	1999	84 (72 to 98)	21 (18 to 26)	49 (40 to 59)	14 (8 to 23)	0 (0 to 0)	162 (139 to 189)	41 (34 to 50)	94 (78 to 114)	27 (15 to 44)	0 (0 to 0)
China	2000	92 (80 to 107)	23 (19 to 27)	55 (46 to 66)	14 (8 to 23)	0 (0 to 0)	177 (154 to 205)	44 (36 to 52)	106 (88 to 127)	27 (15 to 45)	0 (0 to 0)
China	2001	100 (87 to 115)	25 (21 to 30)	61 (51 to 73)	14 (8 to 22)	0 (0 to 0)	192 (166 to 222)	48 (40 to 58)	117 (98 to 140)	26 (15 to 43)	0 (0 to 0)
China	2002	110 (95 to 126)	29 (24 to 35)	67 (56 to 80)	14 (8 to 23)	0 (0 to 0)	212 (184 to 243)	56 (47 to 66)	129 (107 to 153)	26 (15 to 45)	0 (0 to 0)

China	2003	117 (102 to 133)	33 (28 to 39)	71 (59 to 84)	13 (7 to 23)	0 (0 to 0)	226 (196 to 256)	64 (54 to 75)	136 (113 to 161)	26 (14 to 45)	0 (0 to 0)
China	2004	125 (109 to 142)	38 (32 to 44)	74 (61 to 87)	13 (7 to 23)	0 (0 to 0)	240 (209 to 273)	73 (62 to 85)	141 (117 to 168)	26 (14 to 43)	0 (0 to 0)
China	2005	136 (119 to 153)	45 (38 to 52)	77 (64 to 91)	13 (7 to 22)	0 (0 to 0)	261 (229 to 295)	86 (74 to 101)	149 (124 to 175)	26 (14 to 43)	0 (0 to 0)
China	2006	146 (128 to 165)	52 (45 to 61)	80 (67 to 94)	14 (7 to 23)	0 (0 to 0)	280 (246 to 318)	101 (87 to 116)	153 (129 to 181)	26 (14 to 43)	0 (0 to 0)
China	2007	156 (138 to 175)	61 (53 to 71)	81 (68 to 96)	14 (8 to 23)	0 (0 to 0)	300 (265 to 337)	118 (101 to 136)	155 (130 to 184)	26 (15 to 44)	1 (1 to 1)
China	2008	184 (164 to 205)	81 (70 to 93)	87 (74 to 102)	15 (8 to 25)	0 (0 to 0)	353 (315 to 394)	156 (135 to 178)	168 (142 to 196)	29 (16 to 48)	0 (0 to 0)
China	2009	213 (192 to 236)	103 (90 to 116)	94 (80 to 110)	16 (9 to 27)	0 (0 to 0)	410 (369 to 453)	198 (174 to 224)	181 (153 to 212)	31 (17 to 52)	1 (1 to 1)
China	2010	233 (211 to 257)	119 (106 to 134)	98 (83 to 114)	16 (9 to 27)	0 (0 to 0)	449 (406 to 495)	229 (203 to 257)	188 (159 to 220)	31 (17 to 51)	0 (0 to 0)
China	2011	267 (243 to 291)	143 (128 to 160)	107 (91 to 125)	17 (9 to 28)	0 (0 to 0)	513 (467 to 560)	275 (246 to 307)	205 (174 to 239)	32 (18 to 53)	0 (0 to 0)
China	2012	296 (271 to 322)	164 (148 to 183)	115 (99 to 134)	17 (10 to 27)	0 (0 to 0)	569 (521 to 620)	315 (285 to 352)	221 (189 to 258)	33 (19 to 53)	1 (1 to 1)
China	2013	324 (297 to 351)	184 (166 to 203)	123 (107 to 143)	18 (10 to 29)	0 (0 to 0)	624 (572 to 675)	353 (319 to 390)	236 (205 to 274)	34 (20 to 56)	0 (0 to 0)
China	2014	353 (326 to 382)	204 (184 to 225)	130 (112 to 151)	19 (11 to 32)	0 (0 to 0)	679 (626 to 735)	391 (354 to 432)	251 (216 to 291)	37 (21 to 61)	0 (0 to 0)
China	2015	390 (359 to 425)	228 (207 to 253)	140 (120 to 162)	22 (12 to 36)	0 (0 to 0)	750 (690 to 816)	439 (398 to 486)	269 (231 to 311)	42 (23 to 69)	0 (0 to 0)
China	2016	422 (386 to 461)	246 (220 to 274)	151 (127 to 174)	25 (14 to 44)	0 (0 to 0)	811 (742 to 886)	473 (424 to 527)	289 (245 to 335)	49 (26 to 84)	0 (0 to 0)
China	2017	455 (400 to 517)	264 (231 to 298)	161 (134 to 190)	30 (16 to 52)	0 (0 to 0)	875 (769 to 994)	507 (445 to 572)	310 (258 to 365)	57 (30 to 101)	0 (0 to 0)

China	2018	500 (443 to 566)	297 (259 to 336)	172 (143 to 202)	32 (16 to 56)	0 (0 to 0)	962 (851 to 1088)	571 (499 to 647)	330 (274 to 388)	61 (32 to 107)	0 (0 to 0)
China	2019	542 (473 to 617)	328 (285 to 372)	180 (150 to 212)	34 (17 to 59)	0 (0 to 0)	1041 (910 to 1186)	630 (548 to 715)	347 (288 to 408)	65 (34 to 114)	0 (0 to 0)
China	2020	581 (510 to 659)	356 (309 to 407)	189 (157 to 223)	36 (19 to 63)	0 (0 to 0)	1117 (981 to 1267)	685 (593 to 782)	364 (302 to 429)	68 (36 to 120)	0 (0 to 0)
China	2021	614 (535 to 705)	381 (331 to 435)	196 (162 to 231)	37 (19 to 65)	0 (0 to 0)	1180 (1028 to 1356)	732 (635 to 836)	376 (311 to 443)	71 (37 to 125)	0 (0 to 0)
China	2022	647 (572 to 735)	406 (354 to 464)	202 (167 to 238)	39 (20 to 68)	0 (0 to 0)	1244 (1099 to 1412)	781 (681 to 893)	388 (322 to 457)	74 (39 to 130)	0 (0 to 0)
China	2023	683 (596 to 783)	433 (376 to 498)	209 (173 to 246)	40 (21 to 71)	0 (0 to 0)	1312 (1147 to 1505)	833 (722 to 957)	402 (333 to 473)	78 (40 to 136)	0 (0 to 0)
China	2024	719 (630 to 825)	461 (399 to 529)	216 (179 to 255)	42 (22 to 74)	0 (0 to 0)	1383 (1212 to 1586)	886 (768 to 1016)	416 (345 to 490)	81 (42 to 142)	0 (0 to 0)
China	2025	758 (657 to 872)	489 (422 to 564)	224 (186 to 264)	44 (23 to 77)	0 (0 to 0)	1457 (1264 to 1676)	941 (811 to 1084)	431 (357 to 508)	85 (44 to 149)	0 (0 to 0)
China	2026	798 (699 to 917)	520 (448 to 595)	232 (192 to 273)	46 (24 to 81)	0 (0 to 0)	1535 (1343 to 1764)	1000 (862 to 1143)	446 (369 to 526)	88 (46 to 155)	0 (0 to 0)
China	2027	840 (729 to 967)	552 (476 to 635)	240 (199 to 283)	48 (25 to 84)	0 (0 to 0)	1615 (1402 to 1858)	1062 (914 to 1220)	461 (382 to 543)	92 (48 to 161)	0 (0 to 0)

China	2028	885 (765 to 1012)	588 (504 to 679)	247 (205 to 292)	50 (26 to 87)	0 (0 to 0)	1701 (1471 to 1946)	1130 (969 to 1305)	475 (394 to 561)	96 (50 to 168)	0 (0 to 0)
China	2029	933 (811 to 1079)	627 (537 to 725)	254 (211 to 300)	52 (27 to 90)	0 (0 to 0)	1793 (1560 to 2074)	1205 (1032 to 1393)	489 (405 to 576)	100 (52 to 173)	0 (0 to 0)
China	2030	984 (850 to 1132)	669 (572 to 774)	261 (216 to 308)	54 (28 to 93)	0 (0 to 0)	1891 (1634 to 2176)	1286 (1099 to 1489)	502 (415 to 592)	103 (54 to 179)	0 (0 to 0)
China	2031	1038 (894 to 1196)	714 (610 to 828)	268 (222 to 317)	56 (29 to 96)	0 (0 to 0)	1995 (1718 to 2299)	1372 (1173 to 1591)	516 (426 to 609)	107 (56 to 185)	0 (0 to 0)
China	2032	1096 (947 to 1265)	762 (648 to 888)	276 (228 to 325)	58 (30 to 100)	0 (0 to 0)	2106 (1820 to 2433)	1465 (1245 to 1707)	530 (438 to 625)	111 (58 to 193)	0 (0 to 0)
China	2033	1156 (999 to 1345)	812 (688 to 950)	284 (234 to 334)	60 (31 to 104)	0 (0 to 0)	2223 (1920 to 2585)	1562 (1323 to 1826)	546 (450 to 643)	115 (61 to 201)	0 (0 to 0)
China	2034	1219 (1048 to 1410)	865 (732 to 1014)	292 (241 to 344)	62 (33 to 108)	0 (0 to 0)	2343 (2014 to 2711)	1662 (1408 to 1949)	562 (463 to 662)	120 (63 to 209)	0 (0 to 0)
China	2035	1286 (1106 to 1503)	920 (774 to 1081)	301 (248 to 355)	65 (34 to 113)	0 (0 to 0)	2472 (2126 to 2890)	1769 (1488 to 2077)	578 (477 to 683)	124 (65 to 218)	0 (0 to 0)
China	2036	1358 (1168 to 1571)	980 (829 to 1155)	310 (256 to 367)	67 (35 to 118)	0 (0 to 0)	2610 (2246 to 3020)	1884 (1593 to 2220)	597 (492 to 706)	129 (68 to 228)	0 (0 to 0)
China	2037	1437 (1235 to 1683)	1045 (879 to 1229)	322 (265 to 380)	70 (37 to 124)	0 (0 to 0)	2762 (2373 to 3235)	2009 (1690 to 2363)	618 (510 to 731)	135 (71 to 238)	0 (0 to 0)

China	2038	1516 (1301 to 1762)	1110 (932 to 1308)	332 (275 to 393)	73 (39 to 129)	0 (0 to 0)	2914 (2502 to 3387)	2134 (1792 to 2515)	639 (528 to 755)	141 (74 to 248)	0 (0 to 0)
China	2039	1598 (1362 to 1873)	1177 (983 to 1390)	344 (284 to 406)	77 (40 to 135)	0 (0 to 0)	3071 (2619 to 3602)	2263 (1890 to 2673)	661 (546 to 781)	147 (77 to 260)	0 (0 to 0)
China	2040	1680 (1438 to 1974)	1245 (1041 to 1474)	355 (294 to 421)	80 (42 to 141)	0 (0 to 0)	3230 (2765 to 3795)	2394 (2001 to 2833)	683 (565 to 809)	154 (81 to 270)	0 (0 to 0)
China	2041	1767 (1505 to 2069)	1316 (1102 to 1559)	367 (304 to 436)	83 (44 to 147)	0 (0 to 0)	3396 (2893 to 3977)	2530 (2119 to 2997)	706 (584 to 837)	160 (84 to 282)	0 (0 to 0)
China	2042	1855 (1573 to 2174)	1388 (1152 to 1641)	380 (314 to 451)	87 (45 to 153)	0 (0 to 0)	3566 (3025 to 4179)	2669 (2214 to 3155)	730 (603 to 867)	167 (87 to 294)	0 (0 to 0)
China	2043	1947 (1652 to 2302)	1464 (1218 to 1732)	393 (324 to 467)	91 (47 to 160)	0 (0 to 0)	3743 (3175 to 4425)	2814 (2341 to 3330)	755 (624 to 898)	174 (91 to 307)	0 (0 to 0)
China	2044	2042 (1733 to 2406)	1540 (1272 to 1837)	407 (335 to 483)	95 (50 to 167)	0 (0 to 0)	3925 (3331 to 4625)	2960 (2446 to 3532)	782 (644 to 928)	182 (95 to 322)	0 (0 to 0)
China	2045	2140 (1815 to 2532)	1620 (1341 to 1947)	421 (347 to 499)	99 (52 to 175)	0 (0 to 0)	4114 (3490 to 4867)	3113 (2577 to 3743)	810 (666 to 959)	190 (100 to 336)	0 (0 to 0)
China	2046	2239 (1894 to 2654)	1700 (1404 to 2043)	436 (359 to 516)	103 (54 to 182)	0 (0 to 0)	4305 (3641 to 5102)	3268 (2700 to 3928)	838 (689 to 992)	199 (104 to 351)	0 (0 to 0)
China	2047	2341 (1965 to 2777)	1784 (1470 to 2141)	450 (370 to 533)	108 (56 to 190)	0 (0 to 0)	4501 (3777 to 5338)	3429 (2825 to 4116)	865 (712 to 1025)	207 (108 to 365)	0 (0 to 0)

China	2048	2446 (2063 to 2903)	1870 (1539 to 2256)	464 (381 to 549)	112 (59 to 198)	0 (0 to 0)	4702 (3965 to 5581)	3595 (2960 to 4338)	891 (733 to 1055)	215 (113 to 380)	0 (0 to 0)
China	2049	2552 (2146 to 3040)	1959 (1608 to 2366)	477 (392 to 564)	116 (61 to 205)	0 (0 to 0)	4906 (4125 to 5845)	3767 (3092 to 4548)	916 (754 to 1084)	223 (117 to 394)	0 (0 to 0)
China	2050	2662 (2239 to 3173)	2052 (1688 to 2475)	489 (403 to 579)	120 (63 to 213)	0 (0 to 0)	5118 (4304 to 6100)	3946 (3245 to 4758)	941 (774 to 1113)	231 (122 to 409)	0 (0 to 0)
Colombia	1995	315 (279 to 357)	175 (151 to 201)	116 (93 to 143)	24 (14 to 37)	0 (0 to 0)	752 (666 to 852)	417 (361 to 479)	277 (221 to 342)	57 (35 to 87)	1 (1 to 1)
Colombia	1996	348 (312 to 388)	204 (179 to 231)	117 (95 to 142)	26 (16 to 39)	2 (2 to 2)	831 (744 to 927)	487 (428 to 551)	279 (226 to 339)	62 (38 to 92)	4 (4 to 4)
Colombia	1997	363 (327 to 400)	225 (200 to 251)	109 (88 to 132)	27 (18 to 41)	2 (2 to 2)	866 (781 to 954)	536 (477 to 600)	260 (211 to 316)	66 (42 to 97)	4 (4 to 4)
Colombia	1998	345 (313 to 378)	228 (204 to 253)	88 (72 to 106)	28 (18 to 41)	1 (1 to 1)	823 (747 to 902)	544 (488 to 604)	209 (172 to 253)	67 (44 to 99)	2 (2 to 2)
Colombia	1999	313 (286 to 343)	218 (197 to 241)	65 (53 to 80)	29 (19 to 41)	2 (2 to 2)	747 (682 to 818)	520 (471 to 576)	156 (126 to 190)	68 (46 to 97)	4 (4 to 4)
Colombia	2000	263 (239 to 289)	190 (173 to 209)	45 (37 to 56)	27 (18 to 38)	1 (1 to 1)	627 (571 to 690)	453 (412 to 499)	108 (88 to 133)	64 (43 to 90)	1 (1 to 1)
Colombia	2001	251 (230 to 275)	184 (168 to 203)	38 (30 to 48)	27 (18 to 39)	2 (2 to 2)	599 (548 to 656)	439 (400 to 484)	91 (72 to 114)	65 (44 to 92)	5 (5 to 5)
Colombia	2002	246 (224 to 270)	182 (164 to 200)	34 (27 to 43)	27 (18 to 39)	4 (4 to 4)	588 (535 to 643)	434 (392 to 478)	80 (64 to 101)	65 (44 to 93)	9 (9 to 9)
Colombia	2003	249 (226 to 272)	184 (165 to 202)	33 (26 to 41)	28 (19 to 40)	4 (4 to 4)	594 (540 to 648)	438 (394 to 482)	78 (61 to 98)	67 (45 to 96)	10 (10 to 10)
Colombia	2004	260 (236 to 284)	185 (165 to 203)	37 (28 to 47)	28 (19 to 40)	10 (10 to 10)	621 (564 to 677)	440 (394 to 485)	89 (68 to 112)	67 (46 to 95)	25 (25 to 25)
Colombia	2005	268 (243 to 293)	190 (170 to 210)	43 (33 to 54)	28 (20 to 39)	6 (6 to 6)	639 (580 to 698)	454 (407 to 502)	103 (79 to 129)	67 (47 to 94)	14 (14 to 14)

Colombia	2006	302 (275 to 330)	216 (195 to 240)	52 (41 to 65)	31 (21 to 43)	3 (3 to 3)	720 (656 to 787)	516 (465 to 572)	124 (98 to 154)	73 (51 to 103)	7 (7 to 7)
Colombia	2007	326 (298 to 356)	232 (210 to 255)	59 (47 to 73)	33 (23 to 47)	3 (3 to 3)	779 (712 to 850)	553 (501 to 608)	141 (113 to 175)	78 (55 to 112)	7 (7 to 7)
Colombia	2008	338 (309 to 369)	239 (217 to 263)	64 (51 to 78)	34 (24 to 48)	2 (2 to 2)	807 (738 to 882)	571 (519 to 628)	152 (122 to 186)	81 (57 to 115)	4 (4 to 4)
Colombia	2009	371 (342 to 403)	262 (239 to 287)	66 (54 to 80)	38 (27 to 53)	5 (5 to 5)	885 (816 to 961)	625 (570 to 684)	158 (128 to 192)	90 (64 to 126)	12 (12 to 12)
Colombia	2010	386 (356 to 418)	272 (248 to 296)	66 (54 to 80)	41 (29 to 56)	7 (7 to 7)	922 (850 to 998)	649 (593 to 707)	158 (129 to 191)	97 (69 to 133)	17 (17 to 17)
Colombia	2011	399 (369 to 433)	285 (262 to 311)	67 (55 to 81)	45 (32 to 60)	2 (2 to 2)	953 (880 to 1034)	681 (624 to 741)	160 (131 to 194)	106 (77 to 144)	6 (6 to 6)
Colombia	2012	409 (379 to 443)	289 (266 to 315)	70 (57 to 83)	48 (35 to 66)	2 (2 to 2)	976 (903 to 1058)	690 (635 to 751)	167 (135 to 199)	115 (84 to 157)	5 (5 to 5)
Colombia	2013	443 (409 to 477)	306 (281 to 331)	73 (59 to 88)	54 (40 to 73)	9 (9 to 9)	1056 (977 to 1139)	730 (670 to 791)	174 (142 to 211)	130 (94 to 174)	22 (22 to 22)
Colombia	2014	476 (441 to 512)	321 (297 to 348)	76 (61 to 92)	61 (44 to 81)	18 (18 to 18)	1135 (1052 to 1221)	767 (708 to 830)	181 (146 to 219)	145 (104 to 194)	42 (42 to 42)
Colombia	2015	476 (437 to 516)	331 (304 to 361)	77 (63 to 95)	67 (47 to 89)	0 (0 to 0)	1136 (1043 to 1232)	791 (725 to 861)	185 (150 to 226)	159 (113 to 214)	1 (1 to 1)
Colombia	2016	469 (428 to 515)	323 (292 to 354)	77 (61 to 96)	69 (48 to 94)	0 (0 to 0)	1120 (1022 to 1228)	771 (697 to 845)	184 (146 to 229)	164 (114 to 224)	1 (1 to 1)
Colombia	2017	481 (416 to 555)	329 (290 to 367)	78 (61 to 98)	73 (49 to 104)	1 (1 to 1)	1147 (992 to 1325)	784 (691 to 877)	187 (146 to 234)	175 (117 to 248)	1 (1 to 1)

Colombia	2018	491 (425 to 567)	337 (297 to 377)	79 (62 to 99)	75 (50 to 106)	0 (0 to 0)	1172 (1014 to 1353)	803 (708 to 900)	190 (148 to 237)	178 (119 to 252)	1 (1 to 1)
Colombia	2019	512 (442 to 593)	353 (312 to 396)	81 (64 to 102)	78 (52 to 110)	1 (1 to 1)	1222 (1056 to 1416)	842 (745 to 945)	194 (152 to 244)	185 (124 to 261)	1 (1 to 1)
Colombia	2020	534 (460 to 616)	369 (327 to 414)	84 (65 to 105)	81 (54 to 114)	1 (0 to 1)	1274 (1097 to 1471)	880 (780 to 989)	200 (156 to 250)	192 (129 to 273)	1 (1 to 1)
Colombia	2021	551 (475 to 635)	382 (337 to 431)	85 (66 to 106)	83 (55 to 118)	1 (0 to 1)	1314 (1133 to 1516)	912 (803 to 1029)	203 (159 to 254)	198 (132 to 281)	1 (1 to 1)
Colombia	2022	567 (491 to 655)	395 (348 to 446)	87 (68 to 108)	85 (57 to 121)	1 (0 to 1)	1353 (1171 to 1564)	942 (831 to 1064)	207 (161 to 259)	203 (136 to 289)	1 (1 to 1)
Colombia	2023	584 (504 to 677)	408 (358 to 461)	88 (69 to 110)	87 (58 to 124)	1 (0 to 1)	1394 (1202 to 1616)	974 (854 to 1099)	210 (164 to 263)	209 (139 to 296)	1 (1 to 1)
Colombia	2024	599 (516 to 695)	420 (368 to 475)	89 (70 to 112)	90 (60 to 127)	1 (0 to 1)	1430 (1232 to 1659)	1002 (878 to 1132)	213 (167 to 268)	214 (143 to 303)	1 (1 to 1)
Colombia	2025	615 (529 to 715)	432 (378 to 490)	91 (71 to 114)	92 (61 to 130)	1 (0 to 1)	1468 (1264 to 1707)	1032 (903 to 1169)	216 (169 to 272)	219 (146 to 311)	1 (1 to 1)
Colombia	2026	634 (542 to 735)	447 (392 to 506)	92 (72 to 116)	94 (63 to 133)	1 (0 to 1)	1513 (1293 to 1754)	1067 (936 to 1209)	220 (172 to 276)	225 (151 to 318)	1 (1 to 1)
Colombia	2027	652 (559 to 757)	462 (404 to 525)	93 (73 to 117)	97 (65 to 137)	1 (0 to 1)	1556 (1334 to 1807)	1101 (964 to 1253)	223 (175 to 280)	231 (155 to 326)	1 (1 to 2)

Colombia	2028	671 (572 to 779)	477 (417 to 541)	95 (74 to 119)	99 (67 to 140)	1 (0 to 1)	1601 (1365 to 1859)	1137 (994 to 1292)	226 (177 to 284)	237 (159 to 335)	1 (1 to 2)
Colombia	2029	690 (592 to 801)	492 (429 to 559)	96 (75 to 120)	102 (68 to 144)	1 (0 to 1)	1646 (1413 to 1912)	1173 (1024 to 1335)	229 (179 to 287)	243 (163 to 345)	1 (1 to 2)
Colombia	2030	709 (605 to 827)	507 (443 to 576)	97 (76 to 122)	104 (70 to 148)	1 (0 to 1)	1691 (1445 to 1973)	1210 (1057 to 1374)	232 (181 to 291)	249 (167 to 354)	1 (1 to 2)
Colombia	2031	727 (622 to 848)	522 (456 to 595)	98 (77 to 124)	107 (71 to 152)	1 (1 to 1)	1736 (1483 to 2025)	1246 (1089 to 1419)	235 (184 to 295)	255 (171 to 362)	1 (1 to 2)
Colombia	2032	747 (637 to 873)	537 (469 to 613)	100 (79 to 126)	109 (73 to 155)	1 (1 to 1)	1781 (1519 to 2082)	1282 (1119 to 1463)	238 (187 to 300)	261 (175 to 370)	1 (1 to 2)
Colombia	2033	766 (652 to 896)	553 (480 to 631)	101 (79 to 128)	112 (75 to 159)	1 (1 to 1)	1827 (1556 to 2139)	1319 (1145 to 1505)	241 (190 to 305)	267 (180 to 380)	1 (1 to 2)
Colombia	2034	785 (669 to 919)	568 (494 to 651)	102 (81 to 130)	114 (77 to 163)	1 (1 to 1)	1874 (1597 to 2194)	1356 (1179 to 1552)	244 (192 to 309)	273 (184 to 389)	1 (1 to 2)
Colombia	2035	805 (682 to 942)	584 (503 to 667)	104 (82 to 132)	117 (79 to 167)	1 (1 to 1)	1921 (1628 to 2248)	1393 (1200 to 1592)	247 (195 to 314)	279 (189 to 398)	1 (1 to 2)
Colombia	2036	825 (701 to 969)	599 (519 to 688)	105 (83 to 134)	120 (81 to 171)	1 (1 to 1)	1968 (1674 to 2312)	1430 (1238 to 1642)	250 (197 to 319)	286 (194 to 408)	2 (1 to 2)
Colombia	2037	844 (715 to 989)	615 (529 to 703)	106 (84 to 136)	122 (83 to 175)	1 (1 to 1)	2015 (1706 to 2361)	1468 (1262 to 1679)	254 (199 to 324)	292 (199 to 418)	2 (1 to 2)

Colombia	2038	864 (731 to 1016)	631 (544 to 725)	108 (84 to 138)	125 (85 to 179)	1 (1 to 1)	2063 (1744 to 2424)	1505 (1299 to 1729)	257 (202 to 329)	299 (203 to 426)	2 (1 to 2)
Colombia	2039	884 (748 to 1047)	647 (558 to 745)	109 (86 to 140)	128 (87 to 183)	1 (1 to 1)	2111 (1786 to 2499)	1543 (1331 to 1778)	261 (204 to 335)	305 (208 to 436)	2 (1 to 2)
Colombia	2040	905 (766 to 1068)	663 (571 to 763)	111 (87 to 142)	131 (89 to 187)	1 (1 to 1)	2160 (1829 to 2548)	1582 (1362 to 1821)	264 (207 to 339)	312 (212 to 445)	2 (1 to 2)
Colombia	2041	926 (783 to 1091)	679 (583 to 786)	112 (88 to 144)	133 (91 to 191)	1 (1 to 1)	2209 (1868 to 2604)	1621 (1392 to 1875)	267 (209 to 344)	318 (217 to 455)	2 (1 to 2)
Colombia	2042	947 (802 to 1118)	696 (598 to 800)	113 (89 to 146)	136 (93 to 195)	1 (1 to 1)	2259 (1913 to 2668)	1661 (1428 to 1910)	271 (211 to 348)	325 (221 to 465)	2 (1 to 2)
Colombia	2043	968 (819 to 1142)	713 (615 to 822)	115 (89 to 148)	139 (95 to 199)	1 (1 to 1)	2310 (1954 to 2725)	1702 (1468 to 1962)	274 (213 to 353)	332 (226 to 475)	2 (1 to 2)
Colombia	2044	989 (836 to 1164)	730 (626 to 840)	116 (90 to 151)	142 (97 to 204)	1 (1 to 1)	2360 (1996 to 2778)	1742 (1494 to 2003)	277 (216 to 359)	339 (231 to 486)	2 (1 to 2)
Colombia	2045	1010 (854 to 1193)	747 (641 to 860)	118 (91 to 152)	145 (98 to 208)	1 (1 to 1)	2410 (2039 to 2846)	1783 (1530 to 2051)	280 (218 to 364)	346 (235 to 497)	2 (1 to 2)
Colombia	2046	1031 (873 to 1222)	764 (655 to 881)	119 (92 to 154)	148 (101 to 213)	1 (1 to 1)	2461 (2083 to 2915)	1823 (1564 to 2103)	284 (220 to 369)	352 (240 to 508)	2 (1 to 2)
Colombia	2047	1053 (893 to 1245)	782 (670 to 903)	120 (93 to 156)	151 (103 to 217)	1 (1 to 1)	2514 (2132 to 2970)	1865 (1600 to 2156)	287 (222 to 373)	359 (245 to 519)	2 (1 to 2)

Colombia	2048	1075 (912 to 1273)	799 (685 to 928)	122 (94 to 158)	154 (105 to 222)	1 (1 to 1)	2566 (2175 to 3039)	1908 (1634 to 2214)	290 (224 to 378)	367 (250 to 530)	2 (1 to 2)
Colombia	2049	1097 (928 to 1299)	817 (700 to 945)	123 (95 to 160)	157 (107 to 227)	1 (1 to 1)	2618 (2214 to 3100)	1950 (1671 to 2256)	293 (226 to 382)	374 (255 to 541)	2 (1 to 3)
Colombia	2050	1119 (948 to 1330)	835 (714 to 967)	124 (96 to 162)	160 (108 to 231)	1 (1 to 1)	2671 (2262 to 3175)	1992 (1704 to 2309)	296 (228 to 386)	381 (259 to 551)	2 (2 to 3)
Comoros	1995	101 (84 to 122)	11 (8 to 14)	69 (52 to 90)	2 (1 to 3)	19 (19 to 19)	205 (170 to 247)	22 (16 to 29)	140 (106 to 182)	3 (1 to 6)	39 (39 to 39)
Comoros	1996	85 (70 to 105)	10 (7 to 13)	67 (51 to 87)	1 (1 to 3)	7 (7 to 7)	173 (142 to 213)	21 (15 to 27)	136 (104 to 177)	3 (1 to 5)	13 (13 to 13)
Comoros	1997	92 (77 to 110)	10 (8 to 14)	68 (53 to 85)	1 (1 to 3)	13 (13 to 13)	187 (157 to 223)	21 (16 to 28)	137 (107 to 173)	3 (1 to 5)	26 (26 to 26)
Comoros	1998	96 (82 to 113)	10 (7 to 13)	66 (52 to 83)	1 (1 to 2)	19 (19 to 19)	195 (166 to 229)	20 (15 to 27)	134 (106 to 168)	3 (1 to 5)	38 (38 to 38)
Comoros	1999	80 (66 to 97)	10 (7 to 13)	66 (52 to 82)	1 (1 to 2)	3 (3 to 3)	162 (135 to 196)	20 (15 to 26)	134 (107 to 167)	2 (1 to 4)	6 (6 to 6)
Comoros	2000	80 (66 to 95)	10 (7 to 12)	65 (52 to 81)	1 (0 to 2)	4 (4 to 4)	161 (134 to 192)	19 (15 to 25)	132 (106 to 164)	2 (1 to 4)	7 (7 to 7)
Comoros	2001	86 (72 to 102)	11 (9 to 15)	70 (57 to 86)	1 (1 to 2)	4 (4 to 4)	175 (146 to 208)	23 (18 to 30)	142 (115 to 174)	3 (1 to 5)	7 (7 to 7)
Comoros	2002	94 (79 to 111)	13 (10 to 17)	71 (58 to 89)	1 (1 to 3)	8 (8 to 8)	190 (161 to 225)	26 (20 to 34)	145 (117 to 180)	3 (1 to 5)	16 (16 to 16)
Comoros	2003	92 (78 to 107)	12 (9 to 15)	67 (55 to 82)	1 (1 to 2)	11 (11 to 11)	186 (157 to 218)	24 (18 to 31)	137 (111 to 167)	3 (1 to 5)	23 (23 to 23)
Comoros	2004	85 (72 to 100)	11 (8 to 14)	65 (53 to 79)	1 (1 to 2)	8 (8 to 8)	172 (145 to 204)	22 (17 to 29)	132 (107 to 161)	2 (1 to 4)	16 (16 to 16)
Comoros	2005	81 (68 to 96)	11 (8 to 14)	64 (52 to 79)	1 (1 to 2)	5 (5 to 5)	165 (137 to 195)	21 (16 to 28)	131 (105 to 160)	2 (1 to 4)	10 (10 to 10)

Comoros	2006	78 (65 to 93)	10 (8 to 13)	63 (50 to 78)	1 (1 to 2)	4 (4 to 4)	159 (132 to 188)	20 (15 to 27)	128 (102 to 159)	2 (1 to 4)	8 (8 to 8)
Comoros	2007	73 (61 to 88)	9 (7 to 12)	60 (47 to 75)	1 (1 to 2)	4 (4 to 4)	149 (124 to 179)	18 (14 to 24)	121 (96 to 152)	2 (1 to 4)	8 (8 to 8)
Comoros	2008	74 (61 to 89)	9 (7 to 12)	61 (47 to 75)	1 (1 to 2)	3 (3 to 3)	150 (123 to 181)	18 (14 to 24)	123 (96 to 152)	2 (1 to 4)	6 (6 to 6)
Comoros	2009	73 (60 to 88)	8 (6 to 10)	57 (45 to 72)	1 (1 to 2)	7 (7 to 7)	149 (122 to 178)	16 (12 to 21)	116 (90 to 146)	2 (1 to 4)	14 (14 to 14)
Comoros	2010	84 (72 to 99)	8 (6 to 10)	56 (44 to 70)	1 (1 to 2)	19 (19 to 19)	171 (145 to 200)	17 (12 to 21)	114 (89 to 143)	2 (1 to 4)	38 (38 to 38)
Comoros	2011	78 (65 to 93)	9 (7 to 12)	56 (44 to 71)	1 (1 to 2)	11 (11 to 11)	158 (132 to 188)	18 (14 to 24)	114 (89 to 143)	2 (1 to 4)	23 (23 to 23)
Comoros	2012	88 (75 to 102)	9 (7 to 12)	56 (44 to 71)	1 (1 to 2)	21 (21 to 21)	178 (153 to 207)	19 (14 to 24)	114 (89 to 143)	2 (1 to 4)	43 (43 to 43)
Comoros	2013	85 (72 to 100)	9 (7 to 12)	56 (44 to 70)	1 (1 to 2)	18 (18 to 18)	172 (146 to 202)	19 (14 to 24)	113 (88 to 142)	2 (1 to 5)	37 (37 to 37)
Comoros	2014	76 (63 to 90)	9 (7 to 12)	54 (42 to 69)	1 (1 to 2)	12 (12 to 12)	154 (129 to 184)	18 (14 to 24)	110 (85 to 139)	2 (1 to 5)	23 (23 to 23)
Comoros	2015	74 (61 to 89)	10 (8 to 13)	56 (44 to 72)	1 (1 to 2)	7 (7 to 7)	150 (125 to 181)	20 (15 to 26)	113 (88 to 145)	3 (1 to 5)	14 (14 to 14)
Comoros	2016	84 (71 to 100)	10 (8 to 13)	56 (43 to 71)	1 (1 to 3)	16 (16 to 16)	169 (143 to 202)	21 (16 to 27)	113 (87 to 145)	3 (1 to 5)	33 (33 to 33)
Comoros	2017	74 (60 to 91)	10 (7 to 12)	54 (40 to 70)	1 (1 to 2)	9 (9 to 9)	150 (122 to 185)	20 (15 to 25)	109 (81 to 143)	3 (1 to 5)	19 (19 to 19)
Comoros	2018	73 (60 to 89)	10 (7 to 12)	53 (40 to 70)	1 (1 to 2)	9 (9 to 9)	149 (121 to 182)	19 (15 to 25)	109 (81 to 142)	3 (1 to 5)	18 (18 to 18)
Comoros	2019	76 (62 to 93)	12 (9 to 16)	53 (40 to 70)	1 (1 to 3)	9 (9 to 9)	155 (126 to 189)	25 (18 to 33)	108 (81 to 141)	3 (1 to 6)	19 (19 to 19)
Comoros	2020	77 (64 to 94)	12 (9 to 16)	54 (40 to 70)	1 (1 to 3)	10 (9 to 10)	157 (129 to 191)	25 (19 to 33)	109 (82 to 143)	3 (1 to 6)	20 (18 to 21)

Comoros	2021	78 (64 to 95)	13 (9 to 17)	54 (41 to 71)	2 (1 to 3)	10 (9 to 11)	159 (131 to 193)	26 (19 to 34)	110 (82 to 144)	3 (1 to 6)	20 (19 to 22)
Comoros	2022	79 (65 to 96)	13 (9 to 17)	55 (41 to 71)	2 (1 to 3)	10 (9 to 11)	161 (132 to 195)	26 (19 to 35)	111 (83 to 145)	3 (1 to 6)	21 (19 to 23)
Comoros	2023	80 (65 to 98)	13 (10 to 17)	55 (41 to 72)	2 (1 to 3)	10 (9 to 12)	162 (133 to 199)	27 (19 to 35)	111 (84 to 146)	3 (1 to 6)	21 (19 to 23)
Comoros	2024	81 (66 to 98)	13 (10 to 18)	55 (42 to 72)	2 (1 to 3)	10 (9 to 12)	164 (135 to 199)	27 (20 to 36)	112 (84 to 147)	3 (1 to 6)	21 (19 to 24)
Comoros	2025	82 (67 to 99)	14 (10 to 18)	56 (42 to 73)	2 (1 to 3)	11 (9 to 12)	166 (136 to 201)	28 (20 to 37)	113 (85 to 148)	3 (2 to 6)	22 (19 to 25)
Comoros	2026	82 (68 to 100)	14 (10 to 19)	56 (42 to 73)	2 (1 to 3)	11 (10 to 12)	167 (137 to 203)	28 (20 to 38)	114 (86 to 149)	3 (2 to 6)	22 (19 to 25)
Comoros	2027	83 (68 to 101)	14 (10 to 19)	56 (42 to 74)	2 (1 to 3)	11 (10 to 13)	169 (138 to 205)	29 (21 to 39)	115 (86 to 150)	3 (2 to 6)	22 (19 to 26)
Comoros	2028	84 (69 to 102)	14 (10 to 19)	57 (43 to 74)	2 (1 to 3)	11 (10 to 13)	170 (140 to 206)	29 (21 to 39)	115 (87 to 151)	3 (2 to 6)	23 (20 to 27)
Comoros	2029	85 (70 to 104)	15 (10 to 20)	57 (43 to 75)	2 (1 to 3)	11 (10 to 13)	172 (141 to 210)	30 (21 to 40)	116 (87 to 152)	4 (2 to 7)	23 (20 to 27)
Comoros	2030	85 (70 to 104)	15 (11 to 20)	57 (43 to 75)	2 (1 to 3)	12 (10 to 14)	173 (142 to 211)	30 (21 to 41)	116 (87 to 153)	4 (2 to 7)	23 (20 to 28)
Comoros	2031	86 (70 to 106)	15 (11 to 20)	58 (43 to 76)	2 (1 to 3)	12 (10 to 14)	175 (143 to 214)	31 (22 to 41)	117 (88 to 154)	4 (2 to 7)	24 (20 to 29)
Comoros	2032	87 (71 to 106)	15 (11 to 21)	58 (43 to 76)	2 (1 to 3)	12 (10 to 15)	177 (145 to 215)	31 (22 to 42)	118 (88 to 155)	4 (2 to 7)	24 (20 to 30)
Comoros	2033	88 (72 to 107)	16 (11 to 21)	58 (44 to 77)	2 (1 to 3)	12 (10 to 15)	178 (145 to 217)	32 (23 to 43)	118 (88 to 155)	4 (2 to 7)	25 (20 to 31)
Comoros	2034	88 (73 to 108)	16 (11 to 22)	58 (44 to 77)	2 (1 to 3)	13 (10 to 15)	180 (147 to 219)	32 (23 to 44)	118 (89 to 156)	4 (2 to 7)	25 (20 to 31)
Comoros	2035	89 (73 to 108)	16 (11 to 22)	59 (44 to 77)	2 (1 to 4)	13 (10 to 16)	181 (148 to 220)	32 (23 to 45)	119 (89 to 156)	4 (2 to 7)	26 (20 to 32)

Comoros	2036	90 (74 to 109)	16 (11 to 22)	59 (44 to 77)	2 (1 to 4)	13 (10 to 16)	182 (150 to 222)	33 (23 to 45)	119 (89 to 157)	4 (2 to 7)	27 (20 to 33)
Comoros	2037	91 (74 to 110)	16 (11 to 23)	59 (44 to 78)	2 (1 to 4)	13 (10 to 17)	184 (150 to 224)	33 (23 to 47)	119 (90 to 157)	4 (2 to 7)	27 (21 to 34)
Comoros	2038	91 (74 to 111)	17 (11 to 23)	59 (44 to 78)	2 (1 to 4)	14 (10 to 18)	185 (151 to 226)	34 (23 to 47)	120 (90 to 158)	4 (2 to 7)	28 (21 to 36)
Comoros	2039	92 (75 to 112)	17 (12 to 24)	59 (44 to 78)	2 (1 to 4)	14 (10 to 18)	187 (153 to 228)	34 (24 to 48)	120 (90 to 158)	4 (2 to 7)	28 (21 to 36)
Comoros	2040	93 (76 to 113)	17 (12 to 24)	59 (44 to 78)	2 (1 to 4)	14 (11 to 18)	188 (153 to 230)	35 (24 to 49)	120 (90 to 158)	4 (2 to 8)	29 (22 to 37)
Comoros	2041	93 (77 to 114)	17 (12 to 24)	59 (44 to 78)	2 (1 to 4)	15 (11 to 19)	189 (155 to 230)	35 (24 to 49)	120 (90 to 159)	4 (2 to 8)	30 (22 to 38)
Comoros	2042	94 (77 to 115)	18 (12 to 24)	59 (44 to 78)	2 (1 to 4)	15 (11 to 20)	191 (156 to 233)	36 (24 to 49)	120 (90 to 159)	4 (2 to 8)	31 (22 to 40)
Comoros	2043	95 (78 to 115)	18 (12 to 25)	59 (45 to 78)	2 (1 to 4)	16 (11 to 21)	192 (158 to 234)	36 (24 to 51)	120 (91 to 159)	4 (2 to 8)	32 (22 to 42)
Comoros	2044	95 (78 to 116)	18 (12 to 25)	59 (45 to 78)	2 (1 to 4)	16 (11 to 21)	193 (159 to 235)	36 (24 to 51)	120 (91 to 159)	4 (2 to 8)	32 (23 to 43)
Comoros	2045	96 (79 to 117)	18 (12 to 25)	59 (45 to 78)	2 (1 to 4)	16 (11 to 22)	194 (160 to 237)	37 (25 to 51)	120 (91 to 159)	4 (2 to 8)	33 (23 to 44)
Comoros	2046	96 (78 to 118)	18 (12 to 26)	59 (45 to 78)	2 (1 to 4)	17 (11 to 22)	196 (159 to 240)	37 (25 to 52)	120 (91 to 159)	4 (2 to 8)	34 (23 to 45)
Comoros	2047	97 (80 to 118)	19 (12 to 26)	59 (45 to 78)	2 (1 to 4)	17 (11 to 23)	197 (163 to 239)	38 (25 to 53)	120 (91 to 158)	4 (2 to 8)	35 (23 to 47)
Comoros	2048	98 (79 to 120)	19 (12 to 27)	59 (45 to 78)	2 (1 to 4)	18 (12 to 24)	199 (161 to 243)	38 (25 to 54)	120 (90 to 158)	4 (2 to 8)	36 (24 to 50)
Comoros	2049	99 (80 to 120)	19 (12 to 27)	59 (44 to 78)	2 (1 to 4)	18 (12 to 25)	200 (162 to 244)	38 (25 to 55)	120 (90 to 158)	4 (2 to 8)	37 (24 to 50)
Comoros	2050	99 (80 to 121)	19 (13 to 27)	59 (44 to 78)	2 (1 to 4)	19 (12 to 26)	201 (163 to 245)	39 (26 to 56)	120 (90 to 159)	4 (2 to 8)	38 (24 to 53)

Congo (Brazzaville)	1995	46 (37 to 56)	17 (13 to 22)	24 (17 to 32)	1 (0 to 1)	4 (4 to 4)	135 (110 to 165)	50 (38 to 66)	70 (49 to 96)	2 (1 to 4)	13 (13 to 13)
Congo (Brazzaville)	1996	36 (28 to 45)	14 (10 to 18)	21 (15 to 29)	1 (0 to 1)	0 (0 to 0)	106 (85 to 132)	41 (31 to 54)	63 (44 to 85)	2 (1 to 3)	1 (1 to 1)
Congo (Brazzaville)	1997	40 (32 to 49)	15 (12 to 20)	22 (16 to 30)	1 (0 to 1)	2 (2 to 2)	118 (95 to 146)	46 (34 to 59)	66 (46 to 88)	2 (1 to 3)	5 (5 to 5)
Congo (Brazzaville)	1998	41 (33 to 50)	15 (12 to 20)	23 (16 to 31)	1 (0 to 1)	2 (2 to 2)	121 (97 to 148)	45 (34 to 59)	67 (47 to 91)	2 (1 to 4)	6 (6 to 6)
Congo (Brazzaville)	1999	38 (30 to 47)	14 (11 to 19)	22 (15 to 30)	1 (0 to 1)	1 (1 to 1)	111 (88 to 139)	43 (33 to 56)	65 (45 to 89)	2 (1 to 3)	2 (2 to 2)
Congo (Brazzaville)	2000	34 (27 to 43)	13 (10 to 17)	20 (14 to 28)	1 (0 to 1)	0 (0 to 0)	102 (81 to 128)	39 (29 to 50)	61 (43 to 83)	2 (1 to 3)	1 (1 to 1)
Congo (Brazzaville)	2001	37 (30 to 46)	14 (11 to 18)	22 (15 to 30)	1 (0 to 1)	0 (0 to 0)	110 (88 to 137)	42 (32 to 54)	65 (46 to 88)	2 (1 to 4)	1 (1 to 1)
Congo (Brazzaville)	2002	37 (30 to 46)	13 (10 to 17)	23 (16 to 31)	1 (0 to 1)	1 (1 to 1)	110 (89 to 137)	39 (29 to 49)	67 (48 to 91)	2 (1 to 4)	3 (3 to 3)
Congo (Brazzaville)	2003	34 (27 to 42)	11 (8 to 14)	22 (15 to 29)	1 (0 to 1)	1 (1 to 1)	101 (81 to 126)	33 (25 to 42)	64 (46 to 86)	2 (1 to 3)	3 (3 to 3)
Congo (Brazzaville)	2004	34 (28 to 42)	10 (8 to 13)	20 (15 to 28)	1 (0 to 1)	2 (2 to 2)	100 (82 to 123)	31 (23 to 39)	61 (44 to 82)	2 (1 to 3)	7 (7 to 7)

Congo (Brazzaville)	2005	33 (27 to 41)	11 (8 to 14)	20 (14 to 26)	1 (0 to 1)	2 (2 to 2)	99 (80 to 120)	32 (24 to 41)	58 (42 to 78)	2 (1 to 3)	7 (7 to 7)
Congo (Brazzaville)	2006	37 (30 to 45)	13 (10 to 17)	20 (15 to 27)	1 (0 to 1)	3 (3 to 3)	109 (89 to 133)	39 (30 to 51)	60 (44 to 81)	2 (1 to 4)	8 (8 to 8)
Congo (Brazzaville)	2007	40 (33 to 48)	15 (12 to 20)	21 (15 to 28)	1 (0 to 1)	2 (2 to 2)	118 (97 to 142)	46 (35 to 58)	63 (46 to 84)	2 (1 to 4)	7 (7 to 7)
Congo (Brazzaville)	2008	42 (35 to 51)	17 (12 to 21)	21 (15 to 28)	1 (0 to 1)	4 (4 to 4)	125 (104 to 150)	49 (37 to 63)	61 (45 to 82)	2 (1 to 4)	13 (13 to 13)
Congo (Brazzaville)	2009	38 (31 to 46)	15 (11 to 19)	20 (14 to 27)	1 (0 to 1)	3 (3 to 3)	113 (93 to 137)	45 (34 to 58)	58 (42 to 79)	2 (1 to 3)	9 (9 to 9)
Congo (Brazzaville)	2010	44 (37 to 53)	17 (13 to 22)	19 (14 to 26)	1 (0 to 1)	7 (7 to 7)	131 (109 to 156)	51 (38 to 65)	56 (40 to 76)	2 (1 to 3)	22 (22 to 22)
Congo (Brazzaville)	2011	45 (38 to 54)	19 (15 to 25)	19 (13 to 25)	1 (0 to 1)	7 (7 to 7)	135 (112 to 161)	57 (43 to 74)	55 (39 to 75)	2 (1 to 3)	20 (20 to 20)
Congo (Brazzaville)	2012	52 (43 to 62)	24 (18 to 31)	19 (14 to 26)	1 (0 to 1)	7 (7 to 7)	153 (128 to 183)	71 (53 to 93)	58 (41 to 77)	2 (1 to 4)	22 (22 to 22)
Congo (Brazzaville)	2013	57 (48 to 69)	29 (22 to 38)	21 (15 to 28)	1 (1 to 2)	6 (6 to 6)	170 (141 to 205)	87 (65 to 112)	62 (44 to 84)	3 (1 to 6)	18 (18 to 18)
Congo (Brazzaville)	2014	61 (50 to 74)	33 (25 to 43)	23 (16 to 32)	2 (1 to 3)	2 (2 to 2)	180 (148 to 218)	99 (75 to 127)	69 (49 to 94)	5 (2 to 9)	7 (7 to 7)

Congo (Brazzaville)	2015	64 (53 to 77)	33 (25 to 43)	26 (18 to 35)	2 (1 to 3)	3 (3 to 3)	190 (157 to 229)	99 (75 to 127)	77 (54 to 105)	6 (3 to 10)	8 (8 to 8)
Congo (Brazzaville)	2016	68 (56 to 82)	33 (25 to 43)	29 (21 to 40)	2 (1 to 4)	3 (3 to 3)	201 (166 to 243)	98 (75 to 127)	87 (61 to 119)	6 (3 to 12)	10 (10 to 10)
Congo (Brazzaville)	2017	59 (48 to 71)	26 (20 to 33)	27 (19 to 37)	2 (1 to 3)	4 (4 to 4)	175 (143 to 210)	76 (59 to 98)	81 (57 to 110)	5 (2 to 10)	12 (12 to 12)
Congo (Brazzaville)	2018	52 (41 to 63)	20 (15 to 26)	27 (19 to 36)	2 (1 to 3)	4 (4 to 4)	153 (121 to 187)	59 (45 to 77)	79 (55 to 107)	5 (2 to 9)	11 (11 to 11)
Congo (Brazzaville)	2019	54 (43 to 65)	21 (16 to 28)	27 (19 to 37)	2 (1 to 3)	4 (4 to 4)	159 (128 to 194)	63 (47 to 83)	80 (56 to 108)	5 (2 to 9)	11 (11 to 11)
Congo (Brazzaville)	2020	55 (43 to 67)	22 (16 to 29)	27 (19 to 37)	2 (1 to 3)	4 (4 to 4)	162 (127 to 198)	65 (47 to 87)	80 (56 to 109)	5 (2 to 9)	12 (11 to 12)
Congo (Brazzaville)	2021	56 (44 to 68)	22 (16 to 31)	27 (19 to 37)	2 (1 to 3)	4 (4 to 4)	165 (132 to 201)	67 (48 to 91)	81 (57 to 110)	5 (2 to 10)	12 (11 to 13)
Congo (Brazzaville)	2022	57 (44 to 70)	23 (16 to 32)	28 (19 to 38)	2 (1 to 3)	4 (4 to 5)	168 (131 to 209)	68 (48 to 95)	82 (58 to 112)	5 (2 to 10)	12 (11 to 14)
Congo (Brazzaville)	2023	58 (46 to 71)	23 (16 to 33)	28 (20 to 38)	2 (1 to 3)	4 (4 to 5)	171 (136 to 212)	70 (47 to 99)	84 (59 to 114)	5 (2 to 10)	12 (11 to 14)
Congo (Brazzaville)	2024	58 (45 to 74)	24 (16 to 34)	29 (20 to 39)	2 (1 to 3)	4 (4 to 5)	174 (135 to 218)	71 (48 to 101)	85 (60 to 115)	5 (2 to 10)	13 (11 to 14)

Congo (Brazzaville)	2025	59 (47 to 73)	24 (16 to 35)	29 (20 to 39)	2 (1 to 3)	4 (4 to 5)	176 (139 to 218)	71 (49 to 103)	86 (61 to 117)	5 (2 to 10)	13 (11 to 14)
Congo (Brazzaville)	2026	60 (46 to 76)	25 (16 to 36)	29 (21 to 40)	2 (1 to 4)	4 (4 to 5)	179 (137 to 226)	73 (48 to 105)	87 (61 to 118)	6 (3 to 11)	13 (11 to 15)
Congo (Brazzaville)	2027	61 (48 to 77)	25 (16 to 36)	30 (21 to 40)	2 (1 to 4)	4 (4 to 5)	182 (144 to 228)	74 (49 to 108)	89 (62 to 120)	6 (3 to 11)	13 (11 to 15)
Congo (Brazzaville)	2028	62 (48 to 79)	26 (17 to 37)	30 (21 to 41)	2 (1 to 4)	4 (4 to 5)	185 (141 to 235)	76 (50 to 110)	90 (63 to 122)	6 (3 to 11)	13 (11 to 15)
Congo (Brazzaville)	2029	63 (49 to 80)	26 (17 to 39)	31 (22 to 42)	2 (1 to 4)	4 (4 to 5)	188 (147 to 237)	78 (50 to 115)	91 (64 to 123)	6 (3 to 11)	13 (11 to 15)
Congo (Brazzaville)	2030	64 (49 to 84)	27 (17 to 40)	31 (22 to 42)	2 (1 to 4)	4 (4 to 5)	191 (147 to 248)	79 (50 to 119)	93 (65 to 125)	6 (3 to 11)	13 (11 to 16)
Congo (Brazzaville)	2031	66 (51 to 84)	27 (17 to 41)	32 (22 to 43)	2 (1 to 4)	5 (4 to 5)	195 (152 to 248)	81 (51 to 123)	94 (66 to 128)	6 (3 to 12)	13 (11 to 16)
Congo (Brazzaville)	2032	67 (51 to 88)	28 (17 to 43)	32 (23 to 44)	2 (1 to 4)	5 (4 to 6)	198 (150 to 260)	83 (51 to 127)	96 (67 to 130)	6 (3 to 12)	14 (12 to 16)
Congo (Brazzaville)	2033	68 (53 to 87)	28 (17 to 44)	33 (23 to 44)	2 (1 to 4)	5 (4 to 6)	202 (156 to 258)	84 (51 to 132)	97 (68 to 131)	6 (3 to 12)	14 (12 to 17)
Congo (Brazzaville)	2034	69 (52 to 91)	29 (18 to 45)	33 (23 to 45)	2 (1 to 4)	5 (4 to 6)	205 (155 to 270)	86 (53 to 134)	99 (69 to 133)	6 (3 to 12)	14 (12 to 17)

Congo (Brazzaville)	2035	70 (54 to 90)	29 (18 to 47)	34 (24 to 46)	2 (1 to 4)	5 (4 to 6)	208 (160 to 268)	87 (53 to 139)	100 (71 to 136)	7 (3 to 13)	14 (12 to 17)
Congo (Brazzaville)	2036	71 (53 to 94)	30 (18 to 47)	34 (24 to 46)	2 (1 to 4)	5 (4 to 6)	211 (157 to 279)	89 (52 to 141)	102 (72 to 137)	7 (3 to 13)	14 (12 to 18)
Congo (Brazzaville)	2037	72 (55 to 93)	30 (18 to 49)	35 (25 to 47)	2 (1 to 4)	5 (4 to 6)	214 (163 to 277)	90 (54 to 145)	103 (73 to 139)	7 (3 to 13)	14 (12 to 18)
Congo (Brazzaville)	2038	73 (55 to 98)	31 (18 to 50)	35 (25 to 48)	2 (1 to 5)	5 (4 to 6)	217 (162 to 292)	91 (54 to 147)	104 (74 to 141)	7 (3 to 13)	15 (12 to 18)
Congo (Brazzaville)	2039	74 (57 to 97)	31 (18 to 50)	36 (25 to 48)	2 (1 to 5)	5 (4 to 6)	220 (168 to 288)	93 (54 to 149)	105 (74 to 144)	7 (3 to 14)	15 (12 to 19)
Congo (Brazzaville)	2040	75 (55 to 101)	32 (18 to 51)	36 (25 to 49)	2 (1 to 5)	5 (4 to 6)	223 (165 to 299)	94 (54 to 152)	106 (75 to 145)	7 (3 to 14)	15 (12 to 19)
Congo (Brazzaville)	2041	76 (57 to 99)	32 (18 to 52)	36 (25 to 50)	2 (1 to 5)	5 (4 to 7)	225 (169 to 292)	95 (55 to 155)	107 (75 to 147)	7 (3 to 14)	15 (12 to 19)
Congo (Brazzaville)	2042	77 (57 to 104)	32 (19 to 53)	36 (26 to 50)	2 (1 to 5)	5 (4 to 7)	227 (168 to 309)	96 (56 to 156)	108 (76 to 148)	7 (3 to 14)	16 (13 to 20)
Congo (Brazzaville)	2043	77 (58 to 103)	33 (19 to 53)	37 (26 to 50)	2 (1 to 5)	5 (4 to 7)	230 (173 to 304)	97 (55 to 158)	109 (76 to 149)	7 (3 to 14)	16 (13 to 20)
Congo (Brazzaville)	2044	78 (57 to 106)	33 (18 to 54)	37 (26 to 51)	3 (1 to 5)	5 (4 to 7)	232 (170 to 313)	98 (55 to 162)	110 (77 to 150)	7 (3 to 14)	16 (13 to 21)

Congo (Brazzaville)	2045	79 (59 to 105)	33 (18 to 55)	37 (26 to 51)	3 (1 to 5)	5 (4 to 7)	233 (176 to 312)	99 (54 to 163)	110 (77 to 151)	8 (3 to 14)	16 (13 to 21)
Congo (Brazzaville)	2046	79 (58 to 108)	34 (19 to 56)	37 (26 to 51)	3 (1 to 5)	6 (4 to 7)	235 (172 to 321)	100 (56 to 166)	111 (78 to 152)	8 (3 to 15)	17 (13 to 21)
Congo (Brazzaville)	2047	80 (60 to 107)	34 (19 to 57)	38 (26 to 51)	3 (1 to 5)	6 (5 to 8)	237 (177 to 317)	101 (56 to 168)	112 (78 to 153)	8 (4 to 15)	17 (13 to 22)
Congo (Brazzaville)	2048	81 (58 to 110)	34 (19 to 57)	38 (26 to 52)	3 (1 to 5)	6 (5 to 8)	239 (172 to 325)	102 (55 to 170)	112 (79 to 154)	8 (4 to 15)	17 (14 to 23)
Congo (Brazzaville)	2049	81 (61 to 109)	35 (19 to 58)	38 (27 to 52)	3 (1 to 5)	6 (5 to 8)	241 (180 to 325)	103 (56 to 173)	113 (79 to 155)	8 (4 to 15)	17 (14 to 23)
Congo (Brazzaville)	2050	82 (58 to 112)	35 (19 to 59)	38 (27 to 53)	3 (1 to 5)	6 (5 to 8)	243 (173 to 334)	104 (55 to 176)	114 (79 to 156)	8 (4 to 15)	18 (14 to 24)
Costa Rica	1995	413 (375 to 455)	254 (228 to 282)	145 (122 to 171)	11 (6 to 20)	2 (2 to 2)	615 (560 to 678)	378 (340 to 420)	217 (183 to 255)	17 (9 to 29)	4 (4 to 4)
Costa Rica	1996	421 (388 to 457)	261 (238 to 284)	147 (126 to 169)	11 (6 to 19)	3 (3 to 3)	628 (579 to 681)	389 (356 to 424)	219 (188 to 253)	17 (9 to 29)	4 (4 to 4)
Costa Rica	1997	428 (399 to 459)	267 (248 to 288)	146 (127 to 166)	11 (6 to 19)	3 (3 to 3)	638 (595 to 685)	399 (369 to 430)	218 (190 to 247)	16 (9 to 28)	5 (5 to 5)
Costa Rica	1998	458 (428 to 490)	292 (272 to 314)	150 (131 to 169)	11 (6 to 18)	4 (4 to 4)	683 (638 to 730)	436 (406 to 468)	224 (195 to 252)	17 (9 to 27)	6 (6 to 6)
Costa Rica	1999	477 (448 to 507)	308 (287 to 330)	153 (134 to 173)	11 (6 to 18)	5 (5 to 5)	711 (669 to 756)	459 (428 to 493)	228 (199 to 258)	17 (9 to 27)	8 (8 to 8)
Costa Rica	2000	496 (467 to 526)	321 (300 to 344)	157 (138 to 178)	11 (6 to 18)	7 (7 to 7)	740 (697 to 785)	479 (447 to 513)	234 (206 to 265)	17 (10 to 27)	11 (11 to 11)

Costa Rica	2001	509 (480 to 539)	335 (313 to 358)	161 (142 to 181)	11 (6 to 17)	3 (3 to 3)	760 (716 to 804)	499 (466 to 534)	240 (212 to 270)	16 (10 to 26)	4 (4 to 4)
Costa Rica	2002	547 (518 to 577)	362 (339 to 386)	170 (150 to 189)	12 (7 to 20)	3 (3 to 3)	816 (772 to 860)	540 (505 to 576)	253 (224 to 282)	18 (11 to 29)	5 (5 to 5)
Costa Rica	2003	557 (527 to 589)	366 (343 to 390)	174 (156 to 195)	13 (8 to 21)	3 (3 to 3)	830 (786 to 878)	546 (512 to 582)	260 (232 to 291)	20 (11 to 32)	5 (5 to 5)
Costa Rica	2004	570 (540 to 604)	374 (351 to 400)	181 (161 to 201)	13 (8 to 21)	2 (2 to 2)	850 (805 to 901)	558 (524 to 596)	269 (240 to 301)	20 (12 to 32)	3 (3 to 3)
Costa Rica	2005	595 (563 to 630)	390 (366 to 415)	188 (167 to 210)	16 (9 to 24)	1 (1 to 1)	888 (840 to 940)	582 (546 to 618)	281 (249 to 314)	23 (14 to 36)	1 (1 to 1)
Costa Rica	2006	629 (596 to 666)	414 (390 to 439)	196 (175 to 219)	17 (10 to 26)	2 (2 to 2)	938 (889 to 994)	618 (582 to 655)	293 (261 to 327)	25 (16 to 39)	3 (3 to 3)
Costa Rica	2007	675 (641 to 712)	450 (425 to 475)	205 (183 to 229)	18 (11 to 29)	2 (2 to 2)	1007 (956 to 1062)	671 (635 to 709)	306 (273 to 341)	27 (17 to 43)	2 (2 to 2)
Costa Rica	2008	741 (707 to 778)	504 (478 to 532)	214 (192 to 238)	20 (12 to 31)	2 (2 to 2)	1106 (1054 to 1160)	752 (713 to 793)	320 (286 to 355)	30 (18 to 46)	4 (4 to 4)
Costa Rica	2009	792 (757 to 829)	552 (525 to 582)	216 (193 to 240)	22 (14 to 33)	2 (2 to 2)	1181 (1129 to 1236)	824 (783 to 868)	322 (288 to 357)	33 (20 to 50)	3 (3 to 3)
Costa Rica	2010	852 (813 to 890)	605 (576 to 637)	220 (197 to 246)	26 (16 to 38)	1 (1 to 1)	1271 (1212 to 1327)	903 (859 to 949)	328 (294 to 366)	39 (25 to 57)	2 (2 to 2)
Costa Rica	2011	856 (817 to 896)	606 (576 to 637)	218 (195 to 243)	32 (21 to 46)	0 (0 to 0)	1276 (1218 to 1336)	904 (860 to 950)	325 (291 to 363)	47 (31 to 69)	1 (1 to 1)
Costa Rica	2012	870 (829 to 914)	618 (586 to 648)	214 (192 to 239)	38 (25 to 54)	0 (0 to 0)	1298 (1237 to 1363)	921 (874 to 966)	320 (286 to 356)	56 (37 to 81)	0 (0 to 0)

Costa Rica	2013	894 (851 to 937)	638 (606 to 671)	213 (190 to 236)	43 (29 to 61)	0 (0 to 0)	1334 (1269 to 1397)	952 (904 to 1000)	318 (283 to 353)	64 (43 to 91)	1 (1 to 1)
Costa Rica	2014	904 (860 to 948)	647 (615 to 680)	212 (189 to 236)	44 (29 to 64)	0 (0 to 0)	1348 (1283 to 1414)	965 (917 to 1015)	317 (282 to 352)	66 (44 to 95)	1 (1 to 1)
Costa Rica	2015	915 (873 to 960)	660 (629 to 692)	209 (185 to 236)	46 (30 to 68)	1 (1 to 1)	1365 (1302 to 1432)	984 (938 to 1032)	312 (276 to 351)	68 (44 to 101)	1 (1 to 1)
Costa Rica	2016	946 (900 to 993)	668 (633 to 703)	206 (179 to 236)	47 (30 to 70)	25 (25 to 25)	1410 (1342 to 1482)	996 (945 to 1048)	308 (267 to 352)	69 (44 to 104)	37 (37 to 37)
Costa Rica	2017	944 (869 to 1026)	688 (643 to 737)	205 (175 to 240)	50 (31 to 79)	0 (0 to 0)	1408 (1296 to 1530)	1026 (959 to 1099)	306 (261 to 359)	74 (46 to 117)	1 (1 to 1)
Costa Rica	2018	947 (876 to 1035)	689 (640 to 740)	208 (177 to 243)	50 (31 to 79)	0 (0 to 0)	1413 (1306 to 1544)	1028 (954 to 1104)	310 (264 to 362)	75 (46 to 118)	1 (1 to 1)
Costa Rica	2019	988 (906 to 1080)	725 (672 to 783)	210 (179 to 246)	52 (32 to 82)	0 (0 to 0)	1474 (1351 to 1611)	1082 (1002 to 1168)	313 (267 to 366)	78 (48 to 122)	1 (1 to 1)
Costa Rica	2020	1018 (938 to 1115)	750 (695 to 809)	213 (182 to 249)	54 (33 to 84)	0 (0 to 0)	1518 (1398 to 1663)	1119 (1037 to 1207)	318 (271 to 371)	80 (49 to 125)	1 (1 to 1)
Costa Rica	2021	1039 (945 to 1141)	768 (704 to 832)	216 (184 to 252)	55 (34 to 86)	0 (0 to 0)	1550 (1410 to 1703)	1146 (1051 to 1241)	322 (274 to 375)	82 (50 to 128)	1 (1 to 1)
Costa Rica	2022	1060 (966 to 1171)	786 (718 to 858)	218 (186 to 255)	56 (34 to 88)	0 (0 to 0)	1582 (1441 to 1746)	1172 (1070 to 1280)	325 (277 to 380)	83 (51 to 131)	1 (1 to 1)

Costa Rica	2023	1079 (977 to 1196)	802 (727 to 880)	220 (187 to 257)	57 (35 to 89)	0 (0 to 1)	1609 (1458 to 1784)	1196 (1084 to 1313)	328 (280 to 383)	85 (52 to 133)	1 (1 to 1)
Costa Rica	2024	1094 (989 to 1216)	814 (732 to 899)	222 (189 to 259)	58 (36 to 90)	0 (0 to 1)	1631 (1475 to 1813)	1214 (1092 to 1341)	331 (282 to 387)	86 (53 to 135)	1 (1 to 1)
Costa Rica	2025	1108 (994 to 1240)	826 (742 to 920)	224 (190 to 261)	58 (36 to 91)	0 (0 to 1)	1653 (1482 to 1849)	1232 (1107 to 1372)	333 (284 to 390)	87 (54 to 136)	1 (1 to 1)
Costa Rica	2026	1123 (1009 to 1261)	839 (748 to 937)	225 (192 to 263)	59 (37 to 92)	0 (0 to 1)	1676 (1505 to 1881)	1251 (1115 to 1397)	336 (286 to 393)	88 (55 to 138)	1 (1 to 1)
Costa Rica	2027	1139 (1009 to 1284)	852 (753 to 954)	227 (193 to 266)	60 (37 to 94)	0 (0 to 1)	1700 (1506 to 1915)	1271 (1124 to 1423)	339 (288 to 396)	89 (56 to 140)	1 (1 to 1)
Costa Rica	2028	1156 (1027 to 1308)	865 (760 to 979)	229 (195 to 268)	61 (38 to 95)	0 (0 to 1)	1724 (1531 to 1952)	1291 (1134 to 1460)	342 (291 to 400)	91 (56 to 141)	1 (1 to 1)
Costa Rica	2029	1172 (1032 to 1330)	879 (769 to 999)	231 (197 to 270)	62 (38 to 96)	0 (0 to 1)	1748 (1539 to 1983)	1311 (1147 to 1489)	345 (293 to 403)	92 (57 to 143)	1 (1 to 1)
Costa Rica	2030	1189 (1045 to 1352)	893 (778 to 1014)	233 (198 to 273)	63 (39 to 97)	1 (0 to 1)	1773 (1559 to 2017)	1331 (1160 to 1512)	348 (296 to 407)	93 (58 to 145)	1 (1 to 1)
Costa Rica	2031	1205 (1055 to 1376)	906 (785 to 1039)	235 (200 to 276)	64 (39 to 99)	1 (0 to 1)	1798 (1574 to 2052)	1352 (1170 to 1550)	351 (299 to 411)	95 (58 to 148)	1 (1 to 1)
Costa Rica	2032	1223 (1066 to 1395)	920 (792 to 1054)	237 (202 to 278)	64 (40 to 101)	1 (0 to 1)	1824 (1590 to 2080)	1373 (1181 to 1571)	354 (302 to 415)	96 (59 to 150)	1 (0 to 1)

Costa Rica	2033	1240 (1078 to 1420)	935 (804 to 1073)	239 (204 to 281)	65 (40 to 102)	1 (0 to 1)	1850 (1607 to 2118)	1395 (1199 to 1601)	357 (305 to 419)	98 (60 to 152)	1 (0 to 1)
Costa Rica	2034	1259 (1096 to 1446)	951 (811 to 1092)	242 (206 to 283)	66 (41 to 104)	0 (0 to 1)	1879 (1635 to 2156)	1418 (1210 to 1629)	360 (307 to 422)	99 (61 to 155)	1 (0 to 1)
Costa Rica	2035	1278 (1101 to 1475)	966 (819 to 1124)	244 (207 to 285)	68 (42 to 105)	0 (0 to 1)	1906 (1642 to 2200)	1441 (1222 to 1676)	364 (309 to 426)	101 (62 to 157)	1 (0 to 1)
Costa Rica	2036	1298 (1124 to 1501)	983 (836 to 1148)	246 (209 to 288)	69 (42 to 107)	0 (0 to 1)	1936 (1676 to 2239)	1466 (1247 to 1712)	367 (312 to 430)	102 (63 to 159)	1 (0 to 1)
Costa Rica	2037	1317 (1135 to 1530)	999 (849 to 1168)	249 (211 to 291)	70 (43 to 108)	0 (0 to 1)	1965 (1693 to 2281)	1489 (1267 to 1742)	371 (315 to 434)	104 (64 to 162)	1 (0 to 1)
Costa Rica	2038	1338 (1153 to 1548)	1016 (855 to 1188)	251 (213 to 294)	71 (44 to 110)	0 (0 to 1)	1996 (1719 to 2309)	1515 (1275 to 1772)	375 (318 to 439)	106 (65 to 165)	1 (0 to 1)
Costa Rica	2039	1359 (1163 to 1585)	1033 (872 to 1215)	254 (215 to 297)	72 (44 to 112)	0 (0 to 1)	2028 (1734 to 2364)	1541 (1301 to 1812)	378 (321 to 443)	107 (66 to 167)	1 (0 to 1)
Costa Rica	2040	1382 (1188 to 1611)	1052 (886 to 1243)	256 (217 to 300)	73 (45 to 114)	0 (0 to 1)	2061 (1772 to 2403)	1569 (1321 to 1855)	382 (323 to 447)	109 (67 to 170)	1 (0 to 1)
Costa Rica	2041	1403 (1187 to 1652)	1070 (893 to 1274)	259 (219 to 303)	74 (46 to 116)	0 (0 to 1)	2093 (1770 to 2465)	1596 (1332 to 1901)	386 (326 to 452)	111 (68 to 173)	0 (0 to 1)
Costa Rica	2042	1423 (1213 to 1677)	1086 (897 to 1296)	261 (220 to 306)	75 (46 to 117)	0 (0 to 1)	2123 (1809 to 2502)	1621 (1338 to 1933)	389 (328 to 456)	112 (69 to 175)	0 (0 to 1)

Costa Rica	2043	1445 (1216 to 1702)	1104 (915 to 1310)	263 (222 to 309)	77 (47 to 119)	0 (0 to 1)	2155 (1814 to 2539)	1647 (1365 to 1954)	393 (331 to 460)	114 (70 to 177)	0 (0 to 1)
Costa Rica	2044	1464 (1242 to 1723)	1121 (923 to 1337)	265 (223 to 311)	78 (47 to 120)	0 (0 to 1)	2183 (1852 to 2571)	1672 (1377 to 1994)	396 (333 to 464)	116 (71 to 179)	0 (0 to 1)
Costa Rica	2045	1483 (1234 to 1762)	1137 (926 to 1364)	267 (224 to 313)	79 (48 to 122)	0 (0 to 1)	2213 (1841 to 2629)	1696 (1382 to 2034)	399 (335 to 467)	117 (72 to 181)	0 (0 to 1)
Costa Rica	2046	1502 (1264 to 1779)	1153 (944 to 1392)	269 (226 to 315)	80 (49 to 123)	0 (0 to 1)	2240 (1886 to 2654)	1720 (1408 to 2076)	401 (337 to 470)	119 (72 to 184)	0 (0 to 1)
Costa Rica	2047	1521 (1266 to 1815)	1169 (956 to 1416)	271 (227 to 318)	81 (49 to 125)	0 (0 to 1)	2268 (1888 to 2707)	1744 (1425 to 2111)	404 (338 to 474)	120 (73 to 186)	0 (0 to 1)
Costa Rica	2048	1538 (1291 to 1837)	1184 (961 to 1442)	272 (228 to 319)	82 (50 to 126)	0 (0 to 1)	2294 (1926 to 2740)	1766 (1433 to 2150)	406 (339 to 476)	122 (74 to 188)	0 (0 to 1)
Costa Rica	2049	1556 (1283 to 1865)	1199 (970 to 1459)	274 (228 to 321)	83 (50 to 128)	0 (0 to 1)	2321 (1913 to 2782)	1789 (1447 to 2176)	408 (341 to 479)	123 (75 to 191)	0 (0 to 1)
Costa Rica	2050	1574 (1312 to 1886)	1215 (975 to 1470)	275 (229 to 323)	84 (51 to 130)	0 (0 to 1)	2347 (1956 to 2814)	1812 (1454 to 2193)	411 (342 to 482)	125 (76 to 194)	0 (0 to 1)
Croatia	1995	514 (467 to 563)	441 (399 to 489)	58 (46 to 73)	9 (5 to 17)	4 (4 to 4)	959 (873 to 1051)	824 (745 to 913)	109 (85 to 137)	18 (9 to 32)	8 (8 to 8)
Croatia	1996	542 (500 to 585)	465 (426 to 504)	61 (49 to 76)	10 (5 to 18)	5 (5 to 5)	1011 (934 to 1093)	868 (796 to 941)	115 (92 to 143)	19 (10 to 33)	10 (10 to 10)

Croatia	1997	555 (517 to 596)	474 (439 to 512)	66 (53 to 81)	11 (5 to 19)	4 (4 to 4)	1036 (965 to 1114)	886 (820 to 956)	123 (99 to 151)	20 (10 to 35)	7 (7 to 7)
Croatia	1998	604 (565 to 646)	519 (483 to 556)	71 (59 to 86)	12 (6 to 21)	2 (2 to 2)	1128 (1055 to 1207)	969 (902 to 1039)	133 (110 to 161)	22 (11 to 39)	4 (4 to 4)
Croatia	1999	634 (595 to 676)	543 (506 to 580)	79 (66 to 94)	12 (6 to 21)	0 (0 to 0)	1185 (1111 to 1263)	1014 (945 to 1084)	147 (122 to 176)	23 (12 to 40)	0 (0 to 0)
Croatia	2000	652 (611 to 693)	548 (512 to 584)	90 (75 to 107)	13 (7 to 21)	1 (1 to 1)	1217 (1141 to 1295)	1023 (955 to 1091)	169 (140 to 199)	23 (13 to 40)	2 (2 to 2)
Croatia	2001	661 (620 to 703)	545 (508 to 583)	99 (83 to 118)	16 (9 to 26)	1 (1 to 1)	1234 (1158 to 1314)	1017 (949 to 1088)	186 (155 to 220)	29 (16 to 49)	2 (2 to 2)
Croatia	2002	701 (657 to 744)	571 (535 to 609)	111 (93 to 130)	16 (9 to 27)	2 (2 to 2)	1308 (1228 to 1389)	1067 (998 to 1136)	208 (174 to 244)	30 (18 to 51)	3 (3 to 3)
Croatia	2003	732 (686 to 775)	595 (558 to 632)	118 (99 to 139)	16 (9 to 27)	2 (2 to 2)	1367 (1281 to 1448)	1112 (1042 to 1180)	221 (185 to 259)	30 (18 to 50)	5 (5 to 5)
Croatia	2004	769 (723 to 813)	627 (591 to 668)	124 (105 to 144)	16 (10 to 27)	2 (2 to 2)	1436 (1350 to 1519)	1172 (1103 to 1247)	231 (196 to 269)	31 (18 to 50)	3 (3 to 3)
Croatia	2005	821 (775 to 869)	677 (637 to 719)	123 (105 to 142)	17 (10 to 27)	4 (4 to 4)	1533 (1447 to 1624)	1265 (1190 to 1342)	230 (196 to 265)	31 (19 to 50)	7 (7 to 7)
Croatia	2006	901 (858 to 953)	750 (709 to 792)	128 (109 to 147)	18 (11 to 28)	6 (6 to 6)	1683 (1603 to 1780)	1400 (1325 to 1478)	239 (204 to 275)	34 (21 to 52)	11 (11 to 11)

Croatia	2007	980 (936 to 1032)	825 (785 to 873)	135 (116 to 154)	19 (13 to 29)	0 (0 to 0)	1830 (1748 to 1927)	1541 (1465 to 1630)	252 (217 to 288)	36 (24 to 54)	0 (0 to 0)
Croatia	2008	1033 (988 to 1080)	867 (826 to 913)	142 (124 to 163)	24 (16 to 35)	0 (0 to 0)	1928 (1845 to 2016)	1618 (1542 to 1704)	265 (232 to 305)	45 (30 to 65)	0 (0 to 0)
Croatia	2009	1034 (989 to 1082)	863 (823 to 905)	140 (123 to 159)	31 (22 to 43)	0 (0 to 0)	1931 (1847 to 2020)	1612 (1538 to 1691)	261 (229 to 297)	58 (40 to 81)	0 (0 to 0)
Croatia	2010	992 (950 to 1039)	825 (789 to 867)	131 (115 to 149)	36 (26 to 49)	0 (0 to 0)	1853 (1774 to 1940)	1541 (1472 to 1619)	245 (215 to 279)	67 (48 to 91)	0 (0 to 0)
Croatia	2011	966 (922 to 1009)	802 (764 to 841)	119 (104 to 136)	45 (33 to 60)	0 (0 to 0)	1805 (1722 to 1885)	1497 (1427 to 1571)	222 (195 to 254)	85 (62 to 112)	0 (0 to 0)
Croatia	2012	915 (873 to 957)	759 (722 to 797)	106 (92 to 122)	50 (37 to 65)	0 (0 to 0)	1708 (1631 to 1787)	1417 (1349 to 1488)	199 (172 to 229)	93 (69 to 122)	0 (0 to 0)
Croatia	2013	853 (812 to 895)	710 (675 to 749)	93 (80 to 108)	49 (37 to 65)	0 (0 to 0)	1592 (1517 to 1672)	1326 (1260 to 1399)	174 (150 to 201)	92 (68 to 122)	0 (0 to 0)
Croatia	2014	836 (793 to 882)	695 (661 to 735)	92 (79 to 108)	48 (36 to 64)	0 (0 to 0)	1561 (1481 to 1647)	1298 (1234 to 1372)	172 (147 to 201)	90 (67 to 120)	0 (0 to 0)
Croatia	2015	855 (811 to 902)	710 (671 to 752)	94 (78 to 111)	52 (38 to 70)	0 (0 to 0)	1597 (1514 to 1684)	1325 (1253 to 1404)	175 (146 to 207)	97 (71 to 131)	0 (0 to 0)
Croatia	2016	889 (840 to 941)	729 (687 to 773)	96 (79 to 116)	56 (38 to 77)	9 (9 to 9)	1661 (1569 to 1757)	1360 (1282 to 1443)	179 (147 to 216)	104 (72 to 144)	18 (18 to 18)

Croatia	2017	900 (824 to 980)	744 (689 to 801)	98 (79 to 121)	57 (38 to 84)	0 (0 to 0)	1680 (1539 to 1831)	1390 (1286 to 1495)	183 (147 to 227)	107 (71 to 157)	0 (0 to 0)
Croatia	2018	959 (871 to 1060)	797 (731 to 862)	102 (82 to 125)	61 (40 to 89)	0 (0 to 0)	1791 (1627 to 1980)	1488 (1365 to 1610)	190 (153 to 234)	114 (75 to 167)	0 (0 to 0)
Croatia	2019	1008 (911 to 1118)	839 (767 to 919)	105 (85 to 130)	64 (42 to 94)	0 (0 to 0)	1882 (1701 to 2089)	1566 (1432 to 1716)	197 (158 to 243)	119 (79 to 175)	0 (0 to 0)
Croatia	2020	1044 (937 to 1168)	869 (787 to 958)	109 (88 to 135)	66 (44 to 97)	0 (0 to 0)	1949 (1751 to 2180)	1622 (1470 to 1789)	204 (164 to 252)	123 (82 to 181)	0 (0 to 0)
Croatia	2021	1060 (948 to 1176)	882 (798 to 971)	111 (89 to 137)	67 (44 to 99)	0 (0 to 0)	1979 (1770 to 2196)	1647 (1490 to 1814)	206 (166 to 255)	125 (83 to 185)	0 (0 to 0)
Croatia	2022	1075 (963 to 1202)	895 (806 to 988)	112 (90 to 139)	68 (45 to 100)	0 (0 to 0)	2008 (1798 to 2245)	1671 (1504 to 1845)	209 (168 to 259)	127 (84 to 187)	0 (0 to 0)
Croatia	2023	1092 (974 to 1213)	909 (818 to 1003)	113 (91 to 140)	70 (46 to 102)	0 (0 to 0)	2039 (1818 to 2266)	1698 (1528 to 1872)	212 (170 to 262)	130 (86 to 191)	0 (0 to 0)
Croatia	2024	1105 (987 to 1241)	920 (824 to 1023)	114 (92 to 141)	70 (47 to 103)	0 (0 to 0)	2063 (1843 to 2317)	1718 (1539 to 1910)	213 (171 to 264)	132 (87 to 193)	0 (0 to 0)
Croatia	2025	1113 (985 to 1252)	928 (828 to 1035)	115 (92 to 142)	71 (47 to 105)	0 (0 to 0)	2079 (1839 to 2339)	1732 (1546 to 1932)	214 (172 to 265)	133 (88 to 195)	0 (0 to 0)
Croatia	2026	1123 (994 to 1266)	936 (832 to 1041)	115 (92 to 142)	72 (47 to 106)	0 (0 to 0)	2097 (1857 to 2363)	1748 (1553 to 1943)	215 (172 to 266)	134 (89 to 197)	0 (0 to 0)

Croatia	2027	1133 (1002 to 1277)	945 (840 to 1055)	115 (93 to 143)	73 (48 to 107)	0 (0 to 0)	2116 (1871 to 2385)	1764 (1569 to 1969)	216 (173 to 267)	136 (90 to 200)	0 (0 to 0)
Croatia	2028	1143 (1005 to 1296)	954 (844 to 1066)	116 (93 to 144)	74 (49 to 108)	0 (0 to 0)	2135 (1877 to 2420)	1781 (1576 to 1992)	217 (174 to 268)	137 (91 to 201)	0 (0 to 0)
Croatia	2029	1154 (1008 to 1308)	963 (844 to 1080)	116 (93 to 144)	74 (49 to 109)	0 (0 to 0)	2155 (1883 to 2442)	1799 (1577 to 2017)	218 (174 to 269)	139 (91 to 203)	0 (0 to 0)
Croatia	2030	1165 (1011 to 1330)	972 (845 to 1104)	117 (94 to 145)	75 (49 to 110)	0 (0 to 0)	2175 (1889 to 2484)	1816 (1578 to 2061)	219 (175 to 270)	140 (92 to 205)	0 (0 to 0)
Croatia	2031	1176 (1019 to 1354)	982 (855 to 1129)	118 (94 to 146)	76 (50 to 111)	0 (0 to 0)	2196 (1903 to 2529)	1834 (1596 to 2108)	220 (176 to 272)	142 (93 to 208)	0 (0 to 0)
Croatia	2032	1191 (1022 to 1376)	995 (850 to 1146)	119 (95 to 147)	77 (51 to 113)	0 (0 to 0)	2224 (1909 to 2570)	1858 (1588 to 2141)	221 (177 to 274)	144 (95 to 210)	0 (0 to 0)
Croatia	2033	1207 (1031 to 1393)	1009 (858 to 1161)	120 (96 to 148)	78 (51 to 115)	0 (0 to 0)	2255 (1926 to 2601)	1885 (1603 to 2168)	223 (179 to 277)	146 (96 to 214)	0 (0 to 0)
Croatia	2034	1225 (1044 to 1431)	1025 (869 to 1193)	121 (97 to 150)	80 (52 to 117)	0 (0 to 0)	2288 (1949 to 2672)	1913 (1624 to 2228)	226 (181 to 280)	149 (98 to 218)	0 (0 to 0)
Croatia	2035	1243 (1055 to 1449)	1040 (880 to 1213)	122 (98 to 151)	81 (53 to 119)	0 (0 to 0)	2321 (1970 to 2706)	1941 (1643 to 2265)	228 (183 to 283)	152 (99 to 222)	0 (0 to 0)
Croatia	2036	1262 (1067 to 1482)	1056 (894 to 1236)	123 (99 to 153)	83 (54 to 121)	0 (0 to 0)	2356 (1992 to 2768)	1971 (1670 to 2308)	230 (185 to 286)	154 (102 to 226)	0 (0 to 0)

Croatia	2037	1279 (1079 to 1509)	1070 (901 to 1264)	125 (100 to 155)	84 (55 to 123)	0 (0 to 0)	2389 (2015 to 2818)	1999 (1682 to 2360)	233 (187 to 289)	157 (104 to 230)	0 (0 to 0)
Croatia	2038	1297 (1091 to 1544)	1086 (913 to 1288)	126 (101 to 156)	85 (56 to 125)	0 (0 to 0)	2422 (2038 to 2884)	2028 (1705 to 2406)	235 (188 to 292)	159 (105 to 234)	0 (0 to 0)
Croatia	2039	1317 (1101 to 1561)	1103 (924 to 1304)	127 (102 to 158)	87 (57 to 127)	0 (0 to 0)	2459 (2056 to 2915)	2060 (1725 to 2435)	237 (190 to 295)	162 (107 to 238)	0 (0 to 0)
Croatia	2040	1337 (1113 to 1593)	1120 (930 to 1335)	128 (103 to 160)	88 (58 to 129)	0 (0 to 0)	2497 (2079 to 2974)	2092 (1737 to 2494)	240 (192 to 298)	165 (108 to 241)	0 (0 to 0)
Croatia	2041	1356 (1132 to 1622)	1137 (949 to 1364)	130 (104 to 161)	90 (59 to 131)	0 (0 to 0)	2533 (2114 to 3030)	2123 (1773 to 2546)	242 (194 to 301)	168 (110 to 244)	0 (0 to 0)
Croatia	2042	1376 (1141 to 1654)	1153 (957 to 1395)	131 (105 to 163)	91 (60 to 133)	0 (0 to 0)	2569 (2131 to 3088)	2154 (1786 to 2605)	245 (196 to 304)	171 (112 to 248)	0 (0 to 0)
Croatia	2043	1395 (1149 to 1669)	1170 (962 to 1406)	132 (106 to 164)	93 (61 to 135)	0 (0 to 0)	2605 (2146 to 3116)	2184 (1797 to 2625)	247 (198 to 307)	173 (114 to 251)	0 (0 to 0)
Croatia	2044	1413 (1162 to 1698)	1185 (971 to 1423)	133 (107 to 166)	94 (62 to 136)	0 (0 to 0)	2638 (2170 to 3170)	2213 (1814 to 2656)	249 (200 to 309)	176 (115 to 255)	0 (0 to 0)
Croatia	2045	1430 (1180 to 1715)	1200 (983 to 1445)	134 (108 to 167)	96 (63 to 138)	0 (0 to 0)	2671 (2203 to 3202)	2242 (1835 to 2698)	251 (202 to 312)	179 (117 to 258)	0 (0 to 0)
Croatia	2046	1449 (1188 to 1769)	1217 (988 to 1477)	135 (109 to 168)	97 (64 to 140)	0 (0 to 0)	2706 (2219 to 3303)	2272 (1845 to 2759)	253 (203 to 315)	181 (119 to 262)	0 (0 to 0)

Croatia	2047	1468 (1199 to 1789)	1233 (1002 to 1507)	136 (109 to 170)	98 (65 to 142)	0 (0 to 0)	2742 (2239 to 3341)	2303 (1872 to 2815)	255 (204 to 317)	184 (120 to 266)	0 (0 to 0)
Croatia	2048	1487 (1208 to 1825)	1250 (1006 to 1543)	137 (110 to 171)	100 (65 to 144)	0 (0 to 0)	2777 (2257 to 3409)	2334 (1878 to 2882)	257 (206 to 319)	186 (122 to 269)	0 (0 to 0)
Croatia	2049	1507 (1227 to 1855)	1267 (1019 to 1560)	139 (111 to 172)	101 (66 to 147)	0 (0 to 0)	2813 (2292 to 3463)	2366 (1902 to 2914)	259 (207 to 322)	189 (123 to 274)	0 (0 to 0)
Croatia	2050	1527 (1221 to 1887)	1284 (1015 to 1600)	140 (112 to 174)	103 (67 to 149)	0 (0 to 0)	2851 (2281 to 3524)	2398 (1895 to 2987)	261 (209 to 324)	192 (125 to 278)	0 (0 to 0)
Cuba	1995	236 (205 to 272)	191 (162 to 224)	38 (28 to 49)	7 (3 to 13)	0 (0 to 0)	637 (553 to 734)	516 (438 to 606)	103 (76 to 131)	18 (8 to 34)	0 (0 to 0)
Cuba	1996	258 (227 to 294)	211 (181 to 243)	41 (31 to 51)	7 (3 to 13)	0 (0 to 0)	698 (612 to 795)	569 (489 to 656)	109 (83 to 139)	19 (9 to 36)	0 (0 to 0)
Cuba	1997	271 (242 to 306)	221 (193 to 252)	43 (33 to 54)	7 (3 to 13)	0 (0 to 0)	733 (652 to 827)	598 (523 to 680)	115 (89 to 146)	19 (9 to 35)	1 (1 to 1)
Cuba	1998	279 (251 to 311)	227 (201 to 255)	45 (35 to 56)	7 (3 to 12)	0 (0 to 0)	753 (677 to 839)	613 (542 to 690)	122 (95 to 151)	18 (9 to 33)	0 (0 to 0)
Cuba	1999	301 (272 to 334)	244 (218 to 275)	49 (39 to 60)	7 (3 to 12)	1 (1 to 1)	813 (735 to 901)	660 (588 to 742)	132 (105 to 163)	18 (9 to 34)	1 (1 to 1)
Cuba	2000	332 (301 to 366)	269 (241 to 302)	56 (45 to 68)	7 (3 to 13)	0 (0 to 0)	898 (814 to 989)	727 (651 to 815)	151 (122 to 184)	19 (9 to 35)	1 (1 to 1)
Cuba	2001	352 (322 to 387)	282 (254 to 314)	63 (51 to 77)	7 (3 to 13)	0 (0 to 0)	952 (869 to 1046)	763 (686 to 850)	169 (138 to 208)	19 (9 to 34)	1 (1 to 1)
Cuba	2002	378 (344 to 415)	302 (270 to 335)	68 (56 to 83)	7 (3 to 13)	1 (1 to 1)	1021 (930 to 1121)	815 (730 to 905)	184 (151 to 224)	20 (9 to 36)	1 (1 to 1)

Cuba	2003	406 (371 to 443)	324 (292 to 357)	72 (59 to 87)	8 (4 to 14)	1 (1 to 1)	1096 (1003 to 1196)	876 (789 to 966)	195 (159 to 235)	21 (10 to 38)	4 (4 to 4)
Cuba	2004	439 (404 to 476)	356 (324 to 388)	74 (60 to 90)	8 (4 to 15)	1 (1 to 1)	1187 (1092 to 1286)	961 (877 to 1049)	200 (163 to 243)	22 (11 to 40)	4 (4 to 4)
Cuba	2005	550 (510 to 593)	460 (424 to 497)	80 (65 to 95)	10 (5 to 19)	1 (1 to 1)	1487 (1377 to 1601)	1242 (1144 to 1342)	215 (176 to 258)	28 (13 to 50)	2 (2 to 2)
Cuba	2006	653 (611 to 697)	558 (518 to 598)	82 (67 to 98)	12 (6 to 22)	1 (1 to 1)	1764 (1652 to 1882)	1507 (1401 to 1615)	222 (182 to 266)	33 (16 to 59)	3 (3 to 3)
Cuba	2007	783 (736 to 829)	683 (641 to 726)	84 (69 to 101)	14 (7 to 26)	2 (2 to 2)	2114 (1988 to 2241)	1845 (1732 to 1962)	227 (187 to 273)	38 (18 to 70)	4 (4 to 4)
Cuba	2008	929 (880 to 985)	824 (779 to 875)	88 (73 to 105)	17 (8 to 31)	1 (1 to 1)	2511 (2376 to 2662)	2225 (2104 to 2363)	237 (196 to 284)	46 (22 to 85)	3 (3 to 3)
Cuba	2009	961 (912 to 1013)	853 (811 to 900)	88 (73 to 105)	18 (9 to 33)	2 (2 to 2)	2597 (2465 to 2737)	2305 (2192 to 2431)	238 (198 to 285)	48 (23 to 90)	5 (5 to 5)
Cuba	2010	936 (886 to 985)	826 (783 to 869)	90 (76 to 107)	18 (9 to 35)	2 (2 to 2)	2528 (2394 to 2660)	2231 (2116 to 2347)	243 (204 to 289)	48 (23 to 93)	5 (5 to 5)
Cuba	2011	930 (884 to 980)	814 (772 to 856)	96 (81 to 114)	18 (9 to 35)	1 (1 to 1)	2512 (2387 to 2647)	2198 (2087 to 2313)	260 (219 to 308)	49 (24 to 94)	4 (4 to 4)
Cuba	2012	963 (911 to 1013)	839 (793 to 882)	103 (87 to 121)	20 (10 to 37)	1 (1 to 1)	2602 (2461 to 2736)	2266 (2144 to 2382)	279 (236 to 327)	54 (26 to 99)	3 (3 to 3)

Cuba	2013	993 (943 to 1046)	865 (819 to 908)	107 (90 to 126)	20 (10 to 38)	1 (1 to 1)	2683 (2548 to 2825)	2336 (2212 to 2452)	289 (242 to 341)	55 (27 to 104)	4 (4 to 4)
Cuba	2014	1062 (1011 to 1113)	931 (886 to 974)	110 (92 to 130)	21 (10 to 39)	1 (1 to 1)	2870 (2731 to 3008)	2515 (2393 to 2631)	296 (249 to 351)	57 (27 to 106)	2 (2 to 2)
Cuba	2015	1174 (1122 to 1232)	1033 (987 to 1082)	117 (96 to 138)	23 (11 to 43)	1 (1 to 1)	3172 (3031 to 3328)	2792 (2666 to 2924)	315 (261 to 374)	62 (29 to 116)	3 (3 to 3)
Cuba	2016	1181 (1125 to 1242)	1038 (991 to 1092)	119 (98 to 143)	23 (11 to 43)	1 (1 to 1)	3191 (3040 to 3355)	2805 (2676 to 2950)	321 (264 to 387)	63 (29 to 116)	3 (3 to 3)
Cuba	2017	1208 (1129 to 1304)	1060 (996 to 1129)	122 (98 to 149)	25 (11 to 46)	1 (1 to 1)	3262 (3050 to 3522)	2865 (2691 to 3051)	328 (266 to 403)	66 (30 to 125)	3 (3 to 3)
Cuba	2018	1213 (1130 to 1310)	1063 (995 to 1136)	124 (100 to 152)	25 (11 to 46)	1 (1 to 1)	3276 (3052 to 3538)	2873 (2688 to 3068)	334 (270 to 411)	67 (31 to 125)	3 (3 to 3)
Cuba	2019	1250 (1163 to 1351)	1098 (1027 to 1174)	125 (101 to 154)	25 (12 to 48)	1 (1 to 1)	3376 (3142 to 3651)	2967 (2774 to 3173)	338 (274 to 416)	69 (31 to 129)	3 (3 to 3)
Cuba	2020	1292 (1196 to 1397)	1137 (1056 to 1218)	128 (104 to 157)	26 (12 to 49)	1 (1 to 1)	3491 (3230 to 3775)	3071 (2853 to 3290)	346 (280 to 425)	71 (32 to 133)	3 (3 to 3)
Cuba	2021	1322 (1219 to 1438)	1165 (1075 to 1251)	130 (105 to 160)	27 (12 to 50)	1 (1 to 1)	3572 (3294 to 3886)	3146 (2904 to 3380)	351 (284 to 431)	72 (33 to 136)	3 (3 to 3)
Cuba	2022	1362 (1252 to 1479)	1202 (1112 to 1294)	132 (107 to 162)	27 (13 to 52)	1 (1 to 1)	3680 (3383 to 3997)	3248 (3004 to 3496)	355 (288 to 438)	74 (34 to 139)	3 (3 to 3)

Cuba	2023	1403 (1293 to 1524)	1240 (1145 to 1338)	133 (108 to 165)	28 (13 to 53)	1 (1 to 1)	3790 (3493 to 4117)	3351 (3094 to 3616)	360 (292 to 445)	76 (35 to 143)	3 (3 to 4)
Cuba	2024	1443 (1327 to 1572)	1279 (1180 to 1381)	135 (109 to 167)	29 (13 to 54)	1 (1 to 1)	3900 (3584 to 4247)	3455 (3187 to 3731)	364 (295 to 451)	78 (36 to 147)	3 (3 to 4)
Cuba	2025	1484 (1363 to 1623)	1317 (1215 to 1426)	136 (110 to 168)	30 (14 to 56)	1 (1 to 1)	4010 (3681 to 4386)	3560 (3283 to 3853)	368 (298 to 455)	80 (37 to 150)	3 (3 to 4)
Cuba	2026	1528 (1396 to 1671)	1359 (1250 to 1475)	137 (111 to 169)	30 (14 to 57)	1 (1 to 1)	4127 (3771 to 4515)	3671 (3377 to 3984)	371 (301 to 458)	82 (38 to 154)	3 (3 to 4)
Cuba	2027	1573 (1435 to 1722)	1402 (1281 to 1524)	139 (113 to 171)	31 (14 to 58)	1 (1 to 2)	4250 (3877 to 4652)	3788 (3462 to 4118)	375 (304 to 462)	84 (39 to 157)	4 (3 to 4)
Cuba	2028	1622 (1481 to 1774)	1449 (1321 to 1577)	140 (114 to 173)	32 (15 to 59)	1 (1 to 2)	4383 (4002 to 4794)	3915 (3570 to 4262)	378 (307 to 466)	86 (40 to 160)	4 (3 to 4)
Cuba	2029	1673 (1518 to 1836)	1498 (1364 to 1635)	141 (115 to 174)	33 (15 to 61)	1 (1 to 2)	4520 (4101 to 4960)	4048 (3684 to 4417)	381 (309 to 469)	88 (41 to 164)	4 (3 to 4)
Cuba	2030	1724 (1566 to 1899)	1548 (1407 to 1692)	142 (116 to 175)	33 (15 to 62)	1 (1 to 2)	4659 (4231 to 5131)	4181 (3801 to 4570)	384 (312 to 472)	90 (42 to 167)	4 (3 to 5)
Cuba	2031	1775 (1610 to 1958)	1597 (1451 to 1751)	143 (116 to 176)	34 (16 to 63)	1 (1 to 2)	4796 (4350 to 5291)	4314 (3920 to 4732)	386 (314 to 475)	92 (43 to 171)	4 (3 to 5)
Cuba	2032	1824 (1651 to 2023)	1644 (1490 to 1808)	144 (116 to 177)	35 (16 to 64)	1 (1 to 2)	4929 (4462 to 5464)	4442 (4024 to 4884)	389 (315 to 478)	94 (43 to 174)	4 (3 to 5)

Cuba	2033	1873 (1697 to 2079)	1691 (1533 to 1862)	145 (117 to 178)	36 (16 to 66)	2 (1 to 2)	5061 (4584 to 5618)	4568 (4143 to 5031)	392 (317 to 482)	96 (44 to 178)	4 (4 to 5)
Cuba	2034	1921 (1734 to 2128)	1737 (1570 to 1917)	146 (118 to 180)	36 (17 to 67)	2 (1 to 2)	5191 (4684 to 5750)	4694 (4242 to 5179)	395 (318 to 486)	98 (45 to 181)	4 (3 to 5)
Cuba	2035	1969 (1775 to 2191)	1783 (1609 to 1973)	147 (118 to 181)	37 (17 to 68)	2 (1 to 2)	5319 (4795 to 5919)	4817 (4346 to 5331)	398 (320 to 489)	100 (46 to 184)	4 (4 to 5)
Cuba	2036	2016 (1805 to 2252)	1828 (1644 to 2027)	148 (119 to 183)	38 (17 to 69)	2 (0 to 2)	5445 (4876 to 6083)	4938 (4442 to 5477)	401 (322 to 493)	102 (47 to 187)	5 (0 to 6)
Cuba	2037	2060 (1840 to 2302)	1870 (1677 to 2078)	150 (120 to 184)	38 (18 to 71)	2 (0 to 2)	5565 (4971 to 6221)	5053 (4531 to 5614)	404 (324 to 497)	104 (48 to 191)	5 (0 to 6)
Cuba	2038	2103 (1870 to 2356)	1912 (1711 to 2131)	151 (121 to 185)	39 (18 to 72)	2 (0 to 2)	5683 (5052 to 6365)	5165 (4623 to 5758)	407 (327 to 501)	106 (48 to 194)	5 (0 to 6)
Cuba	2039	2146 (1910 to 2406)	1953 (1744 to 2183)	152 (122 to 187)	40 (18 to 73)	2 (0 to 2)	5799 (5162 to 6499)	5276 (4712 to 5898)	411 (329 to 505)	108 (49 to 198)	5 (0 to 6)
Cuba	2040	2189 (1950 to 2466)	1993 (1780 to 2231)	153 (123 to 189)	41 (18 to 75)	2 (0 to 2)	5914 (5269 to 6662)	5386 (4808 to 6027)	414 (331 to 511)	110 (50 to 201)	5 (0 to 7)
Cuba	2041	2232 (1982 to 2511)	2034 (1810 to 2282)	155 (124 to 191)	41 (19 to 76)	2 (0 to 3)	6031 (5356 to 6783)	5496 (4889 to 6166)	419 (334 to 517)	112 (50 to 205)	5 (0 to 7)
Cuba	2042	2277 (2018 to 2567)	2076 (1843 to 2332)	157 (125 to 194)	42 (19 to 77)	2 (0 to 3)	6152 (5451 to 6936)	5609 (4980 to 6301)	424 (338 to 523)	114 (51 to 209)	5 (0 to 7)

Cuba	2043	2320 (2051 to 2612)	2117 (1877 to 2381)	159 (127 to 196)	43 (19 to 79)	2 (0 to 3)	6269 (5540 to 7056)	5719 (5071 to 6433)	429 (343 to 530)	116 (52 to 212)	5 (0 to 7)
Cuba	2044	2363 (2092 to 2672)	2156 (1908 to 2429)	161 (129 to 199)	44 (20 to 80)	2 (0 to 3)	6384 (5651 to 7219)	5826 (5154 to 6562)	435 (348 to 538)	118 (53 to 217)	5 (0 to 8)
Cuba	2045	2406 (2131 to 2728)	2196 (1939 to 2476)	163 (131 to 202)	45 (20 to 82)	2 (0 to 3)	6501 (5758 to 7371)	5934 (5240 to 6689)	442 (354 to 547)	120 (54 to 221)	5 (0 to 8)
Cuba	2046	2447 (2163 to 2776)	2234 (1973 to 2523)	166 (133 to 206)	45 (20 to 83)	2 (0 to 3)	6611 (5845 to 7500)	6036 (5329 to 6815)	448 (359 to 555)	122 (55 to 225)	5 (0 to 8)
Cuba	2047	2488 (2192 to 2827)	2272 (2005 to 2569)	168 (134 to 208)	46 (21 to 85)	2 (0 to 3)	6721 (5922 to 7638)	6139 (5418 to 6942)	453 (363 to 561)	125 (56 to 230)	5 (0 to 8)
Cuba	2048	2527 (2222 to 2879)	2309 (2041 to 2611)	170 (136 to 210)	47 (21 to 86)	2 (0 to 3)	6828 (6004 to 7778)	6239 (5514 to 7055)	458 (367 to 567)	127 (57 to 234)	5 (0 to 8)
Cuba	2049	2566 (2263 to 2924)	2346 (2068 to 2661)	171 (137 to 212)	48 (22 to 88)	2 (0 to 3)	6933 (6114 to 7899)	6337 (5587 to 7190)	463 (371 to 573)	129 (58 to 237)	5 (0 to 9)
Cuba	2050	2606 (2301 to 2964)	2383 (2100 to 2696)	173 (139 to 214)	48 (22 to 89)	2 (0 to 3)	7042 (6217 to 8009)	6439 (5674 to 7285)	468 (374 to 579)	131 (59 to 241)	5 (0 to 9)
Cyprus	1995	718 (666 to 775)	252 (224 to 281)	452 (407 to 499)	15 (9 to 23)	0 (0 to 0)	1080 (1002 to 1165)	379 (337 to 422)	680 (612 to 751)	22 (13 to 35)	0 (0 to 0)
Cyprus	1996	753 (708 to 799)	268 (244 to 294)	469 (435 to 505)	16 (10 to 25)	0 (0 to 0)	1133 (1065 to 1201)	403 (367 to 443)	705 (654 to 759)	25 (15 to 38)	0 (0 to 0)

Cyprus	1997	796 (755 to 838)	295 (272 to 319)	482 (450 to 517)	19 (12 to 29)	0 (0 to 0)	1198 (1135 to 1261)	444 (410 to 480)	726 (676 to 778)	29 (18 to 43)	0 (0 to 0)
Cyprus	1998	837 (798 to 876)	328 (305 to 351)	487 (455 to 522)	22 (14 to 32)	0 (0 to 0)	1259 (1200 to 1318)	493 (459 to 529)	733 (684 to 785)	33 (22 to 47)	0 (0 to 0)
Cyprus	1999	878 (840 to 918)	356 (332 to 379)	495 (463 to 528)	28 (19 to 38)	0 (0 to 0)	1321 (1264 to 1381)	535 (500 to 570)	744 (697 to 794)	41 (29 to 57)	0 (0 to 0)
Cyprus	2000	934 (894 to 975)	388 (364 to 412)	516 (484 to 546)	30 (22 to 41)	0 (0 to 0)	1405 (1345 to 1467)	584 (547 to 620)	776 (728 to 821)	46 (33 to 61)	0 (0 to 0)
Cyprus	2001	974 (934 to 1014)	413 (390 to 438)	526 (496 to 555)	35 (26 to 45)	0 (0 to 0)	1464 (1405 to 1525)	621 (586 to 658)	791 (745 to 835)	52 (39 to 68)	0 (0 to 0)
Cyprus	2002	1008 (970 to 1049)	437 (413 to 460)	528 (501 to 559)	43 (34 to 55)	0 (0 to 0)	1517 (1459 to 1578)	657 (621 to 691)	795 (753 to 841)	65 (51 to 83)	0 (0 to 0)
Cyprus	2003	1038 (997 to 1079)	451 (428 to 475)	522 (492 to 552)	64 (52 to 80)	0 (0 to 0)	1561 (1499 to 1623)	679 (644 to 715)	785 (740 to 830)	97 (78 to 120)	0 (0 to 0)
Cyprus	2004	1030 (989 to 1071)	438 (414 to 460)	515 (487 to 545)	77 (63 to 95)	0 (0 to 0)	1549 (1487 to 1611)	659 (623 to 692)	775 (733 to 820)	115 (94 to 142)	0 (0 to 0)
Cyprus	2005	1072 (1032 to 1113)	454 (430 to 479)	531 (501 to 560)	87 (72 to 106)	0 (0 to 0)	1612 (1552 to 1674)	683 (647 to 720)	798 (753 to 843)	130 (108 to 159)	0 (0 to 0)
Cyprus	2006	1105 (1066 to 1146)	469 (445 to 493)	547 (516 to 576)	90 (75 to 107)	0 (0 to 0)	1663 (1604 to 1724)	705 (669 to 742)	822 (776 to 867)	135 (112 to 161)	0 (0 to 0)

Cyprus	2007	1141 (1099 to 1186)	484 (460 to 507)	568 (537 to 601)	89 (75 to 107)	0 (0 to 0)	1716 (1653 to 1784)	728 (692 to 763)	854 (808 to 904)	134 (112 to 160)	0 (0 to 0)
Cyprus	2008	1221 (1176 to 1265)	523 (498 to 547)	605 (573 to 640)	94 (79 to 111)	0 (0 to 0)	1837 (1769 to 1903)	786 (749 to 823)	910 (862 to 963)	141 (119 to 167)	0 (0 to 0)
Cyprus	2009	1283 (1238 to 1330)	568 (541 to 592)	612 (578 to 646)	104 (89 to 123)	0 (0 to 0)	1930 (1862 to 2000)	854 (814 to 891)	920 (869 to 971)	156 (133 to 185)	0 (0 to 0)
Cyprus	2010	1281 (1235 to 1325)	588 (563 to 613)	577 (546 to 609)	116 (100 to 135)	0 (0 to 0)	1927 (1858 to 1994)	884 (847 to 923)	868 (821 to 916)	175 (150 to 203)	0 (0 to 0)
Cyprus	2011	1271 (1226 to 1312)	588 (564 to 614)	559 (529 to 589)	123 (106 to 143)	0 (0 to 0)	1912 (1844 to 1974)	885 (848 to 924)	841 (795 to 887)	185 (160 to 215)	0 (0 to 0)
Cyprus	2012	1231 (1188 to 1272)	564 (540 to 589)	540 (511 to 570)	126 (109 to 148)	0 (0 to 0)	1851 (1786 to 1913)	848 (813 to 886)	813 (768 to 858)	190 (164 to 222)	0 (0 to 0)
Cyprus	2013	1168 (1127 to 1210)	526 (501 to 551)	517 (489 to 546)	126 (110 to 145)	0 (0 to 0)	1757 (1695 to 1820)	791 (754 to 829)	777 (735 to 821)	189 (165 to 219)	0 (0 to 0)
Cyprus	2014	1169 (1130 to 1211)	518 (493 to 543)	516 (488 to 548)	134 (117 to 154)	0 (0 to 0)	1758 (1699 to 1821)	779 (742 to 817)	777 (734 to 824)	202 (176 to 232)	0 (0 to 0)
Cyprus	2015	1135 (1095 to 1179)	488 (464 to 514)	509 (481 to 540)	138 (120 to 158)	0 (0 to 0)	1707 (1647 to 1773)	734 (698 to 773)	766 (723 to 812)	207 (180 to 237)	0 (0 to 0)
Cyprus	2016	1151 (1107 to 1202)	487 (462 to 514)	520 (488 to 554)	144 (123 to 166)	0 (0 to 0)	1731 (1665 to 1809)	732 (696 to 774)	782 (734 to 834)	216 (186 to 250)	0 (0 to 0)

Cyprus	2017	1184 (1111 to 1261)	500 (468 to 535)	533 (492 to 576)	151 (125 to 181)	0 (0 to 0)	1780 (1671 to 1897)	752 (704 to 804)	802 (739 to 867)	227 (188 to 272)	0 (0 to 0)
Cyprus	2018	1314 (1232 to 1407)	593 (555 to 639)	551 (508 to 596)	169 (140 to 204)	0 (0 to 0)	1976 (1852 to 2116)	892 (834 to 961)	829 (764 to 897)	255 (211 to 307)	0 (0 to 0)
Cyprus	2019	1242 (1164 to 1327)	528 (493 to 569)	556 (512 to 601)	158 (131 to 190)	0 (0 to 0)	1868 (1750 to 1996)	793 (742 to 856)	837 (771 to 904)	238 (197 to 285)	0 (0 to 0)
Cyprus	2020	1277 (1194 to 1366)	547 (509 to 590)	566 (522 to 611)	164 (135 to 197)	0 (0 to 0)	1920 (1796 to 2054)	823 (766 to 888)	851 (784 to 920)	246 (203 to 296)	0 (0 to 0)
Cyprus	2021	1298 (1218 to 1387)	561 (522 to 604)	571 (527 to 618)	167 (138 to 201)	0 (0 to 0)	1953 (1831 to 2086)	843 (785 to 908)	859 (792 to 930)	251 (207 to 302)	0 (0 to 0)
Cyprus	2022	1319 (1232 to 1413)	574 (533 to 618)	576 (531 to 622)	170 (140 to 204)	0 (0 to 0)	1984 (1853 to 2126)	863 (802 to 930)	866 (799 to 935)	256 (211 to 307)	0 (0 to 0)
Cyprus	2023	1337 (1251 to 1433)	586 (542 to 633)	579 (533 to 626)	173 (143 to 207)	0 (0 to 0)	2011 (1882 to 2155)	881 (816 to 951)	871 (801 to 941)	260 (215 to 311)	0 (0 to 0)
Cyprus	2024	1352 (1261 to 1453)	596 (549 to 646)	582 (535 to 630)	175 (144 to 209)	0 (0 to 0)	2034 (1896 to 2185)	896 (826 to 972)	875 (805 to 947)	263 (217 to 315)	0 (0 to 0)
Cyprus	2025	1365 (1276 to 1466)	604 (557 to 655)	584 (537 to 632)	177 (146 to 212)	0 (0 to 0)	2054 (1919 to 2205)	909 (837 to 985)	879 (808 to 951)	266 (220 to 319)	0 (0 to 0)
Cyprus	2026	1379 (1287 to 1482)	614 (565 to 665)	586 (539 to 634)	179 (148 to 215)	0 (0 to 0)	2074 (1936 to 2230)	923 (850 to 1000)	882 (810 to 954)	269 (222 to 323)	0 (0 to 0)

Cyprus	2027	1396 (1299 to 1495)	625 (576 to 677)	589 (541 to 637)	182 (150 to 218)	0 (0 to 0)	2099 (1953 to 2248)	940 (866 to 1018)	886 (814 to 958)	273 (225 to 329)	0 (0 to 0)
Cyprus	2028	1411 (1314 to 1517)	635 (583 to 690)	592 (543 to 641)	184 (151 to 221)	0 (0 to 0)	2122 (1976 to 2282)	955 (876 to 1038)	890 (817 to 964)	277 (227 to 333)	0 (0 to 0)
Cyprus	2029	1431 (1328 to 1539)	649 (595 to 707)	595 (547 to 646)	187 (154 to 225)	0 (0 to 0)	2153 (1997 to 2314)	976 (895 to 1063)	896 (823 to 971)	282 (231 to 339)	0 (0 to 0)
Cyprus	2030	1452 (1350 to 1566)	662 (606 to 722)	599 (550 to 651)	191 (156 to 229)	0 (0 to 0)	2184 (2031 to 2355)	996 (911 to 1086)	901 (827 to 979)	287 (234 to 344)	0 (0 to 0)
Cyprus	2031	1474 (1364 to 1587)	676 (618 to 740)	603 (553 to 656)	194 (158 to 233)	0 (0 to 0)	2216 (2051 to 2388)	1017 (930 to 1112)	907 (832 to 987)	292 (238 to 350)	0 (0 to 0)
Cyprus	2032	1495 (1387 to 1618)	691 (629 to 757)	607 (556 to 662)	197 (161 to 237)	0 (0 to 0)	2249 (2087 to 2434)	1039 (947 to 1139)	913 (837 to 995)	297 (242 to 356)	0 (0 to 0)
Cyprus	2033	1518 (1400 to 1637)	706 (641 to 778)	612 (561 to 667)	201 (164 to 242)	0 (0 to 0)	2284 (2106 to 2462)	1062 (965 to 1170)	920 (843 to 1003)	302 (246 to 364)	0 (0 to 0)
Cyprus	2034	1542 (1424 to 1676)	722 (653 to 797)	616 (564 to 672)	204 (166 to 246)	0 (0 to 0)	2319 (2142 to 2521)	1085 (982 to 1199)	926 (848 to 1010)	307 (250 to 370)	0 (0 to 0)
Cyprus	2035	1564 (1436 to 1694)	737 (665 to 816)	620 (567 to 676)	208 (169 to 250)	0 (0 to 0)	2353 (2161 to 2547)	1108 (1000 to 1228)	932 (853 to 1017)	313 (254 to 376)	0 (0 to 0)
Cyprus	2036	1586 (1461 to 1731)	752 (676 to 835)	623 (570 to 680)	211 (172 to 254)	0 (0 to 0)	2385 (2197 to 2604)	1130 (1017 to 1257)	937 (857 to 1023)	318 (258 to 382)	0 (0 to 0)

Cyprus	2037	1604 (1473 to 1743)	765 (686 to 850)	625 (570 to 683)	214 (174 to 258)	0 (0 to 0)	2413 (2215 to 2621)	1151 (1032 to 1279)	940 (858 to 1027)	322 (262 to 388)	0 (0 to 0)
Cyprus	2038	1623 (1489 to 1780)	779 (698 to 868)	627 (571 to 686)	217 (176 to 262)	0 (0 to 0)	2441 (2240 to 2678)	1171 (1050 to 1305)	944 (859 to 1032)	327 (265 to 394)	0 (0 to 0)
Cyprus	2039	1643 (1501 to 1787)	793 (708 to 887)	630 (573 to 690)	220 (178 to 267)	0 (0 to 0)	2471 (2258 to 2687)	1192 (1065 to 1334)	947 (861 to 1038)	331 (268 to 401)	0 (0 to 0)
Cyprus	2040	1663 (1523 to 1831)	807 (719 to 906)	632 (574 to 694)	223 (181 to 271)	0 (0 to 0)	2501 (2291 to 2753)	1214 (1081 to 1363)	951 (864 to 1043)	336 (272 to 407)	0 (0 to 0)
Cyprus	2041	1682 (1536 to 1837)	821 (731 to 918)	634 (576 to 696)	226 (183 to 274)	0 (0 to 0)	2530 (2310 to 2762)	1235 (1100 to 1381)	954 (866 to 1047)	341 (275 to 413)	0 (0 to 0)
Cyprus	2042	1701 (1551 to 1869)	836 (744 to 937)	636 (577 to 698)	230 (185 to 278)	0 (0 to 0)	2559 (2332 to 2811)	1257 (1119 to 1409)	957 (869 to 1050)	345 (278 to 418)	0 (0 to 0)
Cyprus	2043	1720 (1568 to 1879)	850 (757 to 956)	637 (577 to 700)	232 (187 to 282)	0 (0 to 0)	2586 (2359 to 2826)	1279 (1138 to 1438)	958 (867 to 1052)	350 (281 to 424)	0 (0 to 0)
Cyprus	2044	1738 (1579 to 1915)	865 (767 to 974)	637 (576 to 702)	235 (189 to 286)	0 (0 to 0)	2613 (2375 to 2880)	1302 (1153 to 1464)	958 (866 to 1055)	354 (284 to 431)	0 (0 to 0)
Cyprus	2045	1753 (1596 to 1923)	879 (781 to 992)	636 (574 to 701)	238 (191 to 290)	0 (0 to 0)	2637 (2401 to 2892)	1323 (1175 to 1492)	956 (863 to 1055)	358 (287 to 436)	0 (0 to 0)
Cyprus	2046	1769 (1606 to 1947)	894 (791 to 1009)	635 (572 to 701)	240 (193 to 293)	0 (0 to 0)	2660 (2415 to 2928)	1344 (1190 to 1517)	955 (861 to 1054)	361 (290 to 441)	0 (0 to 0)

Cyprus	2047	1783 (1614 to 1959)	907 (804 to 1025)	633 (570 to 701)	243 (194 to 297)	0 (0 to 0)	2682 (2428 to 2947)	1364 (1210 to 1542)	952 (857 to 1054)	365 (292 to 446)	0 (0 to 0)
Cyprus	2048	1799 (1631 to 1989)	922 (815 to 1041)	632 (568 to 700)	245 (196 to 300)	0 (0 to 0)	2706 (2454 to 2992)	1386 (1226 to 1566)	951 (854 to 1053)	369 (294 to 451)	0 (0 to 0)
Cyprus	2049	1812 (1636 to 1997)	934 (827 to 1056)	630 (565 to 698)	247 (197 to 303)	0 (0 to 0)	2725 (2461 to 3003)	1405 (1243 to 1589)	948 (850 to 1050)	372 (296 to 455)	0 (0 to 0)
Cyprus	2050	1824 (1654 to 2021)	947 (838 to 1071)	628 (562 to 696)	249 (198 to 306)	0 (0 to 0)	2743 (2488 to 3039)	1424 (1261 to 1611)	944 (846 to 1046)	375 (298 to 460)	0 (0 to 0)
Czech Republic	1995	793 (744 to 841)	701 (656 to 746)	78 (63 to 95)	15 (8 to 26)	0 (0 to 0)	1347 (1265 to 1430)	1191 (1115 to 1268)	132 (106 to 162)	25 (13 to 44)	0 (0 to 0)
Czech Republic	1996	759 (721 to 798)	668 (635 to 705)	77 (63 to 92)	14 (8 to 24)	0 (0 to 0)	1289 (1225 to 1356)	1135 (1079 to 1198)	130 (107 to 156)	23 (13 to 41)	0 (0 to 0)
Czech Republic	1997	766 (734 to 802)	674 (643 to 707)	78 (66 to 92)	14 (8 to 24)	0 (0 to 0)	1302 (1247 to 1363)	1146 (1092 to 1201)	133 (111 to 156)	24 (13 to 40)	0 (0 to 0)
Czech Republic	1998	770 (737 to 807)	678 (645 to 710)	79 (66 to 92)	14 (8 to 23)	0 (0 to 0)	1309 (1253 to 1371)	1152 (1096 to 1207)	134 (113 to 156)	23 (14 to 39)	0 (0 to 0)
Czech Republic	1999	781 (745 to 818)	687 (655 to 719)	80 (68 to 93)	14 (8 to 22)	0 (0 to 0)	1327 (1267 to 1389)	1168 (1112 to 1221)	136 (116 to 159)	23 (14 to 37)	0 (0 to 0)
Czech Republic	2000	814 (780 to 849)	716 (685 to 747)	84 (72 to 98)	14 (8 to 21)	0 (0 to 0)	1383 (1325 to 1442)	1217 (1164 to 1269)	142 (122 to 166)	24 (14 to 36)	0 (0 to 0)

Czech Republic	2001	883 (846 to 917)	776 (744 to 809)	90 (78 to 105)	17 (10 to 26)	0 (0 to 0)	1500 (1438 to 1558)	1318 (1265 to 1374)	154 (132 to 178)	28 (17 to 44)	0 (0 to 0)
Czech Republic	2002	936 (900 to 972)	820 (788 to 852)	96 (83 to 111)	19 (12 to 30)	0 (0 to 0)	1590 (1529 to 1653)	1394 (1339 to 1448)	163 (141 to 188)	33 (20 to 51)	0 (0 to 0)
Czech Republic	2003	1047 (1008 to 1089)	917 (881 to 952)	108 (94 to 124)	23 (15 to 35)	0 (0 to 0)	1780 (1713 to 1850)	1557 (1497 to 1617)	183 (159 to 210)	39 (25 to 59)	0 (0 to 0)
Czech Republic	2004	1027 (991 to 1068)	890 (854 to 926)	113 (99 to 129)	24 (16 to 36)	0 (0 to 0)	1745 (1684 to 1815)	1512 (1452 to 1574)	193 (168 to 219)	41 (27 to 61)	0 (0 to 0)
Czech Republic	2005	1092 (1056 to 1130)	936 (903 to 969)	127 (111 to 142)	30 (20 to 43)	0 (0 to 0)	1856 (1795 to 1921)	1590 (1535 to 1647)	215 (189 to 242)	50 (34 to 74)	0 (0 to 0)
Czech Republic	2006	1143 (1105 to 1179)	969 (935 to 1001)	143 (128 to 159)	31 (21 to 44)	0 (0 to 0)	1942 (1877 to 2004)	1646 (1589 to 1701)	243 (217 to 269)	53 (36 to 76)	0 (0 to 0)
Czech Republic	2007	1201 (1161 to 1241)	1004 (970 to 1040)	164 (149 to 181)	32 (22 to 45)	0 (0 to 0)	2040 (1973 to 2109)	1707 (1648 to 1766)	279 (252 to 308)	54 (37 to 77)	0 (0 to 0)
Czech Republic	2008	1260 (1224 to 1298)	1041 (1007 to 1075)	187 (170 to 204)	32 (22 to 44)	0 (0 to 0)	2141 (2079 to 2206)	1770 (1711 to 1827)	317 (289 to 346)	54 (37 to 75)	0 (0 to 0)
Czech Republic	2009	1339 (1301 to 1378)	1108 (1075 to 1141)	202 (184 to 219)	30 (21 to 42)	0 (0 to 0)	2276 (2211 to 2341)	1883 (1827 to 1939)	342 (313 to 373)	51 (35 to 71)	0 (0 to 0)
Czech Republic	2010	1345 (1309 to 1384)	1114 (1081 to 1144)	205 (187 to 223)	27 (19 to 37)	0 (0 to 0)	2286 (2224 to 2352)	1892 (1838 to 1945)	348 (318 to 378)	45 (32 to 63)	0 (0 to 0)

Czech Republic	2011	1375 (1337 to 1415)	1141 (1110 to 1176)	208 (190 to 226)	26 (18 to 35)	0 (0 to 0)	2336 (2272 to 2404)	1939 (1886 to 1998)	353 (322 to 383)	44 (31 to 60)	0 (0 to 0)
Czech Republic	2012	1425 (1389 to 1467)	1186 (1154 to 1221)	210 (193 to 229)	28 (20 to 38)	0 (0 to 0)	2421 (2360 to 2492)	2015 (1961 to 2074)	358 (327 to 388)	48 (34 to 65)	0 (0 to 0)
Czech Republic	2013	1446 (1408 to 1489)	1201 (1168 to 1234)	209 (192 to 227)	36 (26 to 49)	0 (0 to 0)	2458 (2392 to 2530)	2041 (1984 to 2097)	356 (326 to 386)	61 (43 to 84)	0 (0 to 0)
Czech Republic	2014	1490 (1450 to 1534)	1233 (1199 to 1268)	214 (197 to 232)	43 (31 to 59)	0 (0 to 0)	2533 (2464 to 2607)	2096 (2038 to 2155)	364 (335 to 394)	73 (53 to 101)	0 (0 to 0)
Czech Republic	2015	1524 (1482 to 1568)	1256 (1222 to 1293)	221 (203 to 239)	47 (33 to 64)	0 (0 to 0)	2590 (2518 to 2665)	2134 (2077 to 2196)	376 (345 to 407)	80 (57 to 109)	0 (0 to 0)
Czech Republic	2016	1520 (1478 to 1566)	1245 (1211 to 1281)	225 (205 to 247)	49 (34 to 67)	0 (0 to 0)	2583 (2511 to 2661)	2116 (2057 to 2177)	383 (349 to 420)	84 (58 to 114)	0 (0 to 0)
Czech Republic	2017	1585 (1515 to 1665)	1297 (1246 to 1348)	235 (210 to 262)	54 (36 to 76)	0 (0 to 0)	2694 (2575 to 2829)	2203 (2118 to 2291)	399 (357 to 445)	92 (61 to 129)	0 (0 to 0)
Czech Republic	2018	1694 (1616 to 1781)	1397 (1336 to 1455)	241 (216 to 270)	56 (37 to 78)	0 (0 to 0)	2879 (2746 to 3027)	2374 (2271 to 2472)	410 (367 to 458)	94 (63 to 132)	0 (0 to 0)
Czech Republic	2019	1775 (1688 to 1868)	1470 (1399 to 1536)	247 (221 to 276)	57 (38 to 80)	0 (0 to 0)	3016 (2869 to 3174)	2499 (2378 to 2610)	420 (375 to 469)	97 (65 to 136)	0 (0 to 0)
Czech Republic	2020	1839 (1746 to 1942)	1526 (1453 to 1601)	254 (227 to 284)	59 (39 to 83)	0 (0 to 0)	3124 (2968 to 3300)	2593 (2470 to 2721)	431 (385 to 482)	100 (67 to 140)	0 (0 to 0)

Czech Republic	2021	1878 (1777 to 1990)	1561 (1483 to 1642)	257 (229 to 288)	60 (40 to 84)	0 (0 to 0)	3191 (3020 to 3382)	2652 (2519 to 2789)	437 (390 to 489)	102 (68 to 143)	0 (0 to 0)
Czech Republic	2022	1916 (1808 to 2038)	1595 (1509 to 1691)	260 (232 to 291)	61 (41 to 86)	0 (0 to 0)	3256 (3072 to 3463)	2710 (2565 to 2873)	442 (395 to 495)	104 (69 to 147)	0 (0 to 0)
Czech Republic	2023	1959 (1841 to 2094)	1632 (1536 to 1735)	264 (236 to 296)	62 (42 to 88)	0 (0 to 0)	3328 (3129 to 3558)	2773 (2610 to 2948)	449 (401 to 502)	106 (71 to 149)	0 (0 to 0)
Czech Republic	2024	2002 (1879 to 2151)	1670 (1569 to 1787)	269 (240 to 301)	64 (43 to 90)	0 (0 to 0)	3402 (3193 to 3655)	2838 (2666 to 3037)	456 (407 to 511)	108 (72 to 152)	0 (0 to 0)
Czech Republic	2025	2048 (1916 to 2203)	1710 (1600 to 1837)	273 (243 to 306)	65 (44 to 92)	0 (0 to 0)	3481 (3256 to 3743)	2906 (2719 to 3121)	464 (414 to 520)	111 (74 to 156)	0 (0 to 0)
Czech Republic	2026	2095 (1952 to 2270)	1751 (1630 to 1883)	277 (247 to 311)	67 (45 to 94)	0 (0 to 0)	3560 (3316 to 3858)	2975 (2770 to 3199)	471 (420 to 528)	113 (76 to 159)	0 (0 to 0)
Czech Republic	2027	2142 (1991 to 2325)	1792 (1663 to 1939)	282 (251 to 316)	68 (46 to 96)	0 (0 to 0)	3640 (3383 to 3951)	3045 (2826 to 3295)	479 (427 to 536)	116 (78 to 162)	0 (0 to 0)
Czech Republic	2028	2192 (2035 to 2382)	1836 (1700 to 1987)	286 (255 to 320)	70 (47 to 98)	0 (0 to 0)	3724 (3459 to 4048)	3120 (2888 to 3376)	486 (433 to 545)	118 (79 to 166)	0 (0 to 0)
Czech Republic	2029	2248 (2080 to 2452)	1885 (1737 to 2050)	291 (259 to 326)	71 (47 to 100)	0 (0 to 0)	3820 (3534 to 4166)	3204 (2952 to 3483)	495 (440 to 554)	121 (81 to 171)	0 (0 to 0)
Czech Republic	2030	2308 (2120 to 2529)	1938 (1782 to 2119)	297 (264 to 332)	73 (48 to 103)	0 (0 to 0)	3922 (3602 to 4298)	3294 (3028 to 3600)	504 (448 to 564)	124 (82 to 175)	0 (0 to 0)

Czech Republic	2031	2367 (2165 to 2595)	1990 (1818 to 2181)	302 (268 to 338)	75 (49 to 105)	0 (0 to 0)	4022 (3678 to 4410)	3381 (3089 to 3706)	513 (456 to 575)	127 (84 to 179)	0 (0 to 0)
Czech Republic	2032	2425 (2209 to 2677)	2041 (1856 to 2251)	307 (273 to 345)	77 (50 to 108)	0 (0 to 0)	4121 (3753 to 4549)	3468 (3153 to 3825)	522 (464 to 585)	130 (86 to 183)	0 (0 to 0)
Czech Republic	2033	2484 (2254 to 2735)	2093 (1901 to 2305)	313 (277 to 350)	79 (51 to 110)	0 (0 to 0)	4221 (3831 to 4648)	3556 (3230 to 3917)	531 (471 to 595)	134 (87 to 187)	0 (0 to 0)
Czech Republic	2034	2543 (2303 to 2824)	2144 (1940 to 2379)	318 (282 to 356)	80 (53 to 113)	0 (0 to 0)	4321 (3914 to 4799)	3644 (3297 to 4043)	541 (480 to 604)	137 (90 to 191)	0 (0 to 0)
Czech Republic	2035	2601 (2351 to 2885)	2196 (1975 to 2438)	323 (287 to 362)	82 (54 to 115)	0 (0 to 0)	4421 (3995 to 4902)	3731 (3355 to 4142)	549 (487 to 616)	140 (92 to 196)	0 (0 to 0)
Czech Republic	2036	2660 (2396 to 2960)	2248 (2010 to 2508)	328 (291 to 368)	84 (55 to 118)	0 (0 to 0)	4520 (4072 to 5030)	3819 (3416 to 4261)	558 (494 to 626)	143 (94 to 200)	0 (0 to 0)
Czech Republic	2037	2721 (2442 to 3043)	2301 (2058 to 2572)	333 (295 to 375)	86 (56 to 121)	0 (0 to 0)	4623 (4150 to 5171)	3911 (3497 to 4370)	566 (502 to 637)	146 (95 to 205)	0 (0 to 0)
Czech Republic	2038	2779 (2492 to 3108)	2354 (2105 to 2642)	338 (299 to 380)	88 (57 to 123)	0 (0 to 0)	4723 (4234 to 5281)	4001 (3577 to 4489)	574 (509 to 646)	149 (97 to 209)	0 (0 to 0)
Czech Republic	2039	2838 (2544 to 3172)	2407 (2150 to 2711)	341 (303 to 384)	89 (58 to 125)	0 (0 to 0)	4822 (4324 to 5390)	4091 (3653 to 4607)	580 (515 to 653)	151 (99 to 212)	0 (0 to 0)
Czech Republic	2040	2894 (2585 to 3258)	2459 (2189 to 2768)	344 (305 to 388)	91 (60 to 127)	0 (0 to 0)	4918 (4393 to 5536)	4179 (3719 to 4704)	585 (518 to 660)	154 (101 to 216)	0 (0 to 0)

Czech Republic	2041	2951 (2626 to 3314)	2511 (2230 to 2835)	348 (308 to 392)	92 (61 to 129)	0 (0 to 0)	5014 (4462 to 5632)	4267 (3790 to 4818)	591 (523 to 667)	157 (103 to 220)	0 (0 to 0)
Czech Republic	2042	3005 (2681 to 3367)	2560 (2272 to 2900)	351 (310 to 396)	94 (62 to 132)	0 (0 to 0)	5106 (4556 to 5721)	4350 (3861 to 4927)	596 (526 to 673)	159 (105 to 224)	0 (0 to 0)
Czech Republic	2043	3058 (2725 to 3436)	2608 (2312 to 2951)	355 (313 to 401)	95 (63 to 134)	0 (0 to 0)	5196 (4631 to 5838)	4431 (3929 to 5015)	603 (531 to 681)	162 (107 to 227)	0 (0 to 0)
Czech Republic	2044	3110 (2765 to 3500)	2655 (2347 to 3011)	358 (315 to 406)	97 (64 to 136)	0 (0 to 0)	5285 (4698 to 5947)	4511 (3989 to 5116)	609 (535 to 691)	165 (109 to 231)	0 (0 to 0)
Czech Republic	2045	3161 (2801 to 3577)	2700 (2380 to 3065)	362 (316 to 412)	98 (65 to 138)	0 (0 to 0)	5371 (4760 to 6078)	4588 (4044 to 5208)	615 (538 to 700)	167 (111 to 234)	0 (0 to 0)
Czech Republic	2046	3212 (2851 to 3622)	2746 (2423 to 3127)	366 (319 to 417)	100 (66 to 140)	0 (0 to 0)	5458 (4845 to 6155)	4667 (4117 to 5314)	622 (543 to 708)	170 (112 to 239)	0 (0 to 0)
Czech Republic	2047	3261 (2891 to 3682)	2790 (2456 to 3172)	370 (322 to 421)	101 (67 to 143)	0 (0 to 0)	5541 (4912 to 6256)	4740 (4174 to 5389)	628 (547 to 716)	172 (113 to 243)	0 (0 to 0)
Czech Republic	2048	3310 (2935 to 3734)	2833 (2493 to 3221)	374 (325 to 426)	103 (68 to 146)	0 (0 to 0)	5624 (4987 to 6346)	4814 (4236 to 5473)	635 (553 to 724)	175 (115 to 248)	0 (0 to 0)
Czech Republic	2049	3360 (2978 to 3804)	2878 (2536 to 3274)	378 (329 to 432)	105 (69 to 149)	0 (0 to 0)	5710 (5060 to 6464)	4890 (4309 to 5564)	642 (558 to 733)	178 (118 to 253)	0 (0 to 0)
Czech Republic	2050	3411 (3018 to 3866)	2922 (2567 to 3330)	382 (332 to 437)	106 (70 to 151)	0 (0 to 0)	5796 (5128 to 6570)	4965 (4361 to 5658)	650 (565 to 742)	180 (120 to 256)	0 (0 to 0)

Côte d'Ivoire	1995	76 (61 to 92)	10 (8 to 14)	48 (35 to 63)	10 (5 to 19)	7 (7 to 7)	201 (162 to 243)	27 (21 to 37)	127 (93 to 166)	27 (13 to 49)	19 (19 to 19)
Côte d'Ivoire	1996	77 (63 to 93)	12 (9 to 15)	51 (37 to 65)	11 (5 to 19)	4 (4 to 4)	203 (165 to 245)	31 (23 to 41)	133 (98 to 172)	28 (14 to 50)	11 (11 to 11)
Côte d'Ivoire	1997	79 (64 to 95)	13 (10 to 17)	53 (39 to 68)	11 (6 to 20)	2 (2 to 2)	208 (168 to 251)	34 (25 to 45)	139 (103 to 179)	29 (15 to 52)	6 (6 to 6)
Côte d'Ivoire	1998	78 (63 to 94)	13 (9 to 17)	52 (39 to 67)	11 (6 to 20)	3 (3 to 3)	206 (167 to 248)	33 (25 to 45)	137 (102 to 176)	29 (15 to 52)	7 (7 to 7)
Côte d'Ivoire	1999	73 (59 to 88)	12 (9 to 16)	48 (36 to 61)	11 (6 to 19)	2 (2 to 2)	192 (156 to 231)	31 (24 to 42)	125 (95 to 160)	29 (15 to 51)	6 (6 to 6)
Côte d'Ivoire	2000	64 (51 to 78)	11 (8 to 14)	42 (31 to 54)	11 (5 to 19)	1 (1 to 1)	168 (135 to 205)	28 (21 to 37)	109 (82 to 141)	28 (14 to 50)	2 (2 to 2)
Côte d'Ivoire	2001	56 (45 to 69)	9 (7 to 12)	36 (27 to 46)	10 (5 to 18)	1 (1 to 1)	147 (118 to 183)	24 (19 to 32)	94 (71 to 121)	27 (13 to 48)	3 (3 to 3)
Côte d'Ivoire	2002	56 (45 to 69)	9 (7 to 12)	33 (25 to 43)	11 (5 to 19)	3 (3 to 3)	147 (120 to 181)	25 (19 to 33)	88 (66 to 114)	28 (14 to 50)	7 (7 to 7)
Côte d'Ivoire	2003	54 (44 to 66)	9 (7 to 12)	33 (25 to 42)	10 (5 to 18)	2 (2 to 2)	141 (115 to 174)	23 (18 to 30)	86 (65 to 111)	27 (13 to 48)	6 (6 to 6)
Côte d'Ivoire	2004	56 (46 to 69)	9 (7 to 12)	34 (26 to 45)	10 (5 to 18)	3 (3 to 3)	148 (120 to 181)	23 (18 to 30)	90 (68 to 119)	27 (13 to 48)	8 (8 to 8)
Côte d'Ivoire	2005	57 (47 to 70)	9 (7 to 11)	36 (27 to 47)	10 (5 to 18)	3 (3 to 3)	151 (123 to 184)	23 (17 to 29)	95 (72 to 124)	26 (13 to 47)	7 (7 to 7)
Côte d'Ivoire	2006	62 (51 to 76)	9 (7 to 12)	39 (29 to 50)	10 (5 to 18)	4 (4 to 4)	163 (133 to 199)	24 (18 to 31)	102 (77 to 131)	27 (14 to 48)	11 (11 to 11)
Côte d'Ivoire	2007	64 (52 to 78)	9 (7 to 12)	40 (31 to 52)	10 (5 to 19)	4 (4 to 4)	168 (136 to 206)	24 (18 to 31)	106 (81 to 137)	27 (14 to 49)	10 (10 to 10)
Côte d'Ivoire	2008	67 (55 to 81)	9 (7 to 12)	40 (31 to 52)	10 (5 to 18)	7 (7 to 7)	177 (145 to 214)	25 (19 to 32)	106 (81 to 138)	27 (14 to 48)	19 (19 to 19)
Côte d'Ivoire	2009	65 (54 to 80)	10 (7 to 13)	40 (30 to 52)	10 (5 to 18)	6 (6 to 6)	172 (142 to 211)	25 (19 to 33)	105 (79 to 137)	27 (14 to 48)	15 (15 to 15)

Côte d'Ivoire	2010	68 (57 to 83)	10 (7 to 13)	39 (29 to 51)	10 (5 to 19)	9 (9 to 9)	179 (149 to 218)	26 (19 to 34)	103 (77 to 134)	27 (14 to 49)	24 (24 to 24)
Côte d'Ivoire	2011	59 (49 to 72)	9 (7 to 12)	35 (27 to 46)	9 (5 to 17)	5 (5 to 5)	155 (128 to 189)	24 (18 to 31)	93 (71 to 121)	25 (13 to 44)	14 (14 to 14)
Côte d'Ivoire	2012	65 (53 to 78)	11 (9 to 15)	37 (28 to 47)	11 (6 to 19)	6 (6 to 6)	171 (141 to 206)	30 (22 to 39)	96 (73 to 124)	29 (15 to 50)	16 (16 to 16)
Côte d'Ivoire	2013	65 (54 to 78)	12 (9 to 16)	35 (26 to 45)	11 (6 to 19)	7 (7 to 7)	172 (143 to 206)	33 (24 to 42)	92 (69 to 120)	29 (15 to 50)	19 (19 to 19)
Côte d'Ivoire	2014	67 (56 to 80)	13 (10 to 17)	33 (25 to 43)	11 (6 to 20)	10 (10 to 10)	176 (147 to 210)	35 (26 to 46)	86 (65 to 114)	29 (15 to 52)	25 (25 to 25)
Côte d'Ivoire	2015	65 (54 to 79)	15 (11 to 20)	31 (23 to 41)	13 (6 to 22)	6 (6 to 6)	172 (141 to 209)	40 (30 to 52)	82 (61 to 108)	33 (17 to 57)	17 (17 to 17)
Côte d'Ivoire	2016	71 (59 to 86)	17 (13 to 22)	30 (22 to 40)	14 (7 to 24)	10 (10 to 10)	187 (155 to 226)	45 (33 to 58)	79 (59 to 106)	36 (17 to 62)	27 (27 to 27)
Côte d'Ivoire	2017	74 (58 to 92)	19 (14 to 24)	30 (22 to 41)	15 (7 to 25)	11 (11 to 11)	195 (153 to 241)	49 (36 to 64)	79 (58 to 107)	38 (18 to 66)	28 (28 to 28)
Côte d'Ivoire	2018	75 (59 to 95)	19 (14 to 25)	31 (23 to 42)	15 (7 to 26)	10 (10 to 10)	198 (155 to 251)	50 (37 to 65)	82 (60 to 111)	40 (19 to 69)	25 (25 to 25)
Côte d'Ivoire	2019	78 (61 to 97)	19 (14 to 25)	33 (24 to 44)	16 (8 to 28)	9 (9 to 10)	204 (161 to 254)	51 (37 to 67)	86 (63 to 115)	43 (20 to 73)	25 (25 to 25)
Côte d'Ivoire	2020	81 (63 to 103)	20 (15 to 27)	34 (25 to 46)	17 (8 to 29)	9 (9 to 10)	212 (165 to 271)	54 (40 to 70)	89 (66 to 120)	45 (21 to 77)	25 (23 to 26)
Côte d'Ivoire	2021	82 (64 to 103)	21 (15 to 28)	34 (25 to 46)	17 (8 to 30)	9 (9 to 10)	217 (169 to 272)	55 (41 to 73)	91 (67 to 122)	46 (22 to 79)	25 (23 to 26)
Côte d'Ivoire	2022	84 (65 to 108)	22 (16 to 28)	35 (26 to 47)	18 (9 to 31)	9 (9 to 10)	221 (172 to 284)	57 (42 to 75)	92 (68 to 125)	47 (22 to 81)	24 (22 to 27)
Côte d'Ivoire	2023	86 (66 to 108)	22 (16 to 29)	36 (26 to 48)	18 (9 to 32)	9 (8 to 10)	225 (174 to 284)	58 (43 to 77)	94 (69 to 127)	49 (23 to 83)	24 (22 to 27)
Côte d'Ivoire	2024	87 (68 to 112)	23 (17 to 30)	36 (27 to 49)	19 (9 to 33)	9 (8 to 10)	230 (179 to 294)	60 (44 to 80)	96 (71 to 129)	50 (24 to 86)	24 (22 to 27)

Côte d'Ivoire	2025	89 (69 to 113)	24 (17 to 31)	37 (27 to 50)	20 (9 to 34)	9 (8 to 10)	235 (182 to 297)	62 (45 to 82)	98 (72 to 132)	51 (24 to 89)	24 (21 to 27)
Côte d'Ivoire	2026	91 (70 to 117)	24 (18 to 32)	38 (28 to 51)	20 (10 to 35)	9 (8 to 10)	241 (186 to 308)	64 (46 to 85)	100 (73 to 135)	53 (25 to 91)	24 (21 to 27)
Côte d'Ivoire	2027	93 (72 to 118)	25 (18 to 33)	39 (28 to 52)	21 (10 to 36)	9 (8 to 10)	246 (190 to 312)	66 (47 to 88)	102 (75 to 138)	55 (26 to 94)	24 (21 to 27)
Côte d'Ivoire	2028	95 (73 to 124)	26 (18 to 34)	39 (29 to 53)	21 (10 to 37)	9 (8 to 11)	250 (192 to 326)	67 (48 to 90)	103 (76 to 140)	56 (26 to 96)	24 (21 to 28)
Côte d'Ivoire	2029	97 (74 to 123)	26 (19 to 35)	40 (29 to 54)	22 (10 to 37)	9 (8 to 11)	254 (196 to 323)	69 (49 to 93)	105 (76 to 142)	57 (27 to 98)	24 (21 to 28)
Côte d'Ivoire	2030	98 (76 to 128)	27 (19 to 36)	40 (29 to 55)	22 (10 to 38)	9 (8 to 11)	259 (200 to 336)	70 (51 to 96)	106 (77 to 144)	58 (27 to 100)	24 (21 to 29)
Côte d'Ivoire	2031	100 (77 to 129)	27 (20 to 37)	41 (30 to 55)	23 (11 to 39)	9 (8 to 11)	263 (202 to 339)	72 (52 to 97)	107 (78 to 146)	60 (28 to 102)	24 (21 to 29)
Côte d'Ivoire	2032	102 (78 to 132)	28 (20 to 38)	41 (30 to 56)	23 (11 to 40)	9 (8 to 11)	268 (205 to 347)	74 (53 to 99)	109 (79 to 148)	61 (29 to 104)	24 (21 to 29)
Côte d'Ivoire	2033	104 (79 to 132)	29 (20 to 39)	42 (30 to 57)	24 (11 to 40)	9 (8 to 11)	273 (208 to 348)	75 (54 to 103)	110 (80 to 150)	62 (29 to 107)	25 (21 to 30)
Côte d'Ivoire	2034	105 (80 to 138)	29 (21 to 40)	42 (31 to 58)	24 (11 to 41)	9 (8 to 11)	278 (211 to 364)	77 (55 to 105)	112 (81 to 152)	64 (30 to 109)	25 (21 to 30)
Côte d'Ivoire	2035	107 (82 to 138)	30 (21 to 41)	43 (31 to 58)	25 (12 to 42)	9 (8 to 12)	283 (215 to 362)	79 (56 to 107)	113 (82 to 154)	65 (30 to 111)	25 (21 to 31)
Côte d'Ivoire	2036	109 (82 to 144)	31 (22 to 42)	44 (31 to 59)	25 (12 to 43)	10 (8 to 12)	288 (217 to 379)	81 (58 to 111)	115 (83 to 156)	67 (31 to 113)	25 (21 to 31)
Côte d'Ivoire	2037	111 (84 to 144)	31 (22 to 43)	44 (32 to 60)	26 (12 to 44)	10 (8 to 12)	292 (221 to 379)	83 (59 to 114)	116 (84 to 158)	68 (32 to 116)	25 (21 to 31)
Côte d'Ivoire	2038	113 (86 to 148)	32 (23 to 44)	45 (32 to 61)	26 (12 to 45)	10 (8 to 12)	298 (226 to 390)	85 (61 to 117)	118 (85 to 160)	69 (32 to 119)	26 (21 to 33)
Côte d'Ivoire	2039	115 (88 to 150)	33 (24 to 45)	45 (33 to 61)	27 (13 to 46)	10 (8 to 12)	303 (231 to 394)	87 (62 to 118)	119 (86 to 162)	71 (33 to 122)	26 (21 to 33)

Côte d'Ivoire	2040	117 (88 to 155)	34 (24 to 46)	46 (33 to 62)	27 (13 to 47)	10 (8 to 13)	308 (231 to 408)	89 (63 to 121)	121 (87 to 163)	72 (34 to 125)	26 (21 to 33)
Côte d'Ivoire	2041	119 (91 to 155)	35 (25 to 47)	46 (33 to 63)	28 (13 to 49)	10 (8 to 13)	313 (240 to 408)	91 (65 to 123)	122 (88 to 166)	74 (34 to 128)	26 (21 to 33)
Côte d'Ivoire	2042	121 (90 to 159)	35 (25 to 48)	47 (34 to 64)	29 (13 to 50)	10 (8 to 13)	318 (237 to 419)	93 (66 to 127)	123 (89 to 168)	75 (35 to 131)	27 (22 to 34)
Côte d'Ivoire	2043	123 (94 to 160)	36 (26 to 50)	47 (34 to 65)	29 (14 to 51)	10 (8 to 13)	324 (247 to 420)	95 (68 to 131)	125 (90 to 170)	77 (36 to 134)	27 (22 to 35)
Côte d'Ivoire	2044	125 (93 to 167)	37 (26 to 51)	48 (34 to 65)	30 (14 to 52)	10 (8 to 14)	329 (245 to 440)	97 (69 to 135)	126 (91 to 172)	78 (37 to 136)	27 (22 to 36)
Côte d'Ivoire	2045	127 (97 to 167)	38 (27 to 53)	48 (35 to 66)	30 (14 to 53)	10 (8 to 14)	334 (255 to 439)	99 (70 to 138)	127 (91 to 174)	80 (37 to 139)	28 (22 to 36)
Côte d'Ivoire	2046	129 (96 to 174)	39 (27 to 54)	49 (35 to 67)	31 (14 to 54)	11 (8 to 14)	340 (253 to 459)	102 (72 to 143)	129 (92 to 176)	81 (38 to 142)	28 (22 to 36)
Côte d'Ivoire	2047	131 (99 to 172)	39 (28 to 55)	49 (35 to 68)	31 (15 to 55)	11 (9 to 14)	345 (262 to 453)	104 (73 to 146)	130 (93 to 178)	83 (39 to 145)	28 (23 to 38)
Côte d'Ivoire	2048	133 (99 to 182)	40 (28 to 57)	50 (36 to 68)	32 (15 to 56)	11 (9 to 15)	351 (260 to 479)	106 (74 to 151)	131 (94 to 180)	84 (39 to 148)	29 (23 to 38)
Côte d'Ivoire	2049	135 (102 to 179)	41 (29 to 59)	50 (36 to 69)	33 (15 to 57)	11 (9 to 15)	356 (268 to 473)	108 (75 to 155)	133 (95 to 181)	86 (40 to 150)	29 (23 to 38)
Côte d'Ivoire	2050	137 (102 to 188)	42 (29 to 60)	51 (36 to 70)	33 (16 to 58)	11 (9 to 15)	362 (268 to 495)	111 (76 to 159)	134 (96 to 183)	88 (41 to 153)	30 (23 to 40)
DR Congo	1995	19 (14 to 26)	1 (1 to 1)	17 (12 to 23)	1 (1 to 2)	0 (0 to 0)	31 (23 to 41)	1 (1 to 2)	27 (19 to 37)	2 (1 to 4)	1 (1 to 1)
DR Congo	1996	18 (14 to 24)	1 (1 to 1)	16 (11 to 22)	1 (1 to 2)	1 (1 to 1)	30 (22 to 40)	1 (1 to 1)	25 (18 to 35)	2 (1 to 4)	1 (1 to 1)
DR Congo	1997	18 (13 to 24)	1 (1 to 1)	15 (11 to 22)	1 (1 to 2)	0 (0 to 0)	29 (21 to 39)	1 (1 to 1)	25 (17 to 35)	2 (1 to 4)	1 (1 to 1)
DR Congo	1998	16 (12 to 22)	1 (0 to 1)	14 (10 to 19)	1 (1 to 2)	0 (0 to 0)	26 (19 to 35)	1 (1 to 1)	22 (16 to 32)	2 (1 to 4)	1 (1 to 1)

DR Congo	1999	12 (9 to 16)	1 (0 to 1)	10 (7 to 14)	1 (0 to 2)	0 (0 to 0)	20 (15 to 27)	1 (1 to 1)	17 (12 to 23)	2 (1 to 3)	1 (1 to 1)
DR Congo	2000	8 (6 to 11)	0 (0 to 0)	7 (5 to 9)	1 (0 to 2)	1 (1 to 1)	14 (10 to 18)	1 (0 to 1)	11 (7 to 15)	1 (1 to 3)	1 (1 to 1)
DR Congo	2001	10 (7 to 13)	0 (0 to 1)	7 (5 to 10)	1 (0 to 2)	1 (1 to 1)	15 (12 to 20)	1 (1 to 1)	12 (8 to 16)	2 (1 to 3)	1 (1 to 1)
DR Congo	2002	9 (7 to 12)	0 (0 to 1)	7 (5 to 10)	1 (0 to 2)	1 (1 to 1)	15 (11 to 20)	1 (1 to 1)	11 (8 to 16)	2 (1 to 3)	1 (1 to 1)
DR Congo	2003	13 (10 to 17)	1 (1 to 1)	10 (7 to 13)	1 (1 to 3)	1 (1 to 1)	21 (16 to 28)	1 (1 to 2)	15 (11 to 22)	2 (1 to 4)	2 (2 to 2)
DR Congo	2004	13 (10 to 17)	1 (1 to 1)	10 (7 to 14)	1 (1 to 2)	1 (1 to 1)	21 (16 to 28)	1 (1 to 2)	16 (11 to 22)	2 (1 to 4)	2 (2 to 2)
DR Congo	2005	15 (12 to 19)	1 (1 to 1)	10 (7 to 14)	1 (1 to 2)	3 (3 to 3)	24 (19 to 31)	1 (1 to 2)	16 (11 to 23)	2 (1 to 3)	4 (4 to 4)
DR Congo	2006	15 (11 to 18)	1 (1 to 1)	10 (7 to 14)	1 (1 to 2)	2 (2 to 2)	23 (19 to 30)	1 (1 to 2)	16 (11 to 23)	2 (1 to 4)	4 (4 to 4)
DR Congo	2007	14 (11 to 18)	1 (1 to 1)	10 (7 to 13)	1 (1 to 2)	2 (2 to 2)	23 (18 to 29)	2 (1 to 2)	16 (11 to 22)	2 (1 to 4)	4 (4 to 4)
DR Congo	2008	17 (15 to 22)	1 (1 to 2)	9 (6 to 13)	1 (1 to 2)	6 (6 to 6)	28 (23 to 35)	2 (1 to 3)	15 (10 to 20)	2 (1 to 4)	9 (9 to 9)
DR Congo	2009	16 (13 to 19)	1 (1 to 2)	8 (6 to 11)	1 (1 to 2)	5 (5 to 5)	25 (21 to 31)	2 (2 to 3)	13 (9 to 18)	2 (1 to 4)	8 (8 to 8)
DR Congo	2010	17 (14 to 20)	2 (1 to 2)	8 (5 to 10)	1 (1 to 2)	6 (6 to 6)	27 (23 to 32)	3 (2 to 4)	12 (8 to 17)	2 (1 to 4)	10 (10 to 10)
DR Congo	2011	16 (13 to 18)	2 (1 to 2)	7 (5 to 9)	1 (1 to 2)	6 (6 to 6)	25 (22 to 30)	3 (2 to 4)	11 (7 to 15)	2 (1 to 3)	10 (10 to 10)
DR Congo	2012	17 (14 to 20)	2 (2 to 3)	7 (5 to 10)	1 (1 to 2)	7 (7 to 7)	27 (23 to 32)	3 (3 to 4)	11 (8 to 15)	2 (1 to 3)	11 (11 to 11)
DR Congo	2013	17 (14 to 20)	2 (2 to 3)	7 (5 to 10)	1 (1 to 2)	7 (7 to 7)	27 (23 to 32)	4 (3 to 5)	11 (8 to 16)	2 (1 to 3)	11 (11 to 11)

DR Congo	2014	19 (16 to 23)	3 (2 to 4)	8 (5 to 11)	2 (1 to 3)	6 (6 to 6)	31 (26 to 37)	5 (4 to 6)	13 (9 to 18)	3 (1 to 5)	10 (10 to 10)
DR Congo	2015	20 (17 to 23)	3 (2 to 4)	8 (6 to 12)	2 (1 to 3)	6 (6 to 6)	32 (27 to 38)	5 (4 to 7)	14 (9 to 19)	3 (1 to 5)	10 (10 to 10)
DR Congo	2016	19 (16 to 22)	3 (2 to 4)	8 (6 to 11)	2 (1 to 3)	6 (6 to 6)	30 (25 to 36)	4 (3 to 6)	13 (9 to 19)	3 (1 to 5)	10 (10 to 10)
DR Congo	2017	19 (16 to 22)	2 (2 to 3)	8 (6 to 11)	1 (1 to 3)	7 (7 to 7)	30 (26 to 36)	4 (3 to 5)	13 (9 to 18)	2 (1 to 5)	11 (11 to 11)
DR Congo	2018	19 (16 to 23)	3 (2 to 3)	8 (6 to 12)	2 (1 to 3)	6 (6 to 6)	30 (25 to 36)	4 (3 to 5)	13 (9 to 19)	3 (1 to 5)	10 (10 to 10)
DR Congo	2019	19 (16 to 23)	3 (2 to 3)	8 (6 to 12)	2 (1 to 3)	6 (6 to 6)	30 (25 to 36)	4 (3 to 5)	14 (9 to 19)	3 (1 to 5)	10 (10 to 10)
DR Congo	2020	19 (16 to 23)	3 (2 to 3)	8 (6 to 12)	2 (1 to 3)	6 (6 to 7)	31 (26 to 37)	4 (3 to 6)	14 (9 to 19)	3 (1 to 5)	10 (10 to 11)
DR Congo	2021	19 (16 to 23)	3 (2 to 4)	8 (6 to 12)	2 (1 to 3)	7 (6 to 7)	31 (26 to 38)	4 (3 to 6)	14 (9 to 19)	3 (1 to 5)	11 (10 to 11)
DR Congo	2022	20 (16 to 23)	3 (2 to 4)	9 (6 to 12)	2 (1 to 3)	7 (6 to 7)	32 (26 to 38)	4 (3 to 6)	14 (9 to 19)	3 (1 to 5)	11 (10 to 12)
DR Congo	2023	20 (16 to 24)	3 (2 to 4)	9 (6 to 12)	2 (1 to 3)	7 (6 to 7)	32 (26 to 39)	5 (3 to 6)	14 (10 to 19)	3 (1 to 5)	11 (10 to 12)
DR Congo	2024	20 (16 to 24)	3 (2 to 4)	9 (6 to 12)	2 (1 to 3)	7 (6 to 8)	32 (26 to 39)	5 (3 to 6)	14 (10 to 20)	3 (1 to 5)	11 (10 to 12)
DR Congo	2025	20 (16 to 24)	3 (2 to 4)	9 (6 to 12)	2 (1 to 3)	7 (6 to 8)	33 (27 to 40)	5 (3 to 6)	14 (10 to 20)	3 (1 to 5)	11 (10 to 12)
DR Congo	2026	20 (17 to 25)	3 (2 to 4)	9 (6 to 12)	2 (1 to 3)	7 (6 to 8)	33 (27 to 40)	5 (3 to 7)	14 (10 to 20)	3 (1 to 5)	11 (10 to 12)
DR Congo	2027	21 (17 to 25)	3 (2 to 4)	9 (6 to 12)	2 (1 to 3)	7 (6 to 8)	33 (27 to 41)	5 (3 to 7)	14 (10 to 20)	3 (1 to 5)	11 (10 to 13)
DR Congo	2028	21 (17 to 25)	3 (2 to 4)	9 (6 to 13)	2 (1 to 3)	7 (6 to 8)	34 (28 to 41)	5 (3 to 7)	15 (10 to 20)	3 (1 to 6)	11 (10 to 13)

DR Congo	2029	21 (17 to 26)	3 (2 to 4)	9 (6 to 13)	2 (1 to 3)	7 (6 to 8)	34 (28 to 41)	5 (4 to 7)	15 (10 to 21)	3 (1 to 6)	11 (10 to 13)
DR Congo	2030	21 (17 to 26)	3 (2 to 5)	9 (6 to 13)	2 (1 to 4)	7 (6 to 8)	35 (28 to 42)	5 (4 to 8)	15 (10 to 21)	3 (1 to 6)	11 (10 to 13)
DR Congo	2031	22 (18 to 26)	3 (2 to 5)	9 (6 to 13)	2 (1 to 4)	7 (6 to 8)	35 (28 to 43)	5 (4 to 8)	15 (10 to 21)	3 (2 to 6)	11 (10 to 13)
DR Congo	2032	22 (18 to 27)	3 (2 to 5)	9 (6 to 13)	2 (1 to 4)	7 (6 to 9)	35 (29 to 44)	6 (4 to 8)	15 (10 to 21)	3 (2 to 6)	11 (10 to 14)
DR Congo	2033	22 (18 to 27)	4 (2 to 5)	10 (6 to 13)	2 (1 to 4)	7 (6 to 9)	36 (29 to 44)	6 (4 to 8)	15 (10 to 22)	3 (2 to 6)	11 (10 to 14)
DR Congo	2034	23 (18 to 28)	4 (2 to 5)	10 (7 to 13)	2 (1 to 4)	7 (6 to 9)	37 (29 to 45)	6 (4 to 9)	16 (11 to 22)	3 (2 to 6)	12 (10 to 14)
DR Congo	2035	23 (19 to 29)	4 (3 to 5)	10 (7 to 14)	2 (1 to 4)	7 (6 to 9)	37 (30 to 46)	6 (4 to 9)	16 (11 to 22)	3 (2 to 6)	12 (10 to 14)
DR Congo	2036	23 (19 to 29)	4 (3 to 6)	10 (7 to 14)	2 (1 to 4)	7 (6 to 9)	38 (30 to 47)	6 (4 to 9)	16 (11 to 22)	3 (2 to 6)	12 (10 to 15)
DR Congo	2037	24 (19 to 30)	4 (3 to 6)	10 (7 to 14)	2 (1 to 4)	7 (6 to 9)	38 (31 to 48)	7 (4 to 10)	16 (11 to 23)	3 (2 to 6)	12 (10 to 15)
DR Congo	2038	24 (19 to 30)	4 (3 to 6)	10 (7 to 14)	2 (1 to 4)	7 (6 to 9)	39 (31 to 49)	7 (4 to 10)	16 (11 to 23)	4 (2 to 7)	12 (10 to 15)
DR Congo	2039	24 (19 to 30)	4 (3 to 6)	10 (7 to 14)	2 (1 to 4)	8 (6 to 10)	39 (31 to 49)	7 (5 to 10)	17 (11 to 23)	4 (2 to 7)	12 (10 to 15)
DR Congo	2040	25 (20 to 31)	4 (3 to 7)	10 (7 to 14)	2 (1 to 4)	8 (6 to 10)	40 (32 to 50)	7 (5 to 11)	17 (11 to 23)	4 (2 to 7)	12 (10 to 16)
DR Congo	2041	25 (20 to 31)	5 (3 to 7)	10 (7 to 15)	2 (1 to 4)	8 (6 to 10)	40 (33 to 51)	7 (5 to 11)	17 (11 to 24)	4 (2 to 7)	12 (10 to 16)
DR Congo	2042	25 (20 to 32)	5 (3 to 7)	11 (7 to 15)	2 (1 to 4)	8 (6 to 10)	41 (33 to 51)	7 (5 to 11)	17 (12 to 24)	4 (2 to 7)	13 (10 to 16)
DR Congo	2043	26 (21 to 32)	5 (3 to 7)	11 (7 to 15)	2 (1 to 4)	8 (6 to 10)	42 (33 to 52)	8 (5 to 12)	17 (12 to 24)	4 (2 to 7)	13 (10 to 17)

DR Congo	2044	26 (21 to 32)	5 (3 to 7)	11 (7 to 15)	2 (1 to 5)	8 (6 to 10)	42 (34 to 52)	8 (5 to 12)	18 (12 to 24)	4 (2 to 7)	13 (10 to 17)
DR Congo	2045	26 (21 to 33)	5 (3 to 8)	11 (7 to 15)	2 (1 to 5)	8 (6 to 10)	43 (34 to 54)	8 (5 to 12)	18 (12 to 25)	4 (2 to 8)	13 (10 to 17)
DR Congo	2046	27 (21 to 34)	5 (3 to 8)	11 (7 to 15)	3 (1 to 5)	8 (6 to 11)	43 (35 to 54)	8 (5 to 13)	18 (12 to 25)	4 (2 to 8)	13 (10 to 17)
DR Congo	2047	27 (22 to 34)	5 (3 to 8)	11 (7 to 16)	3 (1 to 5)	8 (6 to 11)	44 (35 to 55)	8 (5 to 13)	18 (12 to 25)	4 (2 to 8)	13 (11 to 18)
DR Congo	2048	28 (22 to 35)	5 (3 to 8)	11 (8 to 16)	3 (1 to 5)	8 (7 to 11)	45 (36 to 56)	9 (5 to 14)	18 (12 to 26)	4 (2 to 8)	14 (11 to 18)
DR Congo	2049	28 (22 to 35)	5 (3 to 9)	11 (8 to 16)	3 (1 to 5)	9 (7 to 11)	45 (36 to 57)	9 (5 to 14)	18 (12 to 26)	4 (2 to 8)	14 (11 to 18)
DR Congo	2050	28 (23 to 36)	6 (3 to 9)	12 (8 to 16)	3 (1 to 5)	9 (7 to 12)	46 (37 to 58)	9 (6 to 14)	19 (12 to 26)	4 (2 to 8)	14 (11 to 19)
Denmark	1995	3527 (3395 to 3665)	2875 (2751 to 3005)	604 (564 to 647)	48 (34 to 66)	0 (0 to 0)	3189 (3069 to 3313)	2600 (2487 to 2717)	546 (510 to 585)	43 (31 to 59)	0 (0 to 0)
Denmark	1996	3608 (3515 to 3706)	2941 (2855 to 3030)	616 (585 to 649)	51 (38 to 67)	0 (0 to 0)	3262 (3178 to 3351)	2659 (2581 to 2740)	557 (529 to 586)	46 (34 to 61)	0 (0 to 0)
Denmark	1997	3712 (3615 to 3808)	3029 (2940 to 3118)	629 (600 to 660)	54 (41 to 69)	0 (0 to 0)	3356 (3268 to 3443)	2739 (2658 to 2819)	569 (543 to 597)	49 (37 to 62)	0 (0 to 0)
Denmark	1998	3879 (3781 to 3972)	3180 (3086 to 3270)	640 (612 to 671)	59 (45 to 73)	0 (0 to 0)	3507 (3419 to 3591)	2875 (2791 to 2957)	579 (553 to 607)	53 (41 to 66)	0 (0 to 0)
Denmark	1999	4105 (4010 to 4193)	3394 (3308 to 3480)	649 (622 to 676)	62 (49 to 76)	0 (0 to 0)	3711 (3625 to 3791)	3068 (2991 to 3147)	587 (563 to 611)	56 (44 to 69)	0 (0 to 0)

Denmark	2000	4285 (4192 to 4379)	3564 (3476 to 3655)	657 (632 to 681)	64 (53 to 78)	0 (0 to 0)	3875 (3790 to 3960)	3223 (3143 to 3305)	594 (571 to 616)	58 (48 to 71)	0 (0 to 0)
Denmark	2001	4428 (4332 to 4526)	3697 (3607 to 3792)	665 (642 to 690)	66 (55 to 79)	0 (0 to 0)	4003 (3917 to 4092)	3342 (3261 to 3429)	601 (581 to 624)	60 (49 to 72)	0 (0 to 0)
Denmark	2002	4577 (4476 to 4675)	3832 (3737 to 3924)	676 (654 to 702)	69 (57 to 82)	0 (0 to 0)	4138 (4047 to 4227)	3465 (3378 to 3548)	612 (591 to 635)	62 (52 to 75)	0 (0 to 0)
Denmark	2003	4687 (4588 to 4789)	3925 (3828 to 4020)	690 (668 to 714)	72 (61 to 86)	0 (0 to 0)	4238 (4148 to 4330)	3549 (3461 to 3635)	624 (604 to 646)	65 (55 to 77)	0 (0 to 0)
Denmark	2004	4878 (4780 to 4977)	4084 (3989 to 4185)	717 (693 to 741)	76 (64 to 90)	0 (0 to 0)	4410 (4322 to 4500)	3693 (3607 to 3784)	648 (627 to 670)	69 (58 to 82)	0 (0 to 0)
Denmark	2005	5002 (4907 to 5095)	4186 (4093 to 4277)	736 (712 to 759)	79 (67 to 94)	0 (0 to 0)	4522 (4437 to 4606)	3785 (3701 to 3867)	665 (644 to 687)	72 (61 to 85)	0 (0 to 0)
Denmark	2006	5206 (5107 to 5299)	4363 (4268 to 4453)	758 (734 to 784)	85 (73 to 101)	0 (0 to 0)	4707 (4617 to 4791)	3945 (3859 to 4026)	685 (664 to 709)	77 (66 to 91)	0 (0 to 0)
Denmark	2007	5331 (5231 to 5439)	4471 (4373 to 4572)	769 (747 to 797)	91 (78 to 107)	0 (0 to 0)	4820 (4730 to 4918)	4042 (3954 to 4134)	695 (675 to 720)	83 (70 to 97)	0 (0 to 0)
Denmark	2008	5446 (5345 to 5545)	4575 (4481 to 4672)	774 (749 to 800)	97 (82 to 112)	0 (0 to 0)	4924 (4833 to 5014)	4137 (4052 to 4224)	700 (677 to 723)	87 (74 to 101)	0 (0 to 0)
Denmark	2009	5650 (5546 to 5744)	4756 (4660 to 4848)	792 (766 to 817)	102 (86 to 117)	0 (0 to 0)	5109 (5014 to 5193)	4301 (4214 to 4384)	716 (693 to 738)	92 (78 to 106)	0 (0 to 0)

Denmark	2010	5651 (5545 to 5754)	4745 (4651 to 4846)	803 (779 to 828)	102 (87 to 118)	0 (0 to 0)	5109 (5014 to 5202)	4290 (4205 to 4381)	726 (705 to 748)	93 (79 to 107)	0 (0 to 0)
Denmark	2011	5590 (5493 to 5689)	4684 (4594 to 4780)	802 (776 to 828)	104 (88 to 121)	0 (0 to 0)	5054 (4967 to 5143)	4235 (4154 to 4322)	725 (702 to 748)	94 (80 to 109)	0 (0 to 0)
Denmark	2012	5640 (5539 to 5749)	4736 (4641 to 4839)	797 (771 to 822)	108 (91 to 125)	0 (0 to 0)	5099 (5008 to 5198)	4282 (4196 to 4375)	720 (698 to 743)	97 (82 to 113)	0 (0 to 0)
Denmark	2013	5587 (5487 to 5684)	4697 (4598 to 4789)	780 (756 to 806)	110 (94 to 127)	0 (0 to 0)	5051 (4961 to 5139)	4246 (4157 to 4330)	706 (684 to 728)	99 (85 to 115)	0 (0 to 0)
Denmark	2014	5659 (5562 to 5762)	4764 (4673 to 4862)	780 (756 to 805)	115 (100 to 133)	0 (0 to 0)	5117 (5029 to 5209)	4307 (4225 to 4396)	706 (683 to 728)	104 (90 to 120)	0 (0 to 0)
Denmark	2015	5793 (5687 to 5895)	4879 (4778 to 4974)	792 (765 to 818)	122 (106 to 142)	0 (0 to 0)	5238 (5142 to 5330)	4411 (4320 to 4497)	716 (692 to 740)	111 (96 to 128)	0 (0 to 0)
Denmark	2016	5846 (5746 to 5955)	4918 (4817 to 5022)	801 (774 to 827)	127 (109 to 151)	0 (0 to 0)	5285 (5195 to 5384)	4446 (4355 to 4540)	724 (700 to 748)	115 (98 to 136)	0 (0 to 0)
Denmark	2017	5933 (5782 to 6079)	4987 (4852 to 5118)	814 (779 to 848)	132 (108 to 163)	0 (0 to 0)	5364 (5227 to 5496)	4509 (4387 to 4627)	736 (704 to 767)	120 (98 to 147)	0 (0 to 0)
Denmark	2018	6068 (5908 to 6218)	5111 (4962 to 5252)	821 (786 to 856)	135 (110 to 166)	0 (0 to 0)	5486 (5341 to 5622)	4622 (4486 to 4749)	743 (711 to 774)	122 (99 to 150)	0 (0 to 0)
Denmark	2019	6178 (6004 to 6342)	5210 (5046 to 5362)	831 (795 to 867)	137 (112 to 169)	0 (0 to 0)	5586 (5428 to 5734)	4710 (4562 to 4848)	751 (719 to 784)	124 (101 to 153)	0 (0 to 0)

Denmark	2020	6256 (6071 to 6421)	5275 (5104 to 5432)	841 (805 to 877)	139 (113 to 171)	0 (0 to 0)	5656 (5489 to 5806)	4770 (4615 to 4911)	761 (728 to 793)	126 (103 to 155)	0 (0 to 0)
Denmark	2021	6299 (6120 to 6474)	5312 (5128 to 5478)	846 (810 to 883)	141 (114 to 173)	0 (0 to 0)	5695 (5533 to 5853)	4803 (4637 to 4953)	765 (732 to 798)	127 (103 to 156)	0 (0 to 0)
Denmark	2022	6345 (6150 to 6523)	5352 (5166 to 5530)	851 (815 to 888)	142 (115 to 174)	0 (0 to 0)	5737 (5560 to 5898)	4839 (4671 to 5000)	769 (737 to 803)	128 (104 to 158)	0 (0 to 0)
Denmark	2023	6371 (6176 to 6567)	5378 (5178 to 5563)	851 (815 to 889)	143 (116 to 176)	0 (0 to 0)	5761 (5584 to 5937)	4862 (4682 to 5030)	770 (737 to 804)	129 (105 to 159)	0 (0 to 0)
Denmark	2024	6390 (6191 to 6587)	5398 (5196 to 5590)	850 (813 to 888)	143 (117 to 176)	0 (0 to 0)	5778 (5598 to 5956)	4880 (4698 to 5054)	768 (735 to 803)	129 (105 to 160)	0 (0 to 0)
Denmark	2025	6414 (6215 to 6609)	5422 (5217 to 5621)	848 (810 to 887)	144 (117 to 177)	0 (0 to 0)	5799 (5619 to 5976)	4902 (4717 to 5082)	767 (733 to 802)	130 (106 to 160)	0 (0 to 0)
Denmark	2026	6433 (6216 to 6639)	5443 (5227 to 5646)	846 (808 to 885)	144 (117 to 178)	0 (0 to 0)	5817 (5620 to 6003)	4921 (4726 to 5105)	765 (731 to 800)	130 (106 to 161)	0 (0 to 0)
Denmark	2027	6454 (6242 to 6670)	5466 (5246 to 5677)	844 (806 to 883)	145 (118 to 179)	0 (0 to 0)	5836 (5643 to 6031)	4942 (4743 to 5133)	763 (728 to 798)	131 (106 to 161)	0 (0 to 0)
Denmark	2028	6478 (6252 to 6691)	5490 (5262 to 5714)	842 (803 to 882)	145 (118 to 180)	0 (0 to 0)	5857 (5653 to 6049)	4964 (4757 to 5166)	761 (726 to 797)	131 (107 to 162)	0 (0 to 0)
Denmark	2029	6504 (6275 to 6731)	5518 (5274 to 5746)	841 (801 to 881)	146 (118 to 181)	0 (0 to 0)	5881 (5673 to 6086)	4989 (4768 to 5196)	760 (724 to 797)	132 (107 to 163)	0 (0 to 0)

Denmark	2030	6537 (6302 to 6768)	5550 (5306 to 5793)	840 (800 to 882)	147 (119 to 182)	0 (0 to 0)	5911 (5698 to 6119)	5018 (4797 to 5238)	760 (723 to 797)	133 (107 to 165)	0 (0 to 0)
Denmark	2031	6573 (6328 to 6805)	5585 (5328 to 5832)	841 (799 to 883)	148 (119 to 184)	0 (0 to 0)	5943 (5721 to 6152)	5050 (4818 to 5273)	760 (723 to 799)	134 (108 to 166)	0 (0 to 0)
Denmark	2032	6606 (6363 to 6850)	5616 (5361 to 5871)	841 (799 to 885)	149 (120 to 185)	0 (0 to 0)	5973 (5753 to 6194)	5078 (4847 to 5308)	761 (722 to 800)	134 (109 to 167)	0 (0 to 0)
Denmark	2033	6635 (6392 to 6879)	5644 (5390 to 5900)	842 (798 to 886)	149 (121 to 186)	0 (0 to 0)	5999 (5779 to 6220)	5103 (4873 to 5334)	761 (721 to 801)	135 (109 to 168)	0 (0 to 0)
Denmark	2034	6666 (6394 to 6917)	5673 (5392 to 5943)	843 (797 to 887)	150 (121 to 188)	0 (0 to 0)	6027 (5781 to 6254)	5129 (4875 to 5374)	762 (721 to 802)	136 (110 to 170)	0 (0 to 0)
Denmark	2035	6697 (6427 to 6967)	5702 (5410 to 5983)	843 (797 to 889)	151 (122 to 189)	0 (0 to 0)	6055 (5811 to 6299)	5156 (4892 to 5409)	763 (720 to 804)	137 (110 to 171)	0 (0 to 0)
Denmark	2036	6731 (6440 to 7002)	5734 (5433 to 6023)	845 (796 to 891)	152 (123 to 191)	0 (0 to 0)	6085 (5823 to 6330)	5184 (4912 to 5446)	764 (720 to 806)	137 (111 to 172)	0 (0 to 0)
Denmark	2037	6766 (6466 to 7060)	5767 (5445 to 6060)	846 (797 to 894)	153 (124 to 192)	0 (0 to 0)	6117 (5846 to 6384)	5214 (4923 to 5479)	765 (721 to 808)	138 (112 to 174)	0 (0 to 0)
Denmark	2038	6806 (6490 to 7107)	5804 (5481 to 6103)	849 (798 to 897)	154 (124 to 194)	0 (0 to 0)	6154 (5868 to 6426)	5247 (4956 to 5518)	767 (721 to 811)	139 (113 to 175)	0 (0 to 0)
Denmark	2039	6850 (6520 to 7158)	5844 (5502 to 6157)	851 (799 to 901)	155 (125 to 196)	0 (0 to 0)	6194 (5895 to 6472)	5284 (4974 to 5567)	769 (723 to 815)	141 (113 to 177)	0 (0 to 0)

Denmark	2040	6895 (6566 to 7223)	5885 (5543 to 6217)	854 (801 to 905)	157 (126 to 198)	0 (0 to 0)	6234 (5936 to 6531)	5320 (5012 to 5621)	772 (724 to 818)	142 (114 to 179)	0 (0 to 0)
Denmark	2041	6945 (6606 to 7304)	5930 (5578 to 6295)	857 (803 to 910)	158 (127 to 200)	0 (0 to 0)	6279 (5973 to 6604)	5361 (5043 to 5691)	775 (726 to 822)	143 (115 to 181)	0 (0 to 0)
Denmark	2042	6999 (6651 to 7383)	5978 (5621 to 6357)	861 (805 to 916)	160 (128 to 202)	0 (0 to 0)	6328 (6013 to 6675)	5405 (5082 to 5747)	778 (728 to 828)	144 (116 to 182)	0 (0 to 0)
Denmark	2043	7059 (6689 to 7456)	6032 (5657 to 6436)	866 (809 to 922)	161 (129 to 204)	0 (0 to 0)	6382 (6048 to 6741)	5454 (5115 to 5819)	783 (731 to 834)	146 (117 to 184)	0 (0 to 0)
Denmark	2044	7121 (6743 to 7568)	6087 (5710 to 6528)	871 (813 to 930)	163 (130 to 206)	0 (0 to 0)	6439 (6097 to 6843)	5504 (5163 to 5902)	788 (735 to 840)	147 (118 to 186)	0 (0 to 0)
Denmark	2045	7189 (6782 to 7679)	6147 (5759 to 6635)	877 (817 to 937)	165 (132 to 208)	0 (0 to 0)	6500 (6131 to 6943)	5558 (5207 to 5999)	793 (738 to 848)	149 (119 to 188)	0 (0 to 0)
Denmark	2046	7258 (6833 to 7794)	6209 (5792 to 6722)	883 (820 to 945)	167 (133 to 210)	0 (0 to 0)	6562 (6178 to 7047)	5614 (5237 to 6078)	798 (741 to 854)	151 (120 to 190)	0 (0 to 0)
Denmark	2047	7326 (6885 to 7867)	6269 (5830 to 6804)	889 (824 to 953)	169 (135 to 213)	0 (0 to 0)	6624 (6225 to 7113)	5668 (5271 to 6152)	804 (745 to 861)	152 (122 to 193)	0 (0 to 0)
Denmark	2048	7397 (6925 to 7968)	6331 (5881 to 6889)	895 (828 to 961)	171 (136 to 216)	0 (0 to 0)	6688 (6261 to 7205)	5724 (5318 to 6228)	809 (748 to 869)	154 (123 to 195)	0 (0 to 0)
Denmark	2049	7468 (6981 to 8092)	6394 (5927 to 6990)	901 (832 to 969)	173 (138 to 219)	0 (0 to 0)	6752 (6312 to 7316)	5781 (5359 to 6320)	815 (752 to 876)	156 (125 to 198)	0 (0 to 0)

Denmark	2050	7538 (7022 to 8193)	6456 (5962 to 7085)	907 (836 to 978)	175 (140 to 221)	0 (0 to 0)	6815 (6349 to 7408)	5837 (5390 to 6406)	820 (756 to 884)	158 (126 to 200)	0 (0 to 0)
Djibouti	1995	75 (63 to 91)	27 (21 to 35)	33 (23 to 46)	0 (0 to 1)	15 (15 to 15)	135 (113 to 164)	49 (37 to 63)	59 (42 to 83)	1 (0 to 1)	27 (27 to 27)
Djibouti	1996	60 (49 to 74)	25 (19 to 32)	31 (22 to 43)	0 (0 to 1)	4 (4 to 4)	109 (89 to 133)	45 (34 to 58)	55 (40 to 78)	1 (0 to 1)	8 (8 to 8)
Djibouti	1997	82 (71 to 96)	27 (21 to 35)	31 (22 to 43)	0 (0 to 1)	24 (24 to 24)	148 (127 to 174)	48 (37 to 62)	56 (39 to 77)	1 (0 to 1)	43 (43 to 43)
Djibouti	1998	84 (73 to 98)	27 (21 to 35)	30 (22 to 42)	0 (0 to 1)	26 (26 to 26)	152 (131 to 177)	49 (38 to 63)	55 (39 to 76)	1 (0 to 1)	47 (47 to 47)
Djibouti	1999	69 (57 to 83)	29 (22 to 37)	31 (22 to 43)	0 (0 to 1)	9 (9 to 9)	125 (103 to 150)	52 (40 to 66)	56 (39 to 77)	1 (0 to 1)	16 (16 to 16)
Djibouti	2000	67 (55 to 81)	28 (22 to 36)	31 (22 to 42)	0 (0 to 1)	7 (7 to 7)	120 (100 to 145)	51 (39 to 65)	55 (40 to 75)	1 (0 to 1)	13 (13 to 13)
Djibouti	2001	57 (47 to 70)	26 (20 to 33)	29 (21 to 39)	0 (0 to 1)	2 (2 to 2)	103 (85 to 126)	46 (36 to 59)	53 (38 to 71)	1 (0 to 1)	3 (3 to 3)
Djibouti	2002	60 (50 to 73)	26 (20 to 33)	29 (21 to 39)	0 (0 to 1)	5 (5 to 5)	108 (89 to 131)	48 (37 to 60)	52 (38 to 70)	1 (0 to 1)	8 (8 to 8)
Djibouti	2003	62 (51 to 74)	29 (22 to 36)	28 (21 to 39)	0 (0 to 1)	4 (4 to 4)	111 (92 to 134)	52 (40 to 66)	51 (38 to 69)	1 (0 to 1)	7 (7 to 7)
Djibouti	2004	69 (59 to 82)	30 (23 to 38)	27 (20 to 37)	0 (0 to 1)	12 (12 to 12)	125 (106 to 148)	54 (42 to 68)	49 (37 to 67)	1 (0 to 1)	21 (21 to 21)
Djibouti	2005	75 (65 to 87)	30 (24 to 38)	26 (19 to 35)	0 (0 to 1)	18 (18 to 18)	135 (117 to 157)	55 (43 to 69)	47 (35 to 63)	1 (0 to 1)	33 (33 to 33)
Djibouti	2006	75 (65 to 87)	32 (25 to 40)	24 (18 to 33)	0 (0 to 1)	19 (19 to 19)	136 (118 to 156)	57 (45 to 71)	44 (32 to 60)	1 (0 to 1)	34 (34 to 34)
Djibouti	2007	81 (71 to 93)	35 (27 to 44)	24 (17 to 33)	0 (0 to 1)	23 (23 to 23)	147 (128 to 169)	63 (49 to 79)	43 (31 to 59)	1 (0 to 1)	41 (41 to 41)
Djibouti	2008	82 (71 to 95)	39 (31 to 49)	22 (16 to 30)	0 (0 to 1)	21 (21 to 21)	148 (128 to 171)	70 (55 to 88)	40 (29 to 55)	1 (0 to 1)	37 (37 to 37)

Djibouti	2009	77 (67 to 90)	40 (32 to 51)	21 (15 to 28)	0 (0 to 1)	16 (16 to 16)	139 (120 to 162)	72 (57 to 91)	37 (27 to 51)	1 (0 to 1)	29 (29 to 29)
Djibouti	2010	64 (54 to 75)	37 (29 to 47)	18 (13 to 25)	0 (0 to 1)	8 (8 to 8)	115 (98 to 136)	66 (53 to 85)	33 (24 to 45)	1 (0 to 1)	15 (15 to 15)
Djibouti	2011	66 (56 to 78)	38 (30 to 48)	17 (12 to 24)	1 (0 to 1)	10 (10 to 10)	119 (101 to 140)	68 (53 to 86)	31 (22 to 43)	1 (0 to 2)	19 (19 to 19)
Djibouti	2012	73 (63 to 85)	38 (30 to 49)	17 (12 to 23)	1 (0 to 1)	17 (17 to 17)	131 (114 to 153)	69 (54 to 88)	30 (21 to 42)	1 (1 to 2)	31 (31 to 31)
Djibouti	2013	65 (55 to 77)	37 (29 to 48)	16 (11 to 22)	1 (0 to 1)	11 (11 to 11)	117 (100 to 138)	67 (53 to 87)	29 (20 to 39)	1 (1 to 2)	20 (20 to 20)
Djibouti	2014	64 (54 to 76)	38 (30 to 49)	16 (11 to 22)	1 (0 to 1)	9 (9 to 9)	115 (97 to 137)	68 (53 to 89)	28 (20 to 39)	1 (1 to 2)	17 (17 to 17)
Djibouti	2015	74 (63 to 88)	44 (34 to 57)	17 (12 to 23)	1 (0 to 1)	13 (13 to 13)	133 (113 to 158)	79 (61 to 102)	30 (21 to 42)	1 (1 to 3)	23 (23 to 23)
Djibouti	2016	66 (56 to 78)	37 (28 to 48)	16 (11 to 22)	1 (0 to 1)	13 (13 to 13)	119 (101 to 140)	67 (51 to 86)	29 (19 to 40)	1 (1 to 2)	23 (23 to 23)
Djibouti	2017	57 (48 to 69)	32 (24 to 41)	15 (10 to 21)	0 (0 to 1)	9 (9 to 9)	104 (87 to 124)	58 (44 to 74)	28 (19 to 38)	1 (0 to 2)	17 (17 to 17)
Djibouti	2018	57 (47 to 68)	32 (24 to 41)	16 (11 to 22)	1 (0 to 1)	9 (9 to 9)	102 (86 to 123)	57 (43 to 74)	28 (19 to 39)	1 (0 to 2)	16 (16 to 16)
Djibouti	2019	56 (46 to 67)	30 (23 to 40)	16 (11 to 22)	0 (0 to 1)	9 (9 to 9)	101 (84 to 121)	55 (41 to 71)	29 (20 to 40)	1 (0 to 2)	16 (16 to 16)
Djibouti	2020	57 (48 to 69)	31 (23 to 41)	17 (11 to 23)	1 (0 to 1)	9 (8 to 9)	103 (86 to 125)	57 (42 to 75)	30 (20 to 41)	1 (0 to 2)	16 (15 to 17)
Djibouti	2021	58 (48 to 71)	32 (24 to 42)	17 (11 to 23)	1 (0 to 1)	9 (8 to 10)	105 (87 to 127)	58 (42 to 76)	30 (20 to 42)	1 (0 to 2)	16 (15 to 17)
Djibouti	2022	59 (49 to 71)	32 (23 to 43)	17 (11 to 23)	1 (0 to 1)	9 (8 to 10)	106 (88 to 129)	58 (42 to 77)	30 (21 to 42)	1 (0 to 2)	16 (15 to 18)
Djibouti	2023	59 (49 to 71)	32 (23 to 43)	17 (11 to 23)	1 (0 to 1)	9 (8 to 10)	106 (88 to 129)	58 (42 to 78)	31 (21 to 42)	1 (0 to 2)	16 (15 to 18)

Djibouti	2024	59 (49 to 72)	32 (23 to 43)	17 (12 to 24)	1 (0 to 1)	9 (8 to 10)	107 (88 to 130)	59 (42 to 78)	31 (21 to 42)	1 (0 to 2)	16 (15 to 18)
Djibouti	2025	60 (49 to 73)	33 (24 to 44)	17 (12 to 24)	1 (0 to 1)	9 (8 to 10)	108 (89 to 131)	59 (43 to 79)	31 (21 to 43)	1 (0 to 2)	17 (15 to 19)
Djibouti	2026	60 (49 to 74)	33 (24 to 45)	17 (12 to 24)	1 (0 to 1)	9 (8 to 11)	109 (89 to 133)	60 (43 to 81)	31 (21 to 43)	1 (0 to 2)	17 (15 to 19)
Djibouti	2027	61 (50 to 75)	34 (24 to 46)	17 (12 to 24)	1 (0 to 1)	9 (8 to 11)	110 (90 to 135)	61 (43 to 83)	32 (21 to 43)	1 (0 to 2)	17 (15 to 20)
Djibouti	2028	62 (50 to 76)	34 (24 to 47)	18 (12 to 24)	1 (0 to 1)	9 (8 to 11)	112 (90 to 138)	62 (43 to 85)	32 (22 to 44)	1 (0 to 2)	17 (15 to 20)
Djibouti	2029	62 (50 to 77)	34 (23 to 47)	18 (12 to 25)	1 (0 to 1)	10 (8 to 11)	112 (91 to 139)	62 (42 to 85)	32 (22 to 44)	1 (1 to 2)	17 (15 to 20)
Djibouti	2030	63 (51 to 79)	35 (24 to 48)	18 (12 to 25)	1 (0 to 1)	10 (8 to 12)	113 (92 to 142)	62 (42 to 87)	33 (22 to 45)	1 (1 to 2)	17 (15 to 21)
Djibouti	2031	63 (51 to 79)	35 (24 to 49)	18 (12 to 25)	1 (0 to 1)	10 (8 to 12)	114 (92 to 143)	63 (43 to 89)	33 (22 to 45)	1 (1 to 2)	18 (15 to 21)
Djibouti	2032	64 (51 to 81)	35 (24 to 50)	19 (12 to 26)	1 (0 to 1)	10 (8 to 12)	116 (93 to 146)	64 (43 to 91)	33 (23 to 46)	1 (1 to 2)	18 (15 to 22)
Djibouti	2033	66 (52 to 82)	36 (24 to 52)	19 (13 to 26)	1 (0 to 1)	10 (9 to 12)	118 (94 to 148)	65 (43 to 93)	34 (23 to 47)	1 (1 to 2)	18 (15 to 22)
Djibouti	2034	67 (53 to 85)	37 (25 to 53)	19 (13 to 27)	1 (0 to 1)	10 (9 to 12)	121 (96 to 153)	67 (45 to 96)	35 (23 to 48)	1 (1 to 2)	18 (15 to 22)
Djibouti	2035	68 (54 to 86)	38 (25 to 55)	19 (13 to 27)	1 (0 to 1)	10 (9 to 13)	123 (98 to 155)	68 (45 to 99)	35 (24 to 49)	1 (1 to 2)	19 (15 to 23)
Djibouti	2036	69 (55 to 88)	39 (25 to 56)	20 (13 to 27)	1 (0 to 1)	10 (9 to 13)	125 (99 to 158)	69 (45 to 101)	36 (24 to 49)	1 (1 to 2)	19 (16 to 23)
Djibouti	2037	71 (55 to 91)	39 (26 to 58)	20 (14 to 28)	1 (0 to 1)	11 (9 to 13)	128 (100 to 163)	71 (47 to 105)	36 (24 to 50)	1 (1 to 2)	19 (16 to 23)
Djibouti	2038	72 (56 to 93)	40 (26 to 59)	21 (14 to 28)	1 (0 to 1)	11 (9 to 13)	130 (101 to 167)	73 (47 to 107)	37 (25 to 51)	1 (1 to 2)	19 (16 to 24)

Djibouti	2039	74 (58 to 95)	41 (26 to 60)	21 (14 to 29)	1 (0 to 1)	11 (9 to 14)	133 (104 to 171)	74 (47 to 108)	38 (25 to 52)	1 (1 to 2)	20 (16 to 25)
Djibouti	2040	75 (58 to 96)	42 (27 to 61)	21 (14 to 29)	1 (0 to 1)	11 (9 to 14)	135 (105 to 173)	76 (48 to 110)	38 (26 to 53)	1 (1 to 2)	20 (16 to 25)
Djibouti	2041	76 (60 to 98)	43 (27 to 64)	21 (14 to 30)	1 (0 to 1)	11 (9 to 14)	137 (108 to 177)	77 (49 to 115)	39 (26 to 53)	1 (1 to 2)	20 (16 to 26)
Djibouti	2042	77 (60 to 99)	43 (28 to 64)	22 (15 to 30)	1 (0 to 1)	11 (9 to 15)	139 (108 to 178)	78 (50 to 115)	39 (26 to 54)	1 (1 to 3)	20 (17 to 26)
Djibouti	2043	78 (61 to 100)	44 (28 to 65)	22 (15 to 30)	1 (0 to 1)	12 (9 to 15)	140 (109 to 181)	79 (50 to 117)	39 (27 to 54)	1 (1 to 3)	21 (17 to 27)
Djibouti	2044	79 (61 to 102)	44 (28 to 67)	22 (15 to 30)	1 (0 to 1)	12 (9 to 15)	142 (109 to 183)	80 (50 to 121)	40 (27 to 55)	1 (1 to 3)	21 (17 to 28)
Djibouti	2045	80 (62 to 104)	45 (28 to 67)	22 (15 to 31)	1 (0 to 1)	12 (9 to 15)	144 (111 to 187)	81 (50 to 121)	40 (27 to 55)	1 (1 to 3)	22 (17 to 28)
Djibouti	2046	80 (62 to 104)	45 (28 to 68)	22 (15 to 31)	1 (0 to 1)	12 (10 to 16)	145 (112 to 188)	82 (51 to 123)	40 (27 to 55)	1 (1 to 3)	22 (17 to 28)
Djibouti	2047	81 (62 to 105)	46 (28 to 69)	22 (15 to 31)	1 (0 to 1)	12 (10 to 16)	146 (113 to 190)	82 (51 to 125)	40 (27 to 56)	1 (1 to 3)	22 (18 to 29)
Djibouti	2048	82 (63 to 107)	46 (29 to 70)	22 (15 to 31)	1 (0 to 2)	13 (10 to 17)	148 (113 to 193)	83 (52 to 127)	40 (27 to 56)	2 (1 to 3)	23 (18 to 30)
Djibouti	2049	83 (63 to 108)	47 (28 to 70)	22 (15 to 31)	1 (0 to 2)	13 (10 to 17)	149 (113 to 195)	84 (51 to 127)	40 (27 to 56)	2 (1 to 3)	23 (18 to 31)
Djibouti	2050	83 (63 to 109)	47 (29 to 72)	22 (15 to 31)	1 (0 to 2)	13 (10 to 18)	150 (114 to 197)	85 (51 to 129)	41 (27 to 56)	2 (1 to 3)	24 (19 to 32)
Dominica	1995	372 (336 to 416)	238 (210 to 270)	133 (108 to 158)	1 (0 to 2)	0 (0 to 0)	528 (477 to 590)	338 (297 to 383)	189 (154 to 224)	1 (1 to 3)	0 (0 to 0)
Dominica	1996	375 (344 to 410)	241 (216 to 265)	134 (112 to 156)	1 (0 to 2)	0 (0 to 0)	533 (488 to 582)	341 (307 to 377)	190 (159 to 222)	1 (1 to 2)	0 (0 to 0)
Dominica	1997	381 (352 to 412)	235 (213 to 258)	132 (112 to 152)	1 (0 to 1)	15 (15 to 15)	541 (500 to 585)	333 (303 to 366)	187 (160 to 216)	1 (0 to 2)	21 (21 to 21)

Dominica	1998	398 (368 to 430)	251 (227 to 276)	136 (117 to 156)	0 (0 to 1)	11 (11 to 11)	566 (522 to 610)	356 (323 to 392)	193 (166 to 222)	1 (0 to 1)	16 (16 to 16)
Dominica	1999	385 (355 to 417)	249 (226 to 275)	135 (118 to 156)	0 (0 to 0)	0 (0 to 0)	547 (504 to 592)	354 (320 to 391)	192 (167 to 222)	0 (0 to 1)	1 (1 to 1)
Dominica	2000	383 (352 to 415)	250 (227 to 275)	133 (115 to 153)	0 (0 to 0)	0 (0 to 0)	544 (500 to 589)	355 (322 to 391)	189 (164 to 217)	0 (0 to 0)	0 (0 to 0)
Dominica	2001	326 (300 to 352)	203 (183 to 224)	122 (106 to 141)	0 (0 to 0)	0 (0 to 0)	462 (425 to 499)	288 (260 to 317)	173 (151 to 201)	0 (0 to 0)	1 (1 to 1)
Dominica	2002	324 (298 to 352)	200 (181 to 220)	123 (107 to 143)	0 (0 to 0)	0 (0 to 0)	459 (422 to 500)	284 (257 to 313)	175 (152 to 202)	0 (0 to 0)	1 (1 to 1)
Dominica	2003	343 (317 to 372)	209 (189 to 231)	130 (113 to 150)	0 (0 to 0)	4 (4 to 4)	487 (449 to 528)	296 (268 to 328)	185 (161 to 213)	0 (0 to 0)	6 (6 to 6)
Dominica	2004	347 (319 to 375)	207 (186 to 228)	135 (117 to 154)	0 (0 to 0)	6 (6 to 6)	493 (452 to 533)	294 (264 to 323)	191 (166 to 218)	0 (0 to 0)	8 (8 to 8)
Dominica	2005	359 (329 to 386)	212 (192 to 233)	141 (123 to 159)	0 (0 to 0)	6 (6 to 6)	509 (466 to 548)	300 (272 to 331)	200 (174 to 226)	0 (0 to 1)	9 (9 to 9)
Dominica	2006	370 (341 to 398)	213 (192 to 234)	145 (126 to 165)	1 (0 to 2)	10 (10 to 10)	525 (484 to 566)	303 (272 to 332)	206 (179 to 234)	1 (1 to 3)	14 (14 to 14)
Dominica	2007	410 (379 to 443)	240 (217 to 263)	162 (141 to 183)	2 (1 to 5)	6 (6 to 6)	582 (538 to 629)	340 (308 to 374)	230 (201 to 260)	4 (2 to 7)	9 (9 to 9)
Dominica	2008	449 (417 to 482)	259 (236 to 283)	175 (153 to 197)	4 (2 to 7)	11 (11 to 11)	637 (592 to 685)	367 (335 to 402)	248 (217 to 280)	6 (3 to 11)	16 (16 to 16)
Dominica	2009	452 (420 to 485)	261 (237 to 285)	174 (151 to 197)	5 (2 to 9)	12 (12 to 12)	641 (596 to 689)	370 (336 to 405)	246 (214 to 279)	7 (3 to 13)	18 (18 to 18)
Dominica	2010	481 (447 to 516)	286 (259 to 312)	177 (155 to 201)	6 (3 to 11)	11 (11 to 11)	682 (635 to 732)	406 (368 to 444)	251 (220 to 286)	9 (4 to 16)	16 (16 to 16)
Dominica	2011	452 (422 to 486)	268 (245 to 291)	167 (146 to 190)	6 (3 to 11)	11 (11 to 11)	642 (598 to 690)	381 (348 to 413)	237 (207 to 270)	8 (4 to 15)	16 (16 to 16)
Dominica	2012	459 (428 to 494)	281 (257 to 305)	165 (144 to 189)	6 (3 to 11)	6 (6 to 6)	651 (607 to 701)	399 (365 to 433)	235 (204 to 268)	9 (4 to 16)	9 (9 to 9)

Dominica	2013	442 (412 to 474)	270 (248 to 293)	155 (134 to 175)	6 (3 to 11)	11 (11 to 11)	627 (585 to 672)	384 (352 to 416)	219 (190 to 249)	8 (4 to 15)	16 (16 to 16)
Dominica	2014	433 (404 to 466)	274 (251 to 297)	150 (131 to 171)	6 (3 to 11)	3 (3 to 3)	615 (574 to 662)	389 (357 to 421)	213 (186 to 243)	8 (4 to 15)	5 (5 to 5)
Dominica	2015	431 (401 to 462)	276 (252 to 301)	142 (122 to 163)	6 (3 to 10)	8 (8 to 8)	611 (569 to 656)	391 (357 to 427)	201 (173 to 231)	8 (4 to 15)	11 (11 to 11)
Dominica	2016	476 (437 to 515)	319 (287 to 353)	146 (125 to 170)	6 (3 to 12)	4 (4 to 4)	675 (620 to 732)	453 (408 to 501)	208 (178 to 242)	9 (5 to 17)	5 (5 to 5)
Dominica	2017	493 (445 to 550)	330 (293 to 371)	148 (124 to 177)	7 (3 to 13)	8 (8 to 8)	699 (631 to 780)	469 (416 to 527)	211 (176 to 251)	9 (4 to 18)	11 (11 to 11)
Dominica	2018	515 (462 to 575)	349 (305 to 399)	152 (127 to 181)	7 (3 to 13)	7 (7 to 7)	732 (655 to 817)	496 (433 to 566)	216 (181 to 257)	10 (5 to 19)	10 (10 to 10)
Dominica	2019	507 (452 to 574)	328 (283 to 380)	166 (139 to 197)	7 (3 to 13)	7 (7 to 7)	720 (641 to 815)	465 (401 to 539)	235 (197 to 280)	9 (5 to 19)	10 (10 to 10)
Dominica	2020	533 (473 to 604)	346 (295 to 403)	173 (145 to 206)	7 (3 to 14)	7 (7 to 7)	757 (672 to 858)	491 (419 to 572)	246 (206 to 293)	10 (5 to 20)	10 (9 to 11)
Dominica	2021	546 (481 to 624)	355 (301 to 419)	177 (148 to 210)	7 (4 to 14)	7 (7 to 8)	775 (683 to 886)	504 (427 to 594)	251 (210 to 298)	10 (5 to 20)	10 (10 to 11)
Dominica	2022	558 (487 to 638)	364 (302 to 435)	180 (150 to 214)	7 (4 to 15)	7 (7 to 8)	792 (691 to 906)	516 (429 to 618)	255 (213 to 303)	11 (5 to 21)	10 (10 to 12)
Dominica	2023	570 (494 to 659)	372 (306 to 453)	182 (153 to 218)	8 (4 to 15)	8 (7 to 8)	809 (701 to 936)	528 (434 to 642)	259 (217 to 309)	11 (5 to 21)	11 (10 to 12)
Dominica	2024	581 (497 to 674)	381 (312 to 463)	185 (155 to 221)	8 (4 to 15)	8 (7 to 9)	825 (705 to 957)	541 (443 to 657)	263 (220 to 314)	11 (5 to 22)	11 (10 to 12)
Dominica	2025	593 (512 to 691)	389 (316 to 477)	188 (157 to 225)	8 (4 to 16)	8 (7 to 9)	842 (727 to 981)	553 (448 to 678)	267 (223 to 319)	11 (5 to 22)	11 (10 to 13)
Dominica	2026	605 (512 to 708)	398 (321 to 493)	191 (160 to 228)	8 (4 to 16)	8 (7 to 9)	859 (727 to 1005)	565 (456 to 699)	271 (227 to 323)	11 (6 to 23)	11 (10 to 13)
Dominica	2027	616 (524 to 722)	406 (325 to 506)	193 (162 to 230)	8 (4 to 16)	8 (7 to 9)	874 (744 to 1025)	577 (461 to 718)	274 (230 to 327)	12 (6 to 23)	11 (10 to 13)

Dominica	2028	625 (524 to 742)	413 (326 to 516)	195 (164 to 234)	8 (4 to 16)	8 (7 to 10)	888 (744 to 1053)	587 (463 to 732)	277 (233 to 332)	12 (6 to 23)	12 (10 to 14)
Dominica	2029	635 (533 to 753)	420 (328 to 534)	198 (166 to 237)	8 (4 to 17)	8 (7 to 10)	901 (756 to 1069)	597 (466 to 758)	280 (235 to 336)	12 (6 to 24)	12 (10 to 14)
Dominica	2030	644 (536 to 774)	428 (331 to 545)	200 (168 to 239)	9 (4 to 17)	9 (7 to 10)	915 (761 to 1099)	607 (469 to 773)	283 (238 to 339)	12 (6 to 24)	12 (10 to 15)
Dominica	2031	652 (539 to 781)	433 (333 to 553)	201 (169 to 241)	9 (4 to 17)	9 (8 to 10)	926 (766 to 1108)	615 (473 to 785)	286 (240 to 342)	12 (6 to 25)	12 (11 to 15)
Dominica	2032	661 (548 to 798)	440 (338 to 560)	203 (170 to 243)	9 (4 to 18)	9 (8 to 11)	938 (778 to 1133)	625 (480 to 795)	288 (242 to 345)	13 (6 to 25)	13 (11 to 15)
Dominica	2033	669 (551 to 804)	447 (336 to 578)	204 (172 to 245)	9 (4 to 18)	9 (8 to 11)	950 (782 to 1142)	634 (477 to 821)	290 (244 to 347)	13 (6 to 25)	13 (11 to 16)
Dominica	2034	676 (553 to 823)	452 (341 to 585)	206 (173 to 246)	9 (4 to 18)	9 (8 to 11)	960 (784 to 1169)	642 (484 to 830)	292 (245 to 349)	13 (6 to 25)	13 (11 to 16)
Dominica	2035	683 (558 to 834)	457 (344 to 599)	207 (174 to 247)	9 (4 to 18)	10 (8 to 12)	969 (792 to 1183)	649 (488 to 850)	294 (246 to 350)	13 (6 to 26)	14 (11 to 17)
Dominica	2036	691 (560 to 844)	463 (348 to 607)	208 (174 to 248)	9 (5 to 18)	10 (8 to 12)	980 (795 to 1198)	658 (494 to 861)	295 (248 to 352)	13 (6 to 26)	14 (12 to 18)
Dominica	2037	699 (566 to 863)	470 (345 to 625)	209 (175 to 250)	9 (5 to 18)	10 (8 to 12)	992 (804 to 1225)	667 (490 to 888)	297 (249 to 354)	13 (6 to 26)	14 (12 to 18)
Dominica	2038	708 (571 to 875)	477 (351 to 633)	211 (176 to 252)	10 (5 to 19)	10 (9 to 13)	1005 (810 to 1243)	677 (498 to 899)	300 (250 to 357)	14 (7 to 27)	15 (12 to 19)
Dominica	2039	719 (577 to 895)	486 (353 to 652)	213 (178 to 255)	10 (5 to 19)	11 (9 to 13)	1021 (820 to 1271)	690 (501 to 925)	302 (253 to 362)	14 (7 to 27)	15 (12 to 19)
Dominica	2040	731 (581 to 911)	495 (364 to 667)	215 (179 to 258)	10 (5 to 19)	11 (9 to 14)	1037 (825 to 1294)	703 (517 to 947)	305 (255 to 366)	14 (7 to 28)	15 (13 to 19)

Dominica	2041	744 (592 to 939)	505 (365 to 680)	217 (181 to 260)	10 (5 to 20)	11 (9 to 14)	1056 (840 to 1333)	717 (518 to 965)	308 (257 to 370)	14 (7 to 28)	16 (13 to 20)
Dominica	2042	755 (596 to 949)	514 (368 to 696)	219 (183 to 263)	10 (5 to 20)	11 (9 to 14)	1071 (846 to 1348)	729 (522 to 987)	311 (260 to 374)	14 (7 to 29)	16 (13 to 21)
Dominica	2043	767 (607 to 980)	524 (374 to 718)	221 (185 to 266)	10 (5 to 21)	12 (9 to 15)	1089 (862 to 1391)	744 (531 to 1020)	314 (262 to 378)	15 (7 to 29)	17 (13 to 21)
Dominica	2044	781 (615 to 990)	535 (379 to 740)	224 (186 to 269)	11 (5 to 21)	12 (10 to 16)	1108 (873 to 1405)	759 (537 to 1050)	318 (265 to 381)	15 (7 to 30)	17 (14 to 23)
Dominica	2045	793 (614 to 1020)	544 (375 to 755)	226 (188 to 271)	11 (5 to 21)	12 (10 to 16)	1126 (871 to 1448)	773 (533 to 1072)	321 (267 to 385)	15 (7 to 30)	17 (14 to 22)
Dominica	2046	806 (630 to 1037)	554 (384 to 781)	228 (190 to 275)	11 (5 to 22)	13 (10 to 16)	1145 (894 to 1472)	787 (545 to 1108)	324 (270 to 390)	16 (8 to 31)	18 (14 to 23)
Dominica	2047	819 (631 to 1065)	564 (392 to 804)	231 (192 to 277)	11 (5 to 22)	13 (10 to 17)	1162 (896 to 1512)	801 (556 to 1141)	327 (273 to 394)	16 (8 to 31)	18 (14 to 24)
Dominica	2048	831 (644 to 1087)	574 (397 to 809)	233 (194 to 280)	11 (6 to 22)	13 (10 to 17)	1180 (914 to 1543)	815 (563 to 1148)	330 (275 to 398)	16 (8 to 32)	19 (15 to 25)
Dominica	2049	842 (643 to 1095)	583 (397 to 831)	235 (196 to 283)	11 (6 to 23)	13 (10 to 18)	1196 (912 to 1554)	828 (563 to 1180)	333 (278 to 401)	16 (8 to 32)	19 (15 to 25)
Dominica	2050	855 (657 to 1125)	593 (406 to 849)	237 (197 to 285)	12 (6 to 23)	13 (0 to 19)	1213 (933 to 1597)	841 (577 to 1205)	336 (280 to 405)	17 (8 to 33)	19 (0 to 27)

Dominican Republic	1995	162 (140 to 188)	33 (26 to 40)	110 (91 to 134)	19 (10 to 31)	1 (1 to 1)	386 (333 to 447)	78 (62 to 96)	261 (217 to 318)	44 (24 to 73)	3 (3 to 3)
Dominican Republic	1996	172 (151 to 196)	37 (30 to 46)	110 (93 to 130)	19 (11 to 31)	6 (6 to 6)	409 (360 to 466)	88 (71 to 108)	262 (221 to 308)	46 (26 to 74)	13 (13 to 13)
Dominican Republic	1997	183 (162 to 205)	46 (37 to 56)	115 (98 to 133)	19 (11 to 31)	3 (3 to 3)	435 (385 to 488)	110 (89 to 133)	273 (232 to 316)	46 (27 to 74)	6 (6 to 6)
Dominican Republic	1998	193 (172 to 215)	54 (44 to 64)	115 (98 to 132)	19 (11 to 30)	6 (6 to 6)	459 (408 to 511)	127 (105 to 153)	272 (233 to 314)	44 (26 to 71)	15 (15 to 15)
Dominican Republic	1999	201 (180 to 223)	61 (51 to 73)	114 (98 to 132)	18 (11 to 29)	8 (8 to 8)	479 (427 to 531)	145 (121 to 175)	272 (234 to 313)	43 (26 to 69)	18 (18 to 18)
Dominican Republic	2000	200 (179 to 222)	65 (54 to 77)	114 (99 to 131)	18 (11 to 27)	4 (4 to 4)	476 (427 to 528)	154 (129 to 183)	270 (235 to 311)	42 (25 to 65)	10 (10 to 10)
Dominican Republic	2001	204 (183 to 224)	67 (57 to 79)	114 (100 to 132)	18 (11 to 28)	4 (4 to 4)	484 (434 to 534)	160 (136 to 189)	272 (237 to 313)	42 (25 to 66)	10 (10 to 10)
Dominican Republic	2002	200 (179 to 220)	65 (55 to 77)	113 (98 to 130)	18 (11 to 27)	4 (4 to 4)	475 (426 to 524)	155 (131 to 183)	268 (233 to 308)	42 (25 to 65)	9 (9 to 9)
Dominican Republic	2003	201 (182 to 224)	65 (55 to 76)	114 (98 to 131)	19 (12 to 29)	4 (4 to 4)	479 (432 to 533)	154 (130 to 180)	270 (233 to 311)	45 (27 to 70)	10 (10 to 10)
Dominican Republic	2004	194 (174 to 216)	61 (52 to 71)	108 (93 to 125)	18 (11 to 28)	6 (6 to 6)	462 (415 to 513)	145 (124 to 170)	257 (221 to 297)	44 (27 to 68)	15 (15 to 15)
Dominican Republic	2005	197 (177 to 218)	64 (55 to 75)	105 (90 to 120)	19 (12 to 30)	8 (8 to 8)	468 (422 to 518)	153 (131 to 178)	249 (213 to 286)	46 (28 to 72)	20 (20 to 20)
Dominican Republic	2006	211 (189 to 236)	77 (66 to 89)	107 (92 to 124)	21 (13 to 33)	5 (5 to 5)	501 (449 to 561)	183 (158 to 212)	256 (219 to 294)	50 (30 to 79)	13 (13 to 13)
Dominican Republic	2007	230 (207 to 257)	91 (79 to 105)	110 (95 to 127)	23 (14 to 36)	6 (6 to 6)	548 (492 to 612)	217 (189 to 251)	262 (226 to 303)	55 (33 to 87)	13 (13 to 13)
Dominican Republic	2008	263 (237 to 292)	113 (98 to 130)	118 (102 to 137)	26 (15 to 40)	6 (6 to 6)	625 (563 to 695)	269 (234 to 310)	281 (242 to 325)	61 (37 to 94)	14 (14 to 14)
Dominican Republic	2009	280 (254 to 307)	120 (105 to 137)	121 (105 to 140)	25 (15 to 39)	13 (13 to 13)	665 (605 to 730)	285 (250 to 327)	288 (250 to 333)	60 (36 to 93)	31 (31 to 31)

Dominican Republic	2010	305 (279 to 335)	135 (120 to 152)	131 (115 to 151)	27 (16 to 42)	12 (12 to 12)	726 (665 to 796)	321 (284 to 363)	313 (274 to 360)	63 (38 to 99)	29 (29 to 29)
Dominican Republic	2011	316 (290 to 348)	145 (128 to 164)	138 (120 to 158)	27 (16 to 43)	5 (5 to 5)	751 (689 to 827)	346 (305 to 390)	328 (285 to 375)	65 (39 to 101)	13 (13 to 13)
Dominican Republic	2012	364 (335 to 400)	172 (153 to 194)	152 (132 to 175)	32 (19 to 49)	8 (8 to 8)	865 (796 to 951)	408 (363 to 460)	362 (315 to 415)	77 (46 to 117)	19 (19 to 19)
Dominican Republic	2013	356 (325 to 390)	165 (146 to 185)	152 (133 to 174)	31 (19 to 47)	8 (8 to 8)	846 (774 to 927)	391 (348 to 439)	362 (316 to 413)	74 (45 to 112)	18 (18 to 18)
Dominican Republic	2014	371 (340 to 405)	168 (148 to 188)	161 (140 to 183)	32 (20 to 50)	10 (10 to 10)	883 (809 to 964)	399 (353 to 446)	382 (333 to 436)	77 (47 to 119)	24 (24 to 24)
Dominican Republic	2015	408 (374 to 445)	175 (154 to 196)	171 (149 to 196)	34 (21 to 53)	28 (28 to 28)	971 (890 to 1059)	416 (366 to 466)	407 (355 to 467)	82 (50 to 126)	66 (66 to 66)
Dominican Republic	2016	414 (376 to 455)	188 (166 to 213)	183 (159 to 211)	37 (22 to 57)	6 (6 to 6)	985 (895 to 1081)	447 (394 to 507)	436 (378 to 502)	88 (52 to 136)	15 (15 to 15)
Dominican Republic	2017	436 (383 to 493)	200 (173 to 231)	193 (164 to 227)	39 (22 to 60)	5 (5 to 5)	1037 (911 to 1174)	474 (412 to 549)	459 (391 to 541)	92 (53 to 142)	12 (12 to 12)
Dominican Republic	2018	454 (397 to 514)	206 (177 to 238)	204 (174 to 240)	40 (23 to 62)	5 (5 to 5)	1081 (943 to 1223)	489 (420 to 566)	485 (413 to 571)	95 (55 to 148)	11 (11 to 11)
Dominican Republic	2019	483 (419 to 550)	225 (191 to 264)	212 (180 to 249)	42 (24 to 65)	5 (5 to 5)	1149 (997 to 1308)	535 (454 to 627)	503 (428 to 592)	100 (57 to 155)	11 (11 to 11)
Dominican Republic	2020	506 (437 to 579)	238 (198 to 281)	220 (187 to 259)	44 (25 to 68)	5 (4 to 5)	1204 (1040 to 1377)	566 (471 to 667)	523 (445 to 616)	104 (60 to 162)	11 (10 to 12)
Dominican Republic	2021	523 (450 to 602)	248 (206 to 296)	226 (192 to 266)	45 (26 to 70)	5 (4 to 5)	1245 (1070 to 1432)	590 (489 to 704)	536 (457 to 633)	108 (62 to 167)	11 (10 to 12)

Dominican Republic	2022	543 (464 to 624)	260 (213 to 309)	231 (197 to 273)	47 (27 to 73)	5 (4 to 5)	1291 (1103 to 1484)	617 (506 to 735)	550 (468 to 648)	112 (64 to 174)	11 (10 to 12)
Dominican Republic	2023	564 (484 to 648)	273 (223 to 325)	237 (202 to 279)	49 (28 to 76)	5 (4 to 5)	1340 (1151 to 1541)	649 (531 to 773)	563 (479 to 664)	117 (67 to 182)	11 (10 to 13)
Dominican Republic	2024	585 (503 to 677)	287 (235 to 341)	243 (206 to 286)	51 (29 to 79)	5 (4 to 5)	1390 (1196 to 1609)	682 (559 to 811)	577 (491 to 681)	120 (69 to 187)	11 (10 to 13)
Dominican Republic	2025	606 (519 to 700)	300 (247 to 357)	248 (211 to 293)	52 (30 to 81)	5 (4 to 5)	1440 (1235 to 1665)	714 (587 to 849)	591 (503 to 697)	124 (71 to 192)	11 (10 to 13)
Dominican Republic	2026	626 (536 to 723)	313 (258 to 372)	254 (216 to 300)	54 (31 to 83)	5 (4 to 6)	1489 (1276 to 1719)	745 (614 to 885)	604 (514 to 714)	127 (73 to 197)	12 (10 to 13)
Dominican Republic	2027	647 (554 to 751)	327 (269 to 388)	260 (221 to 307)	55 (32 to 85)	5 (4 to 6)	1539 (1318 to 1785)	778 (639 to 923)	618 (525 to 730)	131 (75 to 203)	12 (10 to 14)
Dominican Republic	2028	669 (570 to 775)	341 (280 to 404)	266 (225 to 314)	57 (32 to 88)	5 (4 to 6)	1591 (1357 to 1842)	812 (666 to 962)	632 (536 to 746)	135 (77 to 209)	12 (10 to 14)
Dominican Republic	2029	691 (592 to 798)	356 (292 to 422)	272 (230 to 320)	58 (33 to 91)	5 (4 to 6)	1643 (1408 to 1897)	847 (695 to 1004)	646 (547 to 762)	138 (79 to 215)	12 (10 to 14)
Dominican Republic	2030	714 (610 to 828)	372 (305 to 441)	277 (235 to 328)	60 (34 to 93)	5 (4 to 6)	1698 (1450 to 1969)	885 (724 to 1048)	660 (559 to 780)	142 (82 to 222)	12 (10 to 14)
Dominican Republic	2031	738 (630 to 855)	388 (317 to 460)	283 (240 to 335)	61 (35 to 96)	5 (4 to 6)	1754 (1499 to 2033)	922 (754 to 1093)	674 (570 to 798)	146 (84 to 228)	12 (10 to 14)

Dominican Republic	2032	763 (649 to 888)	405 (330 to 480)	290 (245 to 343)	63 (36 to 99)	5 (4 to 6)	1814 (1543 to 2111)	962 (785 to 1142)	689 (582 to 817)	150 (86 to 235)	12 (10 to 15)
Dominican Republic	2033	789 (668 to 918)	422 (343 to 501)	297 (250 to 352)	65 (37 to 102)	5 (4 to 6)	1876 (1588 to 2183)	1003 (815 to 1191)	706 (595 to 838)	154 (88 to 242)	12 (11 to 15)
Dominican Republic	2034	816 (694 to 952)	440 (357 to 524)	304 (256 to 361)	67 (38 to 105)	5 (4 to 7)	1940 (1651 to 2265)	1046 (848 to 1246)	723 (609 to 859)	159 (90 to 249)	13 (10 to 16)
Dominican Republic	2035	844 (720 to 984)	458 (371 to 547)	312 (263 to 371)	69 (39 to 108)	5 (4 to 7)	2008 (1712 to 2339)	1090 (883 to 1300)	742 (624 to 882)	164 (92 to 257)	13 (10 to 16)
Dominican Republic	2036	874 (741 to 1015)	478 (386 to 570)	320 (269 to 380)	71 (40 to 111)	5 (0 to 7)	2078 (1762 to 2414)	1136 (918 to 1356)	761 (640 to 904)	169 (95 to 265)	12 (0 to 16)
Dominican Republic	2037	904 (764 to 1053)	498 (401 to 595)	328 (276 to 390)	73 (41 to 115)	5 (0 to 7)	2150 (1816 to 2504)	1183 (954 to 1415)	780 (657 to 928)	174 (97 to 273)	12 (0 to 16)
Dominican Republic	2038	935 (789 to 1091)	518 (417 to 620)	337 (283 to 401)	76 (42 to 118)	5 (0 to 7)	2223 (1878 to 2595)	1231 (992 to 1475)	801 (673 to 954)	180 (100 to 282)	12 (0 to 17)
Dominican Republic	2039	965 (815 to 1126)	538 (433 to 645)	345 (290 to 412)	78 (43 to 122)	5 (0 to 7)	2296 (1938 to 2677)	1279 (1029 to 1535)	820 (689 to 979)	185 (103 to 290)	11 (0 to 16)
Dominican Republic	2040	996 (838 to 1172)	558 (449 to 673)	353 (297 to 422)	80 (45 to 126)	4 (0 to 7)	2369 (1994 to 2787)	1328 (1068 to 1600)	840 (705 to 1004)	191 (106 to 299)	11 (0 to 17)
Dominican Republic	2041	1027 (862 to 1209)	579 (465 to 700)	362 (303 to 433)	82 (46 to 129)	4 (0 to 7)	2443 (2051 to 2875)	1377 (1106 to 1665)	860 (722 to 1029)	196 (109 to 308)	10 (0 to 17)

Dominican Republic	2042	1058 (888 to 1247)	599 (481 to 729)	370 (310 to 443)	85 (47 to 133)	4 (0 to 7)	2517 (2111 to 2967)	1426 (1144 to 1734)	881 (738 to 1054)	202 (112 to 316)	9 (0 to 17)
Dominican Republic	2043	1090 (911 to 1287)	620 (497 to 756)	379 (317 to 454)	87 (49 to 137)	4 (0 to 7)	2591 (2166 to 3060)	1475 (1182 to 1798)	901 (753 to 1079)	207 (116 to 325)	8 (0 to 17)
Dominican Republic	2044	1120 (937 to 1326)	641 (513 to 783)	387 (324 to 464)	90 (50 to 141)	3 (0 to 7)	2665 (2229 to 3154)	1523 (1219 to 1863)	920 (769 to 1103)	213 (119 to 334)	8 (0 to 18)
Dominican Republic	2045	1152 (962 to 1364)	661 (529 to 813)	396 (330 to 474)	92 (51 to 144)	3 (0 to 8)	2740 (2287 to 3244)	1573 (1257 to 1933)	941 (786 to 1128)	219 (122 to 344)	7 (0 to 18)
Dominican Republic	2046	1183 (990 to 1407)	682 (544 to 841)	404 (337 to 485)	95 (52 to 148)	3 (0 to 7)	2814 (2353 to 3346)	1622 (1294 to 2001)	961 (802 to 1153)	225 (125 to 353)	7 (0 to 18)
Dominican Republic	2047	1214 (1013 to 1443)	702 (559 to 869)	413 (343 to 495)	97 (54 to 152)	2 (0 to 8)	2888 (2410 to 3432)	1670 (1330 to 2068)	981 (816 to 1178)	231 (128 to 362)	6 (0 to 18)
Dominican Republic	2048	1246 (1041 to 1488)	723 (574 to 899)	421 (350 to 506)	100 (55 to 157)	2 (0 to 8)	2962 (2475 to 3539)	1718 (1366 to 2138)	1002 (832 to 1203)	237 (131 to 373)	5 (0 to 19)
Dominican Republic	2049	1277 (1068 to 1530)	743 (589 to 925)	430 (356 to 517)	102 (56 to 161)	2 (0 to 8)	3037 (2539 to 3638)	1767 (1402 to 2201)	1022 (847 to 1228)	243 (134 to 383)	5 (0 to 19)
Dominican Republic	2050	1309 (1089 to 1568)	764 (605 to 956)	438 (362 to 527)	105 (58 to 166)	2 (0 to 8)	3113 (2589 to 3729)	1817 (1440 to 2274)	1043 (862 to 1254)	249 (138 to 394)	4 (0 to 19)
Ecuador	1995	203 (178 to 229)	112 (94 to 130)	69 (55 to 84)	20 (11 to 33)	2 (2 to 2)	386 (339 to 436)	213 (178 to 248)	130 (104 to 160)	38 (21 to 62)	4 (4 to 4)
Ecuador	1996	209 (186 to 233)	113 (96 to 130)	74 (60 to 89)	20 (11 to 33)	3 (3 to 3)	397 (353 to 443)	214 (183 to 248)	140 (114 to 170)	38 (22 to 63)	5 (5 to 5)

Ecuador	1997	205 (184 to 227)	103 (88 to 118)	81 (67 to 97)	19 (11 to 31)	2 (2 to 2)	390 (349 to 432)	195 (168 to 224)	154 (127 to 184)	36 (21 to 58)	4 (4 to 4)
Ecuador	1998	200 (179 to 222)	88 (76 to 101)	90 (76 to 107)	17 (10 to 28)	4 (4 to 4)	379 (341 to 423)	167 (144 to 191)	172 (144 to 204)	33 (19 to 54)	8 (8 to 8)
Ecuador	1999	195 (174 to 217)	78 (67 to 89)	100 (85 to 117)	15 (9 to 24)	2 (2 to 2)	370 (331 to 413)	148 (127 to 169)	190 (162 to 223)	28 (17 to 46)	4 (4 to 4)
Ecuador	2000	186 (167 to 207)	67 (58 to 77)	106 (91 to 121)	11 (7 to 18)	2 (2 to 2)	354 (318 to 394)	128 (110 to 147)	201 (173 to 230)	21 (13 to 35)	4 (4 to 4)
Ecuador	2001	191 (173 to 210)	64 (55 to 74)	115 (100 to 131)	10 (6 to 17)	2 (2 to 2)	363 (329 to 399)	122 (105 to 140)	218 (189 to 249)	19 (11 to 31)	4 (4 to 4)
Ecuador	2002	220 (201 to 243)	72 (62 to 83)	136 (118 to 154)	12 (7 to 20)	1 (1 to 1)	419 (381 to 462)	137 (118 to 157)	258 (225 to 293)	23 (13 to 38)	2 (2 to 2)
Ecuador	2003	244 (223 to 268)	75 (65 to 86)	152 (134 to 172)	14 (8 to 23)	2 (2 to 2)	463 (424 to 509)	143 (124 to 164)	289 (256 to 327)	27 (16 to 44)	4 (4 to 4)
Ecuador	2004	270 (246 to 297)	83 (72 to 95)	167 (148 to 188)	17 (10 to 27)	3 (3 to 3)	512 (468 to 564)	158 (138 to 181)	318 (282 to 358)	32 (19 to 52)	5 (5 to 5)
Ecuador	2005	291 (268 to 320)	92 (80 to 105)	178 (158 to 202)	19 (11 to 30)	3 (3 to 3)	554 (509 to 609)	175 (152 to 200)	338 (300 to 384)	36 (21 to 57)	6 (6 to 6)
Ecuador	2006	299 (274 to 327)	98 (86 to 112)	178 (158 to 199)	20 (12 to 31)	4 (4 to 4)	569 (520 to 621)	187 (164 to 212)	338 (300 to 379)	37 (22 to 59)	7 (7 to 7)
Ecuador	2007	315 (289 to 342)	112 (99 to 125)	176 (156 to 198)	22 (13 to 35)	4 (4 to 4)	599 (549 to 650)	213 (189 to 239)	335 (296 to 376)	42 (25 to 67)	9 (9 to 9)
Ecuador	2008	377 (348 to 408)	151 (134 to 168)	193 (171 to 217)	29 (17 to 45)	4 (4 to 4)	716 (661 to 775)	287 (254 to 319)	367 (326 to 412)	55 (32 to 85)	8 (8 to 8)
Ecuador	2009	374 (347 to 404)	155 (137 to 171)	188 (168 to 210)	27 (17 to 43)	3 (3 to 3)	710 (659 to 768)	294 (261 to 326)	358 (320 to 399)	52 (32 to 81)	6 (6 to 6)
Ecuador	2010	409 (380 to 440)	180 (161 to 197)	197 (176 to 221)	29 (19 to 45)	3 (3 to 3)	778 (722 to 837)	341 (307 to 374)	375 (335 to 420)	56 (35 to 86)	6 (6 to 6)
Ecuador	2011	475 (441 to 510)	222 (201 to 243)	216 (194 to 240)	34 (22 to 51)	3 (3 to 3)	902 (838 to 970)	422 (383 to 462)	410 (369 to 457)	64 (41 to 98)	6 (6 to 6)

Ecuador	2012	510 (476 to 545)	248 (226 to 270)	225 (202 to 250)	35 (23 to 54)	2 (2 to 2)	969 (905 to 1037)	471 (429 to 514)	427 (384 to 475)	67 (43 to 102)	4 (4 to 4)
Ecuador	2013	555 (517 to 593)	280 (256 to 305)	235 (212 to 261)	38 (24 to 57)	2 (2 to 2)	1055 (983 to 1127)	532 (486 to 579)	448 (403 to 497)	72 (46 to 109)	4 (4 to 4)
Ecuador	2014	576 (534 to 617)	294 (268 to 320)	240 (215 to 266)	40 (25 to 60)	3 (3 to 3)	1096 (1015 to 1173)	558 (509 to 609)	456 (409 to 506)	75 (48 to 113)	6 (6 to 6)
Ecuador	2015	550 (507 to 593)	279 (253 to 306)	231 (206 to 259)	39 (24 to 60)	1 (1 to 1)	1046 (964 to 1127)	530 (481 to 582)	440 (392 to 493)	74 (46 to 115)	2 (2 to 2)
Ecuador	2016	533 (486 to 578)	272 (244 to 302)	219 (192 to 249)	40 (24 to 63)	1 (1 to 1)	1012 (924 to 1099)	518 (464 to 574)	417 (365 to 472)	76 (46 to 119)	2 (2 to 2)
Ecuador	2017	524 (464 to 591)	271 (238 to 307)	213 (182 to 246)	40 (23 to 62)	1 (1 to 1)	996 (881 to 1124)	515 (453 to 583)	405 (345 to 468)	75 (44 to 119)	2 (2 to 2)
Ecuador	2018	527 (469 to 594)	275 (242 to 311)	211 (180 to 244)	40 (23 to 63)	1 (1 to 1)	1001 (892 to 1129)	523 (460 to 592)	401 (342 to 464)	76 (45 to 120)	2 (2 to 2)
Ecuador	2019	502 (444 to 566)	257 (225 to 291)	207 (176 to 239)	38 (22 to 60)	1 (1 to 1)	955 (843 to 1076)	488 (427 to 554)	393 (335 to 454)	73 (42 to 115)	2 (2 to 2)
Ecuador	2020	502 (446 to 566)	258 (225 to 294)	205 (175 to 237)	38 (22 to 61)	1 (1 to 1)	955 (848 to 1076)	491 (428 to 558)	390 (333 to 451)	73 (43 to 115)	2 (2 to 2)
Ecuador	2021	509 (450 to 577)	263 (230 to 301)	206 (176 to 239)	39 (23 to 61)	1 (1 to 1)	968 (855 to 1096)	500 (437 to 573)	393 (335 to 454)	74 (43 to 117)	2 (2 to 2)
Ecuador	2022	516 (459 to 583)	268 (234 to 308)	208 (177 to 241)	40 (23 to 62)	1 (1 to 1)	981 (873 to 1108)	509 (446 to 585)	395 (337 to 458)	75 (44 to 119)	2 (1 to 2)
Ecuador	2023	522 (460 to 596)	272 (237 to 315)	209 (178 to 242)	40 (24 to 63)	1 (1 to 1)	993 (874 to 1133)	518 (451 to 599)	397 (339 to 459)	76 (45 to 120)	2 (2 to 2)

Ecuador	2024	528 (468 to 600)	277 (242 to 318)	210 (179 to 243)	41 (24 to 64)	1 (1 to 1)	1004 (889 to 1140)	526 (460 to 605)	399 (339 to 462)	77 (45 to 122)	2 (2 to 2)
Ecuador	2025	534 (470 to 607)	281 (245 to 324)	211 (179 to 245)	41 (24 to 65)	1 (1 to 1)	1015 (894 to 1155)	534 (465 to 616)	401 (341 to 466)	78 (46 to 123)	2 (2 to 2)
Ecuador	2026	539 (476 to 612)	285 (248 to 328)	212 (180 to 246)	42 (24 to 66)	1 (1 to 1)	1026 (905 to 1164)	543 (472 to 623)	402 (343 to 468)	79 (47 to 125)	2 (2 to 2)
Ecuador	2027	546 (478 to 621)	290 (252 to 334)	213 (181 to 247)	42 (25 to 67)	1 (1 to 1)	1037 (910 to 1181)	551 (479 to 635)	404 (344 to 470)	80 (47 to 127)	2 (2 to 2)
Ecuador	2028	552 (487 to 629)	295 (254 to 341)	213 (182 to 248)	43 (25 to 68)	1 (1 to 1)	1049 (926 to 1196)	561 (483 to 648)	406 (346 to 472)	81 (48 to 129)	2 (2 to 2)
Ecuador	2029	558 (490 to 638)	300 (258 to 348)	214 (183 to 250)	43 (25 to 69)	1 (1 to 1)	1061 (932 to 1213)	570 (491 to 662)	408 (348 to 475)	82 (48 to 131)	2 (2 to 2)
Ecuador	2030	565 (496 to 646)	305 (261 to 355)	215 (184 to 251)	44 (26 to 70)	1 (1 to 1)	1074 (943 to 1229)	579 (497 to 674)	410 (349 to 477)	83 (49 to 132)	2 (2 to 2)
Ecuador	2031	571 (502 to 655)	309 (264 to 361)	216 (184 to 252)	44 (26 to 71)	1 (1 to 1)	1086 (954 to 1246)	588 (502 to 686)	411 (351 to 480)	84 (50 to 134)	2 (2 to 2)
Ecuador	2032	578 (507 to 662)	314 (268 to 367)	217 (185 to 254)	45 (27 to 72)	1 (1 to 1)	1098 (963 to 1259)	598 (510 to 697)	413 (352 to 483)	85 (50 to 136)	2 (2 to 2)
Ecuador	2033	586 (510 to 673)	320 (273 to 372)	219 (186 to 256)	46 (27 to 73)	1 (1 to 1)	1113 (970 to 1279)	609 (518 to 707)	416 (354 to 487)	87 (51 to 138)	2 (2 to 2)

Ecuador	2034	594 (520 to 684)	326 (278 to 383)	220 (188 to 259)	46 (27 to 74)	1 (1 to 1)	1130 (988 to 1300)	621 (528 to 728)	419 (357 to 491)	88 (52 to 141)	2 (2 to 2)
Ecuador	2035	603 (522 to 694)	332 (282 to 390)	222 (189 to 261)	47 (28 to 75)	1 (1 to 1)	1146 (992 to 1320)	632 (536 to 742)	422 (359 to 495)	90 (53 to 143)	2 (2 to 2)
Ecuador	2036	612 (533 to 704)	339 (287 to 398)	224 (190 to 263)	48 (28 to 77)	1 (1 to 1)	1163 (1014 to 1338)	644 (545 to 757)	426 (361 to 499)	91 (54 to 146)	2 (2 to 3)
Ecuador	2037	620 (537 to 719)	345 (291 to 405)	226 (191 to 265)	49 (29 to 78)	1 (1 to 1)	1179 (1020 to 1368)	656 (553 to 770)	429 (364 to 504)	92 (55 to 149)	2 (2 to 3)
Ecuador	2038	629 (547 to 725)	351 (296 to 414)	227 (193 to 267)	49 (29 to 79)	1 (1 to 1)	1196 (1040 to 1379)	668 (562 to 787)	432 (366 to 508)	94 (56 to 151)	2 (2 to 3)
Ecuador	2039	638 (550 to 743)	358 (301 to 422)	229 (194 to 270)	50 (30 to 81)	1 (1 to 1)	1213 (1046 to 1413)	680 (573 to 802)	435 (369 to 512)	95 (57 to 154)	2 (2 to 3)
Ecuador	2040	647 (560 to 750)	365 (306 to 431)	230 (195 to 272)	51 (30 to 82)	1 (1 to 1)	1230 (1065 to 1425)	693 (581 to 819)	438 (371 to 517)	97 (57 to 156)	2 (2 to 3)
Ecuador	2041	656 (563 to 764)	371 (311 to 439)	232 (197 to 274)	52 (31 to 84)	1 (1 to 1)	1248 (1071 to 1452)	706 (590 to 834)	441 (374 to 521)	98 (58 to 159)	2 (2 to 3)
Ecuador	2042	665 (570 to 772)	378 (316 to 445)	234 (198 to 276)	53 (31 to 85)	1 (1 to 1)	1265 (1084 to 1467)	719 (601 to 845)	444 (376 to 525)	100 (59 to 161)	2 (2 to 3)
Ecuador	2043	674 (576 to 785)	384 (318 to 457)	235 (199 to 278)	53 (31 to 86)	1 (1 to 2)	1282 (1096 to 1493)	731 (605 to 868)	447 (379 to 529)	101 (60 to 163)	2 (2 to 3)

Ecuador	2044	683 (583 to 796)	391 (325 to 465)	237 (201 to 280)	54 (32 to 87)	1 (1 to 2)	1298 (1108 to 1514)	743 (619 to 885)	450 (381 to 532)	103 (60 to 165)	2 (2 to 3)
Ecuador	2045	692 (594 to 808)	398 (328 to 473)	238 (202 to 282)	55 (32 to 88)	1 (1 to 2)	1315 (1129 to 1536)	756 (624 to 899)	453 (383 to 536)	104 (61 to 167)	2 (2 to 3)
Ecuador	2046	701 (596 to 822)	404 (334 to 481)	240 (203 to 284)	56 (33 to 89)	1 (1 to 2)	1332 (1132 to 1563)	769 (636 to 914)	455 (386 to 540)	106 (62 to 170)	2 (2 to 3)
Ecuador	2047	710 (605 to 831)	411 (340 to 491)	241 (204 to 286)	56 (33 to 91)	1 (1 to 2)	1350 (1151 to 1580)	782 (646 to 933)	458 (388 to 544)	107 (63 to 173)	2 (2 to 3)
Ecuador	2048	719 (609 to 845)	418 (345 to 501)	242 (205 to 288)	57 (34 to 92)	1 (1 to 2)	1367 (1158 to 1606)	795 (656 to 952)	461 (390 to 547)	109 (64 to 176)	2 (2 to 3)
Ecuador	2049	728 (618 to 864)	425 (350 to 512)	244 (206 to 290)	58 (34 to 94)	1 (1 to 2)	1384 (1175 to 1642)	808 (666 to 974)	463 (392 to 551)	110 (65 to 178)	3 (2 to 3)
Ecuador	2050	737 (626 to 870)	431 (356 to 521)	245 (207 to 292)	59 (34 to 95)	1 (1 to 2)	1400 (1189 to 1654)	820 (677 to 991)	466 (393 to 555)	112 (66 to 180)	3 (2 to 3)
Egypt	1995	78 (65 to 93)	27 (21 to 35)	47 (36 to 60)	2 (1 to 3)	2 (2 to 2)	356 (296 to 427)	125 (96 to 162)	213 (163 to 277)	7 (3 to 14)	10 (10 to 10)
Egypt	1996	82 (69 to 98)	28 (22 to 36)	50 (39 to 64)	2 (1 to 3)	2 (2 to 2)	375 (314 to 448)	130 (101 to 167)	229 (178 to 293)	8 (4 to 15)	9 (9 to 9)
Egypt	1997	87 (73 to 103)	30 (23 to 38)	54 (43 to 69)	2 (1 to 3)	1 (1 to 1)	400 (336 to 471)	138 (107 to 175)	249 (196 to 314)	8 (4 to 15)	6 (6 to 6)
Egypt	1998	94 (78 to 109)	32 (24 to 40)	58 (46 to 72)	2 (1 to 3)	2 (2 to 2)	428 (359 to 498)	145 (112 to 184)	266 (209 to 329)	9 (4 to 16)	9 (9 to 9)
Egypt	1999	97 (82 to 113)	33 (26 to 42)	61 (48 to 75)	2 (1 to 4)	1 (1 to 1)	443 (373 to 515)	151 (117 to 191)	277 (219 to 343)	9 (4 to 16)	6 (6 to 6)

Egypt	2000	99 (83 to 114)	33 (26 to 42)	62 (49 to 76)	2 (1 to 4)	1 (1 to 1)	453 (382 to 524)	153 (119 to 191)	284 (226 to 348)	10 (5 to 17)	7 (7 to 7)
Egypt	2001	105 (89 to 121)	35 (27 to 44)	66 (53 to 81)	2 (1 to 4)	1 (1 to 1)	479 (405 to 554)	162 (125 to 202)	301 (242 to 370)	10 (5 to 18)	6 (6 to 6)
Egypt	2002	109 (93 to 127)	37 (29 to 46)	69 (56 to 85)	2 (1 to 4)	1 (1 to 1)	501 (424 to 582)	170 (131 to 211)	316 (255 to 387)	10 (5 to 19)	5 (5 to 5)
Egypt	2003	107 (91 to 125)	36 (28 to 45)	68 (55 to 83)	2 (1 to 4)	1 (1 to 1)	489 (415 to 571)	163 (127 to 204)	313 (252 to 382)	10 (5 to 18)	4 (4 to 4)
Egypt	2004	104 (89 to 122)	34 (27 to 43)	66 (53 to 81)	2 (1 to 4)	1 (1 to 1)	476 (406 to 556)	157 (122 to 197)	303 (241 to 370)	10 (5 to 19)	6 (6 to 6)
Egypt	2005	103 (87 to 120)	34 (27 to 42)	65 (52 to 80)	2 (1 to 4)	2 (2 to 2)	472 (400 to 549)	156 (122 to 194)	299 (239 to 366)	10 (5 to 19)	7 (7 to 7)
Egypt	2006	109 (93 to 127)	38 (29 to 47)	67 (54 to 81)	3 (1 to 5)	2 (2 to 2)	501 (427 to 580)	173 (135 to 217)	307 (246 to 373)	12 (6 to 22)	10 (10 to 10)
Egypt	2007	106 (90 to 123)	36 (29 to 46)	66 (53 to 80)	3 (1 to 5)	1 (1 to 1)	486 (414 to 563)	166 (131 to 209)	300 (242 to 364)	12 (6 to 23)	7 (7 to 7)
Egypt	2008	112 (96 to 129)	38 (30 to 48)	68 (55 to 82)	3 (2 to 6)	2 (2 to 2)	511 (437 to 591)	175 (136 to 221)	312 (252 to 378)	15 (7 to 27)	10 (10 to 10)
Egypt	2009	110 (93 to 127)	38 (29 to 47)	68 (55 to 82)	4 (2 to 7)	1 (1 to 1)	504 (428 to 582)	173 (134 to 217)	310 (254 to 377)	17 (8 to 31)	3 (3 to 3)
Egypt	2010	112 (95 to 130)	38 (29 to 47)	69 (55 to 83)	5 (2 to 8)	1 (1 to 1)	510 (433 to 593)	172 (133 to 214)	314 (254 to 381)	21 (10 to 38)	4 (4 to 4)
Egypt	2011	114 (97 to 132)	38 (29 to 47)	70 (56 to 85)	6 (3 to 10)	1 (1 to 1)	522 (442 to 605)	172 (135 to 215)	320 (258 to 391)	26 (13 to 47)	3 (3 to 3)
Egypt	2012	116 (99 to 136)	37 (29 to 47)	72 (57 to 88)	7 (4 to 12)	0 (0 to 0)	533 (451 to 622)	170 (132 to 214)	328 (263 to 402)	32 (16 to 56)	2 (2 to 2)
Egypt	2013	127 (108 to 148)	41 (32 to 51)	77 (62 to 94)	9 (5 to 16)	0 (0 to 0)	581 (496 to 678)	187 (144 to 234)	352 (283 to 429)	41 (21 to 72)	2 (2 to 2)
Egypt	2014	134 (114 to 155)	43 (34 to 54)	80 (64 to 97)	10 (5 to 18)	1 (1 to 1)	612 (523 to 711)	197 (154 to 249)	366 (295 to 443)	46 (24 to 80)	2 (2 to 2)

Egypt	2015	137 (116 to 160)	44 (34 to 55)	82 (66 to 100)	11 (5 to 19)	1 (1 to 1)	628 (532 to 731)	199 (155 to 251)	377 (303 to 460)	49 (25 to 88)	2 (2 to 2)
Egypt	2016	144 (121 to 169)	46 (35 to 58)	86 (67 to 105)	12 (6 to 21)	1 (1 to 1)	658 (556 to 775)	209 (162 to 265)	393 (308 to 483)	53 (26 to 97)	3 (3 to 3)
Egypt	2017	147 (121 to 178)	47 (36 to 60)	88 (68 to 110)	11 (6 to 20)	1 (1 to 1)	675 (552 to 815)	216 (166 to 276)	403 (313 to 504)	52 (26 to 93)	4 (4 to 4)
Egypt	2018	149 (121 to 180)	45 (35 to 58)	91 (70 to 114)	12 (6 to 21)	1 (1 to 1)	681 (553 to 822)	208 (159 to 266)	415 (323 to 520)	54 (27 to 97)	4 (4 to 4)
Egypt	2019	150 (122 to 181)	44 (33 to 56)	93 (72 to 117)	12 (6 to 22)	1 (1 to 1)	687 (558 to 830)	200 (152 to 256)	427 (332 to 534)	56 (28 to 101)	4 (4 to 4)
Egypt	2020	155 (125 to 188)	45 (34 to 58)	96 (75 to 121)	13 (6 to 23)	1 (1 to 1)	709 (574 to 860)	206 (157 to 263)	441 (341 to 552)	58 (29 to 106)	4 (4 to 4)
Egypt	2021	157 (128 to 190)	46 (35 to 58)	98 (76 to 123)	13 (6 to 24)	1 (1 to 1)	719 (586 to 868)	208 (160 to 265)	447 (347 to 561)	60 (30 to 109)	4 (4 to 4)
Egypt	2022	160 (130 to 193)	46 (36 to 59)	99 (77 to 124)	13 (7 to 24)	1 (1 to 1)	731 (595 to 881)	212 (163 to 271)	454 (351 to 568)	61 (30 to 111)	4 (4 to 4)
Egypt	2023	163 (132 to 198)	47 (36 to 60)	101 (78 to 126)	14 (7 to 25)	1 (1 to 1)	745 (606 to 908)	217 (167 to 276)	461 (357 to 578)	62 (31 to 114)	4 (4 to 5)
Egypt	2024	166 (136 to 202)	49 (37 to 62)	103 (79 to 128)	14 (7 to 25)	1 (1 to 1)	760 (621 to 927)	223 (169 to 286)	470 (363 to 588)	64 (32 to 117)	4 (4 to 5)
Egypt	2025	169 (137 to 207)	50 (38 to 64)	104 (81 to 131)	14 (7 to 26)	1 (1 to 1)	775 (626 to 948)	228 (173 to 292)	478 (369 to 598)	65 (32 to 119)	4 (4 to 5)
Egypt	2026	173 (141 to 210)	51 (39 to 65)	106 (82 to 133)	15 (7 to 27)	1 (1 to 1)	792 (644 to 961)	234 (178 to 299)	486 (375 to 611)	67 (33 to 123)	4 (4 to 5)
Egypt	2027	177 (143 to 216)	52 (40 to 67)	108 (84 to 136)	15 (7 to 27)	1 (1 to 1)	808 (653 to 987)	240 (181 to 309)	496 (383 to 622)	68 (34 to 126)	4 (4 to 5)
Egypt	2028	180 (146 to 219)	54 (41 to 69)	110 (85 to 139)	15 (8 to 28)	1 (1 to 1)	825 (667 to 1003)	245 (186 to 316)	505 (390 to 634)	70 (35 to 129)	4 (4 to 5)
Egypt	2029	184 (148 to 224)	55 (42 to 71)	113 (87 to 141)	16 (8 to 29)	1 (1 to 1)	844 (680 to 1027)	252 (190 to 324)	516 (398 to 647)	72 (36 to 132)	4 (4 to 5)

Egypt	2030	189 (153 to 228)	56 (43 to 73)	115 (89 to 145)	16 (8 to 30)	1 (1 to 1)	863 (702 to 1044)	258 (195 to 332)	527 (407 to 661)	74 (37 to 136)	4 (4 to 5)
Egypt	2031	193 (156 to 235)	58 (44 to 75)	118 (91 to 148)	17 (8 to 30)	1 (1 to 1)	883 (712 to 1077)	265 (200 to 342)	539 (416 to 676)	76 (37 to 139)	4 (4 to 5)
Egypt	2032	198 (159 to 239)	60 (45 to 77)	120 (93 to 151)	17 (8 to 31)	1 (1 to 1)	905 (729 to 1096)	273 (208 to 351)	550 (425 to 691)	78 (39 to 143)	4 (4 to 5)
Egypt	2033	203 (164 to 248)	61 (46 to 79)	123 (95 to 154)	17 (9 to 32)	1 (1 to 1)	928 (749 to 1136)	281 (212 to 362)	563 (436 to 707)	80 (40 to 147)	4 (4 to 5)
Egypt	2034	208 (167 to 251)	63 (48 to 81)	126 (97 to 158)	18 (9 to 33)	1 (1 to 1)	952 (766 to 1151)	290 (220 to 372)	576 (446 to 722)	82 (41 to 151)	4 (3 to 5)
Egypt	2035	213 (171 to 263)	65 (49 to 84)	129 (99 to 161)	19 (9 to 34)	1 (1 to 1)	977 (781 to 1204)	299 (226 to 385)	589 (455 to 739)	85 (42 to 156)	4 (4 to 5)
Egypt	2036	218 (176 to 264)	67 (50 to 86)	131 (101 to 165)	19 (9 to 35)	1 (1 to 1)	999 (808 to 1209)	307 (231 to 393)	601 (464 to 755)	87 (43 to 160)	4 (4 to 5)
Egypt	2037	223 (179 to 274)	69 (52 to 88)	134 (103 to 168)	20 (10 to 36)	1 (1 to 1)	1020 (820 to 1255)	315 (238 to 403)	612 (471 to 770)	89 (44 to 163)	4 (4 to 5)
Egypt	2038	228 (184 to 277)	71 (53 to 91)	136 (105 to 172)	20 (10 to 37)	1 (1 to 1)	1042 (842 to 1266)	323 (245 to 415)	623 (479 to 786)	91 (45 to 167)	4 (4 to 5)
Egypt	2039	233 (188 to 285)	73 (55 to 93)	139 (106 to 175)	21 (10 to 38)	1 (1 to 1)	1065 (861 to 1303)	332 (251 to 425)	635 (487 to 802)	94 (47 to 172)	4 (4 to 6)
Egypt	2040	238 (193 to 288)	75 (56 to 96)	142 (108 to 179)	21 (10 to 39)	1 (1 to 1)	1090 (882 to 1317)	342 (256 to 440)	648 (496 to 818)	96 (48 to 177)	4 (4 to 6)
Egypt	2041	244 (196 to 296)	77 (58 to 98)	144 (110 to 183)	22 (11 to 40)	1 (1 to 1)	1115 (899 to 1357)	351 (264 to 450)	661 (505 to 835)	99 (49 to 182)	4 (4 to 6)

Egypt	2042	249 (201 to 302)	79 (59 to 101)	147 (113 to 186)	22 (11 to 41)	1 (1 to 1)	1141 (918 to 1384)	361 (271 to 464)	674 (515 to 853)	102 (50 to 188)	4 (4 to 6)
Egypt	2043	255 (205 to 310)	81 (61 to 104)	150 (115 to 190)	23 (11 to 42)	1 (1 to 1)	1168 (939 to 1417)	372 (278 to 477)	687 (525 to 870)	104 (51 to 193)	4 (4 to 6)
Egypt	2044	261 (210 to 318)	84 (62 to 108)	153 (117 to 194)	23 (11 to 43)	1 (1 to 1)	1195 (962 to 1453)	383 (285 to 496)	701 (536 to 887)	107 (53 to 198)	4 (4 to 6)
Egypt	2045	267 (215 to 325)	86 (64 to 111)	156 (120 to 197)	24 (12 to 44)	1 (1 to 1)	1223 (982 to 1489)	394 (294 to 510)	714 (547 to 903)	110 (54 to 203)	5 (4 to 6)
Egypt	2046	273 (219 to 330)	89 (66 to 115)	159 (122 to 201)	25 (12 to 45)	1 (1 to 1)	1250 (1004 to 1508)	405 (303 to 525)	727 (558 to 921)	113 (55 to 208)	5 (4 to 6)
Egypt	2047	279 (225 to 343)	91 (68 to 119)	162 (124 to 205)	25 (12 to 46)	1 (1 to 1)	1278 (1031 to 1569)	417 (311 to 542)	740 (568 to 937)	116 (56 to 213)	5 (4 to 6)
Egypt	2048	285 (229 to 347)	94 (70 to 122)	165 (126 to 208)	26 (13 to 48)	1 (1 to 1)	1306 (1046 to 1589)	428 (319 to 557)	754 (578 to 954)	119 (58 to 218)	5 (4 to 6)
Egypt	2049	291 (234 to 357)	96 (72 to 125)	168 (128 to 212)	27 (13 to 49)	1 (1 to 1)	1334 (1073 to 1632)	440 (328 to 573)	767 (587 to 970)	122 (59 to 223)	5 (4 to 6)
Egypt	2050	298 (239 to 365)	99 (74 to 128)	171 (130 to 215)	27 (13 to 50)	1 (1 to 1)	1362 (1093 to 1669)	452 (336 to 585)	780 (596 to 985)	125 (61 to 228)	5 (4 to 6)
El Salvador	1995	221 (194 to 250)	76 (64 to 90)	133 (110 to 158)	5 (2 to 9)	8 (8 to 8)	457 (401 to 517)	157 (131 to 186)	274 (227 to 327)	9 (5 to 18)	17 (17 to 17)
El Salvador	1996	248 (222 to 277)	89 (75 to 104)	144 (122 to 169)	6 (3 to 11)	9 (9 to 9)	513 (459 to 572)	183 (155 to 214)	298 (253 to 350)	12 (6 to 23)	19 (19 to 19)

El Salvador	1997	254 (229 to 283)	94 (80 to 109)	149 (128 to 173)	6 (3 to 12)	5 (5 to 5)	524 (473 to 584)	195 (166 to 226)	307 (264 to 358)	13 (7 to 25)	10 (10 to 10)
El Salvador	1998	273 (246 to 302)	107 (92 to 123)	152 (132 to 176)	8 (4 to 14)	6 (6 to 6)	563 (508 to 624)	221 (191 to 254)	314 (272 to 363)	16 (8 to 28)	13 (13 to 13)
El Salvador	1999	280 (253 to 310)	115 (100 to 132)	150 (130 to 174)	9 (5 to 16)	5 (5 to 5)	578 (522 to 640)	238 (206 to 272)	311 (269 to 359)	18 (9 to 32)	11 (11 to 11)
El Salvador	2000	286 (259 to 317)	124 (107 to 141)	149 (129 to 171)	9 (5 to 17)	5 (5 to 5)	592 (536 to 654)	256 (221 to 291)	307 (266 to 353)	20 (10 to 34)	10 (10 to 10)
El Salvador	2001	281 (257 to 309)	122 (107 to 139)	141 (123 to 162)	10 (5 to 17)	8 (8 to 8)	581 (530 to 639)	253 (221 to 287)	292 (253 to 335)	20 (10 to 35)	16 (16 to 16)
El Salvador	2002	278 (252 to 305)	126 (110 to 142)	136 (117 to 158)	10 (5 to 18)	5 (5 to 5)	573 (521 to 631)	261 (228 to 293)	282 (243 to 325)	21 (10 to 36)	10 (10 to 10)
El Salvador	2003	279 (253 to 306)	133 (117 to 150)	132 (113 to 153)	10 (5 to 19)	4 (4 to 4)	577 (523 to 632)	274 (242 to 309)	272 (234 to 316)	22 (11 to 39)	9 (9 to 9)
El Salvador	2004	269 (244 to 294)	131 (116 to 149)	121 (105 to 140)	10 (5 to 18)	5 (5 to 5)	555 (504 to 607)	272 (240 to 308)	251 (217 to 290)	21 (11 to 38)	11 (11 to 11)
El Salvador	2005	272 (247 to 296)	140 (123 to 158)	114 (98 to 131)	11 (6 to 19)	7 (7 to 7)	561 (510 to 612)	289 (255 to 325)	235 (203 to 270)	23 (12 to 40)	14 (14 to 14)
El Salvador	2006	269 (245 to 294)	148 (131 to 167)	104 (90 to 119)	12 (6 to 20)	6 (6 to 6)	556 (506 to 606)	305 (270 to 344)	215 (185 to 247)	24 (13 to 42)	12 (12 to 12)
El Salvador	2007	264 (241 to 288)	148 (131 to 166)	99 (85 to 115)	12 (6 to 21)	6 (6 to 6)	546 (499 to 596)	305 (270 to 344)	204 (175 to 237)	25 (13 to 43)	12 (12 to 12)
El Salvador	2008	267 (244 to 291)	152 (134 to 171)	97 (82 to 113)	12 (7 to 21)	6 (6 to 6)	552 (504 to 602)	314 (278 to 354)	200 (169 to 233)	26 (14 to 44)	13 (13 to 13)
El Salvador	2009	273 (249 to 300)	157 (139 to 177)	96 (81 to 112)	13 (7 to 22)	6 (6 to 6)	564 (514 to 620)	325 (287 to 366)	198 (168 to 231)	27 (14 to 46)	13 (13 to 13)
El Salvador	2010	283 (259 to 311)	163 (144 to 183)	94 (80 to 109)	13 (7 to 22)	13 (13 to 13)	585 (535 to 643)	337 (298 to 379)	194 (165 to 226)	28 (14 to 46)	26 (26 to 26)
El Salvador	2011	283 (257 to 309)	171 (152 to 193)	92 (78 to 107)	14 (7 to 23)	6 (6 to 6)	585 (530 to 638)	354 (314 to 398)	190 (161 to 221)	29 (15 to 47)	13 (13 to 13)

El Salvador	2012	286 (260 to 313)	176 (157 to 197)	89 (76 to 105)	15 (8 to 25)	5 (5 to 5)	590 (537 to 646)	364 (323 to 408)	185 (157 to 218)	30 (16 to 52)	11 (11 to 11)
El Salvador	2013	294 (267 to 322)	184 (163 to 206)	87 (74 to 103)	16 (8 to 27)	7 (7 to 7)	607 (552 to 665)	380 (337 to 425)	180 (152 to 213)	32 (17 to 55)	15 (15 to 15)
El Salvador	2014	293 (266 to 323)	187 (166 to 209)	86 (71 to 102)	17 (9 to 29)	4 (4 to 4)	605 (550 to 666)	386 (343 to 431)	177 (147 to 212)	34 (18 to 59)	8 (8 to 8)
El Salvador	2015	300 (272 to 330)	192 (171 to 215)	85 (69 to 102)	18 (9 to 31)	5 (5 to 5)	620 (562 to 681)	396 (353 to 445)	176 (143 to 210)	37 (19 to 64)	11 (11 to 11)
El Salvador	2016	309 (279 to 341)	198 (175 to 224)	86 (69 to 104)	19 (10 to 34)	6 (6 to 6)	638 (577 to 704)	409 (362 to 463)	177 (143 to 215)	40 (21 to 71)	12 (12 to 12)
El Salvador	2017	315 (275 to 366)	200 (171 to 229)	87 (69 to 107)	21 (11 to 36)	8 (8 to 8)	650 (568 to 756)	412 (354 to 473)	179 (142 to 221)	42 (22 to 74)	17 (17 to 17)
El Salvador	2018	320 (277 to 371)	203 (176 to 234)	88 (70 to 109)	21 (11 to 37)	8 (8 to 8)	661 (571 to 766)	420 (363 to 484)	182 (144 to 225)	43 (23 to 76)	16 (16 to 16)
El Salvador	2019	329 (286 to 384)	210 (182 to 244)	89 (71 to 110)	21 (11 to 38)	8 (8 to 8)	680 (591 to 794)	434 (376 to 504)	185 (146 to 228)	44 (23 to 78)	16 (16 to 16)
El Salvador	2020	335 (289 to 389)	214 (185 to 249)	90 (72 to 112)	22 (12 to 39)	8 (8 to 9)	692 (597 to 805)	443 (382 to 515)	187 (148 to 231)	45 (24 to 80)	17 (16 to 18)
El Salvador	2021	342 (297 to 402)	220 (189 to 256)	92 (73 to 113)	22 (12 to 39)	8 (8 to 9)	707 (614 to 830)	454 (391 to 530)	189 (150 to 234)	46 (24 to 82)	17 (16 to 18)
El Salvador	2022	349 (301 to 408)	225 (193 to 262)	93 (73 to 114)	23 (12 to 40)	8 (8 to 9)	721 (623 to 842)	465 (399 to 541)	191 (152 to 236)	47 (25 to 83)	18 (16 to 19)
El Salvador	2023	356 (309 to 418)	231 (198 to 269)	93 (74 to 115)	23 (12 to 41)	9 (8 to 10)	736 (638 to 864)	477 (409 to 555)	193 (153 to 238)	48 (25 to 85)	18 (16 to 20)
El Salvador	2024	364 (312 to 424)	237 (204 to 276)	94 (75 to 116)	24 (12 to 42)	9 (8 to 10)	751 (645 to 877)	489 (421 to 569)	195 (155 to 240)	49 (26 to 86)	18 (16 to 21)
El Salvador	2025	371 (322 to 436)	243 (209 to 283)	95 (75 to 117)	24 (13 to 43)	9 (8 to 10)	767 (665 to 901)	502 (431 to 584)	196 (156 to 242)	50 (26 to 88)	19 (17 to 21)
El Salvador	2026	379 (326 to 443)	249 (214 to 289)	96 (76 to 118)	25 (13 to 44)	9 (8 to 10)	783 (673 to 916)	515 (443 to 598)	198 (157 to 244)	51 (27 to 90)	19 (17 to 22)

El Salvador	2027	387 (336 to 454)	255 (220 to 295)	96 (77 to 119)	25 (13 to 45)	9 (8 to 11)	799 (695 to 937)	528 (454 to 610)	199 (158 to 245)	52 (27 to 92)	19 (17 to 22)
El Salvador	2028	395 (340 to 461)	262 (226 to 303)	97 (77 to 120)	26 (13 to 45)	10 (8 to 11)	815 (703 to 953)	541 (466 to 626)	201 (159 to 247)	53 (28 to 94)	20 (17 to 23)
El Salvador	2029	403 (349 to 471)	269 (232 to 311)	98 (78 to 120)	26 (14 to 46)	10 (9 to 12)	832 (721 to 974)	555 (479 to 643)	202 (161 to 249)	54 (28 to 96)	20 (18 to 24)
El Salvador	2030	411 (355 to 482)	276 (238 to 318)	99 (78 to 121)	27 (14 to 47)	10 (9 to 12)	850 (733 to 996)	570 (492 to 657)	204 (162 to 251)	56 (29 to 98)	21 (18 to 25)
El Salvador	2031	420 (363 to 493)	283 (245 to 327)	99 (79 to 122)	27 (14 to 48)	10 (9 to 12)	867 (751 to 1018)	584 (505 to 676)	205 (163 to 252)	57 (29 to 100)	21 (18 to 25)
El Salvador	2032	428 (369 to 502)	290 (251 to 336)	100 (79 to 123)	28 (14 to 49)	11 (9 to 13)	885 (762 to 1037)	599 (519 to 695)	207 (164 to 254)	58 (30 to 102)	22 (19 to 26)
El Salvador	2033	437 (378 to 512)	297 (257 to 345)	101 (80 to 124)	29 (15 to 51)	11 (9 to 13)	904 (780 to 1058)	614 (530 to 714)	208 (165 to 256)	59 (31 to 104)	22 (19 to 27)
El Salvador	2034	447 (384 to 525)	305 (263 to 354)	101 (80 to 125)	29 (15 to 52)	11 (9 to 13)	923 (792 to 1085)	630 (543 to 732)	210 (166 to 258)	60 (31 to 107)	23 (19 to 28)
El Salvador	2035	456 (393 to 535)	313 (268 to 364)	102 (81 to 126)	30 (15 to 53)	11 (9 to 14)	942 (812 to 1105)	646 (554 to 752)	211 (167 to 260)	61 (32 to 109)	24 (20 to 29)
El Salvador	2036	465 (397 to 547)	320 (273 to 373)	103 (81 to 127)	30 (16 to 54)	12 (10 to 15)	961 (820 to 1130)	662 (565 to 771)	213 (168 to 262)	63 (32 to 111)	24 (20 to 30)
El Salvador	2037	475 (409 to 559)	328 (280 to 383)	104 (82 to 128)	31 (16 to 55)	12 (10 to 15)	981 (844 to 1155)	678 (578 to 791)	214 (169 to 264)	64 (33 to 114)	25 (20 to 30)
El Salvador	2038	484 (412 to 570)	336 (285 to 392)	104 (82 to 128)	32 (16 to 56)	12 (10 to 15)	1001 (851 to 1178)	695 (590 to 811)	216 (170 to 265)	65 (33 to 116)	25 (21 to 32)
El Salvador	2039	494 (422 to 583)	344 (291 to 403)	105 (83 to 129)	32 (16 to 57)	13 (10 to 16)	1021 (873 to 1205)	711 (602 to 832)	217 (172 to 267)	66 (34 to 119)	26 (22 to 33)
El Salvador	2040	504 (426 to 592)	353 (297 to 414)	106 (84 to 130)	33 (17 to 59)	13 (11 to 16)	1041 (880 to 1222)	728 (615 to 855)	218 (173 to 269)	68 (35 to 121)	27 (22 to 34)

El Salvador	2041	514 (436 to 607)	361 (303 to 424)	106 (84 to 131)	33 (17 to 60)	13 (11 to 17)	1062 (902 to 1255)	746 (625 to 876)	220 (174 to 270)	69 (35 to 124)	28 (23 to 35)
El Salvador	2042	524 (440 to 619)	369 (308 to 435)	107 (85 to 132)	34 (17 to 61)	14 (11 to 17)	1083 (908 to 1279)	763 (636 to 898)	221 (175 to 272)	70 (36 to 126)	28 (23 to 36)
El Salvador	2043	534 (451 to 631)	378 (313 to 445)	108 (85 to 132)	35 (18 to 62)	14 (11 to 18)	1103 (931 to 1304)	780 (646 to 918)	222 (175 to 274)	72 (36 to 129)	29 (24 to 38)
El Salvador	2044	544 (452 to 644)	386 (318 to 455)	108 (85 to 133)	35 (18 to 64)	15 (12 to 19)	1124 (934 to 1330)	797 (656 to 940)	224 (176 to 275)	73 (37 to 131)	30 (24 to 40)
El Salvador	2045	554 (462 to 656)	394 (322 to 465)	109 (86 to 134)	36 (18 to 65)	15 (12 to 19)	1144 (954 to 1355)	814 (666 to 960)	225 (177 to 276)	74 (38 to 134)	31 (25 to 40)
El Salvador	2046	563 (465 to 669)	402 (327 to 475)	109 (86 to 134)	36 (18 to 66)	15 (12 to 20)	1164 (961 to 1383)	831 (676 to 981)	226 (178 to 278)	75 (38 to 137)	32 (25 to 41)
El Salvador	2047	573 (476 to 682)	410 (331 to 485)	110 (87 to 135)	37 (19 to 67)	16 (13 to 21)	1184 (983 to 1409)	848 (685 to 1001)	227 (179 to 279)	77 (39 to 139)	33 (26 to 43)
El Salvador	2048	583 (477 to 692)	418 (336 to 495)	110 (87 to 136)	38 (19 to 69)	16 (13 to 22)	1204 (986 to 1429)	864 (695 to 1024)	228 (180 to 280)	78 (40 to 142)	34 (27 to 45)
El Salvador	2049	592 (489 to 707)	426 (341 to 505)	111 (87 to 136)	38 (19 to 70)	17 (13 to 22)	1223 (1011 to 1461)	880 (704 to 1044)	229 (180 to 282)	79 (40 to 145)	35 (28 to 46)
El Salvador	2050	601 (492 to 714)	434 (345 to 516)	111 (88 to 137)	39 (20 to 72)	17 (14 to 24)	1242 (1016 to 1476)	896 (713 to 1065)	230 (181 to 283)	80 (41 to 148)	36 (29 to 49)

Equatorial Guinea	1995	144 (114 to 177)	9 (6 to 11)	131 (103 to 163)	3 (1 to 5)	2 (2 to 2)	343 (272 to 420)	20 (15 to 27)	312 (244 to 387)	6 (3 to 11)	4 (4 to 4)
Equatorial Guinea	1996	121 (100 to 144)	5 (4 to 7)	102 (82 to 125)	1 (1 to 2)	12 (12 to 12)	287 (238 to 343)	13 (10 to 16)	243 (195 to 298)	3 (2 to 6)	28 (28 to 28)
Equatorial Guinea	1997	136 (113 to 162)	9 (6 to 11)	123 (100 to 149)	2 (1 to 4)	3 (3 to 3)	324 (269 to 384)	20 (15 to 26)	293 (238 to 355)	5 (2 to 9)	6 (6 to 6)
Equatorial Guinea	1998	170 (142 to 201)	14 (11 to 18)	150 (124 to 181)	3 (2 to 6)	3 (3 to 3)	404 (338 to 479)	34 (26 to 44)	355 (294 to 430)	8 (4 to 14)	6 (6 to 6)
Equatorial Guinea	1999	152 (129 to 181)	13 (10 to 16)	133 (111 to 160)	3 (1 to 5)	4 (4 to 4)	362 (308 to 430)	30 (23 to 38)	315 (263 to 380)	7 (4 to 13)	10 (10 to 10)
Equatorial Guinea	2000	176 (152 to 204)	17 (13 to 22)	147 (123 to 174)	4 (2 to 8)	7 (7 to 7)	418 (362 to 485)	41 (32 to 51)	349 (293 to 415)	10 (5 to 18)	18 (18 to 18)
Equatorial Guinea	2001	186 (163 to 214)	17 (13 to 21)	159 (135 to 185)	4 (2 to 8)	6 (6 to 6)	443 (387 to 508)	41 (32 to 51)	377 (321 to 440)	10 (5 to 19)	14 (14 to 14)
Equatorial Guinea	2002	191 (168 to 219)	17 (13 to 21)	166 (142 to 193)	4 (2 to 8)	4 (4 to 4)	455 (399 to 521)	40 (32 to 50)	394 (339 to 458)	11 (5 to 19)	10 (10 to 10)
Equatorial Guinea	2003	197 (173 to 225)	19 (15 to 24)	168 (145 to 195)	5 (3 to 9)	5 (5 to 5)	469 (411 to 534)	46 (36 to 56)	399 (344 to 463)	12 (6 to 22)	12 (12 to 12)
Equatorial Guinea	2004	230 (203 to 262)	29 (23 to 35)	187 (160 to 217)	8 (4 to 14)	7 (7 to 7)	548 (483 to 622)	69 (56 to 83)	445 (381 to 516)	18 (9 to 33)	16 (16 to 16)
Equatorial Guinea	2005	226 (200 to 253)	31 (25 to 36)	174 (149 to 201)	8 (4 to 15)	13 (13 to 13)	537 (475 to 601)	73 (60 to 87)	413 (354 to 478)	19 (10 to 36)	32 (32 to 32)
Equatorial Guinea	2006	240 (214 to 268)	38 (32 to 45)	176 (151 to 202)	10 (5 to 18)	16 (16 to 16)	571 (508 to 637)	90 (75 to 107)	419 (358 to 480)	24 (12 to 43)	39 (39 to 39)
Equatorial Guinea	2007	242 (214 to 272)	46 (38 to 54)	184 (158 to 211)	12 (6 to 22)	0 (0 to 0)	575 (509 to 647)	108 (90 to 128)	437 (375 to 502)	28 (14 to 51)	1 (1 to 1)
Equatorial Guinea	2008	266 (237 to 298)	55 (46 to 64)	196 (169 to 227)	14 (7 to 25)	1 (1 to 1)	631 (564 to 708)	130 (110 to 152)	466 (403 to 540)	34 (16 to 60)	2 (2 to 2)
Equatorial Guinea	2009	325 (291 to 366)	87 (74 to 101)	216 (187 to 250)	22 (11 to 40)	0 (0 to 0)	773 (691 to 869)	206 (175 to 239)	514 (444 to 593)	52 (25 to 95)	1 (1 to 1)

Equatorial Guinea	2010	267 (239 to 302)	69 (59 to 81)	180 (155 to 208)	18 (9 to 32)	0 (0 to 0)	636 (568 to 718)	165 (141 to 192)	428 (369 to 494)	42 (20 to 76)	0 (0 to 0)
Equatorial Guinea	2011	249 (223 to 280)	67 (57 to 78)	165 (143 to 189)	17 (8 to 31)	0 (0 to 0)	593 (529 to 665)	159 (136 to 186)	393 (340 to 450)	41 (20 to 75)	0 (0 to 0)
Equatorial Guinea	2012	273 (245 to 307)	80 (69 to 94)	173 (149 to 199)	20 (10 to 37)	0 (0 to 0)	650 (581 to 729)	190 (164 to 223)	411 (355 to 474)	48 (23 to 89)	1 (1 to 1)
Equatorial Guinea	2013	277 (248 to 310)	68 (59 to 80)	191 (165 to 220)	17 (8 to 32)	0 (0 to 0)	657 (589 to 736)	163 (139 to 191)	453 (392 to 523)	41 (20 to 77)	0 (0 to 0)
Equatorial Guinea	2014	314 (282 to 351)	71 (60 to 83)	225 (197 to 258)	18 (9 to 33)	0 (0 to 0)	747 (671 to 835)	168 (142 to 198)	536 (468 to 614)	42 (20 to 80)	1 (1 to 1)
Equatorial Guinea	2015	365 (326 to 406)	78 (66 to 93)	265 (232 to 302)	19 (9 to 36)	3 (3 to 3)	867 (775 to 966)	187 (157 to 221)	629 (551 to 717)	45 (22 to 86)	6 (6 to 6)
Equatorial Guinea	2016	330 (295 to 374)	63 (52 to 75)	248 (215 to 286)	14 (7 to 27)	6 (6 to 6)	785 (702 to 889)	150 (124 to 178)	589 (511 to 680)	33 (16 to 64)	13 (13 to 13)
Equatorial Guinea	2017	299 (257 to 345)	52 (42 to 63)	230 (194 to 273)	11 (5 to 21)	7 (7 to 7)	711 (611 to 821)	123 (100 to 150)	546 (460 to 649)	25 (12 to 50)	16 (16 to 16)
Equatorial Guinea	2018	279 (242 to 324)	49 (40 to 62)	213 (179 to 253)	10 (5 to 20)	7 (7 to 7)	664 (576 to 770)	118 (95 to 147)	506 (426 to 602)	24 (12 to 47)	16 (15 to 16)
Equatorial Guinea	2019	256 (222 to 295)	43 (34 to 54)	197 (166 to 234)	9 (4 to 18)	7 (7 to 7)	608 (527 to 702)	102 (81 to 129)	468 (394 to 556)	22 (10 to 42)	16 (16 to 17)
Equatorial Guinea	2020	240 (206 to 277)	41 (32 to 52)	183 (154 to 217)	9 (4 to 17)	7 (7 to 8)	569 (490 to 659)	98 (77 to 124)	434 (366 to 516)	21 (10 to 40)	17 (16 to 18)
Equatorial Guinea	2021	247 (214 to 285)	44 (34 to 56)	187 (157 to 222)	9 (4 to 17)	7 (7 to 8)	587 (509 to 678)	104 (81 to 134)	444 (374 to 528)	22 (10 to 41)	17 (16 to 18)
Equatorial Guinea	2022	253 (218 to 292)	47 (36 to 60)	189 (159 to 225)	10 (5 to 18)	7 (6 to 8)	600 (519 to 695)	111 (86 to 143)	450 (379 to 534)	23 (11 to 44)	17 (15 to 19)
Equatorial Guinea	2023	261 (226 to 302)	51 (39 to 65)	193 (163 to 230)	10 (5 to 19)	7 (6 to 8)	620 (536 to 719)	120 (93 to 154)	459 (386 to 546)	24 (11 to 46)	17 (15 to 19)
Equatorial Guinea	2024	273 (235 to 315)	55 (43 to 70)	200 (168 to 238)	11 (5 to 21)	7 (6 to 8)	649 (560 to 748)	131 (102 to 167)	475 (400 to 565)	26 (12 to 49)	16 (15 to 19)

Equatorial Guinea	2025	285 (246 to 331)	60 (47 to 77)	207 (174 to 246)	11 (5 to 22)	7 (6 to 8)	678 (584 to 786)	143 (111 to 182)	493 (414 to 585)	27 (13 to 52)	16 (14 to 19)
Equatorial Guinea	2026	298 (258 to 344)	65 (51 to 83)	214 (181 to 254)	12 (6 to 24)	7 (6 to 8)	708 (614 to 818)	154 (120 to 197)	509 (429 to 605)	29 (14 to 56)	16 (14 to 18)
Equatorial Guinea	2027	311 (268 to 360)	70 (55 to 90)	221 (187 to 263)	13 (6 to 25)	7 (6 to 8)	740 (638 to 857)	167 (130 to 213)	526 (444 to 625)	31 (15 to 60)	16 (14 to 18)
Equatorial Guinea	2028	324 (282 to 375)	76 (59 to 97)	228 (193 to 271)	14 (7 to 27)	7 (6 to 8)	771 (671 to 892)	180 (141 to 230)	543 (458 to 645)	33 (15 to 63)	16 (13 to 18)
Equatorial Guinea	2029	338 (291 to 394)	81 (64 to 104)	235 (198 to 280)	15 (7 to 28)	6 (6 to 8)	803 (691 to 936)	194 (152 to 247)	559 (472 to 665)	34 (16 to 67)	15 (13 to 18)
Equatorial Guinea	2030	352 (304 to 409)	88 (69 to 112)	243 (205 to 289)	15 (7 to 30)	6 (5 to 8)	837 (723 to 972)	209 (163 to 267)	577 (486 to 686)	37 (17 to 71)	15 (13 to 19)
Equatorial Guinea	2031	367 (315 to 429)	94 (74 to 121)	250 (210 to 296)	16 (8 to 32)	6 (5 to 8)	872 (750 to 1019)	224 (175 to 288)	594 (500 to 705)	39 (18 to 75)	15 (12 to 18)
Equatorial Guinea	2032	380 (327 to 444)	101 (79 to 130)	256 (216 to 304)	17 (8 to 33)	6 (0 to 8)	904 (776 to 1056)	239 (187 to 309)	609 (513 to 722)	41 (19 to 79)	15 (0 to 19)
Equatorial Guinea	2033	394 (339 to 461)	107 (84 to 139)	263 (221 to 312)	18 (9 to 35)	6 (0 to 8)	937 (806 to 1095)	255 (199 to 330)	625 (526 to 741)	43 (20 to 84)	14 (0 to 18)
Equatorial Guinea	2034	407 (349 to 476)	113 (88 to 147)	270 (227 to 320)	19 (9 to 37)	6 (0 to 8)	969 (830 to 1131)	269 (209 to 350)	642 (539 to 760)	45 (21 to 87)	13 (0 to 18)
Equatorial Guinea	2035	421 (359 to 493)	120 (93 to 155)	277 (233 to 329)	20 (9 to 38)	5 (0 to 8)	1002 (853 to 1173)	284 (220 to 369)	658 (553 to 782)	47 (22 to 91)	13 (0 to 18)
Equatorial Guinea	2036	435 (370 to 511)	126 (98 to 164)	284 (239 to 338)	20 (10 to 40)	5 (0 to 8)	1034 (879 to 1215)	299 (232 to 389)	675 (567 to 803)	49 (23 to 95)	12 (0 to 19)
Equatorial Guinea	2037	449 (382 to 523)	133 (103 to 174)	291 (244 to 346)	21 (10 to 42)	5 (0 to 8)	1068 (909 to 1245)	315 (244 to 414)	691 (581 to 822)	51 (24 to 100)	11 (0 to 18)

Equatorial Guinea	2038	462 (393 to 550)	139 (107 to 183)	297 (249 to 353)	22 (11 to 44)	4 (0 to 8)	1099 (935 to 1308)	330 (255 to 434)	706 (593 to 840)	53 (25 to 104)	10 (0 to 18)
Equatorial Guinea	2039	477 (407 to 559)	146 (113 to 193)	304 (255 to 361)	23 (11 to 45)	4 (0 to 8)	1134 (967 to 1329)	348 (269 to 460)	722 (606 to 859)	55 (26 to 108)	9 (0 to 19)
Equatorial Guinea	2040	491 (416 to 587)	153 (118 to 204)	310 (260 to 369)	24 (11 to 47)	4 (0 to 8)	1166 (990 to 1395)	364 (281 to 484)	737 (618 to 877)	57 (27 to 112)	8 (0 to 19)
Equatorial Guinea	2041	505 (429 to 590)	160 (123 to 213)	316 (266 to 377)	25 (12 to 49)	3 (0 to 8)	1200 (1021 to 1403)	381 (294 to 507)	752 (631 to 897)	59 (28 to 116)	8 (0 to 19)
Equatorial Guinea	2042	519 (438 to 619)	167 (129 to 224)	323 (270 to 385)	26 (12 to 51)	3 (0 to 8)	1233 (1040 to 1471)	397 (306 to 533)	768 (642 to 915)	61 (29 to 120)	7 (0 to 19)
Equatorial Guinea	2043	532 (449 to 625)	174 (134 to 233)	329 (275 to 393)	27 (13 to 52)	3 (0 to 8)	1265 (1068 to 1485)	413 (318 to 555)	782 (653 to 934)	63 (30 to 124)	7 (0 to 19)
Equatorial Guinea	2044	544 (460 to 654)	180 (138 to 242)	335 (279 to 401)	27 (13 to 54)	2 (0 to 8)	1294 (1093 to 1555)	427 (328 to 576)	797 (664 to 952)	65 (31 to 128)	6 (0 to 19)
Equatorial Guinea	2045	557 (470 to 663)	186 (142 to 252)	341 (284 to 408)	28 (13 to 55)	2 (0 to 8)	1324 (1118 to 1576)	442 (339 to 599)	810 (676 to 969)	67 (32 to 132)	5 (0 to 18)
Equatorial Guinea	2046	568 (477 to 686)	191 (146 to 263)	346 (289 to 414)	29 (14 to 57)	2 (0 to 8)	1351 (1134 to 1630)	455 (348 to 624)	822 (687 to 984)	69 (33 to 135)	5 (0 to 19)
Equatorial Guinea	2047	580 (487 to 694)	197 (150 to 270)	352 (293 to 422)	30 (14 to 58)	2 (0 to 8)	1380 (1158 to 1650)	468 (358 to 643)	837 (697 to 1003)	71 (33 to 139)	4 (0 to 19)

Equatorial Guinea	2048	592 (498 to 719)	202 (154 to 278)	358 (298 to 429)	30 (14 to 60)	2 (0 to 8)	1407 (1185 to 1710)	481 (366 to 660)	850 (708 to 1020)	72 (34 to 142)	4 (0 to 19)
Equatorial Guinea	2049	604 (506 to 730)	208 (158 to 289)	363 (301 to 436)	31 (15 to 62)	2 (0 to 8)	1435 (1202 to 1736)	495 (376 to 687)	862 (717 to 1036)	74 (35 to 146)	4 (0 to 19)
Equatorial Guinea	2050	615 (518 to 757)	214 (162 to 301)	368 (306 to 443)	32 (15 to 63)	2 (0 to 8)	1463 (1232 to 1801)	508 (386 to 715)	875 (727 to 1052)	76 (36 to 150)	4 (0 to 19)
Eritrea	1995	20 (17 to 25)	5 (4 to 7)	10 (7 to 15)	1 (1 to 2)	3 (3 to 3)	27 (22 to 33)	7 (5 to 9)	14 (10 to 20)	2 (1 to 3)	4 (4 to 4)
Eritrea	1996	21 (18 to 26)	5 (4 to 7)	11 (7 to 15)	1 (1 to 2)	4 (4 to 4)	28 (24 to 35)	7 (5 to 9)	14 (10 to 20)	2 (1 to 3)	6 (6 to 6)
Eritrea	1997	19 (15 to 23)	5 (4 to 6)	11 (7 to 15)	1 (1 to 2)	2 (2 to 2)	25 (20 to 31)	7 (5 to 9)	15 (10 to 21)	2 (1 to 3)	2 (2 to 2)
Eritrea	1998	24 (20 to 29)	6 (4 to 8)	12 (8 to 17)	1 (1 to 3)	4 (4 to 4)	32 (26 to 39)	8 (6 to 10)	16 (11 to 23)	2 (1 to 4)	5 (5 to 5)
Eritrea	1999	27 (22 to 32)	7 (5 to 9)	13 (9 to 18)	2 (1 to 3)	5 (5 to 5)	36 (30 to 43)	9 (7 to 12)	17 (12 to 25)	2 (1 to 4)	7 (7 to 7)
Eritrea	2000	27 (23 to 32)	6 (5 to 8)	12 (8 to 17)	1 (1 to 3)	7 (7 to 7)	36 (31 to 44)	8 (6 to 11)	16 (11 to 23)	2 (1 to 4)	10 (10 to 10)
Eritrea	2001	23 (19 to 28)	6 (5 to 8)	12 (8 to 16)	1 (1 to 3)	4 (4 to 4)	31 (26 to 37)	8 (6 to 11)	15 (11 to 22)	2 (1 to 4)	5 (5 to 5)
Eritrea	2002	24 (20 to 28)	6 (4 to 8)	11 (7 to 14)	1 (1 to 2)	6 (6 to 6)	32 (27 to 38)	8 (6 to 10)	14 (10 to 19)	2 (1 to 3)	8 (8 to 8)
Eritrea	2003	26 (22 to 30)	5 (4 to 7)	10 (7 to 13)	1 (1 to 2)	9 (9 to 9)	35 (30 to 40)	7 (5 to 9)	13 (9 to 18)	2 (1 to 3)	13 (13 to 13)
Eritrea	2004	22 (20 to 26)	4 (3 to 6)	8 (6 to 11)	1 (0 to 2)	9 (9 to 9)	30 (26 to 35)	6 (4 to 7)	11 (8 to 15)	1 (1 to 3)	12 (12 to 12)
Eritrea	2005	21 (18 to 24)	4 (3 to 5)	8 (6 to 11)	1 (0 to 2)	8 (8 to 8)	28 (25 to 33)	5 (4 to 6)	11 (8 to 15)	1 (1 to 2)	11 (11 to 11)

Eritrea	2006	15 (12 to 17)	3 (2 to 4)	7 (5 to 10)	1 (0 to 1)	4 (4 to 4)	20 (16 to 23)	4 (3 to 5)	10 (7 to 13)	1 (1 to 2)	5 (5 to 5)
Eritrea	2007	16 (13 to 18)	3 (2 to 4)	7 (5 to 10)	1 (0 to 1)	5 (5 to 5)	21 (18 to 25)	4 (3 to 5)	10 (7 to 13)	1 (0 to 2)	7 (7 to 7)
Eritrea	2008	16 (13 to 18)	2 (2 to 3)	7 (5 to 10)	1 (0 to 1)	6 (6 to 6)	21 (18 to 25)	3 (2 to 4)	9 (7 to 13)	1 (0 to 2)	8 (8 to 8)
Eritrea	2009	14 (12 to 17)	2 (2 to 3)	7 (5 to 9)	1 (0 to 1)	4 (4 to 4)	19 (16 to 22)	3 (2 to 4)	9 (6 to 13)	1 (0 to 2)	5 (5 to 5)
Eritrea	2010	20 (18 to 23)	2 (2 to 3)	7 (5 to 10)	1 (0 to 1)	10 (10 to 10)	27 (24 to 31)	3 (2 to 4)	10 (7 to 13)	1 (0 to 2)	13 (13 to 13)
Eritrea	2011	15 (12 to 18)	2 (2 to 3)	8 (5 to 10)	1 (0 to 1)	4 (4 to 4)	19 (16 to 24)	3 (2 to 4)	10 (7 to 14)	1 (0 to 1)	5 (5 to 5)
Eritrea	2012	17 (15 to 20)	3 (2 to 3)	8 (6 to 11)	1 (0 to 1)	6 (6 to 6)	23 (20 to 27)	3 (3 to 4)	11 (8 to 15)	1 (0 to 1)	8 (8 to 8)
Eritrea	2013	17 (15 to 21)	2 (2 to 3)	8 (6 to 12)	1 (0 to 1)	6 (6 to 6)	23 (20 to 28)	3 (2 to 4)	11 (8 to 16)	1 (0 to 1)	8 (8 to 8)
Eritrea	2014	19 (16 to 23)	2 (2 to 3)	9 (6 to 13)	1 (0 to 1)	6 (6 to 6)	25 (21 to 30)	3 (3 to 4)	12 (9 to 18)	1 (0 to 1)	9 (9 to 9)
Eritrea	2015	17 (13 to 21)	3 (2 to 4)	10 (7 to 14)	1 (0 to 1)	3 (3 to 3)	22 (18 to 28)	4 (3 to 5)	14 (10 to 19)	1 (0 to 1)	4 (4 to 4)
Eritrea	2016	20 (16 to 25)	4 (3 to 5)	12 (8 to 16)	1 (0 to 1)	4 (4 to 4)	27 (22 to 34)	5 (4 to 7)	16 (11 to 22)	1 (0 to 1)	5 (5 to 5)
Eritrea	2017	24 (20 to 30)	5 (4 to 6)	13 (9 to 18)	1 (0 to 1)	6 (6 to 6)	33 (27 to 41)	6 (5 to 8)	18 (12 to 25)	1 (0 to 1)	8 (8 to 8)
Eritrea	2018	25 (20 to 31)	4 (3 to 6)	15 (10 to 20)	1 (0 to 1)	6 (5 to 6)	34 (27 to 42)	6 (4 to 8)	20 (13 to 27)	1 (0 to 1)	7 (7 to 8)
Eritrea	2019	27 (21 to 33)	5 (4 to 7)	15 (10 to 21)	1 (0 to 1)	6 (6 to 6)	36 (29 to 44)	7 (5 to 10)	20 (13 to 28)	1 (0 to 2)	8 (7 to 8)
Eritrea	2020	27 (22 to 33)	6 (4 to 8)	15 (10 to 21)	1 (0 to 1)	6 (5 to 6)	37 (29 to 45)	8 (6 to 10)	20 (14 to 28)	1 (0 to 2)	8 (7 to 8)

Eritrea	2021	28 (22 to 34)	6 (4 to 8)	15 (10 to 22)	1 (0 to 1)	6 (5 to 6)	37 (30 to 46)	8 (6 to 11)	21 (14 to 29)	1 (0 to 2)	8 (7 to 8)
Eritrea	2022	28 (23 to 35)	6 (4 to 8)	16 (11 to 22)	1 (0 to 1)	6 (5 to 6)	38 (30 to 47)	8 (6 to 11)	21 (14 to 29)	1 (0 to 2)	8 (7 to 9)
Eritrea	2023	29 (23 to 36)	6 (5 to 9)	16 (11 to 22)	1 (0 to 1)	6 (5 to 6)	39 (31 to 48)	9 (6 to 12)	21 (14 to 30)	1 (0 to 2)	8 (7 to 9)
Eritrea	2024	29 (23 to 36)	7 (5 to 9)	16 (11 to 23)	1 (0 to 1)	6 (5 to 7)	39 (32 to 48)	9 (6 to 12)	22 (15 to 30)	1 (0 to 2)	8 (7 to 9)
Eritrea	2025	30 (24 to 37)	7 (5 to 9)	17 (11 to 23)	1 (0 to 1)	6 (5 to 7)	40 (32 to 50)	9 (6 to 13)	22 (15 to 31)	1 (0 to 2)	8 (7 to 9)
Eritrea	2026	30 (25 to 38)	7 (5 to 10)	17 (11 to 23)	1 (0 to 1)	6 (5 to 7)	41 (33 to 51)	9 (6 to 13)	23 (15 to 32)	1 (1 to 2)	8 (7 to 9)
Eritrea	2027	31 (24 to 38)	7 (5 to 10)	17 (11 to 24)	1 (0 to 1)	6 (5 to 7)	42 (33 to 52)	10 (7 to 13)	23 (15 to 32)	1 (1 to 2)	8 (7 to 9)
Eritrea	2028	31 (25 to 39)	7 (5 to 10)	17 (12 to 24)	1 (0 to 1)	6 (5 to 7)	42 (34 to 53)	10 (7 to 14)	23 (16 to 33)	1 (1 to 2)	8 (7 to 9)
Eritrea	2029	32 (25 to 40)	8 (5 to 11)	18 (12 to 25)	1 (0 to 2)	6 (5 to 7)	43 (34 to 54)	10 (7 to 14)	24 (16 to 33)	1 (1 to 2)	8 (7 to 9)
Eritrea	2030	33 (26 to 40)	8 (5 to 11)	18 (12 to 25)	1 (0 to 2)	6 (5 to 7)	44 (35 to 54)	11 (7 to 15)	24 (16 to 34)	1 (1 to 2)	8 (7 to 10)
Eritrea	2031	33 (26 to 42)	8 (6 to 12)	18 (12 to 26)	1 (0 to 2)	6 (5 to 7)	45 (35 to 56)	11 (7 to 15)	25 (16 to 35)	1 (1 to 2)	8 (7 to 10)
Eritrea	2032	34 (27 to 43)	8 (6 to 12)	19 (12 to 26)	1 (0 to 2)	6 (5 to 7)	46 (36 to 58)	11 (7 to 16)	25 (17 to 35)	1 (1 to 2)	8 (7 to 10)
Eritrea	2033	35 (27 to 43)	9 (6 to 12)	19 (13 to 27)	1 (0 to 2)	6 (5 to 7)	46 (37 to 58)	12 (8 to 17)	26 (17 to 36)	1 (1 to 2)	8 (7 to 10)
Eritrea	2034	35 (28 to 44)	9 (6 to 13)	19 (13 to 27)	1 (0 to 2)	6 (5 to 8)	47 (37 to 59)	12 (8 to 17)	26 (17 to 37)	1 (1 to 2)	8 (7 to 10)
Eritrea	2035	36 (28 to 45)	9 (6 to 13)	20 (13 to 28)	1 (0 to 2)	6 (5 to 8)	48 (38 to 60)	12 (8 to 17)	26 (18 to 37)	1 (1 to 2)	8 (7 to 10)

Eritrea	2036	37 (29 to 46)	9 (6 to 13)	20 (13 to 28)	1 (0 to 2)	6 (5 to 8)	49 (38 to 61)	13 (8 to 18)	27 (18 to 38)	1 (1 to 2)	8 (7 to 10)
Eritrea	2037	37 (29 to 47)	10 (6 to 14)	20 (14 to 28)	1 (1 to 2)	6 (5 to 8)	50 (39 to 63)	13 (9 to 19)	27 (18 to 38)	1 (1 to 2)	8 (7 to 10)
Eritrea	2038	38 (29 to 49)	10 (6 to 15)	21 (14 to 29)	1 (1 to 2)	6 (5 to 8)	51 (40 to 65)	13 (9 to 20)	28 (19 to 39)	1 (1 to 3)	8 (7 to 11)
Eritrea	2039	39 (30 to 49)	10 (7 to 15)	21 (14 to 29)	1 (1 to 2)	6 (5 to 8)	52 (41 to 65)	14 (9 to 21)	28 (19 to 40)	1 (1 to 3)	9 (7 to 11)
Eritrea	2040	40 (30 to 49)	11 (7 to 16)	21 (14 to 30)	1 (1 to 2)	6 (5 to 8)	53 (41 to 66)	14 (9 to 21)	29 (19 to 40)	1 (1 to 3)	9 (7 to 11)
Eritrea	2041	40 (31 to 51)	11 (7 to 16)	22 (14 to 30)	1 (1 to 2)	7 (5 to 8)	54 (42 to 68)	15 (9 to 22)	29 (19 to 41)	2 (1 to 3)	9 (7 to 11)
Eritrea	2042	41 (32 to 52)	11 (7 to 17)	22 (15 to 31)	1 (1 to 2)	7 (5 to 8)	55 (43 to 69)	15 (10 to 23)	30 (20 to 41)	2 (1 to 3)	9 (7 to 11)
Eritrea	2043	42 (32 to 53)	12 (7 to 18)	22 (15 to 31)	1 (1 to 2)	7 (5 to 9)	56 (44 to 71)	16 (10 to 24)	30 (20 to 42)	2 (1 to 3)	9 (7 to 12)
Eritrea	2044	43 (33 to 53)	12 (8 to 18)	23 (15 to 32)	1 (1 to 2)	7 (5 to 9)	57 (44 to 71)	16 (10 to 24)	30 (20 to 43)	2 (1 to 3)	9 (7 to 12)
Eritrea	2045	43 (34 to 55)	12 (8 to 19)	23 (15 to 32)	1 (1 to 2)	7 (5 to 9)	58 (45 to 73)	17 (10 to 25)	31 (21 to 43)	2 (1 to 3)	9 (7 to 12)
Eritrea	2046	44 (34 to 56)	13 (8 to 20)	23 (16 to 33)	1 (1 to 2)	7 (5 to 9)	59 (46 to 75)	17 (11 to 27)	31 (21 to 44)	2 (1 to 3)	9 (7 to 12)
Eritrea	2047	45 (34 to 58)	13 (8 to 20)	24 (16 to 33)	1 (1 to 2)	7 (5 to 9)	60 (46 to 77)	18 (11 to 27)	32 (21 to 45)	2 (1 to 3)	9 (7 to 13)
Eritrea	2048	46 (35 to 58)	14 (8 to 21)	24 (16 to 34)	1 (1 to 2)	7 (6 to 9)	62 (47 to 78)	18 (11 to 28)	32 (22 to 45)	2 (1 to 3)	10 (7 to 13)
Eritrea	2049	47 (36 to 60)	14 (8 to 22)	24 (16 to 34)	1 (1 to 2)	7 (6 to 10)	63 (48 to 81)	19 (11 to 29)	33 (22 to 46)	2 (1 to 3)	10 (8 to 13)
Eritrea	2050	47 (37 to 61)	14 (8 to 23)	25 (16 to 35)	1 (1 to 2)	7 (6 to 10)	64 (49 to 82)	19 (11 to 30)	33 (22 to 47)	2 (1 to 3)	10 (8 to 13)

Estonia	1995	569 (530 to 610)	486 (449 to 525)	63 (51 to 77)	20 (11 to 34)	0 (0 to 0)	879 (820 to 944)	751 (694 to 811)	98 (79 to 119)	31 (16 to 52)	0 (0 to 0)
Estonia	1996	584 (552 to 619)	496 (466 to 525)	69 (57 to 82)	20 (11 to 34)	0 (0 to 0)	904 (853 to 956)	766 (720 to 811)	106 (88 to 127)	31 (17 to 52)	0 (0 to 0)
Estonia	1997	595 (565 to 628)	500 (473 to 526)	74 (63 to 86)	20 (11 to 33)	0 (0 to 0)	919 (873 to 970)	773 (732 to 814)	114 (97 to 133)	31 (17 to 52)	0 (0 to 0)
Estonia	1998	608 (574 to 641)	503 (476 to 530)	84 (72 to 96)	21 (12 to 35)	0 (0 to 0)	939 (887 to 990)	777 (736 to 819)	129 (112 to 149)	32 (19 to 54)	1 (1 to 1)
Estonia	1999	594 (564 to 625)	479 (454 to 503)	93 (82 to 105)	21 (12 to 34)	1 (1 to 1)	918 (872 to 966)	740 (702 to 778)	144 (126 to 163)	32 (18 to 53)	2 (2 to 2)
Estonia	2000	586 (558 to 617)	460 (438 to 484)	106 (93 to 119)	20 (12 to 33)	0 (0 to 0)	906 (863 to 954)	711 (677 to 748)	164 (144 to 184)	31 (18 to 51)	0 (0 to 0)
Estonia	2001	592 (564 to 621)	457 (434 to 481)	116 (103 to 130)	19 (11 to 31)	0 (0 to 0)	916 (873 to 960)	707 (672 to 743)	179 (160 to 201)	30 (17 to 48)	0 (0 to 0)
Estonia	2002	640 (610 to 669)	488 (463 to 513)	131 (117 to 147)	20 (11 to 32)	0 (0 to 0)	989 (943 to 1035)	755 (716 to 793)	203 (181 to 228)	32 (18 to 50)	0 (0 to 0)
Estonia	2003	693 (663 to 725)	523 (498 to 548)	148 (133 to 165)	21 (12 to 33)	1 (1 to 1)	1071 (1025 to 1120)	808 (770 to 847)	229 (206 to 255)	33 (18 to 51)	2 (2 to 2)
Estonia	2004	752 (721 to 784)	561 (537 to 586)	167 (152 to 185)	22 (13 to 33)	2 (2 to 2)	1162 (1115 to 1213)	867 (830 to 905)	259 (234 to 286)	33 (19 to 51)	3 (3 to 3)
Estonia	2005	828 (798 to 864)	617 (593 to 644)	187 (171 to 205)	21 (13 to 33)	2 (2 to 2)	1280 (1233 to 1335)	954 (917 to 995)	289 (264 to 317)	33 (20 to 51)	4 (4 to 4)
Estonia	2006	898 (868 to 929)	674 (649 to 701)	204 (188 to 221)	20 (12 to 31)	0 (0 to 0)	1388 (1341 to 1437)	1042 (1003 to 1083)	315 (291 to 341)	31 (19 to 48)	0 (0 to 0)
Estonia	2007	990 (960 to 1022)	748 (723 to 774)	220 (204 to 238)	22 (14 to 34)	0 (0 to 0)	1530 (1484 to 1580)	1156 (1118 to 1196)	340 (315 to 367)	34 (21 to 53)	0 (0 to 0)

Estonia	2008	1078 (1047 to 1112)	823 (798 to 851)	229 (214 to 246)	25 (16 to 38)	0 (0 to 0)	1666 (1618 to 1719)	1273 (1234 to 1316)	355 (331 to 380)	39 (25 to 59)	0 (0 to 0)
Estonia	2009	1083 (1049 to 1116)	830 (804 to 858)	228 (211 to 245)	24 (16 to 36)	0 (0 to 0)	1674 (1621 to 1725)	1284 (1242 to 1326)	352 (326 to 379)	38 (24 to 56)	0 (0 to 0)
Estonia	2010	1022 (991 to 1054)	777 (750 to 800)	223 (207 to 240)	22 (14 to 33)	0 (0 to 0)	1580 (1533 to 1629)	1200 (1160 to 1237)	345 (321 to 370)	34 (22 to 51)	0 (0 to 0)
Estonia	2011	1030 (1000 to 1059)	781 (756 to 805)	227 (211 to 244)	22 (14 to 33)	0 (0 to 0)	1592 (1546 to 1638)	1208 (1168 to 1244)	350 (326 to 377)	34 (22 to 52)	0 (0 to 0)
Estonia	2012	1104 (1071 to 1139)	841 (815 to 866)	241 (225 to 259)	22 (15 to 34)	0 (0 to 0)	1707 (1656 to 1760)	1299 (1260 to 1339)	373 (348 to 400)	35 (23 to 52)	0 (0 to 0)
Estonia	2013	1141 (1110 to 1175)	864 (839 to 893)	255 (238 to 272)	22 (14 to 33)	0 (0 to 0)	1764 (1716 to 1817)	1336 (1296 to 1380)	394 (368 to 421)	34 (22 to 52)	0 (0 to 0)
Estonia	2014	1191 (1157 to 1227)	898 (871 to 926)	271 (254 to 289)	22 (14 to 34)	0 (0 to 0)	1841 (1788 to 1897)	1388 (1347 to 1431)	418 (392 to 447)	34 (22 to 52)	0 (0 to 0)
Estonia	2015	1278 (1241 to 1313)	963 (935 to 991)	291 (273 to 311)	23 (14 to 35)	0 (0 to 0)	1975 (1918 to 2030)	1489 (1445 to 1532)	450 (422 to 480)	35 (22 to 54)	0 (0 to 0)
Estonia	2016	1336 (1300 to 1374)	1004 (974 to 1034)	308 (288 to 331)	24 (14 to 36)	0 (0 to 0)	2065 (2010 to 2124)	1552 (1506 to 1598)	476 (445 to 511)	36 (22 to 56)	0 (0 to 0)
Estonia	2017	1400 (1338 to 1462)	1050 (1008 to 1093)	326 (299 to 355)	24 (14 to 38)	0 (0 to 0)	2164 (2069 to 2261)	1622 (1558 to 1690)	504 (461 to 549)	38 (22 to 59)	0 (0 to 0)

Estonia	2018	1451 (1381 to 1523)	1088 (1038 to 1143)	337 (309 to 368)	25 (15 to 39)	0 (0 to 0)	2243 (2135 to 2355)	1682 (1604 to 1768)	522 (477 to 568)	39 (23 to 61)	0 (0 to 0)
Estonia	2019	1511 (1432 to 1589)	1139 (1079 to 1200)	345 (316 to 376)	26 (15 to 41)	0 (0 to 0)	2335 (2213 to 2456)	1761 (1668 to 1856)	533 (488 to 581)	41 (24 to 63)	0 (0 to 0)
Estonia	2020	1557 (1470 to 1644)	1176 (1110 to 1244)	354 (323 to 385)	27 (16 to 42)	0 (0 to 0)	2406 (2272 to 2542)	1817 (1716 to 1923)	547 (500 to 596)	42 (25 to 65)	0 (0 to 0)
Estonia	2021	1584 (1488 to 1680)	1199 (1126 to 1274)	358 (327 to 390)	28 (16 to 43)	0 (0 to 0)	2449 (2300 to 2597)	1853 (1741 to 1969)	553 (505 to 602)	43 (25 to 66)	0 (0 to 0)
Estonia	2022	1611 (1513 to 1710)	1221 (1139 to 1301)	362 (331 to 394)	28 (16 to 43)	0 (0 to 0)	2490 (2339 to 2644)	1887 (1760 to 2011)	560 (511 to 609)	43 (25 to 67)	0 (0 to 0)
Estonia	2023	1633 (1528 to 1747)	1239 (1155 to 1332)	365 (333 to 398)	29 (17 to 44)	0 (0 to 0)	2524 (2361 to 2701)	1916 (1785 to 2060)	564 (515 to 615)	44 (26 to 68)	0 (0 to 0)
Estonia	2024	1650 (1541 to 1768)	1254 (1165 to 1353)	366 (334 to 399)	29 (17 to 45)	0 (0 to 0)	2550 (2382 to 2734)	1939 (1801 to 2092)	567 (517 to 617)	45 (26 to 69)	0 (0 to 0)
Estonia	2025	1668 (1551 to 1790)	1270 (1171 to 1376)	368 (336 to 401)	29 (17 to 45)	0 (0 to 0)	2578 (2397 to 2767)	1964 (1811 to 2126)	569 (519 to 619)	45 (27 to 70)	0 (0 to 0)
Estonia	2026	1689 (1570 to 1815)	1289 (1189 to 1395)	371 (338 to 403)	30 (17 to 46)	0 (0 to 0)	2611 (2427 to 2806)	1993 (1838 to 2156)	573 (522 to 623)	46 (27 to 71)	0 (0 to 0)
Estonia	2027	1714 (1586 to 1847)	1310 (1205 to 1425)	374 (340 to 406)	30 (18 to 47)	0 (0 to 0)	2650 (2452 to 2855)	2026 (1862 to 2202)	578 (525 to 628)	46 (27 to 72)	0 (0 to 0)

Estonia	2028	1743 (1609 to 1884)	1335 (1218 to 1454)	378 (343 to 411)	31 (18 to 47)	0 (0 to 0)	2695 (2487 to 2912)	2063 (1884 to 2248)	584 (531 to 636)	47 (28 to 73)	0 (0 to 0)
Estonia	2029	1776 (1633 to 1921)	1362 (1236 to 1489)	383 (348 to 417)	31 (18 to 49)	0 (0 to 0)	2746 (2524 to 2969)	2106 (1910 to 2302)	592 (538 to 645)	48 (28 to 75)	0 (0 to 0)
Estonia	2030	1812 (1662 to 1970)	1392 (1257 to 1524)	389 (353 to 424)	32 (19 to 50)	0 (0 to 0)	2802 (2569 to 3045)	2151 (1944 to 2356)	601 (546 to 655)	49 (29 to 77)	0 (0 to 0)
Estonia	2031	1852 (1695 to 2017)	1424 (1284 to 1562)	395 (359 to 431)	33 (19 to 51)	0 (0 to 0)	2863 (2620 to 3117)	2201 (1986 to 2414)	611 (555 to 666)	51 (30 to 79)	0 (0 to 0)
Estonia	2032	1891 (1728 to 2061)	1456 (1307 to 1602)	402 (365 to 437)	33 (20 to 52)	0 (0 to 0)	2924 (2672 to 3185)	2251 (2021 to 2476)	621 (564 to 676)	52 (30 to 81)	0 (0 to 0)
Estonia	2033	1930 (1760 to 2104)	1488 (1339 to 1638)	408 (371 to 444)	34 (20 to 53)	0 (0 to 0)	2984 (2720 to 3252)	2300 (2069 to 2532)	630 (573 to 686)	53 (31 to 83)	0 (0 to 0)
Estonia	2034	1968 (1794 to 2156)	1520 (1364 to 1684)	414 (376 to 450)	35 (20 to 55)	0 (0 to 0)	3043 (2774 to 3332)	2349 (2109 to 2602)	640 (581 to 696)	54 (32 to 85)	0 (0 to 0)
Estonia	2035	2007 (1823 to 2200)	1551 (1388 to 1726)	420 (381 to 457)	36 (21 to 56)	0 (0 to 0)	3102 (2818 to 3401)	2398 (2146 to 2668)	649 (590 to 707)	55 (32 to 86)	0 (0 to 0)
Estonia	2036	2046 (1857 to 2247)	1584 (1415 to 1761)	426 (387 to 464)	37 (21 to 57)	0 (0 to 0)	3163 (2870 to 3474)	2448 (2188 to 2723)	658 (598 to 717)	56 (33 to 88)	0 (0 to 0)
Estonia	2037	2085 (1882 to 2288)	1616 (1444 to 1796)	432 (392 to 471)	37 (22 to 58)	0 (0 to 0)	3223 (2909 to 3537)	2498 (2232 to 2776)	667 (606 to 728)	58 (34 to 90)	0 (0 to 0)

Estonia	2038	2124 (1920 to 2341)	1649 (1468 to 1838)	437 (397 to 477)	38 (22 to 60)	0 (0 to 0)	3283 (2969 to 3619)	2548 (2269 to 2841)	676 (613 to 738)	59 (34 to 92)	0 (0 to 0)
Estonia	2039	2164 (1943 to 2387)	1682 (1487 to 1881)	443 (402 to 485)	39 (23 to 61)	0 (0 to 0)	3345 (3003 to 3691)	2600 (2299 to 2907)	685 (621 to 749)	60 (35 to 94)	0 (0 to 0)
Estonia	2040	2203 (1969 to 2440)	1714 (1503 to 1923)	449 (407 to 491)	40 (23 to 62)	0 (0 to 0)	3405 (3044 to 3772)	2650 (2323 to 2972)	694 (629 to 759)	61 (36 to 96)	0 (0 to 0)
Estonia	2041	2242 (2003 to 2487)	1747 (1535 to 1969)	455 (412 to 498)	40 (24 to 64)	0 (0 to 0)	3466 (3096 to 3845)	2701 (2373 to 3044)	703 (636 to 770)	63 (36 to 98)	0 (0 to 0)
Estonia	2042	2281 (2038 to 2537)	1780 (1560 to 2015)	460 (416 to 505)	41 (24 to 65)	0 (0 to 0)	3526 (3150 to 3922)	2751 (2411 to 3115)	711 (644 to 781)	64 (37 to 100)	0 (0 to 0)
Estonia	2043	2320 (2064 to 2585)	1812 (1581 to 2052)	466 (422 to 511)	42 (25 to 66)	0 (0 to 0)	3587 (3190 to 3995)	2802 (2444 to 3172)	720 (652 to 791)	65 (38 to 102)	0 (0 to 0)
Estonia	2044	2360 (2101 to 2630)	1846 (1611 to 2089)	471 (427 to 518)	43 (25 to 67)	0 (0 to 0)	3648 (3248 to 4066)	2853 (2490 to 3229)	728 (660 to 801)	66 (39 to 104)	0 (0 to 0)
Estonia	2045	2399 (2124 to 2670)	1879 (1633 to 2124)	477 (431 to 525)	44 (26 to 69)	0 (0 to 0)	3708 (3283 to 4128)	2904 (2524 to 3283)	737 (667 to 811)	67 (39 to 106)	0 (0 to 0)
Estonia	2046	2436 (2162 to 2723)	1910 (1654 to 2163)	482 (436 to 531)	44 (26 to 70)	0 (0 to 0)	3766 (3342 to 4210)	2953 (2557 to 3344)	745 (674 to 821)	69 (40 to 108)	0 (0 to 0)
Estonia	2047	2475 (2181 to 2774)	1943 (1684 to 2212)	487 (440 to 538)	45 (26 to 71)	0 (0 to 0)	3826 (3371 to 4288)	3003 (2603 to 3420)	753 (681 to 831)	70 (41 to 110)	0 (0 to 0)

Estonia	2048	2511 (2219 to 2823)	1974 (1701 to 2256)	492 (444 to 544)	46 (27 to 72)	0 (0 to 0)	3882 (3431 to 4365)	3051 (2630 to 3488)	760 (687 to 841)	71 (41 to 112)	0 (0 to 0)
Estonia	2049	2548 (2244 to 2875)	2005 (1720 to 2308)	496 (448 to 549)	47 (27 to 74)	0 (0 to 0)	3939 (3469 to 4444)	3100 (2659 to 3568)	767 (693 to 849)	72 (42 to 114)	0 (0 to 0)
Estonia	2050	2586 (2282 to 2933)	2038 (1752 to 2348)	501 (452 to 555)	48 (28 to 75)	0 (0 to 0)	3997 (3528 to 4534)	3150 (2708 to 3630)	774 (699 to 858)	74 (43 to 116)	0 (0 to 0)
eSwatini	1995	131 (108 to 155)	65 (52 to 78)	23 (16 to 32)	40 (23 to 62)	3 (3 to 3)	349 (289 to 414)	172 (139 to 208)	61 (43 to 85)	107 (62 to 166)	8 (8 to 8)
eSwatini	1996	139 (115 to 164)	70 (57 to 84)	24 (17 to 34)	43 (26 to 66)	2 (2 to 2)	370 (308 to 439)	187 (152 to 224)	65 (46 to 90)	114 (69 to 176)	4 (4 to 4)
eSwatini	1997	134 (113 to 156)	67 (55 to 79)	24 (17 to 34)	41 (25 to 61)	2 (2 to 2)	357 (301 to 418)	178 (147 to 211)	64 (45 to 90)	108 (68 to 164)	6 (6 to 6)
eSwatini	1998	138 (118 to 161)	67 (56 to 80)	25 (18 to 34)	40 (26 to 59)	6 (6 to 6)	369 (316 to 429)	180 (151 to 212)	67 (47 to 92)	107 (68 to 157)	15 (15 to 15)
eSwatini	1999	138 (118 to 162)	70 (59 to 83)	26 (18 to 36)	40 (26 to 59)	2 (2 to 2)	369 (314 to 432)	187 (157 to 221)	69 (48 to 95)	107 (69 to 159)	6 (6 to 6)
eSwatini	2000	131 (113 to 152)	67 (56 to 79)	25 (17 to 34)	36 (23 to 54)	3 (3 to 3)	350 (301 to 407)	180 (150 to 212)	66 (47 to 91)	96 (62 to 144)	8 (8 to 8)
eSwatini	2001	132 (113 to 154)	69 (58 to 81)	25 (18 to 34)	36 (22 to 52)	3 (3 to 3)	353 (303 to 411)	184 (155 to 216)	67 (48 to 92)	95 (60 to 140)	7 (7 to 7)
eSwatini	2002	137 (118 to 160)	72 (61 to 85)	26 (18 to 35)	36 (23 to 52)	4 (4 to 4)	367 (316 to 429)	193 (163 to 228)	69 (49 to 95)	96 (61 to 139)	10 (10 to 10)
eSwatini	2003	154 (134 to 176)	78 (67 to 93)	27 (19 to 37)	37 (23 to 53)	12 (12 to 12)	411 (357 to 471)	209 (178 to 248)	71 (52 to 98)	98 (63 to 142)	33 (33 to 33)
eSwatini	2004	174 (151 to 200)	95 (81 to 111)	29 (21 to 40)	42 (27 to 60)	8 (8 to 8)	465 (404 to 533)	255 (216 to 297)	78 (57 to 107)	112 (73 to 161)	20 (20 to 20)
eSwatini	2005	207 (184 to 233)	105 (90 to 121)	30 (22 to 41)	42 (28 to 62)	29 (29 to 29)	552 (491 to 623)	280 (239 to 324)	80 (58 to 110)	113 (74 to 164)	79 (79 to 79)

eSwatini	2006	203 (180 to 229)	110 (95 to 127)	30 (22 to 41)	42 (28 to 61)	21 (21 to 21)	542 (481 to 612)	294 (253 to 340)	81 (59 to 110)	112 (74 to 163)	55 (55 to 55)
eSwatini	2007	233 (209 to 264)	128 (110 to 147)	32 (23 to 43)	45 (30 to 66)	28 (28 to 28)	622 (558 to 705)	341 (295 to 393)	85 (61 to 116)	121 (81 to 177)	76 (76 to 76)
eSwatini	2008	248 (222 to 281)	141 (122 to 163)	32 (24 to 44)	47 (31 to 68)	27 (27 to 27)	662 (592 to 751)	378 (326 to 434)	87 (63 to 118)	125 (84 to 182)	73 (73 to 73)
eSwatini	2009	269 (241 to 301)	147 (127 to 169)	33 (23 to 44)	47 (31 to 68)	42 (42 to 42)	718 (643 to 805)	394 (340 to 452)	87 (62 to 117)	126 (83 to 181)	111 (111 to 111)
eSwatini	2010	290 (263 to 320)	142 (123 to 164)	32 (23 to 43)	46 (31 to 66)	69 (69 to 69)	774 (701 to 856)	380 (329 to 437)	86 (61 to 116)	123 (82 to 176)	185 (185 to 185)
eSwatini	2011	259 (235 to 286)	122 (107 to 141)	30 (22 to 41)	41 (27 to 59)	65 (65 to 65)	693 (627 to 764)	327 (285 to 376)	81 (58 to 108)	110 (72 to 156)	175 (175 to 175)
eSwatini	2012	247 (221 to 277)	125 (109 to 145)	31 (22 to 41)	43 (28 to 63)	48 (48 to 48)	659 (591 to 740)	335 (290 to 386)	82 (59 to 109)	114 (75 to 167)	128 (128 to 128)
eSwatini	2013	258 (233 to 288)	123 (106 to 142)	30 (22 to 40)	43 (27 to 63)	61 (61 to 61)	688 (621 to 770)	329 (282 to 379)	81 (58 to 108)	114 (73 to 168)	164 (164 to 164)
eSwatini	2014	238 (213 to 268)	121 (104 to 140)	30 (21 to 40)	43 (28 to 64)	44 (44 to 44)	636 (569 to 717)	323 (278 to 373)	80 (57 to 107)	115 (74 to 172)	118 (118 to 118)
eSwatini	2015	239 (213 to 271)	120 (103 to 139)	30 (21 to 40)	43 (27 to 64)	46 (46 to 46)	638 (569 to 723)	321 (275 to 371)	79 (57 to 106)	114 (71 to 171)	124 (124 to 124)
eSwatini	2016	285 (258 to 319)	124 (105 to 146)	30 (21 to 40)	43 (26 to 68)	89 (89 to 89)	762 (688 to 851)	330 (280 to 389)	79 (56 to 107)	115 (71 to 182)	237 (237 to 237)
eSwatini	2017	289 (249 to 340)	129 (108 to 153)	30 (21 to 40)	43 (26 to 69)	88 (88 to 88)	773 (666 to 909)	344 (288 to 410)	80 (56 to 108)	115 (68 to 184)	234 (234 to 234)
eSwatini	2018	282 (242 to 333)	124 (104 to 148)	30 (21 to 41)	44 (26 to 70)	85 (85 to 85)	755 (648 to 890)	331 (277 to 394)	80 (56 to 109)	117 (69 to 186)	226 (226 to 226)
eSwatini	2019	282 (242 to 331)	120 (100 to 143)	30 (21 to 41)	44 (26 to 70)	88 (87 to 88)	753 (647 to 885)	321 (268 to 381)	80 (57 to 109)	118 (70 to 188)	234 (232 to 235)
eSwatini	2020	283 (243 to 333)	118 (99 to 140)	30 (21 to 41)	44 (26 to 70)	91 (86 to 96)	755 (650 to 890)	316 (263 to 375)	80 (56 to 108)	117 (69 to 187)	242 (230 to 256)

eSwatini	2021	285 (246 to 333)	118 (98 to 141)	30 (21 to 41)	44 (26 to 70)	92 (87 to 100)	761 (658 to 889)	316 (263 to 376)	81 (57 to 110)	118 (70 to 188)	247 (232 to 267)
eSwatini	2022	290 (248 to 344)	121 (100 to 146)	31 (21 to 42)	45 (27 to 72)	94 (86 to 104)	775 (661 to 918)	323 (268 to 389)	82 (57 to 111)	120 (72 to 193)	250 (230 to 277)
eSwatini	2023	294 (250 to 350)	124 (101 to 149)	31 (22 to 42)	46 (27 to 74)	94 (85 to 106)	787 (668 to 935)	330 (270 to 397)	83 (58 to 112)	123 (73 to 197)	251 (227 to 282)
eSwatini	2024	299 (252 to 357)	126 (103 to 154)	31 (22 to 43)	47 (28 to 75)	94 (85 to 107)	798 (674 to 955)	337 (276 to 412)	84 (59 to 114)	125 (75 to 201)	252 (226 to 285)
eSwatini	2025	303 (258 to 362)	128 (105 to 157)	32 (22 to 43)	48 (28 to 76)	95 (84 to 108)	809 (690 to 967)	343 (280 to 420)	85 (59 to 115)	127 (76 to 204)	254 (225 to 289)
eSwatini	2026	307 (257 to 369)	131 (106 to 163)	32 (22 to 44)	49 (29 to 78)	96 (84 to 109)	821 (687 to 985)	349 (283 to 434)	86 (60 to 117)	130 (77 to 208)	256 (226 to 291)
eSwatini	2027	312 (262 to 376)	133 (107 to 167)	32 (23 to 44)	49 (29 to 79)	97 (85 to 113)	832 (699 to 1004)	356 (285 to 446)	87 (61 to 118)	132 (79 to 211)	258 (227 to 301)
eSwatini	2028	316 (263 to 382)	135 (107 to 169)	33 (23 to 45)	50 (30 to 80)	98 (85 to 115)	844 (703 to 1021)	361 (285 to 452)	88 (61 to 120)	134 (79 to 214)	261 (226 to 307)
eSwatini	2029	321 (269 to 390)	138 (108 to 173)	33 (23 to 45)	51 (30 to 82)	99 (86 to 116)	858 (719 to 1041)	369 (289 to 461)	89 (62 to 121)	137 (81 to 218)	264 (229 to 309)
eSwatini	2030	327 (270 to 399)	141 (110 to 180)	34 (24 to 46)	52 (31 to 84)	100 (85 to 119)	873 (722 to 1067)	377 (294 to 480)	90 (63 to 123)	140 (82 to 223)	266 (228 to 319)
eSwatini	2031	333 (275 to 405)	145 (112 to 184)	34 (24 to 47)	54 (32 to 86)	101 (85 to 120)	890 (735 to 1081)	387 (300 to 491)	91 (64 to 124)	143 (84 to 229)	269 (228 to 320)
eSwatini	2032	339 (278 to 414)	149 (114 to 190)	35 (24 to 47)	55 (32 to 88)	101 (85 to 122)	907 (743 to 1105)	397 (304 to 507)	93 (65 to 126)	147 (86 to 235)	271 (228 to 327)
eSwatini	2033	347 (283 to 425)	153 (117 to 195)	35 (25 to 48)	56 (33 to 90)	103 (85 to 125)	926 (756 to 1135)	407 (312 to 521)	94 (66 to 128)	150 (89 to 241)	274 (228 to 334)
eSwatini	2034	353 (286 to 437)	157 (120 to 201)	36 (25 to 49)	58 (34 to 92)	103 (85 to 127)	944 (765 to 1166)	418 (320 to 537)	96 (67 to 131)	154 (91 to 247)	276 (227 to 340)
eSwatini	2035	360 (294 to 445)	161 (123 to 208)	36 (25 to 50)	59 (35 to 95)	104 (86 to 129)	963 (784 to 1188)	429 (328 to 557)	97 (68 to 133)	158 (93 to 253)	279 (229 to 344)

eSwatini	2036	368 (294 to 457)	165 (126 to 214)	37 (26 to 50)	60 (36 to 97)	105 (86 to 133)	982 (784 to 1220)	440 (336 to 571)	99 (69 to 135)	161 (95 to 259)	282 (230 to 354)
eSwatini	2037	375 (303 to 466)	169 (128 to 219)	38 (26 to 51)	62 (37 to 99)	107 (85 to 132)	1001 (809 to 1245)	451 (341 to 585)	100 (70 to 137)	165 (98 to 265)	285 (228 to 353)
eSwatini	2038	382 (307 to 479)	173 (130 to 226)	38 (27 to 52)	63 (37 to 102)	108 (85 to 139)	1021 (820 to 1279)	462 (348 to 605)	102 (71 to 139)	169 (100 to 272)	288 (226 to 372)
eSwatini	2039	390 (312 to 492)	178 (133 to 235)	39 (27 to 53)	65 (38 to 104)	109 (86 to 140)	1041 (833 to 1315)	474 (356 to 627)	103 (72 to 142)	173 (103 to 278)	290 (230 to 373)
eSwatini	2040	398 (315 to 502)	182 (136 to 239)	39 (27 to 54)	66 (39 to 107)	110 (87 to 143)	1063 (841 to 1342)	486 (362 to 638)	105 (73 to 144)	177 (105 to 285)	294 (231 to 382)
eSwatini	2041	406 (323 to 512)	187 (139 to 245)	40 (28 to 55)	68 (40 to 109)	111 (85 to 142)	1085 (863 to 1368)	499 (371 to 656)	107 (74 to 146)	181 (107 to 291)	298 (227 to 380)
eSwatini	2042	414 (327 to 523)	191 (141 to 254)	40 (28 to 55)	69 (41 to 112)	113 (86 to 147)	1106 (873 to 1398)	511 (376 to 678)	108 (75 to 148)	186 (109 to 298)	301 (229 to 393)
eSwatini	2043	422 (330 to 542)	196 (145 to 260)	41 (29 to 56)	71 (42 to 114)	114 (85 to 152)	1128 (883 to 1447)	524 (387 to 694)	110 (76 to 150)	190 (112 to 305)	305 (228 to 406)
eSwatini	2044	430 (338 to 553)	200 (148 to 265)	42 (29 to 57)	72 (43 to 117)	116 (85 to 158)	1148 (902 to 1476)	535 (395 to 709)	111 (77 to 152)	194 (115 to 312)	309 (227 to 421)
eSwatini	2045	438 (341 to 559)	205 (149 to 272)	42 (29 to 58)	74 (44 to 120)	117 (86 to 156)	1171 (911 to 1494)	547 (397 to 727)	113 (78 to 155)	198 (118 to 319)	313 (229 to 416)

eSwatini	2046	447 (345 to 577)	209 (151 to 277)	43 (30 to 59)	76 (45 to 122)	119 (86 to 159)	1193 (921 to 1542)	560 (403 to 740)	114 (79 to 157)	202 (120 to 327)	318 (229 to 425)
eSwatini	2047	455 (351 to 583)	214 (154 to 288)	43 (30 to 59)	77 (46 to 125)	121 (87 to 164)	1216 (939 to 1558)	572 (411 to 768)	115 (80 to 159)	206 (122 to 334)	323 (233 to 438)
eSwatini	2048	464 (357 to 601)	219 (157 to 293)	44 (30 to 60)	79 (47 to 128)	123 (87 to 165)	1238 (954 to 1605)	584 (420 to 783)	117 (81 to 161)	210 (125 to 341)	328 (231 to 442)
eSwatini	2049	472 (364 to 610)	223 (160 to 305)	44 (31 to 61)	80 (48 to 130)	125 (86 to 173)	1261 (971 to 1629)	596 (428 to 814)	118 (82 to 163)	214 (127 to 348)	333 (228 to 463)
eSwatini	2050	480 (365 to 619)	227 (161 to 308)	45 (31 to 62)	81 (48 to 133)	127 (87 to 177)	1283 (974 to 1655)	606 (430 to 822)	119 (83 to 165)	218 (129 to 354)	340 (233 to 473)
Ethiopia	1995	10 (8 to 12)	4 (3 to 6)	4 (3 to 6)	1 (0 to 1)	1 (1 to 1)	27 (22 to 32)	11 (8 to 15)	10 (7 to 15)	2 (1 to 3)	3 (3 to 3)
Ethiopia	1996	11 (9 to 13)	5 (3 to 6)	4 (3 to 6)	1 (0 to 1)	1 (1 to 1)	29 (24 to 35)	12 (9 to 16)	11 (8 to 15)	2 (1 to 4)	4 (4 to 4)
Ethiopia	1997	11 (9 to 13)	4 (3 to 6)	4 (3 to 6)	1 (0 to 1)	1 (1 to 1)	28 (23 to 33)	12 (9 to 16)	11 (8 to 16)	2 (1 to 4)	3 (3 to 3)
Ethiopia	1998	10 (9 to 12)	4 (3 to 6)	4 (3 to 6)	1 (0 to 1)	1 (1 to 1)	27 (22 to 33)	12 (9 to 15)	11 (8 to 16)	2 (1 to 4)	2 (2 to 2)
Ethiopia	1999	12 (10 to 14)	5 (4 to 6)	5 (3 to 6)	1 (0 to 2)	2 (2 to 2)	31 (26 to 37)	13 (10 to 17)	12 (8 to 17)	2 (1 to 4)	4 (4 to 4)
Ethiopia	2000	12 (10 to 14)	5 (4 to 7)	5 (3 to 6)	1 (0 to 2)	1 (1 to 1)	31 (26 to 37)	13 (10 to 17)	12 (8 to 17)	2 (1 to 4)	4 (4 to 4)
Ethiopia	2001	12 (10 to 14)	5 (4 to 7)	5 (3 to 6)	1 (0 to 1)	2 (2 to 2)	32 (27 to 38)	13 (10 to 17)	12 (8 to 17)	2 (1 to 4)	4 (4 to 4)
Ethiopia	2002	13 (11 to 15)	5 (4 to 7)	5 (3 to 7)	1 (0 to 1)	2 (2 to 2)	34 (28 to 40)	14 (11 to 18)	13 (8 to 18)	2 (1 to 4)	5 (5 to 5)

Ethiopia	2003	14 (12 to 16)	5 (4 to 7)	5 (3 to 7)	1 (0 to 1)	3 (3 to 3)	36 (31 to 43)	13 (10 to 17)	13 (8 to 18)	2 (1 to 3)	8 (8 to 8)
Ethiopia	2004	13 (11 to 15)	4 (3 to 6)	5 (3 to 7)	1 (0 to 1)	3 (3 to 3)	34 (29 to 40)	12 (9 to 15)	13 (8 to 18)	2 (1 to 3)	8 (8 to 8)
Ethiopia	2005	14 (12 to 17)	5 (3 to 6)	5 (3 to 7)	1 (0 to 1)	4 (4 to 4)	37 (32 to 44)	12 (9 to 16)	14 (9 to 19)	2 (1 to 3)	10 (10 to 10)
Ethiopia	2006	17 (14 to 19)	4 (3 to 5)	6 (4 to 8)	1 (0 to 1)	6 (6 to 6)	44 (38 to 51)	11 (8 to 14)	15 (11 to 21)	2 (1 to 3)	15 (15 to 15)
Ethiopia	2007	19 (16 to 22)	4 (3 to 5)	7 (5 to 9)	1 (0 to 1)	7 (7 to 7)	50 (43 to 57)	11 (8 to 14)	18 (12 to 24)	2 (1 to 4)	20 (20 to 20)
Ethiopia	2008	19 (16 to 22)	3 (3 to 4)	7 (5 to 10)	1 (0 to 1)	8 (8 to 8)	50 (43 to 58)	9 (7 to 12)	19 (13 to 26)	2 (1 to 4)	20 (20 to 20)
Ethiopia	2009	20 (17 to 24)	4 (3 to 5)	8 (6 to 11)	1 (0 to 2)	8 (8 to 8)	54 (46 to 62)	9 (7 to 12)	21 (15 to 29)	3 (1 to 5)	21 (21 to 21)
Ethiopia	2010	24 (20 to 27)	4 (3 to 5)	9 (6 to 13)	1 (1 to 3)	9 (9 to 9)	62 (54 to 72)	10 (8 to 13)	24 (17 to 34)	4 (2 to 7)	24 (24 to 24)
Ethiopia	2011	26 (22 to 30)	4 (3 to 5)	10 (7 to 13)	2 (1 to 3)	10 (10 to 10)	68 (58 to 79)	10 (7 to 13)	25 (17 to 36)	4 (2 to 8)	28 (28 to 28)
Ethiopia	2012	25 (22 to 30)	4 (3 to 6)	10 (7 to 14)	2 (1 to 4)	9 (9 to 9)	67 (58 to 79)	12 (9 to 15)	26 (18 to 36)	5 (2 to 10)	24 (24 to 24)
Ethiopia	2013	30 (26 to 35)	5 (4 to 7)	10 (7 to 14)	3 (1 to 5)	12 (12 to 12)	79 (69 to 91)	14 (10 to 18)	27 (19 to 38)	7 (3 to 13)	31 (31 to 31)
Ethiopia	2014	28 (24 to 33)	5 (4 to 7)	10 (7 to 14)	4 (2 to 7)	9 (9 to 9)	73 (63 to 87)	14 (11 to 19)	26 (18 to 36)	9 (5 to 18)	23 (23 to 23)
Ethiopia	2015	28 (24 to 34)	6 (4 to 8)	10 (7 to 14)	4 (2 to 8)	8 (8 to 8)	75 (63 to 89)	16 (12 to 21)	26 (18 to 36)	11 (6 to 22)	21 (21 to 21)
Ethiopia	2016	29 (25 to 35)	7 (5 to 9)	10 (7 to 14)	5 (2 to 9)	8 (8 to 8)	78 (66 to 92)	17 (13 to 23)	26 (18 to 36)	13 (6 to 24)	21 (21 to 21)
Ethiopia	2017	31 (25 to 38)	7 (5 to 9)	10 (7 to 14)	5 (3 to 10)	8 (8 to 8)	81 (67 to 101)	18 (14 to 24)	26 (18 to 37)	14 (7 to 26)	22 (22 to 22)

Ethiopia	2018	30 (25 to 38)	6 (5 to 8)	10 (7 to 14)	6 (3 to 10)	8 (8 to 8)	80 (65 to 101)	17 (13 to 22)	27 (19 to 38)	15 (7 to 27)	21 (21 to 21)
Ethiopia	2019	31 (25 to 39)	6 (5 to 8)	11 (7 to 15)	6 (3 to 11)	8 (8 to 8)	82 (67 to 104)	17 (13 to 22)	29 (20 to 40)	15 (7 to 29)	21 (20 to 21)
Ethiopia	2020	32 (26 to 41)	7 (5 to 9)	11 (8 to 16)	6 (3 to 12)	8 (7 to 8)	85 (69 to 109)	18 (13 to 23)	30 (21 to 42)	16 (8 to 30)	20 (19 to 21)
Ethiopia	2021	33 (27 to 42)	7 (5 to 9)	12 (8 to 16)	7 (3 to 12)	8 (7 to 8)	87 (70 to 111)	18 (14 to 24)	31 (22 to 43)	17 (8 to 32)	20 (19 to 22)
Ethiopia	2022	34 (27 to 43)	7 (5 to 9)	12 (8 to 17)	7 (3 to 13)	7 (7 to 8)	89 (72 to 114)	19 (14 to 25)	32 (22 to 45)	18 (9 to 33)	20 (18 to 22)
Ethiopia	2023	35 (28 to 45)	8 (6 to 10)	13 (9 to 18)	7 (3 to 13)	7 (7 to 8)	92 (73 to 119)	20 (15 to 26)	34 (23 to 47)	19 (9 to 35)	19 (17 to 22)
Ethiopia	2024	36 (29 to 46)	8 (6 to 10)	13 (9 to 18)	7 (4 to 14)	7 (6 to 8)	94 (76 to 122)	21 (15 to 27)	35 (24 to 49)	20 (9 to 37)	19 (17 to 21)
Ethiopia	2025	37 (29 to 48)	8 (6 to 11)	14 (10 to 19)	8 (4 to 15)	7 (6 to 8)	97 (78 to 127)	22 (16 to 29)	37 (25 to 51)	21 (10 to 38)	19 (16 to 21)
Ethiopia	2026	38 (30 to 49)	9 (6 to 12)	14 (10 to 20)	8 (4 to 15)	7 (6 to 8)	100 (79 to 130)	23 (16 to 30)	38 (26 to 53)	22 (10 to 40)	18 (16 to 21)
Ethiopia	2027	39 (31 to 51)	9 (6 to 12)	15 (10 to 21)	9 (4 to 16)	7 (6 to 8)	104 (82 to 135)	24 (17 to 32)	39 (27 to 55)	23 (11 to 42)	18 (16 to 21)
Ethiopia	2028	40 (32 to 52)	9 (7 to 13)	16 (11 to 21)	9 (4 to 17)	7 (6 to 8)	107 (84 to 138)	25 (18 to 33)	41 (28 to 57)	24 (11 to 44)	18 (15 to 21)
Ethiopia	2029	42 (33 to 54)	10 (7 to 13)	16 (11 to 22)	9 (4 to 17)	7 (6 to 8)	110 (86 to 144)	26 (19 to 35)	43 (29 to 59)	25 (12 to 46)	17 (15 to 21)
Ethiopia	2030	43 (34 to 57)	10 (7 to 14)	17 (12 to 23)	10 (5 to 18)	6 (5 to 8)	114 (89 to 149)	27 (19 to 36)	44 (31 to 61)	26 (12 to 48)	17 (14 to 21)
Ethiopia	2031	45 (34 to 58)	11 (8 to 15)	17 (12 to 24)	10 (5 to 19)	6 (5 to 8)	118 (91 to 154)	28 (20 to 38)	46 (32 to 64)	27 (13 to 50)	17 (14 to 20)
Ethiopia	2032	46 (36 to 61)	11 (8 to 15)	18 (12 to 25)	11 (5 to 20)	6 (5 to 8)	122 (95 to 161)	30 (21 to 41)	48 (33 to 66)	28 (14 to 53)	17 (14 to 20)

Ethiopia	2033	48 (37 to 63)	12 (8 to 16)	19 (13 to 26)	11 (5 to 21)	6 (5 to 8)	126 (97 to 167)	31 (21 to 42)	50 (34 to 69)	29 (14 to 55)	16 (13 to 20)
Ethiopia	2034	50 (38 to 66)	12 (8 to 17)	20 (13 to 27)	12 (6 to 22)	6 (5 to 8)	131 (101 to 175)	32 (22 to 45)	52 (36 to 71)	31 (15 to 57)	16 (13 to 20)
Ethiopia	2035	51 (39 to 69)	13 (9 to 18)	20 (14 to 28)	12 (6 to 23)	6 (5 to 8)	136 (103 to 182)	34 (23 to 47)	54 (37 to 74)	32 (15 to 60)	16 (12 to 20)
Ethiopia	2036	53 (41 to 71)	13 (9 to 19)	21 (15 to 29)	13 (6 to 24)	6 (5 to 8)	141 (107 to 188)	35 (24 to 49)	56 (38 to 77)	34 (16 to 62)	16 (12 to 20)
Ethiopia	2037	55 (42 to 75)	14 (9 to 20)	22 (15 to 30)	13 (6 to 25)	6 (4 to 7)	146 (111 to 197)	37 (25 to 52)	58 (40 to 80)	35 (17 to 65)	16 (12 to 20)
Ethiopia	2038	57 (44 to 77)	15 (10 to 21)	23 (16 to 32)	14 (7 to 26)	6 (4 to 8)	152 (115 to 204)	39 (26 to 54)	61 (42 to 84)	37 (18 to 68)	15 (12 to 20)
Ethiopia	2039	60 (45 to 81)	15 (10 to 22)	24 (16 to 33)	15 (7 to 27)	6 (4 to 8)	157 (119 to 214)	40 (27 to 58)	63 (43 to 87)	38 (18 to 71)	15 (11 to 20)
Ethiopia	2040	62 (47 to 84)	16 (11 to 23)	25 (17 to 34)	15 (7 to 28)	6 (4 to 8)	163 (124 to 222)	42 (28 to 60)	66 (45 to 91)	40 (19 to 74)	15 (11 to 20)
Ethiopia	2041	64 (48 to 88)	17 (11 to 24)	26 (18 to 36)	16 (8 to 29)	6 (4 to 8)	170 (127 to 232)	44 (29 to 63)	68 (47 to 95)	42 (20 to 77)	15 (10 to 20)
Ethiopia	2042	67 (50 to 92)	18 (11 to 25)	27 (18 to 37)	17 (8 to 31)	6 (4 to 8)	176 (131 to 243)	46 (30 to 67)	71 (49 to 98)	44 (21 to 81)	15 (10 to 20)
Ethiopia	2043	69 (51 to 96)	18 (12 to 27)	28 (19 to 39)	17 (8 to 32)	6 (4 to 8)	183 (135 to 253)	48 (32 to 70)	74 (51 to 102)	46 (22 to 84)	15 (10 to 20)
Ethiopia	2044	72 (53 to 99)	19 (12 to 28)	29 (20 to 40)	18 (9 to 33)	6 (4 to 8)	190 (140 to 262)	50 (32 to 74)	77 (53 to 107)	48 (23 to 88)	15 (9 to 20)
Ethiopia	2045	75 (55 to 103)	20 (13 to 29)	30 (21 to 42)	19 (9 to 35)	6 (4 to 8)	197 (145 to 273)	53 (34 to 78)	80 (55 to 111)	50 (24 to 92)	15 (9 to 20)
Ethiopia	2046	78 (57 to 107)	21 (13 to 31)	32 (22 to 44)	20 (9 to 36)	5 (3 to 8)	205 (149 to 283)	55 (35 to 82)	84 (57 to 115)	52 (25 to 96)	14 (9 to 20)
Ethiopia	2047	81 (59 to 112)	22 (14 to 33)	33 (23 to 45)	21 (10 to 38)	5 (3 to 8)	213 (155 to 295)	58 (37 to 86)	87 (59 to 120)	54 (26 to 100)	14 (9 to 20)

Ethiopia	2048	84 (60 to 118)	23 (15 to 34)	34 (23 to 47)	21 (10 to 40)	5 (3 to 8)	221 (159 to 310)	60 (39 to 90)	90 (62 to 125)	57 (27 to 104)	14 (9 to 20)
Ethiopia	2049	87 (62 to 122)	24 (15 to 36)	36 (24 to 49)	22 (11 to 41)	5 (3 to 8)	230 (165 to 322)	63 (40 to 94)	94 (64 to 130)	59 (28 to 109)	14 (8 to 20)
Ethiopia	2050	91 (65 to 127)	25 (15 to 38)	37 (25 to 51)	23 (11 to 43)	5 (3 to 8)	239 (171 to 336)	66 (41 to 99)	98 (67 to 135)	62 (29 to 114)	14 (8 to 20)
Federated States of Micronesia	1995	100 (84 to 120)	83 (67 to 102)	16 (11 to 21)	0 (0 to 0)	2 (2 to 2)	96 (81 to 115)	79 (65 to 98)	15 (11 to 21)	0 (0 to 0)	2 (2 to 2)
Federated States of Micronesia	1996	94 (80 to 113)	79 (65 to 97)	15 (11 to 21)	0 (0 to 0)	0 (0 to 0)	91 (77 to 108)	76 (63 to 93)	15 (10 to 20)	0 (0 to 0)	0 (0 to 0)
Federated States of Micronesia	1997	88 (75 to 103)	72 (59 to 87)	15 (10 to 20)	0 (0 to 0)	1 (1 to 1)	84 (72 to 99)	69 (57 to 83)	14 (10 to 19)	0 (0 to 0)	1 (1 to 1)
Federated States of Micronesia	1998	92 (79 to 110)	77 (64 to 92)	15 (10 to 21)	0 (0 to 0)	1 (1 to 1)	89 (76 to 105)	74 (61 to 88)	14 (10 to 20)	0 (0 to 0)	1 (1 to 1)
Federated States of Micronesia	1999	183 (170 to 199)	74 (62 to 89)	15 (10 to 20)	0 (0 to 0)	94 (94 to 94)	176 (163 to 191)	71 (60 to 86)	14 (10 to 19)	0 (0 to 0)	90 (90 to 90)
Federated States of Micronesia	2000	84 (72 to 98)	68 (57 to 82)	14 (10 to 19)	0 (0 to 0)	2 (2 to 2)	81 (69 to 94)	65 (55 to 78)	14 (10 to 18)	0 (0 to 0)	2 (2 to 2)
Federated States of Micronesia	2001	84 (72 to 98)	67 (56 to 80)	14 (10 to 19)	0 (0 to 0)	3 (3 to 3)	81 (70 to 94)	64 (54 to 77)	13 (9 to 18)	0 (0 to 0)	3 (3 to 3)
Federated States of Micronesia	2002	80 (69 to 94)	64 (54 to 76)	13 (10 to 18)	0 (0 to 0)	3 (3 to 3)	77 (66 to 90)	62 (52 to 73)	13 (9 to 17)	0 (0 to 0)	3 (3 to 3)

Federated States of Micronesia	2003	142 (129 to 156)	75 (62 to 89)	14 (10 to 19)	0 (0 to 0)	53 (53 to 53)	136 (123 to 150)	72 (60 to 85)	13 (10 to 18)	0 (0 to 0)	51 (51 to 51)
Federated States of Micronesia	2004	313 (300 to 327)	74 (62 to 88)	13 (10 to 18)	0 (0 to 0)	225 (225 to 225)	300 (288 to 314)	71 (59 to 85)	13 (9 to 18)	0 (0 to 0)	216 (216 to 216)
Federated States of Micronesia	2005	339 (326 to 354)	80 (68 to 94)	13 (10 to 19)	0 (0 to 0)	246 (246 to 246)	326 (313 to 340)	77 (65 to 91)	13 (9 to 18)	0 (0 to 0)	236 (236 to 236)
Federated States of Micronesia	2006	305 (291 to 320)	85 (72 to 100)	13 (9 to 19)	0 (0 to 0)	207 (207 to 207)	293 (279 to 307)	81 (69 to 96)	13 (9 to 18)	0 (0 to 0)	199 (199 to 199)
Federated States of Micronesia	2007	305 (292 to 320)	84 (71 to 98)	13 (9 to 18)	0 (0 to 0)	209 (209 to 209)	293 (280 to 308)	80 (68 to 94)	12 (9 to 17)	0 (0 to 0)	200 (200 to 200)
Federated States of Micronesia	2008	324 (309 to 340)	85 (71 to 100)	12 (9 to 18)	0 (0 to 0)	227 (227 to 227)	311 (297 to 326)	81 (68 to 96)	12 (8 to 17)	0 (0 to 0)	218 (218 to 218)
Federated States of Micronesia	2009	381 (367 to 397)	86 (72 to 102)	12 (8 to 17)	0 (0 to 0)	283 (283 to 283)	366 (352 to 381)	82 (69 to 98)	12 (8 to 16)	0 (0 to 0)	272 (272 to 272)
Federated States of Micronesia	2010	128 (113 to 146)	92 (77 to 108)	12 (8 to 16)	0 (0 to 0)	24 (24 to 24)	123 (108 to 140)	88 (74 to 104)	11 (8 to 16)	0 (0 to 0)	23 (23 to 23)
Federated States of Micronesia	2011	138 (122 to 156)	99 (84 to 116)	12 (8 to 16)	0 (0 to 0)	27 (27 to 27)	132 (117 to 149)	95 (81 to 112)	11 (8 to 16)	0 (0 to 0)	26 (26 to 26)
Federated States of Micronesia	2012	364 (348 to 382)	99 (85 to 117)	11 (8 to 16)	0 (0 to 0)	253 (253 to 253)	349 (334 to 366)	95 (81 to 112)	11 (8 to 15)	0 (0 to 0)	243 (243 to 243)

Federated States of Micronesia	2013	126 (110 to 144)	98 (83 to 115)	11 (8 to 15)	0 (0 to 0)	17 (17 to 17)	121 (106 to 138)	94 (79 to 111)	11 (7 to 14)	0 (0 to 0)	16 (16 to 16)
Federated States of Micronesia	2014	407 (392 to 425)	98 (83 to 116)	11 (8 to 14)	0 (0 to 0)	299 (299 to 299)	391 (376 to 408)	94 (79 to 111)	10 (7 to 14)	0 (0 to 0)	287 (287 to 287)
Federated States of Micronesia	2015	212 (195 to 233)	105 (89 to 126)	11 (8 to 15)	0 (0 to 0)	96 (96 to 96)	204 (188 to 224)	101 (85 to 121)	10 (7 to 14)	0 (0 to 0)	92 (92 to 92)
Federated States of Micronesia	2016	138 (119 to 162)	117 (98 to 140)	11 (8 to 15)	0 (0 to 0)	11 (11 to 11)	133 (114 to 155)	112 (94 to 134)	11 (7 to 15)	0 (0 to 0)	10 (10 to 10)
Federated States of Micronesia	2017	141 (119 to 166)	119 (98 to 144)	11 (8 to 15)	0 (0 to 0)	11 (11 to 11)	136 (115 to 160)	115 (94 to 138)	11 (7 to 15)	0 (0 to 0)	11 (11 to 11)
Federated States of Micronesia	2018	150 (126 to 177)	128 (104 to 155)	11 (8 to 15)	0 (0 to 0)	11 (11 to 11)	144 (121 to 170)	123 (100 to 149)	11 (7 to 15)	0 (0 to 0)	10 (10 to 10)
Federated States of Micronesia	2019	153 (128 to 181)	130 (107 to 158)	11 (8 to 16)	0 (0 to 0)	11 (11 to 11)	147 (123 to 174)	125 (102 to 152)	11 (7 to 15)	0 (0 to 0)	11 (11 to 11)
Federated States of Micronesia	2020	157 (131 to 187)	134 (109 to 163)	11 (8 to 16)	0 (0 to 0)	11 (11 to 12)	151 (126 to 180)	129 (105 to 157)	11 (7 to 15)	0 (0 to 0)	11 (10 to 11)
Federated States of Micronesia	2021	164 (137 to 196)	141 (115 to 171)	11 (8 to 16)	0 (0 to 0)	11 (11 to 12)	157 (132 to 188)	135 (110 to 164)	11 (8 to 15)	0 (0 to 0)	11 (10 to 12)
Federated States of Micronesia	2022	171 (143 to 203)	148 (120 to 181)	12 (8 to 16)	0 (0 to 0)	12 (11 to 13)	164 (137 to 195)	142 (116 to 173)	11 (8 to 16)	0 (0 to 0)	11 (10 to 12)

Federated States of Micronesia	2023	178 (149 to 213)	155 (126 to 189)	12 (8 to 16)	0 (0 to 0)	12 (11 to 13)	171 (143 to 204)	149 (121 to 181)	11 (8 to 16)	0 (0 to 0)	11 (10 to 13)
Federated States of Micronesia	2024	186 (156 to 222)	162 (132 to 198)	12 (8 to 16)	0 (0 to 0)	12 (11 to 13)	178 (150 to 213)	156 (127 to 190)	11 (8 to 16)	0 (0 to 0)	11 (10 to 13)
Federated States of Micronesia	2025	193 (161 to 232)	169 (138 to 208)	12 (8 to 17)	0 (0 to 0)	12 (11 to 14)	185 (155 to 222)	162 (132 to 199)	11 (8 to 16)	0 (0 to 0)	11 (10 to 13)
Federated States of Micronesia	2026	200 (167 to 240)	176 (143 to 216)	12 (8 to 17)	0 (0 to 0)	12 (11 to 14)	192 (160 to 230)	169 (138 to 207)	11 (8 to 16)	0 (0 to 0)	12 (10 to 13)
Federated States of Micronesia	2027	207 (172 to 248)	183 (148 to 224)	12 (8 to 17)	0 (0 to 0)	12 (11 to 14)	198 (165 to 238)	175 (142 to 215)	12 (8 to 16)	0 (0 to 0)	12 (10 to 14)
Federated States of Micronesia	2028	213 (176 to 256)	188 (152 to 231)	12 (8 to 17)	0 (0 to 0)	12 (11 to 14)	204 (169 to 245)	181 (146 to 222)	12 (8 to 16)	0 (0 to 0)	12 (10 to 14)
Federated States of Micronesia	2029	218 (181 to 264)	194 (156 to 239)	12 (8 to 17)	0 (0 to 0)	12 (11 to 15)	210 (174 to 253)	186 (150 to 229)	12 (8 to 16)	0 (0 to 0)	12 (10 to 14)
Federated States of Micronesia	2030	224 (185 to 270)	199 (161 to 245)	12 (8 to 17)	0 (0 to 0)	13 (11 to 15)	215 (178 to 259)	191 (154 to 235)	12 (8 to 16)	0 (0 to 0)	12 (10 to 14)
Federated States of Micronesia	2031	229 (190 to 278)	204 (164 to 252)	12 (8 to 17)	0 (0 to 0)	13 (11 to 15)	220 (182 to 267)	196 (157 to 242)	12 (8 to 16)	0 (0 to 0)	12 (10 to 14)
Federated States of Micronesia	2032	234 (192 to 285)	209 (167 to 259)	12 (8 to 17)	0 (0 to 0)	13 (11 to 16)	224 (185 to 273)	200 (160 to 249)	12 (8 to 16)	0 (0 to 0)	12 (10 to 15)

Federated States of Micronesia	2033	238 (195 to 290)	213 (171 to 264)	12 (8 to 17)	0 (0 to 0)	13 (11 to 16)	229 (187 to 278)	205 (164 to 253)	12 (8 to 16)	0 (0 to 0)	12 (11 to 15)
Federated States of Micronesia	2034	243 (201 to 296)	217 (174 to 270)	12 (8 to 17)	0 (0 to 0)	13 (11 to 16)	233 (192 to 284)	209 (167 to 259)	12 (8 to 16)	0 (0 to 0)	13 (11 to 15)
Federated States of Micronesia	2035	247 (203 to 301)	222 (178 to 276)	12 (8 to 17)	0 (0 to 0)	13 (11 to 16)	237 (195 to 289)	213 (171 to 265)	12 (8 to 16)	0 (0 to 0)	13 (11 to 16)
Federated States of Micronesia	2036	252 (206 to 307)	226 (181 to 281)	12 (8 to 17)	0 (0 to 0)	14 (11 to 17)	241 (198 to 294)	217 (174 to 270)	12 (8 to 16)	0 (0 to 0)	13 (11 to 16)
Federated States of Micronesia	2037	255 (208 to 312)	229 (183 to 285)	12 (8 to 17)	0 (0 to 0)	14 (11 to 17)	245 (200 to 300)	220 (176 to 274)	12 (8 to 16)	0 (0 to 0)	13 (11 to 16)
Federated States of Micronesia	2038	259 (212 to 317)	233 (186 to 290)	12 (8 to 17)	0 (0 to 0)	14 (11 to 17)	249 (204 to 304)	224 (178 to 278)	12 (8 to 16)	0 (0 to 0)	13 (11 to 17)
Federated States of Micronesia	2039	263 (214 to 321)	236 (188 to 295)	12 (9 to 17)	0 (0 to 0)	14 (12 to 18)	252 (206 to 308)	227 (181 to 284)	12 (8 to 16)	0 (0 to 0)	14 (11 to 17)
Federated States of Micronesia	2040	267 (217 to 328)	240 (191 to 300)	12 (9 to 17)	0 (0 to 0)	14 (12 to 18)	256 (208 to 315)	230 (183 to 288)	12 (8 to 16)	0 (0 to 0)	14 (11 to 17)
Federated States of Micronesia	2041	270 (220 to 333)	243 (194 to 304)	12 (9 to 17)	0 (0 to 0)	15 (12 to 18)	260 (211 to 320)	234 (186 to 292)	12 (8 to 16)	0 (0 to 0)	14 (11 to 17)
Federated States of Micronesia	2042	274 (222 to 336)	247 (196 to 310)	12 (9 to 17)	0 (0 to 0)	15 (12 to 19)	263 (213 to 323)	237 (188 to 297)	12 (8 to 16)	0 (0 to 0)	14 (12 to 18)

Federated States of Micronesia	2043	278 (225 to 340)	250 (198 to 313)	13 (9 to 17)	0 (0 to 0)	15 (12 to 19)	267 (216 to 326)	240 (190 to 300)	12 (8 to 17)	0 (0 to 0)	14 (12 to 19)
Federated States of Micronesia	2044	281 (227 to 348)	254 (200 to 318)	13 (9 to 17)	0 (0 to 0)	15 (12 to 20)	270 (218 to 334)	243 (192 to 305)	12 (8 to 17)	0 (0 to 0)	15 (12 to 19)
Federated States of Micronesia	2045	285 (229 to 353)	257 (202 to 323)	13 (9 to 17)	0 (0 to 0)	15 (12 to 20)	274 (220 to 338)	247 (194 to 310)	12 (8 to 17)	0 (0 to 0)	15 (12 to 19)
Federated States of Micronesia	2046	289 (233 to 356)	260 (205 to 327)	13 (9 to 17)	0 (0 to 0)	16 (12 to 20)	277 (223 to 341)	250 (197 to 314)	12 (8 to 17)	0 (0 to 0)	15 (12 to 19)
Federated States of Micronesia	2047	292 (235 to 361)	264 (208 to 333)	13 (9 to 18)	0 (0 to 0)	16 (13 to 21)	281 (225 to 347)	253 (199 to 319)	12 (8 to 17)	0 (0 to 0)	15 (12 to 20)
Federated States of Micronesia	2048	296 (240 to 366)	267 (211 to 337)	13 (9 to 18)	0 (0 to 0)	16 (13 to 21)	284 (230 to 351)	256 (202 to 323)	12 (9 to 17)	0 (0 to 0)	15 (12 to 20)
Federated States of Micronesia	2049	300 (243 to 370)	271 (213 to 342)	13 (9 to 18)	0 (0 to 0)	16 (13 to 21)	288 (233 to 355)	260 (205 to 328)	12 (9 to 17)	0 (0 to 0)	16 (12 to 21)
Federated States of Micronesia	2050	304 (247 to 374)	274 (216 to 346)	13 (9 to 18)	0 (0 to 0)	16 (13 to 22)	292 (237 to 359)	263 (207 to 332)	13 (9 to 17)	0 (0 to 0)	16 (13 to 21)
Fiji	1995	115 (98 to 135)	89 (73 to 106)	16 (11 to 21)	10 (5 to 17)	1 (1 to 1)	208 (176 to 243)	159 (131 to 191)	28 (20 to 39)	18 (9 to 31)	2 (2 to 2)
Fiji	1996	124 (105 to 144)	97 (80 to 114)	16 (12 to 22)	11 (5 to 18)	0 (0 to 0)	222 (190 to 259)	174 (144 to 206)	29 (21 to 39)	19 (10 to 33)	0 (0 to 0)
Fiji	1997	127 (109 to 147)	100 (83 to 117)	17 (12 to 22)	10 (5 to 17)	1 (1 to 1)	228 (197 to 264)	180 (150 to 210)	30 (21 to 40)	18 (9 to 31)	1 (1 to 1)

Fiji	1998	133 (116 to 150)	97 (81 to 113)	16 (11 to 22)	9 (4 to 15)	11 (11 to 11)	239 (209 to 270)	174 (146 to 203)	29 (21 to 40)	15 (8 to 27)	21 (21 to 21)
Fiji	1999	137 (121 to 154)	97 (82 to 113)	17 (12 to 23)	7 (3 to 12)	17 (17 to 17)	247 (218 to 277)	175 (148 to 203)	30 (21 to 41)	12 (6 to 21)	30 (30 to 30)
Fiji	2000	137 (121 to 155)	101 (86 to 117)	17 (12 to 23)	7 (4 to 12)	13 (13 to 13)	247 (218 to 279)	182 (155 to 211)	30 (21 to 42)	13 (7 to 22)	23 (23 to 23)
Fiji	2001	133 (117 to 151)	102 (88 to 119)	18 (12 to 24)	7 (4 to 13)	6 (6 to 6)	240 (210 to 271)	184 (158 to 214)	32 (22 to 44)	13 (7 to 23)	11 (11 to 11)
Fiji	2002	141 (124 to 160)	107 (92 to 125)	18 (13 to 25)	8 (4 to 14)	8 (8 to 8)	253 (224 to 288)	192 (165 to 225)	33 (23 to 45)	14 (7 to 25)	14 (14 to 14)
Fiji	2003	153 (135 to 173)	106 (90 to 124)	19 (13 to 26)	8 (4 to 13)	20 (20 to 20)	275 (244 to 311)	190 (163 to 224)	34 (24 to 46)	14 (8 to 24)	36 (36 to 36)
Fiji	2004	148 (129 to 168)	108 (91 to 126)	20 (14 to 28)	9 (5 to 14)	11 (11 to 11)	266 (233 to 303)	194 (164 to 227)	37 (26 to 50)	16 (8 to 26)	20 (20 to 20)
Fiji	2005	145 (127 to 165)	108 (92 to 127)	21 (15 to 29)	9 (5 to 15)	6 (6 to 6)	262 (229 to 297)	195 (165 to 228)	38 (27 to 52)	17 (9 to 27)	11 (11 to 11)
Fiji	2006	160 (140 to 182)	115 (98 to 134)	24 (17 to 32)	11 (6 to 18)	10 (10 to 10)	288 (253 to 327)	207 (176 to 242)	43 (31 to 58)	20 (11 to 32)	17 (17 to 17)
Fiji	2007	158 (139 to 180)	108 (92 to 127)	26 (19 to 36)	12 (6 to 20)	12 (12 to 12)	285 (250 to 324)	195 (166 to 228)	47 (34 to 64)	21 (11 to 35)	22 (22 to 22)
Fiji	2008	151 (132 to 172)	102 (86 to 119)	28 (20 to 38)	12 (7 to 19)	10 (10 to 10)	273 (238 to 309)	184 (155 to 214)	50 (36 to 68)	21 (12 to 35)	17 (17 to 17)
Fiji	2009	161 (140 to 184)	105 (88 to 123)	33 (24 to 44)	13 (7 to 22)	10 (10 to 10)	289 (252 to 332)	189 (159 to 221)	60 (43 to 79)	24 (13 to 39)	17 (17 to 17)
Fiji	2010	165 (145 to 186)	100 (84 to 118)	35 (25 to 45)	14 (8 to 23)	16 (16 to 16)	297 (260 to 336)	180 (151 to 212)	63 (46 to 82)	25 (14 to 41)	29 (29 to 29)
Fiji	2011	167 (146 to 188)	99 (84 to 116)	36 (26 to 48)	16 (9 to 26)	16 (16 to 16)	300 (263 to 339)	178 (150 to 209)	66 (47 to 87)	29 (16 to 47)	28 (28 to 28)
Fiji	2012	160 (138 to 183)	97 (81 to 113)	37 (26 to 49)	18 (10 to 29)	9 (9 to 9)	288 (249 to 330)	174 (147 to 204)	66 (47 to 88)	32 (18 to 51)	16 (16 to 16)

Fiji	2013	165 (143 to 188)	97 (82 to 114)	36 (26 to 49)	20 (12 to 33)	11 (11 to 11)	296 (257 to 339)	175 (147 to 204)	65 (47 to 88)	36 (21 to 59)	20 (20 to 20)
Fiji	2014	185 (160 to 211)	111 (93 to 131)	38 (27 to 51)	25 (14 to 40)	11 (11 to 11)	333 (288 to 379)	200 (168 to 235)	68 (49 to 92)	44 (25 to 72)	20 (20 to 20)
Fiji	2015	191 (165 to 220)	118 (99 to 139)	39 (27 to 52)	26 (14 to 42)	9 (9 to 9)	344 (297 to 395)	212 (179 to 250)	69 (49 to 94)	47 (26 to 75)	15 (15 to 15)
Fiji	2016	187 (161 to 216)	115 (96 to 138)	37 (26 to 50)	26 (14 to 42)	9 (9 to 9)	336 (290 to 388)	207 (174 to 248)	66 (47 to 89)	46 (26 to 75)	16 (16 to 16)
Fiji	2017	198 (160 to 243)	126 (104 to 152)	36 (26 to 49)	30 (17 to 48)	6 (6 to 6)	356 (288 to 438)	227 (187 to 274)	65 (46 to 88)	53 (30 to 86)	11 (11 to 11)
Fiji	2018	218 (176 to 267)	145 (119 to 174)	37 (26 to 50)	31 (17 to 50)	6 (6 to 6)	393 (316 to 481)	261 (214 to 313)	66 (47 to 90)	55 (31 to 89)	11 (11 to 11)
Fiji	2019	209 (168 to 257)	134 (109 to 162)	38 (27 to 51)	31 (18 to 51)	6 (6 to 6)	376 (302 to 463)	241 (197 to 292)	68 (48 to 92)	57 (32 to 92)	11 (11 to 11)
Fiji	2020	215 (173 to 266)	138 (113 to 168)	39 (28 to 53)	32 (18 to 52)	6 (6 to 7)	388 (311 to 479)	249 (204 to 302)	70 (50 to 95)	58 (33 to 94)	11 (11 to 12)
Fiji	2021	219 (176 to 269)	140 (115 to 170)	39 (28 to 53)	33 (19 to 53)	6 (6 to 7)	394 (317 to 485)	253 (207 to 307)	71 (50 to 96)	59 (34 to 96)	11 (11 to 12)
Fiji	2022	222 (178 to 275)	143 (116 to 173)	40 (28 to 54)	34 (19 to 54)	6 (6 to 7)	400 (320 to 495)	257 (209 to 312)	72 (51 to 97)	60 (34 to 98)	12 (11 to 13)
Fiji	2023	226 (181 to 280)	145 (118 to 177)	40 (29 to 55)	34 (19 to 55)	7 (6 to 7)	407 (326 to 504)	261 (213 to 318)	73 (52 to 98)	61 (35 to 99)	12 (11 to 13)
Fiji	2024	230 (183 to 288)	148 (119 to 183)	41 (29 to 55)	35 (20 to 56)	7 (6 to 7)	414 (329 to 518)	266 (214 to 329)	74 (52 to 100)	62 (36 to 101)	12 (11 to 13)
Fiji	2025	234 (185 to 292)	150 (121 to 185)	41 (30 to 56)	35 (20 to 57)	7 (6 to 8)	421 (334 to 526)	271 (217 to 334)	75 (53 to 101)	64 (36 to 103)	12 (11 to 14)
Fiji	2026	238 (187 to 299)	153 (122 to 190)	42 (30 to 57)	36 (20 to 58)	7 (6 to 8)	428 (337 to 537)	276 (220 to 342)	76 (54 to 103)	65 (37 to 105)	12 (11 to 14)
Fiji	2027	242 (191 to 305)	156 (124 to 195)	43 (30 to 58)	37 (21 to 60)	7 (6 to 8)	436 (343 to 549)	281 (223 to 350)	77 (55 to 104)	66 (38 to 107)	12 (11 to 14)

Fiji	2028	246 (194 to 310)	159 (126 to 199)	43 (31 to 59)	37 (21 to 61)	7 (6 to 8)	443 (349 to 557)	286 (226 to 358)	78 (55 to 106)	67 (38 to 109)	13 (11 to 15)
Fiji	2029	251 (196 to 316)	162 (129 to 204)	44 (31 to 59)	38 (22 to 62)	7 (6 to 8)	451 (354 to 569)	291 (232 to 367)	79 (56 to 107)	68 (39 to 112)	13 (11 to 15)
Fiji	2030	255 (200 to 322)	165 (130 to 208)	44 (32 to 60)	39 (22 to 63)	7 (6 to 9)	459 (360 to 580)	296 (234 to 374)	80 (57 to 109)	70 (39 to 114)	13 (11 to 16)
Fiji	2031	260 (204 to 329)	168 (132 to 213)	45 (32 to 61)	39 (22 to 65)	7 (6 to 9)	468 (366 to 593)	302 (238 to 383)	81 (58 to 110)	71 (40 to 116)	13 (11 to 16)
Fiji	2032	264 (206 to 337)	171 (134 to 217)	46 (32 to 62)	40 (23 to 66)	7 (6 to 9)	476 (371 to 607)	308 (240 to 390)	82 (58 to 112)	72 (41 to 118)	13 (11 to 16)
Fiji	2033	270 (209 to 345)	175 (137 to 222)	46 (33 to 63)	41 (23 to 67)	8 (6 to 9)	485 (377 to 621)	314 (246 to 399)	84 (59 to 113)	74 (42 to 121)	14 (12 to 17)
Fiji	2034	275 (212 to 351)	178 (140 to 227)	47 (33 to 64)	42 (24 to 69)	8 (6 to 9)	495 (382 to 632)	321 (251 to 409)	85 (60 to 115)	75 (43 to 123)	14 (12 to 17)
Fiji	2035	280 (217 to 361)	182 (141 to 232)	48 (34 to 65)	43 (24 to 70)	8 (7 to 10)	504 (390 to 650)	327 (254 to 417)	86 (61 to 117)	77 (44 to 126)	14 (12 to 17)
Fiji	2036	285 (221 to 368)	185 (144 to 236)	49 (34 to 66)	44 (25 to 72)	8 (7 to 10)	514 (397 to 663)	333 (258 to 426)	88 (62 to 119)	78 (45 to 129)	14 (12 to 18)
Fiji	2037	291 (225 to 376)	189 (147 to 243)	49 (35 to 67)	44 (25 to 73)	8 (7 to 10)	523 (404 to 677)	340 (265 to 437)	89 (63 to 121)	80 (45 to 131)	15 (12 to 18)
Fiji	2038	296 (226 to 384)	192 (148 to 249)	50 (35 to 68)	45 (26 to 74)	8 (7 to 10)	532 (407 to 691)	346 (266 to 448)	90 (63 to 123)	81 (46 to 134)	15 (12 to 19)
Fiji	2039	301 (230 to 391)	196 (151 to 255)	51 (36 to 69)	46 (26 to 76)	8 (7 to 11)	541 (414 to 704)	352 (271 to 460)	91 (64 to 124)	83 (47 to 137)	15 (12 to 19)
Fiji	2040	306 (234 to 395)	199 (152 to 258)	51 (36 to 70)	47 (27 to 78)	9 (7 to 11)	551 (421 to 711)	359 (274 to 464)	92 (65 to 126)	84 (48 to 140)	15 (13 to 19)
Fiji	2041	312 (238 to 406)	203 (155 to 266)	52 (37 to 71)	48 (27 to 79)	9 (7 to 11)	561 (429 to 731)	366 (279 to 479)	94 (66 to 128)	86 (49 to 142)	16 (13 to 20)
Fiji	2042	317 (241 to 412)	207 (158 to 271)	53 (37 to 72)	49 (27 to 81)	9 (7 to 11)	571 (434 to 742)	372 (284 to 487)	95 (67 to 130)	87 (49 to 145)	16 (13 to 20)

Fiji	2043	322 (244 to 422)	210 (160 to 275)	53 (37 to 73)	49 (28 to 82)	9 (7 to 12)	580 (440 to 760)	379 (288 to 496)	96 (67 to 132)	89 (50 to 148)	16 (13 to 21)
Fiji	2044	327 (248 to 426)	214 (162 to 277)	54 (38 to 74)	50 (28 to 83)	9 (7 to 12)	589 (446 to 767)	385 (292 to 499)	97 (68 to 133)	90 (50 to 150)	16 (13 to 22)
Fiji	2045	332 (250 to 431)	217 (163 to 282)	55 (38 to 75)	51 (28 to 85)	9 (7 to 12)	597 (450 to 776)	391 (293 to 507)	98 (69 to 135)	92 (51 to 153)	17 (13 to 21)
Fiji	2046	336 (253 to 439)	220 (165 to 287)	55 (39 to 76)	52 (29 to 86)	9 (7 to 12)	605 (456 to 789)	396 (298 to 516)	99 (70 to 136)	93 (51 to 155)	17 (13 to 22)
Fiji	2047	341 (256 to 444)	223 (167 to 292)	56 (39 to 76)	53 (29 to 88)	10 (8 to 13)	614 (460 to 800)	402 (301 to 526)	100 (71 to 138)	95 (52 to 158)	17 (14 to 23)
Fiji	2048	346 (260 to 456)	227 (170 to 298)	56 (40 to 77)	53 (29 to 89)	10 (8 to 13)	624 (468 to 820)	408 (306 to 537)	102 (71 to 139)	96 (53 to 161)	18 (14 to 23)
Fiji	2049	352 (264 to 465)	231 (173 to 305)	57 (40 to 78)	54 (30 to 90)	10 (8 to 13)	633 (476 to 837)	415 (311 to 549)	103 (72 to 141)	98 (53 to 162)	18 (14 to 24)
Fiji	2050	357 (268 to 476)	234 (173 to 313)	58 (41 to 79)	55 (30 to 91)	10 (8 to 14)	643 (482 to 857)	422 (312 to 564)	104 (73 to 143)	99 (54 to 165)	18 (14 to 25)
Finland	1995	2371 (2270 to 2482)	1773 (1677 to 1877)	544 (508 to 585)	54 (38 to 74)	0 (0 to 0)	2324 (2225 to 2432)	1738 (1644 to 1839)	533 (498 to 573)	53 (37 to 73)	0 (0 to 0)
Finland	1996	2423 (2344 to 2502)	1810 (1739 to 1882)	556 (527 to 588)	57 (42 to 75)	0 (0 to 0)	2374 (2297 to 2452)	1774 (1704 to 1845)	545 (516 to 576)	56 (41 to 73)	0 (0 to 0)
Finland	1997	2495 (2419 to 2570)	1869 (1803 to 1936)	566 (538 to 597)	59 (46 to 77)	0 (0 to 0)	2445 (2371 to 2519)	1832 (1766 to 1897)	555 (527 to 585)	58 (45 to 75)	0 (0 to 0)
Finland	1998	2548 (2470 to 2628)	1910 (1838 to 1981)	576 (547 to 606)	62 (49 to 78)	0 (0 to 0)	2497 (2421 to 2575)	1872 (1802 to 1942)	565 (536 to 594)	60 (48 to 76)	0 (0 to 0)
Finland	1999	2642 (2567 to 2716)	1974 (1906 to 2043)	602 (575 to 631)	66 (53 to 81)	0 (0 to 0)	2589 (2516 to 2662)	1934 (1868 to 2002)	590 (563 to 619)	65 (52 to 79)	0 (0 to 0)

Finland	2000	2757 (2688 to 2828)	2057 (1991 to 2122)	630 (604 to 659)	70 (58 to 85)	0 (0 to 0)	2702 (2635 to 2772)	2016 (1951 to 2079)	617 (592 to 646)	69 (57 to 83)	0 (0 to 0)
Finland	2001	2880 (2810 to 2950)	2155 (2090 to 2222)	652 (627 to 678)	74 (62 to 88)	0 (0 to 0)	2823 (2753 to 2891)	2111 (2048 to 2178)	639 (615 to 664)	72 (61 to 86)	0 (0 to 0)
Finland	2002	3068 (3001 to 3137)	2313 (2248 to 2376)	678 (654 to 702)	77 (65 to 91)	0 (0 to 0)	3007 (2941 to 3074)	2267 (2203 to 2329)	664 (641 to 688)	76 (64 to 89)	0 (0 to 0)
Finland	2003	3245 (3172 to 3315)	2466 (2397 to 2534)	699 (674 to 725)	81 (69 to 95)	0 (0 to 0)	3180 (3109 to 3249)	2417 (2349 to 2483)	685 (661 to 710)	79 (67 to 93)	0 (0 to 0)
Finland	2004	3442 (3364 to 3516)	2635 (2562 to 2705)	721 (696 to 749)	85 (72 to 99)	0 (0 to 0)	3373 (3296 to 3446)	2583 (2511 to 2651)	707 (682 to 734)	83 (71 to 97)	0 (0 to 0)
Finland	2005	3601 (3522 to 3681)	2776 (2705 to 2849)	736 (709 to 764)	90 (77 to 104)	0 (0 to 0)	3529 (3451 to 3608)	2720 (2651 to 2792)	721 (695 to 748)	88 (75 to 102)	0 (0 to 0)
Finland	2006	3737 (3660 to 3821)	2896 (2822 to 2971)	747 (720 to 774)	95 (81 to 111)	0 (0 to 0)	3663 (3587 to 3745)	2838 (2766 to 2912)	732 (706 to 759)	93 (79 to 109)	0 (0 to 0)
Finland	2007	3828 (3749 to 3906)	2972 (2897 to 3046)	757 (730 to 784)	99 (85 to 115)	0 (0 to 0)	3751 (3674 to 3827)	2913 (2840 to 2985)	742 (715 to 768)	97 (83 to 113)	0 (0 to 0)
Finland	2008	3942 (3863 to 4021)	3064 (2993 to 3134)	773 (746 to 800)	105 (90 to 122)	0 (0 to 0)	3864 (3786 to 3941)	3003 (2933 to 3071)	758 (731 to 784)	103 (88 to 119)	0 (0 to 0)
Finland	2009	4020 (3938 to 4103)	3125 (3051 to 3195)	785 (759 to 812)	110 (94 to 127)	0 (0 to 0)	3939 (3859 to 4021)	3063 (2990 to 3131)	769 (744 to 796)	108 (92 to 125)	0 (0 to 0)

Finland	2010	4083 (3998 to 4170)	3172 (3095 to 3245)	798 (771 to 825)	113 (97 to 131)	0 (0 to 0)	4002 (3918 to 4087)	3108 (3034 to 3180)	782 (756 to 808)	111 (95 to 128)	0 (0 to 0)
Finland	2011	4153 (4070 to 4234)	3233 (3160 to 3304)	803 (776 to 831)	117 (100 to 134)	0 (0 to 0)	4070 (3988 to 4149)	3169 (3097 to 3238)	787 (761 to 814)	115 (98 to 132)	0 (0 to 0)
Finland	2012	4186 (4102 to 4269)	3270 (3194 to 3345)	797 (769 to 827)	119 (102 to 139)	0 (0 to 0)	4102 (4020 to 4183)	3204 (3130 to 3278)	781 (754 to 810)	117 (100 to 136)	0 (0 to 0)
Finland	2013	4143 (4063 to 4226)	3235 (3159 to 3312)	789 (762 to 820)	119 (103 to 138)	0 (0 to 0)	4060 (3982 to 4141)	3170 (3096 to 3245)	773 (747 to 804)	116 (101 to 135)	0 (0 to 0)
Finland	2014	4231 (4151 to 4315)	3296 (3220 to 3367)	814 (788 to 843)	121 (105 to 143)	0 (0 to 0)	4147 (4068 to 4229)	3230 (3155 to 3300)	797 (772 to 826)	119 (103 to 140)	0 (0 to 0)
Finland	2015	4304 (4221 to 4387)	3332 (3257 to 3405)	850 (824 to 880)	122 (105 to 142)	0 (0 to 0)	4218 (4136 to 4299)	3266 (3192 to 3336)	833 (807 to 862)	119 (103 to 139)	0 (0 to 0)
Finland	2016	4355 (4264 to 4434)	3349 (3274 to 3422)	879 (851 to 910)	127 (108 to 150)	0 (0 to 0)	4268 (4179 to 4346)	3282 (3208 to 3354)	862 (834 to 892)	124 (106 to 147)	0 (0 to 0)
Finland	2017	4386 (4253 to 4523)	3361 (3253 to 3465)	893 (853 to 936)	131 (107 to 159)	0 (0 to 0)	4298 (4168 to 4432)	3294 (3188 to 3396)	875 (835 to 917)	129 (105 to 156)	0 (0 to 0)
Finland	2018	4430 (4277 to 4583)	3394 (3277 to 3514)	904 (864 to 947)	133 (108 to 161)	0 (0 to 0)	4342 (4192 to 4492)	3326 (3211 to 3444)	886 (847 to 928)	130 (106 to 158)	0 (0 to 0)
Finland	2019	4473 (4315 to 4641)	3425 (3294 to 3567)	913 (873 to 957)	135 (110 to 163)	0 (0 to 0)	4383 (4229 to 4549)	3356 (3228 to 3496)	895 (856 to 938)	132 (107 to 160)	0 (0 to 0)

Finland	2020	4540 (4354 to 4719)	3479 (3327 to 3630)	924 (883 to 969)	137 (111 to 165)	0 (0 to 0)	4450 (4267 to 4625)	3410 (3260 to 3557)	906 (866 to 949)	134 (109 to 162)	0 (0 to 0)
Finland	2021	4578 (4394 to 4772)	3511 (3351 to 3673)	929 (888 to 973)	138 (113 to 167)	0 (0 to 0)	4486 (4306 to 4677)	3440 (3284 to 3600)	910 (870 to 954)	135 (110 to 163)	0 (0 to 0)
Finland	2022	4616 (4406 to 4819)	3543 (3367 to 3719)	934 (893 to 978)	139 (113 to 168)	0 (0 to 0)	4524 (4318 to 4723)	3472 (3300 to 3645)	915 (875 to 958)	137 (111 to 165)	0 (0 to 0)
Finland	2023	4665 (4445 to 4883)	3585 (3387 to 3765)	939 (897 to 984)	141 (115 to 171)	0 (0 to 0)	4571 (4356 to 4786)	3513 (3319 to 3690)	920 (879 to 964)	138 (113 to 167)	0 (0 to 0)
Finland	2024	4698 (4471 to 4921)	3615 (3421 to 3819)	941 (898 to 986)	142 (115 to 172)	0 (0 to 0)	4604 (4381 to 4822)	3543 (3352 to 3743)	922 (880 to 966)	139 (113 to 169)	0 (0 to 0)
Finland	2025	4725 (4490 to 4969)	3640 (3428 to 3845)	941 (898 to 987)	143 (116 to 173)	0 (0 to 0)	4630 (4400 to 4870)	3568 (3360 to 3769)	922 (880 to 967)	140 (114 to 170)	0 (0 to 0)
Finland	2026	4763 (4510 to 5008)	3675 (3457 to 3894)	943 (900 to 989)	144 (117 to 175)	0 (0 to 0)	4668 (4420 to 4908)	3602 (3388 to 3816)	924 (882 to 970)	142 (115 to 172)	0 (0 to 0)
Finland	2027	4798 (4558 to 5060)	3707 (3490 to 3931)	945 (901 to 992)	146 (119 to 177)	0 (0 to 0)	4702 (4467 to 4959)	3633 (3421 to 3852)	926 (883 to 972)	143 (116 to 173)	0 (0 to 0)
Finland	2028	4829 (4566 to 5101)	3737 (3509 to 3978)	946 (901 to 994)	147 (119 to 178)	0 (0 to 0)	4733 (4474 to 4999)	3662 (3439 to 3899)	927 (883 to 974)	144 (117 to 174)	0 (0 to 0)
Finland	2029	4859 (4590 to 5154)	3765 (3519 to 4019)	946 (901 to 995)	148 (120 to 179)	0 (0 to 0)	4762 (4499 to 5051)	3690 (3449 to 3939)	927 (883 to 975)	145 (117 to 176)	0 (0 to 0)

Finland	2030	4894 (4595 to 5181)	3798 (3535 to 4051)	948 (901 to 998)	149 (121 to 181)	0 (0 to 0)	4796 (4503 to 5077)	3722 (3464 to 3970)	929 (883 to 978)	146 (118 to 178)	0 (0 to 0)
Finland	2031	4937 (4630 to 5240)	3836 (3557 to 4100)	950 (903 to 1002)	150 (122 to 183)	0 (0 to 0)	4838 (4537 to 5135)	3760 (3486 to 4018)	931 (884 to 982)	147 (119 to 180)	0 (0 to 0)
Finland	2032	4990 (4669 to 5295)	3884 (3594 to 4158)	955 (905 to 1006)	152 (123 to 186)	0 (0 to 0)	4891 (4576 to 5189)	3806 (3522 to 4075)	936 (887 to 986)	149 (121 to 182)	0 (0 to 0)
Finland	2033	5049 (4730 to 5372)	3935 (3639 to 4222)	960 (910 to 1013)	154 (125 to 188)	0 (0 to 0)	4948 (4636 to 5265)	3857 (3566 to 4137)	941 (892 to 992)	151 (122 to 184)	0 (0 to 0)
Finland	2034	5115 (4775 to 5449)	3993 (3691 to 4296)	966 (915 to 1021)	156 (126 to 191)	0 (0 to 0)	5013 (4679 to 5340)	3913 (3617 to 4210)	947 (897 to 1001)	153 (124 to 187)	0 (0 to 0)
Finland	2035	5185 (4852 to 5535)	4054 (3744 to 4366)	972 (920 to 1028)	159 (128 to 194)	0 (0 to 0)	5081 (4755 to 5425)	3973 (3669 to 4279)	953 (901 to 1008)	155 (125 to 190)	0 (0 to 0)
Finland	2036	5257 (4891 to 5617)	4117 (3789 to 4444)	978 (925 to 1037)	161 (130 to 198)	0 (0 to 0)	5152 (4793 to 5504)	4035 (3713 to 4355)	959 (906 to 1016)	158 (127 to 194)	0 (0 to 0)
Finland	2037	5324 (4956 to 5704)	4176 (3827 to 4522)	984 (929 to 1046)	163 (131 to 201)	0 (0 to 0)	5217 (4856 to 5589)	4093 (3750 to 4431)	964 (911 to 1025)	160 (129 to 197)	0 (0 to 0)
Finland	2038	5392 (4993 to 5790)	4237 (3879 to 4597)	990 (933 to 1055)	165 (133 to 204)	0 (0 to 0)	5284 (4893 to 5674)	4152 (3801 to 4505)	970 (915 to 1034)	162 (130 to 199)	0 (0 to 0)
Finland	2039	5467 (5069 to 5881)	4303 (3926 to 4682)	996 (937 to 1064)	168 (134 to 207)	0 (0 to 0)	5358 (4968 to 5763)	4217 (3847 to 4588)	977 (918 to 1043)	165 (132 to 203)	0 (0 to 0)

Finland	2040	5545 (5110 to 5977)	4371 (3984 to 4775)	1003 (941 to 1074)	170 (136 to 210)	0 (0 to 0)	5434 (5007 to 5858)	4284 (3904 to 4679)	983 (922 to 1053)	167 (133 to 206)	0 (0 to 0)
Finland	2041	5625 (5174 to 6079)	4441 (4018 to 4859)	1010 (946 to 1083)	173 (138 to 214)	0 (0 to 0)	5512 (5071 to 5958)	4352 (3937 to 4762)	990 (927 to 1062)	170 (135 to 209)	0 (0 to 0)
Finland	2042	5702 (5239 to 6170)	4509 (4093 to 4949)	1017 (950 to 1092)	176 (140 to 217)	0 (0 to 0)	5588 (5135 to 6047)	4419 (4011 to 4850)	997 (931 to 1070)	172 (137 to 212)	0 (0 to 0)
Finland	2043	5778 (5321 to 6279)	4576 (4151 to 5033)	1024 (955 to 1103)	178 (142 to 220)	0 (0 to 0)	5662 (5215 to 6154)	4484 (4068 to 4932)	1003 (936 to 1081)	175 (139 to 216)	0 (0 to 0)
Finland	2044	5853 (5362 to 6342)	4643 (4212 to 5102)	1030 (960 to 1112)	181 (143 to 223)	0 (0 to 0)	5736 (5255 to 6215)	4550 (4128 to 5000)	1010 (941 to 1090)	177 (141 to 218)	0 (0 to 0)
Finland	2045	5928 (5445 to 6448)	4708 (4273 to 5180)	1037 (965 to 1121)	183 (145 to 226)	0 (0 to 0)	5810 (5336 to 6319)	4614 (4188 to 5077)	1016 (945 to 1099)	180 (142 to 222)	0 (0 to 0)
Finland	2046	6005 (5491 to 6527)	4776 (4324 to 5259)	1043 (969 to 1131)	186 (147 to 230)	0 (0 to 0)	5885 (5381 to 6397)	4680 (4237 to 5154)	1022 (950 to 1108)	182 (144 to 225)	0 (0 to 0)
Finland	2047	6081 (5567 to 6636)	4843 (4371 to 5350)	1050 (973 to 1141)	188 (149 to 233)	0 (0 to 0)	5959 (5456 to 6503)	4746 (4284 to 5243)	1029 (953 to 1118)	185 (146 to 229)	0 (0 to 0)
Finland	2048	6160 (5603 to 6731)	4913 (4414 to 5423)	1056 (977 to 1151)	191 (150 to 237)	0 (0 to 0)	6037 (5491 to 6597)	4815 (4326 to 5315)	1035 (957 to 1128)	187 (147 to 233)	0 (0 to 0)
Finland	2049	6240 (5697 to 6826)	4983 (4470 to 5520)	1063 (981 to 1161)	194 (152 to 241)	0 (0 to 0)	6115 (5583 to 6689)	4884 (4380 to 5410)	1042 (961 to 1138)	190 (149 to 236)	0 (0 to 0)

Finland	2050	6319 (5725 to 6920)	5053 (4524 to 5602)	1069 (985 to 1170)	196 (154 to 245)	0 (0 to 0)	6192 (5610 to 6782)	4952 (4433 to 5490)	1048 (965 to 1146)	192 (151 to 240)	0 (0 to 0)
France	1995	3104 (3032 to 3180)	2462 (2404 to 2522)	226 (199 to 256)	416 (376 to 459)	0 (0 to 0)	3494 (3413 to 3579)	2772 (2706 to 2839)	254 (223 to 289)	468 (423 to 516)	0 (0 to 0)
France	1996	3121 (3063 to 3180)	2474 (2430 to 2516)	222 (198 to 246)	425 (396 to 458)	0 (0 to 0)	3514 (3448 to 3579)	2785 (2735 to 2833)	250 (223 to 277)	478 (446 to 515)	0 (0 to 0)
France	1997	3169 (3112 to 3230)	2511 (2465 to 2556)	221 (200 to 243)	437 (408 to 468)	0 (0 to 0)	3567 (3503 to 3636)	2826 (2775 to 2877)	249 (226 to 274)	492 (459 to 527)	0 (0 to 0)
France	1998	3223 (3166 to 3283)	2550 (2503 to 2596)	225 (206 to 247)	447 (419 to 477)	0 (0 to 0)	3628 (3564 to 3696)	2871 (2817 to 2922)	254 (232 to 278)	504 (472 to 537)	0 (0 to 0)
France	1999	3309 (3255 to 3365)	2615 (2572 to 2659)	234 (215 to 254)	459 (433 to 488)	0 (0 to 0)	3725 (3664 to 3788)	2944 (2895 to 2993)	264 (242 to 286)	517 (487 to 550)	0 (0 to 0)
France	2000	3407 (3355 to 3457)	2691 (2651 to 2731)	244 (226 to 264)	472 (448 to 498)	0 (0 to 0)	3836 (3776 to 3891)	3029 (2984 to 3074)	274 (254 to 297)	532 (505 to 561)	0 (0 to 0)
France	2001	3504 (3455 to 3554)	2768 (2728 to 2808)	252 (234 to 273)	484 (461 to 508)	0 (0 to 0)	3945 (3889 to 4001)	3116 (3071 to 3161)	284 (264 to 308)	545 (519 to 572)	0 (0 to 0)
France	2002	3630 (3576 to 3680)	2869 (2826 to 2909)	261 (243 to 280)	500 (477 to 526)	0 (0 to 0)	4086 (4026 to 4142)	3230 (3181 to 3274)	293 (273 to 316)	563 (536 to 592)	0 (0 to 0)
France	2003	3668 (3620 to 3717)	2892 (2853 to 2932)	269 (250 to 289)	507 (483 to 532)	0 (0 to 0)	4129 (4075 to 4184)	3255 (3212 to 3301)	303 (282 to 325)	571 (544 to 599)	0 (0 to 0)

France	2004	3759 (3709 to 3809)	2954 (2915 to 2997)	283 (265 to 302)	522 (497 to 545)	0 (0 to 0)	4232 (4176 to 4288)	3326 (3281 to 3374)	319 (298 to 340)	587 (559 to 614)	0 (0 to 0)
France	2005	3862 (3811 to 3914)	3021 (2978 to 3062)	308 (289 to 329)	533 (509 to 557)	0 (0 to 0)	4347 (4290 to 4406)	3400 (3353 to 3447)	347 (326 to 370)	600 (573 to 627)	0 (0 to 0)
France	2006	3930 (3878 to 3984)	3050 (3010 to 3093)	347 (326 to 368)	533 (507 to 558)	0 (0 to 0)	4424 (4366 to 4485)	3434 (3388 to 3481)	390 (367 to 414)	600 (570 to 628)	0 (0 to 0)
France	2007	4012 (3958 to 4063)	3094 (3052 to 3138)	379 (358 to 402)	539 (512 to 564)	0 (0 to 0)	4516 (4455 to 4573)	3482 (3435 to 3532)	427 (403 to 452)	606 (577 to 635)	0 (0 to 0)
France	2008	4071 (4020 to 4126)	3122 (3083 to 3164)	405 (384 to 427)	544 (520 to 570)	0 (0 to 0)	4583 (4525 to 4644)	3514 (3471 to 3562)	456 (432 to 481)	612 (585 to 641)	0 (0 to 0)
France	2009	4206 (4150 to 4264)	3217 (3178 to 3260)	427 (404 to 450)	561 (538 to 587)	0 (0 to 0)	4734 (4672 to 4799)	3622 (3577 to 3670)	481 (455 to 507)	632 (606 to 661)	0 (0 to 0)
France	2010	4264 (4208 to 4318)	3253 (3212 to 3298)	437 (413 to 460)	574 (549 to 600)	0 (0 to 0)	4800 (4737 to 4861)	3662 (3615 to 3713)	492 (465 to 518)	646 (617 to 675)	0 (0 to 0)
France	2011	4310 (4257 to 4370)	3280 (3239 to 3325)	441 (419 to 466)	588 (562 to 613)	0 (0 to 0)	4851 (4792 to 4919)	3693 (3646 to 3743)	497 (471 to 525)	662 (633 to 690)	0 (0 to 0)
France	2012	4353 (4301 to 4411)	3314 (3273 to 3357)	441 (417 to 465)	598 (572 to 625)	0 (0 to 0)	4900 (4842 to 4965)	3730 (3685 to 3779)	497 (470 to 524)	673 (644 to 704)	0 (0 to 0)
France	2013	4415 (4360 to 4475)	3370 (3325 to 3414)	440 (416 to 463)	605 (580 to 633)	0 (0 to 0)	4969 (4907 to 5038)	3793 (3743 to 3844)	495 (469 to 521)	681 (653 to 712)	0 (0 to 0)

France	2014	4453 (4398 to 4510)	3410 (3368 to 3455)	437 (415 to 463)	606 (580 to 633)	0 (0 to 0)	5013 (4951 to 5076)	3839 (3791 to 3889)	492 (467 to 521)	682 (653 to 713)	0 (0 to 0)
France	2015	4471 (4412 to 4532)	3432 (3387 to 3479)	432 (409 to 458)	607 (580 to 634)	0 (0 to 0)	5033 (4966 to 5101)	3863 (3813 to 3916)	487 (460 to 516)	683 (653 to 713)	0 (0 to 0)
France	2016	4485 (4427 to 4545)	3449 (3406 to 3491)	428 (404 to 455)	607 (579 to 635)	0 (0 to 0)	5049 (4983 to 5116)	3883 (3834 to 3929)	482 (455 to 512)	684 (652 to 715)	0 (0 to 0)
France	2017	4530 (4455 to 4600)	3490 (3435 to 3543)	428 (392 to 462)	613 (576 to 651)	0 (0 to 0)	5100 (5015 to 5178)	3928 (3867 to 3988)	482 (441 to 520)	690 (648 to 733)	0 (0 to 0)
France	2018	4598 (4526 to 4674)	3542 (3484 to 3602)	433 (396 to 467)	623 (586 to 662)	0 (0 to 0)	5176 (5095 to 5261)	3987 (3922 to 4055)	487 (446 to 525)	702 (659 to 745)	0 (0 to 0)
France	2019	4645 (4567 to 4720)	3577 (3511 to 3642)	437 (400 to 472)	631 (593 to 670)	0 (0 to 0)	5229 (5141 to 5314)	4026 (3952 to 4100)	492 (451 to 531)	710 (667 to 754)	0 (0 to 0)
France	2020	4723 (4639 to 4808)	3639 (3568 to 3712)	442 (405 to 477)	641 (602 to 682)	0 (0 to 0)	5316 (5222 to 5412)	4097 (4017 to 4178)	498 (455 to 537)	722 (677 to 768)	0 (0 to 0)
France	2021	4772 (4688 to 4851)	3679 (3604 to 3758)	445 (408 to 481)	648 (608 to 690)	0 (0 to 0)	5372 (5277 to 5461)	4141 (4057 to 4230)	501 (459 to 541)	730 (684 to 776)	0 (0 to 0)
France	2022	4829 (4742 to 4911)	3725 (3645 to 3805)	448 (410 to 484)	656 (615 to 698)	0 (0 to 0)	5436 (5338 to 5528)	4194 (4103 to 4283)	505 (462 to 545)	738 (692 to 786)	0 (0 to 0)
France	2023	4865 (4774 to 4952)	3755 (3669 to 3841)	449 (411 to 485)	660 (619 to 703)	0 (0 to 0)	5476 (5373 to 5574)	4227 (4130 to 4324)	506 (462 to 546)	743 (696 to 791)	0 (0 to 0)

France	2024	4901 (4810 to 4992)	3785 (3689 to 3881)	450 (412 to 486)	665 (622 to 709)	0 (0 to 0)	5517 (5414 to 5620)	4261 (4152 to 4369)	507 (463 to 547)	749 (700 to 799)	0 (0 to 0)
France	2025	4938 (4844 to 5032)	3817 (3722 to 3909)	452 (413 to 488)	670 (626 to 716)	0 (0 to 0)	5559 (5452 to 5665)	4296 (4190 to 4400)	508 (465 to 549)	754 (705 to 806)	0 (0 to 0)
France	2026	4973 (4872 to 5070)	3846 (3747 to 3947)	453 (415 to 490)	674 (629 to 721)	0 (0 to 0)	5598 (5484 to 5708)	4329 (4218 to 4443)	510 (467 to 551)	759 (708 to 811)	0 (0 to 0)
France	2027	5009 (4914 to 5106)	3876 (3771 to 3981)	454 (416 to 491)	679 (633 to 728)	0 (0 to 0)	5639 (5531 to 5747)	4363 (4245 to 4481)	511 (468 to 553)	764 (712 to 820)	0 (0 to 0)
France	2028	5046 (4948 to 5150)	3907 (3792 to 4018)	455 (417 to 493)	684 (636 to 736)	0 (0 to 0)	5680 (5569 to 5797)	4398 (4268 to 4522)	513 (469 to 555)	770 (716 to 829)	0 (0 to 0)
France	2029	5085 (4979 to 5194)	3939 (3822 to 4048)	457 (418 to 495)	689 (640 to 744)	0 (0 to 0)	5724 (5605 to 5847)	4434 (4302 to 4557)	514 (471 to 557)	775 (720 to 837)	0 (0 to 0)
France	2030	5127 (5026 to 5235)	3974 (3849 to 4085)	459 (420 to 497)	694 (644 to 752)	0 (0 to 0)	5772 (5658 to 5893)	4473 (4333 to 4599)	517 (473 to 559)	782 (725 to 846)	0 (0 to 0)
France	2031	5177 (5074 to 5281)	4014 (3887 to 4137)	461 (423 to 499)	701 (649 to 761)	0 (0 to 0)	5827 (5712 to 5944)	4519 (4375 to 4657)	519 (476 to 562)	789 (730 to 856)	0 (0 to 0)
France	2032	5229 (5125 to 5333)	4057 (3919 to 4182)	464 (426 to 502)	708 (654 to 770)	0 (0 to 0)	5886 (5769 to 6003)	4566 (4412 to 4707)	522 (479 to 565)	797 (736 to 867)	0 (0 to 0)
France	2033	5282 (5174 to 5398)	4100 (3957 to 4237)	467 (428 to 505)	715 (658 to 780)	0 (0 to 0)	5946 (5824 to 6077)	4615 (4454 to 4769)	526 (482 to 569)	805 (741 to 878)	0 (0 to 0)

France	2034	5337 (5226 to 5448)	4144 (3998 to 4280)	470 (430 to 509)	723 (663 to 790)	0 (0 to 0)	6008 (5883 to 6133)	4665 (4501 to 4817)	529 (484 to 573)	813 (746 to 889)	0 (0 to 0)
France	2035	5390 (5262 to 5513)	4187 (4037 to 4338)	473 (433 to 512)	730 (667 to 800)	0 (0 to 0)	6067 (5923 to 6206)	4713 (4544 to 4883)	532 (487 to 576)	821 (751 to 900)	0 (0 to 0)
France	2036	5443 (5317 to 5572)	4231 (4069 to 4383)	476 (436 to 515)	737 (672 to 810)	0 (0 to 0)	6127 (5985 to 6272)	4762 (4581 to 4934)	535 (490 to 580)	830 (757 to 912)	0 (0 to 0)
France	2037	5493 (5364 to 5638)	4271 (4112 to 4423)	478 (438 to 519)	744 (677 to 820)	0 (0 to 0)	6184 (6038 to 6346)	4808 (4629 to 4978)	539 (493 to 584)	838 (762 to 923)	0 (0 to 0)
France	2038	5549 (5400 to 5695)	4315 (4149 to 4479)	482 (440 to 523)	752 (681 to 830)	0 (0 to 0)	6246 (6079 to 6411)	4857 (4670 to 5042)	542 (495 to 589)	846 (767 to 934)	0 (0 to 0)
France	2039	5608 (5447 to 5755)	4362 (4187 to 4533)	485 (443 to 528)	760 (687 to 841)	0 (0 to 0)	6312 (6132 to 6479)	4910 (4713 to 5103)	546 (498 to 595)	856 (773 to 947)	0 (0 to 0)
France	2040	5669 (5508 to 5837)	4411 (4232 to 4583)	489 (445 to 533)	769 (693 to 853)	0 (0 to 0)	6382 (6200 to 6571)	4966 (4763 to 5159)	551 (501 to 600)	866 (780 to 960)	0 (0 to 0)
France	2041	5732 (5557 to 5902)	4461 (4273 to 4643)	493 (449 to 539)	778 (698 to 865)	0 (0 to 0)	6453 (6255 to 6644)	5022 (4810 to 5226)	555 (505 to 606)	876 (786 to 974)	0 (0 to 0)
France	2042	5797 (5616 to 5977)	4512 (4311 to 4695)	497 (452 to 544)	787 (705 to 877)	0 (0 to 0)	6525 (6322 to 6728)	5079 (4853 to 5285)	560 (509 to 613)	886 (794 to 988)	0 (0 to 0)
France	2043	5863 (5670 to 6046)	4564 (4357 to 4760)	502 (456 to 550)	797 (711 to 891)	0 (0 to 0)	6600 (6383 to 6806)	5138 (4905 to 5358)	565 (513 to 619)	897 (800 to 1003)	0 (0 to 0)

France	2044	5931 (5724 to 6126)	4618 (4408 to 4819)	506 (459 to 555)	807 (718 to 905)	0 (0 to 0)	6677 (6443 to 6896)	5198 (4962 to 5425)	570 (516 to 625)	908 (808 to 1018)	0 (0 to 0)
France	2045	6001 (5788 to 6198)	4673 (4459 to 4883)	511 (463 to 561)	817 (725 to 918)	0 (0 to 0)	6755 (6515 to 6977)	5260 (5019 to 5497)	575 (521 to 632)	920 (816 to 1034)	0 (0 to 0)
France	2046	6072 (5847 to 6290)	4728 (4504 to 4952)	516 (467 to 568)	828 (732 to 934)	0 (0 to 0)	6835 (6582 to 7080)	5323 (5070 to 5575)	581 (525 to 639)	932 (825 to 1051)	0 (0 to 0)
France	2047	6147 (5927 to 6372)	4787 (4557 to 5017)	521 (471 to 574)	839 (740 to 950)	0 (0 to 0)	6920 (6672 to 7173)	5389 (5129 to 5647)	587 (531 to 646)	944 (834 to 1069)	0 (0 to 0)
France	2048	6221 (5978 to 6466)	4845 (4612 to 5082)	526 (475 to 579)	850 (748 to 966)	0 (0 to 0)	7003 (6729 to 7279)	5454 (5192 to 5720)	593 (535 to 652)	957 (842 to 1087)	0 (0 to 0)
France	2049	6298 (6033 to 6558)	4905 (4666 to 5151)	532 (480 to 585)	861 (755 to 981)	0 (0 to 0)	7090 (6791 to 7382)	5522 (5252 to 5798)	599 (540 to 659)	970 (850 to 1104)	0 (0 to 0)
France	2050	6379 (6103 to 6660)	4968 (4722 to 5226)	537 (485 to 592)	873 (764 to 999)	0 (0 to 0)	7181 (6869 to 7497)	5593 (5315 to 5883)	605 (546 to 667)	983 (860 to 1124)	0 (0 to 0)
Gabon	1995	338 (301 to 381)	114 (94 to 134)	198 (169 to 236)	26 (15 to 41)	0 (0 to 0)	820 (730 to 925)	275 (228 to 325)	481 (411 to 571)	63 (37 to 99)	0 (0 to 0)
Gabon	1996	326 (294 to 365)	109 (92 to 128)	190 (166 to 220)	25 (15 to 38)	3 (3 to 3)	791 (714 to 886)	265 (223 to 309)	461 (402 to 533)	59 (35 to 93)	6 (6 to 6)
Gabon	1997	345 (314 to 381)	119 (101 to 137)	194 (170 to 220)	26 (16 to 41)	6 (6 to 6)	836 (761 to 923)	288 (245 to 332)	470 (411 to 534)	64 (39 to 100)	15 (15 to 15)
Gabon	1998	398 (362 to 439)	143 (123 to 164)	215 (188 to 242)	32 (19 to 49)	8 (8 to 8)	965 (879 to 1064)	347 (299 to 398)	522 (455 to 586)	76 (46 to 119)	20 (20 to 20)
Gabon	1999	306 (277 to 338)	105 (91 to 120)	175 (152 to 199)	24 (14 to 36)	2 (2 to 2)	741 (672 to 819)	255 (220 to 291)	424 (369 to 482)	57 (34 to 88)	5 (5 to 5)

Gabon	2000	270 (244 to 298)	95 (83 to 109)	151 (131 to 171)	21 (12 to 32)	2 (2 to 2)	653 (593 to 722)	231 (201 to 264)	367 (318 to 416)	51 (30 to 79)	5 (5 to 5)
Gabon	2001	296 (268 to 326)	113 (98 to 129)	156 (135 to 177)	24 (14 to 38)	2 (2 to 2)	717 (650 to 791)	274 (239 to 313)	379 (328 to 429)	59 (35 to 91)	6 (6 to 6)
Gabon	2002	287 (262 to 317)	108 (94 to 124)	151 (131 to 172)	23 (14 to 36)	5 (5 to 5)	697 (635 to 768)	263 (229 to 300)	366 (318 to 416)	56 (33 to 88)	12 (12 to 12)
Gabon	2003	270 (245 to 297)	102 (88 to 116)	143 (125 to 162)	21 (12 to 33)	4 (4 to 4)	654 (594 to 720)	247 (214 to 281)	347 (302 to 393)	51 (30 to 81)	9 (9 to 9)
Gabon	2004	269 (244 to 295)	102 (89 to 116)	139 (122 to 160)	21 (12 to 34)	6 (6 to 6)	651 (591 to 715)	247 (215 to 281)	338 (295 to 387)	51 (30 to 81)	16 (16 to 16)
Gabon	2005	270 (244 to 298)	104 (90 to 119)	138 (120 to 158)	22 (13 to 35)	6 (6 to 6)	654 (592 to 723)	253 (218 to 289)	334 (290 to 383)	52 (31 to 85)	15 (15 to 15)
Gabon	2006	265 (239 to 292)	102 (87 to 117)	132 (115 to 152)	21 (12 to 33)	11 (11 to 11)	643 (579 to 709)	246 (212 to 283)	321 (278 to 368)	50 (29 to 79)	26 (26 to 26)
Gabon	2007	254 (229 to 280)	100 (87 to 115)	126 (109 to 146)	20 (12 to 32)	7 (7 to 7)	616 (556 to 679)	243 (210 to 280)	306 (265 to 354)	48 (29 to 77)	18 (18 to 18)
Gabon	2008	247 (223 to 271)	103 (89 to 118)	119 (102 to 136)	20 (12 to 32)	5 (5 to 5)	599 (540 to 658)	250 (217 to 287)	288 (248 to 330)	49 (29 to 77)	13 (13 to 13)
Gabon	2009	246 (221 to 270)	110 (96 to 126)	107 (92 to 123)	20 (13 to 32)	8 (8 to 8)	596 (536 to 654)	267 (234 to 306)	260 (222 to 299)	50 (30 to 78)	19 (19 to 19)
Gabon	2010	229 (206 to 252)	121 (106 to 138)	83 (70 to 97)	21 (13 to 32)	3 (3 to 3)	555 (499 to 611)	294 (256 to 334)	202 (171 to 235)	51 (31 to 78)	8 (8 to 8)
Gabon	2011	232 (209 to 257)	131 (114 to 148)	75 (63 to 88)	22 (13 to 33)	4 (4 to 4)	562 (507 to 622)	318 (277 to 360)	182 (153 to 214)	53 (32 to 81)	9 (9 to 9)
Gabon	2012	238 (215 to 262)	140 (123 to 157)	72 (60 to 86)	23 (14 to 35)	4 (4 to 4)	577 (521 to 636)	339 (297 to 381)	173 (145 to 207)	55 (34 to 85)	9 (9 to 9)
Gabon	2013	298 (269 to 330)	184 (162 to 208)	79 (66 to 95)	29 (18 to 45)	6 (6 to 6)	723 (651 to 799)	445 (392 to 504)	192 (160 to 232)	71 (44 to 110)	14 (14 to 14)
Gabon	2014	253 (228 to 282)	151 (133 to 171)	69 (57 to 84)	27 (17 to 42)	6 (6 to 6)	614 (554 to 684)	367 (323 to 415)	168 (139 to 203)	65 (41 to 102)	14 (14 to 14)

Gabon	2015	257 (229 to 288)	156 (137 to 178)	68 (56 to 82)	31 (19 to 48)	3 (3 to 3)	624 (555 to 699)	378 (333 to 432)	164 (135 to 200)	74 (45 to 117)	7 (7 to 7)
Gabon	2016	279 (247 to 315)	173 (151 to 199)	70 (56 to 86)	34 (20 to 55)	3 (3 to 3)	678 (598 to 764)	419 (365 to 482)	169 (137 to 209)	82 (49 to 134)	8 (8 to 8)
Gabon	2017	289 (253 to 338)	160 (137 to 186)	66 (53 to 82)	30 (18 to 50)	33 (33 to 33)	702 (612 to 819)	387 (332 to 450)	161 (128 to 199)	74 (43 to 122)	80 (80 to 80)
Gabon	2018	283 (245 to 332)	155 (132 to 184)	65 (52 to 81)	30 (18 to 50)	32 (32 to 32)	686 (593 to 806)	377 (320 to 445)	159 (126 to 197)	73 (43 to 121)	77 (77 to 77)
Gabon	2019	288 (249 to 339)	158 (134 to 187)	67 (53 to 83)	31 (18 to 51)	32 (32 to 32)	697 (603 to 821)	384 (326 to 454)	161 (128 to 200)	74 (44 to 124)	78 (78 to 78)
Gabon	2020	298 (258 to 349)	165 (140 to 196)	68 (54 to 85)	32 (19 to 53)	32 (31 to 34)	722 (625 to 846)	401 (340 to 475)	165 (131 to 205)	77 (45 to 129)	79 (75 to 83)
Gabon	2021	306 (266 to 360)	172 (145 to 203)	69 (55 to 86)	33 (19 to 55)	33 (31 to 35)	743 (645 to 873)	416 (352 to 491)	167 (133 to 208)	79 (46 to 133)	80 (75 to 86)
Gabon	2022	314 (272 to 372)	177 (149 to 209)	70 (55 to 87)	34 (20 to 56)	33 (30 to 37)	762 (660 to 902)	430 (362 to 507)	169 (135 to 210)	81 (48 to 136)	81 (74 to 89)
Gabon	2023	322 (279 to 381)	183 (154 to 216)	71 (56 to 88)	34 (20 to 58)	33 (30 to 37)	780 (676 to 924)	444 (372 to 525)	171 (136 to 213)	83 (49 to 139)	81 (73 to 91)
Gabon	2024	330 (284 to 391)	189 (159 to 224)	72 (57 to 89)	35 (21 to 59)	34 (30 to 38)	800 (688 to 947)	459 (385 to 542)	174 (137 to 216)	85 (50 to 143)	82 (73 to 92)
Gabon	2025	339 (290 to 400)	196 (164 to 232)	73 (57 to 90)	36 (21 to 61)	34 (30 to 39)	821 (704 to 970)	475 (398 to 562)	176 (139 to 219)	88 (51 to 147)	82 (73 to 94)
Gabon	2026	348 (299 to 412)	203 (170 to 241)	73 (58 to 91)	37 (22 to 62)	34 (30 to 39)	843 (724 to 1000)	492 (413 to 584)	178 (141 to 222)	90 (53 to 151)	83 (73 to 95)
Gabon	2027	357 (306 to 422)	210 (175 to 250)	74 (59 to 92)	38 (22 to 64)	35 (30 to 40)	865 (742 to 1024)	508 (424 to 605)	180 (143 to 224)	92 (54 to 155)	84 (74 to 98)
Gabon	2028	366 (313 to 435)	217 (181 to 259)	75 (60 to 94)	39 (23 to 66)	35 (30 to 41)	888 (758 to 1054)	526 (438 to 628)	183 (145 to 227)	95 (55 to 160)	85 (74 to 99)
Gabon	2029	376 (319 to 450)	224 (186 to 269)	76 (60 to 95)	40 (23 to 68)	35 (31 to 41)	912 (775 to 1090)	544 (451 to 652)	185 (146 to 230)	97 (57 to 164)	86 (74 to 100)

Gabon	2030	386 (328 to 460)	232 (191 to 279)	77 (61 to 96)	41 (24 to 70)	36 (31 to 43)	936 (795 to 1115)	562 (462 to 677)	187 (148 to 234)	100 (58 to 169)	86 (74 to 104)
Gabon	2031	396 (334 to 471)	239 (195 to 289)	78 (62 to 98)	42 (25 to 71)	36 (31 to 43)	959 (810 to 1143)	580 (474 to 701)	190 (150 to 237)	102 (60 to 173)	88 (75 to 104)
Gabon	2032	405 (344 to 484)	246 (201 to 297)	79 (62 to 99)	43 (25 to 73)	37 (31 to 44)	982 (834 to 1174)	597 (487 to 721)	192 (152 to 240)	104 (61 to 176)	89 (75 to 107)
Gabon	2033	415 (348 to 498)	254 (207 to 308)	80 (63 to 100)	44 (26 to 74)	37 (32 to 45)	1007 (845 to 1207)	616 (501 to 746)	195 (153 to 243)	107 (62 to 180)	90 (77 to 109)
Gabon	2034	425 (356 to 509)	262 (212 to 317)	81 (64 to 101)	45 (26 to 76)	37 (31 to 46)	1031 (864 to 1233)	634 (515 to 769)	197 (155 to 246)	109 (64 to 184)	91 (76 to 111)
Gabon	2035	435 (364 to 524)	269 (218 to 327)	82 (65 to 103)	46 (27 to 77)	38 (32 to 46)	1056 (883 to 1269)	653 (529 to 792)	199 (157 to 249)	112 (65 to 187)	92 (77 to 112)
Gabon	2036	445 (371 to 538)	276 (223 to 337)	83 (65 to 104)	47 (27 to 79)	38 (32 to 48)	1079 (899 to 1305)	670 (541 to 817)	202 (158 to 253)	114 (67 to 191)	93 (77 to 117)
Gabon	2037	455 (375 to 551)	283 (228 to 347)	84 (66 to 106)	48 (28 to 80)	39 (32 to 48)	1102 (910 to 1337)	687 (553 to 841)	204 (160 to 256)	116 (68 to 194)	95 (78 to 116)
Gabon	2038	464 (384 to 560)	290 (234 to 356)	85 (67 to 107)	49 (29 to 82)	40 (33 to 50)	1125 (932 to 1357)	704 (566 to 863)	206 (162 to 258)	119 (69 to 198)	96 (79 to 120)
Gabon	2039	472 (390 to 572)	297 (238 to 364)	86 (67 to 108)	50 (29 to 83)	40 (33 to 51)	1145 (946 to 1387)	719 (577 to 882)	208 (163 to 261)	121 (71 to 202)	97 (80 to 123)
Gabon	2040	481 (399 to 583)	302 (242 to 371)	87 (68 to 109)	51 (30 to 85)	41 (33 to 51)	1165 (968 to 1413)	733 (586 to 900)	210 (165 to 264)	123 (72 to 206)	99 (80 to 124)

Gabon	2041	488 (405 to 596)	308 (245 to 379)	88 (69 to 110)	51 (30 to 86)	41 (34 to 52)	1184 (982 to 1444)	747 (595 to 919)	212 (166 to 266)	125 (73 to 210)	100 (82 to 126)
Gabon	2042	496 (408 to 607)	313 (249 to 386)	88 (69 to 111)	52 (31 to 88)	42 (34 to 53)	1202 (988 to 1471)	760 (603 to 935)	214 (168 to 268)	126 (74 to 213)	102 (83 to 129)
Gabon	2043	502 (416 to 611)	318 (252 to 391)	89 (70 to 111)	53 (31 to 89)	42 (34 to 55)	1217 (1009 to 1481)	771 (610 to 948)	216 (169 to 270)	128 (75 to 215)	103 (83 to 134)
Gabon	2044	508 (420 to 621)	322 (255 to 396)	90 (70 to 112)	53 (31 to 90)	43 (35 to 57)	1233 (1018 to 1506)	781 (617 to 961)	218 (170 to 272)	129 (76 to 218)	105 (84 to 138)
Gabon	2045	514 (423 to 627)	326 (258 to 402)	90 (71 to 113)	54 (32 to 91)	44 (35 to 56)	1247 (1026 to 1521)	791 (624 to 974)	219 (171 to 275)	131 (76 to 220)	106 (84 to 136)
Gabon	2046	520 (430 to 630)	330 (260 to 407)	91 (71 to 114)	54 (32 to 91)	44 (35 to 57)	1260 (1042 to 1528)	799 (630 to 986)	221 (172 to 277)	132 (77 to 222)	108 (86 to 138)
Gabon	2047	524 (432 to 638)	333 (262 to 411)	92 (71 to 115)	55 (32 to 92)	45 (36 to 60)	1271 (1048 to 1548)	807 (634 to 996)	222 (173 to 279)	133 (78 to 223)	109 (87 to 145)
Gabon	2048	528 (438 to 646)	335 (263 to 414)	92 (72 to 116)	55 (32 to 93)	46 (36 to 61)	1281 (1061 to 1568)	813 (638 to 1004)	224 (174 to 282)	134 (78 to 225)	111 (88 to 147)
Gabon	2049	533 (438 to 650)	338 (265 to 417)	93 (72 to 117)	56 (33 to 93)	46 (37 to 62)	1292 (1062 to 1575)	819 (642 to 1011)	225 (175 to 284)	135 (79 to 226)	113 (89 to 151)
Gabon	2050	537 (445 to 656)	340 (267 to 420)	94 (73 to 118)	56 (33 to 94)	47 (37 to 64)	1303 (1079 to 1590)	826 (646 to 1019)	227 (176 to 286)	136 (80 to 228)	114 (89 to 156)

Georgia	1995	50 (39 to 62)	3 (2 to 4)	47 (36 to 59)	0 (0 to 0)	0 (0 to 0)	142 (112 to 177)	8 (6 to 11)	133 (102 to 168)	0 (0 to 0)	0 (0 to 0)
Georgia	1996	67 (54 to 81)	6 (4 to 8)	60 (47 to 75)	0 (0 to 0)	1 (1 to 1)	189 (153 to 230)	17 (13 to 22)	170 (135 to 213)	0 (0 to 0)	2 (2 to 2)
Georgia	1997	79 (65 to 94)	9 (7 to 12)	69 (55 to 84)	0 (0 to 0)	1 (1 to 1)	224 (185 to 266)	27 (20 to 35)	195 (156 to 237)	0 (0 to 0)	2 (2 to 2)
Georgia	1998	78 (65 to 91)	10 (8 to 13)	66 (54 to 80)	0 (0 to 0)	2 (2 to 2)	220 (184 to 259)	29 (22 to 36)	187 (153 to 226)	0 (0 to 0)	5 (5 to 5)
Georgia	1999	83 (71 to 96)	11 (8 to 14)	68 (56 to 81)	0 (0 to 0)	4 (4 to 4)	234 (200 to 273)	31 (24 to 39)	192 (159 to 229)	0 (0 to 0)	12 (12 to 12)
Georgia	2000	86 (74 to 99)	11 (9 to 14)	70 (59 to 84)	0 (0 to 0)	4 (4 to 4)	243 (210 to 282)	32 (25 to 40)	200 (167 to 237)	0 (0 to 0)	11 (11 to 11)
Georgia	2001	96 (84 to 110)	13 (10 to 17)	78 (66 to 92)	0 (0 to 0)	4 (4 to 4)	271 (237 to 311)	38 (30 to 47)	221 (186 to 260)	0 (0 to 1)	12 (12 to 12)
Georgia	2002	107 (94 to 123)	15 (12 to 19)	87 (74 to 102)	0 (0 to 1)	5 (5 to 5)	303 (266 to 349)	42 (33 to 53)	245 (209 to 290)	1 (1 to 2)	14 (14 to 14)
Georgia	2003	119 (105 to 136)	17 (13 to 21)	98 (84 to 115)	1 (0 to 1)	3 (3 to 3)	338 (297 to 386)	49 (38 to 60)	279 (240 to 325)	2 (1 to 4)	8 (8 to 8)
Georgia	2004	136 (120 to 154)	20 (16 to 25)	111 (95 to 128)	1 (1 to 2)	4 (4 to 4)	385 (340 to 436)	58 (46 to 71)	314 (271 to 364)	4 (2 to 7)	10 (10 to 10)
Georgia	2005	157 (139 to 177)	24 (19 to 30)	122 (106 to 142)	2 (1 to 4)	8 (8 to 8)	444 (393 to 502)	68 (54 to 84)	347 (301 to 401)	6 (3 to 11)	23 (23 to 23)
Georgia	2006	171 (152 to 193)	27 (22 to 33)	131 (114 to 151)	3 (1 to 6)	10 (10 to 10)	484 (430 to 546)	77 (61 to 94)	371 (323 to 428)	9 (4 to 16)	28 (28 to 28)
Georgia	2007	196 (176 to 219)	34 (27 to 42)	147 (128 to 169)	5 (3 to 10)	11 (11 to 11)	557 (499 to 620)	97 (78 to 118)	416 (362 to 478)	15 (7 to 28)	30 (30 to 30)
Georgia	2008	217 (195 to 242)	42 (34 to 51)	160 (140 to 184)	8 (4 to 14)	7 (7 to 7)	615 (552 to 685)	119 (97 to 144)	454 (398 to 523)	21 (11 to 40)	21 (21 to 21)
Georgia	2009	242 (218 to 267)	49 (40 to 58)	173 (152 to 197)	9 (4 to 16)	11 (11 to 11)	686 (619 to 757)	138 (114 to 166)	491 (430 to 559)	25 (12 to 45)	32 (32 to 32)

Georgia	2010	248 (226 to 273)	51 (42 to 61)	179 (158 to 201)	8 (4 to 15)	11 (11 to 11)	704 (641 to 774)	143 (118 to 172)	506 (447 to 571)	24 (12 to 41)	31 (31 to 31)
Georgia	2011	248 (226 to 273)	50 (41 to 60)	177 (157 to 201)	9 (5 to 15)	12 (12 to 12)	704 (640 to 774)	143 (117 to 170)	503 (444 to 569)	25 (13 to 43)	34 (34 to 34)
Georgia	2012	263 (239 to 288)	57 (47 to 68)	183 (162 to 206)	10 (5 to 17)	12 (12 to 12)	745 (677 to 815)	163 (134 to 193)	519 (458 to 583)	28 (14 to 49)	34 (34 to 34)
Georgia	2013	268 (242 to 294)	66 (55 to 78)	183 (162 to 207)	11 (6 to 19)	7 (7 to 7)	759 (687 to 833)	187 (156 to 221)	520 (459 to 586)	32 (17 to 54)	19 (19 to 19)
Georgia	2014	283 (256 to 312)	80 (67 to 94)	184 (163 to 208)	13 (6 to 22)	6 (6 to 6)	802 (727 to 885)	226 (191 to 268)	522 (461 to 590)	36 (18 to 61)	18 (18 to 18)
Georgia	2015	292 (264 to 325)	92 (77 to 109)	177 (156 to 203)	14 (7 to 24)	9 (9 to 9)	828 (750 to 922)	261 (219 to 309)	502 (442 to 574)	39 (20 to 68)	25 (25 to 25)
Georgia	2016	300 (269 to 336)	104 (86 to 124)	176 (151 to 204)	16 (8 to 28)	4 (4 to 4)	850 (762 to 954)	294 (244 to 352)	498 (428 to 579)	45 (23 to 79)	13 (13 to 13)
Georgia	2017	307 (267 to 354)	110 (91 to 132)	173 (145 to 205)	17 (9 to 30)	7 (7 to 7)	870 (757 to 1003)	312 (258 to 375)	490 (412 to 581)	48 (24 to 85)	19 (19 to 19)
Georgia	2018	320 (278 to 368)	118 (97 to 142)	178 (149 to 211)	18 (9 to 32)	7 (7 to 7)	908 (788 to 1045)	335 (276 to 402)	504 (424 to 597)	51 (26 to 90)	19 (19 to 19)
Georgia	2019	338 (294 to 389)	129 (106 to 154)	183 (154 to 217)	19 (10 to 34)	7 (7 to 7)	959 (834 to 1102)	366 (301 to 438)	519 (436 to 615)	54 (27 to 96)	19 (19 to 20)
Georgia	2020	358 (310 to 411)	140 (115 to 167)	190 (159 to 225)	20 (10 to 36)	7 (7 to 7)	1014 (880 to 1166)	397 (326 to 475)	538 (452 to 638)	58 (29 to 102)	20 (19 to 21)
Georgia	2021	371 (321 to 427)	149 (123 to 178)	193 (162 to 229)	21 (11 to 38)	7 (7 to 8)	1053 (910 to 1209)	423 (348 to 504)	548 (461 to 650)	61 (31 to 107)	21 (19 to 22)
Georgia	2022	385 (335 to 442)	159 (131 to 189)	196 (165 to 232)	22 (11 to 40)	7 (7 to 8)	1090 (949 to 1253)	450 (371 to 536)	556 (469 to 659)	63 (32 to 112)	21 (19 to 23)

Georgia	2023	398 (344 to 460)	168 (138 to 201)	199 (167 to 235)	23 (12 to 41)	8 (7 to 9)	1129 (976 to 1304)	478 (392 to 569)	563 (475 to 667)	66 (34 to 117)	22 (20 to 24)
Georgia	2024	412 (359 to 474)	179 (147 to 213)	201 (169 to 238)	24 (12 to 43)	8 (7 to 9)	1168 (1018 to 1343)	507 (416 to 604)	570 (480 to 676)	69 (35 to 122)	22 (20 to 25)
Georgia	2025	427 (369 to 494)	190 (156 to 226)	204 (171 to 242)	25 (13 to 45)	8 (7 to 9)	1210 (1047 to 1400)	538 (443 to 641)	577 (486 to 685)	72 (36 to 127)	23 (20 to 26)
Georgia	2026	442 (382 to 512)	201 (166 to 240)	206 (174 to 244)	27 (13 to 47)	8 (7 to 9)	1254 (1084 to 1452)	571 (470 to 680)	585 (492 to 693)	75 (38 to 133)	23 (20 to 27)
Georgia	2027	460 (400 to 532)	214 (177 to 255)	209 (176 to 248)	28 (14 to 49)	8 (7 to 10)	1303 (1134 to 1508)	607 (502 to 723)	593 (499 to 703)	79 (40 to 138)	24 (21 to 28)
Georgia	2028	479 (413 to 553)	228 (188 to 272)	213 (179 to 252)	29 (15 to 51)	9 (7 to 10)	1357 (1171 to 1567)	647 (533 to 771)	603 (507 to 716)	83 (42 to 145)	24 (21 to 28)
Georgia	2029	499 (431 to 578)	243 (201 to 290)	217 (182 to 257)	31 (15 to 54)	9 (8 to 10)	1416 (1222 to 1640)	690 (569 to 822)	614 (517 to 728)	87 (44 to 152)	25 (21 to 29)
Georgia	2030	521 (449 to 606)	259 (214 to 310)	221 (186 to 262)	32 (16 to 56)	9 (8 to 11)	1477 (1274 to 1718)	735 (607 to 879)	626 (527 to 742)	91 (46 to 160)	25 (22 to 30)
Georgia	2031	544 (466 to 632)	276 (228 to 331)	225 (190 to 267)	34 (17 to 59)	9 (8 to 11)	1543 (1322 to 1792)	783 (648 to 939)	639 (538 to 757)	96 (48 to 168)	26 (22 to 31)
Georgia	2032	568 (486 to 665)	294 (243 to 352)	230 (194 to 273)	36 (18 to 62)	9 (8 to 11)	1612 (1379 to 1884)	833 (689 to 998)	652 (549 to 773)	101 (51 to 176)	26 (22 to 32)

Georgia	2033	593 (508 to 699)	312 (257 to 374)	235 (198 to 278)	37 (19 to 65)	9 (8 to 12)	1682 (1442 to 1982)	884 (730 to 1062)	665 (561 to 789)	106 (53 to 185)	27 (23 to 33)
Georgia	2034	619 (528 to 727)	331 (272 to 398)	240 (202 to 285)	39 (20 to 68)	10 (8 to 12)	1756 (1498 to 2062)	938 (772 to 1127)	680 (573 to 807)	111 (56 to 194)	27 (23 to 33)
Georgia	2035	646 (550 to 755)	350 (288 to 422)	245 (206 to 291)	41 (21 to 72)	10 (8 to 12)	1832 (1561 to 2140)	994 (816 to 1195)	694 (585 to 826)	116 (59 to 203)	28 (23 to 34)
Georgia	2036	674 (573 to 792)	370 (304 to 445)	250 (211 to 298)	43 (22 to 75)	10 (8 to 13)	1910 (1626 to 2247)	1051 (861 to 1263)	709 (598 to 845)	121 (61 to 213)	28 (23 to 36)
Georgia	2037	701 (598 to 821)	391 (320 to 470)	255 (215 to 304)	45 (23 to 78)	10 (8 to 13)	1987 (1696 to 2327)	1107 (907 to 1333)	724 (609 to 863)	127 (64 to 222)	29 (24 to 36)
Georgia	2038	729 (619 to 855)	411 (336 to 495)	260 (219 to 311)	47 (24 to 82)	10 (9 to 13)	2066 (1754 to 2425)	1165 (953 to 1404)	738 (621 to 882)	132 (67 to 232)	30 (24 to 37)
Georgia	2039	757 (642 to 892)	432 (353 to 521)	266 (223 to 318)	49 (25 to 85)	11 (9 to 14)	2146 (1819 to 2530)	1224 (1000 to 1477)	753 (633 to 901)	138 (70 to 242)	30 (25 to 38)
Georgia	2040	785 (666 to 922)	452 (370 to 546)	271 (228 to 324)	51 (26 to 89)	11 (9 to 14)	2226 (1888 to 2614)	1283 (1048 to 1549)	768 (646 to 919)	143 (73 to 252)	31 (25 to 39)
Georgia	2041	813 (687 to 957)	473 (386 to 572)	276 (232 to 330)	53 (27 to 92)	11 (9 to 14)	2305 (1948 to 2714)	1341 (1095 to 1621)	783 (657 to 937)	149 (76 to 261)	31 (26 to 40)
Georgia	2042	841 (710 to 987)	494 (403 to 597)	281 (236 to 337)	55 (28 to 96)	11 (9 to 15)	2384 (2014 to 2798)	1400 (1143 to 1694)	797 (668 to 955)	155 (78 to 271)	32 (26 to 42)

Georgia	2043	868 (727 to 1029)	514 (419 to 622)	286 (240 to 343)	56 (28 to 99)	12 (9 to 15)	2461 (2061 to 2919)	1457 (1189 to 1764)	812 (680 to 973)	160 (81 to 281)	33 (26 to 43)
Georgia	2044	895 (758 to 1056)	534 (436 to 647)	291 (244 to 350)	58 (29 to 102)	12 (9 to 16)	2538 (2151 to 2994)	1514 (1235 to 1834)	826 (691 to 991)	165 (83 to 290)	33 (27 to 44)
Georgia	2045	921 (773 to 1092)	553 (451 to 670)	296 (248 to 356)	60 (30 to 106)	12 (9 to 16)	2612 (2193 to 3096)	1568 (1279 to 1901)	840 (702 to 1009)	171 (85 to 299)	34 (27 to 44)
Georgia	2046	947 (796 to 1121)	572 (467 to 694)	301 (252 to 361)	62 (31 to 109)	12 (10 to 16)	2686 (2258 to 3179)	1622 (1323 to 1967)	854 (714 to 1024)	176 (88 to 309)	35 (27 to 45)
Georgia	2047	973 (818 to 1148)	590 (481 to 716)	306 (256 to 367)	64 (32 to 112)	12 (10 to 17)	2758 (2321 to 3255)	1674 (1365 to 2030)	868 (726 to 1042)	181 (90 to 317)	35 (27 to 48)
Georgia	2048	997 (838 to 1189)	608 (496 to 737)	311 (260 to 374)	66 (33 to 115)	13 (10 to 17)	2828 (2377 to 3372)	1724 (1405 to 2090)	881 (738 to 1060)	186 (93 to 326)	36 (28 to 49)
Georgia	2049	1021 (856 to 1204)	625 (509 to 757)	316 (264 to 380)	67 (34 to 118)	13 (10 to 18)	2895 (2428 to 3413)	1772 (1443 to 2147)	895 (748 to 1079)	191 (95 to 335)	37 (28 to 50)
Georgia	2050	1044 (875 to 1251)	641 (522 to 777)	320 (268 to 387)	69 (34 to 121)	13 (10 to 18)	2961 (2480 to 3548)	1819 (1481 to 2205)	909 (759 to 1097)	196 (98 to 343)	38 (29 to 51)
Germany	1995	3178 (3090 to 3267)	2565 (2491 to 2639)	337 (305 to 370)	276 (243 to 309)	0 (0 to 0)	3647 (3545 to 3748)	2943 (2858 to 3028)	387 (350 to 425)	317 (279 to 354)	0 (0 to 0)
Germany	1996	3240 (3170 to 3308)	2610 (2552 to 2664)	350 (325 to 376)	280 (256 to 305)	0 (0 to 0)	3717 (3637 to 3796)	2995 (2928 to 3056)	401 (373 to 431)	321 (294 to 350)	0 (0 to 0)

Germany	1997	3376 (3309 to 3445)	2703 (2648 to 2759)	374 (351 to 398)	299 (275 to 324)	0 (0 to 0)	3873 (3797 to 3953)	3102 (3038 to 3166)	429 (403 to 456)	343 (316 to 371)	0 (0 to 0)
Germany	1998	3462 (3397 to 3525)	2751 (2698 to 2806)	397 (374 to 420)	314 (289 to 338)	0 (0 to 0)	3973 (3898 to 4044)	3157 (3096 to 3219)	455 (429 to 482)	360 (332 to 388)	0 (0 to 0)
Germany	1999	3553 (3497 to 3613)	2804 (2755 to 2855)	419 (397 to 443)	329 (307 to 353)	0 (0 to 0)	4077 (4012 to 4145)	3218 (3161 to 3276)	481 (456 to 508)	378 (352 to 405)	0 (0 to 0)
Germany	2000	3630 (3569 to 3692)	2847 (2793 to 2901)	441 (419 to 464)	342 (320 to 363)	0 (0 to 0)	4165 (4095 to 4236)	3267 (3205 to 3329)	506 (481 to 532)	392 (367 to 417)	0 (0 to 0)
Germany	2001	3743 (3685 to 3804)	2926 (2876 to 2979)	462 (440 to 483)	355 (333 to 376)	0 (0 to 0)	4295 (4228 to 4365)	3358 (3300 to 3418)	530 (505 to 554)	407 (382 to 432)	0 (0 to 0)
Germany	2002	3797 (3740 to 3854)	2954 (2902 to 3005)	481 (460 to 502)	362 (343 to 385)	0 (0 to 0)	4357 (4291 to 4422)	3390 (3329 to 3448)	552 (528 to 576)	416 (393 to 442)	0 (0 to 0)
Germany	2003	3841 (3781 to 3899)	2966 (2916 to 3021)	502 (481 to 523)	373 (352 to 395)	0 (0 to 0)	4407 (4338 to 4474)	3404 (3346 to 3466)	576 (552 to 600)	428 (404 to 453)	0 (0 to 0)
Germany	2004	3823 (3765 to 3884)	2920 (2868 to 2970)	524 (501 to 546)	379 (357 to 402)	0 (0 to 0)	4387 (4320 to 4456)	3350 (3291 to 3408)	602 (575 to 626)	435 (410 to 461)	0 (0 to 0)
Germany	2005	3905 (3846 to 3971)	2960 (2910 to 3012)	551 (526 to 572)	394 (374 to 417)	0 (0 to 0)	4480 (4413 to 4557)	3396 (3339 to 3456)	632 (604 to 656)	452 (429 to 478)	0 (0 to 0)
Germany	2006	3996 (3939 to 4059)	3015 (2965 to 3068)	574 (549 to 596)	408 (387 to 431)	0 (0 to 0)	4585 (4520 to 4658)	3459 (3402 to 3520)	658 (630 to 684)	468 (444 to 495)	0 (0 to 0)

Germany	2007	4069 (4012 to 4135)	3064 (3013 to 3117)	586 (562 to 610)	419 (396 to 442)	0 (0 to 0)	4669 (4603 to 4745)	3516 (3457 to 3577)	673 (645 to 700)	481 (455 to 507)	0 (0 to 0)
Germany	2008	4215 (4154 to 4277)	3178 (3124 to 3232)	600 (575 to 625)	437 (415 to 459)	0 (0 to 0)	4837 (4766 to 4907)	3647 (3584 to 3709)	688 (660 to 717)	501 (477 to 527)	0 (0 to 0)
Germany	2009	4364 (4301 to 4430)	3299 (3249 to 3352)	614 (590 to 639)	451 (427 to 476)	0 (0 to 0)	5008 (4935 to 5083)	3785 (3728 to 3847)	705 (677 to 733)	518 (490 to 546)	0 (0 to 0)
Germany	2010	4504 (4443 to 4573)	3409 (3358 to 3467)	630 (605 to 655)	466 (440 to 491)	0 (0 to 0)	5168 (5098 to 5248)	3911 (3853 to 3978)	723 (695 to 751)	534 (505 to 563)	0 (0 to 0)
Germany	2011	4531 (4469 to 4594)	3425 (3372 to 3476)	634 (612 to 658)	472 (447 to 497)	0 (0 to 0)	5199 (5128 to 5272)	3930 (3869 to 3989)	728 (702 to 754)	541 (513 to 571)	0 (0 to 0)
Germany	2012	4574 (4507 to 4640)	3466 (3412 to 3523)	633 (609 to 658)	476 (450 to 502)	0 (0 to 0)	5249 (5171 to 5324)	3977 (3915 to 4043)	726 (698 to 755)	546 (516 to 576)	0 (0 to 0)
Germany	2013	4668 (4602 to 4734)	3560 (3507 to 3616)	626 (602 to 651)	482 (456 to 508)	0 (0 to 0)	5356 (5280 to 5432)	4085 (4024 to 4149)	718 (691 to 747)	553 (523 to 583)	0 (0 to 0)
Germany	2014	4754 (4687 to 4824)	3649 (3593 to 3708)	622 (597 to 647)	483 (459 to 508)	0 (0 to 0)	5455 (5378 to 5536)	4187 (4123 to 4255)	713 (685 to 742)	555 (527 to 583)	0 (0 to 0)
Germany	2015	4849 (4784 to 4912)	3736 (3680 to 3794)	627 (603 to 651)	486 (462 to 511)	0 (0 to 0)	5564 (5489 to 5636)	4286 (4223 to 4354)	719 (692 to 746)	558 (530 to 587)	0 (0 to 0)
Germany	2016	4971 (4906 to 5037)	3844 (3788 to 3898)	637 (613 to 664)	490 (465 to 516)	0 (0 to 0)	5704 (5629 to 5780)	4411 (4346 to 4473)	731 (704 to 762)	562 (533 to 592)	0 (0 to 0)

Germany	2017	5110 (4991 to 5225)	3965 (3890 to 4041)	649 (615 to 686)	496 (463 to 531)	0 (0 to 0)	5864 (5727 to 5995)	4550 (4464 to 4637)	745 (706 to 787)	569 (531 to 609)	0 (0 to 0)
Germany	2018	5224 (5115 to 5349)	4061 (3981 to 4144)	656 (621 to 694)	507 (473 to 542)	0 (0 to 0)	5994 (5869 to 6137)	4660 (4568 to 4755)	753 (713 to 796)	581 (542 to 622)	0 (0 to 0)
Germany	2019	5337 (5193 to 5473)	4161 (4064 to 4261)	659 (625 to 698)	516 (482 to 553)	0 (0 to 0)	6124 (5959 to 6280)	4774 (4663 to 4889)	757 (717 to 800)	593 (553 to 634)	0 (0 to 0)
Germany	2020	5448 (5310 to 5601)	4254 (4148 to 4368)	667 (632 to 705)	527 (491 to 564)	0 (0 to 0)	6251 (6093 to 6426)	4881 (4760 to 5012)	766 (726 to 809)	604 (563 to 647)	0 (0 to 0)
Germany	2021	5518 (5362 to 5671)	4313 (4199 to 4428)	671 (636 to 710)	534 (496 to 570)	0 (0 to 0)	6332 (6152 to 6507)	4949 (4818 to 5081)	770 (730 to 815)	612 (570 to 654)	0 (0 to 0)
Germany	2022	5588 (5433 to 5751)	4373 (4249 to 4490)	675 (639 to 714)	540 (502 to 578)	0 (0 to 0)	6412 (6233 to 6599)	5018 (4876 to 5152)	775 (734 to 819)	620 (576 to 663)	0 (0 to 0)
Germany	2023	5670 (5488 to 5840)	4444 (4301 to 4573)	678 (643 to 717)	548 (509 to 586)	0 (0 to 0)	6506 (6297 to 6701)	5099 (4936 to 5248)	778 (737 to 823)	629 (584 to 672)	0 (0 to 0)
Germany	2024	5747 (5549 to 5939)	4510 (4342 to 4658)	681 (645 to 721)	555 (515 to 594)	0 (0 to 0)	6594 (6367 to 6815)	5175 (4982 to 5345)	782 (741 to 827)	637 (591 to 681)	0 (0 to 0)
Germany	2025	5821 (5588 to 6029)	4576 (4388 to 4749)	684 (648 to 724)	561 (521 to 600)	0 (0 to 0)	6679 (6412 to 6918)	5251 (5035 to 5449)	785 (744 to 831)	644 (597 to 688)	0 (0 to 0)
Germany	2026	5893 (5647 to 6131)	4640 (4431 to 4840)	686 (650 to 726)	567 (526 to 606)	0 (0 to 0)	6762 (6479 to 7035)	5324 (5084 to 5554)	787 (746 to 833)	650 (604 to 696)	0 (0 to 0)

Germany	2027	5966 (5673 to 6225)	4705 (4470 to 4928)	689 (653 to 730)	573 (532 to 613)	0 (0 to 0)	6846 (6510 to 7143)	5398 (5129 to 5655)	790 (749 to 837)	657 (610 to 704)	0 (0 to 0)
Germany	2028	6035 (5730 to 6325)	4766 (4503 to 5014)	691 (655 to 732)	578 (538 to 619)	0 (0 to 0)	6925 (6575 to 7258)	5469 (5167 to 5753)	792 (751 to 840)	664 (617 to 711)	0 (0 to 0)
Germany	2029	6099 (5762 to 6415)	4824 (4528 to 5104)	692 (655 to 733)	583 (542 to 626)	0 (0 to 0)	6998 (6611 to 7360)	5535 (5195 to 5856)	794 (752 to 841)	669 (622 to 718)	0 (0 to 0)
Germany	2030	6162 (5794 to 6512)	4881 (4550 to 5190)	693 (656 to 735)	588 (547 to 632)	0 (0 to 0)	7070 (6648 to 7472)	5600 (5221 to 5955)	795 (753 to 843)	675 (628 to 725)	0 (0 to 0)
Germany	2031	6228 (5824 to 6611)	4939 (4583 to 5278)	695 (657 to 737)	594 (551 to 638)	0 (0 to 0)	7146 (6682 to 7586)	5668 (5259 to 6056)	797 (753 to 846)	681 (633 to 732)	0 (0 to 0)
Germany	2032	6301 (5874 to 6717)	5004 (4620 to 5371)	697 (658 to 741)	600 (556 to 645)	0 (0 to 0)	7230 (6739 to 7708)	5742 (5302 to 6162)	800 (755 to 850)	688 (638 to 740)	0 (0 to 0)
Germany	2033	6379 (5914 to 6815)	5072 (4661 to 5463)	700 (661 to 745)	606 (561 to 653)	0 (0 to 0)	7319 (6786 to 7820)	5820 (5348 to 6269)	803 (758 to 854)	696 (644 to 749)	0 (0 to 0)
Germany	2034	6459 (5987 to 6924)	5142 (4707 to 5556)	703 (664 to 749)	614 (567 to 663)	0 (0 to 0)	7412 (6869 to 7945)	5901 (5401 to 6375)	807 (761 to 859)	704 (650 to 760)	0 (0 to 0)
Germany	2035	6545 (6030 to 7034)	5216 (4755 to 5655)	707 (667 to 753)	621 (573 to 673)	0 (0 to 0)	7510 (6919 to 8071)	5986 (5456 to 6488)	812 (765 to 865)	713 (658 to 772)	0 (0 to 0)
Germany	2036	6638 (6102 to 7162)	5297 (4811 to 5762)	712 (670 to 759)	630 (580 to 683)	0 (0 to 0)	7617 (7002 to 8218)	6077 (5521 to 6612)	817 (768 to 871)	723 (665 to 784)	0 (0 to 0)

Germany	2037	6742 (6158 to 7291)	5385 (4873 to 5877)	718 (675 to 766)	639 (587 to 696)	0 (0 to 0)	7736 (7065 to 8365)	6179 (5592 to 6744)	824 (774 to 879)	734 (673 to 798)	0 (0 to 0)
Germany	2038	6858 (6273 to 7438)	5482 (4945 to 6001)	725 (681 to 775)	650 (595 to 710)	0 (0 to 0)	7869 (7198 to 8534)	6290 (5674 to 6885)	832 (782 to 889)	746 (683 to 814)	0 (0 to 0)
Germany	2039	6985 (6340 to 7586)	5589 (5027 to 6135)	734 (689 to 784)	662 (604 to 726)	0 (0 to 0)	8015 (7275 to 8704)	6413 (5768 to 7039)	842 (790 to 900)	760 (693 to 833)	0 (0 to 0)
Germany	2040	7119 (6473 to 7773)	5701 (5114 to 6274)	743 (697 to 795)	675 (615 to 742)	0 (0 to 0)	8169 (7427 to 8919)	6542 (5868 to 7199)	852 (799 to 912)	775 (706 to 852)	0 (0 to 0)
Germany	2041	7258 (6571 to 7902)	5816 (5206 to 6414)	752 (705 to 806)	689 (626 to 758)	0 (0 to 0)	8328 (7540 to 9068)	6674 (5974 to 7359)	863 (809 to 925)	791 (718 to 870)	0 (0 to 0)
Germany	2042	7393 (6700 to 8087)	5929 (5297 to 6554)	762 (714 to 816)	702 (636 to 775)	0 (0 to 0)	8483 (7688 to 9279)	6803 (6078 to 7520)	874 (819 to 937)	806 (729 to 889)	0 (0 to 0)
Germany	2043	7528 (6796 to 8244)	6041 (5386 to 6692)	771 (721 to 827)	716 (646 to 792)	0 (0 to 0)	8637 (7798 to 9459)	6932 (6181 to 7679)	884 (828 to 949)	821 (741 to 908)	0 (0 to 0)
Germany	2044	7661 (6930 to 8397)	6152 (5479 to 6821)	780 (728 to 839)	729 (656 to 810)	0 (0 to 0)	8791 (7952 to 9635)	7059 (6287 to 7827)	895 (836 to 962)	837 (753 to 929)	0 (0 to 0)
Germany	2045	7795 (7024 to 8550)	6263 (5571 to 6951)	789 (736 to 850)	743 (667 to 827)	0 (0 to 0)	8945 (8059 to 9810)	7186 (6392 to 7976)	905 (844 to 975)	853 (765 to 949)	0 (0 to 0)
Germany	2046	7930 (7153 to 8728)	6374 (5664 to 7084)	798 (745 to 862)	757 (678 to 845)	0 (0 to 0)	9099 (8208 to 10015)	7314 (6499 to 8129)	916 (854 to 989)	869 (778 to 970)	0 (0 to 0)

Germany	2047	8063 (7267 to 8876)	6484 (5757 to 7214)	807 (752 to 873)	771 (689 to 864)	0 (0 to 0)	9252 (8338 to 10185)	7440 (6606 to 8278)	927 (863 to 1002)	885 (791 to 992)	0 (0 to 0)
Germany	2048	8194 (7369 to 9017)	6592 (5849 to 7341)	817 (760 to 884)	786 (700 to 884)	0 (0 to 0)	9402 (8456 to 10347)	7563 (6712 to 8423)	937 (872 to 1014)	901 (803 to 1014)	0 (0 to 0)
Germany	2049	8322 (7458 to 9169)	6697 (5946 to 7463)	825 (766 to 895)	800 (710 to 903)	0 (0 to 0)	9549 (8557 to 10521)	7685 (6822 to 8564)	947 (879 to 1027)	918 (815 to 1036)	0 (0 to 0)
Germany	2050	8452 (7607 to 9302)	6803 (6040 to 7583)	834 (773 to 907)	814 (721 to 923)	0 (0 to 0)	9698 (8729 to 10673)	7806 (6931 to 8701)	957 (887 to 1041)	934 (827 to 1059)	0 (0 to 0)
Ghana	1995	28 (23 to 35)	9 (7 to 12)	15 (10 to 21)	3 (1 to 5)	1 (1 to 1)	90 (72 to 110)	29 (21 to 37)	47 (33 to 65)	9 (4 to 17)	5 (5 to 5)
Ghana	1996	29 (23 to 36)	9 (7 to 12)	15 (11 to 21)	3 (1 to 5)	2 (2 to 2)	92 (74 to 114)	30 (22 to 39)	48 (34 to 67)	9 (4 to 17)	5 (5 to 5)
Ghana	1997	33 (27 to 40)	9 (7 to 12)	16 (11 to 22)	3 (1 to 5)	5 (5 to 5)	104 (86 to 127)	29 (22 to 38)	49 (34 to 69)	9 (4 to 16)	17 (17 to 17)
Ghana	1998	30 (24 to 37)	9 (7 to 12)	16 (11 to 22)	3 (1 to 5)	2 (2 to 2)	94 (75 to 116)	29 (22 to 39)	49 (34 to 69)	9 (4 to 16)	6 (6 to 6)
Ghana	1999	31 (26 to 38)	9 (7 to 12)	16 (11 to 22)	3 (1 to 5)	4 (4 to 4)	99 (81 to 121)	29 (22 to 38)	50 (35 to 69)	9 (4 to 16)	11 (11 to 11)
Ghana	2000	32 (26 to 39)	10 (7 to 13)	16 (11 to 23)	3 (1 to 5)	3 (3 to 3)	101 (82 to 123)	30 (23 to 40)	52 (36 to 72)	8 (4 to 15)	10 (10 to 10)
Ghana	2001	36 (30 to 43)	11 (8 to 14)	17 (12 to 24)	3 (1 to 5)	5 (5 to 5)	112 (93 to 135)	33 (25 to 43)	54 (37 to 74)	9 (4 to 16)	17 (17 to 17)
Ghana	2002	32 (26 to 38)	9 (7 to 12)	16 (11 to 22)	2 (1 to 4)	4 (4 to 4)	99 (82 to 121)	28 (22 to 37)	50 (35 to 69)	7 (4 to 13)	14 (14 to 14)
Ghana	2003	37 (31 to 45)	10 (8 to 13)	17 (12 to 23)	3 (1 to 5)	7 (7 to 7)	117 (99 to 141)	33 (25 to 42)	53 (38 to 73)	8 (4 to 15)	23 (23 to 23)

Ghana	2004	44 (37 to 52)	12 (9 to 16)	18 (13 to 25)	3 (1 to 5)	11 (11 to 11)	138 (118 to 163)	39 (30 to 50)	57 (40 to 77)	9 (4 to 16)	34 (34 to 34)
Ghana	2005	46 (40 to 54)	14 (11 to 19)	18 (13 to 25)	3 (1 to 5)	11 (11 to 11)	146 (125 to 171)	45 (34 to 59)	58 (41 to 79)	9 (4 to 17)	34 (34 to 34)
Ghana	2006	49 (42 to 58)	17 (13 to 22)	20 (14 to 26)	3 (1 to 6)	9 (9 to 9)	155 (131 to 182)	54 (41 to 70)	62 (43 to 83)	10 (5 to 18)	29 (29 to 29)
Ghana	2007	55 (47 to 65)	21 (16 to 27)	21 (15 to 29)	4 (2 to 6)	9 (9 to 9)	175 (148 to 206)	67 (51 to 86)	67 (48 to 90)	11 (5 to 20)	30 (30 to 30)
Ghana	2008	61 (52 to 72)	24 (18 to 31)	22 (16 to 30)	4 (2 to 7)	11 (11 to 11)	194 (164 to 228)	76 (58 to 97)	70 (50 to 94)	12 (6 to 21)	35 (35 to 35)
Ghana	2009	65 (55 to 76)	26 (20 to 33)	23 (16 to 31)	4 (2 to 7)	12 (12 to 12)	204 (172 to 241)	83 (63 to 105)	72 (52 to 97)	12 (6 to 21)	37 (37 to 37)
Ghana	2010	70 (59 to 83)	31 (23 to 39)	25 (17 to 33)	4 (2 to 7)	10 (10 to 10)	220 (185 to 261)	97 (73 to 122)	77 (55 to 105)	12 (6 to 22)	33 (33 to 33)
Ghana	2011	74 (63 to 87)	33 (25 to 42)	27 (19 to 37)	4 (2 to 7)	11 (11 to 11)	233 (198 to 276)	104 (79 to 132)	84 (59 to 116)	12 (6 to 21)	34 (34 to 34)
Ghana	2012	81 (69 to 95)	35 (27 to 45)	29 (21 to 40)	4 (2 to 7)	13 (13 to 13)	257 (217 to 300)	111 (84 to 140)	92 (66 to 125)	12 (6 to 21)	42 (42 to 42)
Ghana	2013	80 (68 to 94)	34 (26 to 43)	31 (22 to 42)	4 (2 to 6)	12 (12 to 12)	254 (213 to 297)	106 (81 to 135)	97 (70 to 133)	11 (5 to 20)	39 (39 to 39)
Ghana	2014	75 (62 to 88)	31 (24 to 40)	31 (23 to 43)	4 (2 to 7)	9 (9 to 9)	237 (197 to 279)	98 (75 to 125)	98 (71 to 134)	12 (6 to 22)	28 (28 to 28)
Ghana	2015	72 (61 to 85)	28 (21 to 36)	29 (21 to 40)	4 (2 to 8)	11 (11 to 11)	229 (192 to 269)	89 (68 to 112)	92 (67 to 127)	14 (6 to 25)	34 (34 to 34)
Ghana	2016	70 (59 to 84)	27 (21 to 35)	28 (20 to 38)	6 (3 to 10)	9 (9 to 9)	221 (186 to 264)	86 (65 to 111)	88 (63 to 119)	18 (8 to 32)	30 (30 to 30)
Ghana	2017	66 (53 to 81)	25 (19 to 32)	27 (19 to 37)	6 (3 to 11)	9 (9 to 9)	208 (168 to 254)	78 (59 to 101)	84 (59 to 115)	19 (9 to 35)	27 (27 to 27)
Ghana	2018	72 (59 to 88)	30 (23 to 39)	27 (19 to 37)	6 (3 to 11)	8 (8 to 8)	227 (185 to 277)	96 (73 to 124)	86 (61 to 118)	20 (10 to 36)	26 (25 to 26)

Ghana	2019	77 (62 to 95)	34 (26 to 44)	28 (20 to 39)	7 (3 to 12)	8 (8 to 8)	244 (197 to 298)	107 (81 to 138)	90 (63 to 123)	21 (10 to 38)	25 (25 to 26)
Ghana	2020	80 (65 to 98)	36 (27 to 46)	29 (21 to 40)	7 (3 to 13)	8 (8 to 9)	252 (204 to 309)	112 (85 to 144)	93 (66 to 127)	22 (11 to 40)	25 (24 to 27)
Ghana	2021	82 (67 to 100)	37 (28 to 47)	30 (21 to 41)	7 (4 to 13)	8 (8 to 9)	260 (211 to 317)	117 (88 to 149)	95 (67 to 130)	23 (11 to 41)	26 (24 to 28)
Ghana	2022	85 (69 to 105)	39 (29 to 50)	31 (22 to 42)	7 (4 to 13)	8 (7 to 9)	268 (218 to 331)	122 (93 to 158)	97 (69 to 133)	23 (11 to 43)	26 (23 to 28)
Ghana	2023	88 (71 to 109)	41 (31 to 52)	32 (22 to 43)	8 (4 to 14)	8 (7 to 9)	277 (225 to 343)	128 (96 to 165)	99 (70 to 136)	24 (12 to 44)	26 (23 to 29)
Ghana	2024	91 (73 to 112)	42 (32 to 55)	32 (23 to 44)	8 (4 to 14)	8 (7 to 9)	286 (231 to 352)	134 (100 to 173)	102 (72 to 139)	25 (12 to 45)	26 (23 to 29)
Ghana	2025	94 (75 to 116)	44 (33 to 58)	33 (23 to 45)	8 (4 to 15)	8 (7 to 9)	295 (236 to 364)	140 (103 to 183)	104 (73 to 143)	26 (13 to 47)	26 (23 to 29)
Ghana	2026	97 (78 to 120)	46 (34 to 60)	34 (24 to 46)	8 (4 to 15)	8 (7 to 9)	305 (247 to 379)	146 (108 to 190)	107 (75 to 146)	27 (13 to 48)	26 (22 to 29)
Ghana	2027	100 (80 to 124)	49 (36 to 63)	35 (24 to 47)	9 (4 to 16)	8 (7 to 9)	316 (253 to 391)	153 (113 to 200)	109 (77 to 150)	28 (13 to 50)	25 (22 to 30)
Ghana	2028	103 (82 to 129)	51 (38 to 67)	35 (25 to 48)	9 (4 to 16)	8 (7 to 9)	326 (260 to 406)	161 (118 to 212)	112 (79 to 153)	28 (14 to 51)	26 (22 to 30)
Ghana	2029	107 (85 to 133)	53 (39 to 71)	36 (26 to 50)	9 (5 to 17)	8 (7 to 10)	338 (268 to 418)	168 (123 to 224)	114 (81 to 157)	29 (14 to 52)	26 (22 to 30)
Ghana	2030	111 (87 to 138)	56 (40 to 74)	37 (26 to 51)	10 (5 to 17)	8 (7 to 10)	349 (275 to 434)	176 (127 to 234)	117 (83 to 160)	30 (15 to 54)	26 (22 to 31)
Ghana	2031	114 (90 to 143)	58 (42 to 77)	38 (27 to 52)	10 (5 to 18)	8 (7 to 10)	360 (285 to 450)	184 (134 to 243)	120 (85 to 164)	31 (15 to 56)	26 (22 to 31)
Ghana	2032	118 (93 to 147)	61 (44 to 80)	39 (28 to 53)	10 (5 to 18)	8 (7 to 10)	373 (292 to 465)	192 (140 to 253)	123 (87 to 168)	32 (16 to 57)	26 (22 to 31)
Ghana	2033	123 (96 to 153)	64 (47 to 84)	40 (28 to 55)	11 (5 to 19)	8 (7 to 10)	386 (303 to 484)	201 (147 to 266)	126 (89 to 172)	33 (16 to 59)	26 (22 to 31)

Ghana	2034	127 (99 to 159)	67 (48 to 89)	41 (29 to 56)	11 (5 to 19)	8 (7 to 10)	401 (311 to 503)	211 (152 to 281)	130 (91 to 177)	34 (17 to 61)	26 (22 to 32)
Ghana	2035	132 (103 to 166)	70 (50 to 94)	42 (30 to 58)	11 (5 to 20)	8 (7 to 10)	416 (326 to 523)	222 (158 to 296)	133 (94 to 181)	35 (17 to 63)	26 (22 to 32)
Ghana	2036	137 (106 to 173)	74 (52 to 98)	43 (31 to 59)	12 (6 to 21)	8 (7 to 10)	432 (335 to 546)	233 (165 to 310)	137 (97 to 186)	36 (18 to 65)	26 (22 to 33)
Ghana	2037	142 (110 to 179)	78 (54 to 103)	44 (31 to 61)	12 (6 to 21)	8 (7 to 10)	449 (347 to 566)	245 (172 to 323)	140 (99 to 191)	38 (18 to 67)	26 (22 to 32)
Ghana	2038	148 (115 to 188)	82 (58 to 109)	46 (32 to 62)	12 (6 to 22)	8 (7 to 11)	467 (362 to 593)	257 (182 to 345)	144 (102 to 197)	39 (19 to 70)	26 (22 to 33)
Ghana	2039	154 (119 to 196)	86 (60 to 114)	47 (33 to 64)	13 (6 to 23)	8 (7 to 11)	485 (375 to 618)	270 (190 to 361)	148 (105 to 201)	40 (19 to 72)	26 (22 to 34)
Ghana	2040	160 (123 to 203)	90 (63 to 120)	48 (34 to 65)	13 (6 to 24)	8 (7 to 11)	503 (388 to 640)	284 (200 to 378)	152 (107 to 207)	41 (20 to 74)	27 (22 to 34)
Ghana	2041	166 (128 to 212)	95 (67 to 126)	49 (35 to 67)	14 (6 to 24)	8 (7 to 11)	523 (403 to 668)	298 (210 to 398)	156 (110 to 212)	43 (20 to 77)	27 (22 to 34)
Ghana	2042	173 (132 to 219)	99 (70 to 134)	51 (36 to 69)	14 (7 to 25)	9 (7 to 11)	544 (417 to 692)	313 (222 to 423)	160 (113 to 217)	44 (21 to 79)	27 (22 to 34)
Ghana	2043	179 (138 to 227)	104 (73 to 141)	52 (37 to 71)	14 (7 to 26)	9 (7 to 11)	565 (435 to 716)	329 (232 to 445)	164 (115 to 223)	45 (22 to 82)	27 (22 to 35)
Ghana	2044	186 (142 to 237)	110 (77 to 148)	53 (38 to 72)	15 (7 to 27)	9 (7 to 11)	588 (447 to 748)	346 (243 to 468)	168 (118 to 228)	47 (22 to 84)	27 (22 to 36)
Ghana	2045	194 (148 to 246)	115 (81 to 155)	54 (38 to 74)	15 (7 to 27)	9 (7 to 11)	610 (465 to 776)	363 (255 to 490)	172 (121 to 234)	48 (23 to 87)	27 (22 to 35)
Ghana	2046	201 (153 to 257)	121 (84 to 162)	56 (39 to 76)	16 (8 to 28)	9 (7 to 11)	634 (484 to 811)	382 (266 to 510)	176 (124 to 240)	50 (24 to 89)	28 (22 to 36)
Ghana	2047	209 (158 to 267)	127 (89 to 172)	57 (40 to 78)	16 (8 to 29)	9 (7 to 12)	660 (498 to 843)	401 (281 to 543)	180 (127 to 245)	51 (24 to 92)	28 (22 to 37)
Ghana	2048	217 (166 to 278)	133 (94 to 180)	58 (41 to 80)	17 (8 to 30)	9 (7 to 12)	685 (523 to 876)	421 (295 to 568)	184 (130 to 251)	52 (25 to 94)	28 (22 to 37)

Ghana	2049	226 (172 to 288)	140 (97 to 191)	60 (42 to 82)	17 (8 to 31)	9 (7 to 12)	712 (543 to 910)	441 (307 to 603)	189 (132 to 258)	54 (26 to 97)	28 (22 to 38)
Ghana	2050	234 (177 to 302)	147 (101 to 201)	61 (43 to 84)	18 (8 to 32)	9 (7 to 12)	739 (558 to 953)	462 (318 to 634)	193 (135 to 264)	55 (27 to 99)	28 (22 to 39)
Greece	1995	1288 (1208 to 1368)	661 (604 to 725)	595 (544 to 648)	33 (20 to 49)	0 (0 to 0)	1942 (1821 to 2062)	996 (911 to 1093)	897 (820 to 977)	49 (30 to 74)	0 (0 to 0)
Greece	1996	1297 (1232 to 1367)	671 (623 to 724)	593 (554 to 635)	33 (22 to 48)	0 (0 to 0)	1955 (1856 to 2061)	1012 (939 to 1092)	893 (835 to 957)	50 (33 to 72)	0 (0 to 0)
Greece	1997	1317 (1256 to 1378)	692 (649 to 743)	590 (555 to 627)	34 (23 to 48)	0 (0 to 0)	1985 (1894 to 2078)	1044 (978 to 1120)	890 (837 to 944)	51 (35 to 72)	0 (0 to 0)
Greece	1998	1352 (1292 to 1411)	730 (687 to 777)	586 (551 to 620)	35 (25 to 48)	0 (0 to 0)	2037 (1948 to 2127)	1101 (1035 to 1171)	884 (831 to 935)	53 (37 to 73)	0 (0 to 0)
Greece	1999	1390 (1332 to 1450)	780 (732 to 829)	573 (539 to 607)	37 (27 to 50)	0 (0 to 0)	2095 (2008 to 2186)	1176 (1104 to 1250)	863 (813 to 916)	55 (40 to 75)	0 (0 to 0)
Greece	2000	1443 (1383 to 1503)	849 (800 to 897)	554 (522 to 589)	40 (29 to 52)	0 (0 to 0)	2175 (2085 to 2265)	1280 (1206 to 1352)	835 (786 to 887)	60 (44 to 78)	0 (0 to 0)
Greece	2001	1545 (1487 to 1605)	928 (878 to 978)	574 (541 to 608)	43 (32 to 55)	0 (0 to 0)	2329 (2241 to 2419)	1400 (1324 to 1475)	865 (815 to 917)	65 (49 to 84)	0 (0 to 0)
Greece	2002	1661 (1600 to 1723)	995 (946 to 1044)	618 (587 to 653)	47 (36 to 60)	0 (0 to 0)	2504 (2411 to 2597)	1500 (1426 to 1573)	932 (885 to 984)	71 (55 to 90)	0 (0 to 0)
Greece	2003	1777 (1712 to 1841)	1058 (1009 to 1110)	667 (632 to 702)	52 (40 to 65)	0 (0 to 0)	2678 (2581 to 2775)	1595 (1521 to 1673)	1005 (953 to 1058)	78 (60 to 99)	0 (0 to 0)

Greece	2004	1894 (1831 to 1957)	1130 (1081 to 1182)	707 (672 to 747)	57 (44 to 71)	0 (0 to 0)	2855 (2760 to 2950)	1703 (1630 to 1782)	1066 (1013 to 1126)	85 (66 to 107)	0 (0 to 0)
Greece	2005	1911 (1850 to 1975)	1145 (1096 to 1197)	709 (673 to 746)	57 (45 to 72)	0 (0 to 0)	2881 (2788 to 2978)	1726 (1653 to 1804)	1068 (1015 to 1124)	87 (68 to 108)	0 (0 to 0)
Greece	2006	2009 (1944 to 2078)	1231 (1184 to 1283)	716 (678 to 753)	62 (49 to 77)	0 (0 to 0)	3028 (2930 to 3132)	1856 (1784 to 1934)	1079 (1021 to 1135)	93 (74 to 116)	0 (0 to 0)
Greece	2007	2109 (2037 to 2178)	1332 (1281 to 1382)	710 (673 to 748)	66 (52 to 83)	0 (0 to 0)	3179 (3070 to 3283)	2009 (1931 to 2083)	1071 (1015 to 1127)	100 (78 to 125)	0 (0 to 0)
Greece	2008	2219 (2150 to 2294)	1451 (1398 to 1509)	698 (662 to 735)	71 (56 to 88)	0 (0 to 0)	3345 (3240 to 3458)	2187 (2108 to 2275)	1051 (998 to 1108)	107 (84 to 132)	0 (0 to 0)
Greece	2009	2244 (2174 to 2316)	1504 (1449 to 1563)	666 (632 to 701)	74 (58 to 91)	0 (0 to 0)	3382 (3277 to 3492)	2267 (2184 to 2357)	1004 (953 to 1057)	111 (87 to 137)	0 (0 to 0)
Greece	2010	2075 (2005 to 2142)	1391 (1334 to 1449)	612 (580 to 647)	73 (58 to 89)	0 (0 to 0)	3128 (3023 to 3230)	2097 (2012 to 2185)	922 (875 to 975)	109 (87 to 134)	0 (0 to 0)
Greece	2011	1877 (1815 to 1940)	1238 (1188 to 1295)	569 (537 to 603)	70 (56 to 86)	0 (0 to 0)	2829 (2735 to 2924)	1866 (1791 to 1952)	857 (810 to 909)	106 (85 to 129)	0 (0 to 0)
Greece	2012	1706 (1648 to 1768)	1101 (1053 to 1150)	538 (507 to 571)	68 (54 to 83)	0 (0 to 0)	2572 (2484 to 2666)	1660 (1587 to 1733)	810 (764 to 861)	102 (81 to 126)	0 (0 to 0)
Greece	2013	1645 (1587 to 1708)	1033 (985 to 1084)	543 (512 to 575)	69 (55 to 86)	0 (0 to 0)	2480 (2392 to 2574)	1557 (1485 to 1635)	819 (771 to 867)	104 (83 to 129)	0 (0 to 0)

Greece	2014	1513 (1458 to 1572)	910 (867 to 960)	536 (507 to 568)	67 (53 to 82)	0 (0 to 0)	2281 (2198 to 2370)	1372 (1308 to 1447)	808 (764 to 856)	101 (80 to 124)	0 (0 to 0)
Greece	2015	1554 (1494 to 1620)	927 (879 to 978)	556 (522 to 588)	72 (57 to 88)	0 (0 to 0)	2342 (2253 to 2441)	1397 (1325 to 1474)	838 (787 to 887)	108 (86 to 133)	0 (0 to 0)
Greece	2016	1550 (1481 to 1619)	922 (871 to 978)	555 (518 to 590)	73 (56 to 92)	0 (0 to 0)	2336 (2233 to 2440)	1390 (1313 to 1474)	836 (781 to 890)	111 (85 to 138)	0 (0 to 0)
Greece	2017	1571 (1477 to 1676)	937 (870 to 1008)	557 (511 to 606)	76 (56 to 98)	0 (0 to 0)	2368 (2227 to 2526)	1413 (1311 to 1519)	840 (770 to 913)	115 (85 to 148)	0 (0 to 0)
Greece	2018	1597 (1491 to 1711)	952 (883 to 1024)	567 (520 to 617)	77 (57 to 100)	0 (0 to 0)	2407 (2247 to 2579)	1436 (1331 to 1544)	855 (784 to 930)	117 (86 to 151)	0 (0 to 0)
Greece	2019	1663 (1559 to 1774)	1005 (931 to 1084)	578 (530 to 629)	81 (60 to 105)	0 (0 to 0)	2507 (2350 to 2674)	1514 (1403 to 1633)	871 (798 to 948)	122 (90 to 158)	0 (0 to 0)
Greece	2020	1704 (1583 to 1826)	1031 (953 to 1114)	590 (540 to 643)	83 (61 to 107)	0 (0 to 0)	2569 (2386 to 2753)	1554 (1437 to 1679)	890 (814 to 970)	125 (92 to 161)	0 (0 to 0)
Greece	2021	1719 (1606 to 1836)	1041 (957 to 1126)	593 (543 to 647)	84 (62 to 108)	0 (0 to 0)	2591 (2421 to 2767)	1570 (1443 to 1697)	894 (819 to 975)	126 (93 to 163)	0 (0 to 0)
Greece	2022	1735 (1604 to 1868)	1054 (962 to 1147)	596 (545 to 650)	85 (63 to 109)	0 (0 to 0)	2616 (2418 to 2815)	1589 (1450 to 1729)	899 (822 to 979)	128 (95 to 164)	0 (0 to 0)
Greece	2023	1754 (1624 to 1892)	1068 (973 to 1169)	600 (549 to 654)	86 (64 to 111)	0 (0 to 0)	2644 (2448 to 2853)	1610 (1466 to 1762)	904 (828 to 985)	130 (96 to 167)	0 (0 to 0)

Greece	2024	1765 (1622 to 1916)	1077 (974 to 1194)	601 (551 to 655)	87 (64 to 112)	0 (0 to 0)	2660 (2445 to 2888)	1624 (1468 to 1799)	906 (830 to 987)	131 (97 to 168)	0 (0 to 0)
Greece	2025	1775 (1640 to 1922)	1086 (979 to 1203)	602 (552 to 656)	88 (65 to 113)	0 (0 to 0)	2676 (2472 to 2897)	1637 (1475 to 1814)	907 (831 to 989)	132 (98 to 170)	0 (0 to 0)
Greece	2026	1787 (1633 to 1947)	1095 (982 to 1218)	603 (553 to 657)	88 (65 to 114)	0 (0 to 0)	2694 (2461 to 2935)	1651 (1480 to 1837)	909 (833 to 991)	133 (98 to 172)	0 (0 to 0)
Greece	2027	1799 (1650 to 1960)	1105 (989 to 1232)	605 (554 to 659)	89 (66 to 116)	0 (0 to 0)	2712 (2488 to 2955)	1665 (1491 to 1857)	912 (835 to 993)	135 (99 to 174)	0 (0 to 0)
Greece	2028	1811 (1644 to 1977)	1114 (990 to 1243)	606 (556 to 660)	90 (66 to 117)	0 (0 to 0)	2729 (2478 to 2980)	1680 (1493 to 1873)	914 (837 to 996)	136 (100 to 177)	0 (0 to 0)
Greece	2029	1825 (1672 to 1997)	1126 (1000 to 1266)	608 (556 to 662)	91 (67 to 119)	0 (0 to 0)	2750 (2521 to 3010)	1697 (1508 to 1909)	916 (838 to 998)	137 (101 to 179)	0 (0 to 0)
Greece	2030	1836 (1664 to 2020)	1135 (1013 to 1287)	609 (557 to 663)	92 (67 to 120)	0 (0 to 0)	2768 (2509 to 3046)	1712 (1527 to 1939)	918 (839 to 1000)	139 (102 to 181)	0 (0 to 0)
Greece	2031	1848 (1688 to 2035)	1145 (1018 to 1303)	610 (558 to 665)	93 (68 to 121)	0 (0 to 0)	2786 (2544 to 3068)	1726 (1534 to 1964)	919 (841 to 1002)	140 (102 to 183)	0 (0 to 0)
Greece	2032	1858 (1680 to 2049)	1154 (1022 to 1317)	610 (558 to 666)	94 (69 to 122)	0 (0 to 0)	2801 (2532 to 3089)	1740 (1540 to 1985)	920 (841 to 1004)	141 (103 to 185)	0 (0 to 0)
Greece	2033	1867 (1699 to 2063)	1162 (1024 to 1324)	610 (557 to 667)	94 (69 to 123)	0 (0 to 0)	2814 (2560 to 3110)	1752 (1543 to 1995)	920 (839 to 1005)	142 (104 to 186)	0 (0 to 0)

Greece	2034	1871 (1685 to 2070)	1167 (1023 to 1330)	609 (555 to 666)	95 (69 to 124)	0 (0 to 0)	2821 (2541 to 3120)	1759 (1543 to 2005)	919 (837 to 1004)	143 (105 to 186)	0 (0 to 0)
Greece	2035	1875 (1696 to 2077)	1172 (1025 to 1340)	608 (553 to 665)	95 (70 to 124)	0 (0 to 0)	2827 (2556 to 3130)	1767 (1546 to 2021)	916 (833 to 1002)	144 (105 to 187)	0 (0 to 0)
Greece	2036	1880 (1688 to 2096)	1177 (1027 to 1348)	607 (551 to 665)	96 (70 to 125)	0 (0 to 0)	2834 (2544 to 3160)	1774 (1548 to 2032)	915 (830 to 1002)	145 (106 to 188)	0 (0 to 0)
Greece	2037	1884 (1696 to 2092)	1182 (1030 to 1352)	606 (549 to 664)	96 (70 to 125)	0 (0 to 0)	2841 (2557 to 3154)	1782 (1552 to 2037)	913 (828 to 1001)	145 (106 to 189)	0 (0 to 0)
Greece	2038	1887 (1692 to 2104)	1186 (1028 to 1355)	605 (547 to 664)	97 (71 to 126)	0 (0 to 0)	2845 (2550 to 3171)	1788 (1550 to 2043)	911 (825 to 1000)	146 (106 to 190)	0 (0 to 0)
Greece	2039	1891 (1700 to 2104)	1190 (1032 to 1369)	603 (546 to 662)	97 (71 to 127)	0 (0 to 0)	2850 (2562 to 3172)	1795 (1556 to 2063)	909 (823 to 999)	147 (107 to 191)	0 (0 to 0)
Greece	2040	1893 (1686 to 2115)	1194 (1027 to 1376)	601 (543 to 661)	98 (71 to 128)	0 (0 to 0)	2854 (2542 to 3188)	1801 (1548 to 2074)	906 (819 to 997)	147 (107 to 192)	0 (0 to 0)
Greece	2041	1895 (1697 to 2125)	1198 (1030 to 1385)	599 (541 to 660)	98 (71 to 128)	0 (0 to 0)	2857 (2558 to 3204)	1806 (1553 to 2088)	903 (816 to 995)	148 (107 to 194)	0 (0 to 0)
Greece	2042	1898 (1683 to 2124)	1201 (1026 to 1381)	598 (538 to 658)	99 (72 to 129)	0 (0 to 0)	2860 (2536 to 3201)	1811 (1547 to 2082)	901 (812 to 993)	149 (108 to 195)	0 (0 to 0)
Greece	2043	1898 (1694 to 2129)	1204 (1031 to 1396)	595 (535 to 656)	99 (72 to 130)	0 (0 to 0)	2862 (2554 to 3210)	1815 (1554 to 2105)	897 (807 to 989)	149 (108 to 196)	0 (0 to 0)

Greece	2044	1899 (1680 to 2136)	1207 (1027 to 1402)	593 (532 to 654)	99 (72 to 131)	0 (0 to 0)	2862 (2533 to 3220)	1819 (1548 to 2114)	894 (802 to 986)	150 (108 to 197)	0 (0 to 0)
Greece	2045	1899 (1687 to 2149)	1209 (1026 to 1419)	591 (530 to 652)	100 (72 to 132)	0 (0 to 0)	2862 (2543 to 3239)	1822 (1546 to 2138)	890 (799 to 983)	150 (109 to 198)	0 (0 to 0)
Greece	2046	1900 (1673 to 2148)	1211 (1025 to 1425)	589 (527 to 651)	100 (72 to 133)	0 (0 to 0)	2864 (2522 to 3239)	1826 (1545 to 2148)	887 (794 to 981)	151 (109 to 200)	0 (0 to 0)
Greece	2047	1905 (1679 to 2166)	1217 (1025 to 1430)	587 (525 to 651)	101 (73 to 134)	0 (0 to 0)	2871 (2532 to 3265)	1834 (1546 to 2156)	886 (791 to 982)	152 (110 to 202)	0 (0 to 0)
Greece	2048	1912 (1679 to 2167)	1224 (1033 to 1438)	587 (524 to 652)	101 (73 to 135)	0 (0 to 0)	2883 (2531 to 3267)	1845 (1558 to 2168)	885 (789 to 983)	153 (110 to 204)	0 (0 to 0)
Greece	2049	1922 (1694 to 2193)	1232 (1041 to 1448)	587 (523 to 653)	102 (74 to 137)	0 (0 to 0)	2897 (2554 to 3306)	1858 (1569 to 2183)	885 (789 to 984)	154 (111 to 206)	0 (0 to 0)
Greece	2050	1933 (1683 to 2207)	1242 (1036 to 1474)	588 (522 to 653)	103 (74 to 138)	0 (0 to 0)	2914 (2537 to 3327)	1873 (1562 to 2222)	886 (787 to 985)	156 (112 to 207)	0 (0 to 0)
Greenland	1995	3609 (2786 to 4512)	3608 (2785 to 4512)	0 (0 to 0)	0 (0 to 0)	0 (0 to 0)	2685 (2073 to 3358)	2685 (2072 to 3357)	0 (0 to 0)	0 (0 to 0)	0 (0 to 0)
Greenland	1996	3684 (2902 to 4610)	3683 (2902 to 4610)	0 (0 to 0)	0 (0 to 0)	0 (0 to 0)	2741 (2160 to 3430)	2741 (2159 to 3430)	0 (0 to 0)	0 (0 to 0)	0 (0 to 0)
Greenland	1997	3813 (3063 to 4699)	3812 (3063 to 4698)	0 (0 to 0)	0 (0 to 0)	0 (0 to 0)	2837 (2279 to 3496)	2837 (2279 to 3496)	0 (0 to 0)	0 (0 to 0)	0 (0 to 0)

Greenland	1998	4147 (3401 to 5025)	4146 (3401 to 5024)	0 (0 to 0)	0 (0 to 0)	0 (0 to 0)	3086 (2531 to 3739)	3085 (2530 to 3739)	0 (0 to 0)	0 (0 to 0)	0 (0 to 0)
Greenland	1999	4283 (3640 to 5082)	4282 (3639 to 5081)	0 (0 to 0)	0 (0 to 0)	0 (0 to 0)	3187 (2708 to 3781)	3186 (2708 to 3781)	0 (0 to 0)	0 (0 to 0)	0 (0 to 0)
Greenland	2000	4567 (3984 to 5221)	4566 (3983 to 5221)	0 (0 to 0)	0 (0 to 0)	0 (0 to 0)	3398 (2964 to 3885)	3398 (2964 to 3885)	0 (0 to 0)	0 (0 to 0)	0 (0 to 0)
Greenland	2001	4787 (4319 to 5304)	4787 (4318 to 5303)	0 (0 to 0)	0 (0 to 0)	0 (0 to 0)	3562 (3213 to 3946)	3562 (3213 to 3946)	0 (0 to 0)	0 (0 to 0)	0 (0 to 0)
Greenland	2002	4850 (4515 to 5198)	4850 (4514 to 5197)	0 (0 to 0)	0 (0 to 0)	0 (0 to 0)	3609 (3359 to 3867)	3609 (3359 to 3867)	0 (0 to 0)	0 (0 to 0)	0 (0 to 0)
Greenland	2003	4994 (4782 to 5211)	4994 (4782 to 5211)	0 (0 to 0)	0 (0 to 0)	0 (0 to 0)	3716 (3558 to 3877)	3716 (3558 to 3877)	0 (0 to 0)	0 (0 to 0)	0 (0 to 0)
Greenland	2004	5179 (5025 to 5342)	5179 (5025 to 5341)	0 (0 to 0)	0 (0 to 0)	0 (0 to 0)	3854 (3739 to 3975)	3853 (3739 to 3974)	0 (0 to 0)	0 (0 to 0)	0 (0 to 0)
Greenland	2005	5349 (5193 to 5493)	5348 (5192 to 5493)	0 (0 to 0)	0 (0 to 0)	0 (0 to 0)	3980 (3864 to 4087)	3980 (3863 to 4087)	0 (0 to 0)	0 (0 to 0)	0 (0 to 0)
Greenland	2006	5637 (5484 to 5782)	5636 (5483 to 5782)	0 (0 to 0)	0 (0 to 0)	0 (0 to 0)	4194 (4081 to 4303)	4194 (4080 to 4303)	0 (0 to 0)	0 (0 to 0)	0 (0 to 0)
Greenland	2007	5937 (5783 to 6087)	5936 (5783 to 6086)	0 (0 to 0)	0 (0 to 0)	0 (0 to 0)	4418 (4303 to 4530)	4417 (4303 to 4529)	0 (0 to 0)	0 (0 to 0)	0 (0 to 0)

Greenland	2008	6552 (6393 to 6706)	6552 (6392 to 6705)	0 (0 to 0)	0 (0 to 0)	0 (0 to 0)	4875 (4757 to 4990)	4875 (4757 to 4989)	0 (0 to 0)	0 (0 to 0)	0 (0 to 0)
Greenland	2009	6764 (6602 to 6928)	6764 (6602 to 6928)	0 (0 to 1)	0 (0 to 1)	0 (0 to 0)	5033 (4913 to 5155)	5033 (4912 to 5155)	0 (0 to 0)	0 (0 to 0)	0 (0 to 0)
Greenland	2010	6807 (6640 to 6975)	6806 (6639 to 6975)	0 (0 to 1)	0 (0 to 1)	0 (0 to 0)	5065 (4941 to 5190)	5065 (4940 to 5190)	0 (0 to 0)	0 (0 to 0)	0 (0 to 0)
Greenland	2011	6797 (6630 to 6962)	6797 (6630 to 6962)	0 (0 to 1)	0 (0 to 1)	0 (0 to 0)	5058 (4933 to 5180)	5057 (4933 to 5180)	0 (0 to 0)	0 (0 to 0)	0 (0 to 0)
Greenland	2012	6871 (6716 to 7035)	6870 (6715 to 7035)	0 (0 to 1)	0 (0 to 1)	0 (0 to 0)	5112 (4998 to 5235)	5112 (4997 to 5234)	0 (0 to 0)	0 (0 to 0)	0 (0 to 0)
Greenland	2013	6796 (6635 to 6956)	6796 (6635 to 6955)	0 (0 to 1)	0 (0 to 1)	0 (0 to 0)	5057 (4937 to 5176)	5057 (4937 to 5175)	0 (0 to 0)	0 (0 to 0)	0 (0 to 0)
Greenland	2014	6754 (6594 to 6923)	6754 (6593 to 6922)	0 (0 to 1)	0 (0 to 1)	0 (0 to 0)	5026 (4906 to 5151)	5025 (4906 to 5151)	0 (0 to 0)	0 (0 to 0)	0 (0 to 0)
Greenland	2015	6312 (6160 to 6471)	6311 (6159 to 6471)	0 (0 to 1)	0 (0 to 1)	0 (0 to 0)	4697 (4584 to 4815)	4696 (4583 to 4815)	0 (0 to 0)	0 (0 to 0)	0 (0 to 0)
Greenland	2016	6412 (6202 to 6636)	6411 (6201 to 6635)	0 (0 to 1)	0 (0 to 1)	0 (0 to 0)	4771 (4615 to 4938)	4770 (4614 to 4937)	0 (0 to 0)	0 (0 to 0)	0 (0 to 0)
Greenland	2017	6559 (6196 to 6981)	6558 (6196 to 6980)	0 (0 to 1)	0 (0 to 1)	0 (0 to 0)	4880 (4610 to 5195)	4880 (4610 to 5194)	0 (0 to 0)	0 (0 to 0)	0 (0 to 0)

Greenland	2018	6722 (6341 to 7135)	6722 (6340 to 7134)	0 (0 to 1)	0 (0 to 1)	0 (0 to 0)	5002 (4718 to 5309)	5002 (4718 to 5308)	0 (0 to 0)	0 (0 to 0)	0 (0 to 0)
Greenland	2019	6998 (6592 to 7459)	6997 (6591 to 7458)	0 (0 to 1)	0 (0 to 1)	0 (0 to 0)	5207 (4905 to 5550)	5206 (4905 to 5549)	0 (0 to 1)	0 (0 to 0)	0 (0 to 0)
Greenland	2020	7295 (6847 to 7779)	7295 (6847 to 7777)	0 (0 to 1)	0 (0 to 1)	0 (0 to 0)	5428 (5095 to 5788)	5428 (5095 to 5787)	0 (0 to 1)	0 (0 to 0)	0 (0 to 0)
Greenland	2021	7458 (6992 to 7965)	7458 (6991 to 7964)	0 (0 to 1)	0 (0 to 1)	0 (0 to 0)	5550 (5203 to 5927)	5549 (5202 to 5926)	0 (0 to 1)	0 (0 to 0)	0 (0 to 0)
Greenland	2022	7615 (7153 to 8141)	7614 (7153 to 8140)	0 (0 to 1)	0 (0 to 1)	0 (0 to 0)	5666 (5323 to 6058)	5666 (5322 to 6057)	0 (0 to 1)	0 (0 to 0)	0 (0 to 0)
Greenland	2023	7692 (7215 to 8240)	7692 (7215 to 8239)	0 (0 to 1)	0 (0 to 1)	0 (0 to 0)	5724 (5369 to 6131)	5723 (5368 to 6130)	0 (0 to 1)	0 (0 to 1)	0 (0 to 0)
Greenland	2024	7773 (7266 to 8318)	7772 (7266 to 8316)	0 (0 to 1)	0 (0 to 1)	0 (0 to 0)	5784 (5407 to 6189)	5783 (5407 to 6188)	0 (0 to 1)	0 (0 to 1)	0 (0 to 0)
Greenland	2025	7846 (7338 to 8400)	7845 (7337 to 8399)	0 (0 to 1)	0 (0 to 1)	0 (0 to 0)	5838 (5460 to 6250)	5838 (5460 to 6250)	0 (0 to 1)	0 (0 to 1)	0 (0 to 0)
Greenland	2026	7919 (7384 to 8485)	7919 (7383 to 8484)	0 (0 to 1)	0 (0 to 1)	0 (0 to 0)	5893 (5494 to 6314)	5892 (5494 to 6313)	0 (0 to 1)	0 (0 to 1)	0 (0 to 0)
Greenland	2027	7989 (7445 to 8550)	7988 (7445 to 8549)	0 (0 to 1)	0 (0 to 1)	0 (0 to 0)	5944 (5540 to 6362)	5944 (5540 to 6361)	0 (0 to 1)	0 (0 to 1)	0 (0 to 0)

Greenland	2028	8051 (7531 to 8614)	8050 (7530 to 8613)	0 (0 to 1)	0 (0 to 1)	0 (0 to 0)	5991 (5604 to 6410)	5990 (5603 to 6409)	0 (0 to 1)	0 (0 to 1)	0 (0 to 0)
Greenland	2029	8095 (7553 to 8670)	8094 (7552 to 8669)	0 (0 to 1)	0 (0 to 1)	0 (0 to 0)	6023 (5620 to 6451)	6023 (5620 to 6451)	0 (0 to 1)	0 (0 to 1)	0 (0 to 0)
Greenland	2030	8140 (7578 to 8745)	8139 (7578 to 8743)	0 (0 to 1)	0 (0 to 1)	0 (0 to 0)	6057 (5638 to 6507)	6056 (5638 to 6506)	0 (0 to 1)	0 (0 to 1)	0 (0 to 0)
Greenland	2031	8202 (7622 to 8815)	8201 (7621 to 8815)	0 (0 to 1)	0 (0 to 1)	0 (0 to 0)	6103 (5671 to 6560)	6102 (5671 to 6559)	0 (0 to 1)	0 (0 to 1)	0 (0 to 0)
Greenland	2032	8280 (7684 to 8908)	8279 (7684 to 8906)	0 (0 to 1)	0 (0 to 1)	0 (0 to 0)	6161 (5718 to 6628)	6161 (5718 to 6627)	0 (0 to 1)	0 (0 to 1)	0 (0 to 0)
Greenland	2033	8381 (7780 to 9025)	8381 (7779 to 9023)	0 (0 to 1)	0 (0 to 1)	0 (0 to 0)	6236 (5789 to 6716)	6236 (5788 to 6714)	0 (0 to 1)	0 (0 to 1)	0 (0 to 0)
Greenland	2034	8490 (7862 to 9149)	8489 (7862 to 9148)	0 (0 to 1)	0 (0 to 1)	0 (0 to 0)	6317 (5850 to 6808)	6317 (5850 to 6807)	0 (0 to 1)	0 (0 to 1)	0 (0 to 0)
Greenland	2035	8614 (7948 to 9301)	8613 (7948 to 9300)	0 (0 to 1)	0 (0 to 1)	0 (0 to 0)	6410 (5914 to 6921)	6409 (5914 to 6920)	0 (0 to 1)	0 (0 to 1)	0 (0 to 0)
Greenland	2036	8754 (8071 to 9466)	8753 (8070 to 9464)	0 (0 to 1)	0 (0 to 1)	0 (0 to 0)	6514 (6006 to 7043)	6513 (6005 to 7042)	0 (0 to 1)	0 (0 to 1)	0 (0 to 0)
Greenland	2037	8926 (8230 to 9665)	8925 (8230 to 9665)	0 (0 to 1)	0 (0 to 1)	0 (0 to 0)	6642 (6124 to 7192)	6641 (6124 to 7191)	0 (0 to 1)	0 (0 to 1)	0 (0 to 0)

Greenland	2038	9125 (8427 to 9914)	9124 (8427 to 9913)	0 (0 to 1)	0 (0 to 1)	0 (0 to 0)	6790 (6270 to 7377)	6789 (6270 to 7376)	0 (0 to 1)	0 (0 to 1)	0 (0 to 0)
Greenland	2039	9332 (8605 to 10118)	9331 (8605 to 10118)	0 (0 to 1)	0 (0 to 1)	0 (0 to 0)	6944 (6403 to 7529)	6943 (6403 to 7528)	0 (0 to 1)	0 (0 to 1)	0 (0 to 0)
Greenland	2040	9553 (8831 to 10353)	9552 (8830 to 10352)	0 (0 to 1)	0 (0 to 1)	0 (0 to 0)	7108 (6571 to 7704)	7108 (6570 to 7703)	0 (0 to 1)	0 (0 to 1)	0 (0 to 0)
Greenland	2041	9781 (9004 to 10616)	9780 (9004 to 10615)	0 (0 to 1)	0 (0 to 1)	0 (0 to 0)	7278 (6700 to 7899)	7277 (6700 to 7899)	0 (0 to 1)	0 (0 to 1)	0 (0 to 0)
Greenland	2042	9995 (9207 to 10849)	9994 (9207 to 10847)	0 (0 to 1)	0 (0 to 1)	0 (0 to 0)	7437 (6851 to 8072)	7436 (6851 to 8071)	0 (0 to 1)	0 (0 to 1)	0 (0 to 0)
Greenland	2043	10214 (9413 to 11117)	10213 (9413 to 11115)	0 (0 to 1)	0 (0 to 1)	0 (0 to 0)	7600 (7004 to 8272)	7600 (7004 to 8270)	0 (0 to 1)	0 (0 to 1)	0 (0 to 0)
Greenland	2044	10425 (9563 to 11355)	10424 (9563 to 11354)	0 (0 to 1)	0 (0 to 1)	0 (0 to 0)	7757 (7115 to 8450)	7756 (7115 to 8449)	0 (0 to 1)	0 (0 to 1)	0 (0 to 0)
Greenland	2045	10637 (9729 to 11572)	10636 (9728 to 11571)	0 (0 to 1)	0 (0 to 1)	0 (0 to 0)	7915 (7239 to 8610)	7914 (7238 to 8610)	0 (0 to 1)	0 (0 to 1)	0 (0 to 0)
Greenland	2046	10846 (9913 to 11807)	10845 (9913 to 11806)	0 (0 to 1)	0 (0 to 1)	0 (0 to 0)	8071 (7376 to 8785)	8070 (7376 to 8784)	0 (0 to 1)	0 (0 to 1)	0 (0 to 0)
Greenland	2047	11048 (10086 to 12029)	11047 (10086 to 12026)	0 (0 to 1)	1 (0 to 1)	0 (0 to 0)	8220 (7505 to 8950)	8220 (7505 to 8949)	0 (0 to 1)	0 (0 to 1)	0 (0 to 0)

Greenland	2048	11254 (10300 to 12284)	11253 (10300 to 12282)	0 (0 to 1)	1 (0 to 1)	0 (0 to 0)	8374 (7664 to 9141)	8373 (7664 to 9139)	0 (0 to 1)	0 (0 to 1)	0 (0 to 0)
Greenland	2049	11448 (10478 to 12479)	11447 (10477 to 12477)	0 (0 to 1)	1 (0 to 1)	0 (0 to 0)	8518 (7797 to 9286)	8518 (7796 to 9284)	0 (0 to 1)	0 (0 to 1)	0 (0 to 0)
Greenland	2050	11644 (10646 to 12688)	11643 (10645 to 12687)	0 (0 to 1)	1 (0 to 1)	0 (0 to 0)	8664 (7922 to 9441)	8664 (7921 to 9440)	0 (0 to 1)	0 (0 to 1)	0 (0 to 0)
Grenada	1995	478 (436 to 528)	242 (212 to 277)	221 (190 to 255)	15 (7 to 27)	0 (0 to 0)	699 (636 to 771)	353 (310 to 404)	323 (278 to 373)	22 (11 to 40)	0 (0 to 0)
Grenada	1996	494 (455 to 539)	245 (217 to 277)	233 (203 to 264)	16 (8 to 28)	0 (0 to 0)	721 (664 to 787)	358 (317 to 404)	340 (297 to 385)	23 (11 to 41)	1 (1 to 1)
Grenada	1997	491 (452 to 532)	230 (205 to 256)	241 (213 to 270)	15 (8 to 28)	5 (5 to 5)	717 (660 to 777)	335 (299 to 375)	352 (311 to 394)	23 (11 to 40)	7 (7 to 7)
Grenada	1998	484 (447 to 523)	214 (191 to 238)	251 (224 to 279)	16 (8 to 28)	4 (4 to 4)	707 (652 to 763)	312 (279 to 347)	366 (328 to 407)	23 (11 to 41)	6 (6 to 6)
Grenada	1999	455 (420 to 490)	191 (170 to 211)	249 (221 to 275)	15 (8 to 28)	0 (0 to 0)	665 (613 to 715)	279 (249 to 309)	363 (322 to 402)	22 (11 to 40)	0 (0 to 0)
Grenada	2000	420 (388 to 454)	169 (151 to 188)	236 (210 to 262)	15 (8 to 28)	0 (0 to 0)	614 (566 to 662)	247 (220 to 275)	344 (306 to 382)	22 (11 to 41)	0 (0 to 0)
Grenada	2001	415 (384 to 447)	171 (153 to 190)	229 (204 to 254)	16 (8 to 28)	0 (0 to 0)	606 (561 to 653)	249 (223 to 278)	334 (297 to 371)	23 (12 to 41)	1 (1 to 1)
Grenada	2002	449 (415 to 484)	196 (176 to 218)	235 (210 to 260)	17 (9 to 31)	0 (0 to 0)	655 (607 to 707)	287 (256 to 318)	343 (306 to 380)	25 (13 to 45)	1 (1 to 1)
Grenada	2003	432 (399 to 465)	190 (172 to 211)	222 (198 to 246)	16 (9 to 28)	4 (4 to 4)	631 (583 to 678)	278 (251 to 309)	324 (289 to 360)	24 (13 to 41)	6 (6 to 6)
Grenada	2004	414 (382 to 446)	185 (167 to 207)	208 (186 to 231)	15 (8 to 26)	5 (5 to 5)	605 (558 to 652)	270 (244 to 303)	304 (272 to 337)	22 (12 to 38)	7 (7 to 7)

Grenada	2005	468 (434 to 504)	220 (198 to 245)	226 (203 to 250)	18 (10 to 31)	4 (4 to 4)	684 (634 to 736)	321 (289 to 358)	330 (296 to 365)	26 (15 to 45)	6 (6 to 6)
Grenada	2006	518 (481 to 557)	246 (223 to 273)	244 (221 to 269)	20 (11 to 34)	8 (8 to 8)	757 (703 to 813)	359 (325 to 399)	356 (323 to 393)	29 (16 to 49)	12 (12 to 12)
Grenada	2007	512 (478 to 550)	233 (209 to 259)	250 (226 to 276)	19 (11 to 32)	9 (9 to 9)	748 (698 to 803)	340 (305 to 378)	366 (330 to 403)	28 (16 to 47)	13 (13 to 13)
Grenada	2008	526 (493 to 564)	233 (209 to 258)	262 (237 to 289)	21 (11 to 34)	10 (10 to 10)	768 (720 to 823)	341 (305 to 377)	383 (347 to 422)	30 (16 to 49)	14 (14 to 14)
Grenada	2009	514 (481 to 550)	223 (201 to 246)	261 (237 to 289)	20 (11 to 32)	9 (9 to 9)	750 (703 to 803)	325 (293 to 360)	382 (346 to 422)	30 (17 to 47)	13 (13 to 13)
Grenada	2010	513 (478 to 551)	221 (199 to 246)	266 (241 to 294)	21 (11 to 33)	5 (5 to 5)	750 (699 to 805)	323 (291 to 359)	389 (352 to 429)	30 (17 to 48)	8 (8 to 8)
Grenada	2011	523 (484 to 562)	223 (201 to 249)	272 (245 to 299)	21 (12 to 34)	7 (7 to 7)	763 (707 to 820)	326 (293 to 363)	397 (357 to 437)	30 (17 to 49)	10 (10 to 10)
Grenada	2012	498 (462 to 535)	207 (185 to 229)	263 (236 to 291)	19 (11 to 32)	9 (9 to 9)	727 (674 to 782)	302 (270 to 335)	384 (345 to 426)	28 (16 to 46)	13 (13 to 13)
Grenada	2013	499 (464 to 537)	210 (188 to 233)	261 (237 to 291)	20 (11 to 33)	8 (8 to 8)	729 (678 to 785)	307 (275 to 341)	381 (346 to 425)	29 (16 to 48)	12 (12 to 12)
Grenada	2014	499 (460 to 538)	220 (198 to 245)	256 (231 to 282)	20 (11 to 34)	3 (3 to 3)	728 (672 to 786)	321 (289 to 357)	374 (337 to 412)	29 (16 to 50)	4 (4 to 4)
Grenada	2015	491 (452 to 529)	212 (189 to 238)	252 (227 to 278)	20 (11 to 34)	7 (7 to 7)	717 (661 to 773)	309 (276 to 347)	369 (331 to 407)	29 (16 to 50)	10 (10 to 10)
Grenada	2016	494 (453 to 538)	217 (192 to 248)	255 (225 to 286)	20 (11 to 35)	2 (2 to 2)	722 (661 to 786)	317 (281 to 362)	372 (328 to 418)	29 (16 to 51)	4 (4 to 4)
Grenada	2017	528 (474 to 593)	223 (193 to 258)	265 (230 to 306)	21 (11 to 37)	19 (19 to 19)	772 (692 to 867)	325 (282 to 376)	387 (335 to 446)	31 (16 to 55)	28 (28 to 28)
Grenada	2018	544 (485 to 611)	228 (195 to 267)	275 (238 to 317)	22 (11 to 39)	19 (19 to 19)	794 (708 to 892)	333 (285 to 390)	402 (348 to 463)	32 (17 to 56)	27 (27 to 28)
Grenada	2019	558 (496 to 629)	234 (195 to 276)	282 (244 to 325)	23 (12 to 40)	19 (19 to 19)	815 (724 to 919)	342 (285 to 404)	412 (356 to 475)	33 (17 to 58)	28 (28 to 28)

Grenada	2020	571 (504 to 648)	241 (198 to 288)	288 (249 to 331)	23 (12 to 41)	20 (19 to 21)	834 (737 to 946)	351 (289 to 421)	420 (363 to 484)	34 (18 to 60)	29 (27 to 30)
Grenada	2021	582 (513 to 664)	246 (201 to 301)	292 (252 to 336)	24 (12 to 42)	20 (19 to 22)	849 (749 to 970)	359 (294 to 440)	426 (368 to 491)	35 (18 to 61)	30 (28 to 32)
Grenada	2022	591 (518 to 677)	251 (202 to 310)	295 (256 to 341)	24 (13 to 43)	21 (19 to 23)	864 (756 to 989)	367 (295 to 452)	431 (373 to 498)	35 (19 to 62)	30 (28 to 34)
Grenada	2023	597 (524 to 685)	252 (203 to 310)	299 (259 to 345)	25 (13 to 44)	21 (19 to 24)	872 (766 to 1000)	368 (296 to 452)	437 (379 to 503)	36 (19 to 64)	31 (28 to 35)
Grenada	2024	603 (527 to 688)	254 (204 to 312)	303 (262 to 349)	25 (13 to 44)	22 (19 to 24)	880 (770 to 1005)	371 (298 to 455)	442 (383 to 509)	36 (19 to 64)	32 (28 to 36)
Grenada	2025	608 (533 to 697)	255 (205 to 313)	305 (264 to 352)	25 (13 to 45)	22 (20 to 25)	888 (778 to 1017)	373 (300 to 456)	446 (386 to 514)	37 (19 to 65)	32 (29 to 37)
Grenada	2026	614 (535 to 705)	259 (206 to 321)	307 (266 to 353)	26 (13 to 45)	23 (20 to 26)	897 (781 to 1030)	378 (301 to 469)	448 (389 to 516)	37 (20 to 66)	33 (29 to 37)
Grenada	2027	621 (543 to 717)	263 (211 to 328)	309 (268 to 356)	26 (14 to 46)	23 (20 to 27)	907 (793 to 1047)	384 (307 to 479)	452 (392 to 520)	38 (20 to 67)	34 (29 to 39)
Grenada	2028	628 (543 to 726)	267 (210 to 337)	311 (270 to 359)	26 (14 to 46)	23 (21 to 27)	917 (794 to 1060)	390 (307 to 492)	455 (394 to 524)	38 (20 to 68)	34 (30 to 40)
Grenada	2029	635 (554 to 739)	271 (213 to 347)	313 (272 to 361)	27 (14 to 47)	24 (21 to 28)	927 (809 to 1080)	396 (311 to 507)	457 (397 to 527)	39 (20 to 69)	35 (30 to 41)
Grenada	2030	642 (553 to 748)	275 (214 to 353)	315 (273 to 363)	27 (14 to 48)	24 (21 to 29)	937 (808 to 1093)	402 (313 to 516)	460 (399 to 530)	40 (21 to 70)	36 (31 to 43)
Grenada	2031	648 (561 to 761)	279 (211 to 362)	317 (275 to 365)	27 (14 to 49)	25 (21 to 30)	947 (819 to 1111)	408 (308 to 529)	463 (401 to 533)	40 (21 to 71)	36 (31 to 43)
Grenada	2032	653 (561 to 767)	282 (213 to 369)	318 (276 to 367)	28 (15 to 49)	25 (21 to 31)	954 (819 to 1120)	412 (311 to 539)	465 (403 to 536)	41 (21 to 72)	37 (31 to 45)
Grenada	2033	659 (566 to 782)	285 (216 to 375)	320 (277 to 369)	28 (15 to 50)	26 (22 to 32)	962 (826 to 1142)	416 (316 to 547)	467 (405 to 539)	41 (22 to 73)	38 (32 to 46)
Grenada	2034	664 (567 to 784)	288 (216 to 385)	322 (279 to 371)	28 (15 to 50)	26 (20 to 33)	970 (828 to 1144)	421 (315 to 563)	470 (407 to 542)	42 (22 to 73)	38 (29 to 48)

Grenada	2035	669 (572 to 797)	291 (218 to 386)	323 (280 to 373)	29 (15 to 51)	27 (0 to 33)	978 (835 to 1163)	425 (318 to 564)	472 (409 to 545)	42 (22 to 74)	39 (0 to 49)
Grenada	2036	675 (571 to 800)	294 (219 to 391)	325 (281 to 376)	29 (15 to 52)	26 (0 to 35)	985 (834 to 1168)	430 (320 to 570)	474 (411 to 548)	43 (22 to 75)	39 (0 to 51)
Grenada	2037	680 (574 to 811)	298 (221 to 392)	326 (283 to 378)	29 (16 to 52)	26 (0 to 35)	993 (838 to 1184)	435 (322 to 573)	477 (413 to 552)	43 (23 to 76)	38 (0 to 51)
Grenada	2038	685 (575 to 815)	301 (221 to 402)	329 (284 to 381)	30 (16 to 53)	25 (0 to 36)	1000 (840 to 1191)	439 (323 to 587)	480 (415 to 556)	44 (23 to 77)	37 (0 to 53)
Grenada	2039	691 (576 to 828)	305 (223 to 410)	331 (287 to 384)	30 (16 to 54)	24 (0 to 37)	1009 (841 to 1209)	446 (326 to 599)	483 (419 to 561)	44 (23 to 79)	35 (0 to 54)
Grenada	2040	697 (581 to 835)	309 (221 to 417)	333 (289 to 387)	31 (16 to 55)	24 (0 to 38)	1018 (849 to 1219)	451 (323 to 609)	487 (422 to 565)	45 (24 to 80)	35 (0 to 56)
Grenada	2041	704 (581 to 845)	314 (223 to 424)	336 (291 to 390)	31 (17 to 56)	23 (0 to 39)	1029 (848 to 1234)	459 (326 to 619)	491 (425 to 570)	46 (24 to 81)	34 (0 to 57)
Grenada	2042	711 (588 to 863)	318 (227 to 432)	339 (294 to 393)	32 (17 to 56)	22 (0 to 40)	1038 (858 to 1260)	465 (332 to 630)	495 (429 to 574)	46 (25 to 82)	32 (0 to 58)
Grenada	2043	718 (589 to 872)	324 (229 to 444)	342 (297 to 398)	32 (17 to 57)	20 (0 to 41)	1049 (861 to 1274)	473 (334 to 649)	500 (434 to 581)	47 (25 to 84)	29 (0 to 60)
Grenada	2044	727 (592 to 889)	330 (233 to 450)	346 (300 to 402)	33 (17 to 58)	18 (0 to 43)	1061 (865 to 1298)	481 (341 to 657)	505 (438 to 587)	48 (25 to 85)	27 (0 to 62)
Grenada	2045	734 (601 to 895)	335 (234 to 457)	349 (303 to 406)	33 (18 to 59)	16 (0 to 43)	1072 (878 to 1308)	489 (341 to 668)	510 (442 to 593)	49 (26 to 86)	24 (0 to 62)

Grenada	2046	742 (605 to 914)	340 (237 to 467)	353 (306 to 410)	34 (18 to 60)	15 (0 to 43)	1084 (884 to 1335)	497 (346 to 682)	515 (446 to 599)	49 (26 to 88)	22 (0 to 62)
Grenada	2047	750 (611 to 920)	345 (242 to 473)	356 (308 to 415)	34 (18 to 61)	14 (0 to 45)	1095 (892 to 1344)	504 (354 to 691)	520 (450 to 605)	50 (27 to 89)	20 (0 to 65)
Grenada	2048	759 (614 to 938)	351 (241 to 483)	360 (311 to 419)	35 (19 to 62)	13 (0 to 44)	1108 (896 to 1370)	513 (352 to 705)	525 (455 to 611)	51 (27 to 91)	19 (0 to 64)
Grenada	2049	766 (622 to 949)	356 (245 to 495)	363 (314 to 423)	35 (19 to 63)	12 (0 to 44)	1118 (908 to 1386)	519 (358 to 723)	530 (459 to 617)	52 (28 to 93)	17 (0 to 64)
Grenada	2050	773 (622 to 962)	361 (244 to 506)	366 (317 to 427)	36 (19 to 65)	10 (0 to 45)	1129 (908 to 1405)	527 (356 to 739)	534 (462 to 623)	53 (28 to 94)	15 (0 to 66)
Guam	1995	795 (637 to 988)	557 (415 to 722)	210 (146 to 300)	28 (14 to 51)	0 (0 to 0)	795 (637 to 988)	557 (415 to 722)	210 (146 to 300)	28 (14 to 51)	0 (0 to 0)
Guam	1996	815 (657 to 1012)	572 (433 to 745)	214 (150 to 309)	29 (14 to 53)	0 (0 to 0)	815 (657 to 1012)	572 (433 to 745)	214 (150 to 309)	29 (14 to 53)	0 (0 to 0)
Guam	1997	835 (675 to 1036)	588 (443 to 767)	219 (156 to 311)	28 (14 to 53)	0 (0 to 0)	835 (675 to 1036)	588 (443 to 767)	219 (156 to 311)	28 (14 to 53)	0 (0 to 0)
Guam	1998	845 (682 to 1040)	596 (446 to 784)	222 (156 to 313)	27 (13 to 50)	0 (0 to 0)	845 (682 to 1040)	596 (446 to 784)	222 (156 to 313)	27 (13 to 50)	0 (0 to 0)
Guam	1999	857 (686 to 1055)	606 (455 to 794)	225 (156 to 312)	26 (13 to 46)	0 (0 to 0)	857 (686 to 1055)	606 (455 to 794)	225 (156 to 312)	26 (13 to 46)	0 (0 to 0)
Guam	2000	876 (701 to 1074)	622 (465 to 816)	229 (159 to 323)	24 (12 to 43)	0 (0 to 0)	876 (701 to 1074)	622 (465 to 816)	229 (159 to 323)	24 (12 to 43)	0 (0 to 0)
Guam	2001	893 (717 to 1098)	636 (468 to 832)	234 (162 to 329)	22 (11 to 40)	0 (0 to 0)	893 (717 to 1098)	636 (468 to 832)	234 (162 to 329)	22 (11 to 40)	0 (0 to 0)
Guam	2002	921 (735 to 1131)	655 (488 to 853)	242 (166 to 341)	23 (11 to 42)	0 (0 to 0)	921 (735 to 1131)	655 (488 to 853)	242 (166 to 341)	23 (11 to 42)	0 (0 to 0)

Guam	2003	953 (761 to 1166)	675 (512 to 882)	252 (174 to 356)	26 (12 to 47)	0 (0 to 0)	953 (761 to 1166)	675 (512 to 882)	252 (174 to 356)	26 (12 to 47)	0 (0 to 0)
Guam	2004	1019 (821 to 1243)	722 (546 to 947)	267 (183 to 379)	30 (15 to 57)	0 (0 to 0)	1019 (821 to 1243)	722 (546 to 947)	267 (183 to 379)	30 (15 to 57)	0 (0 to 0)
Guam	2005	1071 (866 to 1307)	756 (573 to 981)	279 (194 to 397)	36 (17 to 66)	0 (0 to 0)	1071 (866 to 1307)	756 (573 to 981)	279 (194 to 397)	36 (17 to 66)	0 (0 to 0)
Guam	2006	1070 (864 to 1314)	748 (567 to 973)	282 (197 to 406)	41 (20 to 76)	0 (0 to 0)	1070 (864 to 1314)	748 (567 to 973)	282 (197 to 406)	41 (20 to 76)	0 (0 to 0)
Guam	2007	1100 (886 to 1356)	761 (572 to 998)	289 (200 to 413)	49 (24 to 91)	0 (0 to 0)	1100 (886 to 1356)	761 (572 to 998)	289 (200 to 413)	49 (24 to 91)	0 (0 to 0)
Guam	2008	1137 (918 to 1408)	777 (584 to 1024)	297 (205 to 426)	63 (30 to 117)	0 (0 to 0)	1137 (918 to 1408)	777 (584 to 1024)	297 (205 to 426)	63 (30 to 117)	0 (0 to 0)
Guam	2009	1162 (933 to 1432)	782 (591 to 1040)	304 (210 to 423)	75 (37 to 141)	0 (0 to 0)	1162 (933 to 1432)	782 (591 to 1040)	304 (210 to 423)	75 (37 to 141)	0 (0 to 0)
Guam	2010	1197 (959 to 1476)	797 (599 to 1044)	311 (213 to 434)	89 (43 to 163)	0 (0 to 0)	1197 (959 to 1476)	797 (599 to 1044)	311 (213 to 434)	89 (43 to 163)	0 (0 to 0)
Guam	2011	1229 (976 to 1515)	810 (612 to 1065)	318 (217 to 439)	101 (49 to 182)	0 (0 to 0)	1229 (976 to 1515)	810 (612 to 1065)	318 (217 to 439)	101 (49 to 182)	0 (0 to 0)
Guam	2012	1279 (1009 to 1569)	837 (636 to 1108)	327 (224 to 458)	115 (55 to 211)	0 (0 to 0)	1279 (1009 to 1569)	837 (636 to 1108)	327 (224 to 458)	115 (55 to 211)	0 (0 to 0)

Guam	2013	1330 (1053 to 1646)	865 (652 to 1138)	336 (232 to 463)	128 (61 to 247)	0 (0 to 0)	1330 (1053 to 1646)	865 (652 to 1138)	336 (232 to 463)	128 (61 to 247)	0 (0 to 0)
Guam	2014	1378 (1095 to 1719)	892 (673 to 1157)	346 (240 to 478)	140 (65 to 268)	0 (0 to 0)	1378 (1095 to 1719)	892 (673 to 1157)	346 (240 to 478)	140 (65 to 268)	0 (0 to 0)
Guam	2015	1413 (1135 to 1765)	911 (687 to 1186)	353 (241 to 481)	149 (70 to 287)	0 (0 to 0)	1413 (1135 to 1765)	911 (687 to 1186)	353 (241 to 481)	149 (70 to 287)	0 (0 to 0)
Guam	2016	1446 (1166 to 1798)	931 (704 to 1202)	358 (247 to 491)	157 (72 to 294)	0 (0 to 0)	1446 (1166 to 1798)	931 (704 to 1202)	358 (247 to 491)	157 (72 to 294)	0 (0 to 0)
Guam	2017	1468 (1143 to 1903)	944 (710 to 1224)	362 (248 to 501)	162 (76 to 296)	0 (0 to 0)	1468 (1143 to 1903)	944 (710 to 1224)	362 (248 to 501)	162 (76 to 296)	0 (0 to 0)
Guam	2018	1490 (1129 to 1921)	962 (722 to 1246)	365 (250 to 504)	164 (77 to 300)	0 (0 to 0)	1490 (1129 to 1921)	962 (722 to 1246)	365 (250 to 504)	164 (77 to 300)	0 (0 to 0)
Guam	2019	1506 (1158 to 1939)	975 (732 to 1266)	365 (250 to 505)	166 (77 to 303)	0 (0 to 0)	1506 (1158 to 1939)	975 (732 to 1266)	365 (250 to 505)	166 (77 to 303)	0 (0 to 0)
Guam	2020	1529 (1159 to 1992)	992 (745 to 1288)	368 (252 to 508)	168 (78 to 308)	0 (0 to 0)	1529 (1159 to 1992)	992 (745 to 1288)	368 (252 to 508)	168 (78 to 308)	0 (0 to 0)
Guam	2021	1536 (1192 to 1988)	999 (752 to 1301)	368 (253 to 509)	169 (79 to 310)	0 (0 to 0)	1536 (1192 to 1988)	999 (752 to 1301)	368 (253 to 509)	169 (79 to 310)	0 (0 to 0)
Guam	2022	1541 (1177 to 2015)	1004 (754 to 1304)	367 (253 to 508)	170 (79 to 311)	0 (0 to 0)	1541 (1177 to 2015)	1004 (754 to 1304)	367 (253 to 508)	170 (79 to 311)	0 (0 to 0)

Guam	2023	1548 (1187 to 1987)	1011 (760 to 1310)	366 (252 to 505)	171 (80 to 312)	0 (0 to 0)	1548 (1187 to 1987)	1011 (760 to 1310)	366 (252 to 505)	171 (80 to 312)	0 (0 to 0)
Guam	2024	1555 (1198 to 2016)	1018 (769 to 1314)	365 (252 to 503)	171 (80 to 314)	0 (0 to 0)	1555 (1198 to 2016)	1018 (769 to 1314)	365 (252 to 503)	171 (80 to 314)	0 (0 to 0)
Guam	2025	1561 (1187 to 2035)	1025 (776 to 1328)	364 (251 to 501)	172 (81 to 315)	0 (0 to 0)	1561 (1187 to 2035)	1025 (776 to 1328)	364 (251 to 501)	172 (81 to 315)	0 (0 to 0)
Guam	2026	1567 (1195 to 2037)	1031 (781 to 1338)	363 (251 to 500)	172 (81 to 316)	0 (0 to 0)	1567 (1195 to 2037)	1031 (781 to 1338)	363 (251 to 500)	172 (81 to 316)	0 (0 to 0)
Guam	2027	1575 (1209 to 2054)	1039 (788 to 1345)	363 (250 to 500)	173 (81 to 318)	0 (0 to 0)	1575 (1209 to 2054)	1039 (788 to 1345)	363 (250 to 500)	173 (81 to 318)	0 (0 to 0)
Guam	2028	1585 (1209 to 2053)	1048 (795 to 1359)	363 (250 to 500)	174 (82 to 320)	0 (0 to 0)	1585 (1209 to 2053)	1048 (795 to 1359)	363 (250 to 500)	174 (82 to 320)	0 (0 to 0)
Guam	2029	1596 (1231 to 2085)	1058 (802 to 1378)	363 (250 to 502)	175 (82 to 323)	0 (0 to 0)	1596 (1231 to 2085)	1058 (802 to 1378)	363 (250 to 502)	175 (82 to 323)	0 (0 to 0)
Guam	2030	1607 (1236 to 2088)	1068 (808 to 1390)	363 (250 to 503)	176 (83 to 326)	0 (0 to 0)	1607 (1236 to 2088)	1068 (808 to 1390)	363 (250 to 503)	176 (83 to 326)	0 (0 to 0)
Guam	2031	1618 (1235 to 2108)	1078 (814 to 1402)	363 (250 to 504)	178 (84 to 328)	0 (0 to 0)	1618 (1235 to 2108)	1078 (814 to 1402)	363 (250 to 504)	178 (84 to 328)	0 (0 to 0)
Guam	2032	1630 (1249 to 2139)	1088 (821 to 1416)	363 (249 to 505)	179 (85 to 331)	0 (0 to 0)	1630 (1249 to 2139)	1088 (821 to 1416)	363 (249 to 505)	179 (85 to 331)	0 (0 to 0)

Guam	2033	1641 (1248 to 2138)	1098 (826 to 1431)	362 (249 to 505)	180 (85 to 334)	0 (0 to 0)	1641 (1248 to 2138)	1098 (826 to 1431)	362 (249 to 505)	180 (85 to 334)	0 (0 to 0)
Guam	2034	1653 (1265 to 2157)	1109 (832 to 1442)	363 (249 to 505)	181 (86 to 336)	0 (0 to 0)	1653 (1265 to 2157)	1109 (832 to 1442)	363 (249 to 505)	181 (86 to 336)	0 (0 to 0)
Guam	2035	1666 (1259 to 2202)	1121 (840 to 1456)	363 (249 to 505)	183 (87 to 340)	0 (0 to 0)	1666 (1259 to 2202)	1121 (840 to 1456)	363 (249 to 505)	183 (87 to 340)	0 (0 to 0)
Guam	2036	1680 (1274 to 2198)	1133 (848 to 1474)	363 (250 to 505)	184 (87 to 343)	0 (0 to 0)	1680 (1274 to 2198)	1133 (848 to 1474)	363 (250 to 505)	184 (87 to 343)	0 (0 to 0)
Guam	2037	1696 (1294 to 2221)	1146 (858 to 1494)	364 (250 to 505)	186 (88 to 347)	0 (0 to 0)	1696 (1294 to 2221)	1146 (858 to 1494)	364 (250 to 505)	186 (88 to 347)	0 (0 to 0)
Guam	2038	1713 (1275 to 2246)	1160 (868 to 1511)	365 (251 to 507)	188 (88 to 351)	0 (0 to 0)	1713 (1275 to 2246)	1160 (868 to 1511)	365 (251 to 507)	188 (88 to 351)	0 (0 to 0)
Guam	2039	1730 (1323 to 2251)	1175 (878 to 1532)	366 (251 to 509)	190 (89 to 355)	0 (0 to 0)	1730 (1323 to 2251)	1175 (878 to 1532)	366 (251 to 509)	190 (89 to 355)	0 (0 to 0)
Guam	2040	1748 (1308 to 2296)	1189 (888 to 1548)	367 (252 to 512)	192 (90 to 359)	0 (0 to 0)	1748 (1308 to 2296)	1189 (888 to 1548)	367 (252 to 512)	192 (90 to 359)	0 (0 to 0)
Guam	2041	1765 (1350 to 2307)	1204 (898 to 1567)	368 (252 to 514)	194 (91 to 363)	0 (0 to 0)	1765 (1350 to 2307)	1204 (898 to 1567)	368 (252 to 514)	194 (91 to 363)	0 (0 to 0)
Guam	2042	1783 (1353 to 2349)	1219 (911 to 1589)	368 (252 to 516)	196 (91 to 367)	0 (0 to 0)	1783 (1353 to 2349)	1219 (911 to 1589)	368 (252 to 516)	196 (91 to 367)	0 (0 to 0)

Guam	2043	1802 (1370 to 2334)	1235 (921 to 1611)	369 (252 to 519)	198 (92 to 370)	0 (0 to 0)	1802 (1370 to 2334)	1235 (921 to 1611)	369 (252 to 519)	198 (92 to 370)	0 (0 to 0)
Guam	2044	1821 (1372 to 2401)	1251 (931 to 1634)	370 (252 to 521)	200 (93 to 373)	0 (0 to 0)	1821 (1372 to 2401)	1251 (931 to 1634)	370 (252 to 521)	200 (93 to 373)	0 (0 to 0)
Guam	2045	1841 (1405 to 2402)	1267 (942 to 1657)	372 (253 to 523)	202 (94 to 375)	0 (0 to 0)	1841 (1405 to 2402)	1267 (942 to 1657)	372 (253 to 523)	202 (94 to 375)	0 (0 to 0)
Guam	2046	1860 (1405 to 2446)	1283 (953 to 1677)	373 (253 to 525)	204 (95 to 379)	0 (0 to 0)	1860 (1405 to 2446)	1283 (953 to 1677)	373 (253 to 525)	204 (95 to 379)	0 (0 to 0)
Guam	2047	1879 (1412 to 2455)	1299 (964 to 1697)	374 (254 to 526)	207 (96 to 384)	0 (0 to 0)	1879 (1412 to 2455)	1299 (964 to 1697)	374 (254 to 526)	207 (96 to 384)	0 (0 to 0)
Guam	2048	1898 (1424 to 2483)	1315 (976 to 1716)	375 (254 to 527)	209 (98 to 388)	0 (0 to 0)	1898 (1424 to 2483)	1315 (976 to 1716)	375 (254 to 527)	209 (98 to 388)	0 (0 to 0)
Guam	2049	1918 (1463 to 2516)	1331 (989 to 1736)	376 (255 to 528)	211 (99 to 393)	0 (0 to 0)	1918 (1463 to 2516)	1331 (989 to 1736)	376 (255 to 528)	211 (99 to 393)	0 (0 to 0)
Guam	2050	1937 (1436 to 2546)	1347 (1000 to 1757)	377 (255 to 530)	213 (100 to 396)	0 (0 to 0)	1937 (1436 to 2546)	1347 (1000 to 1757)	377 (255 to 530)	213 (100 to 396)	0 (0 to 0)
Guatemala	1995	175 (150 to 202)	65 (52 to 79)	103 (83 to 126)	4 (2 to 7)	3 (3 to 3)	325 (279 to 376)	121 (97 to 147)	193 (155 to 234)	7 (3 to 13)	5 (5 to 5)
Guatemala	1996	175 (152 to 199)	64 (52 to 77)	104 (85 to 125)	4 (2 to 7)	3 (3 to 3)	326 (283 to 370)	120 (97 to 144)	193 (159 to 232)	7 (4 to 13)	5 (5 to 5)
Guatemala	1997	192 (171 to 214)	68 (56 to 81)	107 (89 to 126)	4 (2 to 8)	14 (14 to 14)	358 (318 to 399)	126 (104 to 151)	198 (167 to 234)	8 (4 to 14)	26 (26 to 26)

Guatemala	1998	191 (169 to 213)	72 (60 to 87)	110 (94 to 128)	5 (2 to 9)	3 (3 to 3)	355 (316 to 396)	135 (112 to 161)	205 (176 to 239)	9 (4 to 16)	6 (6 to 6)
Guatemala	1999	207 (185 to 230)	76 (64 to 91)	118 (101 to 137)	6 (3 to 11)	7 (7 to 7)	386 (344 to 429)	142 (119 to 169)	220 (189 to 255)	11 (6 to 20)	12 (12 to 12)
Guatemala	2000	218 (195 to 243)	77 (65 to 92)	131 (113 to 150)	7 (3 to 12)	4 (4 to 4)	406 (363 to 453)	144 (121 to 172)	243 (210 to 280)	12 (6 to 22)	7 (7 to 7)
Guatemala	2001	235 (210 to 261)	80 (66 to 94)	143 (122 to 163)	8 (4 to 13)	5 (5 to 5)	438 (391 to 486)	149 (124 to 176)	266 (228 to 304)	14 (7 to 25)	9 (9 to 9)
Guatemala	2002	240 (214 to 266)	79 (66 to 94)	149 (127 to 170)	8 (4 to 14)	3 (3 to 3)	446 (398 to 495)	148 (124 to 174)	277 (237 to 316)	15 (8 to 26)	6 (6 to 6)
Guatemala	2003	257 (231 to 285)	85 (71 to 100)	159 (136 to 181)	9 (5 to 16)	5 (5 to 5)	479 (429 to 531)	158 (133 to 186)	295 (253 to 336)	17 (9 to 29)	9 (9 to 9)
Guatemala	2004	248 (223 to 274)	80 (67 to 94)	156 (135 to 178)	9 (5 to 16)	3 (3 to 3)	462 (415 to 511)	149 (125 to 175)	290 (252 to 331)	18 (9 to 30)	6 (6 to 6)
Guatemala	2005	250 (224 to 278)	80 (67 to 95)	156 (137 to 180)	10 (5 to 17)	3 (3 to 3)	465 (416 to 518)	149 (125 to 177)	291 (254 to 336)	18 (10 to 31)	7 (7 to 7)
Guatemala	2006	260 (233 to 290)	85 (71 to 102)	161 (140 to 187)	10 (5 to 17)	4 (4 to 4)	484 (434 to 540)	159 (133 to 191)	300 (261 to 348)	18 (10 to 32)	8 (8 to 8)
Guatemala	2007	262 (236 to 291)	87 (73 to 104)	160 (139 to 185)	10 (6 to 18)	5 (5 to 5)	488 (440 to 543)	162 (136 to 193)	299 (260 to 344)	19 (11 to 34)	9 (9 to 9)
Guatemala	2008	260 (235 to 288)	86 (73 to 102)	155 (135 to 177)	11 (6 to 20)	7 (7 to 7)	483 (437 to 535)	160 (136 to 190)	290 (251 to 330)	21 (11 to 36)	13 (13 to 13)
Guatemala	2009	258 (233 to 285)	88 (75 to 105)	153 (133 to 174)	11 (6 to 19)	5 (5 to 5)	480 (433 to 531)	164 (140 to 195)	285 (248 to 324)	21 (12 to 36)	10 (10 to 10)
Guatemala	2010	256 (232 to 284)	88 (74 to 105)	150 (131 to 170)	12 (7 to 20)	6 (6 to 6)	477 (431 to 529)	164 (138 to 196)	280 (243 to 317)	22 (12 to 37)	10 (10 to 10)
Guatemala	2011	257 (232 to 285)	89 (75 to 105)	149 (129 to 170)	12 (7 to 21)	7 (7 to 7)	478 (432 to 530)	165 (140 to 196)	277 (240 to 317)	23 (12 to 39)	13 (13 to 13)
Guatemala	2012	253 (227 to 282)	89 (75 to 105)	147 (128 to 169)	13 (7 to 22)	5 (5 to 5)	471 (422 to 524)	165 (140 to 196)	274 (238 to 314)	23 (13 to 40)	9 (9 to 9)

Guatemala	2013	260 (232 to 291)	92 (78 to 110)	147 (127 to 169)	14 (8 to 23)	8 (8 to 8)	485 (433 to 542)	171 (144 to 204)	274 (237 to 315)	25 (14 to 43)	15 (15 to 15)
Guatemala	2014	260 (232 to 291)	95 (80 to 114)	146 (126 to 168)	15 (8 to 26)	4 (4 to 4)	484 (432 to 543)	176 (149 to 211)	272 (234 to 313)	28 (16 to 48)	8 (8 to 8)
Guatemala	2015	276 (247 to 308)	94 (78 to 113)	144 (122 to 167)	16 (9 to 28)	22 (22 to 22)	514 (460 to 574)	175 (146 to 210)	268 (227 to 312)	30 (17 to 52)	40 (40 to 40)
Guatemala	2016	262 (229 to 296)	96 (79 to 116)	144 (120 to 171)	19 (10 to 33)	3 (3 to 3)	487 (426 to 551)	178 (147 to 216)	268 (223 to 318)	36 (19 to 61)	6 (6 to 6)
Guatemala	2017	265 (227 to 311)	96 (78 to 117)	143 (118 to 174)	21 (11 to 37)	4 (4 to 4)	493 (424 to 580)	179 (145 to 219)	267 (219 to 325)	39 (20 to 69)	7 (7 to 7)
Guatemala	2018	269 (228 to 318)	99 (80 to 122)	145 (119 to 175)	21 (11 to 38)	4 (4 to 4)	500 (424 to 591)	184 (148 to 227)	269 (221 to 327)	40 (21 to 70)	7 (7 to 7)
Guatemala	2019	272 (233 to 322)	101 (81 to 124)	146 (120 to 177)	22 (11 to 38)	4 (4 to 4)	507 (433 to 599)	188 (151 to 231)	272 (223 to 330)	41 (21 to 71)	7 (7 to 7)
Guatemala	2020	277 (234 to 329)	103 (83 to 127)	148 (121 to 179)	22 (12 to 39)	4 (3 to 4)	516 (436 to 613)	192 (154 to 236)	275 (226 to 334)	41 (21 to 73)	7 (6 to 7)
Guatemala	2021	282 (240 to 333)	106 (84 to 129)	150 (123 to 181)	23 (12 to 40)	4 (3 to 4)	525 (446 to 619)	197 (157 to 241)	279 (229 to 337)	42 (22 to 74)	7 (6 to 7)
Guatemala	2022	287 (242 to 341)	108 (86 to 133)	151 (124 to 183)	23 (12 to 41)	4 (3 to 4)	534 (451 to 634)	201 (160 to 247)	282 (232 to 342)	43 (22 to 76)	7 (6 to 8)
Guatemala	2023	291 (248 to 344)	111 (88 to 136)	153 (126 to 186)	24 (12 to 42)	4 (3 to 4)	542 (462 to 640)	206 (164 to 253)	285 (234 to 346)	44 (23 to 77)	7 (6 to 8)
Guatemala	2024	296 (250 to 351)	113 (89 to 139)	155 (127 to 188)	24 (13 to 42)	4 (3 to 4)	551 (466 to 654)	210 (166 to 259)	288 (237 to 350)	45 (23 to 79)	7 (6 to 8)
Guatemala	2025	300 (254 to 353)	115 (91 to 143)	156 (128 to 190)	25 (13 to 43)	4 (3 to 4)	559 (473 to 657)	215 (170 to 266)	291 (239 to 353)	46 (24 to 80)	7 (6 to 8)
Guatemala	2026	304 (257 to 361)	118 (93 to 145)	158 (130 to 192)	25 (13 to 44)	4 (3 to 4)	567 (478 to 672)	219 (173 to 269)	294 (242 to 357)	47 (24 to 82)	7 (6 to 8)
Guatemala	2027	309 (262 to 367)	120 (95 to 148)	159 (131 to 193)	26 (13 to 45)	4 (3 to 4)	575 (487 to 683)	224 (176 to 275)	296 (244 to 360)	48 (25 to 83)	7 (6 to 8)

Guatemala	2028	313 (264 to 371)	122 (97 to 152)	161 (132 to 195)	26 (13 to 45)	4 (3 to 5)	583 (491 to 691)	228 (180 to 284)	299 (246 to 363)	49 (25 to 85)	7 (6 to 8)
Guatemala	2029	318 (269 to 381)	125 (99 to 156)	162 (133 to 197)	27 (14 to 46)	4 (3 to 5)	592 (501 to 709)	233 (185 to 290)	302 (248 to 366)	49 (26 to 86)	7 (6 to 9)
Guatemala	2030	322 (270 to 382)	128 (101 to 161)	163 (134 to 198)	27 (14 to 47)	4 (3 to 5)	600 (504 to 712)	238 (187 to 300)	304 (250 to 369)	50 (26 to 88)	7 (6 to 9)
Guatemala	2031	327 (278 to 393)	131 (103 to 166)	165 (135 to 200)	28 (14 to 48)	4 (3 to 5)	609 (517 to 732)	244 (192 to 308)	307 (252 to 372)	51 (27 to 90)	7 (6 to 9)
Guatemala	2032	332 (279 to 395)	134 (105 to 168)	166 (136 to 202)	28 (15 to 49)	4 (3 to 5)	618 (519 to 735)	249 (196 to 312)	309 (254 to 375)	52 (27 to 92)	7 (6 to 9)
Guatemala	2033	337 (285 to 405)	137 (108 to 172)	168 (137 to 204)	29 (15 to 50)	4 (3 to 5)	628 (531 to 754)	255 (201 to 320)	312 (256 to 379)	53 (28 to 94)	8 (6 to 9)
Guatemala	2034	342 (287 to 410)	140 (110 to 175)	169 (139 to 206)	29 (15 to 51)	4 (3 to 5)	638 (534 to 763)	261 (204 to 325)	315 (258 to 383)	54 (28 to 96)	8 (6 to 9)
Guatemala	2035	348 (294 to 421)	143 (113 to 180)	171 (140 to 208)	30 (15 to 52)	4 (3 to 5)	648 (547 to 783)	267 (209 to 335)	318 (261 to 387)	55 (29 to 97)	8 (6 to 9)
Guatemala	2036	354 (296 to 426)	146 (114 to 184)	173 (141 to 210)	30 (16 to 53)	4 (3 to 5)	658 (550 to 793)	273 (213 to 342)	321 (263 to 391)	56 (29 to 100)	8 (6 to 10)
Guatemala	2037	360 (305 to 437)	150 (117 to 189)	174 (143 to 212)	31 (16 to 55)	4 (3 to 5)	670 (567 to 813)	280 (217 to 352)	325 (266 to 395)	57 (30 to 102)	8 (6 to 10)
Guatemala	2038	366 (304 to 440)	154 (119 to 194)	176 (144 to 215)	31 (16 to 56)	4 (4 to 5)	681 (566 to 820)	287 (221 to 362)	328 (269 to 400)	59 (30 to 104)	8 (7 to 10)
Guatemala	2039	371 (312 to 451)	157 (121 to 201)	178 (145 to 217)	32 (17 to 57)	4 (4 to 5)	692 (581 to 841)	293 (226 to 375)	331 (271 to 403)	60 (31 to 106)	8 (7 to 10)
Guatemala	2040	377 (313 to 455)	161 (124 to 205)	179 (146 to 218)	33 (17 to 58)	4 (4 to 6)	702 (582 to 848)	300 (231 to 382)	333 (273 to 407)	61 (31 to 107)	8 (7 to 10)
Guatemala	2041	382 (320 to 467)	165 (127 to 209)	180 (147 to 220)	33 (17 to 59)	4 (4 to 6)	712 (596 to 870)	306 (236 to 389)	335 (274 to 410)	62 (32 to 109)	8 (7 to 11)
Guatemala	2042	387 (321 to 467)	168 (129 to 214)	181 (148 to 222)	34 (17 to 59)	5 (4 to 6)	721 (598 to 870)	313 (240 to 399)	337 (276 to 414)	63 (32 to 111)	8 (7 to 11)

Guatemala	2043	393 (328 to 477)	172 (132 to 218)	182 (149 to 223)	34 (17 to 60)	5 (4 to 6)	731 (611 to 889)	320 (246 to 407)	339 (277 to 416)	63 (33 to 112)	9 (7 to 11)
Guatemala	2044	397 (331 to 479)	175 (134 to 223)	183 (149 to 224)	35 (18 to 61)	5 (4 to 6)	740 (617 to 892)	326 (250 to 414)	341 (278 to 418)	64 (33 to 114)	9 (7 to 11)
Guatemala	2045	402 (337 to 490)	179 (137 to 228)	184 (150 to 225)	35 (18 to 62)	5 (4 to 6)	749 (627 to 913)	333 (256 to 425)	342 (279 to 420)	65 (33 to 116)	9 (7 to 11)
Guatemala	2046	407 (338 to 491)	182 (140 to 235)	185 (151 to 226)	36 (18 to 63)	5 (4 to 6)	758 (630 to 915)	340 (260 to 437)	344 (280 to 421)	66 (34 to 117)	9 (7 to 12)
Guatemala	2047	412 (343 to 501)	186 (143 to 238)	185 (151 to 227)	36 (18 to 64)	5 (4 to 7)	767 (639 to 934)	346 (266 to 444)	345 (281 to 423)	67 (34 to 119)	9 (7 to 12)
Guatemala	2048	417 (344 to 505)	190 (145 to 242)	186 (152 to 229)	36 (19 to 65)	5 (4 to 7)	777 (641 to 940)	353 (269 to 451)	346 (282 to 426)	68 (35 to 120)	9 (7 to 12)
Guatemala	2049	422 (351 to 514)	193 (147 to 249)	187 (152 to 230)	37 (19 to 66)	5 (4 to 7)	786 (654 to 957)	360 (273 to 463)	348 (283 to 429)	69 (35 to 122)	9 (7 to 12)
Guatemala	2050	427 (354 to 517)	197 (149 to 254)	187 (152 to 232)	37 (19 to 66)	5 (4 to 7)	795 (660 to 963)	367 (278 to 473)	349 (284 to 432)	70 (36 to 124)	10 (8 to 13)
Guinea	1995	21 (18 to 27)	2 (1 to 3)	13 (9 to 18)	0 (0 to 0)	7 (7 to 7)	53 (44 to 66)	5 (4 to 6)	32 (23 to 44)	0 (0 to 1)	16 (16 to 16)
Guinea	1996	18 (14 to 23)	2 (2 to 3)	13 (9 to 18)	0 (0 to 0)	2 (2 to 2)	44 (35 to 57)	6 (4 to 7)	33 (23 to 45)	0 (0 to 1)	6 (6 to 6)
Guinea	1997	21 (17 to 26)	2 (2 to 3)	13 (10 to 19)	0 (0 to 0)	5 (5 to 5)	51 (41 to 64)	6 (4 to 8)	33 (23 to 46)	0 (0 to 1)	12 (12 to 12)
Guinea	1998	20 (16 to 25)	2 (2 to 3)	13 (9 to 19)	0 (0 to 0)	4 (4 to 4)	49 (39 to 62)	6 (4 to 8)	33 (23 to 46)	0 (0 to 1)	10 (10 to 10)
Guinea	1999	19 (15 to 24)	3 (2 to 3)	14 (10 to 19)	0 (0 to 0)	3 (3 to 3)	47 (37 to 59)	6 (5 to 8)	34 (24 to 47)	0 (0 to 1)	6 (6 to 6)
Guinea	2000	20 (16 to 25)	3 (2 to 4)	14 (10 to 19)	0 (0 to 0)	3 (3 to 3)	49 (40 to 62)	6 (5 to 9)	34 (24 to 47)	0 (0 to 1)	8 (8 to 8)
Guinea	2001	23 (18 to 28)	3 (2 to 4)	15 (10 to 20)	0 (0 to 0)	5 (5 to 5)	56 (45 to 70)	7 (6 to 10)	36 (25 to 50)	0 (0 to 1)	12 (12 to 12)

Guinea	2002	21 (17 to 27)	3 (2 to 4)	15 (11 to 20)	0 (0 to 0)	3 (3 to 3)	53 (42 to 67)	8 (6 to 10)	36 (26 to 50)	0 (0 to 1)	8 (8 to 8)
Guinea	2003	21 (16 to 26)	3 (2 to 4)	15 (10 to 20)	0 (0 to 0)	3 (3 to 3)	51 (40 to 65)	7 (5 to 9)	36 (25 to 49)	0 (0 to 1)	8 (8 to 8)
Guinea	2004	20 (16 to 26)	2 (2 to 3)	14 (10 to 20)	0 (0 to 0)	4 (4 to 4)	50 (39 to 64)	6 (4 to 8)	35 (24 to 48)	0 (0 to 1)	9 (9 to 9)
Guinea	2005	20 (16 to 25)	2 (2 to 3)	14 (10 to 19)	0 (0 to 0)	4 (4 to 4)	49 (38 to 62)	5 (4 to 7)	34 (24 to 47)	0 (0 to 1)	10 (10 to 10)
Guinea	2006	22 (17 to 27)	2 (2 to 3)	15 (11 to 21)	0 (0 to 0)	4 (4 to 4)	54 (42 to 68)	5 (4 to 7)	37 (26 to 51)	0 (0 to 1)	11 (11 to 11)
Guinea	2007	19 (15 to 25)	2 (1 to 2)	14 (10 to 20)	0 (0 to 0)	3 (3 to 3)	47 (36 to 61)	4 (3 to 6)	35 (24 to 48)	0 (0 to 1)	8 (8 to 8)
Guinea	2008	21 (17 to 27)	2 (2 to 3)	15 (10 to 20)	0 (0 to 0)	4 (4 to 4)	52 (41 to 66)	5 (4 to 7)	36 (25 to 50)	0 (0 to 1)	10 (10 to 10)
Guinea	2009	22 (17 to 28)	3 (2 to 3)	16 (11 to 22)	0 (0 to 0)	3 (3 to 3)	54 (42 to 69)	6 (5 to 8)	40 (28 to 54)	1 (0 to 1)	8 (8 to 8)
Guinea	2010	26 (20 to 32)	3 (3 to 4)	18 (12 to 24)	0 (0 to 1)	4 (4 to 4)	63 (50 to 80)	8 (6 to 11)	43 (31 to 60)	1 (0 to 1)	11 (11 to 11)
Guinea	2011	24 (19 to 31)	3 (3 to 4)	17 (12 to 24)	0 (0 to 1)	3 (3 to 3)	60 (47 to 77)	8 (6 to 10)	43 (31 to 60)	1 (0 to 1)	8 (8 to 8)
Guinea	2012	29 (24 to 37)	4 (3 to 5)	19 (14 to 27)	1 (0 to 1)	5 (5 to 5)	72 (58 to 91)	10 (8 to 13)	48 (34 to 66)	1 (1 to 2)	13 (13 to 13)
Guinea	2013	29 (23 to 36)	4 (3 to 5)	20 (14 to 27)	1 (0 to 2)	4 (4 to 4)	70 (56 to 89)	10 (8 to 13)	49 (35 to 67)	2 (1 to 4)	9 (9 to 9)
Guinea	2014	47 (40 to 55)	5 (4 to 6)	22 (16 to 30)	2 (1 to 4)	18 (18 to 18)	115 (99 to 136)	12 (9 to 15)	55 (39 to 75)	5 (3 to 9)	44 (44 to 44)
Guinea	2015	50 (42 to 59)	5 (4 to 6)	24 (17 to 33)	4 (2 to 7)	17 (17 to 17)	122 (104 to 145)	12 (9 to 16)	58 (42 to 80)	9 (4 to 17)	42 (42 to 42)
Guinea	2016	46 (39 to 55)	5 (4 to 6)	23 (17 to 31)	4 (2 to 7)	14 (14 to 14)	113 (95 to 134)	12 (9 to 16)	57 (41 to 76)	10 (5 to 18)	34 (34 to 34)

Guinea	2017	45 (36 to 55)	6 (5 to 8)	24 (17 to 33)	5 (2 to 8)	10 (10 to 10)	110 (90 to 135)	15 (11 to 19)	60 (42 to 81)	12 (5 to 21)	24 (24 to 24)
Guinea	2018	45 (36 to 55)	6 (4 to 7)	25 (18 to 34)	5 (2 to 8)	9 (9 to 9)	111 (89 to 136)	14 (11 to 18)	62 (44 to 84)	12 (5 to 21)	22 (22 to 23)
Guinea	2019	48 (39 to 59)	7 (5 to 9)	26 (19 to 36)	5 (2 to 10)	9 (9 to 9)	117 (95 to 145)	17 (13 to 22)	65 (46 to 87)	13 (6 to 24)	22 (22 to 22)
Guinea	2020	49 (39 to 61)	7 (6 to 9)	27 (19 to 37)	6 (3 to 10)	9 (9 to 10)	121 (96 to 149)	18 (14 to 23)	67 (47 to 90)	14 (6 to 25)	22 (21 to 23)
Guinea	2021	50 (40 to 62)	8 (6 to 10)	28 (19 to 37)	6 (3 to 10)	9 (8 to 10)	123 (99 to 152)	19 (14 to 24)	68 (48 to 92)	14 (7 to 25)	22 (21 to 24)
Guinea	2022	51 (41 to 63)	8 (6 to 10)	28 (20 to 38)	6 (3 to 11)	9 (8 to 10)	125 (100 to 156)	19 (14 to 25)	69 (49 to 94)	14 (7 to 26)	22 (20 to 25)
Guinea	2023	52 (42 to 64)	8 (6 to 11)	29 (20 to 39)	6 (3 to 11)	9 (8 to 10)	128 (103 to 158)	20 (15 to 26)	71 (50 to 95)	15 (7 to 27)	22 (20 to 25)
Guinea	2024	53 (42 to 66)	8 (6 to 11)	29 (21 to 39)	6 (3 to 11)	9 (8 to 10)	130 (103 to 162)	21 (15 to 27)	72 (51 to 97)	15 (7 to 28)	22 (20 to 25)
Guinea	2025	54 (43 to 67)	9 (6 to 12)	30 (21 to 40)	6 (3 to 12)	9 (8 to 10)	132 (106 to 164)	21 (15 to 28)	73 (52 to 99)	16 (7 to 29)	22 (19 to 25)
Guinea	2026	55 (43 to 69)	9 (6 to 12)	30 (22 to 41)	7 (3 to 12)	9 (8 to 10)	135 (106 to 169)	22 (16 to 30)	75 (53 to 101)	16 (8 to 30)	22 (19 to 25)
Guinea	2027	56 (45 to 70)	9 (7 to 13)	31 (22 to 42)	7 (3 to 12)	9 (8 to 10)	138 (111 to 172)	23 (16 to 31)	77 (54 to 104)	17 (8 to 31)	22 (19 to 25)
Guinea	2028	57 (45 to 72)	10 (7 to 13)	32 (23 to 43)	7 (3 to 13)	9 (8 to 10)	141 (111 to 178)	24 (17 to 32)	79 (56 to 107)	17 (8 to 32)	21 (19 to 25)
Guinea	2029	59 (46 to 74)	10 (7 to 14)	33 (23 to 45)	7 (3 to 13)	9 (7 to 10)	145 (114 to 182)	25 (17 to 34)	81 (57 to 110)	18 (8 to 33)	21 (18 to 25)
Guinea	2030	60 (47 to 77)	10 (7 to 14)	34 (24 to 46)	8 (4 to 14)	9 (7 to 10)	149 (116 to 188)	26 (18 to 36)	83 (59 to 113)	19 (9 to 34)	21 (18 to 25)
Guinea	2031	62 (49 to 78)	11 (7 to 15)	35 (25 to 47)	8 (4 to 14)	9 (7 to 10)	152 (120 to 192)	27 (18 to 37)	86 (60 to 115)	19 (9 to 35)	21 (18 to 25)

Guinea	2032	63 (50 to 81)	11 (8 to 16)	36 (25 to 48)	8 (4 to 15)	8 (7 to 10)	156 (122 to 199)	28 (19 to 38)	88 (62 to 118)	20 (9 to 37)	21 (18 to 25)
Guinea	2033	65 (51 to 82)	12 (8 to 16)	37 (26 to 49)	8 (4 to 15)	8 (7 to 10)	160 (125 to 202)	29 (19 to 40)	90 (64 to 121)	21 (10 to 38)	21 (18 to 25)
Guinea	2034	67 (52 to 86)	12 (8 to 17)	38 (27 to 51)	9 (4 to 16)	8 (7 to 10)	164 (128 to 211)	30 (20 to 42)	92 (65 to 124)	21 (10 to 39)	21 (17 to 25)
Guinea	2035	68 (53 to 87)	12 (8 to 18)	38 (27 to 52)	9 (4 to 16)	8 (7 to 10)	168 (131 to 214)	31 (20 to 43)	95 (67 to 127)	22 (10 to 41)	21 (17 to 25)
Guinea	2036	70 (54 to 90)	13 (8 to 18)	39 (28 to 53)	9 (4 to 17)	8 (7 to 11)	172 (133 to 222)	32 (21 to 45)	97 (69 to 131)	23 (11 to 42)	21 (17 to 26)
Guinea	2037	72 (56 to 91)	13 (9 to 19)	40 (29 to 54)	10 (5 to 18)	8 (7 to 10)	177 (137 to 225)	33 (21 to 47)	99 (70 to 134)	24 (11 to 43)	21 (17 to 25)
Guinea	2038	74 (56 to 95)	14 (9 to 20)	41 (29 to 56)	10 (5 to 18)	8 (7 to 11)	181 (139 to 234)	34 (22 to 49)	102 (72 to 137)	24 (11 to 45)	21 (17 to 26)
Guinea	2039	75 (58 to 96)	14 (9 to 20)	42 (30 to 57)	10 (5 to 19)	8 (7 to 11)	185 (144 to 237)	35 (22 to 50)	104 (74 to 140)	25 (12 to 46)	21 (17 to 26)
Guinea	2040	77 (59 to 100)	15 (9 to 22)	43 (31 to 59)	11 (5 to 19)	8 (7 to 11)	190 (144 to 247)	37 (23 to 53)	107 (75 to 144)	26 (12 to 48)	21 (17 to 26)
Guinea	2041	79 (61 to 102)	15 (10 to 22)	44 (31 to 60)	11 (5 to 20)	8 (7 to 11)	195 (149 to 250)	38 (24 to 55)	109 (77 to 148)	27 (13 to 49)	21 (17 to 26)
Guinea	2042	81 (62 to 105)	16 (10 to 23)	45 (32 to 61)	11 (5 to 21)	8 (7 to 11)	199 (152 to 259)	39 (24 to 57)	112 (79 to 151)	28 (13 to 51)	21 (17 to 26)
Guinea	2043	83 (63 to 107)	16 (10 to 24)	47 (33 to 63)	12 (5 to 21)	8 (7 to 11)	204 (156 to 263)	40 (25 to 59)	114 (81 to 155)	29 (13 to 53)	21 (17 to 27)
Guinea	2044	85 (65 to 111)	17 (11 to 25)	48 (34 to 65)	12 (6 to 22)	8 (7 to 11)	209 (159 to 273)	42 (26 to 62)	117 (83 to 159)	30 (14 to 54)	21 (17 to 27)
Guinea	2045	87 (66 to 113)	17 (11 to 26)	49 (34 to 66)	12 (6 to 23)	8 (7 to 11)	214 (163 to 278)	43 (27 to 64)	120 (85 to 162)	30 (14 to 56)	21 (17 to 27)
Guinea	2046	89 (67 to 116)	18 (11 to 27)	50 (35 to 68)	13 (6 to 24)	8 (7 to 11)	219 (166 to 287)	44 (27 to 66)	123 (87 to 167)	31 (15 to 58)	21 (17 to 27)

Guinea	2047	91 (69 to 119)	19 (11 to 28)	51 (36 to 69)	13 (6 to 24)	8 (7 to 11)	225 (170 to 293)	46 (28 to 69)	126 (88 to 171)	32 (15 to 60)	21 (17 to 28)
Guinea	2048	94 (70 to 122)	19 (11 to 29)	52 (37 to 71)	14 (6 to 25)	8 (7 to 11)	230 (173 to 301)	47 (28 to 72)	128 (90 to 175)	33 (15 to 62)	21 (17 to 28)
Guinea	2049	96 (73 to 125)	20 (12 to 31)	53 (38 to 73)	14 (6 to 26)	9 (7 to 11)	236 (178 to 307)	49 (29 to 75)	131 (92 to 179)	34 (16 to 64)	21 (17 to 28)
Guinea	2050	98 (73 to 130)	20 (12 to 31)	55 (38 to 75)	14 (7 to 27)	9 (7 to 12)	241 (181 to 320)	50 (29 to 77)	134 (94 to 184)	35 (16 to 66)	21 (17 to 28)
Guinea-Bissau	1995	56 (49 to 64)	8 (6 to 11)	22 (16 to 30)	0 (0 to 1)	26 (26 to 26)	133 (117 to 153)	19 (14 to 25)	52 (37 to 71)	1 (0 to 1)	61 (61 to 61)
Guinea-Bissau	1996	35 (28 to 43)	8 (6 to 11)	22 (15 to 29)	0 (0 to 1)	4 (4 to 4)	82 (66 to 101)	20 (15 to 26)	51 (37 to 70)	1 (0 to 1)	10 (10 to 10)
Guinea-Bissau	1997	38 (31 to 47)	11 (8 to 14)	22 (15 to 29)	0 (0 to 1)	6 (6 to 6)	90 (73 to 111)	25 (19 to 33)	51 (37 to 70)	1 (0 to 1)	13 (13 to 13)
Guinea-Bissau	1998	32 (26 to 39)	9 (6 to 11)	17 (12 to 23)	0 (0 to 0)	6 (6 to 6)	76 (63 to 92)	21 (15 to 27)	40 (28 to 55)	0 (0 to 1)	15 (15 to 15)
Guinea-Bissau	1999	29 (23 to 36)	11 (9 to 15)	17 (12 to 23)	0 (0 to 0)	1 (1 to 1)	69 (55 to 86)	27 (20 to 36)	39 (28 to 55)	0 (0 to 0)	2 (2 to 2)
Guinea-Bissau	2000	39 (32 to 47)	16 (12 to 21)	18 (13 to 25)	0 (0 to 0)	4 (4 to 4)	92 (75 to 112)	39 (30 to 51)	43 (30 to 60)	0 (0 to 0)	10 (10 to 10)
Guinea-Bissau	2001	39 (33 to 47)	16 (12 to 21)	17 (12 to 23)	0 (0 to 0)	7 (7 to 7)	94 (78 to 113)	37 (28 to 50)	40 (28 to 54)	0 (0 to 0)	17 (17 to 17)
Guinea-Bissau	2002	34 (28 to 41)	13 (10 to 17)	15 (11 to 20)	0 (0 to 0)	6 (6 to 6)	80 (67 to 97)	31 (23 to 41)	35 (25 to 47)	0 (0 to 0)	14 (14 to 14)
Guinea-Bissau	2003	37 (31 to 45)	15 (12 to 20)	15 (11 to 21)	0 (0 to 0)	6 (6 to 6)	87 (73 to 106)	37 (28 to 48)	36 (26 to 49)	0 (0 to 0)	15 (15 to 15)
Guinea-Bissau	2004	44 (37 to 52)	19 (14 to 24)	17 (12 to 23)	0 (0 to 0)	8 (8 to 8)	104 (88 to 124)	44 (33 to 58)	40 (29 to 54)	0 (0 to 0)	20 (20 to 20)
Guinea-Bissau	2005	43 (36 to 51)	17 (13 to 22)	16 (12 to 22)	0 (0 to 0)	9 (9 to 9)	101 (86 to 120)	40 (30 to 52)	39 (29 to 52)	0 (0 to 0)	23 (23 to 23)

Guinea-Bissau	2006	43 (37 to 50)	15 (12 to 20)	17 (12 to 22)	0 (0 to 0)	11 (11 to 11)	102 (87 to 120)	36 (27 to 47)	40 (29 to 53)	0 (0 to 0)	26 (26 to 26)
Guinea-Bissau	2007	49 (43 to 57)	16 (12 to 21)	18 (13 to 25)	0 (0 to 1)	15 (15 to 15)	118 (102 to 135)	38 (29 to 49)	43 (31 to 58)	1 (0 to 1)	36 (36 to 36)
Guinea-Bissau	2008	43 (37 to 51)	14 (10 to 18)	19 (13 to 25)	1 (0 to 1)	10 (10 to 10)	103 (87 to 120)	33 (25 to 42)	44 (32 to 59)	2 (1 to 3)	24 (24 to 24)
Guinea-Bissau	2009	43 (37 to 50)	11 (9 to 15)	19 (14 to 25)	1 (1 to 2)	11 (11 to 11)	101 (87 to 119)	27 (20 to 35)	45 (33 to 59)	3 (1 to 5)	27 (27 to 27)
Guinea-Bissau	2010	51 (46 to 58)	8 (6 to 10)	19 (14 to 25)	1 (1 to 3)	23 (23 to 23)	122 (108 to 138)	19 (14 to 25)	45 (33 to 60)	3 (2 to 6)	54 (54 to 54)
Guinea-Bissau	2011	38 (33 to 45)	6 (4 to 8)	20 (14 to 27)	2 (1 to 3)	11 (11 to 11)	91 (77 to 108)	14 (10 to 18)	47 (34 to 63)	4 (2 to 7)	26 (26 to 26)
Guinea-Bissau	2012	40 (34 to 47)	4 (3 to 5)	21 (16 to 29)	1 (1 to 3)	13 (13 to 13)	95 (80 to 112)	10 (7 to 13)	51 (37 to 68)	3 (2 to 6)	31 (31 to 31)
Guinea-Bissau	2013	55 (48 to 64)	4 (3 to 5)	25 (18 to 34)	1 (1 to 2)	25 (25 to 25)	131 (114 to 152)	9 (7 to 11)	60 (43 to 80)	3 (2 to 6)	60 (60 to 60)
Guinea-Bissau	2014	52 (43 to 64)	5 (4 to 7)	34 (24 to 46)	2 (1 to 3)	12 (12 to 12)	124 (102 to 152)	12 (9 to 16)	80 (57 to 108)	5 (2 to 8)	28 (28 to 28)
Guinea-Bissau	2015	59 (49 to 71)	5 (4 to 6)	36 (25 to 48)	2 (1 to 3)	17 (17 to 17)	141 (116 to 169)	11 (8 to 15)	85 (60 to 114)	4 (2 to 8)	40 (40 to 40)
Guinea-Bissau	2016	60 (49 to 72)	4 (3 to 6)	36 (26 to 50)	2 (1 to 3)	17 (17 to 17)	141 (116 to 172)	11 (8 to 14)	86 (61 to 118)	4 (2 to 8)	40 (40 to 40)
Guinea-Bissau	2017	60 (49 to 73)	4 (3 to 6)	36 (26 to 49)	2 (1 to 3)	17 (17 to 17)	142 (116 to 172)	10 (8 to 13)	87 (61 to 117)	4 (2 to 8)	40 (40 to 40)
Guinea-Bissau	2018	59 (48 to 73)	5 (3 to 6)	37 (26 to 50)	2 (1 to 3)	16 (16 to 16)	141 (115 to 174)	11 (8 to 15)	87 (62 to 118)	5 (2 to 8)	38 (38 to 38)
Guinea-Bissau	2019	61 (50 to 75)	6 (4 to 7)	38 (27 to 51)	2 (1 to 4)	16 (16 to 16)	145 (119 to 178)	13 (10 to 17)	89 (63 to 121)	5 (2 to 9)	38 (38 to 39)
Guinea-Bissau	2020	62 (50 to 77)	6 (4 to 8)	38 (27 to 52)	2 (1 to 4)	16 (15 to 17)	148 (120 to 182)	14 (10 to 18)	91 (64 to 124)	5 (2 to 9)	38 (37 to 41)

Guinea-Bissau	2021	63 (51 to 77)	6 (4 to 8)	39 (28 to 53)	2 (1 to 4)	16 (15 to 18)	150 (122 to 182)	14 (10 to 19)	93 (65 to 126)	5 (2 to 9)	39 (36 to 42)
Guinea-Bissau	2022	64 (52 to 79)	6 (4 to 8)	39 (28 to 53)	2 (1 to 4)	16 (15 to 18)	152 (123 to 187)	14 (10 to 19)	94 (66 to 127)	5 (2 to 9)	39 (35 to 43)
Guinea-Bissau	2023	65 (52 to 79)	6 (4 to 8)	40 (28 to 54)	2 (1 to 4)	16 (15 to 18)	153 (124 to 188)	15 (11 to 20)	95 (67 to 129)	5 (2 to 9)	38 (35 to 43)
Guinea-Bissau	2024	65 (52 to 80)	6 (4 to 9)	41 (29 to 55)	2 (1 to 4)	16 (14 to 18)	155 (124 to 191)	15 (11 to 21)	96 (68 to 131)	5 (3 to 10)	38 (34 to 43)
Guinea-Bissau	2025	66 (53 to 80)	6 (5 to 9)	41 (29 to 56)	2 (1 to 4)	16 (14 to 18)	157 (126 to 191)	15 (11 to 21)	98 (69 to 133)	5 (3 to 10)	38 (34 to 43)
Guinea-Bissau	2026	67 (53 to 83)	7 (5 to 9)	42 (29 to 57)	2 (1 to 4)	16 (14 to 18)	158 (126 to 197)	16 (11 to 21)	99 (70 to 135)	5 (3 to 10)	38 (33 to 44)
Guinea-Bissau	2027	67 (54 to 82)	7 (5 to 10)	42 (30 to 58)	2 (1 to 4)	16 (14 to 19)	160 (128 to 196)	16 (11 to 23)	101 (71 to 137)	6 (3 to 10)	38 (33 to 44)
Guinea-Bissau	2028	68 (54 to 86)	7 (5 to 10)	43 (30 to 59)	2 (1 to 4)	16 (14 to 19)	163 (128 to 203)	16 (11 to 23)	103 (72 to 139)	6 (3 to 11)	38 (33 to 44)
Guinea-Bissau	2029	69 (56 to 85)	7 (5 to 10)	44 (31 to 60)	2 (1 to 5)	16 (14 to 19)	165 (132 to 202)	17 (12 to 24)	105 (74 to 142)	6 (3 to 11)	38 (33 to 44)
Guinea-Bissau	2030	71 (56 to 89)	7 (5 to 11)	45 (32 to 61)	3 (1 to 5)	16 (14 to 19)	168 (132 to 211)	18 (12 to 25)	106 (75 to 145)	6 (3 to 11)	38 (32 to 45)
Guinea-Bissau	2031	72 (58 to 89)	8 (5 to 11)	46 (32 to 62)	3 (1 to 5)	16 (14 to 19)	171 (137 to 210)	18 (12 to 26)	108 (77 to 147)	6 (3 to 11)	38 (32 to 45)
Guinea-Bissau	2032	73 (57 to 91)	8 (5 to 11)	46 (33 to 63)	3 (1 to 5)	16 (13 to 19)	173 (136 to 217)	19 (12 to 27)	110 (78 to 149)	6 (3 to 12)	38 (32 to 46)
Guinea-Bissau	2033	74 (59 to 91)	8 (5 to 12)	47 (33 to 64)	3 (1 to 5)	16 (13 to 19)	176 (141 to 217)	19 (12 to 28)	112 (79 to 152)	7 (3 to 12)	38 (32 to 46)
Guinea-Bissau	2034	75 (59 to 95)	8 (5 to 12)	48 (34 to 65)	3 (1 to 5)	16 (13 to 19)	179 (141 to 225)	20 (13 to 29)	115 (81 to 155)	7 (3 to 12)	38 (32 to 46)
Guinea-Bissau	2035	77 (61 to 95)	9 (5 to 13)	49 (35 to 67)	3 (1 to 5)	16 (13 to 19)	182 (144 to 225)	21 (13 to 31)	117 (83 to 158)	7 (3 to 13)	38 (32 to 46)

Guinea-Bissau	2036	78 (61 to 98)	9 (6 to 14)	50 (35 to 68)	3 (1 to 5)	16 (13 to 20)	185 (145 to 233)	21 (13 to 33)	119 (84 to 161)	7 (3 to 13)	38 (32 to 48)
Guinea-Bissau	2037	79 (63 to 98)	9 (6 to 14)	51 (36 to 69)	3 (1 to 6)	16 (13 to 20)	189 (150 to 233)	22 (14 to 33)	121 (86 to 164)	7 (4 to 13)	38 (31 to 47)
Guinea-Bissau	2038	81 (63 to 102)	10 (6 to 15)	52 (37 to 71)	3 (2 to 6)	16 (13 to 20)	192 (149 to 242)	23 (14 to 34)	124 (87 to 168)	8 (4 to 14)	38 (31 to 48)
Guinea-Bissau	2039	82 (65 to 101)	10 (6 to 15)	53 (37 to 72)	3 (2 to 6)	16 (13 to 20)	196 (154 to 241)	24 (15 to 37)	126 (89 to 171)	8 (4 to 14)	38 (31 to 49)
Guinea-Bissau	2040	84 (64 to 105)	10 (6 to 16)	54 (38 to 73)	3 (2 to 6)	16 (13 to 21)	199 (153 to 250)	25 (15 to 37)	128 (90 to 174)	8 (4 to 14)	39 (31 to 49)
Guinea-Bissau	2041	85 (67 to 106)	11 (7 to 16)	55 (39 to 75)	3 (2 to 6)	16 (13 to 21)	203 (159 to 251)	25 (15 to 39)	130 (92 to 177)	8 (4 to 15)	39 (31 to 49)
Guinea-Bissau	2042	87 (67 to 109)	11 (7 to 17)	56 (39 to 76)	4 (2 to 6)	16 (13 to 21)	206 (159 to 260)	26 (16 to 41)	133 (94 to 181)	8 (4 to 15)	39 (31 to 50)
Guinea-Bissau	2043	88 (70 to 109)	12 (7 to 18)	57 (40 to 77)	4 (2 to 7)	16 (13 to 22)	210 (165 to 259)	28 (16 to 43)	135 (95 to 184)	9 (4 to 16)	39 (31 to 51)
Guinea-Bissau	2044	90 (69 to 113)	12 (7 to 19)	58 (41 to 79)	4 (2 to 7)	16 (13 to 22)	214 (165 to 268)	29 (17 to 45)	137 (97 to 187)	9 (4 to 16)	39 (32 to 52)
Guinea-Bissau	2045	91 (71 to 113)	12 (7 to 20)	59 (41 to 80)	4 (2 to 7)	17 (13 to 21)	217 (168 to 269)	30 (18 to 47)	139 (98 to 189)	9 (4 to 16)	39 (31 to 51)
Guinea-Bissau	2046	93 (72 to 116)	13 (8 to 21)	59 (42 to 81)	4 (2 to 7)	17 (13 to 22)	221 (171 to 277)	31 (18 to 49)	141 (100 to 192)	9 (4 to 17)	40 (31 to 52)
Guinea-Bissau	2047	95 (74 to 117)	13 (8 to 22)	60 (43 to 82)	4 (2 to 7)	17 (13 to 22)	225 (176 to 279)	32 (19 to 51)	143 (101 to 195)	9 (4 to 17)	40 (31 to 53)
Guinea-Bissau	2048	96 (74 to 120)	14 (8 to 22)	61 (43 to 83)	4 (2 to 7)	17 (13 to 23)	229 (176 to 286)	33 (19 to 53)	146 (103 to 198)	10 (5 to 18)	40 (32 to 54)
Guinea-Bissau	2049	98 (76 to 121)	15 (8 to 23)	62 (44 to 84)	4 (2 to 8)	17 (13 to 23)	233 (180 to 288)	35 (20 to 55)	148 (104 to 201)	10 (5 to 18)	41 (32 to 54)
Guinea-Bissau	2050	100 (76 to 125)	15 (9 to 25)	63 (44 to 86)	4 (2 to 8)	17 (13 to 23)	237 (181 to 298)	36 (20 to 59)	150 (105 to 203)	10 (5 to 18)	41 (32 to 56)

Guyana	1995	126 (108 to 147)	69 (56 to 85)	44 (33 to 58)	5 (3 to 10)	7 (7 to 7)	222 (191 to 259)	122 (99 to 149)	78 (59 to 102)	10 (4 to 17)	12 (12 to 12)
Guyana	1996	130 (113 to 150)	72 (59 to 87)	46 (35 to 59)	6 (3 to 10)	7 (7 to 7)	229 (199 to 265)	127 (105 to 153)	81 (61 to 105)	10 (5 to 18)	12 (12 to 12)
Guyana	1997	136 (119 to 156)	74 (62 to 89)	47 (36 to 60)	6 (3 to 11)	9 (9 to 9)	241 (210 to 275)	131 (109 to 157)	84 (64 to 106)	10 (5 to 19)	16 (16 to 16)
Guyana	1998	130 (114 to 147)	70 (59 to 84)	47 (36 to 60)	5 (3 to 10)	6 (6 to 6)	229 (200 to 259)	124 (103 to 148)	84 (64 to 106)	10 (5 to 18)	11 (11 to 11)
Guyana	1999	124 (109 to 140)	66 (56 to 79)	47 (37 to 59)	5 (2 to 10)	6 (6 to 6)	219 (192 to 248)	117 (98 to 139)	83 (65 to 103)	9 (4 to 17)	10 (10 to 10)
Guyana	2000	127 (111 to 144)	69 (58 to 82)	50 (39 to 62)	5 (2 to 10)	3 (3 to 3)	224 (196 to 254)	122 (102 to 145)	87 (69 to 110)	9 (4 to 17)	5 (5 to 5)
Guyana	2001	131 (115 to 148)	70 (58 to 82)	51 (40 to 63)	5 (2 to 9)	6 (6 to 6)	232 (204 to 262)	123 (103 to 145)	90 (71 to 112)	9 (4 to 16)	10 (10 to 10)
Guyana	2002	128 (113 to 145)	68 (57 to 80)	51 (40 to 63)	5 (2 to 9)	4 (4 to 4)	226 (199 to 256)	120 (101 to 141)	90 (71 to 112)	8 (4 to 16)	8 (8 to 8)
Guyana	2003	144 (128 to 161)	67 (57 to 79)	52 (41 to 65)	5 (2 to 9)	20 (20 to 20)	254 (226 to 284)	119 (100 to 139)	91 (73 to 114)	8 (4 to 15)	35 (35 to 35)
Guyana	2004	164 (148 to 180)	65 (55 to 77)	54 (43 to 66)	5 (2 to 9)	41 (41 to 41)	290 (261 to 318)	115 (97 to 136)	95 (75 to 116)	8 (4 to 15)	72 (72 to 72)
Guyana	2005	164 (148 to 181)	67 (56 to 79)	58 (46 to 70)	5 (3 to 9)	34 (34 to 34)	290 (261 to 319)	118 (99 to 140)	102 (81 to 124)	9 (5 to 17)	60 (60 to 60)
Guyana	2006	180 (164 to 197)	65 (55 to 76)	62 (49 to 75)	6 (3 to 10)	47 (47 to 47)	317 (289 to 347)	115 (96 to 135)	109 (87 to 133)	10 (5 to 18)	83 (83 to 83)
Guyana	2007	174 (159 to 191)	61 (51 to 71)	65 (52 to 78)	6 (3 to 10)	43 (43 to 43)	308 (280 to 337)	107 (90 to 126)	114 (92 to 138)	10 (5 to 18)	76 (76 to 76)
Guyana	2008	188 (172 to 206)	59 (50 to 70)	68 (55 to 83)	6 (3 to 11)	54 (54 to 54)	332 (303 to 363)	105 (88 to 124)	120 (97 to 147)	11 (5 to 19)	96 (96 to 96)
Guyana	2009	192 (175 to 212)	64 (55 to 76)	73 (59 to 88)	6 (3 to 11)	48 (48 to 48)	339 (308 to 373)	114 (97 to 135)	128 (104 to 156)	11 (6 to 20)	85 (85 to 85)

Guyana	2010	193 (176 to 215)	69 (58 to 81)	75 (62 to 92)	7 (3 to 12)	43 (43 to 43)	341 (310 to 379)	121 (102 to 143)	133 (109 to 162)	12 (6 to 21)	75 (75 to 75)
Guyana	2011	199 (180 to 222)	78 (67 to 91)	80 (65 to 96)	7 (4 to 12)	34 (34 to 34)	351 (317 to 391)	138 (118 to 161)	140 (115 to 170)	13 (6 to 22)	60 (60 to 60)
Guyana	2012	206 (186 to 229)	90 (78 to 105)	83 (68 to 101)	8 (4 to 13)	26 (26 to 26)	364 (328 to 404)	159 (137 to 186)	147 (120 to 179)	13 (6 to 24)	45 (45 to 45)
Guyana	2013	216 (193 to 240)	102 (88 to 119)	86 (70 to 104)	8 (4 to 14)	20 (20 to 20)	381 (340 to 424)	181 (155 to 211)	151 (123 to 184)	14 (7 to 24)	35 (35 to 35)
Guyana	2014	230 (206 to 257)	118 (101 to 136)	88 (71 to 107)	8 (4 to 14)	16 (16 to 16)	406 (364 to 453)	208 (179 to 240)	155 (126 to 189)	14 (7 to 25)	28 (28 to 28)
Guyana	2015	226 (202 to 253)	122 (105 to 141)	85 (69 to 104)	8 (4 to 14)	11 (11 to 11)	399 (356 to 447)	215 (186 to 249)	150 (121 to 184)	14 (7 to 25)	20 (20 to 20)
Guyana	2016	248 (221 to 279)	144 (123 to 168)	85 (68 to 106)	8 (4 to 15)	10 (10 to 10)	437 (390 to 493)	254 (217 to 297)	151 (120 to 187)	14 (7 to 26)	18 (18 to 18)
Guyana	2017	258 (226 to 299)	154 (129 to 181)	86 (66 to 110)	8 (4 to 15)	11 (11 to 11)	456 (399 to 528)	271 (228 to 319)	151 (117 to 194)	14 (7 to 27)	19 (19 to 19)
Guyana	2018	263 (224 to 307)	156 (129 to 185)	88 (68 to 113)	8 (4 to 16)	10 (10 to 10)	464 (395 to 542)	275 (228 to 327)	156 (120 to 200)	15 (7 to 27)	18 (18 to 18)
Guyana	2019	287 (248 to 337)	176 (144 to 214)	92 (71 to 118)	9 (4 to 17)	10 (10 to 10)	507 (438 to 595)	310 (254 to 378)	163 (126 to 209)	16 (8 to 30)	18 (18 to 18)
Guyana	2020	503 (419 to 597)	315 (256 to 387)	162 (125 to 208)	16 (8 to 30)	9 (8 to 10)	887 (739 to 1054)	557 (451 to 683)	286 (221 to 367)	29 (14 to 53)	15 (14 to 17)
Guyana	2021	517 (438 to 617)	327 (262 to 408)	164 (127 to 210)	17 (8 to 31)	9 (8 to 10)	912 (774 to 1089)	577 (462 to 720)	289 (224 to 371)	30 (14 to 55)	16 (14 to 18)
Guyana	2022	530 (433 to 640)	338 (262 to 426)	166 (128 to 212)	17 (8 to 31)	9 (8 to 10)	935 (765 to 1129)	596 (463 to 751)	292 (227 to 375)	30 (15 to 56)	16 (14 to 18)
Guyana	2023	543 (451 to 660)	349 (270 to 450)	167 (129 to 214)	17 (8 to 32)	9 (8 to 11)	959 (797 to 1165)	617 (476 to 794)	295 (229 to 378)	31 (15 to 56)	16 (14 to 19)
Guyana	2024	555 (450 to 679)	359 (274 to 464)	169 (130 to 215)	18 (8 to 32)	9 (8 to 11)	980 (794 to 1199)	634 (483 to 818)	298 (230 to 380)	31 (15 to 57)	17 (14 to 19)

Guyana	2025	566 (461 to 696)	369 (277 to 487)	170 (132 to 217)	18 (9 to 33)	10 (8 to 11)	999 (814 to 1229)	651 (489 to 859)	300 (232 to 383)	32 (15 to 58)	17 (14 to 20)
Guyana	2026	577 (463 to 712)	378 (284 to 492)	170 (132 to 217)	18 (9 to 33)	10 (8 to 11)	1018 (816 to 1256)	668 (501 to 868)	301 (233 to 384)	32 (15 to 58)	17 (14 to 20)
Guyana	2027	587 (477 to 729)	388 (291 to 513)	171 (133 to 218)	18 (9 to 34)	10 (8 to 12)	1037 (841 to 1287)	685 (514 to 905)	302 (234 to 385)	32 (16 to 59)	17 (15 to 21)
Guyana	2028	599 (472 to 753)	399 (294 to 531)	172 (133 to 219)	19 (9 to 34)	10 (8 to 12)	1058 (833 to 1329)	704 (519 to 938)	303 (235 to 387)	33 (16 to 60)	18 (15 to 22)
Guyana	2029	610 (488 to 775)	408 (299 to 552)	173 (134 to 220)	19 (9 to 35)	10 (9 to 12)	1076 (862 to 1369)	720 (527 to 975)	305 (236 to 388)	33 (16 to 61)	18 (15 to 22)
Guyana	2030	621 (485 to 793)	418 (300 to 570)	173 (134 to 221)	19 (9 to 35)	10 (9 to 13)	1097 (855 to 1400)	739 (530 to 1006)	306 (236 to 390)	34 (16 to 62)	19 (15 to 22)
Guyana	2031	633 (499 to 813)	429 (307 to 588)	174 (135 to 222)	19 (9 to 36)	11 (9 to 13)	1117 (881 to 1435)	757 (542 to 1038)	307 (238 to 392)	34 (16 to 63)	19 (16 to 23)
Guyana	2032	647 (494 to 842)	441 (311 to 613)	175 (136 to 223)	20 (9 to 36)	11 (9 to 14)	1141 (871 to 1486)	779 (549 to 1082)	309 (239 to 394)	35 (17 to 64)	19 (16 to 24)
Guyana	2033	660 (515 to 848)	453 (320 to 626)	176 (136 to 224)	20 (10 to 37)	11 (9 to 14)	1166 (909 to 1497)	800 (565 to 1104)	311 (241 to 396)	35 (17 to 65)	20 (16 to 24)
Guyana	2034	676 (516 to 876)	467 (326 to 646)	177 (137 to 226)	20 (10 to 37)	11 (9 to 14)	1192 (910 to 1546)	825 (576 to 1141)	312 (242 to 399)	36 (17 to 66)	20 (16 to 25)

Guyana	2035	690 (531 to 900)	480 (333 to 670)	178 (138 to 227)	20 (10 to 38)	12 (9 to 14)	1218 (937 to 1589)	847 (587 to 1182)	314 (244 to 401)	36 (17 to 66)	21 (17 to 25)
Guyana	2036	705 (530 to 937)	493 (345 to 700)	179 (139 to 229)	21 (10 to 38)	12 (10 to 15)	1244 (936 to 1654)	870 (609 to 1236)	316 (246 to 404)	37 (18 to 67)	21 (17 to 27)
Guyana	2037	721 (557 to 955)	507 (356 to 726)	181 (140 to 230)	21 (10 to 39)	12 (10 to 15)	1273 (983 to 1686)	895 (629 to 1281)	319 (247 to 407)	37 (18 to 68)	21 (17 to 27)
Guyana	2038	736 (551 to 982)	521 (362 to 740)	182 (141 to 232)	21 (10 to 39)	12 (10 to 16)	1300 (973 to 1734)	919 (638 to 1307)	321 (249 to 410)	38 (18 to 69)	22 (18 to 28)
Guyana	2039	753 (572 to 1000)	535 (369 to 766)	183 (142 to 234)	22 (10 to 40)	13 (10 to 16)	1329 (1009 to 1765)	945 (652 to 1352)	323 (251 to 413)	38 (19 to 70)	22 (18 to 29)
Guyana	2040	770 (570 to 1032)	550 (374 to 789)	184 (143 to 235)	22 (11 to 40)	13 (10 to 17)	1359 (1006 to 1822)	971 (659 to 1392)	326 (252 to 416)	39 (19 to 71)	23 (18 to 29)
Guyana	2041	786 (593 to 1052)	565 (382 to 817)	186 (144 to 237)	22 (11 to 41)	13 (11 to 17)	1388 (1046 to 1857)	997 (674 to 1442)	328 (254 to 418)	40 (19 to 73)	23 (19 to 30)
Guyana	2042	803 (586 to 1083)	580 (389 to 840)	187 (145 to 239)	23 (11 to 42)	13 (11 to 18)	1417 (1034 to 1911)	1023 (687 to 1483)	330 (255 to 421)	40 (19 to 74)	24 (19 to 31)
Guyana	2043	820 (609 to 1101)	595 (394 to 858)	188 (145 to 240)	23 (11 to 42)	14 (11 to 18)	1447 (1076 to 1943)	1049 (695 to 1515)	333 (257 to 424)	41 (20 to 75)	24 (19 to 32)
Guyana	2044	837 (601 to 1142)	610 (400 to 888)	190 (147 to 242)	23 (11 to 43)	14 (11 to 19)	1477 (1062 to 2016)	1077 (707 to 1567)	335 (259 to 427)	41 (20 to 76)	25 (19 to 33)

Guyana	2045	854 (628 to 1161)	625 (413 to 913)	191 (147 to 244)	24 (12 to 44)	14 (11 to 19)	1507 (1108 to 2049)	1102 (729 to 1612)	337 (260 to 430)	42 (20 to 77)	25 (20 to 33)
Guyana	2046	871 (617 to 1189)	640 (417 to 934)	192 (149 to 245)	24 (12 to 44)	15 (11 to 19)	1538 (1089 to 2098)	1130 (736 to 1649)	339 (262 to 433)	43 (21 to 78)	26 (20 to 34)
Guyana	2047	889 (644 to 1226)	656 (422 to 980)	194 (150 to 247)	25 (12 to 45)	15 (11 to 20)	1569 (1137 to 2164)	1158 (745 to 1731)	342 (265 to 436)	43 (21 to 80)	26 (20 to 36)
Guyana	2048	907 (632 to 1255)	672 (427 to 997)	195 (151 to 249)	25 (12 to 46)	15 (12 to 21)	1602 (1116 to 2215)	1186 (753 to 1760)	344 (267 to 439)	44 (21 to 81)	27 (21 to 36)
Guyana	2049	923 (655 to 1289)	685 (427 to 1026)	197 (153 to 251)	25 (12 to 47)	16 (12 to 21)	1629 (1157 to 2274)	1209 (754 to 1811)	347 (270 to 443)	45 (22 to 82)	27 (21 to 37)
Guyana	2050	939 (646 to 1315)	699 (431 to 1062)	198 (154 to 253)	26 (12 to 47)	16 (12 to 22)	1657 (1141 to 2320)	1234 (761 to 1874)	350 (272 to 446)	45 (22 to 84)	28 (21 to 38)
Haiti	1995	61 (53 to 72)	10 (7 to 13)	26 (18 to 36)	1 (1 to 3)	24 (24 to 24)	150 (129 to 175)	24 (17 to 31)	65 (45 to 89)	4 (2 to 7)	58 (58 to 58)
Haiti	1996	45 (37 to 56)	9 (7 to 12)	27 (19 to 37)	1 (1 to 3)	7 (7 to 7)	111 (91 to 137)	23 (17 to 30)	66 (46 to 91)	4 (2 to 7)	18 (18 to 18)
Haiti	1997	45 (36 to 55)	9 (7 to 12)	26 (19 to 37)	1 (1 to 3)	8 (8 to 8)	109 (89 to 134)	23 (17 to 30)	65 (46 to 90)	3 (2 to 6)	19 (19 to 19)
Haiti	1998	48 (40 to 58)	9 (7 to 11)	25 (18 to 35)	1 (1 to 2)	13 (13 to 13)	117 (99 to 141)	22 (16 to 28)	62 (45 to 85)	3 (2 to 6)	31 (31 to 31)
Haiti	1999	47 (39 to 57)	10 (7 to 12)	26 (19 to 36)	1 (1 to 3)	10 (10 to 10)	116 (96 to 140)	24 (18 to 30)	64 (46 to 87)	4 (2 to 7)	25 (25 to 25)
Haiti	2000	46 (38 to 55)	10 (8 to 13)	26 (19 to 35)	1 (1 to 3)	8 (8 to 8)	112 (92 to 136)	24 (19 to 31)	63 (47 to 86)	4 (2 to 7)	20 (20 to 20)

Haiti	2001	43 (35 to 52)	9 (7 to 12)	25 (18 to 33)	1 (1 to 3)	8 (8 to 8)	105 (86 to 127)	22 (17 to 29)	60 (44 to 81)	3 (2 to 6)	19 (19 to 19)
Haiti	2002	38 (30 to 46)	8 (6 to 10)	23 (17 to 31)	1 (1 to 3)	5 (5 to 5)	92 (74 to 113)	20 (15 to 26)	57 (41 to 76)	3 (2 to 6)	12 (12 to 12)
Haiti	2003	39 (33 to 48)	8 (6 to 10)	22 (16 to 30)	1 (1 to 3)	8 (8 to 8)	97 (80 to 118)	19 (14 to 24)	55 (39 to 74)	4 (2 to 6)	20 (20 to 20)
Haiti	2004	37 (31 to 45)	7 (5 to 9)	20 (15 to 28)	1 (1 to 3)	9 (9 to 9)	91 (76 to 110)	17 (13 to 22)	50 (36 to 67)	3 (1 to 6)	21 (21 to 21)
Haiti	2005	38 (32 to 45)	7 (5 to 9)	20 (14 to 26)	1 (1 to 3)	10 (10 to 10)	93 (79 to 110)	17 (13 to 22)	48 (34 to 64)	4 (2 to 7)	24 (24 to 24)
Haiti	2006	42 (37 to 49)	6 (5 to 8)	18 (13 to 24)	1 (1 to 3)	16 (16 to 16)	103 (89 to 119)	15 (12 to 20)	45 (32 to 60)	4 (2 to 6)	39 (39 to 39)
Haiti	2007	46 (40 to 53)	8 (6 to 10)	19 (14 to 25)	2 (1 to 3)	17 (17 to 17)	112 (98 to 129)	20 (15 to 25)	47 (34 to 62)	4 (2 to 7)	42 (42 to 42)
Haiti	2008	47 (42 to 54)	8 (6 to 11)	19 (13 to 25)	2 (1 to 3)	19 (19 to 19)	115 (102 to 132)	20 (15 to 26)	46 (33 to 62)	4 (2 to 7)	46 (46 to 46)
Haiti	2009	48 (42 to 55)	9 (7 to 11)	19 (14 to 26)	2 (1 to 3)	18 (18 to 18)	117 (102 to 135)	21 (16 to 28)	47 (34 to 64)	4 (2 to 8)	44 (44 to 44)
Haiti	2010	48 (42 to 56)	8 (6 to 10)	19 (14 to 26)	2 (1 to 3)	19 (19 to 19)	118 (104 to 137)	20 (15 to 25)	47 (34 to 64)	4 (2 to 8)	47 (47 to 47)
Haiti	2011	50 (44 to 57)	7 (5 to 9)	19 (14 to 26)	2 (1 to 3)	22 (22 to 22)	122 (108 to 140)	17 (13 to 23)	47 (34 to 65)	4 (2 to 8)	54 (54 to 54)
Haiti	2012	51 (45 to 59)	7 (5 to 9)	20 (15 to 28)	2 (1 to 4)	22 (22 to 22)	125 (110 to 144)	17 (13 to 23)	49 (36 to 68)	5 (2 to 10)	53 (53 to 53)
Haiti	2013	52 (45 to 60)	7 (5 to 9)	20 (15 to 28)	2 (1 to 4)	22 (22 to 22)	126 (111 to 146)	17 (13 to 23)	50 (36 to 69)	6 (3 to 10)	54 (54 to 54)
Haiti	2014	49 (43 to 57)	7 (5 to 9)	20 (14 to 28)	2 (1 to 4)	20 (20 to 20)	120 (105 to 140)	17 (12 to 22)	49 (35 to 69)	6 (3 to 11)	48 (48 to 48)
Haiti	2015	49 (43 to 58)	7 (5 to 9)	21 (15 to 29)	2 (1 to 5)	19 (19 to 19)	121 (104 to 143)	16 (12 to 22)	51 (36 to 72)	6 (3 to 11)	47 (47 to 47)

Haiti	2016	50 (43 to 59)	6 (5 to 9)	21 (15 to 30)	3 (1 to 5)	20 (20 to 20)	123 (107 to 145)	16 (12 to 21)	52 (37 to 72)	6 (3 to 12)	49 (49 to 49)
Haiti	2017	48 (40 to 57)	7 (5 to 9)	22 (15 to 31)	3 (1 to 5)	17 (17 to 17)	117 (99 to 139)	16 (12 to 21)	54 (38 to 75)	6 (3 to 12)	40 (40 to 40)
Haiti	2018	48 (41 to 57)	7 (5 to 10)	22 (16 to 31)	3 (1 to 5)	16 (16 to 16)	118 (100 to 139)	18 (13 to 24)	54 (38 to 76)	7 (3 to 13)	39 (39 to 39)
Haiti	2019	47 (39 to 56)	6 (5 to 8)	22 (15 to 31)	3 (1 to 5)	16 (16 to 16)	115 (96 to 137)	16 (12 to 21)	53 (37 to 75)	6 (3 to 12)	39 (39 to 39)
Haiti	2020	47 (40 to 56)	6 (5 to 8)	22 (15 to 31)	3 (1 to 5)	16 (16 to 17)	115 (98 to 137)	15 (11 to 21)	53 (38 to 75)	6 (3 to 12)	40 (38 to 42)
Haiti	2021	47 (40 to 57)	6 (5 to 8)	22 (15 to 31)	3 (1 to 5)	17 (16 to 18)	116 (98 to 138)	15 (11 to 20)	54 (38 to 75)	6 (3 to 12)	41 (38 to 44)
Haiti	2022	48 (40 to 56)	6 (5 to 8)	22 (15 to 31)	3 (1 to 5)	17 (15 to 19)	117 (99 to 138)	15 (11 to 20)	54 (38 to 75)	7 (3 to 12)	41 (38 to 46)
Haiti	2023	48 (40 to 57)	6 (4 to 8)	22 (15 to 31)	3 (1 to 5)	17 (15 to 19)	117 (99 to 140)	15 (11 to 20)	54 (38 to 75)	7 (3 to 13)	42 (38 to 47)
Haiti	2024	48 (40 to 57)	6 (4 to 8)	22 (15 to 31)	3 (1 to 5)	17 (15 to 19)	118 (99 to 141)	15 (11 to 20)	54 (38 to 76)	7 (3 to 13)	42 (38 to 48)
Haiti	2025	48 (41 to 58)	6 (4 to 8)	22 (16 to 31)	3 (1 to 5)	17 (15 to 20)	118 (99 to 141)	15 (11 to 19)	54 (38 to 76)	7 (3 to 13)	43 (38 to 48)
Haiti	2026	48 (41 to 58)	6 (4 to 8)	22 (16 to 31)	3 (1 to 5)	18 (16 to 20)	119 (100 to 142)	14 (11 to 19)	54 (38 to 76)	7 (3 to 13)	43 (38 to 49)
Haiti	2027	49 (41 to 58)	6 (4 to 8)	22 (16 to 31)	3 (1 to 5)	18 (16 to 21)	119 (100 to 142)	14 (11 to 19)	54 (38 to 76)	7 (3 to 13)	44 (38 to 51)
Haiti	2028	49 (41 to 58)	6 (4 to 8)	22 (16 to 31)	3 (1 to 5)	18 (16 to 21)	120 (101 to 143)	14 (10 to 19)	55 (38 to 77)	7 (3 to 13)	44 (39 to 52)
Haiti	2029	49 (41 to 59)	6 (4 to 8)	22 (16 to 32)	3 (1 to 5)	18 (16 to 21)	121 (101 to 144)	14 (10 to 19)	55 (39 to 77)	7 (3 to 13)	45 (39 to 53)
Haiti	2030	50 (42 to 59)	6 (4 to 8)	22 (16 to 32)	3 (1 to 6)	19 (16 to 22)	122 (102 to 145)	14 (10 to 19)	55 (39 to 77)	7 (3 to 14)	45 (39 to 54)

Haiti	2031	50 (42 to 60)	6 (4 to 8)	23 (16 to 32)	3 (1 to 6)	19 (16 to 22)	122 (102 to 147)	14 (10 to 19)	55 (39 to 78)	7 (3 to 14)	46 (40 to 55)
Haiti	2032	50 (42 to 61)	6 (4 to 8)	23 (16 to 32)	3 (1 to 6)	19 (16 to 23)	123 (104 to 148)	14 (10 to 18)	56 (39 to 78)	7 (3 to 14)	47 (40 to 57)
Haiti	2033	51 (43 to 61)	6 (4 to 8)	23 (16 to 32)	3 (1 to 6)	19 (17 to 24)	125 (105 to 149)	14 (10 to 18)	56 (39 to 78)	7 (3 to 14)	48 (41 to 58)
Haiti	2034	51 (43 to 62)	6 (4 to 8)	23 (16 to 32)	3 (1 to 6)	20 (16 to 24)	126 (106 to 151)	14 (10 to 19)	56 (39 to 79)	8 (3 to 14)	48 (40 to 59)
Haiti	2035	52 (43 to 63)	6 (4 to 8)	23 (16 to 32)	3 (1 to 6)	20 (17 to 24)	127 (106 to 153)	14 (10 to 19)	57 (40 to 79)	8 (3 to 15)	49 (41 to 60)
Haiti	2036	52 (44 to 63)	6 (4 to 8)	23 (16 to 32)	3 (1 to 6)	20 (17 to 25)	128 (107 to 153)	14 (10 to 19)	57 (40 to 80)	8 (4 to 15)	50 (41 to 62)
Haiti	2037	53 (44 to 64)	6 (4 to 8)	23 (16 to 33)	3 (1 to 6)	21 (17 to 25)	129 (108 to 156)	14 (10 to 19)	57 (40 to 80)	8 (4 to 15)	51 (42 to 62)
Haiti	2038	53 (44 to 64)	6 (4 to 8)	24 (16 to 33)	3 (1 to 6)	21 (17 to 26)	131 (108 to 157)	14 (10 to 19)	58 (40 to 81)	8 (4 to 15)	51 (42 to 65)
Haiti	2039	54 (45 to 65)	6 (4 to 8)	24 (16 to 33)	3 (1 to 6)	21 (18 to 27)	132 (110 to 160)	14 (10 to 19)	58 (40 to 81)	8 (4 to 15)	52 (43 to 66)
Haiti	2040	54 (45 to 66)	6 (4 to 8)	24 (17 to 33)	3 (2 to 6)	22 (18 to 27)	133 (111 to 163)	14 (10 to 19)	58 (41 to 82)	8 (4 to 16)	53 (43 to 67)
Haiti	2041	55 (45 to 67)	6 (4 to 8)	24 (17 to 33)	3 (2 to 6)	22 (18 to 28)	135 (111 to 165)	14 (10 to 19)	58 (41 to 82)	8 (4 to 16)	54 (44 to 68)
Haiti	2042	56 (46 to 68)	6 (4 to 8)	24 (17 to 34)	3 (2 to 7)	22 (18 to 28)	136 (113 to 168)	14 (10 to 20)	59 (41 to 82)	8 (4 to 16)	55 (45 to 70)
Haiti	2043	56 (46 to 69)	6 (4 to 8)	24 (17 to 34)	3 (2 to 7)	23 (18 to 30)	138 (114 to 170)	15 (10 to 20)	59 (41 to 83)	8 (4 to 16)	56 (45 to 72)
Haiti	2044	57 (47 to 69)	6 (4 to 8)	24 (17 to 34)	3 (2 to 7)	23 (19 to 31)	139 (115 to 168)	15 (10 to 20)	59 (41 to 83)	9 (4 to 16)	57 (46 to 75)
Haiti	2045	57 (47 to 69)	6 (4 to 8)	24 (17 to 34)	4 (2 to 7)	24 (19 to 30)	141 (116 to 170)	15 (10 to 20)	59 (41 to 83)	9 (4 to 16)	58 (46 to 74)

Haiti	2046	58 (48 to 71)	6 (4 to 8)	24 (17 to 34)	4 (2 to 7)	24 (19 to 31)	142 (118 to 173)	15 (10 to 21)	59 (41 to 83)	9 (4 to 16)	59 (47 to 75)
Haiti	2047	59 (48 to 71)	6 (4 to 9)	24 (17 to 34)	4 (2 to 7)	25 (20 to 32)	144 (119 to 174)	15 (10 to 21)	59 (41 to 84)	9 (4 to 17)	60 (48 to 79)
Haiti	2048	59 (49 to 72)	6 (4 to 9)	24 (17 to 34)	4 (2 to 7)	25 (20 to 33)	145 (119 to 175)	16 (10 to 22)	59 (41 to 84)	9 (4 to 17)	61 (49 to 80)
Haiti	2049	60 (49 to 73)	7 (4 to 9)	24 (17 to 34)	4 (2 to 7)	25 (20 to 34)	147 (121 to 178)	16 (10 to 22)	60 (41 to 84)	9 (4 to 17)	62 (50 to 83)
Haiti	2050	61 (51 to 74)	7 (4 to 9)	24 (17 to 34)	4 (2 to 7)	26 (21 to 35)	149 (124 to 181)	16 (10 to 22)	60 (41 to 84)	9 (4 to 17)	64 (51 to 85)
Honduras	1995	95 (81 to 112)	40 (31 to 50)	45 (34 to 58)	7 (3 to 12)	4 (4 to 4)	200 (169 to 235)	83 (65 to 105)	93 (71 to 121)	14 (7 to 26)	9 (9 to 9)
Honduras	1996	100 (87 to 117)	39 (31 to 49)	45 (36 to 58)	7 (4 to 13)	9 (9 to 9)	210 (182 to 246)	82 (64 to 102)	95 (74 to 122)	14 (7 to 26)	19 (19 to 19)
Honduras	1997	104 (91 to 121)	41 (33 to 51)	47 (37 to 60)	7 (4 to 13)	10 (10 to 10)	219 (190 to 254)	86 (68 to 106)	98 (78 to 125)	14 (7 to 27)	20 (20 to 20)
Honduras	1998	104 (89 to 120)	43 (34 to 53)	49 (39 to 61)	7 (3 to 13)	5 (5 to 5)	218 (187 to 252)	90 (72 to 110)	103 (82 to 129)	14 (7 to 27)	10 (10 to 10)
Honduras	1999	118 (102 to 135)	46 (37 to 56)	53 (42 to 65)	7 (3 to 13)	11 (11 to 11)	247 (213 to 282)	97 (77 to 117)	111 (89 to 137)	15 (7 to 26)	24 (24 to 24)
Honduras	2000	118 (102 to 135)	48 (38 to 59)	55 (44 to 68)	6 (3 to 12)	8 (8 to 8)	247 (213 to 282)	101 (80 to 123)	116 (93 to 143)	13 (6 to 24)	16 (16 to 16)
Honduras	2001	122 (106 to 140)	51 (41 to 62)	59 (48 to 73)	6 (3 to 11)	6 (6 to 6)	255 (222 to 292)	106 (85 to 129)	124 (101 to 153)	13 (6 to 23)	12 (12 to 12)
Honduras	2002	127 (110 to 145)	53 (43 to 65)	63 (52 to 78)	6 (3 to 12)	4 (4 to 4)	266 (231 to 304)	111 (90 to 135)	133 (108 to 164)	13 (6 to 25)	9 (9 to 9)
Honduras	2003	134 (118 to 153)	54 (45 to 66)	67 (54 to 81)	6 (3 to 12)	7 (7 to 7)	282 (247 to 320)	114 (93 to 138)	140 (114 to 170)	13 (6 to 24)	15 (15 to 15)
Honduras	2004	144 (127 to 163)	57 (46 to 69)	71 (58 to 86)	6 (3 to 12)	10 (10 to 10)	302 (266 to 341)	118 (97 to 144)	149 (121 to 180)	13 (6 to 24)	21 (21 to 21)

Honduras	2005	154 (136 to 175)	60 (49 to 73)	76 (62 to 92)	6 (3 to 12)	11 (11 to 11)	323 (285 to 367)	126 (103 to 153)	160 (131 to 192)	13 (6 to 24)	24 (24 to 24)
Honduras	2006	165 (145 to 187)	68 (56 to 82)	83 (69 to 101)	6 (3 to 12)	7 (7 to 7)	345 (303 to 391)	141 (117 to 171)	175 (144 to 211)	14 (7 to 25)	16 (16 to 16)
Honduras	2007	177 (155 to 201)	73 (60 to 89)	89 (73 to 108)	7 (3 to 12)	8 (8 to 8)	370 (324 to 421)	153 (126 to 186)	186 (152 to 225)	14 (7 to 25)	17 (17 to 17)
Honduras	2008	186 (162 to 211)	78 (64 to 95)	93 (77 to 112)	7 (3 to 12)	8 (8 to 8)	390 (340 to 442)	164 (135 to 199)	196 (161 to 234)	14 (7 to 25)	16 (16 to 16)
Honduras	2009	191 (166 to 216)	83 (68 to 101)	93 (77 to 113)	7 (3 to 12)	7 (7 to 7)	399 (347 to 452)	173 (143 to 212)	196 (161 to 236)	14 (7 to 26)	16 (16 to 16)
Honduras	2010	187 (163 to 212)	81 (67 to 98)	92 (76 to 111)	7 (4 to 13)	7 (7 to 7)	391 (341 to 444)	169 (140 to 204)	193 (159 to 233)	15 (8 to 28)	14 (14 to 14)
Honduras	2011	188 (166 to 214)	80 (67 to 97)	93 (77 to 112)	8 (4 to 14)	7 (7 to 7)	394 (347 to 448)	168 (140 to 204)	194 (160 to 234)	16 (8 to 30)	16 (16 to 16)
Honduras	2012	190 (167 to 216)	78 (65 to 94)	94 (78 to 113)	8 (4 to 15)	9 (9 to 9)	397 (349 to 451)	164 (135 to 196)	196 (162 to 237)	17 (9 to 31)	20 (20 to 20)
Honduras	2013	191 (167 to 218)	80 (66 to 95)	95 (78 to 115)	9 (5 to 16)	8 (8 to 8)	400 (349 to 456)	167 (137 to 199)	199 (163 to 240)	19 (9 to 34)	16 (16 to 16)
Honduras	2014	183 (160 to 209)	76 (62 to 91)	92 (75 to 111)	9 (5 to 17)	6 (6 to 6)	383 (334 to 437)	158 (129 to 190)	192 (158 to 233)	19 (10 to 35)	13 (13 to 13)
Honduras	2015	178 (155 to 202)	73 (59 to 87)	90 (72 to 109)	9 (5 to 17)	6 (6 to 6)	373 (325 to 424)	153 (124 to 182)	188 (152 to 229)	19 (10 to 35)	13 (13 to 13)
Honduras	2016	185 (160 to 211)	77 (63 to 93)	92 (74 to 113)	10 (5 to 18)	5 (5 to 5)	388 (335 to 442)	162 (131 to 195)	193 (156 to 237)	21 (11 to 38)	11 (11 to 11)
Honduras	2017	185 (155 to 218)	78 (63 to 95)	93 (74 to 119)	11 (5 to 19)	3 (3 to 3)	387 (324 to 457)	163 (131 to 198)	195 (154 to 249)	22 (11 to 41)	6 (6 to 6)
Honduras	2018	187 (156 to 224)	79 (63 to 96)	95 (75 to 121)	11 (5 to 20)	3 (3 to 3)	392 (326 to 469)	165 (132 to 201)	198 (157 to 253)	23 (11 to 41)	6 (6 to 6)
Honduras	2019	192 (160 to 226)	82 (65 to 99)	96 (76 to 123)	11 (6 to 20)	3 (3 to 3)	401 (335 to 473)	171 (136 to 208)	201 (159 to 256)	23 (12 to 42)	6 (6 to 6)

Honduras	2020	195 (163 to 233)	83 (66 to 101)	98 (77 to 124)	11 (6 to 21)	3 (3 to 3)	409 (342 to 488)	174 (139 to 212)	205 (162 to 260)	24 (12 to 43)	6 (6 to 6)
Honduras	2021	199 (166 to 235)	85 (68 to 105)	99 (79 to 126)	12 (6 to 21)	3 (3 to 3)	417 (348 to 492)	179 (143 to 219)	208 (165 to 265)	24 (12 to 44)	6 (6 to 6)
Honduras	2022	203 (169 to 243)	87 (69 to 107)	101 (80 to 128)	12 (6 to 22)	3 (3 to 3)	425 (353 to 508)	183 (145 to 224)	211 (167 to 268)	25 (13 to 45)	6 (5 to 7)
Honduras	2023	206 (172 to 244)	89 (71 to 109)	102 (81 to 130)	12 (6 to 22)	3 (3 to 3)	432 (360 to 510)	187 (148 to 229)	214 (169 to 272)	26 (13 to 46)	6 (5 to 7)
Honduras	2024	210 (174 to 250)	91 (72 to 112)	103 (82 to 131)	12 (6 to 23)	3 (3 to 3)	439 (364 to 524)	191 (151 to 235)	216 (171 to 275)	26 (13 to 47)	6 (5 to 7)
Honduras	2025	213 (177 to 252)	93 (74 to 115)	104 (82 to 132)	13 (6 to 23)	3 (3 to 3)	445 (371 to 528)	195 (155 to 240)	218 (172 to 277)	27 (13 to 48)	6 (5 to 7)
Honduras	2026	216 (179 to 259)	95 (75 to 118)	105 (83 to 133)	13 (7 to 23)	3 (3 to 3)	452 (375 to 542)	199 (156 to 248)	220 (174 to 279)	27 (14 to 49)	6 (5 to 7)
Honduras	2027	219 (182 to 261)	97 (76 to 121)	106 (84 to 135)	13 (7 to 24)	3 (3 to 3)	459 (381 to 546)	203 (159 to 254)	222 (175 to 282)	28 (14 to 50)	6 (5 to 7)
Honduras	2028	223 (184 to 266)	99 (77 to 125)	107 (85 to 136)	13 (7 to 24)	3 (3 to 3)	466 (385 to 558)	207 (162 to 261)	224 (177 to 285)	28 (14 to 51)	6 (5 to 7)
Honduras	2029	226 (186 to 269)	101 (78 to 126)	108 (85 to 137)	14 (7 to 25)	3 (3 to 3)	472 (390 to 563)	211 (164 to 264)	226 (178 to 288)	29 (14 to 52)	6 (5 to 7)
Honduras	2030	229 (189 to 275)	103 (80 to 130)	109 (86 to 139)	14 (7 to 25)	3 (3 to 4)	479 (396 to 576)	215 (167 to 272)	229 (180 to 291)	29 (15 to 53)	6 (5 to 7)
Honduras	2031	232 (192 to 277)	105 (81 to 132)	110 (87 to 140)	14 (7 to 26)	3 (3 to 4)	486 (401 to 580)	220 (169 to 276)	231 (181 to 293)	30 (15 to 54)	6 (5 to 7)
Honduras	2032	236 (194 to 283)	107 (83 to 135)	111 (87 to 141)	15 (7 to 26)	3 (3 to 4)	494 (406 to 593)	224 (173 to 282)	233 (183 to 295)	31 (15 to 55)	6 (5 to 8)
Honduras	2033	240 (198 to 287)	109 (84 to 136)	112 (88 to 143)	15 (7 to 27)	3 (3 to 4)	502 (414 to 600)	229 (177 to 285)	235 (185 to 299)	31 (16 to 57)	6 (5 to 8)
Honduras	2034	243 (199 to 295)	111 (86 to 141)	114 (89 to 144)	15 (8 to 28)	3 (3 to 4)	509 (418 to 618)	233 (180 to 294)	238 (186 to 302)	32 (16 to 58)	6 (5 to 8)

Honduras	2035	247 (203 to 296)	114 (87 to 144)	115 (90 to 146)	16 (8 to 28)	3 (3 to 4)	517 (425 to 619)	238 (182 to 302)	240 (188 to 305)	32 (16 to 59)	6 (5 to 8)
Honduras	2036	251 (205 to 303)	116 (89 to 147)	116 (91 to 147)	16 (8 to 29)	3 (3 to 4)	525 (430 to 635)	243 (186 to 308)	243 (190 to 309)	33 (17 to 61)	6 (5 to 8)
Honduras	2037	255 (209 to 306)	118 (90 to 150)	117 (91 to 149)	16 (8 to 30)	3 (3 to 4)	534 (438 to 641)	248 (188 to 313)	246 (191 to 312)	34 (17 to 62)	7 (5 to 8)
Honduras	2038	259 (211 to 312)	121 (92 to 153)	119 (92 to 150)	16 (8 to 30)	3 (3 to 4)	542 (441 to 654)	253 (193 to 320)	248 (193 to 315)	35 (17 to 63)	7 (5 to 8)
Honduras	2039	263 (215 to 316)	123 (94 to 156)	120 (93 to 152)	17 (8 to 31)	3 (3 to 4)	550 (449 to 662)	257 (197 to 327)	250 (194 to 318)	35 (17 to 64)	7 (5 to 8)
Honduras	2040	266 (216 to 322)	125 (95 to 158)	121 (94 to 153)	17 (9 to 31)	3 (3 to 4)	557 (451 to 674)	262 (199 to 332)	253 (196 to 320)	36 (18 to 66)	7 (6 to 9)
Honduras	2041	270 (220 to 326)	128 (97 to 163)	122 (94 to 155)	17 (9 to 32)	3 (3 to 4)	566 (460 to 683)	267 (204 to 341)	255 (197 to 323)	37 (18 to 67)	7 (6 to 9)
Honduras	2042	274 (222 to 332)	130 (98 to 167)	123 (95 to 156)	18 (9 to 32)	3 (3 to 4)	574 (465 to 695)	272 (205 to 349)	257 (199 to 326)	37 (19 to 68)	7 (6 to 9)
Honduras	2043	278 (226 to 336)	132 (100 to 170)	124 (96 to 158)	18 (9 to 33)	3 (3 to 4)	581 (472 to 703)	277 (209 to 356)	259 (200 to 330)	38 (19 to 69)	7 (6 to 9)
Honduras	2044	282 (227 to 341)	135 (101 to 172)	125 (96 to 159)	18 (9 to 34)	3 (3 to 4)	589 (475 to 714)	282 (211 to 359)	261 (201 to 334)	39 (19 to 71)	7 (6 to 9)
Honduras	2045	285 (230 to 347)	137 (103 to 176)	126 (97 to 161)	19 (9 to 34)	3 (3 to 4)	597 (482 to 726)	287 (216 to 368)	263 (203 to 336)	39 (19 to 72)	7 (6 to 9)
Honduras	2046	289 (233 to 353)	140 (103 to 179)	127 (97 to 162)	19 (9 to 35)	3 (3 to 4)	605 (487 to 738)	292 (215 to 375)	266 (204 to 339)	40 (20 to 73)	7 (6 to 9)
Honduras	2047	293 (236 to 357)	142 (104 to 182)	128 (98 to 163)	19 (10 to 36)	3 (3 to 5)	613 (495 to 747)	297 (219 to 381)	268 (205 to 342)	41 (20 to 75)	7 (6 to 10)
Honduras	2048	297 (237 to 364)	145 (107 to 186)	129 (99 to 164)	20 (10 to 36)	4 (3 to 5)	621 (495 to 762)	303 (224 to 389)	270 (206 to 344)	41 (20 to 76)	7 (6 to 10)
Honduras	2049	301 (242 to 366)	147 (109 to 191)	130 (99 to 166)	20 (10 to 37)	4 (3 to 5)	629 (506 to 765)	308 (229 to 399)	272 (208 to 347)	42 (21 to 78)	7 (6 to 10)

Honduras	2050	304 (244 to 376)	149 (110 to 195)	131 (100 to 167)	20 (10 to 38)	4 (3 to 5)	637 (510 to 786)	313 (231 to 408)	274 (210 to 349)	43 (21 to 79)	8 (6 to 10)
Hungary	1995	654 (607 to 704)	512 (473 to 555)	113 (95 to 136)	29 (16 to 49)	0 (0 to 0)	1275 (1183 to 1372)	997 (922 to 1081)	221 (185 to 265)	56 (31 to 95)	0 (0 to 0)
Hungary	1996	635 (598 to 676)	486 (457 to 519)	120 (103 to 139)	28 (16 to 46)	1 (1 to 1)	1237 (1165 to 1317)	948 (891 to 1011)	233 (201 to 272)	54 (31 to 90)	3 (3 to 3)
Hungary	1997	640 (605 to 679)	480 (453 to 510)	131 (114 to 151)	28 (16 to 45)	1 (1 to 1)	1248 (1179 to 1322)	936 (883 to 995)	255 (223 to 293)	54 (32 to 87)	3 (3 to 3)
Hungary	1998	671 (634 to 708)	489 (462 to 519)	152 (135 to 173)	29 (17 to 44)	1 (1 to 1)	1307 (1236 to 1380)	953 (900 to 1011)	297 (264 to 338)	56 (33 to 87)	1 (1 to 1)
Hungary	1999	690 (653 to 729)	489 (462 to 518)	173 (155 to 195)	28 (17 to 44)	0 (0 to 0)	1345 (1273 to 1420)	952 (900 to 1010)	337 (301 to 380)	55 (33 to 85)	1 (1 to 1)
Hungary	2000	719 (681 to 759)	498 (470 to 528)	192 (171 to 216)	29 (17 to 43)	0 (0 to 0)	1401 (1326 to 1479)	971 (917 to 1030)	374 (334 to 422)	56 (34 to 84)	1 (1 to 1)
Hungary	2001	771 (731 to 810)	528 (500 to 558)	212 (189 to 237)	30 (19 to 45)	0 (0 to 0)	1502 (1424 to 1578)	1030 (975 to 1088)	414 (369 to 461)	59 (38 to 87)	0 (0 to 0)
Hungary	2002	873 (830 to 914)	604 (573 to 636)	236 (211 to 261)	33 (22 to 48)	0 (0 to 0)	1701 (1618 to 1782)	1178 (1116 to 1239)	459 (412 to 508)	65 (43 to 94)	0 (0 to 0)
Hungary	2003	935 (893 to 977)	648 (616 to 682)	250 (227 to 274)	37 (25 to 52)	0 (0 to 0)	1822 (1740 to 1904)	1263 (1201 to 1329)	488 (442 to 535)	71 (49 to 101)	0 (0 to 0)

Hungary	2004	985 (941 to 1028)	684 (653 to 719)	259 (237 to 282)	41 (29 to 58)	0 (0 to 0)	1919 (1833 to 2003)	1334 (1273 to 1402)	505 (462 to 550)	80 (56 to 113)	0 (0 to 0)
Hungary	2005	1034 (992 to 1079)	720 (689 to 755)	269 (246 to 292)	45 (32 to 62)	0 (0 to 0)	2015 (1933 to 2103)	1404 (1343 to 1471)	524 (480 to 569)	87 (62 to 122)	0 (0 to 0)
Hungary	2006	1067 (1025 to 1110)	742 (712 to 775)	274 (253 to 297)	50 (36 to 68)	0 (0 to 0)	2079 (1997 to 2162)	1447 (1387 to 1511)	535 (493 to 578)	98 (71 to 132)	0 (0 to 0)
Hungary	2007	1024 (985 to 1063)	702 (673 to 732)	270 (249 to 291)	52 (37 to 69)	0 (0 to 0)	1995 (1921 to 2072)	1368 (1312 to 1427)	526 (485 to 567)	101 (73 to 134)	0 (0 to 0)
Hungary	2008	1002 (966 to 1039)	680 (652 to 708)	268 (247 to 288)	54 (40 to 69)	0 (0 to 0)	1952 (1883 to 2026)	1325 (1271 to 1380)	522 (482 to 562)	105 (78 to 135)	0 (0 to 0)
Hungary	2009	984 (950 to 1023)	663 (636 to 693)	266 (247 to 287)	55 (42 to 72)	0 (0 to 0)	1919 (1851 to 1994)	1293 (1239 to 1350)	518 (482 to 559)	108 (81 to 141)	0 (0 to 0)
Hungary	2010	988 (952 to 1028)	658 (629 to 689)	273 (254 to 294)	57 (43 to 73)	0 (0 to 0)	1925 (1855 to 2002)	1283 (1226 to 1343)	532 (495 to 574)	110 (84 to 143)	0 (0 to 0)
Hungary	2011	1004 (964 to 1044)	665 (634 to 695)	282 (262 to 306)	57 (44 to 75)	0 (0 to 0)	1956 (1878 to 2034)	1295 (1236 to 1355)	550 (510 to 596)	111 (85 to 146)	0 (0 to 0)
Hungary	2012	983 (945 to 1024)	645 (617 to 675)	283 (262 to 307)	54 (42 to 72)	0 (0 to 0)	1915 (1842 to 1996)	1258 (1202 to 1316)	551 (510 to 598)	106 (81 to 141)	0 (0 to 0)
Hungary	2013	999 (959 to 1038)	660 (630 to 689)	285 (263 to 310)	53 (40 to 71)	0 (0 to 0)	1947 (1869 to 2023)	1287 (1228 to 1342)	556 (513 to 604)	104 (77 to 138)	0 (0 to 0)

Hungary	2014	1021 (981 to 1060)	681 (649 to 710)	288 (267 to 312)	52 (38 to 69)	0 (0 to 0)	1989 (1912 to 2065)	1327 (1266 to 1383)	561 (520 to 607)	101 (74 to 134)	0 (0 to 0)
Hungary	2015	1061 (1022 to 1103)	716 (687 to 748)	293 (272 to 315)	52 (38 to 69)	0 (0 to 0)	2067 (1991 to 2149)	1396 (1338 to 1457)	571 (530 to 614)	100 (74 to 134)	0 (0 to 0)
Hungary	2016	1061 (1020 to 1100)	718 (688 to 751)	293 (271 to 317)	50 (35 to 67)	0 (0 to 0)	2067 (1989 to 2143)	1399 (1341 to 1464)	572 (529 to 618)	97 (69 to 130)	0 (0 to 0)
Hungary	2017	1107 (1042 to 1180)	757 (717 to 802)	299 (271 to 328)	51 (35 to 71)	0 (0 to 0)	2157 (2030 to 2299)	1476 (1396 to 1564)	583 (528 to 640)	99 (68 to 138)	0 (0 to 0)
Hungary	2018	1161 (1093 to 1235)	794 (748 to 839)	313 (283 to 344)	54 (37 to 75)	0 (0 to 0)	2262 (2130 to 2408)	1548 (1457 to 1636)	610 (552 to 670)	104 (72 to 146)	0 (0 to 0)
Hungary	2019	1222 (1144 to 1308)	838 (786 to 890)	327 (296 to 360)	56 (39 to 78)	0 (0 to 0)	2382 (2230 to 2550)	1634 (1533 to 1734)	638 (577 to 701)	110 (76 to 153)	0 (0 to 0)
Hungary	2020	1265 (1186 to 1354)	868 (812 to 922)	338 (306 to 373)	59 (40 to 82)	0 (0 to 0)	2466 (2311 to 2638)	1692 (1583 to 1797)	660 (597 to 727)	114 (79 to 159)	0 (0 to 0)
Hungary	2021	1286 (1202 to 1379)	883 (826 to 939)	344 (311 to 378)	60 (41 to 83)	0 (0 to 0)	2507 (2342 to 2687)	1720 (1609 to 1829)	670 (606 to 737)	117 (81 to 163)	0 (0 to 0)
Hungary	2022	1309 (1224 to 1399)	898 (839 to 954)	349 (315 to 384)	61 (42 to 85)	0 (0 to 0)	2550 (2385 to 2727)	1750 (1636 to 1860)	681 (615 to 749)	119 (82 to 166)	0 (0 to 0)
Hungary	2023	1323 (1238 to 1416)	909 (847 to 970)	352 (318 to 387)	62 (43 to 87)	0 (0 to 0)	2578 (2413 to 2759)	1771 (1651 to 1891)	685 (619 to 755)	121 (84 to 169)	0 (0 to 0)

Hungary	2024	1338 (1253 to 1433)	921 (858 to 983)	354 (320 to 391)	63 (43 to 88)	0 (0 to 0)	2608 (2441 to 2793)	1795 (1672 to 1916)	691 (623 to 761)	123 (85 to 171)	0 (0 to 0)
Hungary	2025	1353 (1265 to 1446)	932 (870 to 995)	358 (323 to 394)	64 (44 to 88)	0 (0 to 0)	2637 (2464 to 2818)	1816 (1695 to 1939)	697 (629 to 768)	124 (86 to 172)	0 (0 to 0)
Hungary	2026	1370 (1282 to 1466)	945 (882 to 1008)	361 (326 to 397)	65 (45 to 90)	0 (0 to 0)	2670 (2498 to 2857)	1841 (1720 to 1965)	703 (634 to 774)	126 (87 to 175)	0 (0 to 0)
Hungary	2027	1387 (1297 to 1491)	958 (891 to 1025)	364 (328 to 400)	66 (45 to 91)	0 (0 to 0)	2704 (2527 to 2906)	1868 (1736 to 1998)	709 (640 to 780)	128 (88 to 177)	0 (0 to 0)
Hungary	2028	1406 (1312 to 1509)	972 (904 to 1043)	367 (331 to 404)	67 (46 to 92)	0 (0 to 0)	2739 (2557 to 2940)	1895 (1762 to 2033)	715 (645 to 786)	130 (90 to 180)	0 (0 to 0)
Hungary	2029	1424 (1328 to 1530)	987 (915 to 1062)	370 (333 to 407)	68 (47 to 94)	0 (0 to 0)	2776 (2588 to 2983)	1923 (1784 to 2069)	721 (650 to 793)	132 (91 to 183)	0 (0 to 0)
Hungary	2030	1445 (1343 to 1559)	1002 (929 to 1082)	374 (337 to 411)	69 (47 to 95)	0 (0 to 0)	2816 (2617 to 3039)	1954 (1810 to 2108)	729 (656 to 801)	134 (92 to 186)	0 (0 to 0)
Hungary	2031	1469 (1365 to 1582)	1020 (946 to 1105)	379 (341 to 417)	70 (48 to 97)	0 (0 to 0)	2862 (2660 to 3083)	1988 (1843 to 2154)	739 (665 to 812)	136 (94 to 189)	0 (0 to 0)
Hungary	2032	1495 (1384 to 1619)	1039 (960 to 1127)	385 (346 to 423)	71 (49 to 99)	0 (0 to 0)	2914 (2697 to 3155)	2025 (1870 to 2197)	750 (675 to 825)	139 (96 to 193)	0 (0 to 0)
Hungary	2033	1525 (1419 to 1650)	1061 (980 to 1153)	392 (352 to 431)	73 (50 to 101)	0 (0 to 0)	2972 (2765 to 3216)	2067 (1909 to 2247)	763 (687 to 840)	142 (98 to 197)	0 (0 to 0)

Hungary	2034	1555 (1437 to 1690)	1082 (995 to 1175)	398 (358 to 439)	74 (51 to 103)	0 (0 to 0)	3031 (2801 to 3294)	2109 (1939 to 2289)	777 (699 to 855)	145 (100 to 201)	0 (0 to 0)
Hungary	2035	1585 (1466 to 1718)	1104 (1013 to 1199)	405 (365 to 447)	76 (52 to 106)	0 (0 to 0)	3088 (2857 to 3347)	2151 (1974 to 2336)	790 (710 to 871)	148 (102 to 206)	0 (0 to 0)
Hungary	2036	1614 (1488 to 1755)	1125 (1033 to 1224)	412 (370 to 454)	77 (53 to 108)	0 (0 to 0)	3146 (2901 to 3420)	2193 (2013 to 2385)	803 (721 to 886)	151 (104 to 210)	0 (0 to 0)
Hungary	2037	1643 (1515 to 1781)	1145 (1051 to 1247)	418 (375 to 461)	79 (54 to 110)	0 (0 to 0)	3201 (2952 to 3471)	2232 (2048 to 2430)	815 (732 to 898)	154 (106 to 214)	0 (0 to 0)
Hungary	2038	1668 (1537 to 1814)	1164 (1063 to 1267)	424 (381 to 467)	80 (55 to 112)	0 (0 to 0)	3252 (2996 to 3536)	2268 (2072 to 2470)	827 (742 to 911)	156 (108 to 218)	0 (0 to 0)
Hungary	2039	1693 (1558 to 1848)	1181 (1084 to 1286)	430 (385 to 474)	82 (56 to 113)	0 (0 to 0)	3299 (3037 to 3601)	2302 (2112 to 2506)	838 (751 to 924)	159 (109 to 221)	0 (0 to 0)
Hungary	2040	1715 (1564 to 1873)	1197 (1092 to 1304)	435 (390 to 480)	83 (57 to 115)	0 (0 to 0)	3343 (3048 to 3650)	2333 (2128 to 2540)	849 (760 to 936)	161 (111 to 224)	0 (0 to 0)
Hungary	2041	1739 (1602 to 1889)	1214 (1103 to 1325)	441 (395 to 487)	84 (58 to 117)	0 (0 to 0)	3389 (3122 to 3682)	2366 (2149 to 2583)	859 (769 to 950)	164 (112 to 228)	0 (0 to 0)
Hungary	2042	1764 (1614 to 1923)	1232 (1119 to 1350)	447 (400 to 494)	85 (58 to 119)	0 (0 to 0)	3438 (3146 to 3748)	2402 (2180 to 2631)	871 (779 to 963)	166 (114 to 231)	0 (0 to 0)
Hungary	2043	1792 (1641 to 1952)	1252 (1138 to 1372)	453 (405 to 501)	87 (59 to 121)	0 (0 to 0)	3491 (3198 to 3804)	2440 (2217 to 2673)	883 (790 to 976)	169 (115 to 236)	0 (0 to 0)

Hungary	2044	1820 (1659 to 1994)	1272 (1156 to 1397)	459 (410 to 509)	88 (60 to 123)	0 (0 to 0)	3546 (3233 to 3886)	2479 (2252 to 2722)	895 (799 to 992)	172 (117 to 241)	0 (0 to 0)
Hungary	2045	1850 (1694 to 2020)	1294 (1176 to 1424)	466 (415 to 517)	90 (61 to 126)	0 (0 to 0)	3604 (3301 to 3936)	2522 (2291 to 2775)	908 (809 to 1007)	175 (119 to 246)	0 (0 to 0)
Hungary	2046	1881 (1710 to 2056)	1317 (1193 to 1448)	473 (421 to 525)	91 (62 to 129)	0 (0 to 0)	3666 (3332 to 4007)	2566 (2325 to 2823)	921 (820 to 1023)	178 (122 to 251)	0 (0 to 0)
Hungary	2047	1915 (1753 to 2096)	1342 (1214 to 1480)	480 (426 to 534)	93 (64 to 132)	0 (0 to 0)	3732 (3415 to 4085)	2614 (2366 to 2885)	936 (831 to 1040)	182 (124 to 256)	0 (0 to 0)
Hungary	2048	1951 (1775 to 2142)	1368 (1233 to 1510)	488 (433 to 543)	95 (65 to 135)	0 (0 to 0)	3802 (3459 to 4174)	2665 (2403 to 2944)	951 (843 to 1058)	185 (127 to 262)	0 (0 to 0)
Hungary	2049	1989 (1814 to 2178)	1396 (1258 to 1542)	496 (439 to 553)	97 (66 to 138)	0 (0 to 0)	3877 (3534 to 4245)	2720 (2452 to 3006)	967 (856 to 1077)	189 (129 to 269)	0 (0 to 0)
Hungary	2050	2030 (1837 to 2239)	1426 (1276 to 1582)	505 (446 to 563)	99 (68 to 141)	0 (0 to 0)	3956 (3580 to 4363)	2779 (2486 to 3084)	984 (870 to 1097)	194 (132 to 275)	0 (0 to 0)
Iceland	1995	3239 (3053 to 3433)	2456 (2275 to 2643)	783 (733 to 832)	0 (0 to 0)	0 (0 to 0)	2737 (2580 to 2901)	2075 (1923 to 2233)	662 (619 to 703)	0 (0 to 0)	0 (0 to 0)
Iceland	1996	3345 (3190 to 3507)	2553 (2410 to 2713)	792 (753 to 828)	0 (0 to 0)	0 (0 to 0)	2826 (2696 to 2964)	2157 (2036 to 2292)	669 (636 to 700)	0 (0 to 0)	0 (0 to 0)
Iceland	1997	3526 (3376 to 3673)	2721 (2577 to 2868)	806 (770 to 843)	0 (0 to 0)	0 (0 to 0)	2980 (2852 to 3104)	2299 (2178 to 2423)	681 (650 to 712)	0 (0 to 0)	0 (0 to 0)

Iceland	1998	3829 (3679 to 3981)	3003 (2853 to 3154)	826 (791 to 866)	0 (0 to 0)	0 (0 to 0)	3236 (3109 to 3364)	2537 (2410 to 2665)	698 (668 to 732)	0 (0 to 0)	0 (0 to 0)
Iceland	1999	4163 (4008 to 4319)	3319 (3166 to 3469)	844 (810 to 880)	0 (0 to 0)	0 (0 to 0)	3518 (3387 to 3649)	2804 (2675 to 2932)	713 (684 to 744)	0 (0 to 0)	0 (0 to 0)
Iceland	2000	4333 (4184 to 4479)	3484 (3339 to 3633)	849 (818 to 882)	0 (0 to 0)	0 (0 to 0)	3661 (3535 to 3785)	2944 (2821 to 3070)	718 (691 to 745)	0 (0 to 0)	0 (0 to 0)
Iceland	2001	4538 (4387 to 4685)	3677 (3525 to 3822)	861 (830 to 891)	0 (0 to 0)	0 (0 to 0)	3835 (3707 to 3959)	3107 (2979 to 3229)	727 (701 to 753)	0 (0 to 0)	0 (0 to 0)
Iceland	2002	4738 (4587 to 4886)	3868 (3723 to 4012)	869 (838 to 901)	0 (0 to 0)	0 (0 to 0)	4004 (3876 to 4129)	3269 (3146 to 3390)	735 (708 to 761)	0 (0 to 0)	0 (0 to 0)
Iceland	2003	4943 (4790 to 5096)	4061 (3913 to 4212)	874 (843 to 905)	8 (6 to 10)	0 (0 to 0)	4177 (4047 to 4306)	3432 (3306 to 3559)	739 (712 to 765)	6 (5 to 8)	0 (0 to 0)
Iceland	2004	5126 (4969 to 5293)	4194 (4041 to 4354)	886 (854 to 918)	46 (36 to 57)	0 (0 to 0)	4331 (4199 to 4472)	3544 (3415 to 3679)	748 (721 to 776)	39 (31 to 48)	0 (0 to 0)
Iceland	2005	5232 (5068 to 5398)	4251 (4092 to 4415)	884 (853 to 915)	97 (79 to 118)	0 (0 to 0)	4421 (4282 to 4561)	3592 (3457 to 3730)	747 (721 to 773)	82 (67 to 100)	0 (0 to 0)
Iceland	2006	5170 (5017 to 5337)	4194 (4048 to 4354)	860 (830 to 889)	116 (94 to 140)	0 (0 to 0)	4369 (4239 to 4510)	3544 (3421 to 3679)	726 (702 to 752)	98 (80 to 119)	0 (0 to 0)
Iceland	2007	5120 (4961 to 5288)	4169 (4017 to 4332)	842 (812 to 875)	109 (87 to 131)	0 (0 to 0)	4327 (4192 to 4469)	3523 (3395 to 3661)	712 (686 to 739)	92 (74 to 111)	0 (0 to 0)

Iceland	2008	5869 (5684 to 6068)	4853 (4665 to 5038)	905 (874 to 939)	111 (91 to 133)	0 (0 to 0)	4959 (4803 to 5128)	4101 (3942 to 4257)	765 (738 to 793)	94 (77 to 113)	0 (0 to 0)
Iceland	2009	5011 (4841 to 5180)	4083 (3922 to 4243)	846 (817 to 876)	82 (67 to 99)	0 (0 to 0)	4234 (4091 to 4377)	3450 (3314 to 3585)	715 (690 to 740)	69 (56 to 84)	0 (0 to 0)
Iceland	2010	4791 (4631 to 4962)	3869 (3710 to 4029)	851 (821 to 879)	71 (58 to 86)	0 (0 to 0)	4048 (3913 to 4193)	3269 (3135 to 3405)	719 (694 to 743)	60 (49 to 73)	0 (0 to 0)
Iceland	2011	4634 (4485 to 4798)	3713 (3561 to 3867)	855 (826 to 884)	66 (53 to 81)	0 (0 to 0)	3916 (3790 to 4054)	3138 (3009 to 3267)	722 (698 to 747)	56 (45 to 68)	0 (0 to 0)
Iceland	2012	4695 (4544 to 4856)	3758 (3609 to 3915)	870 (839 to 899)	67 (54 to 81)	0 (0 to 0)	3967 (3840 to 4103)	3176 (3049 to 3309)	735 (709 to 760)	56 (46 to 68)	0 (0 to 0)
Iceland	2013	4761 (4616 to 4925)	3814 (3670 to 3976)	878 (848 to 909)	68 (56 to 83)	0 (0 to 0)	4023 (3901 to 4162)	3223 (3101 to 3359)	742 (716 to 768)	58 (48 to 70)	0 (0 to 0)
Iceland	2014	4938 (4792 to 5100)	3977 (3833 to 4136)	888 (859 to 919)	73 (61 to 89)	0 (0 to 0)	4172 (4049 to 4310)	3360 (3239 to 3495)	750 (726 to 776)	62 (51 to 75)	0 (0 to 0)
Iceland	2015	4955 (4801 to 5111)	4002 (3854 to 4152)	877 (847 to 910)	76 (62 to 92)	0 (0 to 0)	4187 (4056 to 4319)	3382 (3257 to 3508)	741 (715 to 769)	64 (53 to 78)	0 (0 to 0)
Iceland	2016	5391 (5220 to 5569)	4398 (4233 to 4566)	907 (874 to 941)	86 (69 to 106)	0 (0 to 0)	4555 (4411 to 4705)	3716 (3577 to 3858)	766 (738 to 795)	73 (59 to 90)	0 (0 to 0)
Iceland	2017	5538 (5290 to 5805)	4529 (4317 to 4765)	917 (872 to 963)	92 (70 to 115)	0 (0 to 0)	4680 (4470 to 4905)	3827 (3648 to 4026)	775 (737 to 813)	77 (60 to 98)	0 (0 to 0)

Iceland	2018	5577 (5300 to 5859)	4552 (4298 to 4811)	931 (885 to 978)	94 (72 to 118)	0 (0 to 0)	4712 (4478 to 4951)	3847 (3632 to 4066)	787 (748 to 827)	79 (61 to 99)	0 (0 to 0)
Iceland	2019	5479 (5198 to 5783)	4465 (4207 to 4738)	921 (876 to 967)	93 (72 to 117)	0 (0 to 0)	4629 (4392 to 4887)	3773 (3555 to 4004)	778 (740 to 817)	79 (60 to 99)	0 (0 to 0)
Iceland	2020	5488 (5199 to 5802)	4473 (4210 to 4748)	921 (875 to 967)	94 (72 to 117)	0 (0 to 0)	4638 (4393 to 4903)	3780 (3557 to 4012)	778 (739 to 817)	79 (61 to 99)	0 (0 to 0)
Iceland	2021	5519 (5229 to 5834)	4499 (4221 to 4805)	926 (879 to 973)	94 (72 to 119)	0 (0 to 0)	4663 (4419 to 4929)	3801 (3567 to 4060)	782 (743 to 822)	80 (61 to 100)	0 (0 to 0)
Iceland	2022	5561 (5257 to 5891)	4536 (4244 to 4832)	931 (882 to 978)	95 (73 to 120)	0 (0 to 0)	4699 (4442 to 4978)	3833 (3586 to 4083)	786 (745 to 827)	80 (62 to 101)	0 (0 to 0)
Iceland	2023	5595 (5271 to 5957)	4565 (4266 to 4908)	934 (886 to 982)	96 (73 to 121)	0 (0 to 0)	4728 (4454 to 5034)	3858 (3605 to 4147)	789 (748 to 830)	81 (62 to 102)	0 (0 to 0)
Iceland	2024	5599 (5256 to 5963)	4570 (4266 to 4902)	932 (885 to 981)	96 (74 to 121)	0 (0 to 0)	4731 (4441 to 5039)	3862 (3605 to 4142)	788 (748 to 829)	81 (62 to 102)	0 (0 to 0)
Iceland	2025	5603 (5257 to 5978)	4576 (4253 to 4923)	931 (882 to 980)	96 (74 to 121)	0 (0 to 0)	4734 (4443 to 5051)	3866 (3593 to 4160)	787 (745 to 828)	81 (62 to 102)	0 (0 to 0)
Iceland	2026	5610 (5240 to 5994)	4584 (4236 to 4947)	930 (880 to 979)	96 (74 to 121)	0 (0 to 0)	4740 (4428 to 5065)	3873 (3580 to 4180)	786 (744 to 827)	81 (62 to 102)	0 (0 to 0)
Iceland	2027	5609 (5242 to 5988)	4584 (4246 to 4947)	929 (880 to 979)	96 (74 to 121)	0 (0 to 0)	4739 (4430 to 5060)	3873 (3588 to 4181)	785 (744 to 827)	81 (62 to 103)	0 (0 to 0)

Iceland	2028	5610 (5215 to 6012)	4585 (4222 to 4957)	929 (880 to 980)	96 (74 to 122)	0 (0 to 0)	4741 (4407 to 5080)	3874 (3568 to 4189)	785 (744 to 828)	81 (62 to 103)	0 (0 to 0)
Iceland	2029	5624 (5234 to 6020)	4597 (4220 to 4978)	930 (881 to 982)	97 (74 to 122)	0 (0 to 0)	4752 (4423 to 5087)	3884 (3566 to 4207)	786 (744 to 830)	82 (63 to 103)	0 (0 to 0)
Iceland	2030	5656 (5264 to 6079)	4625 (4242 to 5026)	934 (883 to 987)	97 (75 to 124)	0 (0 to 0)	4780 (4448 to 5137)	3908 (3584 to 4247)	790 (746 to 834)	82 (63 to 104)	0 (0 to 0)
Iceland	2031	5701 (5289 to 6151)	4662 (4265 to 5081)	941 (889 to 994)	98 (76 to 125)	0 (0 to 0)	4817 (4469 to 5197)	3940 (3604 to 4293)	795 (752 to 840)	83 (64 to 106)	0 (0 to 0)
Iceland	2032	5744 (5323 to 6216)	4699 (4303 to 5127)	946 (894 to 1001)	99 (76 to 126)	0 (0 to 0)	4853 (4498 to 5252)	3970 (3636 to 4333)	799 (755 to 846)	84 (64 to 107)	0 (0 to 0)
Iceland	2033	5783 (5341 to 6244)	4733 (4323 to 5175)	951 (897 to 1008)	100 (77 to 127)	0 (0 to 0)	4887 (4513 to 5276)	3999 (3653 to 4373)	803 (758 to 851)	84 (65 to 108)	0 (0 to 0)
Iceland	2034	5826 (5366 to 6314)	4770 (4342 to 5229)	955 (902 to 1013)	101 (78 to 128)	0 (0 to 0)	4923 (4534 to 5336)	4031 (3669 to 4418)	807 (762 to 856)	85 (66 to 108)	0 (0 to 0)
Iceland	2035	5871 (5399 to 6351)	4810 (4371 to 5266)	960 (905 to 1019)	101 (78 to 129)	0 (0 to 0)	4961 (4562 to 5367)	4064 (3694 to 4449)	811 (765 to 861)	86 (66 to 109)	0 (0 to 0)
Iceland	2036	5920 (5432 to 6442)	4852 (4394 to 5328)	966 (909 to 1026)	102 (79 to 130)	0 (0 to 0)	5002 (4590 to 5443)	4100 (3713 to 4502)	816 (768 to 867)	87 (67 to 110)	0 (0 to 0)
Iceland	2037	5958 (5465 to 6489)	4885 (4420 to 5364)	970 (912 to 1032)	103 (80 to 131)	0 (0 to 0)	5035 (4618 to 5484)	4128 (3735 to 4533)	820 (771 to 872)	87 (67 to 111)	0 (0 to 0)

Iceland	2038	6002 (5494 to 6539)	4923 (4438 to 5423)	975 (915 to 1039)	104 (80 to 133)	0 (0 to 0)	5071 (4642 to 5525)	4160 (3750 to 4583)	824 (773 to 878)	88 (68 to 112)	0 (0 to 0)
Iceland	2039	6053 (5527 to 6603)	4967 (4481 to 5486)	980 (920 to 1047)	105 (81 to 134)	0 (0 to 0)	5115 (4670 to 5580)	4197 (3786 to 4636)	828 (777 to 884)	89 (68 to 113)	0 (0 to 0)
Iceland	2040	6106 (5566 to 6686)	5013 (4509 to 5548)	986 (924 to 1055)	106 (82 to 135)	0 (0 to 0)	5159 (4703 to 5650)	4236 (3810 to 4688)	834 (781 to 891)	90 (69 to 114)	0 (0 to 0)
Iceland	2041	6159 (5617 to 6747)	5059 (4556 to 5625)	993 (929 to 1062)	107 (82 to 136)	0 (0 to 0)	5204 (4747 to 5701)	4275 (3849 to 4753)	839 (785 to 898)	91 (70 to 115)	0 (0 to 0)
Iceland	2042	6214 (5658 to 6838)	5107 (4578 to 5699)	999 (934 to 1071)	108 (83 to 137)	0 (0 to 0)	5251 (4781 to 5778)	4315 (3868 to 4816)	844 (789 to 905)	92 (70 to 116)	0 (0 to 0)
Iceland	2043	6271 (5697 to 6929)	5156 (4626 to 5775)	1005 (939 to 1079)	109 (84 to 138)	0 (0 to 0)	5299 (4814 to 5855)	4357 (3909 to 4880)	849 (794 to 912)	92 (71 to 117)	0 (0 to 0)
Iceland	2044	6329 (5767 to 7021)	5206 (4668 to 5858)	1012 (945 to 1088)	111 (85 to 140)	0 (0 to 0)	5348 (4873 to 5932)	4399 (3944 to 4950)	855 (798 to 919)	93 (72 to 118)	0 (0 to 0)
Iceland	2045	6387 (5810 to 7080)	5257 (4703 to 5922)	1018 (948 to 1096)	112 (86 to 142)	0 (0 to 0)	5397 (4910 to 5982)	4442 (3974 to 5004)	860 (801 to 926)	94 (72 to 120)	0 (0 to 0)
Iceland	2046	6445 (5844 to 7141)	5308 (4732 to 5982)	1024 (954 to 1104)	113 (87 to 143)	0 (0 to 0)	5446 (4938 to 6034)	4485 (3999 to 5055)	865 (806 to 933)	95 (73 to 121)	0 (0 to 0)
Iceland	2047	6504 (5891 to 7229)	5359 (4774 to 6051)	1030 (958 to 1113)	114 (87 to 145)	0 (0 to 0)	5495 (4978 to 6108)	4528 (4034 to 5113)	871 (810 to 940)	96 (74 to 123)	0 (0 to 0)

Iceland	2048	6562 (5938 to 7287)	5410 (4818 to 6110)	1037 (963 to 1121)	115 (88 to 147)	0 (0 to 0)	5545 (5017 to 6158)	4571 (4071 to 5163)	876 (814 to 947)	97 (74 to 124)	0 (0 to 0)
Iceland	2049	6624 (5968 to 7387)	5465 (4848 to 6212)	1043 (967 to 1129)	116 (89 to 149)	0 (0 to 0)	5597 (5043 to 6242)	4618 (4097 to 5249)	881 (818 to 954)	98 (75 to 126)	0 (0 to 0)
Iceland	2050	6691 (6040 to 7448)	5522 (4905 to 6244)	1050 (973 to 1138)	118 (90 to 150)	0 (0 to 0)	5653 (5104 to 6293)	4666 (4144 to 5276)	888 (822 to 962)	100 (76 to 127)	0 (0 to 0)
India	1995	26 (20 to 34)	5 (4 to 6)	19 (14 to 27)	1 (1 to 3)	0 (0 to 0)	100 (78 to 129)	19 (14 to 24)	74 (53 to 102)	5 (2 to 10)	2 (2 to 2)
India	1996	27 (21 to 34)	5 (4 to 7)	20 (14 to 27)	1 (1 to 3)	1 (1 to 1)	104 (80 to 131)	19 (15 to 25)	76 (54 to 104)	6 (3 to 11)	3 (3 to 3)
India	1997	28 (22 to 36)	5 (4 to 7)	21 (15 to 28)	2 (1 to 3)	0 (0 to 0)	107 (83 to 138)	20 (16 to 27)	79 (57 to 108)	6 (3 to 11)	2 (2 to 2)
India	1998	29 (23 to 37)	6 (4 to 8)	21 (16 to 29)	2 (1 to 3)	0 (0 to 0)	112 (87 to 143)	22 (17 to 29)	82 (60 to 112)	6 (3 to 12)	1 (1 to 1)
India	1999	30 (24 to 38)	6 (5 to 8)	22 (16 to 30)	2 (1 to 3)	0 (0 to 0)	117 (91 to 148)	23 (17 to 30)	85 (62 to 116)	7 (3 to 12)	2 (2 to 2)
India	2000	31 (25 to 40)	6 (5 to 8)	23 (17 to 31)	2 (1 to 3)	1 (1 to 1)	120 (94 to 153)	24 (18 to 30)	88 (63 to 119)	7 (3 to 13)	2 (2 to 2)
India	2001	33 (26 to 41)	6 (5 to 8)	24 (17 to 32)	2 (1 to 4)	1 (1 to 1)	125 (99 to 158)	24 (18 to 31)	91 (66 to 124)	7 (3 to 14)	2 (2 to 2)
India	2002	33 (27 to 43)	6 (5 to 8)	24 (18 to 33)	2 (1 to 4)	1 (1 to 1)	128 (102 to 164)	25 (18 to 31)	93 (69 to 126)	8 (4 to 14)	3 (3 to 3)
India	2003	35 (27 to 44)	7 (5 to 9)	25 (18 to 34)	2 (1 to 4)	0 (0 to 0)	133 (105 to 168)	26 (19 to 33)	97 (70 to 130)	9 (4 to 16)	2 (2 to 2)
India	2004	35 (28 to 45)	7 (5 to 9)	25 (18 to 34)	2 (1 to 4)	1 (1 to 1)	135 (106 to 172)	26 (19 to 34)	97 (71 to 131)	9 (4 to 16)	3 (3 to 3)
India	2005	36 (29 to 46)	7 (5 to 9)	26 (19 to 35)	2 (1 to 4)	1 (1 to 1)	139 (110 to 177)	28 (21 to 36)	100 (73 to 134)	9 (4 to 16)	3 (3 to 3)

India	2006	37 (29 to 47)	8 (6 to 10)	27 (20 to 36)	2 (1 to 5)	1 (1 to 1)	144 (113 to 182)	30 (22 to 39)	103 (75 to 138)	9 (5 to 18)	2 (2 to 2)
India	2007	39 (31 to 49)	8 (6 to 11)	27 (20 to 37)	3 (1 to 5)	1 (1 to 1)	150 (119 to 188)	32 (24 to 42)	105 (77 to 141)	10 (5 to 19)	3 (3 to 3)
India	2008	41 (33 to 51)	9 (7 to 12)	28 (21 to 38)	3 (1 to 5)	1 (1 to 1)	158 (125 to 198)	36 (27 to 47)	108 (79 to 146)	11 (5 to 21)	3 (3 to 3)
India	2009	43 (34 to 53)	10 (8 to 13)	29 (21 to 39)	3 (2 to 6)	1 (1 to 1)	165 (131 to 205)	40 (30 to 52)	110 (81 to 150)	12 (6 to 23)	3 (3 to 3)
India	2010	45 (37 to 56)	11 (9 to 15)	30 (21 to 40)	3 (2 to 7)	1 (1 to 1)	173 (140 to 216)	44 (33 to 56)	113 (82 to 153)	13 (6 to 25)	3 (3 to 3)
India	2011	48 (39 to 60)	12 (9 to 16)	31 (23 to 41)	4 (2 to 7)	1 (1 to 1)	184 (149 to 229)	48 (36 to 62)	118 (87 to 159)	15 (7 to 28)	3 (3 to 3)
India	2012	50 (41 to 63)	13 (10 to 17)	33 (24 to 43)	4 (2 to 8)	1 (1 to 1)	193 (158 to 241)	50 (38 to 65)	125 (93 to 167)	16 (8 to 30)	2 (2 to 2)
India	2013	54 (44 to 68)	13 (10 to 18)	35 (26 to 48)	5 (2 to 9)	1 (1 to 1)	207 (169 to 259)	51 (39 to 67)	136 (100 to 183)	17 (8 to 33)	3 (3 to 3)
India	2014	57 (46 to 71)	14 (10 to 18)	37 (28 to 50)	5 (2 to 9)	0 (0 to 0)	218 (177 to 273)	54 (40 to 71)	143 (107 to 193)	19 (9 to 36)	2 (2 to 2)
India	2015	61 (50 to 77)	15 (12 to 20)	39 (29 to 54)	6 (3 to 10)	1 (1 to 1)	234 (190 to 294)	59 (44 to 77)	150 (112 to 205)	22 (10 to 40)	2 (2 to 2)
India	2016	65 (53 to 82)	17 (13 to 22)	41 (31 to 57)	6 (3 to 11)	1 (1 to 1)	249 (202 to 314)	65 (48 to 85)	158 (118 to 217)	24 (12 to 43)	2 (2 to 2)
India	2017	69 (54 to 87)	18 (13 to 24)	43 (32 to 60)	7 (3 to 12)	1 (1 to 1)	265 (209 to 336)	70 (51 to 91)	166 (123 to 230)	26 (13 to 48)	2 (2 to 2)
India	2018	73 (57 to 93)	19 (14 to 25)	45 (34 to 63)	7 (4 to 13)	1 (1 to 1)	278 (219 to 359)	74 (54 to 96)	174 (129 to 242)	28 (14 to 50)	2 (2 to 2)
India	2019	77 (60 to 98)	21 (15 to 28)	47 (35 to 66)	8 (4 to 14)	1 (1 to 1)	295 (231 to 374)	81 (59 to 106)	182 (135 to 252)	30 (15 to 54)	2 (2 to 2)
India	2020	81 (64 to 104)	22 (16 to 30)	50 (37 to 69)	8 (4 to 15)	1 (1 to 1)	311 (245 to 400)	86 (63 to 113)	191 (142 to 265)	32 (16 to 57)	2 (2 to 2)

India	2021	84 (66 to 107)	24 (17 to 31)	52 (38 to 71)	9 (4 to 16)	1 (1 to 1)	324 (255 to 411)	91 (66 to 119)	198 (146 to 274)	33 (16 to 60)	2 (2 to 2)
India	2022	88 (69 to 112)	25 (18 to 33)	53 (39 to 74)	9 (4 to 16)	1 (1 to 1)	337 (266 to 430)	95 (69 to 125)	205 (151 to 284)	35 (17 to 63)	2 (2 to 3)
India	2023	91 (72 to 115)	26 (19 to 34)	55 (41 to 77)	9 (5 to 17)	1 (1 to 1)	350 (276 to 443)	100 (73 to 131)	212 (156 to 294)	36 (18 to 66)	2 (2 to 3)
India	2024	95 (74 to 121)	27 (20 to 36)	57 (42 to 79)	10 (5 to 18)	1 (1 to 1)	363 (285 to 464)	104 (76 to 138)	219 (162 to 303)	38 (19 to 69)	2 (2 to 3)
India	2025	98 (77 to 124)	28 (21 to 37)	59 (44 to 82)	10 (5 to 19)	1 (1 to 1)	377 (297 to 477)	109 (79 to 143)	226 (167 to 313)	39 (19 to 72)	2 (2 to 3)
India	2026	102 (80 to 131)	30 (22 to 39)	61 (45 to 84)	11 (5 to 20)	1 (1 to 1)	391 (306 to 503)	114 (84 to 150)	233 (173 to 324)	41 (20 to 75)	2 (2 to 3)
India	2027	106 (83 to 134)	31 (23 to 41)	63 (47 to 87)	11 (6 to 20)	1 (1 to 1)	406 (320 to 515)	120 (88 to 158)	241 (179 to 334)	43 (21 to 78)	2 (2 to 3)
India	2028	110 (87 to 141)	33 (24 to 43)	65 (48 to 90)	12 (6 to 21)	1 (1 to 1)	422 (333 to 541)	126 (92 to 166)	249 (184 to 345)	45 (22 to 82)	2 (2 to 3)
India	2029	114 (89 to 144)	34 (25 to 46)	67 (50 to 93)	12 (6 to 22)	1 (1 to 1)	439 (343 to 553)	132 (97 to 175)	257 (190 to 357)	47 (23 to 85)	2 (2 to 3)
India	2030	119 (94 to 152)	36 (26 to 48)	69 (51 to 96)	13 (6 to 23)	1 (1 to 1)	456 (360 to 583)	139 (102 to 183)	266 (196 to 368)	49 (24 to 89)	2 (2 to 3)
India	2031	123 (96 to 156)	38 (28 to 50)	72 (53 to 99)	13 (7 to 24)	1 (1 to 1)	473 (370 to 597)	145 (107 to 192)	274 (203 to 379)	51 (26 to 93)	2 (2 to 3)
India	2032	128 (100 to 162)	40 (29 to 52)	74 (55 to 102)	14 (7 to 25)	1 (1 to 1)	492 (386 to 623)	152 (111 to 201)	283 (210 to 391)	53 (27 to 97)	2 (2 to 3)
India	2033	133 (103 to 170)	42 (31 to 55)	76 (56 to 105)	15 (7 to 26)	1 (1 to 1)	511 (395 to 651)	160 (117 to 212)	292 (217 to 404)	56 (28 to 101)	2 (2 to 3)
India	2034	138 (108 to 175)	44 (32 to 58)	79 (58 to 109)	15 (8 to 28)	1 (1 to 1)	531 (415 to 672)	168 (123 to 221)	302 (224 to 417)	58 (29 to 106)	2 (2 to 3)
India	2035	144 (112 to 181)	46 (34 to 61)	81 (60 to 112)	16 (8 to 29)	1 (1 to 1)	551 (429 to 696)	177 (129 to 234)	311 (231 to 430)	61 (30 to 111)	2 (2 to 3)

India	2036	149 (117 to 189)	48 (36 to 64)	83 (62 to 116)	17 (8 to 30)	1 (1 to 1)	572 (447 to 725)	186 (137 to 245)	320 (238 to 444)	64 (32 to 116)	3 (2 to 3)
India	2037	155 (120 to 197)	51 (37 to 67)	86 (64 to 119)	17 (9 to 32)	1 (1 to 1)	593 (461 to 756)	195 (143 to 256)	330 (245 to 457)	66 (33 to 121)	3 (2 to 3)
India	2038	160 (125 to 203)	53 (39 to 70)	88 (66 to 122)	18 (9 to 33)	1 (1 to 1)	615 (480 to 780)	204 (150 to 270)	339 (252 to 470)	69 (34 to 126)	3 (2 to 3)
India	2039	166 (130 to 211)	56 (41 to 74)	91 (67 to 126)	19 (9 to 34)	1 (1 to 1)	637 (497 to 808)	214 (156 to 282)	349 (259 to 483)	72 (36 to 131)	3 (2 to 3)
India	2040	172 (135 to 218)	58 (43 to 77)	93 (69 to 129)	20 (10 to 36)	1 (1 to 1)	660 (519 to 836)	224 (163 to 297)	358 (266 to 497)	75 (37 to 137)	3 (2 to 3)
India	2041	178 (139 to 226)	61 (45 to 81)	96 (71 to 133)	20 (10 to 37)	1 (1 to 1)	685 (533 to 867)	235 (172 to 311)	368 (273 to 511)	78 (39 to 143)	3 (2 to 3)
India	2042	185 (145 to 234)	64 (47 to 85)	99 (73 to 137)	21 (11 to 39)	1 (1 to 1)	709 (557 to 899)	247 (179 to 326)	378 (280 to 525)	82 (41 to 149)	3 (2 to 4)
India	2043	191 (149 to 242)	67 (49 to 89)	101 (75 to 141)	22 (11 to 41)	1 (1 to 1)	735 (572 to 930)	259 (189 to 341)	389 (288 to 540)	85 (42 to 155)	3 (2 to 4)
India	2044	198 (155 to 251)	71 (51 to 93)	104 (77 to 144)	23 (12 to 42)	1 (1 to 1)	761 (597 to 965)	271 (197 to 356)	399 (296 to 554)	88 (44 to 163)	3 (2 to 4)
India	2045	205 (159 to 262)	74 (53 to 97)	107 (79 to 148)	24 (12 to 44)	1 (1 to 1)	788 (610 to 1005)	284 (205 to 372)	409 (303 to 568)	92 (46 to 169)	3 (2 to 4)
India	2046	213 (166 to 271)	77 (56 to 102)	109 (81 to 152)	25 (12 to 46)	1 (1 to 1)	815 (638 to 1039)	297 (216 to 390)	420 (311 to 583)	96 (48 to 176)	3 (2 to 4)
India	2047	220 (170 to 279)	81 (59 to 106)	112 (83 to 156)	26 (13 to 48)	1 (1 to 1)	844 (653 to 1071)	311 (225 to 408)	430 (319 to 598)	99 (49 to 183)	3 (2 to 4)
India	2048	228 (179 to 290)	85 (61 to 111)	115 (85 to 160)	27 (13 to 49)	1 (1 to 1)	874 (686 to 1112)	326 (236 to 427)	441 (326 to 614)	103 (51 to 190)	3 (2 to 4)
India	2049	235 (182 to 300)	89 (64 to 116)	118 (87 to 164)	28 (14 to 51)	1 (1 to 1)	903 (697 to 1153)	341 (247 to 444)	452 (334 to 629)	108 (53 to 197)	3 (2 to 4)
India	2050	244 (192 to 312)	93 (68 to 122)	121 (89 to 168)	29 (15 to 54)	1 (1 to 1)	935 (735 to 1196)	357 (260 to 469)	463 (342 to 644)	112 (56 to 206)	3 (2 to 4)

Indonesia	1995	40 (32 to 50)	11 (8 to 14)	17 (12 to 22)	12 (6 to 20)	1 (1 to 1)	135 (108 to 169)	37 (28 to 47)	55 (40 to 76)	39 (21 to 67)	4 (4 to 4)
Indonesia	1996	41 (33 to 52)	11 (9 to 14)	17 (12 to 24)	12 (7 to 21)	1 (1 to 1)	139 (110 to 175)	38 (30 to 49)	58 (42 to 80)	41 (22 to 70)	2 (2 to 2)
Indonesia	1997	45 (36 to 57)	13 (10 to 16)	18 (14 to 25)	14 (7 to 23)	1 (1 to 1)	152 (121 to 190)	42 (33 to 54)	62 (46 to 85)	45 (24 to 77)	2 (2 to 2)
Indonesia	1998	41 (33 to 52)	11 (9 to 14)	18 (13 to 24)	12 (6 to 20)	1 (1 to 1)	139 (111 to 174)	38 (30 to 48)	59 (43 to 79)	40 (22 to 68)	2 (2 to 2)
Indonesia	1999	41 (33 to 51)	12 (9 to 15)	18 (13 to 24)	11 (6 to 19)	1 (1 to 1)	139 (111 to 173)	39 (30 to 49)	60 (44 to 80)	38 (21 to 65)	2 (2 to 2)
Indonesia	2000	41 (33 to 50)	12 (9 to 15)	18 (13 to 23)	10 (5 to 17)	1 (1 to 1)	137 (109 to 168)	40 (31 to 51)	59 (43 to 79)	34 (18 to 57)	4 (4 to 4)
Indonesia	2001	46 (37 to 56)	15 (11 to 19)	19 (14 to 26)	11 (6 to 19)	1 (1 to 1)	155 (125 to 188)	50 (38 to 63)	65 (48 to 87)	36 (19 to 63)	3 (3 to 3)
Indonesia	2002	44 (36 to 54)	14 (11 to 18)	20 (15 to 26)	10 (5 to 16)	1 (1 to 1)	149 (121 to 182)	48 (37 to 61)	67 (49 to 88)	32 (16 to 55)	2 (2 to 2)
Indonesia	2003	49 (40 to 59)	16 (12 to 20)	22 (17 to 29)	10 (5 to 16)	1 (1 to 1)	164 (134 to 199)	54 (41 to 68)	75 (56 to 98)	33 (16 to 55)	3 (3 to 3)
Indonesia	2004	52 (42 to 63)	17 (13 to 21)	24 (18 to 32)	10 (5 to 17)	1 (1 to 1)	174 (142 to 210)	56 (43 to 71)	82 (62 to 107)	33 (17 to 56)	3 (3 to 3)
Indonesia	2005	56 (47 to 67)	18 (14 to 22)	28 (21 to 37)	10 (5 to 17)	1 (1 to 1)	189 (158 to 226)	59 (46 to 74)	94 (70 to 123)	33 (17 to 56)	3 (3 to 3)
Indonesia	2006	63 (53 to 76)	20 (16 to 26)	31 (24 to 41)	11 (5 to 18)	1 (1 to 1)	213 (178 to 256)	67 (52 to 86)	105 (80 to 138)	36 (18 to 62)	5 (5 to 5)
Indonesia	2007	69 (58 to 83)	22 (17 to 28)	34 (26 to 44)	11 (6 to 20)	1 (1 to 1)	231 (194 to 278)	75 (58 to 95)	115 (88 to 149)	38 (20 to 68)	4 (4 to 4)
Indonesia	2008	75 (63 to 91)	24 (19 to 31)	37 (28 to 48)	13 (7 to 22)	2 (2 to 2)	253 (211 to 305)	82 (63 to 104)	124 (94 to 161)	42 (22 to 75)	5 (5 to 5)
Indonesia	2009	76 (62 to 92)	23 (18 to 29)	38 (29 to 50)	12 (7 to 21)	1 (1 to 1)	254 (210 to 309)	79 (61 to 99)	129 (97 to 168)	42 (22 to 72)	4 (4 to 4)

Indonesia	2010	82 (67 to 99)	24 (19 to 30)	43 (32 to 56)	14 (7 to 23)	1 (1 to 1)	275 (226 to 333)	81 (63 to 102)	144 (109 to 188)	46 (24 to 77)	4 (4 to 4)
Indonesia	2011	90 (74 to 109)	27 (21 to 34)	47 (35 to 61)	15 (8 to 25)	1 (1 to 1)	304 (249 to 366)	91 (71 to 115)	157 (119 to 206)	51 (27 to 85)	4 (4 to 4)
Indonesia	2012	97 (80 to 117)	30 (24 to 38)	48 (37 to 63)	17 (9 to 28)	1 (1 to 1)	325 (268 to 392)	102 (79 to 128)	162 (123 to 210)	57 (31 to 95)	4 (4 to 4)
Indonesia	2013	103 (85 to 123)	34 (26 to 43)	49 (37 to 62)	19 (10 to 32)	1 (1 to 1)	345 (285 to 413)	114 (89 to 143)	163 (124 to 210)	64 (34 to 108)	5 (5 to 5)
Indonesia	2014	108 (89 to 129)	38 (30 to 48)	48 (37 to 63)	21 (11 to 35)	1 (1 to 1)	361 (300 to 435)	129 (100 to 160)	161 (123 to 211)	69 (37 to 118)	3 (3 to 3)
Indonesia	2015	111 (92 to 134)	42 (33 to 53)	47 (35 to 61)	21 (11 to 35)	1 (1 to 1)	372 (310 to 451)	143 (111 to 177)	158 (119 to 206)	69 (37 to 118)	3 (3 to 3)
Indonesia	2016	116 (95 to 141)	48 (36 to 59)	46 (34 to 62)	21 (11 to 36)	1 (1 to 1)	389 (319 to 472)	160 (122 to 199)	156 (115 to 207)	71 (37 to 122)	3 (3 to 3)
Indonesia	2017	120 (94 to 152)	52 (40 to 65)	46 (34 to 62)	21 (11 to 38)	1 (1 to 1)	405 (316 to 511)	175 (134 to 219)	154 (113 to 209)	72 (37 to 127)	3 (3 to 3)
Indonesia	2018	127 (99 to 163)	56 (43 to 71)	47 (35 to 64)	23 (12 to 40)	1 (1 to 1)	427 (334 to 546)	188 (145 to 237)	159 (117 to 216)	77 (40 to 135)	3 (3 to 3)
Indonesia	2019	132 (103 to 167)	58 (45 to 74)	49 (36 to 67)	24 (12 to 42)	1 (1 to 1)	444 (346 to 562)	196 (150 to 248)	165 (121 to 224)	80 (42 to 141)	3 (3 to 3)
Indonesia	2020	139 (108 to 178)	62 (48 to 78)	51 (38 to 69)	25 (13 to 44)	1 (1 to 1)	466 (362 to 599)	208 (160 to 263)	172 (126 to 233)	84 (44 to 147)	3 (3 to 3)
Indonesia	2021	145 (113 to 183)	65 (50 to 82)	52 (39 to 71)	26 (13 to 46)	1 (1 to 1)	485 (378 to 613)	218 (167 to 276)	176 (130 to 239)	88 (45 to 153)	3 (3 to 3)
Indonesia	2022	150 (116 to 193)	68 (52 to 87)	54 (40 to 73)	27 (14 to 48)	1 (1 to 1)	505 (391 to 650)	230 (176 to 291)	181 (133 to 245)	91 (47 to 160)	3 (3 to 3)
Indonesia	2023	157 (122 to 200)	72 (55 to 91)	55 (41 to 75)	28 (15 to 50)	1 (1 to 1)	527 (409 to 673)	243 (186 to 307)	186 (137 to 253)	95 (49 to 167)	3 (3 to 4)
Indonesia	2024	165 (127 to 214)	77 (59 to 97)	57 (42 to 77)	30 (15 to 52)	1 (1 to 1)	553 (428 to 720)	258 (198 to 327)	191 (141 to 259)	100 (52 to 176)	3 (3 to 4)

Indonesia	2025	173 (133 to 217)	82 (63 to 104)	58 (43 to 79)	31 (16 to 55)	1 (1 to 1)	579 (448 to 728)	275 (211 to 349)	196 (144 to 265)	105 (54 to 185)	3 (3 to 4)
Indonesia	2026	181 (140 to 235)	87 (67 to 110)	60 (44 to 81)	33 (17 to 58)	1 (1 to 1)	607 (470 to 789)	292 (224 to 370)	201 (148 to 272)	110 (57 to 193)	3 (3 to 4)
Indonesia	2027	189 (146 to 238)	92 (71 to 117)	61 (45 to 83)	34 (18 to 60)	1 (1 to 1)	634 (491 to 799)	310 (238 to 392)	206 (151 to 279)	115 (60 to 202)	3 (3 to 4)
Indonesia	2028	197 (152 to 255)	98 (75 to 124)	63 (46 to 85)	36 (19 to 63)	1 (1 to 1)	662 (511 to 857)	327 (252 to 415)	211 (155 to 285)	120 (63 to 212)	3 (3 to 4)
Indonesia	2029	206 (158 to 261)	103 (79 to 130)	64 (47 to 87)	37 (20 to 66)	1 (1 to 1)	691 (530 to 877)	346 (267 to 437)	216 (158 to 292)	126 (66 to 221)	3 (3 to 4)
Indonesia	2030	215 (166 to 277)	109 (84 to 138)	66 (48 to 89)	39 (21 to 69)	1 (1 to 1)	722 (558 to 931)	367 (282 to 464)	220 (161 to 298)	131 (69 to 231)	3 (3 to 4)
Indonesia	2031	225 (174 to 287)	116 (89 to 146)	67 (49 to 91)	41 (21 to 72)	1 (1 to 1)	754 (584 to 964)	388 (298 to 491)	225 (165 to 304)	137 (72 to 241)	3 (3 to 4)
Indonesia	2032	234 (182 to 301)	122 (93 to 155)	68 (50 to 92)	43 (22 to 75)	1 (1 to 1)	786 (611 to 1010)	410 (314 to 520)	230 (168 to 310)	143 (75 to 251)	3 (3 to 4)
Indonesia	2033	244 (188 to 312)	129 (98 to 163)	70 (51 to 94)	44 (23 to 78)	1 (1 to 1)	819 (630 to 1049)	432 (331 to 548)	235 (172 to 316)	149 (78 to 262)	4 (3 to 4)
Indonesia	2034	253 (196 to 329)	135 (103 to 171)	71 (52 to 96)	46 (24 to 81)	1 (1 to 1)	851 (657 to 1106)	454 (347 to 575)	239 (175 to 322)	155 (81 to 272)	4 (3 to 4)
Indonesia	2035	263 (201 to 339)	142 (108 to 181)	73 (53 to 98)	48 (25 to 84)	1 (1 to 1)	884 (675 to 1138)	476 (364 to 607)	244 (179 to 329)	161 (84 to 282)	4 (3 to 4)
Indonesia	2036	273 (208 to 358)	148 (113 to 190)	74 (54 to 100)	50 (26 to 87)	1 (1 to 1)	917 (700 to 1201)	498 (378 to 639)	248 (182 to 335)	166 (87 to 292)	4 (3 to 5)
Indonesia	2037	283 (215 to 368)	155 (117 to 198)	75 (55 to 102)	51 (27 to 90)	1 (1 to 1)	950 (721 to 1235)	521 (393 to 667)	253 (185 to 341)	172 (90 to 302)	4 (3 to 5)
Indonesia	2038	293 (224 to 384)	162 (122 to 208)	77 (56 to 103)	53 (28 to 93)	1 (1 to 1)	983 (751 to 1290)	544 (410 to 700)	257 (189 to 347)	178 (94 to 313)	4 (3 to 5)
Indonesia	2039	303 (230 to 395)	169 (127 to 218)	78 (57 to 105)	55 (29 to 96)	1 (1 to 1)	1018 (774 to 1327)	567 (426 to 731)	262 (192 to 353)	184 (97 to 323)	4 (3 to 5)

Indonesia	2040	314 (236 to 412)	176 (131 to 228)	80 (58 to 107)	57 (30 to 100)	1 (1 to 1)	1053 (793 to 1385)	591 (441 to 765)	267 (196 to 360)	191 (100 to 334)	4 (3 to 5)
Indonesia	2041	324 (245 to 424)	183 (136 to 238)	81 (59 to 109)	59 (31 to 103)	1 (1 to 2)	1089 (822 to 1425)	616 (457 to 799)	272 (199 to 367)	197 (104 to 346)	4 (3 to 5)
Indonesia	2042	335 (251 to 445)	191 (141 to 248)	83 (60 to 112)	61 (32 to 107)	1 (1 to 2)	1126 (842 to 1494)	641 (474 to 831)	278 (203 to 375)	204 (107 to 358)	4 (3 to 5)
Indonesia	2043	346 (261 to 453)	198 (146 to 258)	84 (62 to 114)	63 (33 to 110)	1 (1 to 2)	1163 (876 to 1521)	665 (490 to 868)	283 (207 to 382)	210 (111 to 370)	4 (3 to 5)
Indonesia	2044	357 (266 to 466)	206 (151 to 269)	86 (63 to 116)	65 (34 to 114)	1 (1 to 2)	1200 (892 to 1565)	690 (506 to 902)	288 (211 to 390)	217 (115 to 382)	4 (3 to 6)
Indonesia	2045	369 (277 to 485)	213 (156 to 278)	88 (64 to 118)	67 (35 to 117)	1 (1 to 2)	1238 (931 to 1628)	716 (524 to 935)	294 (215 to 398)	224 (118 to 393)	4 (3 to 6)
Indonesia	2046	380 (281 to 499)	221 (161 to 289)	89 (66 to 121)	69 (36 to 120)	1 (1 to 2)	1276 (943 to 1675)	741 (540 to 969)	300 (220 to 406)	230 (122 to 404)	4 (3 to 6)
Indonesia	2047	391 (291 to 521)	228 (166 to 298)	91 (67 to 124)	71 (37 to 124)	1 (1 to 2)	1313 (977 to 1749)	766 (556 to 1002)	306 (224 to 415)	237 (126 to 415)	4 (3 to 6)
Indonesia	2048	403 (296 to 534)	236 (170 to 309)	93 (68 to 126)	73 (39 to 127)	1 (1 to 2)	1352 (992 to 1793)	791 (573 to 1037)	313 (229 to 424)	244 (129 to 427)	4 (4 to 6)
Indonesia	2049	414 (311 to 549)	243 (175 to 319)	95 (70 to 129)	75 (40 to 131)	1 (1 to 2)	1392 (1046 to 1844)	817 (589 to 1070)	319 (234 to 434)	251 (133 to 439)	5 (4 to 6)

Indonesia	2050	427 (310 to 565)	251 (181 to 330)	97 (71 to 132)	77 (41 to 135)	1 (1 to 2)	1433 (1040 to 1898)	844 (607 to 1110)	326 (239 to 444)	259 (137 to 453)	5 (4 to 6)
Iran	1995	167 (145 to 192)	68 (56 to 81)	95 (77 to 115)	5 (2 to 9)	0 (0 to 0)	532 (460 to 609)	215 (179 to 257)	301 (245 to 366)	16 (8 to 28)	0 (0 to 0)
Iran	1996	169 (148 to 192)	69 (58 to 81)	95 (79 to 113)	5 (2 to 9)	1 (1 to 1)	537 (470 to 609)	218 (184 to 259)	302 (252 to 360)	16 (8 to 28)	2 (2 to 2)
Iran	1997	179 (158 to 199)	73 (61 to 85)	100 (84 to 117)	6 (3 to 10)	1 (1 to 1)	569 (504 to 634)	231 (195 to 271)	318 (267 to 373)	18 (9 to 31)	2 (2 to 2)
Iran	1998	189 (168 to 209)	76 (64 to 88)	107 (91 to 124)	6 (3 to 10)	0 (0 to 0)	600 (535 to 666)	241 (205 to 281)	339 (290 to 394)	19 (10 to 33)	1 (1 to 1)
Iran	1999	197 (177 to 217)	76 (65 to 89)	114 (98 to 131)	7 (3 to 11)	0 (0 to 0)	627 (562 to 690)	243 (207 to 282)	362 (312 to 416)	21 (11 to 36)	1 (1 to 1)
Iran	2000	202 (182 to 222)	77 (66 to 88)	119 (103 to 136)	7 (4 to 11)	0 (0 to 0)	644 (580 to 707)	244 (210 to 281)	378 (327 to 432)	21 (11 to 36)	0 (0 to 0)
Iran	2001	211 (191 to 233)	80 (70 to 92)	124 (108 to 141)	7 (4 to 12)	0 (0 to 0)	672 (607 to 739)	254 (221 to 293)	394 (343 to 448)	24 (13 to 39)	1 (1 to 1)
Iran	2002	242 (220 to 266)	95 (83 to 109)	136 (119 to 154)	11 (6 to 18)	0 (0 to 0)	769 (700 to 847)	301 (263 to 347)	433 (380 to 491)	35 (19 to 56)	0 (0 to 0)
Iran	2003	259 (237 to 285)	101 (88 to 116)	145 (128 to 164)	13 (8 to 21)	0 (0 to 0)	824 (752 to 907)	320 (278 to 368)	461 (406 to 523)	43 (24 to 67)	0 (0 to 0)
Iran	2004	263 (239 to 288)	99 (86 to 113)	149 (131 to 168)	15 (9 to 23)	0 (0 to 0)	835 (761 to 915)	315 (275 to 359)	474 (417 to 533)	47 (28 to 73)	0 (0 to 0)
Iran	2005	292 (267 to 319)	113 (99 to 127)	161 (142 to 181)	18 (11 to 29)	1 (1 to 1)	929 (849 to 1015)	358 (315 to 405)	512 (451 to 575)	56 (34 to 91)	3 (3 to 3)
Iran	2006	313 (287 to 342)	124 (109 to 139)	170 (150 to 190)	20 (12 to 32)	0 (0 to 0)	996 (913 to 1086)	393 (348 to 442)	539 (478 to 603)	63 (38 to 101)	1 (1 to 1)
Iran	2007	313 (288 to 341)	119 (106 to 133)	174 (155 to 194)	20 (12 to 33)	0 (0 to 0)	996 (914 to 1083)	378 (335 to 424)	552 (491 to 617)	65 (40 to 104)	0 (0 to 0)

Iran	2008	348 (318 to 378)	130 (116 to 145)	193 (172 to 216)	25 (15 to 39)	0 (0 to 0)	1107 (1011 to 1203)	415 (368 to 462)	614 (547 to 686)	78 (48 to 125)	1 (1 to 1)
Iran	2009	372 (341 to 406)	134 (119 to 150)	210 (187 to 234)	28 (18 to 44)	0 (0 to 0)	1184 (1085 to 1289)	426 (379 to 476)	668 (596 to 744)	90 (56 to 140)	1 (1 to 1)
Iran	2010	395 (364 to 432)	139 (124 to 156)	223 (200 to 249)	33 (21 to 49)	0 (0 to 0)	1257 (1156 to 1375)	443 (393 to 496)	710 (635 to 790)	103 (65 to 155)	0 (0 to 0)
Iran	2011	418 (385 to 455)	152 (135 to 170)	228 (204 to 255)	37 (24 to 55)	0 (0 to 0)	1329 (1224 to 1448)	484 (430 to 540)	726 (647 to 809)	119 (75 to 175)	1 (1 to 1)
Iran	2012	366 (338 to 398)	134 (120 to 150)	198 (177 to 222)	33 (21 to 48)	0 (0 to 0)	1162 (1076 to 1267)	427 (380 to 476)	631 (563 to 705)	104 (67 to 154)	1 (1 to 1)
Iran	2013	371 (345 to 404)	151 (135 to 167)	188 (167 to 210)	33 (22 to 48)	0 (0 to 0)	1181 (1096 to 1283)	479 (428 to 532)	597 (531 to 668)	104 (69 to 153)	0 (0 to 0)
Iran	2014	406 (376 to 440)	185 (167 to 204)	188 (168 to 212)	33 (21 to 50)	0 (0 to 0)	1291 (1195 to 1400)	587 (529 to 650)	598 (536 to 673)	105 (67 to 158)	0 (0 to 0)
Iran	2015	454 (418 to 494)	222 (200 to 245)	197 (175 to 223)	35 (22 to 53)	0 (0 to 0)	1442 (1328 to 1569)	704 (635 to 778)	627 (557 to 709)	111 (70 to 167)	0 (0 to 0)
Iran	2016	528 (487 to 576)	268 (240 to 298)	220 (193 to 250)	40 (25 to 62)	0 (0 to 0)	1679 (1548 to 1830)	853 (762 to 947)	700 (614 to 793)	126 (78 to 197)	0 (0 to 0)
Iran	2017	555 (495 to 618)	285 (251 to 322)	229 (197 to 264)	41 (24 to 65)	0 (0 to 0)	1763 (1574 to 1966)	907 (798 to 1023)	727 (628 to 841)	129 (77 to 205)	0 (0 to 0)

Iran	2018	516 (461 to 577)	262 (231 to 295)	217 (187 to 251)	38 (23 to 60)	0 (0 to 0)	1641 (1465 to 1835)	832 (734 to 936)	689 (595 to 798)	120 (72 to 191)	0 (0 to 0)
Iran	2019	460 (410 to 515)	230 (202 to 261)	196 (169 to 227)	34 (20 to 54)	0 (0 to 0)	1462 (1303 to 1637)	731 (642 to 829)	624 (538 to 722)	107 (64 to 170)	0 (0 to 0)
Iran	2020	462 (410 to 515)	233 (203 to 264)	195 (168 to 226)	34 (20 to 54)	0 (0 to 0)	1470 (1305 to 1639)	741 (646 to 839)	620 (535 to 717)	108 (64 to 172)	0 (0 to 0)
Iran	2021	471 (420 to 527)	241 (211 to 272)	195 (169 to 226)	35 (21 to 55)	0 (0 to 0)	1498 (1336 to 1675)	767 (670 to 865)	620 (536 to 717)	110 (66 to 176)	0 (0 to 0)
Iran	2022	481 (429 to 537)	250 (218 to 282)	196 (169 to 227)	35 (21 to 56)	0 (0 to 0)	1530 (1364 to 1708)	795 (694 to 896)	622 (536 to 721)	113 (67 to 180)	0 (0 to 0)
Iran	2023	492 (438 to 549)	259 (226 to 291)	196 (169 to 227)	36 (22 to 58)	0 (0 to 0)	1563 (1394 to 1745)	825 (720 to 926)	623 (538 to 723)	115 (69 to 184)	0 (0 to 0)
Iran	2024	503 (448 to 560)	269 (235 to 302)	197 (170 to 228)	37 (22 to 59)	0 (0 to 0)	1600 (1425 to 1780)	856 (747 to 961)	625 (539 to 726)	118 (70 to 188)	0 (0 to 0)
Iran	2025	515 (459 to 572)	279 (243 to 313)	197 (170 to 229)	38 (23 to 61)	0 (0 to 0)	1637 (1459 to 1819)	888 (774 to 996)	627 (539 to 729)	121 (72 to 193)	0 (0 to 0)
Iran	2026	527 (470 to 588)	290 (254 to 325)	198 (170 to 230)	39 (23 to 62)	0 (0 to 0)	1676 (1493 to 1869)	923 (807 to 1032)	629 (540 to 730)	124 (73 to 198)	0 (0 to 0)
Iran	2027	540 (480 to 602)	301 (264 to 337)	198 (170 to 230)	40 (24 to 64)	0 (0 to 0)	1716 (1526 to 1915)	958 (839 to 1070)	631 (541 to 732)	127 (75 to 203)	0 (0 to 0)

Iran	2028	552 (493 to 619)	312 (274 to 349)	199 (171 to 231)	41 (24 to 65)	0 (0 to 0)	1756 (1566 to 1967)	993 (871 to 1109)	633 (543 to 733)	130 (77 to 208)	0 (0 to 0)
Iran	2029	567 (505 to 633)	325 (285 to 362)	200 (172 to 232)	42 (25 to 67)	0 (0 to 0)	1802 (1604 to 2011)	1032 (906 to 1151)	636 (545 to 738)	133 (78 to 213)	0 (0 to 0)
Iran	2030	580 (516 to 648)	336 (295 to 375)	201 (172 to 233)	43 (25 to 69)	0 (0 to 0)	1845 (1642 to 2061)	1069 (937 to 1191)	640 (548 to 742)	136 (80 to 218)	0 (0 to 0)
Iran	2031	593 (527 to 663)	347 (305 to 387)	202 (173 to 235)	44 (26 to 70)	0 (0 to 0)	1887 (1677 to 2109)	1105 (970 to 1231)	643 (550 to 746)	139 (82 to 224)	0 (0 to 0)
Iran	2032	606 (539 to 677)	359 (315 to 401)	203 (173 to 236)	45 (26 to 72)	0 (0 to 0)	1928 (1713 to 2153)	1140 (1001 to 1274)	645 (551 to 749)	142 (84 to 228)	0 (0 to 0)
Iran	2033	619 (549 to 691)	369 (324 to 414)	204 (173 to 237)	46 (27 to 73)	0 (0 to 0)	1966 (1746 to 2198)	1174 (1029 to 1315)	647 (551 to 754)	145 (85 to 233)	0 (0 to 0)
Iran	2034	631 (560 to 704)	380 (333 to 425)	204 (173 to 237)	46 (27 to 75)	0 (0 to 0)	2006 (1782 to 2237)	1208 (1059 to 1350)	649 (552 to 755)	148 (87 to 237)	0 (0 to 0)
Iran	2035	643 (571 to 718)	391 (343 to 437)	205 (174 to 238)	47 (28 to 76)	0 (0 to 0)	2044 (1814 to 2284)	1242 (1091 to 1390)	652 (553 to 757)	150 (89 to 241)	0 (0 to 0)
Iran	2036	654 (580 to 729)	400 (352 to 447)	205 (174 to 239)	48 (28 to 77)	0 (0 to 0)	2078 (1843 to 2319)	1272 (1119 to 1423)	652 (553 to 759)	153 (90 to 245)	0 (0 to 1)
Iran	2037	663 (586 to 741)	409 (360 to 457)	205 (173 to 240)	49 (29 to 78)	0 (0 to 0)	2107 (1864 to 2355)	1300 (1144 to 1454)	652 (551 to 762)	155 (91 to 249)	0 (0 to 1)

Iran	2038	672 (596 to 752)	417 (367 to 467)	205 (173 to 241)	49 (29 to 79)	0 (0 to 0)	2136 (1893 to 2391)	1326 (1166 to 1485)	652 (551 to 766)	157 (92 to 252)	0 (0 to 1)
Iran	2039	680 (603 to 762)	425 (374 to 475)	205 (173 to 240)	50 (29 to 80)	0 (0 to 0)	2162 (1918 to 2423)	1351 (1188 to 1511)	652 (551 to 764)	159 (93 to 255)	0 (0 to 1)
Iran	2040	688 (611 to 771)	432 (380 to 484)	205 (173 to 241)	50 (29 to 81)	0 (0 to 0)	2187 (1942 to 2450)	1375 (1207 to 1538)	652 (550 to 766)	160 (94 to 259)	0 (0 to 1)
Iran	2041	694 (614 to 779)	439 (385 to 491)	204 (173 to 241)	51 (30 to 82)	0 (0 to 0)	2207 (1952 to 2477)	1394 (1225 to 1560)	650 (549 to 767)	162 (94 to 261)	0 (0 to 1)
Iran	2042	700 (618 to 785)	445 (391 to 498)	204 (172 to 241)	51 (30 to 83)	0 (0 to 0)	2225 (1966 to 2495)	1414 (1242 to 1584)	648 (547 to 767)	163 (95 to 263)	0 (0 to 1)
Iran	2043	705 (624 to 791)	450 (395 to 504)	203 (171 to 241)	52 (30 to 84)	0 (0 to 0)	2241 (1982 to 2514)	1431 (1257 to 1603)	645 (543 to 766)	164 (95 to 266)	0 (0 to 1)
Iran	2044	708 (625 to 796)	455 (399 to 509)	202 (169 to 241)	52 (30 to 84)	0 (0 to 0)	2252 (1987 to 2531)	1446 (1269 to 1619)	641 (538 to 765)	165 (96 to 267)	1 (0 to 1)
Iran	2045	711 (629 to 797)	458 (403 to 512)	200 (167 to 240)	52 (30 to 84)	0 (0 to 0)	2260 (2000 to 2535)	1458 (1280 to 1629)	637 (531 to 762)	165 (96 to 268)	1 (0 to 1)
Iran	2046	712 (630 to 799)	461 (405 to 515)	199 (165 to 238)	52 (30 to 85)	0 (0 to 0)	2264 (2003 to 2540)	1467 (1288 to 1638)	632 (524 to 757)	165 (96 to 269)	1 (0 to 1)
Iran	2047	713 (630 to 802)	464 (407 to 517)	197 (163 to 236)	52 (30 to 85)	0 (0 to 0)	2266 (2002 to 2551)	1474 (1295 to 1643)	626 (518 to 752)	165 (96 to 269)	1 (0 to 1)

Iran	2048	712 (630 to 800)	464 (408 to 517)	195 (161 to 235)	52 (30 to 84)	0 (0 to 0)	2262 (2002 to 2544)	1477 (1297 to 1644)	620 (512 to 746)	165 (96 to 268)	1 (0 to 1)
Iran	2049	710 (625 to 798)	465 (409 to 518)	193 (159 to 233)	52 (30 to 84)	0 (0 to 0)	2256 (1988 to 2538)	1478 (1301 to 1645)	613 (505 to 740)	165 (96 to 268)	1 (0 to 1)
Iran	2050	707 (624 to 795)	465 (409 to 516)	190 (156 to 231)	52 (30 to 84)	0 (0 to 0)	2247 (1985 to 2526)	1478 (1301 to 1641)	605 (497 to 733)	164 (95 to 267)	1 (0 to 1)
Iraq	1995	69 (55 to 86)	35 (26 to 45)	34 (24 to 49)	0 (0 to 0)	0 (0 to 0)	216 (173 to 268)	108 (82 to 140)	107 (74 to 152)	0 (0 to 0)	1 (1 to 1)
Iraq	1996	71 (57 to 88)	37 (28 to 47)	34 (24 to 48)	0 (0 to 0)	0 (0 to 0)	222 (179 to 276)	115 (88 to 148)	106 (74 to 150)	0 (0 to 0)	1 (1 to 1)
Iraq	1997	80 (64 to 100)	44 (34 to 57)	36 (25 to 50)	0 (0 to 0)	0 (0 to 0)	251 (199 to 313)	138 (106 to 180)	112 (79 to 157)	0 (0 to 0)	0 (0 to 0)
Iraq	1998	99 (79 to 122)	58 (45 to 76)	40 (29 to 55)	0 (0 to 0)	0 (0 to 0)	309 (247 to 381)	182 (140 to 236)	126 (90 to 171)	0 (0 to 0)	0 (0 to 0)
Iraq	1999	98 (79 to 121)	59 (45 to 75)	40 (28 to 54)	0 (0 to 0)	0 (0 to 0)	308 (248 to 378)	183 (142 to 236)	124 (88 to 168)	0 (0 to 0)	0 (0 to 0)
Iraq	2000	101 (82 to 123)	61 (48 to 77)	40 (28 to 53)	0 (0 to 0)	0 (0 to 0)	315 (256 to 385)	190 (149 to 242)	124 (89 to 166)	0 (0 to 0)	0 (0 to 0)
Iraq	2001	107 (88 to 128)	66 (52 to 82)	41 (29 to 55)	0 (0 to 0)	0 (0 to 0)	334 (274 to 402)	206 (162 to 258)	128 (91 to 171)	0 (0 to 0)	0 (0 to 0)
Iraq	2002	107 (89 to 127)	66 (53 to 82)	41 (30 to 54)	0 (0 to 0)	0 (0 to 0)	335 (277 to 399)	207 (166 to 255)	127 (93 to 170)	0 (0 to 0)	0 (0 to 0)
Iraq	2003	85 (71 to 99)	49 (40 to 60)	34 (25 to 45)	0 (0 to 0)	1 (1 to 1)	264 (222 to 311)	154 (125 to 187)	107 (78 to 141)	0 (0 to 0)	3 (3 to 3)
Iraq	2004	131 (111 to 153)	87 (72 to 106)	41 (30 to 53)	0 (0 to 0)	3 (3 to 3)	410 (348 to 478)	273 (224 to 331)	128 (95 to 165)	0 (0 to 0)	9 (9 to 9)
Iraq	2005	128 (112 to 146)	75 (62 to 91)	35 (27 to 46)	0 (0 to 0)	17 (17 to 17)	399 (349 to 456)	236 (194 to 284)	111 (83 to 143)	0 (0 to 0)	52 (52 to 52)

Iraq	2006	119 (103 to 136)	72 (60 to 87)	33 (26 to 43)	0 (0 to 0)	13 (13 to 13)	371 (322 to 426)	225 (186 to 272)	105 (80 to 134)	0 (0 to 0)	41 (41 to 41)
Iraq	2007	116 (100 to 133)	75 (62 to 90)	33 (25 to 43)	0 (0 to 0)	8 (8 to 8)	363 (314 to 417)	234 (193 to 280)	104 (80 to 133)	0 (0 to 0)	24 (24 to 24)
Iraq	2008	133 (114 to 152)	95 (78 to 112)	36 (28 to 45)	0 (0 to 0)	3 (3 to 3)	416 (357 to 476)	296 (244 to 350)	112 (87 to 142)	0 (0 to 0)	9 (9 to 9)
Iraq	2009	143 (123 to 164)	101 (83 to 121)	40 (31 to 50)	0 (0 to 0)	2 (2 to 2)	448 (384 to 514)	317 (259 to 377)	124 (97 to 155)	0 (0 to 0)	8 (8 to 8)
Iraq	2010	134 (115 to 154)	90 (73 to 108)	42 (33 to 53)	0 (0 to 0)	2 (2 to 2)	420 (360 to 481)	280 (230 to 337)	132 (104 to 164)	0 (0 to 0)	7 (7 to 7)
Iraq	2011	127 (109 to 144)	78 (64 to 94)	48 (38 to 59)	0 (0 to 0)	1 (1 to 1)	397 (341 to 451)	244 (199 to 293)	150 (119 to 184)	0 (0 to 0)	3 (3 to 3)
Iraq	2012	131 (113 to 149)	67 (54 to 81)	64 (51 to 78)	0 (0 to 0)	1 (1 to 1)	411 (353 to 467)	208 (170 to 254)	201 (161 to 243)	0 (0 to 0)	2 (2 to 2)
Iraq	2013	151 (129 to 171)	68 (55 to 84)	82 (67 to 99)	0 (0 to 0)	1 (1 to 1)	472 (403 to 535)	212 (171 to 262)	258 (208 to 310)	0 (0 to 0)	2 (2 to 2)
Iraq	2014	152 (130 to 173)	58 (46 to 71)	93 (76 to 111)	0 (0 to 0)	1 (1 to 1)	474 (407 to 541)	180 (145 to 223)	291 (237 to 346)	0 (0 to 0)	3 (3 to 3)
Iraq	2015	162 (140 to 187)	53 (42 to 65)	109 (90 to 130)	0 (0 to 0)	0 (0 to 0)	508 (439 to 586)	165 (132 to 205)	341 (280 to 406)	0 (0 to 0)	1 (1 to 1)
Iraq	2016	185 (160 to 212)	57 (45 to 71)	127 (105 to 152)	0 (0 to 0)	0 (0 to 0)	577 (500 to 663)	179 (142 to 222)	397 (329 to 476)	0 (0 to 0)	1 (1 to 1)
Iraq	2017	195 (166 to 230)	63 (49 to 78)	131 (107 to 160)	0 (0 to 0)	0 (0 to 0)	609 (520 to 719)	197 (154 to 245)	411 (335 to 501)	0 (0 to 0)	2 (2 to 2)
Iraq	2018	190 (161 to 223)	60 (46 to 75)	130 (106 to 158)	0 (0 to 0)	0 (0 to 0)	593 (504 to 697)	186 (145 to 234)	405 (331 to 494)	0 (0 to 0)	2 (2 to 2)
Iraq	2019	214 (183 to 251)	82 (64 to 104)	131 (107 to 159)	0 (0 to 0)	0 (0 to 0)	669 (573 to 786)	258 (200 to 325)	409 (333 to 499)	0 (0 to 0)	2 (2 to 2)
Iraq	2020	220 (187 to 254)	86 (66 to 109)	133 (109 to 163)	0 (0 to 0)	0 (0 to 1)	686 (586 to 796)	267 (206 to 342)	417 (341 to 508)	0 (0 to 0)	2 (1 to 2)

Iraq	2021	224 (190 to 263)	88 (67 to 113)	135 (110 to 165)	0 (0 to 0)	0 (0 to 1)	700 (594 to 823)	276 (211 to 352)	423 (345 to 515)	0 (0 to 0)	2 (1 to 2)
Iraq	2022	228 (194 to 268)	91 (69 to 118)	137 (112 to 167)	0 (0 to 0)	0 (0 to 1)	713 (605 to 837)	283 (216 to 368)	428 (350 to 521)	0 (0 to 0)	2 (1 to 2)
Iraq	2023	231 (194 to 271)	93 (71 to 121)	138 (113 to 168)	0 (0 to 0)	0 (0 to 1)	724 (608 to 846)	290 (221 to 377)	432 (354 to 526)	0 (0 to 0)	2 (1 to 2)
Iraq	2024	235 (199 to 275)	95 (72 to 124)	140 (114 to 170)	0 (0 to 0)	0 (0 to 1)	736 (622 to 860)	297 (224 to 387)	437 (358 to 532)	0 (0 to 0)	2 (1 to 2)
Iraq	2025	239 (202 to 280)	97 (73 to 128)	142 (116 to 172)	0 (0 to 0)	0 (0 to 1)	748 (633 to 876)	303 (227 to 401)	443 (363 to 539)	0 (0 to 0)	2 (1 to 2)
Iraq	2026	242 (204 to 284)	99 (74 to 131)	143 (117 to 175)	0 (0 to 0)	0 (0 to 1)	758 (637 to 887)	308 (230 to 409)	448 (367 to 547)	0 (0 to 0)	2 (1 to 2)
Iraq	2027	246 (205 to 292)	100 (74 to 134)	145 (119 to 177)	0 (0 to 0)	0 (0 to 1)	770 (642 to 912)	314 (232 to 419)	454 (371 to 554)	0 (0 to 0)	2 (1 to 2)
Iraq	2028	250 (210 to 295)	103 (75 to 137)	147 (120 to 180)	0 (0 to 0)	0 (0 to 1)	783 (655 to 923)	321 (234 to 427)	460 (376 to 562)	0 (0 to 0)	2 (1 to 2)
Iraq	2029	254 (214 to 301)	105 (76 to 140)	149 (122 to 182)	0 (0 to 0)	0 (0 to 1)	795 (671 to 941)	328 (236 to 437)	466 (380 to 570)	0 (0 to 0)	2 (1 to 2)
Iraq	2030	258 (214 to 306)	107 (76 to 142)	151 (123 to 184)	0 (0 to 0)	0 (0 to 1)	808 (670 to 958)	334 (239 to 445)	472 (385 to 577)	0 (0 to 0)	2 (1 to 2)
Iraq	2031	262 (217 to 310)	109 (77 to 147)	153 (125 to 187)	0 (0 to 0)	0 (0 to 1)	821 (680 to 969)	340 (242 to 458)	479 (390 to 585)	0 (0 to 0)	2 (1 to 2)
Iraq	2032	266 (220 to 318)	111 (78 to 151)	155 (126 to 190)	0 (0 to 0)	0 (0 to 1)	833 (689 to 995)	347 (244 to 472)	485 (395 to 593)	0 (0 to 0)	2 (1 to 2)
Iraq	2033	271 (222 to 320)	113 (79 to 154)	157 (128 to 193)	0 (0 to 0)	0 (0 to 1)	846 (694 to 1000)	353 (246 to 482)	491 (400 to 603)	0 (0 to 0)	2 (1 to 2)
Iraq	2034	275 (228 to 330)	115 (80 to 157)	159 (129 to 196)	0 (0 to 0)	0 (0 to 1)	859 (712 to 1033)	360 (251 to 492)	498 (405 to 613)	0 (0 to 0)	2 (1 to 2)
Iraq	2035	279 (230 to 335)	117 (81 to 161)	161 (131 to 199)	0 (0 to 0)	0 (0 to 1)	872 (721 to 1049)	366 (254 to 504)	504 (410 to 622)	0 (0 to 0)	2 (1 to 2)

Iraq	2036	283 (231 to 346)	119 (82 to 166)	163 (133 to 202)	0 (0 to 0)	0 (0 to 1)	886 (723 to 1081)	373 (256 to 519)	511 (415 to 632)	0 (0 to 0)	2 (1 to 2)
Iraq	2037	288 (236 to 351)	122 (82 to 171)	166 (134 to 205)	0 (0 to 0)	0 (0 to 1)	900 (738 to 1096)	381 (258 to 534)	518 (419 to 641)	0 (0 to 0)	2 (1 to 2)
Iraq	2038	292 (240 to 352)	124 (84 to 175)	168 (135 to 208)	0 (0 to 0)	0 (0 to 1)	914 (752 to 1102)	388 (262 to 546)	524 (423 to 650)	0 (0 to 0)	2 (1 to 2)
Iraq	2039	297 (241 to 365)	126 (85 to 177)	170 (137 to 211)	0 (0 to 0)	0 (0 to 1)	927 (755 to 1141)	395 (265 to 555)	531 (428 to 659)	0 (0 to 0)	2 (1 to 2)
Iraq	2040	301 (242 to 367)	128 (85 to 182)	172 (138 to 214)	0 (0 to 0)	0 (0 to 1)	940 (757 to 1149)	401 (265 to 568)	537 (432 to 668)	0 (0 to 0)	2 (1 to 2)
Iraq	2041	305 (248 to 375)	131 (86 to 188)	174 (140 to 217)	0 (0 to 0)	0 (0 to 1)	955 (774 to 1172)	409 (270 to 589)	544 (437 to 677)	0 (0 to 0)	2 (1 to 2)
Iraq	2042	309 (246 to 380)	133 (88 to 190)	176 (141 to 219)	0 (0 to 0)	0 (0 to 1)	967 (769 to 1187)	415 (275 to 595)	550 (442 to 686)	0 (0 to 0)	2 (1 to 2)
Iraq	2043	313 (253 to 385)	135 (89 to 196)	178 (143 to 222)	0 (0 to 0)	0 (0 to 1)	979 (791 to 1203)	422 (277 to 613)	556 (447 to 694)	0 (0 to 0)	2 (1 to 2)
Iraq	2044	318 (254 to 391)	137 (89 to 201)	180 (144 to 225)	0 (0 to 0)	0 (0 to 1)	993 (794 to 1223)	429 (278 to 628)	563 (451 to 704)	0 (0 to 0)	2 (1 to 2)
Iraq	2045	323 (258 to 399)	140 (91 to 206)	182 (146 to 228)	0 (0 to 0)	1 (0 to 1)	1009 (805 to 1249)	437 (284 to 645)	570 (456 to 714)	0 (0 to 0)	2 (1 to 2)
Iraq	2046	327 (261 to 409)	142 (92 to 212)	184 (148 to 231)	0 (0 to 0)	1 (0 to 1)	1023 (818 to 1280)	444 (287 to 663)	577 (461 to 723)	0 (0 to 0)	2 (1 to 2)
Iraq	2047	332 (266 to 416)	144 (95 to 216)	187 (149 to 234)	0 (0 to 0)	1 (0 to 1)	1037 (833 to 1301)	452 (296 to 676)	584 (466 to 732)	0 (0 to 0)	2 (1 to 2)
Iraq	2048	336 (270 to 419)	147 (95 to 217)	189 (151 to 237)	0 (0 to 0)	1 (0 to 1)	1051 (845 to 1310)	459 (297 to 678)	590 (471 to 740)	0 (0 to 0)	2 (1 to 2)

Iraq	2049	340 (268 to 427)	149 (97 to 219)	191 (152 to 239)	0 (0 to 0)	1 (0 to 1)	1063 (839 to 1335)	465 (303 to 686)	596 (476 to 748)	0 (0 to 0)	2 (1 to 2)
Iraq	2050	344 (271 to 430)	151 (95 to 226)	193 (154 to 242)	0 (0 to 0)	1 (0 to 1)	1076 (848 to 1345)	471 (298 to 707)	603 (481 to 756)	0 (0 to 0)	2 (1 to 2)
Ireland	1995	1687 (1542 to 1840)	1272 (1149 to 1407)	177 (150 to 207)	238 (186 to 290)	0 (0 to 0)	1841 (1683 to 2008)	1388 (1254 to 1535)	193 (164 to 226)	260 (203 to 317)	0 (0 to 0)
Ireland	1996	1786 (1660 to 1914)	1353 (1240 to 1468)	188 (162 to 216)	245 (204 to 289)	0 (0 to 0)	1949 (1811 to 2088)	1476 (1353 to 1601)	205 (176 to 236)	268 (223 to 315)	0 (0 to 0)
Ireland	1997	1944 (1828 to 2064)	1486 (1381 to 1593)	203 (177 to 227)	255 (216 to 296)	0 (0 to 0)	2121 (1994 to 2252)	1622 (1507 to 1738)	221 (193 to 248)	278 (236 to 324)	0 (0 to 0)
Ireland	1998	2102 (1986 to 2222)	1619 (1511 to 1739)	218 (195 to 243)	264 (227 to 304)	0 (0 to 0)	2293 (2167 to 2425)	1767 (1649 to 1897)	238 (212 to 265)	288 (248 to 332)	0 (0 to 0)
Ireland	1999	2314 (2196 to 2444)	1795 (1686 to 1914)	247 (222 to 273)	273 (236 to 317)	0 (0 to 0)	2525 (2396 to 2667)	1958 (1840 to 2089)	269 (243 to 298)	298 (257 to 346)	0 (0 to 0)
Ireland	2000	2511 (2391 to 2637)	1952 (1839 to 2066)	281 (257 to 307)	278 (243 to 319)	0 (0 to 0)	2740 (2609 to 2877)	2130 (2006 to 2254)	307 (280 to 335)	303 (266 to 348)	0 (0 to 0)
Ireland	2001	2826 (2704 to 2952)	2210 (2098 to 2328)	317 (292 to 344)	298 (261 to 338)	0 (0 to 0)	3083 (2950 to 3221)	2412 (2290 to 2540)	346 (318 to 375)	326 (285 to 369)	0 (0 to 0)
Ireland	2002	3125 (2990 to 3260)	2457 (2333 to 2583)	359 (332 to 387)	308 (272 to 350)	0 (0 to 0)	3410 (3263 to 3557)	2681 (2546 to 2819)	392 (362 to 423)	336 (296 to 382)	0 (0 to 0)

Ireland	2003	3307 (3171 to 3441)	2603 (2477 to 2733)	408 (380 to 440)	296 (261 to 338)	0 (0 to 0)	3609 (3460 to 3755)	2840 (2703 to 2983)	446 (415 to 480)	323 (285 to 368)	0 (0 to 0)
Ireland	2004	3566 (3426 to 3702)	2809 (2685 to 2932)	453 (424 to 485)	304 (266 to 346)	0 (0 to 0)	3892 (3739 to 4039)	3065 (2930 to 3200)	494 (463 to 529)	332 (291 to 378)	0 (0 to 0)
Ireland	2005	3818 (3676 to 3957)	3006 (2880 to 3141)	499 (468 to 530)	313 (275 to 351)	0 (0 to 0)	4166 (4011 to 4317)	3280 (3143 to 3428)	545 (511 to 578)	341 (300 to 383)	0 (0 to 0)
Ireland	2006	4008 (3879 to 4152)	3152 (3026 to 3281)	523 (491 to 554)	332 (295 to 373)	0 (0 to 0)	4373 (4233 to 4530)	3440 (3302 to 3580)	571 (536 to 605)	363 (321 to 407)	0 (0 to 0)
Ireland	2007	4325 (4186 to 4465)	3408 (3280 to 3540)	541 (510 to 573)	377 (337 to 421)	0 (0 to 0)	4720 (4567 to 4872)	3718 (3579 to 3862)	590 (557 to 625)	411 (368 to 460)	0 (0 to 0)
Ireland	2008	4558 (4422 to 4704)	3566 (3438 to 3701)	573 (540 to 604)	419 (378 to 463)	0 (0 to 0)	4973 (4825 to 5133)	3892 (3752 to 4038)	625 (590 to 659)	457 (412 to 505)	0 (0 to 0)
Ireland	2009	4713 (4573 to 4861)	3623 (3494 to 3754)	614 (581 to 647)	476 (436 to 520)	0 (0 to 0)	5142 (4990 to 5304)	3953 (3812 to 4096)	670 (634 to 706)	519 (476 to 568)	0 (0 to 0)
Ireland	2010	5671 (5503 to 5842)	4312 (4160 to 4471)	715 (678 to 753)	644 (592 to 699)	0 (0 to 0)	6188 (6005 to 6374)	4705 (4539 to 4879)	781 (740 to 822)	703 (646 to 763)	0 (0 to 0)
Ireland	2011	4925 (4775 to 5076)	3598 (3462 to 3734)	688 (656 to 723)	639 (593 to 691)	0 (0 to 0)	5374 (5210 to 5539)	3926 (3778 to 4074)	751 (716 to 788)	697 (647 to 754)	0 (0 to 0)
Ireland	2012	4866 (4722 to 5015)	3486 (3356 to 3616)	693 (660 to 727)	687 (642 to 739)	0 (0 to 0)	5310 (5152 to 5472)	3804 (3662 to 3946)	756 (720 to 793)	750 (700 to 806)	0 (0 to 0)

Ireland	2013	4857 (4709 to 5006)	3454 (3322 to 3588)	688 (653 to 723)	715 (668 to 764)	0 (0 to 0)	5300 (5138 to 5462)	3769 (3625 to 3915)	751 (713 to 789)	781 (729 to 833)	0 (0 to 0)
Ireland	2014	4881 (4735 to 5023)	3480 (3350 to 3619)	672 (638 to 708)	728 (678 to 781)	0 (0 to 0)	5326 (5166 to 5481)	3798 (3656 to 3948)	733 (696 to 773)	795 (740 to 852)	0 (0 to 0)
Ireland	2015	4758 (4617 to 4903)	3418 (3281 to 3559)	636 (604 to 670)	704 (656 to 754)	0 (0 to 0)	5192 (5038 to 5351)	3730 (3581 to 3884)	694 (659 to 731)	768 (715 to 823)	0 (0 to 0)
Ireland	2016	4853 (4698 to 5020)	3519 (3383 to 3671)	623 (588 to 659)	711 (666 to 766)	0 (0 to 0)	5295 (5126 to 5478)	3840 (3692 to 4006)	679 (641 to 719)	776 (726 to 835)	0 (0 to 0)
Ireland	2017	4979 (4718 to 5249)	3640 (3460 to 3842)	618 (573 to 667)	721 (654 to 794)	0 (0 to 0)	5433 (5148 to 5728)	3972 (3776 to 4193)	674 (625 to 728)	787 (714 to 866)	0 (0 to 0)
Ireland	2018	5284 (5005 to 5587)	3860 (3661 to 4077)	653 (604 to 704)	771 (700 to 850)	0 (0 to 0)	5766 (5461 to 6096)	4212 (3994 to 4449)	712 (659 to 768)	841 (763 to 927)	0 (0 to 0)
Ireland	2019	5462 (5153 to 5791)	3994 (3774 to 4243)	671 (620 to 724)	797 (723 to 879)	0 (0 to 0)	5960 (5622 to 6319)	4358 (4118 to 4630)	732 (677 to 790)	870 (789 to 960)	0 (0 to 0)
Ireland	2020	5576 (5227 to 5934)	4075 (3810 to 4350)	686 (634 to 741)	815 (739 to 900)	0 (0 to 0)	6085 (5704 to 6475)	4447 (4158 to 4746)	749 (692 to 808)	890 (806 to 982)	0 (0 to 0)
Ireland	2021	5677 (5321 to 6051)	4146 (3875 to 4433)	700 (648 to 757)	832 (753 to 917)	0 (0 to 0)	6195 (5806 to 6603)	4524 (4228 to 4837)	764 (707 to 826)	907 (822 to 1000)	0 (0 to 0)
Ireland	2022	5682 (5311 to 6089)	4145 (3855 to 4461)	703 (650 to 760)	834 (755 to 919)	0 (0 to 0)	6200 (5796 to 6645)	4523 (4207 to 4868)	767 (709 to 830)	910 (824 to 1002)	0 (0 to 0)

Ireland	2023	5682 (5282 to 6104)	4140 (3829 to 4467)	706 (653 to 764)	836 (757 to 921)	0 (0 to 0)	6200 (5764 to 6660)	4518 (4178 to 4874)	770 (712 to 834)	912 (826 to 1005)	0 (0 to 0)
Ireland	2024	5717 (5315 to 6158)	4163 (3845 to 4515)	709 (656 to 767)	845 (765 to 930)	0 (0 to 0)	6239 (5799 to 6719)	4543 (4196 to 4927)	774 (716 to 837)	922 (835 to 1015)	0 (0 to 0)
Ireland	2025	5755 (5339 to 6198)	4191 (3860 to 4543)	711 (657 to 770)	853 (772 to 940)	0 (0 to 0)	6280 (5826 to 6763)	4573 (4212 to 4957)	776 (717 to 840)	931 (843 to 1026)	0 (0 to 0)
Ireland	2026	5795 (5383 to 6260)	4219 (3884 to 4582)	714 (660 to 773)	862 (781 to 951)	0 (0 to 0)	6324 (5874 to 6831)	4603 (4238 to 4999)	779 (720 to 843)	941 (852 to 1038)	0 (0 to 0)
Ireland	2027	5841 (5412 to 6305)	4251 (3911 to 4631)	718 (663 to 776)	873 (789 to 963)	0 (0 to 0)	6374 (5906 to 6880)	4638 (4268 to 5053)	783 (723 to 847)	952 (861 to 1051)	0 (0 to 0)
Ireland	2028	5890 (5454 to 6380)	4286 (3932 to 4660)	721 (667 to 780)	883 (798 to 975)	0 (0 to 0)	6428 (5951 to 6962)	4677 (4290 to 5085)	787 (727 to 851)	963 (871 to 1064)	0 (0 to 0)
Ireland	2029	5991 (5541 to 6473)	4358 (3992 to 4747)	732 (676 to 791)	901 (813 to 998)	0 (0 to 0)	6538 (6047 to 7063)	4756 (4356 to 5179)	798 (738 to 864)	984 (887 to 1089)	0 (0 to 0)
Ireland	2030	6150 (5670 to 6662)	4474 (4091 to 4875)	748 (691 to 809)	928 (835 to 1027)	0 (0 to 0)	6711 (6187 to 7270)	4882 (4464 to 5319)	816 (754 to 883)	1012 (911 to 1121)	0 (0 to 0)
Ireland	2031	6340 (5841 to 6846)	4621 (4229 to 5020)	764 (705 to 827)	954 (859 to 1058)	0 (0 to 0)	6918 (6374 to 7470)	5043 (4615 to 5478)	834 (770 to 902)	1041 (937 to 1154)	0 (0 to 0)
Ireland	2032	6535 (6005 to 7080)	4774 (4360 to 5193)	780 (720 to 844)	981 (880 to 1088)	0 (0 to 0)	7130 (6553 to 7726)	5209 (4757 to 5666)	851 (785 to 921)	1070 (961 to 1188)	0 (0 to 0)

Ireland	2033	6727 (6187 to 7273)	4926 (4495 to 5376)	795 (734 to 861)	1006 (902 to 1118)	0 (0 to 0)	7340 (6752 to 7936)	5375 (4905 to 5866)	867 (801 to 939)	1098 (985 to 1220)	0 (0 to 0)
Ireland	2034	6913 (6339 to 7504)	5072 (4622 to 5533)	809 (746 to 877)	1031 (924 to 1147)	0 (0 to 0)	7543 (6917 to 8188)	5535 (5043 to 6038)	883 (814 to 957)	1125 (1008 to 1252)	0 (0 to 0)
Ireland	2035	7098 (6515 to 7696)	5218 (4742 to 5705)	824 (758 to 893)	1057 (945 to 1177)	0 (0 to 0)	7745 (7109 to 8398)	5694 (5174 to 6225)	899 (827 to 974)	1153 (1031 to 1284)	0 (0 to 0)
Ireland	2036	7286 (6662 to 7926)	5366 (4875 to 5858)	838 (770 to 909)	1082 (966 to 1207)	0 (0 to 0)	7951 (7269 to 8649)	5856 (5320 to 6392)	914 (841 to 992)	1181 (1054 to 1317)	0 (0 to 0)
Ireland	2037	7472 (6842 to 8097)	5512 (5004 to 6034)	852 (783 to 925)	1108 (986 to 1237)	0 (0 to 0)	8153 (7466 to 8836)	6015 (5461 to 6584)	930 (854 to 1010)	1209 (1076 to 1350)	0 (0 to 0)
Ireland	2038	7651 (6994 to 8343)	5652 (5134 to 6197)	865 (795 to 941)	1133 (1006 to 1267)	0 (0 to 0)	8348 (7632 to 9104)	6168 (5602 to 6762)	944 (867 to 1027)	1236 (1098 to 1382)	0 (0 to 0)
Ireland	2039	7830 (7159 to 8525)	5793 (5259 to 6369)	879 (806 to 956)	1158 (1028 to 1300)	0 (0 to 0)	8544 (7812 to 9303)	6321 (5739 to 6950)	959 (880 to 1043)	1264 (1122 to 1418)	0 (0 to 0)
Ireland	2040	8014 (7302 to 8758)	5937 (5370 to 6539)	893 (818 to 972)	1184 (1048 to 1332)	0 (0 to 0)	8744 (7968 to 9557)	6478 (5859 to 7135)	974 (892 to 1061)	1292 (1144 to 1453)	0 (0 to 0)
Ireland	2041	8191 (7461 to 8936)	6075 (5484 to 6695)	906 (829 to 987)	1210 (1068 to 1365)	0 (0 to 0)	8937 (8142 to 9751)	6629 (5984 to 7305)	988 (905 to 1077)	1320 (1166 to 1490)	0 (0 to 0)
Ireland	2042	8359 (7614 to 9157)	6206 (5615 to 6850)	918 (840 to 1002)	1235 (1088 to 1395)	0 (0 to 0)	9121 (8308 to 9992)	6772 (6127 to 7475)	1002 (916 to 1094)	1347 (1187 to 1522)	0 (0 to 0)

Ireland	2043	8534 (7763 to 9340)	6343 (5727 to 7014)	930 (850 to 1018)	1260 (1108 to 1425)	0 (0 to 0)	9312 (8471 to 10191)	6922 (6249 to 7653)	1015 (928 to 1110)	1375 (1210 to 1555)	0 (0 to 0)
Ireland	2044	8710 (7908 to 9564)	6483 (5843 to 7147)	942 (859 to 1031)	1284 (1128 to 1454)	0 (0 to 0)	9504 (8629 to 10436)	7074 (6375 to 7798)	1028 (938 to 1125)	1401 (1231 to 1587)	0 (0 to 0)
Ireland	2045	8882 (8071 to 9741)	6620 (5973 to 7307)	953 (869 to 1045)	1308 (1148 to 1484)	0 (0 to 0)	9692 (8807 to 10629)	7224 (6518 to 7973)	1040 (948 to 1140)	1428 (1252 to 1619)	0 (0 to 0)
Ireland	2046	9063 (8204 to 9962)	6763 (6070 to 7467)	966 (879 to 1061)	1334 (1168 to 1514)	0 (0 to 0)	9889 (8952 to 10870)	7379 (6623 to 8148)	1054 (959 to 1158)	1456 (1275 to 1653)	0 (0 to 0)
Ireland	2047	9246 (8373 to 10175)	6904 (6198 to 7657)	979 (890 to 1078)	1363 (1189 to 1550)	0 (0 to 0)	10089 (9136 to 11103)	7534 (6763 to 8355)	1069 (971 to 1176)	1487 (1298 to 1692)	0 (0 to 0)
Ireland	2048	9439 (8548 to 10418)	7052 (6343 to 7817)	994 (902 to 1094)	1393 (1212 to 1587)	0 (0 to 0)	10300 (9328 to 11368)	7695 (6922 to 8530)	1085 (984 to 1194)	1520 (1323 to 1731)	0 (0 to 0)
Ireland	2049	9632 (8699 to 10578)	7198 (6460 to 7952)	1010 (915 to 1115)	1424 (1236 to 1627)	0 (0 to 0)	10510 (9492 to 11542)	7854 (7050 to 8677)	1102 (998 to 1216)	1554 (1348 to 1776)	0 (0 to 0)
Ireland	2050	9845 (8882 to 10873)	7358 (6589 to 8134)	1028 (930 to 1138)	1459 (1261 to 1672)	0 (0 to 0)	10743 (9692 to 11864)	8029 (7190 to 8876)	1122 (1015 to 1241)	1592 (1376 to 1825)	0 (0 to 0)
Israel	1995	2028 (1949 to 2105)	1371 (1311 to 1429)	607 (559 to 655)	50 (37 to 66)	0 (0 to 0)	1856 (1783 to 1926)	1254 (1200 to 1307)	555 (511 to 599)	46 (34 to 61)	0 (0 to 0)
Israel	1996	2083 (2023 to 2142)	1412 (1368 to 1454)	614 (577 to 652)	57 (44 to 71)	0 (0 to 0)	1906 (1851 to 1960)	1292 (1252 to 1330)	562 (528 to 597)	52 (40 to 65)	0 (0 to 0)

Israel	1997	2111 (2051 to 2171)	1423 (1380 to 1469)	621 (588 to 657)	67 (54 to 81)	0 (0 to 0)	1932 (1877 to 1986)	1302 (1263 to 1344)	568 (538 to 601)	61 (50 to 74)	0 (0 to 0)
Israel	1998	2097 (2036 to 2153)	1394 (1353 to 1437)	625 (592 to 660)	78 (65 to 93)	0 (0 to 0)	1918 (1863 to 1970)	1275 (1238 to 1315)	572 (542 to 604)	71 (59 to 85)	0 (0 to 0)
Israel	1999	2089 (2033 to 2142)	1369 (1329 to 1410)	630 (598 to 662)	90 (76 to 106)	0 (0 to 0)	1911 (1860 to 1960)	1252 (1216 to 1290)	576 (548 to 606)	83 (70 to 97)	0 (0 to 0)
Israel	2000	2149 (2098 to 2197)	1395 (1359 to 1434)	649 (618 to 680)	105 (91 to 123)	0 (0 to 0)	1967 (1920 to 2010)	1276 (1244 to 1312)	594 (566 to 622)	96 (83 to 112)	0 (0 to 0)
Israel	2001	2196 (2146 to 2243)	1417 (1381 to 1455)	660 (630 to 691)	118 (105 to 136)	0 (0 to 0)	2009 (1963 to 2052)	1297 (1264 to 1331)	604 (577 to 632)	108 (96 to 124)	0 (0 to 0)
Israel	2002	2219 (2170 to 2271)	1432 (1394 to 1469)	652 (622 to 682)	135 (120 to 151)	0 (0 to 0)	2030 (1985 to 2078)	1310 (1276 to 1344)	597 (569 to 624)	123 (110 to 139)	0 (0 to 0)
Israel	2003	2158 (2109 to 2208)	1373 (1339 to 1409)	639 (610 to 666)	147 (132 to 163)	0 (0 to 0)	1975 (1930 to 2020)	1256 (1225 to 1289)	584 (558 to 610)	134 (121 to 149)	0 (0 to 0)
Israel	2004	2160 (2110 to 2206)	1356 (1321 to 1391)	640 (614 to 668)	164 (148 to 181)	0 (0 to 0)	1976 (1931 to 2019)	1241 (1209 to 1273)	585 (561 to 611)	150 (136 to 166)	0 (0 to 0)
Israel	2005	2222 (2171 to 2271)	1381 (1344 to 1416)	648 (621 to 678)	192 (177 to 211)	0 (0 to 0)	2033 (1986 to 2078)	1264 (1230 to 1296)	593 (568 to 620)	176 (162 to 193)	0 (0 to 0)
Israel	2006	2299 (2249 to 2348)	1429 (1392 to 1466)	636 (607 to 665)	234 (215 to 253)	0 (0 to 0)	2103 (2058 to 2148)	1308 (1274 to 1341)	582 (555 to 609)	214 (196 to 231)	0 (0 to 0)

Israel	2007	2352 (2300 to 2403)	1455 (1418 to 1492)	631 (603 to 660)	267 (246 to 287)	0 (0 to 0)	2152 (2105 to 2199)	1331 (1297 to 1365)	577 (551 to 604)	244 (225 to 263)	0 (0 to 0)
Israel	2008	2405 (2354 to 2455)	1491 (1455 to 1531)	624 (597 to 652)	290 (269 to 312)	0 (0 to 0)	2200 (2154 to 2246)	1364 (1332 to 1401)	571 (546 to 597)	265 (246 to 286)	0 (0 to 0)
Israel	2009	2452 (2404 to 2502)	1530 (1495 to 1567)	620 (593 to 648)	303 (281 to 327)	0 (0 to 0)	2244 (2199 to 2289)	1400 (1368 to 1434)	567 (542 to 593)	277 (257 to 299)	0 (0 to 0)
Israel	2010	2493 (2440 to 2547)	1571 (1533 to 1610)	613 (585 to 643)	310 (288 to 334)	0 (0 to 0)	2281 (2233 to 2331)	1437 (1403 to 1473)	561 (535 to 588)	284 (264 to 305)	0 (0 to 0)
Israel	2011	2545 (2489 to 2600)	1613 (1574 to 1652)	613 (586 to 642)	319 (298 to 343)	0 (0 to 0)	2329 (2277 to 2379)	1476 (1440 to 1511)	561 (536 to 587)	292 (272 to 314)	0 (0 to 0)
Israel	2012	2629 (2575 to 2682)	1675 (1637 to 1716)	620 (591 to 648)	334 (310 to 359)	0 (0 to 0)	2405 (2356 to 2454)	1533 (1498 to 1570)	567 (540 to 593)	305 (284 to 329)	0 (0 to 0)
Israel	2013	2692 (2637 to 2747)	1725 (1685 to 1763)	625 (596 to 654)	343 (318 to 370)	0 (0 to 0)	2464 (2413 to 2513)	1578 (1542 to 1613)	572 (545 to 598)	314 (291 to 338)	0 (0 to 0)
Israel	2014	2728 (2676 to 2784)	1750 (1711 to 1788)	631 (603 to 658)	347 (322 to 373)	0 (0 to 0)	2496 (2448 to 2547)	1602 (1566 to 1636)	577 (552 to 602)	317 (295 to 342)	0 (0 to 0)
Israel	2015	2751 (2694 to 2810)	1769 (1730 to 1810)	633 (606 to 661)	349 (325 to 374)	0 (0 to 0)	2517 (2464 to 2571)	1619 (1583 to 1656)	579 (554 to 605)	319 (297 to 342)	0 (0 to 0)
Israel	2016	2855 (2797 to 2915)	1844 (1801 to 1888)	650 (619 to 681)	361 (335 to 387)	0 (0 to 0)	2612 (2560 to 2667)	1687 (1648 to 1728)	595 (566 to 623)	330 (306 to 354)	0 (0 to 0)

Israel	2017	2961 (2864 to 3068)	1918 (1859 to 1976)	670 (628 to 712)	374 (338 to 412)	0 (0 to 0)	2710 (2620 to 2807)	1755 (1701 to 1808)	613 (574 to 652)	342 (310 to 377)	0 (0 to 0)
Israel	2018	3044 (2930 to 3153)	1980 (1916 to 2048)	681 (637 to 725)	384 (347 to 422)	0 (0 to 0)	2785 (2681 to 2885)	1811 (1753 to 1874)	623 (583 to 663)	351 (318 to 386)	0 (0 to 0)
Israel	2019	3098 (2985 to 3216)	2018 (1948 to 2087)	689 (646 to 734)	390 (353 to 430)	0 (0 to 0)	2835 (2731 to 2942)	1847 (1782 to 1910)	631 (591 to 672)	357 (323 to 393)	0 (0 to 0)
Israel	2020	3148 (3022 to 3270)	2053 (1980 to 2128)	698 (653 to 743)	397 (359 to 437)	0 (0 to 0)	2880 (2765 to 2992)	1879 (1812 to 1947)	638 (598 to 680)	363 (329 to 400)	0 (0 to 0)
Israel	2021	3198 (3074 to 3322)	2090 (2011 to 2170)	704 (659 to 750)	403 (365 to 445)	0 (0 to 0)	2926 (2813 to 3040)	1913 (1840 to 1986)	644 (603 to 686)	369 (334 to 407)	0 (0 to 0)
Israel	2022	3250 (3117 to 3382)	2129 (2043 to 2213)	711 (665 to 756)	410 (371 to 453)	0 (0 to 0)	2974 (2852 to 3095)	1948 (1870 to 2025)	651 (609 to 692)	375 (339 to 414)	0 (0 to 0)
Israel	2023	3303 (3166 to 3445)	2167 (2076 to 2260)	718 (673 to 764)	417 (376 to 460)	0 (0 to 0)	3022 (2897 to 3152)	1983 (1899 to 2068)	657 (615 to 699)	381 (344 to 421)	0 (0 to 0)
Israel	2024	3357 (3201 to 3506)	2207 (2103 to 2308)	726 (680 to 773)	424 (382 to 468)	0 (0 to 0)	3071 (2929 to 3208)	2020 (1924 to 2112)	664 (622 to 707)	388 (349 to 429)	0 (0 to 0)
Israel	2025	3412 (3252 to 3575)	2247 (2133 to 2360)	733 (687 to 781)	431 (388 to 476)	0 (0 to 0)	3121 (2976 to 3271)	2056 (1952 to 2160)	671 (628 to 715)	394 (355 to 436)	0 (0 to 0)
Israel	2026	3468 (3287 to 3642)	2289 (2163 to 2412)	741 (694 to 790)	438 (394 to 484)	0 (0 to 0)	3173 (3007 to 3332)	2094 (1979 to 2207)	678 (635 to 723)	401 (361 to 443)	0 (0 to 0)

Israel	2027	3527 (3342 to 3717)	2332 (2196 to 2469)	749 (700 to 799)	446 (401 to 494)	0 (0 to 0)	3227 (3057 to 3401)	2134 (2009 to 2259)	685 (641 to 731)	408 (367 to 452)	0 (0 to 0)
Israel	2028	3587 (3385 to 3794)	2375 (2230 to 2526)	758 (709 to 809)	454 (407 to 504)	0 (0 to 0)	3282 (3097 to 3471)	2173 (2040 to 2312)	693 (648 to 740)	415 (373 to 461)	0 (0 to 0)
Israel	2029	3648 (3437 to 3874)	2419 (2259 to 2589)	767 (716 to 819)	462 (414 to 512)	0 (0 to 0)	3338 (3145 to 3545)	2214 (2067 to 2369)	702 (655 to 749)	422 (379 to 469)	0 (0 to 0)
Israel	2030	3711 (3474 to 3960)	2464 (2290 to 2654)	777 (725 to 829)	470 (422 to 523)	0 (0 to 0)	3396 (3178 to 3623)	2255 (2095 to 2429)	711 (664 to 758)	430 (386 to 478)	0 (0 to 0)
Israel	2031	3775 (3535 to 4035)	2510 (2322 to 2713)	786 (733 to 840)	478 (429 to 532)	0 (0 to 0)	3454 (3234 to 3692)	2297 (2124 to 2483)	719 (671 to 768)	438 (393 to 487)	0 (0 to 0)
Israel	2032	3838 (3559 to 4113)	2556 (2357 to 2775)	796 (741 to 851)	487 (435 to 543)	0 (0 to 0)	3512 (3256 to 3764)	2338 (2156 to 2539)	728 (678 to 779)	445 (398 to 497)	0 (0 to 0)
Israel	2033	3902 (3630 to 4211)	2601 (2388 to 2835)	805 (750 to 861)	495 (442 to 554)	0 (0 to 0)	3570 (3321 to 3853)	2380 (2185 to 2594)	737 (686 to 788)	453 (404 to 507)	0 (0 to 0)
Israel	2034	3967 (3675 to 4276)	2649 (2417 to 2903)	815 (757 to 871)	504 (448 to 565)	0 (0 to 0)	3630 (3362 to 3912)	2424 (2211 to 2656)	745 (693 to 797)	461 (410 to 517)	0 (0 to 0)
Israel	2035	4033 (3718 to 4374)	2697 (2450 to 2967)	824 (764 to 882)	512 (456 to 576)	0 (0 to 0)	3690 (3402 to 4002)	2467 (2242 to 2715)	754 (699 to 807)	469 (417 to 527)	0 (0 to 0)
Israel	2036	4097 (3766 to 4467)	2744 (2489 to 3033)	833 (772 to 893)	521 (462 to 587)	0 (0 to 0)	3749 (3446 to 4087)	2510 (2277 to 2775)	762 (706 to 817)	476 (423 to 537)	0 (0 to 0)

Israel	2037	4160 (3825 to 4532)	2790 (2519 to 3095)	841 (780 to 902)	529 (469 to 598)	0 (0 to 0)	3807 (3500 to 4146)	2553 (2304 to 2832)	770 (714 to 825)	484 (429 to 547)	0 (0 to 0)
Israel	2038	4222 (3862 to 4615)	2835 (2556 to 3153)	849 (785 to 912)	537 (476 to 608)	0 (0 to 0)	3863 (3534 to 4223)	2594 (2339 to 2885)	777 (719 to 835)	492 (435 to 556)	0 (0 to 0)
Israel	2039	4282 (3917 to 4694)	2880 (2583 to 3210)	857 (790 to 922)	545 (482 to 618)	0 (0 to 0)	3918 (3584 to 4294)	2635 (2364 to 2937)	784 (723 to 844)	499 (441 to 565)	0 (0 to 0)
Israel	2040	4342 (3953 to 4768)	2925 (2622 to 3269)	864 (795 to 932)	553 (487 to 627)	0 (0 to 0)	3973 (3617 to 4363)	2677 (2399 to 2991)	790 (728 to 853)	506 (446 to 574)	0 (0 to 0)
Israel	2041	4403 (4014 to 4850)	2970 (2662 to 3324)	872 (802 to 942)	561 (493 to 638)	0 (0 to 0)	4028 (3673 to 4438)	2717 (2436 to 3041)	797 (733 to 862)	513 (451 to 584)	0 (0 to 0)
Israel	2042	4462 (4040 to 4916)	3014 (2691 to 3378)	880 (809 to 951)	569 (499 to 649)	0 (0 to 0)	4083 (3697 to 4498)	2757 (2462 to 3091)	805 (740 to 871)	521 (457 to 594)	0 (0 to 0)
Israel	2043	4521 (4094 to 5003)	3056 (2724 to 3436)	888 (816 to 961)	577 (505 to 660)	0 (0 to 0)	4137 (3746 to 4577)	2796 (2493 to 3144)	812 (747 to 879)	528 (462 to 604)	0 (0 to 0)
Israel	2044	4581 (4148 to 5062)	3099 (2754 to 3493)	897 (824 to 971)	585 (510 to 671)	0 (0 to 0)	4192 (3796 to 4632)	2836 (2520 to 3196)	820 (754 to 888)	535 (467 to 614)	0 (0 to 0)
Israel	2045	4640 (4211 to 5145)	3142 (2783 to 3547)	906 (831 to 981)	593 (517 to 683)	0 (0 to 0)	4246 (3853 to 4708)	2874 (2547 to 3246)	829 (760 to 897)	543 (473 to 624)	0 (0 to 0)
Israel	2046	4701 (4251 to 5213)	3184 (2813 to 3599)	915 (838 to 992)	602 (523 to 693)	0 (0 to 0)	4301 (3889 to 4769)	2914 (2574 to 3293)	837 (767 to 908)	551 (478 to 634)	0 (0 to 0)

Israel	2047	4762 (4304 to 5295)	3227 (2844 to 3647)	925 (846 to 1005)	610 (528 to 705)	0 (0 to 0)	4357 (3938 to 4845)	2953 (2602 to 3337)	846 (774 to 919)	558 (483 to 645)	0 (0 to 0)
Israel	2048	4825 (4355 to 5373)	3271 (2882 to 3696)	935 (853 to 1017)	619 (535 to 716)	0 (0 to 0)	4414 (3985 to 4916)	2993 (2637 to 3382)	855 (780 to 931)	566 (489 to 655)	0 (0 to 0)
Israel	2049	4888 (4408 to 5440)	3315 (2915 to 3747)	945 (861 to 1030)	628 (541 to 728)	0 (0 to 0)	4472 (4033 to 4977)	3033 (2667 to 3428)	865 (788 to 942)	574 (495 to 666)	0 (0 to 0)
Israel	2050	4953 (4442 to 5509)	3359 (2950 to 3801)	956 (869 to 1043)	637 (547 to 741)	0 (0 to 0)	4531 (4064 to 5040)	3074 (2700 to 3477)	875 (795 to 954)	583 (500 to 678)	0 (0 to 0)
Italy	1995	2121 (2046 to 2193)	1518 (1459 to 1579)	579 (537 to 621)	24 (14 to 37)	0 (0 to 0)	2604 (2513 to 2694)	1864 (1792 to 1940)	711 (660 to 763)	29 (18 to 45)	0 (0 to 0)
Italy	1996	2193 (2133 to 2250)	1567 (1522 to 1613)	602 (570 to 636)	24 (15 to 38)	0 (0 to 0)	2693 (2620 to 2763)	1924 (1870 to 1981)	739 (700 to 781)	30 (19 to 47)	0 (0 to 0)
Italy	1997	2269 (2216 to 2322)	1616 (1572 to 1665)	628 (596 to 660)	25 (16 to 38)	0 (0 to 0)	2787 (2722 to 2851)	1985 (1931 to 2045)	771 (732 to 811)	30 (19 to 46)	0 (0 to 0)
Italy	1998	2351 (2298 to 2408)	1675 (1631 to 1720)	651 (620 to 681)	25 (16 to 37)	0 (0 to 0)	2887 (2822 to 2958)	2058 (2003 to 2113)	799 (761 to 836)	30 (20 to 45)	0 (0 to 0)
Italy	1999	2428 (2377 to 2485)	1742 (1697 to 1786)	662 (632 to 691)	25 (17 to 36)	0 (0 to 0)	2982 (2920 to 3051)	2139 (2084 to 2193)	813 (776 to 848)	30 (20 to 45)	0 (0 to 0)
Italy	2000	2547 (2493 to 2600)	1855 (1810 to 1898)	668 (642 to 697)	25 (17 to 35)	0 (0 to 0)	3129 (3062 to 3193)	2278 (2223 to 2331)	821 (788 to 856)	30 (21 to 43)	0 (0 to 0)

Italy	2001	2667 (2615 to 2717)	1977 (1932 to 2021)	665 (639 to 695)	25 (18 to 35)	0 (0 to 0)	3275 (3211 to 3337)	2428 (2372 to 2482)	817 (784 to 853)	31 (22 to 42)	0 (0 to 0)
Italy	2002	2681 (2631 to 2734)	2006 (1964 to 2052)	650 (624 to 678)	25 (18 to 34)	0 (0 to 0)	3293 (3231 to 3358)	2464 (2413 to 2520)	798 (767 to 833)	31 (22 to 42)	0 (0 to 0)
Italy	2003	2729 (2675 to 2781)	2063 (2020 to 2107)	640 (613 to 664)	25 (18 to 35)	0 (0 to 0)	3351 (3285 to 3416)	2534 (2481 to 2588)	786 (753 to 816)	31 (23 to 43)	0 (0 to 0)
Italy	2004	2792 (2742 to 2843)	2133 (2089 to 2178)	633 (609 to 658)	26 (19 to 35)	0 (0 to 0)	3429 (3367 to 3492)	2620 (2566 to 2674)	778 (748 to 808)	32 (23 to 43)	0 (0 to 0)
Italy	2005	2863 (2811 to 2914)	2209 (2166 to 2253)	627 (601 to 654)	27 (19 to 36)	0 (0 to 0)	3516 (3452 to 3579)	2713 (2661 to 2767)	770 (738 to 803)	33 (24 to 44)	0 (0 to 0)
Italy	2006	2928 (2874 to 2981)	2273 (2228 to 2317)	627 (602 to 651)	28 (20 to 37)	0 (0 to 0)	3596 (3530 to 3662)	2792 (2736 to 2846)	770 (740 to 800)	34 (25 to 46)	0 (0 to 0)
Italy	2007	2911 (2857 to 2964)	2262 (2215 to 2311)	620 (597 to 645)	29 (21 to 39)	0 (0 to 0)	3575 (3509 to 3641)	2778 (2720 to 2838)	761 (734 to 792)	36 (26 to 48)	0 (0 to 0)
Italy	2008	2932 (2874 to 2988)	2283 (2235 to 2331)	619 (594 to 643)	30 (22 to 41)	0 (0 to 0)	3601 (3529 to 3670)	2804 (2744 to 2863)	760 (729 to 790)	37 (28 to 50)	0 (0 to 0)
Italy	2009	2947 (2892 to 3000)	2301 (2250 to 2346)	614 (590 to 638)	32 (24 to 42)	0 (0 to 0)	3619 (3552 to 3685)	2826 (2763 to 2881)	754 (724 to 784)	40 (30 to 51)	0 (0 to 0)
Italy	2010	2913 (2860 to 2967)	2267 (2216 to 2316)	612 (588 to 636)	34 (26 to 44)	0 (0 to 0)	3578 (3513 to 3644)	2784 (2721 to 2844)	751 (723 to 781)	42 (32 to 54)	0 (0 to 0)

Italy	2011	2882 (2828 to 2936)	2224 (2177 to 2276)	618 (593 to 644)	39 (30 to 50)	0 (0 to 0)	3539 (3474 to 3606)	2732 (2673 to 2795)	759 (728 to 791)	48 (36 to 61)	0 (0 to 0)
Italy	2012	2860 (2805 to 2918)	2190 (2145 to 2238)	620 (596 to 645)	50 (38 to 64)	0 (0 to 0)	3513 (3445 to 3584)	2690 (2634 to 2749)	761 (731 to 792)	62 (47 to 78)	0 (0 to 0)
Italy	2013	2813 (2760 to 2867)	2137 (2091 to 2182)	618 (594 to 643)	57 (45 to 72)	0 (0 to 0)	3455 (3390 to 3521)	2625 (2568 to 2680)	759 (730 to 789)	70 (55 to 88)	0 (0 to 0)
Italy	2014	2807 (2754 to 2861)	2116 (2071 to 2161)	628 (602 to 653)	63 (50 to 78)	0 (0 to 0)	3447 (3382 to 3514)	2599 (2543 to 2653)	771 (739 to 802)	77 (61 to 96)	0 (0 to 0)
Italy	2015	2826 (2769 to 2880)	2115 (2069 to 2159)	644 (620 to 668)	67 (53 to 84)	0 (0 to 0)	3471 (3400 to 3537)	2597 (2541 to 2652)	791 (761 to 821)	82 (65 to 104)	0 (0 to 0)
Italy	2016	2837 (2784 to 2892)	2110 (2066 to 2157)	656 (631 to 682)	71 (55 to 92)	0 (0 to 0)	3484 (3419 to 3552)	2592 (2538 to 2649)	805 (775 to 837)	87 (67 to 113)	0 (0 to 0)
Italy	2017	2879 (2784 to 2971)	2132 (2069 to 2195)	672 (633 to 711)	75 (56 to 102)	0 (0 to 0)	3535 (3419 to 3649)	2618 (2541 to 2696)	825 (778 to 873)	92 (69 to 125)	0 (0 to 0)
Italy	2018	2906 (2804 to 3021)	2152 (2075 to 2231)	678 (640 to 718)	76 (57 to 103)	0 (0 to 0)	3569 (3444 to 3710)	2643 (2549 to 2740)	833 (786 to 882)	93 (70 to 127)	0 (0 to 0)
Italy	2019	2930 (2807 to 3055)	2174 (2081 to 2267)	680 (641 to 719)	77 (57 to 104)	0 (0 to 0)	3599 (3447 to 3752)	2670 (2556 to 2784)	835 (788 to 884)	94 (71 to 128)	0 (0 to 0)
Italy	2020	2968 (2841 to 3103)	2206 (2102 to 2308)	685 (645 to 724)	78 (58 to 106)	0 (0 to 0)	3645 (3489 to 3810)	2709 (2581 to 2834)	841 (793 to 889)	95 (71 to 130)	0 (0 to 0)

Italy	2021	2995 (2854 to 3142)	2230 (2119 to 2343)	687 (647 to 726)	78 (59 to 107)	0 (0 to 0)	3679 (3505 to 3859)	2738 (2602 to 2877)	844 (795 to 891)	96 (72 to 131)	0 (0 to 0)
Italy	2022	3024 (2877 to 3178)	2255 (2137 to 2378)	689 (650 to 728)	79 (59 to 108)	0 (0 to 0)	3714 (3534 to 3903)	2770 (2625 to 2921)	846 (798 to 894)	97 (73 to 133)	0 (0 to 0)
Italy	2023	3043 (2887 to 3207)	2273 (2142 to 2406)	690 (650 to 729)	80 (60 to 109)	0 (0 to 0)	3737 (3545 to 3938)	2792 (2630 to 2955)	847 (798 to 895)	98 (73 to 134)	0 (0 to 0)
Italy	2024	3060 (2898 to 3228)	2289 (2153 to 2424)	690 (650 to 730)	80 (60 to 109)	0 (0 to 0)	3758 (3560 to 3965)	2812 (2645 to 2977)	848 (799 to 896)	99 (74 to 134)	0 (0 to 0)
Italy	2025	3077 (2901 to 3258)	2305 (2159 to 2451)	691 (651 to 730)	81 (60 to 110)	0 (0 to 0)	3779 (3563 to 4001)	2831 (2652 to 3010)	848 (799 to 897)	99 (74 to 135)	0 (0 to 0)
Italy	2026	3093 (2916 to 3282)	2320 (2168 to 2482)	691 (651 to 731)	81 (61 to 111)	0 (0 to 0)	3798 (3581 to 4031)	2849 (2663 to 3048)	849 (800 to 897)	100 (74 to 136)	0 (0 to 0)
Italy	2027	3104 (2916 to 3296)	2332 (2172 to 2496)	691 (651 to 731)	82 (61 to 111)	0 (0 to 0)	3812 (3582 to 4048)	2864 (2667 to 3066)	848 (799 to 897)	100 (75 to 137)	0 (0 to 0)
Italy	2028	3115 (2931 to 3323)	2343 (2181 to 2518)	690 (649 to 730)	82 (61 to 112)	0 (0 to 0)	3826 (3600 to 4082)	2878 (2678 to 3093)	847 (797 to 897)	101 (75 to 137)	0 (0 to 0)
Italy	2029	3123 (2924 to 3337)	2352 (2181 to 2535)	689 (648 to 730)	82 (61 to 112)	0 (0 to 0)	3836 (3591 to 4099)	2889 (2679 to 3114)	846 (796 to 896)	101 (75 to 138)	0 (0 to 0)
Italy	2030	3130 (2927 to 3355)	2360 (2174 to 2551)	687 (645 to 728)	83 (61 to 113)	0 (0 to 0)	3844 (3594 to 4121)	2899 (2670 to 3133)	844 (793 to 895)	102 (76 to 138)	0 (0 to 0)

Italy	2031	3135 (2914 to 3360)	2367 (2178 to 2562)	685 (643 to 728)	83 (62 to 112)	0 (0 to 0)	3850 (3579 to 4126)	2907 (2674 to 3147)	842 (790 to 894)	102 (76 to 138)	0 (0 to 0)
Italy	2032	3139 (2919 to 3379)	2373 (2173 to 2589)	683 (642 to 726)	83 (62 to 112)	0 (0 to 0)	3855 (3586 to 4149)	2914 (2669 to 3180)	839 (788 to 891)	102 (76 to 138)	0 (0 to 0)
Italy	2033	3140 (2917 to 3385)	2376 (2187 to 2582)	681 (639 to 723)	83 (62 to 112)	0 (0 to 0)	3856 (3582 to 4158)	2918 (2686 to 3172)	836 (785 to 888)	102 (76 to 138)	0 (0 to 0)
Italy	2034	3142 (2924 to 3386)	2380 (2188 to 2596)	678 (636 to 721)	83 (62 to 113)	0 (0 to 0)	3858 (3591 to 4158)	2923 (2687 to 3189)	833 (781 to 885)	102 (76 to 138)	0 (0 to 0)
Italy	2035	3141 (2914 to 3389)	2383 (2187 to 2604)	675 (632 to 719)	83 (62 to 113)	0 (0 to 0)	3857 (3579 to 4162)	2926 (2686 to 3198)	829 (777 to 883)	102 (76 to 139)	0 (0 to 0)
Italy	2036	3141 (2920 to 3402)	2385 (2190 to 2611)	672 (628 to 716)	83 (62 to 113)	0 (0 to 0)	3857 (3587 to 4178)	2930 (2689 to 3207)	825 (772 to 880)	102 (76 to 139)	0 (0 to 0)
Italy	2037	3140 (2903 to 3399)	2388 (2182 to 2616)	668 (625 to 713)	83 (62 to 113)	0 (0 to 0)	3856 (3565 to 4175)	2933 (2680 to 3213)	821 (767 to 876)	102 (76 to 139)	0 (0 to 0)
Italy	2038	3139 (2909 to 3410)	2390 (2180 to 2632)	665 (620 to 710)	83 (62 to 114)	0 (0 to 0)	3855 (3573 to 4188)	2936 (2678 to 3233)	817 (761 to 872)	102 (76 to 140)	0 (0 to 0)
Italy	2039	3135 (2885 to 3412)	2391 (2173 to 2631)	661 (615 to 707)	83 (61 to 114)	0 (0 to 0)	3851 (3543 to 4191)	2936 (2668 to 3231)	812 (755 to 869)	102 (75 to 140)	0 (0 to 0)
Italy	2040	3133 (2892 to 3414)	2392 (2169 to 2640)	658 (611 to 704)	83 (61 to 114)	0 (0 to 0)	3848 (3552 to 4193)	2938 (2664 to 3242)	808 (750 to 865)	102 (75 to 140)	0 (0 to 0)

Italy	2041	3132 (2874 to 3415)	2394 (2165 to 2647)	654 (607 to 702)	84 (61 to 114)	0 (0 to 0)	3847 (3530 to 4194)	2940 (2659 to 3251)	804 (745 to 862)	103 (75 to 140)	0 (0 to 0)
Italy	2042	3135 (2884 to 3423)	2400 (2172 to 2650)	652 (604 to 699)	84 (62 to 115)	0 (0 to 0)	3851 (3543 to 4204)	2947 (2667 to 3255)	801 (742 to 859)	103 (76 to 141)	0 (0 to 0)
Italy	2043	3143 (2870 to 3431)	2409 (2180 to 2669)	650 (601 to 698)	84 (62 to 115)	0 (0 to 0)	3860 (3524 to 4213)	2959 (2678 to 3278)	798 (739 to 857)	103 (76 to 142)	0 (0 to 0)
Italy	2044	3155 (2882 to 3452)	2422 (2168 to 2680)	649 (599 to 697)	84 (62 to 116)	0 (0 to 0)	3875 (3539 to 4239)	2974 (2663 to 3291)	797 (736 to 857)	104 (76 to 143)	0 (0 to 0)
Italy	2045	3168 (2876 to 3463)	2436 (2192 to 2710)	648 (597 to 697)	85 (62 to 117)	0 (0 to 0)	3891 (3532 to 4254)	2991 (2692 to 3328)	796 (734 to 857)	104 (76 to 144)	0 (0 to 0)
Italy	2046	3183 (2907 to 3503)	2450 (2191 to 2734)	647 (596 to 698)	85 (62 to 118)	0 (0 to 0)	3909 (3570 to 4302)	3009 (2691 to 3358)	795 (732 to 857)	105 (77 to 145)	0 (0 to 0)
Italy	2047	3201 (2906 to 3503)	2468 (2218 to 2748)	647 (595 to 698)	86 (63 to 119)	0 (0 to 0)	3932 (3569 to 4302)	3031 (2724 to 3375)	795 (731 to 858)	106 (77 to 147)	0 (0 to 0)
Italy	2048	3220 (2933 to 3549)	2486 (2228 to 2767)	648 (596 to 700)	87 (63 to 120)	0 (0 to 0)	3955 (3602 to 4359)	3053 (2736 to 3398)	795 (732 to 859)	106 (77 to 148)	0 (0 to 0)
Italy	2049	3243 (2940 to 3562)	2508 (2243 to 2793)	648 (596 to 701)	87 (63 to 122)	0 (0 to 0)	3983 (3611 to 4374)	3080 (2754 to 3430)	796 (732 to 861)	107 (78 to 149)	0 (0 to 0)
Italy	2050	3266 (2956 to 3610)	2529 (2247 to 2821)	649 (596 to 703)	88 (64 to 123)	0 (0 to 0)	4012 (3630 to 4434)	3106 (2760 to 3465)	797 (732 to 863)	108 (78 to 151)	0 (0 to 0)

Jamaica	1995	252 (223 to 286)	139 (119 to 161)	62 (47 to 79)	36 (22 to 57)	15 (15 to 15)	446 (395 to 508)	247 (210 to 286)	109 (84 to 140)	64 (39 to 101)	26 (26 to 26)
Jamaica	1996	276 (246 to 311)	160 (139 to 183)	67 (52 to 85)	39 (24 to 60)	10 (10 to 10)	489 (437 to 551)	284 (246 to 325)	118 (92 to 150)	69 (43 to 106)	18 (18 to 18)
Jamaica	1997	285 (255 to 318)	165 (144 to 188)	74 (58 to 91)	39 (24 to 58)	8 (8 to 8)	506 (452 to 564)	293 (256 to 334)	130 (104 to 162)	68 (43 to 103)	14 (14 to 14)
Jamaica	1998	283 (255 to 313)	163 (143 to 184)	74 (59 to 91)	37 (23 to 56)	8 (8 to 8)	501 (453 to 556)	290 (253 to 326)	131 (105 to 161)	66 (42 to 99)	15 (15 to 15)
Jamaica	1999	280 (252 to 310)	157 (139 to 178)	78 (64 to 96)	37 (24 to 56)	7 (7 to 7)	497 (446 to 550)	279 (247 to 316)	139 (113 to 169)	66 (43 to 99)	13 (13 to 13)
Jamaica	2000	275 (249 to 305)	149 (131 to 169)	82 (67 to 99)	37 (24 to 54)	8 (8 to 8)	488 (441 to 542)	263 (232 to 300)	145 (119 to 176)	66 (43 to 96)	14 (14 to 14)
Jamaica	2001	284 (258 to 315)	143 (126 to 163)	84 (69 to 101)	37 (25 to 54)	20 (20 to 20)	503 (458 to 558)	254 (224 to 288)	148 (122 to 178)	66 (44 to 96)	35 (35 to 35)
Jamaica	2002	264 (238 to 294)	145 (128 to 164)	79 (65 to 96)	37 (25 to 54)	3 (3 to 3)	468 (422 to 522)	256 (227 to 290)	140 (115 to 169)	66 (45 to 96)	6 (6 to 6)
Jamaica	2003	268 (241 to 296)	146 (130 to 166)	79 (65 to 97)	38 (26 to 55)	4 (4 to 4)	474 (427 to 525)	259 (231 to 294)	140 (115 to 171)	67 (46 to 98)	8 (8 to 8)
Jamaica	2004	269 (242 to 298)	148 (131 to 168)	78 (63 to 94)	37 (25 to 54)	6 (6 to 6)	476 (429 to 528)	263 (233 to 298)	137 (112 to 167)	66 (44 to 96)	10 (10 to 10)
Jamaica	2005	253 (228 to 282)	138 (122 to 156)	75 (61 to 91)	35 (23 to 52)	6 (6 to 6)	449 (404 to 499)	244 (216 to 276)	133 (108 to 161)	62 (41 to 92)	11 (11 to 11)
Jamaica	2006	267 (240 to 295)	146 (130 to 165)	77 (62 to 94)	35 (23 to 53)	8 (8 to 8)	473 (426 to 523)	260 (231 to 293)	136 (110 to 167)	63 (41 to 93)	14 (14 to 14)
Jamaica	2007	276 (248 to 306)	153 (135 to 172)	81 (66 to 99)	35 (23 to 52)	8 (8 to 8)	490 (440 to 542)	271 (239 to 306)	143 (117 to 175)	63 (40 to 92)	13 (13 to 13)
Jamaica	2008	286 (256 to 316)	161 (142 to 183)	80 (65 to 98)	36 (23 to 53)	8 (8 to 8)	507 (455 to 561)	286 (252 to 324)	142 (116 to 174)	64 (41 to 94)	14 (14 to 14)
Jamaica	2009	290 (260 to 321)	171 (150 to 193)	74 (61 to 91)	38 (24 to 54)	7 (7 to 7)	514 (461 to 568)	303 (266 to 342)	131 (108 to 161)	67 (43 to 96)	12 (12 to 12)

Jamaica	2010	278 (251 to 307)	160 (140 to 180)	67 (54 to 82)	36 (23 to 51)	16 (16 to 16)	493 (446 to 545)	283 (248 to 319)	119 (96 to 145)	63 (42 to 90)	28 (28 to 28)
Jamaica	2011	274 (247 to 304)	163 (144 to 183)	63 (51 to 78)	39 (25 to 55)	10 (10 to 10)	486 (438 to 539)	289 (255 to 324)	112 (90 to 138)	68 (44 to 97)	17 (17 to 17)
Jamaica	2012	269 (242 to 299)	161 (142 to 180)	59 (47 to 73)	43 (28 to 61)	6 (6 to 6)	476 (429 to 531)	286 (253 to 319)	104 (83 to 129)	76 (50 to 108)	10 (10 to 10)
Jamaica	2013	272 (245 to 304)	163 (145 to 182)	56 (44 to 70)	45 (30 to 64)	8 (8 to 8)	483 (434 to 539)	290 (257 to 323)	100 (78 to 124)	80 (54 to 114)	14 (14 to 14)
Jamaica	2014	279 (250 to 310)	170 (151 to 190)	57 (44 to 71)	48 (32 to 68)	5 (5 to 5)	496 (444 to 549)	302 (269 to 337)	100 (79 to 126)	85 (57 to 121)	8 (8 to 8)
Jamaica	2015	297 (265 to 330)	184 (164 to 208)	57 (44 to 72)	51 (34 to 74)	5 (5 to 5)	526 (470 to 586)	327 (291 to 368)	101 (79 to 128)	90 (60 to 130)	8 (8 to 8)
Jamaica	2016	314 (280 to 352)	198 (175 to 225)	58 (45 to 74)	54 (36 to 80)	5 (5 to 5)	558 (497 to 624)	351 (310 to 399)	102 (79 to 130)	95 (63 to 141)	9 (9 to 9)
Jamaica	2017	329 (280 to 389)	207 (181 to 240)	58 (44 to 76)	55 (35 to 84)	8 (8 to 8)	583 (497 to 690)	368 (322 to 426)	103 (79 to 135)	98 (62 to 148)	14 (14 to 14)
Jamaica	2018	342 (292 to 405)	218 (191 to 253)	59 (45 to 77)	57 (36 to 87)	7 (7 to 7)	607 (518 to 718)	387 (339 to 448)	105 (80 to 137)	102 (65 to 154)	13 (13 to 13)
Jamaica	2019	345 (294 to 407)	220 (192 to 254)	59 (45 to 78)	58 (37 to 87)	8 (8 to 8)	611 (522 to 722)	390 (340 to 450)	105 (80 to 138)	102 (65 to 155)	14 (14 to 14)
Jamaica	2020	349 (297 to 413)	223 (193 to 257)	60 (46 to 78)	58 (37 to 89)	8 (8 to 8)	619 (527 to 732)	395 (343 to 456)	106 (81 to 139)	104 (66 to 157)	14 (13 to 15)
Jamaica	2021	355 (301 to 421)	227 (195 to 261)	61 (46 to 79)	59 (38 to 90)	8 (8 to 9)	629 (534 to 747)	402 (346 to 464)	107 (82 to 140)	105 (67 to 160)	14 (14 to 16)
Jamaica	2022	360 (305 to 431)	231 (198 to 270)	61 (46 to 80)	60 (38 to 91)	8 (8 to 9)	639 (540 to 764)	409 (351 to 479)	108 (82 to 141)	107 (68 to 162)	15 (14 to 16)
Jamaica	2023	365 (307 to 439)	235 (199 to 275)	61 (47 to 80)	61 (39 to 92)	9 (8 to 10)	648 (544 to 778)	416 (353 to 488)	109 (83 to 142)	108 (69 to 164)	15 (14 to 17)
Jamaica	2024	370 (309 to 445)	238 (201 to 282)	61 (47 to 80)	62 (39 to 93)	9 (8 to 10)	657 (548 to 789)	423 (356 to 500)	109 (83 to 142)	109 (70 to 165)	16 (14 to 18)

Jamaica	2025	375 (312 to 452)	242 (202 to 288)	62 (47 to 80)	62 (40 to 94)	9 (8 to 10)	665 (554 to 801)	429 (358 to 510)	109 (83 to 142)	111 (70 to 167)	16 (14 to 18)
Jamaica	2026	379 (314 to 458)	245 (204 to 292)	62 (47 to 80)	63 (40 to 95)	9 (8 to 11)	673 (557 to 812)	435 (362 to 518)	109 (83 to 142)	112 (71 to 169)	16 (15 to 19)
Jamaica	2027	383 (316 to 464)	248 (206 to 295)	62 (47 to 81)	64 (41 to 96)	10 (8 to 11)	680 (561 to 823)	441 (365 to 522)	110 (83 to 143)	113 (72 to 170)	17 (15 to 20)
Jamaica	2028	387 (318 to 470)	251 (206 to 300)	62 (47 to 81)	64 (41 to 97)	10 (9 to 11)	686 (564 to 833)	445 (366 to 532)	110 (84 to 143)	114 (72 to 172)	17 (15 to 20)
Jamaica	2029	391 (321 to 476)	254 (208 to 305)	62 (47 to 81)	65 (41 to 97)	10 (9 to 12)	693 (569 to 844)	451 (370 to 541)	110 (84 to 143)	115 (73 to 173)	18 (15 to 21)
Jamaica	2030	395 (322 to 482)	257 (209 to 312)	62 (47 to 81)	65 (41 to 98)	10 (9 to 12)	700 (571 to 855)	456 (370 to 553)	110 (84 to 144)	116 (74 to 174)	18 (16 to 22)
Jamaica	2031	398 (324 to 489)	260 (210 to 317)	62 (47 to 81)	66 (42 to 99)	11 (9 to 13)	706 (574 to 867)	461 (373 to 561)	110 (84 to 144)	117 (74 to 175)	19 (16 to 22)
Jamaica	2032	402 (326 to 494)	263 (211 to 320)	62 (47 to 81)	66 (42 to 99)	11 (9 to 13)	713 (578 to 877)	466 (374 to 568)	110 (84 to 144)	118 (74 to 176)	19 (17 to 23)
Jamaica	2033	405 (328 to 500)	265 (211 to 326)	62 (47 to 81)	67 (42 to 100)	11 (10 to 14)	719 (582 to 886)	470 (374 to 579)	110 (84 to 144)	118 (75 to 177)	20 (17 to 24)
Jamaica	2034	408 (327 to 507)	268 (211 to 333)	62 (47 to 81)	67 (42 to 101)	12 (10 to 14)	724 (579 to 900)	475 (375 to 590)	110 (84 to 144)	119 (75 to 179)	21 (17 to 25)
Jamaica	2035	411 (328 to 512)	270 (212 to 337)	62 (47 to 81)	68 (43 to 101)	12 (10 to 15)	729 (582 to 908)	478 (376 to 597)	110 (83 to 144)	120 (75 to 180)	21 (18 to 26)
Jamaica	2036	413 (329 to 513)	271 (213 to 338)	62 (47 to 81)	68 (43 to 102)	12 (10 to 15)	733 (583 to 910)	481 (378 to 599)	110 (83 to 143)	120 (76 to 180)	22 (18 to 27)
Jamaica	2037	416 (329 to 515)	274 (214 to 340)	62 (47 to 81)	68 (43 to 103)	13 (10 to 16)	739 (584 to 913)	485 (380 to 602)	109 (83 to 143)	121 (76 to 182)	23 (19 to 28)
Jamaica	2038	420 (331 to 521)	276 (215 to 343)	62 (47 to 81)	69 (43 to 103)	13 (11 to 17)	744 (586 to 924)	489 (381 to 608)	109 (83 to 143)	122 (77 to 183)	23 (19 to 29)
Jamaica	2039	423 (332 to 529)	278 (215 to 349)	62 (47 to 81)	69 (43 to 104)	14 (11 to 17)	750 (589 to 937)	494 (382 to 620)	109 (83 to 143)	123 (77 to 185)	24 (20 to 30)

Jamaica	2040	426 (334 to 533)	281 (215 to 353)	62 (47 to 81)	70 (44 to 105)	14 (11 to 18)	756 (593 to 945)	498 (382 to 625)	110 (83 to 143)	123 (78 to 187)	25 (20 to 31)
Jamaica	2041	430 (334 to 539)	283 (217 to 356)	62 (47 to 81)	70 (44 to 106)	14 (12 to 18)	762 (592 to 955)	502 (385 to 632)	110 (83 to 144)	124 (78 to 188)	26 (21 to 32)
Jamaica	2042	433 (335 to 543)	286 (216 to 363)	62 (47 to 81)	71 (44 to 107)	15 (12 to 19)	768 (594 to 963)	507 (384 to 643)	110 (83 to 144)	125 (78 to 190)	26 (22 to 34)
Jamaica	2043	437 (338 to 549)	289 (219 to 365)	62 (47 to 81)	71 (44 to 108)	15 (12 to 20)	775 (599 to 973)	512 (388 to 648)	110 (83 to 144)	126 (79 to 192)	27 (22 to 35)
Jamaica	2044	441 (340 to 555)	291 (220 to 373)	62 (47 to 81)	72 (45 to 109)	16 (13 to 21)	782 (603 to 984)	517 (390 to 661)	110 (83 to 144)	127 (79 to 194)	28 (23 to 37)
Jamaica	2045	444 (339 to 557)	293 (220 to 370)	62 (47 to 81)	72 (45 to 110)	16 (13 to 21)	787 (602 to 988)	520 (391 to 655)	110 (83 to 144)	128 (79 to 195)	29 (23 to 38)
Jamaica	2046	447 (343 to 560)	296 (218 to 373)	62 (47 to 82)	72 (45 to 111)	17 (14 to 22)	793 (607 to 993)	525 (387 to 662)	110 (83 to 145)	128 (80 to 197)	30 (24 to 38)
Jamaica	2047	451 (346 to 568)	298 (222 to 378)	62 (47 to 81)	73 (45 to 112)	18 (14 to 23)	799 (614 to 1007)	529 (393 to 670)	110 (83 to 144)	129 (80 to 198)	31 (25 to 41)
Jamaica	2048	453 (350 to 571)	300 (222 to 382)	62 (47 to 81)	73 (45 to 113)	18 (14 to 24)	804 (621 to 1013)	532 (393 to 677)	109 (83 to 144)	130 (80 to 200)	32 (26 to 42)
Jamaica	2049	456 (352 to 579)	302 (226 to 386)	62 (47 to 81)	74 (46 to 113)	19 (15 to 25)	809 (624 to 1027)	536 (400 to 685)	109 (82 to 144)	130 (81 to 201)	33 (26 to 44)
Jamaica	2050	459 (354 to 581)	304 (228 to 390)	61 (46 to 81)	74 (46 to 114)	19 (16 to 26)	814 (627 to 1030)	540 (404 to 692)	109 (82 to 143)	131 (81 to 203)	34 (28 to 46)
Japan	1995	1898 (1798 to 1999)	1538 (1447 to 1633)	289 (261 to 319)	71 (52 to 96)	0 (0 to 0)	2116 (2005 to 2229)	1715 (1614 to 1821)	322 (291 to 356)	80 (58 to 107)	0 (0 to 0)
Japan	1996	1977 (1901 to 2055)	1598 (1526 to 1671)	304 (282 to 329)	74 (56 to 96)	0 (0 to 0)	2205 (2120 to 2292)	1782 (1702 to 1863)	339 (315 to 367)	83 (63 to 107)	0 (0 to 0)
Japan	1997	2026 (1955 to 2104)	1627 (1562 to 1695)	324 (302 to 347)	75 (59 to 94)	0 (0 to 0)	2260 (2180 to 2346)	1815 (1743 to 1890)	361 (337 to 386)	84 (65 to 105)	0 (0 to 0)

Japan	1998	2193 (2119 to 2272)	1761 (1694 to 1836)	352 (331 to 373)	80 (64 to 99)	0 (0 to 0)	2446 (2363 to 2534)	1964 (1889 to 2048)	392 (369 to 416)	89 (71 to 111)	0 (0 to 0)
Japan	1999	2231 (2165 to 2307)	1786 (1725 to 1859)	365 (344 to 385)	80 (64 to 97)	0 (0 to 0)	2488 (2414 to 2573)	1992 (1923 to 2073)	407 (384 to 429)	89 (72 to 108)	0 (0 to 0)
Japan	2000	2411 (2339 to 2487)	1938 (1871 to 2011)	388 (367 to 408)	85 (69 to 101)	0 (0 to 0)	2688 (2608 to 2774)	2162 (2087 to 2243)	432 (409 to 455)	94 (77 to 112)	0 (0 to 0)
Japan	2001	2498 (2429 to 2578)	2012 (1942 to 2086)	402 (382 to 423)	84 (69 to 99)	0 (0 to 0)	2786 (2709 to 2875)	2244 (2166 to 2326)	448 (426 to 471)	94 (77 to 110)	0 (0 to 0)
Japan	2002	2590 (2520 to 2673)	2087 (2018 to 2163)	419 (399 to 439)	85 (70 to 99)	0 (0 to 0)	2888 (2811 to 2981)	2327 (2251 to 2412)	467 (445 to 490)	94 (78 to 110)	0 (0 to 0)
Japan	2003	2668 (2592 to 2752)	2146 (2075 to 2227)	437 (417 to 459)	85 (71 to 99)	0 (0 to 0)	2976 (2891 to 3069)	2394 (2314 to 2484)	487 (465 to 511)	95 (79 to 111)	0 (0 to 0)
Japan	2004	2708 (2627 to 2789)	2178 (2100 to 2253)	445 (425 to 466)	85 (73 to 100)	0 (0 to 0)	3020 (2930 to 3111)	2429 (2342 to 2513)	496 (474 to 520)	95 (81 to 111)	0 (0 to 0)
Japan	2005	2793 (2715 to 2872)	2252 (2174 to 2325)	452 (431 to 473)	89 (76 to 103)	0 (0 to 0)	3114 (3027 to 3203)	2511 (2424 to 2593)	504 (480 to 528)	99 (85 to 115)	0 (0 to 0)
Japan	2006	2843 (2764 to 2919)	2292 (2219 to 2362)	458 (438 to 479)	93 (79 to 109)	0 (0 to 0)	3170 (3082 to 3255)	2556 (2474 to 2634)	510 (488 to 534)	104 (88 to 121)	0 (0 to 0)
Japan	2007	2915 (2840 to 2992)	2357 (2285 to 2430)	459 (439 to 480)	98 (84 to 114)	0 (0 to 0)	3250 (3167 to 3336)	2628 (2549 to 2710)	512 (490 to 535)	110 (94 to 127)	0 (0 to 0)

Japan	2008	2999 (2922 to 3086)	2432 (2357 to 2511)	462 (442 to 483)	105 (89 to 120)	0 (0 to 0)	3344 (3258 to 3441)	2712 (2629 to 2800)	515 (493 to 539)	117 (99 to 134)	0 (0 to 0)
Japan	2009	3207 (3128 to 3295)	2620 (2547 to 2699)	475 (454 to 496)	113 (97 to 129)	0 (0 to 0)	3577 (3489 to 3675)	2921 (2841 to 3010)	530 (506 to 554)	126 (108 to 144)	0 (0 to 0)
Japan	2010	3417 (3336 to 3499)	2816 (2739 to 2892)	484 (464 to 506)	117 (102 to 134)	0 (0 to 0)	3810 (3721 to 3902)	3140 (3055 to 3225)	540 (518 to 565)	130 (113 to 149)	0 (0 to 0)
Japan	2011	3709 (3632 to 3790)	3090 (3013 to 3168)	499 (478 to 520)	120 (103 to 138)	0 (0 to 0)	4137 (4051 to 4226)	3446 (3360 to 3533)	556 (533 to 580)	134 (115 to 154)	0 (0 to 0)
Japan	2012	3931 (3851 to 4012)	3297 (3221 to 3372)	511 (489 to 533)	123 (106 to 142)	0 (0 to 0)	4384 (4295 to 4475)	3677 (3592 to 3761)	570 (545 to 595)	137 (118 to 158)	0 (0 to 0)
Japan	2013	4082 (4001 to 4164)	3436 (3358 to 3511)	520 (498 to 544)	125 (108 to 145)	0 (0 to 0)	4552 (4462 to 4643)	3832 (3745 to 3916)	580 (555 to 606)	140 (120 to 162)	0 (0 to 0)
Japan	2014	4112 (4030 to 4200)	3461 (3378 to 3539)	526 (503 to 550)	125 (109 to 144)	0 (0 to 0)	4586 (4494 to 4684)	3860 (3768 to 3947)	587 (560 to 613)	140 (121 to 160)	0 (0 to 0)
Japan	2015	4146 (4057 to 4234)	3486 (3405 to 3573)	534 (511 to 557)	126 (108 to 145)	0 (0 to 0)	4624 (4525 to 4722)	3888 (3797 to 3985)	595 (570 to 622)	140 (121 to 161)	0 (0 to 0)
Japan	2016	4201 (4114 to 4297)	3532 (3449 to 3623)	541 (518 to 566)	128 (109 to 151)	0 (0 to 0)	4685 (4588 to 4792)	3939 (3847 to 4040)	603 (578 to 631)	143 (122 to 168)	0 (0 to 0)
Japan	2017	4290 (4148 to 4438)	3606 (3495 to 3721)	553 (520 to 588)	131 (107 to 161)	0 (0 to 0)	4784 (4626 to 4950)	4021 (3898 to 4150)	616 (580 to 656)	147 (120 to 180)	0 (0 to 0)

Japan	2018	4319 (4163 to 4491)	3629 (3504 to 3761)	558 (525 to 594)	133 (108 to 163)	0 (0 to 0)	4817 (4642 to 5009)	4047 (3908 to 4194)	622 (586 to 662)	148 (121 to 182)	0 (0 to 0)
Japan	2019	4401 (4242 to 4566)	3702 (3570 to 3839)	564 (531 to 601)	135 (111 to 166)	0 (0 to 0)	4909 (4731 to 5092)	4129 (3981 to 4281)	629 (592 to 670)	151 (123 to 185)	0 (0 to 0)
Japan	2020	4489 (4327 to 4657)	3783 (3641 to 3921)	568 (534 to 606)	138 (113 to 169)	0 (0 to 0)	5006 (4826 to 5194)	4219 (4060 to 4373)	634 (596 to 676)	154 (126 to 188)	0 (0 to 0)
Japan	2021	4573 (4400 to 4754)	3859 (3712 to 4003)	573 (539 to 611)	140 (115 to 172)	0 (0 to 0)	5100 (4907 to 5302)	4304 (4139 to 4464)	640 (601 to 682)	156 (128 to 192)	0 (0 to 0)
Japan	2022	4662 (4478 to 4846)	3941 (3783 to 4095)	578 (543 to 617)	143 (117 to 175)	0 (0 to 0)	5200 (4994 to 5404)	4395 (4219 to 4567)	645 (606 to 688)	159 (131 to 195)	0 (0 to 0)
Japan	2023	4728 (4527 to 4923)	4004 (3836 to 4162)	580 (545 to 619)	145 (119 to 177)	0 (0 to 0)	5273 (5049 to 5490)	4465 (4278 to 4642)	647 (607 to 690)	161 (132 to 197)	0 (0 to 0)
Japan	2024	4796 (4602 to 4999)	4067 (3892 to 4234)	582 (547 to 621)	147 (120 to 179)	0 (0 to 0)	5348 (5132 to 5576)	4535 (4340 to 4722)	649 (610 to 692)	164 (134 to 200)	0 (0 to 0)
Japan	2025	4864 (4651 to 5076)	4131 (3956 to 4301)	584 (549 to 623)	149 (122 to 182)	0 (0 to 0)	5424 (5187 to 5661)	4607 (4412 to 4797)	652 (612 to 695)	166 (136 to 203)	0 (0 to 0)
Japan	2026	4941 (4735 to 5156)	4202 (4020 to 4383)	588 (552 to 627)	151 (123 to 184)	0 (0 to 0)	5510 (5281 to 5750)	4686 (4484 to 4888)	655 (615 to 699)	168 (137 to 205)	0 (0 to 0)
Japan	2027	5028 (4809 to 5260)	4283 (4086 to 4478)	592 (556 to 631)	153 (125 to 187)	0 (0 to 0)	5607 (5363 to 5866)	4776 (4557 to 4994)	660 (620 to 704)	171 (140 to 209)	0 (0 to 0)

Japan	2028	5124 (4886 to 5369)	4371 (4157 to 4586)	597 (560 to 637)	156 (127 to 191)	0 (0 to 0)	5714 (5450 to 5987)	4874 (4637 to 5115)	666 (625 to 711)	174 (142 to 213)	0 (0 to 0)
Japan	2029	5222 (4959 to 5487)	4461 (4225 to 4691)	602 (565 to 643)	159 (130 to 194)	0 (0 to 0)	5824 (5531 to 6119)	4975 (4712 to 5231)	671 (630 to 717)	177 (145 to 216)	0 (0 to 0)
Japan	2030	5321 (5037 to 5597)	4552 (4302 to 4793)	607 (569 to 649)	162 (133 to 197)	0 (0 to 0)	5934 (5617 to 6242)	5077 (4798 to 5345)	677 (635 to 724)	181 (148 to 220)	0 (0 to 0)
Japan	2031	5418 (5118 to 5720)	4641 (4372 to 4904)	612 (573 to 654)	165 (135 to 201)	0 (0 to 0)	6042 (5707 to 6379)	5176 (4876 to 5469)	682 (639 to 729)	184 (151 to 224)	0 (0 to 0)
Japan	2032	5512 (5188 to 5838)	4729 (4439 to 5019)	616 (576 to 659)	167 (137 to 204)	0 (0 to 0)	6148 (5786 to 6510)	5274 (4951 to 5597)	687 (642 to 735)	187 (153 to 227)	0 (0 to 0)
Japan	2033	5606 (5271 to 5957)	4816 (4507 to 5126)	620 (578 to 664)	170 (139 to 207)	0 (0 to 0)	6252 (5879 to 6643)	5371 (5026 to 5717)	691 (645 to 741)	190 (155 to 231)	0 (0 to 0)
Japan	2034	5697 (5336 to 6063)	4901 (4577 to 5234)	623 (581 to 667)	172 (141 to 210)	0 (0 to 0)	6353 (5951 to 6761)	5466 (5105 to 5838)	695 (647 to 744)	192 (157 to 235)	0 (0 to 0)
Japan	2035	5783 (5402 to 6161)	4983 (4630 to 5329)	625 (582 to 671)	175 (143 to 213)	0 (0 to 0)	6450 (6024 to 6871)	5557 (5163 to 5943)	698 (649 to 748)	195 (159 to 237)	0 (0 to 0)
Japan	2036	5871 (5474 to 6265)	5065 (4704 to 5434)	628 (585 to 674)	177 (145 to 216)	0 (0 to 0)	6547 (6104 to 6987)	5649 (5246 to 6060)	700 (652 to 752)	198 (161 to 240)	0 (0 to 0)
Japan	2037	5954 (5548 to 6374)	5144 (4763 to 5530)	630 (587 to 678)	179 (146 to 219)	0 (0 to 0)	6640 (6187 to 7109)	5736 (5311 to 6168)	703 (655 to 756)	200 (163 to 244)	0 (0 to 0)

Japan	2038	6034 (5608 to 6471)	5219 (4821 to 5613)	633 (588 to 681)	182 (148 to 221)	0 (0 to 0)	6729 (6255 to 7217)	5821 (5376 to 6260)	705 (656 to 759)	203 (165 to 247)	0 (0 to 0)
Japan	2039	6111 (5669 to 6565)	5293 (4874 to 5712)	634 (589 to 683)	184 (150 to 224)	0 (0 to 0)	6816 (6322 to 7321)	5903 (5436 to 6370)	707 (657 to 762)	205 (167 to 250)	0 (0 to 0)
Japan	2040	6187 (5721 to 6669)	5365 (4931 to 5784)	636 (590 to 686)	186 (151 to 227)	0 (0 to 0)	6900 (6381 to 7437)	5984 (5499 to 6450)	709 (658 to 765)	207 (169 to 253)	0 (0 to 0)
Japan	2041	6263 (5776 to 6762)	5437 (4989 to 5879)	638 (592 to 690)	188 (153 to 230)	0 (0 to 0)	6985 (6441 to 7541)	6064 (5564 to 6557)	712 (660 to 770)	210 (170 to 256)	0 (0 to 0)
Japan	2042	6342 (5843 to 6841)	5511 (5046 to 5977)	641 (593 to 694)	190 (154 to 232)	0 (0 to 0)	7073 (6517 to 7629)	6146 (5627 to 6665)	715 (662 to 774)	212 (172 to 259)	0 (0 to 0)
Japan	2043	6422 (5914 to 6947)	5585 (5111 to 6057)	644 (596 to 698)	193 (156 to 236)	0 (0 to 0)	7162 (6596 to 7748)	6228 (5700 to 6755)	719 (664 to 779)	215 (174 to 263)	0 (0 to 0)
Japan	2044	6502 (5973 to 7030)	5659 (5169 to 6148)	648 (599 to 704)	195 (158 to 239)	0 (0 to 0)	7252 (6662 to 7840)	6311 (5764 to 6857)	723 (668 to 785)	217 (176 to 267)	0 (0 to 0)
Japan	2045	6583 (6045 to 7133)	5733 (5226 to 6237)	652 (602 to 710)	198 (159 to 243)	0 (0 to 0)	7341 (6741 to 7955)	6393 (5828 to 6956)	728 (671 to 792)	220 (178 to 271)	0 (0 to 0)
Japan	2046	6664 (6100 to 7229)	5807 (5293 to 6315)	657 (605 to 717)	200 (161 to 246)	0 (0 to 0)	7432 (6803 to 8062)	6476 (5903 to 7043)	733 (674 to 799)	223 (180 to 274)	0 (0 to 0)
Japan	2047	6746 (6172 to 7320)	5882 (5345 to 6396)	662 (608 to 724)	203 (163 to 249)	0 (0 to 0)	7524 (6883 to 8163)	6560 (5961 to 7133)	738 (678 to 807)	226 (182 to 278)	0 (0 to 0)

Japan	2048	6829 (6244 to 7406)	5957 (5422 to 6487)	667 (611 to 731)	205 (165 to 253)	0 (0 to 0)	7616 (6964 to 8259)	6644 (6046 to 7235)	744 (682 to 815)	229 (184 to 282)	0 (0 to 0)
Japan	2049	6914 (6327 to 7507)	6034 (5492 to 6588)	672 (616 to 738)	208 (167 to 257)	0 (0 to 0)	7711 (7056 to 8373)	6729 (6124 to 7347)	750 (687 to 822)	232 (186 to 286)	0 (0 to 0)
Japan	2050	7000 (6400 to 7618)	6111 (5553 to 6672)	677 (621 to 744)	211 (169 to 261)	0 (0 to 0)	7806 (7137 to 8496)	6816 (6193 to 7441)	756 (692 to 830)	235 (188 to 291)	0 (0 to 0)
Jordan	1995	298 (268 to 336)	188 (163 to 216)	76 (60 to 95)	28 (16 to 44)	6 (6 to 6)	653 (588 to 738)	413 (359 to 474)	166 (132 to 208)	61 (36 to 97)	13 (13 to 13)
Jordan	1996	302 (273 to 337)	188 (166 to 213)	83 (67 to 101)	29 (17 to 45)	3 (3 to 3)	663 (599 to 741)	412 (365 to 467)	182 (148 to 222)	63 (38 to 98)	6 (6 to 6)
Jordan	1997	311 (282 to 343)	185 (165 to 206)	93 (77 to 111)	29 (19 to 45)	5 (5 to 5)	683 (619 to 753)	405 (362 to 453)	203 (168 to 243)	65 (41 to 99)	10 (10 to 10)
Jordan	1998	332 (302 to 363)	188 (169 to 207)	105 (89 to 123)	32 (21 to 47)	6 (6 to 6)	728 (662 to 796)	412 (370 to 455)	231 (195 to 271)	70 (45 to 104)	14 (14 to 14)
Jordan	1999	351 (322 to 382)	183 (164 to 201)	121 (102 to 140)	35 (23 to 50)	12 (12 to 12)	770 (706 to 839)	402 (360 to 442)	266 (224 to 306)	76 (50 to 111)	27 (27 to 27)
Jordan	2000	379 (348 to 412)	185 (167 to 204)	142 (121 to 163)	42 (28 to 58)	10 (10 to 10)	833 (764 to 903)	407 (366 to 447)	312 (265 to 358)	91 (61 to 128)	22 (22 to 22)
Jordan	2001	395 (364 to 429)	186 (169 to 205)	157 (134 to 179)	43 (29 to 61)	9 (9 to 9)	868 (799 to 943)	409 (370 to 450)	344 (295 to 392)	95 (63 to 135)	20 (20 to 20)
Jordan	2002	411 (379 to 446)	193 (175 to 213)	168 (145 to 192)	41 (27 to 58)	10 (10 to 10)	903 (831 to 978)	423 (384 to 467)	369 (317 to 421)	90 (60 to 128)	22 (22 to 22)
Jordan	2003	415 (381 to 449)	196 (177 to 216)	173 (150 to 197)	35 (23 to 50)	10 (10 to 10)	911 (837 to 985)	431 (388 to 474)	380 (328 to 433)	77 (51 to 111)	22 (22 to 22)
Jordan	2004	402 (369 to 435)	195 (176 to 214)	169 (147 to 194)	29 (19 to 43)	8 (8 to 8)	881 (811 to 955)	428 (387 to 471)	372 (322 to 425)	64 (42 to 95)	18 (18 to 18)
Jordan	2005	403 (370 to 437)	209 (189 to 231)	163 (142 to 188)	26 (16 to 39)	5 (5 to 5)	885 (813 to 959)	459 (415 to 507)	359 (311 to 413)	57 (36 to 86)	10 (10 to 10)

Jordan	2006	386 (355 to 419)	211 (191 to 233)	149 (129 to 172)	22 (14 to 35)	3 (3 to 3)	847 (780 to 919)	463 (419 to 512)	328 (283 to 378)	49 (31 to 76)	7 (7 to 7)
Jordan	2007	406 (375 to 442)	238 (216 to 263)	142 (121 to 165)	23 (15 to 35)	3 (3 to 3)	892 (823 to 970)	523 (474 to 577)	312 (266 to 362)	51 (32 to 76)	6 (6 to 6)
Jordan	2008	423 (390 to 459)	261 (238 to 287)	130 (111 to 153)	26 (16 to 39)	6 (6 to 6)	929 (855 to 1007)	572 (522 to 631)	286 (244 to 336)	58 (36 to 86)	13 (13 to 13)
Jordan	2009	438 (403 to 475)	286 (261 to 313)	116 (98 to 137)	30 (19 to 45)	6 (6 to 6)	960 (886 to 1043)	627 (572 to 688)	254 (215 to 301)	66 (42 to 99)	14 (14 to 14)
Jordan	2010	395 (365 to 429)	262 (239 to 287)	98 (82 to 116)	29 (19 to 44)	6 (6 to 6)	866 (801 to 942)	575 (525 to 630)	215 (181 to 254)	64 (41 to 97)	13 (13 to 13)
Jordan	2011	394 (363 to 428)	264 (241 to 289)	91 (76 to 107)	33 (21 to 49)	6 (6 to 6)	864 (796 to 939)	580 (529 to 634)	199 (167 to 235)	72 (46 to 107)	13 (13 to 13)
Jordan	2012	372 (344 to 404)	243 (220 to 266)	84 (70 to 99)	34 (21 to 49)	12 (12 to 12)	817 (754 to 886)	533 (483 to 584)	184 (154 to 218)	74 (47 to 109)	26 (26 to 26)
Jordan	2013	331 (304 to 360)	207 (187 to 228)	77 (65 to 92)	33 (22 to 49)	13 (13 to 13)	727 (668 to 790)	454 (410 to 499)	170 (142 to 203)	73 (48 to 108)	30 (30 to 30)
Jordan	2014	328 (299 to 359)	200 (181 to 222)	80 (66 to 95)	38 (25 to 56)	10 (10 to 10)	719 (656 to 787)	440 (397 to 488)	175 (145 to 209)	83 (55 to 123)	21 (21 to 21)
Jordan	2015	305 (275 to 340)	176 (157 to 197)	84 (69 to 103)	43 (28 to 64)	2 (2 to 2)	670 (605 to 746)	386 (345 to 433)	185 (151 to 226)	94 (62 to 141)	5 (5 to 5)
Jordan	2016	284 (253 to 320)	152 (134 to 172)	85 (69 to 105)	44 (28 to 65)	4 (4 to 4)	624 (556 to 702)	334 (294 to 378)	186 (152 to 230)	96 (62 to 144)	8 (8 to 8)
Jordan	2017	293 (251 to 344)	149 (129 to 172)	90 (72 to 111)	47 (30 to 73)	7 (7 to 7)	643 (551 to 754)	328 (283 to 378)	198 (158 to 244)	103 (65 to 160)	15 (15 to 15)
Jordan	2018	299 (256 to 354)	154 (133 to 179)	91 (73 to 112)	48 (30 to 74)	6 (6 to 6)	657 (562 to 776)	339 (291 to 393)	199 (160 to 247)	105 (66 to 163)	13 (13 to 13)
Jordan	2019	298 (255 to 352)	153 (132 to 179)	91 (73 to 113)	48 (30 to 74)	6 (6 to 6)	655 (560 to 772)	336 (289 to 393)	201 (161 to 248)	105 (65 to 163)	13 (13 to 13)
Jordan	2020	304 (259 to 360)	156 (133 to 184)	92 (74 to 114)	49 (30 to 75)	6 (6 to 7)	666 (568 to 790)	343 (292 to 403)	203 (162 to 251)	107 (67 to 166)	14 (13 to 14)

Jordan	2021	309 (263 to 364)	160 (135 to 188)	93 (75 to 115)	50 (31 to 77)	6 (6 to 7)	678 (576 to 800)	351 (296 to 413)	204 (164 to 252)	109 (68 to 169)	14 (13 to 15)
Jordan	2022	315 (268 to 377)	164 (139 to 193)	94 (75 to 116)	51 (32 to 79)	7 (6 to 7)	690 (588 to 827)	359 (305 to 425)	206 (165 to 255)	111 (70 to 173)	14 (13 to 16)
Jordan	2023	320 (272 to 381)	168 (142 to 198)	94 (76 to 117)	51 (32 to 80)	7 (6 to 7)	703 (597 to 837)	368 (311 to 434)	207 (166 to 257)	113 (71 to 175)	15 (13 to 16)
Jordan	2024	324 (273 to 390)	170 (143 to 201)	95 (76 to 118)	52 (33 to 81)	7 (6 to 8)	711 (600 to 857)	373 (313 to 441)	209 (168 to 259)	114 (72 to 178)	15 (13 to 17)
Jordan	2025	329 (279 to 392)	173 (145 to 205)	96 (77 to 119)	53 (33 to 83)	7 (6 to 8)	723 (611 to 860)	380 (319 to 451)	211 (169 to 262)	116 (73 to 181)	15 (13 to 17)
Jordan	2026	334 (281 to 401)	176 (147 to 208)	97 (78 to 121)	54 (34 to 84)	7 (6 to 8)	732 (617 to 881)	385 (322 to 456)	213 (171 to 265)	119 (74 to 185)	15 (13 to 17)
Jordan	2027	337 (285 to 402)	178 (148 to 211)	98 (79 to 122)	55 (34 to 85)	7 (6 to 8)	741 (625 to 882)	390 (324 to 463)	215 (173 to 267)	121 (75 to 187)	15 (13 to 17)
Jordan	2028	341 (287 to 413)	180 (148 to 214)	99 (79 to 123)	56 (35 to 87)	7 (6 to 8)	749 (629 to 906)	394 (326 to 470)	217 (174 to 269)	123 (77 to 190)	15 (13 to 18)
Jordan	2029	345 (287 to 414)	182 (149 to 219)	100 (80 to 124)	57 (35 to 88)	7 (6 to 8)	757 (630 to 910)	398 (328 to 481)	219 (175 to 272)	124 (78 to 192)	15 (13 to 18)
Jordan	2030	348 (291 to 427)	183 (150 to 223)	100 (80 to 125)	57 (36 to 89)	7 (6 to 8)	764 (639 to 938)	403 (329 to 491)	220 (176 to 273)	126 (79 to 195)	15 (13 to 18)
Jordan	2031	352 (291 to 426)	185 (150 to 228)	101 (81 to 125)	58 (36 to 90)	7 (6 to 9)	772 (638 to 935)	407 (330 to 500)	221 (177 to 275)	128 (80 to 197)	16 (13 to 19)
Jordan	2032	355 (295 to 439)	188 (151 to 233)	101 (81 to 126)	59 (37 to 91)	7 (6 to 9)	780 (647 to 963)	412 (331 to 511)	223 (178 to 277)	130 (81 to 200)	16 (13 to 19)
Jordan	2033	359 (293 to 438)	190 (151 to 237)	102 (82 to 127)	60 (37 to 92)	7 (6 to 9)	788 (644 to 962)	416 (331 to 520)	224 (179 to 279)	131 (82 to 202)	16 (14 to 20)
Jordan	2034	362 (298 to 449)	192 (151 to 241)	102 (82 to 128)	60 (38 to 93)	7 (6 to 9)	794 (654 to 987)	420 (331 to 530)	225 (180 to 280)	133 (83 to 205)	16 (14 to 20)
Jordan	2035	365 (296 to 452)	193 (151 to 246)	103 (82 to 128)	61 (38 to 95)	8 (6 to 9)	801 (649 to 993)	425 (332 to 541)	226 (180 to 282)	134 (84 to 208)	17 (14 to 20)

Jordan	2036	368 (299 to 463)	195 (151 to 250)	103 (82 to 129)	62 (38 to 96)	8 (6 to 10)	808 (656 to 1017)	429 (332 to 549)	226 (181 to 283)	136 (84 to 210)	17 (14 to 21)
Jordan	2037	371 (298 to 462)	197 (151 to 254)	104 (83 to 130)	63 (39 to 96)	8 (6 to 10)	815 (654 to 1015)	433 (332 to 559)	227 (181 to 285)	137 (85 to 212)	17 (14 to 21)
Jordan	2038	374 (300 to 474)	199 (151 to 259)	104 (83 to 130)	63 (39 to 97)	8 (7 to 10)	821 (659 to 1039)	437 (333 to 568)	228 (182 to 286)	139 (86 to 214)	18 (14 to 22)
Jordan	2039	377 (301 to 470)	201 (152 to 264)	104 (83 to 131)	64 (40 to 98)	8 (7 to 10)	828 (661 to 1031)	441 (334 to 579)	229 (182 to 287)	141 (87 to 216)	18 (14 to 23)
Jordan	2040	380 (304 to 483)	203 (153 to 268)	105 (83 to 131)	65 (40 to 100)	8 (7 to 11)	835 (668 to 1060)	445 (335 to 589)	230 (182 to 288)	142 (88 to 219)	18 (15 to 23)
Jordan	2041	383 (303 to 484)	204 (153 to 273)	105 (83 to 132)	65 (40 to 101)	8 (7 to 11)	841 (665 to 1063)	449 (336 to 600)	230 (183 to 289)	144 (89 to 221)	18 (15 to 24)
Jordan	2042	386 (306 to 497)	206 (153 to 277)	105 (83 to 132)	66 (41 to 102)	9 (7 to 11)	848 (671 to 1090)	453 (337 to 608)	231 (183 to 291)	145 (90 to 223)	19 (15 to 24)
Jordan	2043	390 (306 to 494)	208 (154 to 281)	106 (84 to 133)	67 (41 to 102)	9 (7 to 11)	855 (672 to 1085)	458 (338 to 616)	232 (184 to 292)	147 (90 to 225)	19 (15 to 25)
Jordan	2044	393 (311 to 508)	211 (154 to 285)	106 (84 to 133)	68 (42 to 104)	9 (7 to 12)	863 (682 to 1114)	462 (339 to 625)	233 (184 to 293)	149 (91 to 228)	19 (15 to 26)
Jordan	2045	397 (310 to 510)	213 (155 to 289)	106 (84 to 134)	68 (42 to 105)	9 (7 to 12)	871 (679 to 1118)	467 (341 to 635)	233 (185 to 294)	150 (92 to 232)	20 (15 to 26)
Jordan	2046	400 (314 to 522)	215 (156 to 296)	107 (85 to 135)	69 (43 to 107)	9 (7 to 12)	879 (689 to 1146)	473 (343 to 650)	234 (186 to 296)	152 (93 to 234)	20 (16 to 26)
Jordan	2047	404 (313 to 520)	217 (157 to 300)	107 (85 to 135)	70 (43 to 108)	9 (7 to 12)	887 (686 to 1142)	477 (345 to 658)	235 (186 to 297)	154 (94 to 236)	20 (15 to 27)
Jordan	2048	408 (318 to 534)	220 (159 to 304)	108 (85 to 136)	71 (43 to 109)	9 (7 to 13)	896 (699 to 1172)	483 (348 to 668)	236 (187 to 298)	156 (95 to 240)	21 (16 to 28)
Jordan	2049	412 (319 to 535)	223 (160 to 309)	108 (85 to 136)	72 (44 to 111)	10 (7 to 13)	905 (699 to 1174)	489 (351 to 678)	237 (188 to 299)	158 (96 to 243)	21 (16 to 29)
Jordan	2050	416 (323 to 548)	225 (161 to 312)	109 (86 to 137)	73 (44 to 112)	10 (7 to 13)	914 (709 to 1203)	494 (354 to 685)	238 (188 to 300)	160 (98 to 247)	22 (16 to 29)

Kazakhstan	1995	171 (149 to 199)	94 (78 to 113)	75 (57 to 97)	2 (1 to 4)	0 (0 to 0)	557 (484 to 649)	306 (253 to 368)	243 (186 to 316)	7 (3 to 14)	1 (1 to 1)
Kazakhstan	1996	169 (147 to 195)	93 (78 to 110)	74 (57 to 94)	2 (1 to 4)	1 (1 to 1)	549 (478 to 633)	301 (255 to 356)	239 (187 to 307)	7 (3 to 14)	2 (2 to 2)
Kazakhstan	1997	167 (146 to 191)	92 (78 to 107)	73 (57 to 91)	2 (1 to 4)	1 (1 to 1)	543 (475 to 620)	298 (254 to 347)	236 (187 to 297)	7 (3 to 13)	2 (2 to 2)
Kazakhstan	1998	159 (140 to 180)	86 (73 to 99)	70 (56 to 87)	2 (1 to 3)	1 (1 to 1)	517 (454 to 587)	279 (237 to 322)	229 (181 to 284)	6 (3 to 11)	3 (3 to 3)
Kazakhstan	1999	153 (134 to 172)	81 (69 to 94)	68 (54 to 85)	2 (1 to 3)	2 (2 to 2)	496 (437 to 561)	263 (224 to 305)	223 (176 to 275)	5 (2 to 10)	5 (5 to 5)
Kazakhstan	2000	154 (135 to 173)	82 (70 to 95)	69 (54 to 86)	1 (1 to 2)	1 (1 to 1)	500 (439 to 562)	267 (229 to 309)	224 (177 to 279)	4 (2 to 7)	5 (5 to 5)
Kazakhstan	2001	160 (140 to 180)	88 (75 to 102)	69 (56 to 86)	1 (1 to 2)	2 (2 to 2)	521 (457 to 587)	286 (245 to 331)	226 (181 to 280)	4 (2 to 7)	5 (5 to 5)
Kazakhstan	2002	163 (143 to 183)	90 (78 to 104)	71 (57 to 88)	1 (1 to 2)	1 (1 to 1)	530 (464 to 596)	293 (253 to 338)	231 (186 to 288)	4 (2 to 7)	2 (2 to 2)
Kazakhstan	2003	181 (160 to 204)	103 (89 to 118)	75 (61 to 93)	1 (1 to 2)	1 (1 to 1)	589 (520 to 663)	336 (289 to 383)	245 (200 to 302)	4 (2 to 7)	5 (5 to 5)
Kazakhstan	2004	196 (175 to 219)	117 (101 to 134)	77 (62 to 93)	1 (1 to 2)	1 (1 to 1)	637 (569 to 713)	381 (330 to 435)	249 (203 to 302)	4 (2 to 8)	3 (3 to 3)
Kazakhstan	2005	210 (187 to 235)	133 (116 to 152)	74 (60 to 90)	1 (1 to 3)	1 (1 to 1)	683 (609 to 764)	434 (378 to 496)	242 (196 to 293)	5 (2 to 9)	3 (3 to 3)
Kazakhstan	2006	204 (182 to 227)	134 (117 to 153)	67 (54 to 81)	2 (1 to 3)	1 (1 to 1)	664 (593 to 738)	436 (382 to 499)	219 (176 to 264)	5 (3 to 10)	4 (4 to 4)
Kazakhstan	2007	219 (195 to 243)	151 (133 to 171)	65 (53 to 79)	2 (1 to 4)	1 (1 to 1)	712 (636 to 790)	491 (433 to 558)	212 (171 to 257)	7 (3 to 12)	3 (3 to 3)
Kazakhstan	2008	239 (214 to 265)	171 (151 to 193)	63 (51 to 77)	3 (1 to 5)	2 (2 to 2)	778 (697 to 861)	557 (490 to 628)	206 (165 to 251)	9 (5 to 16)	5 (5 to 5)
Kazakhstan	2009	220 (197 to 244)	155 (136 to 174)	58 (47 to 71)	3 (2 to 6)	3 (3 to 3)	715 (641 to 793)	504 (442 to 565)	190 (152 to 231)	11 (5 to 19)	10 (10 to 10)

Kazakhstan	2010	218 (196 to 242)	151 (133 to 169)	59 (47 to 72)	5 (3 to 9)	3 (3 to 3)	710 (638 to 786)	492 (434 to 551)	191 (154 to 233)	16 (9 to 30)	11 (11 to 11)
Kazakhstan	2011	225 (202 to 250)	155 (136 to 174)	61 (50 to 75)	7 (4 to 12)	2 (2 to 2)	732 (659 to 814)	503 (441 to 566)	199 (161 to 244)	22 (12 to 40)	8 (8 to 8)
Kazakhstan	2012	239 (216 to 266)	161 (142 to 183)	67 (54 to 83)	9 (4 to 15)	3 (3 to 3)	779 (703 to 864)	525 (461 to 594)	218 (175 to 269)	28 (15 to 50)	8 (8 to 8)
Kazakhstan	2013	237 (214 to 263)	156 (137 to 176)	70 (57 to 86)	10 (5 to 17)	2 (2 to 2)	772 (698 to 856)	508 (446 to 574)	227 (184 to 280)	31 (16 to 56)	5 (5 to 5)
Kazakhstan	2014	250 (224 to 278)	164 (145 to 187)	74 (60 to 91)	11 (5 to 20)	1 (1 to 1)	814 (730 to 905)	534 (470 to 607)	240 (195 to 296)	36 (18 to 64)	4 (4 to 4)
Kazakhstan	2015	266 (239 to 296)	170 (148 to 194)	82 (66 to 102)	12 (6 to 22)	1 (1 to 1)	865 (776 to 964)	553 (482 to 631)	268 (216 to 332)	40 (19 to 72)	4 (4 to 4)
Kazakhstan	2016	269 (239 to 304)	165 (143 to 190)	89 (71 to 110)	13 (6 to 22)	2 (2 to 2)	875 (778 to 989)	538 (465 to 620)	288 (229 to 359)	41 (19 to 72)	7 (7 to 7)
Kazakhstan	2017	292 (249 to 340)	181 (155 to 212)	94 (74 to 120)	14 (7 to 25)	2 (2 to 2)	949 (811 to 1105)	590 (505 to 690)	307 (241 to 390)	46 (22 to 82)	6 (6 to 6)
Kazakhstan	2018	260 (224 to 300)	148 (125 to 173)	97 (76 to 124)	12 (6 to 22)	2 (2 to 2)	845 (728 to 975)	482 (408 to 563)	317 (249 to 403)	41 (19 to 72)	5 (5 to 5)
Kazakhstan	2019	275 (234 to 319)	160 (134 to 189)	100 (79 to 127)	13 (6 to 23)	2 (2 to 2)	894 (761 to 1038)	521 (437 to 614)	326 (255 to 413)	42 (20 to 75)	5 (5 to 5)
Kazakhstan	2020	284 (243 to 332)	166 (138 to 197)	103 (81 to 131)	13 (6 to 24)	2 (2 to 2)	925 (789 to 1079)	541 (450 to 641)	336 (263 to 426)	43 (20 to 77)	5 (5 to 6)
Kazakhstan	2021	289 (245 to 339)	169 (141 to 201)	105 (82 to 133)	14 (6 to 24)	2 (2 to 2)	940 (796 to 1102)	550 (459 to 655)	341 (267 to 432)	44 (21 to 79)	6 (5 to 6)
Kazakhstan	2022	294 (248 to 345)	172 (142 to 207)	106 (83 to 135)	14 (6 to 25)	2 (2 to 2)	956 (808 to 1123)	559 (462 to 673)	346 (272 to 438)	45 (21 to 80)	6 (5 to 6)
Kazakhstan	2023	299 (251 to 354)	175 (145 to 212)	108 (85 to 136)	14 (7 to 25)	2 (2 to 2)	972 (817 to 1150)	569 (472 to 690)	351 (276 to 444)	46 (21 to 82)	6 (5 to 7)
Kazakhstan	2024	304 (258 to 360)	178 (147 to 216)	110 (86 to 139)	14 (7 to 26)	2 (2 to 2)	988 (841 to 1172)	579 (477 to 702)	356 (280 to 451)	47 (22 to 83)	6 (5 to 7)

Kazakhstan	2025	309 (260 to 366)	181 (149 to 222)	111 (87 to 141)	15 (7 to 26)	2 (2 to 2)	1005 (845 to 1191)	589 (484 to 721)	362 (284 to 458)	48 (22 to 85)	6 (5 to 7)
Kazakhstan	2026	315 (266 to 373)	185 (151 to 228)	113 (89 to 143)	15 (7 to 27)	2 (2 to 2)	1023 (864 to 1215)	601 (491 to 742)	367 (289 to 465)	49 (23 to 87)	6 (5 to 7)
Kazakhstan	2027	321 (269 to 383)	189 (154 to 231)	115 (90 to 145)	15 (7 to 27)	2 (2 to 2)	1044 (875 to 1246)	614 (501 to 752)	374 (294 to 473)	50 (23 to 89)	6 (5 to 7)
Kazakhstan	2028	328 (276 to 393)	193 (157 to 239)	117 (92 to 148)	16 (7 to 28)	2 (2 to 2)	1067 (897 to 1277)	629 (511 to 779)	381 (300 to 482)	51 (24 to 91)	6 (5 to 7)
Kazakhstan	2029	336 (278 to 405)	198 (159 to 246)	119 (94 to 151)	16 (8 to 29)	2 (2 to 2)	1092 (903 to 1316)	645 (517 to 802)	388 (306 to 493)	53 (25 to 93)	6 (5 to 7)
Kazakhstan	2030	344 (286 to 411)	203 (163 to 254)	122 (96 to 155)	17 (8 to 30)	2 (2 to 2)	1118 (930 to 1339)	661 (529 to 827)	397 (312 to 504)	54 (25 to 96)	6 (5 to 8)
Kazakhstan	2031	352 (289 to 426)	209 (167 to 263)	125 (98 to 158)	17 (8 to 30)	2 (2 to 2)	1146 (940 to 1387)	678 (545 to 855)	405 (318 to 515)	56 (26 to 99)	6 (5 to 8)
Kazakhstan	2032	361 (298 to 433)	214 (170 to 271)	127 (100 to 162)	18 (8 to 31)	2 (2 to 2)	1174 (970 to 1408)	697 (552 to 880)	414 (325 to 527)	57 (27 to 102)	6 (5 to 8)
Kazakhstan	2033	371 (305 to 452)	220 (173 to 279)	130 (102 to 166)	18 (8 to 32)	2 (2 to 2)	1206 (992 to 1469)	717 (562 to 907)	424 (333 to 539)	59 (28 to 106)	7 (6 to 8)
Kazakhstan	2034	381 (316 to 459)	227 (175 to 290)	133 (105 to 170)	19 (9 to 34)	2 (2 to 2)	1240 (1027 to 1492)	738 (570 to 944)	434 (340 to 553)	61 (29 to 109)	7 (5 to 8)

Kazakhstan	2035	392 (319 to 484)	234 (181 to 299)	137 (107 to 174)	19 (9 to 35)	2 (2 to 2)	1275 (1039 to 1574)	761 (589 to 973)	445 (348 to 566)	63 (29 to 113)	7 (5 to 8)
Kazakhstan	2036	402 (335 to 488)	241 (184 to 308)	140 (109 to 178)	20 (9 to 36)	2 (2 to 3)	1309 (1091 to 1587)	783 (600 to 1002)	454 (356 to 579)	65 (30 to 117)	7 (5 to 8)
Kazakhstan	2037	412 (335 to 511)	247 (189 to 319)	142 (112 to 182)	20 (10 to 37)	2 (0 to 3)	1340 (1089 to 1661)	804 (613 to 1038)	463 (363 to 591)	66 (31 to 120)	7 (0 to 8)
Kazakhstan	2038	422 (345 to 517)	253 (193 to 328)	145 (114 to 185)	21 (10 to 38)	2 (0 to 3)	1372 (1123 to 1682)	825 (628 to 1067)	472 (370 to 603)	68 (32 to 124)	6 (0 to 9)
Kazakhstan	2039	431 (350 to 536)	260 (196 to 339)	148 (116 to 189)	22 (10 to 39)	2 (0 to 3)	1403 (1138 to 1744)	845 (639 to 1102)	481 (377 to 615)	70 (33 to 127)	6 (0 to 9)
Kazakhstan	2040	440 (359 to 546)	266 (199 to 344)	151 (118 to 193)	22 (10 to 40)	2 (0 to 3)	1433 (1167 to 1777)	865 (649 to 1121)	490 (383 to 627)	72 (34 to 130)	6 (0 to 9)
Kazakhstan	2041	450 (361 to 565)	272 (203 to 361)	153 (120 to 196)	23 (11 to 41)	2 (0 to 3)	1463 (1175 to 1838)	884 (662 to 1174)	499 (390 to 639)	74 (35 to 133)	6 (0 to 9)
Kazakhstan	2042	459 (369 to 573)	278 (207 to 373)	156 (122 to 200)	23 (11 to 42)	2 (0 to 3)	1494 (1201 to 1863)	906 (672 to 1213)	508 (396 to 650)	76 (35 to 136)	5 (0 to 9)
Kazakhstan	2043	469 (375 to 588)	285 (211 to 377)	159 (124 to 203)	24 (11 to 43)	1 (0 to 3)	1526 (1219 to 1912)	927 (688 to 1228)	517 (402 to 662)	77 (36 to 140)	5 (0 to 9)
Kazakhstan	2044	479 (382 to 601)	292 (217 to 384)	162 (126 to 207)	24 (11 to 44)	1 (0 to 3)	1560 (1241 to 1956)	950 (707 to 1250)	526 (409 to 673)	79 (37 to 143)	4 (0 to 9)

Kazakhstan	2045	490 (388 to 613)	299 (222 to 398)	164 (128 to 210)	25 (12 to 45)	1 (0 to 3)	1593 (1262 to 1996)	974 (722 to 1294)	534 (415 to 685)	81 (38 to 147)	4 (0 to 9)
Kazakhstan	2046	500 (395 to 631)	307 (223 to 409)	167 (130 to 214)	26 (12 to 46)	1 (0 to 3)	1627 (1284 to 2054)	997 (726 to 1330)	543 (422 to 696)	83 (39 to 150)	4 (0 to 9)
Kazakhstan	2047	510 (403 to 649)	313 (228 to 420)	169 (131 to 217)	26 (12 to 47)	1 (0 to 3)	1659 (1310 to 2111)	1019 (743 to 1368)	551 (427 to 707)	85 (40 to 153)	3 (0 to 9)
Kazakhstan	2048	519 (409 to 655)	320 (234 to 428)	172 (133 to 220)	27 (13 to 48)	1 (0 to 3)	1689 (1330 to 2132)	1040 (762 to 1393)	559 (433 to 717)	87 (41 to 156)	3 (0 to 9)
Kazakhstan	2049	529 (418 to 667)	326 (236 to 435)	174 (135 to 223)	27 (13 to 49)	1 (0 to 3)	1721 (1359 to 2169)	1061 (766 to 1417)	567 (439 to 727)	89 (42 to 160)	3 (0 to 9)
Kazakhstan	2050	539 (418 to 678)	333 (238 to 446)	177 (137 to 227)	28 (13 to 50)	1 (0 to 3)	1753 (1362 to 2205)	1084 (775 to 1452)	576 (445 to 738)	91 (43 to 163)	3 (0 to 10)
Kenya	1995	64 (53 to 78)	23 (18 to 30)	30 (21 to 41)	8 (4 to 14)	3 (3 to 3)	123 (102 to 150)	44 (34 to 57)	57 (40 to 80)	15 (7 to 28)	7 (7 to 7)
Kenya	1996	63 (52 to 76)	22 (17 to 28)	29 (21 to 40)	8 (4 to 14)	5 (5 to 5)	121 (100 to 147)	42 (32 to 54)	56 (40 to 76)	14 (7 to 26)	9 (9 to 9)
Kenya	1997	61 (50 to 75)	22 (16 to 28)	29 (21 to 40)	8 (4 to 14)	3 (3 to 3)	118 (96 to 144)	42 (32 to 53)	56 (41 to 77)	15 (7 to 26)	5 (5 to 5)
Kenya	1998	62 (52 to 76)	21 (16 to 27)	29 (22 to 40)	8 (4 to 14)	4 (4 to 4)	120 (100 to 146)	41 (32 to 53)	56 (41 to 77)	15 (7 to 27)	8 (8 to 8)
Kenya	1999	60 (49 to 72)	20 (15 to 26)	29 (21 to 40)	7 (4 to 14)	3 (3 to 3)	115 (95 to 139)	39 (30 to 49)	56 (40 to 76)	14 (7 to 26)	6 (6 to 6)
Kenya	2000	59 (49 to 71)	19 (14 to 24)	29 (21 to 39)	7 (4 to 13)	4 (4 to 4)	113 (94 to 137)	36 (28 to 46)	55 (40 to 74)	14 (7 to 25)	8 (8 to 8)

Kenya	2001	60 (50 to 73)	19 (15 to 24)	29 (21 to 39)	7 (4 to 13)	5 (5 to 5)	116 (97 to 140)	37 (29 to 47)	56 (40 to 75)	14 (7 to 25)	9 (9 to 9)
Kenya	2002	61 (50 to 73)	20 (15 to 25)	29 (21 to 40)	7 (4 to 13)	4 (4 to 4)	117 (97 to 141)	38 (29 to 49)	57 (41 to 77)	14 (7 to 25)	8 (8 to 8)
Kenya	2003	62 (52 to 74)	19 (15 to 25)	30 (21 to 41)	7 (4 to 13)	6 (6 to 6)	120 (100 to 143)	37 (28 to 48)	57 (41 to 78)	14 (7 to 25)	12 (12 to 12)
Kenya	2004	63 (53 to 75)	19 (14 to 24)	29 (21 to 40)	7 (4 to 12)	8 (8 to 8)	121 (102 to 144)	36 (28 to 47)	57 (41 to 77)	13 (7 to 24)	15 (15 to 15)
Kenya	2005	65 (55 to 78)	20 (15 to 26)	30 (22 to 41)	7 (4 to 13)	8 (8 to 8)	125 (106 to 149)	38 (30 to 49)	58 (42 to 78)	14 (7 to 25)	15 (15 to 15)
Kenya	2006	70 (59 to 83)	21 (16 to 26)	30 (21 to 40)	8 (4 to 14)	11 (11 to 11)	134 (114 to 160)	40 (31 to 51)	58 (41 to 78)	15 (7 to 27)	22 (22 to 22)
Kenya	2007	72 (60 to 85)	22 (17 to 28)	30 (21 to 40)	8 (4 to 15)	11 (11 to 11)	138 (116 to 164)	43 (33 to 54)	57 (41 to 78)	16 (8 to 29)	21 (21 to 21)
Kenya	2008	75 (64 to 89)	23 (18 to 30)	29 (21 to 39)	9 (5 to 16)	15 (15 to 15)	145 (124 to 171)	44 (34 to 57)	55 (40 to 75)	17 (9 to 30)	28 (28 to 28)
Kenya	2009	78 (66 to 91)	24 (18 to 31)	28 (20 to 38)	10 (5 to 17)	16 (16 to 16)	149 (128 to 176)	46 (35 to 59)	54 (39 to 74)	18 (9 to 32)	31 (31 to 31)
Kenya	2010	82 (70 to 97)	26 (20 to 34)	28 (21 to 39)	10 (5 to 18)	17 (17 to 17)	158 (135 to 186)	51 (38 to 66)	54 (40 to 75)	20 (10 to 34)	33 (33 to 33)
Kenya	2011	83 (71 to 98)	27 (21 to 35)	28 (20 to 38)	11 (5 to 18)	18 (18 to 18)	160 (137 to 188)	52 (40 to 68)	53 (39 to 73)	20 (10 to 34)	34 (34 to 34)
Kenya	2012	87 (74 to 101)	29 (22 to 37)	28 (20 to 38)	11 (5 to 19)	20 (20 to 20)	167 (143 to 194)	55 (43 to 71)	53 (39 to 73)	21 (11 to 36)	38 (38 to 38)
Kenya	2013	92 (80 to 107)	31 (24 to 39)	28 (20 to 38)	11 (6 to 20)	23 (23 to 23)	178 (153 to 206)	60 (46 to 76)	53 (39 to 72)	22 (11 to 38)	43 (43 to 43)
Kenya	2014	94 (80 to 109)	34 (26 to 44)	27 (20 to 37)	13 (6 to 22)	20 (20 to 20)	180 (155 to 210)	65 (51 to 84)	52 (38 to 70)	24 (12 to 42)	38 (38 to 38)
Kenya	2015	92 (79 to 108)	36 (28 to 46)	26 (19 to 35)	14 (7 to 24)	17 (17 to 17)	178 (151 to 208)	69 (53 to 88)	50 (36 to 68)	26 (13 to 45)	33 (33 to 33)

Kenya	2016	95 (80 to 112)	38 (29 to 49)	25 (18 to 34)	15 (7 to 26)	18 (18 to 18)	183 (154 to 216)	73 (57 to 94)	48 (35 to 66)	28 (14 to 49)	34 (34 to 34)
Kenya	2017	96 (78 to 120)	38 (29 to 49)	24 (17 to 33)	14 (7 to 25)	20 (20 to 20)	185 (150 to 230)	73 (56 to 94)	46 (32 to 64)	28 (14 to 48)	38 (38 to 38)
Kenya	2018	96 (78 to 120)	39 (30 to 50)	24 (17 to 34)	15 (7 to 26)	18 (18 to 18)	185 (150 to 231)	75 (57 to 96)	47 (33 to 65)	29 (14 to 50)	35 (35 to 35)
Kenya	2019	98 (79 to 122)	40 (30 to 51)	25 (18 to 35)	15 (8 to 27)	18 (18 to 18)	189 (152 to 235)	76 (58 to 98)	48 (34 to 67)	29 (15 to 51)	35 (34 to 35)
Kenya	2020	101 (82 to 125)	41 (31 to 53)	26 (18 to 36)	16 (8 to 27)	18 (17 to 19)	194 (157 to 241)	79 (60 to 102)	50 (35 to 69)	30 (15 to 53)	35 (33 to 37)
Kenya	2021	103 (84 to 130)	42 (33 to 54)	26 (19 to 37)	16 (8 to 28)	18 (17 to 20)	199 (161 to 250)	82 (63 to 105)	51 (36 to 70)	31 (16 to 55)	35 (32 to 38)
Kenya	2022	106 (85 to 132)	44 (33 to 56)	27 (19 to 38)	17 (8 to 29)	18 (16 to 20)	203 (163 to 254)	84 (64 to 108)	52 (37 to 72)	32 (16 to 56)	35 (31 to 39)
Kenya	2023	108 (86 to 136)	45 (34 to 58)	28 (20 to 38)	17 (9 to 30)	18 (16 to 20)	208 (166 to 261)	87 (66 to 112)	53 (38 to 74)	33 (17 to 58)	35 (31 to 39)
Kenya	2024	111 (88 to 139)	46 (35 to 60)	28 (20 to 39)	18 (9 to 31)	18 (16 to 20)	213 (169 to 267)	89 (67 to 115)	55 (39 to 76)	34 (17 to 60)	34 (30 to 39)
Kenya	2025	113 (90 to 143)	48 (36 to 61)	29 (21 to 40)	18 (9 to 32)	18 (16 to 20)	217 (173 to 275)	92 (69 to 118)	56 (39 to 77)	35 (18 to 62)	34 (30 to 39)
Kenya	2026	116 (92 to 147)	49 (37 to 64)	30 (21 to 41)	19 (9 to 33)	18 (15 to 20)	223 (177 to 282)	95 (72 to 122)	57 (40 to 79)	36 (18 to 63)	34 (29 to 39)
Kenya	2027	118 (93 to 150)	51 (38 to 65)	30 (21 to 42)	19 (10 to 34)	18 (15 to 20)	228 (180 to 288)	98 (74 to 125)	59 (41 to 81)	37 (19 to 65)	34 (29 to 39)
Kenya	2028	121 (95 to 155)	52 (40 to 68)	31 (22 to 43)	20 (10 to 35)	18 (15 to 21)	233 (183 to 297)	101 (77 to 130)	60 (42 to 83)	38 (19 to 66)	34 (29 to 40)
Kenya	2029	124 (98 to 158)	54 (41 to 70)	32 (22 to 44)	20 (10 to 35)	17 (15 to 21)	238 (188 to 304)	104 (78 to 134)	61 (43 to 85)	39 (20 to 68)	34 (29 to 40)
Kenya	2030	126 (100 to 161)	55 (42 to 72)	32 (23 to 45)	21 (11 to 36)	17 (15 to 21)	243 (192 to 310)	107 (80 to 138)	62 (44 to 87)	40 (20 to 70)	34 (28 to 41)

Kenya	2031	129 (102 to 165)	57 (43 to 73)	33 (23 to 46)	22 (11 to 38)	18 (15 to 21)	249 (197 to 318)	110 (83 to 141)	64 (45 to 89)	41 (21 to 72)	34 (28 to 41)
Kenya	2032	132 (105 to 168)	59 (44 to 76)	34 (24 to 47)	22 (11 to 39)	18 (15 to 22)	254 (202 to 322)	113 (85 to 146)	65 (46 to 90)	42 (21 to 74)	34 (28 to 41)
Kenya	2033	135 (107 to 172)	60 (46 to 78)	34 (24 to 48)	23 (11 to 40)	18 (15 to 22)	260 (206 to 332)	116 (88 to 150)	66 (47 to 92)	43 (22 to 76)	34 (28 to 42)
Kenya	2034	138 (109 to 178)	62 (47 to 81)	35 (25 to 49)	23 (12 to 41)	18 (14 to 22)	265 (209 to 342)	119 (90 to 156)	67 (48 to 93)	45 (22 to 79)	34 (28 to 43)
Kenya	2035	141 (111 to 182)	64 (48 to 83)	36 (25 to 50)	24 (12 to 42)	18 (14 to 22)	271 (213 to 350)	123 (93 to 160)	69 (48 to 95)	46 (23 to 81)	34 (28 to 43)
Kenya	2036	144 (113 to 186)	66 (49 to 86)	36 (26 to 50)	24 (12 to 43)	18 (14 to 22)	277 (217 to 357)	126 (95 to 165)	70 (49 to 97)	47 (24 to 83)	34 (27 to 43)
Kenya	2037	147 (115 to 189)	67 (51 to 88)	37 (26 to 51)	25 (13 to 44)	18 (14 to 22)	283 (221 to 364)	129 (98 to 169)	71 (50 to 99)	48 (24 to 85)	35 (28 to 43)
Kenya	2038	151 (118 to 194)	69 (52 to 90)	38 (27 to 52)	26 (13 to 45)	18 (14 to 23)	290 (227 to 374)	133 (100 to 174)	73 (51 to 101)	49 (25 to 87)	35 (27 to 45)
Kenya	2039	154 (119 to 198)	71 (53 to 93)	38 (27 to 53)	26 (13 to 47)	18 (14 to 23)	296 (229 to 381)	136 (102 to 179)	74 (52 to 102)	50 (25 to 89)	35 (28 to 45)
Kenya	2040	157 (121 to 203)	73 (54 to 96)	39 (27 to 54)	27 (14 to 48)	18 (14 to 24)	302 (233 to 390)	140 (105 to 184)	75 (53 to 104)	52 (26 to 92)	35 (27 to 46)
Kenya	2041	161 (124 to 208)	75 (56 to 98)	40 (28 to 55)	27 (14 to 49)	19 (14 to 24)	309 (238 to 400)	144 (107 to 189)	76 (54 to 106)	53 (27 to 94)	36 (27 to 46)
Kenya	2042	164 (126 to 213)	77 (58 to 101)	40 (28 to 56)	28 (14 to 50)	19 (14 to 24)	316 (242 to 410)	148 (111 to 194)	78 (54 to 108)	54 (27 to 96)	36 (27 to 47)
Kenya	2043	168 (129 to 217)	79 (59 to 104)	41 (29 to 57)	29 (15 to 51)	19 (14 to 25)	323 (249 to 417)	152 (113 to 201)	79 (55 to 109)	55 (28 to 99)	36 (27 to 48)
Kenya	2044	171 (132 to 224)	81 (60 to 108)	42 (29 to 58)	29 (15 to 53)	19 (14 to 26)	330 (254 to 431)	156 (116 to 207)	80 (56 to 111)	57 (29 to 101)	36 (27 to 49)
Kenya	2045	175 (135 to 231)	83 (62 to 112)	42 (30 to 59)	30 (15 to 54)	19 (14 to 26)	337 (259 to 444)	160 (119 to 215)	82 (57 to 113)	58 (29 to 104)	37 (27 to 49)

Kenya	2046	179 (138 to 235)	86 (63 to 115)	43 (30 to 60)	31 (16 to 55)	19 (14 to 26)	345 (265 to 452)	165 (121 to 220)	83 (58 to 115)	59 (30 to 107)	37 (26 to 50)
Kenya	2047	183 (140 to 241)	88 (64 to 117)	44 (31 to 61)	32 (16 to 57)	20 (14 to 27)	352 (270 to 464)	169 (124 to 226)	84 (59 to 117)	61 (31 to 109)	38 (27 to 52)
Kenya	2048	187 (143 to 246)	91 (66 to 122)	45 (31 to 62)	32 (16 to 58)	20 (14 to 27)	360 (275 to 473)	174 (126 to 234)	86 (60 to 120)	62 (32 to 112)	38 (27 to 52)
Kenya	2049	191 (146 to 253)	93 (67 to 126)	45 (32 to 63)	33 (17 to 60)	20 (14 to 28)	368 (281 to 487)	179 (129 to 242)	87 (61 to 122)	64 (32 to 115)	38 (27 to 53)
Kenya	2050	196 (148 to 260)	95 (68 to 129)	46 (32 to 64)	34 (17 to 61)	20 (14 to 28)	376 (285 to 500)	183 (131 to 249)	89 (62 to 124)	65 (33 to 118)	39 (27 to 54)
Kiribati	1995	186 (158 to 218)	137 (115 to 163)	45 (31 to 64)	0 (0 to 1)	3 (3 to 3)	252 (215 to 295)	186 (155 to 221)	61 (42 to 86)	1 (0 to 1)	4 (4 to 4)
Kiribati	1996	178 (152 to 207)	133 (113 to 155)	44 (30 to 62)	0 (0 to 1)	0 (0 to 0)	241 (206 to 280)	180 (153 to 210)	60 (41 to 84)	1 (0 to 1)	0 (0 to 0)
Kiribati	1997	178 (154 to 204)	132 (113 to 151)	44 (30 to 62)	0 (0 to 1)	1 (1 to 1)	241 (208 to 276)	179 (154 to 205)	60 (41 to 84)	0 (0 to 1)	2 (2 to 2)
Kiribati	1998	177 (154 to 204)	132 (115 to 152)	44 (31 to 62)	0 (0 to 0)	1 (1 to 1)	240 (209 to 277)	179 (156 to 205)	60 (41 to 84)	0 (0 to 1)	1 (1 to 1)
Kiribati	1999	180 (157 to 206)	134 (118 to 152)	44 (30 to 62)	0 (0 to 0)	2 (2 to 2)	244 (213 to 279)	181 (160 to 206)	60 (41 to 84)	0 (0 to 0)	3 (3 to 3)
Kiribati	2000	172 (152 to 197)	127 (112 to 144)	43 (29 to 60)	0 (0 to 0)	2 (2 to 2)	233 (205 to 267)	172 (151 to 195)	58 (40 to 82)	0 (0 to 0)	3 (3 to 3)
Kiribati	2001	212 (187 to 240)	160 (141 to 183)	48 (32 to 69)	0 (0 to 0)	4 (4 to 4)	287 (253 to 325)	217 (191 to 248)	65 (44 to 93)	0 (0 to 0)	5 (5 to 5)
Kiribati	2002	213 (188 to 239)	161 (141 to 183)	48 (33 to 69)	0 (0 to 0)	4 (4 to 4)	288 (254 to 324)	218 (191 to 247)	65 (45 to 93)	0 (0 to 0)	5 (5 to 5)
Kiribati	2003	205 (181 to 230)	151 (132 to 172)	47 (32 to 65)	0 (0 to 0)	7 (7 to 7)	277 (245 to 312)	205 (179 to 232)	63 (43 to 89)	0 (0 to 0)	9 (9 to 9)
Kiribati	2004	294 (268 to 322)	165 (144 to 188)	49 (34 to 68)	0 (0 to 0)	80 (80 to 80)	399 (364 to 436)	224 (195 to 255)	66 (46 to 93)	0 (0 to 0)	108 (108 to 108)

Kiribati	2005	244 (219 to 271)	160 (139 to 181)	48 (33 to 66)	0 (0 to 0)	36 (36 to 36)	331 (296 to 367)	216 (188 to 245)	65 (45 to 89)	0 (0 to 0)	49 (49 to 49)
Kiribati	2006	217 (195 to 240)	135 (118 to 153)	44 (31 to 61)	0 (0 to 0)	37 (37 to 37)	293 (264 to 325)	183 (161 to 208)	60 (42 to 82)	0 (0 to 0)	50 (50 to 50)
Kiribati	2007	235 (213 to 259)	132 (116 to 151)	44 (31 to 60)	0 (0 to 0)	59 (59 to 59)	319 (289 to 350)	179 (157 to 204)	59 (42 to 81)	0 (0 to 0)	80 (80 to 80)
Kiribati	2008	278 (256 to 302)	129 (112 to 148)	43 (30 to 59)	0 (0 to 0)	105 (105 to 105)	376 (347 to 409)	175 (152 to 200)	59 (41 to 81)	0 (0 to 0)	142 (142 to 142)
Kiribati	2009	229 (208 to 253)	119 (104 to 137)	42 (29 to 58)	1 (0 to 2)	67 (67 to 67)	311 (282 to 343)	161 (141 to 185)	57 (39 to 78)	1 (1 to 2)	91 (91 to 91)
Kiribati	2010	228 (208 to 250)	109 (95 to 125)	41 (28 to 56)	2 (1 to 4)	76 (76 to 76)	309 (282 to 339)	147 (129 to 169)	55 (38 to 76)	3 (2 to 6)	103 (103 to 103)
Kiribati	2011	217 (197 to 238)	107 (92 to 123)	41 (28 to 57)	4 (2 to 7)	65 (65 to 65)	294 (266 to 323)	145 (125 to 166)	56 (39 to 77)	6 (3 to 10)	88 (88 to 88)
Kiribati	2012	223 (202 to 245)	108 (93 to 123)	42 (29 to 58)	5 (3 to 10)	68 (68 to 68)	302 (274 to 332)	146 (126 to 167)	57 (40 to 79)	7 (4 to 13)	93 (93 to 93)
Kiribati	2013	200 (180 to 222)	103 (89 to 118)	41 (29 to 57)	6 (3 to 11)	50 (50 to 50)	271 (244 to 301)	139 (120 to 160)	56 (39 to 77)	8 (4 to 14)	68 (68 to 68)
Kiribati	2014	187 (165 to 213)	113 (97 to 131)	44 (31 to 61)	7 (3 to 12)	23 (23 to 23)	254 (224 to 288)	153 (132 to 177)	60 (41 to 83)	9 (4 to 17)	32 (32 to 32)
Kiribati	2015	186 (164 to 211)	108 (93 to 125)	43 (30 to 60)	7 (3 to 12)	28 (28 to 28)	251 (223 to 286)	146 (126 to 170)	59 (41 to 82)	9 (4 to 16)	37 (37 to 37)
Kiribati	2016	207 (184 to 235)	118 (101 to 139)	46 (32 to 64)	7 (3 to 13)	36 (36 to 36)	280 (249 to 318)	160 (137 to 188)	62 (43 to 87)	10 (5 to 18)	48 (48 to 48)
Kiribati	2017	214 (188 to 246)	120 (101 to 143)	46 (32 to 64)	7 (3 to 13)	41 (41 to 41)	290 (255 to 334)	163 (137 to 194)	62 (43 to 87)	9 (4 to 17)	56 (56 to 56)
Kiribati	2018	227 (197 to 261)	135 (113 to 161)	46 (32 to 65)	7 (3 to 13)	40 (40 to 40)	308 (267 to 354)	182 (153 to 218)	62 (43 to 87)	10 (5 to 18)	54 (54 to 54)
Kiribati	2019	246 (214 to 284)	152 (126 to 182)	46 (32 to 65)	7 (3 to 13)	41 (41 to 41)	333 (289 to 385)	206 (171 to 246)	63 (43 to 88)	10 (5 to 18)	55 (55 to 55)

Kiribati	2020	245 (211 to 286)	150 (124 to 182)	47 (32 to 66)	7 (3 to 13)	41 (39 to 43)	331 (286 to 387)	203 (168 to 247)	63 (44 to 89)	10 (5 to 18)	55 (53 to 59)
Kiribati	2021	242 (209 to 281)	147 (120 to 178)	46 (32 to 66)	7 (3 to 13)	41 (39 to 45)	328 (283 to 380)	199 (163 to 241)	63 (44 to 89)	10 (5 to 18)	56 (53 to 61)
Kiribati	2022	239 (204 to 280)	143 (116 to 177)	46 (32 to 65)	7 (3 to 13)	42 (38 to 46)	324 (277 to 380)	194 (158 to 240)	63 (44 to 89)	10 (5 to 18)	57 (52 to 63)
Kiribati	2023	236 (201 to 278)	141 (113 to 173)	46 (32 to 65)	7 (3 to 14)	42 (38 to 47)	320 (273 to 377)	190 (153 to 235)	63 (44 to 88)	10 (5 to 18)	57 (52 to 64)
Kiribati	2024	234 (199 to 276)	138 (110 to 170)	46 (32 to 65)	7 (3 to 14)	42 (38 to 48)	316 (270 to 373)	187 (149 to 230)	63 (43 to 88)	10 (5 to 19)	57 (51 to 65)
Kiribati	2025	231 (195 to 273)	135 (107 to 167)	46 (32 to 65)	7 (4 to 14)	42 (38 to 48)	313 (265 to 371)	183 (145 to 226)	63 (43 to 88)	10 (5 to 19)	57 (51 to 65)
Kiribati	2026	230 (197 to 274)	133 (106 to 167)	46 (32 to 65)	7 (4 to 14)	43 (38 to 49)	311 (267 to 371)	181 (144 to 226)	63 (44 to 88)	10 (5 to 19)	58 (51 to 66)
Kiribati	2027	229 (192 to 271)	132 (103 to 166)	46 (32 to 65)	8 (4 to 14)	43 (38 to 50)	310 (260 to 368)	179 (140 to 225)	63 (44 to 89)	10 (5 to 19)	58 (51 to 67)
Kiribati	2028	230 (194 to 274)	132 (104 to 168)	47 (32 to 66)	8 (4 to 14)	43 (38 to 50)	311 (263 to 371)	179 (141 to 228)	63 (44 to 89)	10 (5 to 19)	58 (51 to 68)
Kiribati	2029	231 (194 to 276)	134 (105 to 169)	47 (32 to 66)	8 (4 to 14)	43 (38 to 51)	313 (263 to 375)	181 (142 to 229)	63 (44 to 89)	10 (5 to 19)	59 (51 to 69)
Kiribati	2030	234 (195 to 282)	136 (105 to 173)	47 (32 to 66)	8 (4 to 14)	44 (37 to 52)	317 (264 to 382)	184 (143 to 234)	64 (44 to 90)	11 (5 to 19)	59 (51 to 71)
Kiribati	2031	237 (197 to 285)	138 (107 to 177)	47 (33 to 67)	8 (4 to 14)	44 (38 to 52)	321 (267 to 386)	187 (145 to 240)	64 (44 to 90)	11 (5 to 20)	59 (51 to 71)
Kiribati	2032	241 (202 to 288)	141 (110 to 180)	47 (33 to 67)	8 (4 to 15)	44 (37 to 53)	326 (273 to 391)	191 (148 to 244)	64 (44 to 91)	11 (5 to 20)	60 (51 to 72)
Kiribati	2033	245 (201 to 296)	144 (112 to 186)	48 (33 to 67)	8 (4 to 15)	44 (38 to 54)	332 (273 to 402)	196 (152 to 252)	65 (45 to 91)	11 (5 to 20)	60 (51 to 73)
Kiribati	2034	249 (208 to 300)	148 (114 to 191)	48 (33 to 68)	8 (4 to 15)	45 (37 to 54)	337 (281 to 406)	200 (154 to 259)	65 (45 to 92)	11 (5 to 20)	61 (51 to 74)

Kiribati	2035	252 (205 to 309)	151 (115 to 196)	49 (34 to 69)	8 (4 to 15)	45 (37 to 55)	342 (278 to 419)	204 (155 to 265)	66 (45 to 93)	11 (5 to 21)	61 (51 to 74)
Kiribati	2036	256 (211 to 312)	154 (116 to 201)	49 (34 to 69)	8 (4 to 15)	45 (37 to 57)	347 (285 to 423)	208 (157 to 273)	66 (46 to 94)	11 (5 to 21)	61 (51 to 77)
Kiribati	2037	261 (213 to 325)	157 (118 to 208)	49 (34 to 70)	9 (4 to 16)	45 (38 to 56)	353 (288 to 440)	213 (159 to 282)	67 (46 to 95)	12 (5 to 21)	62 (51 to 75)
Kiribati	2038	265 (215 to 324)	161 (121 to 214)	50 (34 to 70)	9 (4 to 16)	46 (38 to 57)	359 (292 to 440)	218 (163 to 290)	67 (46 to 95)	12 (6 to 22)	62 (51 to 78)
Kiribati	2039	269 (217 to 332)	165 (122 to 219)	50 (35 to 71)	9 (4 to 16)	46 (38 to 58)	365 (294 to 450)	223 (166 to 296)	68 (47 to 96)	12 (6 to 22)	62 (51 to 79)
Kiribati	2040	274 (224 to 339)	169 (126 to 224)	50 (35 to 72)	9 (4 to 16)	46 (38 to 58)	371 (304 to 459)	228 (171 to 304)	68 (47 to 97)	12 (6 to 22)	63 (51 to 79)
Kiribati	2041	279 (224 to 345)	173 (127 to 230)	51 (35 to 72)	9 (4 to 17)	47 (38 to 58)	378 (304 to 467)	234 (172 to 312)	69 (48 to 98)	12 (6 to 23)	63 (52 to 79)
Kiribati	2042	284 (228 to 354)	177 (130 to 237)	51 (35 to 73)	9 (4 to 17)	47 (38 to 59)	385 (309 to 480)	240 (176 to 322)	69 (48 to 98)	12 (6 to 23)	63 (52 to 80)
Kiribati	2043	289 (231 to 359)	181 (132 to 244)	52 (36 to 73)	9 (4 to 17)	47 (38 to 61)	392 (313 to 487)	245 (179 to 330)	70 (48 to 99)	13 (6 to 23)	64 (52 to 83)
Kiribati	2044	294 (237 to 367)	185 (136 to 249)	52 (36 to 74)	9 (4 to 17)	47 (38 to 63)	399 (321 to 498)	251 (184 to 338)	70 (49 to 100)	13 (6 to 24)	64 (52 to 85)
Kiribati	2045	299 (236 to 377)	189 (138 to 257)	52 (36 to 74)	10 (5 to 18)	48 (38 to 61)	405 (320 to 511)	257 (187 to 348)	71 (49 to 101)	13 (6 to 24)	65 (52 to 83)
Kiribati	2046	304 (243 to 382)	194 (141 to 264)	53 (36 to 75)	10 (5 to 18)	48 (38 to 61)	412 (330 to 518)	262 (191 to 357)	71 (49 to 101)	13 (6 to 24)	65 (52 to 83)
Kiribati	2047	309 (246 to 391)	198 (142 to 269)	53 (36 to 75)	10 (5 to 18)	48 (39 to 64)	419 (333 to 530)	269 (193 to 364)	72 (49 to 102)	13 (6 to 25)	66 (52 to 86)
Kiribati	2048	314 (249 to 401)	202 (146 to 278)	53 (37 to 76)	10 (5 to 18)	49 (39 to 64)	426 (337 to 543)	274 (198 to 377)	72 (50 to 102)	14 (6 to 25)	66 (53 to 86)
Kiribati	2049	319 (253 to 407)	207 (149 to 284)	53 (37 to 76)	10 (5 to 19)	49 (39 to 65)	432 (343 to 551)	280 (202 to 385)	72 (50 to 103)	14 (6 to 25)	66 (53 to 88)

Kiribati	2050	324 (252 to 412)	211 (152 to 292)	54 (37 to 76)	10 (5 to 19)	49 (39 to 67)	440 (342 to 558)	286 (206 to 396)	73 (50 to 104)	14 (6 to 26)	67 (53 to 90)
Kuwait	1995	1560 (1402 to 1742)	1268 (1114 to 1447)	274 (240 to 312)	19 (10 to 33)	0 (0 to 0)	3651 (3282 to 4077)	2967 (2607 to 3387)	640 (563 to 730)	44 (24 to 77)	0 (0 to 0)
Kuwait	1996	1475 (1342 to 1623)	1177 (1051 to 1318)	279 (251 to 309)	19 (10 to 32)	0 (0 to 0)	3452 (3142 to 3799)	2755 (2461 to 3084)	653 (587 to 724)	44 (24 to 74)	0 (0 to 0)
Kuwait	1997	1471 (1347 to 1604)	1166 (1046 to 1294)	286 (258 to 314)	19 (11 to 32)	0 (0 to 0)	3442 (3153 to 3754)	2728 (2447 to 3027)	669 (604 to 734)	45 (26 to 74)	0 (0 to 0)
Kuwait	1998	1594 (1468 to 1733)	1268 (1147 to 1398)	305 (278 to 332)	22 (13 to 36)	0 (0 to 0)	3730 (3436 to 4056)	2966 (2685 to 3271)	713 (650 to 778)	51 (30 to 84)	0 (0 to 0)
Kuwait	1999	1375 (1266 to 1493)	1087 (984 to 1201)	268 (245 to 293)	20 (12 to 32)	0 (0 to 0)	3218 (2962 to 3495)	2544 (2302 to 2811)	628 (574 to 686)	46 (27 to 75)	0 (0 to 0)
Kuwait	2000	1211 (1118 to 1312)	954 (859 to 1051)	240 (219 to 260)	18 (11 to 28)	0 (0 to 0)	2835 (2616 to 3071)	2232 (2009 to 2460)	561 (512 to 610)	42 (26 to 66)	0 (0 to 0)
Kuwait	2001	1313 (1204 to 1420)	1049 (941 to 1156)	244 (223 to 265)	20 (12 to 31)	0 (0 to 0)	3072 (2818 to 3324)	2454 (2203 to 2704)	571 (522 to 620)	46 (29 to 72)	0 (0 to 0)
Kuwait	2002	1308 (1199 to 1417)	1046 (939 to 1150)	242 (222 to 263)	21 (13 to 32)	0 (0 to 0)	3062 (2806 to 3317)	2447 (2197 to 2691)	566 (520 to 616)	49 (31 to 76)	0 (0 to 0)
Kuwait	2003	1289 (1179 to 1399)	1031 (926 to 1137)	235 (215 to 255)	23 (14 to 34)	0 (0 to 0)	3016 (2759 to 3274)	2413 (2168 to 2661)	550 (503 to 597)	53 (34 to 80)	0 (0 to 0)

Kuwait	2004	1235 (1128 to 1350)	990 (883 to 1101)	222 (204 to 242)	23 (14 to 35)	0 (0 to 0)	2891 (2639 to 3159)	2317 (2067 to 2578)	521 (477 to 566)	53 (34 to 81)	0 (0 to 0)
Kuwait	2005	1109 (1015 to 1218)	888 (796 to 996)	200 (184 to 219)	21 (13 to 31)	0 (0 to 0)	2595 (2377 to 2850)	2077 (1862 to 2330)	469 (430 to 514)	49 (30 to 73)	0 (0 to 0)
Kuwait	2006	1193 (1090 to 1310)	970 (866 to 1087)	200 (183 to 221)	23 (14 to 34)	0 (0 to 0)	2791 (2551 to 3065)	2269 (2027 to 2544)	469 (429 to 516)	53 (33 to 80)	0 (0 to 0)
Kuwait	2007	1092 (997 to 1204)	888 (791 to 999)	184 (167 to 202)	21 (13 to 31)	0 (0 to 0)	2556 (2333 to 2817)	2077 (1852 to 2338)	430 (391 to 472)	48 (30 to 72)	0 (0 to 0)
Kuwait	2008	1277 (1163 to 1404)	1065 (952 to 1198)	189 (172 to 208)	23 (15 to 35)	0 (0 to 0)	2988 (2722 to 3286)	2492 (2228 to 2804)	442 (402 to 486)	55 (34 to 82)	0 (0 to 0)
Kuwait	2009	1213 (1106 to 1334)	1024 (917 to 1146)	169 (153 to 187)	20 (12 to 31)	0 (0 to 0)	2840 (2588 to 3122)	2397 (2147 to 2682)	396 (358 to 437)	46 (28 to 73)	0 (0 to 0)
Kuwait	2010	1161 (1058 to 1278)	989 (888 to 1109)	153 (137 to 170)	18 (11 to 28)	0 (0 to 0)	2716 (2476 to 2991)	2315 (2078 to 2596)	358 (320 to 399)	43 (26 to 67)	0 (0 to 0)
Kuwait	2011	1111 (1014 to 1221)	951 (855 to 1060)	143 (128 to 160)	17 (10 to 26)	0 (0 to 0)	2600 (2373 to 2857)	2227 (2001 to 2481)	334 (300 to 374)	39 (24 to 61)	0 (0 to 0)
Kuwait	2012	1133 (1035 to 1244)	972 (875 to 1079)	145 (131 to 163)	16 (10 to 25)	0 (0 to 0)	2652 (2422 to 2911)	2274 (2047 to 2526)	340 (307 to 381)	38 (23 to 59)	0 (0 to 0)
Kuwait	2013	1146 (1051 to 1254)	980 (885 to 1081)	152 (137 to 169)	14 (9 to 23)	0 (0 to 0)	2682 (2460 to 2934)	2294 (2071 to 2530)	355 (322 to 396)	33 (20 to 54)	0 (0 to 0)

Kuwait	2014	1255 (1148 to 1378)	1077 (975 to 1194)	167 (151 to 185)	11 (6 to 18)	0 (0 to 0)	2938 (2688 to 3225)	2520 (2281 to 2795)	391 (354 to 434)	26 (15 to 43)	0 (0 to 0)
Kuwait	2015	1417 (1302 to 1553)	1217 (1098 to 1348)	194 (175 to 215)	6 (3 to 10)	0 (0 to 0)	3315 (3047 to 3634)	2847 (2569 to 3155)	454 (409 to 502)	14 (8 to 23)	0 (0 to 0)
Kuwait	2016	1490 (1358 to 1638)	1278 (1147 to 1421)	212 (190 to 234)	1 (0 to 1)	0 (0 to 0)	3488 (3178 to 3833)	2990 (2684 to 3326)	497 (445 to 549)	1 (1 to 2)	0 (0 to 0)
Kuwait	2017	1556 (1400 to 1725)	1336 (1186 to 1499)	220 (194 to 249)	0 (0 to 0)	0 (0 to 0)	3640 (3277 to 4036)	3127 (2775 to 3507)	514 (453 to 582)	0 (0 to 0)	0 (0 to 0)
Kuwait	2018	1563 (1388 to 1754)	1341 (1173 to 1525)	222 (196 to 252)	0 (0 to 0)	0 (0 to 0)	3658 (3248 to 4105)	3138 (2745 to 3568)	520 (459 to 589)	0 (0 to 0)	0 (0 to 0)
Kuwait	2019	1676 (1474 to 1891)	1455 (1257 to 1663)	220 (195 to 250)	0 (0 to 0)	0 (0 to 0)	3922 (3449 to 4424)	3406 (2941 to 3893)	516 (456 to 584)	0 (0 to 0)	0 (0 to 0)
Kuwait	2020	1710 (1507 to 1930)	1488 (1293 to 1714)	221 (195 to 251)	0 (0 to 0)	0 (0 to 0)	4001 (3528 to 4516)	3483 (3026 to 4011)	518 (457 to 587)	0 (0 to 0)	0 (0 to 0)
Kuwait	2021	1728 (1514 to 1957)	1507 (1300 to 1736)	221 (195 to 250)	0 (0 to 0)	0 (0 to 0)	4045 (3543 to 4581)	3527 (3042 to 4062)	517 (457 to 585)	0 (0 to 0)	0 (0 to 0)
Kuwait	2022	1709 (1493 to 1940)	1487 (1283 to 1713)	221 (196 to 250)	0 (0 to 0)	0 (0 to 0)	3999 (3493 to 4540)	3481 (3002 to 4010)	518 (458 to 586)	0 (0 to 0)	0 (0 to 0)
Kuwait	2023	1695 (1477 to 1929)	1474 (1272 to 1712)	221 (195 to 250)	0 (0 to 0)	0 (0 to 0)	3967 (3457 to 4515)	3450 (2976 to 4006)	517 (457 to 584)	0 (0 to 0)	0 (0 to 0)

Kuwait	2024	1682 (1457 to 1913)	1461 (1242 to 1690)	221 (195 to 249)	0 (0 to 0)	0 (0 to 0)	3935 (3409 to 4476)	3419 (2906 to 3956)	516 (456 to 584)	0 (0 to 0)	0 (0 to 0)
Kuwait	2025	1669 (1441 to 1902)	1448 (1223 to 1683)	221 (195 to 249)	0 (0 to 0)	0 (0 to 0)	3906 (3372 to 4451)	3389 (2862 to 3939)	516 (456 to 582)	0 (0 to 0)	0 (0 to 0)
Kuwait	2026	1661 (1433 to 1898)	1441 (1216 to 1675)	221 (195 to 249)	0 (0 to 0)	0 (0 to 0)	3888 (3354 to 4442)	3372 (2846 to 3919)	517 (456 to 583)	0 (0 to 0)	0 (0 to 0)
Kuwait	2027	1655 (1424 to 1893)	1434 (1207 to 1668)	221 (195 to 250)	0 (0 to 0)	0 (0 to 0)	3872 (3333 to 4431)	3355 (2825 to 3903)	517 (456 to 585)	0 (0 to 0)	0 (0 to 0)
Kuwait	2028	1650 (1412 to 1899)	1429 (1197 to 1668)	221 (194 to 251)	0 (0 to 0)	0 (0 to 0)	3861 (3306 to 4444)	3344 (2802 to 3903)	517 (454 to 588)	0 (0 to 0)	0 (0 to 0)
Kuwait	2029	1641 (1406 to 1889)	1420 (1183 to 1663)	221 (194 to 251)	0 (0 to 0)	0 (0 to 0)	3841 (3291 to 4422)	3324 (2768 to 3892)	517 (453 to 588)	0 (0 to 0)	0 (0 to 0)
Kuwait	2030	1630 (1391 to 1890)	1410 (1176 to 1671)	220 (193 to 251)	0 (0 to 0)	0 (0 to 0)	3815 (3256 to 4422)	3299 (2751 to 3910)	516 (452 to 587)	0 (0 to 0)	0 (0 to 0)
Kuwait	2031	1623 (1386 to 1886)	1403 (1172 to 1666)	220 (192 to 252)	0 (0 to 0)	0 (0 to 0)	3798 (3245 to 4415)	3282 (2743 to 3899)	515 (450 to 589)	0 (0 to 0)	0 (0 to 0)
Kuwait	2032	1616 (1376 to 1885)	1396 (1166 to 1669)	220 (191 to 252)	0 (0 to 0)	0 (0 to 0)	3782 (3221 to 4410)	3267 (2729 to 3906)	514 (448 to 590)	0 (0 to 0)	0 (0 to 0)
Kuwait	2033	1608 (1366 to 1885)	1389 (1151 to 1665)	219 (190 to 252)	0 (0 to 0)	0 (0 to 0)	3763 (3197 to 4412)	3250 (2693 to 3896)	513 (445 to 589)	0 (0 to 0)	0 (0 to 0)

Kuwait	2034	1596 (1356 to 1869)	1378 (1142 to 1653)	218 (189 to 253)	0 (0 to 0)	0 (0 to 0)	3736 (3173 to 4373)	3225 (2672 to 3869)	511 (442 to 593)	0 (0 to 0)	0 (0 to 0)
Kuwait	2035	1584 (1342 to 1867)	1367 (1134 to 1646)	217 (187 to 255)	0 (0 to 0)	0 (0 to 0)	3707 (3141 to 4369)	3199 (2654 to 3852)	508 (439 to 596)	0 (0 to 0)	0 (0 to 0)
Kuwait	2036	1570 (1320 to 1849)	1354 (1112 to 1633)	216 (186 to 255)	0 (0 to 0)	0 (0 to 0)	3674 (3090 to 4326)	3169 (2601 to 3823)	505 (435 to 597)	0 (0 to 0)	0 (0 to 0)
Kuwait	2037	1558 (1303 to 1838)	1344 (1090 to 1629)	214 (184 to 256)	0 (0 to 0)	0 (0 to 0)	3646 (3048 to 4303)	3145 (2551 to 3811)	501 (431 to 599)	0 (0 to 0)	0 (0 to 0)
Kuwait	2038	1541 (1275 to 1827)	1328 (1067 to 1615)	212 (183 to 256)	0 (0 to 0)	0 (0 to 0)	3606 (2984 to 4275)	3109 (2497 to 3779)	497 (428 to 599)	0 (0 to 0)	0 (0 to 0)
Kuwait	2039	1524 (1258 to 1808)	1313 (1051 to 1601)	211 (181 to 256)	0 (0 to 0)	0 (0 to 0)	3566 (2944 to 4231)	3073 (2460 to 3748)	493 (424 to 600)	0 (0 to 0)	0 (0 to 0)
Kuwait	2040	1507 (1247 to 1792)	1298 (1044 to 1583)	209 (179 to 256)	0 (0 to 0)	0 (0 to 0)	3526 (2918 to 4193)	3037 (2444 to 3706)	488 (418 to 600)	0 (0 to 0)	0 (0 to 0)
Kuwait	2041	1488 (1228 to 1776)	1282 (1037 to 1570)	206 (176 to 256)	0 (0 to 0)	0 (0 to 0)	3483 (2873 to 4157)	3000 (2427 to 3674)	483 (412 to 598)	0 (0 to 0)	0 (0 to 0)
Kuwait	2042	1467 (1208 to 1756)	1263 (1011 to 1549)	204 (174 to 255)	0 (0 to 0)	0 (0 to 0)	3434 (2827 to 4109)	2957 (2366 to 3625)	477 (407 to 596)	0 (0 to 0)	0 (0 to 0)
Kuwait	2043	1447 (1193 to 1731)	1245 (997 to 1528)	201 (171 to 253)	0 (0 to 0)	0 (0 to 0)	3386 (2791 to 4051)	2915 (2334 to 3576)	471 (401 to 592)	0 (0 to 0)	0 (0 to 0)

Kuwait	2044	1421 (1169 to 1699)	1223 (980 to 1502)	198 (168 to 251)	0 (0 to 0)	0 (0 to 0)	3326 (2737 to 3975)	2862 (2294 to 3516)	463 (393 to 587)	0 (0 to 0)	0 (0 to 0)
Kuwait	2045	1394 (1144 to 1668)	1199 (961 to 1472)	195 (165 to 249)	0 (0 to 0)	0 (0 to 0)	3262 (2678 to 3905)	2806 (2249 to 3445)	456 (386 to 582)	0 (0 to 0)	0 (0 to 0)
Kuwait	2046	1363 (1115 to 1638)	1172 (936 to 1443)	191 (161 to 246)	0 (0 to 0)	0 (0 to 0)	3191 (2609 to 3834)	2744 (2191 to 3376)	447 (377 to 575)	0 (0 to 0)	0 (0 to 0)
Kuwait	2047	1335 (1094 to 1609)	1148 (920 to 1417)	187 (158 to 243)	0 (0 to 0)	0 (0 to 0)	3125 (2559 to 3766)	2687 (2152 to 3317)	438 (369 to 568)	0 (0 to 0)	0 (0 to 0)
Kuwait	2048	1304 (1067 to 1585)	1121 (896 to 1389)	183 (154 to 239)	0 (0 to 0)	0 (0 to 0)	3052 (2497 to 3709)	2624 (2098 to 3252)	428 (359 to 559)	0 (0 to 0)	0 (0 to 0)
Kuwait	2049	1266 (1038 to 1536)	1087 (865 to 1347)	178 (149 to 235)	0 (0 to 0)	0 (0 to 0)	2962 (2430 to 3594)	2544 (2024 to 3153)	418 (350 to 551)	0 (0 to 0)	0 (0 to 0)
Kuwait	2050	1229 (999 to 1489)	1055 (838 to 1306)	174 (146 to 231)	0 (0 to 0)	0 (0 to 0)	2876 (2339 to 3484)	2469 (1960 to 3056)	407 (341 to 541)	0 (0 to 0)	0 (0 to 0)
Kyrgyzstan	1995	45 (37 to 54)	23 (17 to 29)	19 (13 to 26)	0 (0 to 0)	4 (4 to 4)	140 (114 to 168)	70 (53 to 92)	59 (42 to 79)	0 (0 to 0)	11 (11 to 11)
Kyrgyzstan	1996	42 (34 to 50)	20 (15 to 26)	19 (13 to 25)	0 (0 to 0)	3 (3 to 3)	131 (107 to 156)	63 (48 to 82)	59 (42 to 78)	0 (0 to 0)	9 (9 to 9)
Kyrgyzstan	1997	42 (34 to 50)	20 (15 to 26)	19 (14 to 25)	0 (0 to 0)	3 (3 to 3)	130 (106 to 154)	63 (48 to 81)	59 (43 to 78)	0 (0 to 0)	8 (8 to 8)
Kyrgyzstan	1998	45 (37 to 53)	20 (16 to 26)	20 (15 to 27)	0 (0 to 0)	5 (5 to 5)	140 (115 to 165)	63 (48 to 80)	63 (46 to 83)	0 (0 to 0)	14 (14 to 14)
Kyrgyzstan	1999	41 (34 to 49)	19 (15 to 25)	20 (15 to 26)	0 (0 to 0)	2 (2 to 2)	127 (105 to 152)	61 (47 to 78)	61 (45 to 81)	0 (0 to 0)	5 (5 to 5)

Kyrgyzstan	2000	40 (33 to 47)	18 (14 to 23)	19 (14 to 25)	0 (0 to 0)	3 (3 to 3)	123 (102 to 148)	55 (43 to 71)	58 (42 to 77)	0 (0 to 0)	11 (11 to 11)
Kyrgyzstan	2001	37 (31 to 45)	17 (13 to 22)	19 (14 to 25)	0 (0 to 0)	1 (1 to 1)	116 (95 to 140)	53 (41 to 68)	58 (42 to 78)	0 (0 to 0)	5 (5 to 5)
Kyrgyzstan	2002	41 (34 to 49)	18 (14 to 24)	20 (14 to 27)	0 (0 to 0)	3 (3 to 3)	128 (106 to 154)	57 (44 to 74)	62 (45 to 84)	0 (0 to 0)	9 (9 to 9)
Kyrgyzstan	2003	48 (40 to 57)	20 (15 to 26)	22 (16 to 30)	0 (0 to 0)	6 (6 to 6)	149 (125 to 177)	62 (48 to 80)	69 (50 to 93)	0 (0 to 0)	18 (18 to 18)
Kyrgyzstan	2004	51 (42 to 61)	23 (18 to 30)	24 (18 to 33)	0 (0 to 0)	3 (3 to 3)	157 (131 to 189)	71 (54 to 92)	75 (55 to 102)	0 (0 to 0)	11 (11 to 11)
Kyrgyzstan	2005	59 (50 to 69)	27 (20 to 34)	26 (19 to 35)	0 (0 to 0)	6 (6 to 6)	182 (154 to 216)	83 (63 to 107)	81 (60 to 107)	0 (0 to 0)	19 (19 to 19)
Kyrgyzstan	2006	63 (54 to 75)	29 (23 to 38)	28 (20 to 37)	0 (0 to 0)	6 (6 to 6)	197 (167 to 233)	92 (70 to 118)	86 (64 to 114)	0 (0 to 0)	19 (19 to 19)
Kyrgyzstan	2007	66 (56 to 78)	31 (24 to 39)	29 (21 to 37)	0 (0 to 0)	7 (7 to 7)	205 (173 to 243)	95 (73 to 123)	89 (66 to 116)	0 (0 to 0)	21 (21 to 21)
Kyrgyzstan	2008	69 (58 to 82)	31 (24 to 39)	29 (22 to 38)	0 (0 to 0)	9 (9 to 9)	213 (182 to 254)	95 (74 to 123)	91 (68 to 117)	0 (0 to 0)	27 (27 to 27)
Kyrgyzstan	2009	70 (59 to 84)	34 (26 to 43)	30 (23 to 39)	0 (0 to 0)	7 (7 to 7)	219 (184 to 261)	105 (82 to 135)	94 (71 to 122)	0 (0 to 0)	21 (21 to 21)
Kyrgyzstan	2010	74 (63 to 89)	35 (27 to 45)	32 (24 to 41)	0 (0 to 0)	8 (8 to 8)	231 (196 to 275)	109 (85 to 139)	98 (74 to 128)	0 (0 to 0)	24 (24 to 24)
Kyrgyzstan	2011	81 (69 to 96)	38 (29 to 48)	34 (25 to 44)	0 (0 to 0)	9 (9 to 9)	252 (214 to 297)	117 (91 to 148)	106 (79 to 138)	0 (0 to 0)	28 (28 to 28)
Kyrgyzstan	2012	83 (70 to 99)	38 (30 to 49)	37 (28 to 48)	0 (0 to 0)	7 (7 to 7)	258 (217 to 307)	119 (92 to 151)	116 (86 to 149)	0 (0 to 0)	23 (23 to 23)
Kyrgyzstan	2013	84 (71 to 100)	37 (29 to 47)	40 (30 to 52)	0 (0 to 0)	8 (8 to 8)	262 (219 to 311)	114 (89 to 145)	124 (92 to 162)	0 (0 to 0)	23 (23 to 23)
Kyrgyzstan	2014	86 (72 to 103)	36 (28 to 45)	43 (32 to 57)	0 (0 to 0)	8 (8 to 8)	269 (224 to 320)	111 (86 to 140)	133 (100 to 177)	0 (0 to 0)	24 (24 to 24)

Kyrgyzstan	2015	84 (70 to 99)	33 (26 to 43)	43 (32 to 58)	0 (0 to 0)	7 (7 to 7)	260 (217 to 309)	104 (81 to 133)	135 (101 to 179)	0 (0 to 0)	21 (21 to 21)
Kyrgyzstan	2016	83 (69 to 100)	33 (25 to 42)	44 (32 to 58)	0 (0 to 0)	6 (6 to 6)	257 (214 to 309)	103 (79 to 132)	137 (100 to 180)	0 (0 to 0)	18 (18 to 18)
Kyrgyzstan	2017	82 (68 to 99)	32 (24 to 41)	44 (32 to 58)	0 (0 to 0)	7 (7 to 7)	256 (210 to 309)	99 (75 to 128)	136 (99 to 180)	0 (0 to 0)	21 (21 to 21)
Kyrgyzstan	2018	80 (67 to 97)	30 (23 to 38)	44 (32 to 58)	0 (0 to 0)	6 (6 to 6)	250 (207 to 301)	93 (70 to 119)	137 (100 to 181)	0 (0 to 0)	20 (20 to 20)
Kyrgyzstan	2019	83 (68 to 100)	32 (24 to 41)	45 (32 to 59)	0 (0 to 0)	7 (7 to 7)	259 (210 to 312)	100 (76 to 128)	138 (101 to 183)	0 (0 to 0)	20 (20 to 20)
Kyrgyzstan	2020	85 (70 to 103)	33 (25 to 43)	45 (33 to 60)	0 (0 to 0)	7 (6 to 7)	264 (219 to 319)	103 (77 to 135)	141 (103 to 186)	0 (0 to 0)	21 (20 to 22)
Kyrgyzstan	2021	86 (70 to 104)	34 (25 to 45)	45 (33 to 60)	0 (0 to 0)	7 (6 to 7)	268 (218 to 323)	105 (78 to 138)	141 (103 to 187)	0 (0 to 0)	21 (20 to 23)
Kyrgyzstan	2022	87 (72 to 105)	35 (25 to 45)	46 (33 to 61)	0 (0 to 0)	7 (6 to 8)	271 (224 to 327)	108 (79 to 141)	142 (103 to 188)	0 (0 to 0)	21 (20 to 24)
Kyrgyzstan	2023	88 (72 to 107)	36 (25 to 47)	46 (33 to 61)	0 (0 to 0)	7 (6 to 8)	274 (222 to 334)	111 (79 to 146)	142 (104 to 189)	0 (0 to 0)	22 (20 to 24)
Kyrgyzstan	2024	89 (73 to 109)	37 (26 to 48)	46 (33 to 61)	0 (0 to 0)	7 (6 to 8)	278 (228 to 338)	114 (80 to 151)	142 (104 to 189)	0 (0 to 0)	22 (19 to 24)
Kyrgyzstan	2025	91 (73 to 110)	38 (27 to 50)	46 (33 to 61)	0 (0 to 0)	7 (6 to 8)	282 (228 to 341)	117 (83 to 155)	143 (104 to 189)	0 (0 to 0)	22 (20 to 25)
Kyrgyzstan	2026	92 (76 to 112)	39 (28 to 52)	46 (34 to 61)	0 (0 to 0)	7 (6 to 8)	286 (235 to 347)	120 (86 to 160)	143 (104 to 190)	0 (0 to 0)	22 (20 to 25)
Kyrgyzstan	2027	93 (76 to 113)	40 (28 to 53)	46 (34 to 61)	0 (0 to 0)	7 (6 to 8)	290 (235 to 352)	124 (88 to 166)	144 (105 to 191)	0 (0 to 0)	23 (20 to 26)
Kyrgyzstan	2028	95 (78 to 116)	41 (29 to 55)	47 (34 to 62)	0 (0 to 0)	7 (6 to 9)	296 (242 to 361)	128 (91 to 172)	145 (105 to 192)	0 (0 to 0)	23 (20 to 27)
Kyrgyzstan	2029	97 (78 to 118)	43 (30 to 58)	47 (34 to 62)	0 (0 to 0)	7 (6 to 9)	301 (242 to 368)	132 (94 to 179)	146 (106 to 194)	0 (0 to 0)	23 (20 to 27)

Kyrgyzstan	2030	99 (80 to 121)	44 (31 to 60)	48 (35 to 63)	0 (0 to 0)	7 (6 to 9)	307 (250 to 376)	136 (95 to 188)	148 (107 to 196)	0 (0 to 0)	23 (20 to 28)
Kyrgyzstan	2031	101 (81 to 124)	45 (31 to 62)	48 (35 to 64)	0 (0 to 0)	8 (6 to 9)	313 (251 to 384)	140 (97 to 194)	149 (109 to 198)	0 (0 to 0)	24 (20 to 28)
Kyrgyzstan	2032	103 (83 to 127)	46 (32 to 65)	49 (35 to 64)	0 (0 to 0)	8 (7 to 9)	319 (259 to 395)	144 (99 to 201)	151 (110 to 200)	0 (0 to 0)	24 (20 to 29)
Kyrgyzstan	2033	105 (83 to 129)	48 (32 to 67)	49 (36 to 65)	0 (0 to 0)	8 (7 to 9)	325 (258 to 401)	148 (101 to 208)	153 (111 to 202)	0 (0 to 0)	24 (21 to 29)
Kyrgyzstan	2034	107 (86 to 132)	49 (33 to 69)	50 (36 to 66)	0 (0 to 0)	8 (7 to 9)	332 (267 to 410)	153 (103 to 214)	155 (112 to 205)	0 (0 to 0)	24 (20 to 29)
Kyrgyzstan	2035	109 (86 to 134)	50 (34 to 71)	50 (37 to 67)	0 (0 to 0)	8 (7 to 10)	338 (268 to 416)	157 (105 to 220)	157 (114 to 208)	0 (0 to 0)	25 (21 to 30)
Kyrgyzstan	2036	111 (89 to 137)	52 (34 to 73)	51 (37 to 68)	0 (0 to 0)	8 (7 to 10)	344 (276 to 425)	160 (107 to 226)	158 (115 to 211)	0 (0 to 0)	25 (21 to 31)
Kyrgyzstan	2037	112 (88 to 139)	53 (35 to 74)	51 (37 to 69)	0 (0 to 0)	8 (7 to 10)	349 (273 to 433)	164 (109 to 231)	160 (116 to 213)	0 (0 to 0)	25 (21 to 31)
Kyrgyzstan	2038	114 (91 to 141)	54 (36 to 77)	52 (38 to 69)	0 (0 to 0)	8 (7 to 10)	354 (284 to 438)	167 (111 to 240)	161 (117 to 215)	0 (0 to 0)	25 (21 to 32)
Kyrgyzstan	2039	116 (90 to 145)	55 (37 to 78)	52 (38 to 70)	0 (0 to 0)	8 (7 to 10)	360 (280 to 452)	171 (115 to 243)	163 (118 to 218)	0 (0 to 0)	26 (21 to 32)
Kyrgyzstan	2040	117 (94 to 148)	56 (38 to 81)	53 (38 to 71)	0 (0 to 0)	8 (7 to 11)	365 (292 to 459)	175 (118 to 252)	165 (120 to 220)	0 (0 to 0)	26 (21 to 33)
Kyrgyzstan	2041	119 (93 to 148)	57 (38 to 83)	54 (39 to 71)	0 (0 to 0)	8 (7 to 11)	371 (288 to 460)	178 (119 to 257)	166 (121 to 221)	0 (0 to 0)	26 (21 to 33)
Kyrgyzstan	2042	121 (95 to 152)	58 (39 to 84)	54 (39 to 72)	0 (0 to 0)	9 (7 to 11)	376 (296 to 473)	181 (121 to 262)	168 (121 to 223)	0 (0 to 0)	26 (22 to 34)
Kyrgyzstan	2043	123 (96 to 154)	59 (40 to 85)	55 (39 to 72)	0 (0 to 0)	9 (7 to 11)	381 (297 to 479)	185 (124 to 265)	169 (123 to 225)	0 (0 to 0)	27 (22 to 35)
Kyrgyzstan	2044	124 (98 to 156)	60 (40 to 88)	55 (40 to 73)	0 (0 to 0)	9 (7 to 12)	386 (305 to 485)	188 (125 to 272)	171 (124 to 227)	0 (0 to 0)	27 (22 to 36)

Kyrgyzstan	2045	126 (97 to 159)	62 (40 to 91)	55 (40 to 74)	0 (0 to 0)	9 (7 to 11)	391 (303 to 494)	191 (124 to 281)	172 (125 to 229)	0 (0 to 0)	27 (22 to 35)
Kyrgyzstan	2046	127 (100 to 161)	62 (41 to 91)	56 (41 to 74)	0 (0 to 0)	9 (7 to 11)	396 (311 to 500)	194 (127 to 282)	174 (126 to 231)	0 (0 to 0)	28 (22 to 35)
Kyrgyzstan	2047	129 (100 to 165)	64 (41 to 94)	56 (41 to 75)	0 (0 to 0)	9 (7 to 12)	400 (310 to 511)	198 (127 to 292)	175 (127 to 233)	0 (0 to 0)	28 (22 to 37)
Kyrgyzstan	2048	130 (103 to 168)	65 (42 to 96)	57 (41 to 76)	0 (0 to 0)	9 (7 to 12)	405 (320 to 523)	201 (130 to 298)	176 (128 to 235)	0 (0 to 0)	28 (23 to 38)
Kyrgyzstan	2049	132 (101 to 167)	66 (43 to 99)	57 (41 to 76)	0 (0 to 0)	9 (7 to 12)	410 (315 to 518)	204 (133 to 306)	177 (128 to 236)	0 (0 to 0)	29 (23 to 38)
Kyrgyzstan	2050	133 (105 to 172)	67 (43 to 101)	57 (41 to 76)	0 (0 to 0)	9 (7 to 13)	415 (325 to 533)	208 (135 to 315)	178 (129 to 238)	0 (0 to 0)	29 (23 to 39)
Laos	1995	34 (28 to 41)	12 (9 to 16)	18 (12 to 25)	2 (1 to 3)	2 (2 to 2)	101 (83 to 123)	37 (27 to 48)	53 (37 to 74)	5 (3 to 10)	7 (7 to 7)
Laos	1996	33 (27 to 41)	12 (9 to 16)	18 (12 to 24)	2 (1 to 3)	1 (1 to 1)	100 (81 to 121)	37 (28 to 49)	53 (37 to 73)	6 (3 to 10)	4 (4 to 4)
Laos	1997	34 (28 to 42)	13 (9 to 16)	18 (13 to 25)	2 (1 to 4)	1 (1 to 1)	102 (84 to 124)	38 (28 to 49)	54 (38 to 74)	6 (3 to 11)	4 (4 to 4)
Laos	1998	34 (28 to 42)	12 (9 to 16)	18 (13 to 25)	2 (1 to 4)	2 (2 to 2)	103 (84 to 126)	36 (28 to 47)	54 (38 to 74)	7 (3 to 12)	6 (6 to 6)
Laos	1999	36 (29 to 43)	12 (9 to 15)	18 (13 to 25)	3 (1 to 5)	3 (3 to 3)	107 (88 to 130)	36 (27 to 46)	55 (39 to 76)	8 (4 to 14)	8 (8 to 8)
Laos	2000	39 (32 to 47)	12 (9 to 16)	19 (13 to 26)	3 (2 to 6)	5 (5 to 5)	116 (97 to 141)	35 (27 to 46)	56 (40 to 77)	10 (5 to 18)	15 (15 to 15)
Laos	2001	37 (30 to 46)	12 (9 to 16)	19 (14 to 26)	3 (2 to 6)	3 (3 to 3)	111 (90 to 137)	36 (27 to 47)	58 (41 to 79)	9 (5 to 18)	8 (8 to 8)
Laos	2002	35 (28 to 43)	11 (8 to 14)	19 (14 to 26)	3 (2 to 6)	2 (2 to 2)	105 (85 to 129)	32 (24 to 41)	57 (41 to 78)	9 (5 to 17)	7 (7 to 7)
Laos	2003	40 (33 to 49)	11 (8 to 14)	20 (14 to 28)	3 (2 to 6)	6 (6 to 6)	120 (99 to 147)	32 (24 to 41)	60 (43 to 83)	10 (5 to 19)	18 (18 to 18)

Laos	2004	34 (28 to 42)	9 (7 to 12)	18 (13 to 24)	3 (2 to 6)	4 (4 to 4)	103 (85 to 127)	27 (21 to 35)	54 (38 to 73)	10 (5 to 18)	13 (13 to 13)
Laos	2005	38 (31 to 45)	9 (7 to 12)	17 (12 to 24)	4 (2 to 7)	7 (7 to 7)	112 (93 to 135)	28 (22 to 37)	52 (36 to 71)	12 (6 to 21)	21 (21 to 21)
Laos	2006	35 (29 to 42)	9 (7 to 11)	16 (11 to 22)	4 (2 to 8)	6 (6 to 6)	104 (86 to 126)	26 (20 to 34)	47 (33 to 65)	13 (7 to 23)	18 (18 to 18)
Laos	2007	37 (31 to 45)	9 (7 to 11)	15 (11 to 21)	6 (3 to 11)	7 (7 to 7)	111 (91 to 134)	26 (20 to 34)	45 (32 to 62)	18 (9 to 32)	22 (22 to 22)
Laos	2008	38 (31 to 45)	9 (7 to 11)	14 (10 to 20)	6 (3 to 11)	8 (8 to 8)	112 (93 to 135)	26 (20 to 34)	43 (30 to 59)	19 (9 to 34)	24 (24 to 24)
Laos	2009	41 (34 to 49)	11 (8 to 14)	16 (11 to 21)	6 (3 to 12)	8 (8 to 8)	122 (102 to 146)	32 (24 to 42)	48 (33 to 64)	19 (9 to 35)	24 (24 to 24)
Laos	2010	42 (35 to 49)	10 (8 to 14)	17 (12 to 23)	4 (2 to 8)	10 (10 to 10)	124 (105 to 145)	31 (24 to 41)	51 (35 to 68)	13 (6 to 24)	29 (29 to 29)
Laos	2011	39 (33 to 45)	9 (7 to 12)	17 (12 to 23)	2 (1 to 3)	11 (11 to 11)	117 (99 to 135)	28 (21 to 36)	52 (36 to 69)	6 (3 to 10)	32 (32 to 32)
Laos	2012	42 (35 to 49)	11 (8 to 14)	19 (13 to 26)	2 (1 to 3)	10 (10 to 10)	125 (106 to 145)	32 (24 to 41)	57 (40 to 77)	5 (3 to 9)	31 (31 to 31)
Laos	2013	48 (41 to 56)	13 (10 to 17)	22 (15 to 30)	2 (1 to 3)	12 (12 to 12)	145 (122 to 169)	39 (30 to 50)	65 (45 to 88)	5 (2 to 9)	36 (36 to 36)
Laos	2014	51 (43 to 60)	15 (11 to 19)	23 (16 to 32)	2 (1 to 3)	11 (11 to 11)	152 (128 to 180)	44 (34 to 57)	70 (49 to 95)	5 (2 to 9)	34 (34 to 34)
Laos	2015	55 (47 to 66)	17 (13 to 22)	25 (18 to 34)	2 (1 to 3)	12 (12 to 12)	166 (140 to 196)	51 (38 to 66)	75 (53 to 101)	5 (2 to 9)	35 (35 to 35)
Laos	2016	52 (43 to 63)	17 (13 to 22)	25 (18 to 34)	2 (1 to 3)	8 (8 to 8)	155 (129 to 187)	52 (39 to 67)	76 (54 to 103)	5 (2 to 9)	23 (23 to 23)
Laos	2017	58 (48 to 70)	20 (15 to 26)	27 (20 to 38)	2 (1 to 3)	9 (9 to 9)	173 (143 to 210)	59 (44 to 78)	82 (59 to 112)	5 (2 to 9)	27 (27 to 27)
Laos	2018	58 (48 to 71)	19 (14 to 25)	29 (21 to 39)	2 (1 to 3)	8 (8 to 9)	173 (143 to 212)	56 (42 to 74)	87 (62 to 118)	5 (3 to 10)	25 (25 to 25)

Laos	2019	60 (50 to 74)	20 (15 to 26)	30 (22 to 41)	2 (1 to 3)	8 (8 to 9)	180 (148 to 220)	59 (44 to 78)	91 (65 to 124)	5 (3 to 10)	25 (25 to 25)
Laos	2020	62 (51 to 77)	20 (15 to 27)	32 (23 to 43)	2 (1 to 4)	8 (8 to 9)	187 (153 to 230)	61 (46 to 81)	95 (67 to 129)	6 (3 to 11)	25 (24 to 27)
Laos	2021	64 (52 to 79)	21 (16 to 28)	33 (23 to 45)	2 (1 to 4)	9 (8 to 9)	192 (157 to 237)	63 (47 to 83)	98 (70 to 134)	6 (3 to 11)	26 (24 to 28)
Laos	2022	66 (54 to 81)	21 (16 to 28)	34 (24 to 46)	2 (1 to 4)	9 (8 to 9)	197 (161 to 243)	64 (48 to 85)	101 (72 to 138)	6 (3 to 12)	26 (24 to 28)
Laos	2023	67 (55 to 83)	22 (16 to 29)	35 (25 to 48)	2 (1 to 4)	9 (8 to 10)	202 (164 to 249)	65 (49 to 86)	104 (75 to 143)	6 (3 to 12)	26 (23 to 29)
Laos	2024	69 (56 to 85)	22 (17 to 29)	36 (26 to 49)	2 (1 to 4)	9 (8 to 10)	207 (169 to 255)	67 (50 to 88)	108 (77 to 147)	7 (3 to 12)	25 (23 to 29)
Laos	2025	71 (58 to 87)	23 (17 to 30)	37 (26 to 51)	2 (1 to 4)	9 (8 to 10)	212 (173 to 261)	69 (52 to 90)	111 (79 to 152)	7 (3 to 13)	25 (23 to 29)
Laos	2026	73 (59 to 90)	24 (18 to 31)	38 (27 to 53)	2 (1 to 4)	9 (8 to 10)	219 (177 to 270)	71 (53 to 94)	115 (82 to 158)	7 (3 to 13)	26 (23 to 29)
Laos	2027	75 (61 to 93)	25 (19 to 33)	40 (28 to 54)	2 (1 to 5)	9 (8 to 10)	226 (183 to 278)	74 (55 to 98)	119 (85 to 163)	7 (4 to 14)	26 (22 to 30)
Laos	2028	78 (63 to 96)	26 (19 to 34)	41 (29 to 56)	3 (1 to 5)	9 (7 to 10)	233 (189 to 288)	76 (57 to 101)	123 (87 to 168)	8 (4 to 14)	26 (22 to 30)
Laos	2029	80 (65 to 100)	26 (20 to 35)	42 (30 to 58)	3 (1 to 5)	9 (7 to 10)	240 (194 to 298)	79 (59 to 105)	127 (90 to 173)	8 (4 to 15)	26 (22 to 30)
Laos	2030	83 (67 to 103)	27 (20 to 37)	44 (31 to 60)	3 (1 to 5)	9 (7 to 10)	247 (201 to 309)	82 (61 to 110)	131 (93 to 179)	8 (4 to 15)	26 (22 to 31)
Laos	2031	85 (69 to 105)	28 (21 to 39)	45 (32 to 62)	3 (1 to 5)	9 (7 to 10)	255 (208 to 315)	85 (63 to 115)	135 (97 to 185)	8 (4 to 16)	26 (22 to 31)
Laos	2032	88 (71 to 109)	30 (22 to 40)	47 (33 to 64)	3 (1 to 6)	9 (7 to 11)	263 (212 to 327)	88 (65 to 119)	140 (100 to 191)	9 (4 to 17)	26 (22 to 32)
Laos	2033	91 (73 to 113)	31 (23 to 41)	48 (34 to 66)	3 (1 to 6)	9 (7 to 11)	272 (219 to 338)	92 (68 to 124)	145 (103 to 198)	9 (4 to 17)	26 (22 to 32)

Laos	2034	94 (75 to 118)	32 (23 to 43)	50 (36 to 68)	3 (2 to 6)	9 (7 to 11)	282 (226 to 352)	96 (70 to 129)	150 (107 to 205)	9 (5 to 18)	27 (22 to 33)
Laos	2035	97 (78 to 121)	33 (24 to 45)	52 (37 to 71)	3 (2 to 6)	9 (7 to 11)	291 (232 to 362)	99 (72 to 134)	155 (111 to 212)	10 (5 to 19)	27 (22 to 33)
Laos	2036	101 (81 to 126)	35 (25 to 47)	54 (38 to 74)	3 (2 to 6)	9 (7 to 11)	301 (241 to 376)	103 (75 to 141)	161 (114 to 220)	10 (5 to 19)	27 (22 to 34)
Laos	2037	104 (84 to 131)	36 (26 to 49)	56 (40 to 76)	4 (2 to 7)	9 (7 to 11)	312 (251 to 391)	108 (78 to 147)	167 (118 to 229)	11 (5 to 20)	27 (22 to 33)
Laos	2038	108 (87 to 136)	38 (27 to 50)	58 (41 to 79)	4 (2 to 7)	9 (7 to 12)	323 (259 to 406)	112 (81 to 151)	173 (122 to 237)	11 (5 to 21)	27 (22 to 35)
Laos	2039	112 (90 to 141)	39 (28 to 53)	60 (42 to 82)	4 (2 to 7)	9 (7 to 12)	334 (269 to 421)	117 (84 to 160)	178 (126 to 246)	12 (6 to 22)	27 (22 to 35)
Laos	2040	115 (91 to 145)	41 (29 to 56)	62 (43 to 85)	4 (2 to 8)	9 (7 to 12)	345 (273 to 433)	121 (87 to 166)	184 (130 to 255)	12 (6 to 23)	28 (22 to 36)
Laos	2041	119 (94 to 150)	42 (30 to 58)	64 (45 to 88)	4 (2 to 8)	9 (7 to 12)	357 (282 to 450)	126 (90 to 174)	191 (134 to 264)	12 (6 to 24)	28 (22 to 36)
Laos	2042	123 (98 to 154)	44 (31 to 60)	66 (46 to 91)	4 (2 to 8)	9 (7 to 12)	369 (292 to 462)	131 (94 to 180)	197 (139 to 272)	13 (6 to 24)	28 (22 to 36)
Laos	2043	127 (99 to 160)	46 (32 to 63)	68 (48 to 94)	4 (2 to 8)	9 (7 to 12)	381 (296 to 479)	137 (97 to 189)	203 (143 to 280)	13 (7 to 25)	28 (22 to 37)
Laos	2044	132 (104 to 165)	48 (34 to 65)	70 (49 to 97)	5 (2 to 9)	10 (7 to 13)	394 (312 to 494)	142 (101 to 196)	210 (148 to 290)	14 (7 to 26)	29 (22 to 38)
Laos	2045	136 (107 to 170)	49 (35 to 68)	72 (51 to 100)	5 (2 to 9)	10 (7 to 13)	407 (321 to 509)	148 (104 to 205)	216 (152 to 299)	14 (7 to 27)	29 (22 to 38)
Laos	2046	141 (111 to 177)	51 (36 to 70)	74 (52 to 103)	5 (2 to 9)	10 (7 to 13)	421 (332 to 528)	154 (108 to 208)	223 (156 to 309)	15 (7 to 28)	29 (22 to 39)
Laos	2047	145 (115 to 181)	54 (38 to 73)	77 (54 to 106)	5 (3 to 10)	10 (8 to 13)	434 (343 to 540)	160 (113 to 217)	230 (161 to 319)	15 (8 to 29)	29 (23 to 40)
Laos	2048	150 (119 to 187)	56 (39 to 76)	79 (56 to 110)	5 (3 to 10)	10 (8 to 13)	449 (357 to 560)	167 (117 to 228)	236 (166 to 328)	16 (8 to 30)	30 (23 to 40)

Laos	2049	155 (122 to 193)	58 (40 to 81)	81 (57 to 113)	6 (3 to 10)	10 (8 to 14)	463 (364 to 577)	173 (121 to 241)	243 (171 to 337)	17 (8 to 31)	30 (23 to 41)
Laos	2050	160 (126 to 200)	60 (42 to 82)	84 (59 to 116)	6 (3 to 11)	10 (8 to 14)	478 (377 to 598)	181 (125 to 247)	250 (176 to 346)	17 (8 to 32)	30 (23 to 42)
Latvia	1995	331 (300 to 364)	188 (166 to 211)	139 (119 to 163)	3 (2 to 6)	0 (0 to 0)	573 (520 to 631)	325 (288 to 366)	241 (206 to 282)	6 (3 to 11)	1 (1 to 1)
Latvia	1996	340 (314 to 370)	186 (167 to 206)	151 (131 to 173)	3 (2 to 6)	0 (0 to 0)	590 (544 to 641)	323 (290 to 356)	261 (228 to 300)	6 (3 to 11)	0 (0 to 0)
Latvia	1997	363 (338 to 391)	195 (178 to 213)	164 (145 to 185)	4 (2 to 7)	0 (0 to 0)	629 (585 to 678)	338 (309 to 369)	284 (251 to 320)	7 (3 to 12)	0 (0 to 0)
Latvia	1998	391 (366 to 419)	210 (193 to 227)	176 (157 to 196)	5 (2 to 9)	0 (0 to 0)	678 (634 to 727)	365 (335 to 393)	305 (272 to 340)	9 (4 to 15)	0 (0 to 0)
Latvia	1999	418 (391 to 447)	222 (205 to 241)	189 (170 to 209)	6 (3 to 10)	0 (0 to 0)	723 (678 to 774)	384 (354 to 417)	328 (294 to 362)	10 (5 to 18)	1 (1 to 1)
Latvia	2000	427 (401 to 458)	220 (203 to 239)	199 (180 to 219)	7 (4 to 12)	1 (1 to 1)	740 (695 to 793)	381 (352 to 413)	345 (311 to 379)	13 (6 to 21)	1 (1 to 1)
Latvia	2001	457 (429 to 486)	230 (213 to 249)	216 (195 to 237)	9 (5 to 16)	1 (1 to 1)	792 (744 to 842)	399 (369 to 432)	375 (337 to 411)	16 (8 to 27)	2 (2 to 2)
Latvia	2002	506 (476 to 538)	258 (239 to 278)	236 (213 to 258)	11 (6 to 20)	2 (2 to 2)	877 (824 to 933)	446 (414 to 481)	408 (370 to 448)	19 (10 to 34)	4 (4 to 4)
Latvia	2003	544 (513 to 580)	284 (263 to 306)	249 (226 to 272)	12 (6 to 20)	0 (0 to 0)	943 (889 to 1004)	491 (455 to 531)	431 (392 to 472)	20 (11 to 34)	1 (1 to 1)
Latvia	2004	618 (584 to 655)	337 (314 to 362)	265 (241 to 291)	14 (8 to 24)	1 (1 to 1)	1070 (1011 to 1135)	584 (544 to 627)	460 (418 to 505)	25 (14 to 42)	2 (2 to 2)
Latvia	2005	683 (648 to 722)	387 (361 to 414)	280 (255 to 306)	16 (9 to 27)	0 (0 to 0)	1184 (1122 to 1250)	671 (626 to 717)	485 (442 to 530)	28 (16 to 48)	0 (0 to 0)
Latvia	2006	755 (718 to 795)	444 (416 to 472)	293 (269 to 319)	18 (10 to 31)	0 (0 to 0)	1309 (1244 to 1377)	769 (721 to 817)	508 (467 to 552)	32 (18 to 54)	0 (0 to 0)

Latvia	2007	796 (759 to 836)	471 (444 to 499)	306 (283 to 333)	18 (10 to 30)	0 (0 to 0)	1379 (1316 to 1448)	817 (770 to 864)	530 (490 to 577)	31 (17 to 53)	0 (0 to 0)
Latvia	2008	811 (774 to 848)	488 (463 to 514)	305 (283 to 331)	17 (10 to 29)	1 (1 to 1)	1405 (1341 to 1469)	845 (802 to 891)	529 (490 to 573)	30 (17 to 50)	1 (1 to 1)
Latvia	2009	779 (745 to 813)	472 (448 to 497)	292 (270 to 316)	15 (8 to 24)	0 (0 to 0)	1350 (1291 to 1409)	818 (776 to 861)	506 (467 to 548)	26 (14 to 41)	0 (0 to 0)
Latvia	2010	779 (746 to 812)	461 (437 to 487)	280 (259 to 303)	16 (8 to 25)	22 (22 to 22)	1349 (1293 to 1407)	798 (757 to 844)	485 (448 to 525)	27 (15 to 43)	39 (39 to 39)
Latvia	2011	761 (727 to 795)	451 (426 to 476)	272 (252 to 295)	14 (8 to 23)	24 (24 to 24)	1319 (1260 to 1377)	781 (739 to 824)	472 (436 to 510)	25 (13 to 40)	41 (41 to 41)
Latvia	2012	743 (709 to 778)	449 (425 to 474)	281 (260 to 302)	13 (7 to 21)	0 (0 to 0)	1286 (1228 to 1347)	778 (736 to 820)	486 (450 to 524)	22 (12 to 36)	0 (0 to 0)
Latvia	2013	773 (741 to 806)	463 (439 to 487)	298 (277 to 320)	11 (6 to 18)	0 (0 to 0)	1338 (1283 to 1396)	802 (761 to 844)	516 (480 to 554)	20 (11 to 32)	0 (0 to 0)
Latvia	2014	820 (790 to 854)	486 (465 to 509)	325 (303 to 346)	10 (5 to 17)	0 (0 to 0)	1421 (1369 to 1479)	842 (805 to 882)	562 (525 to 599)	17 (9 to 29)	0 (0 to 0)
Latvia	2015	887 (855 to 921)	516 (494 to 539)	361 (340 to 385)	9 (5 to 15)	0 (0 to 0)	1536 (1481 to 1596)	895 (856 to 935)	626 (590 to 668)	15 (8 to 26)	0 (0 to 0)
Latvia	2016	941 (903 to 980)	535 (510 to 560)	398 (373 to 424)	8 (4 to 14)	0 (0 to 0)	1630 (1564 to 1697)	927 (883 to 970)	689 (645 to 735)	14 (7 to 24)	0 (0 to 0)

Latvia	2017	1005 (953 to 1061)	571 (537 to 607)	425 (390 to 462)	8 (4 to 14)	0 (0 to 0)	1741 (1651 to 1839)	990 (930 to 1051)	737 (676 to 800)	14 (7 to 25)	0 (0 to 0)
Latvia	2018	1066 (1009 to 1128)	613 (572 to 654)	445 (408 to 483)	8 (4 to 15)	0 (0 to 0)	1847 (1749 to 1955)	1061 (990 to 1132)	771 (707 to 837)	15 (8 to 26)	0 (0 to 0)
Latvia	2019	1074 (1014 to 1139)	611 (566 to 655)	455 (417 to 494)	9 (5 to 16)	0 (0 to 0)	1861 (1756 to 1973)	1059 (980 to 1134)	788 (722 to 856)	15 (8 to 27)	0 (0 to 0)
Latvia	2020	1107 (1045 to 1175)	631 (583 to 679)	467 (428 to 508)	9 (5 to 16)	0 (0 to 0)	1917 (1810 to 2035)	1094 (1010 to 1177)	808 (741 to 880)	16 (8 to 28)	0 (0 to 0)
Latvia	2021	1127 (1063 to 1201)	645 (593 to 697)	473 (434 to 515)	9 (5 to 17)	0 (0 to 0)	1953 (1841 to 2081)	1117 (1028 to 1208)	820 (752 to 893)	16 (8 to 29)	0 (0 to 0)
Latvia	2022	1147 (1079 to 1223)	658 (605 to 710)	480 (441 to 523)	9 (5 to 17)	0 (0 to 0)	1988 (1870 to 2119)	1140 (1048 to 1230)	832 (763 to 906)	16 (8 to 29)	0 (0 to 0)
Latvia	2023	1167 (1094 to 1250)	671 (612 to 729)	487 (447 to 530)	10 (5 to 17)	0 (0 to 0)	2023 (1895 to 2165)	1162 (1061 to 1263)	844 (775 to 919)	17 (9 to 30)	0 (0 to 0)
Latvia	2024	1178 (1100 to 1261)	678 (620 to 740)	491 (450 to 534)	10 (5 to 18)	0 (0 to 0)	2041 (1906 to 2185)	1175 (1074 to 1283)	850 (779 to 925)	17 (9 to 30)	0 (0 to 0)
Latvia	2025	1186 (1109 to 1272)	684 (623 to 751)	493 (452 to 536)	10 (5 to 18)	0 (0 to 0)	2055 (1922 to 2204)	1185 (1080 to 1301)	853 (782 to 928)	17 (9 to 31)	0 (0 to 0)
Latvia	2026	1198 (1120 to 1285)	692 (629 to 763)	496 (455 to 539)	10 (5 to 18)	0 (0 to 0)	2076 (1940 to 2226)	1199 (1090 to 1321)	860 (788 to 934)	17 (9 to 31)	0 (0 to 0)

Latvia	2027	1214 (1131 to 1306)	703 (639 to 776)	501 (459 to 545)	10 (5 to 18)	0 (0 to 0)	2103 (1960 to 2262)	1217 (1106 to 1345)	868 (795 to 943)	17 (9 to 31)	0 (0 to 0)
Latvia	2028	1233 (1149 to 1327)	715 (647 to 789)	507 (464 to 551)	10 (5 to 18)	0 (0 to 0)	2136 (1990 to 2298)	1239 (1121 to 1367)	879 (805 to 954)	18 (9 to 32)	0 (0 to 0)
Latvia	2029	1254 (1165 to 1352)	729 (657 to 809)	515 (470 to 560)	10 (5 to 19)	0 (0 to 0)	2172 (2019 to 2341)	1263 (1138 to 1402)	892 (815 to 969)	18 (9 to 32)	0 (0 to 0)
Latvia	2030	1278 (1186 to 1377)	744 (669 to 827)	523 (477 to 568)	11 (6 to 19)	0 (0 to 0)	2213 (2054 to 2386)	1289 (1159 to 1433)	905 (827 to 984)	18 (10 to 33)	0 (0 to 0)
Latvia	2031	1302 (1209 to 1407)	760 (681 to 846)	531 (485 to 578)	11 (6 to 19)	0 (0 to 0)	2256 (2095 to 2438)	1317 (1179 to 1466)	920 (840 to 1001)	19 (10 to 33)	0 (0 to 0)
Latvia	2032	1330 (1230 to 1439)	778 (694 to 870)	541 (493 to 588)	11 (6 to 20)	0 (0 to 0)	2304 (2131 to 2492)	1348 (1203 to 1508)	937 (854 to 1019)	19 (10 to 34)	0 (0 to 0)
Latvia	2033	1358 (1254 to 1473)	796 (710 to 894)	551 (501 to 599)	11 (6 to 20)	0 (0 to 0)	2353 (2173 to 2551)	1379 (1229 to 1549)	954 (869 to 1038)	20 (10 to 35)	0 (0 to 0)
Latvia	2034	1389 (1280 to 1510)	816 (724 to 919)	561 (510 to 611)	12 (6 to 21)	0 (0 to 0)	2407 (2217 to 2615)	1414 (1255 to 1591)	973 (884 to 1059)	20 (11 to 36)	0 (0 to 0)
Latvia	2035	1420 (1308 to 1548)	836 (742 to 940)	572 (519 to 623)	12 (6 to 22)	0 (0 to 0)	2461 (2266 to 2681)	1449 (1286 to 1628)	991 (899 to 1080)	21 (11 to 38)	0 (0 to 0)
Latvia	2036	1452 (1336 to 1581)	857 (755 to 966)	583 (529 to 636)	12 (6 to 22)	0 (0 to 0)	2515 (2315 to 2739)	1484 (1308 to 1674)	1010 (916 to 1102)	21 (11 to 39)	0 (0 to 0)

Latvia	2037	1484 (1363 to 1618)	877 (771 to 995)	594 (539 to 649)	13 (7 to 23)	0 (0 to 0)	2570 (2361 to 2803)	1519 (1336 to 1724)	1029 (933 to 1125)	22 (11 to 40)	0 (0 to 0)
Latvia	2038	1514 (1389 to 1656)	897 (791 to 1018)	604 (548 to 661)	13 (7 to 23)	0 (0 to 0)	2623 (2405 to 2869)	1554 (1371 to 1764)	1046 (949 to 1144)	22 (12 to 41)	0 (0 to 0)
Latvia	2039	1545 (1412 to 1695)	917 (808 to 1044)	614 (557 to 673)	13 (7 to 24)	0 (0 to 0)	2676 (2446 to 2936)	1589 (1400 to 1809)	1064 (965 to 1167)	23 (12 to 41)	0 (0 to 0)
Latvia	2040	1577 (1443 to 1736)	939 (823 to 1071)	625 (565 to 686)	14 (7 to 24)	0 (0 to 0)	2732 (2500 to 3007)	1626 (1425 to 1855)	1082 (979 to 1189)	23 (12 to 42)	0 (0 to 0)
Latvia	2041	1607 (1467 to 1773)	958 (837 to 1096)	635 (573 to 698)	14 (7 to 25)	0 (0 to 0)	2784 (2542 to 3071)	1660 (1450 to 1899)	1099 (993 to 1209)	24 (13 to 43)	0 (0 to 0)
Latvia	2042	1637 (1494 to 1803)	978 (852 to 1122)	644 (581 to 708)	14 (7 to 25)	0 (0 to 0)	2836 (2588 to 3124)	1695 (1475 to 1943)	1116 (1007 to 1227)	25 (13 to 44)	0 (0 to 0)
Latvia	2043	1667 (1522 to 1846)	999 (865 to 1148)	654 (589 to 719)	14 (8 to 26)	0 (0 to 0)	2889 (2636 to 3198)	1731 (1499 to 1989)	1133 (1021 to 1246)	25 (13 to 45)	0 (0 to 0)
Latvia	2044	1699 (1544 to 1884)	1020 (878 to 1180)	664 (598 to 731)	15 (8 to 26)	0 (0 to 0)	2944 (2676 to 3264)	1768 (1521 to 2044)	1151 (1035 to 1266)	26 (13 to 46)	0 (0 to 0)
Latvia	2045	1731 (1576 to 1918)	1042 (901 to 1204)	674 (605 to 743)	15 (8 to 27)	0 (0 to 0)	2999 (2730 to 3322)	1806 (1562 to 2086)	1168 (1049 to 1286)	26 (14 to 47)	0 (0 to 0)
Latvia	2046	1763 (1603 to 1960)	1064 (914 to 1235)	684 (613 to 754)	15 (8 to 28)	0 (0 to 0)	3054 (2778 to 3396)	1843 (1583 to 2140)	1184 (1062 to 1307)	27 (14 to 48)	0 (0 to 0)

Latvia	2047	1790 (1627 to 1993)	1082 (930 to 1256)	692 (620 to 765)	16 (8 to 28)	0 (0 to 0)	3101 (2819 to 3453)	1875 (1611 to 2177)	1199 (1073 to 1325)	27 (14 to 49)	0 (0 to 0)
Latvia	2048	1815 (1644 to 2025)	1099 (941 to 1281)	699 (625 to 775)	16 (8 to 29)	0 (0 to 0)	3144 (2848 to 3508)	1904 (1629 to 2219)	1212 (1083 to 1342)	28 (14 to 50)	0 (0 to 0)
Latvia	2049	1836 (1661 to 2050)	1114 (952 to 1307)	706 (629 to 785)	16 (8 to 29)	0 (0 to 0)	3181 (2877 to 3552)	1930 (1650 to 2264)	1222 (1090 to 1359)	28 (14 to 51)	0 (0 to 0)
Latvia	2050	1857 (1675 to 2081)	1129 (960 to 1328)	712 (632 to 795)	16 (8 to 30)	0 (0 to 0)	3217 (2901 to 3605)	1956 (1663 to 2301)	1233 (1095 to 1377)	29 (15 to 51)	0 (0 to 0)
Lebanon	1995	1064 (989 to 1144)	415 (369 to 463)	521 (477 to 571)	122 (94 to 155)	7 (7 to 7)	1684 (1565 to 1811)	656 (584 to 733)	825 (755 to 904)	193 (148 to 246)	11 (11 to 11)
Lebanon	1996	1198 (1132 to 1270)	486 (441 to 536)	570 (530 to 611)	142 (115 to 173)	1 (1 to 1)	1897 (1792 to 2011)	770 (699 to 849)	902 (838 to 967)	224 (182 to 274)	1 (1 to 1)
Lebanon	1997	1147 (1086 to 1213)	444 (405 to 485)	566 (530 to 604)	136 (113 to 163)	2 (2 to 2)	1816 (1719 to 1920)	702 (641 to 768)	895 (839 to 956)	216 (178 to 259)	2 (2 to 2)
Lebanon	1998	1021 (969 to 1078)	350 (319 to 387)	544 (509 to 580)	124 (103 to 148)	2 (2 to 2)	1616 (1534 to 1707)	555 (504 to 612)	862 (806 to 918)	196 (164 to 234)	3 (3 to 3)
Lebanon	1999	953 (900 to 1007)	307 (278 to 341)	525 (491 to 562)	117 (97 to 140)	3 (3 to 3)	1508 (1424 to 1594)	486 (439 to 539)	832 (777 to 889)	185 (154 to 222)	5 (5 to 5)
Lebanon	2000	943 (888 to 996)	307 (275 to 343)	516 (482 to 552)	117 (98 to 140)	2 (2 to 2)	1492 (1406 to 1577)	485 (436 to 543)	817 (764 to 874)	186 (155 to 222)	4 (4 to 4)

Lebanon	2001	868 (822 to 919)	285 (255 to 319)	473 (442 to 506)	108 (90 to 129)	3 (3 to 3)	1374 (1301 to 1455)	450 (403 to 505)	749 (700 to 802)	171 (142 to 204)	4 (4 to 4)
Lebanon	2002	805 (763 to 854)	271 (243 to 302)	430 (401 to 461)	102 (85 to 120)	3 (3 to 3)	1274 (1208 to 1353)	428 (385 to 478)	680 (635 to 730)	161 (134 to 191)	5 (5 to 5)
Lebanon	2003	767 (727 to 812)	268 (241 to 299)	395 (366 to 425)	99 (82 to 119)	5 (5 to 5)	1214 (1150 to 1285)	425 (382 to 472)	625 (579 to 673)	157 (130 to 188)	8 (8 to 8)
Lebanon	2004	738 (696 to 782)	270 (243 to 300)	367 (339 to 395)	98 (80 to 118)	3 (3 to 3)	1168 (1101 to 1238)	428 (384 to 475)	582 (536 to 626)	155 (127 to 187)	4 (4 to 4)
Lebanon	2005	725 (683 to 767)	275 (247 to 303)	351 (322 to 378)	98 (80 to 118)	1 (1 to 1)	1147 (1082 to 1214)	436 (392 to 480)	555 (510 to 598)	155 (126 to 186)	2 (2 to 2)
Lebanon	2006	781 (738 to 827)	309 (278 to 340)	361 (335 to 388)	108 (88 to 130)	2 (2 to 2)	1236 (1168 to 1308)	490 (440 to 539)	572 (530 to 615)	171 (140 to 206)	3 (3 to 3)
Lebanon	2007	819 (774 to 869)	329 (299 to 363)	370 (342 to 400)	116 (95 to 140)	4 (4 to 4)	1297 (1225 to 1375)	521 (473 to 574)	585 (541 to 632)	184 (151 to 222)	6 (6 to 6)
Lebanon	2008	857 (810 to 906)	346 (313 to 380)	377 (348 to 408)	125 (104 to 149)	9 (9 to 9)	1357 (1282 to 1435)	548 (495 to 601)	597 (551 to 646)	198 (164 to 237)	13 (13 to 13)
Lebanon	2009	899 (851 to 948)	373 (339 to 408)	387 (359 to 416)	137 (114 to 162)	2 (2 to 2)	1423 (1346 to 1501)	591 (536 to 646)	612 (569 to 658)	217 (181 to 256)	3 (3 to 3)
Lebanon	2010	932 (881 to 983)	396 (362 to 433)	388 (360 to 417)	144 (121 to 170)	3 (3 to 3)	1475 (1394 to 1555)	627 (572 to 686)	614 (569 to 660)	229 (191 to 269)	5 (5 to 5)

Lebanon	2011	932 (881 to 984)	415 (380 to 452)	368 (339 to 398)	147 (123 to 173)	2 (2 to 2)	1476 (1394 to 1557)	657 (602 to 715)	582 (537 to 630)	233 (194 to 274)	3 (3 to 3)
Lebanon	2012	935 (881 to 990)	444 (408 to 482)	337 (309 to 365)	152 (127 to 178)	2 (2 to 2)	1479 (1394 to 1568)	702 (645 to 763)	533 (489 to 577)	240 (201 to 282)	4 (4 to 4)
Lebanon	2013	890 (840 to 943)	435 (400 to 472)	309 (283 to 334)	145 (121 to 172)	2 (2 to 2)	1409 (1329 to 1492)	688 (633 to 748)	489 (448 to 529)	229 (192 to 272)	3 (3 to 3)
Lebanon	2014	886 (836 to 937)	438 (404 to 476)	300 (276 to 328)	142 (120 to 169)	6 (6 to 6)	1402 (1323 to 1483)	693 (640 to 754)	475 (436 to 519)	224 (190 to 267)	10 (10 to 10)
Lebanon	2015	867 (818 to 918)	432 (397 to 470)	293 (267 to 320)	138 (115 to 164)	3 (3 to 3)	1372 (1294 to 1453)	684 (628 to 744)	464 (422 to 506)	219 (182 to 259)	5 (5 to 5)
Lebanon	2016	899 (842 to 962)	452 (411 to 495)	300 (271 to 332)	144 (116 to 174)	3 (3 to 3)	1423 (1334 to 1522)	715 (651 to 784)	475 (429 to 525)	228 (184 to 275)	5 (5 to 5)
Lebanon	2017	935 (847 to 1028)	473 (420 to 527)	309 (273 to 350)	150 (116 to 188)	4 (4 to 4)	1481 (1341 to 1628)	749 (665 to 835)	489 (432 to 554)	237 (184 to 298)	6 (6 to 6)
Lebanon	2018	953 (857 to 1058)	488 (431 to 549)	310 (274 to 351)	153 (118 to 191)	4 (4 to 4)	1509 (1357 to 1675)	772 (683 to 869)	490 (434 to 556)	241 (187 to 303)	6 (6 to 6)
Lebanon	2019	954 (856 to 1066)	487 (429 to 550)	311 (274 to 353)	153 (119 to 192)	4 (4 to 4)	1511 (1355 to 1687)	771 (678 to 871)	492 (434 to 558)	242 (188 to 303)	6 (6 to 6)
Lebanon	2020	971 (864 to 1091)	497 (434 to 567)	315 (278 to 356)	156 (121 to 196)	4 (3 to 4)	1538 (1367 to 1727)	787 (687 to 898)	498 (440 to 564)	247 (192 to 310)	6 (6 to 6)

Lebanon	2021	987 (876 to 1107)	507 (440 to 580)	318 (281 to 360)	159 (124 to 200)	4 (3 to 4)	1563 (1387 to 1752)	802 (697 to 918)	503 (445 to 570)	252 (196 to 316)	6 (5 to 6)
Lebanon	2022	1002 (888 to 1125)	516 (442 to 600)	321 (284 to 363)	162 (126 to 203)	4 (3 to 4)	1586 (1405 to 1781)	817 (700 to 950)	508 (449 to 575)	256 (199 to 321)	6 (5 to 6)
Lebanon	2023	1018 (891 to 1150)	525 (446 to 609)	324 (287 to 368)	165 (128 to 207)	4 (3 to 4)	1612 (1411 to 1820)	832 (707 to 964)	513 (454 to 582)	261 (203 to 327)	6 (5 to 7)
Lebanon	2024	1032 (902 to 1170)	534 (453 to 623)	327 (289 to 371)	167 (130 to 210)	4 (3 to 4)	1634 (1428 to 1851)	845 (717 to 987)	518 (458 to 587)	265 (206 to 332)	6 (5 to 7)
Lebanon	2025	1048 (920 to 1194)	543 (459 to 639)	331 (292 to 375)	170 (132 to 214)	4 (3 to 4)	1659 (1456 to 1891)	860 (727 to 1012)	523 (462 to 594)	270 (209 to 338)	6 (5 to 7)
Lebanon	2026	1064 (917 to 1220)	553 (458 to 659)	334 (294 to 379)	173 (135 to 218)	4 (3 to 4)	1684 (1452 to 1931)	875 (724 to 1044)	529 (466 to 601)	274 (213 to 345)	6 (5 to 7)
Lebanon	2027	1078 (936 to 1240)	561 (460 to 669)	337 (297 to 383)	176 (137 to 222)	4 (3 to 4)	1707 (1482 to 1963)	888 (728 to 1059)	534 (470 to 607)	279 (217 to 351)	6 (5 to 7)
Lebanon	2028	1093 (943 to 1262)	570 (470 to 688)	341 (300 to 387)	179 (139 to 225)	4 (3 to 4)	1731 (1492 to 1998)	902 (743 to 1089)	540 (475 to 612)	283 (220 to 356)	6 (5 to 7)
Lebanon	2029	1109 (955 to 1285)	580 (469 to 708)	344 (302 to 391)	182 (141 to 229)	4 (3 to 4)	1756 (1512 to 2034)	918 (743 to 1120)	545 (479 to 619)	288 (223 to 362)	6 (5 to 7)
Lebanon	2030	1123 (962 to 1301)	588 (477 to 717)	347 (305 to 394)	184 (142 to 232)	4 (3 to 5)	1777 (1523 to 2059)	930 (756 to 1135)	549 (482 to 623)	291 (226 to 367)	6 (5 to 7)

Lebanon	2031	1136 (974 to 1322)	596 (481 to 731)	350 (307 to 397)	187 (144 to 235)	4 (3 to 5)	1798 (1541 to 2093)	943 (761 to 1158)	554 (486 to 628)	295 (228 to 372)	6 (5 to 7)
Lebanon	2032	1148 (982 to 1336)	603 (486 to 746)	352 (309 to 400)	189 (146 to 237)	4 (3 to 5)	1817 (1554 to 2115)	955 (769 to 1180)	557 (489 to 633)	299 (231 to 376)	6 (5 to 7)
Lebanon	2033	1161 (990 to 1354)	611 (488 to 754)	355 (311 to 403)	191 (148 to 240)	4 (3 to 5)	1838 (1567 to 2143)	968 (773 to 1194)	562 (492 to 639)	303 (234 to 380)	6 (5 to 7)
Lebanon	2034	1174 (997 to 1375)	619 (492 to 765)	358 (313 to 407)	194 (150 to 243)	4 (3 to 5)	1858 (1579 to 2177)	980 (779 to 1211)	566 (496 to 644)	306 (237 to 385)	6 (5 to 8)
Lebanon	2035	1185 (1003 to 1395)	625 (495 to 775)	360 (315 to 410)	196 (151 to 246)	4 (3 to 5)	1876 (1588 to 2209)	990 (783 to 1227)	570 (499 to 649)	310 (240 to 390)	6 (5 to 8)
Lebanon	2036	1194 (1001 to 1410)	631 (496 to 786)	362 (316 to 413)	197 (152 to 248)	4 (3 to 5)	1890 (1585 to 2233)	998 (785 to 1244)	573 (500 to 653)	312 (241 to 393)	6 (5 to 8)
Lebanon	2037	1204 (1017 to 1422)	637 (499 to 798)	364 (317 to 415)	199 (154 to 251)	4 (3 to 5)	1906 (1610 to 2250)	1008 (789 to 1263)	576 (502 to 657)	315 (243 to 397)	6 (5 to 8)
Lebanon	2038	1212 (1015 to 1443)	642 (501 to 807)	365 (318 to 417)	201 (155 to 253)	4 (3 to 5)	1918 (1606 to 2285)	1016 (793 to 1277)	578 (503 to 660)	317 (245 to 401)	6 (5 to 8)
Lebanon	2039	1215 (1024 to 1449)	645 (501 to 819)	365 (318 to 420)	201 (155 to 254)	4 (0 to 5)	1924 (1621 to 2294)	1021 (793 to 1297)	578 (503 to 664)	319 (245 to 402)	6 (0 to 8)
Lebanon	2040	1218 (1016 to 1447)	647 (501 to 818)	365 (317 to 419)	202 (155 to 255)	4 (0 to 5)	1927 (1609 to 2291)	1024 (793 to 1295)	578 (502 to 664)	319 (246 to 404)	6 (0 to 8)

Lebanon	2041	1218 (1013 to 1448)	648 (498 to 822)	364 (316 to 420)	202 (155 to 256)	4 (0 to 5)	1928 (1603 to 2292)	1025 (789 to 1301)	577 (500 to 665)	320 (246 to 405)	7 (0 to 9)
Lebanon	2042	1221 (1014 to 1468)	650 (496 to 838)	364 (315 to 420)	203 (156 to 258)	4 (0 to 6)	1933 (1605 to 2324)	1029 (784 to 1327)	576 (498 to 665)	321 (247 to 408)	7 (0 to 9)
Lebanon	2043	1220 (1009 to 1485)	650 (496 to 844)	363 (313 to 420)	203 (156 to 257)	4 (0 to 6)	1932 (1597 to 2350)	1029 (786 to 1335)	575 (496 to 665)	321 (246 to 408)	7 (0 to 9)
Lebanon	2044	1223 (1001 to 1473)	653 (495 to 846)	362 (312 to 421)	203 (156 to 258)	4 (0 to 6)	1935 (1585 to 2332)	1033 (784 to 1339)	574 (494 to 666)	322 (247 to 409)	7 (0 to 10)
Lebanon	2045	1223 (1005 to 1476)	653 (491 to 863)	362 (310 to 421)	204 (156 to 259)	4 (0 to 6)	1936 (1590 to 2337)	1034 (777 to 1365)	572 (491 to 666)	322 (247 to 410)	7 (0 to 9)
Lebanon	2046	1223 (997 to 1484)	654 (488 to 864)	361 (310 to 421)	204 (156 to 259)	4 (0 to 6)	1936 (1578 to 2349)	1036 (773 to 1368)	571 (491 to 666)	323 (247 to 411)	7 (0 to 9)
Lebanon	2047	1223 (1001 to 1522)	655 (492 to 878)	360 (309 to 421)	204 (157 to 259)	4 (0 to 6)	1937 (1585 to 2409)	1037 (778 to 1390)	569 (489 to 667)	323 (248 to 410)	7 (0 to 10)
Lebanon	2048	1222 (998 to 1510)	655 (489 to 877)	358 (307 to 421)	204 (156 to 259)	4 (0 to 6)	1934 (1580 to 2390)	1037 (774 to 1387)	567 (486 to 667)	323 (247 to 410)	7 (0 to 10)
Lebanon	2049	1222 (994 to 1502)	656 (481 to 879)	357 (305 to 420)	204 (156 to 260)	4 (0 to 6)	1934 (1573 to 2378)	1039 (761 to 1392)	565 (482 to 665)	323 (246 to 411)	7 (0 to 10)
Lebanon	2050	1220 (992 to 1499)	657 (490 to 882)	355 (302 to 419)	204 (155 to 260)	4 (0 to 7)	1932 (1571 to 2372)	1039 (776 to 1396)	562 (479 to 664)	323 (245 to 412)	7 (0 to 11)

Lesotho	1995	41 (33 to 49)	19 (14 to 25)	17 (12 to 23)	3 (1 to 6)	1 (1 to 1)	106 (87 to 128)	50 (38 to 64)	45 (31 to 61)	8 (4 to 15)	3 (3 to 3)
Lesotho	1996	42 (34 to 50)	20 (15 to 25)	17 (12 to 23)	4 (2 to 6)	1 (1 to 1)	108 (89 to 130)	51 (39 to 65)	44 (31 to 60)	9 (4 to 17)	4 (4 to 4)
Lesotho	1997	43 (35 to 51)	20 (15 to 25)	17 (12 to 23)	4 (2 to 8)	2 (2 to 2)	112 (92 to 133)	52 (40 to 66)	44 (31 to 60)	11 (5 to 20)	4 (4 to 4)
Lesotho	1998	44 (37 to 53)	22 (17 to 28)	17 (12 to 23)	5 (2 to 9)	1 (1 to 1)	115 (95 to 139)	57 (43 to 72)	43 (30 to 60)	13 (6 to 24)	3 (3 to 3)
Lesotho	1999	44 (37 to 54)	23 (17 to 29)	16 (11 to 22)	5 (3 to 10)	0 (0 to 0)	116 (96 to 140)	59 (45 to 75)	42 (29 to 58)	14 (7 to 26)	1 (1 to 1)
Lesotho	2000	44 (36 to 52)	21 (17 to 27)	15 (10 to 21)	5 (2 to 9)	2 (2 to 2)	114 (94 to 136)	56 (43 to 71)	39 (27 to 54)	13 (6 to 25)	6 (6 to 6)
Lesotho	2001	48 (40 to 58)	24 (19 to 31)	15 (10 to 21)	5 (2 to 10)	3 (3 to 3)	125 (104 to 150)	64 (49 to 82)	39 (27 to 55)	13 (6 to 26)	9 (9 to 9)
Lesotho	2002	49 (41 to 58)	25 (20 to 32)	15 (10 to 21)	5 (2 to 9)	4 (4 to 4)	127 (107 to 151)	66 (51 to 84)	39 (27 to 54)	12 (6 to 23)	10 (10 to 10)
Lesotho	2003	51 (44 to 60)	26 (20 to 32)	15 (10 to 21)	4 (2 to 7)	7 (7 to 7)	134 (114 to 157)	67 (52 to 84)	39 (27 to 54)	10 (5 to 19)	19 (19 to 19)
Lesotho	2004	51 (44 to 60)	25 (20 to 32)	15 (10 to 20)	3 (1 to 6)	8 (8 to 8)	134 (115 to 156)	66 (52 to 82)	38 (27 to 53)	8 (4 to 15)	22 (22 to 22)
Lesotho	2005	53 (46 to 61)	27 (21 to 33)	15 (11 to 21)	3 (1 to 5)	9 (9 to 9)	138 (119 to 160)	69 (55 to 87)	39 (28 to 54)	7 (3 to 14)	22 (22 to 22)
Lesotho	2006	58 (51 to 68)	31 (24 to 38)	15 (11 to 21)	3 (1 to 5)	10 (10 to 10)	152 (132 to 178)	80 (63 to 100)	40 (28 to 55)	7 (3 to 13)	25 (25 to 25)
Lesotho	2007	69 (61 to 80)	35 (28 to 44)	16 (11 to 22)	2 (1 to 4)	16 (16 to 16)	180 (158 to 209)	92 (73 to 114)	41 (29 to 56)	6 (3 to 11)	41 (41 to 41)
Lesotho	2008	86 (76 to 98)	41 (33 to 51)	17 (12 to 23)	2 (1 to 4)	26 (26 to 26)	224 (198 to 255)	108 (86 to 132)	43 (31 to 59)	5 (2 to 9)	68 (68 to 68)
Lesotho	2009	90 (79 to 104)	49 (39 to 60)	18 (13 to 24)	1 (0 to 2)	22 (22 to 22)	236 (205 to 271)	128 (103 to 156)	46 (33 to 63)	3 (1 to 5)	59 (59 to 59)

Lesotho	2010	106 (95 to 118)	48 (38 to 58)	17 (12 to 24)	0 (0 to 1)	40 (40 to 40)	276 (247 to 308)	124 (99 to 150)	45 (33 to 62)	1 (1 to 2)	105 (105 to 105)
Lesotho	2011	128 (115 to 141)	56 (45 to 67)	18 (13 to 25)	0 (0 to 0)	53 (53 to 53)	333 (299 to 368)	145 (116 to 175)	48 (34 to 66)	0 (0 to 1)	139 (139 to 139)
Lesotho	2012	131 (118 to 145)	60 (49 to 72)	19 (13 to 26)	0 (0 to 0)	52 (52 to 52)	341 (306 to 377)	156 (127 to 188)	49 (34 to 67)	0 (0 to 0)	136 (136 to 136)
Lesotho	2013	148 (134 to 163)	65 (53 to 78)	19 (13 to 26)	0 (0 to 0)	64 (64 to 64)	385 (350 to 425)	169 (138 to 203)	49 (35 to 67)	0 (0 to 0)	167 (167 to 167)
Lesotho	2014	106 (93 to 121)	64 (53 to 78)	18 (13 to 25)	0 (0 to 0)	23 (23 to 23)	276 (242 to 315)	168 (137 to 204)	47 (34 to 65)	0 (0 to 0)	61 (61 to 61)
Lesotho	2015	108 (94 to 124)	68 (56 to 83)	18 (13 to 25)	0 (0 to 0)	21 (21 to 21)	281 (246 to 324)	178 (145 to 217)	48 (34 to 66)	0 (0 to 0)	55 (55 to 55)
Lesotho	2016	121 (107 to 139)	69 (56 to 85)	18 (13 to 26)	0 (0 to 0)	33 (33 to 33)	315 (278 to 362)	180 (145 to 222)	48 (34 to 66)	0 (0 to 0)	87 (87 to 87)
Lesotho	2017	129 (113 to 147)	69 (55 to 86)	19 (13 to 26)	0 (0 to 0)	41 (41 to 41)	336 (296 to 383)	180 (143 to 224)	48 (34 to 67)	0 (0 to 0)	108 (108 to 108)
Lesotho	2018	129 (112 to 147)	71 (56 to 89)	19 (13 to 26)	0 (0 to 0)	39 (39 to 39)	335 (292 to 383)	184 (146 to 231)	49 (35 to 69)	0 (0 to 0)	101 (100 to 102)
Lesotho	2019	132 (116 to 152)	73 (58 to 90)	19 (14 to 27)	0 (0 to 0)	40 (40 to 40)	344 (301 to 395)	189 (150 to 235)	50 (36 to 70)	0 (0 to 0)	104 (103 to 104)
Lesotho	2020	134 (118 to 153)	73 (58 to 91)	19 (14 to 27)	0 (0 to 0)	42 (40 to 44)	350 (307 to 398)	191 (151 to 238)	50 (36 to 70)	0 (0 to 0)	109 (104 to 115)
Lesotho	2021	138 (121 to 159)	75 (60 to 94)	19 (14 to 27)	0 (0 to 0)	43 (40 to 47)	360 (315 to 415)	197 (156 to 246)	51 (36 to 70)	0 (0 to 0)	113 (105 to 122)
Lesotho	2022	142 (125 to 163)	78 (62 to 98)	20 (14 to 27)	0 (0 to 0)	45 (41 to 49)	370 (325 to 424)	203 (161 to 254)	51 (36 to 71)	0 (0 to 0)	116 (106 to 128)
Lesotho	2023	146 (126 to 167)	80 (64 to 101)	20 (14 to 28)	0 (0 to 0)	45 (41 to 51)	379 (329 to 435)	209 (167 to 262)	52 (37 to 72)	0 (0 to 0)	118 (107 to 132)
Lesotho	2024	149 (129 to 172)	83 (66 to 104)	20 (14 to 28)	0 (0 to 0)	46 (41 to 52)	388 (337 to 448)	216 (171 to 270)	52 (37 to 73)	0 (0 to 0)	120 (107 to 135)

Lesotho	2025	152 (132 to 177)	86 (68 to 106)	20 (14 to 28)	0 (0 to 0)	46 (41 to 53)	397 (343 to 461)	223 (177 to 277)	53 (38 to 74)	0 (0 to 0)	121 (108 to 138)
Lesotho	2026	156 (136 to 183)	88 (70 to 110)	20 (15 to 29)	0 (0 to 0)	47 (42 to 54)	406 (353 to 476)	230 (182 to 286)	53 (38 to 74)	0 (0 to 0)	123 (108 to 140)
Lesotho	2027	160 (138 to 185)	91 (72 to 114)	21 (15 to 29)	0 (0 to 0)	48 (42 to 55)	416 (359 to 483)	238 (188 to 296)	54 (38 to 75)	0 (0 to 0)	124 (109 to 144)
Lesotho	2028	164 (140 to 189)	94 (74 to 117)	21 (15 to 29)	0 (0 to 0)	48 (42 to 56)	426 (366 to 494)	246 (194 to 306)	55 (39 to 76)	0 (0 to 0)	126 (110 to 147)
Lesotho	2029	168 (143 to 196)	97 (77 to 122)	21 (15 to 30)	0 (0 to 0)	49 (43 to 57)	437 (374 to 511)	254 (200 to 317)	55 (39 to 77)	0 (0 to 0)	128 (111 to 150)
Lesotho	2030	172 (148 to 199)	101 (79 to 126)	21 (15 to 30)	0 (0 to 0)	50 (43 to 59)	448 (387 to 519)	262 (206 to 328)	56 (40 to 78)	0 (0 to 0)	130 (111 to 154)
Lesotho	2031	176 (150 to 206)	104 (81 to 130)	22 (15 to 30)	0 (0 to 0)	50 (43 to 60)	459 (390 to 536)	271 (211 to 339)	57 (40 to 78)	0 (0 to 0)	132 (112 to 156)
Lesotho	2032	180 (154 to 210)	107 (83 to 135)	22 (16 to 30)	0 (0 to 0)	51 (44 to 62)	470 (401 to 547)	279 (217 to 351)	57 (41 to 79)	0 (0 to 0)	133 (114 to 160)
Lesotho	2033	185 (158 to 216)	110 (86 to 139)	22 (16 to 31)	0 (0 to 0)	52 (44 to 63)	482 (411 to 564)	288 (224 to 361)	58 (41 to 81)	0 (0 to 0)	136 (116 to 164)
Lesotho	2034	189 (160 to 221)	114 (88 to 143)	23 (16 to 31)	0 (0 to 0)	53 (44 to 64)	493 (418 to 577)	297 (230 to 373)	59 (42 to 82)	0 (0 to 0)	138 (115 to 168)
Lesotho	2035	194 (164 to 228)	117 (90 to 148)	23 (16 to 32)	0 (0 to 0)	54 (44 to 65)	505 (427 to 595)	306 (235 to 385)	60 (42 to 83)	0 (0 to 0)	140 (116 to 170)
Lesotho	2036	198 (168 to 233)	121 (93 to 152)	23 (17 to 32)	0 (0 to 0)	54 (45 to 68)	517 (437 to 608)	315 (242 to 397)	60 (43 to 84)	0 (0 to 0)	142 (117 to 178)
Lesotho	2037	203 (170 to 238)	124 (95 to 157)	23 (17 to 33)	0 (0 to 0)	55 (45 to 67)	529 (443 to 621)	324 (248 to 410)	61 (44 to 85)	0 (0 to 0)	144 (116 to 176)
Lesotho	2038	208 (176 to 247)	128 (97 to 162)	24 (17 to 33)	0 (0 to 0)	56 (45 to 71)	542 (458 to 644)	333 (254 to 423)	62 (44 to 87)	0 (0 to 0)	146 (118 to 184)
Lesotho	2039	212 (178 to 252)	131 (100 to 167)	24 (17 to 34)	0 (0 to 0)	57 (45 to 72)	553 (463 to 658)	342 (260 to 435)	63 (45 to 88)	0 (0 to 0)	148 (119 to 187)

Lesotho	2040	217 (180 to 259)	135 (102 to 172)	25 (17 to 34)	0 (0 to 0)	58 (46 to 74)	566 (469 to 676)	352 (267 to 447)	64 (46 to 89)	0 (0 to 0)	150 (120 to 193)
Lesotho	2041	222 (185 to 260)	138 (105 to 176)	25 (18 to 35)	0 (0 to 0)	59 (47 to 75)	579 (483 to 678)	361 (273 to 459)	65 (46 to 91)	0 (0 to 0)	153 (121 to 195)
Lesotho	2042	227 (190 to 268)	142 (107 to 181)	25 (18 to 35)	0 (0 to 0)	59 (47 to 76)	591 (494 to 698)	370 (279 to 471)	66 (47 to 92)	0 (0 to 0)	155 (121 to 198)
Lesotho	2043	231 (192 to 274)	145 (110 to 185)	26 (18 to 36)	0 (0 to 0)	60 (47 to 79)	603 (500 to 714)	379 (286 to 483)	67 (48 to 94)	0 (0 to 0)	157 (122 to 206)
Lesotho	2044	236 (196 to 282)	149 (112 to 190)	26 (19 to 37)	0 (0 to 0)	61 (48 to 80)	616 (511 to 735)	388 (292 to 495)	68 (48 to 95)	0 (0 to 0)	160 (125 to 209)
Lesotho	2045	241 (202 to 287)	152 (114 to 195)	26 (19 to 37)	0 (0 to 0)	62 (47 to 82)	628 (527 to 747)	396 (298 to 507)	69 (49 to 97)	0 (0 to 0)	163 (124 to 214)
Lesotho	2046	246 (204 to 293)	155 (117 to 199)	27 (19 to 38)	0 (0 to 0)	63 (49 to 83)	640 (533 to 765)	405 (304 to 518)	70 (50 to 98)	0 (0 to 0)	165 (127 to 217)
Lesotho	2047	250 (210 to 299)	158 (119 to 203)	27 (19 to 38)	0 (0 to 0)	65 (49 to 87)	653 (547 to 779)	413 (310 to 528)	71 (51 to 100)	0 (0 to 0)	169 (128 to 227)
Lesotho	2048	255 (212 to 306)	161 (121 to 206)	28 (20 to 39)	0 (0 to 0)	66 (50 to 88)	664 (552 to 797)	421 (315 to 538)	72 (51 to 102)	0 (0 to 0)	171 (130 to 230)
Lesotho	2049	259 (216 to 312)	164 (123 to 210)	28 (20 to 40)	0 (0 to 0)	67 (51 to 90)	676 (562 to 814)	428 (320 to 547)	73 (52 to 103)	0 (0 to 0)	174 (132 to 235)
Lesotho	2050	264 (222 to 318)	167 (125 to 214)	28 (20 to 40)	0 (0 to 0)	68 (52 to 92)	688 (577 to 828)	436 (326 to 557)	74 (52 to 105)	0 (0 to 0)	178 (137 to 240)
Liberia	1995	11 (9 to 15)	2 (1 to 2)	9 (6 to 12)	0 (0 to 1)	0 (0 to 0)	23 (17 to 30)	4 (3 to 5)	17 (12 to 24)	1 (0 to 1)	1 (1 to 1)
Liberia	1996	11 (9 to 14)	2 (1 to 2)	8 (6 to 11)	0 (0 to 1)	1 (1 to 1)	22 (17 to 28)	4 (3 to 5)	16 (11 to 22)	1 (0 to 1)	1 (1 to 1)
Liberia	1997	13 (11 to 17)	2 (2 to 3)	9 (6 to 12)	0 (0 to 1)	2 (2 to 2)	27 (22 to 34)	5 (4 to 6)	18 (12 to 24)	1 (0 to 1)	4 (4 to 4)
Liberia	1998	14 (12 to 18)	2 (2 to 3)	9 (6 to 12)	0 (0 to 1)	3 (3 to 3)	29 (23 to 36)	5 (4 to 6)	18 (12 to 24)	1 (0 to 1)	6 (6 to 6)

Liberia	1999	14 (12 to 17)	2 (2 to 3)	9 (6 to 12)	0 (0 to 1)	3 (3 to 3)	29 (23 to 35)	5 (4 to 7)	17 (12 to 24)	1 (0 to 1)	6 (6 to 6)
Liberia	2000	15 (12 to 18)	2 (2 to 3)	8 (6 to 11)	0 (0 to 1)	4 (4 to 4)	30 (25 to 36)	5 (4 to 6)	16 (11 to 22)	1 (0 to 1)	8 (8 to 8)
Liberia	2001	13 (10 to 16)	2 (2 to 3)	8 (5 to 10)	0 (0 to 1)	2 (2 to 2)	25 (21 to 31)	4 (3 to 6)	15 (11 to 21)	1 (0 to 1)	5 (5 to 5)
Liberia	2002	13 (11 to 17)	2 (2 to 3)	9 (6 to 12)	0 (0 to 1)	2 (2 to 2)	27 (22 to 34)	5 (3 to 6)	17 (12 to 24)	1 (0 to 1)	4 (4 to 4)
Liberia	2003	13 (10 to 16)	1 (1 to 2)	8 (5 to 11)	0 (0 to 0)	3 (3 to 3)	25 (20 to 32)	3 (2 to 4)	15 (11 to 22)	1 (0 to 1)	7 (7 to 7)
Liberia	2004	19 (15 to 23)	2 (1 to 2)	11 (8 to 16)	0 (0 to 1)	5 (5 to 5)	38 (31 to 47)	4 (3 to 5)	23 (15 to 32)	1 (0 to 1)	11 (11 to 11)
Liberia	2005	21 (17 to 26)	2 (1 to 2)	13 (9 to 18)	0 (0 to 1)	6 (6 to 6)	42 (34 to 53)	4 (3 to 5)	26 (18 to 36)	1 (0 to 1)	12 (12 to 12)
Liberia	2006	23 (18 to 28)	2 (1 to 3)	13 (9 to 19)	0 (0 to 1)	7 (7 to 7)	45 (37 to 56)	4 (3 to 5)	27 (18 to 38)	1 (0 to 1)	14 (14 to 14)
Liberia	2007	26 (21 to 33)	3 (2 to 4)	16 (11 to 23)	1 (0 to 1)	7 (7 to 7)	52 (42 to 66)	5 (4 to 7)	32 (22 to 45)	1 (1 to 2)	14 (14 to 14)
Liberia	2008	38 (32 to 45)	3 (3 to 5)	17 (12 to 24)	1 (0 to 1)	17 (17 to 17)	76 (65 to 89)	7 (5 to 9)	34 (24 to 47)	1 (1 to 3)	33 (33 to 33)
Liberia	2009	46 (41 to 53)	4 (3 to 5)	17 (12 to 24)	1 (0 to 2)	24 (24 to 24)	92 (81 to 106)	7 (5 to 9)	35 (24 to 49)	2 (1 to 3)	48 (48 to 48)
Liberia	2010	45 (40 to 52)	4 (3 to 5)	18 (12 to 25)	1 (0 to 2)	23 (23 to 23)	91 (80 to 105)	7 (5 to 9)	35 (25 to 50)	2 (1 to 4)	46 (46 to 46)
Liberia	2011	48 (42 to 55)	4 (3 to 5)	19 (13 to 26)	1 (1 to 2)	24 (24 to 24)	96 (85 to 111)	8 (6 to 11)	38 (27 to 53)	3 (1 to 5)	47 (47 to 47)
Liberia	2012	52 (47 to 59)	4 (3 to 5)	19 (14 to 26)	1 (1 to 3)	28 (28 to 28)	105 (94 to 119)	8 (6 to 10)	38 (27 to 53)	3 (1 to 5)	56 (56 to 56)
Liberia	2013	50 (44 to 58)	4 (3 to 5)	21 (15 to 28)	2 (1 to 3)	24 (24 to 24)	101 (89 to 116)	8 (6 to 10)	41 (30 to 56)	4 (2 to 7)	48 (48 to 48)

Liberia	2014	92 (85 to 100)	4 (3 to 6)	23 (17 to 31)	2 (1 to 4)	62 (62 to 62)	185 (172 to 202)	9 (7 to 12)	46 (33 to 63)	5 (2 to 8)	125 (125 to 125)
Liberia	2015	139 (131 to 148)	6 (4 to 8)	25 (18 to 35)	3 (2 to 6)	104 (104 to 104)	278 (264 to 297)	11 (9 to 15)	51 (37 to 70)	7 (3 to 12)	209 (209 to 209)
Liberia	2016	66 (58 to 76)	7 (5 to 9)	26 (19 to 36)	4 (2 to 7)	29 (29 to 29)	132 (116 to 152)	13 (10 to 17)	53 (38 to 72)	8 (4 to 15)	58 (58 to 58)
Liberia	2017	65 (56 to 75)	7 (5 to 10)	26 (19 to 36)	4 (2 to 8)	27 (27 to 27)	130 (112 to 151)	15 (11 to 19)	53 (38 to 72)	9 (4 to 16)	53 (53 to 53)
Liberia	2018	63 (54 to 74)	8 (6 to 10)	26 (19 to 36)	4 (2 to 8)	25 (25 to 25)	127 (108 to 148)	15 (11 to 20)	53 (38 to 72)	9 (4 to 16)	50 (50 to 51)
Liberia	2019	65 (56 to 76)	8 (6 to 11)	26 (19 to 35)	4 (2 to 8)	26 (26 to 26)	130 (112 to 152)	16 (12 to 22)	52 (37 to 71)	9 (4 to 17)	53 (53 to 53)
Liberia	2020	66 (56 to 76)	8 (6 to 11)	25 (18 to 35)	4 (2 to 8)	27 (26 to 29)	132 (112 to 154)	16 (12 to 22)	51 (37 to 70)	9 (5 to 17)	55 (52 to 58)
Liberia	2021	66 (57 to 78)	8 (6 to 11)	26 (19 to 35)	5 (2 to 8)	28 (26 to 30)	133 (115 to 157)	17 (12 to 23)	52 (37 to 70)	9 (5 to 17)	56 (53 to 61)
Liberia	2022	67 (58 to 80)	8 (6 to 12)	26 (19 to 35)	5 (2 to 9)	28 (26 to 31)	135 (116 to 160)	17 (12 to 23)	52 (38 to 71)	9 (5 to 17)	57 (52 to 63)
Liberia	2023	68 (58 to 80)	9 (6 to 12)	26 (19 to 36)	5 (2 to 9)	28 (26 to 32)	137 (116 to 161)	17 (12 to 24)	53 (38 to 72)	9 (5 to 18)	57 (52 to 64)
Liberia	2024	69 (59 to 81)	9 (6 to 13)	27 (19 to 36)	5 (2 to 9)	29 (26 to 32)	138 (117 to 163)	18 (12 to 25)	53 (39 to 72)	10 (5 to 18)	57 (51 to 65)
Liberia	2025	69 (59 to 83)	9 (6 to 13)	27 (19 to 37)	5 (2 to 9)	29 (26 to 33)	139 (118 to 166)	18 (12 to 25)	54 (39 to 73)	10 (5 to 18)	58 (52 to 66)
Liberia	2026	70 (59 to 83)	9 (6 to 13)	27 (20 to 37)	5 (2 to 9)	29 (26 to 33)	141 (119 to 167)	18 (13 to 26)	54 (40 to 74)	10 (5 to 19)	58 (51 to 67)
Liberia	2027	71 (60 to 85)	9 (6 to 13)	27 (20 to 37)	5 (3 to 9)	29 (25 to 34)	143 (121 to 171)	19 (13 to 27)	55 (40 to 75)	10 (5 to 19)	58 (51 to 68)
Liberia	2028	72 (60 to 86)	10 (6 to 14)	28 (20 to 38)	5 (3 to 10)	29 (26 to 34)	144 (121 to 172)	19 (13 to 28)	56 (41 to 76)	10 (5 to 19)	59 (51 to 69)

Liberia	2029	73 (61 to 87)	10 (6 to 14)	28 (20 to 38)	5 (3 to 10)	30 (26 to 35)	146 (123 to 174)	19 (13 to 29)	57 (41 to 77)	11 (5 to 20)	59 (52 to 70)
Liberia	2030	74 (62 to 88)	10 (6 to 15)	28 (21 to 39)	5 (3 to 10)	30 (26 to 36)	148 (124 to 177)	20 (13 to 29)	57 (41 to 78)	11 (5 to 20)	60 (51 to 72)
Liberia	2031	75 (62 to 90)	10 (6 to 15)	29 (21 to 39)	5 (3 to 10)	30 (26 to 36)	150 (124 to 180)	20 (13 to 30)	58 (42 to 79)	11 (5 to 20)	61 (52 to 73)
Liberia	2032	76 (63 to 90)	10 (6 to 16)	29 (21 to 40)	6 (3 to 10)	31 (26 to 37)	152 (126 to 181)	21 (13 to 32)	58 (42 to 79)	11 (6 to 21)	61 (52 to 75)
Liberia	2033	77 (64 to 93)	11 (6 to 16)	29 (21 to 40)	6 (3 to 11)	31 (26 to 38)	154 (128 to 186)	21 (13 to 33)	59 (43 to 80)	11 (6 to 21)	62 (53 to 76)
Liberia	2034	78 (65 to 93)	11 (6 to 16)	30 (22 to 40)	6 (3 to 11)	31 (26 to 38)	156 (130 to 187)	21 (13 to 33)	60 (43 to 81)	12 (6 to 22)	63 (53 to 77)
Liberia	2035	79 (65 to 96)	11 (7 to 17)	30 (22 to 41)	6 (3 to 11)	32 (27 to 39)	158 (131 to 192)	22 (13 to 34)	60 (44 to 82)	12 (6 to 22)	64 (54 to 78)
Liberia	2036	80 (66 to 96)	11 (6 to 18)	30 (22 to 41)	6 (3 to 11)	32 (27 to 41)	160 (132 to 194)	22 (13 to 35)	61 (44 to 83)	12 (6 to 22)	65 (54 to 82)
Liberia	2037	81 (66 to 99)	11 (6 to 18)	31 (22 to 42)	6 (3 to 11)	33 (27 to 40)	163 (133 to 199)	23 (13 to 36)	61 (44 to 84)	12 (6 to 23)	66 (55 to 81)
Liberia	2038	82 (68 to 99)	12 (7 to 18)	31 (22 to 42)	6 (3 to 11)	34 (28 to 42)	165 (136 to 198)	23 (13 to 37)	62 (45 to 85)	12 (6 to 23)	67 (55 to 84)
Liberia	2039	83 (68 to 102)	12 (7 to 19)	31 (22 to 43)	6 (3 to 12)	34 (28 to 43)	167 (136 to 205)	23 (13 to 38)	63 (45 to 86)	13 (6 to 23)	68 (56 to 87)
Liberia	2040	85 (69 to 102)	12 (7 to 19)	31 (23 to 43)	6 (3 to 12)	35 (28 to 44)	170 (139 to 205)	24 (13 to 39)	63 (45 to 87)	13 (6 to 24)	70 (57 to 88)
Liberia	2041	86 (70 to 105)	12 (7 to 20)	32 (23 to 44)	6 (3 to 12)	35 (29 to 45)	172 (140 to 212)	24 (13 to 40)	64 (46 to 87)	13 (6 to 24)	71 (58 to 90)
Liberia	2042	87 (71 to 107)	12 (7 to 20)	32 (23 to 44)	7 (3 to 12)	36 (29 to 46)	175 (142 to 214)	25 (13 to 41)	64 (46 to 88)	13 (7 to 25)	72 (59 to 93)
Liberia	2043	88 (72 to 109)	13 (7 to 21)	32 (23 to 44)	7 (3 to 12)	37 (29 to 48)	177 (145 to 220)	25 (14 to 42)	65 (47 to 89)	13 (7 to 25)	74 (59 to 96)

Liberia	2044	90 (72 to 110)	13 (7 to 21)	33 (24 to 45)	7 (3 to 13)	37 (30 to 49)	180 (146 to 220)	26 (14 to 43)	66 (47 to 90)	14 (7 to 25)	75 (60 to 99)
Liberia	2045	91 (74 to 113)	13 (7 to 22)	33 (24 to 45)	7 (3 to 13)	38 (30 to 49)	182 (149 to 226)	26 (14 to 44)	66 (48 to 91)	14 (7 to 26)	76 (61 to 99)
Liberia	2046	92 (74 to 114)	13 (7 to 23)	33 (24 to 46)	7 (3 to 13)	39 (30 to 50)	185 (149 to 228)	27 (14 to 46)	67 (48 to 92)	14 (7 to 26)	78 (61 to 101)
Liberia	2047	94 (76 to 117)	13 (7 to 23)	34 (24 to 46)	7 (4 to 13)	40 (31 to 52)	188 (153 to 234)	27 (14 to 47)	67 (49 to 93)	14 (7 to 27)	79 (62 to 105)
Liberia	2048	95 (76 to 119)	14 (7 to 24)	34 (24 to 47)	7 (4 to 13)	40 (32 to 54)	191 (153 to 238)	27 (14 to 48)	68 (49 to 94)	15 (7 to 27)	81 (64 to 108)
Liberia	2049	97 (77 to 122)	14 (7 to 24)	34 (25 to 47)	7 (4 to 14)	41 (32 to 55)	194 (156 to 244)	28 (14 to 49)	69 (49 to 95)	15 (7 to 27)	83 (65 to 110)
Liberia	2050	98 (79 to 121)	14 (7 to 26)	34 (25 to 48)	7 (4 to 14)	42 (33 to 57)	197 (158 to 243)	28 (14 to 51)	69 (50 to 96)	15 (7 to 28)	85 (66 to 114)
Libya	1995	656 (588 to 725)	289 (245 to 338)	347 (306 to 394)	21 (9 to 40)	0 (0 to 0)	1157 (1037 to 1278)	509 (432 to 596)	611 (540 to 695)	37 (17 to 71)	0 (0 to 0)
Libya	1996	693 (630 to 756)	315 (271 to 362)	355 (320 to 395)	22 (10 to 42)	0 (0 to 0)	1221 (1111 to 1332)	556 (478 to 639)	627 (564 to 696)	39 (18 to 74)	0 (0 to 0)
Libya	1997	741 (681 to 806)	345 (300 to 393)	372 (337 to 408)	24 (11 to 46)	0 (0 to 0)	1305 (1201 to 1421)	608 (529 to 692)	655 (593 to 720)	42 (19 to 81)	0 (0 to 0)
Libya	1998	754 (695 to 817)	348 (306 to 393)	383 (349 to 420)	23 (11 to 46)	0 (0 to 0)	1329 (1224 to 1440)	614 (539 to 693)	674 (615 to 740)	41 (20 to 81)	0 (0 to 0)
Libya	1999	704 (653 to 761)	323 (287 to 363)	360 (329 to 394)	21 (10 to 42)	0 (0 to 0)	1240 (1150 to 1341)	569 (506 to 639)	634 (580 to 694)	37 (18 to 74)	0 (0 to 0)

Libya	2000	706 (654 to 762)	346 (308 to 386)	338 (308 to 373)	22 (11 to 41)	0 (0 to 0)	1244 (1153 to 1343)	610 (544 to 681)	596 (543 to 658)	38 (19 to 72)	0 (0 to 0)
Libya	2001	808 (749 to 871)	443 (398 to 491)	338 (307 to 372)	26 (13 to 49)	0 (0 to 0)	1424 (1321 to 1535)	782 (702 to 866)	596 (541 to 655)	47 (23 to 86)	0 (0 to 0)
Libya	2002	829 (771 to 898)	494 (446 to 547)	307 (277 to 341)	28 (13 to 51)	0 (0 to 0)	1462 (1359 to 1583)	871 (786 to 964)	542 (487 to 600)	49 (24 to 89)	0 (0 to 0)
Libya	2003	834 (774 to 902)	521 (472 to 575)	283 (254 to 318)	30 (14 to 55)	0 (0 to 0)	1470 (1364 to 1590)	919 (832 to 1013)	499 (448 to 560)	52 (25 to 96)	0 (0 to 0)
Libya	2004	773 (713 to 837)	491 (446 to 545)	252 (225 to 284)	29 (14 to 53)	0 (0 to 0)	1362 (1257 to 1475)	865 (787 to 960)	445 (397 to 501)	51 (24 to 93)	0 (0 to 0)
Libya	2005	619 (568 to 673)	385 (350 to 427)	210 (185 to 237)	24 (11 to 44)	0 (0 to 0)	1091 (1001 to 1187)	679 (618 to 753)	370 (326 to 418)	42 (20 to 77)	0 (0 to 0)
Libya	2006	616 (565 to 669)	389 (352 to 433)	203 (178 to 229)	25 (12 to 47)	0 (0 to 0)	1087 (996 to 1180)	685 (621 to 763)	357 (313 to 404)	44 (21 to 83)	0 (0 to 0)
Libya	2007	620 (572 to 670)	395 (357 to 436)	198 (174 to 224)	27 (13 to 51)	0 (0 to 0)	1092 (1008 to 1182)	696 (630 to 769)	349 (308 to 395)	47 (23 to 90)	1 (1 to 1)
Libya	2008	694 (642 to 753)	451 (408 to 499)	205 (181 to 233)	32 (16 to 61)	6 (6 to 6)	1224 (1131 to 1327)	794 (719 to 880)	362 (320 to 410)	57 (27 to 107)	11 (11 to 11)
Libya	2009	869 (801 to 942)	585 (531 to 647)	239 (211 to 271)	41 (20 to 76)	4 (4 to 4)	1533 (1412 to 1661)	1032 (936 to 1140)	421 (371 to 478)	73 (35 to 134)	6 (6 to 6)

Libya	2010	803 (741 to 869)	539 (490 to 596)	226 (198 to 257)	37 (18 to 70)	0 (0 to 0)	1415 (1306 to 1531)	951 (864 to 1050)	398 (350 to 452)	65 (32 to 123)	0 (0 to 0)
Libya	2011	483 (444 to 524)	300 (272 to 331)	162 (141 to 184)	19 (9 to 36)	2 (2 to 2)	850 (783 to 923)	528 (479 to 584)	285 (249 to 325)	34 (16 to 63)	4 (4 to 4)
Libya	2012	695 (634 to 760)	463 (416 to 518)	204 (176 to 235)	28 (13 to 52)	0 (0 to 0)	1225 (1117 to 1339)	817 (734 to 913)	359 (310 to 415)	49 (23 to 92)	1 (1 to 1)
Libya	2013	680 (614 to 754)	466 (413 to 527)	183 (155 to 215)	29 (14 to 54)	1 (1 to 1)	1198 (1081 to 1329)	822 (728 to 929)	323 (272 to 379)	52 (25 to 95)	1 (1 to 1)
Libya	2014	607 (538 to 681)	427 (368 to 490)	152 (124 to 181)	29 (14 to 52)	0 (0 to 0)	1070 (948 to 1200)	752 (648 to 864)	267 (219 to 320)	50 (25 to 92)	0 (0 to 0)
Libya	2015	546 (474 to 622)	381 (317 to 446)	139 (111 to 173)	25 (12 to 45)	0 (0 to 0)	962 (835 to 1096)	671 (559 to 787)	246 (195 to 305)	44 (22 to 80)	1 (1 to 1)
Libya	2016	506 (431 to 584)	352 (287 to 426)	131 (102 to 167)	23 (11 to 41)	1 (1 to 1)	893 (760 to 1029)	621 (505 to 752)	231 (179 to 295)	40 (19 to 72)	1 (1 to 1)
Libya	2017	466 (392 to 548)	321 (255 to 396)	124 (93 to 162)	20 (10 to 36)	1 (1 to 1)	821 (690 to 966)	566 (449 to 699)	218 (164 to 285)	35 (17 to 64)	2 (2 to 2)
Libya	2018	474 (398 to 563)	325 (253 to 406)	127 (95 to 166)	21 (10 to 39)	1 (1 to 1)	835 (701 to 992)	573 (445 to 716)	223 (168 to 292)	37 (18 to 68)	2 (2 to 2)
Libya	2019	479 (397 to 578)	352 (272 to 447)	105 (79 to 137)	21 (10 to 38)	1 (1 to 1)	844 (699 to 1018)	621 (479 to 788)	185 (138 to 241)	37 (18 to 67)	2 (2 to 2)
Libya	2020	459 (377 to 556)	335 (252 to 434)	103 (77 to 135)	20 (10 to 37)	1 (1 to 1)	810 (665 to 980)	590 (445 to 765)	182 (137 to 238)	35 (17 to 64)	2 (2 to 2)
Libya	2021	434 (358 to 531)	313 (236 to 408)	101 (75 to 131)	19 (9 to 35)	1 (1 to 1)	764 (631 to 936)	551 (417 to 720)	178 (133 to 231)	33 (17 to 61)	2 (2 to 2)
Libya	2022	414 (339 to 509)	296 (221 to 392)	99 (74 to 129)	18 (9 to 34)	1 (1 to 1)	730 (598 to 896)	522 (390 to 691)	174 (131 to 227)	32 (16 to 59)	2 (2 to 3)

Libya	2023	416 (342 to 516)	298 (222 to 395)	98 (73 to 127)	18 (9 to 34)	1 (1 to 2)	733 (603 to 909)	525 (392 to 697)	173 (129 to 225)	32 (16 to 59)	2 (2 to 3)
Libya	2024	420 (338 to 522)	303 (221 to 407)	97 (73 to 126)	19 (9 to 34)	1 (1 to 2)	740 (596 to 919)	533 (390 to 718)	172 (129 to 223)	33 (16 to 60)	2 (2 to 3)
Libya	2025	423 (342 to 530)	307 (220 to 410)	97 (72 to 125)	19 (9 to 34)	1 (1 to 2)	746 (603 to 934)	540 (387 to 723)	170 (128 to 221)	33 (16 to 60)	2 (2 to 3)
Libya	2026	426 (343 to 529)	310 (222 to 424)	96 (72 to 124)	19 (9 to 34)	1 (1 to 2)	750 (605 to 932)	546 (391 to 747)	169 (127 to 219)	33 (16 to 61)	2 (2 to 3)
Libya	2027	428 (346 to 541)	313 (223 to 427)	95 (71 to 124)	19 (9 to 35)	1 (1 to 2)	755 (610 to 953)	551 (392 to 752)	168 (126 to 218)	33 (16 to 61)	3 (2 to 3)
Libya	2028	430 (349 to 541)	315 (227 to 436)	95 (71 to 123)	19 (9 to 35)	1 (1 to 2)	759 (615 to 954)	556 (399 to 768)	167 (125 to 217)	33 (16 to 61)	3 (2 to 3)
Libya	2029	433 (349 to 544)	319 (228 to 434)	94 (71 to 123)	19 (9 to 35)	1 (1 to 2)	764 (615 to 959)	562 (402 to 764)	166 (124 to 216)	34 (16 to 62)	3 (2 to 3)
Libya	2030	436 (348 to 546)	322 (231 to 441)	93 (70 to 122)	19 (9 to 35)	2 (1 to 2)	768 (614 to 963)	567 (407 to 778)	165 (123 to 214)	34 (16 to 62)	3 (2 to 3)
Libya	2031	438 (350 to 558)	324 (232 to 451)	93 (69 to 121)	19 (9 to 35)	2 (1 to 2)	772 (617 to 984)	571 (410 to 795)	164 (122 to 213)	34 (16 to 62)	3 (2 to 3)
Libya	2032	440 (349 to 562)	327 (230 to 457)	92 (69 to 120)	19 (9 to 36)	2 (1 to 2)	775 (615 to 991)	576 (405 to 806)	162 (121 to 212)	34 (16 to 63)	3 (2 to 3)
Libya	2033	441 (348 to 567)	329 (230 to 460)	91 (68 to 119)	19 (9 to 36)	2 (1 to 2)	777 (614 to 1000)	579 (405 to 810)	161 (120 to 210)	34 (16 to 63)	3 (2 to 3)
Libya	2034	441 (349 to 568)	329 (229 to 467)	91 (67 to 118)	19 (9 to 36)	2 (1 to 2)	777 (614 to 1002)	581 (404 to 823)	160 (119 to 208)	34 (16 to 63)	3 (2 to 4)
Libya	2035	441 (348 to 570)	330 (229 to 468)	90 (67 to 117)	19 (9 to 36)	2 (1 to 2)	777 (614 to 1005)	582 (403 to 825)	158 (117 to 206)	34 (16 to 63)	3 (2 to 4)
Libya	2036	440 (351 to 579)	331 (228 to 471)	89 (66 to 116)	19 (9 to 35)	2 (1 to 2)	776 (618 to 1020)	583 (402 to 830)	156 (116 to 204)	34 (16 to 62)	3 (3 to 4)
Libya	2037	438 (344 to 575)	330 (227 to 474)	87 (65 to 115)	19 (9 to 35)	2 (1 to 2)	773 (606 to 1013)	582 (400 to 835)	154 (114 to 202)	34 (16 to 62)	3 (3 to 4)

Libya	2038	437 (341 to 582)	330 (226 to 471)	86 (64 to 113)	19 (9 to 35)	2 (2 to 2)	770 (601 to 1026)	582 (398 to 830)	152 (112 to 199)	34 (16 to 62)	3 (3 to 4)
Libya	2039	436 (341 to 576)	330 (224 to 474)	85 (63 to 112)	19 (9 to 35)	2 (2 to 2)	769 (601 to 1016)	582 (395 to 836)	150 (111 to 197)	33 (16 to 61)	3 (3 to 4)
Libya	2040	436 (336 to 578)	331 (224 to 480)	84 (62 to 111)	19 (9 to 35)	2 (2 to 2)	768 (592 to 1019)	583 (394 to 847)	148 (109 to 195)	33 (16 to 61)	3 (3 to 4)
Libya	2041	434 (333 to 581)	330 (222 to 487)	83 (61 to 110)	19 (9 to 35)	2 (2 to 2)	765 (586 to 1024)	582 (392 to 858)	147 (108 to 193)	33 (16 to 61)	3 (3 to 4)
Libya	2042	433 (330 to 583)	330 (220 to 480)	82 (60 to 108)	19 (9 to 35)	2 (2 to 3)	763 (581 to 1027)	582 (388 to 846)	145 (106 to 191)	33 (16 to 61)	4 (3 to 4)
Libya	2043	432 (331 to 583)	330 (219 to 483)	81 (59 to 107)	19 (9 to 35)	2 (2 to 3)	761 (584 to 1028)	581 (386 to 851)	143 (105 to 189)	33 (16 to 61)	4 (3 to 5)
Libya	2044	431 (327 to 582)	330 (222 to 481)	80 (59 to 106)	19 (9 to 34)	2 (2 to 3)	760 (577 to 1025)	582 (392 to 848)	141 (103 to 187)	33 (16 to 61)	4 (3 to 5)
Libya	2045	430 (330 to 590)	330 (221 to 493)	79 (58 to 105)	19 (9 to 34)	2 (2 to 3)	759 (581 to 1041)	582 (390 to 869)	140 (102 to 185)	33 (16 to 61)	4 (3 to 5)
Libya	2046	428 (321 to 580)	330 (218 to 493)	78 (57 to 104)	19 (9 to 34)	2 (2 to 3)	755 (566 to 1022)	581 (384 to 869)	138 (101 to 183)	33 (16 to 60)	4 (3 to 5)
Libya	2047	427 (319 to 586)	329 (218 to 488)	77 (57 to 103)	18 (9 to 34)	2 (2 to 3)	753 (562 to 1032)	581 (384 to 861)	136 (100 to 181)	33 (16 to 60)	4 (3 to 5)
Libya	2048	425 (318 to 583)	328 (216 to 494)	76 (56 to 101)	18 (9 to 34)	2 (2 to 3)	749 (560 to 1028)	579 (380 to 871)	134 (98 to 178)	32 (16 to 60)	4 (3 to 5)
Libya	2049	423 (313 to 583)	327 (215 to 496)	75 (55 to 100)	18 (9 to 34)	2 (2 to 3)	745 (552 to 1027)	577 (379 to 873)	132 (97 to 176)	32 (16 to 59)	4 (3 to 6)
Libya	2050	420 (306 to 578)	326 (212 to 489)	74 (54 to 99)	18 (9 to 33)	2 (2 to 3)	741 (540 to 1019)	575 (373 to 863)	130 (95 to 174)	32 (15 to 59)	4 (3 to 6)
Lithuania	1995	298 (269 to 330)	205 (180 to 231)	77 (63 to 92)	15 (8 to 25)	1 (1 to 1)	568 (512 to 629)	390 (344 to 441)	147 (120 to 176)	28 (15 to 49)	2 (2 to 2)
Lithuania	1996	315 (288 to 343)	216 (194 to 239)	84 (70 to 97)	15 (8 to 26)	0 (0 to 0)	600 (549 to 654)	412 (370 to 455)	159 (133 to 185)	29 (16 to 49)	0 (0 to 0)

Lithuania	1997	348 (323 to 375)	241 (221 to 264)	91 (78 to 103)	16 (9 to 27)	0 (0 to 0)	664 (616 to 715)	460 (422 to 504)	173 (148 to 197)	31 (17 to 51)	0 (0 to 0)
Lithuania	1998	399 (373 to 426)	281 (259 to 302)	101 (88 to 114)	18 (10 to 29)	0 (0 to 0)	761 (712 to 812)	535 (495 to 576)	192 (168 to 217)	34 (20 to 56)	0 (0 to 0)
Lithuania	1999	428 (401 to 456)	300 (278 to 322)	109 (96 to 123)	18 (11 to 30)	0 (0 to 0)	816 (764 to 870)	572 (530 to 614)	208 (183 to 235)	35 (21 to 58)	0 (0 to 0)
Lithuania	2000	448 (420 to 477)	312 (290 to 334)	117 (104 to 132)	18 (11 to 28)	0 (0 to 0)	853 (800 to 909)	594 (554 to 636)	224 (198 to 252)	34 (21 to 54)	1 (1 to 1)
Lithuania	2001	480 (452 to 509)	337 (314 to 360)	128 (114 to 144)	15 (9 to 24)	0 (0 to 0)	916 (861 to 971)	642 (599 to 687)	244 (218 to 275)	29 (18 to 46)	0 (0 to 0)
Lithuania	2002	514 (485 to 545)	359 (336 to 386)	139 (124 to 155)	14 (9 to 23)	1 (1 to 1)	981 (925 to 1038)	685 (640 to 736)	265 (237 to 296)	27 (17 to 43)	3 (3 to 3)
Lithuania	2003	558 (525 to 590)	384 (360 to 411)	156 (139 to 173)	16 (10 to 26)	2 (2 to 2)	1063 (1001 to 1124)	733 (686 to 783)	297 (265 to 329)	31 (19 to 49)	3 (3 to 3)
Lithuania	2004	593 (560 to 627)	391 (366 to 418)	177 (159 to 196)	23 (14 to 35)	2 (2 to 2)	1130 (1068 to 1196)	745 (697 to 796)	337 (302 to 374)	44 (27 to 66)	5 (5 to 5)
Lithuania	2005	641 (606 to 677)	414 (389 to 442)	197 (178 to 217)	29 (18 to 43)	1 (1 to 1)	1222 (1156 to 1291)	790 (741 to 843)	375 (340 to 413)	56 (35 to 83)	1 (1 to 1)
Lithuania	2006	703 (665 to 742)	456 (429 to 485)	215 (196 to 236)	31 (20 to 47)	1 (1 to 1)	1340 (1268 to 1415)	870 (818 to 925)	409 (373 to 450)	59 (37 to 89)	1 (1 to 1)
Lithuania	2007	789 (752 to 831)	529 (501 to 560)	232 (211 to 255)	29 (17 to 44)	0 (0 to 0)	1505 (1433 to 1584)	1009 (955 to 1067)	442 (403 to 486)	55 (33 to 83)	0 (0 to 0)
Lithuania	2008	875 (836 to 915)	605 (573 to 638)	248 (226 to 272)	20 (12 to 31)	1 (1 to 1)	1667 (1594 to 1744)	1154 (1092 to 1216)	472 (430 to 519)	39 (24 to 59)	3 (3 to 3)

Lithuania	2009	895 (857 to 935)	625 (594 to 659)	249 (229 to 271)	17 (10 to 25)	4 (4 to 4)	1706 (1634 to 1782)	1193 (1133 to 1256)	474 (437 to 517)	32 (20 to 48)	7 (7 to 7)
Lithuania	2010	880 (846 to 919)	613 (585 to 647)	249 (230 to 271)	15 (9 to 23)	3 (3 to 3)	1678 (1614 to 1753)	1170 (1116 to 1234)	475 (439 to 517)	28 (17 to 43)	5 (5 to 5)
Lithuania	2011	921 (884 to 960)	640 (612 to 672)	266 (246 to 288)	15 (9 to 23)	0 (0 to 0)	1756 (1685 to 1831)	1220 (1167 to 1282)	507 (470 to 549)	29 (18 to 44)	0 (0 to 0)
Lithuania	2012	889 (853 to 925)	599 (572 to 627)	276 (256 to 297)	14 (9 to 22)	0 (0 to 0)	1695 (1627 to 1764)	1142 (1091 to 1195)	526 (489 to 566)	28 (17 to 42)	0 (0 to 0)
Lithuania	2013	926 (888 to 961)	616 (589 to 643)	295 (276 to 316)	15 (10 to 24)	0 (0 to 0)	1766 (1693 to 1832)	1174 (1122 to 1227)	563 (525 to 602)	29 (19 to 45)	0 (0 to 0)
Lithuania	2014	975 (940 to 1010)	645 (619 to 673)	313 (293 to 333)	17 (11 to 26)	0 (0 to 0)	1859 (1791 to 1926)	1230 (1181 to 1284)	597 (558 to 634)	32 (20 to 49)	0 (0 to 0)
Lithuania	2015	1042 (1003 to 1078)	688 (661 to 716)	335 (315 to 356)	19 (11 to 29)	0 (0 to 0)	1987 (1913 to 2056)	1312 (1260 to 1365)	638 (600 to 678)	36 (22 to 56)	0 (0 to 0)
Lithuania	2016	1092 (1053 to 1133)	719 (688 to 751)	353 (330 to 376)	20 (12 to 32)	0 (0 to 0)	2082 (2007 to 2160)	1371 (1313 to 1432)	673 (629 to 717)	39 (24 to 61)	0 (0 to 0)
Lithuania	2017	1139 (1081 to 1201)	749 (709 to 794)	367 (339 to 401)	23 (13 to 35)	0 (0 to 0)	2171 (2062 to 2289)	1428 (1351 to 1513)	700 (646 to 764)	43 (25 to 67)	0 (0 to 0)
Lithuania	2018	1209 (1142 to 1279)	805 (758 to 856)	380 (351 to 414)	24 (14 to 37)	0 (0 to 0)	2305 (2177 to 2439)	1535 (1446 to 1632)	725 (669 to 790)	45 (26 to 70)	0 (0 to 0)

Lithuania	2019	1280 (1209 to 1353)	865 (812 to 921)	390 (359 to 424)	24 (14 to 38)	0 (0 to 0)	2440 (2305 to 2580)	1650 (1549 to 1756)	744 (685 to 808)	46 (27 to 72)	0 (0 to 0)
Lithuania	2020	1324 (1246 to 1400)	897 (838 to 959)	401 (370 to 436)	25 (15 to 39)	0 (0 to 0)	2524 (2375 to 2670)	1711 (1598 to 1829)	765 (705 to 832)	48 (28 to 75)	0 (0 to 0)
Lithuania	2021	1351 (1270 to 1436)	918 (854 to 984)	408 (376 to 443)	26 (15 to 40)	0 (0 to 0)	2577 (2421 to 2738)	1750 (1629 to 1875)	778 (717 to 845)	49 (29 to 77)	0 (0 to 0)
Lithuania	2022	1379 (1293 to 1466)	939 (874 to 1005)	414 (381 to 450)	26 (16 to 41)	0 (0 to 0)	2630 (2464 to 2795)	1791 (1667 to 1917)	789 (726 to 859)	50 (30 to 79)	0 (0 to 0)
Lithuania	2023	1405 (1323 to 1497)	959 (888 to 1030)	419 (386 to 456)	27 (16 to 42)	0 (0 to 0)	2679 (2523 to 2853)	1829 (1694 to 1964)	799 (736 to 869)	51 (30 to 80)	0 (0 to 0)
Lithuania	2024	1429 (1340 to 1520)	978 (907 to 1051)	424 (390 to 461)	27 (16 to 43)	0 (0 to 0)	2724 (2555 to 2898)	1865 (1728 to 2003)	808 (744 to 879)	52 (31 to 81)	0 (0 to 0)
Lithuania	2025	1450 (1362 to 1542)	995 (921 to 1067)	427 (393 to 465)	28 (16 to 43)	0 (0 to 0)	2764 (2598 to 2940)	1897 (1757 to 2035)	814 (749 to 886)	53 (31 to 82)	0 (0 to 0)
Lithuania	2026	1473 (1379 to 1567)	1014 (939 to 1089)	431 (397 to 470)	28 (16 to 44)	0 (0 to 0)	2808 (2628 to 2988)	1933 (1790 to 2076)	822 (757 to 895)	54 (31 to 83)	0 (0 to 0)
Lithuania	2027	1501 (1405 to 1605)	1036 (956 to 1117)	436 (402 to 475)	29 (17 to 45)	0 (0 to 0)	2862 (2678 to 3061)	1976 (1823 to 2130)	832 (766 to 905)	55 (32 to 85)	0 (0 to 0)
Lithuania	2028	1531 (1425 to 1641)	1059 (974 to 1145)	442 (407 to 481)	29 (17 to 45)	0 (0 to 0)	2919 (2717 to 3129)	2020 (1857 to 2184)	843 (777 to 918)	56 (33 to 86)	0 (0 to 0)

Lithuania	2029	1562 (1454 to 1676)	1084 (992 to 1174)	448 (412 to 488)	30 (18 to 46)	0 (0 to 0)	2979 (2771 to 3196)	2067 (1892 to 2239)	855 (786 to 931)	57 (33 to 88)	0 (0 to 0)
Lithuania	2030	1595 (1477 to 1713)	1110 (1015 to 1204)	455 (419 to 496)	30 (18 to 47)	0 (0 to 0)	3041 (2816 to 3267)	2116 (1936 to 2296)	868 (798 to 945)	58 (34 to 90)	0 (0 to 0)
Lithuania	2031	1630 (1512 to 1753)	1137 (1035 to 1236)	462 (425 to 503)	31 (18 to 48)	0 (0 to 0)	3107 (2883 to 3343)	2167 (1974 to 2356)	881 (811 to 960)	59 (35 to 92)	0 (0 to 0)
Lithuania	2032	1666 (1540 to 1798)	1165 (1062 to 1267)	469 (432 to 512)	32 (19 to 49)	0 (0 to 0)	3176 (2936 to 3428)	2221 (2024 to 2415)	895 (823 to 975)	61 (36 to 94)	0 (0 to 0)
Lithuania	2033	1703 (1575 to 1842)	1193 (1082 to 1305)	477 (438 to 520)	33 (19 to 50)	0 (0 to 0)	3247 (3004 to 3513)	2275 (2063 to 2489)	910 (835 to 992)	62 (37 to 96)	0 (0 to 0)
Lithuania	2034	1741 (1606 to 1883)	1222 (1107 to 1345)	485 (445 to 529)	33 (20 to 51)	0 (0 to 0)	3319 (3061 to 3591)	2331 (2110 to 2565)	925 (848 to 1008)	63 (37 to 98)	0 (0 to 0)
Lithuania	2035	1780 (1636 to 1937)	1252 (1130 to 1379)	493 (452 to 539)	34 (20 to 52)	0 (0 to 0)	3393 (3120 to 3692)	2388 (2155 to 2629)	940 (862 to 1028)	65 (38 to 100)	0 (0 to 0)
Lithuania	2036	1821 (1667 to 1979)	1284 (1155 to 1422)	502 (459 to 549)	35 (21 to 54)	0 (0 to 0)	3471 (3179 to 3773)	2448 (2203 to 2710)	957 (876 to 1047)	67 (39 to 102)	0 (0 to 0)
Lithuania	2037	1863 (1705 to 2025)	1317 (1181 to 1457)	511 (467 to 559)	36 (21 to 55)	0 (0 to 0)	3552 (3251 to 3860)	2510 (2252 to 2778)	974 (890 to 1066)	68 (40 to 105)	0 (0 to 0)
Lithuania	2038	1908 (1749 to 2082)	1351 (1207 to 1499)	520 (475 to 570)	37 (22 to 57)	0 (0 to 0)	3637 (3335 to 3970)	2575 (2302 to 2858)	992 (906 to 1087)	70 (41 to 108)	0 (0 to 0)

Lithuania	2039	1955 (1793 to 2135)	1387 (1235 to 1544)	530 (484 to 582)	38 (22 to 58)	0 (0 to 0)	3728 (3418 to 4071)	2644 (2354 to 2944)	1011 (923 to 1109)	72 (42 to 111)	0 (0 to 0)
Lithuania	2040	2003 (1825 to 2193)	1423 (1266 to 1585)	541 (493 to 594)	39 (23 to 60)	0 (0 to 0)	3819 (3480 to 4181)	2714 (2414 to 3021)	1031 (940 to 1132)	74 (44 to 115)	0 (0 to 0)
Lithuania	2041	2054 (1873 to 2254)	1462 (1299 to 1629)	552 (503 to 607)	40 (23 to 62)	0 (0 to 0)	3916 (3571 to 4297)	2787 (2477 to 3105)	1052 (958 to 1158)	76 (45 to 119)	0 (0 to 0)
Lithuania	2042	2105 (1904 to 2308)	1501 (1331 to 1679)	563 (512 to 620)	41 (24 to 64)	0 (0 to 0)	4014 (3631 to 4401)	2862 (2539 to 3201)	1074 (976 to 1182)	78 (46 to 122)	0 (0 to 0)
Lithuania	2043	2158 (1955 to 2362)	1541 (1358 to 1719)	574 (522 to 633)	42 (25 to 66)	0 (0 to 0)	4114 (3728 to 4503)	2938 (2588 to 3278)	1095 (995 to 1207)	81 (47 to 126)	0 (0 to 0)
Lithuania	2044	2211 (1997 to 2432)	1582 (1392 to 1777)	586 (532 to 647)	43 (26 to 68)	0 (0 to 0)	4216 (3807 to 4638)	3016 (2654 to 3389)	1118 (1014 to 1233)	83 (49 to 130)	0 (0 to 0)
Lithuania	2045	2264 (2036 to 2500)	1621 (1422 to 1824)	598 (542 to 661)	45 (26 to 70)	0 (0 to 0)	4317 (3881 to 4767)	3092 (2711 to 3479)	1140 (1033 to 1260)	85 (50 to 134)	0 (0 to 0)
Lithuania	2046	2317 (2080 to 2552)	1661 (1453 to 1872)	610 (552 to 674)	46 (27 to 73)	0 (0 to 0)	4418 (3967 to 4865)	3168 (2770 to 3569)	1163 (1052 to 1286)	88 (52 to 139)	0 (0 to 0)
Lithuania	2047	2370 (2134 to 2615)	1701 (1487 to 1918)	621 (561 to 688)	47 (28 to 75)	0 (0 to 0)	4518 (4068 to 4987)	3243 (2834 to 3657)	1184 (1070 to 1312)	90 (53 to 143)	0 (0 to 0)
Lithuania	2048	2422 (2167 to 2679)	1741 (1517 to 1956)	632 (571 to 702)	49 (28 to 77)	0 (0 to 0)	4617 (4132 to 5108)	3319 (2893 to 3729)	1206 (1088 to 1338)	93 (54 to 147)	0 (0 to 0)

Lithuania	2049	2474 (2229 to 2732)	1780 (1548 to 2000)	644 (580 to 716)	50 (29 to 79)	0 (0 to 0)	4716 (4250 to 5210)	3394 (2952 to 3814)	1227 (1107 to 1364)	95 (56 to 151)	0 (0 to 0)
Lithuania	2050	2522 (2256 to 2791)	1817 (1573 to 2045)	654 (589 to 729)	51 (30 to 82)	0 (0 to 0)	4809 (4301 to 5321)	3465 (2998 to 3898)	1247 (1123 to 1389)	97 (57 to 156)	0 (0 to 0)
Luxembourg	1995	3181 (2903 to 3461)	2837 (2574 to 3118)	247 (195 to 306)	98 (56 to 162)	0 (0 to 0)	3109 (2836 to 3382)	2772 (2515 to 3046)	241 (191 to 299)	96 (55 to 159)	0 (0 to 0)
Luxembourg	1996	3324 (3083 to 3555)	2944 (2723 to 3170)	277 (225 to 336)	103 (63 to 161)	0 (0 to 0)	3248 (3012 to 3474)	2877 (2661 to 3097)	271 (220 to 328)	100 (61 to 157)	0 (0 to 0)
Luxembourg	1997	3593 (3376 to 3806)	3164 (2960 to 3365)	320 (269 to 375)	109 (71 to 162)	0 (0 to 0)	3510 (3299 to 3719)	3091 (2892 to 3288)	312 (262 to 366)	107 (69 to 158)	0 (0 to 0)
Luxembourg	1998	3990 (3781 to 4211)	3491 (3288 to 3700)	383 (330 to 441)	116 (79 to 164)	0 (0 to 0)	3899 (3694 to 4115)	3412 (3213 to 3615)	374 (323 to 431)	113 (78 to 160)	0 (0 to 0)
Luxembourg	1999	4604 (4373 to 4836)	3989 (3776 to 4197)	490 (434 to 551)	125 (92 to 166)	0 (0 to 0)	4499 (4273 to 4726)	3898 (3690 to 4101)	479 (424 to 538)	122 (89 to 162)	0 (0 to 0)
Luxembourg	2000	5436 (5200 to 5684)	4606 (4381 to 4835)	691 (627 to 759)	139 (108 to 173)	0 (0 to 0)	5312 (5081 to 5554)	4501 (4281 to 4724)	675 (613 to 742)	136 (105 to 169)	0 (0 to 0)
Luxembourg	2001	6078 (5851 to 6314)	5094 (4873 to 5326)	835 (767 to 906)	149 (120 to 181)	0 (0 to 0)	5939 (5717 to 6169)	4978 (4762 to 5205)	816 (750 to 885)	146 (117 to 177)	0 (0 to 0)
Luxembourg	2002	6870 (6623 to 7120)	5753 (5518 to 5995)	947 (877 to 1018)	170 (141 to 202)	0 (0 to 0)	6713 (6471 to 6958)	5621 (5391 to 5858)	926 (857 to 995)	166 (137 to 198)	0 (0 to 0)

Luxembourg	2003	7306 (7054 to 7577)	6122 (5896 to 6381)	989 (919 to 1058)	195 (166 to 230)	0 (0 to 0)	7139 (6893 to 7403)	5982 (5762 to 6236)	966 (898 to 1034)	191 (162 to 225)	0 (0 to 0)
Luxembourg	2004	7548 (7296 to 7792)	6339 (6109 to 6582)	996 (927 to 1064)	213 (183 to 247)	0 (0 to 0)	7376 (7129 to 7614)	6194 (5970 to 6432)	973 (906 to 1040)	209 (178 to 241)	0 (0 to 0)
Luxembourg	2005	7649 (7397 to 7903)	6441 (6201 to 6685)	976 (907 to 1046)	232 (201 to 268)	0 (0 to 0)	7474 (7228 to 7722)	6293 (6060 to 6532)	954 (886 to 1022)	227 (197 to 261)	0 (0 to 0)
Luxembourg	2006	7411 (7170 to 7667)	6260 (6037 to 6511)	911 (848 to 984)	240 (210 to 276)	0 (0 to 0)	7241 (7006 to 7492)	6116 (5899 to 6362)	890 (828 to 962)	234 (205 to 270)	0 (0 to 0)
Luxembourg	2007	7458 (7219 to 7715)	6360 (6128 to 6609)	835 (772 to 903)	262 (229 to 299)	0 (0 to 0)	7287 (7054 to 7539)	6215 (5987 to 6458)	816 (754 to 882)	256 (224 to 292)	0 (0 to 0)
Luxembourg	2008	7545 (7306 to 7791)	6493 (6250 to 6729)	795 (732 to 862)	257 (225 to 296)	0 (0 to 0)	7372 (7139 to 7613)	6344 (6107 to 6575)	777 (715 to 842)	251 (220 to 290)	0 (0 to 0)
Luxembourg	2009	7759 (7521 to 8023)	6685 (6447 to 6938)	784 (723 to 851)	290 (255 to 330)	0 (0 to 0)	7581 (7349 to 7840)	6532 (6299 to 6779)	766 (706 to 832)	283 (249 to 323)	0 (0 to 0)
Luxembourg	2010	7580 (7323 to 7842)	6496 (6253 to 6736)	769 (704 to 835)	314 (275 to 355)	0 (0 to 0)	7407 (7156 to 7663)	6348 (6110 to 6582)	752 (688 to 816)	307 (268 to 347)	0 (0 to 0)
Luxembourg	2011	7024 (6777 to 7282)	5968 (5730 to 6208)	736 (674 to 796)	320 (281 to 359)	0 (0 to 0)	6863 (6622 to 7115)	5832 (5599 to 6066)	719 (659 to 778)	313 (274 to 351)	0 (0 to 0)
Luxembourg	2012	6815 (6563 to 7066)	5777 (5539 to 6015)	723 (660 to 788)	315 (277 to 355)	0 (0 to 0)	6659 (6413 to 6904)	5645 (5412 to 5878)	707 (644 to 770)	308 (271 to 347)	0 (0 to 0)

Luxembourg	2013	6320 (6073 to 6571)	5370 (5134 to 5625)	691 (628 to 752)	259 (225 to 295)	0 (0 to 0)	6175 (5934 to 6421)	5247 (5017 to 5496)	675 (614 to 735)	253 (220 to 288)	0 (0 to 0)
Luxembourg	2014	6162 (5909 to 6406)	5257 (5012 to 5485)	677 (614 to 738)	228 (196 to 263)	0 (0 to 0)	6022 (5774 to 6259)	5137 (4898 to 5360)	661 (600 to 722)	223 (192 to 257)	0 (0 to 0)
Luxembourg	2015	6087 (5845 to 6326)	5212 (4968 to 5445)	663 (599 to 728)	212 (182 to 246)	0 (0 to 0)	5948 (5712 to 6181)	5093 (4854 to 5321)	648 (586 to 711)	207 (178 to 241)	0 (0 to 0)
Luxembourg	2016	6036 (5783 to 6312)	5179 (4932 to 5428)	655 (585 to 729)	202 (169 to 241)	0 (0 to 0)	5898 (5651 to 6167)	5060 (4819 to 5304)	640 (572 to 712)	198 (165 to 236)	0 (0 to 0)
Luxembourg	2017	6066 (5714 to 6448)	5215 (4906 to 5536)	656 (571 to 748)	195 (153 to 244)	0 (0 to 0)	5928 (5584 to 6301)	5096 (4794 to 5409)	641 (558 to 731)	191 (150 to 238)	0 (0 to 0)
Luxembourg	2018	6114 (5714 to 6525)	5254 (4915 to 5614)	663 (576 to 756)	197 (154 to 247)	0 (0 to 0)	5974 (5583 to 6376)	5134 (4802 to 5486)	648 (563 to 739)	193 (151 to 241)	0 (0 to 0)
Luxembourg	2019	6227 (5813 to 6673)	5357 (4994 to 5742)	669 (582 to 764)	200 (157 to 250)	0 (0 to 0)	6085 (5680 to 6520)	5235 (4880 to 5611)	654 (568 to 747)	196 (154 to 244)	0 (0 to 0)
Luxembourg	2020	6281 (5824 to 6780)	5404 (5013 to 5831)	675 (586 to 770)	202 (159 to 252)	0 (0 to 0)	6138 (5691 to 6625)	5280 (4899 to 5698)	660 (573 to 753)	198 (155 to 246)	0 (0 to 0)
Luxembourg	2021	6331 (5867 to 6831)	5447 (5043 to 5902)	680 (590 to 775)	204 (161 to 254)	0 (0 to 0)	6186 (5733 to 6675)	5322 (4928 to 5767)	664 (577 to 757)	200 (157 to 248)	0 (0 to 0)
Luxembourg	2022	6383 (5878 to 6927)	5492 (5041 to 5964)	684 (593 to 780)	207 (163 to 257)	0 (0 to 0)	6237 (5744 to 6768)	5366 (4926 to 5828)	668 (580 to 762)	202 (159 to 251)	0 (0 to 0)

Luxembourg	2023	6448 (5937 to 7017)	5552 (5068 to 6077)	687 (597 to 783)	209 (164 to 259)	0 (0 to 0)	6300 (5801 to 6856)	5425 (4952 to 5938)	672 (583 to 765)	204 (160 to 253)	0 (0 to 0)
Luxembourg	2024	6488 (5910 to 7086)	5593 (5076 to 6150)	684 (595 to 780)	210 (165 to 260)	0 (0 to 0)	6339 (5775 to 6924)	5466 (4960 to 6010)	669 (581 to 763)	205 (161 to 254)	0 (0 to 0)
Luxembourg	2025	6524 (5947 to 7150)	5633 (5117 to 6213)	681 (592 to 776)	211 (166 to 261)	0 (0 to 0)	6375 (5812 to 6987)	5504 (5000 to 6071)	665 (578 to 758)	206 (162 to 255)	0 (0 to 0)
Luxembourg	2026	6554 (5953 to 7228)	5666 (5119 to 6288)	676 (588 to 770)	211 (166 to 262)	0 (0 to 0)	6404 (5817 to 7063)	5537 (5002 to 6144)	661 (575 to 752)	206 (162 to 256)	0 (0 to 0)
Luxembourg	2027	6586 (5950 to 7283)	5702 (5106 to 6337)	672 (585 to 763)	212 (166 to 264)	0 (0 to 0)	6435 (5814 to 7116)	5572 (4989 to 6192)	656 (571 to 746)	207 (162 to 258)	0 (0 to 0)
Luxembourg	2028	6620 (5971 to 7325)	5741 (5134 to 6414)	667 (581 to 757)	212 (166 to 264)	0 (0 to 0)	6468 (5834 to 7158)	5609 (5017 to 6267)	652 (567 to 740)	207 (162 to 258)	0 (0 to 0)
Luxembourg	2029	6661 (5999 to 7387)	5785 (5184 to 6463)	663 (577 to 752)	213 (166 to 266)	0 (0 to 0)	6509 (5862 to 7219)	5653 (5065 to 6315)	647 (563 to 735)	208 (163 to 260)	0 (0 to 0)
Luxembourg	2030	6708 (6057 to 7439)	5835 (5221 to 6518)	659 (573 to 748)	214 (167 to 267)	0 (0 to 0)	6555 (5918 to 7268)	5702 (5102 to 6369)	644 (559 to 731)	209 (163 to 261)	0 (0 to 0)
Luxembourg	2031	6757 (6051 to 7519)	5887 (5246 to 6573)	655 (571 to 744)	215 (168 to 269)	0 (0 to 0)	6602 (5913 to 7348)	5752 (5126 to 6423)	640 (558 to 727)	210 (164 to 262)	0 (0 to 0)
Luxembourg	2032	6803 (6061 to 7575)	5935 (5239 to 6651)	652 (567 to 740)	216 (169 to 270)	0 (0 to 0)	6647 (5922 to 7401)	5799 (5120 to 6499)	637 (554 to 723)	211 (165 to 264)	0 (0 to 0)

Luxembourg	2033	6845 (6067 to 7660)	5980 (5243 to 6725)	648 (563 to 737)	217 (170 to 271)	0 (0 to 0)	6688 (5928 to 7485)	5843 (5123 to 6571)	633 (551 to 720)	212 (166 to 264)	0 (0 to 0)
Luxembourg	2034	6883 (6109 to 7769)	6021 (5298 to 6828)	644 (561 to 733)	218 (171 to 271)	0 (0 to 0)	6726 (5970 to 7591)	5883 (5177 to 6672)	630 (548 to 716)	213 (167 to 265)	0 (0 to 0)
Luxembourg	2035	6917 (6084 to 7811)	6058 (5275 to 6866)	641 (558 to 728)	218 (171 to 272)	0 (0 to 0)	6759 (5945 to 7633)	5920 (5155 to 6709)	626 (545 to 711)	213 (167 to 265)	0 (0 to 0)
Luxembourg	2036	6957 (6106 to 7884)	6101 (5307 to 6954)	637 (554 to 725)	219 (171 to 273)	0 (0 to 0)	6798 (5967 to 7703)	5961 (5185 to 6795)	623 (542 to 708)	214 (168 to 267)	0 (0 to 0)
Luxembourg	2037	6992 (6159 to 7916)	6137 (5342 to 6995)	634 (553 to 721)	220 (172 to 274)	0 (0 to 0)	6832 (6018 to 7735)	5997 (5220 to 6835)	620 (540 to 704)	215 (168 to 268)	0 (0 to 0)
Luxembourg	2038	7023 (6157 to 7942)	6171 (5363 to 7060)	632 (550 to 719)	221 (173 to 275)	0 (0 to 0)	6863 (6016 to 7761)	6030 (5240 to 6899)	617 (537 to 703)	216 (169 to 269)	0 (0 to 0)
Luxembourg	2039	7057 (6213 to 8025)	6206 (5390 to 7118)	629 (548 to 715)	222 (174 to 278)	0 (0 to 0)	6896 (6071 to 7841)	6064 (5267 to 6955)	615 (535 to 699)	217 (170 to 271)	0 (0 to 0)
Luxembourg	2040	7086 (6215 to 8054)	6237 (5431 to 7148)	627 (545 to 714)	223 (174 to 279)	0 (0 to 0)	6924 (6073 to 7870)	6094 (5307 to 6984)	613 (533 to 698)	218 (170 to 273)	0 (0 to 0)
Luxembourg	2041	7111 (6230 to 8169)	6263 (5429 to 7227)	624 (542 to 713)	223 (174 to 281)	0 (0 to 0)	6948 (6088 to 7982)	6120 (5305 to 7062)	610 (530 to 697)	218 (170 to 274)	0 (0 to 0)
Luxembourg	2042	7140 (6243 to 8169)	6294 (5458 to 7274)	622 (539 to 711)	224 (175 to 282)	0 (0 to 0)	6977 (6100 to 7983)	6150 (5333 to 7108)	608 (527 to 694)	219 (171 to 276)	0 (0 to 0)

Luxembourg	2043	7163 (6299 to 8215)	6318 (5497 to 7323)	620 (537 to 708)	225 (175 to 283)	0 (0 to 0)	6999 (6155 to 8027)	6174 (5371 to 7156)	605 (525 to 692)	220 (171 to 277)	0 (0 to 0)
Luxembourg	2044	7190 (6292 to 8281)	6347 (5507 to 7380)	617 (535 to 706)	226 (176 to 284)	0 (0 to 0)	7026 (6148 to 8092)	6202 (5381 to 7212)	603 (522 to 690)	221 (172 to 278)	0 (0 to 0)
Luxembourg	2045	7215 (6258 to 8284)	6373 (5484 to 7396)	615 (531 to 703)	227 (176 to 286)	0 (0 to 0)	7050 (6115 to 8095)	6228 (5359 to 7227)	601 (519 to 687)	221 (172 to 279)	0 (0 to 0)
Luxembourg	2046	7237 (6270 to 8327)	6398 (5476 to 7447)	612 (528 to 700)	227 (176 to 287)	0 (0 to 0)	7071 (6127 to 8137)	6252 (5351 to 7277)	598 (516 to 684)	222 (172 to 280)	0 (0 to 0)
Luxembourg	2047	7258 (6273 to 8348)	6421 (5489 to 7442)	609 (524 to 698)	228 (177 to 288)	0 (0 to 0)	7092 (6129 to 8157)	6274 (5364 to 7271)	595 (513 to 682)	223 (173 to 282)	0 (0 to 0)
Luxembourg	2048	7281 (6272 to 8420)	6445 (5477 to 7519)	607 (521 to 695)	229 (177 to 290)	0 (0 to 0)	7115 (6128 to 8228)	6298 (5351 to 7347)	593 (509 to 679)	223 (173 to 283)	0 (0 to 0)
Luxembourg	2049	7296 (6268 to 8445)	6462 (5462 to 7557)	604 (518 to 692)	229 (177 to 291)	0 (0 to 0)	7129 (6125 to 8252)	6314 (5337 to 7384)	590 (506 to 676)	224 (173 to 284)	0 (0 to 0)
Luxembourg	2050	7320 (6259 to 8505)	6488 (5492 to 7622)	602 (515 to 690)	230 (178 to 293)	0 (0 to 0)	7152 (6116 to 8311)	6339 (5367 to 7448)	588 (503 to 674)	225 (174 to 286)	0 (0 to 0)
Madagascar	1995	22 (18 to 27)	9 (7 to 11)	9 (6 to 13)	2 (1 to 4)	3 (3 to 3)	82 (67 to 101)	32 (24 to 42)	33 (23 to 49)	7 (3 to 13)	10 (10 to 10)
Madagascar	1996	23 (19 to 28)	9 (7 to 12)	9 (6 to 13)	2 (1 to 4)	3 (3 to 3)	83 (68 to 102)	33 (25 to 43)	33 (23 to 48)	7 (3 to 13)	10 (10 to 10)
Madagascar	1997	22 (18 to 26)	9 (7 to 12)	9 (6 to 13)	2 (1 to 3)	2 (2 to 2)	79 (65 to 97)	32 (24 to 43)	32 (22 to 47)	7 (3 to 13)	8 (8 to 8)

Madagascar	1998	24 (20 to 29)	9 (7 to 12)	9 (6 to 13)	2 (1 to 4)	4 (4 to 4)	87 (72 to 104)	34 (26 to 45)	32 (22 to 47)	7 (3 to 13)	13 (13 to 13)
Madagascar	1999	21 (18 to 26)	9 (7 to 12)	9 (6 to 12)	2 (1 to 3)	2 (2 to 2)	79 (65 to 97)	33 (25 to 44)	32 (22 to 45)	7 (3 to 13)	7 (7 to 7)
Madagascar	2000	22 (18 to 27)	10 (7 to 13)	9 (6 to 12)	2 (1 to 4)	2 (2 to 2)	81 (66 to 100)	35 (27 to 46)	32 (22 to 46)	7 (3 to 13)	7 (7 to 7)
Madagascar	2001	23 (19 to 28)	10 (8 to 13)	9 (6 to 12)	2 (1 to 4)	2 (2 to 2)	84 (69 to 103)	37 (28 to 49)	32 (22 to 45)	7 (3 to 13)	8 (8 to 8)
Madagascar	2002	19 (16 to 24)	8 (6 to 11)	8 (5 to 11)	2 (1 to 3)	2 (2 to 2)	71 (59 to 87)	30 (23 to 39)	28 (19 to 40)	6 (3 to 11)	7 (7 to 7)
Madagascar	2003	23 (20 to 28)	10 (7 to 13)	8 (6 to 12)	2 (1 to 3)	3 (3 to 3)	86 (71 to 103)	36 (27 to 47)	31 (20 to 43)	7 (3 to 12)	13 (13 to 13)
Madagascar	2004	27 (22 to 32)	11 (9 to 15)	9 (6 to 13)	2 (1 to 4)	5 (5 to 5)	98 (82 to 117)	41 (31 to 53)	32 (22 to 47)	8 (4 to 13)	17 (17 to 17)
Madagascar	2005	25 (21 to 29)	10 (7 to 13)	8 (6 to 12)	2 (1 to 3)	5 (5 to 5)	92 (77 to 108)	36 (27 to 46)	31 (21 to 44)	7 (3 to 12)	18 (18 to 18)
Madagascar	2006	24 (20 to 28)	10 (7 to 13)	8 (6 to 12)	2 (1 to 4)	4 (4 to 4)	88 (73 to 104)	36 (27 to 46)	31 (21 to 43)	7 (3 to 13)	14 (14 to 14)
Madagascar	2007	24 (20 to 28)	9 (7 to 12)	8 (6 to 11)	2 (1 to 3)	4 (4 to 4)	87 (73 to 102)	33 (25 to 43)	30 (21 to 42)	7 (3 to 13)	16 (16 to 16)
Madagascar	2008	24 (20 to 28)	9 (7 to 11)	8 (6 to 12)	2 (1 to 4)	5 (5 to 5)	88 (74 to 103)	32 (24 to 41)	31 (21 to 42)	8 (3 to 13)	17 (17 to 17)
Madagascar	2009	20 (17 to 24)	7 (5 to 9)	8 (5 to 10)	2 (1 to 3)	3 (3 to 3)	74 (62 to 88)	27 (20 to 34)	28 (19 to 38)	7 (3 to 12)	12 (12 to 12)
Madagascar	2010	23 (20 to 27)	8 (6 to 10)	8 (5 to 10)	2 (1 to 3)	6 (6 to 6)	84 (72 to 98)	29 (22 to 37)	27 (19 to 38)	7 (3 to 12)	21 (21 to 21)
Madagascar	2011	20 (17 to 24)	8 (6 to 10)	7 (5 to 10)	2 (1 to 3)	3 (3 to 3)	73 (61 to 87)	29 (22 to 38)	25 (18 to 35)	6 (3 to 12)	12 (12 to 12)
Madagascar	2012	19 (16 to 23)	8 (6 to 10)	6 (4 to 9)	2 (1 to 3)	4 (4 to 4)	71 (60 to 84)	28 (21 to 37)	24 (16 to 33)	6 (3 to 11)	13 (13 to 13)

Madagascar	2013	21 (18 to 24)	8 (6 to 11)	6 (4 to 9)	2 (1 to 3)	5 (5 to 5)	76 (64 to 89)	30 (22 to 39)	23 (16 to 33)	6 (3 to 11)	17 (17 to 17)
Madagascar	2014	21 (17 to 24)	9 (6 to 11)	6 (4 to 9)	2 (1 to 3)	4 (4 to 4)	75 (63 to 89)	32 (24 to 42)	23 (16 to 32)	6 (3 to 11)	15 (15 to 15)
Madagascar	2015	21 (18 to 25)	10 (7 to 13)	6 (4 to 9)	2 (1 to 3)	4 (4 to 4)	78 (65 to 92)	35 (26 to 46)	23 (16 to 32)	6 (3 to 11)	14 (14 to 14)
Madagascar	2016	22 (19 to 26)	11 (8 to 14)	6 (4 to 9)	2 (1 to 3)	4 (4 to 4)	82 (68 to 96)	39 (29 to 51)	23 (16 to 32)	6 (3 to 11)	14 (14 to 14)
Madagascar	2017	22 (18 to 27)	11 (8 to 15)	6 (4 to 9)	2 (1 to 3)	3 (3 to 3)	81 (65 to 100)	42 (31 to 54)	23 (16 to 32)	6 (3 to 11)	11 (11 to 11)
Madagascar	2018	22 (18 to 27)	11 (8 to 14)	6 (4 to 9)	2 (1 to 3)	3 (3 to 3)	80 (65 to 99)	40 (30 to 53)	24 (16 to 33)	6 (3 to 11)	10 (10 to 10)
Madagascar	2019	24 (19 to 30)	13 (10 to 17)	7 (5 to 9)	2 (1 to 3)	3 (3 to 3)	88 (70 to 110)	47 (35 to 62)	24 (17 to 34)	6 (3 to 12)	10 (10 to 10)
Madagascar	2020	25 (20 to 31)	13 (10 to 17)	7 (5 to 9)	2 (1 to 3)	3 (3 to 3)	90 (72 to 113)	49 (36 to 64)	25 (17 to 35)	7 (3 to 13)	10 (9 to 11)
Madagascar	2021	25 (20 to 31)	14 (10 to 18)	7 (5 to 10)	2 (1 to 3)	3 (3 to 3)	92 (72 to 115)	50 (37 to 66)	25 (17 to 35)	7 (3 to 13)	10 (9 to 11)
Madagascar	2022	25 (20 to 32)	14 (10 to 18)	7 (5 to 10)	2 (1 to 3)	3 (2 to 3)	92 (74 to 117)	51 (37 to 67)	25 (17 to 35)	7 (3 to 13)	10 (9 to 11)
Madagascar	2023	25 (20 to 32)	14 (10 to 18)	7 (5 to 10)	2 (1 to 4)	3 (2 to 3)	93 (74 to 117)	51 (38 to 68)	25 (18 to 35)	7 (3 to 13)	10 (9 to 11)
Madagascar	2024	26 (20 to 33)	14 (10 to 19)	7 (5 to 10)	2 (1 to 4)	3 (2 to 3)	94 (75 to 120)	52 (38 to 70)	25 (18 to 35)	7 (3 to 13)	10 (9 to 11)
Madagascar	2025	26 (20 to 33)	14 (10 to 19)	7 (5 to 10)	2 (1 to 4)	3 (2 to 3)	94 (74 to 120)	52 (38 to 70)	26 (18 to 36)	7 (3 to 13)	10 (9 to 11)
Madagascar	2026	26 (21 to 33)	15 (11 to 19)	7 (5 to 10)	2 (1 to 4)	3 (2 to 3)	96 (75 to 122)	53 (39 to 71)	26 (18 to 36)	7 (3 to 13)	10 (8 to 11)
Madagascar	2027	27 (21 to 34)	15 (11 to 20)	7 (5 to 10)	2 (1 to 4)	3 (2 to 3)	97 (76 to 124)	55 (40 to 73)	26 (18 to 36)	7 (3 to 14)	10 (8 to 11)

Madagascar	2028	27 (21 to 34)	15 (11 to 20)	7 (5 to 10)	2 (1 to 4)	3 (2 to 3)	99 (77 to 126)	56 (41 to 75)	26 (18 to 36)	7 (3 to 14)	10 (8 to 11)
Madagascar	2029	27 (21 to 35)	16 (11 to 21)	7 (5 to 10)	2 (1 to 4)	3 (2 to 3)	100 (78 to 128)	57 (42 to 77)	26 (18 to 36)	7 (3 to 14)	9 (8 to 11)
Madagascar	2030	28 (22 to 36)	16 (12 to 21)	7 (5 to 10)	2 (1 to 4)	3 (2 to 3)	102 (80 to 130)	59 (43 to 79)	26 (18 to 36)	7 (3 to 14)	9 (8 to 11)
Madagascar	2031	28 (22 to 36)	16 (12 to 22)	7 (5 to 10)	2 (1 to 4)	3 (2 to 3)	103 (81 to 132)	60 (44 to 81)	26 (18 to 36)	8 (3 to 14)	9 (8 to 11)
Madagascar	2032	29 (22 to 37)	17 (12 to 23)	7 (5 to 10)	2 (1 to 4)	3 (2 to 3)	105 (82 to 134)	61 (45 to 82)	26 (18 to 37)	8 (4 to 14)	9 (8 to 11)
Madagascar	2033	29 (23 to 37)	17 (12 to 23)	7 (5 to 10)	2 (1 to 4)	3 (2 to 3)	106 (83 to 136)	63 (46 to 84)	27 (18 to 37)	8 (4 to 15)	9 (8 to 11)
Madagascar	2034	29 (23 to 38)	17 (13 to 23)	7 (5 to 10)	2 (1 to 4)	3 (2 to 3)	108 (84 to 139)	64 (47 to 86)	27 (18 to 37)	8 (4 to 15)	9 (8 to 11)
Madagascar	2035	30 (23 to 38)	18 (13 to 24)	7 (5 to 10)	2 (1 to 4)	3 (2 to 3)	109 (85 to 140)	65 (48 to 88)	27 (18 to 37)	8 (4 to 15)	9 (8 to 11)
Madagascar	2036	30 (24 to 39)	18 (13 to 25)	7 (5 to 10)	2 (1 to 4)	3 (2 to 3)	111 (87 to 143)	67 (49 to 90)	27 (19 to 38)	8 (4 to 15)	9 (8 to 12)
Madagascar	2037	31 (24 to 39)	19 (14 to 25)	7 (5 to 10)	2 (1 to 4)	3 (2 to 3)	113 (87 to 144)	68 (50 to 92)	27 (19 to 38)	8 (4 to 15)	9 (8 to 11)
Madagascar	2038	31 (24 to 40)	19 (14 to 25)	7 (5 to 10)	2 (1 to 4)	3 (2 to 3)	114 (89 to 146)	69 (51 to 93)	27 (19 to 38)	8 (4 to 16)	9 (8 to 12)
Madagascar	2039	32 (25 to 41)	19 (14 to 26)	8 (5 to 11)	2 (1 to 4)	2 (2 to 3)	116 (91 to 150)	71 (52 to 96)	28 (19 to 39)	8 (4 to 16)	9 (7 to 12)
Madagascar	2040	32 (25 to 41)	20 (14 to 27)	8 (5 to 11)	2 (1 to 4)	2 (2 to 3)	118 (92 to 151)	72 (53 to 97)	28 (19 to 39)	9 (4 to 16)	9 (7 to 12)
Madagascar	2041	33 (25 to 42)	20 (15 to 27)	8 (5 to 11)	2 (1 to 4)	2 (2 to 3)	120 (93 to 155)	74 (54 to 99)	28 (19 to 39)	9 (4 to 16)	9 (7 to 11)
Madagascar	2042	33 (26 to 43)	21 (15 to 28)	8 (5 to 11)	2 (1 to 5)	2 (2 to 3)	122 (95 to 157)	75 (55 to 102)	28 (20 to 40)	9 (4 to 17)	9 (7 to 11)

Madagascar	2043	34 (26 to 43)	21 (15 to 28)	8 (5 to 11)	2 (1 to 5)	2 (2 to 3)	124 (96 to 159)	77 (56 to 104)	29 (20 to 40)	9 (4 to 17)	9 (7 to 12)
Madagascar	2044	34 (26 to 44)	21 (16 to 29)	8 (5 to 11)	3 (1 to 5)	2 (2 to 3)	126 (97 to 162)	79 (57 to 105)	29 (20 to 41)	9 (4 to 17)	9 (7 to 12)
Madagascar	2045	35 (27 to 45)	22 (16 to 29)	8 (5 to 11)	3 (1 to 5)	2 (2 to 3)	128 (99 to 165)	80 (58 to 107)	29 (20 to 41)	9 (4 to 18)	9 (7 to 12)
Madagascar	2046	35 (27 to 46)	22 (16 to 30)	8 (6 to 11)	3 (1 to 5)	2 (2 to 3)	130 (100 to 167)	82 (59 to 109)	29 (20 to 41)	10 (4 to 18)	9 (7 to 12)
Madagascar	2047	36 (28 to 47)	23 (16 to 30)	8 (6 to 11)	3 (1 to 5)	2 (2 to 3)	131 (102 to 171)	83 (60 to 111)	30 (20 to 42)	10 (4 to 18)	9 (7 to 12)
Madagascar	2048	36 (28 to 47)	23 (17 to 31)	8 (6 to 11)	3 (1 to 5)	2 (2 to 3)	133 (102 to 172)	84 (61 to 113)	30 (21 to 42)	10 (4 to 19)	9 (7 to 12)
Madagascar	2049	37 (29 to 48)	23 (17 to 31)	8 (6 to 12)	3 (1 to 5)	2 (2 to 3)	135 (105 to 177)	86 (62 to 115)	30 (21 to 43)	10 (5 to 19)	9 (7 to 12)
Madagascar	2050	38 (28 to 48)	24 (17 to 32)	8 (6 to 12)	3 (1 to 5)	2 (2 to 3)	138 (104 to 177)	88 (63 to 117)	31 (21 to 43)	10 (5 to 19)	9 (7 to 12)
Malawi	1995	13 (11 to 15)	4 (3 to 5)	3 (2 to 4)	1 (0 to 2)	5 (5 to 5)	42 (37 to 48)	12 (9 to 15)	10 (7 to 14)	4 (2 to 7)	16 (16 to 16)
Malawi	1996	13 (12 to 15)	3 (2 to 4)	3 (2 to 4)	1 (0 to 2)	6 (6 to 6)	45 (40 to 50)	11 (8 to 14)	10 (7 to 14)	3 (2 to 6)	20 (20 to 20)
Malawi	1997	15 (13 to 17)	3 (3 to 5)	3 (2 to 4)	1 (0 to 2)	7 (7 to 7)	50 (45 to 56)	12 (9 to 15)	10 (7 to 14)	3 (2 to 6)	25 (25 to 25)
Malawi	1998	13 (11 to 14)	3 (3 to 4)	3 (2 to 4)	1 (0 to 2)	6 (6 to 6)	42 (37 to 47)	11 (8 to 15)	9 (6 to 13)	3 (1 to 5)	19 (19 to 19)
Malawi	1999	17 (16 to 19)	3 (3 to 4)	3 (2 to 4)	1 (0 to 1)	10 (10 to 10)	57 (52 to 62)	11 (8 to 15)	8 (6 to 12)	2 (1 to 5)	35 (35 to 35)
Malawi	2000	15 (14 to 17)	3 (3 to 5)	2 (1 to 3)	1 (0 to 1)	9 (9 to 9)	51 (47 to 56)	12 (9 to 15)	7 (5 to 10)	2 (1 to 4)	31 (31 to 31)
Malawi	2001	13 (12 to 14)	3 (2 to 4)	2 (1 to 2)	0 (0 to 1)	8 (8 to 8)	43 (39 to 47)	10 (8 to 13)	6 (4 to 8)	1 (1 to 3)	25 (25 to 25)

Malawi	2002	13 (12 to 14)	4 (3 to 5)	2 (1 to 2)	1 (0 to 1)	7 (7 to 7)	42 (38 to 47)	12 (9 to 15)	6 (4 to 8)	2 (1 to 3)	23 (23 to 23)
Malawi	2003	15 (14 to 17)	4 (3 to 5)	2 (1 to 3)	1 (0 to 1)	9 (9 to 9)	50 (46 to 56)	13 (10 to 17)	6 (4 to 8)	2 (1 to 4)	29 (29 to 29)
Malawi	2004	18 (17 to 20)	4 (3 to 6)	2 (1 to 3)	1 (0 to 2)	12 (12 to 12)	61 (56 to 66)	14 (10 to 18)	6 (4 to 9)	3 (1 to 5)	38 (38 to 38)
Malawi	2005	19 (18 to 21)	4 (3 to 6)	2 (1 to 3)	1 (0 to 2)	12 (12 to 12)	63 (58 to 69)	14 (10 to 19)	6 (4 to 9)	3 (2 to 6)	40 (40 to 40)
Malawi	2006	24 (22 to 26)	4 (3 to 6)	2 (1 to 3)	1 (1 to 3)	16 (16 to 16)	80 (74 to 86)	14 (10 to 18)	7 (5 to 10)	5 (2 to 8)	54 (54 to 54)
Malawi	2007	31 (29 to 33)	5 (4 to 6)	3 (2 to 4)	2 (1 to 4)	21 (21 to 21)	102 (95 to 110)	16 (12 to 21)	9 (6 to 12)	6 (3 to 12)	71 (71 to 71)
Malawi	2008	35 (33 to 38)	6 (4 to 7)	3 (2 to 4)	2 (1 to 4)	24 (24 to 24)	117 (109 to 127)	19 (14 to 25)	10 (7 to 14)	7 (3 to 14)	81 (81 to 81)
Malawi	2009	32 (30 to 35)	6 (4 to 7)	3 (2 to 4)	2 (1 to 4)	22 (22 to 22)	107 (100 to 116)	18 (14 to 24)	10 (7 to 14)	6 (3 to 12)	72 (72 to 72)
Malawi	2010	27 (25 to 30)	6 (4 to 8)	3 (2 to 4)	2 (1 to 3)	17 (17 to 17)	91 (84 to 99)	19 (15 to 25)	10 (7 to 14)	5 (3 to 10)	56 (56 to 56)
Malawi	2011	35 (33 to 37)	6 (4 to 8)	3 (2 to 4)	1 (1 to 3)	24 (24 to 24)	115 (109 to 123)	20 (15 to 26)	10 (7 to 14)	5 (2 to 9)	81 (81 to 81)
Malawi	2012	37 (35 to 39)	6 (5 to 8)	3 (2 to 4)	1 (1 to 2)	26 (26 to 26)	122 (115 to 130)	21 (16 to 27)	10 (7 to 14)	5 (2 to 8)	86 (86 to 86)
Malawi	2013	39 (37 to 42)	8 (6 to 10)	3 (2 to 5)	2 (1 to 3)	27 (27 to 27)	131 (123 to 141)	26 (19 to 34)	11 (8 to 15)	5 (2 to 10)	89 (89 to 89)
Malawi	2014	36 (34 to 39)	8 (6 to 11)	3 (2 to 5)	2 (1 to 3)	23 (23 to 23)	121 (113 to 131)	27 (20 to 36)	11 (8 to 16)	6 (3 to 10)	77 (77 to 77)
Malawi	2015	40 (37 to 44)	10 (7 to 13)	4 (3 to 5)	2 (1 to 4)	25 (25 to 25)	133 (123 to 145)	32 (24 to 42)	13 (9 to 17)	7 (3 to 13)	82 (82 to 82)
Malawi	2016	41 (38 to 44)	10 (7 to 13)	4 (3 to 6)	2 (1 to 4)	24 (24 to 24)	135 (125 to 148)	33 (25 to 44)	13 (9 to 18)	8 (4 to 14)	81 (81 to 81)

Malawi	2017	45 (41 to 51)	11 (8 to 14)	4 (3 to 6)	2 (1 to 4)	28 (28 to 28)	151 (136 to 169)	36 (27 to 47)	14 (10 to 19)	8 (4 to 15)	93 (93 to 93)
Malawi	2018	43 (39 to 48)	10 (7 to 13)	4 (3 to 6)	2 (1 to 4)	26 (26 to 26)	142 (128 to 160)	33 (24 to 43)	14 (10 to 19)	8 (4 to 14)	88 (88 to 88)
Malawi	2019	43 (39 to 48)	10 (7 to 13)	4 (3 to 6)	2 (1 to 4)	26 (26 to 27)	142 (129 to 161)	32 (24 to 44)	14 (10 to 19)	8 (4 to 14)	88 (88 to 88)
Malawi	2020	43 (39 to 49)	10 (7 to 14)	4 (3 to 6)	2 (1 to 4)	26 (25 to 28)	144 (128 to 163)	33 (25 to 45)	14 (10 to 20)	8 (4 to 15)	88 (83 to 93)
Malawi	2021	44 (39 to 49)	10 (8 to 14)	4 (3 to 6)	3 (1 to 4)	26 (25 to 29)	145 (129 to 164)	34 (25 to 46)	15 (10 to 20)	8 (4 to 15)	88 (82 to 95)
Malawi	2022	44 (39 to 50)	11 (8 to 14)	4 (3 to 6)	3 (1 to 5)	26 (24 to 29)	146 (128 to 167)	35 (25 to 47)	15 (10 to 20)	9 (4 to 15)	88 (80 to 97)
Malawi	2023	44 (39 to 51)	11 (8 to 15)	5 (3 to 6)	3 (1 to 5)	26 (24 to 29)	147 (129 to 168)	36 (26 to 48)	15 (11 to 21)	9 (4 to 16)	87 (79 to 97)
Malawi	2024	44 (39 to 51)	11 (8 to 15)	5 (3 to 6)	3 (1 to 5)	26 (23 to 29)	147 (129 to 171)	37 (27 to 50)	15 (11 to 21)	9 (4 to 16)	86 (77 to 97)
Malawi	2025	45 (38 to 52)	11 (8 to 15)	5 (3 to 6)	3 (1 to 5)	26 (23 to 29)	148 (128 to 171)	38 (27 to 51)	16 (11 to 22)	9 (5 to 17)	86 (76 to 97)
Malawi	2026	45 (39 to 52)	12 (8 to 16)	5 (3 to 7)	3 (1 to 5)	26 (23 to 29)	149 (129 to 174)	39 (28 to 52)	16 (11 to 22)	10 (5 to 17)	85 (75 to 97)
Malawi	2027	45 (39 to 53)	12 (9 to 16)	5 (3 to 7)	3 (1 to 5)	25 (22 to 30)	150 (129 to 176)	40 (28 to 54)	16 (11 to 22)	10 (5 to 17)	85 (74 to 98)
Malawi	2028	46 (39 to 53)	12 (9 to 17)	5 (4 to 7)	3 (1 to 5)	25 (22 to 30)	152 (130 to 178)	41 (29 to 55)	17 (12 to 23)	10 (5 to 18)	84 (73 to 98)
Malawi	2029	46 (39 to 54)	13 (9 to 17)	5 (4 to 7)	3 (2 to 6)	25 (22 to 30)	153 (130 to 181)	42 (30 to 57)	17 (12 to 23)	10 (5 to 18)	84 (73 to 98)
Malawi	2030	46 (40 to 55)	13 (9 to 18)	5 (4 to 7)	3 (2 to 6)	25 (22 to 30)	154 (132 to 184)	43 (30 to 59)	17 (12 to 24)	11 (5 to 19)	84 (72 to 100)
Malawi	2031	47 (40 to 56)	13 (9 to 18)	5 (4 to 7)	3 (2 to 6)	25 (21 to 30)	156 (132 to 186)	44 (31 to 60)	18 (12 to 24)	11 (5 to 19)	83 (71 to 99)

Malawi	2032	47 (40 to 56)	14 (10 to 19)	5 (4 to 7)	3 (2 to 6)	25 (21 to 30)	158 (133 to 187)	45 (32 to 63)	18 (13 to 25)	11 (5 to 20)	83 (71 to 101)
Malawi	2033	48 (40 to 58)	14 (10 to 20)	6 (4 to 8)	3 (2 to 6)	25 (21 to 30)	159 (132 to 192)	46 (32 to 65)	18 (13 to 25)	11 (6 to 20)	83 (71 to 100)
Malawi	2034	49 (40 to 58)	14 (10 to 20)	6 (4 to 8)	4 (2 to 6)	25 (21 to 30)	161 (134 to 194)	48 (33 to 67)	19 (13 to 26)	12 (6 to 21)	83 (70 to 101)
Malawi	2035	49 (40 to 60)	15 (10 to 21)	6 (4 to 8)	4 (2 to 6)	25 (21 to 30)	163 (134 to 199)	49 (34 to 70)	19 (13 to 26)	12 (6 to 22)	83 (69 to 101)
Malawi	2036	50 (41 to 61)	15 (10 to 21)	6 (4 to 8)	4 (2 to 7)	25 (21 to 31)	165 (136 to 202)	50 (34 to 70)	20 (14 to 27)	12 (6 to 22)	83 (69 to 104)
Malawi	2037	50 (41 to 61)	16 (11 to 22)	6 (4 to 8)	4 (2 to 7)	25 (21 to 30)	168 (136 to 204)	52 (35 to 73)	20 (14 to 27)	13 (6 to 23)	83 (69 to 101)
Malawi	2038	51 (42 to 63)	16 (11 to 23)	6 (4 to 8)	4 (2 to 7)	25 (20 to 32)	170 (138 to 209)	53 (36 to 75)	20 (14 to 28)	13 (6 to 24)	83 (68 to 105)
Malawi	2039	52 (42 to 63)	16 (11 to 23)	6 (4 to 9)	4 (2 to 7)	25 (21 to 32)	172 (139 to 210)	54 (37 to 77)	21 (15 to 28)	13 (7 to 24)	83 (69 to 105)
Malawi	2040	53 (43 to 65)	17 (11 to 24)	6 (4 to 9)	4 (2 to 7)	25 (20 to 32)	175 (142 to 217)	56 (38 to 79)	21 (15 to 29)	14 (7 to 25)	84 (68 to 106)
Malawi	2041	53 (43 to 66)	17 (12 to 24)	7 (5 to 9)	4 (2 to 8)	25 (20 to 32)	177 (143 to 219)	58 (39 to 81)	22 (15 to 30)	14 (7 to 26)	84 (68 to 106)
Malawi	2042	54 (44 to 67)	18 (12 to 25)	7 (5 to 9)	4 (2 to 8)	25 (20 to 32)	180 (145 to 222)	59 (39 to 84)	22 (15 to 30)	14 (7 to 26)	84 (68 to 106)
Malawi	2043	55 (44 to 68)	18 (12 to 26)	7 (5 to 9)	4 (2 to 8)	25 (20 to 33)	182 (147 to 227)	60 (40 to 87)	23 (16 to 31)	15 (7 to 27)	84 (68 to 109)
Malawi	2044	56 (44 to 69)	19 (12 to 27)	7 (5 to 9)	5 (2 to 8)	25 (20 to 33)	185 (147 to 228)	62 (41 to 89)	23 (16 to 32)	15 (7 to 28)	85 (67 to 110)
Malawi	2045	56 (45 to 70)	19 (13 to 27)	7 (5 to 10)	5 (2 to 9)	26 (20 to 33)	188 (149 to 234)	64 (43 to 91)	23 (16 to 32)	16 (8 to 28)	85 (68 to 111)
Malawi	2046	57 (45 to 72)	20 (13 to 28)	7 (5 to 10)	5 (2 to 9)	26 (20 to 33)	190 (151 to 239)	65 (44 to 93)	24 (17 to 33)	16 (8 to 29)	85 (68 to 110)

Malawi	2047	58 (47 to 73)	20 (13 to 29)	7 (5 to 10)	5 (2 to 9)	26 (20 to 35)	193 (155 to 243)	67 (44 to 95)	24 (17 to 33)	16 (8 to 30)	86 (68 to 116)
Malawi	2048	59 (46 to 74)	21 (14 to 30)	7 (5 to 10)	5 (2 to 9)	26 (21 to 34)	196 (155 to 246)	69 (45 to 99)	25 (17 to 34)	17 (8 to 30)	86 (68 to 114)
Malawi	2049	60 (47 to 76)	21 (14 to 30)	8 (5 to 10)	5 (3 to 9)	26 (21 to 35)	199 (157 to 253)	70 (45 to 101)	25 (17 to 35)	17 (8 to 31)	87 (69 to 115)
Malawi	2050	61 (48 to 77)	22 (14 to 31)	8 (5 to 11)	5 (3 to 10)	26 (21 to 36)	203 (160 to 256)	72 (47 to 105)	26 (18 to 35)	18 (9 to 32)	87 (69 to 118)
Malaysia	1995	132 (114 to 153)	63 (53 to 76)	50 (39 to 64)	17 (9 to 28)	2 (2 to 2)	384 (332 to 447)	183 (153 to 220)	146 (115 to 185)	48 (26 to 83)	7 (7 to 7)
Malaysia	1996	138 (120 to 159)	65 (55 to 78)	52 (41 to 65)	17 (10 to 29)	3 (3 to 3)	401 (350 to 464)	191 (162 to 226)	151 (120 to 191)	50 (28 to 84)	10 (10 to 10)
Malaysia	1997	140 (123 to 161)	65 (56 to 77)	53 (43 to 66)	18 (10 to 30)	4 (4 to 4)	408 (360 to 468)	191 (164 to 224)	154 (125 to 192)	52 (30 to 86)	12 (12 to 12)
Malaysia	1998	135 (118 to 155)	64 (55 to 74)	52 (42 to 65)	18 (10 to 29)	1 (1 to 1)	392 (345 to 450)	186 (159 to 214)	153 (123 to 188)	51 (30 to 84)	2 (2 to 2)
Malaysia	1999	144 (127 to 163)	69 (59 to 79)	55 (45 to 68)	19 (11 to 30)	1 (1 to 1)	419 (369 to 474)	201 (172 to 230)	161 (130 to 197)	55 (33 to 86)	3 (3 to 3)
Malaysia	2000	157 (139 to 177)	76 (65 to 87)	60 (49 to 72)	20 (13 to 32)	1 (1 to 1)	457 (405 to 515)	221 (190 to 253)	174 (142 to 211)	60 (36 to 92)	2 (2 to 2)
Malaysia	2001	170 (151 to 189)	84 (73 to 96)	62 (51 to 75)	22 (14 to 35)	0 (0 to 0)	494 (439 to 551)	245 (212 to 280)	182 (148 to 217)	65 (41 to 101)	1 (1 to 1)
Malaysia	2002	176 (157 to 196)	89 (77 to 100)	64 (52 to 76)	23 (15 to 36)	0 (0 to 0)	512 (458 to 570)	258 (224 to 293)	186 (153 to 222)	68 (43 to 105)	0 (0 to 0)
Malaysia	2003	192 (172 to 213)	98 (85 to 112)	68 (57 to 81)	25 (16 to 39)	0 (0 to 0)	558 (501 to 621)	285 (248 to 326)	199 (165 to 235)	73 (46 to 113)	0 (0 to 0)
Malaysia	2004	197 (177 to 220)	101 (87 to 116)	71 (59 to 83)	25 (16 to 38)	0 (0 to 0)	575 (515 to 641)	293 (254 to 339)	208 (173 to 243)	74 (46 to 112)	0 (0 to 0)
Malaysia	2005	204 (183 to 227)	104 (91 to 119)	75 (62 to 87)	25 (16 to 39)	0 (0 to 0)	595 (533 to 660)	304 (266 to 348)	217 (181 to 253)	74 (47 to 113)	0 (0 to 0)

Malaysia	2006	222 (200 to 245)	116 (102 to 133)	79 (67 to 93)	27 (17 to 40)	0 (0 to 0)	646 (582 to 714)	336 (296 to 387)	231 (194 to 270)	78 (50 to 116)	0 (0 to 0)
Malaysia	2007	235 (212 to 259)	123 (108 to 142)	83 (70 to 97)	28 (18 to 42)	0 (0 to 0)	683 (616 to 755)	360 (315 to 413)	241 (203 to 282)	82 (52 to 123)	0 (0 to 0)
Malaysia	2008	251 (226 to 277)	134 (117 to 153)	87 (74 to 102)	30 (19 to 45)	0 (0 to 0)	730 (658 to 806)	389 (342 to 447)	253 (215 to 297)	87 (55 to 130)	0 (0 to 0)
Malaysia	2009	269 (241 to 298)	145 (128 to 164)	91 (77 to 107)	33 (21 to 49)	0 (0 to 0)	783 (701 to 867)	422 (374 to 479)	265 (225 to 312)	95 (62 to 143)	0 (0 to 0)
Malaysia	2010	267 (241 to 295)	141 (125 to 160)	93 (79 to 109)	32 (21 to 48)	0 (0 to 0)	776 (702 to 859)	412 (365 to 465)	271 (230 to 317)	94 (61 to 141)	0 (0 to 0)
Malaysia	2011	292 (266 to 323)	155 (139 to 175)	102 (86 to 119)	35 (23 to 52)	0 (0 to 0)	851 (774 to 940)	452 (404 to 510)	296 (251 to 346)	102 (66 to 153)	0 (0 to 0)
Malaysia	2012	320 (292 to 352)	172 (153 to 193)	111 (95 to 129)	38 (24 to 56)	0 (0 to 0)	932 (850 to 1026)	500 (445 to 561)	323 (276 to 375)	110 (71 to 164)	0 (0 to 0)
Malaysia	2013	340 (309 to 372)	182 (163 to 204)	119 (101 to 137)	39 (25 to 58)	0 (0 to 0)	989 (899 to 1083)	530 (473 to 593)	346 (294 to 400)	113 (72 to 168)	0 (0 to 0)
Malaysia	2014	360 (327 to 396)	192 (172 to 215)	128 (109 to 148)	40 (26 to 58)	0 (0 to 0)	1049 (952 to 1152)	559 (501 to 625)	372 (316 to 432)	117 (75 to 170)	0 (0 to 0)
Malaysia	2015	381 (347 to 419)	200 (179 to 225)	137 (117 to 160)	43 (27 to 64)	0 (0 to 0)	1109 (1011 to 1222)	584 (522 to 656)	400 (341 to 466)	125 (79 to 186)	0 (0 to 0)
Malaysia	2016	390 (353 to 430)	201 (178 to 227)	144 (123 to 169)	45 (28 to 68)	0 (0 to 0)	1135 (1027 to 1254)	584 (519 to 660)	420 (357 to 492)	130 (81 to 197)	0 (0 to 0)
Malaysia	2017	409 (353 to 475)	208 (183 to 239)	153 (125 to 183)	47 (28 to 73)	0 (0 to 0)	1190 (1029 to 1382)	607 (532 to 696)	445 (364 to 532)	137 (83 to 214)	0 (0 to 0)
Malaysia	2018	436 (377 to 507)	227 (198 to 259)	159 (130 to 190)	50 (30 to 78)	0 (0 to 0)	1270 (1099 to 1476)	660 (577 to 754)	463 (378 to 554)	146 (88 to 228)	0 (0 to 0)

Malaysia	2019	458 (394 to 534)	241 (209 to 276)	164 (134 to 196)	53 (32 to 82)	0 (0 to 0)	1334 (1146 to 1555)	702 (610 to 804)	479 (389 to 572)	153 (93 to 239)	0 (0 to 0)
Malaysia	2020	478 (413 to 558)	253 (220 to 292)	170 (138 to 203)	55 (33 to 86)	0 (0 to 0)	1393 (1203 to 1624)	738 (641 to 851)	494 (403 to 590)	160 (97 to 250)	0 (0 to 0)
Malaysia	2021	496 (428 to 578)	265 (230 to 306)	174 (141 to 207)	57 (35 to 89)	0 (0 to 0)	1445 (1247 to 1682)	773 (670 to 890)	505 (412 to 604)	166 (101 to 259)	0 (0 to 0)
Malaysia	2022	514 (443 to 602)	278 (241 to 320)	177 (145 to 212)	59 (36 to 93)	0 (0 to 0)	1498 (1290 to 1752)	809 (702 to 932)	517 (421 to 618)	172 (104 to 269)	0 (0 to 1)
Malaysia	2023	533 (459 to 624)	290 (252 to 335)	181 (147 to 217)	61 (37 to 96)	0 (0 to 0)	1552 (1336 to 1816)	846 (733 to 977)	528 (429 to 631)	178 (108 to 279)	0 (0 to 1)
Malaysia	2024	552 (476 to 647)	303 (261 to 352)	185 (150 to 221)	63 (38 to 99)	0 (0 to 0)	1607 (1387 to 1885)	883 (760 to 1026)	538 (437 to 643)	185 (112 to 289)	0 (0 to 1)
Malaysia	2025	571 (491 to 669)	317 (273 to 368)	189 (154 to 226)	66 (40 to 102)	0 (0 to 0)	1662 (1429 to 1947)	922 (794 to 1072)	549 (447 to 657)	191 (116 to 298)	0 (0 to 1)
Malaysia	2026	590 (507 to 692)	330 (283 to 384)	192 (157 to 230)	68 (41 to 106)	0 (0 to 0)	1719 (1477 to 2015)	961 (825 to 1118)	560 (456 to 670)	197 (119 to 308)	0 (0 to 1)
Malaysia	2027	610 (523 to 714)	344 (296 to 400)	196 (160 to 234)	70 (42 to 109)	0 (0 to 0)	1778 (1524 to 2080)	1003 (862 to 1165)	571 (466 to 683)	204 (123 to 318)	0 (0 to 1)
Malaysia	2028	631 (541 to 739)	359 (308 to 418)	200 (163 to 239)	72 (44 to 113)	0 (0 to 0)	1839 (1575 to 2152)	1045 (898 to 1217)	582 (474 to 696)	211 (127 to 328)	0 (0 to 1)

Malaysia	2029	653 (559 to 764)	374 (321 to 437)	204 (166 to 244)	75 (45 to 116)	0 (0 to 0)	1901 (1629 to 2226)	1089 (934 to 1273)	593 (484 to 709)	218 (131 to 339)	0 (0 to 1)
Malaysia	2030	673 (575 to 790)	388 (332 to 454)	207 (170 to 248)	77 (46 to 120)	0 (0 to 0)	1960 (1674 to 2299)	1130 (967 to 1321)	604 (494 to 724)	225 (135 to 349)	0 (0 to 1)
Malaysia	2031	694 (593 to 812)	403 (343 to 469)	211 (173 to 252)	80 (48 to 123)	0 (0 to 0)	2020 (1728 to 2366)	1173 (1000 to 1367)	616 (502 to 735)	232 (139 to 358)	0 (0 to 1)
Malaysia	2032	715 (614 to 843)	417 (357 to 488)	215 (176 to 258)	82 (49 to 127)	0 (0 to 0)	2082 (1788 to 2454)	1215 (1040 to 1421)	627 (512 to 751)	239 (143 to 369)	0 (0 to 1)
Malaysia	2033	736 (631 to 873)	432 (368 to 507)	219 (179 to 263)	84 (51 to 130)	0 (0 to 0)	2143 (1837 to 2542)	1258 (1070 to 1476)	639 (523 to 765)	246 (148 to 380)	0 (0 to 1)
Malaysia	2034	757 (647 to 894)	447 (379 to 527)	224 (183 to 268)	87 (52 to 134)	0 (0 to 0)	2205 (1885 to 2604)	1301 (1105 to 1534)	651 (533 to 780)	253 (152 to 391)	0 (0 to 1)
Malaysia	2035	779 (660 to 925)	462 (390 to 546)	228 (186 to 272)	89 (54 to 138)	0 (0 to 0)	2269 (1922 to 2693)	1345 (1136 to 1590)	664 (543 to 793)	260 (156 to 402)	0 (0 to 1)
Malaysia	2036	800 (684 to 948)	477 (401 to 564)	232 (190 to 277)	92 (55 to 142)	0 (0 to 0)	2331 (1993 to 2760)	1388 (1167 to 1644)	676 (552 to 807)	267 (160 to 413)	0 (0 to 1)
Malaysia	2037	822 (701 to 974)	492 (411 to 585)	236 (193 to 282)	94 (56 to 146)	0 (0 to 0)	2395 (2041 to 2838)	1432 (1198 to 1703)	688 (564 to 822)	275 (164 to 425)	0 (0 to 1)
Malaysia	2038	845 (716 to 1003)	507 (424 to 604)	241 (197 to 288)	97 (58 to 150)	0 (0 to 0)	2460 (2084 to 2922)	1477 (1236 to 1759)	701 (575 to 838)	282 (168 to 437)	0 (0 to 1)

Malaysia	2039	868 (737 to 1029)	523 (437 to 625)	245 (202 to 293)	99 (59 to 154)	0 (0 to 0)	2527 (2146 to 2997)	1524 (1274 to 1820)	714 (587 to 853)	290 (172 to 447)	0 (0 to 1)
Malaysia	2040	892 (754 to 1062)	540 (448 to 646)	250 (206 to 298)	102 (61 to 158)	0 (0 to 0)	2599 (2195 to 3092)	1573 (1303 to 1882)	728 (599 to 869)	298 (177 to 459)	0 (0 to 1)
Malaysia	2041	917 (776 to 1090)	557 (461 to 669)	255 (209 to 304)	105 (63 to 162)	0 (0 to 0)	2672 (2261 to 3175)	1623 (1343 to 1947)	742 (609 to 886)	306 (182 to 471)	0 (0 to 1)
Malaysia	2042	943 (795 to 1120)	575 (475 to 693)	260 (213 to 311)	108 (64 to 166)	0 (0 to 0)	2746 (2315 to 3261)	1675 (1385 to 2019)	757 (620 to 907)	315 (187 to 483)	0 (0 to 1)
Malaysia	2043	969 (814 to 1161)	593 (491 to 712)	265 (216 to 317)	111 (66 to 171)	0 (0 to 0)	2822 (2372 to 3381)	1727 (1429 to 2073)	772 (630 to 923)	323 (192 to 497)	0 (0 to 1)
Malaysia	2044	995 (834 to 1193)	611 (503 to 739)	270 (220 to 324)	114 (67 to 176)	0 (0 to 0)	2898 (2428 to 3474)	1779 (1464 to 2151)	787 (642 to 943)	332 (197 to 512)	0 (0 to 1)
Malaysia	2045	1022 (856 to 1223)	629 (519 to 757)	275 (224 to 330)	117 (69 to 181)	0 (0 to 0)	2976 (2494 to 3563)	1833 (1510 to 2205)	802 (654 to 961)	341 (201 to 526)	0 (0 to 1)
Malaysia	2046	1048 (879 to 1258)	648 (534 to 781)	281 (229 to 336)	120 (71 to 185)	0 (0 to 0)	3054 (2561 to 3664)	1887 (1554 to 2274)	817 (666 to 979)	350 (207 to 539)	0 (0 to 1)
Malaysia	2047	1075 (899 to 1281)	666 (549 to 803)	286 (233 to 342)	123 (73 to 190)	0 (0 to 0)	3132 (2618 to 3730)	1941 (1598 to 2338)	832 (679 to 997)	358 (212 to 552)	0 (0 to 1)
Malaysia	2048	1102 (923 to 1312)	685 (564 to 829)	291 (237 to 349)	126 (75 to 194)	0 (0 to 0)	3210 (2688 to 3820)	1995 (1644 to 2415)	847 (691 to 1017)	367 (218 to 566)	0 (0 to 1)

Malaysia	2049	1129 (938 to 1347)	704 (579 to 854)	296 (241 to 355)	129 (77 to 199)	0 (0 to 0)	3289 (2733 to 3925)	2050 (1687 to 2487)	862 (703 to 1034)	376 (224 to 579)	0 (0 to 1)
Malaysia	2050	1157 (966 to 1383)	724 (597 to 878)	301 (246 to 361)	132 (79 to 204)	0 (0 to 0)	3370 (2812 to 4029)	2108 (1739 to 2557)	877 (715 to 1051)	385 (230 to 593)	0 (0 to 1)
Maldives	1995	591 (535 to 647)	145 (118 to 173)	380 (338 to 425)	66 (43 to 97)	0 (0 to 0)	885 (801 to 970)	217 (177 to 259)	569 (507 to 636)	99 (65 to 146)	0 (0 to 0)
Maldives	1996	575 (529 to 623)	147 (122 to 174)	362 (330 to 397)	65 (44 to 93)	0 (0 to 0)	860 (792 to 932)	221 (183 to 261)	541 (494 to 594)	98 (67 to 139)	0 (0 to 0)
Maldives	1997	578 (537 to 622)	157 (132 to 183)	352 (322 to 383)	66 (45 to 92)	4 (4 to 4)	865 (805 to 931)	235 (198 to 274)	527 (482 to 573)	99 (68 to 138)	5 (5 to 5)
Maldives	1998	590 (548 to 632)	175 (149 to 202)	348 (318 to 378)	67 (47 to 90)	0 (0 to 0)	883 (820 to 946)	262 (223 to 302)	521 (476 to 567)	100 (70 to 135)	0 (0 to 0)
Maldives	1999	592 (550 to 637)	186 (159 to 212)	342 (311 to 372)	64 (46 to 85)	1 (1 to 1)	886 (823 to 954)	278 (238 to 318)	511 (465 to 557)	95 (68 to 127)	2 (2 to 2)
Maldives	2000	566 (524 to 609)	183 (158 to 209)	326 (296 to 356)	55 (40 to 74)	1 (1 to 1)	847 (785 to 912)	274 (237 to 313)	488 (444 to 533)	82 (60 to 111)	2 (2 to 2)
Maldives	2001	558 (515 to 601)	180 (155 to 207)	314 (287 to 344)	63 (47 to 85)	1 (1 to 1)	835 (771 to 901)	269 (232 to 309)	470 (429 to 515)	95 (70 to 128)	1 (1 to 1)
Maldives	2002	576 (533 to 621)	185 (161 to 212)	314 (288 to 344)	77 (57 to 102)	0 (0 to 0)	863 (799 to 930)	277 (240 to 317)	470 (432 to 515)	115 (85 to 153)	1 (1 to 1)
Maldives	2003	591 (549 to 635)	193 (168 to 219)	321 (295 to 350)	76 (57 to 99)	0 (0 to 0)	885 (822 to 951)	289 (252 to 328)	481 (442 to 524)	114 (85 to 148)	1 (1 to 1)
Maldives	2004	609 (566 to 652)	205 (179 to 231)	334 (306 to 365)	69 (51 to 92)	1 (1 to 1)	912 (847 to 976)	306 (268 to 346)	501 (458 to 546)	104 (77 to 137)	1 (1 to 1)
Maldives	2005	707 (658 to 757)	268 (237 to 299)	361 (330 to 394)	76 (56 to 101)	2 (2 to 2)	1058 (985 to 1133)	401 (355 to 447)	540 (494 to 590)	114 (84 to 152)	3 (3 to 3)

Maldives	2006	775 (724 to 829)	328 (296 to 362)	368 (338 to 401)	78 (57 to 103)	1 (1 to 1)	1161 (1084 to 1241)	492 (443 to 542)	551 (506 to 600)	117 (86 to 155)	1 (1 to 1)
Maldives	2007	860 (808 to 915)	411 (373 to 447)	373 (344 to 404)	70 (51 to 93)	6 (6 to 6)	1288 (1209 to 1370)	615 (559 to 670)	559 (515 to 604)	105 (77 to 140)	9 (9 to 9)
Maldives	2008	965 (910 to 1024)	510 (468 to 551)	376 (345 to 406)	76 (56 to 101)	2 (2 to 2)	1444 (1362 to 1533)	764 (701 to 825)	563 (517 to 609)	114 (83 to 151)	3 (3 to 3)
Maldives	2009	910 (859 to 963)	501 (463 to 539)	343 (312 to 373)	62 (45 to 82)	4 (4 to 4)	1362 (1286 to 1442)	750 (694 to 807)	513 (468 to 559)	92 (67 to 123)	7 (7 to 7)
Maldives	2010	834 (788 to 881)	454 (420 to 490)	314 (289 to 342)	63 (46 to 84)	4 (4 to 4)	1249 (1181 to 1319)	679 (628 to 733)	470 (432 to 512)	94 (69 to 126)	6 (6 to 6)
Maldives	2011	806 (759 to 856)	426 (392 to 460)	290 (265 to 318)	80 (60 to 106)	10 (10 to 10)	1207 (1137 to 1281)	638 (587 to 689)	435 (396 to 476)	120 (89 to 158)	15 (15 to 15)
Maldives	2012	794 (745 to 845)	427 (394 to 461)	264 (239 to 292)	96 (73 to 124)	7 (7 to 7)	1189 (1116 to 1265)	639 (590 to 690)	396 (357 to 437)	144 (110 to 185)	10 (10 to 10)
Maldives	2013	781 (732 to 832)	443 (410 to 478)	240 (215 to 266)	96 (73 to 123)	2 (2 to 2)	1170 (1095 to 1246)	663 (614 to 715)	360 (321 to 398)	144 (109 to 184)	4 (4 to 4)
Maldives	2014	888 (835 to 946)	556 (516 to 596)	232 (205 to 257)	99 (76 to 128)	1 (1 to 1)	1330 (1251 to 1417)	832 (773 to 893)	347 (307 to 385)	149 (114 to 192)	2 (2 to 2)
Maldives	2015	928 (873 to 986)	624 (585 to 667)	209 (183 to 234)	93 (70 to 122)	2 (2 to 2)	1390 (1307 to 1476)	935 (876 to 999)	313 (274 to 351)	139 (104 to 183)	3 (3 to 3)

Maldives	2016	1037 (974 to 1104)	729 (679 to 777)	210 (182 to 241)	97 (71 to 130)	2 (2 to 2)	1552 (1459 to 1653)	1091 (1016 to 1164)	314 (272 to 360)	145 (106 to 195)	2 (2 to 2)
Maldives	2017	988 (905 to 1081)	702 (644 to 761)	200 (170 to 234)	85 (60 to 121)	0 (0 to 0)	1479 (1355 to 1618)	1051 (964 to 1139)	300 (255 to 350)	128 (90 to 181)	0 (0 to 0)
Maldives	2018	1083 (984 to 1198)	787 (714 to 867)	205 (174 to 239)	90 (64 to 128)	0 (0 to 0)	1621 (1473 to 1793)	1179 (1069 to 1298)	307 (260 to 357)	135 (95 to 191)	0 (0 to 0)
Maldives	2019	1147 (1032 to 1275)	839 (750 to 930)	213 (181 to 248)	95 (67 to 134)	0 (0 to 0)	1717 (1545 to 1910)	1256 (1123 to 1392)	319 (271 to 372)	142 (100 to 201)	0 (0 to 0)
Maldives	2020	1206 (1076 to 1354)	885 (786 to 995)	222 (188 to 259)	99 (70 to 140)	0 (0 to 0)	1806 (1611 to 2027)	1325 (1178 to 1490)	332 (282 to 388)	149 (105 to 210)	0 (0 to 0)
Maldives	2021	1233 (1088 to 1397)	905 (789 to 1026)	226 (192 to 263)	101 (71 to 143)	0 (0 to 0)	1846 (1629 to 2091)	1356 (1181 to 1536)	338 (287 to 394)	152 (107 to 214)	0 (0 to 0)
Maldives	2022	1268 (1119 to 1437)	935 (817 to 1068)	229 (195 to 267)	103 (73 to 146)	0 (0 to 0)	1899 (1675 to 2152)	1401 (1223 to 1599)	344 (292 to 401)	155 (109 to 219)	0 (0 to 0)
Maldives	2023	1308 (1149 to 1484)	968 (839 to 1104)	233 (198 to 272)	106 (74 to 150)	0 (0 to 0)	1958 (1721 to 2222)	1449 (1257 to 1653)	349 (297 to 408)	159 (112 to 225)	0 (0 to 0)
Maldives	2024	1350 (1177 to 1533)	1003 (872 to 1146)	237 (201 to 278)	109 (77 to 154)	0 (0 to 0)	2021 (1762 to 2295)	1502 (1305 to 1716)	355 (301 to 416)	163 (115 to 231)	0 (0 to 0)
Maldives	2025	1393 (1220 to 1582)	1040 (906 to 1192)	241 (205 to 283)	112 (79 to 159)	0 (0 to 0)	2086 (1826 to 2368)	1557 (1356 to 1784)	361 (307 to 424)	167 (118 to 238)	0 (0 to 0)

Maldives	2026	1438 (1261 to 1631)	1079 (938 to 1239)	245 (208 to 288)	114 (80 to 162)	0 (0 to 0)	2153 (1888 to 2442)	1615 (1405 to 1855)	367 (312 to 432)	171 (120 to 242)	0 (0 to 0)
Maldives	2027	1484 (1302 to 1697)	1119 (973 to 1287)	249 (212 to 294)	116 (82 to 165)	0 (0 to 0)	2223 (1949 to 2541)	1675 (1457 to 1927)	373 (317 to 439)	174 (123 to 247)	0 (0 to 0)
Maldives	2028	1534 (1344 to 1752)	1162 (1010 to 1340)	254 (215 to 300)	119 (84 to 168)	0 (0 to 0)	2297 (2013 to 2624)	1739 (1513 to 2007)	380 (322 to 449)	177 (125 to 252)	0 (0 to 0)
Maldives	2029	1586 (1389 to 1820)	1206 (1048 to 1395)	259 (219 to 305)	121 (85 to 172)	0 (0 to 0)	2374 (2079 to 2725)	1806 (1569 to 2089)	387 (328 to 457)	181 (128 to 257)	0 (0 to 0)
Maldives	2030	1638 (1430 to 1884)	1251 (1083 to 1446)	263 (223 to 311)	123 (87 to 176)	0 (0 to 0)	2452 (2141 to 2821)	1873 (1622 to 2166)	394 (333 to 466)	185 (131 to 263)	0 (0 to 0)
Maldives	2031	1690 (1476 to 1942)	1296 (1120 to 1498)	268 (226 to 318)	126 (89 to 180)	0 (0 to 0)	2531 (2210 to 2908)	1941 (1678 to 2243)	401 (339 to 476)	189 (133 to 269)	0 (0 to 0)
Maldives	2032	1742 (1517 to 2013)	1341 (1158 to 1549)	273 (230 to 324)	129 (91 to 184)	0 (0 to 0)	2608 (2271 to 3015)	2007 (1734 to 2319)	408 (344 to 485)	192 (136 to 275)	0 (0 to 0)
Maldives	2033	1794 (1564 to 2061)	1385 (1192 to 1603)	277 (233 to 331)	131 (92 to 187)	0 (0 to 0)	2686 (2342 to 3087)	2074 (1784 to 2400)	415 (350 to 495)	196 (138 to 281)	0 (0 to 0)
Maldives	2034	1844 (1605 to 2137)	1429 (1226 to 1653)	282 (236 to 337)	134 (94 to 191)	0 (0 to 0)	2761 (2403 to 3199)	2139 (1836 to 2476)	422 (353 to 505)	200 (141 to 286)	0 (0 to 0)
Maldives	2035	1893 (1652 to 2181)	1471 (1258 to 1705)	286 (238 to 342)	136 (96 to 194)	0 (0 to 0)	2835 (2473 to 3265)	2202 (1884 to 2554)	429 (357 to 513)	204 (144 to 291)	0 (0 to 0)

Maldives	2036	1940 (1682 to 2238)	1511 (1293 to 1752)	291 (241 to 348)	138 (98 to 198)	0 (0 to 0)	2905 (2519 to 3352)	2263 (1936 to 2624)	435 (361 to 521)	207 (146 to 296)	0 (0 to 0)
Maldives	2037	1987 (1730 to 2300)	1552 (1326 to 1802)	295 (245 to 354)	141 (99 to 201)	0 (0 to 0)	2976 (2591 to 3444)	2323 (1986 to 2698)	441 (367 to 531)	211 (149 to 301)	0 (0 to 0)
Maldives	2038	2033 (1771 to 2358)	1591 (1357 to 1852)	299 (247 to 362)	143 (101 to 204)	0 (0 to 0)	3045 (2652 to 3531)	2382 (2032 to 2773)	448 (370 to 541)	214 (150 to 306)	0 (0 to 1)
Maldives	2039	2079 (1804 to 2419)	1630 (1387 to 1901)	303 (250 to 369)	146 (102 to 207)	0 (0 to 0)	3113 (2702 to 3621)	2440 (2077 to 2847)	454 (375 to 552)	218 (153 to 311)	0 (0 to 1)
Maldives	2040	2122 (1833 to 2467)	1666 (1417 to 1947)	307 (253 to 374)	148 (104 to 211)	0 (0 to 0)	3177 (2745 to 3695)	2495 (2122 to 2916)	460 (379 to 561)	222 (155 to 315)	0 (0 to 1)
Maldives	2041	2163 (1872 to 2514)	1701 (1445 to 1992)	311 (255 to 381)	150 (105 to 214)	0 (0 to 0)	3238 (2803 to 3764)	2547 (2164 to 2982)	466 (382 to 570)	225 (157 to 320)	0 (0 to 1)
Maldives	2042	2202 (1905 to 2558)	1734 (1470 to 2032)	315 (258 to 386)	152 (107 to 217)	0 (0 to 0)	3296 (2852 to 3831)	2596 (2202 to 3043)	472 (386 to 578)	228 (160 to 325)	0 (0 to 1)
Maldives	2043	2237 (1930 to 2602)	1764 (1493 to 2067)	319 (260 to 391)	155 (108 to 220)	0 (0 to 0)	3349 (2890 to 3896)	2641 (2236 to 3095)	477 (389 to 586)	231 (161 to 329)	0 (0 to 1)
Maldives	2044	2267 (1958 to 2632)	1789 (1514 to 2099)	322 (262 to 395)	156 (109 to 222)	0 (0 to 0)	3394 (2931 to 3941)	2678 (2267 to 3143)	482 (392 to 592)	234 (163 to 333)	0 (0 to 1)
Maldives	2045	2293 (1970 to 2667)	1810 (1532 to 2126)	324 (263 to 399)	158 (110 to 224)	0 (0 to 0)	3433 (2949 to 3994)	2711 (2294 to 3183)	486 (394 to 597)	237 (165 to 336)	0 (0 to 1)

Maldives	2046	2314 (1985 to 2688)	1828 (1546 to 2147)	327 (264 to 402)	159 (111 to 226)	0 (0 to 0)	3465 (2972 to 4025)	2737 (2315 to 3215)	489 (396 to 601)	239 (166 to 339)	0 (0 to 1)
Maldives	2047	2333 (1990 to 2713)	1843 (1558 to 2164)	329 (265 to 406)	161 (112 to 228)	0 (0 to 0)	3493 (2980 to 4063)	2760 (2332 to 3241)	492 (397 to 608)	241 (167 to 342)	0 (0 to 1)
Maldives	2048	2344 (2015 to 2730)	1852 (1564 to 2173)	330 (266 to 410)	162 (112 to 229)	0 (0 to 0)	3509 (3017 to 4088)	2773 (2342 to 3254)	494 (398 to 614)	242 (168 to 343)	0 (0 to 1)
Maldives	2049	2347 (2012 to 2723)	1854 (1565 to 2175)	331 (265 to 413)	162 (112 to 230)	0 (0 to 0)	3515 (3013 to 4078)	2777 (2344 to 3256)	495 (397 to 618)	243 (168 to 344)	0 (0 to 1)
Maldives	2050	2351 (2007 to 2747)	1856 (1567 to 2175)	331 (264 to 415)	163 (113 to 230)	0 (0 to 0)	3520 (3005 to 4113)	2780 (2346 to 3257)	496 (396 to 622)	243 (168 to 345)	0 (0 to 1)
Mali	1995	43 (33 to 54)	7 (5 to 9)	31 (23 to 42)	1 (0 to 1)	4 (4 to 4)	115 (90 to 144)	18 (14 to 24)	84 (61 to 114)	2 (1 to 3)	10 (10 to 10)
Mali	1996	36 (28 to 45)	7 (5 to 9)	25 (18 to 34)	1 (0 to 1)	3 (3 to 3)	96 (76 to 120)	19 (14 to 25)	68 (49 to 91)	2 (1 to 4)	8 (8 to 8)
Mali	1997	34 (27 to 42)	7 (5 to 9)	23 (17 to 31)	1 (0 to 1)	4 (4 to 4)	92 (74 to 113)	19 (14 to 24)	62 (44 to 82)	2 (1 to 4)	10 (10 to 10)
Mali	1998	32 (25 to 40)	7 (6 to 10)	23 (17 to 31)	1 (0 to 1)	1 (1 to 1)	86 (68 to 108)	20 (15 to 26)	61 (45 to 83)	2 (1 to 4)	3 (3 to 3)
Mali	1999	33 (26 to 40)	7 (5 to 9)	22 (16 to 29)	1 (0 to 1)	3 (3 to 3)	87 (70 to 108)	19 (15 to 25)	58 (42 to 79)	2 (1 to 3)	8 (8 to 8)
Mali	2000	33 (27 to 41)	8 (6 to 10)	22 (16 to 29)	1 (0 to 1)	3 (3 to 3)	89 (72 to 110)	20 (15 to 26)	59 (43 to 79)	2 (1 to 3)	8 (8 to 8)
Mali	2001	35 (29 to 43)	8 (6 to 10)	22 (16 to 29)	1 (0 to 1)	5 (5 to 5)	95 (78 to 115)	21 (16 to 27)	59 (43 to 77)	2 (1 to 3)	13 (13 to 13)
Mali	2002	33 (27 to 41)	8 (6 to 11)	22 (16 to 29)	1 (0 to 1)	2 (2 to 2)	90 (73 to 109)	23 (17 to 30)	59 (43 to 78)	2 (1 to 3)	6 (6 to 6)

Mali	2003	38 (31 to 46)	9 (7 to 12)	22 (16 to 30)	1 (0 to 1)	5 (5 to 5)	101 (83 to 123)	25 (19 to 33)	60 (44 to 81)	2 (1 to 4)	14 (14 to 14)
Mali	2004	38 (31 to 45)	9 (7 to 12)	22 (16 to 29)	1 (0 to 1)	5 (5 to 5)	101 (84 to 122)	25 (19 to 33)	59 (43 to 79)	2 (1 to 4)	14 (14 to 14)
Mali	2005	38 (32 to 46)	9 (7 to 11)	22 (16 to 29)	1 (0 to 1)	7 (7 to 7)	103 (86 to 123)	24 (18 to 31)	59 (43 to 78)	2 (1 to 4)	18 (18 to 18)
Mali	2006	38 (32 to 46)	8 (6 to 11)	23 (16 to 30)	1 (0 to 1)	7 (7 to 7)	103 (85 to 122)	22 (17 to 29)	61 (44 to 80)	2 (1 to 4)	18 (18 to 18)
Mali	2007	38 (32 to 45)	7 (5 to 9)	22 (16 to 29)	1 (0 to 1)	8 (8 to 8)	102 (85 to 121)	19 (14 to 24)	60 (43 to 79)	2 (1 to 4)	22 (22 to 22)
Mali	2008	37 (31 to 44)	6 (4 to 7)	21 (15 to 28)	1 (0 to 1)	10 (10 to 10)	100 (84 to 118)	15 (12 to 20)	57 (41 to 74)	2 (1 to 3)	26 (26 to 26)
Mali	2009	37 (31 to 44)	6 (4 to 8)	21 (15 to 28)	1 (0 to 1)	10 (10 to 10)	101 (84 to 119)	16 (12 to 21)	57 (40 to 76)	2 (1 to 4)	26 (26 to 26)
Mali	2010	37 (32 to 43)	5 (4 to 7)	18 (13 to 24)	1 (0 to 1)	13 (13 to 13)	101 (86 to 116)	14 (11 to 19)	49 (35 to 66)	2 (1 to 4)	35 (35 to 35)
Mali	2011	34 (29 to 39)	5 (4 to 7)	16 (11 to 21)	1 (0 to 1)	12 (12 to 12)	91 (78 to 105)	14 (10 to 18)	43 (31 to 57)	2 (1 to 4)	33 (33 to 33)
Mali	2012	28 (25 to 33)	4 (3 to 6)	13 (9 to 17)	1 (0 to 1)	11 (11 to 11)	76 (66 to 88)	11 (8 to 15)	34 (24 to 46)	2 (1 to 3)	30 (30 to 30)
Mali	2013	28 (25 to 33)	6 (4 to 7)	12 (9 to 16)	1 (0 to 1)	10 (10 to 10)	76 (66 to 88)	15 (11 to 19)	33 (24 to 44)	2 (1 to 4)	27 (27 to 27)
Mali	2014	29 (25 to 33)	6 (5 to 8)	12 (8 to 16)	1 (0 to 1)	11 (11 to 11)	78 (68 to 90)	17 (12 to 22)	31 (23 to 42)	2 (1 to 3)	28 (28 to 28)
Mali	2015	30 (26 to 35)	7 (5 to 9)	11 (8 to 16)	1 (0 to 1)	11 (11 to 11)	80 (70 to 93)	19 (14 to 25)	31 (22 to 42)	2 (1 to 4)	29 (29 to 29)
Mali	2016	31 (28 to 36)	8 (6 to 11)	11 (8 to 16)	1 (0 to 1)	11 (11 to 11)	84 (74 to 97)	22 (17 to 29)	31 (22 to 42)	2 (1 to 3)	29 (29 to 29)
Mali	2017	32 (27 to 37)	9 (7 to 12)	11 (8 to 16)	1 (0 to 1)	10 (10 to 10)	85 (73 to 100)	25 (19 to 32)	31 (22 to 43)	2 (1 to 4)	27 (27 to 27)

Mali	2018	31 (26 to 36)	9 (6 to 11)	12 (8 to 16)	1 (0 to 1)	10 (10 to 10)	82 (70 to 97)	23 (17 to 30)	31 (22 to 44)	2 (1 to 4)	26 (26 to 26)
Mali	2019	33 (28 to 39)	11 (8 to 14)	12 (8 to 17)	1 (0 to 1)	9 (9 to 10)	88 (75 to 104)	29 (21 to 38)	32 (23 to 44)	2 (1 to 4)	25 (25 to 26)
Mali	2020	33 (28 to 39)	11 (8 to 15)	12 (9 to 17)	1 (0 to 1)	9 (9 to 10)	89 (76 to 106)	30 (22 to 40)	32 (23 to 45)	2 (1 to 4)	25 (24 to 27)
Mali	2021	34 (28 to 40)	11 (8 to 15)	12 (9 to 17)	1 (0 to 1)	9 (9 to 10)	91 (76 to 108)	30 (22 to 41)	33 (23 to 46)	2 (1 to 4)	25 (24 to 27)
Mali	2022	34 (29 to 41)	12 (8 to 16)	12 (9 to 18)	1 (0 to 1)	9 (8 to 10)	92 (78 to 109)	31 (23 to 42)	33 (24 to 47)	2 (1 to 4)	25 (23 to 28)
Mali	2023	34 (29 to 41)	12 (9 to 16)	13 (9 to 18)	1 (0 to 2)	9 (8 to 10)	92 (77 to 110)	32 (23 to 42)	34 (24 to 48)	2 (1 to 4)	25 (22 to 27)
Mali	2024	35 (29 to 42)	12 (9 to 16)	13 (9 to 18)	1 (0 to 2)	9 (8 to 10)	93 (79 to 112)	32 (24 to 44)	35 (25 to 49)	2 (1 to 4)	24 (22 to 27)
Mali	2025	35 (29 to 42)	12 (9 to 17)	13 (9 to 18)	1 (0 to 2)	9 (8 to 10)	95 (79 to 114)	33 (24 to 46)	35 (25 to 50)	2 (1 to 4)	24 (21 to 27)
Mali	2026	36 (30 to 44)	13 (9 to 18)	13 (10 to 19)	1 (0 to 2)	9 (8 to 10)	96 (80 to 117)	34 (24 to 48)	36 (26 to 50)	2 (1 to 4)	23 (21 to 27)
Mali	2027	36 (30 to 44)	13 (9 to 19)	14 (10 to 19)	1 (0 to 2)	9 (8 to 10)	97 (81 to 119)	35 (25 to 50)	37 (26 to 51)	2 (1 to 4)	23 (20 to 27)
Mali	2028	37 (30 to 45)	14 (9 to 19)	14 (10 to 19)	1 (0 to 2)	8 (7 to 10)	99 (81 to 121)	36 (25 to 52)	37 (26 to 52)	3 (1 to 5)	23 (20 to 26)
Mali	2029	37 (30 to 46)	14 (9 to 20)	14 (10 to 20)	1 (0 to 2)	8 (7 to 10)	100 (82 to 123)	37 (25 to 54)	38 (27 to 53)	3 (1 to 5)	22 (19 to 26)
Mali	2030	38 (31 to 46)	14 (10 to 21)	14 (10 to 20)	1 (0 to 2)	8 (7 to 10)	102 (83 to 125)	38 (26 to 55)	39 (27 to 54)	3 (1 to 5)	22 (19 to 26)
Mali	2031	38 (31 to 48)	15 (10 to 21)	15 (10 to 21)	1 (0 to 2)	8 (7 to 10)	103 (83 to 129)	40 (26 to 57)	39 (28 to 55)	3 (1 to 5)	22 (19 to 26)
Mali	2032	39 (32 to 49)	15 (10 to 22)	15 (11 to 21)	1 (1 to 2)	8 (7 to 10)	105 (86 to 131)	41 (27 to 60)	40 (28 to 56)	3 (1 to 5)	21 (18 to 26)

Mali	2033	40 (32 to 50)	16 (10 to 23)	15 (11 to 21)	1 (1 to 2)	8 (7 to 10)	107 (85 to 133)	42 (27 to 61)	41 (29 to 57)	3 (1 to 5)	21 (18 to 26)
Mali	2034	40 (33 to 51)	16 (10 to 23)	16 (11 to 22)	1 (1 to 2)	8 (7 to 9)	108 (88 to 137)	43 (28 to 62)	42 (30 to 58)	3 (1 to 5)	21 (18 to 25)
Mali	2035	41 (32 to 52)	16 (10 to 24)	16 (11 to 22)	1 (1 to 2)	8 (6 to 9)	110 (87 to 140)	44 (28 to 65)	42 (30 to 59)	3 (1 to 5)	21 (17 to 25)
Mali	2036	42 (34 to 53)	17 (11 to 25)	16 (11 to 22)	1 (1 to 2)	8 (6 to 9)	112 (90 to 142)	45 (29 to 67)	43 (31 to 60)	3 (2 to 6)	20 (17 to 25)
Mali	2037	43 (33 to 54)	17 (11 to 26)	16 (12 to 23)	1 (1 to 2)	8 (6 to 9)	114 (89 to 145)	47 (30 to 70)	44 (31 to 62)	3 (2 to 6)	20 (17 to 25)
Mali	2038	43 (35 to 56)	18 (11 to 27)	17 (12 to 23)	1 (1 to 2)	7 (6 to 9)	116 (93 to 149)	48 (30 to 73)	45 (32 to 63)	3 (2 to 6)	20 (16 to 25)
Mali	2039	44 (34 to 57)	18 (12 to 28)	17 (12 to 24)	1 (1 to 2)	7 (6 to 9)	119 (92 to 153)	50 (31 to 75)	46 (33 to 64)	3 (2 to 6)	20 (16 to 25)
Mali	2040	45 (36 to 58)	19 (12 to 30)	17 (12 to 25)	1 (1 to 2)	7 (6 to 9)	121 (96 to 155)	51 (32 to 79)	47 (33 to 66)	3 (2 to 6)	19 (16 to 25)
Mali	2041	46 (36 to 60)	20 (12 to 31)	18 (13 to 25)	1 (1 to 2)	7 (6 to 9)	124 (96 to 162)	53 (33 to 83)	48 (34 to 68)	4 (2 to 7)	19 (15 to 24)
Mali	2042	47 (37 to 62)	20 (13 to 32)	18 (13 to 26)	1 (1 to 3)	7 (6 to 9)	126 (99 to 165)	55 (34 to 86)	49 (35 to 69)	4 (2 to 7)	19 (15 to 24)
Mali	2043	48 (37 to 63)	21 (13 to 33)	19 (13 to 26)	1 (1 to 3)	7 (5 to 9)	129 (99 to 170)	57 (35 to 89)	50 (36 to 71)	4 (2 to 7)	19 (15 to 24)
Mali	2044	49 (38 to 66)	22 (13 to 35)	19 (14 to 27)	1 (1 to 3)	7 (5 to 9)	133 (102 to 177)	59 (35 to 94)	52 (36 to 73)	4 (2 to 7)	18 (15 to 24)
Mali	2045	51 (38 to 68)	23 (14 to 36)	20 (14 to 28)	2 (1 to 3)	7 (5 to 9)	136 (102 to 181)	61 (37 to 98)	53 (37 to 74)	4 (2 to 7)	18 (14 to 24)
Mali	2046	52 (39 to 70)	23 (14 to 38)	20 (14 to 28)	2 (1 to 3)	7 (5 to 9)	139 (106 to 188)	63 (38 to 102)	54 (38 to 76)	4 (2 to 8)	18 (14 to 23)
Mali	2047	53 (40 to 71)	24 (15 to 39)	21 (15 to 29)	2 (1 to 3)	7 (5 to 9)	143 (108 to 192)	65 (39 to 106)	56 (39 to 78)	4 (2 to 8)	18 (14 to 23)

Mali	2048	55 (41 to 74)	25 (15 to 41)	21 (15 to 30)	2 (1 to 3)	6 (5 to 9)	147 (110 to 198)	68 (41 to 111)	57 (40 to 80)	4 (2 to 8)	17 (13 to 23)
Mali	2049	56 (42 to 76)	26 (16 to 43)	22 (15 to 31)	2 (1 to 3)	6 (5 to 9)	151 (112 to 205)	70 (42 to 116)	59 (41 to 82)	5 (2 to 8)	17 (13 to 23)
Mali	2050	58 (43 to 78)	27 (16 to 45)	22 (16 to 31)	2 (1 to 3)	6 (5 to 8)	155 (115 to 209)	73 (43 to 121)	60 (43 to 84)	5 (2 to 9)	17 (13 to 23)
Malta	1995	934 (873 to 998)	679 (629 to 731)	227 (196 to 262)	28 (17 to 43)	0 (0 to 0)	1436 (1343 to 1535)	1045 (967 to 1124)	349 (301 to 403)	43 (26 to 67)	0 (0 to 0)
Malta	1996	985 (934 to 1038)	716 (676 to 758)	241 (214 to 272)	28 (18 to 42)	0 (0 to 0)	1515 (1437 to 1596)	1101 (1040 to 1165)	371 (329 to 419)	43 (27 to 65)	0 (0 to 0)
Malta	1997	1048 (1001 to 1101)	763 (725 to 804)	257 (231 to 284)	28 (19 to 42)	0 (0 to 0)	1612 (1540 to 1693)	1174 (1115 to 1236)	395 (356 to 437)	43 (29 to 65)	0 (0 to 0)
Malta	1998	1107 (1060 to 1158)	799 (763 to 840)	280 (254 to 308)	27 (18 to 40)	0 (0 to 0)	1702 (1630 to 1780)	1229 (1173 to 1292)	431 (390 to 473)	42 (28 to 61)	0 (0 to 0)
Malta	1999	1159 (1112 to 1212)	828 (792 to 870)	305 (278 to 333)	25 (17 to 37)	0 (0 to 0)	1782 (1710 to 1864)	1274 (1218 to 1338)	470 (428 to 512)	38 (26 to 56)	0 (0 to 0)
Malta	2000	1233 (1184 to 1284)	877 (839 to 919)	335 (308 to 364)	22 (15 to 33)	0 (0 to 0)	1897 (1820 to 1974)	1348 (1291 to 1413)	515 (474 to 560)	34 (23 to 51)	0 (0 to 0)
Malta	2001	1299 (1249 to 1351)	911 (869 to 954)	367 (340 to 397)	21 (14 to 30)	0 (0 to 0)	1997 (1921 to 2077)	1400 (1337 to 1467)	565 (523 to 611)	32 (22 to 46)	0 (0 to 0)
Malta	2002	1396 (1345 to 1449)	975 (934 to 1019)	400 (371 to 430)	21 (14 to 30)	0 (0 to 0)	2147 (2068 to 2228)	1500 (1436 to 1566)	615 (571 to 661)	32 (22 to 46)	0 (0 to 0)

Malta	2003	1548 (1493 to 1603)	1084 (1041 to 1127)	438 (408 to 469)	26 (18 to 37)	0 (0 to 0)	2381 (2296 to 2466)	1667 (1601 to 1734)	674 (627 to 722)	40 (28 to 57)	0 (0 to 0)
Malta	2004	1567 (1512 to 1621)	1085 (1045 to 1128)	452 (423 to 484)	30 (21 to 42)	0 (0 to 0)	2410 (2325 to 2493)	1669 (1607 to 1734)	696 (651 to 744)	46 (32 to 64)	0 (0 to 0)
Malta	2005	1669 (1615 to 1722)	1148 (1103 to 1191)	487 (458 to 519)	33 (24 to 45)	0 (0 to 0)	2566 (2483 to 2649)	1766 (1697 to 1831)	749 (704 to 799)	51 (37 to 69)	0 (0 to 0)
Malta	2006	1711 (1658 to 1762)	1162 (1121 to 1203)	513 (482 to 546)	36 (26 to 47)	0 (0 to 0)	2631 (2550 to 2709)	1787 (1724 to 1850)	789 (742 to 839)	55 (40 to 73)	0 (0 to 0)
Malta	2007	1692 (1638 to 1742)	1129 (1089 to 1172)	526 (495 to 558)	37 (27 to 50)	0 (0 to 0)	2601 (2519 to 2680)	1736 (1674 to 1803)	809 (761 to 858)	57 (42 to 76)	0 (0 to 0)
Malta	2008	1702 (1649 to 1754)	1120 (1080 to 1162)	543 (512 to 575)	39 (29 to 52)	0 (0 to 0)	2617 (2536 to 2697)	1722 (1661 to 1787)	835 (788 to 884)	61 (45 to 80)	0 (0 to 0)
Malta	2009	1682 (1629 to 1735)	1094 (1052 to 1135)	547 (518 to 577)	41 (30 to 53)	0 (0 to 0)	2586 (2505 to 2668)	1682 (1617 to 1746)	841 (797 to 888)	63 (47 to 82)	0 (0 to 0)
Malta	2010	1727 (1673 to 1779)	1120 (1078 to 1162)	565 (535 to 596)	42 (31 to 55)	0 (0 to 0)	2656 (2573 to 2736)	1722 (1657 to 1786)	869 (823 to 917)	65 (48 to 85)	0 (0 to 0)
Malta	2011	1789 (1734 to 1844)	1160 (1120 to 1202)	587 (555 to 617)	43 (32 to 57)	0 (0 to 0)	2752 (2667 to 2836)	1783 (1722 to 1848)	902 (853 to 949)	66 (49 to 88)	0 (0 to 0)
Malta	2012	1916 (1861 to 1977)	1248 (1206 to 1294)	624 (591 to 655)	45 (34 to 60)	0 (0 to 0)	2947 (2862 to 3040)	1919 (1854 to 1990)	959 (909 to 1008)	69 (52 to 92)	0 (0 to 0)

Malta	2013	2050 (1993 to 2108)	1324 (1281 to 1367)	680 (648 to 715)	46 (36 to 60)	0 (0 to 0)	3153 (3066 to 3243)	2036 (1970 to 2103)	1046 (997 to 1099)	71 (55 to 93)	0 (0 to 0)
Malta	2014	2261 (2204 to 2320)	1420 (1375 to 1466)	791 (755 to 829)	50 (38 to 64)	0 (0 to 0)	3477 (3389 to 3568)	2184 (2115 to 2254)	1216 (1162 to 1274)	77 (59 to 99)	0 (0 to 0)
Malta	2015	2500 (2439 to 2565)	1552 (1505 to 1602)	893 (856 to 933)	55 (41 to 71)	0 (0 to 0)	3844 (3751 to 3945)	2386 (2315 to 2463)	1373 (1316 to 1434)	85 (64 to 110)	0 (0 to 0)
Malta	2016	2622 (2557 to 2690)	1629 (1579 to 1678)	937 (896 to 975)	57 (42 to 75)	0 (0 to 0)	4033 (3932 to 4137)	2505 (2428 to 2581)	1441 (1378 to 1499)	87 (65 to 115)	0 (0 to 0)
Malta	2017	2831 (2731 to 2939)	1778 (1708 to 1850)	991 (936 to 1045)	62 (45 to 86)	0 (0 to 0)	4353 (4199 to 4519)	2734 (2626 to 2846)	1524 (1440 to 1607)	95 (69 to 132)	0 (0 to 0)
Malta	2018	3024 (2903 to 3144)	1936 (1852 to 2019)	1024 (967 to 1081)	64 (46 to 89)	0 (0 to 0)	4651 (4465 to 4835)	2977 (2848 to 3105)	1575 (1488 to 1662)	99 (71 to 136)	0 (0 to 0)
Malta	2019	3202 (3070 to 3343)	2079 (1978 to 2180)	1056 (997 to 1114)	67 (48 to 92)	0 (0 to 0)	4924 (4722 to 5142)	3197 (3042 to 3353)	1624 (1533 to 1713)	102 (74 to 141)	0 (0 to 0)
Malta	2020	3300 (3147 to 3454)	2148 (2034 to 2268)	1084 (1022 to 1144)	69 (50 to 94)	0 (0 to 0)	5075 (4840 to 5312)	3303 (3128 to 3489)	1667 (1572 to 1759)	106 (76 to 145)	0 (0 to 0)
Malta	2021	3355 (3200 to 3525)	2186 (2061 to 2318)	1099 (1036 to 1160)	70 (51 to 96)	0 (0 to 0)	5160 (4922 to 5421)	3362 (3170 to 3565)	1690 (1594 to 1784)	108 (78 to 148)	0 (0 to 0)
Malta	2022	3440 (3268 to 3609)	2252 (2123 to 2389)	1116 (1052 to 1178)	72 (52 to 99)	0 (0 to 0)	5290 (5026 to 5550)	3463 (3264 to 3673)	1717 (1618 to 1812)	110 (80 to 152)	0 (0 to 0)

Malta	2023	3500 (3340 to 3675)	2301 (2170 to 2440)	1125 (1061 to 1187)	73 (53 to 101)	0 (0 to 0)	5382 (5137 to 5652)	3539 (3336 to 3752)	1731 (1632 to 1825)	112 (81 to 155)	0 (0 to 0)
Malta	2024	3543 (3357 to 3740)	2335 (2188 to 2502)	1134 (1070 to 1196)	74 (53 to 102)	0 (0 to 0)	5448 (5163 to 5752)	3590 (3365 to 3847)	1744 (1645 to 1840)	114 (82 to 157)	0 (0 to 0)
Malta	2025	3585 (3402 to 3790)	2369 (2214 to 2537)	1141 (1078 to 1204)	75 (54 to 104)	0 (0 to 0)	5514 (5232 to 5828)	3643 (3405 to 3901)	1755 (1658 to 1852)	115 (83 to 159)	0 (0 to 0)
Malta	2026	3647 (3449 to 3856)	2419 (2262 to 2589)	1152 (1088 to 1216)	76 (55 to 106)	0 (0 to 0)	5609 (5304 to 5930)	3720 (3478 to 3982)	1771 (1672 to 1870)	117 (85 to 162)	0 (0 to 0)
Malta	2027	3726 (3529 to 3947)	2480 (2315 to 2656)	1168 (1101 to 1233)	78 (56 to 108)	0 (0 to 0)	5730 (5428 to 6071)	3814 (3560 to 4085)	1796 (1694 to 1897)	120 (86 to 166)	0 (0 to 0)
Malta	2028	3820 (3599 to 4045)	2551 (2372 to 2738)	1189 (1121 to 1256)	80 (58 to 111)	0 (0 to 0)	5875 (5535 to 6221)	3923 (3648 to 4210)	1829 (1724 to 1932)	123 (89 to 170)	0 (0 to 0)
Malta	2029	3918 (3694 to 4158)	2623 (2428 to 2825)	1212 (1140 to 1282)	82 (59 to 114)	0 (0 to 0)	6025 (5681 to 6394)	4034 (3734 to 4345)	1864 (1754 to 1971)	126 (91 to 175)	0 (0 to 0)
Malta	2030	4020 (3768 to 4277)	2699 (2493 to 2919)	1237 (1161 to 1309)	84 (61 to 117)	0 (0 to 0)	6182 (5794 to 6577)	4150 (3834 to 4489)	1902 (1785 to 2013)	130 (93 to 179)	0 (0 to 0)
Malta	2031	4121 (3874 to 4397)	2772 (2551 to 2999)	1262 (1182 to 1337)	87 (62 to 120)	0 (0 to 0)	6337 (5957 to 6762)	4264 (3923 to 4613)	1940 (1819 to 2056)	133 (96 to 184)	0 (0 to 0)
Malta	2032	4226 (3940 to 4505)	2850 (2622 to 3094)	1287 (1207 to 1365)	89 (64 to 123)	0 (0 to 0)	6499 (6060 to 6928)	4382 (4033 to 4758)	1980 (1857 to 2100)	137 (98 to 190)	0 (0 to 0)

Malta	2033	4338 (4062 to 4643)	2931 (2684 to 3191)	1315 (1232 to 1396)	92 (66 to 127)	0 (0 to 0)	6672 (6247 to 7140)	4508 (4127 to 4907)	2023 (1894 to 2147)	141 (101 to 195)	0 (0 to 0)
Malta	2034	4450 (4134 to 4764)	3013 (2751 to 3285)	1343 (1256 to 1427)	94 (67 to 130)	0 (0 to 0)	6844 (6358 to 7327)	4633 (4231 to 5053)	2066 (1931 to 2195)	145 (104 to 201)	0 (0 to 0)
Malta	2035	4567 (4268 to 4903)	3097 (2822 to 3386)	1373 (1282 to 1460)	97 (69 to 134)	0 (0 to 0)	7023 (6564 to 7540)	4763 (4340 to 5207)	2111 (1971 to 2245)	149 (106 to 207)	0 (0 to 0)
Malta	2036	4687 (4332 to 5044)	3184 (2888 to 3490)	1403 (1308 to 1493)	99 (71 to 138)	0 (0 to 0)	7208 (6662 to 7757)	4897 (4441 to 5366)	2158 (2012 to 2296)	153 (109 to 212)	0 (0 to 0)
Malta	2037	4809 (4483 to 5184)	3273 (2955 to 3598)	1434 (1335 to 1529)	102 (73 to 142)	0 (0 to 0)	7395 (6895 to 7972)	5033 (4545 to 5533)	2205 (2053 to 2351)	157 (112 to 218)	0 (0 to 0)
Malta	2038	4930 (4524 to 5329)	3362 (3024 to 3705)	1463 (1361 to 1563)	105 (75 to 146)	0 (0 to 0)	7581 (6957 to 8196)	5170 (4651 to 5698)	2250 (2094 to 2404)	161 (115 to 224)	0 (0 to 0)
Malta	2039	5055 (4680 to 5490)	3455 (3104 to 3824)	1493 (1388 to 1600)	108 (77 to 149)	0 (0 to 0)	7775 (7197 to 8443)	5313 (4773 to 5880)	2296 (2135 to 2461)	166 (118 to 230)	0 (0 to 0)
Malta	2040	5179 (4752 to 5617)	3546 (3179 to 3933)	1522 (1413 to 1634)	110 (79 to 153)	0 (0 to 0)	7965 (7308 to 8638)	5454 (4890 to 6049)	2341 (2174 to 2512)	170 (121 to 236)	0 (0 to 0)
Malta	2041	5303 (4882 to 5777)	3638 (3260 to 4043)	1552 (1439 to 1667)	113 (81 to 157)	0 (0 to 0)	8156 (7508 to 8884)	5595 (5013 to 6217)	2387 (2213 to 2564)	174 (124 to 242)	0 (0 to 0)
Malta	2042	5429 (4950 to 5914)	3731 (3334 to 4154)	1582 (1466 to 1701)	116 (83 to 161)	0 (0 to 0)	8349 (7613 to 9094)	5738 (5127 to 6389)	2432 (2254 to 2616)	179 (127 to 248)	0 (0 to 0)

Malta	2043	5556 (5121 to 6072)	3825 (3410 to 4271)	1612 (1492 to 1735)	119 (85 to 165)	0 (0 to 0)	8545 (7875 to 9338)	5883 (5244 to 6569)	2479 (2294 to 2669)	183 (130 to 254)	0 (0 to 0)
Malta	2044	5685 (5175 to 6213)	3921 (3500 to 4389)	1642 (1516 to 1770)	122 (87 to 169)	0 (0 to 0)	8743 (7958 to 9555)	6030 (5382 to 6750)	2525 (2331 to 2722)	187 (133 to 261)	0 (0 to 0)
Malta	2045	5812 (5339 to 6375)	4016 (3572 to 4503)	1671 (1541 to 1806)	125 (89 to 174)	0 (0 to 0)	8938 (8211 to 9804)	6176 (5494 to 6925)	2570 (2370 to 2778)	192 (136 to 267)	0 (0 to 0)
Malta	2046	5938 (5392 to 6486)	4109 (3637 to 4617)	1701 (1564 to 1839)	128 (90 to 178)	0 (0 to 0)	9131 (8292 to 9975)	6320 (5594 to 7101)	2615 (2405 to 2828)	196 (139 to 273)	0 (0 to 0)
Malta	2047	6063 (5555 to 6661)	4203 (3715 to 4726)	1730 (1586 to 1872)	130 (92 to 182)	0 (0 to 0)	9325 (8543 to 10243)	6464 (5713 to 7268)	2661 (2440 to 2880)	201 (142 to 280)	0 (0 to 0)
Malta	2048	6189 (5618 to 6770)	4296 (3789 to 4834)	1759 (1608 to 1909)	133 (94 to 186)	0 (0 to 0)	9518 (8640 to 10412)	6607 (5828 to 7434)	2705 (2473 to 2936)	205 (145 to 287)	0 (0 to 0)
Malta	2049	6315 (5785 to 6951)	4390 (3867 to 4950)	1789 (1631 to 1946)	136 (96 to 190)	0 (0 to 0)	9712 (8896 to 10690)	6751 (5947 to 7613)	2751 (2508 to 2993)	209 (148 to 293)	0 (0 to 0)
Malta	2050	6444 (5841 to 7081)	4486 (3966 to 5058)	1819 (1654 to 1981)	139 (98 to 194)	0 (0 to 0)	9910 (8982 to 10890)	6898 (6100 to 7778)	2798 (2544 to 3046)	214 (151 to 299)	0 (0 to 0)
Marshall Islands	1995	330 (288 to 374)	230 (196 to 268)	65 (49 to 83)	25 (13 to 42)	10 (10 to 10)	323 (283 to 367)	225 (192 to 263)	64 (48 to 82)	24 (13 to 41)	10 (10 to 10)
Marshall Islands	1996	316 (280 to 354)	214 (186 to 247)	60 (45 to 77)	24 (13 to 40)	18 (18 to 18)	309 (275 to 347)	210 (182 to 242)	59 (44 to 75)	23 (13 to 39)	17 (17 to 17)
Marshall Islands	1997	314 (283 to 349)	214 (189 to 242)	57 (44 to 72)	23 (13 to 39)	21 (21 to 21)	308 (277 to 342)	210 (186 to 238)	55 (43 to 70)	23 (13 to 39)	20 (20 to 20)

Marshall Islands	1998	315 (286 to 346)	207 (183 to 232)	54 (42 to 68)	23 (13 to 38)	31 (31 to 31)	308 (281 to 339)	203 (179 to 227)	52 (41 to 67)	22 (13 to 37)	31 (31 to 31)
Marshall Islands	1999	399 (371 to 429)	208 (186 to 232)	52 (41 to 66)	23 (13 to 37)	116 (116 to 116)	391 (363 to 421)	204 (183 to 228)	51 (40 to 64)	22 (12 to 36)	113 (113 to 113)
Marshall Islands	2000	355 (324 to 388)	242 (217 to 269)	56 (44 to 69)	26 (15 to 41)	32 (32 to 32)	348 (318 to 381)	237 (213 to 264)	55 (43 to 68)	25 (15 to 40)	31 (31 to 31)
Marshall Islands	2001	340 (308 to 375)	245 (221 to 273)	58 (45 to 71)	27 (15 to 43)	10 (10 to 10)	334 (302 to 367)	241 (217 to 268)	57 (44 to 70)	26 (15 to 42)	10 (10 to 10)
Marshall Islands	2002	322 (293 to 353)	215 (194 to 241)	58 (45 to 72)	25 (15 to 40)	23 (23 to 23)	316 (287 to 346)	211 (190 to 237)	57 (45 to 71)	25 (15 to 39)	23 (23 to 23)
Marshall Islands	2003	336 (309 to 365)	188 (168 to 211)	62 (49 to 76)	23 (14 to 37)	64 (64 to 64)	329 (303 to 358)	184 (165 to 207)	60 (48 to 74)	23 (13 to 36)	62 (62 to 62)
Marshall Islands	2004	521 (495 to 549)	176 (159 to 199)	63 (50 to 78)	22 (13 to 36)	259 (259 to 259)	511 (485 to 539)	173 (156 to 195)	62 (49 to 76)	22 (13 to 35)	254 (254 to 254)
Marshall Islands	2005	682 (651 to 716)	216 (193 to 243)	71 (57 to 89)	27 (16 to 44)	367 (367 to 367)	668 (638 to 702)	212 (190 to 239)	70 (56 to 87)	27 (16 to 43)	360 (360 to 360)
Marshall Islands	2006	433 (407 to 460)	171 (152 to 192)	64 (51 to 80)	22 (13 to 35)	176 (176 to 176)	424 (399 to 451)	168 (149 to 189)	63 (50 to 78)	21 (13 to 34)	172 (172 to 172)
Marshall Islands	2007	471 (443 to 501)	198 (176 to 223)	68 (54 to 85)	23 (14 to 37)	181 (181 to 181)	461 (434 to 491)	194 (172 to 218)	67 (53 to 84)	23 (14 to 36)	178 (178 to 178)
Marshall Islands	2008	487 (459 to 518)	184 (163 to 206)	67 (53 to 83)	22 (13 to 35)	214 (214 to 214)	478 (450 to 508)	181 (159 to 202)	66 (52 to 82)	21 (12 to 34)	210 (210 to 210)
Marshall Islands	2009	498 (471 to 529)	179 (158 to 201)	68 (54 to 85)	21 (12 to 34)	229 (229 to 229)	488 (462 to 519)	175 (155 to 197)	67 (53 to 83)	21 (12 to 34)	225 (225 to 225)
Marshall Islands	2010	301 (275 to 332)	177 (156 to 198)	72 (57 to 89)	21 (12 to 33)	31 (31 to 31)	295 (269 to 326)	173 (153 to 195)	71 (56 to 88)	20 (11 to 33)	31 (31 to 31)
Marshall Islands	2011	321 (294 to 353)	190 (169 to 213)	75 (59 to 93)	20 (11 to 33)	37 (37 to 37)	315 (288 to 346)	186 (165 to 209)	73 (58 to 91)	20 (11 to 32)	36 (36 to 36)
Marshall Islands	2012	494 (465 to 526)	200 (179 to 225)	75 (60 to 93)	20 (11 to 32)	198 (198 to 198)	484 (456 to 515)	197 (175 to 220)	74 (59 to 91)	19 (11 to 31)	194 (194 to 194)

Marshall Islands	2013	342 (313 to 375)	220 (198 to 245)	77 (61 to 95)	20 (11 to 34)	23 (23 to 23)	335 (307 to 368)	216 (194 to 240)	76 (60 to 93)	20 (11 to 33)	23 (23 to 23)
Marshall Islands	2014	430 (401 to 463)	209 (187 to 232)	75 (60 to 92)	19 (11 to 32)	127 (127 to 127)	421 (393 to 454)	205 (184 to 228)	73 (58 to 90)	19 (10 to 31)	124 (124 to 124)
Marshall Islands	2015	410 (378 to 446)	232 (207 to 260)	77 (61 to 96)	20 (11 to 34)	81 (81 to 81)	402 (371 to 437)	227 (203 to 255)	75 (59 to 94)	20 (11 to 33)	79 (79 to 79)
Marshall Islands	2016	353 (318 to 394)	240 (212 to 271)	76 (59 to 95)	20 (11 to 34)	18 (18 to 18)	346 (312 to 386)	235 (208 to 266)	74 (58 to 94)	19 (10 to 34)	17 (17 to 17)
Marshall Islands	2017	416 (370 to 472)	259 (223 to 298)	77 (60 to 99)	21 (11 to 35)	59 (59 to 59)	408 (362 to 462)	254 (218 to 293)	76 (59 to 97)	21 (11 to 35)	58 (58 to 58)
Marshall Islands	2018	404 (356 to 459)	248 (212 to 286)	79 (61 to 101)	21 (11 to 35)	57 (57 to 57)	396 (349 to 450)	243 (208 to 280)	77 (60 to 99)	20 (10 to 34)	56 (56 to 56)
Marshall Islands	2019	438 (383 to 500)	277 (237 to 323)	80 (62 to 102)	22 (12 to 38)	58 (58 to 59)	429 (375 to 490)	272 (232 to 316)	78 (61 to 100)	22 (11 to 37)	57 (57 to 57)
Marshall Islands	2020	447 (390 to 513)	284 (242 to 330)	81 (63 to 103)	23 (12 to 38)	59 (56 to 63)	438 (383 to 503)	278 (238 to 324)	79 (62 to 101)	22 (12 to 37)	58 (55 to 61)
Marshall Islands	2021	458 (399 to 530)	292 (248 to 343)	82 (64 to 105)	23 (12 to 39)	60 (57 to 65)	449 (391 to 520)	287 (243 to 336)	80 (62 to 103)	23 (12 to 38)	59 (56 to 64)
Marshall Islands	2022	468 (404 to 539)	300 (252 to 353)	83 (64 to 106)	24 (12 to 40)	61 (56 to 68)	459 (397 to 529)	294 (247 to 346)	81 (63 to 104)	23 (12 to 39)	60 (55 to 66)
Marshall Islands	2023	478 (413 to 551)	308 (258 to 365)	84 (65 to 108)	24 (13 to 41)	62 (56 to 69)	469 (405 to 540)	302 (253 to 358)	82 (64 to 105)	24 (12 to 40)	61 (55 to 68)
Marshall Islands	2024	489 (419 to 568)	317 (266 to 377)	85 (66 to 109)	25 (13 to 42)	62 (56 to 70)	479 (411 to 557)	311 (260 to 369)	83 (65 to 107)	24 (13 to 41)	61 (55 to 69)
Marshall Islands	2025	499 (427 to 580)	325 (271 to 391)	86 (67 to 111)	25 (13 to 42)	63 (56 to 71)	490 (419 to 569)	319 (265 to 383)	84 (66 to 108)	25 (13 to 42)	62 (55 to 70)
Marshall Islands	2026	510 (436 to 597)	334 (278 to 401)	87 (68 to 112)	26 (13 to 43)	63 (56 to 72)	500 (427 to 585)	327 (272 to 393)	85 (66 to 110)	25 (13 to 42)	62 (55 to 71)
Marshall Islands	2027	521 (442 to 611)	343 (281 to 414)	88 (68 to 113)	26 (14 to 44)	64 (56 to 74)	511 (434 to 599)	336 (276 to 405)	86 (67 to 111)	26 (13 to 43)	63 (55 to 73)

Marshall Islands	2028	533 (453 to 628)	353 (291 to 428)	89 (69 to 115)	27 (14 to 45)	65 (56 to 75)	523 (444 to 616)	346 (286 to 420)	87 (68 to 112)	26 (14 to 44)	63 (55 to 74)
Marshall Islands	2029	544 (462 to 640)	361 (294 to 438)	90 (70 to 116)	27 (14 to 46)	65 (56 to 76)	533 (453 to 627)	354 (288 to 429)	89 (69 to 114)	27 (14 to 45)	64 (55 to 75)
Marshall Islands	2030	554 (468 to 648)	370 (302 to 447)	91 (71 to 118)	28 (14 to 47)	66 (56 to 79)	544 (459 to 636)	362 (296 to 439)	90 (69 to 115)	27 (14 to 46)	65 (55 to 77)
Marshall Islands	2031	565 (477 to 664)	378 (308 to 458)	92 (72 to 119)	28 (15 to 47)	67 (57 to 79)	554 (468 to 651)	370 (302 to 449)	91 (70 to 116)	28 (14 to 46)	65 (56 to 78)
Marshall Islands	2032	575 (484 to 677)	386 (310 to 468)	94 (72 to 120)	29 (15 to 48)	67 (57 to 82)	564 (475 to 664)	378 (304 to 459)	92 (71 to 118)	28 (15 to 47)	66 (56 to 80)
Marshall Islands	2033	585 (490 to 688)	394 (315 to 478)	94 (73 to 121)	29 (15 to 49)	68 (58 to 83)	574 (480 to 674)	386 (309 to 469)	93 (71 to 119)	29 (15 to 48)	67 (57 to 81)
Marshall Islands	2034	595 (494 to 702)	401 (321 to 494)	95 (73 to 123)	30 (15 to 50)	69 (58 to 84)	584 (485 to 688)	393 (315 to 485)	93 (72 to 120)	29 (15 to 49)	68 (57 to 82)
Marshall Islands	2035	605 (502 to 716)	409 (328 to 499)	96 (74 to 124)	30 (16 to 51)	70 (58 to 85)	594 (492 to 701)	401 (322 to 490)	94 (73 to 121)	30 (15 to 50)	69 (57 to 84)
Marshall Islands	2036	616 (509 to 729)	417 (333 to 507)	97 (75 to 125)	31 (16 to 52)	71 (59 to 89)	604 (499 to 714)	409 (327 to 497)	95 (73 to 122)	30 (16 to 51)	69 (57 to 87)
Marshall Islands	2037	626 (521 to 739)	425 (337 to 520)	98 (75 to 126)	31 (16 to 53)	72 (59 to 88)	613 (511 to 724)	416 (330 to 510)	96 (74 to 124)	31 (16 to 52)	70 (58 to 86)
Marshall Islands	2038	636 (527 to 756)	433 (341 to 530)	99 (76 to 127)	32 (16 to 54)	73 (60 to 91)	624 (517 to 741)	424 (334 to 519)	97 (75 to 125)	31 (16 to 53)	71 (59 to 90)
Marshall Islands	2039	646 (535 to 769)	441 (347 to 541)	100 (77 to 128)	32 (17 to 55)	74 (61 to 93)	633 (525 to 754)	432 (340 to 530)	98 (75 to 126)	31 (16 to 54)	72 (59 to 91)
Marshall Islands	2040	657 (541 to 782)	449 (352 to 552)	101 (77 to 129)	33 (17 to 56)	75 (61 to 95)	644 (530 to 767)	440 (345 to 541)	99 (76 to 127)	32 (17 to 55)	73 (60 to 93)
Marshall Islands	2041	668 (551 to 797)	458 (360 to 562)	102 (78 to 130)	33 (17 to 57)	76 (62 to 96)	655 (540 to 782)	449 (353 to 551)	100 (76 to 128)	32 (17 to 55)	74 (61 to 94)
Marshall Islands	2042	680 (564 to 810)	467 (369 to 574)	102 (78 to 132)	34 (17 to 57)	77 (63 to 98)	666 (553 to 794)	458 (362 to 563)	100 (77 to 129)	33 (17 to 56)	75 (61 to 96)

Marshall Islands	2043	692 (569 to 831)	476 (374 to 589)	103 (79 to 133)	34 (18 to 58)	78 (63 to 102)	678 (557 to 814)	467 (366 to 577)	101 (77 to 130)	33 (17 to 57)	77 (62 to 100)
Marshall Islands	2044	704 (582 to 850)	486 (382 to 608)	104 (80 to 134)	34 (18 to 59)	79 (64 to 105)	690 (571 to 833)	477 (374 to 596)	102 (78 to 131)	34 (18 to 58)	78 (63 to 103)
Marshall Islands	2045	717 (597 to 860)	497 (389 to 616)	105 (80 to 135)	35 (18 to 60)	81 (64 to 103)	703 (585 to 843)	487 (382 to 604)	103 (79 to 132)	34 (18 to 59)	79 (63 to 101)
Marshall Islands	2046	731 (607 to 885)	507 (399 to 631)	106 (81 to 136)	35 (18 to 61)	82 (65 to 105)	716 (595 to 868)	497 (391 to 619)	104 (79 to 134)	35 (18 to 60)	80 (64 to 103)
Marshall Islands	2047	745 (614 to 906)	519 (408 to 650)	107 (82 to 138)	36 (19 to 62)	83 (66 to 109)	730 (602 to 888)	508 (400 to 637)	105 (80 to 135)	35 (18 to 61)	82 (65 to 107)
Marshall Islands	2048	758 (624 to 927)	529 (416 to 665)	108 (83 to 139)	37 (19 to 63)	84 (67 to 112)	743 (611 to 909)	519 (408 to 652)	106 (81 to 136)	36 (19 to 61)	83 (66 to 110)
Marshall Islands	2049	772 (633 to 943)	541 (422 to 680)	109 (84 to 140)	37 (19 to 64)	86 (68 to 113)	757 (620 to 925)	530 (414 to 667)	107 (82 to 138)	36 (19 to 62)	84 (67 to 111)
Marshall Islands	2050	787 (642 to 951)	552 (430 to 694)	110 (84 to 142)	38 (20 to 65)	87 (68 to 118)	771 (630 to 932)	541 (422 to 680)	108 (83 to 139)	37 (19 to 63)	85 (67 to 115)
Mauritania	1995	55 (45 to 68)	11 (9 to 15)	34 (24 to 47)	2 (1 to 4)	8 (8 to 8)	192 (157 to 238)	39 (30 to 51)	119 (85 to 164)	7 (3 to 13)	27 (27 to 27)
Mauritania	1996	57 (47 to 70)	12 (9 to 15)	35 (25 to 47)	2 (1 to 4)	8 (8 to 8)	198 (164 to 244)	41 (31 to 52)	122 (89 to 166)	7 (3 to 14)	29 (29 to 29)
Mauritania	1997	51 (42 to 63)	11 (8 to 14)	34 (25 to 45)	2 (1 to 4)	4 (4 to 4)	178 (145 to 219)	38 (29 to 49)	117 (86 to 159)	7 (3 to 13)	16 (16 to 16)
Mauritania	1998	51 (42 to 62)	10 (8 to 14)	34 (25 to 46)	2 (1 to 4)	5 (5 to 5)	178 (145 to 218)	36 (28 to 47)	119 (87 to 160)	7 (3 to 13)	16 (16 to 16)
Mauritania	1999	49 (39 to 61)	10 (7 to 13)	34 (25 to 46)	2 (1 to 4)	4 (4 to 4)	172 (137 to 212)	34 (25 to 44)	119 (87 to 159)	7 (3 to 13)	13 (13 to 13)
Mauritania	2000	49 (40 to 61)	8 (6 to 11)	33 (24 to 45)	2 (1 to 3)	6 (6 to 6)	172 (138 to 214)	29 (22 to 38)	116 (84 to 157)	6 (3 to 12)	21 (21 to 21)
Mauritania	2001	49 (39 to 61)	9 (7 to 11)	34 (25 to 46)	2 (1 to 3)	5 (5 to 5)	170 (136 to 212)	30 (23 to 39)	118 (87 to 161)	6 (3 to 11)	16 (16 to 16)

Mauritania	2002	51 (41 to 63)	9 (7 to 12)	34 (25 to 47)	2 (1 to 3)	6 (6 to 6)	178 (144 to 220)	33 (25 to 43)	119 (87 to 162)	6 (3 to 11)	19 (19 to 19)
Mauritania	2003	55 (45 to 67)	11 (8 to 14)	35 (25 to 47)	2 (1 to 3)	8 (8 to 8)	193 (157 to 235)	37 (28 to 49)	122 (89 to 165)	7 (3 to 12)	27 (27 to 27)
Mauritania	2004	51 (41 to 62)	10 (8 to 13)	32 (24 to 43)	2 (1 to 3)	7 (7 to 7)	177 (144 to 216)	35 (26 to 46)	113 (83 to 150)	6 (3 to 11)	23 (23 to 23)
Mauritania	2005	47 (37 to 57)	11 (8 to 14)	31 (23 to 42)	2 (1 to 3)	3 (3 to 3)	163 (131 to 201)	37 (28 to 49)	110 (80 to 147)	6 (3 to 11)	10 (10 to 10)
Mauritania	2006	47 (39 to 58)	11 (9 to 15)	31 (22 to 41)	2 (1 to 3)	4 (4 to 4)	165 (135 to 202)	39 (30 to 51)	107 (78 to 142)	7 (3 to 12)	13 (13 to 13)
Mauritania	2007	50 (42 to 60)	12 (9 to 16)	30 (22 to 40)	2 (1 to 4)	6 (6 to 6)	175 (145 to 211)	42 (32 to 56)	105 (77 to 139)	7 (3 to 13)	21 (21 to 21)
Mauritania	2008	50 (42 to 60)	13 (10 to 17)	29 (21 to 39)	2 (1 to 4)	6 (6 to 6)	176 (146 to 211)	44 (34 to 59)	102 (75 to 135)	7 (3 to 13)	22 (22 to 22)
Mauritania	2009	47 (39 to 56)	12 (9 to 16)	28 (20 to 37)	2 (1 to 4)	5 (5 to 5)	163 (135 to 196)	43 (33 to 57)	97 (71 to 128)	7 (3 to 12)	16 (16 to 16)
Mauritania	2010	44 (37 to 53)	12 (9 to 16)	26 (19 to 35)	2 (1 to 4)	4 (4 to 4)	155 (128 to 184)	43 (33 to 56)	90 (66 to 121)	7 (3 to 13)	15 (15 to 15)
Mauritania	2011	44 (36 to 52)	13 (10 to 17)	25 (18 to 33)	2 (1 to 4)	4 (4 to 4)	152 (127 to 182)	45 (35 to 59)	87 (63 to 116)	7 (4 to 14)	13 (13 to 13)
Mauritania	2012	47 (38 to 56)	16 (12 to 20)	26 (19 to 34)	3 (1 to 5)	3 (3 to 3)	163 (134 to 197)	55 (42 to 71)	91 (67 to 120)	9 (4 to 16)	9 (9 to 9)
Mauritania	2013	50 (41 to 60)	17 (13 to 22)	26 (19 to 34)	3 (1 to 5)	5 (5 to 5)	174 (145 to 209)	59 (45 to 76)	90 (66 to 120)	9 (4 to 16)	17 (17 to 17)
Mauritania	2014	56 (47 to 67)	20 (15 to 26)	28 (20 to 37)	3 (2 to 6)	5 (5 to 5)	196 (163 to 234)	69 (53 to 90)	98 (71 to 130)	11 (5 to 20)	17 (17 to 17)
Mauritania	2015	60 (50 to 72)	22 (17 to 28)	30 (21 to 39)	4 (2 to 7)	5 (5 to 5)	211 (176 to 252)	78 (60 to 99)	103 (75 to 138)	12 (6 to 23)	17 (17 to 17)
Mauritania	2016	58 (48 to 69)	21 (16 to 27)	29 (21 to 38)	3 (2 to 6)	5 (5 to 5)	202 (169 to 242)	73 (56 to 94)	100 (74 to 134)	11 (5 to 20)	17 (17 to 17)

Mauritania	2017	61 (50 to 74)	22 (17 to 28)	29 (21 to 39)	3 (2 to 6)	7 (7 to 7)	213 (175 to 257)	75 (58 to 97)	101 (74 to 137)	11 (5 to 20)	25 (25 to 25)
Mauritania	2018	61 (50 to 75)	22 (16 to 28)	29 (22 to 40)	3 (2 to 6)	7 (7 to 7)	213 (173 to 263)	75 (58 to 97)	102 (75 to 139)	11 (5 to 21)	24 (24 to 24)
Mauritania	2019	63 (51 to 76)	22 (17 to 29)	30 (22 to 41)	3 (2 to 6)	7 (7 to 7)	219 (179 to 267)	77 (59 to 100)	106 (78 to 144)	11 (6 to 21)	24 (24 to 24)
Mauritania	2020	65 (52 to 79)	23 (17 to 30)	31 (23 to 43)	3 (2 to 6)	7 (7 to 7)	226 (183 to 278)	80 (61 to 104)	110 (81 to 149)	12 (6 to 22)	24 (23 to 25)
Mauritania	2021	66 (54 to 81)	24 (18 to 31)	32 (24 to 43)	3 (2 to 6)	7 (6 to 7)	230 (187 to 282)	82 (62 to 107)	112 (82 to 152)	12 (6 to 23)	24 (23 to 26)
Mauritania	2022	67 (55 to 82)	24 (18 to 32)	33 (24 to 44)	4 (2 to 7)	7 (6 to 8)	235 (191 to 287)	84 (63 to 110)	114 (84 to 155)	12 (6 to 23)	24 (22 to 26)
Mauritania	2023	69 (56 to 84)	25 (19 to 32)	33 (25 to 45)	4 (2 to 7)	7 (6 to 8)	239 (195 to 293)	87 (65 to 113)	116 (86 to 158)	13 (6 to 24)	24 (21 to 26)
Mauritania	2024	70 (57 to 86)	25 (19 to 34)	34 (25 to 46)	4 (2 to 7)	7 (6 to 8)	245 (198 to 300)	89 (67 to 117)	119 (88 to 161)	13 (6 to 24)	24 (21 to 27)
Mauritania	2025	72 (58 to 88)	26 (20 to 34)	35 (26 to 47)	4 (2 to 7)	7 (6 to 8)	250 (203 to 306)	92 (69 to 120)	122 (90 to 165)	13 (6 to 25)	23 (21 to 27)
Mauritania	2026	73 (59 to 90)	27 (20 to 35)	36 (26 to 48)	4 (2 to 7)	7 (6 to 8)	255 (208 to 315)	94 (70 to 123)	124 (92 to 169)	14 (7 to 26)	23 (20 to 26)
Mauritania	2027	75 (60 to 92)	28 (21 to 36)	36 (27 to 50)	4 (2 to 8)	7 (6 to 8)	261 (211 to 322)	97 (72 to 127)	127 (94 to 173)	14 (7 to 26)	23 (20 to 27)
Mauritania	2028	76 (62 to 95)	28 (21 to 37)	37 (28 to 51)	4 (2 to 8)	7 (6 to 8)	267 (216 to 332)	99 (74 to 131)	130 (96 to 177)	14 (7 to 27)	23 (20 to 27)
Mauritania	2029	78 (63 to 97)	29 (22 to 39)	38 (28 to 52)	4 (2 to 8)	7 (6 to 8)	273 (222 to 339)	102 (76 to 137)	133 (98 to 180)	15 (7 to 28)	23 (20 to 27)
Mauritania	2030	80 (65 to 99)	30 (22 to 40)	39 (29 to 53)	4 (2 to 8)	6 (6 to 8)	279 (226 to 345)	105 (78 to 141)	136 (101 to 184)	15 (8 to 29)	23 (19 to 27)
Mauritania	2031	82 (66 to 101)	31 (23 to 41)	40 (29 to 54)	4 (2 to 8)	6 (6 to 8)	285 (230 to 354)	108 (79 to 145)	139 (103 to 188)	16 (8 to 29)	23 (19 to 27)

Mauritania	2032	83 (67 to 104)	32 (23 to 43)	41 (30 to 55)	5 (2 to 9)	6 (5 to 8)	291 (233 to 362)	111 (81 to 149)	142 (105 to 192)	16 (8 to 30)	23 (19 to 27)
Mauritania	2033	85 (68 to 105)	32 (24 to 44)	41 (31 to 56)	5 (2 to 9)	6 (6 to 8)	297 (238 to 366)	113 (83 to 154)	144 (107 to 196)	16 (8 to 31)	23 (19 to 27)
Mauritania	2034	87 (69 to 108)	33 (24 to 45)	42 (31 to 57)	5 (2 to 9)	6 (5 to 8)	302 (242 to 379)	116 (84 to 158)	147 (109 to 200)	17 (8 to 32)	23 (19 to 27)
Mauritania	2035	88 (70 to 109)	34 (25 to 47)	43 (32 to 58)	5 (2 to 9)	6 (5 to 8)	308 (245 to 380)	119 (86 to 164)	150 (111 to 203)	17 (8 to 33)	22 (19 to 27)
Mauritania	2036	90 (72 to 113)	35 (25 to 48)	44 (32 to 59)	5 (2 to 10)	6 (5 to 8)	314 (250 to 395)	121 (88 to 167)	152 (113 to 207)	18 (9 to 33)	22 (19 to 28)
Mauritania	2037	91 (73 to 114)	36 (26 to 49)	44 (33 to 60)	5 (3 to 10)	6 (5 to 8)	320 (254 to 397)	124 (90 to 172)	155 (114 to 211)	18 (9 to 34)	23 (19 to 27)
Mauritania	2038	93 (74 to 117)	36 (26 to 50)	45 (33 to 61)	5 (3 to 10)	6 (5 to 8)	326 (258 to 410)	127 (91 to 173)	158 (116 to 215)	18 (9 to 35)	23 (19 to 28)
Mauritania	2039	95 (75 to 118)	37 (27 to 51)	46 (34 to 63)	5 (3 to 10)	6 (5 to 8)	332 (263 to 411)	130 (93 to 179)	160 (118 to 219)	19 (9 to 36)	23 (18 to 29)
Mauritania	2040	97 (76 to 122)	38 (27 to 53)	47 (34 to 64)	5 (3 to 10)	6 (5 to 8)	338 (265 to 427)	133 (94 to 185)	163 (119 to 223)	19 (9 to 36)	23 (18 to 29)
Mauritania	2041	98 (78 to 123)	39 (27 to 54)	48 (35 to 65)	6 (3 to 11)	6 (5 to 8)	344 (273 to 429)	135 (95 to 189)	166 (121 to 227)	20 (10 to 37)	23 (18 to 29)
Mauritania	2042	100 (79 to 128)	40 (28 to 55)	48 (35 to 66)	6 (3 to 11)	6 (5 to 8)	350 (277 to 446)	138 (97 to 193)	169 (123 to 230)	20 (10 to 38)	23 (18 to 29)
Mauritania	2043	102 (81 to 129)	40 (28 to 56)	49 (36 to 67)	6 (3 to 11)	7 (5 to 9)	357 (282 to 452)	141 (99 to 196)	172 (126 to 234)	20 (10 to 39)	23 (18 to 30)
Mauritania	2044	104 (82 to 132)	41 (29 to 58)	50 (37 to 68)	6 (3 to 11)	7 (5 to 9)	363 (285 to 461)	144 (102 to 203)	175 (128 to 238)	21 (10 to 40)	23 (18 to 30)
Mauritania	2045	106 (83 to 134)	42 (30 to 59)	51 (37 to 69)	6 (3 to 12)	7 (5 to 8)	370 (290 to 467)	148 (103 to 208)	178 (130 to 242)	21 (10 to 41)	23 (18 to 29)
Mauritania	2046	108 (84 to 136)	43 (30 to 61)	52 (38 to 71)	6 (3 to 12)	7 (5 to 8)	378 (294 to 475)	151 (105 to 213)	182 (132 to 247)	22 (11 to 41)	23 (18 to 30)

Mauritania	2047	110 (86 to 140)	44 (31 to 63)	53 (38 to 72)	6 (3 to 12)	7 (5 to 9)	385 (301 to 489)	154 (107 to 221)	185 (134 to 252)	22 (11 to 42)	23 (18 to 30)
Mauritania	2048	112 (88 to 142)	45 (31 to 64)	54 (39 to 73)	7 (3 to 12)	7 (5 to 9)	392 (308 to 496)	158 (109 to 225)	189 (137 to 256)	23 (11 to 43)	23 (18 to 31)
Mauritania	2049	114 (89 to 147)	46 (32 to 66)	55 (40 to 75)	7 (3 to 13)	7 (5 to 9)	400 (311 to 514)	161 (111 to 229)	192 (139 to 261)	23 (11 to 44)	23 (18 to 31)
Mauritania	2050	117 (92 to 147)	47 (33 to 67)	56 (40 to 76)	7 (3 to 13)	7 (5 to 9)	408 (322 to 515)	165 (115 to 235)	195 (141 to 266)	24 (12 to 45)	23 (18 to 32)
Mauritius	1995	123 (108 to 141)	67 (56 to 80)	50 (39 to 63)	6 (3 to 11)	0 (0 to 0)	265 (232 to 305)	144 (121 to 173)	108 (85 to 136)	13 (7 to 23)	0 (0 to 0)
Mauritius	1996	132 (117 to 151)	71 (60 to 84)	52 (42 to 64)	6 (3 to 11)	2 (2 to 2)	285 (252 to 326)	154 (130 to 181)	113 (91 to 138)	14 (7 to 23)	5 (5 to 5)
Mauritius	1997	134 (119 to 152)	74 (63 to 87)	54 (45 to 65)	6 (3 to 11)	0 (0 to 0)	290 (258 to 328)	159 (136 to 187)	117 (96 to 140)	13 (7 to 23)	1 (1 to 1)
Mauritius	1998	141 (126 to 158)	78 (67 to 91)	57 (48 to 68)	6 (3 to 10)	0 (0 to 0)	305 (273 to 341)	168 (144 to 196)	124 (104 to 147)	12 (6 to 22)	1 (1 to 1)
Mauritius	1999	147 (132 to 163)	81 (69 to 93)	61 (52 to 71)	5 (3 to 9)	0 (0 to 0)	318 (286 to 352)	174 (150 to 201)	132 (112 to 154)	11 (6 to 19)	0 (0 to 0)
Mauritius	2000	165 (150 to 182)	90 (78 to 104)	70 (60 to 80)	5 (2 to 8)	0 (0 to 0)	356 (323 to 393)	195 (169 to 224)	151 (129 to 173)	10 (5 to 17)	0 (0 to 0)
Mauritius	2001	176 (160 to 193)	95 (83 to 108)	77 (66 to 88)	4 (2 to 7)	0 (0 to 0)	380 (345 to 416)	205 (179 to 233)	166 (142 to 190)	9 (5 to 16)	0 (0 to 0)
Mauritius	2002	191 (174 to 209)	102 (89 to 115)	85 (74 to 98)	4 (2 to 8)	0 (0 to 0)	414 (376 to 452)	220 (192 to 248)	184 (160 to 211)	10 (5 to 16)	0 (0 to 0)
Mauritius	2003	206 (189 to 226)	107 (95 to 121)	94 (82 to 108)	5 (2 to 8)	0 (0 to 0)	446 (407 to 488)	232 (204 to 262)	203 (177 to 234)	10 (5 to 18)	0 (0 to 0)
Mauritius	2004	220 (200 to 240)	111 (97 to 125)	104 (91 to 119)	5 (3 to 9)	0 (0 to 0)	476 (432 to 519)	240 (210 to 271)	225 (196 to 257)	11 (6 to 20)	1 (1 to 1)
Mauritius	2005	232 (211 to 254)	110 (97 to 124)	117 (101 to 133)	5 (3 to 9)	0 (0 to 0)	502 (456 to 548)	238 (209 to 268)	252 (219 to 286)	11 (6 to 21)	1 (1 to 1)

Mauritius	2006	247 (226 to 268)	110 (97 to 124)	131 (116 to 148)	5 (3 to 10)	1 (1 to 1)	533 (489 to 579)	237 (209 to 267)	283 (250 to 319)	12 (6 to 21)	1 (1 to 1)
Mauritius	2007	267 (246 to 288)	112 (99 to 126)	148 (132 to 165)	6 (3 to 10)	1 (1 to 1)	576 (531 to 623)	242 (214 to 271)	320 (285 to 357)	13 (7 to 22)	2 (2 to 2)
Mauritius	2008	297 (273 to 320)	120 (107 to 134)	170 (152 to 189)	6 (3 to 11)	0 (0 to 0)	641 (590 to 691)	260 (230 to 289)	368 (329 to 409)	14 (7 to 24)	0 (0 to 0)
Mauritius	2009	332 (307 to 357)	136 (121 to 152)	188 (169 to 209)	7 (4 to 12)	1 (1 to 1)	718 (663 to 772)	295 (261 to 329)	406 (366 to 451)	16 (9 to 27)	2 (2 to 2)
Mauritius	2010	352 (325 to 379)	147 (130 to 164)	195 (175 to 216)	8 (4 to 13)	2 (2 to 2)	760 (702 to 819)	318 (281 to 355)	421 (378 to 467)	17 (9 to 28)	4 (4 to 4)
Mauritius	2011	371 (344 to 397)	158 (141 to 176)	202 (182 to 223)	9 (5 to 14)	2 (2 to 2)	801 (744 to 859)	342 (304 to 379)	436 (392 to 481)	19 (11 to 31)	3 (3 to 3)
Mauritius	2012	389 (361 to 415)	167 (150 to 185)	209 (188 to 230)	11 (6 to 17)	2 (2 to 2)	839 (780 to 897)	361 (324 to 400)	451 (406 to 498)	23 (13 to 36)	4 (4 to 4)
Mauritius	2013	438 (408 to 470)	191 (172 to 213)	230 (208 to 253)	16 (9 to 24)	2 (2 to 2)	947 (881 to 1015)	412 (371 to 460)	497 (450 to 546)	34 (19 to 52)	4 (4 to 4)
Mauritius	2014	481 (449 to 514)	210 (189 to 232)	248 (226 to 273)	21 (12 to 33)	2 (2 to 2)	1039 (969 to 1111)	453 (409 to 500)	537 (488 to 590)	46 (27 to 72)	4 (4 to 4)
Mauritius	2015	534 (497 to 571)	234 (211 to 258)	269 (243 to 295)	30 (18 to 47)	1 (1 to 1)	1153 (1074 to 1234)	506 (457 to 558)	581 (525 to 638)	65 (39 to 100)	1 (1 to 1)
Mauritius	2016	575 (533 to 620)	253 (225 to 283)	283 (254 to 314)	38 (23 to 60)	1 (1 to 1)	1243 (1150 to 1340)	547 (486 to 611)	611 (548 to 679)	82 (50 to 129)	2 (2 to 2)
Mauritius	2017	606 (541 to 677)	265 (231 to 300)	294 (259 to 335)	43 (26 to 68)	3 (3 to 3)	1309 (1170 to 1462)	573 (500 to 647)	636 (560 to 723)	94 (56 to 147)	6 (6 to 6)
Mauritius	2018	643 (574 to 719)	287 (250 to 324)	306 (270 to 349)	46 (27 to 72)	3 (3 to 3)	1388 (1241 to 1552)	621 (540 to 700)	662 (583 to 753)	100 (59 to 155)	6 (6 to 6)

Mauritius	2019	669 (601 to 747)	301 (262 to 341)	318 (280 to 362)	48 (29 to 75)	3 (3 to 3)	1446 (1297 to 1615)	650 (566 to 737)	686 (605 to 782)	104 (62 to 162)	6 (6 to 6)
Mauritius	2020	704 (626 to 786)	320 (276 to 366)	330 (291 to 376)	51 (30 to 79)	3 (3 to 3)	1520 (1353 to 1698)	691 (596 to 790)	714 (629 to 813)	109 (65 to 171)	6 (6 to 7)
Mauritius	2021	730 (650 to 826)	336 (290 to 385)	339 (298 to 386)	53 (31 to 82)	3 (3 to 3)	1578 (1404 to 1784)	726 (627 to 831)	732 (644 to 833)	114 (68 to 177)	6 (6 to 7)
Mauritius	2022	756 (673 to 851)	352 (304 to 402)	347 (305 to 394)	55 (32 to 85)	3 (3 to 3)	1633 (1455 to 1837)	760 (657 to 869)	749 (658 to 852)	118 (70 to 184)	7 (6 to 7)
Mauritius	2023	783 (697 to 880)	368 (318 to 422)	354 (311 to 404)	57 (34 to 89)	3 (3 to 3)	1690 (1506 to 1900)	796 (687 to 911)	765 (673 to 872)	122 (73 to 191)	7 (6 to 7)
Mauritius	2024	809 (718 to 910)	386 (333 to 442)	362 (318 to 413)	59 (35 to 92)	3 (3 to 4)	1748 (1550 to 1965)	833 (720 to 956)	782 (688 to 892)	127 (75 to 198)	7 (6 to 8)
Mauritius	2025	836 (745 to 943)	403 (347 to 465)	369 (325 to 421)	61 (36 to 95)	3 (3 to 4)	1806 (1609 to 2037)	870 (750 to 1004)	798 (702 to 910)	131 (78 to 205)	7 (6 to 8)
Mauritius	2026	863 (766 to 977)	420 (360 to 485)	377 (330 to 429)	63 (37 to 98)	3 (3 to 4)	1864 (1654 to 2111)	907 (778 to 1048)	814 (714 to 928)	136 (81 to 212)	7 (6 to 8)
Mauritius	2027	890 (793 to 1004)	437 (376 to 505)	384 (337 to 438)	65 (38 to 101)	3 (3 to 4)	1922 (1713 to 2169)	945 (811 to 1091)	830 (729 to 946)	140 (83 to 219)	7 (6 to 8)
Mauritius	2028	916 (811 to 1036)	455 (390 to 529)	391 (343 to 446)	67 (40 to 104)	3 (3 to 4)	1978 (1752 to 2237)	982 (844 to 1143)	845 (740 to 964)	144 (86 to 225)	7 (6 to 9)

Mauritius	2029	942 (833 to 1070)	472 (405 to 553)	398 (348 to 453)	69 (41 to 107)	3 (0 to 4)	2036 (1800 to 2312)	1020 (876 to 1195)	859 (752 to 979)	149 (88 to 232)	7 (0 to 9)
Mauritius	2030	969 (855 to 1106)	491 (420 to 574)	404 (354 to 461)	71 (42 to 110)	3 (0 to 4)	2094 (1846 to 2389)	1060 (908 to 1239)	874 (764 to 996)	153 (91 to 238)	7 (0 to 9)
Mauritius	2031	996 (878 to 1136)	509 (434 to 596)	411 (358 to 469)	73 (43 to 113)	3 (0 to 4)	2151 (1897 to 2453)	1099 (937 to 1287)	888 (774 to 1014)	158 (94 to 245)	6 (0 to 9)
Mauritius	2032	1022 (892 to 1173)	526 (447 to 619)	418 (363 to 478)	75 (45 to 117)	3 (0 to 4)	2208 (1926 to 2533)	1137 (965 to 1336)	902 (783 to 1032)	162 (96 to 252)	6 (0 to 9)
Mauritius	2033	1048 (921 to 1197)	544 (460 to 639)	425 (368 to 486)	77 (46 to 120)	2 (0 to 4)	2264 (1990 to 2586)	1175 (995 to 1381)	917 (794 to 1049)	167 (99 to 259)	5 (0 to 10)
Mauritius	2034	1074 (943 to 1227)	561 (472 to 661)	432 (374 to 494)	79 (47 to 123)	2 (0 to 5)	2320 (2036 to 2650)	1213 (1020 to 1427)	932 (808 to 1068)	171 (101 to 266)	4 (0 to 10)
Mauritius	2035	1100 (968 to 1265)	579 (487 to 681)	439 (381 to 503)	81 (48 to 126)	2 (0 to 5)	2377 (2090 to 2733)	1250 (1051 to 1470)	947 (822 to 1086)	176 (104 to 273)	4 (0 to 10)
Mauritius	2036	1127 (987 to 1296)	596 (500 to 705)	446 (386 to 511)	83 (49 to 130)	2 (0 to 5)	2434 (2131 to 2799)	1287 (1081 to 1523)	963 (834 to 1104)	180 (106 to 281)	3 (0 to 10)
Mauritius	2037	1154 (1005 to 1324)	614 (518 to 728)	453 (392 to 519)	86 (50 to 134)	1 (0 to 5)	2493 (2170 to 2861)	1327 (1119 to 1573)	978 (847 to 1120)	185 (109 to 289)	3 (0 to 10)
Mauritius	2038	1182 (1029 to 1365)	633 (532 to 746)	460 (398 to 528)	88 (52 to 137)	1 (0 to 5)	2554 (2223 to 2949)	1367 (1149 to 1611)	994 (860 to 1140)	190 (112 to 297)	3 (0 to 10)

Mauritius	2039	1212 (1054 to 1391)	652 (547 to 771)	468 (404 to 537)	90 (53 to 141)	1 (0 to 5)	2618 (2276 to 3004)	1409 (1183 to 1666)	1011 (873 to 1159)	195 (115 to 304)	2 (0 to 10)
Mauritius	2040	1243 (1076 to 1445)	673 (562 to 799)	476 (411 to 547)	93 (55 to 144)	1 (0 to 5)	2684 (2325 to 3122)	1453 (1215 to 1726)	1029 (889 to 1182)	200 (118 to 311)	2 (0 to 10)
Mauritius	2041	1274 (1102 to 1479)	693 (578 to 827)	485 (418 to 557)	95 (56 to 148)	1 (0 to 5)	2753 (2381 to 3194)	1498 (1248 to 1787)	1047 (903 to 1204)	206 (121 to 319)	2 (0 to 11)
Mauritius	2042	1307 (1127 to 1517)	715 (594 to 850)	494 (425 to 568)	98 (57 to 152)	1 (0 to 5)	2824 (2434 to 3277)	1545 (1284 to 1836)	1066 (918 to 1227)	211 (124 to 328)	2 (0 to 11)
Mauritius	2043	1341 (1159 to 1547)	738 (611 to 878)	502 (432 to 578)	100 (59 to 156)	1 (0 to 5)	2897 (2504 to 3341)	1594 (1319 to 1897)	1085 (934 to 1249)	217 (127 to 338)	1 (0 to 11)
Mauritius	2044	1376 (1182 to 1587)	761 (628 to 914)	511 (440 to 589)	103 (60 to 161)	1 (0 to 5)	2972 (2553 to 3429)	1645 (1356 to 1974)	1103 (950 to 1273)	223 (130 to 347)	1 (0 to 11)
Mauritius	2045	1412 (1220 to 1641)	786 (649 to 944)	520 (446 to 600)	106 (62 to 165)	1 (0 to 5)	3050 (2634 to 3544)	1698 (1401 to 2039)	1122 (965 to 1297)	229 (134 to 356)	1 (0 to 11)
Mauritius	2046	1449 (1241 to 1692)	811 (667 to 976)	529 (453 to 611)	109 (64 to 170)	1 (0 to 5)	3130 (2681 to 3655)	1751 (1441 to 2108)	1142 (979 to 1319)	235 (137 to 367)	1 (0 to 12)
Mauritius	2047	1486 (1287 to 1721)	835 (688 to 1008)	538 (460 to 621)	112 (65 to 175)	0 (0 to 5)	3210 (2780 to 3717)	1805 (1487 to 2178)	1162 (995 to 1342)	242 (141 to 377)	1 (0 to 12)
Mauritius	2048	1524 (1304 to 1774)	860 (709 to 1037)	548 (468 to 634)	115 (67 to 180)	0 (0 to 6)	3293 (2817 to 3832)	1859 (1531 to 2240)	1185 (1012 to 1369)	248 (145 to 388)	1 (0 to 12)

Mauritius	2049	1564 (1347 to 1808)	886 (731 to 1073)	559 (477 to 646)	118 (69 to 185)	0 (0 to 6)	3378 (2909 to 3906)	1914 (1578 to 2317)	1207 (1030 to 1395)	255 (149 to 399)	1 (0 to 12)
Mauritius	2050	1603 (1379 to 1874)	911 (749 to 1104)	570 (485 to 659)	121 (71 to 190)	0 (0 to 6)	3462 (2980 to 4049)	1968 (1618 to 2385)	1231 (1048 to 1423)	262 (153 to 411)	1 (0 to 12)
Mexico	1995	306 (277 to 337)	129 (112 to 150)	171 (147 to 198)	4 (2 to 8)	2 (2 to 2)	631 (571 to 696)	267 (231 to 309)	352 (303 to 408)	9 (4 to 16)	3 (3 to 3)
Mexico	1996	315 (288 to 344)	137 (121 to 155)	172 (151 to 194)	5 (3 to 9)	1 (1 to 1)	650 (594 to 708)	283 (249 to 320)	354 (310 to 400)	10 (5 to 18)	3 (3 to 3)
Mexico	1997	333 (307 to 360)	149 (133 to 167)	176 (156 to 196)	6 (3 to 11)	2 (2 to 2)	686 (632 to 742)	308 (274 to 345)	362 (322 to 405)	12 (6 to 22)	4 (4 to 4)
Mexico	1998	353 (328 to 380)	159 (142 to 177)	181 (162 to 202)	7 (4 to 11)	6 (6 to 6)	728 (676 to 783)	328 (293 to 364)	374 (334 to 417)	14 (8 to 23)	13 (13 to 13)
Mexico	1999	373 (347 to 400)	172 (155 to 191)	192 (172 to 213)	8 (4 to 14)	1 (1 to 1)	770 (716 to 824)	355 (320 to 393)	396 (355 to 439)	17 (9 to 28)	2 (2 to 2)
Mexico	2000	400 (370 to 428)	179 (162 to 199)	206 (186 to 230)	10 (5 to 16)	4 (4 to 4)	824 (762 to 882)	370 (334 to 410)	425 (384 to 473)	20 (11 to 34)	9 (9 to 9)
Mexico	2001	419 (389 to 449)	184 (167 to 204)	222 (201 to 246)	11 (6 to 19)	1 (1 to 1)	864 (801 to 926)	380 (344 to 420)	458 (414 to 507)	23 (13 to 39)	3 (3 to 3)
Mexico	2002	443 (412 to 473)	189 (171 to 208)	239 (216 to 263)	13 (7 to 21)	2 (2 to 2)	914 (850 to 975)	390 (353 to 428)	493 (445 to 542)	26 (15 to 43)	5 (5 to 5)
Mexico	2003	485 (453 to 517)	206 (187 to 226)	264 (239 to 289)	14 (8 to 23)	0 (0 to 0)	1000 (934 to 1066)	425 (385 to 466)	544 (492 to 596)	30 (17 to 48)	1 (1 to 1)
Mexico	2004	500 (466 to 533)	211 (192 to 231)	273 (249 to 300)	15 (9 to 24)	0 (0 to 0)	1030 (961 to 1099)	434 (395 to 476)	563 (513 to 618)	32 (19 to 49)	1 (1 to 1)
Mexico	2005	513 (481 to 551)	217 (198 to 237)	278 (254 to 307)	17 (10 to 26)	1 (1 to 1)	1057 (992 to 1137)	447 (407 to 489)	573 (523 to 633)	35 (21 to 54)	2 (2 to 2)

Mexico	2006	521 (488 to 557)	223 (205 to 244)	277 (252 to 306)	19 (12 to 29)	1 (1 to 1)	1073 (1005 to 1147)	460 (422 to 504)	570 (520 to 630)	40 (24 to 61)	3 (3 to 3)
Mexico	2007	526 (493 to 563)	231 (212 to 254)	271 (247 to 299)	22 (14 to 33)	1 (1 to 1)	1083 (1015 to 1159)	477 (438 to 524)	559 (508 to 617)	45 (28 to 69)	2 (2 to 2)
Mexico	2008	552 (515 to 594)	255 (234 to 279)	269 (243 to 296)	27 (17 to 41)	1 (1 to 1)	1138 (1062 to 1224)	525 (482 to 576)	554 (502 to 610)	56 (36 to 85)	2 (2 to 2)
Mexico	2009	527 (490 to 567)	248 (227 to 271)	250 (224 to 276)	29 (18 to 44)	0 (0 to 0)	1087 (1009 to 1169)	511 (469 to 559)	516 (462 to 569)	59 (38 to 90)	1 (1 to 1)
Mexico	2010	537 (500 to 574)	259 (238 to 283)	242 (217 to 265)	30 (20 to 46)	6 (6 to 6)	1106 (1030 to 1183)	534 (490 to 584)	498 (448 to 547)	63 (40 to 94)	11 (11 to 11)
Mexico	2011	543 (505 to 581)	271 (249 to 296)	234 (210 to 258)	32 (20 to 49)	5 (5 to 5)	1118 (1040 to 1197)	559 (513 to 611)	483 (433 to 531)	66 (42 to 101)	10 (10 to 10)
Mexico	2012	553 (515 to 591)	284 (261 to 310)	233 (210 to 256)	34 (21 to 51)	3 (3 to 3)	1140 (1062 to 1219)	585 (538 to 640)	479 (432 to 529)	70 (44 to 106)	6 (6 to 6)
Mexico	2013	551 (516 to 588)	288 (264 to 315)	228 (206 to 253)	35 (21 to 53)	1 (1 to 1)	1136 (1063 to 1213)	593 (544 to 649)	470 (424 to 521)	72 (44 to 109)	2 (2 to 2)
Mexico	2014	558 (523 to 596)	294 (271 to 320)	226 (204 to 250)	37 (23 to 58)	1 (1 to 1)	1151 (1078 to 1229)	607 (558 to 660)	466 (420 to 516)	76 (47 to 119)	1 (1 to 1)
Mexico	2015	563 (524 to 601)	296 (271 to 322)	227 (204 to 252)	38 (23 to 60)	1 (1 to 1)	1160 (1081 to 1239)	611 (558 to 663)	468 (421 to 520)	79 (48 to 123)	2 (2 to 2)

Mexico	2016	568 (526 to 613)	298 (269 to 327)	229 (204 to 257)	40 (24 to 63)	1 (1 to 1)	1172 (1083 to 1263)	615 (554 to 675)	473 (420 to 529)	83 (50 to 129)	1 (1 to 1)
Mexico	2017	562 (502 to 629)	291 (259 to 326)	229 (198 to 263)	40 (24 to 66)	1 (1 to 1)	1158 (1035 to 1297)	601 (533 to 672)	473 (409 to 543)	83 (49 to 136)	2 (2 to 2)
Mexico	2018	571 (511 to 637)	298 (263 to 334)	231 (200 to 265)	41 (24 to 67)	1 (1 to 1)	1176 (1053 to 1314)	613 (541 to 688)	476 (412 to 547)	85 (50 to 139)	2 (2 to 2)
Mexico	2019	571 (507 to 641)	299 (264 to 335)	230 (199 to 264)	41 (24 to 68)	1 (1 to 1)	1176 (1045 to 1321)	615 (544 to 690)	474 (410 to 544)	85 (50 to 139)	2 (2 to 2)
Mexico	2020	575 (514 to 643)	301 (267 to 339)	231 (200 to 264)	42 (24 to 68)	1 (1 to 1)	1185 (1060 to 1325)	621 (550 to 698)	476 (412 to 545)	86 (50 to 140)	2 (2 to 2)
Mexico	2021	583 (519 to 655)	308 (272 to 346)	232 (201 to 265)	42 (25 to 69)	1 (1 to 1)	1201 (1069 to 1349)	634 (561 to 713)	478 (413 to 547)	87 (51 to 143)	2 (2 to 2)
Mexico	2022	591 (528 to 661)	314 (277 to 354)	233 (201 to 267)	43 (25 to 70)	1 (1 to 1)	1217 (1089 to 1361)	646 (572 to 730)	480 (415 to 550)	89 (52 to 145)	2 (2 to 2)
Mexico	2023	598 (532 to 673)	320 (282 to 361)	234 (202 to 268)	44 (25 to 71)	1 (1 to 1)	1233 (1096 to 1388)	659 (580 to 744)	482 (417 to 553)	90 (52 to 147)	2 (2 to 2)
Mexico	2024	604 (540 to 677)	324 (286 to 366)	235 (203 to 269)	44 (26 to 72)	1 (1 to 1)	1246 (1113 to 1395)	668 (589 to 755)	484 (419 to 555)	91 (53 to 149)	2 (2 to 2)
Mexico	2025	610 (542 to 689)	329 (289 to 374)	236 (204 to 271)	45 (26 to 73)	1 (1 to 1)	1258 (1117 to 1420)	678 (597 to 771)	486 (421 to 558)	92 (53 to 151)	2 (2 to 2)

Mexico	2026	616 (550 to 692)	334 (292 to 379)	236 (205 to 272)	45 (26 to 74)	1 (1 to 1)	1270 (1134 to 1426)	688 (603 to 781)	487 (422 to 560)	93 (54 to 152)	2 (2 to 2)
Mexico	2027	623 (553 to 703)	338 (296 to 383)	237 (206 to 272)	46 (27 to 75)	1 (1 to 1)	1283 (1139 to 1448)	698 (611 to 790)	489 (424 to 561)	94 (55 to 154)	2 (2 to 2)
Mexico	2028	629 (561 to 706)	343 (301 to 388)	238 (206 to 273)	46 (27 to 76)	1 (1 to 1)	1296 (1156 to 1456)	708 (620 to 800)	491 (425 to 564)	96 (56 to 156)	2 (2 to 2)
Mexico	2029	635 (563 to 719)	348 (305 to 395)	239 (207 to 275)	47 (27 to 77)	1 (1 to 1)	1309 (1160 to 1482)	718 (630 to 814)	492 (426 to 566)	97 (56 to 158)	2 (2 to 2)
Mexico	2030	641 (569 to 721)	353 (308 to 401)	240 (207 to 276)	47 (27 to 77)	1 (1 to 1)	1322 (1172 to 1486)	728 (635 to 828)	494 (426 to 568)	98 (57 to 160)	2 (2 to 3)
Mexico	2031	648 (574 to 734)	358 (313 to 408)	240 (207 to 277)	48 (28 to 78)	1 (1 to 1)	1335 (1183 to 1512)	738 (645 to 840)	495 (427 to 571)	99 (57 to 162)	2 (2 to 3)
Mexico	2032	654 (581 to 735)	364 (317 to 413)	241 (208 to 278)	49 (28 to 79)	1 (1 to 1)	1349 (1198 to 1515)	749 (653 to 852)	497 (428 to 573)	100 (58 to 164)	2 (2 to 3)
Mexico	2033	661 (584 to 749)	369 (322 to 419)	242 (209 to 279)	49 (28 to 80)	1 (1 to 1)	1363 (1204 to 1544)	761 (663 to 863)	499 (430 to 575)	101 (58 to 166)	2 (2 to 3)
Mexico	2034	668 (590 to 757)	374 (325 to 428)	243 (209 to 281)	50 (29 to 81)	1 (1 to 1)	1377 (1217 to 1560)	772 (671 to 883)	500 (432 to 578)	103 (59 to 168)	2 (2 to 3)
Mexico	2035	675 (593 to 769)	380 (331 to 436)	244 (210 to 282)	50 (29 to 82)	1 (1 to 1)	1391 (1222 to 1584)	783 (682 to 898)	502 (433 to 581)	104 (60 to 170)	2 (2 to 3)

Mexico	2036	682 (602 to 775)	386 (334 to 442)	245 (211 to 283)	51 (29 to 83)	1 (1 to 1)	1407 (1240 to 1597)	795 (688 to 912)	504 (434 to 584)	105 (61 to 172)	2 (2 to 3)
Mexico	2037	690 (604 to 785)	392 (336 to 451)	246 (211 to 285)	52 (30 to 84)	1 (1 to 1)	1422 (1245 to 1619)	807 (692 to 930)	506 (435 to 587)	107 (61 to 174)	2 (2 to 3)
Mexico	2038	697 (613 to 797)	397 (342 to 458)	246 (212 to 286)	52 (30 to 85)	1 (1 to 1)	1438 (1264 to 1642)	819 (704 to 945)	508 (436 to 590)	108 (62 to 176)	2 (2 to 3)
Mexico	2039	705 (617 to 801)	403 (345 to 463)	247 (212 to 287)	53 (30 to 86)	1 (1 to 2)	1452 (1272 to 1650)	831 (711 to 953)	510 (438 to 592)	109 (62 to 178)	2 (2 to 3)
Mexico	2040	712 (625 to 814)	409 (349 to 469)	248 (213 to 289)	54 (31 to 88)	1 (1 to 2)	1468 (1288 to 1677)	843 (719 to 966)	512 (440 to 596)	111 (63 to 180)	2 (2 to 3)
Mexico	2041	720 (629 to 820)	415 (354 to 477)	249 (214 to 290)	54 (31 to 89)	1 (1 to 2)	1485 (1297 to 1690)	856 (730 to 984)	514 (441 to 598)	112 (64 to 183)	3 (2 to 3)
Mexico	2042	728 (636 to 834)	421 (359 to 485)	250 (215 to 292)	55 (32 to 90)	1 (1 to 2)	1500 (1310 to 1719)	868 (740 to 999)	516 (443 to 601)	113 (65 to 185)	3 (2 to 3)
Mexico	2043	736 (641 to 840)	427 (364 to 493)	251 (216 to 293)	56 (32 to 91)	1 (1 to 2)	1516 (1321 to 1731)	881 (750 to 1017)	518 (445 to 604)	115 (66 to 187)	3 (2 to 3)
Mexico	2044	743 (649 to 851)	433 (368 to 500)	253 (217 to 295)	56 (32 to 92)	1 (1 to 2)	1532 (1337 to 1753)	892 (759 to 1031)	520 (447 to 607)	116 (66 to 189)	3 (2 to 4)
Mexico	2045	750 (648 to 859)	439 (374 to 506)	254 (218 to 296)	57 (32 to 93)	1 (1 to 2)	1547 (1335 to 1770)	904 (770 to 1042)	523 (449 to 610)	117 (67 to 191)	3 (2 to 3)

Mexico	2046	758 (664 to 869)	445 (379 to 514)	255 (218 to 297)	58 (33 to 94)	1 (1 to 2)	1563 (1368 to 1791)	916 (782 to 1060)	525 (450 to 613)	119 (67 to 193)	3 (2 to 4)
Mexico	2047	766 (661 to 876)	451 (380 to 522)	256 (219 to 299)	58 (33 to 94)	1 (1 to 2)	1579 (1361 to 1805)	929 (784 to 1075)	527 (452 to 616)	120 (68 to 195)	3 (2 to 4)
Mexico	2048	774 (674 to 890)	457 (386 to 530)	257 (220 to 301)	59 (33 to 95)	1 (1 to 2)	1595 (1390 to 1835)	941 (796 to 1092)	529 (453 to 620)	122 (69 to 197)	3 (2 to 4)
Mexico	2049	781 (671 to 893)	463 (389 to 537)	258 (221 to 302)	60 (34 to 97)	1 (1 to 2)	1611 (1382 to 1840)	954 (801 to 1107)	531 (455 to 622)	123 (69 to 199)	3 (2 to 4)
Mexico	2050	789 (686 to 908)	468 (395 to 544)	259 (221 to 303)	60 (34 to 98)	1 (1 to 2)	1626 (1415 to 1871)	965 (814 to 1122)	533 (456 to 624)	124 (70 to 202)	3 (2 to 4)
Moldova	1995	103 (86 to 121)	71 (57 to 87)	30 (22 to 41)	1 (1 to 2)	0 (0 to 0)	239 (201 to 283)	165 (133 to 202)	71 (52 to 95)	3 (1 to 5)	0 (0 to 0)
Moldova	1996	99 (84 to 117)	67 (55 to 83)	31 (22 to 40)	1 (1 to 2)	0 (0 to 0)	231 (195 to 273)	157 (127 to 192)	71 (52 to 94)	3 (1 to 5)	0 (0 to 0)
Moldova	1997	94 (80 to 111)	62 (51 to 75)	31 (23 to 40)	1 (1 to 2)	0 (0 to 0)	219 (187 to 258)	144 (118 to 175)	71 (53 to 94)	3 (1 to 5)	1 (1 to 1)
Moldova	1998	76 (65 to 90)	46 (38 to 57)	29 (22 to 37)	1 (0 to 2)	1 (1 to 1)	178 (151 to 209)	108 (88 to 132)	67 (51 to 87)	2 (1 to 4)	1 (1 to 1)
Moldova	1999	66 (56 to 77)	34 (28 to 42)	27 (20 to 35)	1 (0 to 1)	4 (4 to 4)	154 (131 to 180)	80 (65 to 98)	62 (47 to 82)	2 (1 to 3)	10 (10 to 10)
Moldova	2000	68 (58 to 79)	35 (28 to 42)	29 (22 to 38)	1 (0 to 1)	3 (3 to 3)	158 (135 to 184)	80 (65 to 98)	68 (52 to 88)	2 (1 to 3)	7 (7 to 7)
Moldova	2001	70 (60 to 81)	34 (28 to 42)	32 (25 to 41)	1 (0 to 1)	3 (3 to 3)	163 (140 to 189)	80 (65 to 97)	75 (58 to 96)	2 (1 to 3)	6 (6 to 6)
Moldova	2002	81 (69 to 93)	41 (33 to 49)	38 (30 to 47)	1 (0 to 1)	1 (1 to 1)	188 (161 to 218)	94 (76 to 114)	88 (69 to 110)	2 (1 to 3)	3 (3 to 3)

Moldova	2003	96 (83 to 110)	49 (40 to 58)	44 (35 to 54)	1 (0 to 1)	2 (2 to 2)	223 (193 to 256)	113 (92 to 136)	102 (81 to 127)	2 (1 to 3)	5 (5 to 5)
Moldova	2004	116 (101 to 133)	60 (50 to 73)	51 (41 to 62)	0 (0 to 1)	4 (4 to 4)	271 (236 to 309)	140 (115 to 169)	119 (96 to 145)	1 (1 to 2)	10 (10 to 10)
Moldova	2005	139 (122 to 158)	71 (59 to 84)	61 (49 to 74)	0 (0 to 1)	7 (7 to 7)	324 (284 to 369)	164 (138 to 196)	142 (114 to 172)	1 (0 to 2)	17 (17 to 17)
Moldova	2006	156 (137 to 177)	81 (68 to 96)	72 (58 to 87)	0 (0 to 1)	3 (3 to 3)	364 (319 to 413)	188 (158 to 224)	167 (135 to 202)	1 (0 to 2)	8 (8 to 8)
Moldova	2007	176 (155 to 199)	89 (76 to 106)	81 (66 to 99)	0 (0 to 1)	5 (5 to 5)	411 (360 to 464)	208 (176 to 247)	189 (153 to 230)	1 (0 to 2)	13 (13 to 13)
Moldova	2008	189 (166 to 214)	94 (80 to 111)	89 (72 to 107)	1 (0 to 1)	6 (6 to 6)	440 (387 to 498)	218 (186 to 259)	207 (168 to 249)	1 (1 to 2)	14 (14 to 14)
Moldova	2009	205 (181 to 230)	98 (83 to 115)	94 (76 to 113)	1 (1 to 2)	12 (12 to 12)	477 (421 to 536)	228 (193 to 268)	218 (178 to 263)	3 (1 to 5)	29 (29 to 29)
Moldova	2010	206 (182 to 230)	98 (82 to 115)	92 (76 to 111)	2 (1 to 3)	14 (14 to 14)	479 (425 to 537)	227 (191 to 267)	214 (177 to 259)	4 (2 to 7)	33 (33 to 33)
Moldova	2011	203 (179 to 227)	99 (83 to 116)	92 (76 to 112)	2 (1 to 4)	10 (10 to 10)	474 (418 to 530)	231 (194 to 270)	215 (177 to 260)	5 (2 to 8)	23 (23 to 23)
Moldova	2012	205 (181 to 229)	100 (84 to 118)	92 (76 to 111)	2 (1 to 4)	11 (11 to 11)	477 (421 to 534)	234 (196 to 274)	214 (178 to 258)	5 (2 to 9)	25 (25 to 25)
Moldova	2013	206 (182 to 231)	102 (86 to 120)	93 (77 to 111)	2 (1 to 4)	9 (9 to 9)	480 (424 to 538)	238 (200 to 279)	216 (178 to 260)	5 (2 to 9)	22 (22 to 22)
Moldova	2014	219 (194 to 246)	108 (91 to 127)	95 (79 to 115)	2 (1 to 4)	13 (13 to 13)	510 (453 to 572)	251 (211 to 296)	222 (184 to 268)	5 (3 to 10)	31 (31 to 31)
Moldova	2015	219 (193 to 248)	105 (88 to 124)	97 (79 to 118)	2 (1 to 4)	14 (14 to 14)	509 (449 to 577)	244 (204 to 290)	226 (185 to 275)	5 (3 to 10)	34 (34 to 34)
Moldova	2016	211 (183 to 241)	105 (87 to 126)	97 (78 to 119)	2 (1 to 4)	7 (7 to 7)	492 (427 to 561)	244 (203 to 293)	225 (181 to 278)	6 (3 to 10)	17 (17 to 17)
Moldova	2017	215 (184 to 250)	109 (89 to 132)	97 (76 to 121)	2 (1 to 5)	6 (6 to 6)	500 (428 to 583)	254 (208 to 308)	226 (178 to 282)	6 (3 to 11)	14 (14 to 14)

Moldova	2018	223 (192 to 261)	114 (94 to 140)	100 (78 to 124)	3 (1 to 5)	6 (6 to 6)	519 (447 to 607)	267 (218 to 325)	232 (183 to 289)	6 (3 to 11)	14 (13 to 14)
Moldova	2019	235 (200 to 274)	124 (102 to 152)	102 (80 to 127)	3 (1 to 5)	6 (6 to 6)	547 (465 to 639)	290 (237 to 354)	237 (186 to 295)	6 (3 to 12)	14 (14 to 14)
Moldova	2020	242 (209 to 283)	129 (104 to 157)	105 (82 to 130)	3 (1 to 5)	6 (6 to 7)	565 (488 to 660)	300 (243 to 366)	244 (191 to 303)	7 (3 to 12)	15 (14 to 15)
Moldova	2021	247 (209 to 288)	131 (106 to 161)	106 (84 to 132)	3 (1 to 5)	6 (6 to 7)	575 (486 to 671)	306 (246 to 375)	247 (195 to 307)	7 (3 to 13)	15 (14 to 16)
Moldova	2022	251 (216 to 292)	134 (106 to 164)	107 (84 to 133)	3 (1 to 5)	7 (6 to 7)	584 (502 to 681)	312 (247 to 382)	249 (197 to 310)	7 (3 to 13)	16 (14 to 17)
Moldova	2023	254 (214 to 299)	136 (109 to 168)	108 (85 to 134)	3 (1 to 6)	7 (6 to 8)	593 (499 to 695)	318 (253 to 391)	252 (198 to 313)	7 (3 to 13)	16 (14 to 18)
Moldova	2024	258 (222 to 304)	139 (112 to 173)	109 (85 to 135)	3 (1 to 6)	7 (6 to 8)	602 (516 to 708)	324 (261 to 403)	254 (199 to 316)	7 (3 to 13)	16 (15 to 19)
Moldova	2025	264 (222 to 310)	143 (114 to 177)	110 (86 to 137)	3 (1 to 6)	7 (6 to 8)	614 (517 to 723)	333 (266 to 413)	257 (201 to 320)	7 (3 to 13)	17 (15 to 19)
Moldova	2026	267 (229 to 312)	146 (116 to 180)	111 (87 to 138)	3 (1 to 6)	7 (7 to 8)	623 (533 to 727)	340 (270 to 420)	259 (203 to 322)	7 (3 to 14)	17 (15 to 20)
Moldova	2027	272 (228 to 322)	149 (118 to 185)	112 (88 to 140)	3 (1 to 6)	8 (7 to 9)	634 (532 to 750)	347 (275 to 431)	261 (205 to 325)	7 (3 to 14)	18 (16 to 21)
Moldova	2028	277 (235 to 326)	152 (121 to 191)	113 (89 to 141)	3 (2 to 6)	8 (7 to 9)	645 (548 to 759)	355 (281 to 444)	264 (207 to 329)	8 (4 to 14)	18 (16 to 21)
Moldova	2029	282 (236 to 335)	156 (123 to 196)	115 (90 to 143)	3 (2 to 6)	8 (7 to 10)	658 (549 to 780)	364 (286 to 458)	267 (210 to 333)	8 (4 to 14)	19 (16 to 22)
Moldova	2030	288 (245 to 340)	160 (125 to 201)	116 (92 to 145)	3 (2 to 6)	8 (7 to 10)	671 (570 to 792)	373 (290 to 469)	271 (213 to 337)	8 (4 to 15)	19 (16 to 23)
Moldova	2031	294 (245 to 349)	164 (127 to 206)	118 (93 to 147)	3 (2 to 7)	8 (7 to 10)	684 (570 to 812)	382 (297 to 479)	275 (216 to 342)	8 (4 to 15)	20 (17 to 24)
Moldova	2032	300 (254 to 356)	168 (131 to 213)	119 (94 to 149)	4 (2 to 7)	9 (7 to 10)	699 (592 to 828)	392 (304 to 495)	278 (220 to 347)	8 (4 to 16)	20 (17 to 24)

Moldova	2033	306 (254 to 364)	172 (134 to 218)	121 (95 to 151)	4 (2 to 7)	9 (8 to 11)	713 (592 to 848)	402 (311 to 507)	282 (222 to 351)	8 (4 to 16)	21 (18 to 25)
Moldova	2034	313 (264 to 374)	177 (136 to 224)	123 (97 to 153)	4 (2 to 7)	9 (8 to 11)	728 (614 to 870)	412 (317 to 522)	286 (225 to 357)	9 (4 to 16)	21 (18 to 26)
Moldova	2035	319 (264 to 382)	181 (140 to 232)	125 (98 to 155)	4 (2 to 7)	9 (8 to 12)	744 (616 to 890)	423 (326 to 539)	290 (229 to 362)	9 (4 to 17)	22 (18 to 27)
Moldova	2036	326 (275 to 388)	186 (143 to 238)	126 (100 to 157)	4 (2 to 7)	10 (8 to 12)	759 (642 to 905)	433 (334 to 555)	294 (232 to 366)	9 (4 to 17)	23 (19 to 28)
Moldova	2037	332 (274 to 397)	191 (147 to 244)	128 (101 to 159)	4 (2 to 8)	10 (8 to 12)	774 (638 to 926)	444 (342 to 569)	298 (235 to 371)	9 (4 to 17)	23 (19 to 28)
Moldova	2038	338 (285 to 403)	195 (149 to 250)	129 (102 to 161)	4 (2 to 8)	10 (8 to 13)	788 (663 to 939)	454 (347 to 582)	301 (237 to 375)	10 (4 to 18)	24 (19 to 30)
Moldova	2039	344 (283 to 415)	199 (152 to 257)	130 (103 to 163)	4 (2 to 8)	11 (8 to 13)	802 (660 to 967)	464 (353 to 599)	304 (240 to 379)	10 (4 to 18)	24 (20 to 31)
Moldova	2040	350 (294 to 421)	204 (154 to 264)	132 (104 to 164)	4 (2 to 8)	11 (9 to 14)	816 (686 to 981)	474 (359 to 615)	307 (242 to 383)	10 (5 to 19)	25 (20 to 32)
Moldova	2041	356 (292 to 431)	208 (158 to 268)	133 (105 to 166)	4 (2 to 8)	11 (9 to 14)	830 (681 to 1003)	484 (368 to 625)	310 (245 to 387)	10 (5 to 19)	26 (21 to 33)
Moldova	2042	362 (302 to 437)	212 (161 to 274)	134 (106 to 168)	4 (2 to 8)	11 (9 to 15)	843 (705 to 1018)	494 (374 to 637)	312 (246 to 391)	10 (5 to 19)	27 (21 to 34)
Moldova	2043	368 (300 to 447)	216 (162 to 280)	135 (107 to 169)	4 (2 to 8)	12 (9 to 15)	856 (698 to 1040)	503 (377 to 653)	315 (248 to 394)	10 (5 to 20)	27 (22 to 36)
Moldova	2044	373 (311 to 452)	220 (166 to 288)	136 (107 to 170)	5 (2 to 9)	12 (10 to 16)	869 (724 to 1052)	513 (387 to 670)	317 (250 to 397)	11 (5 to 20)	28 (23 to 37)
Moldova	2045	378 (308 to 457)	224 (168 to 293)	137 (108 to 171)	5 (2 to 9)	13 (10 to 16)	881 (719 to 1065)	522 (392 to 684)	320 (252 to 399)	11 (5 to 20)	29 (23 to 38)
Moldova	2046	384 (319 to 465)	228 (172 to 300)	138 (109 to 172)	5 (2 to 9)	13 (10 to 17)	894 (744 to 1083)	531 (402 to 699)	321 (253 to 401)	11 (5 to 21)	30 (24 to 39)
Moldova	2047	389 (316 to 474)	232 (175 to 305)	139 (109 to 173)	5 (2 to 9)	13 (10 to 18)	905 (737 to 1104)	540 (408 to 711)	323 (254 to 403)	11 (5 to 21)	31 (24 to 42)

Moldova	2048	393 (328 to 477)	235 (177 to 311)	139 (109 to 174)	5 (2 to 9)	14 (11 to 18)	915 (763 to 1111)	548 (412 to 724)	324 (255 to 404)	11 (5 to 21)	32 (25 to 43)
Moldova	2049	396 (320 to 485)	238 (179 to 315)	139 (109 to 174)	5 (2 to 9)	14 (11 to 19)	924 (746 to 1130)	555 (417 to 733)	324 (255 to 405)	11 (5 to 22)	33 (26 to 44)
Moldova	2050	400 (333 to 487)	241 (179 to 320)	139 (109 to 174)	5 (2 to 9)	15 (12 to 20)	931 (777 to 1134)	561 (418 to 745)	325 (255 to 405)	12 (5 to 22)	34 (27 to 46)
Mongolia	1995	50 (41 to 59)	38 (30 to 47)	7 (5 to 10)	2 (1 to 4)	2 (2 to 2)	173 (142 to 205)	132 (103 to 163)	25 (18 to 34)	8 (4 to 14)	9 (9 to 9)
Mongolia	1996	52 (43 to 62)	41 (32 to 50)	7 (5 to 10)	2 (1 to 4)	2 (2 to 2)	181 (150 to 216)	141 (112 to 176)	26 (18 to 35)	7 (3 to 13)	7 (7 to 7)
Mongolia	1997	55 (45 to 65)	43 (35 to 54)	8 (6 to 11)	2 (1 to 4)	1 (1 to 1)	190 (157 to 227)	151 (121 to 188)	27 (19 to 37)	7 (3 to 13)	4 (4 to 4)
Mongolia	1998	62 (52 to 74)	48 (39 to 60)	9 (6 to 12)	2 (1 to 3)	3 (3 to 3)	216 (182 to 257)	168 (136 to 207)	30 (22 to 42)	7 (3 to 12)	11 (11 to 11)
Mongolia	1999	64 (54 to 75)	47 (39 to 58)	10 (7 to 13)	2 (1 to 4)	4 (4 to 4)	222 (189 to 261)	164 (134 to 203)	34 (24 to 46)	8 (4 to 15)	15 (15 to 15)
Mongolia	2000	66 (56 to 79)	49 (40 to 61)	12 (9 to 17)	3 (1 to 5)	2 (2 to 2)	231 (195 to 273)	172 (138 to 211)	43 (31 to 58)	10 (5 to 18)	7 (7 to 7)
Mongolia	2001	72 (62 to 84)	49 (39 to 60)	14 (10 to 19)	3 (1 to 6)	7 (7 to 7)	252 (216 to 293)	170 (137 to 210)	49 (35 to 66)	10 (5 to 19)	23 (23 to 23)
Mongolia	2002	67 (57 to 79)	48 (39 to 60)	15 (11 to 21)	3 (1 to 6)	1 (1 to 1)	235 (198 to 276)	169 (137 to 207)	54 (39 to 73)	10 (5 to 19)	2 (2 to 2)
Mongolia	2003	66 (56 to 78)	46 (37 to 56)	16 (12 to 22)	3 (1 to 5)	2 (2 to 2)	231 (195 to 270)	159 (130 to 193)	56 (42 to 76)	9 (4 to 17)	7 (7 to 7)
Mongolia	2004	68 (58 to 80)	46 (38 to 56)	18 (13 to 24)	3 (1 to 5)	2 (2 to 2)	237 (200 to 277)	161 (132 to 196)	62 (46 to 83)	9 (4 to 18)	5 (5 to 5)
Mongolia	2005	71 (60 to 82)	47 (38 to 57)	19 (14 to 26)	3 (1 to 5)	3 (3 to 3)	248 (209 to 287)	162 (134 to 198)	68 (50 to 91)	9 (4 to 16)	9 (9 to 9)
Mongolia	2006	78 (66 to 91)	53 (43 to 64)	21 (16 to 28)	3 (1 to 5)	2 (2 to 2)	272 (231 to 317)	183 (151 to 223)	73 (54 to 97)	9 (4 to 17)	7 (7 to 7)

Mongolia	2007	100 (85 to 116)	67 (56 to 82)	24 (18 to 32)	3 (1 to 6)	6 (6 to 6)	348 (297 to 404)	234 (193 to 284)	83 (62 to 111)	11 (5 to 21)	20 (20 to 20)
Mongolia	2008	107 (91 to 124)	73 (60 to 88)	26 (19 to 34)	3 (1 to 6)	5 (5 to 5)	371 (316 to 433)	254 (210 to 308)	89 (66 to 119)	10 (4 to 20)	17 (17 to 17)
Mongolia	2009	100 (86 to 116)	66 (55 to 79)	26 (19 to 34)	2 (1 to 5)	6 (6 to 6)	349 (300 to 404)	229 (190 to 277)	90 (67 to 117)	9 (4 to 16)	21 (21 to 21)
Mongolia	2010	103 (90 to 118)	60 (50 to 73)	27 (20 to 36)	2 (1 to 4)	13 (13 to 13)	359 (313 to 409)	210 (175 to 254)	95 (70 to 124)	8 (3 to 15)	46 (46 to 46)
Mongolia	2011	118 (103 to 135)	71 (60 to 85)	34 (25 to 44)	3 (1 to 5)	11 (11 to 11)	412 (358 to 470)	248 (208 to 297)	117 (87 to 153)	9 (4 to 18)	37 (37 to 37)
Mongolia	2012	128 (111 to 147)	76 (64 to 90)	39 (30 to 50)	3 (1 to 5)	10 (10 to 10)	445 (387 to 510)	266 (222 to 315)	136 (103 to 176)	10 (5 to 18)	33 (33 to 33)
Mongolia	2013	140 (121 to 160)	80 (67 to 95)	45 (34 to 58)	3 (2 to 6)	11 (11 to 11)	486 (422 to 557)	278 (232 to 330)	157 (120 to 204)	11 (5 to 20)	40 (40 to 40)
Mongolia	2014	146 (126 to 168)	85 (71 to 102)	49 (38 to 64)	4 (2 to 7)	8 (8 to 8)	508 (440 to 586)	296 (247 to 355)	171 (131 to 222)	13 (6 to 23)	28 (28 to 28)
Mongolia	2015	145 (125 to 167)	83 (68 to 100)	50 (38 to 65)	4 (2 to 7)	8 (8 to 8)	505 (437 to 581)	288 (237 to 348)	173 (132 to 226)	14 (7 to 26)	29 (29 to 29)
Mongolia	2016	169 (145 to 195)	98 (80 to 120)	53 (40 to 70)	5 (3 to 9)	13 (13 to 13)	588 (505 to 680)	340 (278 to 419)	186 (140 to 242)	18 (9 to 32)	45 (45 to 45)
Mongolia	2017	162 (139 to 188)	88 (71 to 109)	50 (37 to 66)	5 (2 to 9)	20 (20 to 20)	563 (484 to 653)	305 (248 to 380)	173 (129 to 230)	16 (8 to 30)	68 (68 to 68)
Mongolia	2018	162 (140 to 190)	88 (70 to 108)	51 (38 to 68)	5 (2 to 9)	18 (18 to 19)	564 (486 to 660)	305 (245 to 376)	178 (133 to 237)	17 (8 to 31)	64 (64 to 64)
Mongolia	2019	175 (150 to 204)	98 (80 to 122)	53 (40 to 71)	5 (3 to 10)	18 (18 to 19)	610 (522 to 709)	343 (278 to 423)	185 (138 to 247)	18 (9 to 34)	64 (63 to 65)
Mongolia	2020	182 (157 to 213)	103 (83 to 128)	55 (41 to 73)	5 (3 to 10)	18 (17 to 19)	634 (545 to 741)	360 (289 to 445)	192 (143 to 255)	19 (9 to 35)	64 (61 to 68)
Mongolia	2021	187 (160 to 220)	107 (85 to 133)	56 (42 to 75)	6 (3 to 10)	19 (18 to 20)	652 (558 to 767)	372 (295 to 464)	195 (145 to 259)	20 (10 to 36)	65 (61 to 71)

Mongolia	2022	192 (163 to 225)	110 (87 to 137)	57 (42 to 76)	6 (3 to 11)	19 (18 to 21)	669 (568 to 782)	384 (303 to 479)	198 (147 to 264)	20 (10 to 37)	67 (61 to 74)
Mongolia	2023	197 (168 to 231)	114 (90 to 142)	58 (43 to 77)	6 (3 to 11)	19 (17 to 22)	686 (585 to 806)	397 (313 to 495)	202 (150 to 268)	21 (10 to 38)	67 (61 to 75)
Mongolia	2024	201 (170 to 235)	117 (91 to 146)	59 (44 to 78)	6 (3 to 11)	20 (17 to 22)	701 (591 to 820)	406 (316 to 510)	205 (152 to 272)	21 (10 to 39)	68 (61 to 77)
Mongolia	2025	205 (175 to 241)	119 (93 to 149)	60 (45 to 80)	6 (3 to 11)	20 (18 to 23)	713 (608 to 840)	414 (324 to 519)	209 (156 to 278)	22 (11 to 40)	69 (61 to 78)
Mongolia	2026	208 (179 to 245)	121 (94 to 151)	61 (45 to 81)	6 (3 to 12)	20 (18 to 23)	725 (622 to 852)	421 (329 to 526)	212 (158 to 282)	22 (11 to 41)	70 (62 to 80)
Mongolia	2027	212 (179 to 250)	123 (97 to 155)	62 (46 to 83)	6 (3 to 12)	20 (18 to 24)	738 (625 to 871)	428 (336 to 539)	217 (161 to 287)	22 (11 to 42)	71 (62 to 82)
Mongolia	2028	216 (183 to 254)	126 (98 to 158)	64 (47 to 84)	7 (3 to 12)	21 (18 to 24)	753 (637 to 884)	437 (341 to 551)	221 (165 to 293)	23 (11 to 42)	72 (62 to 84)
Mongolia	2029	221 (187 to 258)	128 (99 to 163)	65 (48 to 86)	7 (3 to 12)	21 (18 to 25)	769 (652 to 898)	446 (346 to 567)	226 (168 to 299)	23 (11 to 43)	73 (63 to 85)
Mongolia	2030	226 (191 to 267)	131 (101 to 167)	67 (50 to 88)	7 (3 to 13)	21 (18 to 25)	786 (664 to 929)	457 (353 to 583)	232 (172 to 307)	24 (12 to 44)	74 (63 to 88)
Mongolia	2031	231 (193 to 276)	134 (103 to 173)	68 (51 to 91)	7 (3 to 13)	21 (18 to 25)	805 (672 to 961)	468 (360 to 604)	238 (177 to 316)	25 (12 to 46)	75 (64 to 89)
Mongolia	2032	237 (199 to 280)	138 (105 to 179)	70 (52 to 93)	7 (4 to 13)	22 (18 to 26)	824 (694 to 975)	479 (367 to 625)	244 (181 to 325)	25 (12 to 47)	75 (64 to 91)
Mongolia	2033	243 (205 to 290)	141 (108 to 186)	72 (53 to 96)	7 (4 to 14)	22 (19 to 27)	845 (714 to 1011)	492 (375 to 647)	250 (186 to 334)	26 (13 to 48)	76 (65 to 92)
Mongolia	2034	249 (208 to 299)	146 (110 to 193)	74 (55 to 99)	8 (4 to 14)	22 (18 to 27)	869 (725 to 1040)	507 (382 to 671)	258 (191 to 344)	27 (13 to 50)	77 (64 to 93)
Mongolia	2035	256 (215 to 308)	150 (112 to 201)	76 (56 to 101)	8 (4 to 15)	22 (19 to 27)	893 (750 to 1073)	522 (391 to 700)	265 (197 to 353)	28 (14 to 51)	78 (65 to 94)
Mongolia	2036	263 (219 to 318)	154 (115 to 207)	78 (58 to 104)	8 (4 to 15)	23 (19 to 28)	917 (762 to 1109)	537 (400 to 722)	273 (202 to 363)	28 (14 to 53)	78 (65 to 98)

Mongolia	2037	270 (223 to 330)	159 (117 to 216)	80 (59 to 107)	8 (4 to 16)	23 (19 to 28)	940 (777 to 1148)	552 (407 to 754)	280 (207 to 372)	29 (14 to 55)	79 (65 to 97)
Mongolia	2038	277 (229 to 340)	163 (119 to 223)	82 (61 to 110)	9 (4 to 16)	23 (19 to 29)	964 (796 to 1182)	567 (414 to 775)	287 (212 to 382)	30 (15 to 56)	80 (66 to 100)
Mongolia	2039	284 (233 to 349)	167 (121 to 231)	84 (62 to 113)	9 (4 to 17)	23 (19 to 29)	987 (810 to 1216)	582 (423 to 803)	294 (216 to 392)	31 (15 to 58)	81 (66 to 102)
Mongolia	2040	290 (238 to 356)	171 (124 to 238)	86 (64 to 115)	9 (4 to 17)	24 (19 to 30)	1011 (828 to 1240)	597 (432 to 827)	301 (221 to 401)	32 (16 to 59)	82 (67 to 103)
Mongolia	2041	297 (239 to 374)	176 (127 to 245)	88 (65 to 118)	9 (5 to 17)	24 (20 to 30)	1036 (832 to 1303)	613 (442 to 852)	307 (226 to 410)	32 (16 to 61)	83 (68 to 105)
Mongolia	2042	305 (247 to 379)	181 (130 to 253)	90 (66 to 121)	10 (5 to 18)	24 (20 to 31)	1061 (862 to 1319)	629 (452 to 882)	314 (231 to 420)	33 (16 to 62)	84 (68 to 107)
Mongolia	2043	312 (254 to 392)	185 (132 to 261)	92 (68 to 124)	10 (5 to 18)	24 (20 to 32)	1086 (884 to 1364)	645 (460 to 908)	321 (236 to 430)	34 (17 to 64)	85 (69 to 110)
Mongolia	2044	319 (259 to 404)	190 (136 to 270)	94 (69 to 126)	10 (5 to 19)	25 (20 to 33)	1111 (901 to 1407)	662 (473 to 939)	328 (242 to 440)	35 (17 to 65)	86 (70 to 113)
Mongolia	2045	327 (263 to 412)	195 (138 to 277)	96 (71 to 129)	10 (5 to 19)	25 (20 to 32)	1137 (916 to 1433)	679 (479 to 963)	335 (247 to 449)	36 (18 to 67)	87 (69 to 112)
Mongolia	2046	334 (269 to 428)	200 (141 to 284)	98 (72 to 132)	11 (5 to 20)	25 (20 to 33)	1163 (936 to 1492)	696 (490 to 989)	342 (252 to 459)	37 (18 to 69)	88 (70 to 113)
Mongolia	2047	341 (275 to 436)	205 (144 to 293)	100 (74 to 134)	11 (5 to 20)	26 (20 to 34)	1189 (959 to 1517)	713 (500 to 1020)	349 (257 to 468)	38 (18 to 70)	90 (71 to 118)

Mongolia	2048	349 (281 to 447)	210 (147 to 301)	102 (75 to 137)	11 (5 to 21)	26 (21 to 35)	1215 (979 to 1556)	731 (511 to 1047)	355 (261 to 477)	38 (19 to 72)	91 (72 to 121)
Mongolia	2049	357 (284 to 459)	215 (150 to 310)	104 (76 to 140)	11 (6 to 21)	27 (21 to 35)	1242 (988 to 1597)	749 (522 to 1081)	362 (266 to 486)	39 (19 to 74)	92 (73 to 123)
Mongolia	2050	365 (290 to 473)	220 (155 to 319)	106 (78 to 142)	12 (6 to 22)	27 (21 to 37)	1270 (1011 to 1647)	767 (538 to 1112)	368 (271 to 495)	40 (20 to 75)	94 (74 to 127)
Montenegro	1995	204 (163 to 251)	131 (98 to 175)	65 (45 to 92)	7 (3 to 13)	1 (1 to 1)	471 (377 to 580)	303 (226 to 404)	150 (104 to 212)	16 (8 to 30)	2 (2 to 2)
Montenegro	1996	225 (179 to 281)	148 (112 to 194)	69 (48 to 97)	8 (4 to 15)	0 (0 to 0)	521 (413 to 650)	342 (259 to 448)	161 (111 to 225)	18 (9 to 34)	0 (0 to 0)
Montenegro	1997	235 (188 to 292)	155 (117 to 208)	72 (49 to 102)	8 (4 to 15)	0 (0 to 0)	545 (434 to 676)	358 (271 to 481)	167 (114 to 236)	19 (9 to 36)	0 (0 to 0)
Montenegro	1998	250 (201 to 309)	165 (125 to 218)	76 (52 to 107)	9 (4 to 16)	0 (0 to 0)	578 (464 to 715)	381 (290 to 504)	176 (121 to 248)	21 (10 to 38)	0 (0 to 0)
Montenegro	1999	246 (199 to 308)	161 (122 to 211)	76 (53 to 106)	9 (4 to 16)	0 (0 to 0)	570 (459 to 712)	373 (283 to 488)	177 (123 to 246)	20 (10 to 37)	0 (0 to 0)
Montenegro	2000	258 (209 to 322)	169 (128 to 222)	80 (56 to 112)	9 (5 to 17)	0 (0 to 0)	597 (482 to 744)	390 (297 to 514)	185 (129 to 260)	21 (11 to 40)	1 (1 to 1)
Montenegro	2001	282 (226 to 352)	185 (140 to 244)	86 (60 to 121)	10 (5 to 19)	0 (0 to 0)	651 (524 to 815)	428 (323 to 564)	199 (139 to 279)	24 (12 to 44)	0 (0 to 0)
Montenegro	2002	294 (237 to 366)	192 (144 to 251)	90 (63 to 127)	11 (5 to 21)	1 (1 to 1)	681 (548 to 847)	443 (333 to 580)	209 (146 to 294)	26 (13 to 48)	3 (3 to 3)
Montenegro	2003	333 (268 to 413)	220 (164 to 288)	100 (70 to 141)	13 (7 to 25)	0 (0 to 0)	771 (619 to 957)	508 (380 to 666)	231 (161 to 325)	31 (16 to 58)	1 (1 to 1)
Montenegro	2004	346 (281 to 427)	222 (164 to 289)	103 (73 to 146)	14 (7 to 26)	7 (7 to 7)	802 (649 to 987)	513 (380 to 669)	239 (168 to 338)	32 (16 to 61)	17 (17 to 17)
Montenegro	2005	355 (285 to 441)	229 (171 to 301)	108 (76 to 152)	15 (7 to 27)	2 (2 to 2)	821 (659 to 1020)	530 (395 to 696)	250 (176 to 353)	35 (17 to 63)	6 (6 to 6)

Montenegro	2006	432 (350 to 534)	276 (206 to 362)	122 (86 to 172)	19 (9 to 35)	15 (15 to 15)	999 (809 to 1235)	638 (476 to 837)	283 (198 to 399)	43 (21 to 81)	35 (35 to 35)
Montenegro	2007	482 (387 to 600)	312 (235 to 407)	134 (91 to 188)	22 (10 to 41)	13 (13 to 13)	1114 (896 to 1387)	723 (544 to 942)	311 (212 to 436)	50 (24 to 94)	31 (31 to 31)
Montenegro	2008	564 (454 to 699)	371 (277 to 489)	150 (102 to 211)	26 (13 to 50)	17 (17 to 17)	1304 (1051 to 1617)	857 (640 to 1131)	348 (235 to 488)	60 (29 to 115)	38 (38 to 38)
Montenegro	2009	550 (442 to 679)	367 (274 to 486)	152 (103 to 211)	26 (13 to 49)	5 (5 to 5)	1272 (1022 to 1571)	848 (635 to 1124)	352 (238 to 489)	60 (29 to 114)	11 (11 to 11)
Montenegro	2010	539 (430 to 672)	356 (265 to 471)	152 (103 to 211)	25 (12 to 48)	5 (5 to 5)	1246 (995 to 1555)	823 (612 to 1091)	352 (238 to 489)	59 (28 to 110)	13 (13 to 13)
Montenegro	2011	551 (439 to 685)	362 (268 to 477)	156 (105 to 215)	26 (13 to 48)	7 (7 to 7)	1274 (1015 to 1586)	837 (619 to 1104)	360 (244 to 498)	60 (30 to 111)	17 (17 to 17)
Montenegro	2012	550 (436 to 679)	361 (265 to 481)	157 (107 to 218)	26 (13 to 49)	5 (5 to 5)	1272 (1009 to 1570)	835 (614 to 1113)	364 (247 to 504)	61 (30 to 112)	12 (12 to 12)
Montenegro	2013	568 (452 to 703)	374 (277 to 497)	162 (110 to 227)	27 (13 to 51)	4 (4 to 4)	1313 (1046 to 1627)	866 (640 to 1151)	374 (253 to 525)	64 (30 to 118)	10 (10 to 10)
Montenegro	2014	569 (453 to 715)	375 (277 to 502)	164 (111 to 233)	28 (13 to 53)	3 (3 to 3)	1318 (1049 to 1655)	868 (642 to 1162)	379 (257 to 539)	64 (31 to 122)	7 (7 to 7)
Montenegro	2015	609 (485 to 765)	405 (303 to 548)	172 (117 to 246)	30 (15 to 58)	2 (2 to 2)	1410 (1122 to 1769)	936 (702 to 1268)	399 (270 to 570)	70 (34 to 134)	5 (5 to 5)

Montenegro	2016	646 (516 to 810)	429 (321 to 582)	180 (122 to 256)	33 (16 to 62)	4 (4 to 4)	1494 (1194 to 1875)	993 (743 to 1347)	416 (282 to 594)	76 (36 to 143)	9 (9 to 9)
Montenegro	2017	672 (518 to 870)	450 (332 to 605)	187 (126 to 272)	35 (17 to 65)	0 (0 to 0)	1555 (1198 to 2012)	1042 (768 to 1401)	432 (293 to 629)	80 (39 to 151)	1 (1 to 1)
Montenegro	2018	715 (553 to 921)	482 (358 to 652)	195 (132 to 284)	37 (18 to 70)	0 (0 to 0)	1654 (1281 to 2132)	1115 (829 to 1509)	452 (306 to 656)	85 (41 to 161)	1 (1 to 1)
Montenegro	2019	715 (550 to 930)	477 (355 to 647)	200 (135 to 291)	37 (18 to 70)	0 (0 to 0)	1654 (1273 to 2151)	1104 (822 to 1497)	463 (313 to 673)	86 (41 to 162)	1 (1 to 1)
Montenegro	2020	737 (574 to 961)	494 (367 to 668)	204 (138 to 298)	38 (18 to 72)	0 (0 to 0)	1705 (1329 to 2223)	1142 (849 to 1547)	473 (320 to 689)	89 (43 to 167)	1 (1 to 1)
Montenegro	2021	750 (579 to 989)	504 (372 to 681)	207 (139 to 301)	39 (19 to 74)	0 (0 to 0)	1735 (1341 to 2288)	1165 (861 to 1575)	478 (323 to 696)	90 (43 to 171)	1 (1 to 1)
Montenegro	2022	763 (589 to 989)	514 (380 to 693)	209 (141 to 305)	40 (19 to 75)	0 (0 to 0)	1765 (1364 to 2289)	1189 (880 to 1603)	484 (326 to 705)	92 (44 to 174)	1 (1 to 1)
Montenegro	2023	777 (598 to 1017)	524 (388 to 705)	212 (142 to 309)	41 (20 to 77)	0 (0 to 0)	1797 (1383 to 2354)	1213 (897 to 1632)	490 (329 to 714)	94 (45 to 177)	1 (1 to 1)
Montenegro	2024	790 (611 to 1020)	534 (395 to 718)	214 (144 to 312)	41 (20 to 78)	0 (0 to 0)	1828 (1415 to 2359)	1237 (914 to 1662)	495 (333 to 722)	95 (46 to 181)	1 (1 to 1)
Montenegro	2025	804 (619 to 1051)	545 (402 to 730)	217 (146 to 315)	42 (20 to 80)	0 (0 to 0)	1860 (1432 to 2433)	1261 (931 to 1689)	501 (337 to 729)	97 (47 to 184)	1 (1 to 1)

Montenegro	2026	818 (634 to 1053)	556 (410 to 745)	219 (147 to 319)	43 (21 to 81)	0 (0 to 0)	1892 (1466 to 2436)	1286 (949 to 1725)	507 (341 to 738)	99 (48 to 188)	1 (1 to 1)
Montenegro	2027	832 (635 to 1092)	566 (419 to 760)	221 (149 to 323)	44 (21 to 83)	0 (0 to 1)	1925 (1470 to 2526)	1310 (969 to 1760)	512 (345 to 748)	101 (48 to 191)	1 (1 to 1)
Montenegro	2028	846 (649 to 1095)	577 (427 to 774)	224 (151 to 328)	44 (21 to 84)	0 (0 to 1)	1958 (1503 to 2533)	1336 (988 to 1790)	518 (350 to 758)	103 (49 to 194)	1 (1 to 1)
Montenegro	2029	862 (659 to 1120)	589 (435 to 790)	227 (153 to 332)	45 (22 to 86)	0 (0 to 1)	1994 (1525 to 2591)	1363 (1006 to 1827)	525 (355 to 768)	105 (50 to 198)	1 (1 to 1)
Montenegro	2030	877 (674 to 1143)	601 (443 to 806)	229 (155 to 335)	46 (22 to 87)	0 (0 to 1)	2029 (1559 to 2645)	1391 (1024 to 1865)	531 (359 to 775)	107 (51 to 202)	1 (1 to 1)
Montenegro	2031	893 (681 to 1164)	613 (452 to 823)	232 (157 to 338)	47 (22 to 89)	0 (0 to 1)	2066 (1576 to 2694)	1419 (1046 to 1904)	537 (364 to 783)	109 (52 to 206)	1 (1 to 1)
Montenegro	2032	909 (699 to 1189)	626 (462 to 840)	235 (159 to 343)	48 (23 to 91)	0 (0 to 1)	2104 (1618 to 2751)	1448 (1070 to 1944)	544 (368 to 793)	111 (53 to 211)	1 (1 to 1)
Montenegro	2033	927 (704 to 1212)	639 (472 to 859)	238 (161 to 347)	49 (23 to 93)	0 (0 to 1)	2144 (1628 to 2804)	1479 (1092 to 1988)	551 (373 to 804)	113 (54 to 215)	1 (1 to 1)
Montenegro	2034	944 (718 to 1236)	653 (480 to 877)	241 (163 to 351)	50 (24 to 94)	1 (0 to 1)	2185 (1661 to 2860)	1510 (1111 to 2030)	558 (378 to 813)	115 (55 to 218)	1 (1 to 1)
Montenegro	2035	962 (730 to 1252)	667 (489 to 896)	244 (166 to 356)	51 (24 to 96)	1 (0 to 1)	2227 (1689 to 2897)	1543 (1131 to 2074)	566 (383 to 823)	118 (56 to 222)	1 (1 to 1)

Montenegro	2036	981 (748 to 1280)	681 (498 to 916)	248 (168 to 360)	52 (25 to 98)	1 (0 to 1)	2271 (1730 to 2962)	1576 (1153 to 2119)	573 (389 to 833)	120 (57 to 226)	1 (1 to 2)
Montenegro	2037	999 (756 to 1304)	695 (508 to 934)	251 (170 to 364)	53 (25 to 100)	1 (0 to 1)	2313 (1750 to 3017)	1608 (1175 to 2162)	581 (394 to 842)	122 (58 to 231)	1 (1 to 2)
Montenegro	2038	1018 (777 to 1324)	709 (518 to 953)	254 (172 to 368)	54 (25 to 102)	1 (0 to 1)	2355 (1798 to 3065)	1640 (1199 to 2206)	588 (399 to 852)	125 (59 to 235)	1 (1 to 2)
Montenegro	2039	1036 (785 to 1365)	723 (527 to 973)	257 (174 to 373)	55 (26 to 104)	1 (0 to 1)	2398 (1817 to 3158)	1674 (1219 to 2251)	596 (404 to 863)	127 (60 to 240)	1 (1 to 2)
Montenegro	2040	1055 (808 to 1377)	738 (536 to 992)	261 (177 to 378)	56 (26 to 106)	1 (0 to 1)	2441 (1870 to 3187)	1707 (1240 to 2295)	603 (409 to 874)	130 (61 to 245)	1 (1 to 2)
Montenegro	2041	1074 (805 to 1412)	752 (547 to 1013)	264 (179 to 383)	57 (27 to 108)	1 (0 to 1)	2485 (1863 to 3266)	1741 (1265 to 2343)	611 (414 to 886)	132 (62 to 250)	1 (1 to 2)
Montenegro	2042	1093 (840 to 1423)	767 (557 to 1033)	267 (182 to 388)	58 (27 to 110)	1 (0 to 1)	2529 (1945 to 3293)	1775 (1290 to 2391)	618 (420 to 897)	135 (63 to 255)	1 (1 to 2)
Montenegro	2043	1112 (839 to 1457)	782 (568 to 1053)	271 (184 to 393)	59 (28 to 112)	1 (0 to 1)	2574 (1942 to 3371)	1809 (1315 to 2437)	626 (425 to 909)	137 (65 to 260)	1 (0 to 2)
Montenegro	2044	1131 (867 to 1472)	796 (579 to 1073)	274 (186 to 398)	60 (28 to 115)	1 (0 to 1)	2618 (2006 to 3406)	1843 (1340 to 2483)	634 (430 to 921)	140 (66 to 266)	1 (0 to 2)
Montenegro	2045	1150 (864 to 1512)	811 (590 to 1092)	277 (188 to 403)	62 (29 to 117)	1 (0 to 1)	2662 (2000 to 3499)	1876 (1365 to 2527)	642 (434 to 932)	142 (67 to 270)	1 (0 to 2)

Montenegro	2046	1170 (896 to 1532)	826 (601 to 1112)	281 (190 to 408)	63 (29 to 119)	1 (0 to 1)	2707 (2073 to 3544)	1911 (1390 to 2573)	650 (439 to 944)	145 (68 to 275)	1 (0 to 2)
Montenegro	2047	1189 (892 to 1553)	841 (611 to 1131)	284 (192 to 413)	64 (30 to 121)	1 (0 to 1)	2751 (2064 to 3593)	1945 (1414 to 2618)	657 (444 to 955)	148 (69 to 280)	1 (0 to 2)
Montenegro	2048	1208 (922 to 1581)	855 (622 to 1151)	287 (194 to 418)	65 (31 to 123)	1 (0 to 1)	2795 (2133 to 3658)	1979 (1439 to 2663)	665 (448 to 967)	150 (71 to 285)	1 (0 to 2)
Montenegro	2049	1228 (925 to 1606)	870 (633 to 1170)	291 (196 to 423)	66 (31 to 125)	1 (0 to 1)	2840 (2141 to 3716)	2014 (1464 to 2708)	672 (453 to 978)	153 (72 to 290)	1 (0 to 2)
Montenegro	2050	1247 (946 to 1623)	885 (643 to 1191)	294 (198 to 428)	67 (32 to 128)	1 (0 to 1)	2885 (2189 to 3756)	2048 (1489 to 2755)	680 (458 to 990)	155 (73 to 295)	1 (0 to 2)
Morocco	1995	49 (40 to 59)	15 (11 to 19)	30 (22 to 40)	3 (1 to 5)	1 (1 to 1)	135 (109 to 163)	40 (31 to 52)	83 (61 to 111)	7 (4 to 14)	4 (4 to 4)
Morocco	1996	52 (43 to 63)	15 (11 to 19)	31 (24 to 42)	3 (2 to 6)	2 (2 to 2)	143 (117 to 174)	41 (31 to 53)	86 (65 to 115)	10 (5 to 17)	6 (6 to 6)
Morocco	1997	54 (44 to 66)	15 (12 to 19)	33 (25 to 43)	5 (2 to 8)	1 (1 to 1)	148 (122 to 181)	42 (32 to 53)	90 (68 to 120)	13 (6 to 23)	3 (3 to 3)
Morocco	1998	60 (50 to 74)	16 (13 to 21)	35 (26 to 46)	7 (3 to 12)	2 (2 to 2)	166 (138 to 203)	45 (35 to 57)	97 (73 to 127)	18 (9 to 32)	6 (6 to 6)
Morocco	1999	63 (52 to 75)	17 (13 to 21)	37 (28 to 48)	8 (4 to 15)	1 (1 to 1)	174 (144 to 207)	46 (36 to 58)	101 (78 to 131)	23 (12 to 40)	4 (4 to 4)
Morocco	2000	73 (61 to 87)	19 (15 to 24)	40 (31 to 51)	12 (6 to 20)	2 (2 to 2)	200 (168 to 240)	52 (41 to 65)	110 (86 to 141)	32 (16 to 55)	6 (6 to 6)
Morocco	2001	80 (66 to 97)	22 (17 to 27)	44 (34 to 56)	14 (7 to 24)	1 (1 to 1)	219 (183 to 267)	59 (46 to 74)	120 (94 to 153)	38 (20 to 66)	2 (2 to 2)
Morocco	2002	88 (75 to 106)	24 (19 to 30)	48 (38 to 61)	15 (8 to 26)	1 (1 to 1)	243 (205 to 292)	67 (53 to 83)	132 (103 to 167)	41 (21 to 71)	3 (3 to 3)

Morocco	2003	93 (79 to 112)	26 (20 to 32)	51 (40 to 65)	14 (8 to 25)	2 (2 to 2)	255 (216 to 307)	71 (55 to 89)	141 (110 to 178)	39 (21 to 68)	4 (4 to 4)
Morocco	2004	98 (83 to 116)	28 (23 to 35)	55 (43 to 69)	13 (7 to 23)	1 (1 to 1)	269 (229 to 318)	78 (62 to 97)	152 (120 to 191)	36 (19 to 62)	3 (3 to 3)
Morocco	2005	111 (96 to 130)	34 (27 to 42)	61 (49 to 77)	11 (6 to 20)	5 (5 to 5)	306 (263 to 357)	93 (74 to 115)	169 (134 to 211)	31 (16 to 54)	13 (13 to 13)
Morocco	2006	115 (99 to 134)	39 (31 to 47)	65 (52 to 81)	8 (4 to 13)	3 (3 to 3)	316 (273 to 370)	106 (86 to 130)	180 (143 to 224)	21 (11 to 37)	8 (8 to 8)
Morocco	2007	123 (107 to 144)	45 (36 to 55)	70 (56 to 87)	7 (4 to 12)	2 (2 to 2)	340 (294 to 397)	124 (100 to 151)	193 (154 to 239)	18 (10 to 32)	5 (5 to 5)
Morocco	2008	139 (121 to 161)	52 (43 to 64)	76 (61 to 92)	6 (3 to 11)	5 (5 to 5)	383 (332 to 443)	144 (117 to 176)	208 (168 to 254)	18 (9 to 31)	13 (13 to 13)
Morocco	2009	149 (130 to 171)	57 (47 to 71)	80 (63 to 98)	6 (3 to 11)	5 (5 to 5)	409 (357 to 471)	158 (130 to 194)	219 (175 to 270)	17 (9 to 31)	15 (15 to 15)
Morocco	2010	152 (132 to 175)	60 (49 to 74)	83 (66 to 102)	6 (3 to 11)	2 (2 to 2)	418 (364 to 481)	166 (135 to 205)	228 (182 to 281)	17 (9 to 29)	7 (7 to 7)
Morocco	2011	162 (142 to 186)	66 (54 to 82)	87 (70 to 106)	6 (3 to 11)	3 (3 to 3)	446 (390 to 512)	182 (148 to 225)	239 (193 to 292)	17 (9 to 30)	8 (8 to 8)
Morocco	2012	165 (144 to 189)	69 (56 to 85)	88 (71 to 107)	6 (3 to 11)	2 (2 to 2)	455 (396 to 521)	191 (155 to 234)	243 (196 to 294)	17 (9 to 31)	5 (5 to 5)
Morocco	2013	168 (146 to 192)	69 (56 to 83)	87 (70 to 107)	6 (3 to 11)	6 (6 to 6)	463 (401 to 528)	189 (155 to 228)	240 (193 to 293)	17 (9 to 31)	17 (17 to 17)
Morocco	2014	167 (144 to 191)	68 (56 to 83)	88 (70 to 107)	6 (3 to 11)	6 (6 to 6)	460 (397 to 525)	187 (153 to 227)	241 (193 to 295)	16 (8 to 30)	16 (16 to 16)
Morocco	2015	161 (139 to 186)	67 (55 to 81)	87 (69 to 109)	5 (3 to 10)	2 (2 to 2)	442 (382 to 511)	183 (150 to 224)	240 (191 to 300)	15 (7 to 27)	4 (4 to 4)
Morocco	2016	169 (144 to 195)	68 (55 to 85)	88 (69 to 112)	5 (3 to 10)	7 (7 to 7)	464 (397 to 536)	188 (152 to 233)	244 (191 to 308)	15 (7 to 27)	18 (18 to 18)
Morocco	2017	171 (145 to 205)	71 (57 to 88)	90 (70 to 114)	5 (3 to 10)	5 (5 to 5)	471 (398 to 564)	194 (156 to 242)	249 (191 to 314)	15 (8 to 28)	13 (13 to 13)

Morocco	2018	174 (145 to 209)	72 (58 to 90)	92 (71 to 116)	6 (3 to 11)	4 (4 to 4)	480 (400 to 575)	199 (160 to 248)	253 (195 to 319)	16 (8 to 29)	12 (12 to 12)
Morocco	2019	179 (150 to 213)	75 (60 to 93)	94 (72 to 118)	6 (3 to 11)	5 (5 to 5)	492 (414 to 587)	206 (166 to 256)	257 (198 to 324)	16 (8 to 30)	12 (12 to 13)
Morocco	2020	184 (155 to 221)	77 (62 to 97)	96 (74 to 121)	6 (3 to 11)	5 (4 to 5)	506 (426 to 607)	213 (170 to 266)	264 (203 to 332)	16 (8 to 31)	13 (12 to 13)
Morocco	2021	188 (157 to 224)	79 (63 to 99)	98 (75 to 123)	6 (3 to 11)	5 (4 to 5)	516 (433 to 616)	218 (174 to 272)	269 (207 to 338)	17 (8 to 31)	13 (12 to 14)
Morocco	2022	191 (160 to 228)	81 (64 to 101)	99 (76 to 125)	6 (3 to 12)	5 (4 to 5)	526 (441 to 627)	222 (177 to 278)	274 (210 to 343)	17 (9 to 32)	13 (12 to 14)
Morocco	2023	195 (163 to 233)	83 (66 to 104)	101 (78 to 127)	6 (3 to 12)	5 (4 to 5)	536 (448 to 641)	227 (182 to 285)	278 (213 to 350)	18 (9 to 33)	13 (12 to 15)
Morocco	2024	198 (167 to 236)	84 (68 to 106)	103 (79 to 129)	7 (3 to 12)	5 (4 to 5)	546 (459 to 650)	232 (186 to 291)	283 (217 to 356)	18 (9 to 33)	13 (12 to 15)
Morocco	2025	203 (169 to 241)	87 (69 to 109)	104 (80 to 131)	7 (3 to 12)	5 (4 to 6)	558 (466 to 664)	238 (190 to 299)	287 (221 to 362)	18 (9 to 34)	14 (12 to 15)
Morocco	2026	207 (174 to 248)	89 (71 to 112)	106 (82 to 134)	7 (3 to 13)	5 (4 to 6)	570 (478 to 683)	245 (195 to 308)	292 (225 to 368)	19 (10 to 35)	14 (12 to 16)
Morocco	2027	211 (176 to 252)	92 (73 to 116)	108 (83 to 136)	7 (4 to 13)	5 (4 to 6)	582 (485 to 694)	252 (200 to 319)	296 (228 to 375)	19 (10 to 35)	14 (12 to 16)
Morocco	2028	216 (181 to 257)	94 (75 to 119)	109 (84 to 139)	7 (4 to 13)	5 (4 to 6)	594 (498 to 709)	260 (207 to 327)	301 (232 to 382)	20 (10 to 36)	14 (12 to 16)
Morocco	2029	221 (184 to 265)	97 (77 to 123)	111 (85 to 141)	7 (4 to 13)	5 (4 to 6)	607 (506 to 728)	267 (211 to 340)	305 (235 to 388)	20 (10 to 37)	14 (12 to 17)
Morocco	2030	225 (187 to 270)	100 (78 to 127)	113 (87 to 144)	7 (4 to 14)	5 (4 to 6)	620 (516 to 742)	275 (215 to 348)	310 (238 to 395)	20 (10 to 38)	14 (12 to 17)
Morocco	2031	230 (190 to 277)	103 (80 to 130)	114 (88 to 146)	8 (4 to 14)	5 (4 to 6)	633 (523 to 763)	283 (220 to 358)	314 (242 to 402)	21 (11 to 38)	15 (12 to 17)
Morocco	2032	235 (195 to 283)	106 (82 to 135)	116 (89 to 148)	8 (4 to 14)	5 (5 to 7)	646 (537 to 777)	291 (226 to 373)	319 (246 to 408)	21 (11 to 39)	15 (13 to 18)

Morocco	2033	239 (198 to 290)	108 (84 to 140)	118 (91 to 150)	8 (4 to 14)	6 (5 to 7)	659 (544 to 798)	298 (230 to 386)	323 (249 to 414)	22 (11 to 40)	15 (13 to 19)
Morocco	2034	244 (202 to 292)	111 (86 to 145)	119 (92 to 152)	8 (4 to 15)	6 (5 to 7)	672 (555 to 804)	306 (235 to 398)	328 (253 to 419)	22 (11 to 41)	15 (13 to 19)
Morocco	2035	249 (206 to 302)	114 (88 to 149)	121 (93 to 154)	8 (4 to 15)	6 (5 to 7)	685 (566 to 831)	314 (241 to 409)	332 (256 to 425)	23 (12 to 42)	16 (13 to 19)
Morocco	2036	254 (211 to 309)	117 (89 to 153)	122 (94 to 157)	8 (4 to 15)	6 (5 to 7)	698 (581 to 851)	322 (245 to 422)	337 (260 to 431)	23 (12 to 42)	16 (13 to 20)
Morocco	2037	258 (212 to 316)	120 (91 to 157)	124 (95 to 159)	9 (4 to 16)	6 (5 to 7)	711 (584 to 871)	330 (251 to 431)	341 (263 to 437)	24 (12 to 43)	16 (13 to 20)
Morocco	2038	263 (217 to 321)	123 (92 to 163)	125 (97 to 161)	9 (5 to 16)	6 (5 to 8)	725 (598 to 885)	339 (254 to 449)	345 (266 to 443)	24 (12 to 44)	17 (13 to 21)
Morocco	2039	269 (218 to 332)	126 (94 to 168)	127 (98 to 163)	9 (5 to 16)	6 (5 to 8)	739 (600 to 913)	348 (260 to 462)	350 (269 to 449)	25 (13 to 45)	17 (13 to 21)
Morocco	2040	274 (225 to 336)	130 (97 to 173)	129 (99 to 165)	9 (5 to 17)	6 (5 to 8)	754 (619 to 923)	357 (266 to 477)	355 (272 to 455)	25 (13 to 46)	17 (13 to 22)
Morocco	2041	280 (227 to 347)	133 (98 to 179)	131 (100 to 168)	9 (5 to 17)	6 (5 to 8)	770 (626 to 956)	367 (270 to 494)	359 (276 to 462)	26 (13 to 47)	17 (13 to 23)
Morocco	2042	285 (233 to 351)	137 (100 to 185)	132 (102 to 170)	10 (5 to 18)	6 (5 to 8)	785 (641 to 967)	377 (276 to 510)	364 (280 to 467)	26 (13 to 48)	18 (13 to 23)
Morocco	2043	291 (236 to 363)	141 (103 to 191)	134 (103 to 172)	10 (5 to 18)	7 (5 to 9)	801 (650 to 1000)	387 (283 to 526)	370 (283 to 473)	27 (14 to 49)	18 (14 to 24)
Morocco	2044	297 (241 to 367)	144 (105 to 197)	136 (104 to 174)	10 (5 to 18)	7 (5 to 9)	817 (662 to 1009)	397 (289 to 543)	374 (286 to 478)	28 (14 to 50)	18 (14 to 24)
Morocco	2045	303 (244 to 381)	148 (107 to 203)	138 (105 to 176)	10 (5 to 19)	7 (5 to 9)	834 (673 to 1050)	407 (294 to 558)	379 (289 to 484)	28 (14 to 51)	19 (14 to 25)
Morocco	2046	309 (248 to 384)	152 (108 to 210)	140 (106 to 178)	10 (5 to 19)	7 (5 to 9)	850 (682 to 1056)	418 (298 to 578)	384 (292 to 491)	29 (14 to 53)	19 (14 to 25)
Morocco	2047	315 (251 to 398)	156 (111 to 216)	141 (107 to 181)	11 (5 to 19)	7 (5 to 10)	867 (689 to 1094)	429 (305 to 595)	389 (296 to 498)	29 (15 to 54)	20 (14 to 26)

Morocco	2048	321 (256 to 400)	160 (113 to 222)	143 (109 to 183)	11 (5 to 20)	7 (5 to 10)	884 (704 to 1102)	440 (310 to 611)	394 (299 to 504)	30 (15 to 55)	20 (14 to 27)
Morocco	2049	328 (260 to 416)	164 (115 to 229)	145 (110 to 186)	11 (6 to 20)	7 (5 to 10)	901 (717 to 1145)	452 (316 to 631)	399 (303 to 511)	31 (15 to 56)	20 (15 to 28)
Morocco	2050	334 (264 to 420)	168 (116 to 236)	147 (112 to 188)	11 (6 to 21)	8 (5 to 10)	918 (726 to 1155)	463 (321 to 650)	404 (307 to 518)	31 (16 to 57)	21 (15 to 28)
Mozambique	1995	12 (11 to 14)	5 (4 to 6)	2 (1 to 2)	0 (0 to 0)	5 (5 to 5)	32 (29 to 37)	13 (10 to 17)	5 (3 to 7)	0 (0 to 1)	14 (14 to 14)
Mozambique	1996	14 (13 to 16)	5 (4 to 6)	2 (1 to 2)	0 (0 to 0)	8 (8 to 8)	39 (35 to 43)	13 (10 to 17)	5 (3 to 7)	0 (0 to 1)	21 (21 to 21)
Mozambique	1997	14 (12 to 15)	6 (4 to 7)	2 (1 to 3)	0 (0 to 0)	6 (6 to 6)	37 (33 to 42)	15 (11 to 19)	5 (3 to 7)	0 (0 to 1)	17 (17 to 17)
Mozambique	1998	13 (12 to 16)	6 (4 to 8)	2 (1 to 2)	0 (0 to 0)	6 (6 to 6)	36 (32 to 42)	16 (12 to 20)	5 (3 to 7)	0 (0 to 1)	16 (16 to 16)
Mozambique	1999	15 (13 to 17)	6 (5 to 8)	2 (1 to 2)	0 (0 to 0)	6 (6 to 6)	39 (35 to 45)	17 (13 to 22)	5 (3 to 7)	0 (0 to 1)	17 (17 to 17)
Mozambique	2000	16 (14 to 18)	7 (5 to 9)	2 (1 to 2)	0 (0 to 0)	7 (7 to 7)	43 (38 to 50)	19 (14 to 25)	5 (3 to 7)	0 (0 to 1)	19 (19 to 19)
Mozambique	2001	18 (16 to 21)	8 (6 to 11)	2 (1 to 3)	0 (0 to 0)	7 (7 to 7)	48 (42 to 56)	23 (17 to 30)	5 (3 to 7)	0 (0 to 1)	20 (20 to 20)
Mozambique	2002	19 (17 to 22)	8 (6 to 11)	2 (1 to 3)	0 (0 to 0)	9 (9 to 9)	52 (46 to 60)	22 (16 to 29)	5 (4 to 7)	0 (0 to 1)	24 (24 to 24)
Mozambique	2003	20 (18 to 23)	8 (6 to 11)	2 (1 to 3)	0 (0 to 0)	10 (10 to 10)	55 (49 to 63)	22 (17 to 30)	5 (4 to 8)	0 (0 to 1)	27 (27 to 27)
Mozambique	2004	23 (21 to 26)	8 (6 to 11)	2 (1 to 3)	0 (0 to 0)	13 (13 to 13)	62 (56 to 70)	22 (17 to 29)	5 (4 to 8)	0 (0 to 1)	34 (34 to 34)
Mozambique	2005	23 (20 to 25)	8 (6 to 10)	2 (1 to 3)	0 (0 to 0)	12 (12 to 12)	61 (55 to 68)	22 (16 to 28)	5 (4 to 8)	0 (0 to 1)	33 (33 to 33)
Mozambique	2006	24 (22 to 27)	8 (6 to 10)	2 (1 to 3)	0 (0 to 0)	14 (14 to 14)	65 (59 to 72)	22 (16 to 28)	6 (4 to 8)	1 (0 to 1)	37 (37 to 37)

Mozambique	2007	27 (25 to 29)	7 (5 to 9)	2 (1 to 3)	0 (0 to 0)	18 (18 to 18)	74 (69 to 79)	18 (14 to 23)	5 (4 to 8)	1 (0 to 1)	50 (50 to 50)
Mozambique	2008	30 (29 to 32)	5 (4 to 6)	2 (1 to 3)	0 (0 to 0)	23 (23 to 23)	81 (77 to 85)	13 (10 to 17)	5 (3 to 7)	1 (0 to 1)	62 (62 to 62)
Mozambique	2009	26 (25 to 28)	4 (3 to 6)	2 (1 to 3)	0 (0 to 1)	20 (20 to 20)	71 (68 to 75)	11 (9 to 15)	5 (3 to 7)	1 (0 to 1)	54 (54 to 54)
Mozambique	2010	27 (26 to 29)	4 (3 to 5)	2 (1 to 3)	0 (0 to 1)	21 (21 to 21)	73 (71 to 77)	10 (8 to 13)	5 (3 to 7)	1 (0 to 1)	58 (58 to 58)
Mozambique	2011	27 (26 to 28)	4 (3 to 5)	2 (1 to 2)	0 (0 to 1)	21 (21 to 21)	72 (69 to 76)	10 (8 to 14)	5 (3 to 7)	1 (0 to 2)	56 (56 to 56)
Mozambique	2012	29 (27 to 30)	5 (4 to 6)	2 (1 to 3)	0 (0 to 1)	22 (22 to 22)	77 (74 to 82)	13 (10 to 17)	5 (3 to 7)	1 (0 to 2)	58 (58 to 58)
Mozambique	2013	32 (30 to 34)	6 (5 to 8)	2 (1 to 3)	0 (0 to 1)	23 (23 to 23)	85 (81 to 91)	17 (13 to 22)	5 (4 to 7)	1 (1 to 2)	62 (62 to 62)
Mozambique	2014	39 (36 to 41)	8 (6 to 11)	2 (1 to 3)	1 (0 to 1)	28 (28 to 28)	104 (98 to 112)	23 (17 to 29)	6 (4 to 8)	1 (1 to 2)	74 (74 to 74)
Mozambique	2015	31 (29 to 34)	8 (6 to 10)	2 (1 to 3)	0 (0 to 1)	21 (21 to 21)	85 (79 to 91)	21 (17 to 28)	5 (4 to 8)	1 (1 to 2)	57 (57 to 57)
Mozambique	2016	33 (31 to 36)	8 (6 to 10)	2 (1 to 3)	0 (0 to 1)	23 (23 to 23)	90 (85 to 97)	21 (16 to 27)	5 (4 to 8)	1 (1 to 2)	63 (63 to 63)
Mozambique	2017	34 (32 to 36)	8 (6 to 10)	2 (1 to 3)	0 (0 to 1)	24 (24 to 24)	91 (85 to 98)	20 (16 to 27)	5 (4 to 8)	1 (1 to 2)	64 (64 to 64)
Mozambique	2018	33 (31 to 35)	8 (6 to 10)	2 (1 to 3)	0 (0 to 1)	22 (22 to 22)	88 (82 to 95)	21 (16 to 28)	5 (4 to 7)	1 (1 to 2)	61 (61 to 61)
Mozambique	2019	34 (32 to 37)	9 (7 to 12)	2 (1 to 3)	0 (0 to 1)	23 (23 to 23)	92 (85 to 100)	24 (18 to 33)	5 (4 to 7)	1 (1 to 2)	61 (61 to 61)
Mozambique	2020	34 (32 to 38)	9 (7 to 13)	2 (1 to 3)	0 (0 to 1)	23 (21 to 24)	93 (86 to 102)	25 (18 to 34)	5 (4 to 8)	1 (1 to 2)	61 (58 to 64)
Mozambique	2021	35 (31 to 39)	10 (7 to 13)	2 (1 to 3)	0 (0 to 1)	23 (21 to 24)	94 (85 to 105)	26 (19 to 36)	6 (4 to 8)	1 (1 to 2)	61 (57 to 66)

Mozambique	2022	35 (31 to 39)	10 (7 to 14)	2 (1 to 3)	0 (0 to 1)	22 (21 to 25)	95 (85 to 106)	27 (19 to 38)	6 (4 to 8)	1 (1 to 2)	61 (55 to 67)
Mozambique	2023	35 (31 to 41)	11 (7 to 15)	2 (2 to 3)	1 (0 to 1)	22 (20 to 25)	96 (84 to 109)	28 (19 to 39)	6 (4 to 8)	1 (1 to 3)	60 (54 to 67)
Mozambique	2024	36 (31 to 40)	11 (7 to 15)	2 (2 to 3)	1 (0 to 1)	22 (20 to 25)	96 (85 to 108)	29 (20 to 41)	6 (4 to 9)	1 (1 to 3)	59 (53 to 67)
Mozambique	2025	36 (31 to 41)	11 (8 to 15)	2 (2 to 3)	1 (0 to 1)	22 (19 to 24)	96 (84 to 111)	30 (20 to 42)	6 (4 to 9)	1 (1 to 3)	58 (52 to 66)
Mozambique	2026	36 (31 to 41)	11 (8 to 16)	2 (2 to 4)	1 (0 to 1)	21 (19 to 24)	96 (84 to 110)	31 (21 to 43)	7 (5 to 9)	2 (1 to 3)	58 (51 to 66)
Mozambique	2027	36 (31 to 42)	12 (8 to 16)	3 (2 to 4)	1 (0 to 1)	21 (18 to 24)	97 (84 to 113)	31 (21 to 43)	7 (5 to 10)	2 (1 to 3)	57 (50 to 66)
Mozambique	2028	36 (31 to 41)	12 (8 to 17)	3 (2 to 4)	1 (0 to 1)	21 (18 to 25)	97 (85 to 112)	32 (22 to 45)	7 (5 to 10)	2 (1 to 3)	57 (49 to 66)
Mozambique	2029	36 (31 to 42)	12 (8 to 17)	3 (2 to 4)	1 (0 to 1)	21 (18 to 25)	98 (84 to 115)	32 (22 to 46)	7 (5 to 10)	2 (1 to 3)	56 (48 to 66)
Mozambique	2030	36 (32 to 42)	12 (8 to 17)	3 (2 to 4)	1 (0 to 1)	21 (18 to 25)	98 (85 to 114)	33 (23 to 47)	8 (5 to 11)	2 (1 to 3)	56 (48 to 67)
Mozambique	2031	37 (31 to 43)	13 (9 to 18)	3 (2 to 4)	1 (0 to 1)	21 (17 to 25)	99 (83 to 117)	34 (23 to 48)	8 (5 to 11)	2 (1 to 4)	56 (47 to 67)
Mozambique	2032	37 (32 to 43)	13 (9 to 18)	3 (2 to 4)	1 (0 to 1)	20 (17 to 25)	100 (85 to 117)	35 (24 to 49)	8 (6 to 12)	2 (1 to 4)	55 (47 to 67)
Mozambique	2033	37 (32 to 45)	13 (9 to 19)	3 (2 to 4)	1 (0 to 1)	20 (17 to 25)	101 (86 to 121)	35 (24 to 50)	8 (6 to 12)	2 (1 to 4)	55 (46 to 67)
Mozambique	2034	38 (33 to 45)	13 (9 to 19)	3 (2 to 5)	1 (0 to 1)	20 (17 to 25)	102 (88 to 122)	36 (25 to 51)	9 (6 to 12)	2 (1 to 4)	55 (45 to 68)
Mozambique	2035	38 (32 to 45)	14 (9 to 20)	3 (2 to 5)	1 (0 to 2)	20 (17 to 25)	103 (87 to 123)	37 (25 to 53)	9 (6 to 13)	2 (1 to 4)	55 (45 to 68)
Mozambique	2036	39 (33 to 46)	14 (10 to 20)	3 (2 to 5)	1 (0 to 2)	20 (17 to 26)	105 (88 to 125)	38 (26 to 54)	9 (6 to 13)	2 (1 to 4)	55 (45 to 69)

Mozambique	2037	39 (33 to 47)	15 (10 to 21)	3 (2 to 5)	1 (0 to 2)	20 (16 to 25)	106 (89 to 128)	39 (27 to 56)	9 (6 to 13)	2 (1 to 4)	55 (44 to 68)
Mozambique	2038	40 (33 to 48)	15 (10 to 21)	4 (2 to 5)	1 (0 to 2)	20 (16 to 26)	108 (89 to 130)	40 (28 to 58)	10 (7 to 14)	2 (1 to 5)	55 (44 to 70)
Mozambique	2039	40 (33 to 49)	15 (10 to 22)	4 (3 to 5)	1 (0 to 2)	20 (16 to 26)	109 (90 to 133)	41 (28 to 60)	10 (7 to 14)	2 (1 to 5)	55 (44 to 70)
Mozambique	2040	41 (34 to 50)	16 (11 to 23)	4 (3 to 5)	1 (0 to 2)	21 (16 to 27)	111 (92 to 135)	43 (29 to 62)	10 (7 to 15)	3 (1 to 5)	55 (44 to 72)
Mozambique	2041	42 (34 to 52)	16 (11 to 24)	4 (3 to 6)	1 (0 to 2)	21 (16 to 26)	113 (92 to 139)	44 (30 to 65)	10 (7 to 15)	3 (1 to 5)	56 (44 to 71)
Mozambique	2042	43 (35 to 52)	17 (11 to 25)	4 (3 to 6)	1 (0 to 2)	21 (16 to 27)	115 (94 to 142)	46 (31 to 67)	11 (7 to 16)	3 (1 to 5)	56 (44 to 72)
Mozambique	2043	43 (35 to 54)	18 (12 to 26)	4 (3 to 6)	1 (1 to 2)	21 (16 to 27)	117 (94 to 146)	47 (32 to 70)	11 (8 to 16)	3 (1 to 5)	56 (44 to 73)
Mozambique	2044	44 (36 to 55)	18 (12 to 27)	4 (3 to 6)	1 (1 to 2)	21 (16 to 28)	120 (98 to 148)	49 (33 to 73)	11 (8 to 16)	3 (1 to 6)	56 (44 to 74)
Mozambique	2045	45 (37 to 57)	19 (13 to 28)	4 (3 to 6)	1 (1 to 2)	21 (16 to 28)	122 (99 to 153)	51 (34 to 75)	12 (8 to 17)	3 (1 to 6)	56 (43 to 74)
Mozambique	2046	46 (37 to 59)	20 (13 to 29)	4 (3 to 6)	1 (1 to 2)	21 (16 to 28)	125 (100 to 159)	53 (35 to 79)	12 (8 to 17)	3 (2 to 6)	57 (44 to 75)
Mozambique	2047	47 (38 to 60)	20 (13 to 31)	5 (3 to 7)	1 (1 to 2)	21 (16 to 29)	128 (103 to 162)	55 (36 to 83)	12 (9 to 18)	3 (2 to 6)	57 (44 to 78)
Mozambique	2048	48 (38 to 61)	21 (14 to 32)	5 (3 to 7)	1 (1 to 2)	21 (16 to 28)	131 (103 to 164)	57 (37 to 86)	13 (9 to 18)	3 (2 to 6)	57 (44 to 77)
Mozambique	2049	50 (40 to 63)	22 (14 to 34)	5 (3 to 7)	1 (1 to 2)	21 (17 to 29)	134 (108 to 171)	59 (39 to 91)	13 (9 to 19)	3 (2 to 7)	58 (45 to 79)
Mozambique	2050	51 (40 to 65)	23 (15 to 35)	5 (3 to 7)	1 (1 to 2)	22 (17 to 30)	137 (109 to 176)	62 (40 to 95)	13 (9 to 19)	4 (2 to 7)	58 (45 to 81)
Myanmar	1995	4 (3 to 5)	1 (1 to 1)	3 (2 to 5)	0 (0 to 0)	0 (0 to 0)	22 (16 to 29)	4 (3 to 5)	18 (12 to 25)	0 (0 to 0)	0 (0 to 0)

Myanmar	1996	4 (3 to 5)	1 (1 to 1)	3 (2 to 5)	0 (0 to 0)	0 (0 to 0)	22 (16 to 29)	4 (3 to 5)	18 (12 to 25)	0 (0 to 0)	0 (0 to 0)
Myanmar	1997	4 (3 to 5)	1 (0 to 1)	3 (2 to 5)	0 (0 to 0)	0 (0 to 0)	21 (16 to 28)	3 (3 to 4)	18 (12 to 24)	0 (0 to 0)	0 (0 to 0)
Myanmar	1998	4 (3 to 5)	1 (0 to 1)	3 (2 to 5)	0 (0 to 0)	0 (0 to 0)	22 (16 to 29)	3 (2 to 4)	18 (13 to 25)	0 (0 to 0)	0 (0 to 0)
Myanmar	1999	4 (3 to 6)	1 (0 to 1)	4 (3 to 5)	0 (0 to 0)	0 (0 to 0)	24 (18 to 32)	3 (2 to 4)	20 (14 to 28)	0 (0 to 0)	1 (1 to 1)
Myanmar	2000	5 (4 to 7)	1 (1 to 1)	4 (3 to 6)	0 (0 to 0)	0 (0 to 0)	28 (21 to 38)	4 (3 to 5)	24 (17 to 33)	0 (0 to 0)	1 (1 to 1)
Myanmar	2001	6 (4 to 8)	1 (1 to 1)	5 (4 to 7)	0 (0 to 0)	0 (0 to 0)	32 (24 to 43)	4 (3 to 5)	28 (20 to 39)	0 (0 to 0)	1 (1 to 1)
Myanmar	2002	7 (5 to 9)	1 (1 to 1)	6 (4 to 8)	0 (0 to 0)	0 (0 to 0)	38 (29 to 51)	4 (3 to 6)	32 (23 to 44)	0 (0 to 0)	2 (2 to 2)
Myanmar	2003	9 (7 to 11)	1 (1 to 1)	7 (5 to 9)	0 (0 to 0)	1 (1 to 1)	46 (36 to 59)	5 (4 to 6)	37 (27 to 51)	0 (0 to 0)	4 (4 to 4)
Myanmar	2004	9 (7 to 12)	1 (1 to 1)	8 (5 to 10)	0 (0 to 0)	1 (1 to 1)	50 (38 to 65)	5 (4 to 7)	41 (30 to 56)	0 (0 to 0)	4 (4 to 4)
Myanmar	2005	11 (8 to 14)	1 (1 to 1)	9 (6 to 12)	0 (0 to 0)	1 (1 to 1)	57 (44 to 74)	5 (4 to 7)	46 (33 to 62)	0 (0 to 0)	6 (6 to 6)
Myanmar	2006	11 (9 to 15)	1 (1 to 2)	10 (7 to 13)	0 (0 to 0)	1 (1 to 1)	62 (47 to 81)	7 (5 to 9)	52 (37 to 71)	0 (0 to 0)	3 (3 to 3)
Myanmar	2007	13 (10 to 17)	1 (1 to 2)	11 (8 to 14)	0 (0 to 0)	1 (1 to 1)	70 (53 to 91)	7 (5 to 9)	57 (41 to 78)	0 (0 to 0)	6 (6 to 6)
Myanmar	2008	13 (10 to 17)	1 (1 to 2)	11 (8 to 15)	0 (0 to 0)	1 (1 to 1)	72 (55 to 93)	7 (5 to 9)	59 (42 to 80)	0 (0 to 0)	6 (6 to 6)
Myanmar	2009	14 (11 to 18)	1 (1 to 2)	12 (8 to 16)	0 (0 to 0)	1 (1 to 1)	76 (59 to 99)	8 (6 to 10)	62 (44 to 84)	0 (0 to 0)	7 (7 to 7)
Myanmar	2010	16 (12 to 20)	2 (1 to 2)	12 (9 to 16)	0 (0 to 0)	2 (2 to 2)	84 (65 to 107)	9 (7 to 12)	66 (48 to 89)	0 (0 to 0)	8 (8 to 8)

Myanmar	2011	16 (13 to 21)	2 (2 to 3)	13 (10 to 18)	0 (0 to 0)	1 (1 to 1)	89 (69 to 113)	11 (9 to 15)	71 (51 to 96)	0 (0 to 0)	6 (6 to 6)
Myanmar	2012	23 (19 to 29)	3 (3 to 5)	17 (12 to 23)	0 (0 to 0)	3 (3 to 3)	126 (100 to 156)	19 (14 to 25)	92 (66 to 122)	0 (0 to 0)	15 (15 to 15)
Myanmar	2013	31 (24 to 38)	5 (4 to 7)	22 (16 to 29)	0 (0 to 0)	3 (3 to 3)	165 (132 to 205)	28 (21 to 36)	119 (87 to 158)	0 (0 to 0)	18 (18 to 18)
Myanmar	2014	40 (32 to 49)	7 (5 to 9)	28 (20 to 37)	0 (0 to 0)	5 (5 to 5)	215 (174 to 267)	39 (29 to 51)	151 (109 to 200)	0 (0 to 0)	25 (25 to 25)
Myanmar	2015	45 (36 to 57)	8 (6 to 11)	34 (24 to 45)	0 (0 to 0)	3 (3 to 3)	245 (196 to 307)	46 (34 to 59)	182 (131 to 244)	0 (0 to 0)	18 (18 to 18)
Myanmar	2016	50 (39 to 62)	8 (6 to 11)	37 (27 to 49)	0 (0 to 0)	5 (5 to 5)	268 (212 to 335)	44 (33 to 57)	198 (145 to 264)	0 (0 to 0)	26 (26 to 26)
Myanmar	2017	52 (41 to 66)	8 (6 to 11)	39 (28 to 53)	0 (0 to 0)	4 (4 to 4)	279 (220 to 357)	45 (34 to 58)	212 (153 to 285)	0 (0 to 0)	22 (22 to 22)
Myanmar	2018	54 (43 to 69)	9 (6 to 11)	42 (30 to 56)	0 (0 to 0)	4 (4 to 4)	293 (230 to 371)	47 (35 to 61)	225 (163 to 302)	0 (0 to 0)	21 (21 to 21)
Myanmar	2019	57 (44 to 73)	9 (7 to 12)	44 (32 to 59)	0 (0 to 0)	4 (4 to 4)	308 (240 to 393)	50 (37 to 66)	237 (171 to 318)	0 (0 to 0)	21 (21 to 21)
Myanmar	2020	60 (47 to 76)	10 (7 to 13)	46 (33 to 62)	0 (0 to 0)	4 (4 to 4)	323 (252 to 410)	53 (39 to 71)	249 (181 to 334)	0 (0 to 0)	21 (20 to 23)
Myanmar	2021	62 (49 to 79)	10 (7 to 14)	48 (35 to 64)	0 (0 to 0)	4 (4 to 4)	336 (262 to 425)	56 (40 to 75)	259 (187 to 347)	0 (0 to 0)	21 (20 to 23)
Myanmar	2022	65 (50 to 83)	11 (8 to 15)	50 (36 to 67)	0 (0 to 0)	4 (4 to 4)	349 (270 to 445)	59 (42 to 80)	269 (195 to 361)	0 (0 to 0)	22 (20 to 24)
Myanmar	2023	67 (52 to 85)	11 (8 to 16)	52 (37 to 70)	0 (0 to 0)	4 (4 to 4)	362 (281 to 460)	62 (43 to 84)	279 (202 to 376)	0 (0 to 0)	22 (19 to 24)
Myanmar	2024	70 (54 to 89)	12 (8 to 17)	54 (39 to 73)	0 (0 to 0)	4 (4 to 4)	376 (290 to 479)	65 (46 to 90)	290 (210 to 391)	0 (0 to 0)	21 (19 to 24)
Myanmar	2025	72 (56 to 92)	13 (9 to 18)	56 (41 to 75)	0 (0 to 0)	4 (4 to 5)	391 (303 to 497)	68 (47 to 95)	301 (219 to 406)	0 (0 to 0)	21 (19 to 24)

Myanmar	2026	75 (58 to 96)	13 (9 to 19)	58 (42 to 78)	0 (0 to 0)	4 (4 to 5)	406 (315 to 516)	72 (48 to 100)	313 (228 to 422)	0 (0 to 0)	21 (19 to 24)
Myanmar	2027	78 (61 to 99)	14 (9 to 20)	60 (44 to 81)	0 (0 to 0)	4 (3 to 5)	421 (327 to 536)	75 (50 to 107)	325 (236 to 437)	0 (0 to 0)	21 (19 to 25)
Myanmar	2028	81 (63 to 103)	15 (10 to 21)	62 (45 to 84)	0 (0 to 0)	4 (3 to 5)	437 (340 to 558)	79 (52 to 113)	337 (245 to 452)	0 (0 to 0)	22 (19 to 25)
Myanmar	2029	84 (65 to 107)	15 (10 to 22)	65 (47 to 87)	0 (0 to 0)	4 (3 to 5)	453 (351 to 576)	83 (54 to 118)	349 (254 to 467)	0 (0 to 0)	22 (19 to 25)
Myanmar	2030	87 (68 to 111)	16 (10 to 23)	67 (49 to 90)	0 (0 to 0)	4 (3 to 5)	470 (365 to 597)	86 (56 to 127)	362 (263 to 484)	0 (0 to 0)	22 (19 to 26)
Myanmar	2031	90 (69 to 115)	17 (11 to 25)	69 (50 to 93)	0 (0 to 0)	4 (3 to 5)	487 (374 to 621)	91 (58 to 134)	375 (272 to 503)	0 (0 to 0)	22 (19 to 26)
Myanmar	2032	94 (72 to 120)	18 (11 to 26)	72 (52 to 97)	0 (0 to 0)	4 (3 to 5)	505 (389 to 650)	95 (59 to 141)	388 (282 to 521)	0 (0 to 0)	22 (19 to 27)
Myanmar	2033	97 (75 to 124)	18 (11 to 27)	74 (54 to 100)	0 (0 to 0)	4 (3 to 5)	523 (402 to 671)	99 (62 to 148)	401 (291 to 539)	0 (0 to 0)	22 (19 to 27)
Myanmar	2034	100 (77 to 128)	19 (12 to 29)	77 (56 to 103)	0 (0 to 0)	4 (3 to 5)	541 (418 to 689)	104 (64 to 157)	415 (301 to 557)	0 (0 to 0)	22 (19 to 27)
Myanmar	2035	104 (79 to 132)	20 (13 to 30)	79 (58 to 107)	0 (0 to 0)	4 (3 to 5)	559 (428 to 714)	108 (68 to 164)	429 (311 to 576)	0 (0 to 0)	22 (19 to 27)
Myanmar	2036	107 (82 to 137)	21 (13 to 32)	82 (60 to 111)	0 (0 to 0)	4 (3 to 5)	579 (440 to 738)	113 (68 to 175)	443 (321 to 598)	0 (0 to 0)	23 (19 to 28)
Myanmar	2037	111 (84 to 142)	22 (13 to 34)	85 (62 to 114)	0 (0 to 0)	4 (3 to 5)	599 (454 to 765)	118 (70 to 185)	458 (332 to 617)	0 (0 to 0)	23 (19 to 28)
Myanmar	2038	115 (88 to 146)	23 (14 to 36)	88 (64 to 118)	0 (0 to 0)	4 (3 to 5)	620 (475 to 786)	124 (73 to 193)	473 (343 to 638)	0 (0 to 0)	23 (19 to 29)
Myanmar	2039	119 (90 to 151)	24 (14 to 38)	91 (66 to 122)	0 (0 to 0)	4 (4 to 5)	641 (487 to 813)	129 (75 to 208)	489 (355 to 660)	0 (0 to 0)	23 (19 to 29)
Myanmar	2040	123 (94 to 157)	25 (15 to 41)	94 (68 to 126)	0 (0 to 0)	4 (4 to 5)	664 (506 to 848)	135 (79 to 221)	506 (367 to 681)	0 (0 to 0)	23 (19 to 29)

Myanmar	2041	128 (96 to 164)	26 (15 to 42)	97 (70 to 131)	0 (0 to 0)	4 (4 to 6)	688 (519 to 883)	141 (80 to 228)	524 (380 to 705)	0 (0 to 0)	23 (19 to 30)
Myanmar	2042	132 (99 to 170)	27 (16 to 45)	101 (73 to 135)	0 (0 to 0)	4 (4 to 6)	714 (537 to 918)	148 (85 to 241)	542 (393 to 730)	0 (0 to 0)	24 (19 to 30)
Myanmar	2043	137 (104 to 176)	29 (16 to 47)	104 (75 to 140)	0 (0 to 0)	4 (4 to 6)	740 (560 to 948)	155 (88 to 252)	562 (406 to 757)	0 (0 to 0)	24 (19 to 31)
Myanmar	2044	142 (107 to 182)	30 (17 to 48)	108 (78 to 145)	0 (0 to 0)	4 (4 to 6)	768 (577 to 981)	162 (90 to 261)	582 (420 to 784)	0 (0 to 0)	24 (19 to 32)
Myanmar	2045	148 (110 to 187)	31 (17 to 51)	112 (81 to 151)	0 (0 to 0)	4 (4 to 6)	796 (593 to 1009)	169 (92 to 277)	602 (435 to 814)	0 (0 to 0)	24 (19 to 31)
Myanmar	2046	153 (114 to 194)	33 (18 to 53)	116 (83 to 156)	0 (0 to 0)	5 (4 to 6)	825 (617 to 1047)	177 (96 to 288)	624 (450 to 844)	0 (0 to 0)	24 (19 to 31)
Myanmar	2047	159 (118 to 203)	34 (18 to 57)	120 (86 to 162)	0 (0 to 0)	5 (4 to 6)	856 (637 to 1095)	185 (100 to 306)	646 (465 to 874)	0 (0 to 0)	24 (19 to 32)
Myanmar	2048	165 (121 to 210)	36 (19 to 59)	124 (89 to 168)	0 (0 to 0)	5 (4 to 6)	888 (654 to 1131)	194 (103 to 321)	669 (481 to 906)	0 (0 to 0)	25 (20 to 33)
Myanmar	2049	171 (126 to 219)	38 (20 to 63)	128 (92 to 174)	0 (0 to 0)	5 (4 to 6)	920 (678 to 1180)	202 (109 to 341)	693 (498 to 940)	0 (0 to 0)	25 (20 to 33)
Myanmar	2050	177 (132 to 229)	39 (21 to 66)	133 (96 to 181)	0 (0 to 0)	5 (4 to 6)	955 (710 to 1233)	212 (115 to 354)	718 (516 to 975)	0 (0 to 0)	25 (20 to 34)
Namibia	1995	383 (343 to 423)	207 (180 to 236)	33 (24 to 45)	135 (107 to 168)	7 (7 to 7)	762 (681 to 842)	411 (358 to 470)	67 (48 to 90)	269 (213 to 334)	15 (15 to 15)
Namibia	1996	385 (348 to 421)	210 (187 to 235)	34 (25 to 45)	137 (114 to 163)	5 (5 to 5)	766 (692 to 836)	417 (371 to 468)	67 (49 to 90)	273 (226 to 324)	10 (10 to 10)
Namibia	1997	392 (359 to 425)	211 (188 to 236)	33 (25 to 45)	139 (119 to 162)	8 (8 to 8)	780 (714 to 845)	420 (375 to 470)	66 (49 to 89)	276 (237 to 322)	17 (17 to 17)
Namibia	1998	396 (366 to 430)	215 (193 to 238)	33 (25 to 44)	143 (123 to 167)	5 (5 to 5)	788 (727 to 855)	427 (383 to 474)	66 (49 to 88)	285 (245 to 333)	10 (10 to 10)
Namibia	1999	389 (358 to 422)	210 (189 to 231)	32 (24 to 42)	142 (122 to 166)	5 (5 to 5)	773 (711 to 839)	417 (375 to 460)	64 (47 to 84)	283 (244 to 330)	9 (9 to 9)

Namibia	2000	376 (347 to 407)	201 (180 to 223)	30 (22 to 39)	140 (121 to 163)	5 (5 to 5)	747 (691 to 810)	399 (357 to 443)	60 (45 to 79)	278 (242 to 323)	10 (10 to 10)
Namibia	2001	384 (355 to 415)	206 (185 to 228)	30 (22 to 39)	142 (123 to 164)	7 (7 to 7)	764 (707 to 825)	410 (369 to 454)	59 (44 to 77)	282 (245 to 326)	13 (13 to 13)
Namibia	2002	395 (366 to 427)	214 (193 to 237)	31 (23 to 40)	143 (125 to 166)	7 (7 to 7)	786 (728 to 850)	425 (384 to 472)	61 (45 to 80)	285 (248 to 329)	15 (15 to 15)
Namibia	2003	410 (380 to 445)	221 (200 to 244)	32 (23 to 42)	146 (127 to 168)	11 (11 to 11)	815 (756 to 884)	440 (398 to 485)	63 (47 to 83)	291 (252 to 335)	22 (22 to 22)
Namibia	2004	416 (386 to 448)	220 (199 to 241)	32 (24 to 42)	145 (126 to 166)	19 (19 to 19)	827 (767 to 892)	437 (395 to 480)	64 (48 to 84)	288 (250 to 330)	38 (38 to 38)
Namibia	2005	411 (380 to 444)	214 (193 to 235)	33 (24 to 43)	143 (125 to 165)	22 (22 to 22)	818 (757 to 883)	425 (385 to 466)	65 (48 to 86)	285 (248 to 327)	43 (43 to 43)
Namibia	2006	430 (399 to 462)	209 (188 to 228)	33 (24 to 43)	145 (126 to 167)	43 (43 to 43)	855 (793 to 919)	415 (374 to 454)	65 (49 to 86)	289 (250 to 332)	86 (86 to 86)
Namibia	2007	440 (410 to 472)	204 (185 to 224)	34 (25 to 45)	148 (128 to 170)	53 (53 to 53)	875 (815 to 939)	407 (368 to 446)	68 (50 to 89)	295 (255 to 338)	106 (106 to 106)
Namibia	2008	445 (415 to 478)	205 (185 to 226)	37 (27 to 48)	155 (133 to 178)	49 (49 to 49)	886 (826 to 951)	407 (369 to 449)	73 (54 to 95)	308 (264 to 354)	98 (98 to 98)
Namibia	2009	476 (444 to 510)	199 (180 to 219)	40 (29 to 51)	161 (139 to 185)	76 (76 to 76)	946 (883 to 1015)	396 (358 to 436)	79 (58 to 102)	321 (276 to 369)	150 (150 to 150)
Namibia	2010	490 (457 to 524)	203 (184 to 224)	44 (33 to 57)	174 (151 to 198)	69 (69 to 69)	975 (908 to 1042)	404 (365 to 445)	88 (65 to 113)	346 (300 to 394)	137 (137 to 137)
Namibia	2011	518 (482 to 554)	221 (199 to 243)	49 (37 to 63)	199 (174 to 225)	49 (49 to 49)	1031 (960 to 1101)	440 (397 to 483)	98 (74 to 125)	396 (347 to 447)	97 (97 to 97)
Namibia	2012	529 (497 to 564)	214 (193 to 234)	49 (37 to 63)	201 (176 to 226)	65 (65 to 65)	1052 (988 to 1121)	425 (384 to 466)	98 (73 to 126)	400 (351 to 450)	129 (129 to 129)
Namibia	2013	529 (496 to 564)	222 (202 to 242)	48 (37 to 63)	209 (184 to 235)	49 (49 to 49)	1051 (986 to 1121)	441 (401 to 481)	96 (73 to 124)	416 (366 to 467)	98 (98 to 98)

Namibia	2014	570 (533 to 610)	245 (223 to 268)	48 (37 to 63)	234 (207 to 261)	43 (43 to 43)	1133 (1061 to 1213)	486 (443 to 534)	96 (73 to 125)	466 (412 to 520)	85 (85 to 85)
Namibia	2015	593 (551 to 636)	260 (235 to 288)	48 (36 to 63)	256 (228 to 285)	29 (29 to 29)	1179 (1095 to 1264)	517 (467 to 572)	95 (72 to 125)	510 (453 to 566)	57 (57 to 57)
Namibia	2016	578 (533 to 624)	254 (227 to 285)	46 (34 to 61)	241 (211 to 274)	37 (37 to 37)	1149 (1060 to 1241)	506 (451 to 567)	91 (67 to 121)	479 (419 to 545)	73 (73 to 73)
Namibia	2017	553 (488 to 629)	241 (212 to 274)	43 (31 to 58)	223 (187 to 268)	46 (46 to 46)	1100 (971 to 1252)	479 (423 to 546)	86 (63 to 115)	444 (371 to 533)	91 (91 to 91)
Namibia	2018	542 (478 to 617)	234 (206 to 267)	42 (31 to 57)	221 (185 to 266)	44 (44 to 45)	1077 (950 to 1226)	465 (410 to 530)	84 (61 to 113)	440 (368 to 528)	88 (88 to 89)
Namibia	2019	538 (474 to 613)	232 (203 to 266)	42 (30 to 56)	219 (183 to 263)	46 (45 to 46)	1070 (943 to 1219)	462 (404 to 530)	83 (60 to 111)	435 (364 to 522)	91 (90 to 91)
Namibia	2020	541 (475 to 617)	233 (203 to 266)	42 (30 to 56)	220 (184 to 264)	46 (44 to 49)	1075 (945 to 1227)	463 (403 to 530)	83 (60 to 111)	437 (366 to 526)	92 (87 to 97)
Namibia	2021	547 (480 to 626)	235 (204 to 270)	42 (31 to 56)	223 (187 to 269)	47 (44 to 51)	1088 (954 to 1245)	467 (405 to 536)	84 (61 to 112)	444 (372 to 534)	93 (88 to 101)
Namibia	2022	554 (484 to 635)	238 (206 to 273)	43 (31 to 57)	227 (190 to 273)	47 (43 to 52)	1102 (963 to 1264)	473 (409 to 543)	85 (61 to 113)	451 (377 to 543)	94 (86 to 104)
Namibia	2023	562 (492 to 643)	242 (209 to 277)	43 (31 to 57)	230 (192 to 278)	48 (43 to 53)	1118 (978 to 1278)	480 (415 to 551)	85 (62 to 114)	457 (383 to 552)	95 (86 to 106)

Namibia	2024	570 (499 to 654)	246 (212 to 284)	43 (32 to 58)	234 (195 to 282)	48 (43 to 54)	1134 (992 to 1302)	489 (422 to 564)	86 (63 to 116)	464 (389 to 561)	95 (85 to 107)
Namibia	2025	579 (507 to 666)	250 (215 to 290)	44 (32 to 59)	237 (198 to 287)	48 (43 to 54)	1151 (1009 to 1325)	497 (427 to 577)	87 (63 to 117)	471 (394 to 571)	95 (85 to 108)
Namibia	2026	588 (514 to 675)	255 (219 to 296)	44 (32 to 59)	241 (201 to 291)	48 (43 to 55)	1170 (1022 to 1343)	507 (436 to 588)	88 (64 to 118)	479 (400 to 580)	96 (85 to 109)
Namibia	2027	598 (521 to 689)	260 (223 to 302)	45 (33 to 60)	245 (204 to 296)	48 (43 to 56)	1189 (1037 to 1371)	517 (444 to 601)	89 (65 to 119)	487 (406 to 588)	96 (85 to 112)
Namibia	2028	608 (528 to 704)	265 (227 to 311)	45 (33 to 61)	249 (208 to 300)	49 (43 to 57)	1209 (1051 to 1401)	528 (452 to 618)	90 (66 to 121)	494 (414 to 596)	97 (85 to 113)
Namibia	2029	619 (538 to 717)	271 (232 to 318)	46 (33 to 61)	253 (211 to 305)	49 (43 to 58)	1231 (1070 to 1425)	539 (461 to 633)	91 (66 to 122)	503 (420 to 607)	98 (85 to 115)
Namibia	2030	630 (546 to 729)	277 (236 to 325)	46 (34 to 62)	257 (215 to 311)	49 (42 to 59)	1253 (1085 to 1450)	551 (470 to 646)	92 (67 to 123)	511 (427 to 618)	98 (84 to 118)
Namibia	2031	641 (555 to 744)	283 (240 to 333)	47 (34 to 63)	261 (218 to 317)	50 (43 to 59)	1275 (1103 to 1479)	563 (477 to 662)	93 (68 to 125)	519 (433 to 630)	99 (85 to 118)
Namibia	2032	652 (564 to 758)	290 (246 to 342)	47 (34 to 63)	265 (221 to 323)	50 (43 to 61)	1298 (1121 to 1508)	576 (489 to 680)	94 (68 to 126)	528 (440 to 642)	100 (85 to 121)
Namibia	2033	665 (574 to 773)	297 (252 to 350)	48 (35 to 64)	270 (225 to 328)	51 (43 to 61)	1323 (1141 to 1536)	590 (502 to 697)	95 (69 to 128)	537 (448 to 652)	101 (86 to 122)

Namibia	2034	680 (586 to 793)	305 (258 to 362)	49 (35 to 65)	275 (229 to 335)	51 (43 to 62)	1351 (1164 to 1576)	606 (513 to 720)	97 (70 to 130)	547 (456 to 665)	102 (85 to 123)
Namibia	2035	694 (597 to 807)	313 (266 to 371)	49 (36 to 66)	280 (233 to 341)	51 (43 to 63)	1380 (1188 to 1605)	623 (528 to 737)	98 (71 to 131)	557 (464 to 678)	102 (85 to 125)
Namibia	2036	708 (607 to 824)	321 (271 to 380)	50 (36 to 67)	285 (237 to 348)	52 (43 to 65)	1408 (1208 to 1639)	639 (540 to 755)	99 (72 to 133)	567 (471 to 691)	103 (85 to 129)
Namibia	2037	723 (622 to 840)	330 (278 to 391)	51 (37 to 68)	290 (240 to 354)	52 (43 to 64)	1437 (1237 to 1671)	655 (553 to 777)	101 (73 to 134)	577 (478 to 704)	104 (86 to 127)
Namibia	2038	737 (630 to 859)	338 (284 to 398)	51 (37 to 68)	295 (244 to 360)	53 (43 to 66)	1467 (1254 to 1709)	673 (565 to 792)	102 (74 to 136)	587 (485 to 716)	105 (86 to 132)
Namibia	2039	752 (644 to 877)	347 (291 to 411)	52 (38 to 69)	300 (247 to 368)	53 (44 to 67)	1496 (1281 to 1744)	690 (579 to 817)	103 (75 to 138)	597 (491 to 731)	106 (87 to 134)
Namibia	2040	768 (655 to 900)	356 (298 to 423)	52 (38 to 70)	305 (251 to 374)	54 (44 to 68)	1526 (1303 to 1790)	708 (592 to 842)	104 (76 to 140)	607 (499 to 744)	107 (87 to 135)
Namibia	2041	784 (668 to 919)	366 (304 to 435)	53 (38 to 71)	311 (255 to 381)	54 (44 to 68)	1559 (1329 to 1827)	727 (605 to 864)	106 (76 to 141)	618 (506 to 757)	108 (88 to 136)
Namibia	2042	800 (685 to 945)	375 (311 to 449)	54 (39 to 72)	316 (259 to 387)	55 (45 to 69)	1590 (1362 to 1879)	746 (619 to 894)	107 (77 to 143)	628 (514 to 770)	109 (89 to 138)
Namibia	2043	816 (693 to 965)	385 (319 to 461)	54 (39 to 73)	321 (263 to 394)	55 (45 to 72)	1622 (1379 to 1918)	766 (634 to 917)	108 (78 to 145)	638 (522 to 783)	110 (89 to 143)

Namibia	2044	832 (707 to 988)	395 (324 to 472)	55 (40 to 74)	326 (267 to 401)	56 (45 to 74)	1655 (1406 to 1964)	786 (645 to 939)	109 (79 to 147)	649 (530 to 796)	111 (90 to 147)
Namibia	2045	849 (719 to 1006)	405 (332 to 486)	56 (40 to 75)	332 (270 to 407)	56 (45 to 72)	1689 (1429 to 2001)	806 (659 to 967)	111 (80 to 149)	660 (538 to 810)	112 (89 to 144)
Namibia	2046	866 (730 to 1032)	416 (339 to 502)	56 (41 to 75)	337 (274 to 414)	57 (45 to 73)	1722 (1452 to 2053)	827 (675 to 999)	112 (81 to 150)	670 (544 to 824)	113 (90 to 145)
Namibia	2047	883 (743 to 1052)	426 (347 to 517)	57 (41 to 76)	342 (277 to 421)	58 (46 to 76)	1756 (1477 to 2092)	847 (691 to 1028)	113 (82 to 152)	681 (550 to 838)	115 (91 to 151)
Namibia	2048	900 (755 to 1079)	437 (355 to 533)	57 (41 to 77)	348 (280 to 428)	58 (47 to 77)	1790 (1501 to 2145)	869 (706 to 1060)	114 (82 to 153)	691 (557 to 851)	116 (93 to 153)
Namibia	2049	917 (767 to 1097)	448 (363 to 543)	58 (42 to 78)	353 (283 to 436)	59 (47 to 78)	1824 (1525 to 2182)	891 (722 to 1079)	115 (83 to 155)	701 (563 to 866)	117 (93 to 155)
Namibia	2050	935 (782 to 1124)	459 (373 to 561)	58 (42 to 79)	358 (287 to 443)	60 (47 to 81)	1860 (1556 to 2236)	914 (742 to 1116)	116 (84 to 156)	711 (571 to 881)	119 (94 to 160)
Nepal	1995	20 (15 to 25)	4 (3 to 5)	13 (9 to 19)	2 (1 to 3)	1 (1 to 1)	65 (49 to 82)	13 (10 to 17)	43 (30 to 60)	6 (3 to 11)	2 (2 to 2)
Nepal	1996	21 (16 to 27)	4 (3 to 5)	14 (10 to 20)	2 (1 to 3)	1 (1 to 1)	68 (53 to 87)	13 (10 to 18)	46 (32 to 64)	6 (3 to 11)	3 (3 to 3)
Nepal	1997	23 (18 to 29)	4 (3 to 6)	15 (10 to 21)	2 (1 to 3)	2 (2 to 2)	73 (57 to 94)	14 (10 to 18)	48 (33 to 67)	6 (3 to 11)	6 (6 to 6)
Nepal	1998	23 (18 to 30)	4 (3 to 6)	15 (10 to 21)	2 (1 to 3)	2 (2 to 2)	76 (59 to 96)	14 (11 to 19)	48 (34 to 68)	6 (3 to 11)	7 (7 to 7)
Nepal	1999	22 (18 to 28)	4 (3 to 5)	14 (10 to 20)	2 (1 to 3)	2 (2 to 2)	72 (57 to 92)	13 (10 to 18)	46 (32 to 64)	6 (3 to 11)	7 (7 to 7)

Nepal	2000	20 (16 to 26)	4 (3 to 5)	13 (9 to 18)	2 (1 to 3)	2 (2 to 2)	66 (52 to 82)	13 (9 to 16)	42 (29 to 58)	5 (2 to 10)	6 (6 to 6)
Nepal	2001	22 (18 to 28)	4 (3 to 6)	13 (9 to 19)	2 (1 to 3)	2 (2 to 2)	71 (57 to 89)	14 (10 to 18)	43 (30 to 60)	6 (3 to 11)	8 (8 to 8)
Nepal	2002	22 (18 to 27)	5 (3 to 6)	14 (10 to 19)	2 (1 to 3)	2 (2 to 2)	71 (57 to 89)	15 (11 to 19)	44 (31 to 61)	6 (3 to 11)	7 (7 to 7)
Nepal	2003	23 (18 to 28)	4 (3 to 6)	14 (10 to 19)	2 (1 to 3)	3 (3 to 3)	73 (60 to 91)	14 (11 to 18)	44 (31 to 61)	6 (3 to 11)	10 (10 to 10)
Nepal	2004	23 (18 to 28)	5 (3 to 6)	14 (10 to 19)	2 (1 to 3)	3 (3 to 3)	73 (59 to 90)	15 (11 to 19)	44 (31 to 61)	6 (3 to 11)	8 (8 to 8)
Nepal	2005	23 (19 to 29)	5 (4 to 6)	14 (10 to 19)	2 (1 to 4)	3 (3 to 3)	75 (61 to 93)	16 (12 to 20)	44 (31 to 61)	6 (3 to 12)	10 (10 to 10)
Nepal	2006	23 (19 to 28)	5 (4 to 6)	13 (9 to 18)	2 (1 to 3)	3 (3 to 3)	75 (61 to 91)	16 (12 to 20)	43 (30 to 59)	6 (3 to 11)	10 (10 to 10)
Nepal	2007	25 (21 to 31)	5 (4 to 7)	15 (11 to 21)	2 (1 to 4)	3 (3 to 3)	81 (67 to 100)	17 (13 to 22)	49 (35 to 66)	6 (3 to 12)	10 (10 to 10)
Nepal	2008	28 (24 to 34)	5 (4 to 7)	16 (12 to 22)	2 (1 to 4)	5 (5 to 5)	92 (76 to 110)	17 (13 to 22)	53 (38 to 71)	7 (3 to 12)	15 (15 to 15)
Nepal	2009	32 (26 to 38)	6 (5 to 8)	18 (13 to 25)	3 (1 to 5)	4 (4 to 4)	102 (84 to 122)	21 (16 to 26)	60 (43 to 79)	9 (4 to 17)	13 (13 to 13)
Nepal	2010	35 (29 to 42)	6 (5 to 8)	20 (14 to 26)	3 (2 to 6)	5 (5 to 5)	112 (94 to 134)	21 (16 to 27)	64 (46 to 85)	11 (5 to 21)	16 (16 to 16)
Nepal	2011	36 (30 to 44)	7 (5 to 8)	21 (15 to 28)	4 (2 to 7)	4 (4 to 4)	117 (97 to 142)	21 (16 to 27)	68 (49 to 90)	13 (6 to 24)	14 (14 to 14)
Nepal	2012	38 (32 to 47)	7 (5 to 9)	23 (17 to 31)	4 (2 to 8)	4 (4 to 4)	124 (102 to 153)	23 (17 to 29)	74 (54 to 98)	14 (7 to 26)	13 (13 to 13)
Nepal	2013	41 (33 to 50)	7 (5 to 9)	24 (18 to 33)	4 (2 to 7)	5 (5 to 5)	131 (108 to 162)	23 (17 to 29)	78 (57 to 105)	13 (6 to 24)	17 (17 to 17)
Nepal	2014	45 (37 to 55)	8 (6 to 10)	27 (19 to 36)	4 (2 to 8)	6 (6 to 6)	146 (120 to 178)	25 (19 to 32)	86 (63 to 116)	14 (7 to 27)	20 (20 to 20)

Nepal	2015	47 (38 to 58)	9 (7 to 11)	28 (21 to 38)	5 (2 to 9)	5 (5 to 5)	152 (124 to 186)	28 (21 to 35)	91 (67 to 122)	16 (8 to 31)	17 (17 to 17)
Nepal	2016	47 (38 to 58)	9 (7 to 12)	29 (21 to 39)	5 (2 to 10)	4 (4 to 4)	151 (123 to 186)	30 (23 to 39)	93 (68 to 124)	16 (8 to 31)	12 (12 to 12)
Nepal	2017	50 (40 to 63)	11 (8 to 14)	31 (22 to 41)	5 (2 to 9)	4 (4 to 4)	162 (128 to 204)	36 (27 to 46)	99 (71 to 134)	15 (7 to 28)	12 (12 to 12)
Nepal	2018	53 (43 to 67)	13 (10 to 16)	32 (23 to 44)	5 (2 to 10)	3 (3 to 3)	172 (137 to 217)	41 (31 to 53)	104 (75 to 141)	17 (8 to 31)	11 (11 to 11)
Nepal	2019	55 (43 to 69)	13 (10 to 16)	34 (24 to 46)	5 (2 to 10)	3 (3 to 3)	177 (140 to 222)	41 (31 to 52)	109 (79 to 148)	17 (8 to 32)	11 (10 to 11)
Nepal	2020	58 (45 to 72)	13 (10 to 17)	35 (26 to 48)	5 (3 to 10)	3 (3 to 3)	186 (146 to 234)	43 (33 to 55)	114 (83 to 155)	18 (8 to 33)	11 (10 to 11)
Nepal	2021	60 (47 to 75)	14 (10 to 18)	36 (27 to 49)	6 (3 to 11)	3 (3 to 4)	192 (153 to 243)	45 (34 to 58)	118 (86 to 160)	18 (9 to 35)	11 (10 to 12)
Nepal	2022	62 (49 to 77)	15 (11 to 19)	38 (27 to 51)	6 (3 to 11)	3 (3 to 4)	199 (160 to 249)	47 (35 to 61)	121 (88 to 165)	19 (9 to 36)	11 (10 to 12)
Nepal	2023	64 (51 to 79)	15 (11 to 20)	39 (28 to 53)	6 (3 to 12)	3 (3 to 4)	205 (163 to 256)	49 (37 to 63)	125 (91 to 170)	20 (9 to 37)	11 (10 to 12)
Nepal	2024	66 (53 to 82)	16 (12 to 21)	40 (29 to 54)	6 (3 to 12)	3 (3 to 4)	212 (170 to 266)	51 (38 to 66)	129 (94 to 175)	21 (10 to 39)	11 (10 to 12)
Nepal	2025	68 (54 to 85)	17 (12 to 21)	41 (30 to 56)	7 (3 to 12)	3 (3 to 4)	219 (173 to 273)	54 (40 to 69)	133 (96 to 180)	22 (10 to 40)	11 (9 to 12)
Nepal	2026	70 (55 to 88)	17 (13 to 22)	42 (31 to 57)	7 (3 to 13)	3 (3 to 4)	226 (178 to 285)	56 (41 to 72)	137 (99 to 185)	22 (10 to 42)	11 (9 to 12)
Nepal	2027	72 (57 to 91)	18 (13 to 23)	44 (32 to 59)	7 (3 to 13)	3 (3 to 4)	233 (184 to 293)	58 (43 to 76)	141 (102 to 191)	23 (11 to 44)	11 (9 to 13)
Nepal	2028	74 (59 to 95)	19 (14 to 24)	45 (33 to 61)	7 (3 to 14)	3 (3 to 4)	240 (190 to 306)	60 (45 to 78)	145 (105 to 196)	24 (11 to 45)	11 (9 to 13)
Nepal	2029	77 (60 to 97)	19 (14 to 25)	46 (34 to 62)	8 (4 to 15)	3 (3 to 4)	248 (195 to 312)	63 (47 to 81)	149 (108 to 201)	25 (12 to 47)	11 (9 to 13)

Nepal	2030	79 (62 to 100)	20 (15 to 26)	47 (35 to 64)	8 (4 to 15)	3 (3 to 4)	255 (199 to 321)	65 (49 to 84)	153 (112 to 207)	26 (12 to 49)	11 (9 to 13)
Nepal	2031	82 (63 to 103)	21 (16 to 27)	49 (36 to 66)	8 (4 to 16)	3 (3 to 4)	263 (204 to 333)	68 (51 to 87)	157 (115 to 212)	27 (12 to 50)	11 (9 to 14)
Nepal	2032	84 (64 to 106)	22 (16 to 28)	50 (37 to 68)	9 (4 to 16)	4 (3 to 4)	271 (208 to 342)	70 (52 to 91)	162 (118 to 218)	28 (13 to 52)	11 (10 to 14)
Nepal	2033	87 (66 to 110)	23 (17 to 29)	51 (38 to 70)	9 (4 to 17)	4 (3 to 4)	280 (214 to 355)	73 (54 to 95)	166 (121 to 224)	29 (13 to 54)	12 (10 to 14)
Nepal	2034	89 (69 to 113)	24 (17 to 31)	53 (39 to 71)	9 (4 to 17)	4 (3 to 4)	288 (224 to 366)	76 (56 to 99)	171 (125 to 231)	30 (14 to 56)	12 (10 to 14)
Nepal	2035	92 (72 to 116)	24 (18 to 32)	54 (40 to 73)	10 (4 to 18)	4 (3 to 4)	297 (232 to 375)	79 (58 to 102)	175 (128 to 237)	31 (14 to 58)	12 (10 to 14)
Nepal	2036	95 (74 to 120)	25 (19 to 33)	56 (41 to 75)	10 (5 to 18)	4 (3 to 5)	306 (237 to 388)	82 (60 to 107)	180 (132 to 243)	32 (15 to 59)	12 (10 to 15)
Nepal	2037	98 (76 to 126)	26 (19 to 34)	57 (42 to 78)	10 (5 to 19)	4 (3 to 5)	316 (245 to 406)	85 (63 to 111)	185 (135 to 250)	33 (15 to 61)	12 (10 to 15)
Nepal	2038	101 (79 to 127)	27 (20 to 36)	59 (43 to 80)	11 (5 to 20)	4 (3 to 5)	325 (255 to 411)	88 (64 to 116)	190 (139 to 257)	34 (16 to 64)	12 (10 to 16)
Nepal	2039	104 (81 to 132)	28 (20 to 37)	61 (44 to 82)	11 (5 to 20)	4 (3 to 5)	335 (260 to 427)	92 (66 to 119)	195 (142 to 264)	35 (16 to 66)	12 (10 to 16)
Nepal	2040	107 (83 to 136)	29 (21 to 38)	62 (45 to 84)	11 (5 to 21)	4 (3 to 5)	344 (267 to 438)	95 (69 to 124)	200 (146 to 271)	36 (17 to 68)	13 (10 to 16)
Nepal	2041	110 (86 to 138)	30 (22 to 40)	64 (46 to 86)	12 (5 to 22)	4 (3 to 5)	354 (278 to 446)	98 (72 to 129)	205 (149 to 277)	38 (17 to 70)	13 (10 to 16)
Nepal	2042	113 (88 to 144)	32 (23 to 41)	65 (47 to 88)	12 (6 to 23)	4 (3 to 5)	364 (284 to 465)	102 (74 to 133)	211 (153 to 284)	39 (18 to 73)	13 (10 to 17)
Nepal	2043	116 (90 to 147)	33 (24 to 43)	67 (48 to 90)	12 (6 to 23)	4 (3 to 5)	374 (291 to 475)	105 (76 to 138)	216 (156 to 291)	40 (19 to 75)	13 (10 to 17)
Nepal	2044	119 (93 to 152)	34 (24 to 44)	68 (50 to 92)	13 (6 to 24)	4 (3 to 5)	384 (300 to 490)	109 (78 to 143)	221 (160 to 298)	41 (19 to 78)	13 (10 to 18)

Nepal	2045	122 (95 to 156)	35 (25 to 46)	70 (51 to 95)	13 (6 to 25)	4 (3 to 6)	395 (306 to 503)	113 (80 to 148)	226 (164 to 305)	43 (20 to 81)	14 (11 to 18)
Nepal	2046	126 (98 to 158)	36 (26 to 47)	72 (52 to 97)	14 (6 to 26)	4 (3 to 6)	405 (318 to 511)	116 (83 to 153)	231 (168 to 313)	44 (20 to 83)	14 (11 to 18)
Nepal	2047	129 (101 to 162)	37 (27 to 49)	73 (53 to 99)	14 (7 to 27)	4 (3 to 6)	416 (325 to 523)	120 (86 to 158)	237 (172 to 320)	45 (21 to 86)	14 (11 to 19)
Nepal	2048	132 (104 to 168)	38 (27 to 51)	75 (54 to 101)	14 (7 to 27)	4 (3 to 6)	427 (336 to 542)	124 (88 to 163)	242 (176 to 327)	46 (22 to 88)	14 (11 to 19)
Nepal	2049	135 (104 to 173)	40 (28 to 52)	77 (56 to 104)	15 (7 to 28)	5 (4 to 6)	437 (337 to 557)	128 (91 to 168)	247 (179 to 334)	48 (22 to 90)	15 (11 to 20)
Nepal	2050	139 (106 to 176)	41 (29 to 53)	78 (57 to 106)	15 (7 to 29)	5 (4 to 6)	448 (342 to 568)	132 (93 to 173)	253 (183 to 341)	49 (23 to 93)	15 (12 to 21)
Netherlands	1995	3920 (3780 to 4064)	2440 (2331 to 2551)	485 (436 to 536)	995 (911 to 1087)	0 (0 to 0)	4385 (4228 to 4546)	2729 (2607 to 2854)	543 (488 to 600)	1113 (1019 to 1216)	0 (0 to 0)
Netherlands	1996	3775 (3670 to 3884)	2370 (2293 to 2449)	489 (450 to 529)	916 (857 to 981)	0 (0 to 0)	4222 (4106 to 4345)	2651 (2565 to 2740)	547 (503 to 592)	1025 (959 to 1098)	0 (0 to 0)
Netherlands	1997	3780 (3681 to 3892)	2410 (2338 to 2486)	484 (449 to 520)	885 (827 to 945)	0 (0 to 0)	4228 (4117 to 4354)	2696 (2615 to 2781)	542 (502 to 582)	990 (925 to 1057)	0 (0 to 0)
Netherlands	1998	3703 (3605 to 3806)	2414 (2341 to 2490)	457 (424 to 492)	832 (777 to 889)	0 (0 to 0)	4142 (4032 to 4257)	2700 (2619 to 2785)	511 (474 to 550)	931 (869 to 994)	0 (0 to 0)
Netherlands	1999	3609 (3516 to 3703)	2408 (2335 to 2484)	424 (394 to 456)	777 (721 to 832)	0 (0 to 0)	4037 (3933 to 4142)	2694 (2612 to 2779)	474 (440 to 510)	869 (807 to 931)	0 (0 to 0)
Netherlands	2000	3536 (3442 to 3631)	2418 (2343 to 2490)	395 (366 to 425)	723 (671 to 774)	0 (0 to 0)	3956 (3850 to 4062)	2705 (2621 to 2786)	442 (409 to 475)	809 (750 to 866)	0 (0 to 0)

Netherlands	2001	3618 (3526 to 3715)	2511 (2437 to 2584)	384 (356 to 414)	724 (674 to 774)	0 (0 to 0)	4047 (3944 to 4155)	2809 (2726 to 2891)	429 (398 to 463)	809 (754 to 866)	0 (0 to 0)
Netherlands	2002	3786 (3692 to 3886)	2643 (2568 to 2721)	382 (354 to 410)	760 (710 to 814)	0 (0 to 0)	4235 (4130 to 4347)	2957 (2872 to 3043)	427 (396 to 459)	851 (794 to 911)	0 (0 to 0)
Netherlands	2003	3943 (3849 to 4042)	2750 (2675 to 2832)	385 (358 to 413)	808 (755 to 863)	0 (0 to 0)	4411 (4306 to 4522)	3077 (2992 to 3168)	431 (400 to 462)	904 (844 to 966)	0 (0 to 0)
Netherlands	2004	4035 (3941 to 4139)	2796 (2724 to 2872)	388 (361 to 414)	852 (795 to 911)	0 (0 to 0)	4514 (4408 to 4629)	3128 (3047 to 3212)	434 (403 to 464)	953 (889 to 1019)	0 (0 to 0)
Netherlands	2005	4111 (4015 to 4208)	2841 (2767 to 2919)	385 (357 to 412)	885 (834 to 940)	0 (0 to 0)	4599 (4492 to 4707)	3178 (3095 to 3265)	431 (400 to 461)	990 (933 to 1051)	0 (0 to 0)
Netherlands	2006	4287 (4191 to 4382)	2964 (2887 to 3039)	382 (356 to 407)	942 (888 to 995)	0 (0 to 0)	4796 (4688 to 4901)	3316 (3229 to 3399)	427 (399 to 456)	1053 (993 to 1113)	0 (0 to 0)
Netherlands	2007	4406 (4302 to 4507)	3017 (2941 to 3092)	388 (362 to 415)	1001 (947 to 1058)	0 (0 to 0)	4928 (4812 to 5041)	3375 (3289 to 3459)	434 (405 to 464)	1120 (1059 to 1183)	0 (0 to 0)
Netherlands	2008	4551 (4451 to 4652)	3068 (2994 to 3148)	414 (388 to 442)	1069 (1015 to 1126)	0 (0 to 0)	5091 (4979 to 5204)	3432 (3349 to 3521)	463 (434 to 494)	1195 (1135 to 1259)	0 (0 to 0)
Netherlands	2009	4753 (4653 to 4854)	3182 (3109 to 3261)	433 (406 to 460)	1138 (1082 to 1195)	0 (0 to 0)	5317 (5205 to 5429)	3560 (3478 to 3648)	484 (454 to 515)	1273 (1211 to 1336)	0 (0 to 0)
Netherlands	2010	4866 (4761 to 4970)	3257 (3175 to 3335)	452 (426 to 480)	1157 (1099 to 1221)	0 (0 to 0)	5443 (5326 to 5560)	3643 (3552 to 3730)	506 (477 to 537)	1294 (1229 to 1365)	0 (0 to 0)

Netherlands	2011	4913 (4807 to 5020)	3293 (3211 to 3368)	478 (450 to 507)	1142 (1083 to 1202)	0 (0 to 0)	5496 (5377 to 5615)	3684 (3592 to 3767)	535 (504 to 568)	1277 (1211 to 1345)	0 (0 to 0)
Netherlands	2012	4987 (4885 to 5090)	3348 (3272 to 3425)	516 (487 to 549)	1123 (1065 to 1176)	0 (0 to 0)	5578 (5464 to 5693)	3745 (3660 to 3831)	577 (544 to 614)	1256 (1191 to 1316)	0 (0 to 0)
Netherlands	2013	5055 (4953 to 5160)	3403 (3321 to 3487)	557 (527 to 594)	1096 (1037 to 1154)	0 (0 to 0)	5655 (5540 to 5772)	3807 (3715 to 3900)	623 (590 to 664)	1225 (1160 to 1291)	0 (0 to 0)
Netherlands	2014	5020 (4917 to 5123)	3358 (3273 to 3442)	573 (542 to 607)	1089 (1030 to 1149)	0 (0 to 0)	5615 (5500 to 5731)	3757 (3661 to 3850)	641 (606 to 679)	1218 (1152 to 1285)	0 (0 to 0)
Netherlands	2015	5047 (4941 to 5154)	3318 (3240 to 3398)	576 (544 to 610)	1153 (1097 to 1214)	0 (0 to 0)	5645 (5527 to 5766)	3712 (3625 to 3801)	644 (609 to 683)	1289 (1227 to 1358)	0 (0 to 0)
Netherlands	2016	5065 (4960 to 5179)	3283 (3199 to 3365)	574 (541 to 611)	1208 (1150 to 1270)	0 (0 to 0)	5666 (5548 to 5794)	3672 (3578 to 3764)	643 (605 to 683)	1351 (1286 to 1421)	0 (0 to 0)
Netherlands	2017	5143 (4950 to 5341)	3305 (3195 to 3416)	574 (533 to 621)	1264 (1190 to 1348)	0 (0 to 0)	5753 (5537 to 5974)	3696 (3574 to 3821)	642 (596 to 694)	1414 (1332 to 1508)	0 (0 to 0)
Netherlands	2018	5226 (5002 to 5461)	3357 (3224 to 3499)	583 (541 to 630)	1286 (1212 to 1373)	0 (0 to 0)	5846 (5595 to 6109)	3755 (3607 to 3914)	652 (605 to 705)	1439 (1356 to 1536)	0 (0 to 0)
Netherlands	2019	5387 (5149 to 5647)	3472 (3314 to 3635)	592 (549 to 639)	1323 (1247 to 1414)	0 (0 to 0)	6026 (5760 to 6317)	3884 (3708 to 4067)	662 (614 to 715)	1480 (1395 to 1581)	0 (0 to 0)
Netherlands	2020	5469 (5203 to 5755)	3525 (3335 to 3717)	600 (556 to 648)	1344 (1264 to 1436)	0 (0 to 0)	6118 (5820 to 6437)	3944 (3730 to 4157)	671 (622 to 725)	1504 (1414 to 1606)	0 (0 to 0)

Netherlands	2021	5528 (5234 to 5818)	3564 (3363 to 3761)	605 (561 to 654)	1359 (1278 to 1452)	0 (0 to 0)	6183 (5855 to 6508)	3986 (3761 to 4207)	677 (627 to 732)	1520 (1429 to 1624)	0 (0 to 0)
Netherlands	2022	5595 (5291 to 5908)	3609 (3402 to 3834)	610 (564 to 659)	1376 (1292 to 1471)	0 (0 to 0)	6259 (5919 to 6608)	4038 (3806 to 4288)	683 (631 to 738)	1539 (1445 to 1645)	0 (0 to 0)
Netherlands	2023	5669 (5356 to 6010)	3660 (3421 to 3898)	615 (569 to 665)	1393 (1306 to 1491)	0 (0 to 0)	6341 (5991 to 6722)	4094 (3827 to 4360)	688 (636 to 744)	1558 (1461 to 1668)	0 (0 to 0)
Netherlands	2024	5744 (5397 to 6120)	3714 (3461 to 3967)	620 (573 to 671)	1410 (1320 to 1513)	0 (0 to 0)	6426 (6038 to 6846)	4154 (3871 to 4437)	694 (641 to 750)	1578 (1476 to 1692)	0 (0 to 0)
Netherlands	2025	5805 (5454 to 6160)	3755 (3494 to 4010)	625 (577 to 675)	1425 (1331 to 1532)	0 (0 to 0)	6493 (6101 to 6890)	4201 (3908 to 4486)	699 (645 to 755)	1594 (1488 to 1714)	0 (0 to 0)
Netherlands	2026	5858 (5497 to 6233)	3792 (3524 to 4054)	628 (581 to 678)	1437 (1339 to 1549)	0 (0 to 0)	6553 (6149 to 6972)	4242 (3942 to 4535)	703 (650 to 758)	1608 (1497 to 1732)	0 (0 to 0)
Netherlands	2027	5901 (5524 to 6304)	3823 (3547 to 4101)	631 (583 to 680)	1448 (1344 to 1562)	0 (0 to 0)	6601 (6179 to 7052)	4276 (3968 to 4587)	705 (653 to 761)	1619 (1504 to 1747)	0 (0 to 0)
Netherlands	2028	5940 (5532 to 6358)	3851 (3561 to 4143)	633 (584 to 683)	1457 (1351 to 1576)	0 (0 to 0)	6645 (6188 to 7112)	4307 (3983 to 4634)	708 (654 to 764)	1630 (1511 to 1763)	0 (0 to 0)
Netherlands	2029	5980 (5571 to 6400)	3879 (3580 to 4176)	635 (585 to 686)	1466 (1357 to 1588)	0 (0 to 0)	6689 (6231 to 7159)	4339 (4004 to 4671)	710 (655 to 767)	1640 (1518 to 1776)	0 (0 to 0)
Netherlands	2030	6023 (5611 to 6462)	3910 (3596 to 4224)	637 (587 to 689)	1477 (1364 to 1602)	0 (0 to 0)	6738 (6277 to 7228)	4373 (4022 to 4725)	713 (657 to 771)	1652 (1526 to 1792)	0 (0 to 0)

Netherlands	2031	6075 (5634 to 6538)	3946 (3621 to 4279)	640 (589 to 694)	1489 (1373 to 1622)	0 (0 to 0)	6796 (6302 to 7314)	4414 (4051 to 4786)	716 (659 to 776)	1666 (1536 to 1814)	0 (0 to 0)
Netherlands	2032	6132 (5691 to 6615)	3985 (3651 to 4332)	644 (592 to 698)	1503 (1383 to 1642)	0 (0 to 0)	6859 (6366 to 7399)	4457 (4084 to 4845)	720 (662 to 781)	1681 (1547 to 1837)	0 (0 to 0)
Netherlands	2033	6187 (5708 to 6675)	4023 (3669 to 4383)	647 (594 to 703)	1516 (1391 to 1661)	0 (0 to 0)	6920 (6385 to 7466)	4500 (4104 to 4903)	724 (665 to 786)	1696 (1556 to 1858)	0 (0 to 0)
Netherlands	2034	6240 (5763 to 6770)	4060 (3687 to 4433)	651 (597 to 707)	1529 (1400 to 1679)	0 (0 to 0)	6980 (6447 to 7573)	4542 (4125 to 4959)	728 (667 to 791)	1710 (1566 to 1878)	0 (0 to 0)
Netherlands	2035	6295 (5798 to 6840)	4099 (3716 to 4495)	654 (599 to 712)	1542 (1410 to 1698)	0 (0 to 0)	7042 (6486 to 7652)	4585 (4157 to 5028)	732 (670 to 797)	1725 (1577 to 1899)	0 (0 to 0)
Netherlands	2036	6360 (5830 to 6946)	4144 (3753 to 4555)	658 (602 to 717)	1558 (1419 to 1718)	0 (0 to 0)	7115 (6521 to 7770)	4635 (4198 to 5095)	736 (673 to 802)	1743 (1587 to 1922)	0 (0 to 0)
Netherlands	2037	6434 (5885 to 7031)	4195 (3788 to 4631)	663 (607 to 723)	1576 (1431 to 1743)	0 (0 to 0)	7197 (6583 to 7865)	4692 (4237 to 5180)	742 (678 to 808)	1763 (1601 to 1950)	0 (0 to 0)
Netherlands	2038	6519 (5959 to 7168)	4253 (3829 to 4703)	669 (612 to 729)	1597 (1446 to 1771)	0 (0 to 0)	7292 (6666 to 8018)	4758 (4283 to 5261)	748 (685 to 815)	1787 (1617 to 1981)	0 (0 to 0)
Netherlands	2039	6616 (6015 to 7247)	4320 (3876 to 4783)	675 (618 to 737)	1621 (1463 to 1799)	0 (0 to 0)	7401 (6728 to 8106)	4832 (4336 to 5351)	756 (692 to 825)	1814 (1636 to 2013)	0 (0 to 0)
Netherlands	2040	6723 (6109 to 7396)	4392 (3935 to 4872)	683 (625 to 747)	1648 (1484 to 1834)	0 (0 to 0)	7520 (6833 to 8273)	4913 (4402 to 5450)	764 (699 to 835)	1843 (1660 to 2051)	0 (0 to 0)

Netherlands	2041	6837 (6192 to 7545)	4470 (4002 to 4971)	691 (633 to 755)	1676 (1506 to 1871)	0 (0 to 0)	7648 (6927 to 8440)	5001 (4477 to 5560)	773 (708 to 845)	1875 (1685 to 2093)	0 (0 to 0)
Netherlands	2042	6955 (6299 to 7679)	4550 (4069 to 5071)	699 (641 to 765)	1705 (1529 to 1908)	0 (0 to 0)	7779 (7046 to 8590)	5090 (4551 to 5673)	782 (717 to 855)	1907 (1710 to 2134)	0 (0 to 0)
Netherlands	2043	7076 (6386 to 7823)	4633 (4135 to 5165)	708 (648 to 775)	1735 (1551 to 1947)	0 (0 to 0)	7915 (7143 to 8751)	5182 (4626 to 5778)	792 (725 to 867)	1941 (1735 to 2178)	0 (0 to 0)
Netherlands	2044	7204 (6504 to 7982)	4720 (4208 to 5264)	717 (656 to 784)	1767 (1575 to 1989)	0 (0 to 0)	8059 (7275 to 8929)	5280 (4707 to 5889)	802 (734 to 877)	1977 (1762 to 2225)	0 (0 to 0)
Netherlands	2045	7336 (6626 to 8119)	4810 (4283 to 5365)	726 (663 to 795)	1800 (1600 to 2032)	0 (0 to 0)	8206 (7412 to 9082)	5380 (4791 to 6002)	812 (742 to 889)	2014 (1790 to 2273)	0 (0 to 0)
Netherlands	2046	7472 (6728 to 8297)	4902 (4364 to 5469)	736 (670 to 807)	1834 (1625 to 2075)	0 (0 to 0)	8358 (7526 to 9281)	5484 (4881 to 6117)	823 (750 to 903)	2051 (1818 to 2321)	0 (0 to 0)
Netherlands	2047	7608 (6842 to 8443)	4995 (4446 to 5572)	745 (677 to 819)	1868 (1651 to 2119)	0 (0 to 0)	8511 (7654 to 9444)	5587 (4973 to 6233)	834 (758 to 916)	2090 (1846 to 2370)	0 (0 to 0)
Netherlands	2048	7746 (6960 to 8592)	5089 (4528 to 5676)	755 (685 to 830)	1902 (1676 to 2164)	0 (0 to 0)	8665 (7785 to 9611)	5692 (5065 to 6350)	845 (766 to 929)	2128 (1875 to 2421)	0 (0 to 0)
Netherlands	2049	7887 (7061 to 8754)	5184 (4611 to 5782)	765 (692 to 842)	1938 (1702 to 2213)	0 (0 to 0)	8822 (7898 to 9792)	5799 (5157 to 6468)	856 (774 to 942)	2167 (1904 to 2476)	0 (0 to 0)
Netherlands	2050	8028 (7188 to 8955)	5281 (4694 to 5888)	775 (698 to 854)	1973 (1728 to 2261)	0 (0 to 0)	8980 (8040 to 10017)	5907 (5251 to 6587)	866 (781 to 955)	2207 (1933 to 2529)	0 (0 to 0)

New Zealand	1995	2244 (2156 to 2336)	1682 (1607 to 1762)	340 (306 to 380)	222 (190 to 259)	0 (0 to 0)	2243 (2155 to 2335)	1681 (1606 to 1761)	340 (306 to 380)	222 (190 to 258)	0 (0 to 0)
New Zealand	1996	2275 (2205 to 2345)	1703 (1648 to 1761)	345 (315 to 378)	227 (199 to 256)	0 (0 to 0)	2274 (2204 to 2345)	1703 (1648 to 1760)	345 (315 to 378)	227 (199 to 256)	0 (0 to 0)
New Zealand	1997	2338 (2270 to 2405)	1750 (1696 to 1807)	354 (328 to 383)	234 (208 to 260)	0 (0 to 0)	2337 (2269 to 2404)	1750 (1695 to 1807)	354 (328 to 382)	234 (208 to 260)	0 (0 to 0)
New Zealand	1998	2400 (2334 to 2471)	1793 (1737 to 1852)	368 (341 to 395)	239 (216 to 267)	0 (0 to 0)	2399 (2333 to 2470)	1793 (1736 to 1851)	368 (341 to 395)	239 (216 to 267)	0 (0 to 0)
New Zealand	1999	2455 (2386 to 2521)	1831 (1777 to 1886)	381 (355 to 407)	244 (222 to 270)	0 (0 to 0)	2454 (2385 to 2520)	1830 (1777 to 1885)	380 (355 to 407)	244 (221 to 270)	0 (0 to 0)
New Zealand	2000	2475 (2413 to 2537)	1837 (1786 to 1890)	392 (367 to 417)	246 (225 to 268)	0 (0 to 0)	2474 (2412 to 2536)	1837 (1786 to 1889)	392 (367 to 417)	245 (225 to 268)	0 (0 to 0)
New Zealand	2001	2517 (2460 to 2581)	1865 (1813 to 1917)	409 (383 to 433)	243 (222 to 266)	0 (0 to 0)	2516 (2459 to 2580)	1864 (1812 to 1916)	409 (383 to 433)	243 (222 to 266)	0 (0 to 0)
New Zealand	2002	2637 (2574 to 2701)	1971 (1919 to 2026)	421 (395 to 445)	245 (224 to 268)	0 (0 to 0)	2636 (2573 to 2700)	1970 (1918 to 2026)	420 (395 to 445)	245 (224 to 268)	0 (0 to 0)
New Zealand	2003	2748 (2686 to 2811)	2077 (2021 to 2133)	421 (398 to 447)	249 (228 to 270)	0 (0 to 0)	2747 (2685 to 2810)	2077 (2020 to 2132)	421 (397 to 447)	249 (228 to 270)	0 (0 to 0)
New Zealand	2004	2861 (2799 to 2926)	2189 (2130 to 2246)	418 (396 to 444)	255 (233 to 276)	0 (0 to 0)	2860 (2798 to 2925)	2188 (2130 to 2245)	418 (395 to 444)	255 (233 to 276)	0 (0 to 0)

New Zealand	2005	3019 (2953 to 3083)	2328 (2273 to 2385)	421 (399 to 447)	269 (247 to 293)	0 (0 to 0)	3018 (2952 to 3082)	2327 (2272 to 2384)	421 (399 to 447)	269 (247 to 293)	0 (0 to 0)
New Zealand	2006	3164 (3099 to 3229)	2457 (2399 to 2517)	422 (398 to 446)	285 (264 to 309)	0 (0 to 0)	3163 (3098 to 3228)	2456 (2398 to 2516)	422 (398 to 446)	285 (264 to 308)	0 (0 to 0)
New Zealand	2007	3256 (3195 to 3322)	2543 (2484 to 2603)	413 (389 to 436)	300 (278 to 322)	0 (0 to 0)	3255 (3194 to 3321)	2543 (2483 to 2602)	412 (389 to 436)	300 (278 to 322)	0 (0 to 0)
New Zealand	2008	3373 (3305 to 3437)	2631 (2573 to 2688)	425 (402 to 452)	317 (294 to 340)	0 (0 to 0)	3372 (3304 to 3436)	2630 (2572 to 2687)	425 (402 to 452)	317 (294 to 340)	0 (0 to 0)
New Zealand	2009	3486 (3421 to 3551)	2720 (2662 to 2777)	431 (408 to 456)	335 (311 to 359)	0 (0 to 0)	3484 (3419 to 3550)	2719 (2661 to 2776)	431 (407 to 456)	334 (311 to 359)	0 (0 to 0)
New Zealand	2010	3637 (3573 to 3712)	2850 (2792 to 2910)	439 (415 to 465)	348 (323 to 373)	0 (0 to 0)	3636 (3571 to 3710)	2849 (2791 to 2909)	439 (415 to 465)	348 (323 to 373)	0 (0 to 0)
New Zealand	2011	3678 (3608 to 3747)	2889 (2829 to 2950)	445 (421 to 472)	345 (321 to 369)	0 (0 to 0)	3677 (3607 to 3746)	2888 (2828 to 2949)	445 (421 to 471)	344 (321 to 369)	0 (0 to 0)
New Zealand	2012	3667 (3599 to 3737)	2870 (2813 to 2927)	452 (429 to 478)	345 (320 to 371)	0 (0 to 0)	3666 (3597 to 3736)	2869 (2812 to 2926)	452 (428 to 478)	345 (320 to 370)	0 (0 to 0)
New Zealand	2013	3732 (3663 to 3801)	2898 (2835 to 2951)	468 (443 to 493)	366 (341 to 392)	0 (0 to 0)	3730 (3661 to 3799)	2897 (2834 to 2950)	468 (442 to 493)	366 (341 to 392)	0 (0 to 0)
New Zealand	2014	3813 (3739 to 3886)	2925 (2864 to 2987)	491 (465 to 518)	397 (371 to 426)	0 (0 to 0)	3811 (3738 to 3885)	2924 (2863 to 2986)	491 (464 to 518)	397 (370 to 426)	0 (0 to 0)

New Zealand	2015	3909 (3839 to 3981)	2948 (2888 to 3011)	518 (492 to 545)	443 (416 to 472)	0 (0 to 0)	3908 (3838 to 3980)	2947 (2887 to 3010)	517 (491 to 545)	443 (416 to 472)	0 (0 to 0)
New Zealand	2016	3998 (3921 to 4074)	2992 (2930 to 3053)	540 (512 to 569)	465 (438 to 494)	0 (0 to 0)	3997 (3920 to 4072)	2991 (2929 to 3052)	540 (512 to 569)	465 (438 to 494)	0 (0 to 0)
New Zealand	2017	4068 (3970 to 4174)	3050 (2966 to 3135)	555 (519 to 596)	462 (426 to 503)	0 (0 to 0)	4066 (3969 to 4172)	3049 (2965 to 3134)	555 (519 to 596)	462 (425 to 503)	0 (0 to 0)
New Zealand	2018	4167 (4051 to 4284)	3132 (3032 to 3238)	562 (525 to 604)	473 (435 to 515)	0 (0 to 0)	4166 (4050 to 4283)	3131 (3031 to 3237)	562 (525 to 604)	473 (435 to 515)	0 (0 to 0)
New Zealand	2019	4242 (4102 to 4374)	3197 (3082 to 3315)	565 (527 to 606)	481 (443 to 523)	0 (0 to 0)	4241 (4101 to 4373)	3195 (3081 to 3314)	565 (527 to 606)	480 (442 to 523)	0 (0 to 0)
New Zealand	2020	4291 (4151 to 4436)	3236 (3109 to 3365)	569 (532 to 611)	486 (448 to 529)	0 (0 to 0)	4290 (4149 to 4434)	3235 (3108 to 3363)	569 (531 to 611)	486 (448 to 529)	0 (0 to 0)
New Zealand	2021	4340 (4198 to 4476)	3276 (3141 to 3407)	572 (534 to 614)	492 (453 to 535)	0 (0 to 0)	4338 (4197 to 4475)	3275 (3140 to 3406)	572 (534 to 614)	491 (453 to 535)	0 (0 to 0)
New Zealand	2022	4397 (4245 to 4542)	3324 (3181 to 3462)	575 (536 to 618)	498 (459 to 541)	0 (0 to 0)	4395 (4243 to 4540)	3323 (3180 to 3461)	575 (536 to 618)	498 (459 to 541)	0 (0 to 0)
New Zealand	2023	4442 (4288 to 4592)	3362 (3205 to 3507)	577 (538 to 620)	502 (463 to 547)	0 (0 to 0)	4440 (4286 to 4590)	3361 (3203 to 3506)	577 (538 to 620)	502 (463 to 547)	0 (0 to 0)
New Zealand	2024	4478 (4313 to 4633)	3395 (3232 to 3545)	577 (537 to 619)	506 (467 to 552)	0 (0 to 0)	4476 (4311 to 4631)	3394 (3230 to 3544)	577 (537 to 619)	506 (466 to 552)	0 (0 to 0)

New Zealand	2025	4512 (4342 to 4663)	3426 (3258 to 3583)	577 (537 to 620)	509 (469 to 557)	0 (0 to 0)	4510 (4340 to 4661)	3425 (3257 to 3582)	576 (537 to 619)	509 (469 to 557)	0 (0 to 0)
New Zealand	2026	4553 (4385 to 4706)	3462 (3294 to 3620)	577 (538 to 620)	514 (471 to 561)	0 (0 to 0)	4551 (4383 to 4704)	3461 (3293 to 3619)	577 (538 to 620)	513 (471 to 561)	0 (0 to 0)
New Zealand	2027	4598 (4414 to 4767)	3501 (3322 to 3669)	579 (539 to 622)	518 (475 to 568)	0 (0 to 0)	4597 (4412 to 4765)	3500 (3321 to 3668)	579 (539 to 622)	518 (475 to 567)	0 (0 to 0)
New Zealand	2028	4648 (4460 to 4822)	3543 (3360 to 3711)	581 (540 to 625)	524 (480 to 574)	0 (0 to 0)	4646 (4458 to 4820)	3542 (3359 to 3710)	581 (540 to 625)	524 (480 to 573)	0 (0 to 0)
New Zealand	2029	4699 (4513 to 4881)	3586 (3403 to 3760)	583 (541 to 628)	529 (484 to 582)	0 (0 to 0)	4697 (4512 to 4880)	3585 (3401 to 3759)	583 (541 to 628)	529 (484 to 582)	0 (0 to 0)
New Zealand	2030	4755 (4562 to 4936)	3634 (3440 to 3815)	586 (543 to 631)	535 (489 to 589)	0 (0 to 0)	4754 (4560 to 4934)	3632 (3439 to 3814)	586 (543 to 631)	535 (489 to 588)	0 (0 to 0)
New Zealand	2031	4809 (4606 to 4999)	3678 (3481 to 3870)	589 (545 to 635)	541 (494 to 596)	0 (0 to 0)	4807 (4604 to 4997)	3677 (3480 to 3869)	589 (545 to 634)	541 (494 to 595)	0 (0 to 0)
New Zealand	2032	4859 (4649 to 5070)	3720 (3514 to 3922)	592 (547 to 639)	547 (499 to 603)	0 (0 to 0)	4857 (4647 to 5068)	3719 (3512 to 3921)	592 (547 to 638)	547 (498 to 602)	0 (0 to 0)
New Zealand	2033	4907 (4672 to 5136)	3760 (3542 to 3969)	594 (548 to 642)	553 (502 to 610)	0 (0 to 0)	4905 (4671 to 5134)	3759 (3540 to 3968)	594 (548 to 642)	552 (502 to 610)	0 (0 to 0)
New Zealand	2034	4955 (4712 to 5183)	3800 (3574 to 4016)	597 (550 to 647)	558 (506 to 617)	0 (0 to 0)	4953 (4710 to 5181)	3799 (3572 to 4015)	596 (550 to 647)	558 (506 to 617)	0 (0 to 0)

New Zealand	2035	5002 (4770 to 5217)	3839 (3607 to 4061)	599 (551 to 649)	563 (509 to 625)	0 (0 to 0)	5000 (4769 to 5215)	3838 (3606 to 4060)	599 (551 to 649)	563 (509 to 625)	0 (0 to 0)
New Zealand	2036	5051 (4815 to 5286)	3880 (3640 to 4111)	602 (552 to 653)	569 (513 to 634)	0 (0 to 0)	5049 (4813 to 5284)	3879 (3638 to 4110)	601 (552 to 652)	569 (513 to 634)	0 (0 to 0)
New Zealand	2037	5101 (4840 to 5344)	3921 (3687 to 4168)	605 (554 to 657)	575 (517 to 643)	0 (0 to 0)	5100 (4839 to 5342)	3920 (3686 to 4166)	605 (554 to 657)	575 (517 to 643)	0 (0 to 0)
New Zealand	2038	5154 (4898 to 5413)	3964 (3717 to 4206)	609 (557 to 663)	582 (521 to 652)	0 (0 to 0)	5152 (4896 to 5411)	3962 (3716 to 4205)	608 (557 to 663)	581 (521 to 652)	0 (0 to 0)
New Zealand	2039	5204 (4936 to 5468)	4003 (3742 to 4260)	613 (560 to 667)	588 (525 to 660)	0 (0 to 0)	5202 (4934 to 5466)	4002 (3741 to 4259)	612 (560 to 667)	588 (525 to 660)	0 (0 to 0)
New Zealand	2040	5254 (4991 to 5509)	4043 (3771 to 4302)	617 (564 to 671)	594 (530 to 669)	0 (0 to 0)	5252 (4989 to 5507)	4041 (3769 to 4300)	617 (563 to 671)	594 (529 to 669)	0 (0 to 0)
New Zealand	2041	5307 (5037 to 5580)	4084 (3809 to 4350)	621 (567 to 677)	601 (535 to 679)	0 (0 to 0)	5305 (5035 to 5578)	4083 (3808 to 4349)	621 (567 to 677)	601 (534 to 679)	0 (0 to 0)
New Zealand	2042	5359 (5077 to 5640)	4125 (3838 to 4411)	626 (571 to 684)	608 (539 to 690)	0 (0 to 0)	5357 (5075 to 5638)	4123 (3836 to 4410)	625 (571 to 683)	608 (539 to 690)	0 (0 to 0)
New Zealand	2043	5412 (5127 to 5696)	4166 (3878 to 4462)	631 (575 to 689)	615 (544 to 701)	0 (0 to 0)	5410 (5125 to 5693)	4164 (3876 to 4460)	631 (574 to 689)	615 (543 to 701)	0 (0 to 0)
New Zealand	2044	5468 (5142 to 5769)	4209 (3902 to 4516)	636 (579 to 697)	623 (549 to 711)	0 (0 to 0)	5466 (5140 to 5767)	4208 (3901 to 4515)	636 (578 to 696)	623 (549 to 711)	0 (0 to 0)

New Zealand	2045	5524 (5204 to 5846)	4251 (3936 to 4577)	642 (583 to 704)	630 (554 to 721)	0 (0 to 0)	5522 (5203 to 5844)	4250 (3935 to 4575)	641 (582 to 704)	630 (554 to 721)	0 (0 to 0)
New Zealand	2046	5579 (5250 to 5905)	4294 (3969 to 4637)	647 (587 to 712)	638 (559 to 733)	0 (0 to 0)	5577 (5248 to 5903)	4292 (3967 to 4635)	647 (587 to 712)	638 (559 to 732)	0 (0 to 0)
New Zealand	2047	5634 (5282 to 5990)	4336 (3999 to 4675)	653 (591 to 719)	646 (564 to 744)	0 (0 to 0)	5632 (5280 to 5988)	4334 (3998 to 4673)	653 (591 to 719)	646 (564 to 744)	0 (0 to 0)
New Zealand	2048	5691 (5297 to 6076)	4379 (4018 to 4733)	659 (596 to 727)	654 (569 to 756)	0 (0 to 0)	5689 (5296 to 6074)	4377 (4016 to 4731)	659 (595 to 727)	654 (569 to 756)	0 (0 to 0)
New Zealand	2049	5750 (5370 to 6123)	4423 (4067 to 4781)	665 (600 to 737)	662 (574 to 767)	0 (0 to 0)	5748 (5368 to 6120)	4421 (4066 to 4779)	665 (600 to 736)	662 (574 to 767)	0 (0 to 0)
New Zealand	2050	5811 (5416 to 6178)	4470 (4096 to 4830)	671 (605 to 744)	671 (581 to 779)	0 (0 to 0)	5809 (5414 to 6175)	4468 (4094 to 4828)	671 (604 to 744)	670 (580 to 779)	0 (0 to 0)
Nicaragua	1995	89 (76 to 105)	39 (31 to 50)	38 (28 to 51)	3 (1 to 5)	9 (9 to 9)	245 (207 to 288)	108 (85 to 136)	105 (76 to 139)	7 (4 to 13)	24 (24 to 24)
Nicaragua	1996	93 (80 to 107)	37 (29 to 46)	39 (29 to 51)	3 (1 to 5)	14 (14 to 14)	254 (219 to 294)	101 (80 to 126)	106 (78 to 138)	7 (4 to 13)	40 (40 to 40)
Nicaragua	1997	85 (73 to 99)	33 (26 to 41)	38 (28 to 50)	3 (1 to 5)	12 (12 to 12)	234 (201 to 272)	90 (71 to 112)	105 (78 to 138)	7 (3 to 13)	32 (32 to 32)
Nicaragua	1998	84 (73 to 98)	32 (25 to 40)	38 (29 to 50)	3 (1 to 5)	11 (11 to 11)	231 (199 to 268)	87 (70 to 109)	105 (79 to 137)	7 (4 to 14)	31 (31 to 31)
Nicaragua	1999	98 (86 to 112)	33 (26 to 40)	38 (29 to 50)	3 (1 to 5)	25 (25 to 25)	270 (237 to 306)	90 (71 to 110)	105 (80 to 136)	8 (4 to 14)	67 (67 to 67)
Nicaragua	2000	89 (77 to 102)	36 (28 to 44)	38 (28 to 49)	3 (1 to 6)	12 (12 to 12)	243 (210 to 280)	98 (78 to 120)	103 (77 to 133)	8 (4 to 15)	34 (34 to 34)

Nicaragua	2001	86 (75 to 99)	36 (29 to 44)	37 (28 to 47)	3 (2 to 6)	11 (11 to 11)	236 (204 to 271)	98 (79 to 119)	100 (76 to 130)	9 (4 to 16)	29 (29 to 29)
Nicaragua	2002	87 (75 to 100)	35 (29 to 43)	37 (28 to 48)	3 (1 to 6)	11 (11 to 11)	238 (207 to 273)	97 (78 to 118)	101 (77 to 131)	8 (4 to 16)	31 (31 to 31)
Nicaragua	2003	91 (79 to 104)	36 (29 to 44)	38 (29 to 48)	3 (2 to 6)	13 (13 to 13)	249 (218 to 286)	100 (81 to 121)	103 (79 to 133)	9 (4 to 16)	37 (37 to 37)
Nicaragua	2004	96 (84 to 110)	39 (32 to 47)	39 (30 to 51)	3 (1 to 5)	15 (15 to 15)	263 (229 to 303)	107 (87 to 130)	108 (83 to 139)	8 (4 to 14)	40 (40 to 40)
Nicaragua	2005	102 (90 to 118)	43 (35 to 51)	41 (32 to 53)	3 (1 to 5)	16 (16 to 16)	281 (246 to 322)	117 (95 to 141)	113 (87 to 145)	7 (4 to 13)	44 (44 to 44)
Nicaragua	2006	106 (93 to 121)	44 (36 to 53)	43 (33 to 55)	2 (1 to 4)	16 (16 to 16)	289 (254 to 332)	120 (98 to 145)	118 (91 to 150)	7 (3 to 12)	44 (44 to 44)
Nicaragua	2007	114 (100 to 131)	48 (40 to 59)	46 (36 to 59)	2 (1 to 5)	17 (17 to 17)	313 (274 to 360)	132 (109 to 161)	127 (98 to 162)	7 (3 to 12)	47 (47 to 47)
Nicaragua	2008	121 (106 to 139)	52 (43 to 64)	48 (37 to 61)	3 (1 to 5)	18 (18 to 18)	332 (290 to 382)	143 (118 to 175)	132 (103 to 167)	7 (3 to 13)	50 (50 to 50)
Nicaragua	2009	123 (108 to 140)	55 (45 to 66)	48 (38 to 61)	3 (1 to 5)	17 (17 to 17)	336 (295 to 384)	149 (123 to 181)	132 (104 to 168)	7 (4 to 14)	47 (47 to 47)
Nicaragua	2010	120 (104 to 137)	56 (46 to 68)	48 (38 to 62)	3 (1 to 5)	13 (13 to 13)	329 (286 to 376)	154 (126 to 187)	133 (103 to 169)	8 (4 to 14)	35 (35 to 35)
Nicaragua	2011	128 (111 to 147)	63 (52 to 75)	50 (39 to 63)	3 (1 to 5)	12 (12 to 12)	350 (303 to 402)	173 (143 to 207)	137 (106 to 173)	8 (4 to 15)	32 (32 to 32)
Nicaragua	2012	137 (119 to 156)	70 (58 to 83)	52 (40 to 66)	3 (1 to 6)	12 (12 to 12)	374 (325 to 427)	191 (159 to 228)	142 (110 to 181)	9 (4 to 16)	32 (32 to 32)
Nicaragua	2013	146 (127 to 167)	76 (63 to 91)	54 (41 to 68)	3 (2 to 6)	13 (13 to 13)	400 (348 to 457)	209 (172 to 248)	147 (113 to 188)	9 (4 to 17)	35 (35 to 35)
Nicaragua	2014	154 (135 to 176)	84 (70 to 100)	56 (43 to 71)	4 (2 to 7)	11 (11 to 11)	423 (369 to 482)	231 (192 to 274)	152 (117 to 194)	10 (5 to 18)	29 (29 to 29)
Nicaragua	2015	166 (145 to 189)	93 (77 to 111)	57 (43 to 75)	4 (2 to 7)	12 (12 to 12)	454 (396 to 518)	254 (211 to 304)	157 (119 to 204)	10 (5 to 18)	33 (33 to 33)

Nicaragua	2016	184 (161 to 210)	104 (86 to 125)	60 (45 to 79)	4 (2 to 7)	16 (16 to 16)	504 (442 to 576)	284 (235 to 343)	165 (123 to 217)	11 (5 to 19)	44 (44 to 44)
Nicaragua	2017	188 (161 to 222)	110 (90 to 133)	62 (45 to 83)	4 (2 to 7)	13 (13 to 13)	516 (441 to 608)	300 (247 to 366)	170 (125 to 227)	11 (5 to 20)	35 (35 to 35)
Nicaragua	2018	183 (157 to 215)	107 (88 to 131)	59 (43 to 79)	4 (2 to 7)	13 (12 to 13)	501 (429 to 590)	294 (242 to 359)	162 (119 to 217)	10 (5 to 19)	34 (34 to 35)
Nicaragua	2019	176 (151 to 207)	103 (85 to 126)	56 (41 to 75)	4 (2 to 7)	13 (13 to 13)	482 (414 to 568)	283 (233 to 344)	153 (113 to 205)	10 (5 to 18)	36 (35 to 36)
Nicaragua	2020	175 (150 to 207)	103 (85 to 125)	55 (40 to 74)	4 (2 to 7)	13 (13 to 14)	481 (412 to 567)	283 (232 to 344)	151 (111 to 202)	10 (5 to 18)	37 (35 to 39)
Nicaragua	2021	178 (152 to 209)	105 (86 to 128)	55 (41 to 74)	4 (2 to 7)	14 (13 to 15)	487 (416 to 573)	288 (236 to 350)	152 (111 to 204)	10 (5 to 19)	37 (35 to 41)
Nicaragua	2022	181 (155 to 213)	107 (88 to 131)	56 (41 to 75)	4 (2 to 7)	14 (13 to 15)	495 (425 to 584)	294 (240 to 359)	153 (112 to 205)	10 (5 to 19)	38 (35 to 42)
Nicaragua	2023	184 (157 to 217)	110 (90 to 134)	56 (41 to 75)	4 (2 to 7)	14 (13 to 16)	503 (431 to 594)	301 (246 to 367)	154 (113 to 206)	10 (5 to 19)	39 (35 to 43)
Nicaragua	2024	188 (161 to 221)	113 (93 to 137)	57 (42 to 76)	4 (2 to 7)	14 (13 to 16)	514 (441 to 605)	309 (254 to 375)	155 (114 to 209)	11 (5 to 19)	39 (35 to 44)
Nicaragua	2025	191 (164 to 225)	116 (94 to 140)	57 (42 to 77)	4 (2 to 7)	14 (13 to 16)	525 (449 to 618)	318 (258 to 384)	157 (116 to 211)	11 (5 to 20)	40 (35 to 45)
Nicaragua	2026	195 (167 to 230)	119 (97 to 143)	58 (42 to 78)	4 (2 to 7)	15 (13 to 17)	534 (458 to 630)	325 (267 to 393)	158 (116 to 212)	11 (5 to 20)	40 (35 to 46)
Nicaragua	2027	199 (170 to 234)	122 (99 to 147)	58 (43 to 78)	4 (2 to 8)	15 (13 to 17)	544 (467 to 640)	333 (272 to 403)	159 (117 to 214)	11 (5 to 21)	41 (36 to 47)
Nicaragua	2028	202 (174 to 238)	125 (102 to 150)	58 (43 to 79)	4 (2 to 8)	15 (13 to 18)	555 (477 to 653)	342 (279 to 411)	160 (118 to 216)	12 (5 to 21)	41 (36 to 48)
Nicaragua	2029	206 (177 to 243)	128 (104 to 155)	59 (44 to 79)	4 (2 to 8)	15 (13 to 18)	565 (484 to 665)	350 (285 to 424)	161 (119 to 218)	12 (5 to 21)	42 (36 to 49)
Nicaragua	2030	210 (180 to 247)	131 (106 to 158)	59 (44 to 80)	4 (2 to 8)	16 (13 to 19)	576 (492 to 677)	359 (292 to 433)	163 (120 to 219)	12 (6 to 22)	43 (37 to 51)

Nicaragua	2031	214 (184 to 252)	134 (109 to 162)	60 (44 to 81)	4 (2 to 8)	16 (14 to 19)	587 (503 to 690)	367 (300 to 443)	164 (121 to 221)	12 (6 to 22)	43 (37 to 52)
Nicaragua	2032	218 (187 to 256)	137 (112 to 165)	60 (45 to 81)	5 (2 to 8)	16 (14 to 20)	597 (511 to 701)	376 (306 to 452)	165 (122 to 223)	12 (6 to 23)	44 (38 to 54)
Nicaragua	2033	222 (190 to 260)	140 (114 to 169)	61 (45 to 82)	5 (2 to 8)	16 (14 to 20)	608 (520 to 714)	384 (312 to 463)	166 (123 to 224)	13 (6 to 23)	45 (38 to 55)
Nicaragua	2034	226 (193 to 265)	143 (117 to 173)	61 (45 to 82)	5 (2 to 9)	17 (14 to 20)	618 (530 to 726)	393 (320 to 474)	167 (124 to 225)	13 (6 to 23)	46 (38 to 56)
Nicaragua	2035	230 (197 to 268)	146 (119 to 177)	61 (45 to 83)	5 (2 to 9)	17 (14 to 21)	629 (539 to 734)	401 (327 to 485)	168 (124 to 226)	13 (6 to 24)	47 (39 to 57)
Nicaragua	2036	234 (200 to 274)	150 (122 to 180)	62 (45 to 83)	5 (2 to 9)	17 (14 to 22)	640 (548 to 751)	410 (334 to 493)	169 (125 to 228)	13 (6 to 24)	48 (39 to 60)
Nicaragua	2037	238 (204 to 280)	153 (124 to 183)	62 (46 to 84)	5 (2 to 9)	18 (15 to 22)	651 (558 to 767)	419 (340 to 503)	170 (125 to 229)	13 (6 to 25)	49 (40 to 59)
Nicaragua	2038	242 (207 to 283)	156 (126 to 188)	62 (46 to 84)	5 (2 to 9)	18 (15 to 23)	662 (567 to 775)	428 (346 to 514)	171 (125 to 230)	14 (6 to 25)	50 (41 to 62)
Nicaragua	2039	245 (210 to 289)	159 (129 to 192)	63 (46 to 84)	5 (2 to 9)	18 (15 to 23)	672 (574 to 792)	436 (353 to 525)	172 (126 to 231)	14 (6 to 25)	51 (42 to 64)
Nicaragua	2040	249 (212 to 292)	162 (131 to 196)	63 (46 to 85)	5 (2 to 9)	19 (15 to 24)	683 (581 to 799)	445 (360 to 536)	172 (127 to 232)	14 (6 to 26)	52 (42 to 65)
Nicaragua	2041	253 (216 to 297)	165 (133 to 200)	63 (46 to 85)	5 (2 to 10)	19 (16 to 24)	693 (591 to 814)	453 (365 to 548)	173 (127 to 233)	14 (7 to 26)	53 (43 to 67)
Nicaragua	2042	257 (218 to 301)	168 (136 to 203)	63 (47 to 85)	5 (2 to 10)	20 (16 to 25)	704 (597 to 824)	462 (373 to 557)	174 (128 to 233)	14 (7 to 26)	54 (44 to 68)
Nicaragua	2043	261 (222 to 304)	172 (139 to 207)	64 (47 to 85)	5 (2 to 10)	20 (16 to 26)	714 (609 to 833)	470 (380 to 567)	174 (128 to 234)	15 (7 to 27)	55 (45 to 72)
Nicaragua	2044	265 (226 to 309)	175 (141 to 211)	64 (47 to 86)	5 (2 to 10)	21 (17 to 27)	725 (619 to 847)	479 (387 to 578)	175 (128 to 235)	15 (7 to 27)	56 (46 to 75)
Nicaragua	2045	268 (229 to 314)	178 (143 to 215)	64 (47 to 86)	5 (3 to 10)	21 (17 to 27)	736 (628 to 862)	488 (393 to 590)	175 (129 to 235)	15 (7 to 28)	58 (46 to 74)

Nicaragua	2046	272 (232 to 319)	181 (147 to 220)	64 (47 to 86)	6 (3 to 10)	22 (17 to 28)	746 (635 to 873)	496 (402 to 602)	175 (129 to 235)	15 (7 to 28)	59 (47 to 75)
Nicaragua	2047	276 (235 to 323)	184 (149 to 222)	64 (47 to 86)	6 (3 to 10)	22 (18 to 29)	756 (645 to 885)	505 (409 to 609)	176 (128 to 235)	15 (7 to 28)	61 (48 to 80)
Nicaragua	2048	280 (240 to 327)	187 (152 to 225)	64 (47 to 86)	6 (3 to 11)	23 (18 to 30)	766 (657 to 897)	513 (416 to 617)	176 (129 to 235)	16 (7 to 29)	62 (50 to 81)
Nicaragua	2049	283 (242 to 333)	190 (154 to 229)	64 (47 to 86)	6 (3 to 11)	23 (18 to 31)	776 (664 to 913)	521 (423 to 629)	176 (128 to 236)	16 (7 to 29)	64 (51 to 84)
Nicaragua	2050	287 (245 to 337)	193 (157 to 232)	64 (47 to 86)	6 (3 to 11)	24 (19 to 32)	786 (672 to 924)	529 (430 to 637)	176 (128 to 236)	16 (7 to 29)	65 (52 to 88)
Niger	1995	23 (18 to 30)	5 (4 to 7)	14 (10 to 21)	2 (1 to 3)	3 (3 to 3)	62 (49 to 78)	13 (10 to 17)	37 (25 to 54)	4 (2 to 8)	7 (7 to 7)
Niger	1996	22 (18 to 28)	5 (4 to 6)	14 (9 to 20)	1 (1 to 3)	3 (3 to 3)	59 (46 to 74)	12 (9 to 16)	36 (23 to 52)	4 (2 to 7)	7 (7 to 7)
Niger	1997	22 (18 to 28)	5 (4 to 7)	14 (9 to 20)	2 (1 to 3)	2 (2 to 2)	59 (46 to 74)	13 (10 to 18)	36 (24 to 52)	4 (2 to 7)	5 (5 to 5)
Niger	1998	23 (18 to 29)	5 (4 to 7)	14 (10 to 20)	2 (1 to 3)	2 (2 to 2)	61 (48 to 76)	14 (10 to 18)	37 (25 to 52)	4 (2 to 8)	6 (6 to 6)
Niger	1999	22 (17 to 28)	5 (4 to 7)	14 (10 to 20)	2 (1 to 3)	1 (1 to 1)	58 (46 to 74)	14 (11 to 19)	37 (26 to 52)	4 (2 to 7)	3 (3 to 3)
Niger	2000	22 (17 to 28)	5 (4 to 6)	14 (9 to 19)	1 (1 to 3)	2 (2 to 2)	57 (44 to 73)	13 (10 to 16)	36 (25 to 51)	4 (2 to 7)	4 (4 to 4)
Niger	2001	23 (18 to 28)	5 (4 to 6)	14 (10 to 20)	1 (1 to 3)	2 (2 to 2)	59 (47 to 75)	13 (10 to 17)	37 (26 to 52)	4 (2 to 7)	6 (6 to 6)
Niger	2002	23 (19 to 30)	5 (4 to 7)	14 (10 to 20)	2 (1 to 3)	2 (2 to 2)	62 (50 to 77)	14 (10 to 17)	38 (26 to 53)	4 (2 to 7)	7 (7 to 7)
Niger	2003	25 (20 to 31)	5 (4 to 7)	14 (10 to 20)	1 (1 to 3)	4 (4 to 4)	65 (53 to 81)	13 (10 to 17)	37 (26 to 51)	4 (2 to 7)	11 (11 to 11)
Niger	2004	25 (20 to 31)	6 (4 to 7)	14 (10 to 20)	1 (1 to 3)	3 (3 to 3)	65 (53 to 80)	15 (11 to 19)	37 (26 to 52)	4 (2 to 7)	9 (9 to 9)

Niger	2005	25 (20 to 31)	6 (5 to 8)	14 (10 to 19)	1 (1 to 3)	3 (3 to 3)	66 (54 to 81)	17 (13 to 21)	37 (26 to 51)	4 (2 to 7)	9 (9 to 9)
Niger	2006	28 (23 to 33)	7 (5 to 8)	14 (10 to 19)	1 (1 to 3)	6 (6 to 6)	72 (60 to 87)	17 (13 to 22)	37 (26 to 51)	4 (2 to 7)	15 (15 to 15)
Niger	2007	27 (23 to 33)	7 (5 to 9)	14 (10 to 20)	2 (1 to 3)	5 (5 to 5)	72 (60 to 87)	18 (14 to 24)	37 (27 to 52)	4 (2 to 7)	12 (12 to 12)
Niger	2008	29 (24 to 35)	7 (5 to 9)	14 (10 to 20)	1 (1 to 3)	6 (6 to 6)	76 (64 to 91)	18 (14 to 24)	37 (27 to 51)	4 (2 to 7)	17 (17 to 17)
Niger	2009	26 (22 to 32)	7 (5 to 9)	14 (10 to 19)	1 (1 to 3)	4 (4 to 4)	69 (58 to 85)	18 (13 to 23)	37 (26 to 51)	4 (2 to 7)	11 (11 to 11)
Niger	2010	24 (20 to 30)	6 (4 to 8)	13 (9 to 18)	1 (1 to 2)	4 (4 to 4)	64 (53 to 79)	16 (12 to 21)	35 (25 to 48)	3 (1 to 6)	10 (10 to 10)
Niger	2011	23 (19 to 28)	5 (4 to 7)	12 (9 to 17)	1 (0 to 2)	5 (5 to 5)	61 (51 to 75)	14 (10 to 19)	33 (24 to 45)	2 (1 to 4)	12 (12 to 12)
Niger	2012	22 (18 to 27)	6 (4 to 8)	13 (9 to 18)	1 (0 to 1)	2 (2 to 2)	57 (46 to 70)	15 (11 to 20)	34 (24 to 46)	2 (1 to 3)	7 (7 to 7)
Niger	2013	24 (19 to 29)	6 (5 to 8)	13 (9 to 18)	1 (0 to 1)	4 (4 to 4)	62 (51 to 76)	17 (12 to 22)	34 (24 to 46)	2 (1 to 3)	10 (10 to 10)
Niger	2014	25 (21 to 31)	7 (5 to 9)	13 (10 to 19)	1 (0 to 1)	4 (4 to 4)	66 (55 to 81)	18 (13 to 23)	35 (25 to 49)	2 (1 to 3)	12 (12 to 12)
Niger	2015	26 (21 to 32)	7 (5 to 9)	14 (10 to 19)	1 (0 to 1)	4 (4 to 4)	68 (55 to 83)	18 (13 to 24)	37 (26 to 51)	2 (1 to 4)	10 (10 to 10)
Niger	2016	25 (20 to 30)	6 (5 to 8)	14 (9 to 19)	1 (0 to 1)	4 (4 to 4)	65 (52 to 80)	17 (13 to 22)	36 (25 to 50)	2 (1 to 3)	10 (10 to 10)
Niger	2017	26 (21 to 33)	7 (6 to 10)	14 (10 to 20)	1 (0 to 1)	4 (4 to 4)	69 (56 to 86)	19 (15 to 26)	38 (26 to 53)	2 (1 to 3)	11 (11 to 11)
Niger	2018	28 (22 to 34)	9 (6 to 11)	15 (10 to 21)	1 (0 to 1)	4 (4 to 4)	73 (58 to 90)	23 (17 to 30)	39 (27 to 54)	2 (1 to 4)	10 (10 to 10)
Niger	2019	29 (23 to 36)	9 (7 to 12)	15 (10 to 21)	1 (0 to 1)	4 (4 to 4)	75 (61 to 94)	24 (18 to 32)	40 (27 to 55)	2 (1 to 4)	10 (10 to 10)

Niger	2020	29 (23 to 36)	10 (7 to 13)	15 (11 to 21)	1 (0 to 2)	4 (3 to 4)	77 (61 to 94)	25 (19 to 33)	40 (28 to 56)	2 (1 to 4)	10 (9 to 10)
Niger	2021	30 (24 to 37)	10 (7 to 13)	15 (11 to 22)	1 (0 to 2)	4 (3 to 4)	78 (62 to 97)	26 (19 to 34)	41 (28 to 57)	2 (1 to 4)	9 (9 to 10)
Niger	2022	30 (24 to 37)	10 (8 to 13)	16 (11 to 22)	1 (0 to 2)	4 (3 to 4)	79 (62 to 97)	27 (20 to 35)	41 (28 to 57)	2 (1 to 4)	9 (9 to 10)
Niger	2023	30 (24 to 38)	10 (8 to 14)	16 (11 to 22)	1 (0 to 2)	3 (3 to 4)	80 (64 to 99)	27 (20 to 36)	41 (29 to 58)	2 (1 to 4)	9 (8 to 10)
Niger	2024	31 (24 to 38)	11 (8 to 14)	16 (11 to 22)	1 (0 to 2)	3 (3 to 4)	81 (64 to 99)	28 (20 to 37)	42 (29 to 58)	2 (1 to 4)	9 (8 to 10)
Niger	2025	31 (25 to 39)	11 (8 to 15)	16 (11 to 22)	1 (0 to 2)	3 (3 to 4)	82 (65 to 101)	29 (21 to 38)	42 (29 to 59)	2 (1 to 4)	9 (8 to 10)
Niger	2026	31 (25 to 39)	11 (8 to 15)	16 (11 to 23)	1 (0 to 2)	3 (3 to 4)	82 (65 to 102)	29 (21 to 38)	42 (29 to 59)	2 (1 to 5)	9 (8 to 10)
Niger	2027	32 (25 to 39)	11 (8 to 15)	16 (11 to 23)	1 (0 to 2)	3 (3 to 4)	83 (66 to 103)	30 (22 to 39)	43 (30 to 60)	2 (1 to 5)	8 (7 to 10)
Niger	2028	32 (25 to 40)	12 (9 to 15)	16 (11 to 23)	1 (0 to 2)	3 (3 to 4)	85 (67 to 105)	31 (22 to 40)	43 (30 to 60)	2 (1 to 5)	8 (7 to 10)
Niger	2029	33 (26 to 41)	12 (9 to 16)	17 (11 to 23)	1 (0 to 2)	3 (3 to 4)	86 (68 to 107)	32 (23 to 42)	44 (30 to 61)	3 (1 to 5)	8 (7 to 9)
Niger	2030	33 (26 to 41)	12 (9 to 16)	17 (12 to 23)	1 (0 to 2)	3 (3 to 4)	87 (68 to 108)	33 (24 to 43)	44 (30 to 62)	3 (1 to 5)	8 (7 to 9)
Niger	2031	34 (27 to 42)	13 (9 to 17)	17 (12 to 24)	1 (0 to 2)	3 (3 to 4)	88 (70 to 110)	34 (24 to 44)	44 (31 to 62)	3 (1 to 5)	8 (7 to 9)
Niger	2032	34 (27 to 42)	13 (10 to 17)	17 (12 to 24)	1 (1 to 2)	3 (2 to 4)	90 (70 to 110)	34 (25 to 45)	45 (31 to 63)	3 (1 to 5)	8 (6 to 9)
Niger	2033	35 (28 to 43)	13 (10 to 18)	17 (12 to 24)	1 (1 to 2)	3 (2 to 3)	91 (72 to 114)	35 (26 to 47)	45 (31 to 63)	3 (1 to 5)	7 (6 to 9)
Niger	2034	35 (27 to 43)	14 (10 to 19)	17 (12 to 24)	1 (1 to 2)	3 (2 to 3)	92 (72 to 114)	36 (26 to 49)	46 (32 to 64)	3 (1 to 5)	7 (6 to 9)

Niger	2035	36 (28 to 45)	14 (10 to 19)	18 (12 to 25)	1 (1 to 2)	3 (2 to 3)	94 (74 to 117)	37 (27 to 50)	46 (32 to 64)	3 (1 to 5)	7 (6 to 9)
Niger	2036	36 (28 to 45)	15 (11 to 19)	18 (12 to 25)	1 (1 to 2)	3 (2 to 3)	95 (75 to 118)	38 (28 to 51)	46 (32 to 65)	3 (1 to 6)	7 (6 to 9)
Niger	2037	37 (29 to 46)	15 (11 to 20)	18 (12 to 25)	1 (1 to 2)	3 (2 to 3)	96 (76 to 120)	39 (28 to 52)	47 (32 to 65)	3 (1 to 6)	7 (6 to 9)
Niger	2038	37 (29 to 46)	15 (11 to 20)	18 (12 to 25)	1 (1 to 2)	3 (2 to 3)	98 (76 to 121)	40 (29 to 54)	47 (33 to 66)	3 (2 to 6)	7 (6 to 9)
Niger	2039	38 (30 to 47)	16 (11 to 21)	18 (13 to 25)	1 (1 to 2)	3 (2 to 3)	99 (78 to 123)	41 (30 to 55)	48 (33 to 66)	3 (2 to 6)	7 (6 to 9)
Niger	2040	38 (30 to 48)	16 (12 to 22)	18 (13 to 26)	1 (1 to 2)	3 (2 to 3)	101 (79 to 125)	42 (30 to 56)	48 (33 to 67)	3 (2 to 6)	7 (5 to 8)
Niger	2041	39 (30 to 48)	17 (12 to 22)	19 (13 to 26)	1 (1 to 2)	2 (2 to 3)	102 (80 to 127)	44 (31 to 58)	49 (34 to 68)	3 (2 to 6)	7 (5 to 8)
Niger	2042	40 (31 to 49)	17 (12 to 23)	19 (13 to 26)	1 (1 to 2)	2 (2 to 3)	104 (81 to 129)	45 (32 to 60)	49 (34 to 69)	3 (2 to 6)	6 (5 to 8)
Niger	2043	40 (32 to 50)	18 (13 to 24)	19 (13 to 26)	1 (1 to 2)	2 (2 to 3)	106 (83 to 132)	46 (33 to 62)	50 (34 to 69)	4 (2 to 7)	6 (5 to 8)
Niger	2044	41 (32 to 51)	18 (13 to 24)	19 (13 to 27)	1 (1 to 3)	2 (2 to 3)	108 (84 to 134)	48 (34 to 64)	51 (35 to 70)	4 (2 to 7)	6 (5 to 8)
Niger	2045	42 (33 to 53)	19 (13 to 25)	20 (13 to 27)	1 (1 to 3)	2 (2 to 3)	110 (86 to 138)	49 (35 to 67)	51 (35 to 71)	4 (2 to 7)	6 (5 to 8)
Niger	2046	43 (33 to 53)	19 (14 to 26)	20 (14 to 28)	1 (1 to 3)	2 (2 to 3)	112 (87 to 140)	51 (36 to 68)	52 (35 to 72)	4 (2 to 7)	6 (5 to 8)
Niger	2047	44 (34 to 55)	20 (14 to 27)	20 (14 to 28)	2 (1 to 3)	2 (2 to 3)	115 (89 to 143)	52 (37 to 71)	53 (36 to 73)	4 (2 to 7)	6 (5 to 8)
Niger	2048	45 (35 to 56)	21 (14 to 28)	20 (14 to 28)	2 (1 to 3)	2 (2 to 3)	117 (91 to 147)	54 (37 to 74)	53 (37 to 74)	4 (2 to 8)	6 (5 to 8)
Niger	2049	46 (35 to 57)	21 (15 to 29)	21 (14 to 29)	2 (1 to 3)	2 (2 to 3)	120 (93 to 150)	55 (39 to 76)	54 (37 to 75)	4 (2 to 8)	6 (4 to 8)

Niger	2050	47 (36 to 58)	22 (15 to 30)	21 (14 to 29)	2 (1 to 3)	2 (2 to 3)	122 (95 to 153)	57 (40 to 79)	55 (38 to 77)	4 (2 to 8)	6 (4 to 8)
Nigeria	1995	38 (30 to 47)	8 (6 to 10)	27 (20 to 36)	2 (1 to 4)	0 (0 to 0)	103 (81 to 129)	22 (17 to 28)	74 (54 to 99)	6 (3 to 12)	1 (1 to 1)
Nigeria	1996	37 (29 to 46)	8 (6 to 10)	27 (19 to 36)	2 (1 to 4)	0 (0 to 0)	100 (79 to 125)	21 (16 to 26)	73 (53 to 98)	6 (3 to 11)	1 (1 to 1)
Nigeria	1997	38 (30 to 46)	8 (6 to 10)	27 (20 to 36)	2 (1 to 4)	0 (0 to 0)	103 (82 to 127)	22 (17 to 28)	74 (54 to 98)	6 (3 to 11)	1 (1 to 1)
Nigeria	1998	39 (31 to 48)	8 (6 to 11)	28 (20 to 36)	2 (1 to 4)	1 (1 to 1)	106 (85 to 130)	23 (18 to 29)	76 (56 to 99)	6 (3 to 11)	1 (1 to 1)
Nigeria	1999	39 (31 to 48)	9 (7 to 11)	28 (20 to 36)	2 (1 to 4)	1 (1 to 1)	107 (85 to 131)	24 (18 to 31)	75 (55 to 99)	6 (3 to 11)	2 (2 to 2)
Nigeria	2000	40 (32 to 50)	9 (7 to 12)	27 (20 to 36)	2 (1 to 4)	2 (2 to 2)	110 (89 to 135)	25 (19 to 32)	74 (55 to 98)	6 (3 to 11)	5 (5 to 5)
Nigeria	2001	45 (36 to 55)	11 (9 to 14)	30 (22 to 41)	3 (1 to 5)	1 (1 to 1)	122 (98 to 151)	31 (24 to 39)	82 (61 to 111)	7 (3 to 13)	3 (3 to 3)
Nigeria	2002	42 (34 to 53)	9 (7 to 12)	30 (22 to 39)	2 (1 to 4)	1 (1 to 1)	116 (94 to 144)	26 (20 to 33)	81 (61 to 108)	6 (3 to 10)	3 (3 to 3)
Nigeria	2003	57 (46 to 71)	13 (10 to 17)	41 (31 to 53)	2 (1 to 5)	1 (1 to 1)	157 (127 to 193)	36 (27 to 45)	111 (84 to 144)	7 (3 to 12)	4 (4 to 4)
Nigeria	2004	62 (51 to 75)	14 (11 to 18)	44 (33 to 57)	2 (1 to 4)	2 (2 to 2)	170 (139 to 206)	38 (29 to 48)	120 (91 to 155)	6 (3 to 12)	6 (6 to 6)
Nigeria	2005	68 (56 to 83)	15 (12 to 19)	48 (37 to 62)	2 (1 to 4)	3 (3 to 3)	186 (152 to 226)	41 (32 to 53)	131 (100 to 168)	6 (3 to 12)	7 (7 to 7)
Nigeria	2006	62 (51 to 75)	12 (9 to 15)	45 (34 to 57)	2 (1 to 4)	4 (4 to 4)	170 (139 to 205)	32 (25 to 41)	123 (94 to 156)	5 (2 to 10)	10 (10 to 10)
Nigeria	2007	73 (59 to 88)	15 (11 to 19)	52 (40 to 66)	3 (1 to 5)	4 (4 to 4)	199 (162 to 240)	40 (30 to 51)	142 (108 to 180)	7 (3 to 13)	10 (10 to 10)
Nigeria	2008	68 (55 to 82)	12 (9 to 15)	49 (37 to 62)	2 (1 to 4)	5 (5 to 5)	185 (150 to 224)	32 (24 to 41)	134 (102 to 170)	6 (3 to 11)	13 (13 to 13)

Nigeria	2009	72 (59 to 86)	12 (9 to 15)	51 (39 to 66)	2 (1 to 4)	7 (7 to 7)	195 (160 to 235)	31 (24 to 40)	140 (107 to 179)	6 (3 to 12)	18 (18 to 18)
Nigeria	2010	72 (59 to 86)	12 (9 to 15)	53 (41 to 67)	2 (1 to 4)	5 (5 to 5)	196 (162 to 236)	32 (24 to 40)	145 (111 to 183)	6 (3 to 12)	13 (13 to 13)
Nigeria	2011	73 (60 to 88)	12 (9 to 15)	54 (42 to 68)	2 (1 to 4)	5 (5 to 5)	199 (163 to 240)	32 (25 to 41)	148 (114 to 186)	6 (3 to 12)	13 (13 to 13)
Nigeria	2012	71 (58 to 86)	11 (8 to 14)	53 (41 to 67)	2 (1 to 4)	6 (6 to 6)	195 (160 to 235)	30 (23 to 39)	144 (111 to 183)	5 (2 to 10)	15 (15 to 15)
Nigeria	2013	75 (61 to 89)	11 (8 to 14)	54 (41 to 69)	2 (1 to 4)	8 (8 to 8)	203 (167 to 242)	30 (23 to 40)	148 (113 to 188)	5 (2 to 10)	21 (21 to 21)
Nigeria	2014	75 (61 to 91)	11 (9 to 15)	56 (43 to 71)	2 (1 to 3)	6 (6 to 6)	205 (167 to 247)	31 (23 to 40)	154 (117 to 194)	5 (2 to 9)	16 (16 to 16)
Nigeria	2015	77 (63 to 93)	11 (9 to 15)	58 (43 to 73)	2 (1 to 3)	7 (7 to 7)	212 (173 to 255)	31 (23 to 40)	158 (119 to 200)	4 (2 to 8)	19 (19 to 19)
Nigeria	2016	72 (58 to 88)	10 (8 to 13)	56 (42 to 71)	1 (1 to 2)	5 (5 to 5)	198 (159 to 240)	27 (21 to 36)	153 (115 to 194)	3 (1 to 6)	15 (15 to 15)
Nigeria	2017	78 (62 to 96)	11 (9 to 15)	60 (45 to 78)	1 (0 to 2)	5 (5 to 5)	212 (170 to 262)	31 (23 to 40)	163 (123 to 212)	3 (1 to 5)	15 (15 to 15)
Nigeria	2018	78 (62 to 97)	12 (9 to 16)	60 (45 to 78)	1 (0 to 2)	5 (5 to 5)	213 (170 to 265)	32 (24 to 43)	165 (124 to 213)	3 (1 to 5)	14 (14 to 14)
Nigeria	2019	77 (62 to 96)	11 (8 to 15)	60 (45 to 78)	1 (0 to 2)	5 (5 to 5)	211 (169 to 262)	31 (22 to 41)	164 (123 to 213)	3 (1 to 5)	14 (14 to 14)
Nigeria	2020	77 (61 to 97)	11 (8 to 16)	60 (45 to 78)	1 (0 to 2)	5 (5 to 5)	211 (167 to 264)	31 (22 to 43)	163 (123 to 213)	3 (1 to 5)	14 (13 to 15)
Nigeria	2021	78 (62 to 97)	12 (8 to 16)	60 (45 to 78)	1 (0 to 2)	5 (5 to 6)	213 (170 to 265)	32 (23 to 43)	164 (124 to 214)	3 (1 to 5)	14 (13 to 15)
Nigeria	2022	79 (62 to 97)	12 (8 to 17)	61 (46 to 79)	1 (0 to 2)	5 (5 to 6)	215 (170 to 266)	32 (22 to 45)	166 (124 to 216)	3 (1 to 5)	14 (13 to 16)
Nigeria	2023	79 (62 to 98)	12 (8 to 17)	61 (46 to 80)	1 (0 to 2)	5 (5 to 6)	217 (170 to 269)	33 (23 to 47)	167 (126 to 218)	3 (1 to 5)	14 (13 to 16)

Nigeria	2024	80 (63 to 100)	12 (8 to 18)	62 (47 to 81)	1 (0 to 2)	5 (5 to 6)	219 (173 to 272)	34 (22 to 49)	169 (127 to 220)	3 (1 to 5)	14 (13 to 16)
Nigeria	2025	81 (64 to 101)	13 (8 to 18)	62 (47 to 81)	1 (1 to 2)	5 (5 to 6)	222 (174 to 276)	35 (23 to 50)	170 (128 to 222)	3 (1 to 5)	14 (12 to 16)
Nigeria	2026	82 (65 to 101)	13 (9 to 19)	63 (47 to 82)	1 (1 to 2)	5 (4 to 6)	224 (177 to 277)	35 (23 to 51)	172 (130 to 225)	3 (1 to 6)	14 (12 to 16)
Nigeria	2027	83 (65 to 103)	13 (9 to 19)	64 (48 to 83)	1 (1 to 2)	5 (4 to 6)	226 (177 to 280)	36 (24 to 52)	174 (131 to 227)	3 (1 to 6)	14 (12 to 16)
Nigeria	2028	84 (67 to 104)	13 (9 to 19)	64 (49 to 84)	1 (1 to 2)	5 (4 to 6)	229 (182 to 285)	36 (24 to 53)	176 (133 to 230)	3 (1 to 6)	14 (12 to 16)
Nigeria	2029	85 (67 to 105)	13 (9 to 20)	65 (49 to 85)	1 (1 to 2)	5 (4 to 6)	231 (182 to 287)	37 (24 to 54)	178 (134 to 232)	3 (1 to 6)	14 (12 to 16)
Nigeria	2030	85 (68 to 107)	14 (9 to 20)	66 (50 to 86)	1 (1 to 2)	5 (4 to 6)	233 (185 to 291)	37 (25 to 54)	179 (135 to 234)	3 (2 to 6)	14 (12 to 16)
Nigeria	2031	86 (68 to 107)	14 (9 to 20)	66 (50 to 87)	1 (1 to 2)	5 (4 to 6)	236 (186 to 293)	38 (25 to 55)	181 (137 to 237)	3 (2 to 6)	13 (12 to 16)
Nigeria	2032	87 (69 to 110)	14 (9 to 21)	67 (51 to 88)	1 (1 to 2)	5 (4 to 6)	238 (189 to 300)	38 (25 to 56)	183 (138 to 240)	3 (2 to 6)	13 (11 to 16)
Nigeria	2033	88 (69 to 109)	14 (9 to 21)	68 (51 to 89)	1 (1 to 2)	5 (4 to 6)	241 (189 to 299)	39 (26 to 57)	185 (139 to 242)	3 (2 to 6)	13 (11 to 16)
Nigeria	2034	89 (71 to 112)	14 (10 to 21)	68 (51 to 90)	1 (1 to 2)	5 (4 to 6)	243 (193 to 305)	39 (26 to 58)	187 (140 to 245)	4 (2 to 6)	13 (11 to 16)
Nigeria	2035	90 (71 to 112)	15 (10 to 22)	69 (52 to 90)	1 (1 to 2)	5 (4 to 6)	246 (193 to 307)	40 (26 to 59)	188 (141 to 247)	4 (2 to 6)	13 (11 to 16)
Nigeria	2036	91 (72 to 114)	15 (10 to 22)	70 (52 to 92)	1 (1 to 2)	5 (4 to 6)	248 (196 to 312)	41 (27 to 60)	190 (143 to 250)	4 (2 to 7)	13 (11 to 17)
Nigeria	2037	92 (72 to 113)	15 (10 to 23)	70 (53 to 93)	1 (1 to 2)	5 (4 to 6)	251 (196 to 310)	41 (27 to 61)	192 (144 to 253)	4 (2 to 7)	13 (11 to 16)
Nigeria	2038	93 (73 to 117)	15 (10 to 23)	71 (53 to 94)	1 (1 to 2)	5 (4 to 6)	253 (200 to 320)	42 (28 to 63)	194 (145 to 256)	4 (2 to 7)	13 (11 to 17)

Nigeria	2039	94 (73 to 117)	16 (10 to 23)	72 (54 to 95)	1 (1 to 3)	5 (4 to 6)	256 (200 to 320)	43 (28 to 64)	196 (146 to 259)	4 (2 to 7)	13 (11 to 17)
Nigeria	2040	95 (75 to 119)	16 (10 to 24)	73 (54 to 96)	1 (1 to 3)	5 (4 to 6)	260 (204 to 326)	43 (28 to 65)	199 (148 to 262)	4 (2 to 7)	13 (11 to 17)
Nigeria	2041	96 (75 to 121)	16 (11 to 24)	74 (55 to 97)	1 (1 to 3)	5 (4 to 6)	263 (206 to 330)	44 (29 to 67)	201 (150 to 266)	4 (2 to 7)	13 (11 to 17)
Nigeria	2042	98 (77 to 123)	16 (11 to 25)	75 (56 to 98)	1 (1 to 3)	5 (4 to 6)	266 (210 to 336)	45 (30 to 68)	204 (152 to 268)	4 (2 to 7)	13 (11 to 17)
Nigeria	2043	99 (78 to 125)	17 (11 to 26)	76 (56 to 100)	1 (1 to 3)	5 (4 to 6)	270 (212 to 340)	46 (30 to 70)	207 (154 to 272)	4 (2 to 7)	13 (10 to 17)
Nigeria	2044	100 (79 to 127)	17 (11 to 26)	77 (57 to 101)	2 (1 to 3)	5 (4 to 6)	274 (216 to 347)	47 (31 to 71)	210 (156 to 276)	4 (2 to 8)	13 (10 to 17)
Nigeria	2045	102 (80 to 128)	18 (11 to 27)	78 (58 to 102)	2 (1 to 3)	5 (4 to 6)	279 (217 to 350)	48 (31 to 73)	213 (159 to 280)	4 (2 to 8)	13 (10 to 17)
Nigeria	2046	104 (82 to 131)	18 (12 to 27)	79 (59 to 104)	2 (1 to 3)	5 (4 to 6)	283 (223 to 359)	49 (32 to 74)	217 (161 to 285)	4 (2 to 8)	13 (10 to 17)
Nigeria	2047	105 (82 to 132)	18 (12 to 28)	81 (60 to 106)	2 (1 to 3)	5 (4 to 6)	288 (224 to 360)	50 (33 to 76)	220 (164 to 290)	4 (2 to 8)	13 (10 to 18)
Nigeria	2048	107 (84 to 136)	19 (12 to 28)	82 (61 to 108)	2 (1 to 3)	5 (4 to 6)	292 (228 to 373)	51 (33 to 77)	223 (166 to 295)	5 (2 to 8)	13 (10 to 18)
Nigeria	2049	109 (85 to 135)	19 (13 to 29)	83 (62 to 110)	2 (1 to 3)	5 (4 to 6)	297 (232 to 368)	52 (34 to 79)	227 (169 to 300)	5 (2 to 8)	13 (10 to 18)
Nigeria	2050	110 (87 to 141)	20 (13 to 30)	84 (63 to 112)	2 (1 to 3)	5 (4 to 6)	302 (236 to 384)	54 (35 to 81)	230 (172 to 306)	5 (2 to 9)	13 (10 to 18)
North Korea	1995	65 (53 to 79)	39 (30 to 49)	24 (17 to 34)	2 (1 to 3)	0 (0 to 0)	38 (31 to 46)	23 (17 to 29)	14 (10 to 20)	1 (1 to 2)	0 (0 to 0)
North Korea	1996	62 (51 to 76)	37 (29 to 47)	24 (17 to 33)	2 (1 to 3)	0 (0 to 0)	36 (30 to 44)	22 (17 to 27)	14 (10 to 20)	1 (0 to 2)	0 (0 to 0)
North Korea	1997	60 (50 to 74)	36 (28 to 44)	23 (16 to 33)	2 (1 to 3)	0 (0 to 0)	35 (29 to 43)	21 (16 to 26)	14 (9 to 19)	1 (0 to 2)	0 (0 to 0)

North Korea	1998	62 (50 to 75)	36 (29 to 45)	24 (16 to 34)	1 (1 to 3)	0 (0 to 0)	36 (29 to 44)	21 (17 to 26)	14 (10 to 20)	1 (0 to 2)	0 (0 to 0)
North Korea	1999	65 (53 to 79)	38 (30 to 48)	25 (17 to 35)	1 (1 to 3)	0 (0 to 0)	38 (31 to 46)	22 (17 to 28)	14 (10 to 20)	1 (0 to 2)	0 (0 to 0)
North Korea	2000	65 (54 to 79)	39 (30 to 48)	25 (17 to 36)	1 (1 to 3)	0 (0 to 0)	38 (31 to 46)	23 (18 to 28)	15 (10 to 21)	1 (0 to 2)	0 (0 to 0)
North Korea	2001	67 (55 to 81)	40 (31 to 50)	26 (18 to 37)	1 (1 to 3)	0 (0 to 0)	39 (32 to 47)	23 (18 to 29)	15 (10 to 21)	1 (0 to 2)	0 (0 to 0)
North Korea	2002	68 (56 to 84)	40 (31 to 51)	26 (18 to 37)	1 (1 to 2)	0 (0 to 0)	40 (32 to 49)	23 (18 to 30)	15 (10 to 22)	1 (0 to 1)	0 (0 to 0)
North Korea	2003	69 (57 to 85)	41 (32 to 52)	27 (18 to 38)	1 (1 to 3)	0 (0 to 0)	40 (33 to 50)	24 (19 to 30)	16 (11 to 22)	1 (0 to 2)	0 (0 to 0)
North Korea	2004	71 (57 to 87)	41 (33 to 52)	28 (19 to 39)	1 (1 to 3)	0 (0 to 0)	41 (33 to 51)	24 (19 to 30)	16 (11 to 23)	1 (0 to 2)	0 (0 to 0)
North Korea	2005	73 (59 to 89)	43 (33 to 54)	29 (19 to 40)	1 (1 to 3)	0 (0 to 0)	42 (35 to 52)	25 (19 to 31)	17 (11 to 23)	1 (0 to 2)	0 (0 to 0)
North Korea	2006	72 (58 to 87)	42 (33 to 54)	28 (19 to 39)	1 (1 to 3)	0 (0 to 0)	42 (34 to 51)	25 (19 to 31)	16 (11 to 23)	1 (0 to 2)	0 (0 to 0)
North Korea	2007	71 (58 to 87)	42 (32 to 53)	28 (19 to 39)	1 (1 to 3)	0 (0 to 0)	42 (34 to 50)	24 (19 to 31)	16 (11 to 23)	1 (0 to 2)	0 (0 to 0)
North Korea	2008	72 (58 to 88)	43 (33 to 55)	28 (19 to 39)	2 (1 to 3)	0 (0 to 0)	42 (34 to 52)	25 (19 to 32)	16 (11 to 23)	1 (0 to 2)	0 (0 to 0)
North Korea	2009	72 (58 to 88)	43 (33 to 54)	28 (19 to 39)	2 (1 to 3)	0 (0 to 0)	42 (34 to 51)	25 (19 to 32)	16 (11 to 23)	1 (0 to 2)	0 (0 to 0)
North Korea	2010	72 (58 to 88)	43 (33 to 55)	27 (19 to 38)	2 (1 to 3)	0 (0 to 0)	42 (34 to 51)	25 (19 to 32)	16 (11 to 22)	1 (0 to 2)	0 (0 to 0)
North Korea	2011	72 (59 to 89)	43 (33 to 56)	27 (19 to 38)	2 (1 to 3)	0 (0 to 0)	42 (34 to 52)	25 (19 to 33)	16 (11 to 22)	1 (0 to 2)	0 (0 to 0)
North Korea	2012	73 (59 to 89)	44 (33 to 57)	27 (18 to 39)	2 (1 to 3)	0 (0 to 0)	43 (34 to 52)	26 (20 to 33)	16 (11 to 23)	1 (0 to 2)	0 (0 to 0)

North Korea	2013	73 (60 to 90)	44 (33 to 58)	27 (18 to 38)	2 (1 to 3)	0 (0 to 0)	43 (35 to 53)	26 (19 to 34)	16 (11 to 22)	1 (0 to 2)	0 (0 to 0)
North Korea	2014	74 (60 to 91)	45 (34 to 59)	27 (18 to 38)	2 (1 to 3)	0 (0 to 0)	43 (35 to 53)	26 (20 to 34)	16 (11 to 22)	1 (0 to 2)	0 (0 to 0)
North Korea	2015	74 (60 to 90)	45 (34 to 59)	27 (18 to 38)	2 (1 to 3)	0 (0 to 0)	43 (35 to 53)	26 (20 to 34)	16 (11 to 22)	1 (0 to 2)	0 (0 to 0)
North Korea	2016	75 (60 to 92)	46 (34 to 60)	27 (18 to 38)	2 (1 to 3)	0 (0 to 0)	44 (35 to 54)	27 (20 to 35)	16 (11 to 22)	1 (0 to 2)	0 (0 to 0)
North Korea	2017	77 (60 to 96)	47 (34 to 63)	27 (19 to 38)	2 (1 to 3)	1 (1 to 1)	45 (35 to 56)	28 (20 to 37)	16 (11 to 22)	1 (0 to 2)	0 (0 to 0)
North Korea	2018	78 (61 to 98)	48 (35 to 64)	27 (19 to 38)	2 (1 to 3)	0 (0 to 0)	45 (35 to 57)	28 (20 to 37)	16 (11 to 22)	1 (0 to 2)	0 (0 to 0)
North Korea	2019	79 (61 to 98)	49 (35 to 65)	28 (19 to 39)	2 (1 to 3)	1 (0 to 1)	46 (36 to 57)	28 (21 to 38)	16 (11 to 23)	1 (0 to 2)	0 (0 to 0)
North Korea	2020	80 (62 to 101)	50 (36 to 66)	28 (20 to 39)	2 (1 to 3)	1 (0 to 1)	47 (36 to 59)	29 (21 to 39)	16 (11 to 23)	1 (0 to 2)	0 (0 to 0)
North Korea	2021	81 (63 to 102)	51 (37 to 67)	28 (20 to 39)	2 (1 to 3)	1 (0 to 1)	47 (37 to 60)	30 (22 to 39)	16 (11 to 23)	1 (0 to 2)	0 (0 to 0)
North Korea	2022	82 (64 to 102)	52 (38 to 68)	28 (20 to 40)	2 (1 to 3)	1 (0 to 1)	48 (37 to 60)	30 (22 to 40)	17 (11 to 23)	1 (0 to 2)	0 (0 to 0)
North Korea	2023	83 (65 to 105)	53 (38 to 70)	28 (20 to 40)	2 (1 to 3)	1 (1 to 1)	49 (38 to 61)	31 (22 to 41)	17 (12 to 23)	1 (0 to 2)	0 (0 to 0)
North Korea	2024	84 (65 to 106)	53 (39 to 71)	28 (20 to 40)	2 (1 to 3)	1 (1 to 1)	49 (38 to 62)	31 (23 to 41)	17 (12 to 23)	1 (0 to 2)	0 (0 to 0)
North Korea	2025	85 (66 to 107)	54 (40 to 72)	28 (20 to 40)	2 (1 to 3)	1 (1 to 1)	50 (39 to 62)	32 (23 to 42)	17 (12 to 23)	1 (0 to 2)	0 (0 to 0)
North Korea	2026	86 (66 to 108)	55 (40 to 73)	28 (20 to 40)	2 (1 to 4)	1 (1 to 1)	50 (39 to 63)	32 (23 to 42)	17 (12 to 23)	1 (0 to 2)	0 (0 to 0)
North Korea	2027	86 (67 to 108)	55 (41 to 73)	28 (20 to 40)	2 (1 to 4)	1 (1 to 1)	50 (39 to 63)	32 (24 to 43)	17 (12 to 23)	1 (0 to 2)	0 (0 to 0)

North Korea	2028	87 (67 to 109)	56 (41 to 74)	28 (20 to 40)	2 (1 to 4)	1 (1 to 1)	50 (39 to 64)	32 (24 to 43)	17 (11 to 23)	1 (0 to 2)	0 (0 to 0)
North Korea	2029	87 (68 to 109)	56 (41 to 74)	28 (20 to 40)	2 (1 to 4)	1 (1 to 1)	51 (40 to 63)	33 (24 to 43)	16 (11 to 23)	1 (0 to 2)	0 (0 to 0)
North Korea	2030	87 (68 to 110)	56 (41 to 74)	28 (20 to 39)	2 (1 to 4)	1 (1 to 1)	51 (39 to 64)	33 (24 to 43)	16 (11 to 23)	1 (0 to 2)	0 (0 to 1)
North Korea	2031	87 (68 to 109)	57 (42 to 75)	28 (19 to 39)	2 (1 to 4)	1 (1 to 1)	51 (40 to 64)	33 (24 to 44)	16 (11 to 23)	1 (0 to 2)	0 (0 to 1)
North Korea	2032	87 (68 to 110)	57 (42 to 75)	28 (19 to 39)	2 (1 to 4)	1 (1 to 1)	51 (40 to 64)	33 (24 to 44)	16 (11 to 23)	1 (0 to 2)	0 (0 to 1)
North Korea	2033	87 (68 to 110)	57 (42 to 75)	28 (19 to 39)	2 (1 to 4)	1 (1 to 1)	51 (40 to 64)	33 (24 to 44)	16 (11 to 23)	1 (0 to 2)	0 (0 to 1)
North Korea	2034	87 (67 to 110)	57 (42 to 75)	27 (19 to 38)	2 (1 to 4)	1 (1 to 1)	51 (39 to 64)	33 (24 to 44)	16 (11 to 22)	1 (0 to 2)	1 (0 to 1)
North Korea	2035	87 (68 to 109)	57 (42 to 75)	27 (19 to 38)	2 (1 to 4)	1 (1 to 1)	51 (40 to 64)	33 (24 to 44)	16 (11 to 22)	1 (0 to 2)	1 (0 to 1)
North Korea	2036	87 (67 to 110)	57 (42 to 75)	27 (19 to 38)	2 (1 to 4)	1 (1 to 1)	51 (39 to 64)	33 (24 to 44)	16 (11 to 22)	1 (0 to 2)	1 (0 to 1)
North Korea	2037	87 (68 to 110)	57 (42 to 75)	27 (19 to 38)	2 (1 to 4)	1 (1 to 1)	51 (39 to 64)	33 (24 to 44)	16 (11 to 22)	1 (0 to 2)	1 (0 to 1)
North Korea	2038	87 (67 to 110)	58 (42 to 76)	27 (19 to 38)	2 (1 to 4)	1 (1 to 1)	51 (39 to 64)	34 (24 to 44)	16 (11 to 22)	1 (0 to 2)	1 (1 to 1)
North Korea	2039	88 (68 to 111)	58 (42 to 76)	27 (18 to 38)	2 (1 to 4)	1 (1 to 1)	51 (39 to 64)	34 (25 to 45)	16 (11 to 22)	1 (1 to 2)	1 (1 to 1)
North Korea	2040	88 (68 to 111)	58 (42 to 77)	27 (18 to 38)	2 (1 to 4)	1 (1 to 1)	51 (39 to 65)	34 (25 to 45)	16 (11 to 22)	1 (1 to 2)	1 (1 to 1)
North Korea	2041	89 (69 to 112)	59 (43 to 78)	27 (18 to 38)	2 (1 to 4)	1 (1 to 2)	52 (40 to 65)	34 (25 to 45)	16 (11 to 22)	1 (1 to 2)	1 (1 to 1)
North Korea	2042	90 (68 to 113)	60 (43 to 79)	27 (18 to 38)	2 (1 to 4)	1 (1 to 2)	52 (40 to 66)	35 (25 to 46)	16 (11 to 22)	1 (1 to 2)	1 (1 to 1)

North Korea	2043	90 (70 to 113)	60 (44 to 79)	27 (18 to 38)	2 (1 to 4)	1 (1 to 2)	53 (41 to 66)	35 (26 to 46)	16 (11 to 22)	1 (1 to 2)	1 (1 to 1)
North Korea	2044	91 (70 to 115)	61 (44 to 80)	27 (19 to 38)	2 (1 to 4)	1 (1 to 2)	53 (41 to 67)	35 (26 to 47)	16 (11 to 22)	1 (1 to 2)	1 (1 to 1)
North Korea	2045	92 (71 to 116)	61 (45 to 81)	27 (19 to 38)	2 (1 to 4)	1 (1 to 2)	53 (42 to 67)	36 (26 to 47)	16 (11 to 22)	1 (1 to 2)	1 (1 to 1)
North Korea	2046	92 (70 to 118)	62 (45 to 82)	27 (19 to 38)	2 (1 to 4)	1 (1 to 2)	54 (41 to 69)	36 (26 to 48)	16 (11 to 22)	1 (1 to 2)	1 (1 to 1)
North Korea	2047	93 (72 to 117)	62 (45 to 83)	27 (19 to 38)	2 (1 to 4)	2 (1 to 2)	54 (42 to 68)	36 (27 to 48)	16 (11 to 22)	1 (1 to 2)	1 (1 to 1)
North Korea	2048	94 (71 to 119)	63 (46 to 83)	27 (19 to 38)	2 (1 to 4)	2 (1 to 2)	55 (42 to 69)	37 (27 to 49)	16 (11 to 22)	1 (1 to 2)	1 (1 to 1)
North Korea	2049	95 (73 to 118)	64 (46 to 84)	27 (19 to 39)	2 (1 to 4)	2 (1 to 2)	55 (42 to 69)	37 (27 to 49)	16 (11 to 22)	1 (1 to 2)	1 (1 to 1)
North Korea	2050	95 (73 to 120)	64 (47 to 85)	27 (19 to 39)	2 (1 to 4)	2 (1 to 2)	55 (42 to 70)	37 (27 to 49)	16 (11 to 22)	1 (1 to 2)	1 (1 to 1)
North Macedonia	1995	194 (157 to 238)	120 (89 to 157)	61 (42 to 86)	6 (3 to 12)	6 (6 to 6)	524 (425 to 642)	326 (242 to 424)	166 (114 to 232)	17 (8 to 31)	16 (16 to 16)
North Macedonia	1996	193 (157 to 237)	121 (90 to 159)	62 (42 to 87)	6 (3 to 12)	4 (4 to 4)	523 (425 to 641)	328 (244 to 429)	167 (115 to 234)	17 (8 to 32)	11 (11 to 11)
North Macedonia	1997	195 (158 to 240)	123 (92 to 161)	63 (43 to 88)	7 (3 to 12)	3 (3 to 3)	527 (427 to 648)	333 (248 to 436)	169 (117 to 239)	18 (8 to 32)	7 (7 to 7)
North Macedonia	1998	197 (160 to 242)	126 (93 to 164)	64 (44 to 90)	7 (3 to 12)	1 (1 to 1)	533 (433 to 655)	339 (252 to 443)	172 (118 to 243)	18 (9 to 33)	4 (4 to 4)
North Macedonia	1999	222 (181 to 271)	137 (103 to 180)	68 (46 to 95)	7 (3 to 14)	10 (10 to 10)	600 (488 to 733)	370 (277 to 486)	185 (125 to 256)	20 (9 to 38)	26 (26 to 26)
North Macedonia	2000	228 (185 to 280)	147 (110 to 193)	73 (49 to 101)	8 (4 to 15)	0 (0 to 0)	617 (499 to 758)	397 (299 to 521)	197 (132 to 274)	22 (10 to 40)	1 (1 to 1)
North Macedonia	2001	258 (208 to 315)	164 (123 to 216)	79 (53 to 109)	9 (4 to 17)	5 (5 to 5)	697 (563 to 852)	444 (334 to 583)	214 (144 to 295)	25 (12 to 45)	15 (15 to 15)

North Macedonia	2002	262 (211 to 319)	168 (127 to 219)	82 (56 to 113)	10 (5 to 18)	1 (1 to 1)	707 (571 to 864)	455 (343 to 591)	222 (150 to 307)	26 (12 to 48)	4 (4 to 4)
North Macedonia	2003	261 (212 to 319)	166 (126 to 217)	84 (57 to 117)	10 (5 to 18)	2 (2 to 2)	706 (574 to 862)	449 (341 to 588)	226 (154 to 315)	27 (12 to 48)	4 (4 to 4)
North Macedonia	2004	267 (217 to 327)	167 (127 to 222)	87 (59 to 120)	10 (5 to 19)	3 (3 to 3)	723 (586 to 884)	452 (342 to 601)	234 (159 to 324)	28 (13 to 50)	9 (9 to 9)
North Macedonia	2005	277 (223 to 340)	172 (131 to 228)	91 (62 to 126)	11 (5 to 20)	3 (3 to 3)	749 (602 to 920)	465 (354 to 616)	245 (167 to 340)	29 (14 to 54)	9 (9 to 9)
North Macedonia	2006	288 (231 to 357)	177 (135 to 234)	94 (64 to 132)	11 (5 to 20)	4 (4 to 4)	777 (625 to 964)	479 (366 to 633)	255 (173 to 358)	31 (14 to 55)	12 (12 to 12)
North Macedonia	2007	307 (248 to 381)	188 (141 to 250)	100 (67 to 143)	12 (6 to 22)	6 (6 to 6)	829 (670 to 1030)	509 (382 to 675)	270 (182 to 387)	34 (15 to 59)	17 (17 to 17)
North Macedonia	2008	334 (267 to 412)	209 (158 to 275)	108 (73 to 153)	14 (7 to 25)	3 (3 to 3)	902 (722 to 1114)	566 (426 to 742)	291 (197 to 414)	38 (18 to 66)	7 (7 to 7)
North Macedonia	2009	343 (274 to 423)	216 (162 to 281)	111 (76 to 155)	15 (7 to 26)	1 (1 to 1)	926 (740 to 1143)	583 (437 to 758)	300 (205 to 418)	39 (18 to 71)	4 (4 to 4)
North Macedonia	2010	349 (279 to 428)	219 (163 to 286)	113 (76 to 157)	15 (7 to 27)	2 (2 to 2)	944 (755 to 1158)	591 (441 to 772)	306 (207 to 425)	40 (19 to 72)	6 (6 to 6)
North Macedonia	2011	353 (283 to 432)	219 (164 to 288)	115 (77 to 163)	15 (7 to 27)	4 (4 to 4)	953 (764 to 1169)	593 (444 to 778)	310 (209 to 442)	41 (19 to 72)	10 (10 to 10)
North Macedonia	2012	364 (292 to 447)	229 (170 to 299)	118 (81 to 167)	16 (8 to 29)	1 (1 to 1)	985 (791 to 1209)	620 (461 to 807)	320 (218 to 452)	43 (21 to 79)	3 (3 to 3)
North Macedonia	2013	372 (300 to 455)	233 (174 to 304)	120 (83 to 170)	16 (8 to 30)	3 (3 to 3)	1007 (810 to 1230)	629 (471 to 822)	326 (225 to 460)	44 (21 to 81)	8 (8 to 8)
North Macedonia	2014	388 (312 to 478)	244 (183 to 321)	125 (87 to 177)	17 (8 to 32)	1 (1 to 1)	1048 (844 to 1293)	660 (494 to 867)	338 (234 to 479)	47 (22 to 87)	3 (3 to 3)
North Macedonia	2015	410 (329 to 503)	259 (195 to 341)	131 (90 to 182)	19 (9 to 34)	2 (2 to 2)	1109 (890 to 1361)	700 (526 to 921)	353 (242 to 493)	50 (24 to 92)	6 (6 to 6)

North Macedonia	2016	417 (335 to 512)	263 (199 to 346)	133 (91 to 188)	19 (9 to 36)	1 (1 to 1)	1127 (904 to 1385)	712 (537 to 934)	360 (247 to 508)	52 (25 to 96)	3 (3 to 3)
North Macedonia	2017	433 (342 to 550)	274 (208 to 359)	137 (94 to 195)	20 (10 to 37)	1 (1 to 1)	1170 (925 to 1487)	741 (562 to 972)	372 (254 to 526)	54 (26 to 101)	2 (2 to 2)
North Macedonia	2018	437 (343 to 560)	275 (209 to 360)	141 (96 to 200)	20 (10 to 38)	1 (1 to 1)	1181 (928 to 1513)	742 (564 to 972)	382 (261 to 539)	55 (26 to 103)	2 (2 to 2)
North Macedonia	2019	472 (369 to 600)	303 (230 to 397)	146 (100 to 206)	22 (11 to 41)	1 (1 to 1)	1275 (999 to 1621)	819 (621 to 1073)	395 (270 to 556)	59 (28 to 111)	2 (2 to 2)
North Macedonia	2020	490 (383 to 623)	316 (240 to 414)	151 (104 to 213)	23 (11 to 42)	1 (1 to 1)	1325 (1034 to 1684)	853 (649 to 1119)	408 (280 to 575)	62 (29 to 115)	2 (2 to 2)
North Macedonia	2021	499 (392 to 636)	322 (245 to 423)	152 (105 to 215)	23 (11 to 43)	1 (1 to 1)	1349 (1059 to 1718)	871 (663 to 1144)	412 (285 to 580)	63 (30 to 117)	2 (2 to 3)
North Macedonia	2022	507 (398 to 645)	329 (250 to 431)	154 (106 to 217)	24 (11 to 44)	1 (1 to 1)	1371 (1076 to 1744)	889 (675 to 1165)	416 (287 to 586)	64 (31 to 119)	2 (2 to 3)
North Macedonia	2023	517 (408 to 655)	336 (255 to 440)	156 (107 to 219)	24 (12 to 45)	1 (1 to 1)	1397 (1103 to 1771)	909 (690 to 1191)	420 (289 to 591)	65 (31 to 122)	3 (2 to 3)
North Macedonia	2024	526 (413 to 660)	344 (261 to 450)	157 (108 to 221)	24 (12 to 46)	1 (1 to 1)	1423 (1116 to 1783)	929 (706 to 1218)	425 (293 to 598)	66 (32 to 124)	3 (2 to 3)
North Macedonia	2025	537 (420 to 687)	352 (268 to 462)	159 (110 to 224)	25 (12 to 47)	1 (1 to 1)	1452 (1135 to 1856)	952 (723 to 1250)	429 (297 to 605)	68 (32 to 127)	3 (2 to 3)

North Macedonia	2026	549 (431 to 697)	362 (275 to 476)	161 (111 to 227)	26 (12 to 48)	1 (1 to 1)	1485 (1164 to 1883)	979 (743 to 1287)	434 (300 to 613)	69 (33 to 130)	3 (2 to 3)
North Macedonia	2027	562 (443 to 709)	372 (281 to 488)	162 (112 to 229)	26 (13 to 49)	1 (1 to 1)	1518 (1198 to 1916)	1006 (760 to 1320)	439 (303 to 620)	71 (34 to 133)	3 (2 to 3)
North Macedonia	2028	574 (446 to 724)	382 (288 to 500)	164 (113 to 232)	27 (13 to 50)	1 (1 to 1)	1552 (1205 to 1957)	1033 (778 to 1352)	444 (306 to 628)	72 (35 to 136)	3 (2 to 3)
North Macedonia	2029	587 (458 to 744)	393 (296 to 513)	166 (115 to 235)	27 (13 to 51)	1 (1 to 1)	1587 (1238 to 2010)	1061 (800 to 1388)	449 (310 to 636)	74 (35 to 139)	3 (3 to 3)
North Macedonia	2030	600 (466 to 759)	403 (304 to 527)	168 (116 to 238)	28 (13 to 53)	1 (1 to 1)	1623 (1259 to 2051)	1090 (821 to 1426)	454 (313 to 644)	75 (36 to 142)	3 (3 to 4)
North Macedonia	2031	614 (485 to 776)	414 (311 to 541)	170 (117 to 241)	28 (14 to 54)	1 (1 to 1)	1659 (1311 to 2098)	1119 (842 to 1462)	459 (317 to 653)	77 (37 to 145)	3 (3 to 4)
North Macedonia	2032	628 (499 to 797)	425 (319 to 556)	172 (119 to 245)	29 (14 to 55)	1 (1 to 1)	1697 (1350 to 2153)	1150 (862 to 1502)	465 (320 to 662)	79 (38 to 149)	3 (3 to 4)
North Macedonia	2033	642 (505 to 809)	437 (327 to 570)	174 (120 to 248)	30 (14 to 56)	1 (1 to 1)	1735 (1366 to 2187)	1180 (883 to 1540)	471 (324 to 671)	81 (39 to 152)	3 (3 to 4)
North Macedonia	2034	657 (514 to 834)	448 (336 to 587)	177 (121 to 252)	30 (15 to 58)	1 (1 to 1)	1775 (1389 to 2254)	1212 (908 to 1588)	477 (328 to 681)	82 (40 to 156)	3 (3 to 4)
North Macedonia	2035	672 (525 to 847)	460 (345 to 603)	179 (123 to 255)	31 (15 to 59)	1 (1 to 2)	1816 (1419 to 2288)	1244 (932 to 1630)	484 (332 to 690)	84 (41 to 159)	3 (3 to 4)

North Macedonia	2036	687 (546 to 875)	473 (355 to 617)	181 (125 to 259)	32 (15 to 60)	1 (1 to 2)	1858 (1475 to 2364)	1278 (960 to 1668)	491 (337 to 700)	86 (42 to 163)	3 (3 to 4)
North Macedonia	2037	703 (551 to 888)	485 (364 to 635)	184 (126 to 262)	33 (16 to 62)	1 (1 to 2)	1901 (1489 to 2400)	1312 (985 to 1718)	497 (341 to 709)	88 (43 to 167)	4 (3 to 4)
North Macedonia	2038	720 (566 to 912)	498 (373 to 651)	187 (128 to 266)	33 (16 to 63)	1 (1 to 2)	1945 (1531 to 2464)	1347 (1008 to 1761)	504 (346 to 718)	90 (44 to 170)	4 (3 to 5)
North Macedonia	2039	736 (581 to 921)	511 (384 to 667)	189 (130 to 269)	34 (17 to 64)	1 (1 to 2)	1990 (1571 to 2489)	1383 (1037 to 1804)	512 (350 to 728)	92 (45 to 174)	4 (3 to 5)
North Macedonia	2040	753 (596 to 947)	525 (393 to 682)	192 (131 to 273)	35 (17 to 66)	1 (1 to 2)	2035 (1611 to 2560)	1418 (1062 to 1844)	519 (355 to 739)	95 (46 to 177)	4 (3 to 5)
North Macedonia	2041	770 (604 to 974)	538 (401 to 701)	195 (133 to 278)	36 (17 to 67)	1 (1 to 2)	2081 (1633 to 2633)	1455 (1085 to 1895)	526 (360 to 750)	97 (47 to 181)	4 (3 to 5)
North Macedonia	2042	787 (612 to 995)	552 (412 to 718)	197 (135 to 282)	37 (18 to 68)	1 (1 to 2)	2127 (1656 to 2688)	1491 (1114 to 1941)	533 (364 to 761)	99 (48 to 184)	4 (3 to 5)
North Macedonia	2043	804 (627 to 1015)	565 (420 to 736)	200 (137 to 286)	37 (18 to 69)	2 (1 to 2)	2173 (1696 to 2743)	1528 (1136 to 1988)	540 (370 to 772)	101 (49 to 188)	4 (3 to 5)
North Macedonia	2044	820 (635 to 1048)	579 (430 to 751)	202 (138 to 290)	38 (18 to 70)	2 (1 to 2)	2218 (1716 to 2832)	1564 (1162 to 2030)	546 (373 to 783)	103 (50 to 191)	4 (3 to 5)
North Macedonia	2045	837 (648 to 1067)	592 (442 to 768)	204 (140 to 294)	39 (19 to 72)	2 (1 to 2)	2263 (1751 to 2885)	1601 (1195 to 2077)	553 (378 to 793)	105 (51 to 194)	4 (3 to 6)

North Macedonia	2046	854 (667 to 1082)	606 (450 to 790)	207 (141 to 297)	40 (19 to 73)	2 (1 to 2)	2308 (1802 to 2924)	1637 (1215 to 2134)	559 (382 to 804)	107 (52 to 198)	4 (3 to 6)
North Macedonia	2047	870 (673 to 1114)	619 (459 to 807)	209 (143 to 300)	40 (20 to 75)	2 (1 to 2)	2351 (1820 to 3011)	1672 (1242 to 2181)	565 (386 to 811)	109 (53 to 202)	5 (4 to 6)
North Macedonia	2048	885 (689 to 1116)	632 (469 to 823)	211 (144 to 303)	41 (20 to 76)	2 (1 to 2)	2394 (1862 to 3016)	1707 (1269 to 2226)	570 (389 to 820)	111 (54 to 205)	5 (4 to 6)
North Macedonia	2049	901 (699 to 1146)	645 (479 to 837)	213 (145 to 307)	42 (20 to 77)	2 (1 to 2)	2436 (1890 to 3097)	1742 (1294 to 2264)	576 (393 to 829)	113 (55 to 209)	5 (4 to 6)
North Macedonia	2050	917 (721 to 1169)	657 (488 to 853)	215 (147 to 310)	43 (20 to 79)	2 (1 to 2)	2477 (1950 to 3160)	1777 (1319 to 2304)	581 (396 to 837)	115 (55 to 213)	5 (4 to 7)
Northern Mariana Islands	1995	517 (417 to 639)	356 (269 to 464)	146 (97 to 206)	16 (7 to 28)	0 (0 to 0)	517 (417 to 639)	356 (269 to 464)	146 (97 to 206)	16 (7 to 28)	0 (0 to 0)
Northern Mariana Islands	1996	516 (414 to 637)	355 (268 to 469)	145 (95 to 207)	16 (7 to 28)	0 (0 to 0)	516 (414 to 637)	355 (268 to 469)	145 (95 to 207)	16 (7 to 28)	0 (0 to 0)
Northern Mariana Islands	1997	508 (411 to 634)	351 (267 to 468)	143 (96 to 205)	15 (7 to 26)	0 (0 to 0)	508 (411 to 634)	351 (267 to 468)	143 (96 to 205)	15 (7 to 26)	0 (0 to 0)
Northern Mariana Islands	1998	489 (393 to 615)	338 (255 to 454)	138 (94 to 197)	13 (6 to 24)	0 (0 to 0)	489 (393 to 615)	338 (255 to 454)	138 (94 to 197)	13 (6 to 24)	0 (0 to 0)
Northern Mariana Islands	1999	469 (372 to 590)	323 (244 to 440)	134 (92 to 194)	12 (5 to 21)	0 (0 to 0)	469 (372 to 590)	323 (244 to 440)	134 (92 to 194)	12 (5 to 21)	0 (0 to 0)

Northern Mariana Islands	2000	455 (362 to 578)	314 (234 to 426)	131 (91 to 190)	10 (5 to 18)	0 (0 to 0)	455 (362 to 578)	314 (234 to 426)	131 (91 to 190)	10 (5 to 18)	0 (0 to 0)
Northern Mariana Islands	2001	451 (362 to 574)	310 (233 to 420)	132 (92 to 189)	9 (4 to 16)	0 (0 to 0)	451 (362 to 574)	310 (233 to 420)	132 (92 to 189)	9 (4 to 16)	0 (0 to 0)
Northern Mariana Islands	2002	464 (373 to 588)	318 (239 to 433)	136 (93 to 198)	9 (4 to 17)	0 (0 to 0)	464 (373 to 588)	318 (239 to 433)	136 (93 to 198)	9 (4 to 17)	0 (0 to 0)
Northern Mariana Islands	2003	491 (393 to 617)	336 (253 to 442)	144 (100 to 208)	11 (5 to 20)	0 (0 to 0)	491 (393 to 617)	336 (253 to 442)	144 (100 to 208)	11 (5 to 20)	0 (0 to 0)
Northern Mariana Islands	2004	514 (410 to 643)	350 (262 to 463)	151 (106 to 217)	12 (6 to 23)	0 (0 to 0)	514 (410 to 643)	350 (262 to 463)	151 (106 to 217)	12 (6 to 23)	0 (0 to 0)
Northern Mariana Islands	2005	507 (402 to 633)	341 (251 to 444)	153 (106 to 219)	13 (6 to 25)	0 (0 to 0)	507 (402 to 633)	341 (251 to 444)	153 (106 to 219)	13 (6 to 25)	0 (0 to 0)
Northern Mariana Islands	2006	512 (407 to 636)	341 (252 to 443)	155 (107 to 223)	16 (7 to 28)	0 (0 to 0)	512 (407 to 636)	341 (252 to 443)	155 (107 to 223)	16 (7 to 28)	0 (0 to 0)
Northern Mariana Islands	2007	507 (404 to 620)	334 (249 to 436)	155 (108 to 224)	18 (9 to 33)	0 (0 to 0)	507 (404 to 620)	334 (249 to 436)	155 (108 to 224)	18 (9 to 33)	0 (0 to 0)
Northern Mariana Islands	2008	479 (386 to 588)	310 (230 to 402)	149 (104 to 213)	21 (10 to 38)	0 (0 to 0)	479 (386 to 588)	310 (230 to 402)	149 (104 to 213)	21 (10 to 38)	0 (0 to 0)
Northern Mariana Islands	2009	426 (345 to 528)	267 (199 to 347)	138 (96 to 197)	21 (10 to 39)	0 (0 to 0)	426 (345 to 528)	267 (199 to 347)	138 (96 to 197)	21 (10 to 39)	0 (0 to 0)

Northern Mariana Islands	2010	425 (343 to 526)	264 (198 to 344)	136 (95 to 193)	24 (12 to 45)	0 (0 to 0)	425 (343 to 526)	264 (198 to 344)	136 (95 to 193)	24 (12 to 45)	0 (0 to 0)
Northern Mariana Islands	2011	402 (325 to 495)	247 (184 to 322)	130 (90 to 185)	25 (12 to 47)	0 (0 to 0)	402 (325 to 495)	247 (184 to 322)	130 (90 to 185)	25 (12 to 47)	0 (0 to 0)
Northern Mariana Islands	2012	401 (326 to 493)	245 (184 to 319)	129 (88 to 181)	27 (13 to 51)	0 (0 to 0)	401 (326 to 493)	245 (184 to 319)	129 (88 to 181)	27 (13 to 51)	0 (0 to 0)
Northern Mariana Islands	2013	410 (332 to 503)	250 (189 to 328)	130 (90 to 184)	30 (14 to 56)	0 (0 to 0)	410 (332 to 503)	250 (189 to 328)	130 (90 to 184)	30 (14 to 56)	0 (0 to 0)
Northern Mariana Islands	2014	435 (352 to 530)	266 (203 to 349)	135 (93 to 190)	34 (16 to 62)	0 (0 to 0)	435 (352 to 530)	266 (203 to 349)	135 (93 to 190)	34 (16 to 62)	0 (0 to 0)
Northern Mariana Islands	2015	481 (390 to 590)	296 (227 to 391)	145 (100 to 202)	40 (19 to 73)	0 (0 to 0)	481 (390 to 590)	296 (227 to 391)	145 (100 to 202)	40 (19 to 73)	0 (0 to 0)
Northern Mariana Islands	2016	603 (489 to 742)	380 (288 to 501)	170 (117 to 238)	53 (26 to 100)	0 (0 to 0)	603 (489 to 742)	380 (288 to 501)	170 (117 to 238)	53 (26 to 100)	0 (0 to 0)
Northern Mariana Islands	2017	752 (585 to 992)	483 (366 to 638)	199 (135 to 279)	70 (34 to 131)	0 (0 to 0)	752 (585 to 992)	483 (366 to 638)	199 (135 to 279)	70 (34 to 131)	0 (0 to 0)
Northern Mariana Islands	2018	768 (589 to 1018)	499 (378 to 669)	197 (134 to 278)	72 (34 to 134)	0 (0 to 0)	768 (589 to 1018)	499 (378 to 669)	197 (134 to 278)	72 (34 to 134)	0 (0 to 0)
Northern Mariana Islands	2019	776 (593 to 1024)	507 (382 to 682)	197 (134 to 277)	72 (34 to 135)	0 (0 to 0)	776 (593 to 1024)	507 (382 to 682)	197 (134 to 277)	72 (34 to 135)	0 (0 to 0)

Northern Mariana Islands	2020	790 (603 to 1058)	520 (389 to 702)	198 (134 to 279)	73 (35 to 137)	0 (0 to 0)	790 (603 to 1058)	520 (389 to 702)	198 (134 to 279)	73 (35 to 137)	0 (0 to 0)
Northern Mariana Islands	2021	808 (617 to 1076)	532 (398 to 716)	201 (137 to 284)	75 (36 to 140)	0 (0 to 0)	808 (617 to 1076)	532 (398 to 716)	201 (137 to 284)	75 (36 to 140)	0 (0 to 0)
Northern Mariana Islands	2022	820 (627 to 1088)	541 (406 to 731)	203 (139 to 287)	76 (36 to 141)	0 (0 to 0)	820 (627 to 1088)	541 (406 to 731)	203 (139 to 287)	76 (36 to 141)	0 (0 to 0)
Northern Mariana Islands	2023	827 (630 to 1106)	546 (409 to 737)	204 (139 to 285)	76 (37 to 142)	0 (0 to 0)	827 (630 to 1106)	546 (409 to 737)	204 (139 to 285)	76 (37 to 142)	0 (0 to 0)
Northern Mariana Islands	2024	829 (631 to 1105)	549 (409 to 740)	204 (139 to 286)	76 (37 to 141)	0 (0 to 0)	829 (631 to 1105)	549 (409 to 740)	204 (139 to 286)	76 (37 to 141)	0 (0 to 0)
Northern Mariana Islands	2025	828 (634 to 1100)	549 (408 to 740)	204 (139 to 285)	76 (37 to 141)	0 (0 to 0)	828 (634 to 1100)	549 (408 to 740)	204 (139 to 285)	76 (37 to 141)	0 (0 to 0)
Northern Mariana Islands	2026	825 (629 to 1098)	548 (408 to 739)	202 (137 to 281)	75 (36 to 140)	0 (0 to 0)	825 (629 to 1098)	548 (408 to 739)	202 (137 to 281)	75 (36 to 140)	0 (0 to 0)
Northern Mariana Islands	2027	823 (627 to 1098)	549 (408 to 742)	200 (135 to 279)	75 (36 to 140)	0 (0 to 0)	823 (627 to 1098)	549 (408 to 742)	200 (135 to 279)	75 (36 to 140)	0 (0 to 0)
Northern Mariana Islands	2028	820 (625 to 1090)	548 (408 to 739)	197 (134 to 276)	75 (36 to 140)	0 (0 to 0)	820 (625 to 1090)	548 (408 to 739)	197 (134 to 276)	75 (36 to 140)	0 (0 to 0)
Northern Mariana Islands	2029	814 (621 to 1077)	545 (405 to 731)	194 (131 to 273)	74 (36 to 139)	0 (0 to 0)	814 (621 to 1077)	545 (405 to 731)	194 (131 to 273)	74 (36 to 139)	0 (0 to 0)

Northern Mariana Islands	2030	806 (614 to 1071)	542 (402 to 727)	191 (130 to 268)	73 (35 to 138)	0 (0 to 0)	806 (614 to 1071)	542 (402 to 727)	191 (130 to 268)	73 (35 to 138)	0 (0 to 0)
Northern Mariana Islands	2031	799 (613 to 1066)	539 (399 to 724)	188 (128 to 263)	73 (35 to 138)	0 (0 to 0)	799 (613 to 1066)	539 (399 to 724)	188 (128 to 263)	73 (35 to 138)	0 (0 to 0)
Northern Mariana Islands	2032	793 (606 to 1065)	536 (396 to 722)	185 (126 to 258)	72 (35 to 137)	0 (0 to 0)	793 (606 to 1065)	536 (396 to 722)	185 (126 to 258)	72 (35 to 137)	0 (0 to 0)
Northern Mariana Islands	2033	788 (601 to 1049)	534 (394 to 721)	183 (124 to 255)	71 (35 to 136)	0 (0 to 0)	788 (601 to 1049)	534 (394 to 721)	183 (124 to 255)	71 (35 to 136)	0 (0 to 0)
Northern Mariana Islands	2034	782 (593 to 1053)	531 (392 to 720)	180 (122 to 251)	71 (34 to 134)	0 (0 to 0)	782 (593 to 1053)	531 (392 to 720)	180 (122 to 251)	71 (34 to 134)	0 (0 to 0)
Northern Mariana Islands	2035	777 (589 to 1035)	529 (389 to 717)	177 (120 to 248)	70 (34 to 133)	0 (0 to 0)	777 (589 to 1035)	529 (389 to 717)	177 (120 to 248)	70 (34 to 133)	0 (0 to 0)
Northern Mariana Islands	2036	773 (589 to 1028)	528 (386 to 717)	175 (118 to 245)	70 (34 to 132)	0 (0 to 0)	773 (589 to 1028)	528 (386 to 717)	175 (118 to 245)	70 (34 to 132)	0 (0 to 0)
Northern Mariana Islands	2037	772 (584 to 1035)	529 (387 to 721)	174 (117 to 243)	70 (34 to 132)	0 (0 to 0)	772 (584 to 1035)	529 (387 to 721)	174 (117 to 243)	70 (34 to 132)	0 (0 to 0)
Northern Mariana Islands	2038	773 (585 to 1035)	530 (388 to 726)	173 (116 to 241)	70 (34 to 132)	0 (0 to 0)	773 (585 to 1035)	530 (388 to 726)	173 (116 to 241)	70 (34 to 132)	0 (0 to 0)
Northern Mariana Islands	2039	771 (583 to 1034)	530 (387 to 727)	172 (115 to 239)	70 (34 to 131)	0 (0 to 0)	771 (583 to 1034)	530 (387 to 727)	172 (115 to 239)	70 (34 to 131)	0 (0 to 0)

Northern Mariana Islands	2040	771 (584 to 1032)	531 (387 to 727)	171 (114 to 237)	70 (34 to 131)	0 (0 to 0)	771 (584 to 1032)	531 (387 to 727)	171 (114 to 237)	70 (34 to 131)	0 (0 to 0)
Northern Mariana Islands	2041	775 (587 to 1037)	535 (390 to 733)	170 (114 to 237)	70 (34 to 131)	0 (0 to 0)	775 (587 to 1037)	535 (390 to 733)	170 (114 to 237)	70 (34 to 131)	0 (0 to 0)
Northern Mariana Islands	2042	784 (591 to 1055)	542 (395 to 745)	171 (115 to 238)	71 (35 to 133)	0 (0 to 0)	784 (591 to 1055)	542 (395 to 745)	171 (115 to 238)	71 (35 to 133)	0 (0 to 0)
Northern Mariana Islands	2043	799 (605 to 1080)	553 (403 to 762)	174 (116 to 241)	72 (35 to 135)	0 (0 to 0)	799 (605 to 1080)	553 (403 to 762)	174 (116 to 241)	72 (35 to 135)	0 (0 to 0)
Northern Mariana Islands	2044	815 (612 to 1103)	566 (412 to 779)	176 (118 to 244)	74 (36 to 138)	0 (0 to 0)	815 (612 to 1103)	566 (412 to 779)	176 (118 to 244)	74 (36 to 138)	0 (0 to 0)
Northern Mariana Islands	2045	833 (629 to 1126)	579 (422 to 797)	179 (120 to 248)	75 (37 to 140)	0 (0 to 0)	833 (629 to 1126)	579 (422 to 797)	179 (120 to 248)	75 (37 to 140)	0 (0 to 0)
Northern Mariana Islands	2046	852 (642 to 1151)	593 (432 to 816)	182 (122 to 252)	77 (37 to 143)	0 (0 to 0)	852 (642 to 1151)	593 (432 to 816)	182 (122 to 252)	77 (37 to 143)	0 (0 to 0)
Northern Mariana Islands	2047	871 (657 to 1169)	607 (443 to 836)	185 (124 to 257)	79 (38 to 147)	0 (0 to 0)	871 (657 to 1169)	607 (443 to 836)	185 (124 to 257)	79 (38 to 147)	0 (0 to 0)
Northern Mariana Islands	2048	890 (664 to 1201)	621 (453 to 855)	188 (126 to 261)	80 (39 to 150)	0 (0 to 0)	890 (664 to 1201)	621 (453 to 855)	188 (126 to 261)	80 (39 to 150)	0 (0 to 0)
Northern Mariana Islands	2049	910 (682 to 1225)	636 (464 to 875)	192 (128 to 266)	82 (40 to 154)	0 (0 to 0)	910 (682 to 1225)	636 (464 to 875)	192 (128 to 266)	82 (40 to 154)	0 (0 to 0)

Northern Mariana Islands	2050	929 (698 to 1264)	650 (474 to 895)	195 (129 to 271)	84 (41 to 157)	0 (0 to 0)	929 (698 to 1264)	650 (474 to 895)	195 (129 to 271)	84 (41 to 157)	0 (0 to 0)
Norway	1995	4163 (3947 to 4390)	3432 (3227 to 3648)	718 (661 to 773)	12 (6 to 22)	0 (0 to 0)	4089 (3878 to 4312)	3372 (3170 to 3584)	706 (650 to 759)	12 (6 to 22)	0 (0 to 0)
Norway	1996	4462 (4276 to 4632)	3658 (3481 to 3820)	790 (747 to 837)	13 (7 to 24)	0 (0 to 0)	4383 (4200 to 4550)	3593 (3420 to 3752)	776 (733 to 822)	13 (7 to 23)	0 (0 to 0)
Norway	1997	4902 (4745 to 5064)	3984 (3825 to 4141)	903 (861 to 950)	15 (8 to 26)	0 (0 to 0)	4815 (4661 to 4974)	3914 (3758 to 4068)	887 (846 to 933)	14 (8 to 26)	0 (0 to 0)
Norway	1998	5349 (5187 to 5512)	4353 (4192 to 4507)	980 (938 to 1024)	16 (9 to 28)	0 (0 to 0)	5255 (5095 to 5415)	4276 (4118 to 4428)	963 (921 to 1006)	16 (9 to 27)	0 (0 to 0)
Norway	1999	5532 (5376 to 5690)	4520 (4365 to 4667)	996 (953 to 1042)	17 (9 to 28)	0 (0 to 0)	5435 (5281 to 5589)	4440 (4288 to 4585)	978 (937 to 1023)	16 (9 to 28)	0 (0 to 0)
Norway	2000	5554 (5393 to 5719)	4556 (4404 to 4713)	981 (937 to 1026)	17 (10 to 28)	0 (0 to 0)	5456 (5297 to 5618)	4476 (4326 to 4630)	964 (921 to 1008)	17 (9 to 27)	0 (0 to 0)
Norway	2001	5801 (5639 to 5967)	4790 (4632 to 4949)	996 (954 to 1040)	16 (10 to 26)	0 (0 to 0)	5699 (5540 to 5861)	4705 (4550 to 4862)	978 (937 to 1022)	16 (9 to 25)	0 (0 to 0)
Norway	2002	6192 (6019 to 6368)	5133 (4970 to 5307)	1043 (1002 to 1085)	17 (10 to 28)	0 (0 to 0)	6083 (5912 to 6256)	5042 (4882 to 5213)	1024 (984 to 1065)	16 (10 to 27)	0 (0 to 0)
Norway	2003	6409 (6253 to 6594)	5324 (5177 to 5501)	1069 (1030 to 1110)	16 (10 to 26)	0 (0 to 0)	6296 (6142 to 6477)	5230 (5085 to 5404)	1050 (1011 to 1090)	16 (9 to 26)	0 (0 to 0)

Norway	2004	6319 (6167 to 6484)	5246 (5095 to 5405)	1058 (1017 to 1101)	15 (9 to 25)	0 (0 to 0)	6208 (6058 to 6370)	5153 (5005 to 5310)	1039 (999 to 1081)	15 (9 to 24)	0 (0 to 0)
Norway	2005	6200 (6040 to 6359)	5154 (5003 to 5313)	1031 (991 to 1072)	15 (9 to 24)	0 (0 to 0)	6090 (5933 to 6247)	5063 (4915 to 5219)	1013 (974 to 1053)	15 (9 to 23)	0 (0 to 0)
Norway	2006	6104 (5929 to 6273)	5090 (4929 to 5255)	1000 (960 to 1042)	14 (9 to 23)	0 (0 to 0)	5996 (5824 to 6162)	5000 (4842 to 5162)	982 (943 to 1024)	14 (9 to 23)	0 (0 to 0)
Norway	2007	6176 (6007 to 6360)	5173 (5011 to 5347)	989 (949 to 1035)	14 (9 to 23)	0 (0 to 0)	6067 (5901 to 6248)	5081 (4922 to 5253)	972 (932 to 1017)	14 (9 to 22)	0 (0 to 0)
Norway	2008	6245 (6075 to 6435)	5249 (5086 to 5429)	982 (940 to 1025)	14 (9 to 23)	0 (0 to 0)	6135 (5967 to 6321)	5157 (4996 to 5333)	964 (923 to 1007)	14 (9 to 22)	0 (0 to 0)
Norway	2009	6546 (6367 to 6737)	5528 (5365 to 5708)	1002 (960 to 1045)	17 (10 to 27)	0 (0 to 0)	6431 (6255 to 6618)	5430 (5271 to 5607)	984 (943 to 1027)	17 (10 to 26)	0 (0 to 0)
Norway	2010	6511 (6336 to 6686)	5504 (5340 to 5677)	988 (946 to 1030)	18 (11 to 28)	0 (0 to 0)	6396 (6224 to 6568)	5407 (5246 to 5577)	970 (929 to 1012)	18 (11 to 28)	0 (0 to 0)
Norway	2011	6523 (6348 to 6693)	5522 (5353 to 5681)	979 (938 to 1021)	22 (14 to 33)	0 (0 to 0)	6408 (6236 to 6575)	5425 (5258 to 5581)	962 (921 to 1003)	21 (13 to 32)	0 (0 to 0)
Norway	2012	6569 (6405 to 6739)	5572 (5409 to 5741)	973 (935 to 1011)	24 (16 to 36)	0 (0 to 0)	6453 (6291 to 6620)	5473 (5313 to 5640)	956 (919 to 993)	24 (16 to 36)	0 (0 to 0)
Norway	2013	6749 (6569 to 6917)	5740 (5566 to 5902)	984 (943 to 1023)	25 (17 to 38)	0 (0 to 0)	6629 (6453 to 6795)	5638 (5468 to 5798)	967 (927 to 1005)	25 (17 to 37)	0 (0 to 0)

Norway	2014	7063 (6895 to 7246)	6020 (5855 to 6197)	1018 (977 to 1062)	26 (17 to 39)	0 (0 to 0)	6939 (6773 to 7118)	5914 (5751 to 6087)	1000 (960 to 1043)	25 (17 to 38)	0 (0 to 0)
Norway	2015	7588 (7413 to 7785)	6483 (6315 to 6682)	1078 (1037 to 1117)	27 (18 to 41)	0 (0 to 0)	7454 (7282 to 7648)	6369 (6204 to 6564)	1059 (1019 to 1097)	27 (18 to 41)	0 (0 to 0)
Norway	2016	7966 (7795 to 8162)	6810 (6645 to 6998)	1128 (1084 to 1173)	28 (18 to 43)	0 (0 to 0)	7825 (7657 to 8018)	6689 (6528 to 6874)	1108 (1065 to 1153)	28 (18 to 43)	0 (0 to 0)
Norway	2017	8102 (7841 to 8368)	6924 (6683 to 7174)	1149 (1090 to 1214)	29 (18 to 44)	0 (0 to 0)	7959 (7703 to 8220)	6801 (6565 to 7048)	1129 (1070 to 1193)	28 (18 to 44)	0 (0 to 0)
Norway	2018	8076 (7826 to 8353)	6891 (6656 to 7142)	1156 (1097 to 1221)	29 (18 to 44)	0 (0 to 0)	7933 (7687 to 8205)	6769 (6538 to 7016)	1136 (1077 to 1199)	28 (18 to 44)	0 (0 to 0)
Norway	2019	8266 (7989 to 8562)	7066 (6805 to 7330)	1171 (1111 to 1237)	30 (18 to 46)	0 (0 to 0)	8120 (7847 to 8411)	6941 (6685 to 7201)	1150 (1092 to 1215)	29 (18 to 45)	0 (0 to 0)
Norway	2020	8455 (8157 to 8766)	7234 (6966 to 7514)	1191 (1130 to 1258)	30 (19 to 47)	0 (0 to 0)	8306 (8013 to 8611)	7107 (6843 to 7381)	1170 (1110 to 1236)	30 (19 to 46)	0 (0 to 0)
Norway	2021	8579 (8279 to 8910)	7349 (7066 to 7655)	1199 (1137 to 1267)	31 (19 to 47)	0 (0 to 0)	8427 (8133 to 8753)	7219 (6941 to 7520)	1178 (1117 to 1245)	30 (19 to 47)	0 (0 to 0)
Norway	2022	8670 (8358 to 9011)	7435 (7138 to 7746)	1204 (1142 to 1272)	31 (19 to 48)	0 (0 to 0)	8517 (8210 to 8852)	7304 (7012 to 7609)	1183 (1122 to 1249)	30 (19 to 47)	0 (0 to 0)
Norway	2023	8758 (8442 to 9106)	7525 (7216 to 7844)	1202 (1139 to 1268)	31 (20 to 48)	0 (0 to 0)	8603 (8293 to 8945)	7392 (7089 to 7705)	1180 (1119 to 1246)	31 (19 to 48)	0 (0 to 0)

Norway	2024	8834 (8495 to 9195)	7605 (7289 to 7941)	1198 (1136 to 1265)	32 (20 to 49)	0 (0 to 0)	8678 (8345 to 9033)	7470 (7160 to 7801)	1177 (1116 to 1243)	31 (19 to 48)	0 (0 to 0)
Norway	2025	8905 (8561 to 9269)	7680 (7350 to 8028)	1193 (1132 to 1260)	32 (20 to 49)	0 (0 to 0)	8748 (8410 to 9106)	7545 (7220 to 7886)	1172 (1112 to 1237)	31 (20 to 48)	0 (0 to 0)
Norway	2026	8984 (8608 to 9368)	7763 (7402 to 8130)	1189 (1128 to 1255)	32 (20 to 50)	0 (0 to 0)	8826 (8456 to 9203)	7626 (7272 to 7987)	1168 (1108 to 1233)	31 (20 to 49)	0 (0 to 0)
Norway	2027	9066 (8667 to 9483)	7848 (7463 to 8241)	1186 (1123 to 1252)	32 (20 to 50)	0 (0 to 0)	8906 (8514 to 9315)	7710 (7331 to 8095)	1165 (1104 to 1230)	32 (20 to 49)	0 (0 to 0)
Norway	2028	9152 (8726 to 9588)	7936 (7512 to 8360)	1183 (1120 to 1250)	33 (20 to 50)	0 (0 to 0)	8990 (8572 to 9419)	7796 (7379 to 8213)	1162 (1100 to 1228)	32 (20 to 49)	0 (0 to 0)
Norway	2029	9234 (8785 to 9717)	8022 (7572 to 8481)	1180 (1116 to 1247)	33 (21 to 51)	0 (0 to 0)	9071 (8630 to 9545)	7880 (7439 to 8331)	1159 (1096 to 1225)	32 (20 to 50)	0 (0 to 0)
Norway	2030	9313 (8824 to 9819)	8103 (7632 to 8589)	1176 (1113 to 1244)	33 (21 to 51)	0 (0 to 0)	9148 (8668 to 9646)	7960 (7498 to 8438)	1156 (1093 to 1222)	32 (21 to 50)	0 (0 to 0)
Norway	2031	9389 (8890 to 9955)	8183 (7688 to 8709)	1173 (1109 to 1241)	33 (21 to 51)	0 (0 to 0)	9223 (8733 to 9780)	8038 (7552 to 8555)	1152 (1089 to 1219)	33 (21 to 50)	0 (0 to 0)
Norway	2032	9462 (8913 to 10037)	8259 (7723 to 8819)	1169 (1104 to 1237)	33 (21 to 52)	0 (0 to 0)	9295 (8756 to 9860)	8113 (7587 to 8664)	1149 (1085 to 1216)	33 (21 to 51)	0 (0 to 0)
Norway	2033	9526 (8953 to 10161)	8327 (7764 to 8927)	1165 (1099 to 1233)	34 (21 to 52)	0 (0 to 0)	9358 (8795 to 9981)	8180 (7627 to 8769)	1145 (1080 to 1211)	33 (21 to 51)	0 (0 to 0)

Norway	2034	9590 (8961 to 10237)	8395 (7804 to 9035)	1161 (1094 to 1229)	34 (21 to 52)	0 (0 to 0)	9421 (8803 to 10056)	8247 (7667 to 8876)	1141 (1075 to 1208)	33 (21 to 51)	0 (0 to 0)
Norway	2035	9652 (9003 to 10340)	8461 (7836 to 9132)	1157 (1089 to 1225)	34 (21 to 53)	0 (0 to 0)	9481 (8844 to 10157)	8311 (7698 to 8971)	1137 (1069 to 1203)	33 (21 to 52)	0 (0 to 0)
Norway	2036	9713 (9028 to 10416)	8525 (7868 to 9223)	1153 (1084 to 1221)	34 (22 to 53)	0 (0 to 0)	9541 (8868 to 10232)	8375 (7729 to 9060)	1133 (1065 to 1199)	34 (21 to 52)	0 (0 to 0)
Norway	2037	9777 (9072 to 10533)	8592 (7908 to 9316)	1150 (1079 to 1218)	34 (22 to 54)	0 (0 to 0)	9604 (8912 to 10347)	8441 (7768 to 9152)	1130 (1060 to 1197)	34 (21 to 53)	0 (0 to 0)
Norway	2038	9853 (9127 to 10619)	8670 (7955 to 9426)	1148 (1076 to 1217)	35 (22 to 54)	0 (0 to 0)	9679 (8966 to 10431)	8517 (7814 to 9259)	1128 (1057 to 1196)	34 (21 to 53)	0 (0 to 0)
Norway	2039	9926 (9150 to 10758)	8745 (8001 to 9531)	1146 (1072 to 1217)	35 (22 to 55)	0 (0 to 0)	9751 (8988 to 10568)	8590 (7860 to 9363)	1126 (1053 to 1196)	34 (21 to 54)	0 (0 to 0)
Norway	2040	10002 (9212 to 10831)	8822 (8053 to 9638)	1145 (1069 to 1217)	35 (22 to 55)	0 (0 to 0)	9826 (9050 to 10640)	8666 (7911 to 9467)	1125 (1051 to 1196)	35 (22 to 54)	0 (0 to 0)
Norway	2041	10083 (9271 to 10981)	8903 (8108 to 9746)	1144 (1067 to 1218)	36 (22 to 56)	0 (0 to 0)	9905 (9107 to 10787)	8746 (7965 to 9574)	1124 (1048 to 1197)	35 (22 to 55)	0 (0 to 0)
Norway	2042	10162 (9310 to 11046)	8983 (8164 to 9853)	1143 (1064 to 1218)	36 (22 to 56)	0 (0 to 0)	9982 (9146 to 10851)	8824 (8020 to 9679)	1123 (1046 to 1197)	35 (22 to 55)	0 (0 to 0)
Norway	2043	10248 (9387 to 11160)	9069 (8227 to 9964)	1144 (1064 to 1220)	36 (22 to 57)	0 (0 to 0)	10067 (9221 to 10963)	8908 (8082 to 9788)	1123 (1045 to 1198)	35 (22 to 56)	0 (0 to 0)

Norway	2044	10336 (9450 to 11282)	9155 (8293 to 10071)	1144 (1063 to 1223)	36 (22 to 57)	0 (0 to 0)	10153 (9283 to 11082)	8994 (8147 to 9893)	1124 (1044 to 1201)	36 (22 to 56)	0 (0 to 0)
Norway	2045	10425 (9532 to 11384)	9244 (8364 to 10184)	1145 (1062 to 1225)	37 (23 to 58)	0 (0 to 0)	10241 (9363 to 11183)	9080 (8216 to 10004)	1125 (1044 to 1204)	36 (22 to 57)	0 (0 to 0)
Norway	2046	10513 (9583 to 11492)	9330 (8434 to 10289)	1146 (1061 to 1228)	37 (23 to 58)	0 (0 to 0)	10327 (9413 to 11289)	9165 (8285 to 10107)	1126 (1042 to 1206)	36 (22 to 57)	0 (0 to 0)
Norway	2047	10596 (9684 to 11585)	9411 (8501 to 10386)	1147 (1060 to 1231)	37 (23 to 59)	0 (0 to 0)	10409 (9513 to 11380)	9245 (8351 to 10203)	1127 (1042 to 1209)	37 (23 to 58)	0 (0 to 0)
Norway	2048	10672 (9723 to 11636)	9487 (8564 to 10476)	1147 (1059 to 1232)	38 (23 to 59)	0 (0 to 0)	10484 (9551 to 11431)	9320 (8413 to 10291)	1127 (1040 to 1211)	37 (23 to 58)	0 (0 to 0)
Norway	2049	10744 (9802 to 11769)	9559 (8626 to 10561)	1147 (1058 to 1235)	38 (23 to 59)	0 (0 to 0)	10555 (9629 to 11561)	9390 (8474 to 10374)	1127 (1039 to 1213)	37 (23 to 58)	0 (0 to 0)
Norway	2050	10815 (9858 to 11835)	9629 (8686 to 10644)	1147 (1055 to 1237)	38 (24 to 60)	0 (0 to 0)	10624 (9684 to 11626)	9459 (8533 to 10456)	1127 (1036 to 1215)	38 (23 to 59)	0 (0 to 0)
Oman	1995	616 (555 to 681)	510 (452 to 574)	66 (50 to 86)	41 (24 to 62)	0 (0 to 0)	1580 (1424 to 1746)	1307 (1158 to 1471)	169 (129 to 219)	104 (62 to 160)	0 (0 to 0)
Oman	1996	595 (540 to 651)	490 (442 to 544)	65 (51 to 84)	39 (24 to 58)	0 (0 to 0)	1525 (1385 to 1669)	1256 (1133 to 1394)	168 (131 to 214)	100 (63 to 149)	0 (0 to 0)
Oman	1997	601 (550 to 653)	492 (447 to 543)	68 (54 to 86)	40 (26 to 60)	0 (0 to 0)	1539 (1410 to 1673)	1262 (1146 to 1393)	174 (138 to 221)	103 (66 to 153)	0 (0 to 0)

Oman	1998	632 (581 to 685)	516 (473 to 568)	73 (58 to 92)	43 (28 to 63)	0 (0 to 0)	1619 (1490 to 1757)	1323 (1212 to 1456)	187 (148 to 235)	110 (72 to 162)	0 (0 to 0)
Oman	1999	609 (561 to 658)	497 (455 to 541)	71 (57 to 89)	41 (27 to 61)	0 (0 to 0)	1561 (1439 to 1688)	1273 (1165 to 1387)	183 (146 to 228)	106 (69 to 157)	0 (0 to 0)
Oman	2000	590 (543 to 638)	482 (442 to 524)	68 (53 to 85)	40 (26 to 59)	0 (0 to 0)	1512 (1391 to 1635)	1236 (1134 to 1343)	174 (137 to 218)	102 (65 to 151)	0 (0 to 0)
Oman	2001	597 (547 to 647)	491 (450 to 534)	66 (52 to 82)	40 (26 to 60)	0 (0 to 0)	1531 (1403 to 1659)	1258 (1154 to 1368)	169 (134 to 211)	104 (67 to 153)	0 (0 to 0)
Oman	2002	575 (529 to 622)	474 (434 to 517)	62 (49 to 78)	39 (26 to 58)	0 (0 to 0)	1475 (1355 to 1593)	1214 (1112 to 1324)	160 (126 to 199)	101 (67 to 148)	0 (0 to 0)
Oman	2003	541 (496 to 587)	444 (405 to 486)	59 (47 to 74)	38 (25 to 55)	0 (0 to 0)	1387 (1272 to 1505)	1138 (1039 to 1246)	152 (121 to 189)	97 (64 to 142)	0 (0 to 0)
Oman	2004	538 (491 to 584)	440 (400 to 484)	59 (47 to 73)	39 (26 to 56)	0 (0 to 0)	1379 (1258 to 1497)	1128 (1025 to 1240)	151 (121 to 187)	99 (66 to 144)	0 (0 to 0)
Oman	2005	482 (439 to 525)	392 (354 to 433)	54 (44 to 67)	36 (24 to 51)	0 (0 to 0)	1236 (1125 to 1345)	1005 (906 to 1110)	139 (112 to 171)	91 (62 to 130)	0 (0 to 0)
Oman	2006	485 (442 to 529)	394 (355 to 434)	54 (44 to 67)	37 (25 to 53)	0 (0 to 0)	1244 (1132 to 1356)	1010 (910 to 1114)	139 (112 to 171)	95 (65 to 135)	0 (0 to 0)
Oman	2007	485 (445 to 526)	393 (356 to 432)	54 (44 to 66)	38 (26 to 52)	0 (0 to 0)	1244 (1140 to 1348)	1008 (912 to 1108)	140 (113 to 169)	96 (67 to 134)	0 (0 to 0)

Oman	2008	463 (427 to 501)	373 (340 to 408)	54 (44 to 65)	36 (25 to 49)	0 (0 to 0)	1187 (1094 to 1283)	956 (871 to 1045)	138 (112 to 167)	92 (65 to 125)	0 (0 to 0)
Oman	2009	536 (495 to 579)	437 (399 to 476)	59 (49 to 71)	41 (29 to 54)	0 (0 to 0)	1374 (1270 to 1483)	1120 (1022 to 1221)	151 (125 to 182)	104 (75 to 139)	0 (0 to 0)
Oman	2010	508 (469 to 546)	417 (380 to 454)	55 (45 to 66)	37 (26 to 49)	0 (0 to 0)	1303 (1201 to 1399)	1069 (975 to 1164)	140 (116 to 169)	94 (68 to 127)	0 (0 to 0)
Oman	2011	515 (475 to 556)	428 (391 to 467)	51 (42 to 61)	36 (26 to 48)	0 (0 to 0)	1320 (1217 to 1425)	1098 (1003 to 1197)	130 (107 to 157)	92 (66 to 124)	0 (0 to 0)
Oman	2012	535 (494 to 577)	453 (415 to 491)	47 (38 to 56)	35 (25 to 48)	0 (0 to 0)	1370 (1267 to 1480)	1162 (1064 to 1258)	120 (98 to 144)	89 (63 to 124)	0 (0 to 0)
Oman	2013	554 (511 to 595)	480 (440 to 520)	42 (34 to 52)	32 (23 to 45)	0 (0 to 0)	1419 (1310 to 1526)	1229 (1128 to 1333)	108 (88 to 132)	82 (58 to 116)	0 (0 to 0)
Oman	2014	612 (567 to 657)	539 (496 to 583)	41 (33 to 51)	32 (23 to 45)	0 (0 to 0)	1569 (1454 to 1683)	1381 (1271 to 1495)	105 (85 to 131)	82 (58 to 115)	0 (0 to 0)
Oman	2015	691 (639 to 743)	613 (567 to 659)	43 (35 to 54)	35 (25 to 49)	0 (0 to 0)	1772 (1639 to 1906)	1572 (1453 to 1690)	110 (89 to 138)	90 (64 to 127)	0 (0 to 0)
Oman	2016	723 (670 to 778)	642 (590 to 695)	44 (35 to 55)	37 (25 to 53)	0 (0 to 0)	1852 (1717 to 1995)	1646 (1513 to 1782)	113 (89 to 142)	94 (64 to 136)	0 (0 to 0)
Oman	2017	664 (624 to 711)	588 (534 to 644)	42 (32 to 54)	34 (22 to 51)	0 (0 to 0)	1702 (1600 to 1822)	1507 (1368 to 1650)	108 (83 to 138)	87 (57 to 130)	0 (0 to 0)

Oman	2018	659 (615 to 712)	583 (525 to 648)	42 (32 to 54)	34 (22 to 51)	0 (0 to 0)	1690 (1577 to 1824)	1495 (1346 to 1661)	107 (83 to 137)	87 (57 to 130)	0 (0 to 0)
Oman	2019	616 (571 to 672)	543 (483 to 610)	41 (31 to 52)	32 (21 to 48)	0 (0 to 0)	1580 (1464 to 1723)	1393 (1237 to 1563)	104 (80 to 133)	83 (54 to 123)	0 (0 to 0)
Oman	2020	621 (570 to 683)	547 (484 to 620)	41 (32 to 53)	33 (21 to 49)	0 (0 to 0)	1592 (1460 to 1751)	1403 (1240 to 1589)	106 (81 to 135)	84 (55 to 125)	0 (0 to 0)
Oman	2021	628 (570 to 695)	554 (483 to 630)	41 (32 to 53)	33 (21 to 49)	0 (0 to 0)	1609 (1461 to 1782)	1419 (1238 to 1615)	106 (82 to 135)	84 (55 to 125)	0 (0 to 0)
Oman	2022	625 (566 to 694)	550 (479 to 628)	41 (32 to 53)	33 (21 to 49)	0 (0 to 0)	1601 (1450 to 1778)	1411 (1227 to 1609)	106 (82 to 136)	84 (55 to 126)	0 (0 to 0)
Oman	2023	623 (560 to 696)	549 (472 to 632)	41 (32 to 53)	33 (22 to 49)	0 (0 to 0)	1597 (1435 to 1785)	1406 (1210 to 1619)	106 (82 to 136)	85 (55 to 126)	0 (0 to 0)
Oman	2024	620 (553 to 697)	546 (463 to 632)	42 (32 to 53)	33 (22 to 49)	0 (0 to 0)	1590 (1418 to 1787)	1399 (1188 to 1620)	107 (82 to 136)	85 (56 to 126)	0 (0 to 0)
Oman	2025	621 (551 to 703)	546 (463 to 639)	42 (32 to 54)	33 (22 to 49)	0 (0 to 0)	1592 (1412 to 1802)	1400 (1186 to 1639)	107 (83 to 138)	85 (56 to 127)	0 (0 to 0)
Oman	2026	621 (550 to 706)	546 (459 to 642)	42 (32 to 54)	33 (22 to 50)	0 (0 to 0)	1592 (1409 to 1808)	1399 (1178 to 1646)	108 (83 to 138)	85 (56 to 127)	0 (0 to 0)
Oman	2027	622 (547 to 711)	546 (457 to 645)	42 (33 to 54)	33 (22 to 50)	0 (0 to 0)	1593 (1402 to 1823)	1399 (1172 to 1654)	108 (83 to 139)	86 (56 to 127)	0 (0 to 0)

Oman	2028	622 (546 to 716)	546 (457 to 651)	42 (33 to 54)	34 (22 to 50)	0 (0 to 0)	1595 (1400 to 1835)	1401 (1171 to 1669)	109 (84 to 140)	86 (57 to 128)	0 (0 to 0)
Oman	2029	624 (545 to 725)	547 (456 to 658)	43 (33 to 55)	34 (22 to 50)	0 (0 to 0)	1599 (1396 to 1858)	1403 (1168 to 1688)	109 (84 to 140)	87 (57 to 129)	0 (0 to 0)
Oman	2030	626 (549 to 731)	549 (455 to 662)	43 (33 to 55)	34 (22 to 51)	0 (0 to 0)	1606 (1406 to 1875)	1408 (1166 to 1698)	110 (85 to 141)	87 (57 to 130)	0 (0 to 0)
Oman	2031	629 (548 to 735)	551 (456 to 667)	43 (33 to 55)	34 (23 to 51)	0 (0 to 0)	1612 (1406 to 1884)	1413 (1168 to 1709)	111 (85 to 142)	88 (58 to 131)	0 (0 to 0)
Oman	2032	631 (546 to 735)	553 (455 to 671)	44 (33 to 56)	35 (23 to 52)	0 (0 to 0)	1618 (1400 to 1884)	1417 (1167 to 1719)	112 (86 to 143)	89 (58 to 132)	0 (0 to 0)
Oman	2033	634 (547 to 744)	555 (454 to 674)	44 (34 to 56)	35 (23 to 52)	0 (0 to 0)	1624 (1403 to 1908)	1422 (1163 to 1729)	112 (86 to 145)	89 (59 to 133)	0 (0 to 0)
Oman	2034	636 (548 to 750)	557 (452 to 682)	44 (34 to 57)	35 (23 to 52)	0 (0 to 0)	1630 (1404 to 1923)	1427 (1157 to 1747)	113 (87 to 146)	90 (59 to 134)	0 (0 to 0)
Oman	2035	639 (543 to 754)	559 (449 to 685)	44 (34 to 58)	35 (23 to 53)	0 (0 to 0)	1638 (1393 to 1932)	1433 (1152 to 1757)	114 (88 to 147)	91 (60 to 135)	0 (0 to 0)
Oman	2036	641 (545 to 761)	561 (446 to 691)	45 (34 to 58)	36 (23 to 53)	0 (0 to 0)	1643 (1396 to 1951)	1437 (1144 to 1770)	115 (88 to 149)	91 (60 to 137)	0 (0 to 0)
Oman	2037	643 (543 to 766)	562 (448 to 697)	45 (34 to 58)	36 (23 to 54)	0 (0 to 0)	1647 (1391 to 1964)	1440 (1150 to 1786)	115 (88 to 150)	92 (60 to 138)	0 (0 to 0)

Oman	2038	643 (545 to 768)	562 (447 to 698)	45 (35 to 59)	36 (24 to 54)	0 (0 to 0)	1648 (1396 to 1970)	1440 (1145 to 1790)	116 (88 to 151)	92 (60 to 138)	0 (0 to 0)
Oman	2039	644 (543 to 768)	562 (444 to 701)	45 (35 to 59)	36 (24 to 54)	0 (0 to 0)	1650 (1391 to 1969)	1442 (1138 to 1796)	116 (89 to 151)	93 (61 to 138)	0 (0 to 0)
Oman	2040	643 (536 to 765)	561 (438 to 692)	45 (35 to 59)	36 (24 to 54)	0 (0 to 0)	1649 (1373 to 1960)	1439 (1122 to 1774)	116 (89 to 151)	93 (61 to 139)	0 (0 to 0)
Oman	2041	642 (534 to 772)	560 (435 to 703)	45 (34 to 59)	36 (24 to 54)	0 (0 to 0)	1645 (1368 to 1980)	1436 (1115 to 1803)	117 (88 to 151)	93 (61 to 139)	0 (0 to 0)
Oman	2042	640 (531 to 772)	558 (433 to 705)	45 (34 to 59)	36 (24 to 54)	0 (0 to 0)	1640 (1361 to 1979)	1430 (1110 to 1806)	117 (88 to 152)	93 (61 to 139)	0 (0 to 0)
Oman	2043	638 (527 to 774)	556 (429 to 706)	45 (34 to 60)	36 (24 to 54)	0 (0 to 0)	1634 (1350 to 1985)	1425 (1100 to 1811)	116 (88 to 153)	93 (61 to 139)	0 (0 to 0)
Oman	2044	634 (524 to 768)	552 (426 to 698)	45 (34 to 60)	36 (24 to 54)	0 (0 to 0)	1624 (1344 to 1968)	1415 (1091 to 1790)	116 (87 to 153)	93 (61 to 138)	0 (0 to 0)
Oman	2045	628 (516 to 765)	547 (421 to 695)	45 (34 to 60)	36 (24 to 54)	0 (0 to 0)	1611 (1323 to 1960)	1402 (1078 to 1782)	116 (87 to 153)	93 (60 to 137)	0 (0 to 0)
Oman	2046	624 (511 to 764)	543 (416 to 696)	45 (33 to 60)	36 (23 to 53)	0 (0 to 0)	1599 (1310 to 1957)	1391 (1065 to 1783)	115 (86 to 154)	92 (60 to 137)	0 (0 to 0)
Oman	2047	618 (503 to 754)	537 (407 to 686)	45 (33 to 60)	36 (23 to 53)	0 (0 to 0)	1584 (1288 to 1932)	1377 (1044 to 1758)	115 (85 to 153)	92 (60 to 136)	0 (0 to 0)

Oman	2048	612 (494 to 749)	532 (400 to 679)	45 (33 to 60)	36 (23 to 53)	0 (0 to 0)	1568 (1266 to 1921)	1362 (1027 to 1741)	114 (84 to 153)	91 (59 to 135)	0 (0 to 0)
Oman	2049	604 (488 to 740)	524 (395 to 673)	44 (32 to 59)	35 (23 to 52)	0 (0 to 0)	1548 (1252 to 1897)	1344 (1013 to 1725)	113 (83 to 152)	90 (59 to 133)	0 (0 to 0)
Oman	2050	596 (480 to 727)	517 (390 to 658)	44 (32 to 59)	35 (23 to 52)	0 (0 to 0)	1527 (1230 to 1863)	1325 (1000 to 1686)	112 (82 to 151)	89 (58 to 132)	0 (0 to 0)
Pakistan	1995	25 (20 to 32)	7 (5 to 10)	16 (11 to 22)	1 (0 to 1)	1 (1 to 1)	109 (87 to 137)	31 (24 to 41)	69 (48 to 96)	3 (2 to 6)	5 (5 to 5)
Pakistan	1996	26 (21 to 33)	8 (6 to 10)	16 (12 to 23)	1 (0 to 1)	2 (2 to 2)	114 (90 to 142)	33 (24 to 43)	71 (50 to 97)	3 (2 to 6)	7 (7 to 7)
Pakistan	1997	25 (20 to 32)	7 (6 to 10)	16 (11 to 23)	1 (0 to 1)	1 (1 to 1)	109 (87 to 137)	32 (24 to 41)	69 (49 to 97)	3 (2 to 6)	5 (5 to 5)
Pakistan	1998	25 (20 to 32)	8 (6 to 10)	16 (12 to 23)	1 (0 to 1)	1 (1 to 1)	109 (87 to 138)	33 (25 to 43)	70 (50 to 98)	3 (2 to 6)	3 (3 to 3)
Pakistan	1999	25 (20 to 31)	8 (6 to 10)	16 (12 to 23)	1 (0 to 1)	1 (1 to 1)	108 (86 to 135)	33 (25 to 43)	70 (50 to 97)	3 (2 to 6)	3 (3 to 3)
Pakistan	2000	25 (20 to 32)	8 (6 to 11)	16 (12 to 23)	1 (0 to 1)	0 (0 to 0)	109 (87 to 136)	35 (26 to 45)	69 (50 to 97)	3 (1 to 6)	2 (2 to 2)
Pakistan	2001	25 (20 to 31)	8 (6 to 10)	15 (11 to 21)	1 (0 to 1)	1 (1 to 1)	105 (85 to 132)	33 (25 to 42)	65 (47 to 91)	3 (1 to 5)	4 (4 to 4)
Pakistan	2002	26 (21 to 31)	8 (6 to 10)	16 (11 to 21)	1 (0 to 1)	1 (1 to 1)	110 (89 to 135)	34 (25 to 44)	67 (48 to 92)	3 (1 to 6)	6 (6 to 6)
Pakistan	2003	24 (19 to 29)	7 (5 to 9)	16 (11 to 21)	1 (0 to 1)	1 (1 to 1)	101 (81 to 125)	28 (21 to 37)	67 (48 to 90)	3 (1 to 5)	3 (3 to 3)
Pakistan	2004	25 (20 to 30)	6 (5 to 8)	16 (12 to 22)	1 (0 to 1)	1 (1 to 1)	106 (84 to 130)	26 (20 to 34)	70 (50 to 94)	3 (1 to 5)	6 (6 to 6)
Pakistan	2005	27 (21 to 33)	6 (5 to 8)	18 (13 to 25)	1 (0 to 1)	1 (1 to 1)	114 (91 to 142)	26 (20 to 34)	79 (57 to 107)	3 (2 to 6)	6 (6 to 6)

Pakistan	2006	29 (23 to 36)	6 (5 to 8)	20 (15 to 28)	1 (0 to 1)	2 (2 to 2)	125 (99 to 156)	26 (19 to 34)	88 (63 to 118)	3 (2 to 6)	7 (7 to 7)
Pakistan	2007	32 (25 to 40)	7 (5 to 9)	22 (16 to 30)	1 (0 to 2)	2 (2 to 2)	136 (107 to 170)	29 (21 to 37)	95 (67 to 128)	4 (2 to 7)	9 (9 to 9)
Pakistan	2008	32 (25 to 40)	7 (5 to 9)	22 (16 to 30)	1 (0 to 2)	2 (2 to 2)	138 (109 to 172)	29 (22 to 38)	95 (67 to 130)	4 (2 to 7)	11 (11 to 11)
Pakistan	2009	30 (24 to 38)	6 (5 to 8)	20 (14 to 28)	1 (0 to 1)	3 (3 to 3)	129 (102 to 164)	27 (20 to 35)	87 (62 to 119)	3 (2 to 6)	11 (11 to 11)
Pakistan	2010	30 (24 to 37)	7 (5 to 9)	20 (14 to 27)	1 (0 to 1)	3 (3 to 3)	128 (101 to 160)	29 (21 to 37)	85 (59 to 115)	3 (2 to 6)	11 (11 to 11)
Pakistan	2011	28 (22 to 35)	7 (5 to 9)	19 (13 to 26)	1 (0 to 1)	2 (2 to 2)	120 (94 to 151)	29 (22 to 38)	81 (56 to 111)	3 (2 to 6)	7 (7 to 7)
Pakistan	2012	30 (24 to 38)	8 (6 to 10)	19 (13 to 27)	1 (0 to 1)	2 (2 to 2)	129 (103 to 163)	33 (24 to 43)	83 (57 to 114)	3 (2 to 6)	11 (11 to 11)
Pakistan	2013	31 (25 to 40)	8 (6 to 10)	20 (14 to 27)	1 (0 to 1)	3 (3 to 3)	135 (108 to 171)	34 (26 to 45)	85 (59 to 118)	4 (2 to 6)	13 (13 to 13)
Pakistan	2014	31 (25 to 39)	8 (6 to 10)	20 (14 to 28)	1 (0 to 2)	2 (2 to 2)	133 (106 to 169)	35 (26 to 45)	86 (60 to 119)	4 (2 to 7)	9 (9 to 9)
Pakistan	2015	33 (26 to 42)	9 (6 to 11)	21 (14 to 29)	1 (1 to 2)	3 (3 to 3)	141 (113 to 179)	37 (28 to 48)	88 (62 to 124)	4 (2 to 8)	11 (11 to 11)
Pakistan	2016	35 (28 to 44)	9 (7 to 12)	21 (15 to 30)	1 (1 to 3)	3 (3 to 3)	151 (121 to 188)	40 (30 to 51)	91 (64 to 128)	6 (3 to 11)	13 (13 to 13)
Pakistan	2017	37 (29 to 46)	10 (8 to 14)	22 (15 to 31)	2 (1 to 3)	3 (3 to 3)	159 (126 to 199)	45 (34 to 58)	95 (67 to 132)	8 (4 to 14)	12 (12 to 12)
Pakistan	2018	38 (30 to 48)	11 (8 to 14)	23 (16 to 32)	2 (1 to 3)	3 (3 to 3)	165 (130 to 205)	48 (35 to 62)	98 (69 to 137)	8 (4 to 14)	11 (11 to 11)
Pakistan	2019	39 (31 to 49)	11 (8 to 15)	23 (16 to 32)	2 (1 to 3)	3 (3 to 3)	167 (132 to 209)	49 (36 to 64)	100 (70 to 138)	8 (4 to 14)	11 (11 to 11)
Pakistan	2020	39 (31 to 49)	11 (8 to 15)	23 (16 to 32)	2 (1 to 3)	3 (3 to 3)	169 (133 to 211)	49 (36 to 65)	100 (70 to 139)	8 (4 to 15)	11 (11 to 12)

Pakistan	2021	40 (32 to 50)	12 (9 to 16)	24 (17 to 33)	2 (1 to 4)	3 (3 to 3)	172 (135 to 217)	50 (37 to 67)	102 (71 to 142)	8 (4 to 15)	11 (11 to 12)
Pakistan	2022	41 (32 to 51)	12 (9 to 16)	24 (17 to 34)	2 (1 to 4)	3 (2 to 3)	176 (138 to 221)	52 (38 to 69)	104 (73 to 145)	9 (4 to 16)	12 (11 to 13)
Pakistan	2023	42 (33 to 53)	12 (9 to 17)	25 (17 to 34)	2 (1 to 4)	3 (2 to 3)	180 (141 to 227)	54 (39 to 72)	106 (75 to 148)	9 (4 to 16)	12 (10 to 13)
Pakistan	2024	43 (34 to 54)	13 (9 to 17)	25 (18 to 35)	2 (1 to 4)	3 (2 to 3)	184 (144 to 233)	55 (39 to 74)	108 (77 to 151)	9 (5 to 16)	12 (10 to 13)
Pakistan	2025	44 (34 to 56)	13 (9 to 18)	26 (18 to 36)	2 (1 to 4)	3 (2 to 3)	189 (148 to 239)	57 (41 to 76)	111 (78 to 154)	9 (5 to 17)	12 (10 to 13)
Pakistan	2026	45 (35 to 57)	14 (10 to 18)	26 (19 to 37)	2 (1 to 4)	3 (2 to 3)	193 (151 to 244)	58 (42 to 78)	113 (80 to 158)	10 (5 to 17)	12 (10 to 13)
Pakistan	2027	46 (36 to 58)	14 (10 to 19)	27 (19 to 37)	2 (1 to 4)	3 (2 to 3)	197 (154 to 250)	60 (42 to 80)	116 (82 to 161)	10 (5 to 18)	12 (10 to 14)
Pakistan	2028	47 (37 to 59)	14 (10 to 19)	27 (19 to 38)	2 (1 to 4)	3 (2 to 3)	202 (158 to 256)	62 (44 to 84)	118 (83 to 164)	10 (5 to 18)	12 (10 to 14)
Pakistan	2029	48 (38 to 61)	15 (10 to 20)	28 (20 to 39)	2 (1 to 4)	3 (2 to 3)	206 (162 to 261)	64 (45 to 87)	121 (85 to 168)	10 (5 to 19)	12 (10 to 14)
Pakistan	2030	49 (38 to 62)	15 (11 to 21)	29 (20 to 40)	2 (1 to 4)	3 (2 to 3)	211 (164 to 267)	65 (46 to 90)	123 (87 to 171)	11 (5 to 19)	12 (10 to 14)
Pakistan	2031	50 (39 to 63)	16 (11 to 22)	29 (21 to 41)	3 (1 to 5)	3 (2 to 3)	216 (167 to 272)	67 (47 to 93)	126 (89 to 175)	11 (5 to 20)	12 (10 to 14)
Pakistan	2032	51 (40 to 65)	16 (11 to 22)	30 (21 to 42)	3 (1 to 5)	3 (2 to 3)	221 (172 to 280)	69 (48 to 95)	129 (91 to 179)	11 (6 to 20)	12 (10 to 14)
Pakistan	2033	53 (41 to 67)	17 (11 to 23)	31 (22 to 42)	3 (1 to 5)	3 (2 to 3)	226 (176 to 287)	71 (49 to 98)	131 (93 to 183)	12 (6 to 21)	12 (10 to 15)
Pakistan	2034	54 (42 to 69)	17 (12 to 24)	31 (22 to 43)	3 (1 to 5)	3 (2 to 3)	231 (180 to 295)	73 (50 to 101)	134 (94 to 187)	12 (6 to 21)	12 (10 to 15)
Pakistan	2035	55 (43 to 70)	17 (12 to 24)	32 (22 to 44)	3 (1 to 5)	3 (2 to 4)	237 (183 to 300)	75 (51 to 105)	137 (96 to 191)	12 (6 to 22)	12 (10 to 15)

Pakistan	2036	56 (44 to 71)	18 (12 to 25)	33 (23 to 45)	3 (1 to 5)	3 (2 to 4)	242 (188 to 306)	77 (52 to 109)	140 (98 to 195)	12 (6 to 22)	12 (10 to 16)
Pakistan	2037	58 (45 to 74)	18 (12 to 26)	33 (23 to 46)	3 (1 to 5)	3 (2 to 4)	248 (192 to 316)	79 (54 to 112)	143 (101 to 199)	13 (6 to 23)	13 (10 to 15)
Pakistan	2038	59 (46 to 75)	19 (13 to 27)	34 (24 to 47)	3 (2 to 5)	3 (2 to 4)	254 (196 to 323)	81 (55 to 115)	146 (103 to 203)	13 (6 to 24)	13 (10 to 16)
Pakistan	2039	60 (46 to 77)	19 (13 to 27)	35 (24 to 48)	3 (2 to 6)	3 (2 to 4)	259 (200 to 331)	83 (57 to 118)	149 (105 to 208)	13 (7 to 24)	13 (10 to 16)
Pakistan	2040	62 (48 to 79)	20 (13 to 28)	35 (25 to 49)	3 (2 to 6)	3 (2 to 4)	264 (205 to 339)	85 (57 to 121)	152 (107 to 212)	14 (7 to 25)	13 (10 to 16)
Pakistan	2041	63 (48 to 80)	20 (14 to 29)	36 (25 to 50)	3 (2 to 6)	3 (2 to 4)	270 (208 to 346)	88 (59 to 124)	155 (109 to 216)	14 (7 to 25)	13 (10 to 17)
Pakistan	2042	64 (49 to 82)	21 (14 to 30)	37 (26 to 51)	3 (2 to 6)	3 (2 to 4)	276 (213 to 354)	90 (60 to 128)	158 (111 to 220)	14 (7 to 26)	13 (11 to 17)
Pakistan	2043	65 (51 to 84)	21 (14 to 31)	38 (26 to 52)	3 (2 to 6)	3 (2 to 4)	281 (218 to 360)	92 (62 to 131)	161 (113 to 224)	15 (7 to 26)	13 (11 to 17)
Pakistan	2044	67 (51 to 85)	22 (15 to 31)	38 (27 to 53)	3 (2 to 6)	3 (3 to 4)	287 (221 to 367)	94 (63 to 135)	164 (115 to 228)	15 (7 to 27)	14 (11 to 18)
Pakistan	2045	68 (52 to 87)	22 (15 to 32)	39 (27 to 54)	4 (2 to 6)	3 (3 to 4)	293 (225 to 374)	96 (64 to 139)	167 (117 to 232)	15 (8 to 28)	14 (11 to 18)
Pakistan	2046	69 (53 to 89)	23 (16 to 33)	40 (28 to 55)	4 (2 to 7)	3 (3 to 4)	299 (229 to 381)	99 (67 to 143)	171 (119 to 236)	16 (8 to 28)	14 (11 to 18)
Pakistan	2047	71 (54 to 91)	23 (16 to 34)	40 (28 to 56)	4 (2 to 7)	3 (3 to 4)	304 (233 to 392)	101 (67 to 147)	174 (121 to 240)	16 (8 to 29)	14 (11 to 19)
Pakistan	2048	72 (55 to 92)	24 (16 to 35)	41 (29 to 57)	4 (2 to 7)	3 (3 to 4)	310 (238 to 397)	103 (68 to 152)	177 (123 to 244)	16 (8 to 29)	14 (11 to 19)
Pakistan	2049	74 (56 to 94)	25 (16 to 36)	42 (29 to 58)	4 (2 to 7)	3 (3 to 4)	316 (241 to 405)	106 (70 to 155)	180 (125 to 248)	17 (8 to 30)	14 (11 to 19)
Pakistan	2050	75 (57 to 96)	25 (17 to 37)	43 (29 to 59)	4 (2 to 7)	3 (3 to 5)	322 (245 to 415)	108 (71 to 159)	183 (127 to 252)	17 (8 to 31)	15 (12 to 20)

Palestine	1995	232 (190 to 291)	77 (60 to 97)	94 (67 to 127)	56 (31 to 94)	6 (6 to 6)	89 (73 to 111)	29 (23 to 37)	36 (26 to 48)	21 (12 to 36)	2 (2 to 2)
Palestine	1996	236 (198 to 287)	75 (60 to 94)	92 (67 to 122)	55 (32 to 90)	13 (13 to 13)	90 (76 to 110)	29 (23 to 36)	35 (26 to 47)	21 (12 to 34)	5 (5 to 5)
Palestine	1997	250 (213 to 297)	79 (63 to 97)	94 (71 to 120)	59 (37 to 93)	19 (19 to 19)	96 (81 to 113)	30 (24 to 37)	36 (27 to 46)	23 (14 to 36)	7 (7 to 7)
Palestine	1998	260 (223 to 304)	84 (68 to 102)	97 (76 to 120)	65 (42 to 97)	14 (14 to 14)	99 (85 to 116)	32 (26 to 39)	37 (29 to 46)	25 (16 to 37)	5 (5 to 5)
Palestine	1999	271 (237 to 309)	88 (73 to 105)	100 (82 to 121)	70 (49 to 99)	13 (13 to 13)	103 (91 to 118)	34 (28 to 40)	38 (31 to 46)	27 (19 to 38)	5 (5 to 5)
Palestine	2000	263 (236 to 294)	84 (71 to 99)	97 (82 to 115)	67 (50 to 87)	15 (15 to 15)	101 (90 to 113)	32 (27 to 38)	37 (31 to 44)	26 (19 to 33)	6 (6 to 6)
Palestine	2001	247 (224 to 273)	81 (70 to 94)	93 (80 to 108)	64 (50 to 80)	10 (10 to 10)	95 (86 to 104)	31 (27 to 36)	36 (31 to 41)	24 (19 to 31)	4 (4 to 4)
Palestine	2002	239 (218 to 263)	79 (69 to 91)	89 (78 to 102)	60 (48 to 75)	11 (11 to 11)	91 (83 to 100)	30 (26 to 35)	34 (30 to 39)	23 (18 to 29)	4 (4 to 4)
Palestine	2003	269 (248 to 294)	89 (78 to 102)	94 (83 to 108)	63 (50 to 78)	22 (22 to 22)	103 (95 to 112)	34 (30 to 39)	36 (32 to 41)	24 (19 to 30)	8 (8 to 8)
Palestine	2004	308 (285 to 335)	108 (95 to 122)	107 (95 to 122)	71 (57 to 87)	21 (21 to 21)	118 (109 to 128)	41 (36 to 47)	41 (36 to 47)	27 (22 to 33)	8 (8 to 8)
Palestine	2005	333 (309 to 360)	120 (107 to 135)	117 (104 to 132)	77 (63 to 95)	18 (18 to 18)	127 (118 to 138)	46 (41 to 52)	45 (40 to 50)	30 (24 to 36)	7 (7 to 7)
Palestine	2006	338 (314 to 365)	121 (108 to 135)	125 (112 to 139)	76 (63 to 92)	16 (16 to 16)	129 (120 to 140)	46 (41 to 52)	48 (43 to 53)	29 (24 to 35)	6 (6 to 6)
Palestine	2007	364 (340 to 391)	127 (113 to 142)	139 (125 to 155)	77 (63 to 93)	21 (21 to 21)	139 (130 to 149)	49 (43 to 54)	53 (48 to 59)	29 (24 to 36)	8 (8 to 8)
Palestine	2008	389 (363 to 417)	135 (122 to 151)	151 (136 to 168)	83 (68 to 100)	20 (20 to 20)	149 (139 to 159)	52 (47 to 58)	58 (52 to 64)	32 (26 to 38)	8 (8 to 8)
Palestine	2009	403 (376 to 430)	144 (129 to 160)	162 (146 to 179)	84 (69 to 101)	13 (13 to 13)	154 (144 to 165)	55 (49 to 61)	62 (56 to 69)	32 (27 to 39)	5 (5 to 5)

Palestine	2010	401 (375 to 427)	142 (128 to 158)	162 (146 to 178)	81 (67 to 98)	16 (16 to 16)	153 (143 to 163)	54 (49 to 60)	62 (56 to 68)	31 (26 to 38)	6 (6 to 6)
Palestine	2011	407 (380 to 435)	146 (130 to 162)	162 (146 to 180)	81 (66 to 97)	18 (18 to 18)	155 (145 to 166)	56 (50 to 62)	62 (56 to 69)	31 (25 to 37)	7 (7 to 7)
Palestine	2012	393 (364 to 421)	146 (131 to 164)	155 (138 to 172)	78 (62 to 96)	14 (14 to 14)	150 (139 to 161)	56 (50 to 63)	59 (53 to 66)	30 (24 to 37)	5 (5 to 5)
Palestine	2013	368 (339 to 398)	143 (126 to 161)	144 (127 to 163)	72 (55 to 90)	10 (10 to 10)	141 (130 to 152)	54 (48 to 62)	55 (48 to 62)	27 (21 to 34)	4 (4 to 4)
Palestine	2014	344 (310 to 377)	131 (113 to 150)	135 (115 to 156)	68 (50 to 88)	11 (11 to 11)	131 (119 to 144)	50 (43 to 57)	51 (44 to 60)	26 (19 to 34)	4 (4 to 4)
Palestine	2015	341 (301 to 385)	132 (111 to 154)	135 (112 to 162)	69 (48 to 95)	6 (6 to 6)	130 (115 to 147)	50 (42 to 59)	51 (43 to 62)	26 (18 to 36)	2 (2 to 2)
Palestine	2016	349 (305 to 402)	135 (111 to 162)	136 (109 to 170)	71 (47 to 105)	6 (6 to 6)	133 (117 to 154)	52 (42 to 62)	52 (42 to 65)	27 (18 to 40)	2 (2 to 2)
Palestine	2017	365 (300 to 436)	137 (110 to 167)	137 (107 to 177)	71 (44 to 110)	19 (19 to 19)	139 (115 to 167)	52 (42 to 64)	53 (41 to 68)	27 (17 to 42)	7 (7 to 7)
Palestine	2018	359 (297 to 439)	135 (109 to 165)	135 (105 to 173)	71 (44 to 109)	18 (18 to 18)	137 (113 to 168)	52 (42 to 63)	52 (40 to 66)	27 (17 to 42)	7 (7 to 7)
Palestine	2019	368 (301 to 441)	140 (110 to 171)	137 (107 to 177)	73 (45 to 112)	18 (18 to 18)	141 (115 to 168)	53 (42 to 65)	53 (41 to 67)	28 (17 to 43)	7 (7 to 7)
Palestine	2020	380 (311 to 465)	145 (115 to 179)	141 (109 to 181)	75 (47 to 116)	18 (17 to 19)	145 (119 to 178)	56 (44 to 68)	54 (42 to 69)	29 (18 to 44)	7 (7 to 7)
Palestine	2021	389 (317 to 469)	150 (117 to 185)	143 (111 to 183)	78 (48 to 119)	19 (17 to 20)	149 (121 to 179)	57 (45 to 71)	55 (42 to 70)	30 (18 to 46)	7 (7 to 8)
Palestine	2022	399 (326 to 490)	156 (123 to 191)	144 (112 to 185)	80 (50 to 123)	19 (17 to 21)	152 (125 to 187)	60 (47 to 73)	55 (43 to 71)	31 (19 to 47)	7 (7 to 8)
Palestine	2023	409 (332 to 494)	162 (127 to 199)	146 (114 to 188)	82 (51 to 127)	19 (17 to 21)	156 (127 to 189)	62 (48 to 76)	56 (43 to 72)	31 (20 to 48)	7 (6 to 8)
Palestine	2024	419 (341 to 520)	168 (132 to 208)	148 (115 to 190)	85 (53 to 131)	19 (17 to 21)	160 (130 to 199)	64 (50 to 80)	57 (44 to 73)	32 (20 to 50)	7 (6 to 8)

Palestine	2025	431 (347 to 522)	174 (137 to 217)	150 (116 to 193)	88 (54 to 135)	19 (17 to 21)	165 (133 to 200)	67 (52 to 83)	58 (45 to 74)	33 (21 to 52)	7 (6 to 8)
Palestine	2026	442 (359 to 547)	181 (139 to 226)	152 (118 to 196)	90 (56 to 139)	19 (16 to 21)	169 (137 to 209)	69 (53 to 86)	58 (45 to 75)	34 (21 to 53)	7 (6 to 8)
Palestine	2027	454 (363 to 551)	188 (144 to 234)	154 (120 to 199)	93 (58 to 144)	19 (16 to 22)	173 (139 to 211)	72 (55 to 90)	59 (46 to 76)	36 (22 to 55)	7 (6 to 8)
Palestine	2028	466 (375 to 582)	195 (149 to 246)	156 (121 to 202)	96 (59 to 148)	19 (16 to 22)	178 (143 to 223)	75 (57 to 94)	60 (46 to 77)	37 (23 to 57)	7 (6 to 8)
Palestine	2029	479 (383 to 587)	203 (156 to 255)	158 (123 to 205)	99 (61 to 153)	19 (16 to 22)	183 (146 to 225)	78 (60 to 98)	61 (47 to 79)	38 (23 to 58)	7 (6 to 8)
Palestine	2030	492 (393 to 615)	211 (160 to 267)	160 (125 to 208)	102 (63 to 158)	19 (16 to 22)	188 (150 to 235)	80 (61 to 102)	61 (48 to 79)	39 (24 to 60)	7 (6 to 9)
Palestine	2031	505 (399 to 621)	219 (167 to 277)	162 (126 to 210)	105 (65 to 163)	19 (16 to 22)	193 (152 to 238)	84 (64 to 106)	62 (48 to 80)	40 (25 to 62)	7 (6 to 9)
Palestine	2032	518 (412 to 650)	226 (173 to 286)	164 (128 to 213)	108 (67 to 168)	19 (16 to 23)	198 (157 to 249)	87 (66 to 109)	63 (49 to 82)	41 (26 to 64)	7 (6 to 9)
Palestine	2033	530 (418 to 656)	234 (179 to 298)	166 (129 to 216)	111 (69 to 173)	19 (16 to 23)	203 (160 to 251)	89 (69 to 114)	63 (49 to 82)	43 (27 to 66)	7 (6 to 9)
Palestine	2034	543 (430 to 685)	242 (183 to 308)	168 (130 to 218)	114 (71 to 178)	19 (16 to 23)	208 (165 to 262)	92 (70 to 118)	64 (50 to 83)	44 (27 to 68)	7 (6 to 9)
Palestine	2035	556 (440 to 690)	250 (188 to 318)	170 (131 to 220)	117 (73 to 183)	19 (16 to 23)	212 (168 to 264)	95 (72 to 122)	65 (50 to 84)	45 (28 to 70)	7 (6 to 9)
Palestine	2036	569 (451 to 723)	258 (193 to 329)	171 (132 to 223)	121 (75 to 188)	19 (16 to 24)	217 (172 to 276)	98 (74 to 126)	66 (51 to 85)	46 (29 to 72)	7 (6 to 9)
Palestine	2037	582 (457 to 719)	266 (199 to 339)	173 (134 to 225)	124 (77 to 193)	19 (16 to 24)	222 (175 to 275)	102 (76 to 130)	66 (51 to 86)	47 (29 to 74)	7 (6 to 9)
Palestine	2038	595 (469 to 759)	274 (204 to 352)	175 (135 to 227)	127 (79 to 198)	19 (16 to 24)	227 (179 to 290)	105 (78 to 134)	67 (52 to 87)	49 (30 to 76)	7 (6 to 9)
Palestine	2039	607 (477 to 762)	281 (211 to 364)	176 (136 to 229)	130 (81 to 202)	19 (16 to 25)	232 (182 to 291)	108 (81 to 139)	67 (52 to 88)	50 (31 to 77)	7 (6 to 9)

Palestine	2040	619 (484 to 794)	289 (213 to 376)	178 (137 to 231)	133 (82 to 208)	20 (16 to 25)	237 (185 to 304)	111 (82 to 144)	68 (52 to 88)	51 (31 to 79)	7 (6 to 9)
Palestine	2041	631 (492 to 794)	297 (219 to 388)	179 (138 to 233)	136 (83 to 212)	20 (16 to 25)	241 (188 to 303)	113 (84 to 148)	68 (53 to 89)	52 (32 to 81)	8 (6 to 10)
Palestine	2042	643 (501 to 830)	304 (225 to 398)	180 (138 to 235)	139 (85 to 217)	20 (16 to 25)	246 (192 to 317)	116 (86 to 152)	69 (53 to 90)	53 (32 to 83)	8 (6 to 10)
Palestine	2043	654 (511 to 831)	311 (229 to 412)	181 (139 to 236)	141 (86 to 222)	20 (16 to 26)	250 (195 to 317)	119 (87 to 157)	69 (53 to 90)	54 (33 to 85)	8 (6 to 10)
Palestine	2044	666 (518 to 867)	319 (235 to 425)	182 (140 to 238)	144 (88 to 226)	20 (16 to 26)	255 (198 to 332)	122 (90 to 163)	70 (53 to 91)	55 (34 to 86)	8 (6 to 10)
Palestine	2045	678 (527 to 859)	327 (241 to 435)	184 (141 to 240)	147 (90 to 230)	20 (16 to 26)	259 (201 to 328)	125 (92 to 166)	70 (54 to 92)	56 (34 to 88)	8 (6 to 10)
Palestine	2046	690 (532 to 900)	335 (242 to 441)	185 (141 to 241)	150 (92 to 235)	20 (16 to 26)	264 (203 to 344)	128 (93 to 169)	71 (54 to 92)	57 (35 to 90)	8 (6 to 10)
Palestine	2047	702 (546 to 890)	343 (248 to 448)	186 (142 to 243)	153 (94 to 240)	21 (16 to 27)	269 (209 to 340)	131 (95 to 171)	71 (54 to 93)	59 (36 to 92)	8 (6 to 10)
Palestine	2048	715 (548 to 933)	351 (255 to 460)	187 (143 to 245)	156 (95 to 245)	21 (16 to 28)	273 (209 to 357)	134 (97 to 176)	71 (55 to 93)	60 (36 to 94)	8 (6 to 11)
Palestine	2049	728 (561 to 927)	360 (260 to 474)	188 (144 to 246)	160 (97 to 250)	21 (16 to 28)	278 (215 to 354)	137 (99 to 181)	72 (55 to 94)	61 (37 to 95)	8 (6 to 11)
Palestine	2050	741 (568 to 969)	368 (267 to 488)	189 (145 to 247)	163 (99 to 255)	21 (17 to 29)	283 (217 to 370)	141 (102 to 187)	72 (55 to 95)	62 (38 to 97)	8 (6 to 11)
Panama	1995	491 (451 to 534)	328 (295 to 362)	142 (122 to 165)	19 (11 to 30)	3 (3 to 3)	807 (741 to 876)	538 (484 to 595)	233 (201 to 270)	31 (18 to 49)	5 (5 to 5)
Panama	1996	455 (423 to 486)	291 (266 to 317)	136 (119 to 154)	17 (10 to 27)	11 (11 to 11)	747 (694 to 798)	478 (437 to 521)	223 (196 to 254)	27 (17 to 44)	18 (18 to 18)
Panama	1997	486 (455 to 517)	320 (296 to 344)	140 (123 to 157)	19 (12 to 30)	7 (7 to 7)	797 (746 to 849)	525 (485 to 566)	229 (203 to 258)	31 (19 to 48)	12 (12 to 12)
Panama	1998	509 (478 to 539)	337 (313 to 364)	145 (129 to 162)	21 (14 to 31)	5 (5 to 5)	835 (784 to 885)	554 (515 to 597)	238 (212 to 267)	35 (22 to 52)	9 (9 to 9)

Panama	1999	511 (481 to 544)	338 (314 to 364)	145 (129 to 162)	22 (15 to 33)	5 (5 to 5)	839 (790 to 894)	556 (516 to 598)	238 (212 to 266)	37 (24 to 54)	8 (8 to 8)
Panama	2000	517 (487 to 549)	343 (318 to 368)	147 (131 to 164)	23 (15 to 34)	4 (4 to 4)	849 (799 to 902)	563 (523 to 604)	241 (215 to 270)	38 (25 to 56)	7 (7 to 7)
Panama	2001	519 (489 to 551)	342 (319 to 368)	149 (132 to 165)	24 (16 to 35)	5 (5 to 5)	853 (802 to 905)	562 (524 to 604)	244 (217 to 271)	39 (26 to 57)	8 (8 to 8)
Panama	2002	521 (492 to 555)	341 (317 to 367)	151 (135 to 168)	24 (16 to 34)	6 (6 to 6)	856 (808 to 911)	559 (520 to 603)	248 (221 to 276)	39 (26 to 56)	9 (9 to 9)
Panama	2003	541 (511 to 574)	355 (331 to 383)	157 (139 to 175)	25 (17 to 36)	3 (3 to 3)	888 (839 to 943)	583 (543 to 628)	258 (229 to 287)	41 (28 to 59)	5 (5 to 5)
Panama	2004	552 (521 to 587)	360 (334 to 388)	163 (145 to 181)	25 (17 to 36)	3 (3 to 3)	906 (856 to 964)	591 (549 to 636)	267 (239 to 298)	42 (27 to 59)	5 (5 to 5)
Panama	2005	574 (542 to 608)	374 (349 to 401)	171 (153 to 189)	26 (17 to 38)	3 (3 to 3)	943 (891 to 999)	615 (573 to 659)	280 (251 to 310)	43 (29 to 62)	5 (5 to 5)
Panama	2006	621 (589 to 658)	409 (384 to 438)	182 (164 to 201)	27 (18 to 39)	2 (2 to 2)	1020 (967 to 1080)	672 (630 to 719)	299 (270 to 329)	45 (30 to 64)	4 (4 to 4)
Panama	2007	668 (634 to 706)	444 (417 to 474)	194 (176 to 213)	28 (19 to 39)	2 (2 to 2)	1097 (1042 to 1160)	729 (686 to 779)	318 (288 to 349)	45 (31 to 65)	4 (4 to 4)
Panama	2008	738 (704 to 778)	496 (467 to 528)	211 (190 to 231)	29 (20 to 41)	2 (2 to 2)	1211 (1156 to 1277)	814 (766 to 866)	346 (313 to 380)	48 (32 to 68)	4 (4 to 4)
Panama	2009	763 (727 to 800)	504 (476 to 535)	219 (200 to 240)	29 (20 to 41)	10 (10 to 10)	1252 (1194 to 1313)	828 (782 to 879)	360 (328 to 393)	48 (33 to 68)	16 (16 to 16)
Panama	2010	801 (764 to 841)	530 (500 to 561)	237 (217 to 258)	32 (23 to 45)	2 (2 to 2)	1315 (1254 to 1381)	871 (820 to 921)	388 (356 to 423)	53 (37 to 74)	3 (3 to 3)

Panama	2011	839 (798 to 880)	544 (514 to 575)	257 (236 to 280)	36 (25 to 50)	2 (2 to 2)	1378 (1311 to 1445)	893 (843 to 944)	422 (387 to 460)	59 (42 to 82)	4 (4 to 4)
Panama	2012	879 (836 to 923)	553 (524 to 585)	279 (257 to 302)	43 (30 to 58)	5 (5 to 5)	1444 (1373 to 1515)	909 (860 to 961)	457 (421 to 497)	70 (50 to 94)	8 (8 to 8)
Panama	2013	957 (916 to 1002)	600 (566 to 633)	299 (276 to 324)	51 (36 to 70)	7 (7 to 7)	1572 (1504 to 1645)	985 (929 to 1039)	491 (454 to 533)	84 (60 to 114)	12 (12 to 12)
Panama	2014	988 (945 to 1034)	617 (583 to 653)	307 (283 to 333)	59 (42 to 79)	5 (5 to 5)	1622 (1552 to 1698)	1013 (957 to 1072)	504 (465 to 546)	96 (69 to 130)	8 (8 to 8)
Panama	2015	1030 (983 to 1076)	643 (611 to 679)	316 (292 to 342)	64 (46 to 86)	6 (6 to 6)	1691 (1614 to 1767)	1056 (1003 to 1115)	520 (479 to 561)	105 (76 to 141)	10 (10 to 10)
Panama	2016	1089 (1038 to 1143)	687 (649 to 727)	331 (303 to 361)	69 (49 to 93)	1 (1 to 1)	1788 (1704 to 1877)	1128 (1065 to 1193)	544 (498 to 592)	114 (81 to 153)	2 (2 to 2)
Panama	2017	1147 (1067 to 1235)	713 (666 to 765)	359 (322 to 399)	74 (51 to 104)	0 (0 to 0)	1883 (1752 to 2028)	1171 (1094 to 1257)	590 (529 to 655)	121 (84 to 170)	0 (0 to 0)
Panama	2018	1173 (1094 to 1262)	728 (679 to 780)	369 (331 to 410)	76 (53 to 106)	0 (0 to 0)	1926 (1796 to 2072)	1196 (1115 to 1282)	605 (543 to 672)	124 (86 to 175)	0 (0 to 0)
Panama	2019	1187 (1099 to 1284)	729 (678 to 786)	380 (341 to 421)	78 (54 to 110)	0 (0 to 0)	1949 (1804 to 2108)	1197 (1113 to 1291)	623 (559 to 692)	128 (89 to 181)	0 (0 to 0)
Panama	2020	1239 (1147 to 1346)	762 (701 to 826)	395 (354 to 438)	82 (57 to 115)	0 (0 to 0)	2034 (1883 to 2210)	1252 (1152 to 1356)	648 (581 to 719)	134 (93 to 188)	0 (0 to 0)

Panama	2021	1276 (1171 to 1392)	787 (718 to 859)	405 (363 to 450)	84 (58 to 118)	0 (0 to 0)	2095 (1923 to 2285)	1292 (1179 to 1410)	665 (596 to 738)	138 (96 to 194)	0 (0 to 0)
Panama	2022	1312 (1203 to 1431)	811 (735 to 890)	414 (371 to 460)	87 (60 to 121)	0 (0 to 0)	2155 (1976 to 2349)	1333 (1207 to 1461)	680 (609 to 755)	143 (99 to 199)	0 (0 to 0)
Panama	2023	1347 (1225 to 1479)	835 (754 to 921)	423 (379 to 470)	89 (62 to 125)	0 (0 to 0)	2212 (2012 to 2428)	1371 (1238 to 1513)	695 (622 to 772)	147 (101 to 205)	0 (0 to 0)
Panama	2024	1378 (1258 to 1512)	854 (767 to 948)	432 (387 to 480)	92 (63 to 129)	0 (0 to 0)	2262 (2066 to 2483)	1402 (1259 to 1557)	710 (636 to 789)	151 (104 to 211)	0 (0 to 0)
Panama	2025	1409 (1274 to 1547)	873 (786 to 968)	441 (395 to 491)	94 (65 to 132)	0 (0 to 0)	2313 (2093 to 2540)	1434 (1290 to 1590)	724 (648 to 806)	155 (107 to 216)	0 (0 to 0)
Panama	2026	1443 (1316 to 1585)	895 (806 to 993)	450 (402 to 501)	97 (67 to 135)	0 (0 to 0)	2369 (2161 to 2602)	1470 (1324 to 1631)	740 (661 to 823)	159 (110 to 222)	0 (0 to 0)
Panama	2027	1477 (1332 to 1631)	918 (820 to 1022)	460 (411 to 512)	100 (69 to 139)	0 (0 to 0)	2425 (2187 to 2678)	1507 (1346 to 1678)	755 (675 to 840)	164 (113 to 229)	0 (0 to 0)
Panama	2028	1513 (1373 to 1675)	941 (834 to 1053)	469 (419 to 523)	102 (71 to 143)	0 (0 to 0)	2484 (2255 to 2751)	1546 (1370 to 1729)	770 (688 to 859)	168 (116 to 235)	0 (0 to 0)
Panama	2029	1549 (1391 to 1721)	965 (847 to 1084)	478 (428 to 533)	105 (73 to 147)	0 (0 to 0)	2543 (2284 to 2825)	1585 (1391 to 1780)	786 (703 to 876)	173 (119 to 241)	0 (0 to 0)
Panama	2030	1588 (1429 to 1766)	992 (867 to 1122)	488 (437 to 544)	108 (75 to 151)	0 (0 to 0)	2608 (2346 to 2899)	1629 (1424 to 1843)	801 (717 to 893)	177 (122 to 248)	0 (0 to 0)

Panama	2031	1630 (1450 to 1827)	1021 (888 to 1161)	498 (445 to 554)	111 (76 to 155)	0 (0 to 0)	2676 (2381 to 3000)	1677 (1459 to 1906)	817 (731 to 910)	182 (125 to 255)	0 (0 to 0)
Panama	2032	1671 (1493 to 1873)	1051 (907 to 1203)	507 (454 to 566)	114 (78 to 159)	0 (0 to 0)	2744 (2451 to 3075)	1725 (1489 to 1975)	833 (745 to 929)	187 (128 to 261)	0 (0 to 0)
Panama	2033	1713 (1520 to 1923)	1080 (931 to 1239)	517 (462 to 577)	117 (80 to 164)	0 (0 to 0)	2813 (2496 to 3158)	1773 (1529 to 2035)	849 (759 to 948)	191 (132 to 269)	0 (0 to 0)
Panama	2034	1756 (1555 to 1975)	1109 (952 to 1271)	527 (471 to 589)	120 (82 to 169)	0 (0 to 0)	2883 (2553 to 3242)	1821 (1564 to 2087)	866 (774 to 967)	196 (135 to 277)	0 (0 to 0)
Panama	2035	1798 (1592 to 2022)	1139 (978 to 1316)	537 (480 to 600)	123 (84 to 173)	0 (0 to 0)	2953 (2615 to 3320)	1870 (1606 to 2161)	882 (789 to 985)	202 (139 to 284)	0 (0 to 0)
Panama	2036	1842 (1627 to 2079)	1169 (999 to 1353)	547 (489 to 612)	126 (86 to 178)	0 (0 to 0)	3024 (2672 to 3414)	1920 (1640 to 2221)	898 (803 to 1005)	207 (142 to 292)	0 (0 to 0)
Panama	2037	1883 (1661 to 2143)	1198 (1020 to 1388)	556 (496 to 623)	129 (88 to 182)	0 (0 to 0)	3092 (2727 to 3518)	1966 (1675 to 2279)	914 (815 to 1024)	212 (145 to 298)	0 (0 to 0)
Panama	2038	1927 (1695 to 2183)	1229 (1042 to 1426)	567 (504 to 635)	132 (90 to 186)	0 (0 to 0)	3165 (2783 to 3585)	2017 (1711 to 2342)	930 (828 to 1043)	217 (148 to 305)	0 (0 to 0)
Panama	2039	1972 (1734 to 2252)	1259 (1065 to 1477)	577 (512 to 647)	136 (93 to 190)	0 (0 to 0)	3237 (2847 to 3698)	2068 (1749 to 2426)	947 (841 to 1063)	223 (152 to 313)	0 (0 to 0)
Panama	2040	2016 (1759 to 2309)	1290 (1085 to 1514)	587 (521 to 659)	139 (95 to 195)	0 (0 to 0)	3310 (2888 to 3792)	2118 (1782 to 2486)	964 (856 to 1083)	228 (156 to 321)	0 (0 to 0)

Panama	2041	2062 (1797 to 2354)	1321 (1111 to 1557)	598 (530 to 672)	143 (97 to 201)	0 (0 to 0)	3386 (2950 to 3865)	2170 (1824 to 2556)	982 (871 to 1103)	234 (160 to 329)	0 (0 to 0)
Panama	2042	2110 (1837 to 2414)	1354 (1140 to 1596)	609 (540 to 684)	146 (100 to 206)	0 (0 to 0)	3464 (3016 to 3963)	2224 (1871 to 2621)	1000 (886 to 1124)	241 (164 to 338)	0 (0 to 0)
Panama	2043	2158 (1883 to 2464)	1388 (1164 to 1630)	620 (549 to 698)	150 (103 to 211)	0 (0 to 0)	3544 (3092 to 4046)	2278 (1912 to 2676)	1018 (902 to 1147)	247 (169 to 347)	0 (0 to 0)
Panama	2044	2206 (1911 to 2531)	1420 (1193 to 1666)	632 (559 to 712)	154 (106 to 217)	0 (0 to 0)	3623 (3138 to 4157)	2332 (1959 to 2735)	1037 (919 to 1169)	253 (173 to 356)	0 (0 to 0)
Panama	2045	2257 (1953 to 2588)	1455 (1216 to 1717)	643 (569 to 726)	158 (108 to 224)	0 (0 to 0)	3706 (3206 to 4250)	2389 (1997 to 2819)	1056 (935 to 1191)	260 (178 to 367)	0 (0 to 0)
Panama	2046	2308 (1987 to 2663)	1490 (1240 to 1777)	655 (579 to 739)	162 (111 to 229)	0 (0 to 0)	3789 (3263 to 4373)	2447 (2036 to 2918)	1075 (951 to 1214)	267 (183 to 376)	0 (0 to 0)
Panama	2047	2359 (2036 to 2720)	1525 (1264 to 1807)	667 (589 to 753)	167 (114 to 236)	0 (0 to 0)	3874 (3343 to 4467)	2505 (2075 to 2967)	1095 (968 to 1237)	274 (188 to 388)	0 (0 to 0)
Panama	2048	2410 (2074 to 2783)	1560 (1284 to 1850)	679 (600 to 767)	171 (118 to 242)	0 (0 to 0)	3958 (3406 to 4570)	2562 (2108 to 3038)	1115 (985 to 1260)	281 (193 to 398)	0 (0 to 0)
Panama	2049	2463 (2116 to 2844)	1597 (1328 to 1895)	691 (610 to 782)	176 (120 to 248)	0 (0 to 0)	4044 (3474 to 4670)	2622 (2180 to 3112)	1135 (1001 to 1284)	288 (198 to 408)	0 (0 to 0)
Panama	2050	2519 (2173 to 2922)	1636 (1350 to 1961)	703 (620 to 796)	180 (123 to 255)	0 (0 to 0)	4137 (3569 to 4798)	2686 (2217 to 3219)	1155 (1019 to 1308)	296 (202 to 418)	0 (0 to 0)

Papua New Guinea	1995	36 (29 to 45)	32 (24 to 41)	3 (2 to 4)	0 (0 to 0)	1 (1 to 1)	55 (43 to 68)	48 (37 to 62)	4 (3 to 6)	0 (0 to 0)	2 (2 to 2)
Papua New Guinea	1996	45 (38 to 55)	33 (25 to 42)	3 (2 to 4)	0 (0 to 0)	9 (9 to 9)	69 (57 to 83)	50 (38 to 64)	5 (3 to 7)	0 (0 to 0)	14 (14 to 14)
Papua New Guinea	1997	45 (37 to 54)	33 (25 to 43)	3 (2 to 5)	0 (0 to 0)	8 (8 to 8)	68 (56 to 81)	51 (39 to 65)	5 (3 to 7)	0 (0 to 0)	12 (12 to 12)
Papua New Guinea	1998	42 (35 to 51)	33 (25 to 41)	3 (2 to 5)	0 (0 to 0)	7 (7 to 7)	64 (52 to 78)	49 (38 to 62)	5 (3 to 7)	0 (0 to 0)	10 (10 to 10)
Papua New Guinea	1999	54 (46 to 63)	33 (26 to 42)	4 (2 to 5)	0 (0 to 0)	17 (17 to 17)	82 (70 to 96)	50 (39 to 64)	5 (4 to 8)	0 (0 to 0)	26 (26 to 26)
Papua New Guinea	2000	52 (45 to 61)	32 (24 to 40)	4 (2 to 5)	0 (0 to 0)	17 (17 to 17)	79 (68 to 92)	48 (37 to 61)	5 (4 to 8)	0 (0 to 0)	26 (26 to 26)
Papua New Guinea	2001	44 (37 to 52)	32 (24 to 40)	4 (3 to 5)	0 (0 to 0)	8 (8 to 8)	66 (56 to 79)	48 (37 to 60)	6 (4 to 8)	0 (0 to 0)	13 (13 to 13)
Papua New Guinea	2002	45 (38 to 53)	30 (23 to 38)	4 (3 to 5)	0 (0 to 0)	11 (11 to 11)	68 (58 to 80)	46 (35 to 58)	6 (4 to 8)	0 (0 to 0)	16 (16 to 16)
Papua New Guinea	2003	43 (36 to 51)	28 (22 to 36)	4 (3 to 5)	0 (0 to 0)	10 (10 to 10)	65 (55 to 77)	43 (33 to 54)	6 (4 to 8)	0 (0 to 0)	16 (16 to 16)
Papua New Guinea	2004	44 (37 to 52)	29 (23 to 37)	4 (3 to 5)	0 (0 to 0)	11 (11 to 11)	67 (57 to 79)	45 (34 to 56)	6 (4 to 8)	0 (0 to 0)	16 (16 to 16)
Papua New Guinea	2005	46 (39 to 54)	31 (24 to 39)	4 (3 to 5)	0 (0 to 0)	11 (11 to 11)	69 (59 to 82)	47 (36 to 59)	6 (4 to 8)	0 (0 to 0)	17 (17 to 17)
Papua New Guinea	2006	44 (38 to 52)	29 (23 to 37)	4 (3 to 5)	0 (0 to 0)	11 (11 to 11)	67 (57 to 79)	44 (35 to 56)	6 (4 to 8)	0 (0 to 0)	17 (17 to 17)
Papua New Guinea	2007	43 (36 to 51)	29 (23 to 37)	4 (3 to 5)	0 (0 to 0)	10 (10 to 10)	65 (55 to 77)	44 (34 to 55)	6 (4 to 8)	0 (0 to 0)	15 (15 to 15)
Papua New Guinea	2008	45 (39 to 53)	29 (22 to 37)	4 (3 to 6)	0 (0 to 0)	12 (12 to 12)	69 (59 to 81)	44 (34 to 55)	6 (4 to 8)	0 (0 to 0)	19 (19 to 19)
Papua New Guinea	2009	51 (44 to 60)	33 (26 to 42)	5 (3 to 6)	0 (0 to 0)	14 (14 to 14)	78 (67 to 91)	50 (39 to 63)	7 (5 to 10)	0 (0 to 0)	21 (21 to 21)

Papua New Guinea	2010	49 (43 to 57)	29 (23 to 36)	5 (3 to 6)	0 (0 to 0)	16 (16 to 16)	74 (65 to 86)	44 (34 to 55)	7 (5 to 10)	0 (0 to 0)	24 (24 to 24)
Papua New Guinea	2011	56 (49 to 64)	31 (24 to 38)	5 (3 to 7)	0 (0 to 0)	21 (21 to 21)	85 (75 to 97)	46 (36 to 58)	7 (5 to 10)	0 (0 to 0)	32 (32 to 32)
Papua New Guinea	2012	62 (55 to 71)	35 (27 to 43)	5 (3 to 7)	0 (0 to 0)	23 (23 to 23)	94 (83 to 108)	53 (42 to 66)	7 (5 to 10)	0 (0 to 0)	34 (34 to 34)
Papua New Guinea	2013	65 (56 to 74)	41 (32 to 50)	5 (3 to 7)	0 (0 to 0)	19 (19 to 19)	98 (85 to 113)	62 (49 to 76)	7 (5 to 11)	0 (0 to 0)	29 (29 to 29)
Papua New Guinea	2014	64 (54 to 74)	43 (34 to 54)	5 (4 to 7)	0 (0 to 0)	15 (15 to 15)	97 (82 to 112)	65 (51 to 81)	8 (5 to 11)	0 (0 to 0)	23 (23 to 23)
Papua New Guinea	2015	59 (50 to 70)	40 (31 to 50)	5 (4 to 7)	0 (0 to 0)	14 (14 to 14)	90 (76 to 106)	61 (47 to 76)	8 (5 to 11)	0 (0 to 0)	22 (22 to 22)
Papua New Guinea	2016	55 (46 to 66)	41 (31 to 51)	5 (4 to 8)	0 (0 to 0)	9 (9 to 9)	84 (69 to 100)	62 (47 to 77)	8 (6 to 11)	0 (0 to 0)	14 (14 to 14)
Papua New Guinea	2017	54 (44 to 64)	40 (30 to 50)	5 (4 to 7)	0 (0 to 0)	9 (9 to 9)	82 (67 to 98)	60 (46 to 75)	8 (5 to 11)	0 (0 to 0)	13 (13 to 13)
Papua New Guinea	2018	62 (50 to 75)	48 (36 to 61)	5 (4 to 7)	0 (0 to 0)	9 (8 to 9)	94 (76 to 113)	73 (55 to 92)	8 (5 to 11)	0 (0 to 0)	13 (13 to 13)
Papua New Guinea	2019	61 (50 to 74)	48 (36 to 60)	5 (4 to 7)	0 (0 to 0)	8 (8 to 9)	93 (76 to 113)	72 (55 to 91)	8 (6 to 11)	0 (0 to 0)	13 (13 to 13)
Papua New Guinea	2020	63 (51 to 76)	48 (37 to 62)	5 (4 to 8)	0 (0 to 0)	9 (8 to 9)	95 (77 to 115)	74 (56 to 94)	8 (6 to 11)	0 (0 to 0)	13 (12 to 14)
Papua New Guinea	2021	63 (52 to 77)	49 (37 to 63)	5 (4 to 8)	0 (0 to 0)	9 (8 to 10)	96 (78 to 117)	75 (57 to 95)	8 (6 to 12)	0 (0 to 0)	13 (13 to 14)
Papua New Guinea	2022	64 (53 to 78)	50 (38 to 63)	6 (4 to 8)	0 (0 to 0)	9 (8 to 10)	98 (80 to 119)	76 (58 to 96)	8 (6 to 12)	0 (0 to 0)	14 (12 to 15)
Papua New Guinea	2023	65 (53 to 79)	50 (38 to 64)	6 (4 to 8)	0 (0 to 0)	9 (8 to 10)	99 (80 to 120)	76 (58 to 98)	8 (6 to 12)	0 (0 to 0)	14 (12 to 15)
Papua New Guinea	2024	66 (54 to 80)	51 (39 to 66)	6 (4 to 8)	0 (0 to 0)	9 (8 to 10)	100 (81 to 121)	77 (59 to 100)	9 (6 to 12)	0 (0 to 0)	14 (12 to 16)

Papua New Guinea	2025	67 (54 to 81)	52 (40 to 66)	6 (4 to 8)	0 (0 to 0)	9 (8 to 10)	101 (82 to 123)	78 (60 to 100)	9 (6 to 12)	0 (0 to 0)	14 (12 to 16)
Papua New Guinea	2026	67 (55 to 82)	52 (40 to 67)	6 (4 to 8)	0 (0 to 0)	9 (8 to 11)	102 (83 to 125)	79 (61 to 102)	9 (6 to 12)	0 (0 to 0)	14 (12 to 16)
Papua New Guinea	2027	68 (55 to 83)	53 (40 to 68)	6 (4 to 8)	0 (0 to 0)	9 (8 to 11)	103 (84 to 126)	80 (61 to 103)	9 (6 to 13)	0 (0 to 0)	14 (12 to 16)
Papua New Guinea	2028	69 (56 to 85)	54 (41 to 69)	6 (4 to 8)	0 (0 to 0)	9 (8 to 11)	105 (84 to 129)	82 (62 to 105)	9 (6 to 13)	0 (0 to 0)	14 (12 to 16)
Papua New Guinea	2029	70 (57 to 86)	55 (41 to 70)	6 (4 to 8)	0 (0 to 0)	9 (8 to 11)	106 (86 to 130)	83 (63 to 106)	9 (6 to 13)	0 (0 to 0)	14 (12 to 17)
Papua New Guinea	2030	71 (57 to 87)	56 (42 to 72)	6 (4 to 9)	0 (0 to 0)	9 (8 to 11)	108 (87 to 133)	84 (64 to 109)	9 (6 to 13)	0 (0 to 0)	14 (12 to 17)
Papua New Guinea	2031	72 (58 to 90)	57 (43 to 74)	6 (4 to 9)	0 (0 to 0)	9 (8 to 11)	110 (88 to 136)	86 (65 to 112)	9 (7 to 13)	0 (0 to 0)	14 (12 to 17)
Papua New Guinea	2032	74 (59 to 91)	58 (43 to 75)	6 (4 to 9)	0 (0 to 0)	9 (8 to 11)	112 (89 to 139)	88 (66 to 114)	10 (7 to 14)	0 (0 to 0)	14 (12 to 17)
Papua New Guinea	2033	75 (60 to 94)	59 (45 to 77)	6 (4 to 9)	0 (0 to 0)	10 (8 to 11)	114 (92 to 142)	90 (68 to 117)	10 (7 to 14)	0 (0 to 0)	14 (12 to 17)
Papua New Guinea	2034	77 (61 to 96)	61 (45 to 79)	7 (5 to 9)	0 (0 to 0)	10 (8 to 12)	116 (93 to 145)	92 (69 to 120)	10 (7 to 14)	0 (0 to 0)	14 (12 to 18)
Papua New Guinea	2035	78 (62 to 98)	62 (46 to 81)	7 (5 to 10)	0 (0 to 0)	10 (8 to 12)	119 (95 to 149)	94 (70 to 123)	10 (7 to 14)	0 (0 to 0)	15 (12 to 18)
Papua New Guinea	2036	80 (63 to 100)	64 (47 to 82)	7 (5 to 10)	0 (0 to 0)	10 (8 to 12)	121 (96 to 151)	96 (72 to 125)	10 (7 to 15)	0 (0 to 0)	15 (12 to 18)
Papua New Guinea	2037	82 (65 to 101)	65 (49 to 84)	7 (5 to 10)	0 (0 to 0)	10 (8 to 12)	124 (98 to 154)	99 (74 to 128)	11 (7 to 15)	0 (0 to 0)	15 (12 to 18)
Papua New Guinea	2038	84 (66 to 105)	67 (50 to 87)	7 (5 to 10)	0 (0 to 0)	10 (8 to 12)	127 (101 to 159)	101 (75 to 132)	11 (7 to 15)	0 (0 to 0)	15 (12 to 18)
Papua New Guinea	2039	86 (68 to 107)	69 (51 to 90)	7 (5 to 10)	0 (0 to 0)	10 (8 to 12)	130 (103 to 163)	104 (78 to 136)	11 (8 to 16)	0 (0 to 0)	15 (12 to 19)

Papua New Guinea	2040	88 (69 to 110)	70 (53 to 92)	7 (5 to 10)	0 (0 to 0)	10 (8 to 12)	133 (105 to 166)	107 (80 to 140)	11 (8 to 16)	0 (0 to 0)	15 (12 to 19)
Papua New Guinea	2041	90 (71 to 112)	72 (54 to 95)	8 (5 to 11)	0 (0 to 0)	10 (8 to 12)	136 (107 to 170)	109 (82 to 143)	12 (8 to 16)	0 (0 to 0)	15 (12 to 19)
Papua New Guinea	2042	92 (72 to 115)	74 (55 to 97)	8 (5 to 11)	0 (0 to 0)	10 (8 to 12)	139 (109 to 175)	112 (83 to 147)	12 (8 to 17)	0 (0 to 0)	15 (12 to 19)
Papua New Guinea	2043	94 (74 to 118)	76 (56 to 100)	8 (5 to 11)	0 (0 to 0)	10 (8 to 13)	142 (112 to 178)	115 (85 to 152)	12 (8 to 17)	0 (0 to 0)	15 (12 to 19)
Papua New Guinea	2044	95 (75 to 120)	78 (57 to 102)	8 (6 to 11)	0 (0 to 0)	10 (8 to 13)	145 (114 to 183)	118 (87 to 155)	12 (8 to 17)	0 (0 to 0)	15 (12 to 20)
Papua New Guinea	2045	97 (76 to 123)	79 (58 to 104)	8 (6 to 12)	0 (0 to 0)	10 (8 to 13)	148 (115 to 186)	120 (89 to 158)	12 (9 to 18)	0 (0 to 0)	15 (12 to 19)
Papua New Guinea	2046	99 (77 to 125)	81 (60 to 107)	8 (6 to 12)	0 (0 to 0)	10 (8 to 13)	151 (117 to 190)	123 (91 to 163)	13 (9 to 18)	0 (0 to 0)	15 (12 to 19)
Papua New Guinea	2047	101 (79 to 127)	83 (61 to 109)	9 (6 to 12)	0 (0 to 0)	10 (8 to 13)	154 (119 to 193)	126 (92 to 166)	13 (9 to 18)	0 (0 to 0)	15 (12 to 20)
Papua New Guinea	2048	103 (80 to 130)	85 (62 to 111)	9 (6 to 12)	0 (0 to 0)	10 (8 to 13)	157 (121 to 198)	128 (95 to 168)	13 (9 to 19)	0 (0 to 0)	15 (12 to 20)
Papua New Guinea	2049	105 (82 to 133)	87 (63 to 114)	9 (6 to 13)	0 (0 to 0)	10 (8 to 13)	160 (124 to 202)	131 (95 to 173)	13 (9 to 19)	0 (0 to 0)	15 (12 to 20)
Papua New Guinea	2050	108 (83 to 136)	88 (64 to 116)	9 (6 to 13)	0 (0 to 0)	10 (8 to 14)	163 (126 to 207)	134 (97 to 176)	14 (9 to 19)	0 (0 to 0)	15 (12 to 21)
Paraguay	1995	162 (140 to 186)	68 (56 to 82)	82 (66 to 101)	12 (6 to 21)	0 (0 to 0)	389 (337 to 447)	164 (134 to 197)	196 (159 to 244)	28 (14 to 50)	0 (0 to 0)
Paraguay	1996	167 (146 to 190)	72 (60 to 86)	82 (67 to 99)	13 (7 to 23)	0 (0 to 0)	402 (353 to 457)	173 (145 to 207)	197 (161 to 239)	32 (17 to 56)	0 (0 to 0)
Paraguay	1997	176 (157 to 199)	76 (64 to 89)	83 (69 to 99)	16 (9 to 27)	1 (1 to 1)	424 (377 to 479)	183 (155 to 215)	200 (166 to 238)	39 (22 to 64)	2 (2 to 2)
Paraguay	1998	184 (164 to 206)	79 (66 to 92)	83 (70 to 99)	17 (10 to 27)	5 (5 to 5)	443 (394 to 497)	190 (160 to 221)	201 (168 to 239)	41 (24 to 66)	11 (11 to 11)

Paraguay	1999	191 (170 to 215)	80 (68 to 94)	85 (72 to 101)	19 (11 to 30)	6 (6 to 6)	459 (410 to 517)	193 (163 to 227)	206 (174 to 243)	46 (26 to 73)	15 (15 to 15)
Paraguay	2000	192 (171 to 217)	79 (67 to 92)	88 (75 to 104)	21 (12 to 34)	4 (4 to 4)	462 (412 to 522)	189 (162 to 222)	213 (181 to 251)	50 (29 to 81)	10 (10 to 10)
Paraguay	2001	182 (161 to 206)	72 (62 to 85)	86 (74 to 101)	21 (12 to 34)	2 (2 to 2)	439 (389 to 496)	174 (149 to 204)	208 (177 to 243)	52 (30 to 83)	6 (6 to 6)
Paraguay	2002	174 (154 to 196)	67 (58 to 79)	84 (70 to 98)	21 (12 to 34)	2 (2 to 2)	419 (371 to 471)	163 (140 to 189)	201 (169 to 236)	51 (29 to 81)	4 (4 to 4)
Paraguay	2003	168 (149 to 190)	65 (55 to 75)	82 (69 to 96)	20 (12 to 31)	2 (2 to 2)	405 (358 to 457)	156 (133 to 182)	197 (166 to 230)	47 (28 to 75)	5 (5 to 5)
Paraguay	2004	169 (149 to 189)	65 (56 to 76)	83 (69 to 97)	19 (11 to 29)	2 (2 to 2)	406 (360 to 454)	157 (135 to 184)	199 (167 to 235)	45 (27 to 70)	4 (4 to 4)
Paraguay	2005	170 (151 to 189)	67 (58 to 78)	83 (71 to 99)	18 (10 to 27)	2 (2 to 2)	408 (363 to 456)	161 (139 to 187)	201 (170 to 238)	42 (25 to 65)	4 (4 to 4)
Paraguay	2006	176 (157 to 196)	72 (61 to 83)	86 (73 to 102)	17 (10 to 27)	2 (2 to 2)	424 (377 to 472)	172 (148 to 199)	207 (176 to 245)	41 (24 to 65)	4 (4 to 4)
Paraguay	2007	180 (162 to 201)	74 (63 to 85)	88 (75 to 103)	16 (9 to 26)	3 (3 to 3)	434 (390 to 483)	178 (153 to 205)	211 (181 to 249)	38 (23 to 62)	7 (7 to 7)
Paraguay	2008	187 (168 to 206)	77 (66 to 88)	90 (77 to 106)	15 (9 to 24)	4 (4 to 4)	449 (404 to 497)	186 (160 to 212)	218 (186 to 255)	37 (22 to 59)	10 (10 to 10)
Paraguay	2009	210 (189 to 232)	93 (81 to 106)	97 (84 to 113)	16 (10 to 26)	4 (4 to 4)	505 (455 to 558)	223 (194 to 255)	233 (201 to 272)	39 (24 to 63)	9 (9 to 9)
Paraguay	2010	228 (206 to 250)	102 (89 to 116)	104 (90 to 120)	17 (11 to 28)	5 (5 to 5)	548 (497 to 603)	245 (215 to 279)	249 (216 to 288)	42 (26 to 68)	12 (12 to 12)
Paraguay	2011	256 (233 to 281)	118 (103 to 134)	116 (100 to 133)	20 (12 to 32)	3 (3 to 3)	617 (562 to 677)	284 (248 to 324)	278 (240 to 320)	48 (29 to 77)	6 (6 to 6)
Paraguay	2012	311 (282 to 341)	145 (127 to 166)	138 (119 to 159)	24 (15 to 40)	3 (3 to 3)	748 (678 to 821)	349 (305 to 400)	332 (287 to 382)	59 (36 to 95)	8 (8 to 8)
Paraguay	2013	312 (283 to 342)	142 (124 to 161)	143 (123 to 164)	25 (15 to 40)	2 (2 to 2)	750 (682 to 824)	341 (299 to 388)	343 (297 to 395)	60 (37 to 95)	6 (6 to 6)

Paraguay	2014	341 (308 to 375)	158 (138 to 180)	154 (134 to 177)	28 (17 to 46)	2 (2 to 2)	822 (742 to 903)	380 (332 to 433)	370 (322 to 425)	68 (42 to 110)	4 (4 to 4)
Paraguay	2015	365 (330 to 405)	170 (147 to 196)	161 (139 to 185)	32 (19 to 52)	2 (2 to 2)	879 (795 to 975)	409 (355 to 471)	388 (334 to 447)	77 (47 to 125)	4 (4 to 4)
Paraguay	2016	373 (336 to 415)	172 (149 to 199)	164 (139 to 191)	35 (20 to 57)	2 (2 to 2)	899 (809 to 1000)	415 (358 to 479)	395 (335 to 459)	83 (49 to 137)	5 (5 to 5)
Paraguay	2017	389 (338 to 453)	179 (154 to 210)	170 (140 to 200)	38 (22 to 63)	2 (2 to 2)	937 (813 to 1091)	432 (372 to 506)	408 (338 to 482)	91 (52 to 151)	6 (6 to 6)
Paraguay	2018	397 (346 to 463)	182 (156 to 214)	174 (144 to 205)	39 (22 to 64)	2 (2 to 2)	955 (832 to 1114)	439 (375 to 515)	418 (346 to 493)	94 (54 to 155)	5 (5 to 5)
Paraguay	2019	394 (345 to 462)	179 (153 to 210)	174 (144 to 205)	39 (23 to 65)	2 (2 to 2)	948 (831 to 1111)	431 (368 to 505)	418 (346 to 493)	94 (54 to 156)	5 (5 to 5)
Paraguay	2020	409 (358 to 476)	188 (160 to 220)	178 (147 to 210)	40 (23 to 67)	2 (2 to 2)	984 (861 to 1146)	452 (386 to 530)	429 (355 to 507)	97 (56 to 162)	5 (5 to 6)
Paraguay	2021	420 (364 to 490)	195 (166 to 229)	181 (149 to 214)	42 (24 to 69)	2 (2 to 2)	1011 (876 to 1181)	470 (401 to 551)	435 (360 to 514)	100 (58 to 167)	5 (5 to 6)
Paraguay	2022	431 (373 to 506)	202 (173 to 237)	183 (152 to 216)	43 (25 to 71)	2 (2 to 3)	1037 (899 to 1218)	488 (416 to 572)	441 (365 to 521)	103 (59 to 171)	5 (5 to 6)
Paraguay	2023	442 (383 to 517)	210 (179 to 247)	186 (154 to 219)	44 (25 to 73)	2 (2 to 3)	1065 (921 to 1245)	506 (432 to 594)	447 (370 to 528)	106 (61 to 176)	6 (5 to 6)
Paraguay	2024	453 (388 to 526)	218 (186 to 256)	188 (155 to 222)	45 (26 to 75)	2 (2 to 3)	1092 (935 to 1267)	525 (447 to 617)	452 (374 to 534)	109 (62 to 181)	6 (5 to 6)
Paraguay	2025	465 (398 to 545)	226 (193 to 265)	190 (157 to 225)	46 (27 to 77)	2 (2 to 3)	1119 (958 to 1311)	544 (464 to 639)	458 (379 to 541)	112 (64 to 186)	6 (5 to 6)

Paraguay	2026	476 (407 to 556)	234 (200 to 276)	192 (159 to 227)	48 (27 to 80)	2 (2 to 3)	1147 (980 to 1339)	563 (481 to 664)	463 (382 to 548)	115 (66 to 192)	6 (5 to 7)
Paraguay	2027	489 (417 to 569)	242 (207 to 286)	195 (161 to 230)	49 (28 to 82)	2 (2 to 3)	1177 (1003 to 1369)	584 (498 to 689)	469 (387 to 555)	118 (68 to 197)	6 (5 to 7)
Paraguay	2028	501 (428 to 586)	251 (214 to 296)	197 (163 to 233)	50 (29 to 84)	2 (2 to 3)	1207 (1030 to 1410)	605 (515 to 713)	475 (392 to 562)	121 (70 to 203)	6 (5 to 7)
Paraguay	2029	514 (440 to 601)	260 (222 to 308)	200 (165 to 236)	52 (30 to 87)	2 (2 to 3)	1239 (1060 to 1446)	627 (534 to 742)	481 (397 to 570)	125 (71 to 209)	6 (5 to 7)
Paraguay	2030	528 (451 to 619)	270 (229 to 320)	202 (167 to 240)	53 (30 to 89)	2 (2 to 3)	1271 (1087 to 1490)	650 (553 to 771)	487 (401 to 577)	128 (73 to 215)	6 (5 to 7)
Paraguay	2031	542 (464 to 638)	279 (237 to 331)	205 (168 to 243)	55 (31 to 92)	3 (2 to 3)	1304 (1118 to 1536)	673 (570 to 797)	494 (406 to 585)	132 (75 to 222)	6 (5 to 7)
Paraguay	2032	555 (472 to 653)	289 (245 to 343)	208 (171 to 246)	56 (32 to 95)	3 (2 to 3)	1338 (1138 to 1572)	696 (591 to 826)	500 (411 to 593)	135 (77 to 228)	6 (5 to 7)
Paraguay	2033	570 (485 to 669)	299 (253 to 355)	211 (173 to 250)	58 (33 to 97)	3 (2 to 3)	1372 (1167 to 1611)	720 (609 to 854)	508 (416 to 602)	139 (79 to 235)	6 (5 to 7)
Paraguay	2034	585 (496 to 691)	309 (261 to 367)	214 (175 to 254)	59 (34 to 100)	3 (2 to 3)	1408 (1196 to 1663)	744 (630 to 883)	515 (422 to 611)	143 (81 to 241)	6 (5 to 8)
Paraguay	2035	600 (510 to 705)	319 (270 to 379)	217 (177 to 258)	61 (35 to 103)	3 (2 to 3)	1444 (1228 to 1699)	768 (651 to 912)	522 (427 to 620)	147 (83 to 247)	6 (5 to 8)

Paraguay	2036	614 (521 to 721)	329 (279 to 391)	220 (180 to 261)	62 (35 to 105)	3 (2 to 3)	1479 (1254 to 1735)	793 (672 to 942)	529 (433 to 630)	151 (85 to 254)	6 (5 to 8)
Paraguay	2037	629 (536 to 741)	339 (287 to 405)	223 (182 to 265)	64 (36 to 108)	3 (2 to 3)	1514 (1290 to 1785)	817 (692 to 975)	536 (439 to 637)	154 (87 to 260)	7 (5 to 8)
Paraguay	2038	643 (546 to 760)	349 (296 to 417)	226 (184 to 268)	66 (37 to 110)	3 (2 to 3)	1549 (1316 to 1830)	841 (712 to 1004)	543 (444 to 645)	158 (89 to 266)	7 (5 to 8)
Paraguay	2039	658 (562 to 777)	360 (303 to 429)	228 (187 to 271)	67 (38 to 113)	3 (2 to 4)	1585 (1354 to 1872)	866 (730 to 1034)	550 (449 to 653)	162 (91 to 273)	7 (6 to 8)
Paraguay	2040	673 (575 to 793)	370 (312 to 442)	231 (189 to 275)	69 (39 to 116)	3 (2 to 4)	1621 (1385 to 1910)	891 (750 to 1064)	557 (454 to 661)	166 (93 to 279)	7 (6 to 9)
Paraguay	2041	688 (588 to 814)	380 (319 to 455)	234 (191 to 278)	70 (39 to 119)	3 (2 to 4)	1657 (1417 to 1960)	916 (769 to 1095)	564 (460 to 669)	170 (95 to 286)	7 (6 to 9)
Paraguay	2042	703 (598 to 828)	391 (328 to 467)	237 (193 to 281)	72 (40 to 121)	3 (2 to 4)	1693 (1439 to 1994)	941 (790 to 1124)	571 (465 to 678)	173 (97 to 292)	7 (6 to 9)
Paraguay	2043	717 (606 to 852)	401 (335 to 479)	240 (195 to 285)	74 (41 to 124)	3 (2 to 4)	1728 (1459 to 2052)	966 (808 to 1155)	577 (468 to 686)	177 (99 to 298)	7 (6 to 9)
Paraguay	2044	733 (617 to 866)	412 (343 to 494)	243 (197 to 289)	75 (42 to 127)	3 (2 to 4)	1765 (1487 to 2085)	992 (826 to 1189)	584 (474 to 695)	181 (101 to 305)	7 (6 to 10)
Paraguay	2045	748 (629 to 890)	423 (351 to 507)	245 (199 to 292)	77 (43 to 129)	3 (2 to 4)	1800 (1514 to 2143)	1018 (844 to 1222)	590 (480 to 703)	185 (103 to 312)	7 (6 to 9)

Paraguay	2046	763 (641 to 909)	433 (359 to 520)	248 (201 to 295)	78 (44 to 132)	3 (2 to 4)	1837 (1543 to 2190)	1044 (865 to 1251)	597 (485 to 710)	189 (105 to 319)	7 (6 to 10)
Paraguay	2047	777 (658 to 929)	444 (368 to 532)	250 (203 to 298)	80 (44 to 135)	3 (2 to 4)	1871 (1584 to 2238)	1069 (886 to 1281)	602 (489 to 717)	193 (107 to 325)	8 (6 to 10)
Paraguay	2048	791 (667 to 947)	454 (377 to 544)	253 (205 to 301)	81 (45 to 138)	3 (3 to 4)	1906 (1606 to 2280)	1094 (908 to 1310)	608 (493 to 726)	196 (109 to 332)	8 (6 to 10)
Paraguay	2049	806 (684 to 961)	465 (384 to 557)	255 (207 to 305)	83 (46 to 140)	3 (3 to 4)	1940 (1646 to 2315)	1119 (925 to 1341)	614 (497 to 735)	200 (111 to 338)	8 (6 to 10)
Paraguay	2050	820 (689 to 977)	475 (392 to 569)	257 (208 to 308)	84 (47 to 142)	3 (3 to 4)	1974 (1660 to 2352)	1144 (945 to 1369)	619 (502 to 743)	203 (113 to 343)	8 (6 to 11)
Peru	1995	165 (143 to 191)	81 (68 to 96)	62 (47 to 81)	17 (9 to 29)	5 (5 to 5)	344 (298 to 397)	168 (141 to 200)	130 (98 to 168)	35 (19 to 59)	11 (11 to 11)
Peru	1996	168 (146 to 191)	83 (70 to 98)	63 (49 to 79)	18 (9 to 29)	3 (3 to 3)	349 (304 to 398)	174 (146 to 203)	132 (102 to 164)	37 (19 to 61)	6 (6 to 6)
Peru	1997	173 (151 to 197)	87 (74 to 102)	64 (51 to 79)	18 (10 to 31)	3 (3 to 3)	359 (315 to 410)	181 (155 to 211)	134 (106 to 165)	38 (21 to 64)	6 (6 to 6)
Peru	1998	175 (154 to 198)	89 (77 to 104)	63 (51 to 78)	19 (10 to 31)	4 (4 to 4)	364 (321 to 413)	185 (161 to 216)	132 (105 to 162)	39 (21 to 64)	8 (8 to 8)
Peru	1999	179 (158 to 202)	92 (80 to 106)	64 (51 to 78)	19 (11 to 31)	4 (4 to 4)	372 (329 to 420)	192 (166 to 221)	133 (106 to 163)	40 (22 to 65)	7 (7 to 7)
Peru	2000	183 (162 to 207)	94 (82 to 108)	65 (52 to 80)	20 (11 to 32)	4 (4 to 4)	380 (336 to 431)	196 (171 to 225)	134 (109 to 167)	41 (23 to 67)	9 (9 to 9)
Peru	2001	184 (163 to 207)	96 (84 to 110)	64 (52 to 80)	20 (12 to 32)	3 (3 to 3)	383 (339 to 431)	200 (175 to 228)	134 (109 to 167)	42 (24 to 68)	7 (7 to 7)
Peru	2002	188 (166 to 212)	99 (87 to 114)	64 (53 to 79)	20 (12 to 33)	4 (4 to 4)	392 (345 to 441)	207 (181 to 237)	134 (110 to 165)	43 (24 to 69)	8 (8 to 8)

Peru	2003	194 (171 to 217)	104 (91 to 119)	65 (53 to 80)	21 (12 to 34)	4 (4 to 4)	403 (356 to 451)	216 (189 to 249)	135 (110 to 166)	43 (25 to 70)	9 (9 to 9)
Peru	2004	193 (171 to 217)	103 (90 to 118)	67 (55 to 82)	19 (11 to 30)	4 (4 to 4)	402 (357 to 451)	214 (187 to 246)	140 (114 to 171)	39 (22 to 63)	9 (9 to 9)
Peru	2005	201 (179 to 225)	106 (92 to 121)	72 (59 to 88)	19 (11 to 30)	4 (4 to 4)	418 (372 to 467)	220 (191 to 251)	151 (123 to 183)	39 (22 to 62)	8 (8 to 8)
Peru	2006	204 (183 to 228)	104 (91 to 119)	79 (65 to 96)	19 (11 to 30)	3 (3 to 3)	425 (380 to 473)	216 (189 to 247)	164 (136 to 199)	39 (22 to 62)	6 (6 to 6)
Peru	2007	213 (190 to 239)	105 (91 to 120)	86 (72 to 105)	18 (10 to 30)	3 (3 to 3)	443 (396 to 496)	219 (190 to 251)	179 (149 to 219)	38 (21 to 62)	6 (6 to 6)
Peru	2008	234 (211 to 261)	115 (99 to 131)	95 (80 to 115)	19 (11 to 31)	5 (5 to 5)	488 (439 to 543)	239 (206 to 272)	198 (166 to 239)	40 (23 to 64)	11 (11 to 11)
Peru	2009	251 (226 to 278)	126 (109 to 143)	100 (85 to 121)	20 (11 to 32)	5 (5 to 5)	522 (471 to 579)	261 (227 to 297)	209 (177 to 252)	41 (24 to 66)	11 (11 to 11)
Peru	2010	263 (238 to 291)	135 (118 to 153)	104 (88 to 124)	20 (12 to 33)	4 (4 to 4)	547 (494 to 605)	281 (246 to 319)	216 (184 to 258)	42 (24 to 68)	8 (8 to 8)
Peru	2011	267 (242 to 294)	140 (123 to 159)	104 (88 to 125)	20 (12 to 34)	2 (2 to 2)	555 (503 to 612)	292 (257 to 330)	217 (184 to 260)	42 (25 to 70)	4 (4 to 4)
Peru	2012	282 (256 to 308)	153 (135 to 172)	105 (87 to 126)	21 (12 to 35)	2 (2 to 2)	586 (533 to 642)	318 (281 to 357)	219 (182 to 261)	45 (26 to 73)	5 (5 to 5)
Peru	2013	299 (270 to 328)	169 (149 to 189)	104 (87 to 124)	24 (14 to 38)	2 (2 to 2)	621 (562 to 683)	351 (311 to 392)	217 (181 to 258)	49 (28 to 79)	5 (5 to 5)
Peru	2014	312 (282 to 343)	184 (163 to 205)	102 (85 to 121)	25 (14 to 40)	1 (1 to 1)	649 (586 to 714)	383 (338 to 426)	212 (176 to 252)	52 (30 to 83)	2 (2 to 2)
Peru	2015	327 (295 to 361)	194 (170 to 216)	99 (81 to 118)	26 (15 to 42)	9 (9 to 9)	681 (615 to 752)	403 (354 to 450)	206 (169 to 246)	55 (31 to 88)	18 (18 to 18)
Peru	2016	322 (287 to 360)	198 (173 to 223)	96 (79 to 117)	27 (15 to 45)	1 (1 to 1)	669 (598 to 749)	411 (360 to 465)	201 (163 to 244)	55 (31 to 93)	2 (2 to 2)
Peru	2017	330 (283 to 384)	206 (177 to 236)	96 (76 to 120)	28 (15 to 46)	1 (1 to 1)	687 (589 to 799)	428 (368 to 492)	200 (159 to 249)	58 (31 to 95)	2 (2 to 2)

Peru	2018	342 (296 to 401)	215 (183 to 247)	98 (78 to 123)	29 (15 to 47)	1 (1 to 1)	712 (616 to 834)	446 (381 to 515)	205 (163 to 255)	59 (32 to 98)	1 (1 to 1)
Peru	2019	350 (299 to 408)	220 (187 to 254)	100 (80 to 125)	29 (16 to 48)	1 (1 to 1)	728 (623 to 849)	458 (390 to 528)	208 (166 to 260)	61 (33 to 100)	1 (1 to 1)
Peru	2020	362 (313 to 424)	229 (195 to 264)	103 (82 to 128)	30 (16 to 50)	1 (1 to 1)	754 (651 to 882)	476 (406 to 550)	214 (170 to 267)	63 (34 to 103)	1 (1 to 2)
Peru	2021	368 (314 to 432)	233 (198 to 270)	104 (83 to 129)	31 (17 to 51)	1 (1 to 1)	766 (653 to 899)	485 (412 to 562)	216 (172 to 269)	64 (35 to 105)	1 (1 to 2)
Peru	2022	374 (323 to 441)	238 (202 to 275)	105 (83 to 131)	31 (17 to 52)	1 (1 to 1)	779 (672 to 918)	495 (420 to 571)	218 (174 to 272)	65 (35 to 107)	1 (1 to 2)
Peru	2023	381 (324 to 448)	243 (205 to 283)	106 (84 to 132)	32 (17 to 53)	1 (1 to 1)	792 (673 to 932)	505 (427 to 589)	220 (175 to 274)	66 (36 to 109)	1 (1 to 2)
Peru	2024	387 (331 to 454)	248 (208 to 287)	107 (85 to 133)	32 (18 to 54)	1 (1 to 1)	806 (689 to 945)	516 (434 to 596)	222 (177 to 276)	67 (36 to 111)	1 (1 to 2)
Peru	2025	394 (334 to 463)	253 (212 to 293)	108 (86 to 134)	33 (18 to 55)	1 (1 to 1)	820 (694 to 964)	526 (441 to 610)	224 (178 to 279)	69 (37 to 114)	1 (1 to 2)
Peru	2026	401 (343 to 471)	258 (216 to 301)	109 (86 to 135)	34 (18 to 56)	1 (1 to 1)	835 (714 to 981)	537 (450 to 627)	226 (180 to 282)	70 (38 to 116)	2 (1 to 2)
Peru	2027	408 (345 to 480)	264 (222 to 309)	110 (87 to 137)	34 (19 to 57)	1 (1 to 1)	850 (717 to 999)	549 (461 to 642)	228 (181 to 285)	71 (39 to 118)	2 (1 to 2)
Peru	2028	416 (353 to 489)	270 (228 to 316)	111 (88 to 138)	35 (19 to 58)	1 (1 to 1)	866 (734 to 1018)	561 (474 to 658)	231 (183 to 288)	72 (39 to 120)	2 (1 to 2)
Peru	2029	425 (358 to 505)	276 (233 to 325)	112 (89 to 140)	36 (19 to 59)	1 (1 to 1)	884 (744 to 1050)	575 (484 to 676)	234 (185 to 292)	74 (40 to 123)	2 (1 to 2)
Peru	2030	434 (369 to 514)	283 (238 to 335)	114 (90 to 142)	36 (19 to 60)	1 (1 to 1)	903 (768 to 1069)	589 (494 to 696)	237 (188 to 296)	75 (40 to 125)	2 (1 to 2)
Peru	2031	443 (374 to 529)	290 (243 to 346)	115 (91 to 144)	37 (20 to 62)	1 (1 to 1)	922 (779 to 1100)	604 (507 to 719)	240 (190 to 300)	77 (41 to 128)	2 (1 to 2)
Peru	2032	453 (387 to 540)	298 (249 to 354)	117 (93 to 146)	38 (20 to 63)	1 (1 to 1)	943 (806 to 1124)	619 (518 to 737)	243 (193 to 304)	79 (42 to 131)	2 (1 to 2)

Peru	2033	464 (390 to 554)	306 (255 to 363)	119 (94 to 148)	39 (21 to 64)	1 (1 to 1)	965 (812 to 1153)	636 (531 to 756)	247 (196 to 309)	81 (43 to 134)	2 (1 to 2)
Peru	2034	475 (403 to 565)	314 (261 to 371)	120 (96 to 151)	40 (21 to 66)	1 (1 to 1)	988 (839 to 1175)	653 (543 to 773)	251 (199 to 314)	83 (44 to 138)	2 (1 to 2)
Peru	2035	486 (407 to 582)	322 (267 to 386)	122 (97 to 153)	41 (22 to 68)	1 (1 to 1)	1011 (846 to 1212)	671 (556 to 803)	254 (202 to 319)	84 (45 to 141)	2 (1 to 2)
Peru	2036	497 (424 to 596)	331 (275 to 396)	124 (98 to 156)	42 (22 to 69)	1 (1 to 1)	1035 (881 to 1241)	688 (571 to 824)	258 (205 to 324)	86 (46 to 145)	2 (1 to 2)
Peru	2037	509 (422 to 611)	340 (280 to 406)	126 (100 to 158)	43 (23 to 71)	1 (1 to 1)	1060 (879 to 1272)	707 (583 to 845)	262 (208 to 329)	89 (47 to 148)	2 (1 to 2)
Peru	2038	521 (440 to 621)	349 (287 to 421)	128 (102 to 161)	44 (23 to 73)	1 (1 to 1)	1085 (915 to 1293)	726 (597 to 876)	266 (211 to 334)	91 (48 to 151)	2 (1 to 2)
Peru	2039	534 (443 to 640)	358 (295 to 431)	130 (103 to 163)	45 (24 to 75)	1 (1 to 1)	1110 (921 to 1332)	745 (615 to 897)	270 (214 to 340)	93 (49 to 155)	2 (1 to 2)
Peru	2040	546 (460 to 654)	367 (301 to 445)	132 (104 to 166)	46 (24 to 76)	1 (1 to 1)	1135 (958 to 1360)	764 (627 to 926)	274 (217 to 346)	95 (50 to 159)	2 (1 to 2)
Peru	2041	558 (461 to 673)	376 (308 to 458)	134 (106 to 169)	47 (25 to 78)	1 (1 to 1)	1161 (960 to 1399)	783 (642 to 953)	278 (220 to 351)	97 (51 to 163)	2 (1 to 2)
Peru	2042	570 (478 to 684)	386 (313 to 468)	136 (107 to 171)	48 (25 to 80)	1 (1 to 1)	1186 (995 to 1423)	803 (650 to 974)	282 (223 to 356)	99 (52 to 166)	2 (1 to 2)
Peru	2043	582 (479 to 704)	395 (319 to 478)	138 (109 to 173)	49 (26 to 82)	1 (1 to 1)	1212 (996 to 1464)	822 (663 to 995)	286 (226 to 361)	102 (54 to 170)	2 (1 to 2)

Peru	2044	595 (499 to 712)	405 (327 to 489)	139 (110 to 176)	50 (26 to 84)	1 (1 to 1)	1238 (1038 to 1482)	842 (680 to 1017)	290 (230 to 367)	104 (55 to 174)	2 (1 to 2)
Peru	2045	607 (497 to 734)	414 (333 to 500)	141 (112 to 179)	51 (27 to 86)	1 (1 to 1)	1263 (1035 to 1527)	861 (692 to 1040)	294 (233 to 372)	106 (56 to 178)	2 (1 to 2)
Peru	2046	619 (514 to 745)	423 (340 to 511)	143 (113 to 181)	52 (27 to 87)	1 (1 to 1)	1288 (1070 to 1551)	881 (707 to 1064)	298 (236 to 377)	108 (57 to 182)	2 (1 to 2)
Peru	2047	632 (517 to 766)	433 (349 to 527)	145 (114 to 184)	53 (28 to 89)	1 (1 to 1)	1314 (1075 to 1595)	901 (727 to 1097)	301 (238 to 382)	110 (58 to 186)	2 (1 to 2)
Peru	2048	644 (533 to 778)	442 (357 to 535)	147 (116 to 186)	54 (28 to 91)	1 (1 to 1)	1340 (1110 to 1618)	921 (742 to 1114)	305 (241 to 386)	113 (59 to 190)	2 (1 to 2)
Peru	2049	657 (535 to 799)	452 (366 to 550)	148 (117 to 188)	55 (29 to 93)	1 (1 to 1)	1366 (1113 to 1663)	940 (761 to 1143)	309 (244 to 391)	115 (61 to 194)	2 (1 to 2)
Peru	2050	669 (555 to 808)	462 (373 to 560)	150 (119 to 190)	56 (30 to 95)	1 (0 to 1)	1393 (1155 to 1680)	961 (776 to 1165)	313 (247 to 396)	117 (62 to 198)	2 (0 to 3)
Philippines	1995	52 (43 to 63)	20 (15 to 25)	26 (19 to 35)	4 (2 to 9)	2 (2 to 2)	147 (121 to 178)	55 (42 to 71)	73 (53 to 97)	13 (6 to 24)	6 (6 to 6)
Philippines	1996	53 (44 to 64)	21 (16 to 27)	26 (19 to 35)	5 (2 to 9)	1 (1 to 1)	149 (123 to 179)	59 (45 to 75)	74 (55 to 97)	14 (7 to 26)	2 (2 to 2)
Philippines	1997	54 (44 to 64)	22 (17 to 28)	26 (19 to 34)	5 (3 to 10)	1 (1 to 1)	151 (124 to 181)	61 (47 to 78)	73 (54 to 95)	15 (7 to 28)	2 (2 to 2)
Philippines	1998	52 (42 to 61)	21 (16 to 27)	24 (18 to 32)	5 (3 to 10)	1 (1 to 1)	145 (119 to 172)	58 (45 to 75)	68 (50 to 89)	15 (7 to 28)	3 (3 to 3)
Philippines	1999	52 (42 to 62)	21 (16 to 27)	24 (17 to 31)	6 (3 to 11)	1 (1 to 1)	146 (119 to 173)	59 (44 to 75)	66 (49 to 87)	16 (8 to 30)	4 (4 to 4)

Philippines	2000	52 (43 to 62)	21 (16 to 27)	23 (18 to 31)	6 (3 to 11)	1 (1 to 1)	146 (121 to 174)	59 (45 to 75)	66 (49 to 87)	17 (8 to 31)	3 (3 to 3)
Philippines	2001	52 (43 to 62)	20 (16 to 26)	24 (18 to 31)	6 (3 to 11)	1 (1 to 1)	145 (120 to 174)	58 (45 to 73)	67 (50 to 88)	17 (8 to 32)	4 (4 to 4)
Philippines	2002	51 (43 to 62)	20 (15 to 25)	25 (19 to 32)	6 (3 to 12)	1 (1 to 1)	145 (120 to 173)	56 (43 to 71)	69 (52 to 91)	18 (9 to 33)	2 (2 to 2)
Philippines	2003	56 (46 to 68)	21 (16 to 26)	27 (21 to 36)	7 (3 to 12)	1 (1 to 1)	158 (130 to 190)	58 (45 to 74)	77 (58 to 101)	20 (10 to 35)	3 (3 to 3)
Philippines	2004	60 (50 to 73)	21 (17 to 27)	30 (23 to 40)	7 (4 to 13)	1 (1 to 1)	169 (140 to 205)	60 (47 to 76)	85 (64 to 111)	21 (10 to 37)	3 (3 to 3)
Philippines	2005	67 (56 to 81)	22 (17 to 29)	35 (26 to 45)	8 (4 to 14)	2 (2 to 2)	190 (157 to 229)	63 (49 to 81)	98 (73 to 127)	22 (11 to 40)	7 (7 to 7)
Philippines	2006	74 (61 to 89)	24 (18 to 31)	39 (29 to 51)	8 (4 to 15)	2 (2 to 2)	207 (171 to 250)	66 (52 to 87)	111 (83 to 143)	23 (11 to 43)	7 (7 to 7)
Philippines	2007	78 (64 to 94)	25 (19 to 32)	44 (33 to 56)	9 (4 to 16)	1 (1 to 1)	220 (181 to 264)	69 (54 to 90)	122 (92 to 158)	25 (13 to 46)	3 (3 to 3)
Philippines	2008	82 (68 to 98)	25 (19 to 33)	46 (35 to 60)	10 (5 to 17)	1 (1 to 1)	231 (191 to 277)	71 (55 to 92)	131 (99 to 168)	27 (14 to 47)	3 (3 to 3)
Philippines	2009	89 (74 to 107)	27 (21 to 35)	50 (39 to 64)	11 (6 to 19)	1 (1 to 1)	251 (208 to 300)	76 (59 to 98)	141 (108 to 180)	30 (16 to 55)	4 (4 to 4)
Philippines	2010	94 (78 to 111)	28 (21 to 36)	53 (41 to 67)	12 (6 to 21)	2 (2 to 2)	265 (218 to 313)	77 (60 to 101)	149 (116 to 188)	33 (17 to 58)	5 (5 to 5)
Philippines	2011	95 (79 to 113)	26 (20 to 35)	54 (43 to 68)	13 (6 to 22)	2 (2 to 2)	268 (223 to 317)	74 (58 to 97)	153 (120 to 191)	35 (18 to 61)	5 (5 to 5)
Philippines	2012	101 (85 to 120)	28 (22 to 37)	57 (45 to 72)	14 (7 to 24)	2 (2 to 2)	285 (238 to 338)	79 (61 to 104)	162 (128 to 203)	40 (21 to 67)	5 (5 to 5)
Philippines	2013	106 (89 to 127)	29 (22 to 39)	60 (47 to 74)	15 (8 to 25)	2 (2 to 2)	298 (250 to 356)	83 (63 to 109)	168 (132 to 208)	42 (22 to 70)	5 (5 to 5)
Philippines	2014	109 (90 to 129)	31 (23 to 41)	61 (47 to 76)	15 (8 to 26)	2 (2 to 2)	306 (255 to 363)	87 (66 to 115)	171 (134 to 214)	43 (23 to 73)	5 (5 to 5)

Philippines	2015	118 (98 to 139)	35 (27 to 46)	64 (50 to 81)	16 (9 to 27)	3 (3 to 3)	331 (276 to 391)	97 (75 to 129)	181 (140 to 228)	45 (24 to 77)	7 (7 to 7)
Philippines	2016	124 (103 to 149)	38 (30 to 51)	68 (52 to 87)	17 (9 to 30)	1 (1 to 1)	350 (289 to 419)	108 (84 to 143)	191 (146 to 244)	48 (25 to 83)	3 (3 to 3)
Philippines	2017	133 (107 to 168)	41 (32 to 55)	71 (54 to 91)	18 (9 to 32)	3 (3 to 3)	374 (300 to 472)	117 (90 to 155)	200 (153 to 256)	51 (26 to 90)	7 (7 to 7)
Philippines	2018	143 (116 to 179)	47 (36 to 62)	74 (57 to 95)	20 (10 to 35)	2 (2 to 2)	404 (325 to 503)	132 (102 to 175)	209 (160 to 268)	56 (29 to 99)	7 (7 to 7)
Philippines	2019	148 (118 to 184)	48 (37 to 64)	77 (59 to 99)	20 (11 to 36)	2 (2 to 2)	417 (331 to 519)	135 (105 to 179)	217 (166 to 278)	57 (30 to 102)	7 (7 to 7)
Philippines	2020	156 (126 to 196)	51 (40 to 68)	80 (61 to 103)	22 (11 to 38)	2 (2 to 3)	438 (355 to 551)	144 (112 to 191)	226 (173 to 290)	61 (32 to 107)	7 (7 to 7)
Philippines	2021	160 (128 to 199)	53 (41 to 71)	82 (63 to 105)	22 (12 to 39)	2 (2 to 3)	450 (361 to 561)	149 (116 to 198)	231 (177 to 296)	62 (33 to 110)	7 (7 to 7)
Philippines	2022	164 (132 to 204)	55 (43 to 73)	84 (64 to 108)	23 (12 to 40)	2 (2 to 3)	462 (371 to 575)	155 (120 to 205)	236 (180 to 303)	64 (33 to 113)	7 (6 to 8)
Philippines	2023	168 (134 to 211)	57 (44 to 76)	86 (65 to 110)	23 (12 to 41)	2 (2 to 3)	474 (378 to 593)	160 (125 to 214)	241 (184 to 309)	66 (34 to 117)	7 (6 to 8)
Philippines	2024	173 (140 to 216)	59 (46 to 79)	87 (67 to 112)	24 (13 to 43)	2 (2 to 3)	487 (393 to 609)	166 (129 to 222)	246 (187 to 315)	68 (35 to 120)	7 (6 to 8)
Philippines	2025	178 (141 to 222)	61 (48 to 82)	89 (68 to 114)	25 (13 to 44)	2 (2 to 3)	500 (398 to 626)	173 (134 to 230)	251 (191 to 322)	70 (36 to 123)	7 (6 to 8)
Philippines	2026	182 (147 to 227)	64 (49 to 85)	91 (69 to 117)	26 (13 to 45)	3 (2 to 3)	513 (414 to 638)	179 (139 to 239)	256 (195 to 328)	72 (37 to 127)	7 (6 to 8)
Philippines	2027	187 (149 to 235)	66 (51 to 88)	93 (71 to 119)	26 (14 to 47)	3 (2 to 3)	527 (418 to 662)	185 (144 to 247)	261 (199 to 335)	74 (39 to 131)	7 (6 to 8)
Philippines	2028	193 (156 to 241)	68 (53 to 91)	95 (72 to 122)	27 (14 to 48)	3 (2 to 3)	542 (438 to 679)	192 (149 to 256)	266 (203 to 342)	76 (40 to 135)	7 (6 to 8)
Philippines	2029	198 (157 to 249)	71 (55 to 94)	97 (74 to 124)	28 (15 to 50)	3 (2 to 3)	557 (443 to 702)	199 (154 to 266)	272 (207 to 349)	79 (41 to 140)	7 (6 to 8)

Philippines	2030	204 (165 to 256)	73 (57 to 98)	99 (75 to 127)	29 (15 to 51)	3 (2 to 3)	573 (463 to 719)	207 (160 to 276)	278 (212 to 357)	81 (42 to 145)	7 (6 to 9)
Philippines	2031	210 (167 to 265)	76 (59 to 101)	101 (77 to 130)	30 (16 to 53)	3 (2 to 3)	590 (471 to 745)	214 (167 to 285)	284 (216 to 365)	84 (44 to 149)	7 (6 to 9)
Philippines	2032	216 (175 to 272)	79 (61 to 105)	103 (79 to 133)	31 (16 to 55)	3 (2 to 3)	607 (492 to 766)	222 (172 to 295)	290 (221 to 373)	87 (45 to 154)	7 (6 to 9)
Philippines	2033	222 (177 to 281)	82 (63 to 109)	106 (80 to 135)	32 (17 to 56)	3 (2 to 3)	624 (499 to 789)	231 (178 to 306)	297 (226 to 381)	90 (47 to 159)	7 (6 to 9)
Philippines	2034	229 (185 to 288)	85 (65 to 113)	108 (82 to 138)	33 (17 to 58)	3 (2 to 3)	643 (520 to 811)	239 (184 to 319)	304 (231 to 389)	93 (48 to 164)	7 (6 to 9)
Philippines	2035	235 (189 to 298)	88 (68 to 118)	110 (84 to 141)	34 (18 to 60)	3 (2 to 3)	662 (533 to 838)	249 (191 to 331)	311 (236 to 397)	96 (50 to 169)	7 (6 to 9)
Philippines	2036	242 (194 to 309)	92 (70 to 122)	113 (86 to 144)	35 (18 to 62)	3 (2 to 3)	682 (547 to 871)	258 (198 to 344)	318 (242 to 406)	99 (51 to 175)	7 (6 to 9)
Philippines	2037	250 (200 to 316)	95 (73 to 127)	116 (88 to 148)	36 (19 to 64)	3 (2 to 3)	702 (562 to 888)	268 (205 to 356)	325 (247 to 415)	102 (53 to 181)	7 (6 to 9)
Philippines	2038	257 (206 to 327)	99 (75 to 131)	118 (90 to 151)	37 (19 to 66)	3 (2 to 3)	723 (580 to 920)	277 (212 to 370)	333 (253 to 425)	105 (55 to 187)	7 (6 to 9)
Philippines	2039	265 (212 to 336)	102 (78 to 136)	121 (92 to 155)	39 (20 to 69)	3 (2 to 3)	744 (597 to 945)	287 (220 to 382)	341 (259 to 436)	109 (57 to 193)	7 (6 to 9)
Philippines	2040	272 (219 to 348)	106 (81 to 141)	124 (94 to 158)	40 (21 to 71)	3 (2 to 3)	766 (617 to 980)	298 (227 to 397)	349 (265 to 445)	112 (58 to 199)	8 (6 to 10)
Philippines	2041	280 (225 to 356)	110 (84 to 146)	127 (96 to 162)	41 (21 to 73)	3 (2 to 3)	789 (634 to 1000)	309 (236 to 411)	357 (271 to 455)	116 (60 to 204)	8 (6 to 10)
Philippines	2042	289 (233 to 370)	114 (87 to 151)	130 (99 to 166)	43 (22 to 75)	3 (2 to 3)	813 (656 to 1041)	320 (244 to 426)	365 (278 to 466)	120 (62 to 212)	8 (6 to 10)
Philippines	2043	297 (237 to 379)	118 (89 to 157)	133 (101 to 170)	44 (23 to 78)	3 (2 to 4)	836 (666 to 1067)	331 (252 to 442)	374 (284 to 478)	124 (64 to 219)	8 (6 to 10)
Philippines	2044	306 (245 to 386)	122 (92 to 163)	136 (103 to 174)	45 (23 to 80)	3 (2 to 4)	861 (689 to 1087)	344 (260 to 458)	382 (291 to 489)	128 (66 to 226)	8 (6 to 10)

Philippines	2045	315 (251 to 405)	127 (96 to 169)	139 (105 to 178)	47 (24 to 83)	3 (2 to 4)	887 (705 to 1140)	356 (270 to 475)	391 (297 to 501)	132 (68 to 234)	8 (6 to 10)
Philippines	2046	324 (260 to 414)	131 (99 to 175)	142 (108 to 182)	48 (25 to 86)	3 (2 to 4)	912 (731 to 1165)	369 (280 to 492)	400 (303 to 512)	136 (70 to 241)	8 (6 to 10)
Philippines	2047	333 (266 to 425)	136 (103 to 180)	145 (110 to 186)	50 (26 to 88)	3 (2 to 4)	938 (748 to 1195)	382 (289 to 508)	408 (309 to 524)	140 (72 to 248)	8 (6 to 10)
Philippines	2048	343 (274 to 437)	140 (106 to 187)	148 (112 to 191)	51 (26 to 90)	3 (2 to 4)	965 (771 to 1230)	395 (298 to 526)	417 (316 to 536)	145 (75 to 254)	8 (6 to 10)
Philippines	2049	352 (278 to 449)	145 (109 to 193)	151 (114 to 195)	53 (27 to 93)	3 (2 to 4)	991 (783 to 1263)	408 (308 to 543)	426 (322 to 548)	149 (77 to 261)	8 (6 to 10)
Philippines	2050	362 (288 to 461)	150 (113 to 200)	155 (117 to 199)	55 (28 to 95)	3 (2 to 4)	1018 (809 to 1297)	422 (318 to 562)	435 (329 to 561)	154 (79 to 268)	8 (6 to 11)
Poland	1995	305 (277 to 335)	211 (189 to 235)	91 (75 to 110)	3 (1 to 5)	0 (0 to 0)	693 (629 to 760)	478 (430 to 533)	208 (170 to 249)	7 (3 to 12)	1 (1 to 1)
Poland	1996	337 (311 to 366)	233 (212 to 255)	100 (86 to 119)	3 (2 to 6)	1 (1 to 1)	765 (707 to 831)	528 (482 to 578)	228 (194 to 270)	7 (4 to 13)	1 (1 to 1)
Poland	1997	350 (327 to 376)	238 (220 to 257)	107 (93 to 126)	3 (2 to 6)	1 (1 to 1)	794 (742 to 853)	540 (500 to 585)	244 (210 to 286)	7 (4 to 13)	2 (2 to 2)
Poland	1998	366 (344 to 392)	246 (228 to 265)	117 (101 to 135)	3 (2 to 6)	0 (0 to 0)	832 (781 to 890)	559 (519 to 601)	265 (229 to 306)	8 (4 to 13)	0 (0 to 0)
Poland	1999	383 (359 to 409)	260 (242 to 278)	119 (103 to 136)	3 (2 to 6)	0 (0 to 0)	869 (816 to 929)	590 (549 to 632)	271 (234 to 310)	8 (4 to 14)	0 (0 to 0)
Poland	2000	399 (376 to 425)	273 (255 to 294)	122 (106 to 140)	4 (2 to 6)	0 (0 to 0)	907 (854 to 966)	621 (580 to 667)	278 (242 to 317)	8 (4 to 14)	0 (0 to 0)
Poland	2001	430 (406 to 456)	297 (278 to 318)	127 (110 to 144)	5 (3 to 9)	1 (1 to 1)	976 (921 to 1036)	675 (632 to 722)	288 (250 to 326)	12 (6 to 20)	1 (1 to 1)
Poland	2002	452 (426 to 478)	312 (292 to 334)	129 (112 to 146)	10 (5 to 16)	0 (0 to 0)	1026 (968 to 1086)	709 (663 to 758)	294 (255 to 331)	22 (12 to 35)	1 (1 to 1)

Poland	2003	469 (443 to 497)	322 (302 to 345)	134 (116 to 151)	13 (8 to 20)	0 (0 to 0)	1064 (1006 to 1128)	732 (686 to 784)	303 (264 to 344)	29 (17 to 45)	0 (0 to 0)
Poland	2004	481 (454 to 510)	328 (308 to 349)	138 (120 to 156)	15 (9 to 22)	0 (0 to 0)	1092 (1030 to 1157)	746 (700 to 793)	313 (273 to 353)	33 (20 to 50)	0 (0 to 0)
Poland	2005	506 (477 to 537)	347 (326 to 368)	141 (125 to 160)	17 (11 to 26)	0 (0 to 0)	1149 (1084 to 1220)	788 (741 to 837)	321 (283 to 364)	39 (25 to 59)	0 (0 to 0)
Poland	2006	541 (512 to 571)	374 (353 to 396)	147 (129 to 166)	20 (13 to 30)	0 (0 to 0)	1229 (1163 to 1298)	850 (801 to 900)	333 (294 to 377)	46 (30 to 67)	0 (0 to 0)
Poland	2007	581 (551 to 613)	406 (384 to 430)	152 (135 to 171)	23 (15 to 33)	0 (0 to 0)	1319 (1252 to 1391)	921 (871 to 976)	346 (307 to 388)	52 (35 to 75)	0 (0 to 0)
Poland	2008	631 (600 to 663)	446 (424 to 471)	159 (142 to 177)	26 (18 to 37)	0 (0 to 0)	1433 (1362 to 1505)	1012 (962 to 1070)	361 (322 to 402)	60 (40 to 84)	0 (0 to 0)
Poland	2009	667 (638 to 699)	475 (453 to 498)	164 (148 to 181)	29 (20 to 40)	0 (0 to 0)	1516 (1450 to 1587)	1078 (1030 to 1132)	372 (335 to 410)	66 (45 to 90)	0 (0 to 0)
Poland	2010	697 (667 to 728)	497 (476 to 519)	167 (152 to 184)	33 (24 to 45)	0 (0 to 0)	1584 (1514 to 1654)	1129 (1081 to 1178)	380 (345 to 417)	75 (53 to 101)	0 (0 to 0)
Poland	2011	707 (678 to 738)	501 (481 to 523)	169 (154 to 187)	37 (27 to 48)	0 (0 to 0)	1606 (1541 to 1675)	1138 (1092 to 1189)	385 (350 to 424)	84 (62 to 109)	0 (0 to 0)
Poland	2012	718 (691 to 747)	505 (485 to 527)	172 (156 to 189)	41 (31 to 53)	0 (0 to 0)	1631 (1570 to 1696)	1147 (1102 to 1196)	390 (354 to 430)	94 (71 to 120)	0 (0 to 0)

Poland	2013	736 (710 to 764)	515 (495 to 536)	175 (159 to 192)	46 (36 to 59)	0 (0 to 0)	1672 (1612 to 1736)	1171 (1124 to 1218)	397 (362 to 436)	104 (81 to 134)	0 (0 to 0)
Poland	2014	762 (735 to 793)	531 (510 to 552)	178 (162 to 196)	52 (40 to 67)	0 (0 to 0)	1730 (1670 to 1800)	1206 (1159 to 1254)	405 (369 to 444)	119 (92 to 152)	0 (0 to 0)
Poland	2015	796 (766 to 829)	552 (529 to 575)	185 (169 to 204)	59 (45 to 75)	0 (0 to 0)	1808 (1739 to 1882)	1254 (1201 to 1305)	421 (383 to 463)	133 (102 to 171)	0 (0 to 0)
Poland	2016	830 (793 to 865)	573 (548 to 596)	192 (174 to 212)	65 (49 to 86)	0 (0 to 0)	1884 (1802 to 1965)	1300 (1244 to 1354)	437 (395 to 481)	147 (112 to 195)	0 (0 to 0)
Poland	2017	882 (827 to 945)	609 (576 to 644)	202 (179 to 229)	71 (51 to 96)	0 (0 to 0)	2003 (1879 to 2145)	1382 (1308 to 1462)	459 (407 to 519)	161 (117 to 219)	0 (0 to 0)
Poland	2018	936 (872 to 1005)	648 (613 to 686)	213 (189 to 240)	75 (55 to 102)	0 (0 to 0)	2126 (1980 to 2281)	1472 (1392 to 1558)	483 (429 to 545)	170 (124 to 232)	0 (0 to 0)
Poland	2019	996 (932 to 1066)	696 (656 to 737)	221 (196 to 249)	80 (58 to 108)	0 (0 to 0)	2262 (2116 to 2421)	1580 (1490 to 1674)	502 (445 to 566)	181 (132 to 246)	0 (0 to 0)
Poland	2020	1034 (961 to 1110)	723 (682 to 765)	228 (202 to 257)	83 (60 to 112)	0 (0 to 0)	2348 (2183 to 2522)	1643 (1549 to 1738)	518 (460 to 584)	188 (137 to 254)	0 (0 to 0)
Poland	2021	1058 (990 to 1134)	742 (700 to 785)	232 (206 to 261)	84 (62 to 115)	0 (0 to 0)	2404 (2248 to 2574)	1686 (1589 to 1784)	526 (468 to 594)	192 (140 to 260)	0 (0 to 0)
Poland	2022	1084 (1007 to 1165)	762 (717 to 809)	235 (209 to 265)	87 (63 to 117)	0 (0 to 0)	2462 (2287 to 2647)	1731 (1627 to 1836)	535 (476 to 602)	196 (143 to 266)	0 (0 to 0)

Poland	2023	1115 (1040 to 1200)	787 (740 to 836)	239 (213 to 269)	89 (65 to 121)	0 (0 to 0)	2532 (2362 to 2726)	1788 (1681 to 1899)	543 (483 to 611)	202 (147 to 274)	0 (0 to 0)
Poland	2024	1148 (1064 to 1235)	814 (766 to 864)	243 (216 to 273)	91 (67 to 124)	0 (0 to 0)	2607 (2416 to 2805)	1848 (1739 to 1962)	552 (490 to 621)	208 (151 to 281)	0 (0 to 0)
Poland	2025	1182 (1101 to 1275)	841 (791 to 895)	247 (219 to 278)	94 (69 to 127)	0 (0 to 0)	2684 (2500 to 2895)	1910 (1797 to 2033)	561 (498 to 632)	213 (156 to 289)	0 (0 to 0)
Poland	2026	1217 (1130 to 1313)	869 (816 to 925)	251 (223 to 283)	97 (70 to 131)	0 (0 to 0)	2765 (2566 to 2981)	1974 (1853 to 2101)	571 (506 to 643)	220 (160 to 297)	0 (0 to 0)
Poland	2027	1254 (1167 to 1356)	899 (841 to 958)	256 (227 to 289)	100 (73 to 135)	0 (0 to 0)	2849 (2650 to 3079)	2041 (1911 to 2176)	582 (516 to 656)	226 (165 to 306)	0 (0 to 0)
Poland	2028	1294 (1198 to 1403)	930 (870 to 993)	262 (232 to 295)	103 (74 to 139)	0 (0 to 0)	2940 (2721 to 3187)	2112 (1976 to 2255)	595 (526 to 670)	233 (169 to 316)	0 (0 to 0)
Poland	2029	1336 (1239 to 1455)	962 (897 to 1031)	268 (237 to 302)	106 (77 to 144)	0 (0 to 0)	3035 (2814 to 3304)	2186 (2038 to 2342)	609 (538 to 686)	241 (174 to 326)	0 (0 to 0)
Poland	2030	1381 (1274 to 1498)	996 (928 to 1073)	275 (243 to 310)	109 (79 to 149)	0 (0 to 0)	3135 (2894 to 3402)	2263 (2108 to 2436)	624 (551 to 704)	249 (180 to 337)	0 (0 to 0)
Poland	2031	1426 (1313 to 1559)	1031 (957 to 1113)	282 (248 to 318)	113 (82 to 153)	0 (0 to 0)	3238 (2982 to 3540)	2342 (2173 to 2527)	639 (564 to 722)	257 (186 to 348)	0 (0 to 0)
Poland	2032	1472 (1352 to 1600)	1067 (990 to 1153)	288 (254 to 326)	117 (85 to 158)	0 (0 to 0)	3343 (3070 to 3633)	2423 (2247 to 2619)	655 (577 to 741)	265 (192 to 359)	0 (0 to 0)

Poland	2033	1518 (1392 to 1662)	1103 (1018 to 1194)	295 (260 to 334)	120 (87 to 163)	0 (0 to 0)	3447 (3162 to 3775)	2504 (2312 to 2711)	670 (591 to 759)	273 (197 to 371)	0 (0 to 0)
Poland	2034	1564 (1432 to 1708)	1138 (1047 to 1235)	302 (266 to 342)	124 (89 to 168)	0 (0 to 0)	3553 (3253 to 3880)	2586 (2379 to 2806)	686 (604 to 777)	281 (203 to 382)	0 (0 to 0)
Poland	2035	1612 (1470 to 1770)	1176 (1080 to 1278)	309 (272 to 351)	128 (92 to 173)	0 (0 to 0)	3662 (3340 to 4019)	2670 (2452 to 2902)	702 (617 to 796)	290 (209 to 394)	0 (0 to 0)
Poland	2036	1660 (1509 to 1821)	1213 (1110 to 1323)	316 (277 to 359)	131 (95 to 179)	0 (0 to 0)	3770 (3427 to 4135)	2754 (2521 to 3005)	717 (630 to 815)	299 (216 to 406)	0 (0 to 0)
Poland	2037	1709 (1557 to 1890)	1251 (1145 to 1371)	323 (283 to 367)	135 (98 to 184)	0 (0 to 0)	3882 (3537 to 4292)	2842 (2600 to 3114)	733 (643 to 833)	307 (223 to 418)	0 (0 to 0)
Poland	2038	1759 (1598 to 1937)	1291 (1178 to 1417)	329 (289 to 375)	139 (101 to 189)	0 (0 to 0)	3994 (3629 to 4399)	2931 (2675 to 3218)	747 (656 to 851)	316 (228 to 430)	0 (0 to 0)
Poland	2039	1809 (1645 to 2005)	1331 (1212 to 1461)	335 (294 to 381)	143 (103 to 195)	0 (0 to 0)	4108 (3735 to 4554)	3022 (2753 to 3319)	761 (669 to 866)	325 (234 to 442)	0 (0 to 0)
Poland	2040	1860 (1674 to 2059)	1372 (1246 to 1512)	341 (299 to 388)	147 (106 to 200)	0 (0 to 0)	4223 (3802 to 4677)	3115 (2830 to 3435)	774 (680 to 881)	334 (241 to 455)	0 (0 to 0)
Poland	2041	1911 (1727 to 2119)	1414 (1285 to 1559)	347 (305 to 395)	151 (109 to 206)	0 (0 to 0)	4341 (3923 to 4813)	3210 (2917 to 3540)	787 (692 to 897)	343 (247 to 467)	0 (0 to 0)
Poland	2042	1963 (1769 to 2184)	1456 (1317 to 1610)	352 (310 to 402)	155 (111 to 211)	0 (0 to 0)	4458 (4017 to 4960)	3306 (2992 to 3658)	800 (703 to 913)	352 (252 to 480)	0 (0 to 0)

Poland	2043	2014 (1814 to 2238)	1498 (1353 to 1661)	358 (314 to 409)	159 (113 to 217)	0 (0 to 0)	4575 (4120 to 5083)	3402 (3073 to 3771)	813 (713 to 928)	360 (257 to 492)	0 (0 to 0)
Poland	2044	2066 (1858 to 2301)	1540 (1389 to 1716)	363 (318 to 415)	163 (116 to 222)	0 (0 to 0)	4692 (4219 to 5227)	3498 (3155 to 3898)	824 (723 to 943)	369 (263 to 505)	0 (0 to 0)
Poland	2045	2117 (1900 to 2357)	1583 (1421 to 1760)	368 (322 to 421)	166 (119 to 228)	0 (0 to 0)	4808 (4316 to 5353)	3595 (3228 to 3996)	835 (732 to 957)	378 (269 to 517)	0 (0 to 0)
Poland	2046	2169 (1950 to 2421)	1626 (1463 to 1813)	372 (326 to 428)	170 (121 to 233)	0 (0 to 0)	4925 (4428 to 5499)	3693 (3323 to 4118)	846 (741 to 971)	387 (275 to 530)	0 (0 to 0)
Poland	2047	2220 (1997 to 2490)	1668 (1500 to 1861)	377 (330 to 433)	174 (123 to 239)	0 (0 to 0)	5041 (4536 to 5655)	3789 (3406 to 4228)	856 (749 to 984)	395 (280 to 542)	0 (0 to 0)
Poland	2048	2271 (2042 to 2544)	1712 (1538 to 1910)	381 (333 to 438)	178 (126 to 244)	0 (0 to 0)	5158 (4639 to 5778)	3888 (3492 to 4338)	866 (757 to 995)	404 (286 to 554)	0 (0 to 0)
Poland	2049	2322 (2083 to 2598)	1755 (1571 to 1963)	385 (336 to 443)	182 (129 to 249)	0 (0 to 0)	5273 (4732 to 5900)	3985 (3569 to 4458)	875 (763 to 1006)	413 (292 to 566)	0 (0 to 0)
Poland	2050	2373 (2127 to 2658)	1798 (1614 to 2006)	389 (339 to 448)	185 (131 to 255)	0 (0 to 0)	5389 (4831 to 6037)	4083 (3666 to 4555)	884 (770 to 1017)	421 (298 to 578)	0 (0 to 0)
Portugal	1995	1082 (1021 to 1149)	758 (707 to 812)	304 (272 to 337)	19 (12 to 29)	0 (0 to 0)	1572 (1483 to 1669)	1102 (1027 to 1179)	442 (395 to 490)	28 (17 to 43)	0 (0 to 0)
Portugal	1996	1148 (1099 to 1199)	810 (769 to 855)	316 (290 to 341)	22 (14 to 32)	0 (0 to 0)	1667 (1596 to 1741)	1176 (1117 to 1241)	459 (421 to 495)	32 (20 to 46)	0 (0 to 0)

Portugal	1997	1219 (1173 to 1268)	865 (824 to 905)	329 (306 to 354)	26 (17 to 36)	0 (0 to 0)	1770 (1703 to 1842)	1256 (1197 to 1314)	477 (444 to 514)	37 (25 to 52)	0 (0 to 0)
Portugal	1998	1313 (1266 to 1362)	935 (897 to 977)	347 (324 to 372)	31 (22 to 42)	0 (0 to 0)	1907 (1839 to 1979)	1358 (1302 to 1419)	504 (471 to 540)	45 (32 to 61)	0 (0 to 0)
Portugal	1999	1429 (1382 to 1479)	1018 (979 to 1061)	371 (348 to 395)	40 (30 to 51)	0 (0 to 0)	2075 (2007 to 2149)	1479 (1422 to 1541)	538 (506 to 574)	58 (43 to 74)	0 (0 to 0)
Portugal	2000	1577 (1529 to 1629)	1122 (1081 to 1165)	395 (373 to 419)	60 (47 to 75)	0 (0 to 0)	2291 (2221 to 2366)	1630 (1571 to 1692)	574 (541 to 608)	87 (68 to 108)	0 (0 to 0)
Portugal	2001	1685 (1636 to 1735)	1202 (1159 to 1247)	406 (384 to 429)	77 (62 to 94)	0 (0 to 0)	2448 (2376 to 2520)	1747 (1684 to 1812)	589 (558 to 623)	112 (90 to 137)	0 (0 to 0)
Portugal	2002	1736 (1689 to 1785)	1241 (1200 to 1285)	405 (384 to 427)	89 (74 to 107)	0 (0 to 0)	2521 (2454 to 2593)	1803 (1743 to 1866)	588 (557 to 621)	130 (107 to 156)	0 (0 to 0)
Portugal	2003	1789 (1743 to 1844)	1275 (1234 to 1318)	414 (392 to 435)	100 (85 to 119)	0 (0 to 0)	2599 (2531 to 2678)	1853 (1792 to 1914)	601 (570 to 632)	146 (123 to 173)	0 (0 to 0)
Portugal	2004	1846 (1799 to 1899)	1310 (1269 to 1353)	429 (406 to 450)	107 (91 to 126)	0 (0 to 0)	2682 (2614 to 2758)	1903 (1844 to 1965)	623 (590 to 654)	156 (132 to 184)	0 (0 to 0)
Portugal	2005	1903 (1853 to 1956)	1342 (1302 to 1388)	450 (428 to 473)	110 (93 to 130)	0 (0 to 0)	2764 (2692 to 2841)	1950 (1891 to 2015)	654 (621 to 687)	160 (135 to 189)	0 (0 to 0)
Portugal	2006	1899 (1849 to 1951)	1322 (1280 to 1364)	467 (445 to 492)	110 (94 to 129)	0 (0 to 0)	2758 (2686 to 2833)	1920 (1859 to 1982)	679 (647 to 714)	160 (136 to 187)	0 (0 to 0)

Portugal	2007	1919 (1868 to 1973)	1325 (1282 to 1368)	484 (461 to 509)	110 (94 to 129)	0 (0 to 0)	2787 (2714 to 2866)	1924 (1862 to 1987)	704 (670 to 740)	160 (136 to 187)	0 (0 to 0)
Portugal	2008	1941 (1892 to 1993)	1336 (1296 to 1377)	495 (471 to 519)	111 (95 to 132)	0 (0 to 0)	2820 (2748 to 2896)	1940 (1882 to 2000)	719 (685 to 754)	161 (138 to 191)	0 (0 to 0)
Portugal	2009	2019 (1968 to 2073)	1398 (1354 to 1440)	506 (483 to 528)	115 (99 to 136)	0 (0 to 0)	2933 (2858 to 3011)	2030 (1966 to 2092)	735 (702 to 768)	168 (144 to 197)	0 (0 to 0)
Portugal	2010	2043 (1989 to 2099)	1411 (1368 to 1455)	514 (490 to 537)	119 (102 to 140)	0 (0 to 0)	2968 (2889 to 3049)	2049 (1987 to 2113)	746 (712 to 780)	173 (148 to 203)	0 (0 to 0)
Portugal	2011	1926 (1878 to 1977)	1305 (1265 to 1344)	506 (483 to 529)	115 (98 to 137)	0 (0 to 0)	2797 (2728 to 2872)	1896 (1838 to 1953)	735 (702 to 769)	167 (142 to 198)	0 (0 to 0)
Portugal	2012	1807 (1759 to 1855)	1203 (1163 to 1243)	494 (473 to 517)	110 (94 to 131)	0 (0 to 0)	2625 (2555 to 2695)	1747 (1690 to 1805)	718 (686 to 751)	160 (136 to 190)	0 (0 to 0)
Portugal	2013	1778 (1729 to 1829)	1180 (1138 to 1221)	488 (467 to 512)	110 (94 to 132)	0 (0 to 0)	2583 (2511 to 2656)	1714 (1653 to 1773)	709 (678 to 744)	160 (136 to 192)	0 (0 to 0)
Portugal	2014	1811 (1763 to 1863)	1202 (1161 to 1245)	496 (473 to 520)	113 (96 to 134)	0 (0 to 0)	2631 (2560 to 2706)	1746 (1687 to 1809)	721 (686 to 755)	164 (139 to 194)	0 (0 to 0)
Portugal	2015	1805 (1751 to 1854)	1195 (1152 to 1237)	499 (475 to 523)	111 (94 to 133)	0 (0 to 0)	2622 (2543 to 2694)	1735 (1674 to 1796)	725 (691 to 760)	162 (136 to 193)	0 (0 to 0)
Portugal	2016	1800 (1749 to 1853)	1187 (1146 to 1231)	503 (478 to 527)	109 (90 to 134)	0 (0 to 0)	2614 (2540 to 2691)	1725 (1665 to 1788)	731 (695 to 766)	159 (131 to 194)	0 (0 to 0)

Portugal	2017	1889 (1797 to 1988)	1254 (1191 to 1316)	520 (485 to 554)	115 (90 to 145)	0 (0 to 0)	2744 (2610 to 2888)	1821 (1731 to 1912)	756 (705 to 805)	168 (131 to 210)	0 (0 to 0)
Portugal	2018	1904 (1798 to 2012)	1255 (1176 to 1329)	532 (497 to 567)	117 (91 to 146)	0 (0 to 0)	2765 (2611 to 2923)	1822 (1708 to 1931)	773 (721 to 823)	169 (132 to 212)	0 (0 to 0)
Portugal	2019	1947 (1828 to 2074)	1285 (1198 to 1377)	543 (506 to 579)	119 (93 to 150)	0 (0 to 0)	2829 (2655 to 3012)	1867 (1740 to 2001)	788 (735 to 841)	174 (135 to 218)	0 (0 to 0)
Portugal	2020	1989 (1859 to 2130)	1315 (1218 to 1423)	552 (515 to 589)	122 (95 to 153)	0 (0 to 0)	2889 (2700 to 3094)	1910 (1770 to 2067)	802 (749 to 856)	177 (139 to 222)	0 (0 to 0)
Portugal	2021	2015 (1868 to 2165)	1333 (1218 to 1446)	558 (521 to 595)	124 (97 to 155)	0 (0 to 0)	2927 (2714 to 3144)	1937 (1769 to 2100)	810 (757 to 864)	180 (140 to 225)	0 (0 to 0)
Portugal	2022	2040 (1892 to 2197)	1351 (1237 to 1473)	563 (526 to 601)	126 (98 to 158)	0 (0 to 0)	2963 (2747 to 3192)	1962 (1797 to 2139)	818 (764 to 873)	183 (142 to 229)	0 (0 to 0)
Portugal	2023	2056 (1898 to 2222)	1362 (1241 to 1495)	567 (530 to 605)	127 (99 to 160)	0 (0 to 0)	2986 (2757 to 3227)	1978 (1803 to 2171)	823 (769 to 879)	185 (144 to 233)	0 (0 to 0)
Portugal	2024	2069 (1905 to 2245)	1371 (1240 to 1513)	570 (532 to 608)	129 (101 to 162)	0 (0 to 0)	3005 (2767 to 3261)	1991 (1801 to 2198)	828 (773 to 883)	187 (146 to 235)	0 (0 to 0)
Portugal	2025	2081 (1908 to 2275)	1379 (1237 to 1539)	572 (535 to 611)	130 (102 to 164)	0 (0 to 0)	3023 (2771 to 3304)	2003 (1797 to 2236)	831 (777 to 887)	189 (148 to 238)	0 (0 to 0)
Portugal	2026	2092 (1910 to 2296)	1386 (1232 to 1554)	575 (537 to 615)	131 (103 to 166)	0 (0 to 0)	3038 (2774 to 3335)	2012 (1789 to 2257)	835 (781 to 893)	191 (149 to 241)	0 (0 to 0)

Portugal	2027	2100 (1908 to 2313)	1391 (1233 to 1569)	577 (539 to 618)	132 (104 to 167)	0 (0 to 0)	3051 (2771 to 3360)	2020 (1790 to 2279)	838 (783 to 898)	192 (150 to 242)	0 (0 to 0)
Portugal	2028	2109 (1910 to 2337)	1396 (1225 to 1590)	579 (541 to 620)	134 (105 to 169)	0 (0 to 0)	3063 (2775 to 3394)	2028 (1779 to 2310)	841 (786 to 901)	194 (152 to 245)	0 (0 to 0)
Portugal	2029	2118 (1908 to 2351)	1402 (1226 to 1599)	581 (542 to 622)	135 (106 to 170)	0 (0 to 0)	3076 (2772 to 3415)	2037 (1781 to 2322)	844 (787 to 904)	196 (153 to 246)	0 (0 to 0)
Portugal	2030	2127 (1918 to 2371)	1408 (1230 to 1621)	582 (542 to 624)	136 (107 to 171)	0 (0 to 0)	3089 (2785 to 3444)	2046 (1787 to 2355)	846 (788 to 906)	198 (155 to 248)	0 (0 to 0)
Portugal	2031	2136 (1918 to 2379)	1415 (1229 to 1626)	583 (543 to 625)	137 (107 to 172)	0 (0 to 0)	3102 (2786 to 3456)	2056 (1785 to 2362)	847 (789 to 908)	199 (156 to 250)	0 (0 to 0)
Portugal	2032	2147 (1923 to 2401)	1424 (1235 to 1648)	584 (543 to 626)	138 (108 to 173)	0 (0 to 0)	3118 (2793 to 3488)	2068 (1793 to 2394)	849 (789 to 910)	201 (157 to 252)	0 (0 to 0)
Portugal	2033	2153 (1929 to 2417)	1429 (1238 to 1655)	585 (543 to 628)	139 (109 to 175)	0 (0 to 0)	3127 (2803 to 3510)	2076 (1798 to 2404)	849 (788 to 912)	202 (158 to 254)	0 (0 to 0)
Portugal	2034	2161 (1928 to 2443)	1436 (1246 to 1684)	585 (543 to 630)	140 (109 to 176)	0 (0 to 0)	3140 (2800 to 3549)	2086 (1810 to 2446)	850 (789 to 915)	203 (159 to 256)	0 (0 to 0)
Portugal	2035	2171 (1933 to 2449)	1444 (1246 to 1689)	586 (542 to 630)	141 (110 to 178)	0 (0 to 0)	3153 (2808 to 3558)	2098 (1809 to 2453)	851 (788 to 916)	205 (160 to 258)	0 (0 to 0)
Portugal	2036	2178 (1932 to 2472)	1450 (1237 to 1712)	586 (542 to 631)	142 (110 to 178)	0 (0 to 0)	3163 (2807 to 3591)	2106 (1797 to 2487)	852 (787 to 917)	206 (160 to 259)	0 (0 to 0)

Portugal	2037	2184 (1939 to 2483)	1455 (1241 to 1716)	587 (542 to 633)	143 (111 to 179)	0 (0 to 0)	3173 (2817 to 3606)	2113 (1803 to 2493)	852 (788 to 919)	207 (161 to 260)	0 (0 to 0)
Portugal	2038	2189 (1933 to 2492)	1459 (1236 to 1718)	587 (542 to 633)	144 (112 to 181)	0 (0 to 0)	3180 (2808 to 3620)	2119 (1796 to 2496)	852 (788 to 920)	209 (162 to 263)	0 (0 to 0)
Portugal	2039	2194 (1933 to 2508)	1464 (1240 to 1746)	586 (541 to 634)	144 (112 to 182)	0 (0 to 0)	3187 (2808 to 3643)	2126 (1801 to 2537)	852 (786 to 922)	210 (162 to 264)	0 (0 to 0)
Portugal	2040	2198 (1932 to 2516)	1468 (1229 to 1748)	586 (540 to 636)	145 (112 to 183)	0 (0 to 0)	3193 (2806 to 3655)	2132 (1785 to 2540)	851 (785 to 923)	210 (163 to 266)	0 (0 to 0)
Portugal	2041	2199 (1932 to 2524)	1469 (1234 to 1757)	585 (539 to 635)	145 (112 to 184)	0 (0 to 0)	3194 (2806 to 3666)	2134 (1793 to 2552)	849 (783 to 923)	211 (163 to 267)	0 (0 to 0)
Portugal	2042	2203 (1929 to 2541)	1472 (1226 to 1763)	584 (538 to 637)	146 (113 to 185)	0 (0 to 0)	3200 (2802 to 3691)	2139 (1781 to 2561)	849 (781 to 925)	212 (164 to 269)	0 (0 to 0)
Portugal	2043	2207 (1929 to 2549)	1476 (1230 to 1771)	584 (536 to 637)	147 (114 to 186)	0 (0 to 0)	3206 (2802 to 3702)	2144 (1787 to 2572)	848 (779 to 925)	213 (166 to 270)	0 (0 to 0)
Portugal	2044	2214 (1930 to 2548)	1482 (1230 to 1779)	584 (535 to 639)	148 (115 to 187)	0 (0 to 0)	3215 (2803 to 3701)	2152 (1787 to 2584)	848 (778 to 928)	215 (167 to 272)	0 (0 to 0)
Portugal	2045	2223 (1927 to 2563)	1489 (1239 to 1786)	585 (535 to 641)	149 (116 to 189)	0 (0 to 0)	3229 (2799 to 3723)	2163 (1799 to 2594)	849 (777 to 931)	216 (169 to 275)	0 (0 to 0)
Portugal	2046	2235 (1941 to 2588)	1499 (1243 to 1813)	585 (535 to 643)	150 (117 to 192)	0 (0 to 0)	3246 (2820 to 3759)	2178 (1805 to 2633)	850 (776 to 934)	218 (170 to 278)	0 (0 to 0)

Portugal	2047	2246 (1946 to 2597)	1507 (1250 to 1816)	587 (535 to 646)	152 (118 to 194)	0 (0 to 0)	3262 (2827 to 3772)	2190 (1816 to 2638)	852 (777 to 939)	220 (171 to 282)	0 (0 to 0)
Portugal	2048	2262 (1952 to 2618)	1520 (1249 to 1835)	589 (536 to 650)	153 (119 to 197)	0 (0 to 0)	3285 (2835 to 3803)	2207 (1814 to 2666)	855 (779 to 944)	223 (173 to 286)	0 (0 to 0)
Portugal	2049	2278 (1966 to 2654)	1532 (1257 to 1848)	591 (538 to 653)	155 (120 to 200)	0 (0 to 0)	3309 (2856 to 3855)	2225 (1825 to 2685)	858 (781 to 949)	225 (174 to 290)	0 (0 to 0)
Portugal	2050	2299 (1983 to 2669)	1548 (1262 to 1875)	594 (539 to 657)	157 (121 to 202)	0 (0 to 0)	3339 (2880 to 3877)	2249 (1833 to 2723)	862 (783 to 955)	228 (176 to 294)	0 (0 to 0)
Puerto Rico	1995	814 (652 to 1008)	573 (429 to 756)	206 (141 to 288)	35 (17 to 66)	0 (0 to 0)	1028 (823 to 1272)	724 (541 to 955)	260 (177 to 364)	44 (22 to 83)	0 (0 to 0)
Puerto Rico	1996	833 (673 to 1035)	587 (438 to 776)	210 (143 to 292)	36 (17 to 66)	0 (0 to 0)	1052 (849 to 1306)	742 (554 to 980)	265 (181 to 369)	45 (22 to 83)	0 (0 to 0)
Puerto Rico	1997	859 (692 to 1062)	607 (451 to 800)	216 (147 to 303)	36 (18 to 68)	0 (0 to 0)	1084 (874 to 1341)	766 (570 to 1010)	272 (186 to 382)	46 (22 to 85)	0 (0 to 0)
Puerto Rico	1998	872 (701 to 1079)	616 (463 to 815)	219 (149 to 310)	36 (18 to 69)	0 (0 to 0)	1100 (885 to 1362)	778 (584 to 1029)	277 (188 to 391)	45 (23 to 87)	0 (0 to 0)
Puerto Rico	1999	901 (726 to 1105)	638 (483 to 832)	226 (155 to 317)	37 (18 to 69)	0 (0 to 0)	1137 (916 to 1396)	806 (610 to 1050)	285 (195 to 401)	46 (22 to 87)	0 (0 to 0)
Puerto Rico	2000	905 (731 to 1109)	640 (489 to 826)	228 (158 to 319)	36 (17 to 66)	0 (0 to 0)	1142 (922 to 1401)	809 (618 to 1043)	288 (199 to 403)	45 (22 to 83)	0 (0 to 0)

Puerto Rico	2001	947 (765 to 1165)	674 (517 to 865)	237 (166 to 337)	37 (18 to 67)	0 (0 to 0)	1196 (966 to 1472)	851 (653 to 1093)	299 (210 to 426)	47 (22 to 84)	0 (0 to 0)
Puerto Rico	2002	985 (800 to 1204)	702 (543 to 899)	245 (170 to 349)	38 (18 to 69)	0 (0 to 0)	1244 (1010 to 1520)	887 (686 to 1135)	309 (215 to 441)	48 (22 to 88)	0 (0 to 0)
Puerto Rico	2003	1013 (828 to 1231)	722 (560 to 920)	251 (174 to 359)	40 (19 to 74)	0 (0 to 0)	1279 (1046 to 1555)	912 (707 to 1162)	317 (219 to 454)	50 (24 to 93)	0 (0 to 0)
Puerto Rico	2004	1060 (872 to 1276)	756 (599 to 949)	260 (180 to 367)	43 (20 to 80)	0 (0 to 0)	1338 (1101 to 1611)	955 (757 to 1198)	329 (227 to 463)	54 (25 to 101)	0 (0 to 0)
Puerto Rico	2005	1087 (907 to 1297)	776 (623 to 962)	266 (183 to 371)	46 (21 to 84)	0 (0 to 0)	1373 (1145 to 1638)	980 (786 to 1215)	335 (231 to 468)	58 (26 to 107)	0 (0 to 0)
Puerto Rico	2006	1102 (925 to 1295)	786 (638 to 959)	268 (184 to 372)	48 (22 to 87)	0 (0 to 0)	1391 (1168 to 1635)	992 (806 to 1211)	338 (233 to 469)	61 (28 to 109)	0 (0 to 0)
Puerto Rico	2007	1118 (950 to 1296)	798 (660 to 954)	270 (183 to 376)	50 (24 to 90)	0 (0 to 0)	1411 (1200 to 1637)	1007 (833 to 1205)	340 (232 to 474)	63 (31 to 113)	0 (0 to 0)
Puerto Rico	2008	1116 (968 to 1277)	796 (677 to 930)	268 (183 to 371)	51 (24 to 92)	0 (0 to 0)	1409 (1222 to 1612)	1006 (855 to 1175)	339 (231 to 468)	65 (30 to 116)	0 (0 to 0)
Puerto Rico	2009	1127 (987 to 1281)	806 (707 to 920)	268 (182 to 376)	53 (24 to 92)	0 (0 to 0)	1423 (1247 to 1617)	1018 (892 to 1161)	339 (230 to 475)	67 (31 to 117)	0 (0 to 0)
Puerto Rico	2010	1133 (1008 to 1266)	810 (736 to 895)	269 (184 to 378)	54 (25 to 95)	0 (0 to 0)	1430 (1272 to 1599)	1023 (929 to 1130)	339 (232 to 477)	68 (31 to 120)	0 (0 to 0)

Puerto Rico	2011	1143 (1027 to 1271)	817 (762 to 882)	270 (184 to 380)	55 (25 to 99)	0 (0 to 0)	1443 (1296 to 1605)	1032 (962 to 1113)	341 (232 to 480)	70 (32 to 125)	0 (0 to 0)
Puerto Rico	2012	1169 (1062 to 1298)	838 (793 to 886)	274 (186 to 381)	58 (27 to 106)	0 (0 to 0)	1476 (1341 to 1639)	1058 (1001 to 1118)	345 (235 to 481)	73 (35 to 133)	0 (0 to 0)
Puerto Rico	2013	1192 (1085 to 1324)	857 (815 to 899)	276 (187 to 388)	60 (29 to 112)	0 (0 to 0)	1506 (1369 to 1672)	1082 (1029 to 1135)	349 (236 to 489)	75 (36 to 142)	0 (0 to 0)
Puerto Rico	2014	1224 (1114 to 1355)	882 (834 to 926)	280 (189 to 395)	62 (30 to 113)	0 (0 to 0)	1546 (1407 to 1711)	1114 (1053 to 1170)	353 (239 to 498)	79 (38 to 143)	0 (0 to 0)
Puerto Rico	2015	1214 (1101 to 1351)	874 (815 to 933)	278 (189 to 394)	62 (30 to 114)	0 (0 to 0)	1533 (1390 to 1706)	1103 (1029 to 1177)	351 (238 to 498)	78 (38 to 144)	0 (0 to 0)
Puerto Rico	2016	1218 (1087 to 1369)	876 (798 to 957)	279 (190 to 399)	63 (29 to 116)	0 (0 to 0)	1538 (1372 to 1729)	1106 (1008 to 1209)	353 (240 to 504)	79 (37 to 147)	0 (0 to 0)
Puerto Rico	2017	1276 (1101 to 1487)	921 (811 to 1034)	289 (197 to 408)	66 (31 to 125)	0 (0 to 0)	1611 (1390 to 1878)	1163 (1024 to 1306)	365 (248 to 515)	84 (39 to 157)	0 (0 to 0)
Puerto Rico	2018	1394 (1204 to 1617)	1034 (909 to 1160)	289 (197 to 408)	71 (33 to 133)	0 (0 to 0)	1760 (1521 to 2041)	1306 (1148 to 1465)	365 (248 to 516)	89 (41 to 167)	0 (0 to 0)
Puerto Rico	2019	1446 (1251 to 1679)	1082 (950 to 1217)	291 (198 to 411)	73 (34 to 136)	0 (0 to 0)	1826 (1579 to 2120)	1367 (1199 to 1537)	367 (250 to 519)	92 (42 to 172)	0 (0 to 0)
Puerto Rico	2020	1480 (1276 to 1709)	1111 (976 to 1254)	295 (201 to 416)	74 (34 to 139)	0 (0 to 0)	1868 (1611 to 2158)	1403 (1233 to 1583)	372 (254 to 525)	94 (43 to 176)	0 (0 to 0)

Puerto Rico	2021	1509 (1302 to 1750)	1136 (999 to 1277)	297 (203 to 420)	75 (35 to 141)	0 (0 to 0)	1905 (1644 to 2210)	1434 (1261 to 1613)	376 (256 to 531)	95 (44 to 178)	0 (0 to 0)
Puerto Rico	2022	1536 (1324 to 1784)	1159 (1016 to 1306)	300 (204 to 425)	77 (35 to 143)	0 (0 to 0)	1939 (1672 to 2252)	1464 (1283 to 1649)	378 (258 to 537)	97 (45 to 180)	0 (0 to 0)
Puerto Rico	2023	1562 (1349 to 1818)	1182 (1036 to 1336)	302 (206 to 428)	78 (36 to 145)	0 (0 to 0)	1972 (1703 to 2296)	1493 (1308 to 1687)	381 (260 to 541)	98 (45 to 183)	0 (0 to 0)
Puerto Rico	2024	1587 (1372 to 1849)	1204 (1054 to 1359)	304 (207 to 431)	79 (36 to 146)	0 (0 to 0)	2004 (1732 to 2335)	1520 (1331 to 1716)	384 (262 to 545)	100 (46 to 185)	0 (0 to 0)
Puerto Rico	2025	1611 (1391 to 1869)	1226 (1071 to 1389)	306 (209 to 434)	80 (37 to 148)	0 (0 to 0)	2035 (1757 to 2360)	1548 (1352 to 1754)	386 (263 to 548)	101 (46 to 186)	0 (0 to 0)
Puerto Rico	2026	1637 (1410 to 1903)	1248 (1091 to 1410)	308 (211 to 437)	81 (37 to 149)	0 (0 to 0)	2067 (1780 to 2403)	1576 (1378 to 1780)	388 (266 to 551)	102 (47 to 188)	0 (0 to 0)
Puerto Rico	2027	1661 (1429 to 1934)	1270 (1106 to 1436)	310 (212 to 438)	82 (38 to 151)	0 (0 to 0)	2098 (1804 to 2442)	1603 (1397 to 1813)	391 (268 to 554)	104 (48 to 191)	0 (0 to 0)
Puerto Rico	2028	1686 (1448 to 1963)	1291 (1120 to 1459)	311 (213 to 440)	83 (38 to 153)	0 (0 to 0)	2128 (1828 to 2479)	1630 (1414 to 1842)	393 (270 to 556)	105 (48 to 193)	0 (0 to 0)
Puerto Rico	2029	1711 (1475 to 2000)	1313 (1136 to 1489)	313 (215 to 442)	84 (39 to 155)	0 (0 to 0)	2160 (1862 to 2525)	1658 (1435 to 1880)	395 (271 to 558)	106 (49 to 196)	0 (0 to 0)
Puerto Rico	2030	1742 (1499 to 2034)	1341 (1160 to 1524)	315 (216 to 445)	85 (39 to 158)	0 (0 to 0)	2199 (1892 to 2568)	1694 (1465 to 1924)	398 (272 to 561)	108 (49 to 200)	0 (0 to 0)

Puerto Rico	2031	1774 (1526 to 2082)	1370 (1184 to 1561)	317 (217 to 447)	87 (40 to 161)	0 (0 to 0)	2239 (1926 to 2629)	1730 (1496 to 1971)	400 (275 to 565)	110 (50 to 203)	0 (0 to 0)
Puerto Rico	2032	1796 (1540 to 2095)	1390 (1209 to 1584)	318 (219 to 450)	88 (40 to 163)	0 (0 to 0)	2267 (1945 to 2645)	1755 (1526 to 2000)	401 (276 to 568)	111 (51 to 205)	0 (0 to 0)
Puerto Rico	2033	1816 (1561 to 2123)	1408 (1221 to 1601)	319 (219 to 451)	89 (41 to 164)	0 (0 to 0)	2293 (1971 to 2680)	1778 (1541 to 2021)	403 (277 to 570)	112 (51 to 207)	0 (0 to 0)
Puerto Rico	2034	1835 (1577 to 2153)	1425 (1234 to 1625)	320 (220 to 453)	89 (41 to 165)	0 (0 to 0)	2316 (1991 to 2718)	1799 (1558 to 2052)	404 (278 to 572)	113 (52 to 209)	0 (0 to 0)
Puerto Rico	2035	1852 (1597 to 2169)	1441 (1251 to 1645)	321 (221 to 455)	90 (42 to 166)	0 (0 to 0)	2338 (2017 to 2738)	1819 (1580 to 2077)	405 (279 to 575)	114 (52 to 210)	0 (0 to 0)
Puerto Rico	2036	1869 (1606 to 2190)	1456 (1264 to 1662)	322 (221 to 457)	91 (42 to 168)	0 (0 to 0)	2360 (2027 to 2765)	1839 (1596 to 2099)	406 (280 to 577)	115 (53 to 212)	0 (0 to 0)
Puerto Rico	2037	1883 (1616 to 2196)	1470 (1273 to 1680)	322 (222 to 458)	92 (42 to 169)	0 (0 to 0)	2378 (2040 to 2772)	1856 (1607 to 2121)	407 (280 to 578)	116 (53 to 213)	0 (0 to 0)
Puerto Rico	2038	1901 (1632 to 2216)	1485 (1286 to 1703)	323 (222 to 458)	92 (43 to 170)	0 (0 to 0)	2400 (2060 to 2798)	1875 (1623 to 2150)	408 (281 to 579)	117 (54 to 215)	0 (0 to 0)
Puerto Rico	2039	1918 (1643 to 2252)	1501 (1298 to 1726)	324 (223 to 459)	93 (43 to 171)	0 (0 to 0)	2422 (2075 to 2844)	1895 (1639 to 2179)	409 (282 to 580)	117 (54 to 216)	0 (0 to 0)
Puerto Rico	2040	1935 (1655 to 2262)	1517 (1310 to 1744)	325 (224 to 461)	94 (44 to 172)	0 (0 to 0)	2444 (2090 to 2856)	1915 (1654 to 2202)	410 (282 to 582)	118 (55 to 217)	0 (0 to 0)

Puerto Rico	2041	1953 (1669 to 2281)	1532 (1320 to 1765)	326 (224 to 463)	95 (44 to 173)	0 (0 to 0)	2466 (2108 to 2880)	1935 (1667 to 2229)	411 (283 to 585)	119 (55 to 219)	0 (0 to 0)
Puerto Rico	2042	1972 (1687 to 2307)	1549 (1334 to 1772)	327 (225 to 466)	95 (44 to 175)	0 (0 to 0)	2490 (2131 to 2913)	1956 (1684 to 2237)	413 (284 to 588)	121 (56 to 221)	0 (0 to 0)
Puerto Rico	2043	1991 (1696 to 2327)	1566 (1352 to 1805)	328 (226 to 466)	96 (45 to 176)	0 (0 to 0)	2513 (2142 to 2938)	1978 (1707 to 2279)	414 (285 to 589)	122 (56 to 222)	0 (0 to 0)
Puerto Rico	2044	2010 (1709 to 2353)	1584 (1360 to 1821)	329 (226 to 467)	97 (45 to 178)	0 (0 to 0)	2538 (2158 to 2972)	2000 (1718 to 2299)	416 (286 to 590)	123 (57 to 224)	0 (0 to 0)
Puerto Rico	2045	2029 (1723 to 2382)	1601 (1376 to 1847)	330 (227 to 468)	98 (45 to 179)	0 (0 to 0)	2562 (2176 to 3008)	2022 (1738 to 2332)	417 (287 to 591)	124 (57 to 226)	0 (0 to 0)
Puerto Rico	2046	2048 (1744 to 2396)	1618 (1388 to 1866)	331 (228 to 469)	99 (45 to 181)	0 (0 to 0)	2586 (2202 to 3025)	2043 (1753 to 2356)	418 (287 to 592)	125 (57 to 228)	0 (0 to 0)
Puerto Rico	2047	2067 (1757 to 2411)	1635 (1401 to 1882)	332 (228 to 470)	100 (46 to 183)	0 (0 to 0)	2610 (2219 to 3044)	2065 (1769 to 2377)	419 (288 to 593)	126 (58 to 230)	0 (0 to 0)
Puerto Rico	2048	2087 (1775 to 2433)	1653 (1418 to 1902)	333 (229 to 471)	101 (46 to 184)	0 (0 to 0)	2635 (2241 to 3072)	2087 (1790 to 2401)	421 (289 to 594)	127 (58 to 233)	0 (0 to 0)
Puerto Rico	2049	2106 (1789 to 2455)	1670 (1428 to 1925)	334 (230 to 472)	101 (47 to 186)	0 (0 to 0)	2659 (2259 to 3100)	2108 (1803 to 2430)	422 (290 to 597)	128 (59 to 235)	0 (0 to 0)
Puerto Rico	2050	2125 (1802 to 2477)	1687 (1448 to 1945)	336 (231 to 474)	102 (47 to 188)	0 (0 to 0)	2683 (2275 to 3127)	2130 (1829 to 2456)	424 (291 to 599)	129 (60 to 237)	0 (0 to 0)

Qatar	1995	1565 (1420 to 1712)	882 (762 to 1003)	575 (520 to 632)	108 (62 to 176)	0 (0 to 0)	2998 (2721 to 3279)	1690 (1460 to 1922)	1101 (997 to 1210)	207 (119 to 337)	0 (0 to 0)
Qatar	1996	1599 (1474 to 1731)	927 (820 to 1042)	559 (520 to 603)	113 (70 to 178)	0 (0 to 0)	3064 (2824 to 3317)	1776 (1570 to 1996)	1071 (996 to 1155)	217 (134 to 341)	0 (0 to 0)
Qatar	1997	1700 (1578 to 1827)	1005 (893 to 1120)	569 (533 to 611)	127 (83 to 188)	0 (0 to 0)	3258 (3023 to 3500)	1925 (1711 to 2145)	1091 (1021 to 1170)	242 (160 to 359)	0 (0 to 0)
Qatar	1998	1776 (1657 to 1903)	1064 (956 to 1172)	574 (538 to 612)	138 (98 to 192)	0 (0 to 0)	3403 (3174 to 3646)	2039 (1832 to 2246)	1099 (1031 to 1173)	264 (188 to 368)	0 (0 to 0)
Qatar	1999	1667 (1556 to 1778)	1015 (914 to 1118)	515 (483 to 549)	137 (105 to 177)	0 (0 to 0)	3193 (2980 to 3406)	1945 (1750 to 2142)	986 (925 to 1051)	262 (201 to 339)	0 (0 to 0)
Qatar	2000	1610 (1507 to 1722)	1010 (915 to 1107)	459 (429 to 490)	141 (116 to 170)	0 (0 to 0)	3084 (2888 to 3299)	1934 (1753 to 2121)	880 (821 to 938)	270 (221 to 327)	0 (0 to 0)
Qatar	2001	1717 (1609 to 1828)	1125 (1025 to 1222)	443 (412 to 474)	149 (127 to 172)	0 (0 to 0)	3290 (3083 to 3503)	2155 (1964 to 2341)	849 (789 to 907)	285 (243 to 330)	0 (0 to 0)
Qatar	2002	1821 (1710 to 1928)	1242 (1138 to 1339)	430 (401 to 461)	149 (130 to 169)	0 (0 to 0)	3488 (3277 to 3694)	2380 (2180 to 2565)	823 (768 to 883)	285 (248 to 324)	0 (0 to 0)
Qatar	2003	1961 (1845 to 2070)	1408 (1303 to 1512)	411 (382 to 442)	142 (123 to 162)	0 (0 to 0)	3756 (3534 to 3965)	2697 (2497 to 2896)	787 (732 to 846)	271 (236 to 310)	0 (0 to 0)
Qatar	2004	2033 (1919 to 2142)	1505 (1398 to 1611)	390 (361 to 420)	137 (119 to 159)	0 (0 to 0)	3894 (3677 to 4104)	2884 (2678 to 3087)	748 (692 to 805)	263 (228 to 304)	0 (0 to 0)

Qatar	2005	1875 (1766 to 1983)	1404 (1301 to 1509)	349 (321 to 380)	122 (105 to 142)	0 (0 to 0)	3592 (3383 to 3799)	2689 (2492 to 2892)	668 (615 to 729)	235 (202 to 273)	0 (0 to 0)
Qatar	2006	1759 (1648 to 1871)	1319 (1212 to 1423)	323 (296 to 353)	116 (99 to 136)	0 (0 to 0)	3370 (3158 to 3585)	2527 (2322 to 2727)	620 (566 to 677)	223 (190 to 261)	0 (0 to 0)
Qatar	2007	1599 (1491 to 1710)	1190 (1091 to 1298)	297 (272 to 325)	111 (94 to 129)	0 (0 to 0)	3063 (2856 to 3277)	2280 (2091 to 2486)	570 (521 to 623)	213 (180 to 248)	0 (0 to 0)
Qatar	2008	1327 (1238 to 1425)	967 (881 to 1058)	260 (236 to 286)	100 (85 to 117)	0 (0 to 0)	2542 (2371 to 2731)	1853 (1687 to 2027)	498 (453 to 547)	191 (162 to 223)	0 (0 to 0)
Qatar	2009	1480 (1379 to 1594)	1082 (984 to 1194)	269 (244 to 296)	129 (110 to 151)	0 (0 to 0)	2836 (2642 to 3054)	2072 (1886 to 2287)	516 (467 to 568)	248 (211 to 289)	0 (0 to 0)
Qatar	2010	1388 (1291 to 1497)	1023 (933 to 1129)	236 (213 to 261)	130 (111 to 150)	0 (0 to 0)	2660 (2474 to 2869)	1960 (1788 to 2162)	452 (408 to 499)	248 (214 to 288)	0 (0 to 0)
Qatar	2011	1385 (1289 to 1489)	1053 (961 to 1160)	202 (179 to 226)	130 (112 to 150)	0 (0 to 0)	2653 (2470 to 2853)	2017 (1842 to 2222)	387 (343 to 433)	249 (215 to 287)	0 (0 to 0)
Qatar	2012	1484 (1390 to 1598)	1178 (1083 to 1285)	169 (149 to 193)	137 (118 to 157)	0 (0 to 0)	2843 (2664 to 3062)	2256 (2074 to 2462)	324 (285 to 370)	263 (227 to 301)	0 (0 to 0)
Qatar	2013	1510 (1413 to 1615)	1237 (1144 to 1339)	141 (122 to 163)	132 (115 to 150)	0 (0 to 0)	2893 (2707 to 3094)	2369 (2192 to 2566)	270 (234 to 312)	254 (220 to 288)	0 (0 to 0)
Qatar	2014	1779 (1673 to 1896)	1491 (1387 to 1601)	139 (120 to 162)	150 (130 to 169)	0 (0 to 0)	3409 (3204 to 3633)	2856 (2658 to 3068)	267 (229 to 310)	287 (249 to 323)	0 (0 to 0)

Qatar	2015	2169 (2043 to 2307)	1827 (1702 to 1954)	157 (135 to 183)	185 (163 to 208)	0 (0 to 0)	4156 (3914 to 4420)	3500 (3262 to 3744)	301 (258 to 350)	355 (313 to 399)	0 (0 to 0)
Qatar	2016	2153 (2017 to 2293)	1796 (1670 to 1928)	163 (139 to 190)	194 (171 to 218)	0 (0 to 0)	4125 (3863 to 4392)	3441 (3199 to 3694)	312 (266 to 365)	372 (328 to 418)	0 (0 to 0)
Qatar	2017	1958 (1780 to 2155)	1612 (1468 to 1766)	159 (133 to 190)	187 (161 to 219)	0 (0 to 0)	3750 (3410 to 4128)	3088 (2812 to 3383)	305 (254 to 364)	358 (308 to 419)	0 (0 to 0)
Qatar	2018	1864 (1652 to 2111)	1518 (1341 to 1718)	159 (132 to 189)	187 (161 to 220)	0 (0 to 0)	3572 (3165 to 4044)	2908 (2569 to 3291)	305 (254 to 363)	358 (308 to 421)	0 (0 to 0)
Qatar	2019	1998 (1726 to 2297)	1646 (1406 to 1898)	163 (135 to 193)	190 (163 to 223)	0 (0 to 0)	3828 (3307 to 4401)	3153 (2693 to 3636)	311 (258 to 370)	364 (313 to 427)	0 (0 to 0)
Qatar	2020	2175 (1844 to 2547)	1810 (1513 to 2135)	170 (141 to 202)	196 (168 to 230)	0 (0 to 0)	4167 (3532 to 4879)	3467 (2899 to 4090)	325 (270 to 386)	375 (322 to 441)	0 (0 to 0)
Qatar	2021	2313 (1948 to 2758)	1944 (1598 to 2344)	172 (143 to 207)	197 (169 to 232)	0 (0 to 0)	4432 (3732 to 5283)	3724 (3060 to 4491)	330 (274 to 396)	377 (324 to 444)	0 (0 to 0)
Qatar	2022	2454 (2051 to 2948)	2082 (1712 to 2542)	175 (144 to 209)	198 (170 to 233)	0 (0 to 0)	4702 (3930 to 5648)	3988 (3280 to 4871)	335 (276 to 401)	379 (326 to 446)	0 (0 to 0)
Qatar	2023	2620 (2170 to 3185)	2236 (1823 to 2752)	178 (145 to 213)	206 (177 to 243)	0 (0 to 0)	5020 (4157 to 6102)	4283 (3493 to 5272)	341 (278 to 408)	396 (340 to 465)	0 (0 to 0)
Qatar	2024	2648 (2194 to 3210)	2260 (1842 to 2777)	181 (147 to 217)	208 (178 to 245)	0 (0 to 0)	5074 (4203 to 6149)	4330 (3529 to 5320)	346 (281 to 416)	398 (341 to 469)	0 (0 to 0)

Qatar	2025	2684 (2220 to 3279)	2292 (1868 to 2850)	183 (149 to 220)	209 (179 to 246)	0 (0 to 0)	5142 (4253 to 6281)	4391 (3579 to 5460)	350 (285 to 421)	401 (343 to 472)	0 (0 to 0)
Qatar	2026	2855 (2343 to 3493)	2452 (1973 to 3040)	185 (150 to 223)	218 (187 to 257)	0 (0 to 0)	5469 (4489 to 6692)	4697 (3779 to 5823)	354 (287 to 428)	418 (358 to 493)	0 (0 to 0)
Qatar	2027	3016 (2445 to 3722)	2603 (2079 to 3256)	187 (151 to 225)	226 (193 to 267)	0 (0 to 0)	5778 (4684 to 7130)	4986 (3983 to 6237)	359 (289 to 432)	433 (370 to 511)	0 (0 to 0)
Qatar	2028	3163 (2551 to 3935)	2741 (2177 to 3462)	189 (151 to 228)	233 (199 to 275)	0 (0 to 0)	6059 (4887 to 7539)	5251 (4170 to 6632)	361 (290 to 438)	447 (381 to 526)	0 (0 to 0)
Qatar	2029	3306 (2664 to 4165)	2876 (2289 to 3674)	190 (151 to 232)	240 (205 to 283)	0 (0 to 0)	6334 (5103 to 7980)	5510 (4385 to 7039)	364 (290 to 445)	460 (392 to 541)	0 (0 to 0)
Qatar	2030	3458 (2762 to 4411)	3018 (2378 to 3913)	192 (152 to 236)	248 (212 to 291)	0 (0 to 0)	6624 (5291 to 8451)	5782 (4556 to 7497)	368 (290 to 452)	474 (405 to 558)	0 (0 to 0)
Qatar	2031	3623 (2882 to 4624)	3173 (2479 to 4136)	194 (153 to 240)	256 (218 to 300)	0 (0 to 0)	6940 (5521 to 8859)	6078 (4749 to 7923)	372 (292 to 459)	490 (418 to 575)	0 (0 to 0)
Qatar	2032	3795 (2974 to 4830)	3335 (2572 to 4337)	196 (153 to 243)	264 (225 to 309)	0 (0 to 0)	7271 (5697 to 9254)	6389 (4927 to 8309)	376 (293 to 466)	505 (430 to 592)	0 (0 to 0)
Qatar	2033	3989 (3102 to 5131)	3518 (2708 to 4608)	198 (153 to 247)	273 (232 to 319)	0 (0 to 0)	7642 (5944 to 9830)	6741 (5188 to 8828)	380 (293 to 473)	522 (445 to 612)	0 (0 to 0)
Qatar	2034	4194 (3258 to 5413)	3713 (2836 to 4883)	200 (152 to 251)	281 (239 to 330)	0 (0 to 0)	8035 (6242 to 10370)	7114 (5433 to 9355)	383 (292 to 481)	539 (459 to 632)	0 (0 to 0)

Qatar	2035	4395 (3418 to 5657)	3904 (2988 to 5116)	201 (151 to 254)	289 (245 to 340)	0 (0 to 0)	8419 (6548 to 10839)	7480 (5724 to 9801)	385 (289 to 487)	554 (469 to 651)	0 (0 to 0)
Qatar	2036	4576 (3529 to 5894)	4077 (3083 to 5338)	202 (150 to 257)	296 (249 to 349)	0 (0 to 0)	8766 (6760 to 11292)	7811 (5907 to 10226)	387 (287 to 493)	568 (478 to 668)	0 (0 to 0)
Qatar	2037	4738 (3613 to 6160)	4233 (3169 to 5596)	202 (149 to 260)	303 (254 to 357)	0 (0 to 0)	9077 (6922 to 11800)	8110 (6071 to 10720)	388 (285 to 498)	580 (486 to 683)	0 (0 to 0)
Qatar	2038	4868 (3698 to 6341)	4358 (3236 to 5789)	203 (148 to 263)	307 (257 to 363)	0 (0 to 0)	9326 (7085 to 12149)	8349 (6200 to 11090)	389 (283 to 503)	589 (493 to 696)	0 (0 to 0)
Qatar	2039	4978 (3778 to 6515)	4464 (3321 to 5949)	203 (146 to 264)	311 (260 to 369)	0 (0 to 0)	9537 (7237 to 12482)	8551 (6361 to 11396)	389 (281 to 505)	597 (498 to 706)	0 (0 to 0)
Qatar	2040	5087 (3845 to 6682)	4569 (3390 to 6099)	203 (145 to 265)	316 (263 to 374)	0 (0 to 0)	9746 (7366 to 12801)	8753 (6494 to 11684)	388 (279 to 507)	604 (504 to 717)	0 (0 to 0)
Qatar	2041	5195 (3910 to 6887)	4673 (3450 to 6331)	203 (144 to 266)	319 (266 to 380)	0 (0 to 0)	9952 (7491 to 13194)	8952 (6610 to 12129)	388 (276 to 509)	612 (509 to 727)	0 (0 to 0)
Qatar	2042	5290 (3955 to 7064)	4766 (3467 to 6471)	202 (143 to 266)	322 (268 to 384)	0 (0 to 0)	10135 (7578 to 13533)	9131 (6643 to 12397)	386 (273 to 509)	617 (513 to 735)	0 (0 to 0)
Qatar	2043	5380 (4047 to 7156)	4855 (3569 to 6567)	200 (140 to 267)	325 (270 to 387)	0 (0 to 0)	10306 (7753 to 13709)	9301 (6838 to 12581)	384 (269 to 511)	622 (516 to 742)	0 (0 to 0)
Qatar	2044	5475 (4096 to 7212)	4950 (3633 to 6623)	199 (138 to 266)	327 (271 to 391)	0 (0 to 0)	10489 (7848 to 13817)	9483 (6960 to 12688)	381 (264 to 509)	626 (519 to 748)	0 (0 to 0)

Qatar	2045	5548 (4126 to 7270)	5024 (3673 to 6743)	197 (135 to 264)	327 (271 to 393)	0 (0 to 0)	10629 (7904 to 13928)	9626 (7036 to 12918)	377 (258 to 506)	627 (520 to 752)	0 (0 to 0)
Qatar	2046	5604 (4145 to 7346)	5083 (3664 to 6763)	194 (131 to 262)	327 (271 to 393)	0 (0 to 0)	10736 (7940 to 14074)	9738 (7019 to 12956)	372 (251 to 502)	627 (519 to 753)	0 (0 to 0)
Qatar	2047	5646 (4183 to 7457)	5128 (3736 to 6868)	191 (128 to 260)	326 (270 to 391)	0 (0 to 0)	10816 (8013 to 14286)	9825 (7157 to 13157)	366 (245 to 499)	625 (517 to 750)	0 (0 to 0)
Qatar	2048	5657 (4169 to 7473)	5145 (3718 to 6886)	188 (124 to 258)	324 (268 to 389)	0 (0 to 0)	10837 (7987 to 14317)	9857 (7122 to 13192)	359 (237 to 494)	621 (513 to 745)	0 (0 to 0)
Qatar	2049	5654 (4170 to 7512)	5150 (3713 to 6945)	183 (120 to 254)	321 (265 to 386)	0 (0 to 0)	10833 (7988 to 14391)	9867 (7114 to 13305)	351 (229 to 487)	614 (507 to 739)	0 (0 to 0)
Qatar	2050	5626 (4120 to 7435)	5131 (3686 to 6857)	179 (115 to 250)	316 (260 to 381)	0 (0 to 0)	10778 (7894 to 14244)	9830 (7061 to 13136)	343 (221 to 478)	605 (498 to 730)	0 (0 to 0)
Romania	1995	180 (158 to 205)	132 (113 to 154)	43 (33 to 56)	3 (1 to 5)	2 (2 to 2)	407 (357 to 464)	298 (255 to 348)	98 (74 to 127)	7 (3 to 12)	5 (5 to 5)
Romania	1996	189 (168 to 212)	140 (122 to 160)	45 (34 to 57)	3 (1 to 5)	1 (1 to 1)	426 (379 to 478)	316 (276 to 361)	101 (77 to 128)	7 (3 to 12)	3 (3 to 3)
Romania	1997	196 (177 to 218)	148 (132 to 166)	44 (35 to 56)	3 (1 to 5)	1 (1 to 1)	443 (399 to 493)	335 (298 to 375)	100 (79 to 126)	7 (3 to 12)	1 (1 to 1)
Romania	1998	201 (182 to 222)	152 (136 to 170)	44 (35 to 55)	3 (1 to 5)	2 (2 to 2)	454 (412 to 501)	343 (306 to 383)	100 (79 to 124)	7 (3 to 12)	4 (4 to 4)
Romania	1999	214 (194 to 235)	165 (147 to 182)	44 (35 to 54)	3 (2 to 6)	1 (1 to 1)	482 (437 to 532)	373 (332 to 412)	100 (79 to 123)	7 (3 to 13)	3 (3 to 3)
Romania	2000	227 (207 to 250)	179 (160 to 197)	45 (36 to 55)	3 (2 to 6)	0 (0 to 0)	513 (468 to 564)	404 (362 to 445)	102 (81 to 125)	8 (4 to 14)	0 (0 to 0)

Romania	2001	248 (226 to 271)	196 (177 to 215)	47 (38 to 58)	4 (2 to 7)	1 (1 to 1)	560 (510 to 612)	443 (400 to 486)	107 (86 to 132)	8 (4 to 15)	2 (2 to 2)
Romania	2002	276 (252 to 301)	220 (199 to 241)	51 (41 to 63)	4 (2 to 8)	1 (1 to 1)	624 (569 to 679)	496 (450 to 545)	116 (93 to 142)	10 (5 to 17)	2 (2 to 2)
Romania	2003	306 (281 to 333)	244 (224 to 267)	57 (47 to 70)	4 (2 to 8)	1 (1 to 1)	691 (635 to 751)	552 (505 to 602)	129 (105 to 157)	10 (5 to 18)	1 (1 to 1)
Romania	2004	350 (324 to 378)	276 (254 to 299)	68 (56 to 83)	5 (2 to 8)	1 (1 to 1)	790 (731 to 853)	623 (574 to 676)	154 (127 to 188)	10 (5 to 19)	3 (3 to 3)
Romania	2005	364 (338 to 391)	289 (266 to 312)	71 (59 to 87)	4 (2 to 7)	1 (1 to 1)	822 (764 to 883)	652 (600 to 704)	160 (133 to 196)	9 (4 to 16)	1 (1 to 1)
Romania	2006	396 (369 to 426)	316 (293 to 342)	76 (63 to 93)	3 (2 to 7)	0 (0 to 0)	895 (834 to 961)	714 (661 to 772)	172 (142 to 210)	8 (4 to 15)	1 (1 to 1)
Romania	2007	428 (400 to 458)	342 (318 to 367)	80 (67 to 98)	3 (2 to 6)	2 (2 to 2)	965 (904 to 1034)	772 (717 to 829)	181 (151 to 220)	8 (4 to 15)	4 (4 to 4)
Romania	2008	461 (432 to 493)	369 (343 to 396)	88 (73 to 105)	3 (2 to 7)	1 (1 to 1)	1041 (976 to 1112)	833 (775 to 893)	198 (164 to 238)	8 (4 to 15)	3 (3 to 3)
Romania	2009	458 (429 to 490)	362 (336 to 390)	92 (76 to 109)	3 (1 to 6)	1 (1 to 1)	1033 (968 to 1106)	817 (758 to 879)	207 (173 to 247)	7 (3 to 13)	2 (2 to 2)
Romania	2010	457 (428 to 491)	359 (333 to 387)	94 (79 to 112)	3 (1 to 5)	1 (1 to 1)	1033 (967 to 1108)	810 (752 to 873)	213 (178 to 253)	7 (3 to 12)	3 (3 to 3)
Romania	2011	450 (421 to 483)	343 (319 to 369)	95 (81 to 112)	3 (2 to 6)	9 (9 to 9)	1017 (951 to 1090)	775 (719 to 834)	215 (182 to 254)	7 (3 to 13)	20 (20 to 20)
Romania	2012	445 (416 to 477)	345 (320 to 373)	96 (80 to 114)	3 (2 to 6)	1 (1 to 1)	1004 (940 to 1078)	778 (723 to 843)	217 (182 to 257)	7 (4 to 14)	2 (2 to 2)

Romania	2013	477 (446 to 510)	358 (333 to 386)	98 (82 to 117)	3 (2 to 6)	18 (18 to 18)	1076 (1008 to 1152)	807 (752 to 871)	221 (185 to 264)	8 (4 to 14)	40 (40 to 40)
Romania	2014	490 (459 to 524)	377 (352 to 405)	101 (85 to 121)	4 (2 to 7)	8 (8 to 8)	1106 (1036 to 1183)	851 (795 to 914)	229 (191 to 274)	9 (4 to 16)	18 (18 to 18)
Romania	2015	520 (487 to 558)	407 (380 to 435)	108 (90 to 129)	4 (2 to 8)	1 (1 to 1)	1173 (1098 to 1258)	919 (857 to 983)	243 (202 to 292)	10 (5 to 19)	2 (2 to 2)
Romania	2016	535 (499 to 577)	418 (387 to 451)	111 (91 to 135)	5 (2 to 9)	1 (1 to 1)	1207 (1126 to 1302)	943 (873 to 1017)	251 (205 to 305)	11 (6 to 21)	1 (1 to 1)
Romania	2017	585 (535 to 641)	456 (416 to 502)	119 (96 to 147)	5 (3 to 10)	4 (4 to 4)	1320 (1207 to 1446)	1030 (938 to 1132)	269 (217 to 332)	12 (6 to 23)	9 (9 to 9)
Romania	2018	640 (583 to 703)	506 (457 to 557)	125 (100 to 154)	6 (3 to 11)	4 (4 to 4)	1446 (1316 to 1588)	1142 (1032 to 1257)	282 (227 to 347)	13 (7 to 25)	8 (8 to 8)
Romania	2019	691 (627 to 760)	551 (496 to 612)	130 (105 to 160)	6 (3 to 12)	4 (4 to 4)	1559 (1414 to 1716)	1244 (1119 to 1381)	293 (236 to 361)	14 (7 to 27)	8 (8 to 9)
Romania	2020	720 (649 to 797)	575 (513 to 645)	134 (108 to 166)	6 (3 to 12)	4 (4 to 4)	1626 (1466 to 1798)	1299 (1158 to 1455)	303 (245 to 375)	15 (7 to 28)	9 (8 to 9)
Romania	2021	740 (664 to 823)	592 (526 to 667)	137 (111 to 170)	7 (3 to 13)	4 (4 to 4)	1670 (1499 to 1858)	1336 (1186 to 1506)	310 (250 to 384)	15 (7 to 28)	9 (8 to 10)
Romania	2022	760 (677 to 850)	608 (534 to 687)	141 (113 to 174)	7 (3 to 13)	4 (4 to 5)	1715 (1529 to 1918)	1373 (1205 to 1550)	317 (255 to 393)	15 (8 to 29)	9 (9 to 10)

Romania	2023	783 (698 to 876)	628 (553 to 714)	144 (116 to 178)	7 (3 to 13)	4 (4 to 5)	1767 (1575 to 1978)	1417 (1248 to 1613)	325 (261 to 401)	16 (8 to 30)	10 (9 to 11)
Romania	2024	806 (716 to 902)	647 (566 to 736)	147 (119 to 182)	7 (4 to 14)	4 (4 to 5)	1820 (1617 to 2036)	1461 (1278 to 1662)	333 (268 to 411)	16 (8 to 31)	10 (9 to 11)
Romania	2025	829 (735 to 935)	667 (582 to 764)	151 (122 to 187)	7 (4 to 14)	4 (0 to 5)	1872 (1658 to 2111)	1505 (1314 to 1724)	341 (274 to 423)	17 (8 to 32)	9 (0 to 11)
Romania	2026	852 (750 to 966)	686 (591 to 787)	155 (124 to 193)	8 (4 to 14)	3 (0 to 5)	1923 (1694 to 2181)	1548 (1334 to 1776)	349 (281 to 435)	17 (9 to 33)	8 (0 to 12)
Romania	2027	875 (771 to 992)	706 (608 to 812)	159 (127 to 197)	8 (4 to 15)	2 (0 to 5)	1976 (1739 to 2239)	1594 (1372 to 1833)	358 (287 to 445)	18 (9 to 34)	5 (0 to 12)
Romania	2028	900 (788 to 1027)	728 (624 to 842)	163 (131 to 202)	8 (4 to 15)	2 (0 to 5)	2032 (1779 to 2319)	1643 (1408 to 1902)	367 (295 to 456)	19 (9 to 35)	4 (0 to 12)
Romania	2029	927 (807 to 1059)	750 (640 to 872)	167 (135 to 208)	8 (4 to 16)	1 (0 to 5)	2092 (1820 to 2389)	1694 (1444 to 1968)	377 (304 to 469)	19 (9 to 36)	2 (0 to 12)
Romania	2030	955 (819 to 1104)	774 (650 to 915)	172 (138 to 214)	9 (4 to 17)	1 (0 to 5)	2155 (1849 to 2492)	1746 (1468 to 2065)	387 (312 to 482)	20 (10 to 38)	1 (0 to 12)
Romania	2031	984 (843 to 1141)	798 (667 to 946)	176 (142 to 219)	9 (4 to 17)	0 (0 to 5)	2221 (1903 to 2575)	1802 (1507 to 2134)	397 (320 to 495)	20 (10 to 39)	1 (0 to 12)
Romania	2032	1010 (862 to 1177)	821 (682 to 977)	180 (145 to 225)	9 (5 to 18)	0 (0 to 5)	2281 (1946 to 2656)	1852 (1540 to 2206)	407 (327 to 508)	21 (10 to 40)	1 (0 to 12)

Romania	2033	1038 (882 to 1214)	844 (697 to 1009)	184 (148 to 229)	10 (5 to 18)	0 (0 to 5)	2343 (1990 to 2740)	1906 (1573 to 2278)	415 (334 to 517)	22 (11 to 41)	0 (0 to 12)
Romania	2034	1064 (895 to 1250)	866 (705 to 1041)	187 (150 to 233)	10 (5 to 19)	0 (0 to 5)	2401 (2021 to 2822)	1956 (1592 to 2350)	423 (339 to 526)	22 (11 to 42)	0 (0 to 12)
Romania	2035	1090 (912 to 1290)	889 (720 to 1077)	191 (152 to 238)	10 (5 to 19)	0 (0 to 5)	2460 (2058 to 2911)	2007 (1626 to 2432)	430 (344 to 537)	23 (11 to 43)	0 (0 to 12)
Romania	2036	1117 (930 to 1324)	912 (736 to 1112)	194 (155 to 242)	10 (5 to 19)	0 (0 to 6)	2520 (2099 to 2989)	2059 (1662 to 2509)	437 (351 to 547)	23 (11 to 44)	1 (0 to 13)
Romania	2037	1144 (945 to 1366)	936 (751 to 1153)	197 (158 to 247)	10 (5 to 20)	0 (0 to 6)	2583 (2132 to 3084)	2114 (1695 to 2602)	445 (356 to 558)	24 (12 to 45)	1 (0 to 13)
Romania	2038	1172 (969 to 1411)	960 (770 to 1191)	201 (161 to 252)	11 (5 to 20)	0 (0 to 6)	2645 (2187 to 3184)	2168 (1738 to 2687)	453 (363 to 569)	24 (12 to 46)	1 (0 to 14)
Romania	2039	1201 (990 to 1449)	986 (786 to 1225)	204 (163 to 257)	11 (5 to 21)	0 (0 to 6)	2711 (2235 to 3270)	2224 (1773 to 2766)	461 (369 to 580)	25 (12 to 47)	1 (0 to 14)
Romania	2040	1229 (1002 to 1488)	1009 (800 to 1254)	208 (166 to 261)	11 (5 to 21)	0 (0 to 7)	2773 (2261 to 3360)	2278 (1806 to 2832)	469 (375 to 589)	25 (12 to 48)	1 (0 to 15)
Romania	2041	1258 (1031 to 1530)	1035 (816 to 1297)	212 (169 to 265)	11 (6 to 22)	0 (0 to 7)	2840 (2326 to 3454)	2336 (1841 to 2927)	478 (381 to 598)	26 (13 to 49)	1 (0 to 15)
Romania	2042	1288 (1046 to 1580)	1061 (835 to 1324)	215 (172 to 269)	12 (6 to 22)	0 (0 to 7)	2908 (2361 to 3567)	2394 (1886 to 2989)	486 (388 to 608)	26 (13 to 50)	1 (0 to 15)

Romania	2043	1319 (1063 to 1616)	1088 (850 to 1364)	219 (175 to 274)	12 (6 to 22)	0 (0 to 7)	2977 (2399 to 3647)	2455 (1918 to 3078)	495 (395 to 618)	27 (13 to 51)	1 (0 to 15)
Romania	2044	1348 (1090 to 1664)	1113 (860 to 1411)	223 (178 to 278)	12 (6 to 23)	0 (0 to 7)	3044 (2460 to 3756)	2511 (1941 to 3186)	504 (402 to 628)	27 (14 to 52)	1 (0 to 16)
Romania	2045	1380 (1110 to 1698)	1140 (881 to 1448)	227 (181 to 284)	12 (6 to 23)	0 (0 to 7)	3115 (2505 to 3833)	2573 (1988 to 3268)	513 (409 to 641)	28 (14 to 53)	1 (0 to 16)
Romania	2046	1413 (1137 to 1746)	1168 (903 to 1491)	232 (185 to 290)	13 (6 to 24)	0 (0 to 8)	3190 (2567 to 3940)	2637 (2038 to 3365)	523 (417 to 654)	29 (14 to 54)	1 (0 to 17)
Romania	2047	1445 (1161 to 1795)	1195 (912 to 1518)	236 (188 to 296)	13 (6 to 25)	0 (0 to 8)	3261 (2620 to 4053)	2698 (2060 to 3427)	533 (424 to 668)	30 (15 to 56)	1 (0 to 17)
Romania	2048	1480 (1179 to 1845)	1225 (936 to 1560)	241 (191 to 303)	13 (7 to 25)	0 (0 to 8)	3340 (2662 to 4164)	2766 (2114 to 3521)	543 (432 to 683)	30 (15 to 57)	1 (0 to 17)
Romania	2049	1516 (1212 to 1885)	1256 (959 to 1613)	246 (195 to 309)	14 (7 to 26)	0 (0 to 8)	3422 (2736 to 4254)	2835 (2164 to 3640)	555 (441 to 697)	31 (15 to 59)	1 (0 to 18)
Romania	2050	1553 (1241 to 1936)	1288 (976 to 1660)	251 (199 to 315)	14 (7 to 27)	0 (0 to 9)	3506 (2801 to 4370)	2907 (2204 to 3746)	566 (450 to 711)	32 (16 to 61)	1 (0 to 19)
Russia	1995	307 (276 to 342)	209 (184 to 237)	79 (64 to 96)	19 (11 to 32)	0 (0 to 0)	823 (740 to 916)	561 (492 to 636)	212 (172 to 257)	51 (29 to 86)	0 (0 to 0)
Russia	1996	303 (276 to 333)	203 (182 to 228)	80 (67 to 95)	19 (11 to 32)	0 (0 to 0)	811 (741 to 892)	544 (487 to 612)	215 (180 to 255)	52 (30 to 85)	0 (0 to 0)
Russia	1997	320 (295 to 348)	211 (192 to 234)	86 (73 to 101)	22 (13 to 34)	1 (1 to 1)	858 (790 to 934)	566 (513 to 626)	231 (196 to 270)	58 (34 to 92)	3 (3 to 3)

Russia	1998	307 (283 to 333)	196 (179 to 216)	89 (76 to 102)	21 (13 to 34)	1 (1 to 1)	823 (759 to 893)	526 (479 to 579)	237 (204 to 274)	58 (35 to 91)	2 (2 to 2)
Russia	1999	294 (271 to 319)	182 (165 to 199)	90 (78 to 102)	22 (13 to 34)	0 (0 to 0)	788 (726 to 856)	487 (443 to 534)	242 (209 to 275)	59 (36 to 91)	1 (1 to 1)
Russia	2000	305 (281 to 330)	183 (168 to 200)	94 (81 to 107)	27 (16 to 41)	0 (0 to 0)	816 (754 to 885)	492 (451 to 536)	252 (218 to 286)	71 (43 to 110)	1 (1 to 1)
Russia	2001	323 (298 to 349)	191 (175 to 209)	101 (89 to 116)	30 (19 to 47)	0 (0 to 0)	866 (798 to 934)	512 (469 to 560)	272 (238 to 310)	81 (50 to 126)	1 (1 to 1)
Russia	2002	352 (325 to 379)	208 (189 to 226)	111 (97 to 126)	33 (21 to 50)	0 (0 to 0)	943 (871 to 1016)	557 (506 to 605)	298 (261 to 339)	88 (56 to 135)	1 (1 to 1)
Russia	2003	365 (338 to 393)	215 (196 to 235)	118 (102 to 134)	32 (20 to 49)	0 (0 to 0)	977 (907 to 1052)	577 (526 to 629)	315 (275 to 359)	85 (54 to 131)	0 (0 to 0)
Russia	2004	369 (342 to 398)	218 (200 to 239)	121 (106 to 137)	29 (18 to 45)	0 (0 to 0)	988 (916 to 1066)	585 (535 to 640)	324 (284 to 367)	78 (49 to 120)	0 (0 to 0)
Russia	2005	392 (365 to 423)	236 (217 to 258)	127 (111 to 144)	29 (18 to 45)	0 (0 to 0)	1052 (978 to 1135)	633 (581 to 691)	341 (298 to 387)	77 (47 to 120)	1 (1 to 1)
Russia	2006	419 (392 to 451)	255 (235 to 277)	135 (119 to 153)	28 (17 to 44)	1 (1 to 1)	1124 (1050 to 1207)	684 (630 to 743)	362 (318 to 411)	76 (46 to 119)	2 (2 to 2)
Russia	2007	472 (439 to 507)	293 (270 to 316)	150 (132 to 170)	29 (18 to 45)	1 (1 to 1)	1265 (1177 to 1360)	784 (724 to 848)	401 (353 to 454)	77 (47 to 121)	3 (3 to 3)
Russia	2008	496 (464 to 532)	309 (286 to 333)	160 (141 to 182)	26 (16 to 41)	1 (1 to 1)	1329 (1242 to 1426)	828 (765 to 893)	428 (378 to 486)	70 (43 to 110)	3 (3 to 3)
Russia	2009	511 (480 to 545)	320 (296 to 346)	169 (150 to 190)	22 (13 to 34)	1 (1 to 1)	1371 (1287 to 1461)	858 (794 to 927)	453 (402 to 508)	59 (36 to 91)	1 (1 to 1)

Russia	2010	499 (470 to 532)	311 (290 to 334)	169 (151 to 187)	19 (11 to 29)	0 (0 to 0)	1338 (1259 to 1426)	834 (777 to 895)	453 (403 to 502)	50 (30 to 78)	1 (1 to 1)
Russia	2011	505 (476 to 539)	315 (295 to 338)	172 (154 to 190)	17 (10 to 26)	0 (0 to 0)	1352 (1276 to 1445)	845 (790 to 905)	462 (414 to 510)	46 (28 to 71)	0 (0 to 0)
Russia	2012	533 (504 to 565)	334 (314 to 356)	183 (165 to 201)	17 (10 to 26)	0 (0 to 0)	1428 (1350 to 1514)	894 (841 to 955)	489 (442 to 538)	45 (27 to 70)	0 (0 to 0)
Russia	2013	551 (523 to 583)	342 (321 to 363)	193 (176 to 212)	16 (9 to 26)	0 (0 to 0)	1477 (1402 to 1561)	916 (859 to 973)	518 (472 to 568)	43 (25 to 69)	0 (0 to 0)
Russia	2014	562 (533 to 595)	343 (321 to 365)	204 (185 to 225)	15 (9 to 24)	0 (0 to 0)	1507 (1427 to 1596)	918 (861 to 977)	548 (495 to 603)	41 (23 to 65)	0 (0 to 0)
Russia	2015	561 (529 to 599)	332 (309 to 355)	214 (191 to 238)	15 (8 to 24)	0 (0 to 0)	1504 (1418 to 1606)	891 (829 to 952)	572 (513 to 637)	40 (23 to 63)	0 (0 to 0)
Russia	2016	570 (531 to 616)	331 (303 to 359)	224 (197 to 252)	15 (8 to 24)	0 (0 to 0)	1528 (1424 to 1650)	888 (813 to 962)	600 (528 to 675)	40 (23 to 64)	0 (0 to 0)
Russia	2017	574 (526 to 630)	328 (295 to 364)	230 (198 to 267)	15 (8 to 24)	0 (0 to 0)	1537 (1409 to 1688)	880 (791 to 974)	617 (529 to 714)	40 (21 to 64)	0 (0 to 0)
Russia	2018	572 (522 to 626)	322 (288 to 356)	235 (202 to 272)	15 (8 to 25)	0 (0 to 0)	1534 (1399 to 1679)	862 (771 to 953)	631 (541 to 730)	41 (21 to 66)	0 (0 to 0)
Russia	2019	596 (545 to 655)	342 (308 to 378)	239 (204 to 276)	16 (8 to 25)	0 (0 to 0)	1598 (1461 to 1755)	916 (824 to 1013)	639 (548 to 740)	42 (22 to 67)	0 (0 to 0)

Russia	2020	611 (557 to 673)	351 (315 to 389)	244 (209 to 282)	16 (8 to 26)	0 (0 to 0)	1637 (1494 to 1803)	941 (843 to 1041)	653 (559 to 755)	43 (23 to 69)	0 (0 to 0)
Russia	2021	617 (564 to 681)	356 (319 to 394)	245 (210 to 283)	16 (9 to 26)	0 (0 to 0)	1652 (1510 to 1824)	953 (854 to 1056)	656 (562 to 759)	44 (23 to 70)	0 (0 to 0)
Russia	2022	620 (563 to 684)	358 (320 to 397)	246 (211 to 285)	16 (9 to 27)	0 (0 to 0)	1662 (1510 to 1832)	958 (858 to 1064)	659 (564 to 763)	44 (23 to 71)	0 (0 to 0)
Russia	2023	624 (570 to 688)	360 (322 to 400)	247 (212 to 286)	17 (9 to 27)	0 (0 to 0)	1672 (1528 to 1843)	964 (862 to 1072)	663 (567 to 767)	45 (24 to 72)	0 (0 to 0)
Russia	2024	627 (569 to 692)	362 (323 to 402)	248 (213 to 288)	17 (9 to 27)	0 (0 to 0)	1681 (1524 to 1854)	969 (865 to 1078)	666 (569 to 772)	45 (24 to 73)	0 (0 to 0)
Russia	2025	632 (576 to 699)	366 (325 to 407)	250 (213 to 289)	17 (9 to 28)	0 (0 to 0)	1695 (1545 to 1872)	980 (872 to 1089)	669 (572 to 775)	46 (24 to 74)	0 (0 to 0)
Russia	2026	639 (578 to 704)	370 (329 to 413)	251 (215 to 291)	17 (9 to 28)	0 (0 to 0)	1712 (1550 to 1887)	991 (881 to 1108)	674 (576 to 780)	47 (25 to 75)	0 (0 to 0)
Russia	2027	647 (587 to 717)	375 (333 to 421)	254 (217 to 294)	18 (9 to 29)	0 (0 to 0)	1733 (1573 to 1922)	1006 (892 to 1128)	680 (581 to 788)	47 (25 to 77)	0 (0 to 0)
Russia	2028	657 (593 to 727)	382 (339 to 430)	257 (220 to 297)	18 (10 to 29)	0 (0 to 0)	1760 (1590 to 1948)	1024 (908 to 1153)	688 (588 to 797)	48 (25 to 79)	0 (0 to 0)
Russia	2029	668 (605 to 743)	389 (344 to 438)	260 (222 to 302)	18 (10 to 30)	0 (0 to 0)	1791 (1622 to 1990)	1044 (921 to 1175)	698 (596 to 808)	49 (26 to 80)	0 (0 to 0)

Russia	2030	681 (612 to 756)	398 (349 to 449)	265 (226 to 306)	19 (10 to 31)	0 (0 to 0)	1825 (1639 to 2025)	1065 (936 to 1204)	709 (605 to 821)	51 (27 to 82)	0 (0 to 0)
Russia	2031	695 (629 to 776)	407 (356 to 462)	269 (230 to 311)	19 (10 to 31)	0 (0 to 0)	1862 (1685 to 2079)	1090 (955 to 1238)	721 (616 to 834)	52 (27 to 84)	0 (0 to 0)
Russia	2032	709 (636 to 790)	416 (361 to 475)	273 (234 to 316)	20 (11 to 32)	0 (0 to 0)	1900 (1705 to 2116)	1114 (969 to 1273)	733 (627 to 847)	53 (28 to 86)	0 (0 to 0)
Russia	2033	724 (653 to 808)	426 (371 to 488)	278 (238 to 321)	20 (11 to 33)	0 (0 to 0)	1940 (1750 to 2165)	1140 (995 to 1307)	745 (636 to 861)	54 (29 to 88)	0 (0 to 0)
Russia	2034	740 (662 to 825)	436 (379 to 499)	283 (242 to 327)	21 (11 to 34)	0 (0 to 0)	1982 (1774 to 2212)	1168 (1015 to 1338)	759 (648 to 876)	56 (30 to 90)	0 (0 to 0)
Russia	2035	756 (679 to 846)	446 (386 to 513)	288 (246 to 333)	21 (11 to 35)	0 (0 to 0)	2026 (1820 to 2267)	1196 (1036 to 1373)	772 (660 to 891)	57 (31 to 93)	0 (0 to 0)
Russia	2036	772 (687 to 862)	457 (393 to 525)	293 (251 to 338)	22 (12 to 36)	0 (0 to 0)	2067 (1841 to 2310)	1224 (1054 to 1406)	785 (672 to 907)	59 (31 to 95)	0 (0 to 0)
Russia	2037	786 (701 to 882)	466 (400 to 539)	297 (255 to 343)	22 (12 to 36)	0 (0 to 0)	2106 (1879 to 2363)	1249 (1072 to 1443)	797 (682 to 920)	60 (32 to 98)	0 (0 to 0)
Russia	2038	799 (709 to 896)	475 (405 to 551)	301 (258 to 348)	23 (12 to 37)	0 (0 to 0)	2141 (1899 to 2401)	1273 (1087 to 1478)	807 (690 to 932)	61 (33 to 100)	0 (0 to 0)
Russia	2039	812 (720 to 915)	484 (411 to 564)	305 (261 to 352)	23 (12 to 38)	0 (0 to 0)	2175 (1930 to 2453)	1296 (1103 to 1511)	816 (699 to 944)	63 (33 to 102)	0 (0 to 0)

Russia	2040	824 (730 to 925)	492 (417 to 574)	308 (264 to 356)	24 (13 to 39)	0 (0 to 0)	2209 (1956 to 2478)	1319 (1117 to 1538)	826 (708 to 954)	64 (34 to 104)	0 (0 to 0)
Russia	2041	837 (742 to 951)	501 (423 to 585)	312 (267 to 360)	24 (13 to 40)	0 (0 to 0)	2242 (1987 to 2548)	1342 (1134 to 1568)	835 (716 to 965)	65 (35 to 106)	0 (0 to 0)
Russia	2042	849 (747 to 956)	509 (428 to 598)	315 (270 to 364)	25 (13 to 40)	0 (0 to 0)	2275 (2003 to 2562)	1365 (1147 to 1602)	843 (723 to 975)	66 (35 to 108)	0 (0 to 0)
Russia	2043	861 (761 to 985)	518 (434 to 606)	318 (272 to 368)	25 (13 to 41)	0 (0 to 0)	2308 (2039 to 2638)	1389 (1162 to 1625)	852 (728 to 987)	67 (36 to 110)	0 (0 to 0)
Russia	2044	873 (767 to 989)	527 (440 to 617)	321 (274 to 372)	26 (14 to 42)	0 (0 to 0)	2340 (2057 to 2650)	1411 (1179 to 1653)	860 (734 to 998)	69 (37 to 112)	0 (0 to 0)
Russia	2045	885 (777 to 1008)	535 (446 to 628)	324 (277 to 376)	26 (14 to 43)	0 (0 to 0)	2372 (2082 to 2702)	1435 (1196 to 1684)	868 (741 to 1007)	70 (37 to 114)	0 (0 to 0)
Russia	2046	896 (785 to 1016)	543 (455 to 639)	326 (279 to 379)	26 (14 to 43)	0 (0 to 0)	2401 (2103 to 2723)	1456 (1219 to 1713)	874 (747 to 1014)	71 (38 to 116)	0 (0 to 0)
Russia	2047	906 (796 to 1036)	551 (463 to 650)	329 (280 to 381)	27 (14 to 44)	0 (0 to 0)	2429 (2133 to 2777)	1476 (1239 to 1741)	881 (752 to 1021)	72 (39 to 118)	0 (0 to 0)
Russia	2048	916 (802 to 1041)	559 (468 to 659)	331 (282 to 384)	27 (15 to 45)	0 (0 to 0)	2456 (2150 to 2791)	1497 (1253 to 1765)	886 (755 to 1029)	73 (39 to 119)	0 (0 to 0)
Russia	2049	925 (812 to 1060)	566 (474 to 668)	332 (283 to 387)	28 (15 to 45)	0 (0 to 0)	2480 (2175 to 2841)	1516 (1271 to 1790)	891 (758 to 1037)	74 (40 to 121)	0 (0 to 0)

Russia	2050	934 (815 to 1062)	572 (478 to 678)	334 (284 to 390)	28 (15 to 46)	0 (0 to 0)	2504 (2185 to 2846)	1534 (1282 to 1816)	895 (761 to 1045)	75 (40 to 123)	0 (0 to 0)
Rwanda	1995	18 (15 to 22)	7 (5 to 9)	5 (3 to 7)	3 (1 to 5)	3 (3 to 3)	53 (44 to 64)	20 (15 to 26)	15 (10 to 20)	8 (4 to 16)	10 (10 to 10)
Rwanda	1996	17 (14 to 21)	7 (5 to 9)	5 (3 to 7)	3 (1 to 5)	3 (3 to 3)	52 (42 to 62)	20 (15 to 26)	15 (10 to 20)	8 (4 to 16)	9 (9 to 9)
Rwanda	1997	18 (15 to 22)	6 (5 to 8)	5 (3 to 6)	3 (1 to 5)	5 (5 to 5)	54 (46 to 64)	18 (13 to 24)	14 (9 to 19)	8 (4 to 14)	14 (14 to 14)
Rwanda	1998	17 (14 to 20)	5 (4 to 7)	5 (3 to 6)	2 (1 to 4)	5 (5 to 5)	50 (42 to 59)	16 (12 to 21)	13 (9 to 18)	7 (3 to 13)	14 (14 to 14)
Rwanda	1999	16 (14 to 19)	5 (4 to 7)	5 (3 to 6)	2 (1 to 4)	4 (4 to 4)	48 (40 to 57)	16 (12 to 21)	13 (9 to 18)	7 (3 to 13)	12 (12 to 12)
Rwanda	2000	14 (12 to 17)	4 (3 to 5)	4 (3 to 6)	2 (1 to 3)	4 (4 to 4)	41 (35 to 49)	12 (9 to 15)	12 (8 to 16)	5 (2 to 10)	13 (13 to 13)
Rwanda	2001	15 (13 to 18)	4 (3 to 5)	4 (3 to 6)	2 (1 to 3)	5 (5 to 5)	44 (38 to 52)	12 (9 to 15)	12 (8 to 17)	5 (2 to 10)	15 (15 to 15)
Rwanda	2002	17 (15 to 20)	5 (4 to 6)	5 (3 to 6)	2 (1 to 4)	6 (6 to 6)	51 (44 to 60)	15 (11 to 19)	14 (9 to 19)	6 (3 to 11)	17 (17 to 17)
Rwanda	2003	20 (17 to 23)	6 (4 to 8)	5 (4 to 7)	2 (1 to 4)	7 (7 to 7)	59 (51 to 68)	18 (13 to 23)	15 (11 to 21)	6 (3 to 11)	20 (20 to 20)
Rwanda	2004	27 (24 to 31)	7 (5 to 8)	6 (4 to 8)	2 (1 to 4)	13 (13 to 13)	81 (72 to 91)	19 (14 to 25)	18 (12 to 25)	6 (3 to 12)	37 (37 to 37)
Rwanda	2005	31 (27 to 35)	7 (5 to 9)	7 (5 to 10)	3 (1 to 5)	14 (14 to 14)	90 (80 to 103)	22 (16 to 28)	21 (14 to 29)	7 (4 to 15)	41 (41 to 41)
Rwanda	2006	36 (33 to 41)	7 (6 to 10)	7 (5 to 10)	3 (1 to 6)	18 (18 to 18)	107 (96 to 120)	22 (17 to 29)	21 (15 to 30)	9 (4 to 17)	54 (54 to 54)
Rwanda	2007	40 (36 to 45)	8 (6 to 11)	7 (5 to 10)	4 (2 to 7)	20 (20 to 20)	117 (105 to 132)	25 (19 to 32)	21 (14 to 29)	11 (5 to 21)	60 (60 to 60)
Rwanda	2008	50 (46 to 56)	9 (7 to 12)	7 (4 to 9)	4 (2 to 8)	30 (30 to 30)	148 (135 to 164)	27 (20 to 35)	19 (13 to 27)	13 (6 to 24)	89 (89 to 89)

Rwanda	2009	51 (47 to 57)	10 (7 to 13)	6 (4 to 8)	5 (2 to 9)	31 (31 to 31)	151 (138 to 168)	29 (22 to 38)	17 (12 to 24)	14 (6 to 26)	91 (91 to 91)
Rwanda	2010	56 (51 to 62)	11 (8 to 14)	5 (4 to 8)	5 (2 to 10)	34 (34 to 34)	165 (151 to 183)	32 (24 to 42)	16 (11 to 22)	15 (7 to 29)	101 (101 to 101)
Rwanda	2011	58 (53 to 64)	12 (9 to 16)	5 (3 to 7)	6 (3 to 10)	35 (35 to 35)	170 (156 to 188)	36 (27 to 46)	15 (10 to 21)	16 (8 to 30)	104 (104 to 104)
Rwanda	2012	54 (50 to 61)	13 (9 to 17)	4 (3 to 6)	5 (3 to 11)	32 (32 to 32)	160 (147 to 178)	37 (28 to 49)	13 (9 to 19)	16 (8 to 31)	94 (94 to 94)
Rwanda	2013	50 (45 to 56)	13 (10 to 18)	4 (3 to 6)	5 (2 to 10)	27 (27 to 27)	147 (134 to 166)	39 (30 to 52)	12 (8 to 17)	16 (7 to 30)	80 (80 to 80)
Rwanda	2014	54 (49 to 60)	14 (11 to 19)	4 (3 to 5)	5 (2 to 10)	31 (31 to 31)	160 (145 to 177)	42 (32 to 55)	11 (8 to 16)	15 (7 to 29)	91 (91 to 91)
Rwanda	2015	45 (40 to 51)	15 (11 to 19)	4 (3 to 5)	5 (2 to 9)	22 (22 to 22)	133 (118 to 150)	44 (33 to 57)	11 (7 to 15)	15 (7 to 28)	64 (64 to 64)
Rwanda	2016	43 (38 to 49)	15 (11 to 20)	3 (2 to 5)	5 (2 to 9)	19 (19 to 19)	127 (112 to 144)	45 (33 to 59)	10 (7 to 14)	14 (7 to 27)	57 (57 to 57)
Rwanda	2017	45 (39 to 53)	16 (12 to 21)	3 (2 to 5)	5 (2 to 9)	21 (21 to 21)	133 (114 to 157)	47 (35 to 62)	10 (7 to 14)	15 (7 to 27)	61 (61 to 61)
Rwanda	2018	45 (38 to 55)	18 (13 to 23)	4 (2 to 5)	5 (3 to 10)	19 (19 to 19)	134 (112 to 162)	53 (39 to 69)	10 (7 to 14)	16 (8 to 30)	55 (55 to 55)
Rwanda	2019	47 (39 to 57)	19 (14 to 25)	4 (3 to 5)	6 (3 to 11)	18 (18 to 18)	137 (114 to 168)	56 (41 to 73)	11 (8 to 15)	17 (8 to 31)	54 (53 to 54)
Rwanda	2020	48 (40 to 59)	20 (15 to 27)	4 (3 to 5)	6 (3 to 11)	18 (17 to 19)	141 (117 to 175)	60 (44 to 79)	11 (8 to 16)	18 (9 to 33)	52 (50 to 55)
Rwanda	2021	49 (40 to 61)	21 (15 to 28)	4 (3 to 6)	6 (3 to 12)	18 (17 to 19)	144 (117 to 181)	62 (45 to 82)	12 (8 to 16)	19 (9 to 34)	52 (49 to 56)
Rwanda	2022	50 (41 to 63)	22 (16 to 29)	4 (3 to 6)	7 (3 to 12)	17 (16 to 19)	147 (119 to 186)	65 (47 to 86)	12 (9 to 17)	19 (9 to 36)	51 (47 to 57)
Rwanda	2023	51 (41 to 66)	23 (16 to 30)	4 (3 to 6)	7 (3 to 12)	17 (16 to 19)	150 (121 to 193)	67 (48 to 90)	12 (9 to 17)	20 (10 to 37)	51 (46 to 57)

Rwanda	2024	52 (42 to 67)	24 (17 to 32)	4 (3 to 6)	7 (3 to 13)	17 (15 to 19)	153 (123 to 196)	70 (50 to 94)	13 (9 to 18)	21 (10 to 38)	50 (45 to 57)
Rwanda	2025	53 (43 to 68)	25 (18 to 33)	4 (3 to 6)	7 (4 to 13)	17 (15 to 19)	157 (125 to 199)	73 (52 to 97)	13 (9 to 18)	21 (10 to 39)	50 (44 to 57)
Rwanda	2026	54 (43 to 69)	26 (18 to 34)	5 (3 to 6)	7 (4 to 14)	17 (15 to 19)	160 (126 to 204)	75 (54 to 100)	14 (10 to 19)	22 (11 to 41)	49 (43 to 56)
Rwanda	2027	56 (44 to 71)	27 (19 to 36)	5 (3 to 7)	8 (4 to 14)	16 (14 to 19)	164 (128 to 210)	78 (55 to 105)	14 (10 to 19)	23 (11 to 42)	48 (43 to 56)
Rwanda	2028	57 (44 to 73)	28 (19 to 38)	5 (3 to 7)	8 (4 to 15)	16 (14 to 19)	167 (130 to 216)	81 (57 to 111)	14 (10 to 20)	24 (12 to 44)	48 (42 to 56)
Rwanda	2029	58 (45 to 76)	29 (20 to 39)	5 (4 to 7)	8 (4 to 15)	16 (14 to 19)	171 (133 to 223)	84 (59 to 116)	15 (10 to 21)	24 (12 to 45)	48 (41 to 56)
Rwanda	2030	60 (46 to 78)	30 (21 to 41)	5 (4 to 7)	9 (4 to 16)	16 (14 to 19)	175 (136 to 229)	87 (62 to 120)	15 (11 to 21)	25 (12 to 47)	47 (41 to 57)
Rwanda	2031	61 (47 to 81)	31 (21 to 42)	5 (4 to 8)	9 (4 to 16)	16 (14 to 19)	180 (138 to 238)	91 (63 to 125)	16 (11 to 22)	26 (13 to 48)	47 (40 to 56)
Rwanda	2032	63 (48 to 83)	32 (22 to 44)	5 (4 to 8)	9 (5 to 17)	16 (13 to 19)	184 (141 to 244)	94 (65 to 131)	16 (11 to 23)	27 (13 to 50)	47 (40 to 56)
Rwanda	2033	64 (49 to 86)	33 (23 to 46)	6 (4 to 8)	10 (5 to 18)	16 (13 to 19)	189 (145 to 254)	98 (67 to 136)	17 (12 to 24)	28 (14 to 52)	46 (39 to 56)
Rwanda	2034	66 (50 to 88)	35 (24 to 48)	6 (4 to 8)	10 (5 to 18)	16 (13 to 19)	194 (147 to 259)	102 (69 to 142)	17 (12 to 24)	29 (14 to 54)	46 (38 to 56)
Rwanda	2035	68 (52 to 91)	36 (25 to 50)	6 (4 to 9)	10 (5 to 19)	16 (13 to 19)	200 (152 to 267)	106 (72 to 147)	18 (12 to 25)	30 (15 to 56)	46 (38 to 56)
Rwanda	2036	70 (53 to 94)	37 (25 to 52)	6 (4 to 9)	11 (5 to 20)	15 (13 to 19)	205 (155 to 275)	110 (75 to 154)	18 (13 to 26)	32 (15 to 58)	45 (38 to 57)
Rwanda	2037	72 (54 to 96)	39 (26 to 54)	6 (4 to 9)	11 (5 to 20)	15 (13 to 19)	211 (159 to 284)	114 (78 to 159)	19 (13 to 27)	33 (16 to 60)	45 (37 to 55)
Rwanda	2038	74 (55 to 100)	40 (27 to 56)	7 (5 to 9)	12 (6 to 21)	15 (13 to 19)	217 (162 to 295)	119 (80 to 166)	19 (13 to 28)	34 (17 to 63)	45 (37 to 57)

Rwanda	2039	76 (56 to 103)	42 (28 to 59)	7 (5 to 10)	12 (6 to 22)	15 (12 to 19)	223 (166 to 304)	124 (84 to 173)	20 (14 to 29)	35 (17 to 65)	45 (37 to 56)
Rwanda	2040	78 (58 to 108)	44 (30 to 61)	7 (5 to 10)	12 (6 to 23)	15 (12 to 19)	230 (170 to 318)	129 (87 to 180)	21 (14 to 30)	37 (18 to 68)	44 (36 to 56)
Rwanda	2041	81 (59 to 109)	46 (31 to 64)	7 (5 to 10)	13 (6 to 24)	15 (12 to 19)	238 (174 to 322)	134 (91 to 189)	21 (15 to 31)	38 (18 to 70)	44 (36 to 56)
Rwanda	2042	83 (61 to 113)	48 (32 to 68)	7 (5 to 11)	13 (6 to 25)	15 (12 to 19)	245 (180 to 332)	140 (95 to 199)	22 (15 to 32)	39 (19 to 73)	44 (36 to 56)
Rwanda	2043	86 (62 to 117)	50 (33 to 71)	8 (5 to 11)	14 (7 to 26)	15 (12 to 20)	253 (183 to 346)	146 (98 to 208)	23 (15 to 33)	41 (20 to 76)	44 (35 to 58)
Rwanda	2044	89 (64 to 121)	52 (35 to 73)	8 (5 to 11)	14 (7 to 27)	15 (12 to 19)	262 (189 to 357)	153 (102 to 215)	23 (16 to 34)	42 (20 to 79)	44 (35 to 57)
Rwanda	2045	92 (66 to 127)	54 (36 to 77)	8 (6 to 12)	15 (7 to 28)	15 (12 to 19)	270 (196 to 373)	159 (106 to 226)	24 (16 to 35)	44 (21 to 82)	44 (35 to 56)
Rwanda	2046	95 (68 to 130)	56 (38 to 80)	8 (6 to 12)	15 (7 to 29)	15 (12 to 19)	279 (201 to 382)	166 (112 to 235)	25 (17 to 36)	45 (22 to 85)	44 (34 to 57)
Rwanda	2047	98 (71 to 136)	59 (39 to 84)	9 (6 to 13)	16 (8 to 30)	15 (12 to 19)	289 (208 to 400)	173 (115 to 246)	25 (17 to 37)	47 (23 to 88)	43 (34 to 57)
Rwanda	2048	102 (73 to 141)	62 (41 to 88)	9 (6 to 13)	17 (8 to 31)	15 (12 to 20)	299 (215 to 414)	181 (120 to 259)	26 (18 to 38)	49 (24 to 92)	43 (34 to 58)
Rwanda	2049	105 (76 to 145)	64 (43 to 92)	9 (6 to 13)	17 (8 to 32)	15 (12 to 20)	310 (223 to 427)	189 (126 to 270)	27 (18 to 40)	50 (24 to 95)	43 (34 to 58)
Rwanda	2050	109 (78 to 151)	67 (45 to 96)	9 (6 to 14)	18 (9 to 33)	15 (11 to 20)	321 (230 to 446)	198 (132 to 284)	28 (19 to 41)	52 (25 to 98)	43 (34 to 59)
Saint Lucia	1995	533 (485 to 586)	188 (164 to 216)	303 (267 to 344)	43 (26 to 65)	0 (0 to 0)	721 (656 to 793)	254 (222 to 292)	409 (362 to 466)	58 (35 to 88)	0 (0 to 0)
Saint Lucia	1996	571 (529 to 619)	192 (171 to 216)	305 (274 to 337)	43 (27 to 66)	32 (32 to 32)	772 (716 to 837)	259 (231 to 292)	412 (371 to 456)	58 (37 to 90)	43 (43 to 43)
Saint Lucia	1997	533 (497 to 574)	186 (166 to 207)	296 (269 to 326)	42 (27 to 62)	9 (9 to 9)	721 (672 to 777)	251 (224 to 280)	401 (363 to 441)	57 (36 to 84)	12 (12 to 12)

Saint Lucia	1998	526 (489 to 563)	187 (167 to 208)	291 (265 to 318)	42 (28 to 61)	6 (6 to 6)	711 (662 to 762)	252 (226 to 281)	394 (359 to 430)	57 (38 to 83)	8 (8 to 8)
Saint Lucia	1999	531 (494 to 571)	191 (172 to 212)	296 (270 to 324)	43 (29 to 63)	1 (1 to 1)	719 (668 to 772)	258 (232 to 287)	401 (366 to 438)	58 (39 to 85)	1 (1 to 1)
Saint Lucia	2000	522 (485 to 561)	183 (164 to 203)	297 (271 to 326)	42 (28 to 60)	0 (0 to 0)	706 (656 to 759)	248 (222 to 275)	402 (366 to 441)	56 (37 to 81)	1 (1 to 1)
Saint Lucia	2001	512 (478 to 550)	175 (156 to 194)	294 (268 to 324)	42 (28 to 60)	1 (1 to 1)	693 (646 to 745)	237 (211 to 262)	398 (363 to 438)	57 (38 to 81)	1 (1 to 1)
Saint Lucia	2002	523 (489 to 563)	175 (157 to 194)	302 (275 to 331)	45 (30 to 64)	2 (2 to 2)	707 (661 to 761)	237 (212 to 263)	408 (372 to 447)	61 (40 to 87)	2 (2 to 2)
Saint Lucia	2003	530 (495 to 569)	173 (155 to 193)	308 (282 to 338)	46 (31 to 65)	3 (3 to 3)	717 (669 to 769)	234 (210 to 261)	417 (381 to 457)	62 (42 to 88)	5 (5 to 5)
Saint Lucia	2004	539 (505 to 580)	175 (157 to 194)	314 (288 to 344)	46 (31 to 66)	4 (4 to 4)	729 (684 to 785)	236 (213 to 263)	425 (389 to 466)	63 (42 to 89)	6 (6 to 6)
Saint Lucia	2005	570 (533 to 611)	185 (167 to 204)	331 (304 to 362)	48 (33 to 68)	5 (5 to 5)	770 (721 to 826)	250 (225 to 277)	448 (411 to 490)	66 (44 to 93)	7 (7 to 7)
Saint Lucia	2006	570 (532 to 611)	176 (158 to 195)	340 (312 to 371)	45 (31 to 63)	9 (9 to 9)	770 (719 to 827)	238 (214 to 264)	460 (422 to 502)	60 (41 to 85)	12 (12 to 12)
Saint Lucia	2007	563 (527 to 601)	170 (154 to 189)	347 (320 to 376)	41 (28 to 58)	6 (6 to 6)	762 (713 to 813)	230 (208 to 256)	469 (433 to 509)	55 (38 to 79)	7 (7 to 7)
Saint Lucia	2008	599 (563 to 638)	188 (170 to 210)	357 (328 to 388)	42 (28 to 59)	12 (12 to 12)	810 (762 to 863)	255 (230 to 283)	483 (444 to 524)	56 (38 to 79)	16 (16 to 16)
Saint Lucia	2009	614 (576 to 653)	205 (186 to 227)	353 (326 to 382)	42 (28 to 59)	14 (14 to 14)	831 (780 to 883)	277 (251 to 307)	478 (441 to 517)	57 (38 to 80)	19 (19 to 19)
Saint Lucia	2010	591 (553 to 631)	206 (186 to 228)	332 (305 to 361)	41 (28 to 60)	13 (13 to 13)	800 (748 to 853)	279 (252 to 308)	449 (413 to 488)	55 (37 to 80)	17 (17 to 17)
Saint Lucia	2011	708 (668 to 749)	230 (209 to 254)	334 (307 to 364)	45 (30 to 66)	99 (99 to 99)	957 (903 to 1013)	311 (282 to 344)	452 (415 to 492)	61 (41 to 89)	134 (134 to 134)
Saint Lucia	2012	693 (652 to 737)	246 (223 to 272)	323 (294 to 352)	46 (31 to 68)	78 (78 to 78)	937 (882 to 996)	333 (301 to 368)	437 (398 to 476)	62 (42 to 92)	106 (106 to 106)

Saint Lucia	2013	589 (552 to 627)	226 (205 to 249)	289 (262 to 315)	40 (27 to 59)	34 (34 to 34)	797 (747 to 849)	306 (277 to 336)	391 (355 to 426)	54 (36 to 80)	46 (46 to 46)
Saint Lucia	2014	566 (527 to 607)	234 (212 to 258)	270 (244 to 296)	40 (26 to 58)	22 (22 to 22)	765 (713 to 820)	316 (287 to 349)	365 (330 to 400)	54 (36 to 78)	30 (30 to 30)
Saint Lucia	2015	545 (507 to 587)	233 (211 to 257)	255 (228 to 280)	37 (23 to 54)	21 (21 to 21)	738 (686 to 794)	316 (285 to 348)	344 (308 to 379)	49 (31 to 74)	29 (29 to 29)
Saint Lucia	2016	562 (519 to 605)	237 (211 to 262)	252 (225 to 282)	34 (21 to 52)	39 (39 to 39)	760 (702 to 818)	320 (285 to 354)	341 (304 to 381)	47 (29 to 70)	52 (52 to 52)
Saint Lucia	2017	549 (494 to 609)	246 (215 to 278)	244 (211 to 279)	34 (20 to 53)	26 (26 to 26)	743 (668 to 824)	333 (291 to 376)	329 (286 to 377)	46 (27 to 72)	35 (35 to 35)
Saint Lucia	2018	547 (493 to 608)	247 (214 to 282)	241 (209 to 276)	34 (20 to 54)	24 (24 to 25)	740 (667 to 823)	335 (289 to 381)	326 (283 to 373)	47 (27 to 73)	33 (33 to 33)
Saint Lucia	2019	563 (504 to 630)	260 (223 to 299)	242 (211 to 277)	35 (21 to 55)	25 (25 to 26)	762 (682 to 852)	352 (301 to 404)	328 (285 to 375)	47 (28 to 75)	34 (34 to 35)
Saint Lucia	2020	579 (518 to 647)	269 (230 to 308)	248 (215 to 283)	36 (21 to 57)	26 (25 to 28)	783 (700 to 876)	364 (310 to 417)	335 (291 to 383)	49 (29 to 77)	35 (34 to 37)
Saint Lucia	2021	590 (523 to 662)	275 (234 to 317)	250 (217 to 286)	37 (22 to 58)	27 (25 to 29)	798 (707 to 895)	372 (316 to 428)	339 (294 to 387)	50 (29 to 78)	37 (34 to 40)
Saint Lucia	2022	600 (533 to 675)	282 (237 to 327)	252 (219 to 288)	38 (22 to 59)	28 (26 to 31)	812 (721 to 913)	382 (320 to 443)	341 (297 to 390)	51 (30 to 80)	38 (35 to 42)
Saint Lucia	2023	611 (541 to 690)	290 (243 to 338)	254 (221 to 290)	38 (22 to 60)	29 (26 to 32)	826 (731 to 934)	392 (329 to 457)	344 (299 to 393)	52 (30 to 81)	39 (35 to 44)
Saint Lucia	2024	621 (549 to 701)	297 (248 to 349)	256 (222 to 293)	39 (23 to 61)	30 (27 to 33)	841 (742 to 949)	402 (335 to 472)	346 (301 to 396)	52 (31 to 83)	40 (36 to 45)
Saint Lucia	2025	632 (557 to 718)	305 (255 to 359)	257 (224 to 294)	39 (23 to 62)	30 (27 to 35)	855 (753 to 971)	413 (344 to 485)	348 (303 to 398)	53 (31 to 84)	41 (37 to 47)
Saint Lucia	2026	643 (564 to 727)	313 (259 to 371)	258 (225 to 296)	40 (24 to 63)	31 (27 to 36)	869 (762 to 984)	424 (350 to 502)	349 (304 to 401)	54 (32 to 85)	42 (37 to 48)
Saint Lucia	2027	653 (568 to 747)	321 (262 to 381)	259 (225 to 297)	40 (24 to 64)	32 (28 to 37)	883 (769 to 1010)	434 (355 to 516)	350 (305 to 402)	55 (32 to 87)	43 (38 to 50)

Saint Lucia	2028	664 (580 to 753)	330 (268 to 393)	260 (226 to 298)	41 (24 to 65)	33 (28 to 39)	898 (784 to 1018)	446 (362 to 532)	352 (306 to 403)	55 (33 to 88)	45 (38 to 52)
Saint Lucia	2029	674 (585 to 772)	338 (276 to 404)	260 (226 to 299)	41 (24 to 66)	34 (29 to 40)	912 (791 to 1045)	458 (373 to 546)	352 (306 to 405)	56 (33 to 89)	46 (39 to 54)
Saint Lucia	2030	685 (595 to 781)	347 (280 to 414)	261 (226 to 299)	42 (25 to 66)	35 (30 to 42)	926 (805 to 1056)	469 (379 to 559)	353 (306 to 405)	57 (33 to 90)	48 (40 to 57)
Saint Lucia	2031	696 (600 to 800)	356 (284 to 430)	261 (226 to 300)	42 (25 to 67)	36 (31 to 44)	942 (812 to 1082)	482 (384 to 581)	353 (306 to 406)	57 (34 to 91)	49 (41 to 59)
Saint Lucia	2032	707 (611 to 813)	365 (292 to 446)	261 (226 to 301)	43 (25 to 68)	38 (31 to 46)	956 (827 to 1099)	494 (395 to 603)	354 (306 to 407)	58 (34 to 92)	51 (42 to 62)
Saint Lucia	2033	717 (616 to 832)	374 (297 to 455)	261 (226 to 301)	43 (25 to 69)	39 (32 to 48)	970 (833 to 1126)	506 (402 to 615)	354 (306 to 407)	58 (34 to 93)	53 (44 to 65)
Saint Lucia	2034	728 (625 to 841)	383 (303 to 466)	261 (225 to 302)	44 (26 to 69)	40 (33 to 49)	985 (845 to 1137)	518 (410 to 630)	354 (305 to 408)	59 (35 to 94)	55 (45 to 67)
Saint Lucia	2035	740 (631 to 863)	392 (311 to 478)	261 (225 to 302)	44 (26 to 70)	42 (34 to 51)	1000 (854 to 1167)	531 (421 to 647)	354 (305 to 408)	60 (35 to 95)	57 (46 to 69)
Saint Lucia	2036	750 (640 to 870)	401 (312 to 489)	261 (226 to 302)	45 (26 to 71)	43 (34 to 55)	1015 (865 to 1177)	542 (422 to 662)	354 (305 to 409)	60 (35 to 95)	58 (46 to 74)
Saint Lucia	2037	761 (645 to 883)	410 (318 to 503)	262 (225 to 303)	45 (26 to 71)	45 (35 to 56)	1029 (872 to 1195)	554 (431 to 680)	354 (305 to 410)	61 (36 to 96)	60 (47 to 76)
Saint Lucia	2038	771 (646 to 893)	417 (323 to 514)	262 (225 to 304)	45 (27 to 72)	46 (36 to 59)	1042 (874 to 1208)	564 (437 to 695)	354 (304 to 411)	61 (36 to 98)	63 (48 to 79)
Saint Lucia	2039	781 (659 to 912)	425 (328 to 524)	262 (225 to 305)	46 (27 to 73)	47 (36 to 62)	1056 (891 to 1234)	575 (444 to 709)	355 (304 to 412)	62 (36 to 99)	64 (49 to 85)

Saint Lucia	2040	792 (665 to 925)	434 (334 to 539)	263 (225 to 306)	46 (27 to 74)	49 (35 to 64)	1071 (900 to 1251)	587 (452 to 729)	355 (304 to 413)	63 (37 to 100)	66 (48 to 87)
Saint Lucia	2041	803 (669 to 943)	442 (341 to 551)	263 (225 to 306)	47 (27 to 75)	50 (0 to 67)	1086 (904 to 1276)	598 (461 to 746)	356 (305 to 415)	64 (37 to 101)	68 (0 to 90)
Saint Lucia	2042	813 (676 to 957)	451 (346 to 562)	264 (226 to 308)	48 (28 to 76)	51 (0 to 70)	1100 (914 to 1295)	610 (468 to 760)	357 (306 to 416)	64 (38 to 103)	69 (0 to 95)
Saint Lucia	2043	824 (680 to 973)	459 (351 to 578)	265 (226 to 308)	48 (28 to 77)	52 (0 to 74)	1114 (919 to 1315)	621 (475 to 782)	358 (306 to 417)	65 (38 to 104)	70 (0 to 100)
Saint Lucia	2044	834 (692 to 987)	467 (355 to 587)	265 (226 to 309)	49 (28 to 78)	52 (0 to 78)	1128 (936 to 1335)	632 (480 to 794)	359 (306 to 418)	66 (38 to 106)	71 (0 to 105)
Saint Lucia	2045	845 (697 to 1002)	476 (359 to 601)	266 (227 to 310)	49 (29 to 79)	54 (0 to 79)	1143 (942 to 1356)	644 (485 to 813)	360 (307 to 419)	67 (39 to 107)	73 (0 to 107)
Saint Lucia	2046	855 (703 to 1016)	484 (364 to 613)	267 (227 to 311)	50 (29 to 80)	54 (0 to 81)	1157 (951 to 1375)	655 (492 to 829)	361 (308 to 421)	68 (39 to 109)	73 (0 to 109)
Saint Lucia	2047	865 (707 to 1035)	492 (369 to 623)	268 (228 to 313)	51 (29 to 81)	55 (0 to 84)	1171 (956 to 1400)	666 (499 to 842)	362 (308 to 423)	69 (40 to 110)	74 (0 to 114)
Saint Lucia	2048	876 (719 to 1053)	501 (375 to 640)	269 (228 to 314)	51 (30 to 83)	55 (0 to 90)	1185 (973 to 1424)	677 (507 to 865)	364 (309 to 425)	70 (40 to 112)	75 (0 to 121)
Saint Lucia	2049	885 (723 to 1065)	508 (381 to 652)	270 (229 to 316)	52 (30 to 84)	56 (0 to 92)	1197 (978 to 1441)	687 (516 to 882)	365 (310 to 427)	70 (41 to 113)	75 (0 to 124)

Saint Lucia	2050	895 (731 to 1078)	516 (386 to 665)	271 (229 to 317)	53 (31 to 85)	56 (0 to 98)	1211 (989 to 1459)	698 (523 to 899)	366 (310 to 429)	71 (41 to 115)	76 (0 to 132)
Saint Vincent and the Grenadines	1995	266 (234 to 302)	157 (133 to 182)	100 (81 to 127)	5 (3 to 9)	4 (4 to 4)	423 (371 to 480)	249 (212 to 288)	159 (128 to 201)	8 (4 to 15)	6 (6 to 6)
Saint Vincent and the Grenadines	1996	270 (240 to 302)	159 (138 to 180)	100 (81 to 125)	5 (3 to 9)	6 (6 to 6)	429 (381 to 480)	252 (219 to 287)	159 (129 to 198)	9 (4 to 15)	10 (10 to 10)
Saint Vincent and the Grenadines	1997	283 (253 to 311)	172 (150 to 193)	103 (84 to 125)	6 (3 to 10)	1 (1 to 1)	449 (402 to 495)	274 (239 to 307)	164 (134 to 199)	9 (5 to 16)	2 (2 to 2)
Saint Vincent and the Grenadines	1998	271 (243 to 299)	166 (147 to 185)	98 (80 to 118)	6 (3 to 10)	1 (1 to 1)	430 (386 to 474)	263 (233 to 294)	156 (127 to 187)	9 (4 to 17)	2 (2 to 2)
Saint Vincent and the Grenadines	1999	266 (241 to 291)	167 (149 to 186)	92 (76 to 110)	6 (3 to 11)	1 (1 to 1)	422 (382 to 463)	265 (236 to 296)	146 (120 to 175)	10 (5 to 17)	1 (1 to 1)
Saint Vincent and the Grenadines	2000	247 (223 to 272)	161 (144 to 179)	80 (65 to 96)	6 (3 to 11)	0 (0 to 0)	393 (355 to 433)	256 (228 to 285)	127 (103 to 152)	9 (4 to 17)	1 (1 to 1)
Saint Vincent and the Grenadines	2001	243 (221 to 269)	161 (143 to 180)	76 (62 to 91)	6 (3 to 12)	0 (0 to 0)	386 (350 to 427)	255 (227 to 285)	120 (99 to 144)	10 (5 to 18)	1 (1 to 1)

Saint Vincent and the Grenadines	2002	253 (228 to 279)	167 (149 to 188)	77 (64 to 93)	8 (4 to 14)	0 (0 to 0)	402 (362 to 444)	266 (237 to 298)	123 (102 to 148)	13 (6 to 22)	0 (0 to 0)
Saint Vincent and the Grenadines	2003	264 (237 to 289)	171 (153 to 192)	82 (67 to 99)	9 (4 to 16)	2 (2 to 2)	418 (376 to 459)	271 (242 to 304)	130 (107 to 157)	14 (6 to 25)	3 (3 to 3)
Saint Vincent and the Grenadines	2004	263 (238 to 288)	166 (148 to 186)	85 (69 to 103)	9 (4 to 16)	3 (3 to 3)	418 (377 to 458)	263 (235 to 295)	135 (110 to 164)	14 (7 to 26)	5 (5 to 5)
Saint Vincent and the Grenadines	2005	272 (246 to 301)	169 (152 to 190)	90 (74 to 108)	10 (4 to 18)	4 (4 to 4)	432 (391 to 477)	269 (241 to 301)	143 (117 to 172)	15 (7 to 28)	6 (6 to 6)
Saint Vincent and the Grenadines	2006	280 (254 to 307)	167 (148 to 188)	93 (76 to 111)	10 (5 to 18)	10 (10 to 10)	444 (404 to 487)	265 (235 to 299)	148 (120 to 177)	16 (8 to 29)	15 (15 to 15)
Saint Vincent and the Grenadines	2007	288 (260 to 316)	172 (153 to 193)	98 (81 to 117)	11 (6 to 21)	7 (7 to 7)	457 (413 to 503)	274 (243 to 307)	155 (128 to 185)	18 (9 to 33)	11 (11 to 11)
Saint Vincent and the Grenadines	2008	299 (272 to 329)	178 (157 to 199)	100 (83 to 119)	12 (6 to 21)	10 (10 to 10)	475 (431 to 523)	282 (249 to 315)	159 (131 to 189)	18 (9 to 34)	16 (16 to 16)

Saint Vincent and the Grenadines	2009	315 (286 to 344)	188 (167 to 210)	103 (86 to 123)	12 (6 to 23)	12 (12 to 12)	500 (455 to 546)	299 (266 to 333)	163 (136 to 195)	19 (9 to 36)	19 (19 to 19)
Saint Vincent and the Grenadines	2010	314 (286 to 343)	188 (169 to 209)	103 (87 to 123)	12 (6 to 22)	11 (11 to 11)	498 (454 to 544)	298 (268 to 332)	163 (138 to 196)	19 (9 to 35)	18 (18 to 18)
Saint Vincent and the Grenadines	2011	307 (280 to 336)	185 (166 to 207)	102 (85 to 122)	12 (6 to 21)	9 (9 to 9)	488 (445 to 534)	293 (263 to 329)	162 (136 to 194)	18 (9 to 34)	14 (14 to 14)
Saint Vincent and the Grenadines	2012	305 (277 to 333)	180 (161 to 202)	101 (84 to 119)	11 (6 to 19)	13 (13 to 13)	484 (440 to 529)	286 (256 to 321)	160 (134 to 190)	17 (9 to 31)	20 (20 to 20)
Saint Vincent and the Grenadines	2013	327 (298 to 358)	197 (176 to 220)	105 (87 to 124)	11 (6 to 21)	13 (13 to 13)	520 (473 to 568)	313 (280 to 350)	167 (139 to 198)	18 (9 to 33)	21 (21 to 21)
Saint Vincent and the Grenadines	2014	341 (309 to 373)	202 (180 to 226)	106 (88 to 127)	12 (6 to 21)	21 (21 to 21)	541 (491 to 592)	321 (286 to 359)	169 (140 to 202)	18 (9 to 33)	33 (33 to 33)
Saint Vincent and the Grenadines	2015	342 (313 to 372)	192 (171 to 215)	103 (84 to 122)	11 (6 to 19)	37 (37 to 37)	544 (497 to 591)	305 (271 to 341)	163 (134 to 194)	17 (9 to 31)	59 (59 to 59)

Saint Vincent and the Grenadines	2016	339 (307 to 372)	196 (172 to 222)	103 (83 to 125)	11 (6 to 19)	29 (29 to 29)	538 (487 to 590)	312 (273 to 352)	164 (132 to 198)	17 (9 to 30)	45 (45 to 45)
Saint Vincent and the Grenadines	2017	335 (293 to 382)	208 (180 to 239)	105 (83 to 129)	11 (6 to 20)	10 (10 to 10)	532 (465 to 606)	331 (285 to 380)	167 (132 to 205)	18 (9 to 31)	16 (16 to 16)
Saint Vincent and the Grenadines	2018	342 (300 to 391)	214 (185 to 247)	108 (85 to 132)	12 (6 to 20)	10 (9 to 10)	543 (477 to 621)	339 (294 to 392)	171 (135 to 209)	18 (9 to 32)	15 (15 to 15)
Saint Vincent and the Grenadines	2019	350 (307 to 399)	219 (189 to 252)	110 (87 to 135)	12 (6 to 21)	10 (10 to 10)	556 (487 to 634)	347 (300 to 400)	175 (138 to 214)	19 (10 to 33)	15 (15 to 16)
Saint Vincent and the Grenadines	2020	361 (314 to 413)	226 (195 to 261)	113 (89 to 138)	12 (6 to 21)	10 (10 to 11)	573 (499 to 655)	359 (309 to 414)	179 (141 to 219)	19 (10 to 34)	16 (15 to 17)
Saint Vincent and the Grenadines	2021	369 (322 to 421)	232 (198 to 268)	114 (90 to 140)	12 (6 to 22)	10 (10 to 11)	586 (512 to 669)	369 (314 to 426)	181 (143 to 222)	20 (10 to 35)	16 (15 to 18)
Saint Vincent and the Grenadines	2022	377 (330 to 432)	238 (203 to 279)	116 (92 to 142)	13 (7 to 22)	11 (10 to 12)	599 (524 to 686)	378 (322 to 444)	184 (146 to 225)	20 (10 to 35)	17 (15 to 19)

Saint Vincent and the Grenadines	2023	385 (336 to 443)	244 (207 to 286)	118 (93 to 144)	13 (7 to 23)	11 (10 to 12)	612 (533 to 704)	387 (329 to 454)	187 (148 to 229)	21 (11 to 36)	17 (16 to 19)
Saint Vincent and the Grenadines	2024	393 (342 to 454)	249 (212 to 292)	119 (94 to 145)	13 (7 to 23)	11 (10 to 13)	624 (543 to 721)	396 (337 to 464)	189 (150 to 231)	21 (11 to 37)	18 (16 to 20)
Saint Vincent and the Grenadines	2025	400 (347 to 463)	255 (215 to 300)	120 (95 to 147)	13 (7 to 23)	12 (10 to 13)	636 (551 to 736)	405 (342 to 477)	191 (151 to 233)	21 (11 to 37)	18 (16 to 21)
Saint Vincent and the Grenadines	2026	408 (353 to 472)	261 (219 to 308)	122 (96 to 148)	14 (7 to 24)	12 (10 to 14)	647 (561 to 750)	414 (348 to 489)	193 (153 to 236)	21 (11 to 37)	19 (17 to 22)
Saint Vincent and the Grenadines	2027	415 (360 to 480)	266 (224 to 314)	123 (97 to 150)	14 (7 to 24)	12 (11 to 14)	660 (572 to 763)	423 (356 to 499)	195 (154 to 238)	22 (11 to 38)	19 (17 to 23)
Saint Vincent and the Grenadines	2028	423 (365 to 490)	272 (227 to 321)	124 (98 to 152)	14 (7 to 24)	13 (11 to 15)	672 (580 to 778)	432 (361 to 510)	198 (155 to 241)	22 (11 to 39)	20 (18 to 24)
Saint Vincent and the Grenadines	2029	431 (372 to 498)	278 (231 to 328)	126 (99 to 154)	14 (7 to 25)	13 (11 to 15)	684 (591 to 790)	441 (367 to 521)	200 (157 to 244)	22 (12 to 39)	21 (18 to 25)

Saint Vincent and the Grenadines	2030	439 (377 to 507)	283 (235 to 335)	127 (100 to 156)	14 (7 to 25)	14 (11 to 16)	696 (599 to 806)	450 (374 to 532)	202 (159 to 248)	23 (12 to 40)	21 (18 to 26)
Saint Vincent and the Grenadines	2031	446 (384 to 516)	289 (240 to 345)	129 (101 to 158)	15 (8 to 25)	14 (12 to 17)	709 (610 to 820)	459 (381 to 547)	204 (161 to 251)	23 (12 to 40)	22 (19 to 27)
Saint Vincent and the Grenadines	2032	454 (389 to 524)	295 (243 to 350)	130 (102 to 160)	15 (8 to 26)	14 (12 to 18)	721 (617 to 833)	468 (387 to 556)	206 (162 to 254)	23 (12 to 41)	23 (19 to 28)
Saint Vincent and the Grenadines	2033	461 (395 to 534)	300 (247 to 356)	131 (103 to 162)	15 (8 to 26)	15 (13 to 18)	733 (627 to 847)	477 (392 to 566)	208 (163 to 257)	24 (12 to 41)	24 (20 to 29)
Saint Vincent and the Grenadines	2034	469 (399 to 543)	306 (252 to 364)	132 (104 to 164)	15 (8 to 26)	16 (13 to 19)	744 (634 to 863)	485 (401 to 577)	210 (165 to 260)	24 (12 to 42)	25 (20 to 30)
Saint Vincent and the Grenadines	2035	476 (404 to 555)	311 (256 to 375)	133 (105 to 166)	15 (8 to 27)	16 (13 to 20)	756 (642 to 881)	494 (407 to 595)	212 (166 to 263)	24 (13 to 43)	26 (21 to 31)
Saint Vincent and the Grenadines	2036	483 (412 to 564)	316 (259 to 380)	135 (105 to 167)	16 (8 to 27)	17 (13 to 21)	767 (655 to 896)	502 (412 to 603)	214 (167 to 266)	25 (13 to 43)	27 (21 to 33)

Saint Vincent and the Grenadines	2037	490 (417 to 576)	321 (264 to 387)	136 (106 to 169)	16 (8 to 27)	17 (14 to 22)	778 (662 to 914)	510 (419 to 615)	215 (168 to 268)	25 (13 to 44)	28 (22 to 34)
Saint Vincent and the Grenadines	2038	497 (421 to 583)	326 (266 to 396)	137 (107 to 170)	16 (8 to 28)	18 (14 to 23)	789 (669 to 926)	518 (423 to 628)	217 (169 to 269)	25 (13 to 44)	29 (23 to 37)
Saint Vincent and the Grenadines	2039	504 (426 to 591)	331 (272 to 401)	138 (107 to 171)	16 (8 to 28)	19 (15 to 24)	800 (676 to 938)	526 (431 to 637)	219 (170 to 271)	26 (13 to 45)	30 (23 to 38)
Saint Vincent and the Grenadines	2040	511 (432 to 602)	337 (274 to 407)	139 (108 to 172)	16 (8 to 29)	19 (15 to 25)	812 (687 to 956)	535 (435 to 646)	220 (172 to 274)	26 (13 to 46)	31 (24 to 39)
Saint Vincent and the Grenadines	2041	519 (436 to 611)	342 (276 to 416)	140 (109 to 174)	17 (9 to 29)	20 (16 to 26)	824 (692 to 970)	543 (438 to 661)	222 (173 to 276)	26 (14 to 46)	32 (25 to 41)
Saint Vincent and the Grenadines	2042	526 (441 to 624)	348 (280 to 425)	141 (110 to 176)	17 (9 to 29)	21 (16 to 27)	836 (700 to 990)	552 (444 to 675)	224 (174 to 279)	27 (14 to 47)	33 (25 to 43)
Saint Vincent and the Grenadines	2043	534 (444 to 635)	354 (282 to 435)	142 (110 to 177)	17 (9 to 30)	22 (16 to 28)	849 (705 to 1009)	561 (448 to 691)	226 (175 to 281)	27 (14 to 47)	35 (26 to 45)

Saint Vincent and the Grenadines	2044	542 (451 to 648)	359 (286 to 446)	143 (111 to 178)	17 (9 to 30)	23 (16 to 30)	862 (716 to 1029)	571 (455 to 709)	227 (177 to 283)	28 (14 to 48)	36 (26 to 48)
Saint Vincent and the Grenadines	2045	551 (458 to 661)	365 (292 to 454)	144 (112 to 180)	18 (9 to 31)	23 (17 to 31)	875 (727 to 1050)	580 (463 to 720)	229 (178 to 286)	28 (14 to 49)	37 (27 to 50)
Saint Vincent and the Grenadines	2046	559 (466 to 669)	371 (296 to 462)	145 (113 to 181)	18 (9 to 31)	24 (17 to 32)	888 (739 to 1062)	590 (471 to 734)	231 (179 to 288)	28 (14 to 50)	39 (28 to 51)
Saint Vincent and the Grenadines	2047	567 (470 to 678)	377 (299 to 472)	147 (114 to 183)	18 (9 to 32)	25 (18 to 34)	901 (747 to 1077)	599 (475 to 750)	233 (181 to 291)	29 (15 to 50)	40 (29 to 54)
Saint Vincent and the Grenadines	2048	576 (479 to 690)	383 (304 to 479)	148 (115 to 185)	18 (9 to 32)	26 (18 to 35)	915 (760 to 1097)	609 (483 to 761)	235 (182 to 294)	29 (15 to 51)	42 (29 to 56)
Saint Vincent and the Grenadines	2049	585 (483 to 703)	390 (309 to 488)	149 (116 to 187)	19 (9 to 33)	27 (19 to 37)	929 (767 to 1117)	619 (490 to 776)	237 (184 to 297)	30 (15 to 52)	43 (30 to 59)
Saint Vincent and the Grenadines	2050	593 (491 to 714)	396 (313 to 496)	150 (117 to 188)	19 (10 to 33)	28 (19 to 39)	942 (779 to 1133)	629 (498 to 788)	239 (185 to 299)	30 (15 to 53)	45 (31 to 62)
Samoa	1995	151 (129 to 177)	125 (105 to 149)	22 (15 to 30)	0 (0 to 0)	3 (3 to 3)	202 (172 to 236)	168 (141 to 199)	30 (21 to 41)	0 (0 to 0)	4 (4 to 4)

Samoa	1996	152 (131 to 177)	128 (109 to 150)	22 (15 to 30)	0 (0 to 0)	2 (2 to 2)	204 (175 to 236)	172 (146 to 200)	30 (21 to 41)	0 (0 to 0)	2 (2 to 2)
Samoa	1997	141 (122 to 162)	119 (103 to 137)	21 (14 to 28)	0 (0 to 0)	1 (1 to 1)	189 (163 to 216)	159 (138 to 184)	28 (19 to 38)	0 (0 to 0)	1 (1 to 1)
Samoa	1998	139 (122 to 158)	117 (101 to 135)	20 (14 to 27)	0 (0 to 0)	1 (1 to 1)	186 (163 to 212)	157 (135 to 180)	27 (19 to 36)	0 (0 to 0)	2 (2 to 2)
Samoa	1999	153 (135 to 173)	129 (113 to 148)	21 (15 to 28)	0 (0 to 0)	4 (4 to 4)	205 (180 to 232)	173 (151 to 198)	28 (19 to 37)	0 (0 to 0)	5 (5 to 5)
Samoa	2000	162 (145 to 180)	121 (105 to 139)	20 (14 to 26)	0 (0 to 0)	21 (21 to 21)	217 (194 to 241)	162 (141 to 185)	26 (18 to 35)	0 (0 to 0)	29 (29 to 29)
Samoa	2001	153 (135 to 172)	121 (105 to 139)	21 (15 to 28)	0 (0 to 0)	11 (11 to 11)	205 (181 to 230)	162 (140 to 185)	28 (20 to 38)	0 (0 to 0)	15 (15 to 15)
Samoa	2002	161 (143 to 181)	126 (109 to 145)	23 (16 to 31)	0 (0 to 0)	12 (12 to 12)	215 (191 to 242)	169 (146 to 194)	30 (22 to 41)	0 (0 to 0)	16 (16 to 16)
Samoa	2003	169 (151 to 189)	123 (106 to 141)	24 (17 to 32)	0 (0 to 0)	22 (22 to 22)	226 (202 to 252)	165 (142 to 189)	32 (23 to 43)	0 (0 to 0)	30 (30 to 30)
Samoa	2004	176 (158 to 194)	125 (108 to 142)	24 (18 to 33)	0 (0 to 0)	26 (26 to 26)	235 (211 to 260)	167 (145 to 191)	33 (24 to 44)	0 (0 to 0)	35 (35 to 35)
Samoa	2005	196 (175 to 218)	142 (124 to 162)	27 (19 to 36)	0 (0 to 0)	27 (27 to 27)	262 (235 to 292)	190 (165 to 217)	36 (26 to 48)	0 (0 to 0)	36 (36 to 36)
Samoa	2006	207 (189 to 229)	140 (123 to 159)	27 (19 to 37)	0 (0 to 0)	41 (41 to 41)	277 (253 to 306)	187 (164 to 213)	36 (26 to 49)	0 (0 to 0)	54 (54 to 54)
Samoa	2007	211 (190 to 234)	154 (136 to 174)	28 (21 to 39)	0 (0 to 0)	29 (29 to 29)	282 (255 to 312)	206 (182 to 233)	38 (27 to 52)	0 (0 to 0)	38 (38 to 38)
Samoa	2008	207 (188 to 230)	148 (131 to 168)	28 (20 to 37)	0 (0 to 0)	31 (31 to 31)	277 (251 to 307)	199 (175 to 225)	37 (27 to 50)	0 (0 to 0)	41 (41 to 41)
Samoa	2009	216 (196 to 240)	157 (139 to 178)	28 (20 to 38)	0 (0 to 1)	30 (30 to 30)	289 (263 to 321)	211 (186 to 238)	37 (27 to 51)	0 (0 to 1)	41 (41 to 41)
Samoa	2010	265 (244 to 290)	169 (150 to 190)	29 (21 to 39)	1 (0 to 2)	66 (66 to 66)	355 (327 to 388)	227 (201 to 255)	38 (28 to 52)	1 (1 to 2)	88 (88 to 88)

Samoa	2011	215 (194 to 238)	160 (142 to 179)	28 (20 to 38)	1 (1 to 2)	26 (26 to 26)	287 (260 to 318)	214 (189 to 240)	37 (27 to 50)	2 (1 to 3)	34 (34 to 34)
Samoa	2012	260 (240 to 282)	161 (143 to 181)	28 (20 to 38)	2 (1 to 3)	69 (69 to 69)	348 (321 to 378)	216 (191 to 242)	37 (27 to 50)	2 (1 to 4)	93 (93 to 93)
Samoa	2013	262 (241 to 284)	161 (142 to 182)	28 (20 to 38)	2 (1 to 4)	71 (71 to 71)	350 (323 to 380)	215 (190 to 243)	37 (27 to 51)	2 (1 to 5)	96 (96 to 96)
Samoa	2014	227 (204 to 253)	179 (157 to 203)	29 (21 to 39)	2 (1 to 4)	17 (17 to 17)	303 (272 to 338)	239 (211 to 271)	39 (28 to 53)	3 (1 to 5)	23 (23 to 23)
Samoa	2015	248 (225 to 274)	172 (150 to 196)	28 (20 to 38)	2 (1 to 4)	46 (46 to 46)	331 (301 to 366)	229 (200 to 262)	37 (27 to 50)	3 (1 to 5)	62 (62 to 62)
Samoa	2016	228 (202 to 253)	174 (150 to 199)	28 (21 to 38)	2 (1 to 4)	24 (24 to 24)	305 (270 to 339)	232 (200 to 266)	38 (27 to 51)	3 (1 to 5)	32 (32 to 32)
Samoa	2017	222 (194 to 254)	173 (147 to 202)	28 (20 to 38)	2 (1 to 4)	19 (19 to 19)	298 (259 to 339)	232 (197 to 270)	38 (27 to 51)	3 (1 to 5)	26 (26 to 26)
Samoa	2018	217 (189 to 249)	169 (143 to 197)	28 (20 to 39)	2 (1 to 4)	18 (18 to 18)	291 (253 to 333)	226 (191 to 263)	38 (27 to 52)	3 (1 to 5)	25 (25 to 25)
Samoa	2019	228 (198 to 259)	178 (151 to 207)	29 (21 to 40)	2 (1 to 4)	19 (18 to 19)	305 (265 to 347)	238 (202 to 277)	39 (28 to 53)	3 (1 to 5)	25 (25 to 25)
Samoa	2020	236 (205 to 269)	185 (156 to 218)	30 (21 to 41)	2 (1 to 4)	19 (18 to 20)	316 (274 to 360)	248 (209 to 292)	40 (29 to 55)	3 (1 to 5)	25 (24 to 26)
Samoa	2021	239 (207 to 275)	188 (157 to 222)	30 (22 to 41)	2 (1 to 4)	19 (18 to 20)	320 (277 to 367)	251 (210 to 296)	41 (29 to 55)	3 (1 to 5)	25 (24 to 27)
Samoa	2022	241 (206 to 279)	190 (158 to 224)	30 (22 to 42)	2 (1 to 4)	19 (17 to 21)	323 (276 to 374)	254 (211 to 300)	41 (29 to 56)	3 (1 to 6)	25 (23 to 28)
Samoa	2023	243 (209 to 283)	191 (158 to 227)	31 (22 to 42)	2 (1 to 4)	19 (17 to 21)	325 (279 to 379)	255 (212 to 304)	41 (29 to 56)	3 (1 to 6)	26 (23 to 29)
Samoa	2024	244 (208 to 284)	192 (158 to 228)	31 (22 to 42)	2 (1 to 4)	19 (17 to 22)	326 (279 to 380)	257 (212 to 305)	41 (29 to 56)	3 (1 to 6)	26 (23 to 29)
Samoa	2025	245 (210 to 287)	193 (159 to 231)	31 (22 to 42)	2 (1 to 4)	19 (17 to 22)	328 (281 to 385)	258 (213 to 309)	41 (29 to 56)	3 (1 to 6)	26 (23 to 30)

Samoa	2026	246 (210 to 289)	194 (160 to 233)	31 (22 to 42)	2 (1 to 4)	20 (17 to 22)	329 (280 to 387)	259 (214 to 312)	41 (29 to 56)	3 (1 to 6)	26 (23 to 30)
Samoa	2027	247 (209 to 291)	194 (158 to 235)	31 (22 to 42)	2 (1 to 4)	20 (17 to 23)	330 (280 to 389)	260 (212 to 315)	41 (29 to 56)	3 (1 to 6)	26 (23 to 30)
Samoa	2028	247 (210 to 291)	195 (159 to 236)	30 (22 to 42)	2 (1 to 4)	20 (17 to 23)	331 (281 to 389)	260 (213 to 315)	41 (29 to 56)	3 (1 to 6)	27 (23 to 31)
Samoa	2029	248 (208 to 293)	195 (158 to 238)	30 (22 to 42)	2 (1 to 4)	20 (17 to 23)	331 (278 to 392)	261 (212 to 318)	41 (29 to 56)	3 (1 to 6)	27 (23 to 31)
Samoa	2030	248 (209 to 293)	195 (159 to 238)	30 (21 to 41)	2 (1 to 4)	20 (17 to 24)	332 (280 to 393)	261 (213 to 319)	40 (29 to 55)	3 (1 to 6)	27 (23 to 32)
Samoa	2031	249 (208 to 296)	196 (158 to 238)	30 (21 to 41)	2 (1 to 4)	20 (17 to 24)	333 (279 to 396)	262 (212 to 319)	40 (29 to 55)	3 (1 to 6)	27 (23 to 32)
Samoa	2032	249 (207 to 297)	196 (158 to 241)	30 (21 to 41)	2 (1 to 4)	20 (17 to 25)	333 (278 to 397)	262 (212 to 322)	40 (29 to 55)	3 (1 to 6)	27 (23 to 33)
Samoa	2033	250 (208 to 300)	197 (157 to 245)	30 (21 to 41)	2 (1 to 4)	21 (18 to 25)	335 (278 to 402)	264 (210 to 328)	40 (29 to 55)	3 (1 to 6)	28 (24 to 34)
Samoa	2034	251 (208 to 304)	198 (158 to 247)	30 (21 to 41)	2 (1 to 4)	21 (17 to 25)	336 (278 to 407)	265 (211 to 330)	40 (29 to 55)	3 (1 to 6)	28 (23 to 34)
Samoa	2035	252 (208 to 306)	199 (157 to 249)	30 (21 to 41)	2 (1 to 4)	21 (18 to 26)	338 (279 to 409)	266 (211 to 333)	40 (29 to 55)	3 (1 to 6)	28 (23 to 34)
Samoa	2036	253 (207 to 307)	200 (157 to 250)	30 (21 to 41)	2 (1 to 4)	21 (18 to 26)	339 (277 to 411)	267 (210 to 335)	40 (29 to 55)	3 (1 to 6)	28 (23 to 35)
Samoa	2037	254 (207 to 306)	200 (156 to 250)	30 (21 to 41)	2 (1 to 4)	21 (18 to 26)	340 (277 to 409)	268 (208 to 335)	40 (29 to 55)	3 (1 to 6)	29 (24 to 35)
Samoa	2038	256 (209 to 309)	202 (155 to 252)	30 (21 to 41)	2 (1 to 4)	21 (18 to 27)	342 (279 to 414)	270 (207 to 338)	40 (29 to 55)	3 (2 to 6)	29 (24 to 36)
Samoa	2039	258 (207 to 314)	203 (156 to 257)	30 (22 to 41)	2 (1 to 4)	22 (18 to 27)	345 (277 to 420)	272 (209 to 343)	40 (29 to 55)	3 (2 to 6)	29 (24 to 37)
Samoa	2040	260 (211 to 315)	205 (158 to 257)	30 (22 to 42)	2 (1 to 4)	22 (18 to 27)	348 (282 to 421)	275 (211 to 344)	41 (29 to 56)	3 (2 to 6)	29 (24 to 37)

Samoa	2041	262 (212 to 319)	207 (160 to 263)	31 (22 to 42)	2 (1 to 4)	22 (18 to 27)	351 (284 to 427)	277 (214 to 351)	41 (29 to 56)	3 (2 to 6)	29 (24 to 37)
Samoa	2042	265 (213 to 325)	210 (160 to 266)	31 (22 to 42)	2 (1 to 5)	22 (18 to 28)	354 (285 to 435)	280 (214 to 356)	41 (29 to 56)	3 (2 to 6)	29 (24 to 37)
Samoa	2043	267 (216 to 325)	212 (161 to 268)	31 (22 to 42)	3 (1 to 5)	22 (18 to 28)	357 (289 to 435)	283 (215 to 359)	41 (30 to 56)	3 (2 to 6)	29 (24 to 38)
Samoa	2044	270 (215 to 335)	214 (163 to 275)	31 (22 to 42)	3 (1 to 5)	22 (18 to 29)	361 (288 to 448)	287 (218 to 368)	42 (30 to 57)	3 (2 to 6)	29 (24 to 39)
Samoa	2045	273 (221 to 335)	217 (165 to 277)	31 (22 to 43)	3 (1 to 5)	22 (18 to 28)	365 (295 to 448)	290 (221 to 371)	42 (30 to 57)	3 (2 to 6)	30 (24 to 38)
Samoa	2046	275 (221 to 342)	219 (167 to 283)	32 (23 to 43)	3 (1 to 5)	22 (18 to 28)	369 (295 to 457)	293 (223 to 378)	42 (30 to 58)	3 (2 to 6)	30 (24 to 38)
Samoa	2047	278 (223 to 347)	222 (168 to 288)	32 (23 to 43)	3 (1 to 5)	22 (18 to 29)	372 (298 to 465)	296 (225 to 385)	43 (30 to 58)	4 (2 to 6)	30 (24 to 39)
Samoa	2048	281 (222 to 349)	224 (168 to 290)	32 (23 to 44)	3 (1 to 5)	22 (18 to 29)	376 (297 to 466)	299 (225 to 388)	43 (31 to 59)	4 (2 to 7)	30 (24 to 39)
Samoa	2049	283 (224 to 355)	226 (169 to 294)	32 (23 to 44)	3 (1 to 5)	22 (18 to 30)	379 (300 to 475)	303 (226 to 394)	43 (31 to 59)	4 (2 to 7)	30 (24 to 40)
Samoa	2050	286 (228 to 358)	228 (171 to 299)	32 (23 to 44)	3 (1 to 5)	22 (18 to 30)	382 (305 to 480)	306 (229 to 400)	43 (31 to 59)	4 (2 to 7)	30 (24 to 40)
Saudi Arabia	1995	612 (555 to 671)	331 (290 to 379)	198 (166 to 232)	83 (64 to 107)	0 (0 to 0)	1546 (1401 to 1694)	835 (732 to 958)	501 (419 to 585)	210 (161 to 270)	0 (0 to 0)
Saudi Arabia	1996	631 (578 to 681)	355 (316 to 399)	193 (165 to 221)	83 (66 to 104)	0 (0 to 0)	1593 (1460 to 1720)	897 (798 to 1008)	487 (417 to 559)	208 (167 to 262)	0 (0 to 0)
Saudi Arabia	1997	674 (625 to 721)	400 (362 to 442)	190 (166 to 216)	84 (69 to 102)	0 (0 to 0)	1702 (1578 to 1821)	1009 (914 to 1117)	480 (420 to 545)	213 (174 to 258)	0 (0 to 0)

Saudi Arabia	1998	720 (674 to 766)	448 (410 to 489)	186 (164 to 210)	85 (70 to 102)	0 (0 to 0)	1817 (1701 to 1935)	1132 (1035 to 1235)	471 (415 to 530)	215 (177 to 257)	0 (0 to 0)
Saudi Arabia	1999	742 (696 to 788)	489 (451 to 531)	172 (153 to 194)	80 (67 to 97)	0 (0 to 0)	1873 (1759 to 1989)	1235 (1138 to 1342)	434 (386 to 490)	203 (169 to 246)	0 (0 to 0)
Saudi Arabia	2000	824 (781 to 874)	575 (536 to 620)	166 (148 to 188)	83 (68 to 100)	0 (0 to 0)	2081 (1972 to 2208)	1451 (1352 to 1565)	420 (373 to 476)	210 (173 to 252)	0 (0 to 0)
Saudi Arabia	2001	854 (807 to 910)	614 (571 to 660)	159 (140 to 181)	82 (68 to 98)	0 (0 to 0)	2158 (2039 to 2297)	1550 (1442 to 1667)	401 (353 to 456)	208 (172 to 248)	0 (0 to 0)
Saudi Arabia	2002	816 (768 to 866)	592 (549 to 637)	147 (130 to 167)	77 (64 to 92)	0 (0 to 0)	2060 (1939 to 2186)	1494 (1387 to 1609)	371 (329 to 421)	194 (162 to 233)	0 (0 to 0)
Saudi Arabia	2003	797 (750 to 847)	580 (539 to 623)	140 (124 to 159)	77 (65 to 91)	0 (0 to 0)	2013 (1893 to 2140)	1464 (1361 to 1573)	354 (314 to 401)	195 (164 to 230)	0 (0 to 0)
Saudi Arabia	2004	784 (738 to 832)	569 (529 to 611)	135 (120 to 151)	80 (68 to 94)	0 (0 to 0)	1980 (1863 to 2102)	1437 (1337 to 1542)	340 (303 to 382)	203 (171 to 237)	0 (0 to 0)
Saudi Arabia	2005	754 (711 to 800)	543 (505 to 582)	128 (113 to 142)	83 (71 to 96)	0 (0 to 0)	1904 (1795 to 2021)	1371 (1276 to 1470)	322 (286 to 359)	210 (180 to 244)	0 (0 to 0)
Saudi Arabia	2006	741 (700 to 784)	525 (489 to 564)	125 (111 to 140)	90 (77 to 104)	0 (0 to 0)	1870 (1767 to 1980)	1327 (1235 to 1425)	316 (279 to 353)	227 (195 to 263)	0 (0 to 0)
Saudi Arabia	2007	749 (710 to 789)	518 (482 to 554)	128 (114 to 143)	103 (89 to 118)	0 (0 to 0)	1892 (1794 to 1992)	1308 (1217 to 1400)	324 (289 to 360)	259 (224 to 299)	0 (0 to 0)

Saudi Arabia	2008	724 (683 to 762)	481 (448 to 515)	130 (117 to 144)	112 (98 to 128)	0 (0 to 0)	1827 (1725 to 1925)	1215 (1131 to 1301)	328 (295 to 364)	284 (249 to 324)	0 (0 to 0)
Saudi Arabia	2009	824 (780 to 871)	542 (504 to 582)	143 (128 to 159)	139 (122 to 158)	0 (0 to 0)	2081 (1969 to 2200)	1369 (1273 to 1470)	362 (324 to 401)	351 (309 to 398)	0 (0 to 0)
Saudi Arabia	2010	792 (749 to 836)	516 (481 to 556)	138 (123 to 153)	138 (123 to 155)	0 (0 to 0)	2000 (1892 to 2110)	1303 (1215 to 1404)	348 (312 to 386)	349 (310 to 392)	0 (0 to 0)
Saudi Arabia	2011	872 (827 to 919)	582 (543 to 623)	141 (126 to 156)	150 (133 to 168)	0 (0 to 0)	2203 (2089 to 2322)	1469 (1371 to 1573)	355 (319 to 394)	378 (337 to 425)	0 (0 to 0)
Saudi Arabia	2012	940 (896 to 988)	639 (600 to 683)	145 (130 to 161)	156 (139 to 176)	0 (0 to 0)	2374 (2263 to 2496)	1613 (1516 to 1725)	367 (329 to 408)	394 (352 to 443)	0 (0 to 0)
Saudi Arabia	2013	1040 (988 to 1090)	718 (676 to 762)	154 (138 to 170)	169 (151 to 188)	0 (0 to 0)	2627 (2494 to 2753)	1812 (1706 to 1924)	389 (348 to 430)	426 (382 to 475)	0 (0 to 0)
Saudi Arabia	2014	1216 (1163 to 1269)	845 (800 to 892)	172 (154 to 190)	199 (179 to 221)	0 (0 to 0)	3070 (2936 to 3206)	2134 (2020 to 2253)	434 (389 to 480)	502 (453 to 557)	0 (0 to 0)
Saudi Arabia	2015	1324 (1267 to 1383)	907 (859 to 955)	191 (172 to 212)	226 (205 to 249)	0 (0 to 0)	3343 (3201 to 3492)	2289 (2170 to 2412)	483 (434 to 535)	571 (517 to 628)	0 (0 to 0)
Saudi Arabia	2016	1315 (1251 to 1376)	882 (827 to 938)	200 (178 to 222)	234 (210 to 258)	0 (0 to 0)	3321 (3158 to 3476)	2227 (2088 to 2368)	505 (450 to 561)	590 (531 to 653)	0 (0 to 0)
Saudi Arabia	2017	1206 (1107 to 1310)	789 (724 to 853)	196 (172 to 222)	222 (191 to 254)	0 (0 to 0)	3046 (2796 to 3307)	1993 (1829 to 2153)	494 (434 to 560)	560 (482 to 641)	0 (0 to 0)

Saudi Arabia	2018	1309 (1200 to 1429)	875 (799 to 953)	197 (174 to 224)	237 (204 to 272)	0 (0 to 0)	3306 (3031 to 3609)	2209 (2019 to 2407)	499 (438 to 567)	599 (515 to 686)	0 (0 to 0)
Saudi Arabia	2019	1382 (1259 to 1507)	940 (853 to 1025)	196 (172 to 223)	246 (212 to 282)	0 (0 to 0)	3489 (3179 to 3807)	2373 (2153 to 2589)	495 (434 to 563)	622 (535 to 713)	0 (0 to 0)
Saudi Arabia	2020	1422 (1293 to 1561)	973 (877 to 1071)	197 (173 to 224)	252 (217 to 288)	0 (0 to 0)	3590 (3265 to 3941)	2457 (2215 to 2705)	498 (437 to 567)	635 (547 to 728)	0 (0 to 0)
Saudi Arabia	2021	1427 (1294 to 1569)	977 (885 to 1076)	197 (173 to 225)	252 (217 to 290)	0 (0 to 0)	3604 (3268 to 3961)	2468 (2234 to 2717)	499 (437 to 568)	637 (548 to 731)	0 (0 to 0)
Saudi Arabia	2022	1433 (1293 to 1580)	982 (882 to 1093)	198 (173 to 226)	253 (218 to 291)	0 (0 to 0)	3620 (3266 to 3991)	2481 (2226 to 2761)	499 (437 to 570)	640 (549 to 736)	0 (0 to 0)
Saudi Arabia	2023	1493 (1346 to 1653)	1033 (922 to 1152)	198 (174 to 226)	262 (225 to 301)	0 (0 to 0)	3771 (3398 to 4175)	2610 (2328 to 2910)	500 (438 to 571)	661 (568 to 761)	0 (0 to 0)
Saudi Arabia	2024	1555 (1396 to 1716)	1087 (968 to 1209)	198 (174 to 226)	270 (232 to 311)	0 (0 to 0)	3927 (3525 to 4334)	2744 (2444 to 3054)	501 (439 to 571)	682 (585 to 786)	0 (0 to 0)
Saudi Arabia	2025	1613 (1448 to 1789)	1137 (1014 to 1262)	199 (174 to 226)	277 (238 to 320)	0 (0 to 0)	4073 (3657 to 4518)	2871 (2560 to 3188)	502 (439 to 570)	700 (600 to 809)	0 (0 to 0)
Saudi Arabia	2026	1646 (1477 to 1833)	1166 (1039 to 1306)	199 (174 to 226)	281 (241 to 325)	0 (0 to 0)	4157 (3729 to 4628)	2944 (2623 to 3298)	502 (438 to 571)	711 (608 to 822)	0 (0 to 0)
Saudi Arabia	2027	1681 (1503 to 1873)	1196 (1065 to 1342)	199 (174 to 227)	286 (244 to 330)	0 (0 to 0)	4245 (3796 to 4729)	3020 (2688 to 3388)	503 (439 to 572)	722 (617 to 834)	0 (0 to 0)

Saudi Arabia	2028	1716 (1531 to 1935)	1226 (1085 to 1398)	199 (174 to 226)	290 (248 to 336)	0 (0 to 0)	4333 (3866 to 4885)	3097 (2739 to 3530)	503 (439 to 572)	733 (626 to 847)	0 (0 to 0)
Saudi Arabia	2029	1751 (1554 to 2002)	1257 (1104 to 1453)	199 (174 to 227)	295 (251 to 340)	0 (0 to 0)	4422 (3925 to 5055)	3175 (2789 to 3669)	503 (439 to 574)	744 (634 to 860)	0 (0 to 0)
Saudi Arabia	2030	1786 (1577 to 2080)	1288 (1123 to 1526)	199 (174 to 228)	299 (254 to 345)	0 (0 to 0)	4511 (3983 to 5253)	3253 (2837 to 3853)	503 (438 to 575)	755 (642 to 871)	0 (0 to 0)
Saudi Arabia	2031	1821 (1596 to 2157)	1319 (1143 to 1597)	199 (173 to 228)	303 (258 to 351)	0 (0 to 0)	4599 (4030 to 5447)	3330 (2886 to 4033)	503 (437 to 576)	766 (652 to 886)	0 (0 to 0)
Saudi Arabia	2032	1855 (1620 to 2238)	1349 (1158 to 1672)	199 (173 to 229)	308 (262 to 356)	0 (0 to 0)	4685 (4090 to 5651)	3406 (2924 to 4223)	503 (436 to 578)	777 (661 to 900)	0 (0 to 0)
Saudi Arabia	2033	1887 (1636 to 2311)	1377 (1173 to 1739)	199 (172 to 230)	311 (264 to 361)	0 (0 to 0)	4764 (4131 to 5835)	3476 (2962 to 4391)	502 (435 to 582)	786 (668 to 913)	0 (0 to 0)
Saudi Arabia	2034	1917 (1653 to 2385)	1404 (1187 to 1820)	198 (172 to 231)	315 (267 to 366)	0 (0 to 0)	4841 (4175 to 6023)	3545 (2997 to 4595)	501 (433 to 584)	795 (675 to 924)	0 (0 to 0)
Saudi Arabia	2035	1946 (1668 to 2463)	1430 (1202 to 1888)	198 (171 to 231)	318 (270 to 370)	0 (0 to 0)	4913 (4211 to 6220)	3611 (3036 to 4769)	500 (431 to 583)	803 (681 to 934)	0 (0 to 0)
Saudi Arabia	2036	1972 (1678 to 2539)	1454 (1212 to 1965)	197 (170 to 231)	321 (272 to 373)	0 (0 to 0)	4980 (4238 to 6413)	3672 (3060 to 4961)	498 (429 to 582)	810 (686 to 942)	0 (0 to 0)
Saudi Arabia	2037	1998 (1690 to 2618)	1478 (1220 to 2037)	196 (169 to 231)	324 (273 to 377)	0 (0 to 0)	5045 (4267 to 6612)	3731 (3080 to 5143)	496 (427 to 584)	817 (689 to 953)	0 (0 to 0)

Saudi Arabia	2038	2021 (1699 to 2701)	1499 (1227 to 2114)	196 (168 to 232)	326 (274 to 381)	0 (0 to 0)	5104 (4291 to 6820)	3787 (3099 to 5339)	494 (424 to 585)	824 (693 to 963)	0 (0 to 0)
Saudi Arabia	2039	2042 (1707 to 2772)	1519 (1232 to 2188)	195 (166 to 231)	328 (276 to 384)	0 (0 to 0)	5156 (4311 to 7001)	3836 (3110 to 5524)	491 (420 to 584)	829 (696 to 969)	0 (0 to 0)
Saudi Arabia	2040	2059 (1709 to 2839)	1536 (1234 to 2254)	193 (165 to 232)	330 (277 to 386)	0 (0 to 0)	5199 (4315 to 7168)	3879 (3116 to 5691)	488 (416 to 586)	832 (700 to 974)	0 (0 to 0)
Saudi Arabia	2041	2073 (1708 to 2906)	1551 (1235 to 2318)	192 (163 to 232)	331 (278 to 388)	0 (0 to 0)	5235 (4313 to 7338)	3916 (3118 to 5854)	484 (412 to 586)	835 (701 to 980)	0 (0 to 0)
Saudi Arabia	2042	2084 (1708 to 2963)	1563 (1235 to 2374)	190 (162 to 232)	331 (278 to 389)	0 (0 to 0)	5264 (4313 to 7482)	3947 (3120 to 5996)	481 (408 to 585)	837 (702 to 983)	0 (0 to 0)
Saudi Arabia	2043	2092 (1700 to 3023)	1572 (1235 to 2434)	189 (160 to 231)	332 (278 to 389)	0 (0 to 0)	5284 (4293 to 7634)	3971 (3118 to 6147)	476 (404 to 584)	837 (702 to 983)	0 (0 to 0)
Saudi Arabia	2044	2097 (1694 to 3065)	1579 (1232 to 2479)	187 (158 to 230)	331 (277 to 389)	0 (0 to 0)	5294 (4279 to 7739)	3987 (3110 to 6261)	471 (399 to 581)	836 (699 to 982)	0 (0 to 0)
Saudi Arabia	2045	2097 (1681 to 3103)	1582 (1224 to 2533)	185 (156 to 228)	330 (276 to 389)	0 (0 to 0)	5294 (4245 to 7835)	3994 (3091 to 6396)	466 (394 to 576)	834 (696 to 982)	0 (0 to 0)
Saudi Arabia	2046	2093 (1671 to 3147)	1582 (1217 to 2566)	182 (154 to 227)	329 (274 to 388)	0 (0 to 0)	5286 (4220 to 7946)	3996 (3074 to 6480)	460 (389 to 574)	831 (692 to 981)	0 (0 to 0)
Saudi Arabia	2047	2088 (1653 to 3166)	1580 (1209 to 2594)	180 (152 to 226)	327 (272 to 387)	0 (0 to 0)	5272 (4174 to 7994)	3991 (3053 to 6549)	455 (384 to 570)	827 (687 to 976)	0 (0 to 0)

Saudi Arabia	2048	2080 (1643 to 3179)	1577 (1201 to 2615)	178 (150 to 224)	326 (270 to 385)	0 (0 to 0)	5254 (4149 to 8028)	3982 (3033 to 6605)	449 (378 to 566)	822 (681 to 973)	0 (0 to 0)
Saudi Arabia	2049	2071 (1630 to 3191)	1572 (1192 to 2639)	176 (148 to 222)	324 (268 to 384)	0 (0 to 0)	5231 (4116 to 8059)	3970 (3009 to 6664)	444 (373 to 560)	817 (676 to 969)	0 (0 to 0)
Saudi Arabia	2050	2060 (1610 to 3224)	1565 (1181 to 2640)	173 (146 to 219)	321 (265 to 382)	0 (0 to 0)	5202 (4067 to 8142)	3952 (2982 to 6666)	438 (368 to 554)	812 (669 to 964)	0 (0 to 0)
Senegal	1995	44 (36 to 54)	13 (10 to 17)	23 (16 to 32)	4 (2 to 7)	4 (4 to 4)	114 (93 to 141)	33 (25 to 43)	59 (42 to 83)	10 (5 to 18)	11 (11 to 11)
Senegal	1996	43 (35 to 54)	13 (10 to 17)	23 (16 to 32)	4 (2 to 7)	3 (3 to 3)	113 (91 to 140)	34 (26 to 45)	59 (42 to 83)	10 (5 to 18)	9 (9 to 9)
Senegal	1997	44 (36 to 54)	13 (10 to 17)	22 (16 to 31)	4 (2 to 7)	5 (5 to 5)	115 (94 to 140)	33 (25 to 43)	58 (41 to 80)	9 (5 to 17)	14 (14 to 14)
Senegal	1998	45 (37 to 54)	13 (10 to 17)	22 (16 to 31)	3 (2 to 6)	7 (7 to 7)	117 (97 to 141)	33 (25 to 43)	58 (41 to 80)	9 (4 to 16)	17 (17 to 17)
Senegal	1999	46 (38 to 55)	14 (10 to 18)	23 (16 to 31)	3 (1 to 6)	6 (6 to 6)	119 (98 to 144)	36 (27 to 47)	59 (42 to 81)	8 (4 to 15)	15 (15 to 15)
Senegal	2000	43 (35 to 52)	14 (10 to 18)	22 (16 to 30)	3 (1 to 5)	5 (5 to 5)	111 (92 to 136)	35 (27 to 46)	57 (41 to 78)	7 (3 to 13)	12 (12 to 12)
Senegal	2001	48 (40 to 58)	15 (12 to 20)	23 (16 to 31)	3 (1 to 5)	7 (7 to 7)	126 (105 to 152)	40 (30 to 52)	60 (42 to 82)	7 (3 to 13)	19 (19 to 19)
Senegal	2002	45 (38 to 55)	15 (11 to 19)	22 (16 to 31)	3 (1 to 5)	5 (5 to 5)	118 (98 to 143)	39 (29 to 50)	59 (42 to 80)	7 (3 to 13)	14 (14 to 14)
Senegal	2003	53 (45 to 63)	16 (12 to 21)	23 (17 to 31)	3 (2 to 6)	11 (11 to 11)	138 (117 to 163)	42 (32 to 55)	60 (43 to 82)	8 (4 to 15)	29 (29 to 29)
Senegal	2004	55 (48 to 65)	17 (13 to 22)	22 (16 to 30)	4 (2 to 7)	13 (13 to 13)	145 (124 to 169)	44 (33 to 57)	58 (42 to 79)	9 (5 to 17)	33 (33 to 33)
Senegal	2005	53 (46 to 63)	17 (13 to 23)	20 (14 to 27)	4 (2 to 7)	12 (12 to 12)	139 (119 to 163)	45 (34 to 59)	52 (37 to 72)	11 (5 to 19)	31 (31 to 31)

Senegal	2006	55 (47 to 64)	18 (14 to 23)	19 (14 to 27)	4 (2 to 8)	13 (13 to 13)	144 (124 to 168)	47 (35 to 61)	50 (36 to 69)	12 (6 to 22)	35 (35 to 35)
Senegal	2007	49 (42 to 59)	17 (13 to 22)	19 (14 to 27)	5 (2 to 9)	8 (8 to 8)	128 (108 to 153)	45 (34 to 58)	50 (36 to 69)	12 (6 to 24)	21 (21 to 21)
Senegal	2008	51 (43 to 60)	16 (12 to 21)	19 (14 to 26)	5 (2 to 9)	11 (11 to 11)	133 (113 to 156)	42 (32 to 55)	51 (36 to 69)	12 (6 to 24)	28 (28 to 28)
Senegal	2009	54 (46 to 63)	16 (12 to 21)	21 (15 to 29)	5 (2 to 9)	12 (12 to 12)	140 (119 to 165)	41 (31 to 54)	55 (39 to 75)	13 (6 to 24)	31 (31 to 31)
Senegal	2010	53 (44 to 63)	15 (11 to 20)	23 (16 to 31)	5 (2 to 9)	10 (10 to 10)	138 (115 to 164)	40 (30 to 51)	60 (43 to 81)	13 (6 to 24)	26 (26 to 26)
Senegal	2011	57 (48 to 68)	15 (12 to 20)	25 (18 to 34)	5 (2 to 9)	11 (11 to 11)	148 (125 to 176)	40 (30 to 52)	65 (48 to 90)	13 (6 to 24)	29 (29 to 29)
Senegal	2012	59 (50 to 71)	15 (11 to 19)	27 (19 to 37)	5 (2 to 9)	13 (13 to 13)	154 (130 to 184)	39 (29 to 50)	69 (50 to 96)	12 (6 to 23)	34 (34 to 34)
Senegal	2013	58 (48 to 69)	14 (11 to 18)	28 (20 to 38)	4 (2 to 8)	11 (11 to 11)	150 (125 to 180)	37 (28 to 47)	72 (51 to 99)	12 (5 to 22)	30 (30 to 30)
Senegal	2014	60 (50 to 72)	15 (11 to 19)	29 (20 to 39)	4 (2 to 8)	13 (13 to 13)	157 (131 to 187)	38 (28 to 48)	75 (53 to 102)	11 (5 to 22)	33 (33 to 33)
Senegal	2015	60 (50 to 72)	15 (11 to 19)	30 (21 to 41)	4 (2 to 8)	11 (11 to 11)	156 (129 to 189)	38 (28 to 49)	78 (55 to 108)	11 (5 to 21)	30 (30 to 30)
Senegal	2016	60 (49 to 73)	15 (11 to 19)	31 (22 to 43)	5 (2 to 8)	10 (10 to 10)	156 (128 to 189)	39 (29 to 50)	80 (56 to 111)	12 (6 to 22)	25 (25 to 25)
Senegal	2017	65 (55 to 78)	14 (10 to 18)	31 (22 to 43)	5 (2 to 9)	15 (15 to 15)	170 (142 to 203)	37 (27 to 48)	80 (56 to 112)	13 (6 to 24)	40 (40 to 40)
Senegal	2018	66 (55 to 78)	15 (11 to 19)	32 (22 to 44)	5 (2 to 10)	14 (14 to 14)	171 (143 to 204)	38 (28 to 49)	83 (58 to 115)	13 (6 to 25)	37 (37 to 37)
Senegal	2019	68 (56 to 82)	16 (12 to 20)	33 (23 to 45)	5 (2 to 10)	14 (14 to 14)	176 (146 to 213)	41 (30 to 53)	85 (59 to 118)	14 (6 to 26)	37 (36 to 37)
Senegal	2020	70 (58 to 84)	16 (12 to 21)	34 (24 to 47)	6 (3 to 10)	14 (13 to 15)	181 (152 to 218)	42 (31 to 55)	88 (61 to 122)	15 (7 to 27)	37 (35 to 39)

Senegal	2021	70 (58 to 85)	16 (12 to 21)	34 (24 to 48)	6 (3 to 10)	14 (13 to 15)	183 (150 to 222)	43 (32 to 55)	89 (62 to 124)	15 (7 to 27)	37 (34 to 40)
Senegal	2022	71 (59 to 86)	16 (12 to 21)	35 (24 to 48)	6 (3 to 11)	14 (13 to 16)	185 (154 to 223)	43 (31 to 55)	90 (63 to 125)	15 (7 to 28)	37 (33 to 40)
Senegal	2023	71 (59 to 86)	16 (12 to 21)	35 (25 to 49)	6 (3 to 11)	14 (13 to 16)	186 (153 to 225)	43 (31 to 55)	92 (64 to 127)	15 (7 to 28)	36 (33 to 41)
Senegal	2024	72 (60 to 86)	17 (12 to 21)	36 (25 to 49)	6 (3 to 11)	14 (12 to 16)	187 (157 to 225)	43 (32 to 56)	93 (65 to 129)	15 (7 to 28)	36 (32 to 41)
Senegal	2025	72 (60 to 88)	17 (12 to 21)	36 (25 to 50)	6 (3 to 11)	14 (12 to 16)	189 (156 to 229)	43 (32 to 56)	94 (66 to 130)	15 (7 to 28)	36 (32 to 41)
Senegal	2026	73 (60 to 88)	17 (12 to 22)	37 (26 to 51)	6 (3 to 11)	14 (12 to 16)	190 (156 to 229)	43 (32 to 56)	95 (67 to 132)	16 (7 to 29)	36 (32 to 41)
Senegal	2027	74 (60 to 90)	17 (12 to 22)	37 (26 to 52)	6 (3 to 11)	14 (12 to 16)	192 (156 to 233)	44 (32 to 57)	97 (67 to 134)	16 (7 to 29)	36 (31 to 42)
Senegal	2028	74 (62 to 90)	17 (12 to 22)	38 (26 to 52)	6 (3 to 11)	14 (12 to 16)	193 (160 to 234)	44 (32 to 57)	98 (68 to 137)	16 (7 to 29)	36 (31 to 42)
Senegal	2029	75 (62 to 91)	17 (12 to 22)	38 (27 to 53)	6 (3 to 11)	14 (12 to 16)	195 (162 to 236)	44 (32 to 57)	99 (69 to 139)	16 (8 to 30)	35 (31 to 42)
Senegal	2030	76 (62 to 92)	17 (12 to 22)	39 (27 to 54)	6 (3 to 12)	14 (12 to 16)	197 (161 to 240)	44 (32 to 58)	101 (71 to 141)	16 (8 to 30)	35 (30 to 42)
Senegal	2031	77 (63 to 94)	17 (13 to 22)	39 (28 to 55)	6 (3 to 12)	14 (11 to 16)	199 (164 to 246)	45 (33 to 59)	103 (72 to 143)	17 (8 to 31)	35 (30 to 42)
Senegal	2032	78 (64 to 95)	17 (13 to 23)	40 (28 to 56)	6 (3 to 12)	13 (11 to 16)	202 (166 to 247)	45 (33 to 60)	105 (73 to 146)	17 (8 to 31)	35 (30 to 42)
Senegal	2033	79 (64 to 98)	18 (13 to 23)	41 (29 to 57)	7 (3 to 12)	13 (11 to 16)	206 (167 to 254)	46 (34 to 61)	107 (75 to 149)	17 (8 to 32)	35 (29 to 42)
Senegal	2034	80 (66 to 98)	18 (13 to 24)	42 (29 to 58)	7 (3 to 13)	13 (11 to 17)	209 (171 to 256)	47 (34 to 62)	110 (77 to 152)	18 (8 to 33)	35 (29 to 43)
Senegal	2035	82 (67 to 101)	18 (13 to 24)	43 (30 to 60)	7 (3 to 13)	13 (11 to 16)	212 (174 to 262)	48 (35 to 63)	112 (78 to 155)	18 (9 to 33)	35 (28 to 42)

Senegal	2036	83 (68 to 101)	19 (14 to 25)	44 (31 to 61)	7 (3 to 13)	13 (11 to 17)	216 (176 to 263)	49 (35 to 64)	114 (80 to 159)	18 (9 to 34)	35 (28 to 43)
Senegal	2037	84 (70 to 104)	19 (14 to 25)	45 (31 to 62)	7 (3 to 13)	13 (11 to 16)	220 (181 to 270)	50 (36 to 65)	117 (82 to 162)	19 (9 to 35)	34 (27 to 42)
Senegal	2038	86 (71 to 105)	20 (14 to 26)	46 (32 to 64)	7 (3 to 14)	13 (10 to 17)	224 (184 to 274)	51 (37 to 67)	119 (84 to 165)	19 (9 to 36)	34 (27 to 44)
Senegal	2039	88 (71 to 108)	20 (14 to 27)	47 (33 to 65)	8 (4 to 14)	13 (10 to 17)	228 (185 to 282)	52 (38 to 69)	122 (85 to 169)	20 (9 to 37)	34 (27 to 44)
Senegal	2040	89 (72 to 110)	20 (15 to 27)	48 (34 to 66)	8 (4 to 15)	13 (10 to 17)	233 (187 to 287)	53 (39 to 71)	125 (87 to 173)	20 (10 to 38)	34 (27 to 45)
Senegal	2041	91 (74 to 111)	21 (15 to 28)	49 (34 to 68)	8 (4 to 15)	13 (10 to 17)	237 (193 to 290)	55 (40 to 73)	127 (89 to 176)	21 (10 to 39)	34 (26 to 44)
Senegal	2042	93 (75 to 114)	22 (16 to 29)	50 (35 to 69)	8 (4 to 15)	13 (10 to 17)	242 (196 to 296)	56 (40 to 75)	130 (91 to 180)	21 (10 to 40)	34 (26 to 44)
Senegal	2043	95 (76 to 117)	22 (16 to 30)	51 (36 to 70)	8 (4 to 16)	13 (10 to 17)	247 (198 to 306)	58 (41 to 78)	133 (93 to 183)	22 (10 to 41)	34 (26 to 44)
Senegal	2044	97 (79 to 119)	23 (16 to 31)	52 (37 to 72)	9 (4 to 16)	13 (10 to 17)	252 (206 to 310)	60 (43 to 80)	136 (95 to 187)	23 (11 to 42)	34 (25 to 45)
Senegal	2045	99 (79 to 123)	24 (17 to 32)	53 (37 to 73)	9 (4 to 17)	13 (10 to 17)	257 (206 to 321)	62 (44 to 83)	138 (97 to 191)	23 (11 to 44)	34 (25 to 46)
Senegal	2046	101 (82 to 123)	24 (17 to 33)	54 (38 to 75)	9 (4 to 17)	13 (10 to 17)	263 (213 to 321)	64 (45 to 86)	141 (99 to 196)	24 (11 to 45)	34 (25 to 45)
Senegal	2047	103 (82 to 129)	25 (18 to 34)	55 (39 to 77)	9 (4 to 18)	13 (10 to 18)	269 (213 to 335)	66 (46 to 89)	144 (101 to 200)	25 (12 to 46)	34 (25 to 46)
Senegal	2048	105 (86 to 129)	26 (18 to 35)	56 (40 to 78)	10 (5 to 18)	13 (9 to 18)	275 (224 to 337)	68 (48 to 92)	147 (103 to 204)	25 (12 to 48)	34 (25 to 46)
Senegal	2049	108 (87 to 134)	27 (19 to 37)	58 (40 to 80)	10 (5 to 19)	13 (9 to 18)	281 (226 to 350)	70 (49 to 97)	150 (105 to 209)	26 (12 to 49)	34 (24 to 47)
Senegal	2050	110 (89 to 136)	28 (20 to 38)	59 (41 to 82)	10 (5 to 20)	13 (9 to 18)	287 (231 to 354)	73 (51 to 100)	153 (107 to 213)	27 (13 to 51)	34 (25 to 47)

Serbia	1995	153 (134 to 175)	100 (85 to 117)	46 (36 to 58)	7 (4 to 13)	0 (0 to 0)	384 (336 to 437)	250 (212 to 293)	115 (90 to 146)	18 (9 to 33)	0 (0 to 0)
Serbia	1996	156 (139 to 176)	102 (88 to 117)	47 (37 to 58)	7 (4 to 13)	0 (0 to 0)	390 (348 to 439)	254 (221 to 292)	117 (92 to 145)	18 (9 to 34)	0 (0 to 0)
Serbia	1997	168 (151 to 188)	111 (98 to 126)	49 (40 to 61)	8 (4 to 14)	0 (0 to 0)	421 (378 to 471)	277 (244 to 315)	124 (99 to 152)	20 (10 to 36)	0 (0 to 0)
Serbia	1998	175 (158 to 194)	115 (102 to 130)	51 (41 to 62)	8 (4 to 15)	0 (0 to 0)	438 (395 to 486)	288 (255 to 325)	129 (103 to 154)	21 (11 to 37)	0 (0 to 0)
Serbia	1999	173 (157 to 191)	113 (100 to 126)	51 (41 to 61)	8 (4 to 14)	2 (2 to 2)	434 (392 to 478)	282 (251 to 316)	127 (104 to 152)	20 (11 to 36)	4 (4 to 4)
Serbia	2000	186 (168 to 203)	122 (110 to 136)	53 (44 to 63)	9 (4 to 15)	2 (2 to 2)	465 (420 to 507)	306 (275 to 341)	133 (109 to 158)	22 (11 to 38)	5 (5 to 5)
Serbia	2001	205 (186 to 224)	137 (123 to 152)	57 (47 to 68)	9 (5 to 16)	2 (2 to 2)	513 (464 to 561)	343 (308 to 381)	143 (117 to 170)	23 (12 to 40)	4 (4 to 4)
Serbia	2002	258 (234 to 283)	178 (161 to 198)	67 (55 to 79)	11 (6 to 20)	3 (3 to 3)	646 (587 to 707)	445 (402 to 495)	167 (137 to 197)	28 (14 to 49)	7 (7 to 7)
Serbia	2003	279 (255 to 303)	189 (172 to 210)	74 (62 to 87)	11 (6 to 20)	4 (4 to 4)	697 (637 to 759)	474 (430 to 525)	184 (154 to 217)	29 (15 to 50)	10 (10 to 10)
Serbia	2004	302 (277 to 328)	202 (183 to 223)	85 (72 to 99)	12 (6 to 21)	3 (3 to 3)	756 (693 to 820)	506 (459 to 558)	213 (181 to 249)	30 (16 to 52)	8 (8 to 8)
Serbia	2005	330 (304 to 357)	216 (195 to 236)	100 (85 to 116)	13 (7 to 22)	2 (2 to 2)	826 (761 to 893)	539 (488 to 592)	249 (214 to 290)	32 (17 to 55)	6 (6 to 6)
Serbia	2006	379 (349 to 410)	242 (221 to 264)	120 (103 to 138)	14 (7 to 24)	3 (3 to 3)	947 (874 to 1025)	606 (553 to 662)	299 (258 to 345)	35 (18 to 60)	7 (7 to 7)
Serbia	2007	414 (381 to 445)	260 (237 to 282)	138 (120 to 158)	14 (7 to 25)	2 (2 to 2)	1035 (954 to 1114)	650 (593 to 707)	346 (300 to 395)	35 (18 to 61)	4 (4 to 4)
Serbia	2008	450 (418 to 485)	280 (256 to 306)	154 (134 to 175)	13 (7 to 23)	2 (2 to 2)	1126 (1046 to 1214)	700 (641 to 765)	386 (336 to 439)	34 (18 to 57)	6 (6 to 6)

Serbia	2009	452 (422 to 486)	277 (254 to 303)	159 (140 to 180)	12 (6 to 20)	4 (4 to 4)	1131 (1057 to 1216)	694 (634 to 759)	399 (350 to 451)	29 (15 to 51)	9 (9 to 9)
Serbia	2010	454 (424 to 488)	278 (254 to 303)	164 (143 to 187)	10 (5 to 17)	2 (2 to 2)	1135 (1061 to 1222)	695 (636 to 757)	411 (358 to 467)	25 (13 to 42)	5 (5 to 5)
Serbia	2011	457 (427 to 490)	275 (252 to 299)	167 (145 to 188)	9 (4 to 15)	6 (6 to 6)	1142 (1068 to 1227)	689 (631 to 747)	417 (363 to 471)	22 (11 to 38)	14 (14 to 14)
Serbia	2012	469 (438 to 502)	284 (260 to 308)	172 (151 to 193)	8 (4 to 15)	4 (4 to 4)	1173 (1095 to 1257)	710 (652 to 770)	432 (379 to 483)	21 (10 to 37)	10 (10 to 10)
Serbia	2013	460 (429 to 492)	274 (252 to 297)	176 (155 to 198)	8 (4 to 13)	2 (2 to 2)	1151 (1072 to 1231)	686 (630 to 743)	442 (388 to 496)	19 (10 to 33)	4 (4 to 4)
Serbia	2014	462 (429 to 496)	273 (250 to 297)	182 (160 to 205)	7 (4 to 12)	1 (1 to 1)	1157 (1073 to 1240)	682 (625 to 743)	455 (401 to 514)	18 (9 to 31)	2 (2 to 2)
Serbia	2015	452 (417 to 486)	263 (239 to 289)	182 (160 to 206)	7 (3 to 12)	1 (1 to 1)	1132 (1044 to 1216)	657 (598 to 722)	456 (400 to 515)	17 (9 to 29)	2 (2 to 2)
Serbia	2016	461 (420 to 499)	265 (239 to 292)	187 (162 to 215)	7 (3 to 12)	3 (3 to 3)	1154 (1052 to 1248)	663 (597 to 732)	468 (406 to 537)	17 (9 to 30)	6 (6 to 6)
Serbia	2017	465 (423 to 512)	264 (234 to 295)	191 (162 to 224)	7 (3 to 12)	4 (4 to 4)	1163 (1059 to 1281)	660 (586 to 738)	477 (404 to 560)	17 (8 to 30)	9 (9 to 9)
Serbia	2018	493 (450 to 540)	283 (252 to 316)	199 (169 to 234)	7 (4 to 13)	3 (3 to 3)	1233 (1125 to 1352)	707 (630 to 791)	499 (423 to 586)	18 (9 to 32)	8 (8 to 8)

Serbia	2019	515 (469 to 563)	297 (264 to 334)	207 (176 to 243)	8 (4 to 13)	3 (3 to 3)	1289 (1173 to 1410)	744 (661 to 835)	518 (439 to 607)	19 (9 to 33)	9 (8 to 9)
Serbia	2020	539 (490 to 593)	312 (277 to 350)	216 (183 to 253)	8 (4 to 14)	3 (3 to 4)	1350 (1225 to 1483)	781 (693 to 877)	540 (458 to 633)	20 (10 to 35)	9 (8 to 9)
Serbia	2021	554 (505 to 608)	322 (286 to 362)	219 (186 to 257)	8 (4 to 14)	4 (3 to 4)	1385 (1263 to 1522)	807 (715 to 906)	549 (466 to 644)	20 (10 to 36)	9 (8 to 10)
Serbia	2022	566 (516 to 620)	331 (293 to 372)	223 (190 to 262)	8 (4 to 15)	4 (3 to 4)	1417 (1292 to 1552)	828 (732 to 931)	559 (475 to 656)	21 (10 to 36)	9 (8 to 10)
Serbia	2023	578 (525 to 638)	338 (298 to 380)	228 (193 to 267)	8 (4 to 15)	4 (3 to 4)	1446 (1313 to 1597)	845 (745 to 952)	570 (484 to 669)	21 (11 to 37)	9 (9 to 11)
Serbia	2024	590 (533 to 651)	345 (304 to 389)	232 (197 to 273)	9 (4 to 15)	4 (3 to 4)	1475 (1334 to 1630)	862 (761 to 973)	582 (494 to 682)	22 (11 to 38)	10 (9 to 11)
Serbia	2025	602 (544 to 662)	351 (309 to 397)	237 (201 to 278)	9 (4 to 16)	4 (3 to 4)	1505 (1362 to 1658)	879 (773 to 992)	594 (504 to 696)	22 (11 to 39)	10 (9 to 11)
Serbia	2026	614 (554 to 679)	359 (315 to 405)	242 (206 to 284)	9 (5 to 16)	4 (4 to 5)	1537 (1386 to 1699)	897 (789 to 1013)	607 (515 to 711)	23 (11 to 40)	10 (9 to 12)
Serbia	2027	627 (564 to 692)	366 (323 to 415)	248 (210 to 290)	9 (5 to 16)	4 (4 to 5)	1568 (1411 to 1730)	915 (808 to 1039)	620 (526 to 727)	23 (12 to 41)	10 (9 to 12)
Serbia	2028	639 (578 to 703)	373 (327 to 425)	253 (215 to 296)	9 (5 to 17)	4 (4 to 5)	1600 (1445 to 1759)	933 (818 to 1063)	633 (537 to 741)	24 (12 to 42)	11 (9 to 12)

Serbia	2029	653 (585 to 727)	380 (333 to 433)	258 (219 to 302)	10 (5 to 17)	4 (4 to 5)	1633 (1464 to 1819)	952 (833 to 1084)	646 (549 to 756)	24 (12 to 43)	11 (9 to 13)
Serbia	2030	666 (596 to 743)	388 (338 to 442)	264 (224 to 309)	10 (5 to 17)	4 (4 to 5)	1666 (1492 to 1858)	970 (845 to 1106)	660 (560 to 773)	25 (12 to 44)	11 (9 to 13)
Serbia	2031	679 (611 to 752)	396 (343 to 452)	269 (228 to 316)	10 (5 to 18)	5 (4 to 5)	1700 (1528 to 1882)	990 (859 to 1131)	674 (572 to 790)	25 (13 to 45)	11 (10 to 14)
Serbia	2032	693 (620 to 774)	403 (348 to 463)	275 (233 to 322)	10 (5 to 18)	5 (4 to 6)	1735 (1552 to 1936)	1010 (872 to 1159)	688 (584 to 807)	26 (13 to 46)	12 (10 to 14)
Serbia	2033	708 (633 to 788)	412 (355 to 473)	281 (239 to 329)	11 (5 to 19)	5 (4 to 6)	1771 (1584 to 1973)	1030 (887 to 1183)	702 (597 to 824)	27 (13 to 47)	12 (10 to 14)
Serbia	2034	722 (645 to 801)	420 (360 to 484)	287 (244 to 336)	11 (5 to 19)	5 (4 to 6)	1808 (1614 to 2005)	1052 (901 to 1211)	717 (610 to 841)	27 (13 to 48)	12 (10 to 15)
Serbia	2035	737 (659 to 820)	429 (366 to 496)	292 (249 to 343)	11 (6 to 20)	5 (4 to 6)	1845 (1649 to 2053)	1073 (916 to 1240)	731 (622 to 858)	28 (14 to 49)	13 (10 to 15)
Serbia	2036	752 (672 to 836)	438 (373 to 507)	298 (253 to 350)	11 (6 to 20)	5 (4 to 6)	1883 (1682 to 2091)	1095 (933 to 1269)	746 (634 to 876)	28 (14 to 51)	13 (11 to 16)
Serbia	2037	767 (683 to 864)	446 (379 to 519)	303 (258 to 356)	12 (6 to 21)	5 (4 to 6)	1918 (1708 to 2161)	1117 (948 to 1298)	759 (646 to 891)	29 (14 to 52)	13 (11 to 16)
Serbia	2038	781 (698 to 876)	455 (385 to 529)	309 (263 to 363)	12 (6 to 21)	5 (4 to 7)	1955 (1746 to 2192)	1139 (964 to 1323)	772 (657 to 907)	30 (15 to 53)	14 (11 to 17)

Serbia	2039	797 (709 to 892)	465 (393 to 540)	314 (267 to 369)	12 (6 to 22)	6 (5 to 7)	1993 (1775 to 2233)	1163 (982 to 1352)	786 (669 to 924)	30 (15 to 54)	14 (11 to 18)
Serbia	2040	812 (723 to 910)	475 (400 to 554)	319 (272 to 376)	12 (6 to 22)	6 (5 to 7)	2033 (1809 to 2278)	1188 (1000 to 1386)	799 (680 to 940)	31 (15 to 56)	14 (12 to 18)
Serbia	2041	829 (735 to 932)	485 (408 to 568)	325 (276 to 382)	13 (6 to 23)	6 (5 to 7)	2074 (1839 to 2331)	1214 (1020 to 1422)	813 (692 to 956)	32 (16 to 57)	15 (12 to 18)
Serbia	2042	846 (747 to 948)	496 (415 to 583)	331 (281 to 389)	13 (6 to 23)	6 (5 to 8)	2116 (1868 to 2372)	1242 (1038 to 1460)	827 (703 to 972)	32 (16 to 58)	15 (12 to 19)
Serbia	2043	863 (761 to 965)	508 (424 to 599)	336 (285 to 395)	13 (7 to 24)	6 (5 to 8)	2160 (1904 to 2415)	1270 (1062 to 1498)	841 (714 to 989)	33 (16 to 59)	15 (12 to 20)
Serbia	2044	882 (774 to 996)	520 (433 to 613)	342 (290 to 402)	14 (7 to 24)	6 (5 to 8)	2206 (1937 to 2493)	1301 (1085 to 1535)	856 (726 to 1005)	34 (17 to 61)	15 (12 to 21)
Serbia	2045	900 (798 to 1014)	532 (441 to 632)	348 (295 to 408)	14 (7 to 25)	6 (0 to 8)	2252 (1997 to 2538)	1332 (1103 to 1581)	870 (738 to 1022)	35 (17 to 62)	16 (0 to 21)
Serbia	2046	919 (809 to 1038)	545 (451 to 645)	353 (300 to 415)	14 (7 to 25)	6 (0 to 9)	2299 (2024 to 2598)	1364 (1129 to 1614)	884 (751 to 1039)	35 (18 to 64)	16 (0 to 21)
Serbia	2047	938 (830 to 1061)	559 (462 to 665)	359 (305 to 422)	14 (7 to 26)	6 (0 to 9)	2348 (2078 to 2655)	1398 (1156 to 1665)	898 (762 to 1057)	36 (18 to 65)	16 (0 to 22)
Serbia	2048	959 (844 to 1087)	573 (472 to 686)	365 (309 to 429)	15 (7 to 26)	6 (0 to 9)	2399 (2112 to 2719)	1434 (1182 to 1716)	913 (774 to 1075)	37 (18 to 66)	16 (0 to 23)

Serbia	2049	979 (861 to 1111)	587 (485 to 699)	370 (314 to 437)	15 (8 to 27)	6 (0 to 9)	2450 (2154 to 2781)	1470 (1214 to 1749)	927 (787 to 1093)	38 (19 to 68)	15 (0 to 24)
Serbia	2050	1000 (879 to 1134)	602 (496 to 716)	376 (319 to 444)	15 (8 to 28)	6 (0 to 10)	2503 (2199 to 2837)	1507 (1241 to 1792)	942 (798 to 1112)	39 (19 to 69)	15 (0 to 25)
Seychelles	1995	630 (586 to 679)	380 (343 to 425)	111 (89 to 135)	0 (0 to 0)	138 (138 to 138)	1163 (1083 to 1256)	703 (634 to 785)	205 (164 to 250)	0 (0 to 0)	256 (256 to 256)
Seychelles	1996	526 (487 to 571)	412 (380 to 451)	113 (93 to 137)	0 (0 to 0)	1 (1 to 1)	972 (901 to 1055)	762 (703 to 834)	209 (172 to 253)	0 (0 to 0)	2 (2 to 2)
Seychelles	1997	570 (531 to 612)	440 (409 to 477)	113 (94 to 136)	0 (0 to 0)	17 (17 to 17)	1054 (982 to 1131)	814 (756 to 881)	209 (175 to 251)	0 (0 to 0)	31 (31 to 31)
Seychelles	1998	599 (560 to 639)	463 (431 to 499)	115 (96 to 138)	0 (0 to 0)	20 (20 to 20)	1106 (1034 to 1182)	855 (796 to 923)	213 (178 to 256)	0 (0 to 0)	38 (38 to 38)
Seychelles	1999	567 (530 to 604)	444 (411 to 478)	115 (97 to 135)	0 (0 to 0)	8 (8 to 8)	1047 (979 to 1116)	821 (760 to 883)	212 (180 to 249)	0 (0 to 0)	14 (14 to 14)
Seychelles	2000	582 (544 to 620)	445 (413 to 480)	118 (101 to 138)	0 (0 to 0)	18 (18 to 18)	1075 (1005 to 1146)	823 (764 to 888)	219 (186 to 255)	0 (0 to 0)	33 (33 to 33)
Seychelles	2001	511 (475 to 547)	385 (357 to 415)	122 (103 to 143)	0 (0 to 0)	3 (3 to 3)	944 (879 to 1011)	712 (659 to 767)	225 (190 to 264)	0 (0 to 0)	6 (6 to 6)
Seychelles	2002	567 (528 to 604)	436 (402 to 470)	125 (106 to 146)	0 (0 to 0)	5 (5 to 5)	1047 (976 to 1117)	806 (743 to 868)	231 (196 to 269)	0 (0 to 0)	10 (10 to 10)
Seychelles	2003	525 (491 to 559)	380 (353 to 410)	121 (102 to 141)	0 (0 to 0)	23 (23 to 23)	970 (907 to 1032)	703 (652 to 758)	224 (189 to 260)	0 (0 to 0)	43 (43 to 43)

Seychelles	2004	479 (448 to 510)	341 (316 to 368)	121 (102 to 141)	0 (0 to 0)	16 (16 to 16)	884 (828 to 942)	631 (583 to 681)	223 (189 to 261)	0 (0 to 0)	30 (30 to 30)
Seychelles	2005	496 (462 to 529)	350 (324 to 377)	130 (111 to 152)	0 (0 to 0)	15 (15 to 15)	916 (854 to 977)	647 (600 to 696)	241 (205 to 281)	0 (0 to 0)	28 (28 to 28)
Seychelles	2006	530 (495 to 567)	384 (355 to 414)	144 (122 to 168)	0 (0 to 0)	3 (3 to 3)	980 (914 to 1047)	709 (656 to 766)	265 (225 to 311)	0 (0 to 0)	5 (5 to 5)
Seychelles	2007	547 (508 to 584)	390 (360 to 422)	155 (132 to 180)	0 (0 to 0)	2 (2 to 2)	1012 (940 to 1078)	722 (665 to 780)	287 (244 to 333)	0 (0 to 0)	3 (3 to 3)
Seychelles	2008	421 (391 to 452)	273 (251 to 296)	146 (125 to 168)	0 (0 to 0)	2 (2 to 2)	778 (722 to 835)	504 (464 to 546)	271 (232 to 310)	0 (0 to 0)	3 (3 to 3)
Seychelles	2009	478 (443 to 512)	314 (288 to 340)	162 (140 to 186)	0 (0 to 0)	2 (2 to 2)	884 (819 to 946)	580 (533 to 628)	299 (258 to 343)	0 (0 to 0)	5 (5 to 5)
Seychelles	2010	523 (487 to 559)	347 (319 to 375)	168 (146 to 191)	1 (1 to 2)	7 (7 to 7)	966 (901 to 1032)	641 (590 to 693)	310 (269 to 354)	2 (1 to 4)	12 (12 to 12)
Seychelles	2011	579 (542 to 618)	396 (367 to 426)	177 (155 to 201)	3 (2 to 6)	3 (3 to 3)	1071 (1003 to 1142)	732 (678 to 788)	327 (286 to 372)	6 (3 to 11)	6 (6 to 6)
Seychelles	2012	636 (599 to 675)	444 (414 to 476)	181 (159 to 206)	6 (3 to 11)	4 (4 to 4)	1175 (1108 to 1248)	821 (765 to 879)	334 (295 to 380)	12 (6 to 20)	8 (8 to 8)
Seychelles	2013	654 (618 to 694)	463 (433 to 494)	179 (159 to 202)	6 (3 to 10)	6 (6 to 6)	1208 (1143 to 1282)	856 (801 to 913)	331 (294 to 373)	11 (6 to 19)	10 (10 to 10)
Seychelles	2014	635 (601 to 671)	449 (422 to 477)	181 (161 to 203)	5 (3 to 9)	1 (1 to 1)	1174 (1111 to 1240)	829 (780 to 882)	334 (297 to 376)	9 (5 to 16)	1 (1 to 1)
Seychelles	2015	656 (619 to 694)	464 (434 to 494)	184 (163 to 208)	7 (3 to 11)	1 (1 to 1)	1212 (1144 to 1282)	858 (802 to 914)	340 (301 to 384)	12 (6 to 21)	2 (2 to 2)

Seychelles	2016	740 (694 to 786)	535 (500 to 572)	194 (169 to 222)	10 (5 to 18)	1 (1 to 1)	1368 (1283 to 1453)	988 (924 to 1058)	359 (312 to 410)	19 (10 to 34)	1 (1 to 1)
Seychelles	2017	754 (697 to 817)	548 (505 to 595)	193 (163 to 225)	12 (6 to 21)	1 (1 to 1)	1394 (1288 to 1510)	1013 (933 to 1100)	357 (302 to 416)	22 (11 to 39)	1 (1 to 1)
Seychelles	2018	810 (740 to 881)	602 (547 to 659)	195 (166 to 227)	12 (6 to 22)	0 (0 to 0)	1496 (1368 to 1628)	1113 (1012 to 1217)	361 (307 to 419)	23 (12 to 40)	0 (0 to 0)
Seychelles	2019	833 (758 to 917)	621 (559 to 688)	200 (170 to 232)	13 (6 to 22)	0 (0 to 0)	1540 (1401 to 1694)	1148 (1034 to 1272)	369 (313 to 428)	23 (12 to 41)	0 (0 to 0)
Seychelles	2020	856 (773 to 946)	639 (567 to 715)	204 (174 to 238)	13 (7 to 23)	0 (0 to 0)	1583 (1429 to 1748)	1181 (1048 to 1321)	378 (321 to 439)	24 (12 to 43)	0 (0 to 0)
Seychelles	2021	870 (781 to 972)	650 (573 to 736)	207 (176 to 240)	13 (7 to 24)	0 (0 to 0)	1609 (1443 to 1796)	1201 (1059 to 1360)	383 (325 to 444)	25 (13 to 44)	0 (0 to 0)
Seychelles	2022	886 (789 to 991)	662 (578 to 756)	210 (178 to 243)	14 (7 to 24)	0 (0 to 0)	1637 (1458 to 1832)	1224 (1068 to 1397)	388 (329 to 450)	25 (13 to 44)	0 (0 to 0)
Seychelles	2023	899 (799 to 1020)	673 (583 to 778)	212 (180 to 246)	14 (7 to 24)	0 (0 to 0)	1661 (1476 to 1884)	1243 (1077 to 1437)	392 (333 to 455)	26 (13 to 45)	0 (0 to 0)
Seychelles	2024	911 (798 to 1035)	683 (583 to 794)	215 (182 to 249)	14 (7 to 25)	0 (0 to 0)	1684 (1475 to 1914)	1261 (1078 to 1468)	397 (337 to 460)	26 (13 to 46)	0 (0 to 0)
Seychelles	2025	925 (813 to 1063)	693 (590 to 814)	217 (185 to 253)	14 (7 to 25)	0 (0 to 0)	1709 (1502 to 1965)	1281 (1091 to 1504)	402 (341 to 467)	26 (13 to 46)	0 (0 to 0)

Seychelles	2026	937 (813 to 1071)	702 (594 to 825)	220 (186 to 255)	14 (7 to 26)	0 (0 to 0)	1731 (1502 to 1980)	1298 (1099 to 1525)	406 (344 to 472)	27 (14 to 47)	0 (0 to 0)
Seychelles	2027	951 (823 to 1110)	714 (599 to 850)	222 (188 to 259)	15 (7 to 26)	0 (0 to 0)	1757 (1521 to 2052)	1319 (1107 to 1572)	411 (348 to 478)	27 (14 to 48)	0 (0 to 0)
Seychelles	2028	964 (827 to 1115)	725 (604 to 860)	225 (190 to 262)	15 (8 to 26)	0 (0 to 0)	1782 (1528 to 2061)	1339 (1116 to 1590)	416 (352 to 484)	28 (14 to 49)	0 (0 to 0)
Seychelles	2029	978 (844 to 1145)	736 (610 to 876)	228 (192 to 265)	15 (8 to 27)	0 (0 to 0)	1808 (1560 to 2116)	1360 (1127 to 1619)	421 (355 to 490)	28 (14 to 49)	0 (0 to 0)
Seychelles	2030	992 (846 to 1161)	747 (615 to 901)	230 (194 to 269)	15 (8 to 27)	0 (0 to 0)	1834 (1563 to 2146)	1380 (1136 to 1665)	425 (359 to 496)	28 (14 to 50)	0 (0 to 0)
Seychelles	2031	1006 (857 to 1187)	758 (617 to 923)	233 (197 to 271)	16 (8 to 27)	0 (0 to 0)	1860 (1584 to 2194)	1400 (1141 to 1706)	430 (364 to 502)	29 (15 to 51)	0 (0 to 0)
Seychelles	2032	1021 (859 to 1204)	769 (621 to 938)	236 (199 to 275)	16 (8 to 28)	0 (0 to 0)	1886 (1587 to 2225)	1421 (1147 to 1734)	436 (368 to 508)	29 (15 to 51)	0 (0 to 0)
Seychelles	2033	1035 (876 to 1225)	781 (632 to 960)	239 (201 to 278)	16 (8 to 28)	0 (0 to 0)	1913 (1619 to 2264)	1443 (1167 to 1775)	441 (371 to 514)	30 (15 to 52)	0 (0 to 0)
Seychelles	2034	1051 (875 to 1247)	793 (635 to 978)	241 (203 to 282)	16 (8 to 29)	0 (0 to 0)	1943 (1618 to 2304)	1466 (1174 to 1807)	446 (375 to 520)	30 (15 to 53)	0 (0 to 0)
Seychelles	2035	1066 (895 to 1266)	805 (641 to 998)	244 (205 to 285)	17 (8 to 29)	0 (0 to 0)	1970 (1654 to 2341)	1488 (1184 to 1844)	451 (380 to 526)	31 (15 to 54)	0 (0 to 0)

Seychelles	2036	1082 (894 to 1295)	818 (649 to 1018)	247 (208 to 288)	17 (8 to 30)	0 (0 to 0)	2000 (1652 to 2393)	1511 (1199 to 1882)	457 (384 to 532)	31 (16 to 55)	0 (0 to 0)
Seychelles	2037	1098 (914 to 1324)	830 (653 to 1041)	250 (210 to 291)	17 (9 to 30)	0 (0 to 0)	2029 (1689 to 2447)	1535 (1207 to 1925)	462 (389 to 538)	32 (16 to 56)	0 (0 to 0)
Seychelles	2038	1115 (924 to 1349)	844 (663 to 1061)	253 (213 to 294)	17 (9 to 31)	0 (0 to 0)	2061 (1708 to 2494)	1561 (1225 to 1962)	468 (393 to 544)	32 (16 to 56)	0 (0 to 0)
Seychelles	2039	1132 (937 to 1371)	858 (669 to 1088)	256 (215 to 298)	18 (9 to 31)	0 (0 to 0)	2092 (1732 to 2534)	1586 (1237 to 2011)	474 (397 to 551)	33 (16 to 57)	0 (0 to 0)
Seychelles	2040	1149 (938 to 1391)	871 (671 to 1099)	260 (217 to 303)	18 (9 to 32)	0 (0 to 0)	2123 (1734 to 2570)	1610 (1241 to 2032)	480 (402 to 559)	33 (17 to 58)	0 (0 to 0)
[truncated: 492,544 more chars]
